# De novo transcriptome assembly of *Dalbergia sissoo* Roxb. (*Fabaceae*) under *Botryodiplodia theobromae*-induced dieback disease

**DOI:** 10.1038/s41598-023-45982-8

**Published:** 2023-11-22

**Authors:** Ummul Buneen Zafar, Muhammad Shahzaib, Rana Muhammad Atif, Sultan Habibullah Khan, Muhammad Zeeshan Niaz, Khalid Shahzad, Nighat Chughtai, Faisal Saeed Awan, Muhammad Tehseen Azhar, Iqrar Ahmad Rana

**Affiliations:** 1 Centre of Agricultural Biochemistry and Biotechnology, University of Agriculture, Faisalabad, Faisalabad, 38000 Punjab Pakistan; 2 Centre for Advanced Studies in Agriculture and Food Security, University of Agriculture, Faisalabad, Faisalabad, 38000 Punjab Pakistan; 3 Department of Plant Breeding and Genetics, University of Agriculture, Faisalabad, Faisalabad, 38000 Punjab Pakistan; 4 National Center for Genome Editing (Gene Editing of Biological Agents for Nutritional, Biochemicals and Therapeutic Purposes), University of Agriculture, Faisalabad, Punjab Pakistan; 5 Plant Pathology Research Institute, Ayub Agriculture Research Institute, Faisalabad, 38850 Punjab Pakistan; 6 Punjab Forestry Research Institute, Faisalabad, 37620 Punjab Pakistan

**Keywords:** Transcriptomics, Gene expression profiling

## Abstract

*Dalbergia sissoo* Roxb. (Shisham) is a timber-producing species of economic, cultural, and medicinal importance in the Indian subcontinent. In the past few decades, Shisham's dieback disease caused by the fungus *Botryodiplodia theobromae* has become an evolving issue in the subcontinent endangering its survival. To gain insights into this issue, a standard transcriptome assembly was deployed to assess the response of *D. sissoo* at the transcriptomic level under the stress of *B. theobromae* infection. For RNA isolation, the control and infected leaf tissue samples were taken from 1-year-old greenhouse-grown *D. sissoo* plants after 20 days of stem-base spore inoculation. cDNA synthesis was performed from these freshly isolated RNA samples that were then sent for sequencing. About 18.14 Gb (Giga base) of data was generated using the BGISEQ-500 sequencing platform. In terms of Unigenes, 513,821 were identified after a combined assembly of all samples and then filtering the abundance. The total length of Unigenes, their average length, N50, and GC-content were 310,523,693 bp, 604 bp, 1,101 bp, and 39.95% respectively. The Unigenes were annotated using 7 functional databases i.e., 200,355 (NR: 38.99%), 164,973 (NT: 32.11%), 123,733 (Swissprot: 24.08%), 142,580 (KOG: 27.75%), 139,588 (KEGG: 27.17%), 99,752 (GO: 19.41%), and 137,281 (InterPro: 26.72%). Furthermore, the Transdecoder detected 115,762 CDS. In terms of SSR (Simple Sequence Repeat) markers, 62,863 of them were distributed on 51,508 Unigenes and on the predicted 4673 TF (Transcription Factor) coding Unigenes. A total of 16,018 up- and 19,530 down-regulated Differentially Expressed Genes (DEGs) were also identified. Moreover, the Plant Resistance Genes (PRGs) had a count of 9230. We are hopeful that in the future, these identified Unigenes, SSR markers, DEGs and PRGs will provide the prerequisites for managing Shisham dieback disease, its breeding, and in tree improvement programs.

## Introduction

*Dalbergia sissoo* Roxb. (Shisham), also known as the Indian Rosewood, belongs to the family *Fabaceae*. It is indigenous to the Indian subcontinent and is characterized by the leathery textures of its leaves, pink-white flowers, and the crookedness of the tree itself. The timber heartwood of *D. sissoo* is generally used for making high-grade furniture. Its stem bark has been found to contain dalbergichromene, a neoflavonoid of great significance^1^. In its native habitat, *D. sissoo* can be ranged under the threatened circle for being a high-value timber-producing species^2^. In terms of carbon sequestration, it has been observed that the trees of *D. sissoo* have an average sequestration capacity of 7.56 tha^−1^ (metric tons per hectare) with a carbon intake value of 27.735t CO_2_ (metric tons CO_2_)^3^. As a whole, *D. sissoo* and other *Dalbergia* tree species determine the extent of relative biodiversity in their native habitats, and they also have a significant economic and ecological impact. Due to these reasons, it is of foremost importance to establish the basis for strategies that can help in the conservation of *D. sissoo* under abiotic and biotic stresses^4^. The implementation of such programs can provide in-depth data on the precursors of resistance against the dieback disease. The genetic diversity analysis of *D. sissoo*, on the other hand, will help us ensure its survival under environmental stresses and conserve its precious natural resources, especially in the native regions^5,6^.

In Pakistan, the timber wood harvested from *D. sissoo* plays a vital role in the economy of the country through the woodwork industry. Recently, a constant decline in the population of *D. sissoo* has been observed in the eleven districts of Punjab with a mortality rate of 25–30%. The fungus spp. *B. theobromae* is the main culprit associated with infecting both the underground as well as the aerial parts of the tree. In different studies, when inoculated with *B. theobromae*, the healthy *D. sissoo* plants produced typical symptoms of dieback disease such as wilting and yellowing of leaves, cankers of the stem, and dieback on the branches to various degrees^7,8^. Furthermore, as a countermeasure, the fungicides were experimented with in vitro and it was found that 100 ppm was the optimum concentration of Topsin-M in reducing the growth rate of fungal mycelia. Additionally, the same *D. sissoo* decline patterns have also been observed in different areas of Bangladesh^9^. Another study isolated the contaminated samples from different parts of the *D. sissoo* tree, cultured them on Potato Dextrose Agar (PDA) and Czapek dox agar media, and examined all of them simultaneously through microscopic observation. They found that *B. theobromae* was the most frequently isolated fungus from infection suggesting its strong association with the Shisham dieback^10^.

Novel marker-assisted selection processes have shown unmatched potential for assessing the underlying genomic changes, identification of genes/germplasm for biotic and abiotic stress tolerance, genetic diversity, and natural variability among the tree species^11–16^. Several studies have reported molecular markers for *D. sissoo* germplasm such as RAPD and ISSR markers for genetic diversity analysis^17–23^. SSR markers have also proven to be very helpful in evaluating the structure and genetic diversity of plants^24–30^. The SSR markers have many significant comparative advantages such as their transferability to closely related species, reproducibility, abundance, co-dominance properties, and a relatively higher degree of subsequent polymorphism. SSR markers are generally divided into two categories based on their origin of derivation. First, there are conventional SSR markers identified from the genomic sequences, and second, there are Expressed Sequence Tag—Simple Sequence Repeats (EST-SSRs) which can be identified through transcribed RNA sequences^31^. These EST-SSR markers were previously identified and developed using traditional approaches. These approaches were costly, time-consuming, and laborious. Everything changed when transcriptome sequencing (RNA-seq) based on Next Generation Sequencing (NGS) was introduced. Various successful and comprehensive studies on SSR marker development have been performed on different tree species (no reference genome) through the utilization of NGS-based RNA-seq^32–34^. The transcriptomic analysis through the RNA-seq data can also be used to explore and mine the data for molecular and genetic breeding opportunities for threatened species through RNA-transcripts-based genome-wide analysis^35,36^.

In this study, we present the first transcriptome of *Dalbergia sissoo* under the stress of *B. theobromae* infection assembled using the BGISEQ-500 sequencing platform. The BGISEQ-500 sequencing platform was used due to the superior relative factors such as cost-effectiveness, high throughput, short-read sequencing, rapid turnaround, ease of use, phasing information, versatility for different applications, and customization options. There is no report to our knowledge that has comprehensively identified SSR markers, Differentially Expressed Genes (DEGs), and Plant Resistance Genes (PRGs) in *D. sissoo* under the stress from *B. theobromae* infection, simultaneously. Somewhat related work is reported on the ornamental tree of Japanese apricot (*prunus mume*). In this study, 1,212 total DEGs and Differentially Methylated Regions (DMR) involved in influencing the biosynthesis of anthocyanins in the chimera of flower color were characterized^37^. Similarly, the DEGs identified in Masson pine (*Pinus massoniana*) and recently in pine wood (*Pinus thunbergia*) conferred resistance against the pine wood nematode that causes the Pine wilt disease^38,39^. Moreover, 1,573 DEGs responded to the drought stress in the mahaleb cherry (*Prunus mahaleb*)^40^. In Mexican Lime (*Citrus aurantifolia*), the DEGs that responded to the biotic stress from Huanglongbing-causing *Candidatus Liberibacter asiaticus* were also identified^41^. We aimed to expand the transcriptomic resource library available for *D. sissoo* through the inclusion of high-quality transcriptome sequencing data under the stress of *B. theobromae* infection. Most of the research on this disease to date is either on conventional plant pathogenic interactions or the use of RAPD and Est-SSRs identified in related species, no genomics tools developed endogenously are present. The findings of this research will help in filling this gap. The discovery of putative DEGs can especially help us understanding the significant disease-related processes like biomarker discovery, pathway-mediating biological processes, disease classification, and subtyping using the generated up- and down-regulated gene expression datasets. The numerous identified PRGs, SSR markers, and Unigenes in conjunction with the genomic data, on the whole, will help in ensuring the survival of *D. sissoo* under environmental stresses, the conservation of its germplasms, and facilitate the hybridization and breeding programs of the future.

## Results

### Sequence read filtering

Before the beginning of the downstream analysis, all the sequenced reads were filtered for low-quality, adaptor-polluted, and high content of unknown base (N) reads. In terms of clean read quality metrics, a total of 124.83 million (M) raw reads were obtained, out of which 120.95 M were clean reads (Table 1) (see Supplementary Figure S1**)**. Moving on sample-wise, Rep1-Control had 30.91 M, Rep1-Disease contained 45.61 M, and Rep2-Disease had 44.43 M clean reads. Their Q20 percentage was 96.52%, 96.69%, and 96.81% respectively.

**Table 1:** Clean reads quality metrics.

Sample	Total raw reads (M)	Total clean reads (M)	Total clean bases (Gb)	Clean reads Q20 (%)	Clean reads Q30 (%)	Clean reads ratio (%)
Rep1-Control	31.93	30.91	4.64	96.52	91.64	96.81
Rep1-Disease	47.33	45.61	6.84	96.69	92.23	96.38
Rep2-Disease	45.57	44.43	6.66	96.81	92.20	97.49

### BGISEQ-500 transcriptome sequencing, and de novo assembly

As this was a project without a reference genome, the reference sequence was obtained after the clean sequenced reads were assembled using Trinity^42^ for subsequent analysis. In total, 1,000,549 individual transcripts and 513,821 Unigenes were obtained. They had a mean length of 483 bp (N50 = 818 bp) and 604 bp (N50 = 1,101 bp) respectively (Table 2) (see Supplementary Figure S2a). The number of Unigenes assembled for Rep1-Control was 205,077, 136,502 for Rep1-Disease, and 221,789 for Rep2-Disease (Table 3) (see Supplementary Table S1). The lengths of the Unigenes ranged from 1179 bp to 251,253 bp with 310,253,693 nucleotides in total (see Supplementary Figure** S2b**).

**Table 2:** Quality metrics of transcripts.

Sample	Total number	Total length	Mean length	N50	N70	N90	GC (%)
Rep1-Control	354,390	146,146,365	412	467	267	197	37.88
Rep1-Disease	259,336	144,905,195	558	1,151	399	211	40.14
Rep2-Disease	386,823	197,303,972	510	836	344	210	40.77

**Table 3:** Quality metrics of Unigenes.

Sample	Total number	Total length	Mean length	N50	N70	N90	GC (%)
Rep1-Control	205,077	108,527,100	529	817	345	232	38.47
Rep1-Disease	136,702	99,406,573	727	1,465	669	258	40.47
Rep2-Disease	221,789	151,540,658	683	1,285	530	261	40.95
All-Uni gene	513,821	310,523,693	604	1,101	418	242	39.95

### Functional annotation of Unigenes

After the completion of assembly, 7 functional databases were used to functionally annotate the Unigenes. These functional databases include NR, NT, GO, KOG, KEGG, SwissProt, and InterPro. Out of all the 513,821 Unigenes, 223,132 (43.43%) Unigenes were successfully annotated in at least one of the seven databases. Moreover, 52,244 (10.36%) of them showed annotations in all seven of the databases and the Unigene-associated functional pathway maps (Table 4) (see **Supplementary File S1**).

**Table 4:** Annotation summary.

Values	Total	NT	NR	Swiss-Prot	KEGG	KOG	InterPro	GO	Intersection	Overall
Number	513,821	200,355	164,973	123,733	139,588	142,580	137,281	99,752	53,244	223,132
Percentage	100%	38.99%	32.11%	24.08%	27.17%	27.75%	26.72%	19.41%	10.36%	43.33%

**Figure 1:**
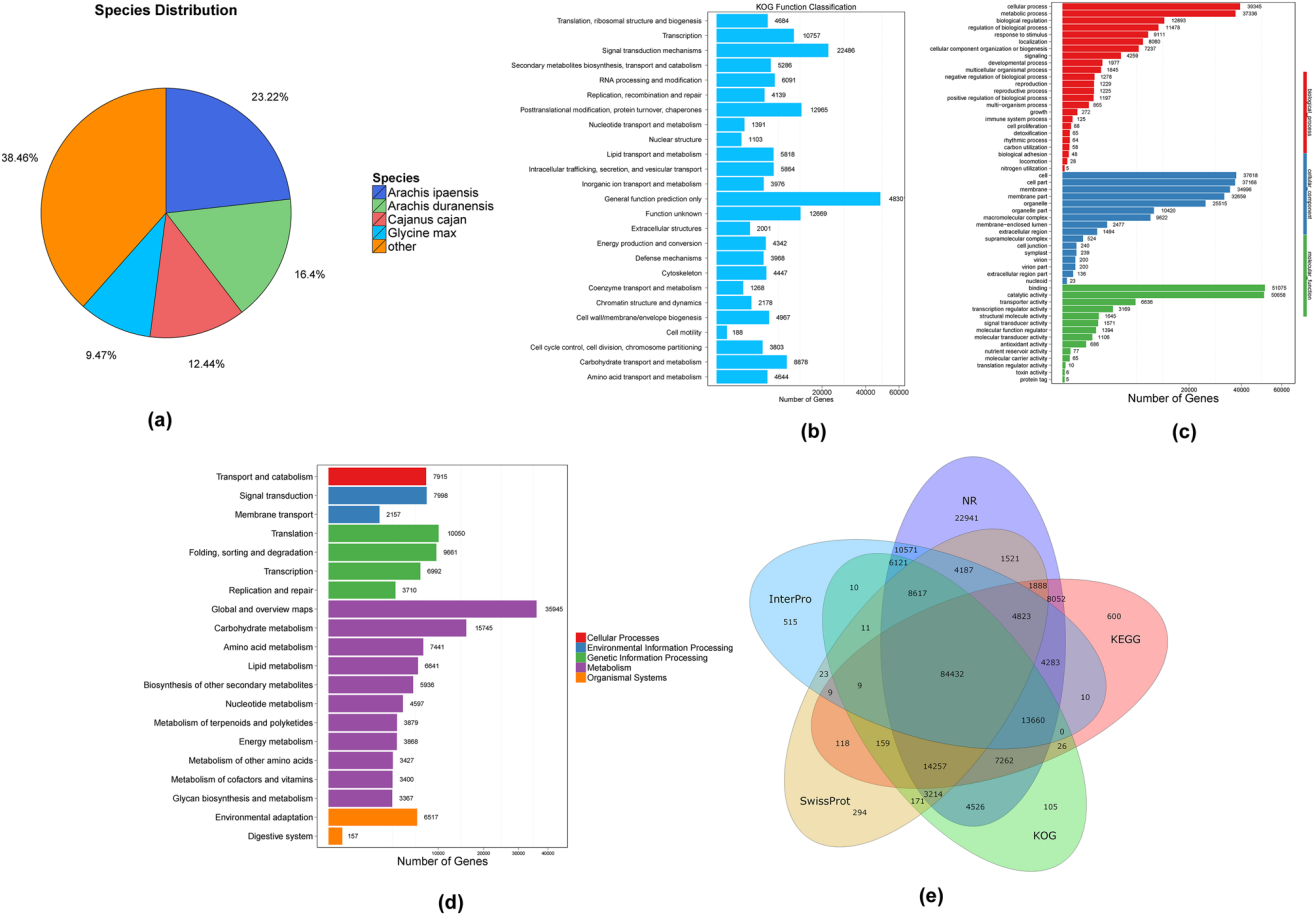
(**a**) Distribution of NR annotated species. (**b**) Functional distribution of KOG annotation. The X-axis represents the number of Unigenes and the Y-axis represents the KOG functional category. (**c**) Functional distribution of GO annotation. The X-axis represents the number of Unigenes and the Y-axis represents the Gene Ontology functional category. (**d**) Functional distribution of KEGG annotation. The X-axis represents the number of Unigenes and the Y-axis represents the KEGG functional category. KEGG metabolic pathway is categorized into 7 branches: Cellular Processes, Environmental Information Processing, Genetic Information Processing, Human Disease, Metabolism, Organismal Systems, and Drug Development. (**e**) Venn diagram between NR, KOG, KEGG, Swissprot, and Interpro.

The NT and NR databases are the official nucleic acid and protein databases of the NCBI, respectively. In terms of NT functional annotations using BLAST (Basic Local Alignment Search Tool), NT constitutes 164,973 (32.11%) while NR had 200,355 (38.9%) functional annotations. The Unigene annotation ratio in the NR database was also calculated for different species (Fig. 1a). In terms of functional classification through KOG (euKaryotic Ortholog Groups), the calculations of 142,580 Unigenes were distributed among 25 functional groups. The largest group was the ‘general function prediction only’ group with 48,301 (33.87%) Unigene annotations. Following this, the signal transduction mechanism group had 22,486 (15.77%), post-translational modification, protein turnover, and chaperones group had 12,965 (9.09%), and the unknown function group had 12,669 (8.88%) Unigene annotations (Fig. 1b). The Unigenes that were successfully aligned to the NR database through KOG were then annotated using the GO (Gene Ontology)^43^ database using Blast2GO^44^. The GO terms distribution was calculated using three categories viz. the biological process, cellular component, and molecular functions (Fig. 1c). In total, 99,752 (19.41%) Unigenes were annotated from the GO database. In the biological processes category, 39,345 (39.44%) Unigenes were annotated in the cellular process, 37,336 (37.42%) in the metabolic process, and 12,893 (12.92%) in biological regulation. Only 5 Unigenes were annotated in the biological process of nitrogen fixation. In the cellular components category, 37,618 (37.71%) were annotated in the cell, 37,168 (37.26%) were in the cell part, and 34,996 (35.08%) were in the membrane. Lastly, in the molecular functions category, 51,075 (51.20%) were annotated in binding, 50,658 (50.78%) in catalytic activity, and 6,636 (6.65%) in the transporter activity. Only 6 and 5 Unigenes were annotated in the molecular functions of toxin activity and protein tag, respectively.

The KEGG^45^ database was also used to annotate and distribute the Unigenes at KEGG Level 1 and KEGG Level 2. In total, 139,588 Unigenes were annotated and all pathways were condensed into 5 clades (cellular processes, environmental information processing, genetic information processing, metabolism, organismal systems) and 20 subgroups (Fig. 1d). Among all the subgroups and clades, the ‘global and overview maps’ group from the Metabolism clade had 35,945 (25.75%) Unigenes. The ‘translation’ group from the genetic information processing clade had 10,050 (7.2%) Unigenes. Similarly, the ‘signal transduction group’ from the environmental information processing clade had 7,998 (5.73%) Unigenes and the ‘transport and catabolism singular group’ from the cellular processes clade had 7,915 (5.67%) Unigenes. Lastly, the ‘environmental adaptation’ group from organismal systems clade had 6,517 (4.67%) Unigenes. Additionally, the identified Unigenes were also aligned to the InterPro database using InterProscan software. Furthermore, the Unigene annotations were also performed through Swiss-Prot because the database is based upon the manually reviewed, high-quality annotated, and non-redundant protein sequences from UniProt Knowledgebase (UniProtKB). The annotation results from all the databases have also been illustrated using a Venn^46^ diagrammatical representation (Fig. 1e).

**Figure 2:**
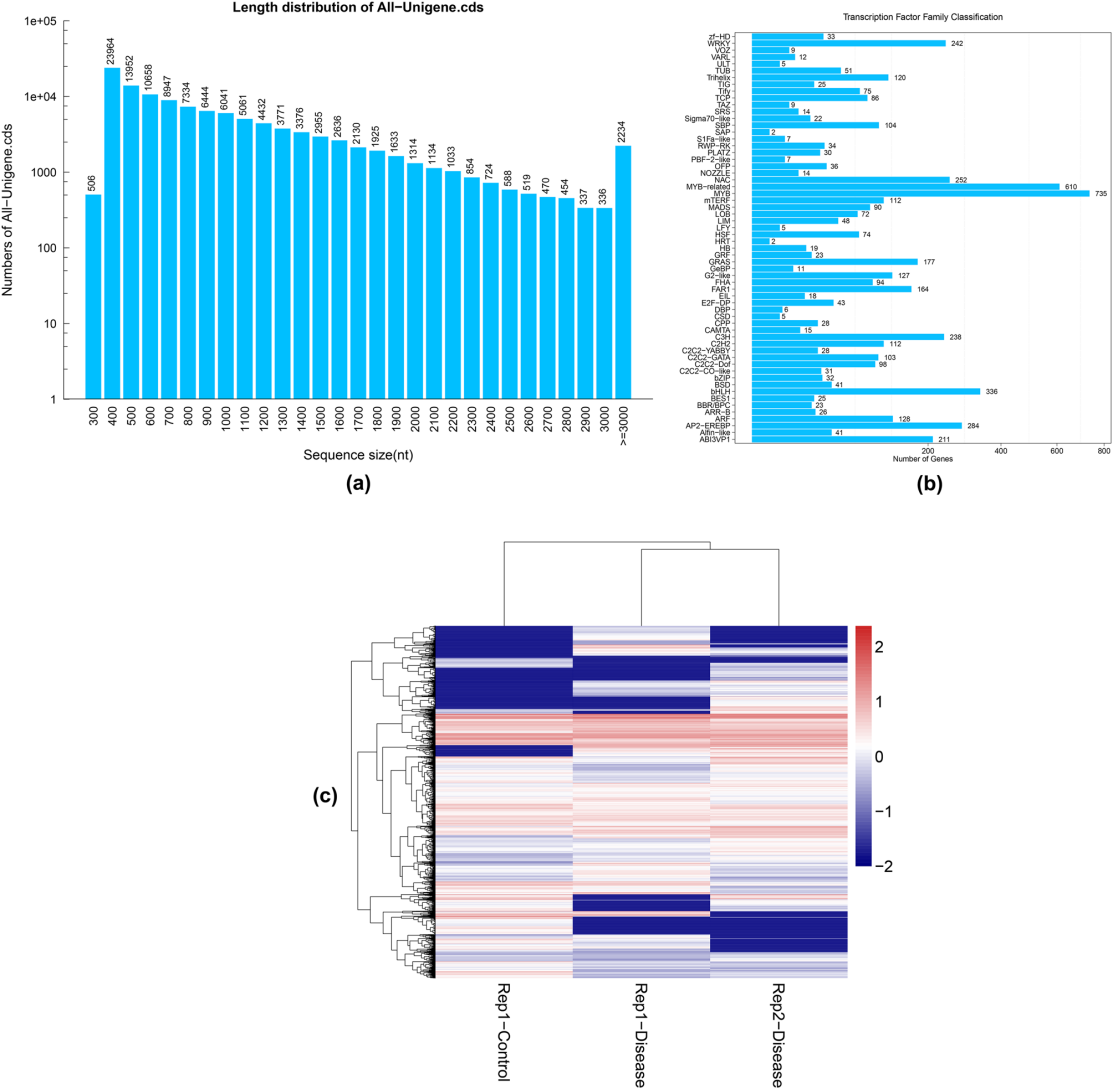
(**a**) CDS length distribution. The X-axis represents the length of CDS and the Y-axis represents the number of CDS. (**b**) Transcription Factor Family Classification of Unigenes. The X-axis represents the number of Unigenes and the Y-axis represents the family of TF. (**c**) Distribution of TF expression level. Each column represents a sample and each row represents a transcription factor.

The CDS (Coding DNA Sequences) were identified in the candidate coding regions using the TransDecoder software. The longest identified ORF (Open Reading Frame) was curated through BLAST^47^ against hmmscan^48^ and Swiss-Prot to predict CDS using Pfam protein homology sequences. The total number of identified CDS was 115,762 with a total length of 107,534,790 bp (Fig. 2a) (see Supplementary Table S2a–S2b). The minimum and maximum CDS lengths were 297 and 15,342 respectively. They also had an overall N50 of 1,209 bp with a GC percentage of 44.49% (see Supplementary Table S3). Similarly, a total of 4,673 Transcription Factor (TF) encoding genes were also predicted. The predicted Unigenes were classified into TF families. The most prominent families with their number of predicted Unigenes were MYB (735), MYB-related (610), bHLH (336), AP2-EREBP (284), and NAC (252) (Fig. 2b). The distribution of expression level of TFs among Rep1-Control, Rep1-Disease, and Rep2-Disease was also analyzed (Fig. 2c).

### Unigene SSR identification and plant disease resistance gene identification

In terms of SSR markers, 62,863 of them were identified from 513,821 Unigenes (310,523,693 bp) with a distribution density of 202.44 SSRs per Megabase (Mb). In total, 51,508 Unigenes contained SSRs. The maximum number of bases interrupting 2 SSRs in a compound microsatellite was 100. Moreover, the number of sequences containing more than 1 SSR was 8809 and the number of SSRs present in compound formation was 4,175. In terms of repeat motifs, the most abundant of them were di-nucleotide (23,119, 36.78%), followed by tri-nucleotide (18,519, 29.46%) and mono-nucleotide (17,592, 27.98%). These three motif types were observed to be the most dominant constituting about 94.22% of the total. Only 1,410, 1,159, and 1,064 motif repeats were shown by pentanucleotides, hexanucleotides, and quad-nucleotides respectively (see Supplementary Table S4). A total of 289 different repeat motifs were detected with the mono-nucleotide (A/T)_n_ accounting for 26.95% of the total. Similarly, 16,944 motif repeats were followed by the di-nucleotides (AG/CT)_n_ (11,867, 18.88%), (AT/AT)_n_ (5,827, 9.27%), and (AC/GT)_n_ (5,342, 8.50%). Moreover, the significant repeats among the tri-nucleotides were shown by (AAG/CTT)_n_ (4,333, 6.89%) and (AAT/ATT)_n_ (3,586, 5.70%) (see Supplementary Figure S6). Additionally, a total of 17,364 primers for each Unigene were also designed using Primer3 through the identified Unigene SSR sequences (see Supplementary Table S5). Furthermore, the plant disease resistance gene identification analysis was performed through the Plant Resistance Gene database (PRGdb)^49,50^. It revealed numerous disease-resistance genes in multiple species such as *Populus trichocarpa*, and *Brassica rapa* subsp. *Pekinensis*, and *Vitis vinifera* and more (see Supplementary Table S6).

#### Unigene expression

**Figure 3:**
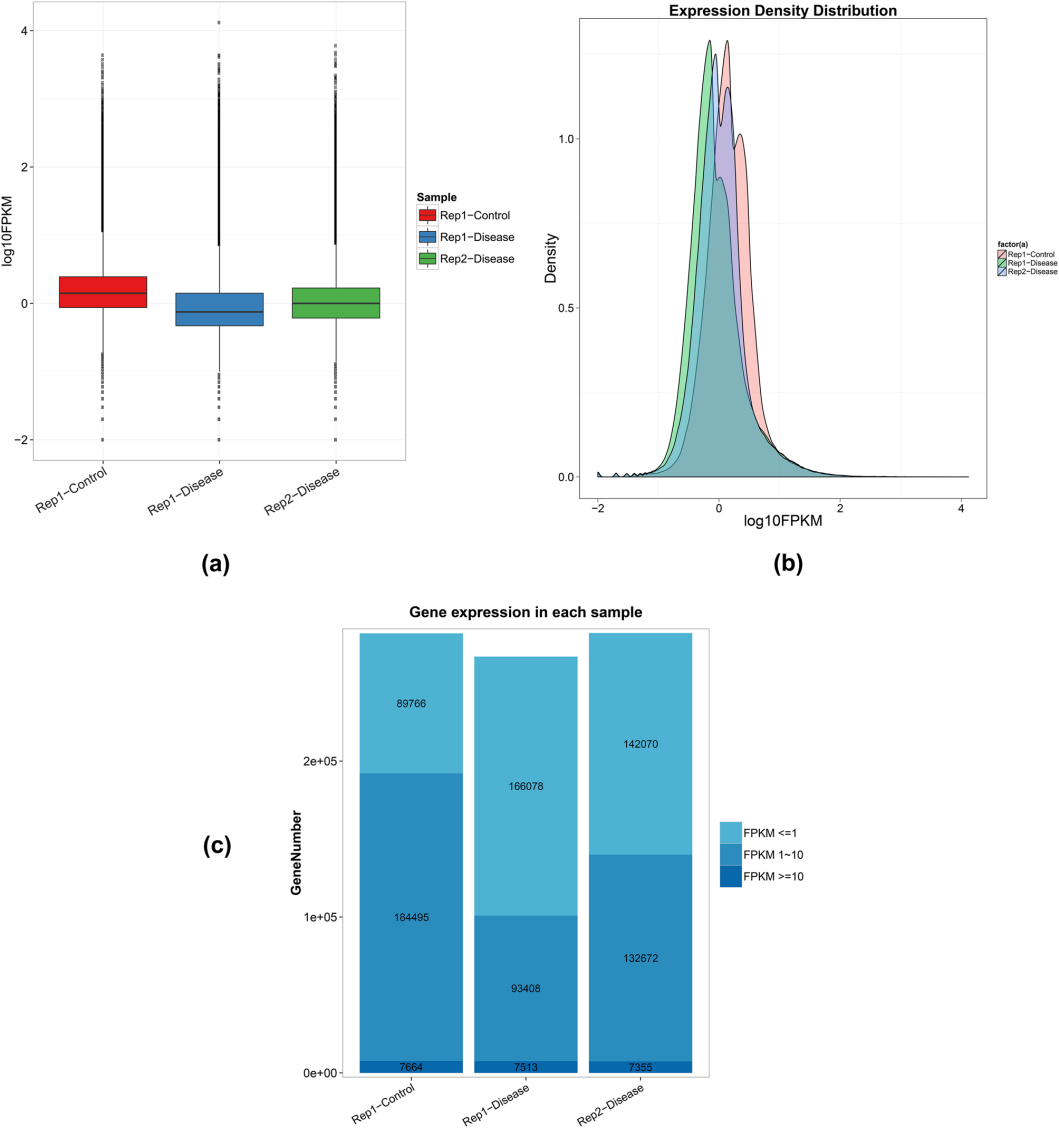
(**a**) Box plot of Gene expression. The X-axis represents the sample name, and the Y-axis is the log_10_FPKM value. (**b**) Expression Density Distribution. The X-axis represents the log_10_FPKM value, and the Y-axis represents the expression density, which means the ratio of gene amount under the specific expression level to the total number of expressed genes. (**c**) Gene expression distribution. The X-axis represents the sample name, and the Y-axis represents the gene amount. The depth of the color refers to different gene expression levels: FPKM <  = 1 means extremely low expression level, and 110 means high expression level.

To assess the expression level of the genes, Bowtie2^51^ was first used to assemble the clean reads into Unigenes as described before, and then RSEM^52^ was used to calculate the gene expression level (see Supplementary Table S1). The results of an all-sample alignment showed that for 18,142,156,800 bases, the number of total reads was 120,947,712, with 77,457,276 (64.04%) total mapped reads, and 16,761,366 (13.86%) unique reads. The distribution of Unigene expression level was assessed by illustrating the dispersion of FPKM expression datasets through Box-plots^53^ (Fig. 3a). In terms of dispersion and skewness, all the samples are almost in the same Inter Quartile Range (IQR) but vary in terms of their median. The deviation of the median between the control and disease samples showed different expression levels of the genes. Moreover, the genes in all the samples showed almost the same distribution of the FPKM expression outliers. The gene expression density graph with log_10_FPKM clearly shows the tendency of gene abundance changing with expression quantity. It also mirrors the concentration interval of relative gene expression in all samples (Fig. 3b). Comparatively, the Rep1-Control showed a slightly different second peak of expression density distribution which shows the difference in the level of relative gene expression. For a more intuitive representation of expression values, the intervals were created between different FPKM values (FPKM <  = 1, FPKM: 1–10, FPKM >  = 10), and the gene amount was calculated for each interval (Fig. 3c). Compared with Rep1-Control, for FPKM <  = 1, Rep1-Disease and Rep2-Disease had 1.85- and 1.58-times higher expression levels, respectively. For FPKM: 1 ~ 10, Rep1-Disease and Rep2-Disease had 1.98- and 1.39-times lower expression levels, respectively. Lastly, For the FPKM >  = 10, there were no significant differences between the control and disease samples. The higher and lower expression values at different FPKM intervals show the subsequent up and downregulation of genes.

### Differentially expressed genes (DEGs)

#### Detection

**Figure 4:**
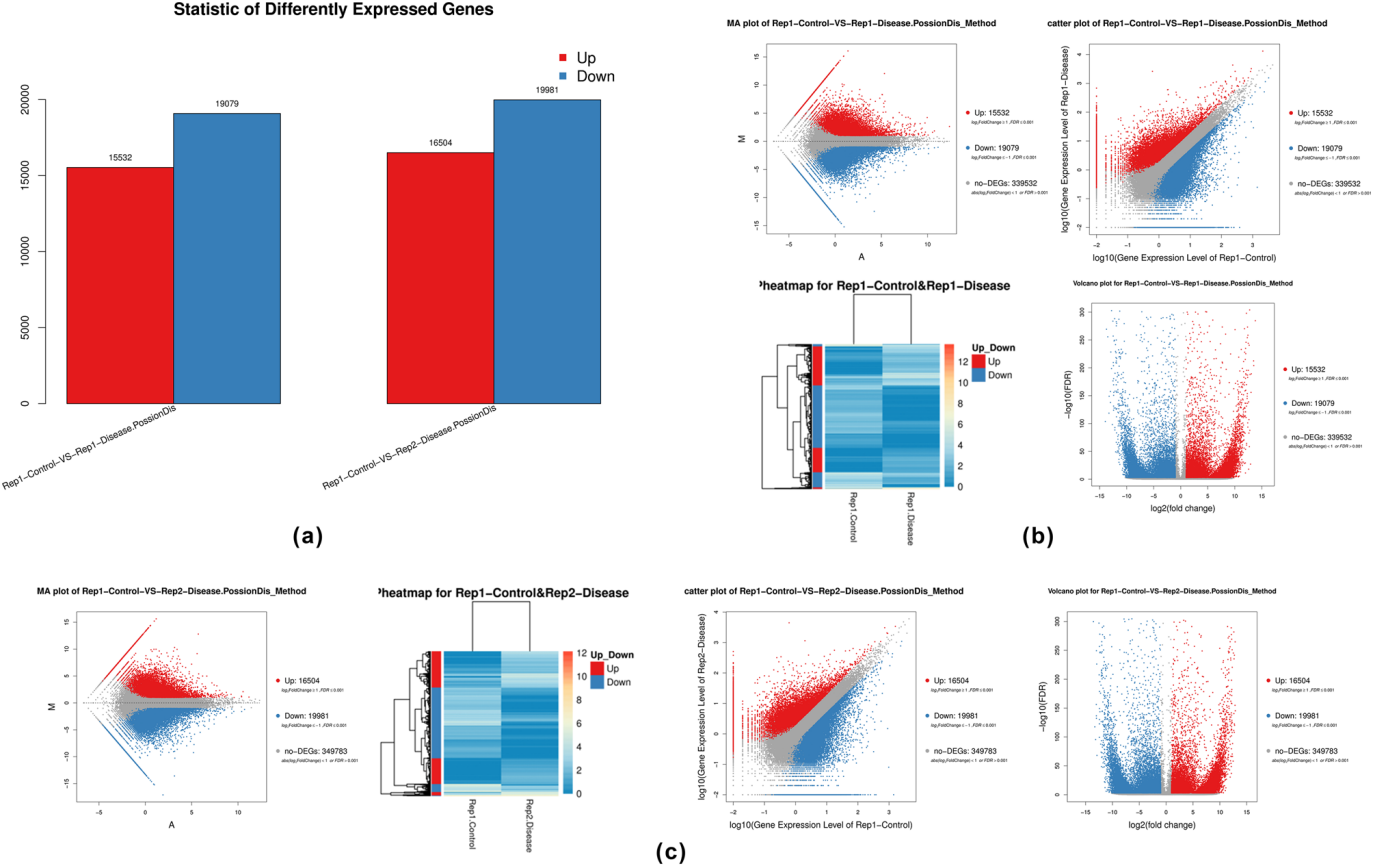
(**a**) Summary of DEGs. The X-axis represents the comparison functionality between each group and the Y-axis represents DEG numbers. The red color represents upregulated DEGs. The blue color represents downregulated DEGs. (**b, c**) MA plot. The X-axis represents value A (log_2_ transformed mean expression level) and the Y-axis represents value M (log_2_transformed fold change). Red dots represent upregulated DEG. Blue dots represent downregulated DEG. Black points represent non-DEGs. **Scatter plot.** XY-axis represents the log_10_ transformed gene expression level, the blue color represents the up-regulated genes, the red color represents the down-regulated genes, and the grey color represents the non-significant differential genes. Volcano plot. The X-axis represents -log_10_ transformed significance the Y-axis represents log_2_ transformed fold change. Red points represent upregulated DEG. Blue points represent downregulated DEG. Black points represent non-DEGs. Heatmap plot. The X-axis represents the sample name and the Y-axis represents DEGs. The dark color means a high expression level while the light color means a low expression level.

The Differentially Expressed Genes or DEGs can be identified based on their expression level between different samples. For this purpose, the Poisson distance correction or distribution algorithm^54,55^ was deployed using PoissonDis to detect DEGs. Rep1-Control sample was compared to both the Rep1-Disease and Rep2-Disease samples using log_2_FoldChange values of expression (see Supplementary File S2). In summary, the Rep1-Control vs. Rep1-Disease comparison group exhibited 15,532 up- and 19,079 down-regulated DEGs. Similarly, the Rep1-Control vs. Rep2-Disease group showed 16,504 up- and 19,981 down-regulated DEGs (Fig. 4a). This data has also been represented in terms of MA, scatter, volcano, and heatmap plots (Fig. 4b,c).

#### GO (gene ontology) analysis

**Figure 5:**
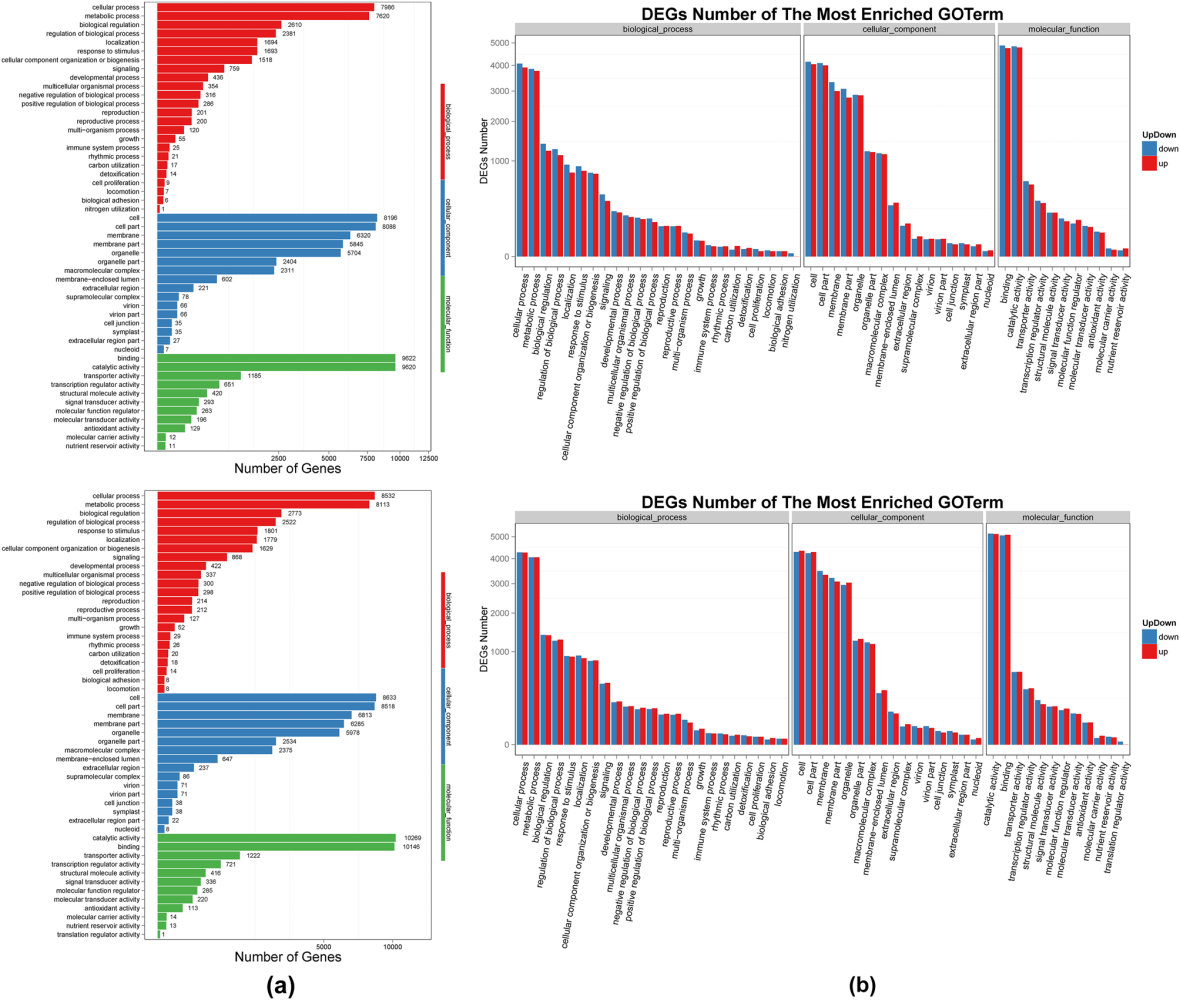
(**a**) GO classification of DEGs. The X-axis represents the number of DEG and the Y-axis represents the GO term. (**b**) GO classification of up-regulate and down-regulate **genes.** The X-axis represents the GO term and the Y-axis represents the amount of up/down-regulated genes.

The DEGs were classified according to GO functional enrichment terms into three subsequent categories: biological process, cellular component, and molecular function (see Supplementary File S3). In the Rep1-Control vs. Rep1-Disease group, the cellular and metabolic process GO terms were prominent in the biological process category. Similarly, the cell and cell part were prominent in the cellular component category, and binding and catalytic activity were prominent in the category of molecular function (Fig. 5a). The same trend of GO terms prominence was also observed in the Rep1-Control vs. Rep2-Disease group. The up- and down-regulated DEGs were also represented according to the classification of enriched GO terms and an almost equal distribution was observed in all the processes (Fig. 5b). Additionally, the GO functional enrichment DAGs (Directed Acyclic Graphs) in both the aforementioned groups of DEGs showed numerous significantly enriched pathways based on the calculated p-values.

#### Pathway and protein-interaction network analysis

**Figure 6:**
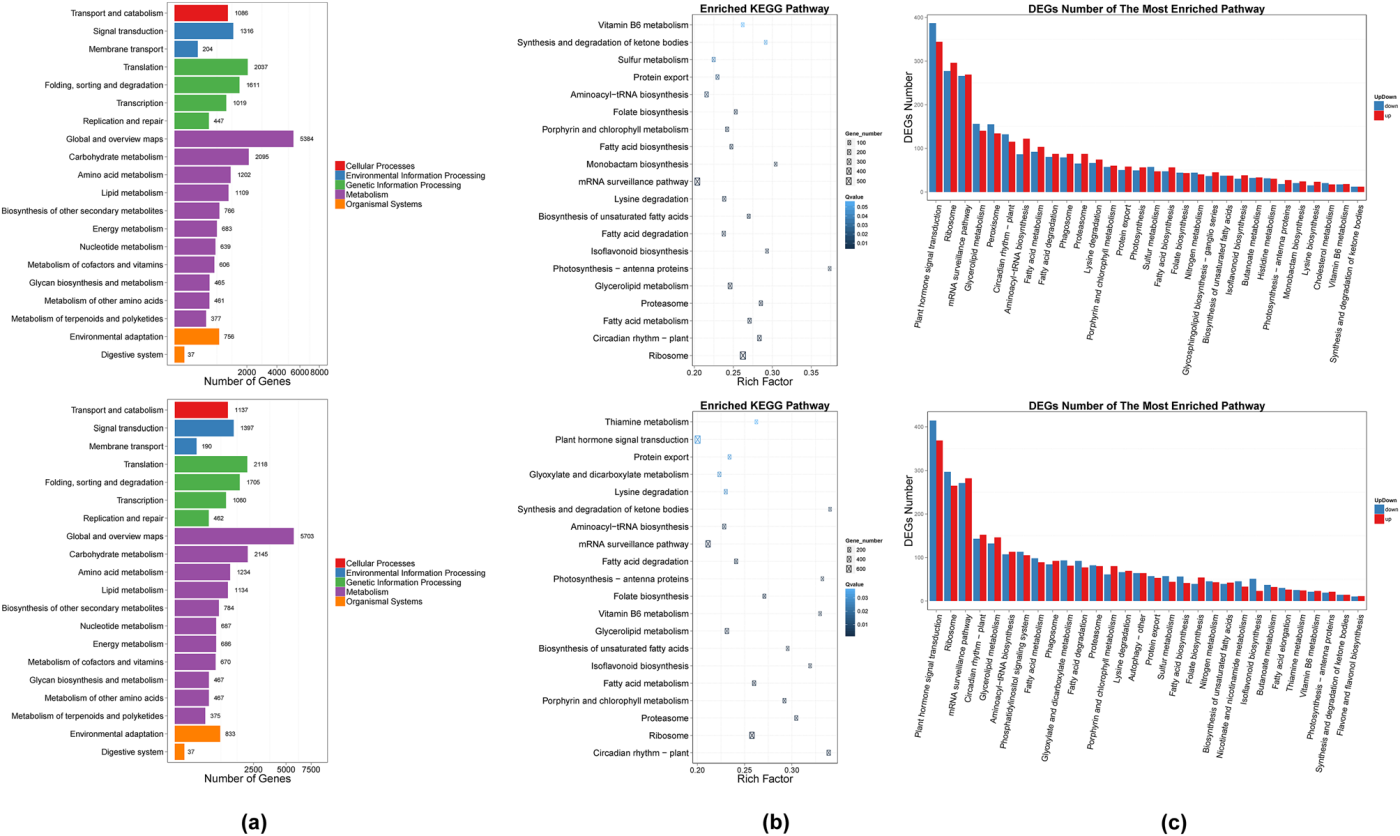
(**a**) Pathway classification of DEGs. The X-axis represents the number of DEG and the Y-axis represents the functional classification of KEGG. There are 7 branches for KEGG pathways: Cellular Processes, Environmental Information Processing, Genetic Information Processing, Human Disease (For animals only), Metabolism, Organismal Systems, and Drug Development. (**b**) Pathway functional enrichment of DEGs. The X-axis represents the enrichment factor and the Y-axis represents the pathway name. The color indicates the q-value (high: white, low: blue), and the lower q value indicates the more significant enrichment. Point size indicates the DEG number (The bigger dots refer to a larger amount). Rich Factor refers to the value of enrichment factor, which is the quotient of foreground value (the number of DEGs) and background value (total Gene amount). The larger the value, the more significant the enrichment. (**c**) Pathway functional enrichment results for up/down-regulation genes. The X-axis represents the terms of Pathway and the Y-axis represents the number of up/down-regulation genes.

KEGG^45^ database was used to classify the DEGs according to their functional enrichment and pathways. Like the pathway classification and distribution patterns of Unigenes, DEGs in both Rep1-Control vs. Rep1-Disease and Rep1-Control vs. Rep2-Disease group showed a similar trend with 5 individual clades (cellular processes, environmental information processing, genetic information processing, metabolism, and organismal systems) (Fig. 6a). The KEGG pathway enrichment is based on the intermittent rich factor and Q-value^56,57^. The Rep1-Control vs. Rep1-Disease group showed ‘Photosynthesis – antenna proteins’, Monobactum biosynthesis, and Isoflavonoid biosynthesis pathway as the major ones. Similarly, in Rep1-Control vs. Rep2-Disease group, ‘Circadian rhythm—plant’, Synthesis and degradation of ketone bodies, ‘Photosynthesis – antenna proteins’, Vitamin B6 metabolism, and Isoflavonoid biosynthesis pathway were the prominent ones (Fig. 6b). The KEGG pathway maps for each DEG give extensive detail about the underlying pathways and their mechanisms (see Supplementary File S4). Additionally, the enriched KEGG pathways were also represented in terms of both up and downregulated DEGs (Fig. 6c). The three major and significant pathways were Plant hormone signal transduction, Ribosome, and mRNA surveillance pathway. In these pathways, the first one had more down-regulated DEGs while the latter two had more up-regulated DEGs. The protein-interaction network for Rep1-Control vs. Rep1-Disease and Rep1-Control vs. Rep2-Disease group showed the up and downregulation of Unigenes along with the significance and intensity of the protein-interaction network on an individual gene level (see Supplementary File S5).

## Discussion

Shisham (*Dalbergia sissoo*) is a timber-producing deciduous tree species of vast economic importance. Its timber has been used in building furniture and other wood crafts in the Indian sub-continent. It has remained one of the pillars of its economy since old age. The ecological impact of *D. sissoo* comes from its involvement in the associated ways such as soil erosion control, carbon sequestration, water quality maintenance, ecosystem services, and genetic diversity preservation. It also has great medicinal importance in this region and therefore is of great cultural importance as well^58,59^. Even though it is an important tree species with enormous germplasm resources, there is a lack of genomic data under the biotic stress of Shisham dieback. This data is an essential prerequisite for ensuring survival, future breeding, and genetic improvement programs. The diversity of *D. sissoo* is also another endangered factor due to habitat loss and fragmentation, and it requires more attention from the international scientific community for proper preservation and conservation of the germplasm resources.

Fortunately, modern Next Generation Sequencing (NGS) technologies have enabled us to develop large datasets of genomic data for breeding and crop improvement programs both cost-effectively and in an timely-efficient manner^60,61^. In our study, we used the BGISEQ-500 sequencing platform to perform the transcriptome sequencing of a total RNA equimolar mixture isolated from the young leaf tissue samples of the *D. sissoo* tree. The output cDNA library was normalized to maximize the chances of finding less abundant mRNA and robust sampling of complex transcripts^62^. The significant overlapping in paired-end cDNA sequencing for tree species with no reference genome was a critical part of the assembly. In total, 18.14 Gb data was generated. After sequence read filtration treatment, 310,523,693 (96.89%) clean reads were obtained. These clean reads were further assembled using Trinity into 513,821 Unigenes for the reconstruction of reference genomes lacking non-model tree species^63^. The high quality and precision of assembly were confirmed through the N50 value of 1101 bp with an average length of 604 bp. Compared with a recent transcriptome assembly that utilized the Illumina® HiSeq 4000 sequencing platform, our BGISEQ-500-generated paired-end filtered reads were 5.2% more in number (96.89% vs. 91.7%)^64^. Overall, a robust and precise analysis pipeline was implemented to easily assess and report the transcriptome datasets and decipher the underlying molecular mechanisms and disease pathways.

The TransDecoder CDS prediction revealed that most of the Unigenes code for the ORFs of medium to long lengths. The presence of a respective amount of hits (38.99%) in the NR database shows that a significant number of CDS codes for proteins. The CDS that did not exhibit a hit might be very short in length, lack functionally conserved domain, or were part of non-coding RNAs^65^. In the functional annotation results from the NR database, it was revealed that *D. sissoo* shared maximum similarity with an herb named *Arachis ipaensis*. Both of them originate from the same subfamily of *Faboideae*, suggesting it might serve as a reference genome for *D. sissoo* and other closely related *Fabaceae* species in the future. The GO and KEGG functional term enrichment analysis placed the Unigenes in three categories *i.e*., biological process, cellular component, and molecular function. The major Unigene-enriched pathways include carbohydrate metabolism, translation, transport and catabolism, signal transduction, and environmental adaptation. The studies on the transcriptomes of other tree species duly support the above-mentioned findings^66–72^.

In terms of the generalized FPKM expression level of assembled Unigenes, the top three expressing Unigenes for Rep1-Control were CL9241.Contig10_All, CL20392.Contig2_All, and Unigene15134_All with functional pathway annotations in Ribulose bisphosphate carboxylase small chain 1, Chlorophyll a-b binding protein of LHCII type 1, and serine protease inhibitor-like precursor, respectively. These genes are involved in crucial photosynthetic functions and enzymatic processes^73–75^. There was a partially significant change in the expression of these genes in Rep1-Disease and Rep2-Disease suggesting their moderately associative nature with the biotic stress induced by *B. theobromae*. In Rep1-Disease, the top three expressing Unigenes were CL2816.Contig6_All, CL9241.Contig5_All, and CL33174.Contig2_All with functional pathway annotations in Trypsin inhibitor, Ribulose bisphosphate carboxylase small chain 1, and Photosystem I P700 chlorophyll an apoprotein A2, respectively. When compared to Rep1-Control, all three had a 6.22-, 18.74-, and 1.41-times higher expression level, respectively. These extreme changes in the expression level suggest that the aforementioned protein pathways might have undergone dynamic regulation changes in their functions in response to the biotic stress induced by *B. theobromae*. These functions include plant defense responses and regulation of photosynthetic processes^73,76–78^.

Similarly, in Rep2-Disease, the top three expressing Unigenes were CL9241.Contig10_All, CL33174.Contig2_All, and Unigene176088_All with functional pathway annotations in Ribulose bisphosphate carboxylase small chain 1, photosystem I P700 chlorophyll a apoprotein A2, and Serine protease inhibitor-like precursor. When compared to Rep1-Control, all three had a 1.38-, 1.63-, and 7165.29 times higher expression level, respectively. Aligning with the previous speculation, the first two pathway annotations have similar functions such as plant defense responses and regulation of photosynthetic processes^73,75–78^. However, the third extremely high expression (Unigene176088_All: 7165.29 times the expression of Rep1-Control) pathway annotation of serine protease inhibitor-like precursor has a diverse range of sub-classes and functions. These include extreme defense responses against pathogens by Bowman-Birk Inhibitors (BBI) and Kunitz-type inhibitors, and in abiotic stressors, plant growth, and development processes by Serine Protease Inhibitor-like Proteins (SPIs)^75,79–82^. All these findings suggest that all these genes dynamically change their functions through a significant increase in their expression level to cope with the biotic stress induced by *B. theobromae*.

In terms of the log_2_FoldChange expression level of identified DEGs, a significant up and downregulation of genes was observed when both the disease samples were grouped comparatively with the controlled sample. This speculates the dynamic change in the level of expression of different genes under the biotic stress induced by *B. theobromae*. For instance, in the Rep1-Control vs. Rep1-Disease group, the top three up-regulated DEGs were CL18436.Contig5_All, CL11636.Contig1_All, and CL34903.Contig2_All with functional pathway annotations in Aquaporin PIP-type 7a, Histone H3.3, and Kunitz trypsin inhibitor precursor, respectively. These protein pathways are involved in water transport across plasma membranes, defense responses against pathogens, and other abiotic stressors, respectively^79,80,83–86^. In the same group, the top 3 down-regulated DEGs were CL67374.Contig1_All, Unigene263_All, and CL24897.Contig1_All. All these have functional pathway annotations in tRNA-dihydrouridine (20) synthase [NAD(P) +]-like, Pentatricopeptide repeat-containing protein, and HIRA protein, respectively. These protein pathways are involved in response against abiotic stressors and regulation of gene expression^87–90^. Similarly, in the Rep1-Control vs. Rep2-Disease group, the top three up-regulated DEGs were CL8007.Contig2_All, CL30985.Contig9_All, and CL5191.Contig7_All. These have functional pathway annotations in SRC1-like protein, Isoflavone reductase homolog, and NADH dehydrogenase subunit 5. These protein pathways are involved in response against environmental stressors, pathogen defense, and plant growth and development processes, respectively^91–95^. In the same group, the top three down-regulated DEGs were CL39346.Contig3_All, CL7867.Contig15_All, and CL7253.Contig6_All. These have functional pathway annotations in 29 kDa (kilo-Dalton) ribonucleoprotein A, β-galactosidase 17, and Phosphatase 2C 79 protein, respectively. These protein pathways are involved in stress response, and plant growth and development processes^96–103^.

In summary, PIP-type 7a aquaporins have an active involvement in plant defense responses. They are mainly involved in the regulation of water transport across the plasma membrane and thus maintain the water balance of the cell. Due to this active involvement, PIP-type 7a aquaporins can be a potential improvement target to improve the resistance of *D. sissoo* against *B. theobromae* infection. Similarly, Histone H3.3, Kunitz trypsin inhibitor precursor, Pentatricopeptide repeat-containing protein, SRC1-like protein, Isoflavone reductase homolog, 29 kDa ribonucleoprotein A, NADH dehydrogenase subunit 5, β-galactosidase 17, and Phosphatase 2C 79 protein have shown strong association with plant–microbe interaction processes, plant-defense response, cellular expansion, elongation, division and proliferation control, enzyme, secondary metabolite, and hormonal regulations under disease stress, respectively.

DEGs were also identified using the Plant Resistance Gene (PRG) database^49,50^ and the assembled Unigenes. In Rep1-Control, the top three genes with the highest FPKM expression values were Unigene176148_All (*Medicago truncatula*), and CL44494.Contig2_All (*Medicago truncatula*), and Unigene6841_All (*Oryza sativa*). These have pathway annotations in Heat shock cognate protein 70–1, Disease resistance protein (TIR-NBS-LRR class) family, and RNI-like superfamily protein, respectively. These pathways are involved in cellular functions like stress responses, pathogen defense, and plant growth and development processes, respectively^104–109^. In Rep1-Disease, the top three genes with the highest FPKM expression values were CL44494.Contig2_All (*Medicago truncatula*), Unigene176148_All (*Medicago truncatula*), and CL58125.Contig2_All (*Glycine max*). These have pathway annotations in the Disease resistance protein (TIR-NBS-LRR class) family, Heat shock cognate protein 70–1, and pleiotropic drug resistance 12, respectively. These pathways are involved in functions like pathogen defense and stress responses^105–111^. Similarly, in Rep2-Disease, the top three genes with the highest FPKM expression values were Unigene123966_All (*Medicago truncatula*), Unigene177302_All (*Oryza sativa*), and CL44494.Contig2_All (*Medicago truncatula*). These have pathway annotations in Heat shock cognate protein 70–1, RNI-like superfamily protein, and Disease resistance protein (TIR-NBS-LRR class) family, respectively. Similar to Rep1-Disease, these protein pathways have functions like stress responses, pathogen defense, and plant growth and development, respectively^104–109^. Compared to Rep1-Control, there was a significant change in the expression level of the aforementioned genes which indicates the dynamic expression adjustments resulting due to the stress induced by *B. theobromae*.

In this study, we have identified Unigenes in the transcriptome of *D. sissoo* under normal and *B. theobromae*-induced stress. We analyzed their GO enrichment terms and KEGG functional pathway annotations, identified their SSR markers, and cross-matched all the Unigenes in 7 functional databases. The CDS and transcription factors were predicted along with expression density. Furthermore, we detected the DEGs, analyzed their GO terms, and KEGG pathways, mapped the protein–protein interaction networks, and curated the Plant Resistance Genes (PRGs) from the assembled Unigenes. Additionally, the FPKM and Fold Change gene expression of Unigenes, DEGs, and PRGs were also comparatively evaluated. On the whole, all this robust, precise, and valuable transcriptomic information will lay the foundations needed to ensure the survival of *D. sissoo* through crop improvement programs, genetic breeding, increased biodiversity, and preserved germplasm resources.

## Conclusions

Our study on the transcriptome of *D. sissoo* demonstrates how the stress from dieback disease induced by *B. theobromae* affects the level of gene expression. In terms of de novo transcriptome sequencing, we generated about 18.14 Gb bases in total and 310,523,693 bp clean reads. In total, 513,821 Unigenes were curated with an average length of 604 bp, an N50 of 1,101 bp, and a GC content of 39.95%. All the Unigenes were aligned with 7 functional databases and annotations were given as follows: 200,355 (NR: 38.99%), 164,973 (NT: 32.11%), 123,733 (Swissprot: 24.08%), 142,580 (KOG: 27.75%), 139,588 (KEGG: 27.17%), 99,752 (GO: 19.41%), and 137,281 (InterPro: 26.72%). The GO term enrichment and KEGG functional pathway analysis shed light on the important underlying stress response mechanisms. Moreover, 115,762 CDS and 4,673 TF coding genes were also identified along with 62,863 SSR markers distributed on 51,508 Unigenes. The significant up and downregulation of 16,018 up- and 19,530 down-regulated DEGs showed the gene expression shifts under the biotic stress conditions. The evaluation of change in the relative expression levels in the case of both the DEGs and 9,230 identified PRGs hints at the dynamic shift in the underlying gene expression levels in response to disease stress. Our primary objective has been the expansion of the transcriptomic resource library accessible for *D. sissoo*. We have achieved this goal by incorporating high-quality transcriptome sequencing data, specifically gathered under the stress of *B. theobromae* infection. The identification of potential DEGs carries significant implications for various disease-related processes, such as biomarker discovery, comprehension of underlying pathway-mediated biological phenomena, disease categorization, and subtyping. All this will be made possible by the generated gene expression datasets highlighting up- and down-regulated genes. The multitude of PRGs, SSR markers, and Unigenes, when combined with genomic data, collectively contributes to the survival of *D. sissoo* under environmental stressors, preservation of germplasms, and the facilitation of future hybridization and breeding initiatives.

## Material and methods

### Plant materials preparation and RNA extraction

The fungus *B. theobromae* was isolated from the confirmed sources and cultured on BAM Media M127: Potato Dextrose Agar (PDA) according to established protocols^112^. The culture was examined microscopically to assess the viability of the fungus through septation incidence and the shape of spores. After proper assessment, the spore solution was prepared in sterile water and the inoculation in the stem-base portion of greenhouse-grown one-year-old *D. sissoo* vegetative plantations was performed according to standard protocols^113^. The plantations were grown at a temperature of 25 °C, 70% humidity, a light intensity of 350 µmol m^−2^ s^−1^ Photosynthetically Active Radiation (PAR), and with a 12-h light and 12-h dark photoperiod. After successful inoculation, three leaf samples were isolated after 20 days^114,115^, one from the control and two from the diseased one^116–118^. The leaf samples were enclosed in desiccated plastic bags and stored at − 30 °C for subsequent extraction. Vegetative plantations of *D. sissoo* were provided by the Punjab Forestry Research Institute (PFRI), Gatwala, Faisalabad. RNA (Ribonucleic acid) was isolated from the three young leaf samples using an RNA Purification Kit according to the manufacturer’s instructions (Thermo Fisher Scientific – Waltham, Massachusetts, United States). After extraction, the concentration and quality of RNA for cDNA synthesis were accurately determined using NanoDrop™ 8000 Spectrophotometer (Thermo Fisher Scientific – Waltham, Massachusetts, United States), Agilent 2100 Bioanalyzer system, and Agilent RNA 6000 Nano Kit (Agilent Technologies – Santa Clara, California, United States) and finally, it was stored at − 80 °C.

### Sequence read filtering, BGISEQ-500 transcriptome sequencing, and de novo assembly

Extracted RNA from three young leaf samples was used for cDNA synthesis and transcriptome sequencing through the BGISEQ-500 sequencing platform at BGI Genomics Co., Ltd., Shenzhen, Guangdong, China. This sequencing platform has a Paired-End (PE) read length capacity of 100 bp. The raw generated reads were subjected to a filter treatment by implementing strict principles and protocols^119,120^. After filtration, the ‘clean reads’ are stored in FASTQ format for further analysis^121^. Trinity v2.0.6 was utilized (Parameters: –min_contig_length 150 –CPU 8 –min_kmer_cov 3 –min_glue 3 –bfly_opts '-V 5 –edge-thr = 0.1 –stderr ') to carry out the assembly of clean reads with removed PCR duplications to improve efficiency and Tgicl v2.0.6 was used (Parameters: -l 40 -c 10 -v 25 -O '-repeat_stringency 0.95 -minmatch 35 -minscore 35') to cluster the large transcripts to Unigenes^42,122–124^. Trinity partitions the sequence data using its independent three modules *i.e*., Inchworm, Chrysalis, and Butterfly into many individual de Bruijn graphs. The de Bruijn graphs were used for the representation of the transcriptional complexity of a gene or locus and then Trinity processes each graph simultaneously and independently to extract splicing isoforms of full length^125^. These splicing isoforms are then used to sort out transcripts derived from paralogous genes. The final Unigenes were assembled using Tgicl gene family clustering for each sample for further downstream analysis^126,127^. When several Unigenes have a similarity of more than 70%, they are placed in one unified cluster while the remaining clustered Unigenes are placed as singletons. Furthermore, the RNA-seq transcripts used here have already been submitted to Sequence Read Archive or SRA (https://www.ncbi.nlm.nih.gov/sra) and Gene Expression Omnibus or GEO datasets (https://www.ncbi.nlm.nih.gov/geo/) of the National Center for Biotechnology Information or NCBI (https://www.ncbi.nlm.nih.gov/) under GEO accession ID: GSE220535 and BioProject ID: PRJNA910142. Their sample-wise accession number is GSM6806574 (Leaf Control R1), GSM6806575 (Leaf Treatment R1), and GSM6806576 (Leaf Treatment R2) with SRA IDs SRX18528739 (Rep1—Control), SRX18538740 (Rep1—Treatment), and SRX18538741 (Rep2—Treatment)^128,129^.

### Functional annotation of Unigenes

The curated Unigenes were analyzed by comparing and searching against public functional databases such as NT (The Nucleotide database) (ftp://ftp.ncbi.nlm.nih.gov/blast/db), NR (The Protein database) (ftp://ftp.ncbi.nlm.nih.gov/blast/db), GO (Gene Ontology) (http://geneontology.org), KOG (EuKaryotic Orthologous Groups) (ftp://ftp.ncbi.nih.gov/pub/COG/KOG), KEGG (Kyoto Encyclopedia of Genes and Genomes) (http://www.genome.jp/kegg), SwissProt (http://ftp.ebi.ac.uk/pub/databases/swissprot) and InterPro (http://www.ebi.ac.uk/interpro)^43,45,130,131^. Unigenes were aligned using BLAST-N v2.2.23 (http://blast.ncbi.nlm.nih.gov/Blast.cgi), BLAST-X v2.2.23 (http://blast.ncbi.nlm.nih.gov/Blast.cgi), Diamond v0.8.31 (https://github.com/bbuchfink/diamond), Blast2GO v2.5.0 (https://www.blast2go.com), InterProScan5 v5.11–51.0 (https://code.google.com/p/interproscan/wiki/Introduction)^44,47,132,133^. All the software tools were used at their default set perameters. Candidate coding areas were curated for Coding sequences or CDs using TransDecoder v3.0.1 (https://transdecoder.github.io). The longest Open Reading Frames or ORFs were extracted and then the coding regions were predicted using HMMscan and Swiss-Prot using BLAST search for protein homologous sequences in Pfam^48,134^. For the prediction of Unigene Transcription Factors (TFs), each Unigene was exploited using getorf version: EMBOSS: 6.5.7.0 (Parameters: -minsize 150) (http://genome.csdb.cn/cgi-bin/emboss/help/getorf)^135^. TFs were aligned to ORFs using hmmsearch v3.0 (http://hmmer.org) at default parameters and the guidelines provided by PlmTFDB (http://plntfdb.bio.uni-potsdam.de/v3.0/)^136^.

### Unigene SSR identification, expression, and plant disease resistance gene identification

MISA v1.0 (Parameters: 1–12,2–6,3–5,4–5,5–4,6–4 100 150) (http://pgrc.ipk-gatersleben.de/misa) was used to identify SSRs among Unigenes and then Primer3 v2.2.2 (http://bioinfo.ut.ee/primer3) was used to design primers for each identified SSR at default parameters^137,138^. The clean reads were mapped and aligned with the identified Unigenes using Bowtie2 v2.2.5 (Parameters: -q –phred64 –sensitive –dpad 0 –gbar 99,999,999 –mp 1,1 –np 1 –score-min L,0,-0.1 -I 1 -X 1000 –nomixed –no-discordant -p 1 -k 200) (http://bowtie-bio.sourceforge.net/Bowtie2/index.shtml) and then the level of gene expression was calculated with RSEM v1.2.12 (http://deweylab.biostat.wisc.edu/RSEM) in FPKM (Fragments Per Kilobase of transcript per Million mapped reads)^51,52,139^. The hierarchal clustering was performed using its hculust function at default parameters^140^. Finally, plant disease resistance genes were detected using Diamond (BLAST) on the identified Unigenes in the PRG database v2.0 (http://prgdb.crg.eu/)^49,50^. Potential disease resistance genes were finalized by evaluating the query coverage and identity values.

### Detection, GO, KEGG, and protein-interaction network analysis of differentially expressed genes (DEGs)

PoissonDis (Parameters: Fold Change >  = 2.00 and FDR <  = 0.001) was used to detect DEGs based on the Poisson distribution^54,55,141,142^. The results of PoissonDis were visualized using a comparative histogram, MA^143–145^, scatter^146^, volcano^147,148^, and heatmap plots^149^. GO (Gene Ontology) annotations and the official classification were used to classify the DEGs and functional enrichment was checked using phyper, a function of R. The p-value was calculated using the hypergeometric test and the False Discovery Rate (FDR) was then calculated for each *p* value^56,141,150^. The GO terms that have an FDR value less than 0.01 were defined as significantly enriched^43,151^. The KEGG annotation results were also used to classify DEGs according to official classification. phyper was used again to check the significant functional enrichment with the abovementioned hypergeometric p-value and FDR calculations^45^. To analyze the protein–protein interaction network, the DEGs were mapped to the STRING database v10.0 (http://string-db.org/)^152^.

### Supplementary Information

Supplementary Information 1.

Supplementary Information 2.

Supplementary Information 3.

Supplementary Information 4.

Supplementary Information 5.

Supplementary Table S1.

Supplementary Table S2.

Supplementary Table S3.

Supplementary Table S4.

Supplementary Table S5.

Supplementary Table S6.

Supplementary Figure S1.

Supplementary Figure S2.

Supplementary Figure S3.

## Data Availability

The raw sequencing data and assembled transcriptome generated in this study have been deposited in the Sequence Read Archive or SRA (https://www.ncbi.nlm.nih.gov/sra) and Gene Expression Omnibus or GEO datasets (https://www.ncbi.nlm.nih.gov/geo/) of the National Center for Biotechnology Information or NCBI (https://www.ncbi.nlm.nih.gov/) under GEO accession ID: GSE220535 and BioProject ID: PRJNA910142. Their accession number is GSM6806574 (Leaf Control R1), GSM6806575 (Leaf Treatment R1), and GSM6806576 (Leaf Treatment R2) with SRA IDs SRX18528739 (Rep1—Control), SRX18538740 (Rep1—Treatment), and SRX18538741 (Rep2—Treatment). Researchers can access the data and associated metadata for further analysis and validation. Detailed information on data retrieval and processing can be found in the Materials and Methods section of this paper.
