# Supplementary material for: De novo transcriptome assembly of Dalbergia sissoo Roxb. (Fabaceae) under Botryodiplodia theobromae-induced dieback disease
Source: Sci Rep. 2023 Nov 22;13:20503. doi: 10.1038/s41598-023-45982-8 (PMC10665356; doi:10.1038/s41598-023-45982-8)
Supplement: Supplementary file 1 — Supplementary Information 1. [file 41598_2023_45982_MOESM1_ESM.pdf]

Supplementary File S1: Dataset of pathway maps in all the Unigenes.

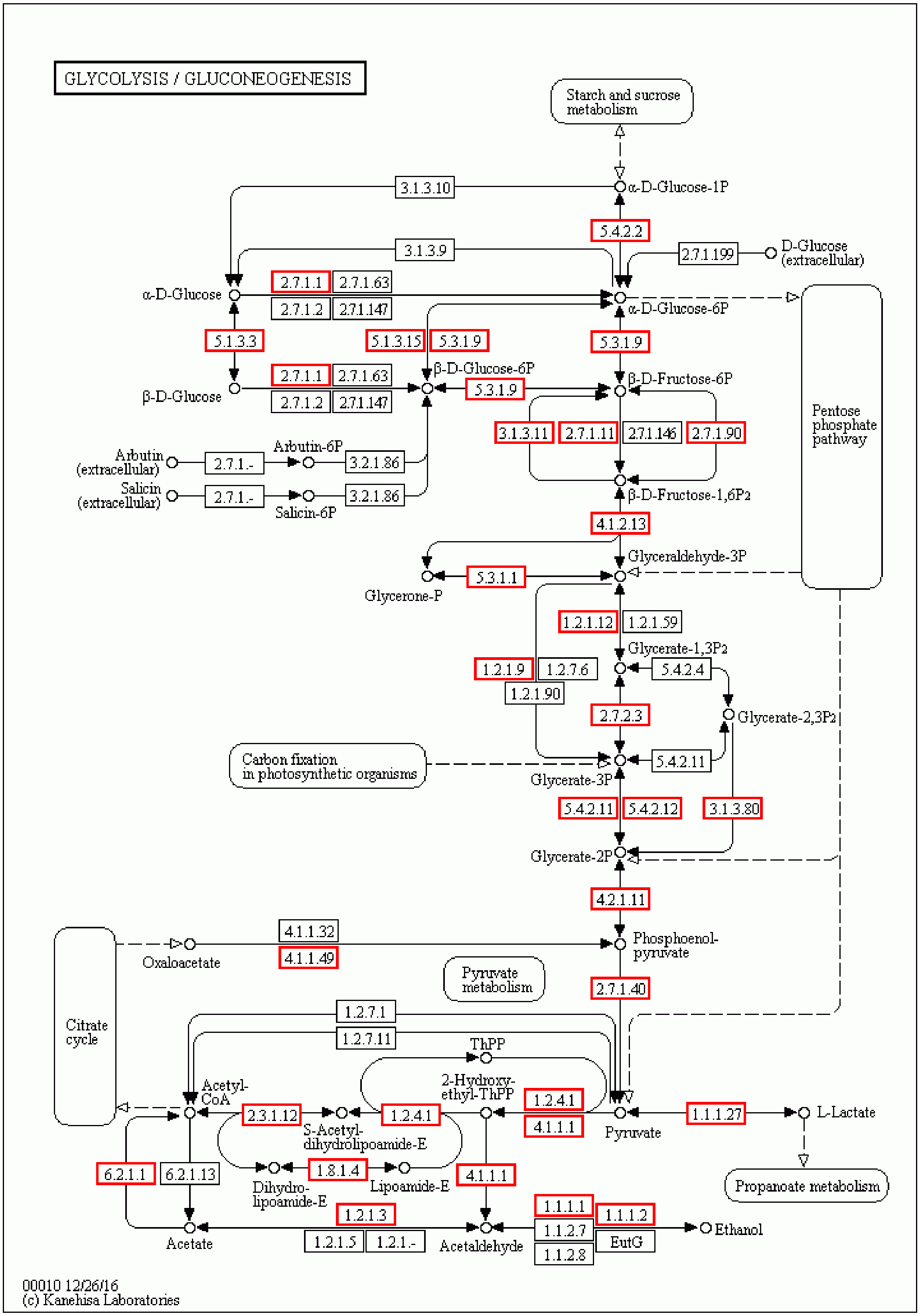

GLYCOLYSIS / GLUCONEOGENESIS
Starch and sucrose metabolism
α-D-Glucose-1P
3.1.3.10
5.4.2.2
3.1.3.9
2.7.1.199
D-Glucose (extracellular)
α-D-Glucose
2.7.1.1
2.7.1.63
2.7.1.2
2.7.1.147
α-D-Glucose-6P
5.1.3.3
5.1.3.15
5.3.1.9
5.3.1.9
β-D-Glucose-6P
β-D-Fructose-6P
β-D-Glucose
2.7.1.1
2.7.1.63
2.7.1.2
2.7.1.147
5.3.1.9
3.1.3.11
2.7.1.11
2.7.1.146
2.7.1.90
Pentose phosphate pathway
Arbutin (extracellular)
2.7.1.-
Arbutin-6P
3.2.1.86
Salicin (extracellular)
2.7.1.-
Salicin-6P
3.2.1.86
β-D-Fructose-1,6P2
4.1.2.13
Glyceraldehyde-3P
5.3.1.1
Glycerone-P
1.2.1.12
1.2.1.59
Glycerate-1,3P2
1.2.1.9
1.2.7.6
5.4.2.4
1.2.1.90
2.7.2.3
Glycerate-2,3P2
Carbon fixation in photosynthetic organisms
5.4.2.11
Glycerate-3P
5.4.2.11
5.4.2.12
3.1.3.80
Glycerate-2P
4.2.1.11
4.1.1.32
4.1.1.49
Phosphoenol-pyruvate
Oxaloacetate
Citrate cycle
Pyruvate metabolism
2.7.1.40
1.2.7.1
1.2.7.11
ThPP
Acetyl-CoA
2-Hydroxy-ethyl-ThPP
1.2.4.1
2.3.1.12
1.2.4.1
4.1.1.1
Pyruvate
1.1.1.27
L-Lactate
S-Acetyl-dihydrolipoamide-E
6.2.1.1
6.2.1.13
Dihydro-lipoamide-E
1.8.1.4
Lipoamide-E
4.1.1.1
Propanoate metabolism
1.2.1.3
1.1.1.1
1.1.1.2
Ethanol
1.2.1.5
1.2.1.-
1.1.2.7
EutG
Acetate
Acetaldehyde
1.1.2.8
00010 12/26/16
(c) Kanehisa Laboratories

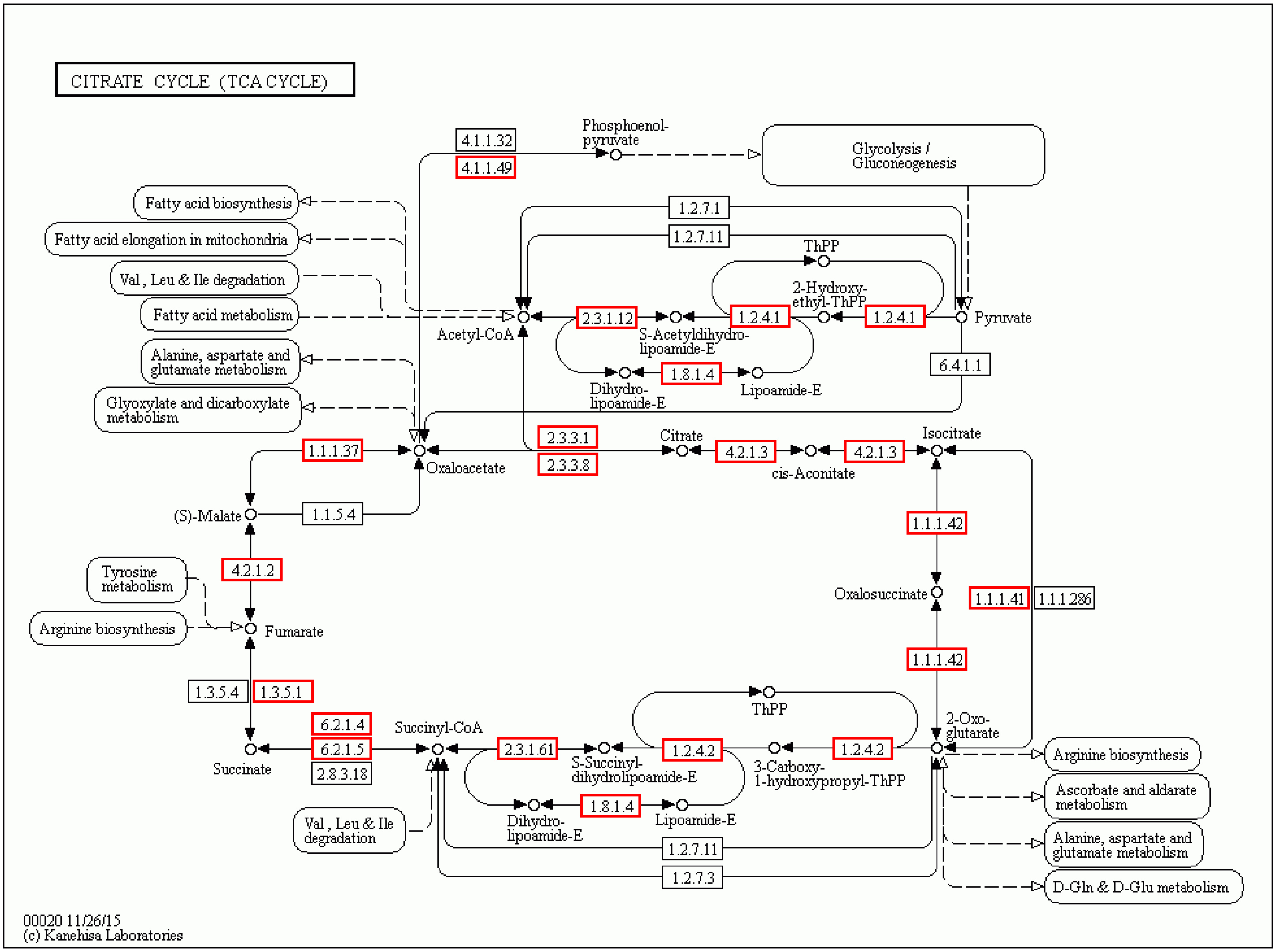
CITRATE CYCLE (TCA CYCLE)
Phosphoenol-pyruvate
Glycolysis / Gluconeogenesis
4.1.1.32
4.1.1.49
Fatty acid biosynthesis
Fatty acid elongation in mitochondria
Val, Leu & Ile degradation
Fatty acid metabolism
Alanine, aspartate and glutamate metabolism
Glyoxylate and dicarboxylate metabolism
1.2.7.1
1.2.7.11
ThPP
2-Hydroxy-ethyl-ThPP
Acetyl-CoA
2.3.1.12
S-Acetyldihydro-lipoamide-E
1.2.4.1
1.2.4.1
Pyruvate
1.8.1.4
Dihydro-lipoamide-E
Lipoamide-E
6.4.1.1
1.1.1.37
Oxaloacetate
2.3.3.1
2.3.3.8
Citrate
4.2.1.3
cis-Aconitate
4.2.1.3
Isocitrate
(S)-Malate
1.1.5.4
1.1.1.42
4.2.1.2
Oxalosuccinate
1.1.1.41
1.1.1.286
Tyrosine metabolism
Arginine biosynthesis
Fumarate
1.1.1.42
1.3.5.4
1.3.5.1
ThPP
6.2.1.4
Succinyl-CoA
2-Oxo-glutarate
6.2.1.5
2.3.1.61
1.2.4.2
1.2.4.2
Arginine biosynthesis
Succinate
2.8.3.18
S-Succinyl-dihydrolipoamide-E
3-Carboxy-1-hydroxypropyl-ThPP
Ascorbate and aldarate metabolism
Val, Leu & Ile degradation
1.8.1.4
Dihydro-lipoamide-E
Lipoamide-E
Alanine, aspartate and glutamate metabolism
1.2.7.11
1.2.7.3
D-Gln & D-Glu metabolism
00020 11/26/15
(c) Kanehisa Laboratories

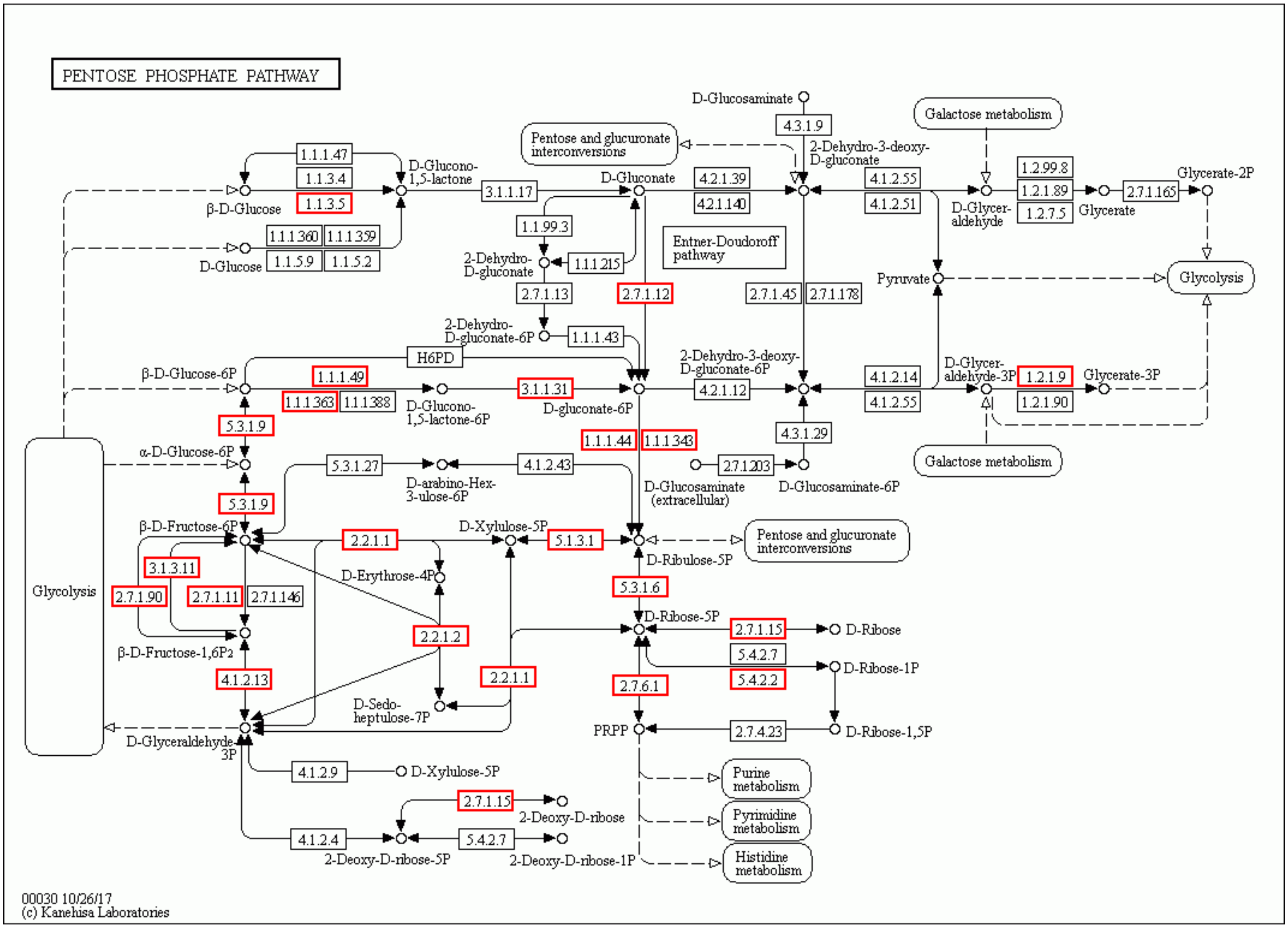
PENTOSE PHOSPHATE PATHWAY
D-Glucosaminate
4.3.1.9
Galactose metabolism
Pentose and glucuronate interconversions
2-Dehydro-3-deoxy-D-gluconate
1.1.1.47
1.1.3.4
1.1.3.5
D-Glucono-1,5-lactone
3.1.1.17
D-Gluconate
4.2.1.39
4.2.1.140
4.1.2.55
4.1.2.51
1.2.99.8
1.2.1.89
1.2.7.5
Glycerate-2P
2.7.1.165
Glycerate
D-Glyceraldehyde
β-D-Glucose
1.1.1.360
1.1.1.359
D-Glucose
1.1.5.9
1.1.5.2
1.1.99.3
2-Dehydro-D-gluconate
1.1.1.215
Entner-Doudoroff pathway
Pyruvate
Glycolysis
2.7.1.13
2.7.1.12
2.7.1.45
2.7.1.178
2-Dehydro-D-gluconate-6P
1.1.1.43
H6PD
2-Dehydro-3-deoxy-D-gluconate-6P
β-D-Glucose-6P
1.1.1.49
1.1.1.363
1.1.1.388
D-Glucono-1,5-lactone-6P
3.1.1.31
D-gluconate-6P
4.2.1.12
4.1.2.14
4.1.2.55
D-Glyceraldehyde-3P
1.2.1.9
1.2.1.90
Glycerate-3P
5.3.1.9
4.3.1.29
1.1.1.44
1.1.1.343
Galactose metabolism
α-D-Glucose-6P
5.3.1.27
D-arabino-Hex-3-ulose-6P
4.1.2.43
2.7.1.203
D-Glucosaminate (extracellular)
D-Glucosamínate-6P
5.3.1.9
β-D-Fructose-6P
2.2.1.1
D-Xylulose-5P
5.1.3.1
Pentose and glucuronate interconversions
D-Ribulose-5P
3.1.3.11
D-Erythrose-4P
Glycolysis
2.7.1.90
2.7.1.11
2.7.1.146
5.3.1.6
D-Ribose-5P
2.7.1.15
D-Ribose
2.2.1.2
5.4.2.7
β-D-Fructose-1,6P2
D-Ribose-1P
4.1.2.13
2.2.1.1
2.7.6.1
5.4.2.2
D-Sedo-heptulose-7P
PRPP
2.7.4.23
D-Ribose-1,5P
D-Glyceraldehyde-3P
4.1.2.9
D-Xylulose-5P
Purine metabolism
2.7.1.15
2-Deoxy-D-ribose
Pyrimidine metabolism
4.1.2.4
5.4.2.7
2-Deoxy-D-ribose-5P
2-Deoxy-D-ribose-1P
Histidine metabolism
00030 10/26/17
(c) Kanehisa Laboratories

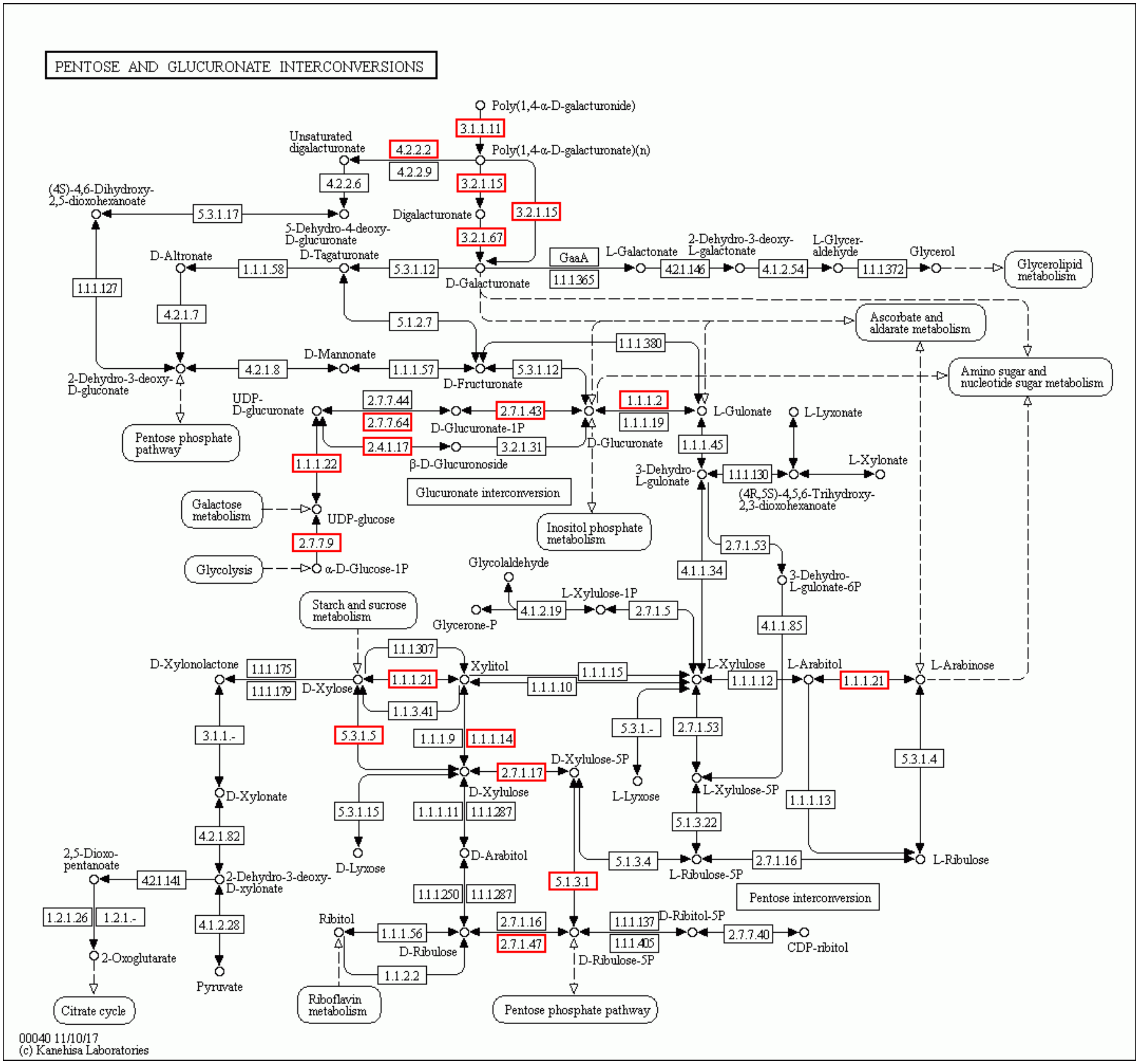

PENTOSE AND GLUCURONATE INTERCONVERSIONS
Poly(1,4-α-D-galacturonide)
3.1.1.11
Poly(1,4-α-D-galacturonate)(n)
Unsaturated digalacturonate
4.2.2.2
4.2.2.9
4.2.2.6
3.2.1.15
3.2.1.15
Digalacturonate
3.2.1.67
(4S)-4,6-Dihydroxy-2,5-dioxohexanoate
5.3.1.17
5-Dehydro-4-deoxy-D-glucuronate
D-Altronate
1.1.1.58
D-Tagaturonate
5.3.1.12
D-Galacturonate
GaaA
1.1.1.365
L-Galactonate
4.2.1.146
2-Dehydro-3-deoxy-L-galactonate
4.1.2.54
L-Glyceraldehyde
1.1.1.372
Glycerol
Glycerolipid metabolism
1.1.1.127
4.2.1.7
5.1.2.7
Ascorbate and aldarate metabolism
1.1.1.380
2-Dehydro-3-deoxy-D-gluconate
4.2.1.8
D-Mannonate
1.1.1.57
D-Fructuronate
5.3.1.12
Amino sugar and nucleotide sugar metabolism
UDP-D-glucuronate
2.7.7.44
2.7.7.64
2.7.1.43
D-Glucuronate-1P
1.1.1.2
1.1.1.19
L-Gulonate
L-Lyxonate
Pentose phosphate pathway
2.4.1.17
3.2.1.31
β-D-Glucuronoside
D-Glucuronate
1.1.1.45
1.1.1.22
3-Dehydro-L-gulonate
1.1.1.130
L-Xylonate
(4R,5S)-4,5,6-Trihydroxy-2,3-dioxohexanoate
Glucuronate interconversion
Galactose metabolism
UDP-glucose
Inositol phosphate metabolism
2.7.7.9
2.7.1.53
Glycolysis
α-D-Glucose-1P
Glycolaldehyde
4.1.1.34
3-Dehydro-L-gulonate-6P
L-Xylulose-1P
4.1.2.19
2.7.1.5
Starch and sucrose metabolism
Glycerone-P
4.1.1.85
1.1.1.307
Xylitol
L-Xylulose
L-Arabitol
L-Arabinose
D-Xylonolactone
1.1.1.175
1.1.1.179
D-Xylose
1.1.1.21
1.1.1.15
1.1.1.10
1.1.1.12
1.1.1.21
1.1.3.41
3.1.1.-
5.3.1.5
1.1.1.9
1.1.1.14
5.3.1.-
2.7.1.53
5.3.1.4
2.7.1.17
D-Xylulose-5P
D-Xylulose
L-Lyxose
L-Xylulose-5P
D-Xylonate
5.3.1.15
1.1.1.11
1.1.1.287
1.1.1.13
4.2.1.82
5.1.3.22
D-Arabitol
2,5-Dioxopentanoate
4.2.1.141
2-Dehydro-3-deoxy-D-xylonate
D-Lyxose
5.1.3.4
L-Ribulose-5P
2.7.1.16
L-Ribulose
5.1.3.1
1.1.1.250
1.1.1.287
Pentose interconversion
1.2.1.26
1.2.1.-
4.1.2.28
Ribitol
1.1.1.56
2.7.1.16
1.1.1.137
D-Ribitol-5P
2.7.7.40
D-Ribulose
2.7.1.47
1.1.1.405
CDP-ribitol
2-Oxoglutarate
D-Ribulose-5P
Pyruvate
1.1.2.2
Citrate cycle
Riboflavin metabolism
Pentose phosphate pathway
00040 11/10/17
(c) Kanehisa Laboratories

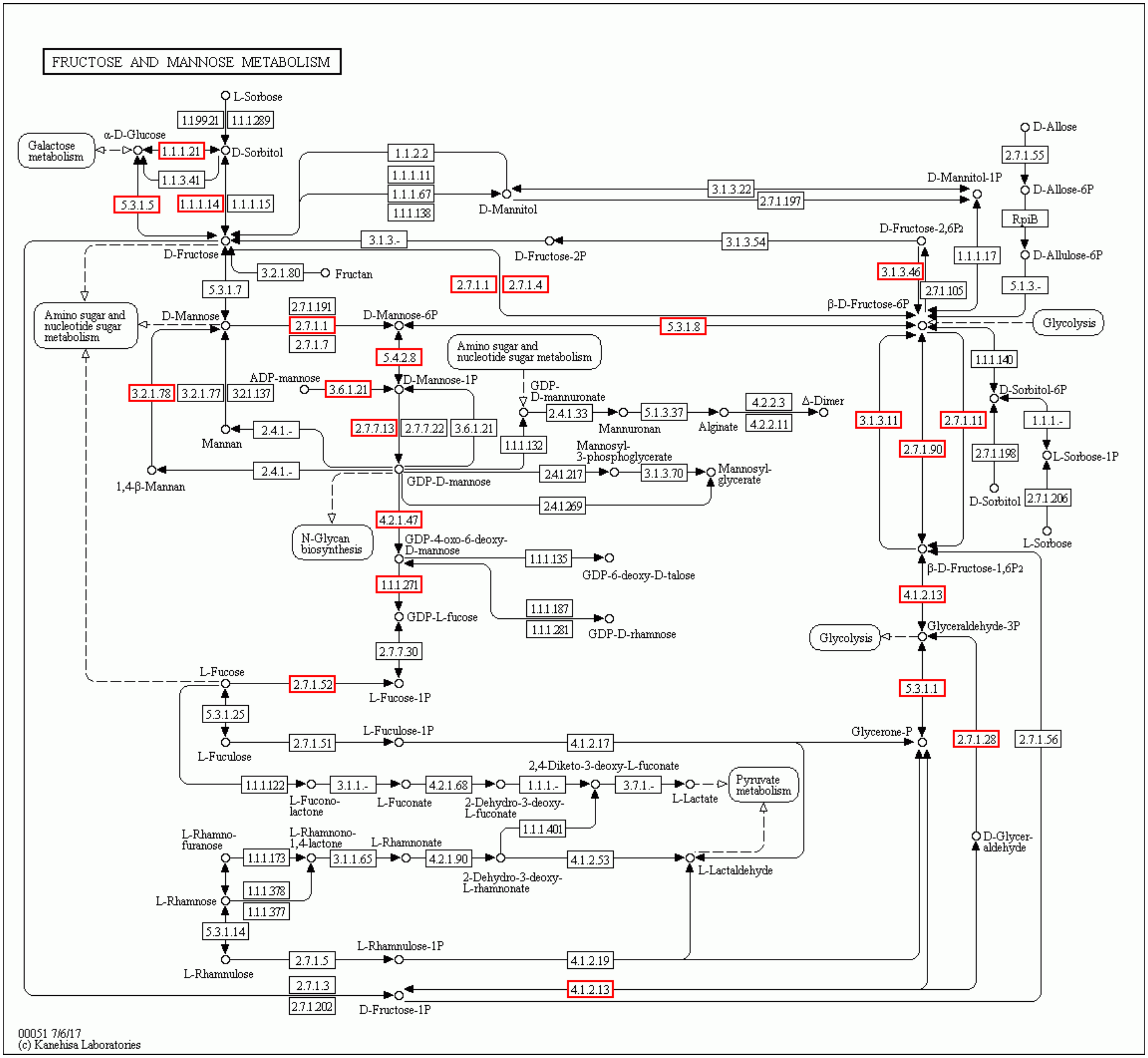
FRUCTOSE AND MANNOSE METABOLISM
L-Sorbose
1.1.99.21
1.1.1.289
α-D-Glucose
Galactose metabolism
1.1.1.21
D-Sorbitol
1.1.3.41
5.3.1.5
1.1.1.14
1.1.1.15
1.1.2.2
1.1.1.11
1.1.1.67
1.1.1.138
D-Mannitol
3.1.3.22
2.7.1.197
D-Mannitol-1P
D-Allose
2.7.1.55
D-Allose-6P
RpiB
D-Allulose-6P
1.1.1.17
5.1.3.-
3.1.3.-
D-Fructose
D-Fructose-2P
3.1.3.54
D-Fructose-2,6P2
3.1.3.46
2.7.1.105
3.2.1.80
Fructan
5.3.1.7
2.7.1.1
2.7.1.4
β-D-Fructose-6P
Glycolysis
Amino sugar and nucleotide sugar metabolism
D-Mannose
2.7.1.191
2.7.1.1
2.7.1.7
D-Mannose-6P
5.3.1.8
5.4.2.8
Amino sugar and nucleotide sugar metabolism
1.1.1.140
D-Sorbitol-6P
3.2.1.78
3.2.1.77
3.2.1.137
ADP-mannose
3.6.1.21
D-Mannose-1P
GDP-D-mannuronate
2.4.1.33
Mannuronan
5.1.3.37
Alginate
4.2.2.3
4.2.2.11
Δ-Dimer
3.1.3.11
2.7.1.11
1.1.1.-
2.4.1.-
Mannan
2.7.7.13
2.7.7.22
3.6.1.21
1.1.1.132
Mannosyl-3-phosphoglycerate
2.7.1.90
2.7.1.198
L-Sorbose-1P
1,4-β-Mannan
2.4.1.-
GDP-D-mannose
2.4.1.217
3.1.3.70
Mannosyl-glycerate
D-Sorbitol
2.7.1.206
2.4.1.269
4.2.1.47
N-Glycan biosynthesis
GDP-4-oxo-6-deoxy-D-mannose
1.1.1.135
GDP-6-deoxy-D-talose
L-Sorbose
β-D-Fructose-1,6P2
1.1.1.271
1.1.1.187
1.1.1.281
GDP-L-fucose
GDP-D-rhamnose
4.1.2.13
Glycolysis
Glyceraldehyde-3P
2.7.7.30
L-Fucose
2.7.1.52
L-Fucose-1P
5.3.1.1
5.3.1.25
L-Fuculose-1P
2.7.1.51
4.1.2.17
Glycerone-P
2.7.1.28
2.7.1.56
L-Fuculose
2,4-Diketo-3-deoxy-L-fuconate
1.1.1.122
3.1.1.-
4.2.1.68
1.1.1.-
3.7.1.-
L-Fucono-lactone
L-Fuconate
2-Dehydro-3-deoxy-L-fuconate
L-Lactate
Pyruvate metabolism
1.1.1.401
L-Rhamno-furanose
L-Rhamnono-1,4-lactone
L-Rhamnonate
1.1.1.173
3.1.1.65
4.2.1.90
4.1.2.53
D-Glycer-aldehyde
2-Dehydro-3-deoxy-L-rhamnonate
L-Lactaldehyde
1.1.1.378
L-Rhamnose
1.1.1.377
5.3.1.14
L-Rhamnulose-1P
2.7.1.5
4.1.2.19
L-Rhamnulose
2.7.1.3
4.1.2.13
2.7.1.202
D-Fructose-1P
00051 7/6/17
(c) Kanehisa Laboratories

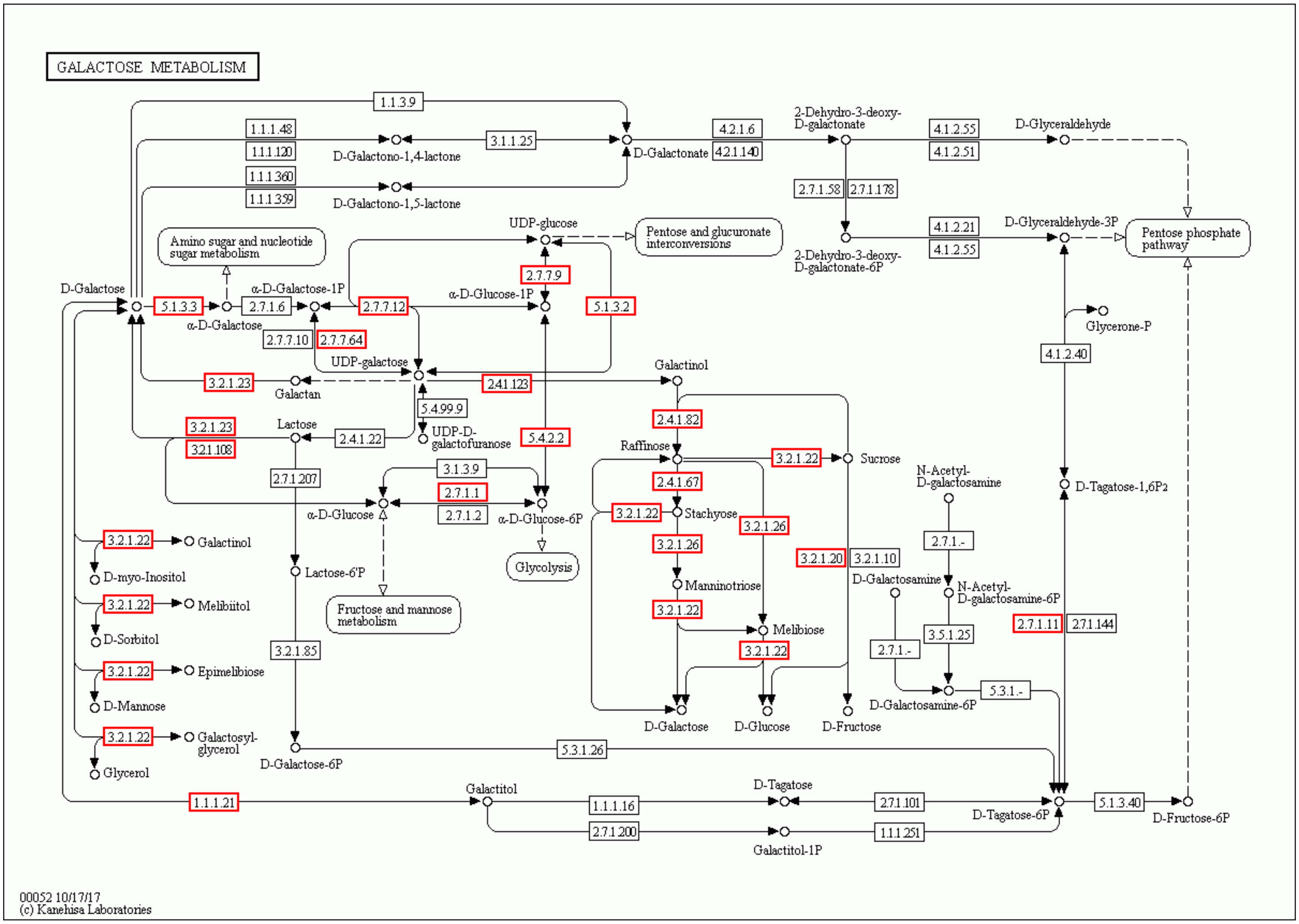
GALACTOSE METABOLISM
1.1.3.9
1.1.1.48
1.1.1.120
3.1.1.25
D-Galactono-1,4-lactone
1.1.1.360
1.1.1.359
D-Galactono-1,5-lactone
D-Galactonate
4.2.1.6
4.2.1.140
2-Dehydro-3-deoxy-D-galactonate
4.1.2.55
4.1.2.51
D-Glyceraldehyde
2.7.1.58
2.7.1.178
4.1.2.21
4.1.2.55
2-Dehydro-3-deoxy-D-galactonate-6P
D-Glyceraldehyde-3P
Pentose phosphate pathway
UDP-glucose
Pentose and glucuronate interconversions
Amino sugar and nucleotide sugar metabolism
D-Galactose
5.1.3.3
2.7.1.6
α-D-Galactose-1P
α-D-Galactose
2.7.7.12
2.7.7.9
α-D-Glucose-1P
5.1.3.2
2.7.7.10
2.7.7.64
UDP-galactose
Glycerone-P
4.1.2.40
3.2.1.23
Galactan
2.4.1.123
Galactinol
5.4.99.9
UDP-D-galactofuranose
3.2.1.23
3.2.1.108
Lactose
2.4.1.22
5.4.2.2
2.4.1.82
Raffinose
3.2.1.22
Sucrose
3.1.3.9
2.7.1.1
2.7.1.2
2.7.1.207
α-D-Glucose
α-D-Glucose-6P
2.4.1.67
3.2.1.22
Stachyose
3.2.1.26
N-Acetyl-D-galactosamine
D-Tagatose-1,6P2
Glycolysis
3.2.1.26
2.7.1.-
3.2.1.22
Galactinol
D-myo-Inositol
Lactose-6'P
3.2.1.20
3.2.1.10
D-Galactosamine
N-Acetyl-D-galactosamine-6P
Manninotriose
Fructose and mannose metabolism
3.2.1.22
Melibiitol
D-Sorbitol
3.2.1.22
Melibiose
2.7.1.11
2.7.1.144
3.5.1.25
3.2.1.22
2.7.1.-
3.2.1.85
3.2.1.22
Epimelibiose
D-Mannose
5.3.1.-
D-Galactosamine-6P
D-Galactose
D-Glucose
D-Fructose
3.2.1.22
Galactosyl-glycerol
Glycerol
D-Galactose-6P
5.3.1.26
1.1.1.21
Galactitol
1.1.1.16
D-Tagatose
2.7.1.101
D-Tagatose-6P
5.1.3.40
D-Fructose-6P
2.7.1.200
Galactitol-1P
1.1.1.251
00052 10/17/17
(c) Kanehisa Laboratories

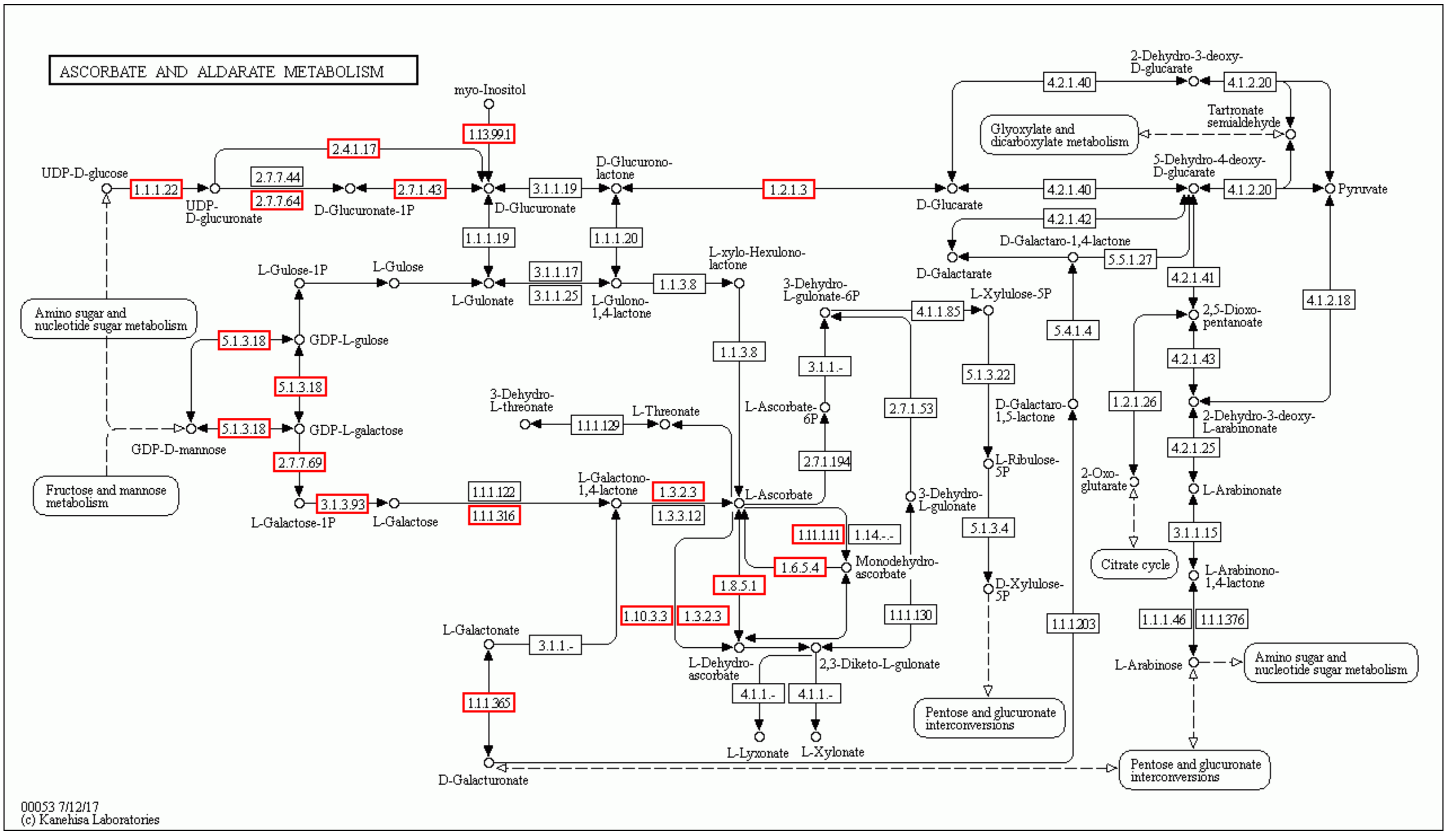
ASCORBATE AND ALDARATE METABOLISM
myo-Inositol
1.13.99.1
2.4.1.17
UDP-D-glucose
1.1.1.22
UDP-D-glucuronate
2.7.7.44
2.7.7.64
D-Glucuronate-1P
2.7.1.43
D-Glucuronate
3.1.1.19
D-Glucurono-lactone
1.2.1.3
D-Glucarate
4.2.1.40
2-Dehydro-3-deoxy-D-glucarate
4.1.2.20
Tartronate semialdehyde
Glyoxylate and dicarboxylate metabolism
5-Dehydro-4-deoxy-D-glucarate
4.2.1.40
4.1.2.20
Pyruvate
4.2.1.42
D-Galactaro-1,4-lactone
D-Galactarate
5.5.1.27
4.2.1.41
1.1.1.19
1.1.1.20
L-Gulose-1P
L-Gulose
3.1.1.17
3.1.1.25
L-Gulonate
L-Gulono-1,4-lactone
1.1.3.8
L-xylo-Hexulono-lactone
3-Dehydro-L-gulonate-6P
4.1.1.85
L-Xylulose-5P
2,5-Dioxo-pentanoate
4.1.2.18
Amino sugar and nucleotide sugar metabolism
5.1.3.18
GDP-L-gulose
1.1.3.8
5.4.1.4
3.1.1.-
5.1.3.22
4.2.1.43
5.1.3.18
3-Dehydro-L-threonate
1.1.1.129
L-Threonate
L-Ascorbate-6P
2.7.1.53
D-Galactaro-1,5-lactone
1.2.1.26
2-Dehydro-3-deoxy-L-arabinonate
5.1.3.18
GDP-D-mannose
GDP-L-galactose
2.7.1.194
L-Ribulose-5P
4.2.1.25
2.7.7.69
L-Galactono-1,4-lactone
1.3.2.3
1.3.3.12
L-Ascorbate
3-Dehydro-L-gulonate
2-Oxo-glutarate
L-Arabinonate
Fructose and mannose metabolism
3.1.3.93
L-Galactose-1P
L-Galactose
1.1.1.122
1.1.1.316
5.1.3.4
1.11.1.11
1.14.-.-
3.1.1.15
1.6.5.4
Monodehydro-ascorbate
D-Xylulose-5P
Citrate cycle
L-Arabinono-1,4-lactone
1.8.5.1
L-Galactonate
1.10.3.3
1.3.2.3
1.1.1.130
1.1.1.203
1.1.1.46
1.1.1.376
3.1.1.-
L-Dehydro-ascorbate
2,3-Diketo-L-gulonate
L-Arabinose
Amino sugar and nucleotide sugar metabolism
1.1.1.365
4.1.1.-
4.1.1.-
Pentose and glucuronate interconversions
L-Lyxonate
L-Xylonate
D-Galacturonate
Pentose and glucuronate interconversions
00053 7/12/17
(c) Kanehisa Laboratories

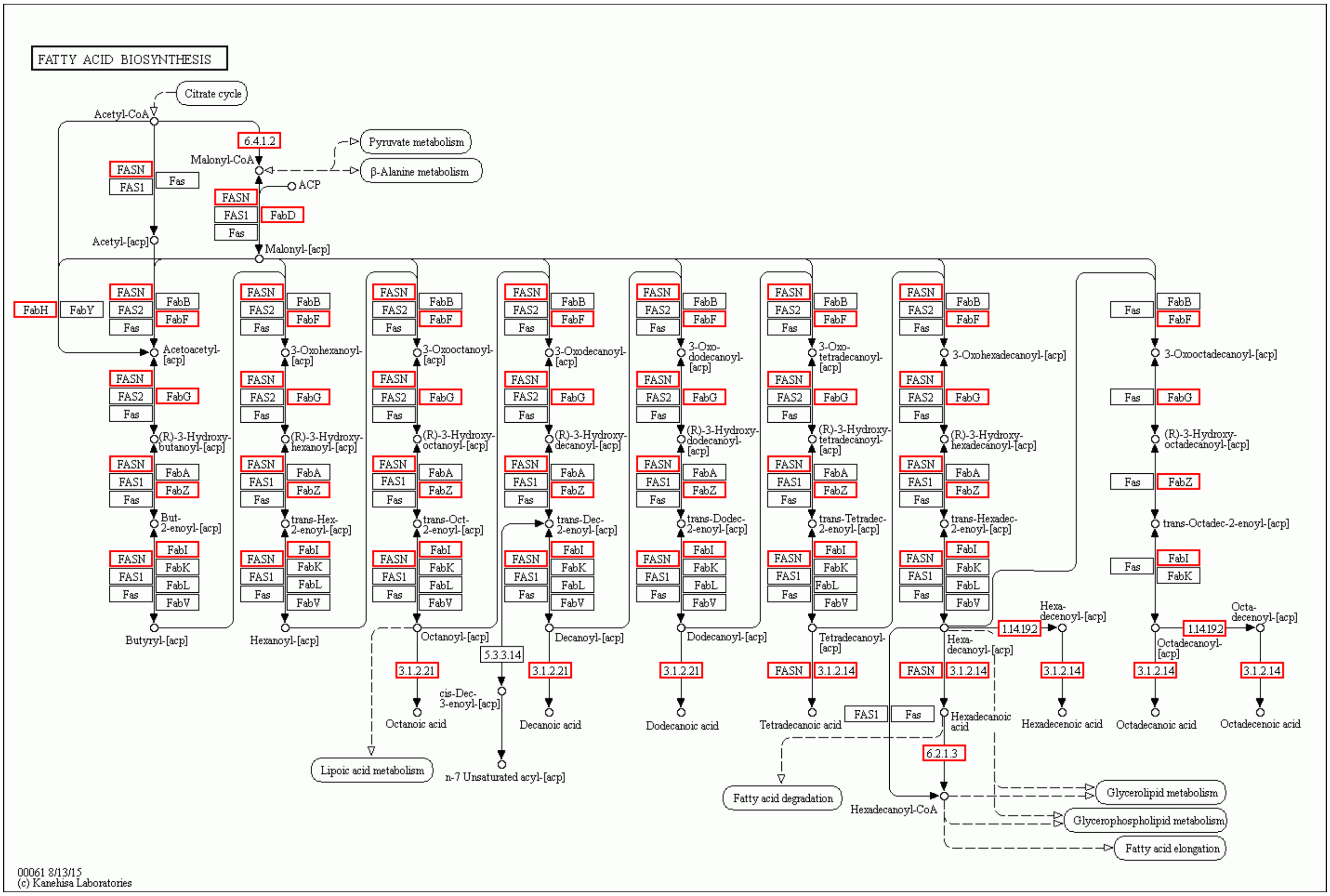
FATTY ACID BIOSYNTHESIS
Citrate cycle
Acetyl-CoA
6.4.1.2
Malonyl-CoA
Pyruvate metabolism
β-Alanine metabolism
ACP
FASN
FAS1
Fas
FabD
Acetyl-[acp]
Malonyl-[acp]
FabH
FabY
FAS2
FabB
FabF
Acetoacetyl-[acp]
3-Oxohexanoyl-[acp]
3-Oxooctanoyl-[acp]
3-Oxodecanoyl-[acp]
3-Oxo-dodecanoyl-[acp]
3-Oxo-tetradecanoyl-[acp]
3-Oxohexadecanoyl-[acp]
3-Oxooctadecanoyl-[acp]
FabG
(R)-3-Hydroxy-butanoyl-[acp]
(R)-3-Hydroxy-hexanoyl-[acp]
(R)-3-Hydroxy-octanoyl-[acp]
(R)-3-Hydroxy-decanoyl-[acp]
(R)-3-Hydroxy-dodecanoyl-[acp]
(R)-3-Hydroxy-tetradecanoyl-[acp]
(R)-3-Hydroxy-hexadecanoyl-[acp]
(R)-3-Hydroxy-octadecanoyl-[acp]
FabA
FabZ
But-2-enoyl-[acp]
trans-Hex-2-enoyl-[acp]
trans-Oct-2-enoyl-[acp]
trans-Dec-2-enoyl-[acp]
trans-Dodec-2-enoyl-[acp]
trans-Tetradec-2-enoyl-[acp]
trans-Hexadec-2-enoyl-[acp]
trans-Octadec-2-enoyl-[acp]
FabI
FabK
FabL
FabV
Butyryl-[acp]
Hexanoyl-[acp]
Octanoyl-[acp]
Decanoyl-[acp]
Dodecanoyl-[acp]
Tetradecanoyl-[acp]
Hexa-decanoyl-[acp]
Octadecanoyl-[acp]
Hexa-decenoyl-[acp]
Octa-decenoyl-[acp]
1.14.19.2
5.3.3.14
3.1.2.21
3.1.2.14
cis-Dec-3-enoyl-[acp]
Octanoic acid
Decanoic acid
Dodecanoic acid
Tetradecanoic acid
Hexadecanoic acid
Hexadecenoic acid
Octadecanoic acid
Octadecenoic acid
6.2.1.3
Lipoic acid metabolism
n-7 Unsaturated acyl-[acp]
Fatty acid degradation
Hexadecanoyl-CoA
Glycerolipid metabolism
Glycerophospholipid metabolism
Fatty acid elongation
00061 8/13/15
(c) Kanehisa Laboratories

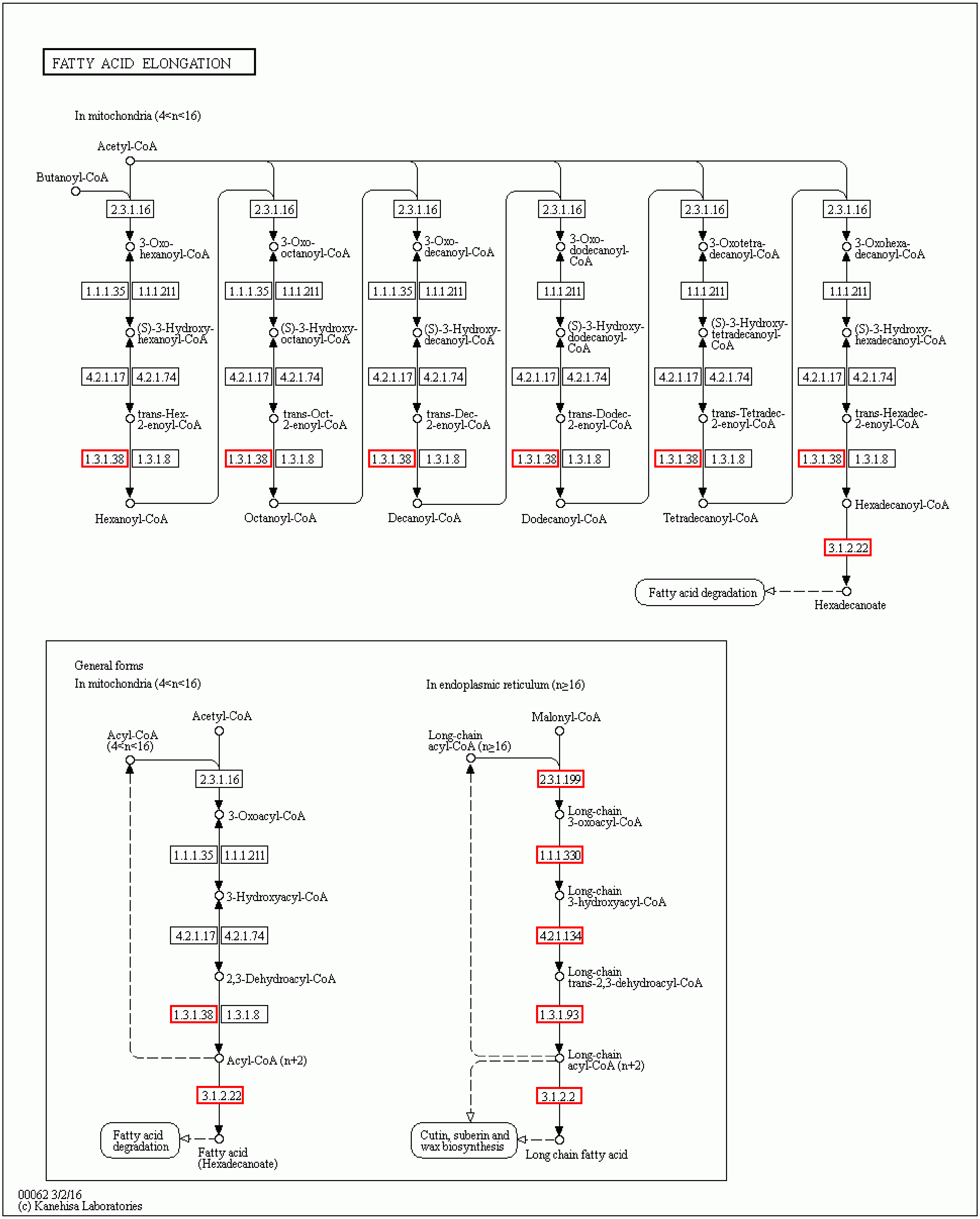
FATTY ACID ELONGATION
In mitochondria (4<n<16)
Acetyl-CoA
Butanoyl-CoA
2.3.1.16
3-Oxo-hexanoyl-CoA
3-Oxo-octanoyl-CoA
3-Oxo-decanoyl-CoA
3-Oxo-dodecanoyl-CoA
3-Oxotetra-decanoyl-CoA
3-Oxohexa-decanoyl-CoA
1.1.1.35
1.1.1.211
(S)-3-Hydroxy-hexanoyl-CoA
(S)-3-Hydroxy-octanoyl-CoA
(S)-3-Hydroxy-decanoyl-CoA
(S)-3-Hydroxy-dodecanoyl-CoA
(S)-3-Hydroxy-tetradecanoyl-CoA
(S)-3-Hydroxy-hexadecanoyl-CoA
4.2.1.17
4.2.1.74
trans-Hex-2-enoyl-CoA
trans-Oct-2-enoyl-CoA
trans-Dec-2-enoyl-CoA
trans-Dodec-2-enoyl-CoA
trans-Tetradec-2-enoyl-CoA
trans-Hexadec-2-enoyl-CoA
1.3.1.38
1.3.1.8
Hexanoyl-CoA
Octanoyl-CoA
Decanoyl-CoA
Dodecanoyl-CoA
Tetradecanoyl-CoA
Hexadecanoyl-CoA
3.1.2.22
Fatty acid degradation
Hexadecanoate
General forms
In mitochondria (4<n<16)
Acetyl-CoA
Acyl-CoA (4<n<16)
2.3.1.16
3-Oxoacyl-CoA
1.1.1.35
1.1.1.211
3-Hydroxyacyl-CoA
4.2.1.17
4.2.1.74
2,3-Dehydroacyl-CoA
1.3.1.38
1.3.1.8
Acyl-CoA (n+2)
3.1.2.22
Fatty acid degradation
Fatty acid (Hexadecanoate)
In endoplasmic reticulum (n≥16)
Malonyl-CoA
Long-chain acyl-CoA (n≥16)
2.3.1.199
Long-chain 3-oxoacyl-CoA
1.1.1.330
Long-chain 3-hydroxyacyl-CoA
4.2.1.134
Long-chain trans-2,3-dehydroacyl-CoA
1.3.1.93
Long-chain acyl-CoA (n+2)
3.1.2.2
Cutin, suberin and wax biosynthesis
Long chain fatty acid
00062 3/2/16
(c) Kanehisa Laboratories

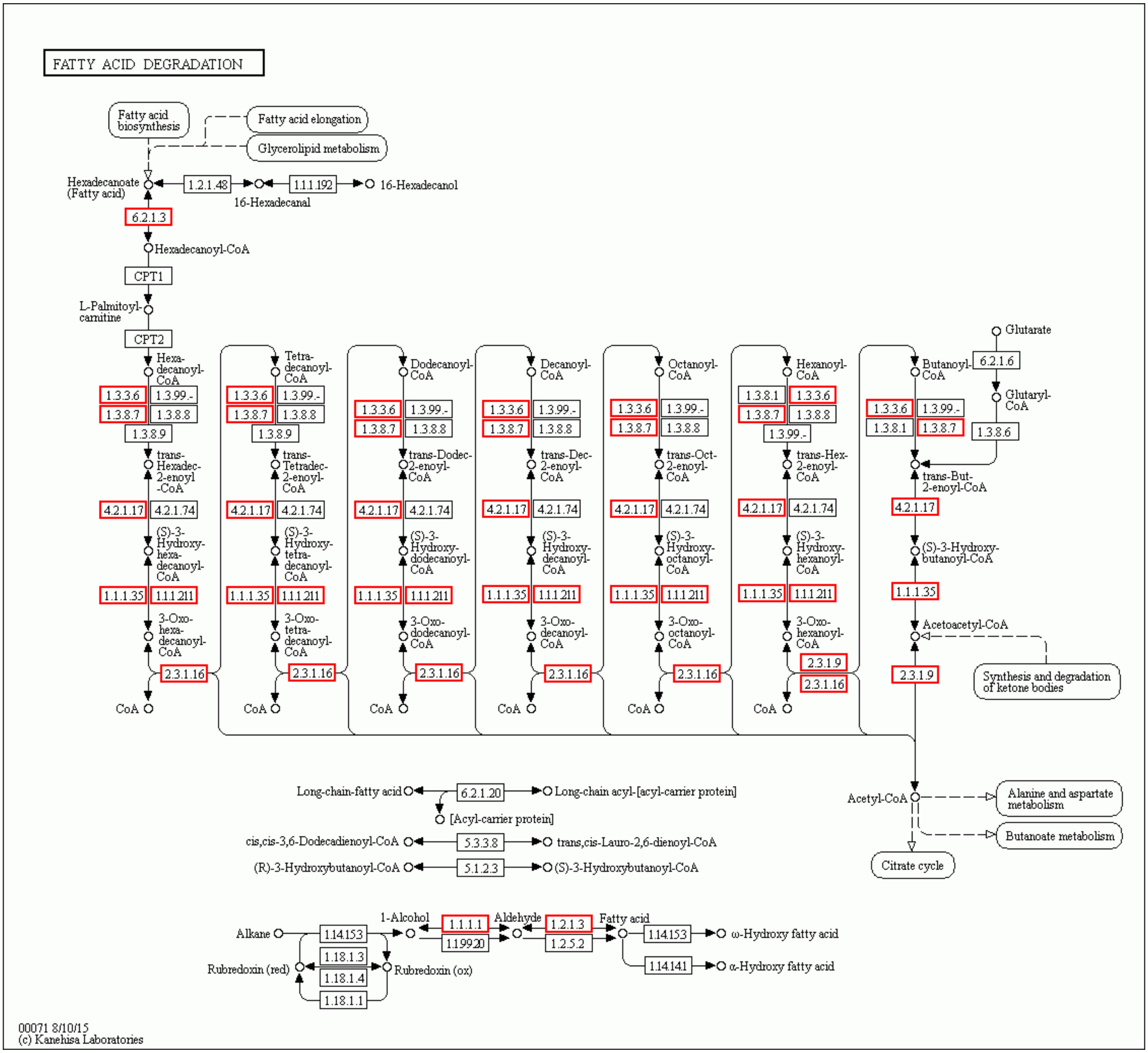

FATTY ACID DEGRADATION
Fatty acid biosynthesis
Fatty acid elongation
Glycerolipid metabolism
Hexadecanoate (Fatty acid)
1.2.1.48
1.1.1.192
16-Hexadecanol
16-Hexadecanal
6.2.1.3
Hexadecanoyl-CoA
CPT1
L-Palmitoyl-carnitine
CPT2
Hexa-decanoyl-CoA
Tetra-decanoyl-CoA
Dodecanoyl-CoA
Decanoyl-CoA
Octanoyl-CoA
Hexanoyl-CoA
Butanoyl-CoA
Glutarate
6.2.1.6
Glutaryl-CoA
1.3.8.6
1.3.3.6
1.3.99.-
1.3.8.7
1.3.8.8
1.3.8.9
1.3.8.1
trans-Hexadec-2-enoyl-CoA
trans-Tetradec-2-enoyl-CoA
trans-Dodec-2-enoyl-CoA
trans-Dec-2-enoyl-CoA
trans-Oct-2-enoyl-CoA
trans-Hex-2-enoyl-CoA
trans-But-2-enoyl-CoA
4.2.1.17
4.2.1.74
(S)-3-Hydroxy-hexa-decanoyl-CoA
(S)-3-Hydroxy-tetra-decanoyl-CoA
(S)-3-Hydroxy-dodecanoyl-CoA
(S)-3-Hydroxy-decanoyl-CoA
(S)-3-Hydroxy-octanoyl-CoA
(S)-3-Hydroxy-hexanoyl-CoA
(S)-3-Hydroxy-butanoyl-CoA
1.1.1.35
1.1.1.211
3-Oxo-hexa-decanoyl-CoA
3-Oxo-tetra-decanoyl-CoA
3-Oxo-dodecanoyl-CoA
3-Oxo-decanoyl-CoA
3-Oxo-octanoyl-CoA
3-Oxo-hexanoyl-CoA
Acetoacetyl-CoA
2.3.1.16
2.3.1.9
CoA
Synthesis and degradation of ketone bodies
Long-chain-fatty acid
6.2.1.20
Long-chain acyl-[acyl-carrier protein]
[Acyl-carrier protein]
cis,cis-3,6-Dodecadienoyl-CoA
5.3.3.8
trans,cis-Lauro-2,6-dienoyl-CoA
(R)-3-Hydroxybutanoyl-CoA
5.1.2.3
(S)-3-Hydroxybutanoyl-CoA
Acetyl-CoA
Alanine and aspartate metabolism
Butanoate metabolism
Citrate cycle
Alkane
1.14.15.3
1-Alcohol
1.1.1.1
1.1.99.20
Aldehyde
1.2.1.3
1.2.5.2
Fatty acid
1.14.15.3
ω-Hydroxy fatty acid
1.14.14.1
α-Hydroxy fatty acid
1.18.1.3
1.18.1.4
1.18.1.1
Rubredoxin (red)
Rubredoxin (ox)
00071 8/10/15
(c) Kanehisa Laboratories

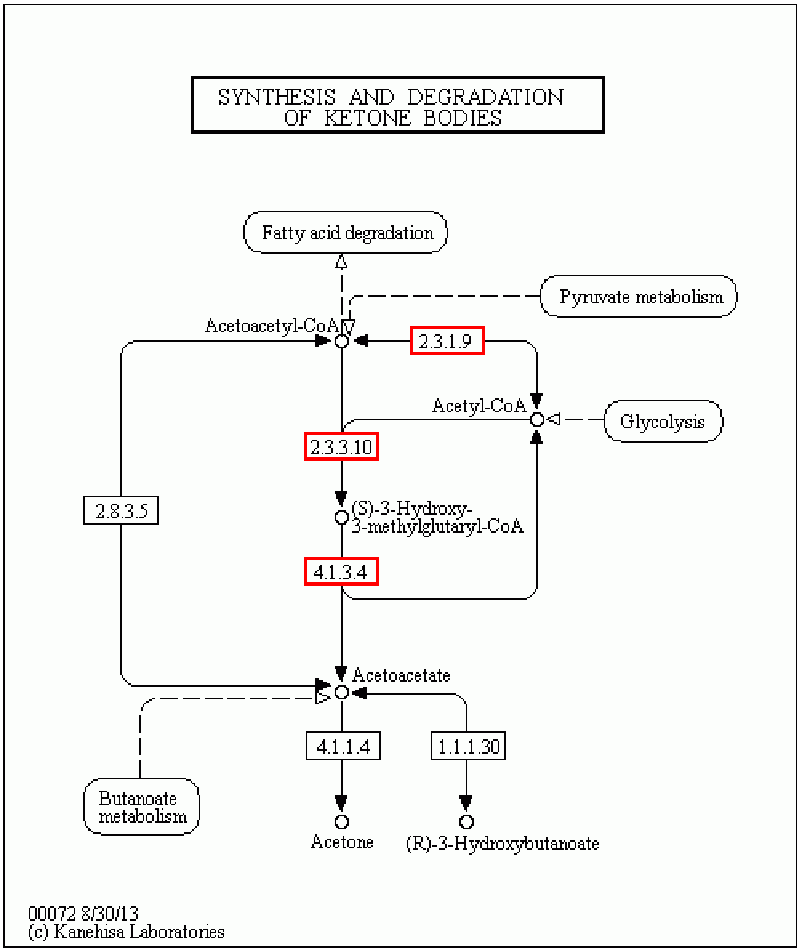
SYNTHESIS AND DEGRADATION OF KETONE BODIES
Fatty acid degradation
Pyruvate metabolism
Acetoacetyl-CoA
2.3.1.9
Acetyl-CoA
Glycolysis
2.3.3.10
2.8.3.5
(S)-3-Hydroxy-3-methylglutaryl-CoA
4.1.3.4
Acetoacetate
4.1.1.4
1.1.1.30
Butanoate metabolism
Acetone
(R)-3-Hydroxybutanoate
00072 8/30/13
(c) Kanehisa Laboratories

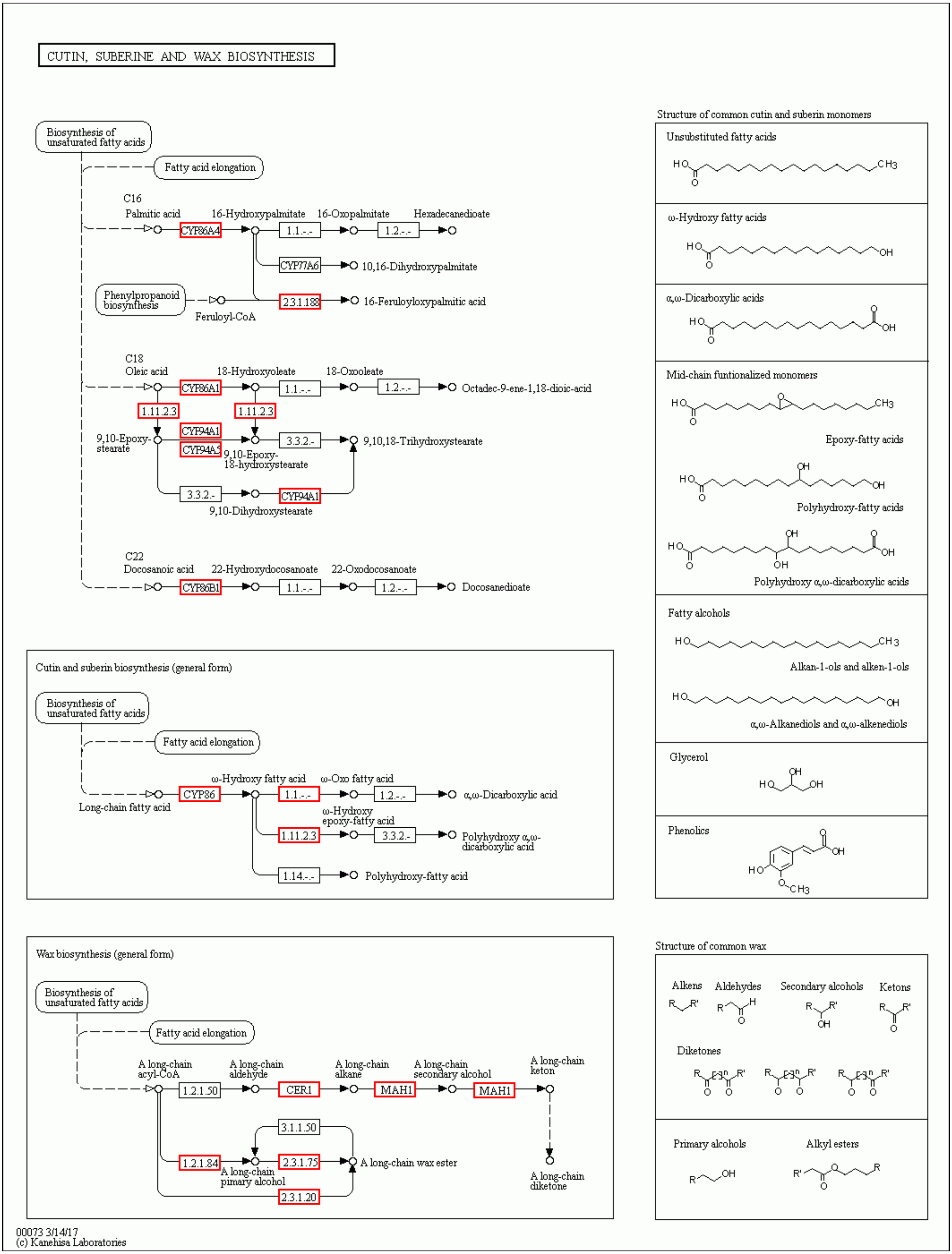
CUTIN, SUBERINE AND WAX BIOSYNTHESIS
Biosynthesis of unsaturated fatty acids
Fatty acid elongation
C16
Palmitic acid
CYP86A4
16-Hydroxypalmitate
1.1.-.-
16-Oxopalmitate
1.2.-.-
Hexadecanedioate
CYP77A6
10,16-Dihydroxypalmitate
Phenylpropanoid biosynthesis
Feruloyl-CoA
2.3.1.188
16-Feruloyloxypalmitic acid
C18
Oleic acid
CYP86A1
18-Hydroxyoleate
1.1.-.-
18-Oxooleate
1.2.-.-
Octadec-9-ene-1,18-dioic-acid
1.11.2.3
1.11.2.3
9,10-Epoxy-stearate
CYP94A1
CYP94A5
3.3.2.-
9,10,18-Trihydroxystearate
9,10-Epoxy-18-hydroxystearate
3.3.2.-
CYP94A1
9,10-Dihydroxystearate
C22
Docosanoic acid
CYP86B1
22-Hydroxydocosanoate
1.1.-.-
22-Oxodocosanoate
1.2.-.-
Docosanedioate
Cutin and suberin biosynthesis (general form)
Biosynthesis of unsaturated fatty acids
Fatty acid elongation
Long-chain fatty acid
CYP86
ω-Hydroxy fatty acid
1.1.-.-
ω-Oxo fatty acid
1.2.-.-
α,ω-Dicarboxylic acid
1.11.2.3
ω-Hydroxy epoxy-fatty acid
3.3.2.-
Polyhydroxy α,ω-dicarboxylic acid
1.14.-.-
Polyhydroxy-fatty acid
Wax biosynthesis (general form)
Biosynthesis of unsaturated fatty acids
Fatty acid elongation
A long-chain acyl-CoA
1.2.1.50
A long-chain aldehyde
CER1
A long-chain alkane
MAH1
A long-chain secondary alcohol
MAH1
A long-chain keton
A long-chain diketone
3.1.1.50
1.2.1.84
A long-chain pimary alcohol
2.3.1.75
A long-chain wax ester
2.3.1.20
Structure of common cutin and suberin monomers
Unsubstituted fatty acids
ω-Hydroxy fatty acids
α,ω-Dicarboxylic acids
Mid-chain funtionalized monomers
Epoxy-fatty acids
Polyhydroxy-fatty acids
Polyhydroxy α,ω-dicarboxylic acids
Fatty alcohols
Alkan-1-ols and alken-1-ols
α,ω-Alkanediols and α,ω-alkenediols
Glycerol
Phenolics
Structure of common wax
Alkens
Aldehydes
Secondary alcohols
Ketons
Diketones
Primary alcohols
Alkyl esters
00073 3/14/17
(c) Kanehisa Laboratories

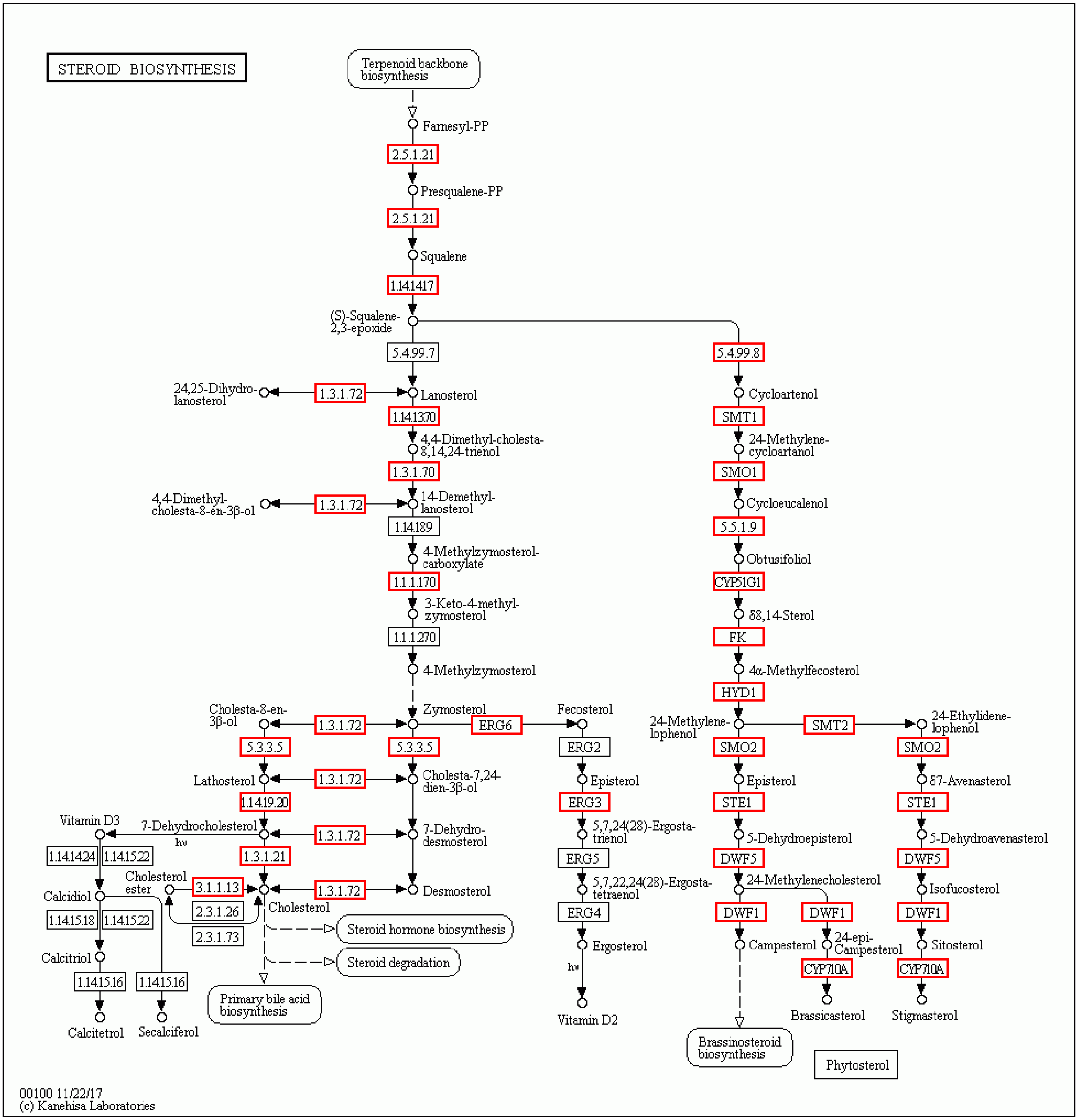

STEROID BIOSYNTHESIS
Terpenoid backbone biosynthesis
Farnesyl-PP
2.5.1.21
Presqualene-PP
2.5.1.21
Squalene
1.14.14.17
(S)-Squalene-2,3-epoxide
5.4.99.7
5.4.99.8
24,25-Dihydro-lanosterol
1.3.1.72
Lanosterol
Cycloartenol
1.14.13.70
SMT1
4,4-Dimethyl-cholesta-8,14,24-trienol
24-Methylene-cycloartanol
1.3.1.70
SMO1
4,4-Dimethyl-cholesta-8-en-3β-ol
1.3.1.72
14-Demethyl-lanosterol
Cycloeucalenol
1.14.18.9
5.5.1.9
4-Methylzymosterol-carboxylate
Obtusifoliol
1.1.1.170
CYP51G1
3-Keto-4-methyl-zymosterol
δ8,14-Sterol
1.1.1.270
FK
4-Methylzymosterol
4α-Methylfecosterol
HYD1
Cholesta-8-en-3β-ol
1.3.1.72
Zymosterol
ERG6
Fecosterol
24-Methylene-lophenol
SMT2
24-Ethylidene-lophenol
5.3.3.5
5.3.3.5
ERG2
SMO2
SMO2
Lathosterol
1.3.1.72
Cholesta-7,24-dien-3β-ol
Episterol
Episterol
δ7-Avenasterol
1.14.19.20
ERG3
STE1
STE1
Vitamin D3
7-Dehydrocholesterol
1.3.1.72
7-Dehydro-desmosterol
5,7,24(28)-Ergosta-trienol
5-Dehydroepisterol
5-Dehydroavenasterol
hν
1.14.14.24
1.14.15.22
1.3.1.21
ERG5
DWF5
DWF5
Calcidiol
Cholesterol ester
3.1.1.13
1.3.1.72
Desmosterol
5,7,22,24(28)-Ergosta-tetraenol
24-Methylenecholesterol
Isofucosterol
Cholesterol
ERG4
DWF1
DWF1
DWF1
1.14.15.18
1.14.15.22
2.3.1.26
Steroid hormone biosynthesis
2.3.1.73
Ergosterol
Campesterol
24-epi-Campesterol
Sitosterol
Calcitriol
Steroid degradation
hν
CYP710A
CYP710A
1.14.15.16
1.14.15.16
Primary bile acid biosynthesis
Calcitetrol
Secalciferol
Vitamin D2
Brassicasterol
Stigmasterol
Brassinosteroid biosynthesis
Phytosterol
00100 11/22/17
(c) Kanehisa Laboratories

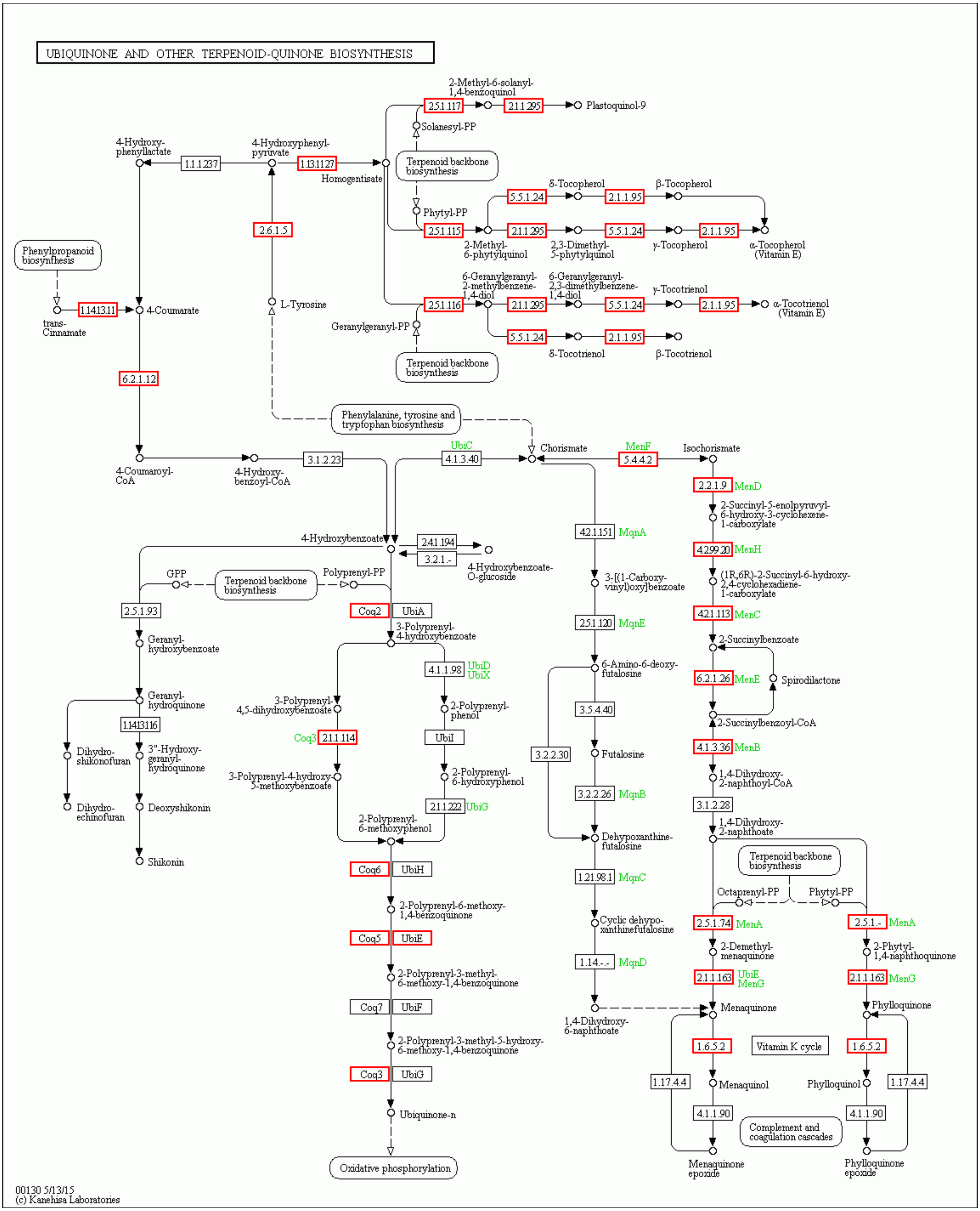
UBIQUINONE AND OTHER TERPENOID-QUINONE BIOSYNTHESIS
2-Methyl-6-solanyl-1,4-benzoquinol
2.5.1.117
2.1.1.295
Plastoquinol-9
Solanesyl-PP
4-Hydroxy-phenyllactate
1.1.1.237
4-Hydroxyphenyl-pyruvate
1.13.11.27
Homogentisate
Terpenoid backbone biosynthesis
δ-Tocopherol
β-Tocopherol
5.5.1.24
2.1.1.95
Phytyl-PP
2.6.1.5
2.5.1.115
2.1.1.295
5.5.1.24
2.1.1.95
2-Methyl-6-phytylquinol
2,3-Dimethyl-5-phytylquinol
γ-Tocopherol
α-Tocopherol (Vitamin E)
Phenylpropanoid biosynthesis
6-Geranylgeranyl-2-methylbenzene-1,4-diol
6-Geranylgeranyl-2,3-dimethylbenzene-1,4-diol
γ-Tocotrienol
1.14.13.11
4-Coumarate
L-Tyrosine
2.5.1.116
2.1.1.295
5.5.1.24
2.1.1.95
α-Tocotrienol (Vitamin E)
trans-Cinnamate
Geranylgeranyl-PP
5.5.1.24
2.1.1.95
δ-Tocotrienol
β-Tocotrienol
Terpenoid backbone biosynthesis
6.2.1.12
Phenylalanine, tyrosine and tryptophan biosynthesis
UbiC
MenF
Chorismate
Isochorismate
4.1.3.40
5.4.4.2
4-Coumaroyl-CoA
4-Hydroxy-benzoyl-CoA
3.1.2.23
2.2.1.9
MenD
2-Succinyl-5-enolpyruvyl-6-hydroxy-3-cyclohexene-1-carboxylate
4-Hydroxybenzoate
2.4.1.194
3.2.1.-
4.2.1.151
MqnA
4.2.99.20
MenH
4-Hydroxybenzoate-O-glucoside
GPP
Terpenoid backbone biosynthesis
Polyprenyl-PP
3-[(1-Carboxy-vinyl)oxy]benzoate
(1R,6R)-2-Succinyl-6-hydroxy-2,4-cyclohexadiene-1-carboxylate
2.5.1.93
Coq2
UbiA
4.2.1.113
MenC
Geranyl-hydroxybenzoate
3-Polyprenyl-4-hydroxybenzoate
2.5.1.120
MqnE
2-Succinylbenzoate
4.1.1.98
UbiD
UbiX
6-Amino-6-deoxy-futalosine
6.2.1.26
MenE
Spirodilactone
Geranyl-hydroquinone
3-Polyprenyl-4,5-dihydroxybenzoate
2-Polyprenyl-phenol
3.5.4.40
2-Succinylbenzoyl-CoA
1.14.13.116
Coq3
2.1.1.114
UbiI
3.2.2.30
Futalosine
4.1.3.36
MenB
Dihydro-shikonofuran
3''-Hydroxy-geranyl-hydroquinone
3-Polyprenyl-4-hydroxy-5-methoxybenzoate
2-Polyprenyl-6-hydroxyphenol
1,4-Dihydroxy-2-naphthoyl-CoA
3.2.2.26
MqnB
Dihydro-echinofuran
Deoxyshikonin
2.1.1.222
UbiG
3.1.2.28
2-Polyprenyl-6-methoxyphenol
1,4-Dihydroxy-2-naphthoate
Dehypoxanthine-futalosine
Shikonin
Terpenoid backbone biosynthesis
Coq6
UbiH
1.21.98.1
MqnC
Octaprenyl-PP
Phytyl-PP
2-Polyprenyl-6-methoxy-1,4-benzoquinone
Cyclic dehypo-xanthinefutalosine
2.5.1.74
MenA
2.5.1.-
MenA
Coq5
UbiE
2-Demethyl-menaquinone
2-Phytyl-1,4-naphthoquinone
2-Polyprenyl-3-methyl-6-methoxy-1,4-benzoquinone
1.14.-.-
MqnD
2.1.1.163
UbiE
MenG
2.1.1.163
MenG
Coq7
UbiF
Menaquinone
Phylloquinone
1,4-Dihydroxy-6-naphthoate
2-Polyprenyl-3-methyl-5-hydroxy-6-methoxy-1,4-benzoquinone
1.6.5.2
Vitamin K cycle
1.6.5.2
Coq3
UbiG
1.17.4.4
Menaquinol
Phylloquinol
1.17.4.4
Ubiquinone-n
4.1.1.90
4.1.1.90
Complement and coagulation cascades
Oxidative phosphorylation
Menaquinone epoxide
Phylloquinone epoxide
00130 5/13/15
(c) Kanehisa Laboratories

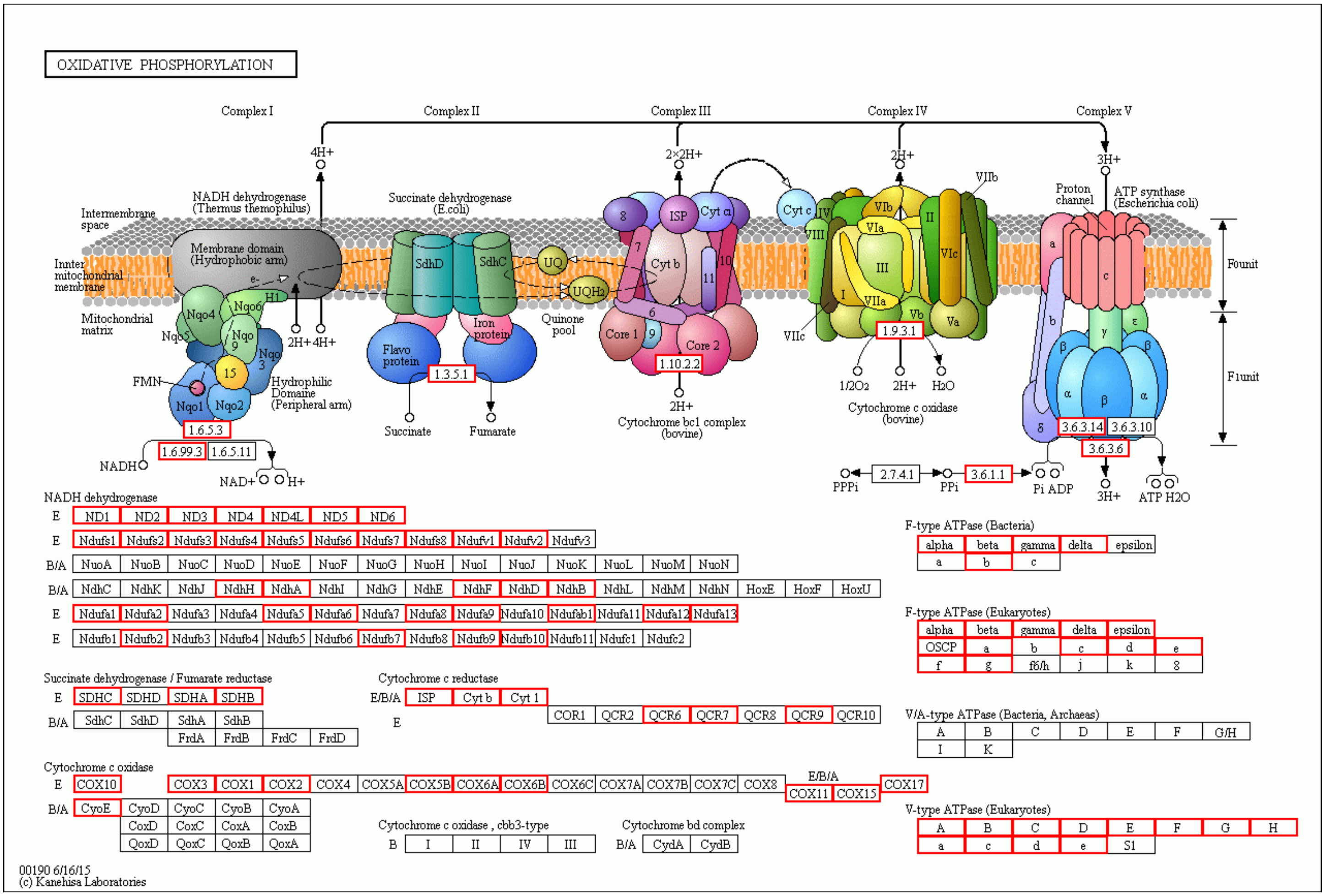
OXIDATIVE PHOSPHORYLATION
Complex I
Complex II
Complex III
Complex IV
Complex V
4H+
2×2H+
2H+
3H+
Intermembrane space
Inner mitochondrial membrane
Mitochondrial matrix
NADH dehydrogenase (Thermus themophilus)
Membrane domain (Hydrophobic arm)
e-
Nqo4
Nqo6
H1
Nqo5
Nqo 9
Nqo 3
15
FMN
Nqo1
Nqo2
Hydrophilic Domaine (Peripheral arm)
2H+
4H+
1.6.5.3
1.6.99.3
1.6.5.11
NADH
NAD+
H+
Succinate dehydrogenase (E.coli)
SdhD
SdhC
Iron protein
Flavo protein
1.3.5.1
Succinate
Fumarate
UQ
UQH2
Quinone pool
8
ISP
Cyt c1
7
Cyt b
10
11
6
Core 1
9
Core 2
1.10.2.2
2H+
Cytochrome bc1 complex (bovine)
Cyt c
IV
VIII
VIb
VIa
II
VIIb
VIc
III
I
VIIa
Vb
Va
VIIc
1.9.3.1
1/2O2
2H+
H2O
Cytochrome c oxidase (bovine)
Proton channel
ATP synthase (Escherichia coli)
a
c
b
γ
ε
β
α
δ
Founit
F1unit
3.6.3.14
3.6.3.10
3.6.3.6
PPPi
2.7.4.1
PPi
3.6.1.1
Pi ADP
3H+
ATP H2O
NADH dehydrogenase
E ND1 ND2 ND3 ND4 ND4L ND5 ND6
E Ndufs1 Ndufs2 Ndufs3 Ndufs4 Ndufs5 Ndufs6 Ndufs7 Ndufs8 Ndufv1 Ndufv2 Ndufv3
B/A NuoA NuoB NuoC NuoD NuoE NuoF NuoG NuoH NuoI NuoJ NuoK NuoL NuoM NuoN
B/A NdhC NdhK NdhJ NdhH NdhA NdhI NdhG NdhE NdhF NdhD NdhB NdhL NdhM NdhN HoxE HoxF HoxU
E Ndufa1 Ndufa2 Ndufa3 Ndufa4 Ndufa5 Ndufa6 Ndufa7 Ndufa8 Ndufa9 Ndufa10 Ndufab1 Ndufa11 Ndufa12 Ndufa13
E Ndufb1 Ndufb2 Ndufb3 Ndufb4 Ndufb5 Ndufb6 Ndufb7 Ndufb8 Ndufb9 Ndufb10 Ndufb11 Ndufc1 Ndufc2
Succinate dehydrogenase / Fumarate reductase
E SDHC SDHD SDHA SDHB
B/A SdhC SdhD SdhA SdhB
FrdA FrdB FrdC FrdD
Cytochrome c reductase
E/B/A ISP Cyt b Cyt 1
E COR1 QCR2 QCR6 QCR7 QCR8 QCR9 QCR10
Cytochrome c oxidase
E COX10 COX3 COX1 COX2 COX4 COX5A COX5B COX6A COX6B COX6C COX7A COX7B COX7C COX8
E/B/A COX11 COX15 COX17
B/A CyoE CyoD CyoC CyoB CyoA
CoxD CoxC CoxA CoxB
QoxD QoxC QoxB QoxA
Cytochrome c oxidase , cbb3-type
B I II IV III
Cytochrome bd complex
B/A CydA CydB
F-type ATPase (Bacteria)
alpha beta gamma delta epsilon
a b c
F-type ATPase (Eukaryotes)
alpha beta gamma delta epsilon
OSCP a b c d e
f g f6/h j k 8
V/A-type ATPase (Bacteria, Archaeas)
A B C D E F G/H
I K
V-type ATPase (Eukaryotes)
A B C D E F G H
a c d e S1
00190 6/16/15
(c) Kanehisa Laboratories

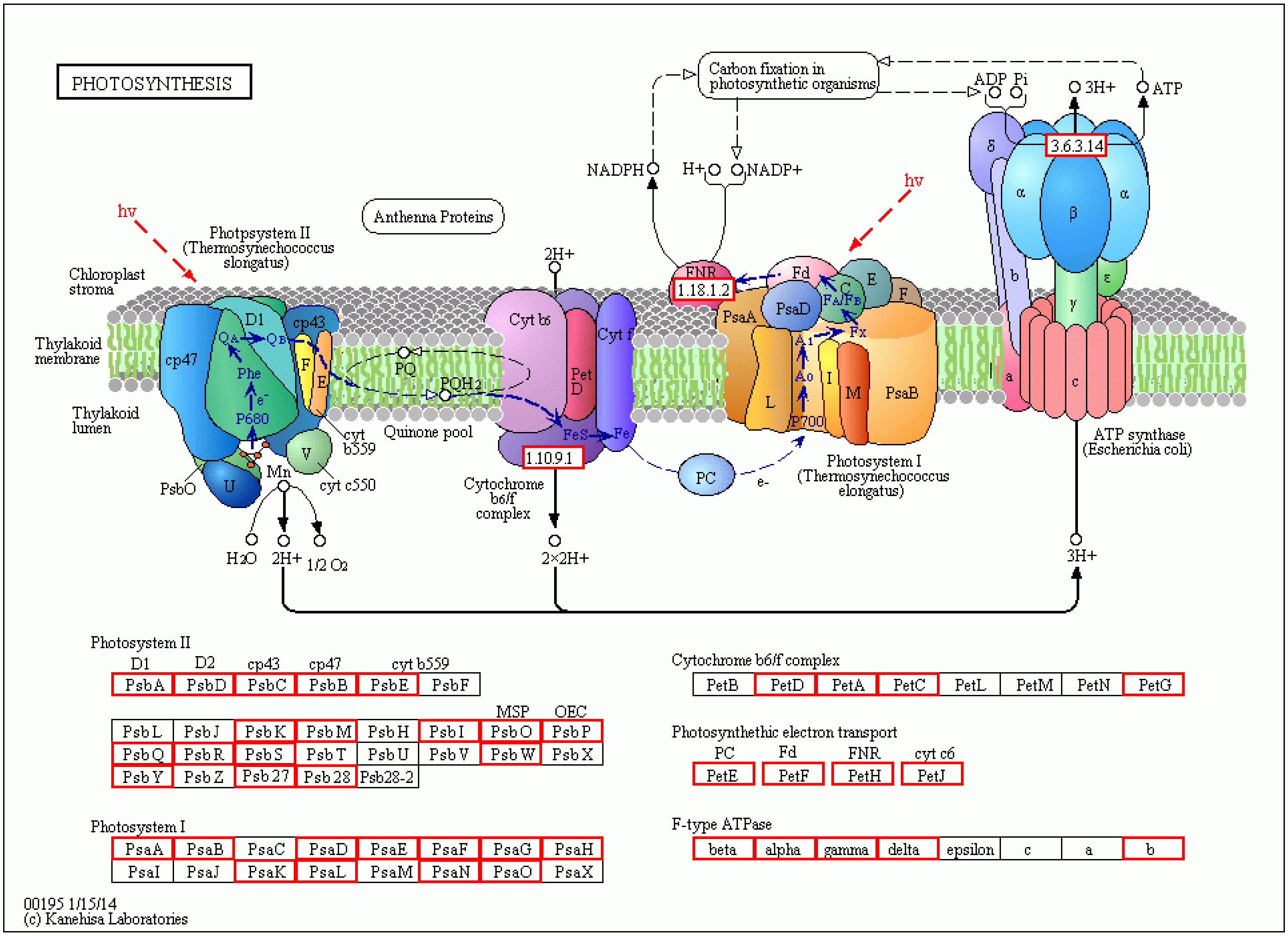
PHOTOSYNTHESIS
Carbon fixation in photosynthetic organisms
ADP Pi
3H+
ATP
NADPH
H+
NADP+
hν
Anthenna Proteins
Photpsystem II (Thermosynechococcus elongatus)
Chloroplast stroma
Thylakoid membrane
Thylakoid lumen
D1
cp43
cp47
QA
QB
Phe
P680
F
E
PQ
PQH2
V
PsbO
U
Mn
cyt b559
Quinone pool
cyt c550
H2O
2H+
1/2 O2
2H+
Cyt b6
Pet D
Cyt f
FeS
Fe
1.10.9.1
Cytochrome b6/f complex
2×2H+
FNR
1.18.1.2
Fd
E
C
FA/FB
F
PsaA
PsaD
Fx
A1
A0
I
L
M
PsaB
P700
PC
e-
Photosystem I (Thermosynechococcus elongatus)
3.6.3.14
δ
α
β
α
b
ε
γ
a
c
ATP synthase (Escherichia coli)
3H+
Photosystem II
D1 D2 cp43 cp47 cyt b559
PsbA PsbD PsbC PsbB PsbE PsbF
MSP OEC
PsbL PsbJ PsbK PsbM PsbH PsbI PsbO PsbP
PsbQ PsbR PsbS PsbT PsbU PsbV PsbW PsbX
PsbY PsbZ Psb27 Psb28 Psb28-2
Photosystem I
PsaA PsaB PsaC PsaD PsaE PsaF PsaG PsaH
PsaI PsaJ PsaK PsaL PsaM PsaN PsaO PsaX
Cytochrome b6/f complex
PetB PetD PetA PetC PetL PetM PetN PetG
Photosynthethic electron transport
PC Fd FNR cyt c6
PetE PetF PetH PetJ
F-type ATPase
beta alpha gamma delta epsilon c a b
00195 1/15/14
(c) Kanehisa Laboratories

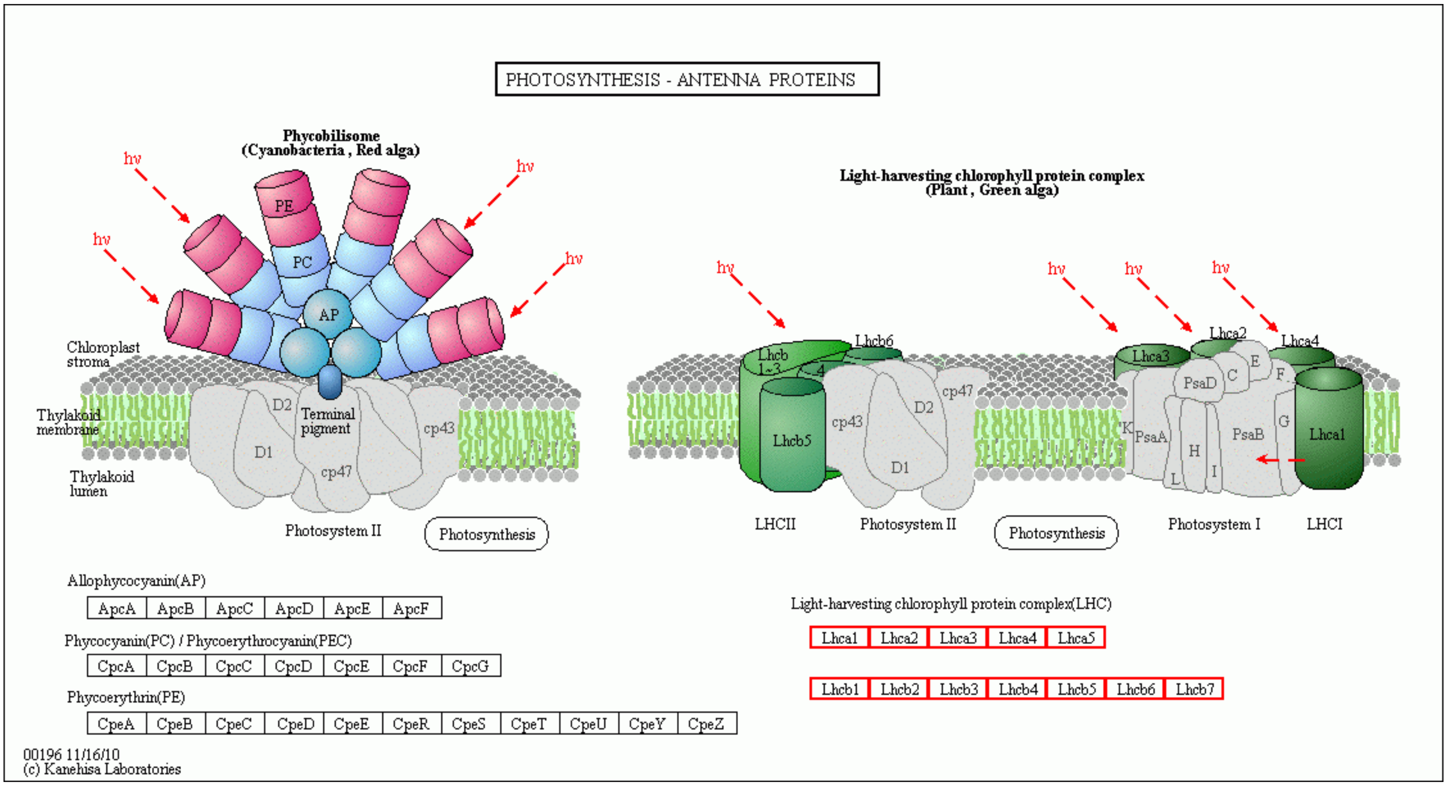
PHOTOSYNTHESIS - ANTENNA PROTEINS
Phycobilisome (Cyanobacteria , Red alga)
hν
PE
PC
AP
Chloroplast stroma
Thylakoid membrane
Thylakoid lumen
D2
D1
Terminal pigment
cp43
cp47
Photosystem II
Photosynthesis
Light-harvesting chlorophyll protein complex (Plant , Green alga)
Lhcb6
Lhcb 1-3
Lhcb5
cp43
cp47
D2
D1
Lhca3
Lhca2
Lhca4
PsaD
C
E
F
K
PsaA
H
L
I
PsaB
G
Lhca1
LHCII
Photosystem II
Photosynthesis
Photosystem I
LHCI
Allophycocyanin(AP)
ApcA ApcB ApcC ApcD ApcE ApcF
Phycocyanin(PC) / Phycoerythrocyanin(PEC)
CpcA CpcB CpcC CpcD CpcE CpcF CpcG
Phycoerythrin(PE)
CpeA CpeB CpeC CpeD CpeE CpeR CpeS CpeT CpeU CpeY CpeZ
Light-harvesting chlorophyll protein complex(LHC)
Lhca1 Lhca2 Lhca3 Lhca4 Lhca5
Lhcb1 Lhcb2 Lhcb3 Lhcb4 Lhcb5 Lhcb6 Lhcb7
00196 11/16/10
(c) Kanehisa Laboratories

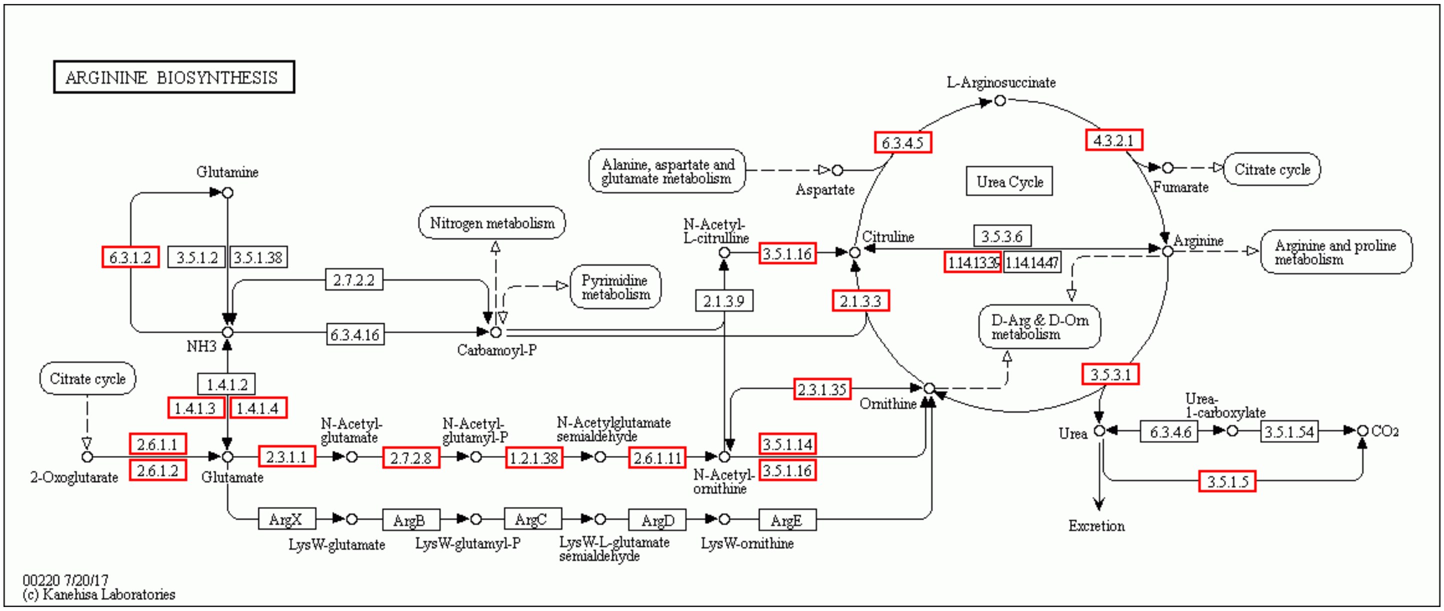
ARGININE BIOSYNTHESIS
L-Arginosuccinate
6.3.4.5
4.3.2.1
Alanine, aspartate and glutamate metabolism
Aspartate
Urea Cycle
Citrate cycle
Fumarate
Glutamine
Nitrogen metabolism
N-Acetyl-L-citrulline
3.5.1.16
Citruline
3.5.3.6
1.14.13.39
1.14.14.47
Arginine
Arginine and proline metabolism
6.3.1.2
3.5.1.2
3.5.1.38
2.7.2.2
Pyrimidine metabolism
2.1.3.9
2.1.3.3
D-Arg & D-Orn metabolism
NH3
6.3.4.16
Carbamoyl-P
Citrate cycle
1.4.1.2
1.4.1.3
1.4.1.4
2.3.1.35
3.5.3.1
Ornithine
Urea-1-carboxylate
2.6.1.1
2.6.1.2
2-Oxoglutarate
Glutamate
2.3.1.1
N-Acetyl-glutamate
2.7.2.8
N-Acetyl-glutamyl-P
1.2.1.38
N-Acetylglutamate semialdehyde
2.6.1.11
N-Acetyl-ornithine
3.5.1.14
3.5.1.16
Urea
6.3.4.6
3.5.1.54
CO2
3.5.1.5
Excretion
ArgX
ArgB
ArgC
ArgD
ArgE
LysW-glutamate
LysW-glutamyl-P
LysW-L-glutamate semialdehyde
LysW-ornithine
00220 7/20/17
(c) Kanehisa Laboratories

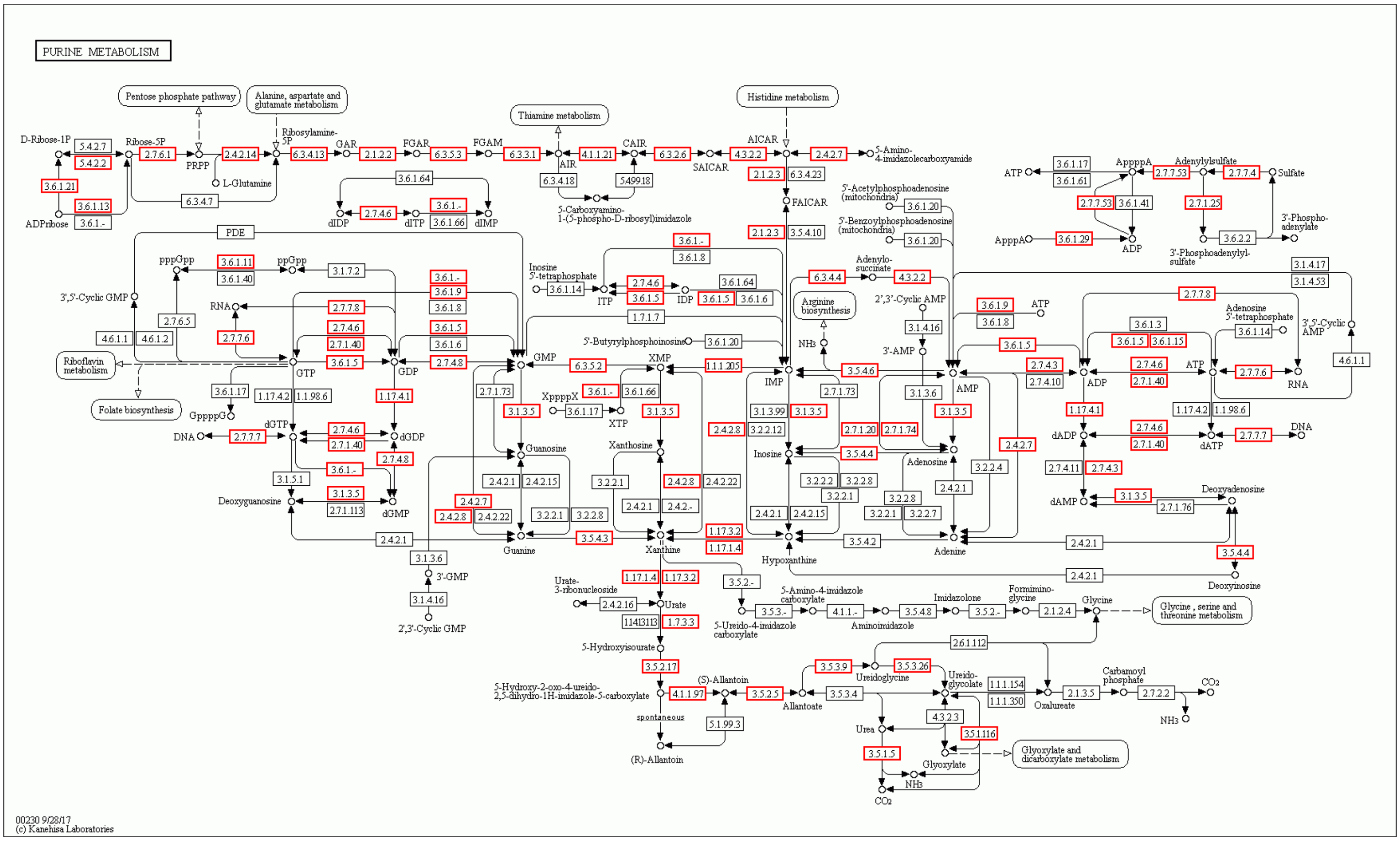
PURINE METABOLISM
Pentose phosphate pathway
Alanine, aspartate and glutamate metabolism
Thiamine metabolism
Histidine metabolism
D-Ribose-1P
Ribose-5P
PRPP
Ribosylamine-5P
GAR
FGAR
FGAM
AIR
CAIR
SAICAR
AICAR
5-Amino-4-imidazolecarboxyamide
L-Glutamine
ADPribose
5-Carboxyamino-1-(5-phospho-D-ribosyl)imidazole
FAICAR
dIDP
dITP
dIMP
PDE
pppGpp
ppGpp
3',5'-Cyclic GMP
RNA
Riboflavin metabolism
Folate biosynthesis
GTP
GDP
GMP
GppppG
dGTP
dGDP
DNA
Deoxyguanosine
dGMP
Guanosine
Guanine
3'-GMP
2',3'-Cyclic GMP
Inosine 5'-tetraphosphate
ITP
IDP
5'-Butyrylphosphoinosine
XpppX
XTP
XMP
Xanthosine
Xanthine
Urate-3-ribonucleoside
Urate
5-Hydroxyisourate
5-Hydroxy-2-oxo-4-ureido-2,5-dihydro-1H-imidazole-5-carboxylate
(S)-Allantoin
(R)-Allantoin
spontaneous
Allantoate
Ureidoglycine
Ureido-glycolate
Urea
Glyoxylate
NH3
CO2
Glyoxylate and dicarboxylate metabolism
Oxalureate
Carbamoyl phosphate
Glycine
Glycine, serine and threonine metabolism
5-Amino-4-imidazole carboxylate
5-Ureido-4-imidazole carboxylate
Aminoimidazole
Imidazolone
Formimino-glycine
Arginine biosynthesis
IMP
Inosine
Hypoxanthine
Adenylo-succinate
5'-Acetylphosphoadenosine (mitochondria)
5'-Benzoylphosphoadenosine (mitochondria)
2',3'-Cyclic AMP
3'-AMP
AMP
Adenosine
Adenine
ADP
ATP
RNA
Adenosine 5'-tetraphosphate
3',5'-Cyclic AMP
dADP
dATP
DNA
dAMP
Deoxyadenosine
Deoxyinosine
ApppA
AppppA
Adenylylsulfate
Sulfate
3'-Phospho-adenylate
3'-Phosphoadenylyl-sulfate
00230 9/28/17
(c) Kanehisa Laboratories

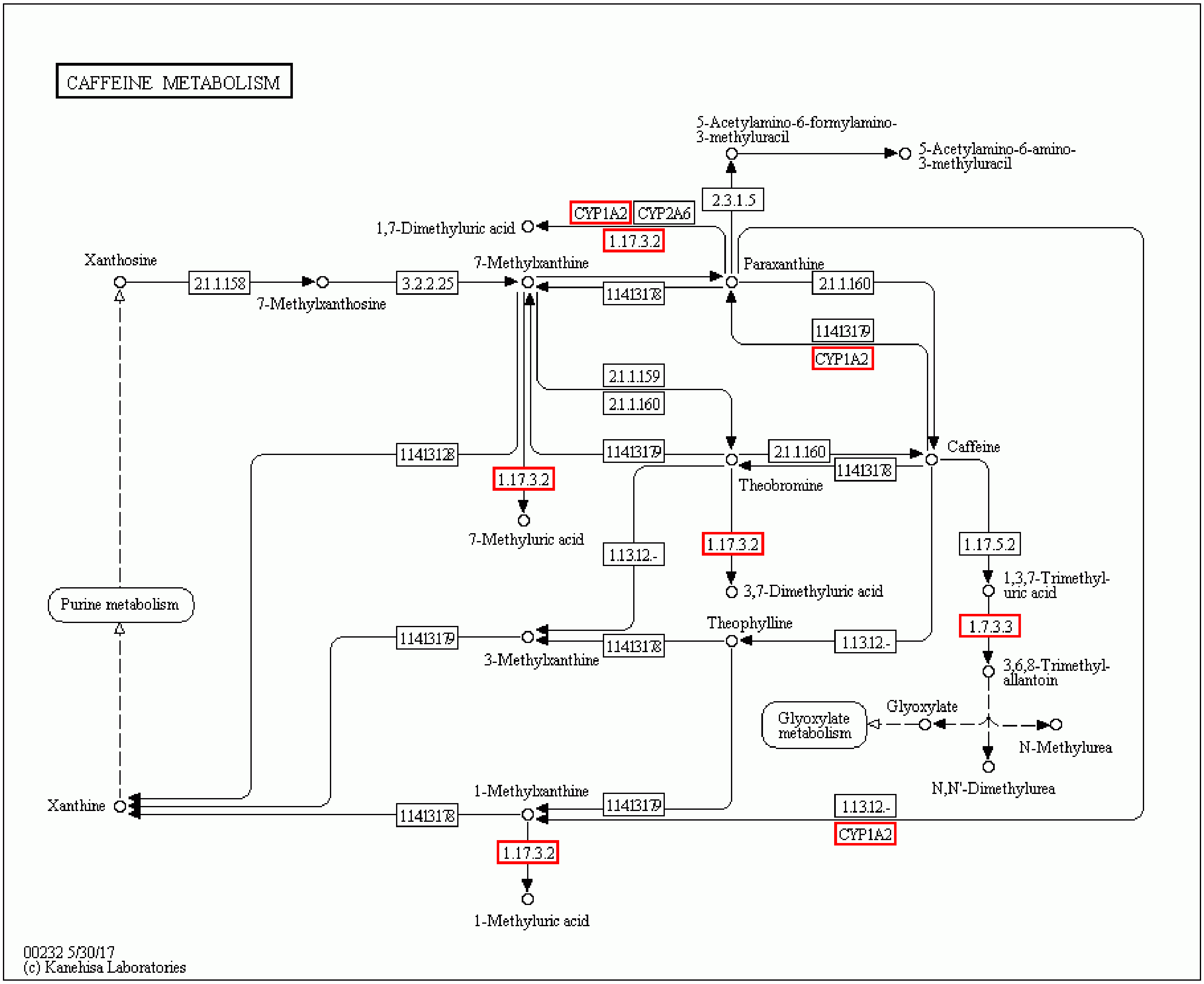

CAFFEINE METABOLISM
5-Acetylamino-6-formylamino-3-methyluracil
5-Acetylamino-6-amino-3-methyluracil
2.3.1.5
CYP1A2
CYP2A6
1,7-Dimethyluric acid
1.17.3.2
Xanthosine
2.1.1.158
7-Methylxanthosine
3.2.2.25
7-Methylxanthine
1.14.13.178
Paraxanthine
2.1.1.160
1.14.13.179
CYP1A2
2.1.1.159
2.1.1.160
1.14.13.128
1.14.13.179
2.1.1.160
Caffeine
1.14.13.178
Theobromine
1.17.3.2
7-Methyluric acid
1.13.12.-
1.17.3.2
3,7-Dimethyluric acid
1.17.5.2
1,3,7-Trimethyluric acid
Purine metabolism
Theophylline
1.7.3.3
1.14.13.179
1.14.13.178
3-Methylxanthine
1.13.12.-
3,6,8-Trimethylallantoin
Glyoxylate metabolism
Glyoxylate
N-Methylurea
N,N'-Dimethylurea
1-Methylxanthine
1.14.13.179
Xanthine
1.14.13.178
1.13.12.-
CYP1A2
1.17.3.2
1-Methyluric acid
00232 5/30/17
(c) Kanehisa Laboratories

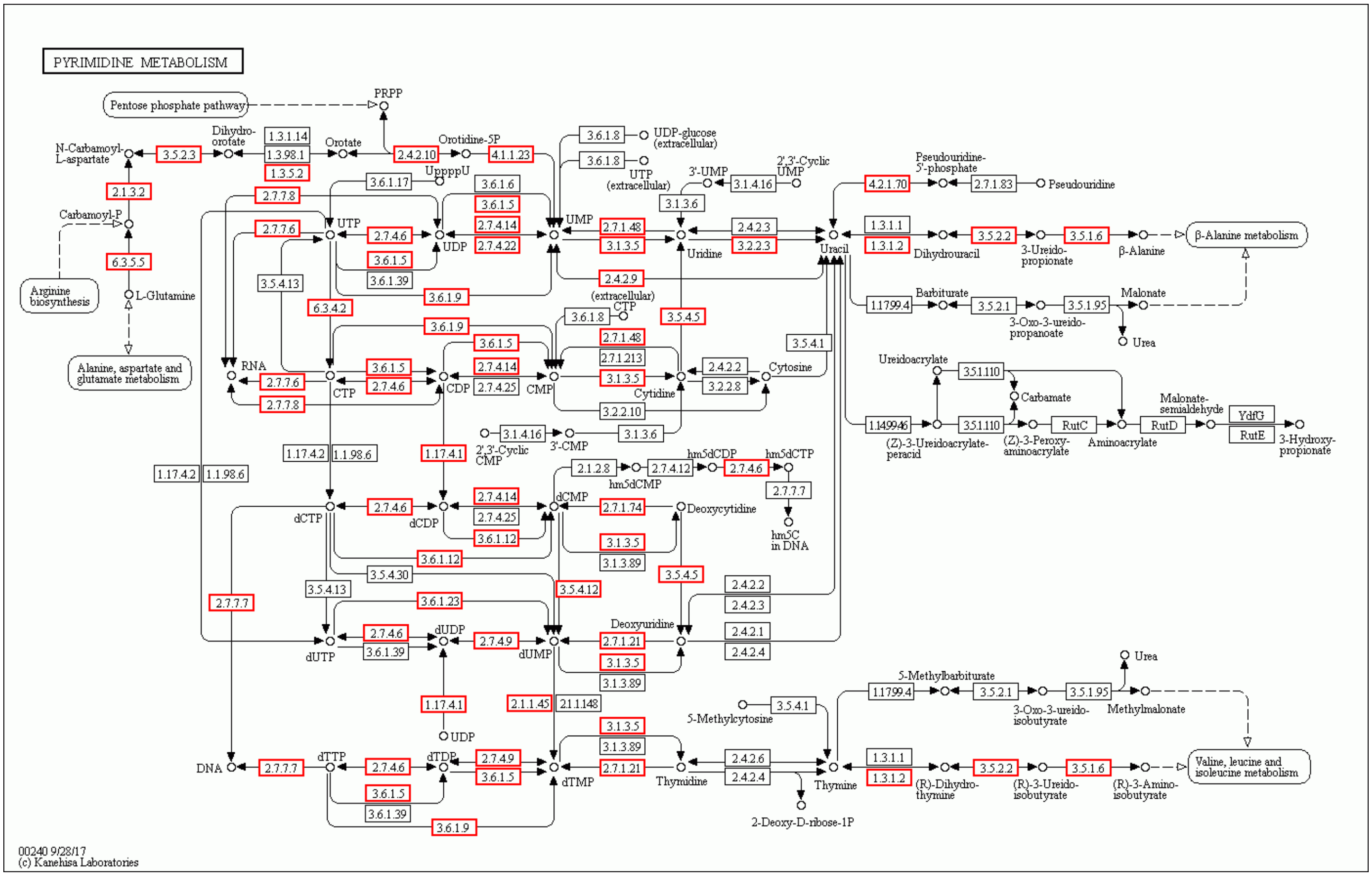
PYRIMIDINE METABOLISM
Pentose phosphate pathway
PRPP
N-Carbamoyl-L-aspartate
Dihydro-orotate
Orotate
Orotidine-5P
UppppU
UDP-glucose (extracellular)
UTP (extracellular)
3'-UMP
2',3'-Cyclic UMP
Pseudouridine-5'-phosphate
Pseudouridine
Carbamoyl-P
L-Glutamine
Arginine biosynthesis
Alanine, aspartate and glutamate metabolism
UTP
UDP
UMP
Uridine
Uracil
Dihydrouracil
3-Ureido-propionate
β-Alanine
β-Alanine metabolism
Barbiturate
3-Oxo-3-ureido-propanoate
Malonate
Urea
(extracellular) CTP
RNA
CTP
CDP
CMP
Cytidine
Cytosine
Ureidoacrylate
Carbamate
(Z)-3-Ureidoacrylate-peracid
(Z)-3-Peroxy-aminoacrylate
Aminoacrylate
Malonate-semialdehyde
3-Hydroxy-propionate
RutC
RutD
YdfG
RutE
2',3'-Cyclic CMP
3'-CMP
hm5dCDP
hm5dCTP
hm5dCMP
hm5C in DNA
dCTP
dCDP
dCMP
Deoxycytidine
dUTP
dUDP
dUMP
Deoxyuridine
5-Methylbarbiturate
3-Oxo-3-ureido-isobutyrate
Methylmalonate
5-Methylcytosine
UDP
DNA
dTTP
dTDP
dTMP
Thymidine
Thymine
(R)-Dihydro-thymine
(R)-3-Ureido-isobutyrate
(R)-3-Amino-isobutyrate
Valine, leucine and isoleucine metabolism
2-Deoxy-D-ribose-1P
3.5.2.3
1.3.1.14
1.3.98.1
1.3.5.2
2.4.2.10
4.1.1.23
2.1.3.2
6.3.5.5
2.7.7.8
2.7.7.6
6.3.4.2
3.5.4.13
3.6.1.17
3.6.1.6
3.6.1.5
2.7.4.6
2.7.4.14
2.7.4.22
3.6.1.39
3.6.1.9
3.6.1.8
3.1.4.16
3.1.3.6
2.7.1.48
3.1.3.5
2.4.2.3
3.2.2.3
2.4.2.9
3.5.4.5
4.2.1.70
2.7.1.83
1.3.1.1
1.3.1.2
3.5.2.2
3.5.1.6
1.17.99.4
3.5.2.1
3.5.1.95
3.5.4.1
2.7.1.213
2.4.2.2
3.2.2.8
3.2.2.10
3.5.1.110
1.14.99.46
1.17.4.2
1.1.98.6
1.17.4.1
2.1.2.8
2.7.4.12
2.7.7.7
2.7.1.74
3.6.1.12
2.7.4.25
3.1.3.89
3.5.4.30
3.5.4.12
3.6.1.23
2.7.4.9
2.7.1.21
2.4.2.1
2.4.2.4
2.1.1.45
2.1.1.148
2.4.2.6
00240 9/28/17
(c) Kanehisa Laboratories

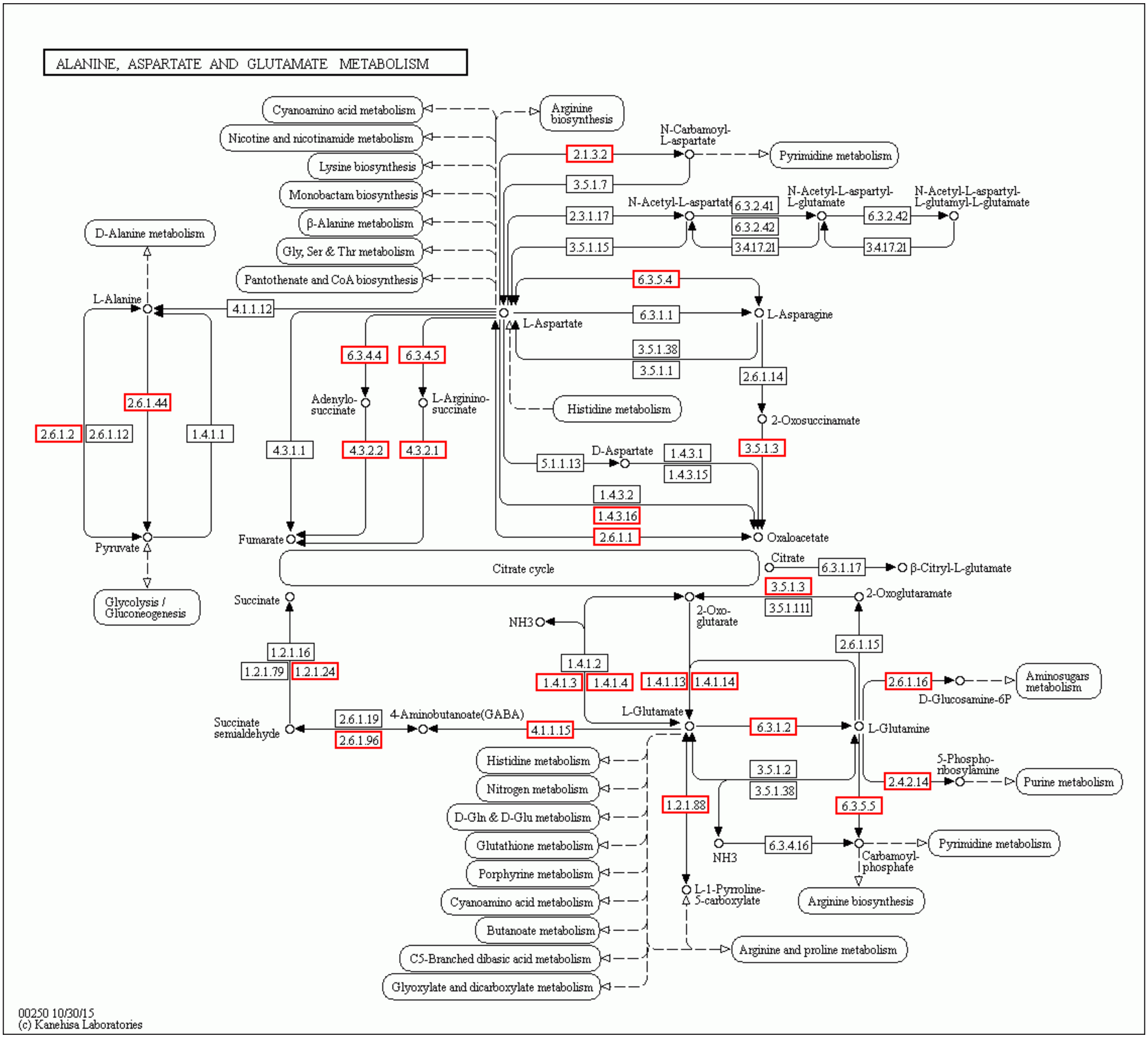
ALANINE, ASPARTATE AND GLUTAMATE METABOLISM
Cyanoamino acid metabolism
Nicotine and nicotinamide metabolism
Lysine biosynthesis
Monobactam biosynthesis
β-Alanine metabolism
Gly, Ser & Thr metabolism
Pantothenate and CoA biosynthesis
Arginine biosynthesis
N-Carbamoyl-L-aspartate
Pyrimidine metabolism
2.1.3.2
3.5.1.7
2.3.1.17
3.5.1.15
N-Acetyl-L-aspartate
6.3.2.41
6.3.2.42
3.4.17.21
N-Acetyl-L-aspartyl-L-glutamate
6.3.2.42
3.4.17.21
N-Acetyl-L-aspartyl-L-glutamyl-L-glutamate
D-Alanine metabolism
L-Alanine
4.1.1.12
6.3.5.4
L-Aspartate
6.3.1.1
L-Asparagine
3.5.1.38
3.5.1.1
6.3.4.4
6.3.4.5
Adenylo-succinate
L-Arginino-succinate
2.6.1.44
2.6.1.2
2.6.1.12
1.4.1.1
2.6.1.14
Histidine metabolism
2-Oxosuccinamate
4.3.1.1
4.3.2.2
4.3.2.1
5.1.1.13
D-Aspartate
1.4.3.1
1.4.3.15
3.5.1.3
1.4.3.2
1.4.3.16
2.6.1.1
Pyruvate
Fumarate
Oxaloacetate
Glycolysis / Gluconeogenesis
Citrate cycle
Citrate
6.3.1.17
β-Citryl-L-glutamate
3.5.1.3
3.5.1.111
2-Oxoglutaramate
Succinate
NH3
2-Oxo-glutarate
1.2.1.16
1.2.1.79
1.2.1.24
1.4.1.2
1.4.1.3
1.4.1.4
1.4.1.13
1.4.1.14
2.6.1.15
2.6.1.16
Aminosugars metabolism
D-Glucosamine-6P
Succinate semialdehyde
2.6.1.19
2.6.1.96
4-Aminobutanoate(GABA)
4.1.1.15
L-Glutamate
6.3.1.2
L-Glutamine
5-Phospho-ribosylamine
2.4.2.14
Purine metabolism
Histidine metabolism
Nitrogen metabolism
D-Gln & D-Glu metabolism
Glutathione metabolism
Porphyrine metabolism
Cyanoamino acid metabolism
Butanoate metabolism
C5-Branched dibasic acid metabolism
Glyoxylate and dicarboxylate metabolism
3.5.1.2
3.5.1.38
1.2.1.88
6.3.5.5
NH3
6.3.4.16
Carbamoyl-phosphate
Pyrimidine metabolism
L-1-Pyrroline-5-carboxylate
Arginine biosynthesis
Arginine and proline metabolism
00250 10/30/15
(c) Kanehisa Laboratories

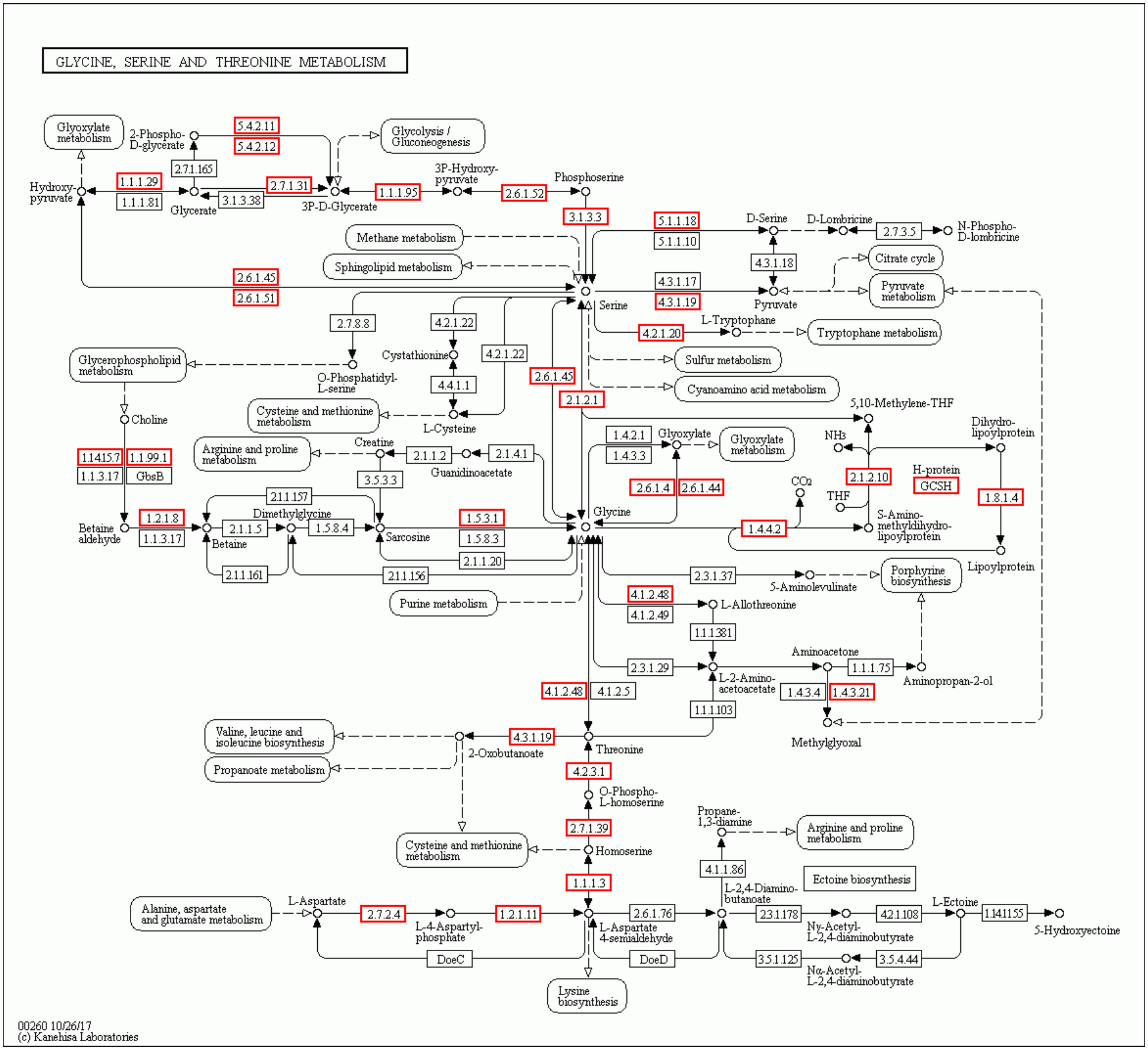
GLYCINE, SERINE AND THREONINE METABOLISM
Glyoxylate metabolism
2-Phospho-D-glycerate
5.4.2.11
5.4.2.12
Glycolysis / Gluconeogenesis
2.7.1.165
1.1.1.29
1.1.1.81
Hydroxy-pyruvate
Glycerate
3.1.3.38
2.7.1.31
3P-D-Glycerate
1.1.1.95
3P-Hydroxy-pyruvate
2.6.1.52
Phosphoserine
3.1.3.3
5.1.1.18
5.1.1.10
D-Serine
D-Lombricine
2.7.3.5
N-Phospho-D-lombricine
Methane metabolism
Sphingolipid metabolism
4.3.1.18
Citrate cycle
2.6.1.45
2.6.1.51
4.3.1.17
4.3.1.19
Pyruvate
Pyruvate metabolism
Serine
2.7.8.8
4.2.1.22
4.2.1.20
L-Tryptophane
Tryptophane metabolism
Glycerophospholipid metabolism
Cystathionine
4.2.1.22
Sulfur metabolism
O-Phosphatidyl-L-serine
4.4.1.1
2.6.1.45
Cyanoamino acid metabolism
Choline
Cysteine and methionine metabolism
L-Cysteine
2.1.2.1
5,10-Methylene-THF
Dihydro-lipoylprotein
1.1.4.15.7
1.1.99.1
Arginine and proline metabolism
Creatine
2.1.1.2
2.1.4.1
Guanidinoacetate
1.4.2.1
1.4.3.3
Glyoxylate
Glyoxylate metabolism
NH3
H-protein
GCSH
1.1.3.17
GbsB
3.5.3.3
2.1.2.10
2.1.1.157
2.6.1.4
2.6.1.44
CO2
THF
1.8.1.4
Betaine aldehyde
1.2.1.8
Dimethylglycine
1.5.3.1
Glycine
S-Amino-methyldihydro-lipoylprotein
2.1.1.5
1.5.8.4
Sarcosine
1.5.8.3
1.4.4.2
1.1.3.17
Betaine
2.1.1.20
Lipoylprotein
2.1.1.161
2.1.1.156
2.3.1.37
Porphyrine biosynthesis
5-Aminolevulinate
Purine metabolism
4.1.2.48
4.1.2.49
L-Allothreonine
1.1.1.381
Aminoacetone
2.3.1.29
L-2-Amino-acetoacetate
1.1.1.75
Aminopropan-2-ol
4.1.2.48
4.1.2.5
1.4.3.4
1.4.3.21
1.1.1.103
Valine, leucine and isoleucine biosynthesis
4.3.1.19
Threonine
Methylglyoxal
2-Oxobutanoate
Propanoate metabolism
4.2.3.1
O-Phospho-L-homoserine
2.7.1.39
Propane-1,3-diamine
Arginine and proline metabolism
Cysteine and methionine metabolism
Homoserine
4.1.1.86
Ectoine biosynthesis
1.1.1.3
L-2,4-Diamino-butanoate
Alanine, aspartate and glutamate metabolism
L-Aspartate
2.7.2.4
1.2.1.11
2.6.1.76
2.3.1.178
4.2.1.108
L-Ectoine
1.14.11.55
5-Hydroxyectoine
L-4-Aspartyl-phosphate
L-Aspartate 4-semialdehyde
Nγ-Acetyl-L-2,4-diaminobutyrate
DoeC
DoeD
3.5.1.125
3.5.4.44
Nα-Acetyl-L-2,4-diaminobutyrate
Lysine biosynthesis
00260 10/26/17
(c) Kanehisa Laboratories

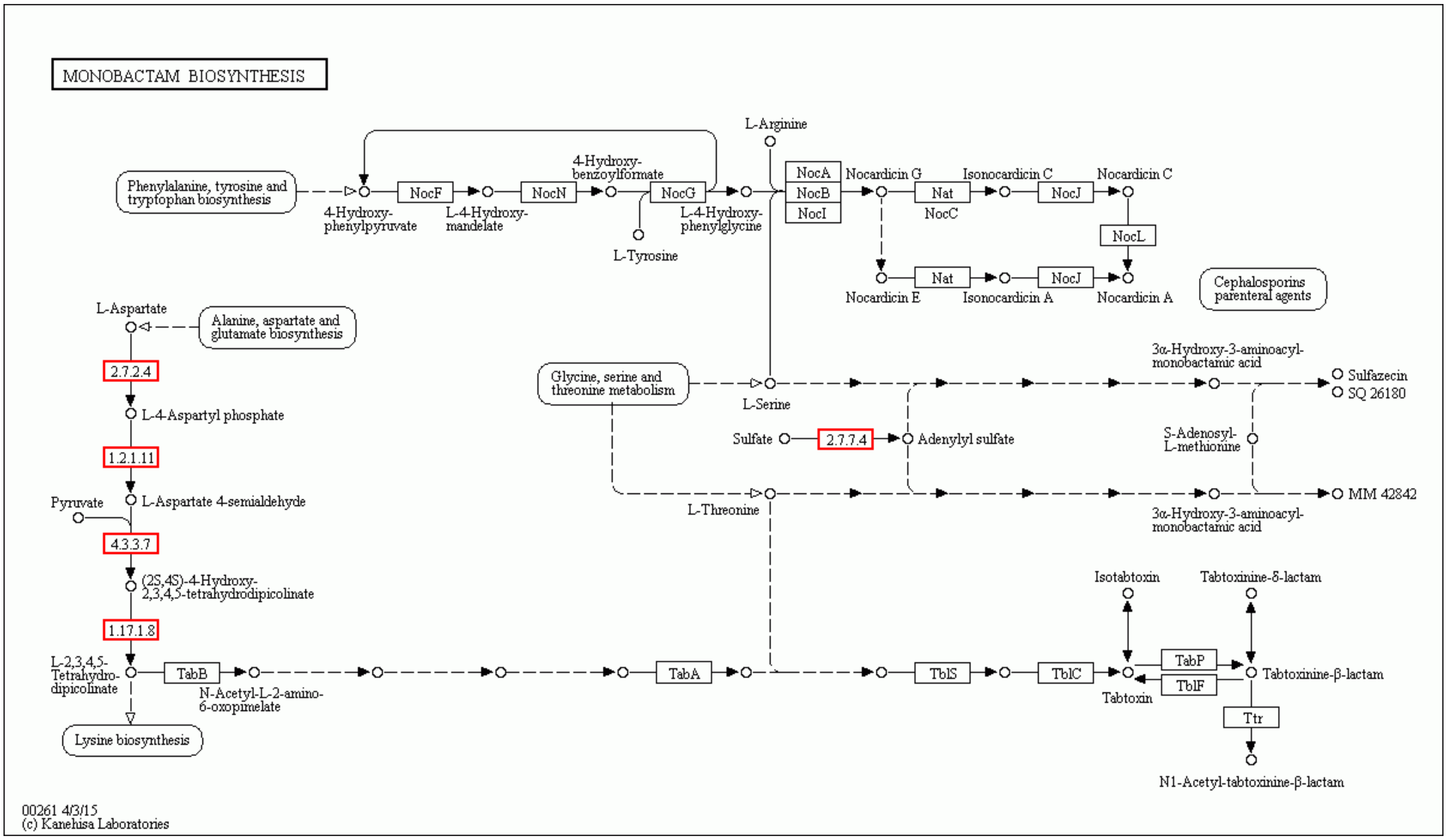

MONOBACTAM BIOSYNTHESIS
Phenylalanine, tyrosine and tryptophan biosynthesis
4-Hydroxy-phenylpyruvate
NocF
L-4-Hydroxy-mandelate
NocN
4-Hydroxy-benzoylformate
NocG
L-Tyrosine
L-4-Hydroxy-phenylglycine
L-Arginine
NocA
NocB
NocI
Nocardicin G
Nat
NocC
Isonocardicin C
NocJ
Nocardicin C
NocL
Nocardicin E
Isonocardicin A
Nocardicin A
Cephalosporins parenteral agents
L-Aspartate
Alanine, aspartate and glutamate biosynthesis
2.7.2.4
L-4-Aspartyl phosphate
1.2.1.11
L-Aspartate 4-semialdehyde
Pyruvate
4.3.3.7
(2S,4S)-4-Hydroxy-2,3,4,5-tetrahydrodipicolinate
1.17.1.8
L-2,3,4,5-Tetrahydro-dipicolinate
TabB
N-Acetyl-L-2-amino-6-oxopimelate
Lysine biosynthesis
Glycine, serine and threonine metabolism
L-Serine
Sulfate
2.7.7.4
Adenylyl sulfate
L-Threonine
3α-Hydroxy-3-aminoacyl-monobactamic acid
Sulfazecin
SQ 26180
S-Adenosyl-L-methionine
MM 42842
TabA
TblS
TblC
Isotabtoxin
Tabtoxinine-δ-lactam
TabP
TblF
Tabtoxin
Tabtoxinine-β-lactam
Ttr
N1-Acetyl-tabtoxinine-β-lactam
00261 4/3/15
(c) Kanehisa Laboratories

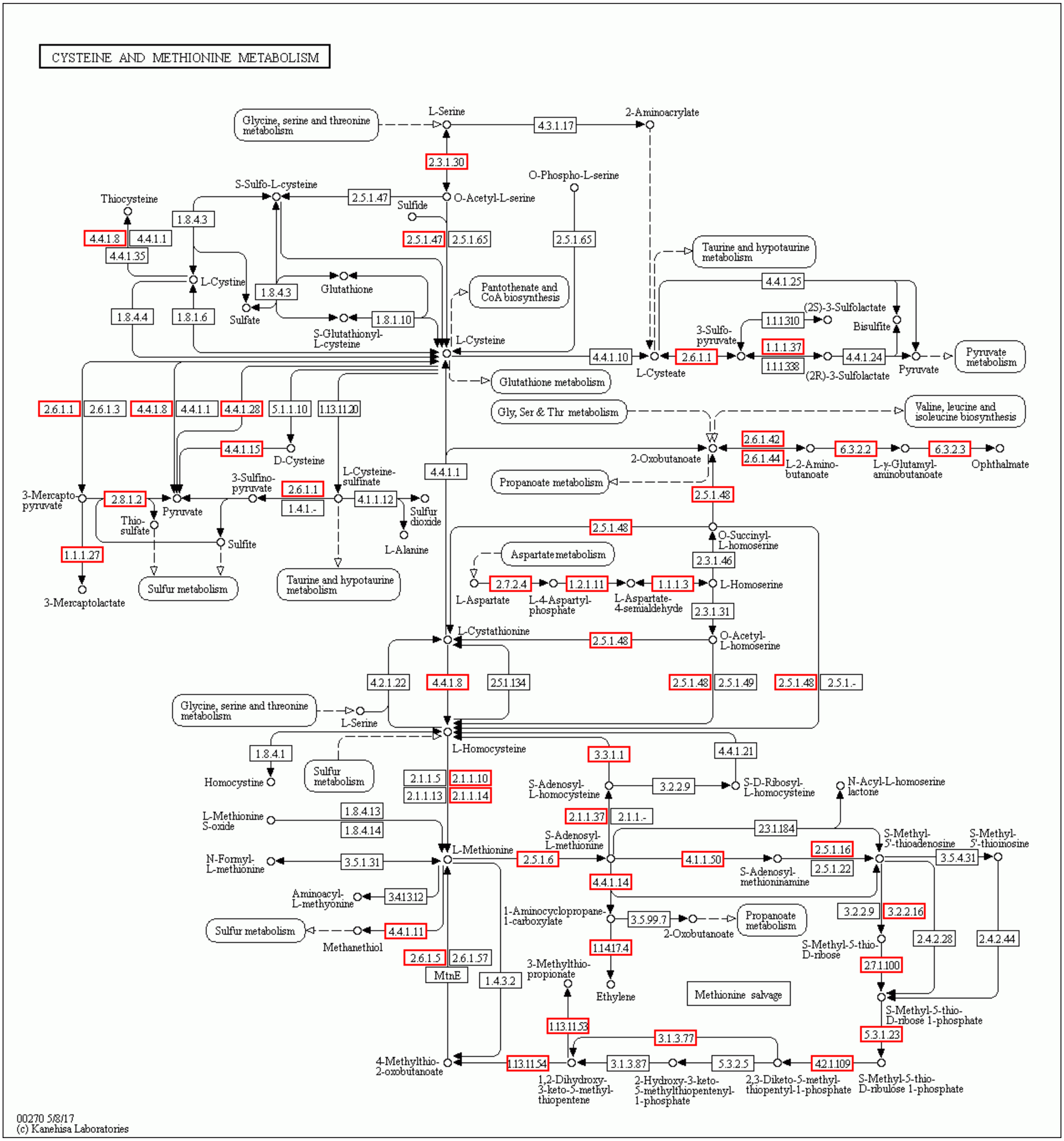
CYSTEINE AND METHIONINE METABOLISM
Glycine, serine and threonine metabolism
L-Serine
4.3.1.17
2-Aminoacrylate
2.3.1.30
S-Sulfo-L-cysteine
2.5.1.47
O-Acetyl-L-serine
O-Phospho-L-serine
Thiocysteine
1.8.4.3
Sulfide
4.4.1.8
4.4.1.1
4.4.1.35
2.5.1.47
2.5.1.65
2.5.1.65
Taurine and hypotaurine metabolism
L-Cystine
Glutathione
1.8.4.3
Pantothenate and CoA biosynthesis
4.4.1.25
1.8.4.4
1.8.1.6
Sulfate
S-Glutathionyl-L-cysteine
1.8.1.10
L-Cysteine
(2S)-3-Sulfolactate
1.1.1.310
Bisulfite
3-Sulfo-pyruvate
1.1.1.37
4.4.1.10
2.6.1.1
1.1.1.338
4.4.1.24
Pyruvate metabolism
L-Cysteate
(2R)-3-Sulfolactate
Pyruvate
Glutathione metabolism
2.6.1.1
2.6.1.3
4.4.1.8
4.4.1.1
4.4.1.28
5.1.1.10
1.13.11.20
Gly, Ser & Thr metabolism
Valine, leucine and isoleucine biosynthesis
2.6.1.42
6.3.2.2
6.3.2.3
4.4.1.15
D-Cysteine
2-Oxobutanoate
2.6.1.44
L-2-Amino-butanoate
L-γ-Glutamyl-aminobutanoate
Ophthalmate
L-Cysteine-sulfinate
4.4.1.1
Propanoate metabolism
3-Sulfino-pyruvate
2.6.1.1
3-Mercapto-pyruvate
2.8.1.2
Pyruvate
1.4.1.-
4.1.1.12
Sulfur dioxide
2.5.1.48
Thio-sulfate
Sulfite
L-Alanine
2.5.1.48
O-Succinyl-L-homoserine
1.1.1.27
2.3.1.46
Aspartate metabolism
Sulfur metabolism
Taurine and hypotaurine metabolism
2.7.2.4
1.2.1.11
1.1.1.3
L-Homoserine
3-Mercaptolactate
L-Aspartate
L-4-Aspartyl-phosphate
L-Aspartate-4-semialdehyde
2.3.1.31
L-Cystathionine
2.5.1.48
O-Acetyl-L-homoserine
4.2.1.22
4.4.1.8
2.5.1.134
2.5.1.48
2.5.1.49
2.5.1.48
2.5.1.-
Glycine, serine and threonine metabolism
L-Serine
L-Homocysteine
1.8.4.1
4.4.1.21
3.3.1.1
Sulfur metabolism
Homocystine
2.1.1.5
2.1.1.10
S-Adenosyl-L-homocysteine
3.2.2.9
S-D-Ribosyl-L-homocysteine
N-Acyl-L-homoserine lactone
2.1.1.13
2.1.1.14
1.8.4.13
2.1.1.37
2.1.1.-
L-Methionine S-oxide
1.8.4.14
2.3.1.184
S-Adenosyl-L-methionine
S-Methyl-5'-thioadenosine
S-Methyl-5'-thioinosine
L-Methionine
2.5.1.16
N-Formyl-L-methionine
3.5.1.31
2.5.1.6
4.1.1.50
S-Adenosyl-methioninamine
3.5.4.31
2.5.1.22
4.4.1.14
Aminoacyl-L-methyonine
3.4.13.12
1-Aminocyclopropane-1-carboxylate
3.5.99.7
2-Oxobutanoate
Propanoate metabolism
3.2.2.9
3.2.2.16
Sulfur metabolism
4.4.1.11
S-Methyl-5-thio-D-ribose
2.4.2.28
2.4.2.44
Methanethiol
1.14.17.4
2.6.1.5
2.6.1.57
3-Methylthio-propionate
2.7.1.100
MtnE
1.4.3.2
Ethylene
Methionine salvage
S-Methyl-5-thio-D-ribose 1-phosphate
1.13.11.53
3.1.3.77
5.3.1.23
4-Methylthio-2-oxobutanoate
1.13.11.54
3.1.3.87
5.3.2.5
4.2.1.109
1,2-Dihydroxy-3-keto-5-methyl-thiopentene
2-Hydroxy-3-keto-5-methylthiopentenyl-1-phosphate
2,3-Diketo-5-methyl-thiopentyl-1-phosphate
S-Methyl-5-thio-D-ribulose 1-phosphate
00270 5/8/17
(c) Kanehisa Laboratories

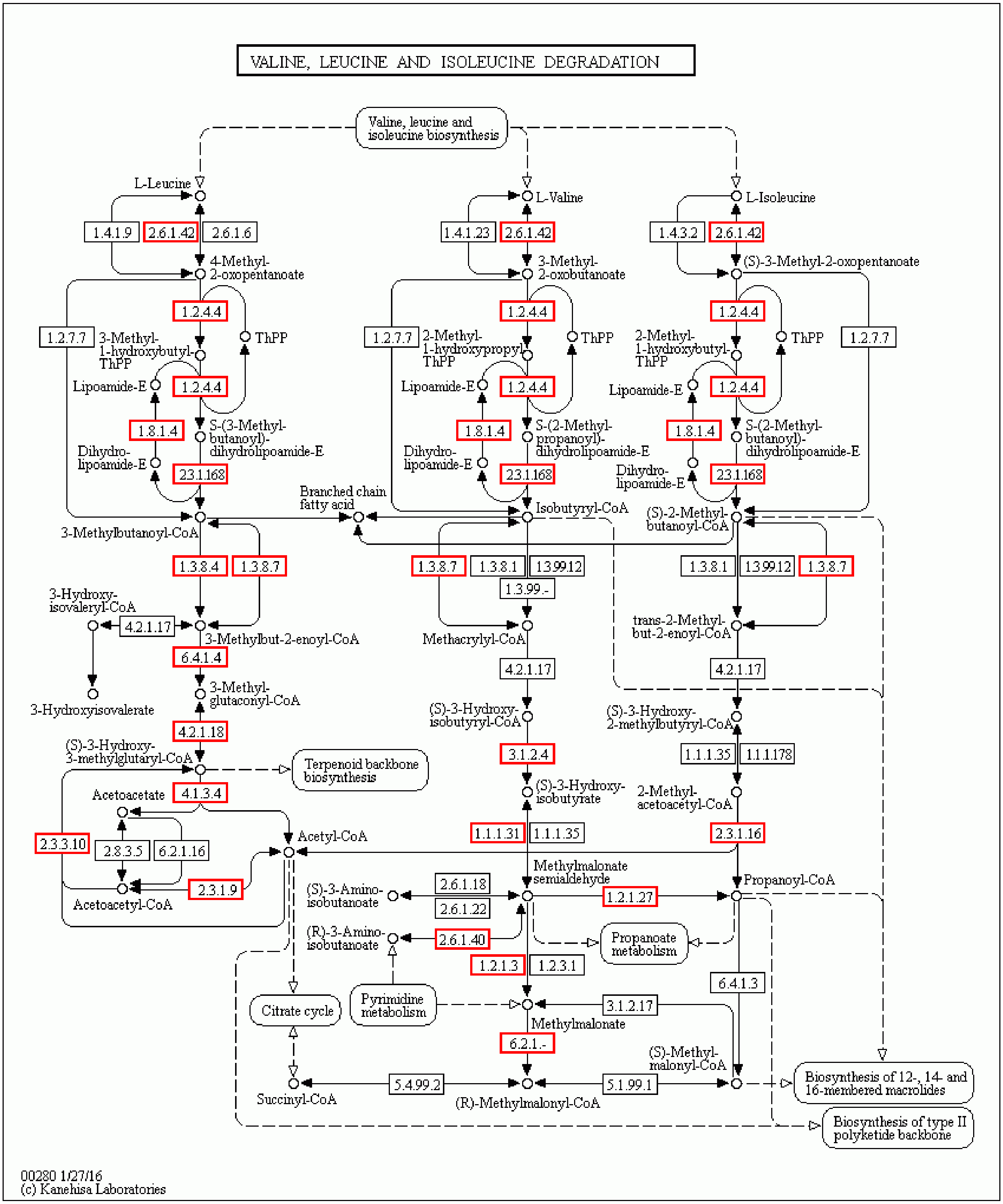

VALINE, LEUCINE AND ISOLEUCINE DEGRADATION
Valine, leucine and isoleucine biosynthesis
L-Leucine
L-Valine
L-Isoleucine
1.4.1.9
2.6.1.42
2.6.1.6
1.4.1.23
1.4.3.2
4-Methyl-2-oxopentanoate
3-Methyl-2-oxobutanoate
(S)-3-Methyl-2-oxopentanoate
1.2.4.4
1.2.7.7
ThPP
3-Methyl-1-hydroxybutyl-ThPP
2-Methyl-1-hydroxypropyl-ThPP
2-Methyl-1-hydroxybutyl-ThPP
Lipoamide-E
1.8.1.4
S-(3-Methylbutanoyl)-dihydrolipoamide-E
S-(2-Methylpropanoyl)-dihydrolipoamide-E
S-(2-Methylbutanoyl)-dihydrolipoamide-E
Dihydrolipoamide-E
2.3.1.168
Branched chain fatty acid
3-Methylbutanoyl-CoA
Isobutyryl-CoA
(S)-2-Methylbutanoyl-CoA
1.3.8.4
1.3.8.7
1.3.8.1
1.3.99.12
1.3.99.-
3-Hydroxyisovaleryl-CoA
4.2.1.17
3-Methylbut-2-enoyl-CoA
Methacrylyl-CoA
trans-2-Methylbut-2-enoyl-CoA
6.4.1.4
3-Hydroxyisovalerate
3-Methylglutaconyl-CoA
4.2.1.18
(S)-3-Hydroxyisobutyryl-CoA
(S)-3-Hydroxy-2-methylbutyryl-CoA
(S)-3-Hydroxy-3-methylglutaryl-CoA
Terpenoid backbone biosynthesis
3.1.2.4
1.1.1.35
1.1.1.178
Acetoacetate
4.1.3.4
(S)-3-Hydroxyisobutyrate
2-Methylacetoacetyl-CoA
2.3.3.10
2.8.3.5
6.2.1.16
Acetyl-CoA
1.1.1.31
2.3.1.16
Methylmalonate semialdehyde
Propanoyl-CoA
2.3.1.9
Acetoacetyl-CoA
(S)-3-Aminoisobutanoate
2.6.1.18
2.6.1.22
1.2.1.27
(R)-3-Aminoisobutanoate
2.6.1.40
Propanoate metabolism
1.2.1.3
1.2.3.1
6.4.1.3
Citrate cycle
Pyrimidine metabolism
3.1.2.17
Methylmalonate
6.2.1.-
(S)-Methylmalonyl-CoA
Biosynthesis of 12-, 14- and 16-membered macrolides
Succinyl-CoA
5.4.99.2
(R)-Methylmalonyl-CoA
5.1.99.1
Biosynthesis of type II polyketide backbone
00280 1/27/16
(c) Kanehisa Laboratories

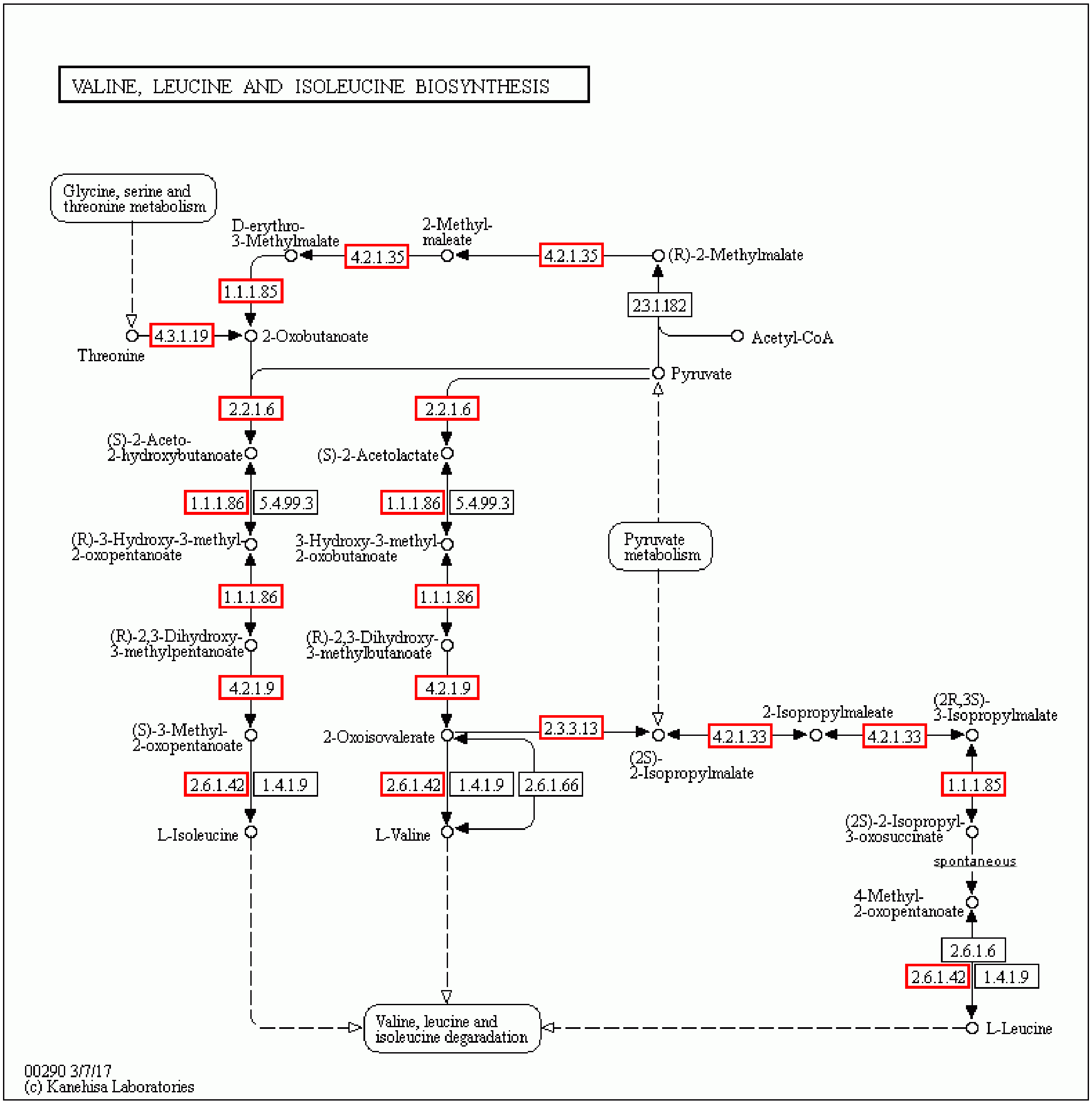

VALINE, LEUCINE AND ISOLEUCINE BIOSYNTHESIS
Glycine, serine and threonine metabolism
D-erythro-3-Methylmalate
2-Methyl-maleate
4.2.1.35
4.2.1.35
(R)-2-Methylmalate
1.1.1.85
2.3.1.182
Threonine
4.3.1.19
2-Oxobutanoate
Acetyl-CoA
Pyruvate
2.2.1.6
2.2.1.6
(S)-2-Aceto-2-hydroxybutanoate
(S)-2-Acetolactate
1.1.1.86
5.4.99.3
1.1.1.86
5.4.99.3
(R)-3-Hydroxy-3-methyl-2-oxopentanoate
3-Hydroxy-3-methyl-2-oxobutanoate
Pyruvate metabolism
1.1.1.86
1.1.1.86
(R)-2,3-Dihydroxy-3-methylpentanoate
(R)-2,3-Dihydroxy-3-methylbutanoate
4.2.1.9
4.2.1.9
(S)-3-Methyl-2-oxopentanoate
2-Oxoisovalerate
2.3.3.13
(2S)-2-Isopropylmalate
4.2.1.33
2-Isopropylmaleate
4.2.1.33
(2R,3S)-3-Isopropylmalate
2.6.1.42
1.4.1.9
2.6.1.42
1.4.1.9
2.6.1.66
1.1.1.85
L-Isoleucine
L-Valine
(2S)-2-Isopropyl-3-oxosuccinate
spontaneous
4-Methyl-2-oxopentanoate
2.6.1.6
2.6.1.42
1.4.1.9
Valine, leucine and isoleucine degaradation
L-Leucine
00290 3/7/17
(c) Kanehisa Laboratories

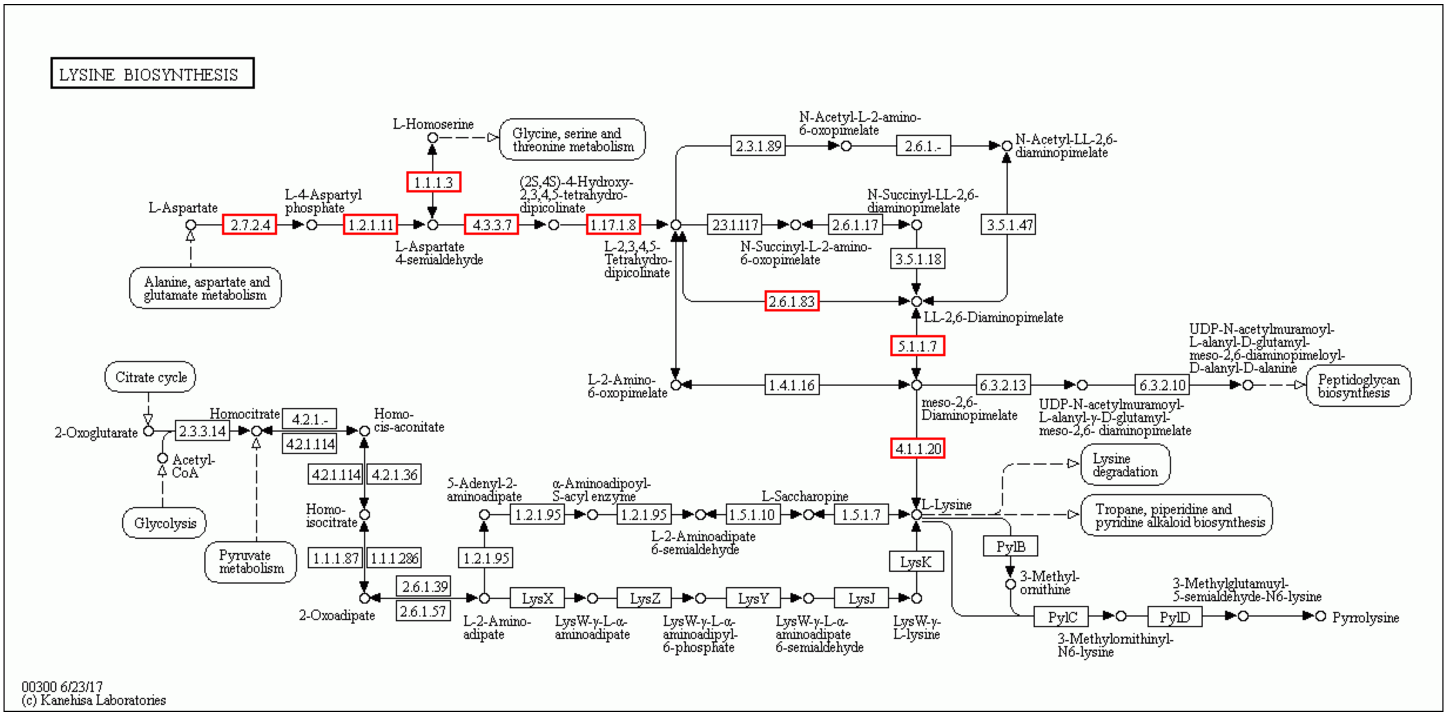
LYSINE BIOSYNTHESIS
L-Homoserine
Glycine, serine and threonine metabolism
N-Acetyl-L-2-amino-6-oxopimelate
2.3.1.89
2.6.1.-
N-Acetyl-LL-2,6-diaminopimelate
1.1.1.3
(2S,4S)-4-Hydroxy-2,3,4,5-tetrahydro-dipicolinate
L-Aspartate
2.7.2.4
L-4-Aspartyl phosphate
1.2.1.11
4.3.3.7
1.17.1.8
2.3.1.117
2.6.1.17
N-Succinyl-LL-2,6-diaminopimelate
3.5.1.47
L-Aspartate 4-semialdehyde
L-2,3,4,5-Tetrahydro-dipicolinate
N-Succinyl-L-2-amino-6-oxopimelate
3.5.1.18
Alanine, aspartate and glutamate metabolism
2.6.1.83
LL-2,6-Diaminopimelate
5.1.1.7
UDP-N-acetylmuramoyl-L-alanyl-D-glutamyl-meso-2,6-diaminopimeloyl-D-alanyl-D-alanine
Citrate cycle
L-2-Amino-6-oxopimelate
1.4.1.16
6.3.2.13
6.3.2.10
Peptidoglycan biosynthesis
Homocitrate
4.2.1.-
Homo-cis-aconitate
meso-2,6-Diaminopimelate
UDP-N-acetylmuramoyl-L-alanyl-γ-D-glutamyl-meso-2,6- diaminopimelate
2-Oxoglutarate
2.3.3.14
4.2.1.114
Acetyl-CoA
4.2.1.114
4.2.1.36
4.1.1.20
Lysine degradation
5-Adenyl-2-aminoadipate
α-Aminoadipoyl-S-acyl enzyme
L-Saccharopine
L-Lysine
Glycolysis
Homo-isocitrate
1.2.1.95
1.2.1.95
1.5.1.10
1.5.1.7
Tropane, piperidine and pyridine alkaloid biosynthesis
L-2-Aminoadipate 6-semialdehyde
PylB
Pyruvate metabolism
1.1.1.87
1.1.1.286
1.2.1.95
LysK
3-Methyl-ornithine
2.6.1.39
3-Methylglutamoyl-5-semialdehyde-N6-lysine
LysX
LysZ
LysY
LysJ
2.6.1.57
PylC
PylD
Pyrrolysine
2-Oxoadipate
L-2-Amino-adipate
LysW-γ-L-α-aminoadipate
LysW-γ-L-α-aminoadipyl-6-phosphate
LysW-γ-L-α-aminoadipate 6-semialdehyde
LysW-γ-L-lysine
3-Methylornithinyl-N6-lysine
00300 6/23/17
(c) Kanehisa Laboratories

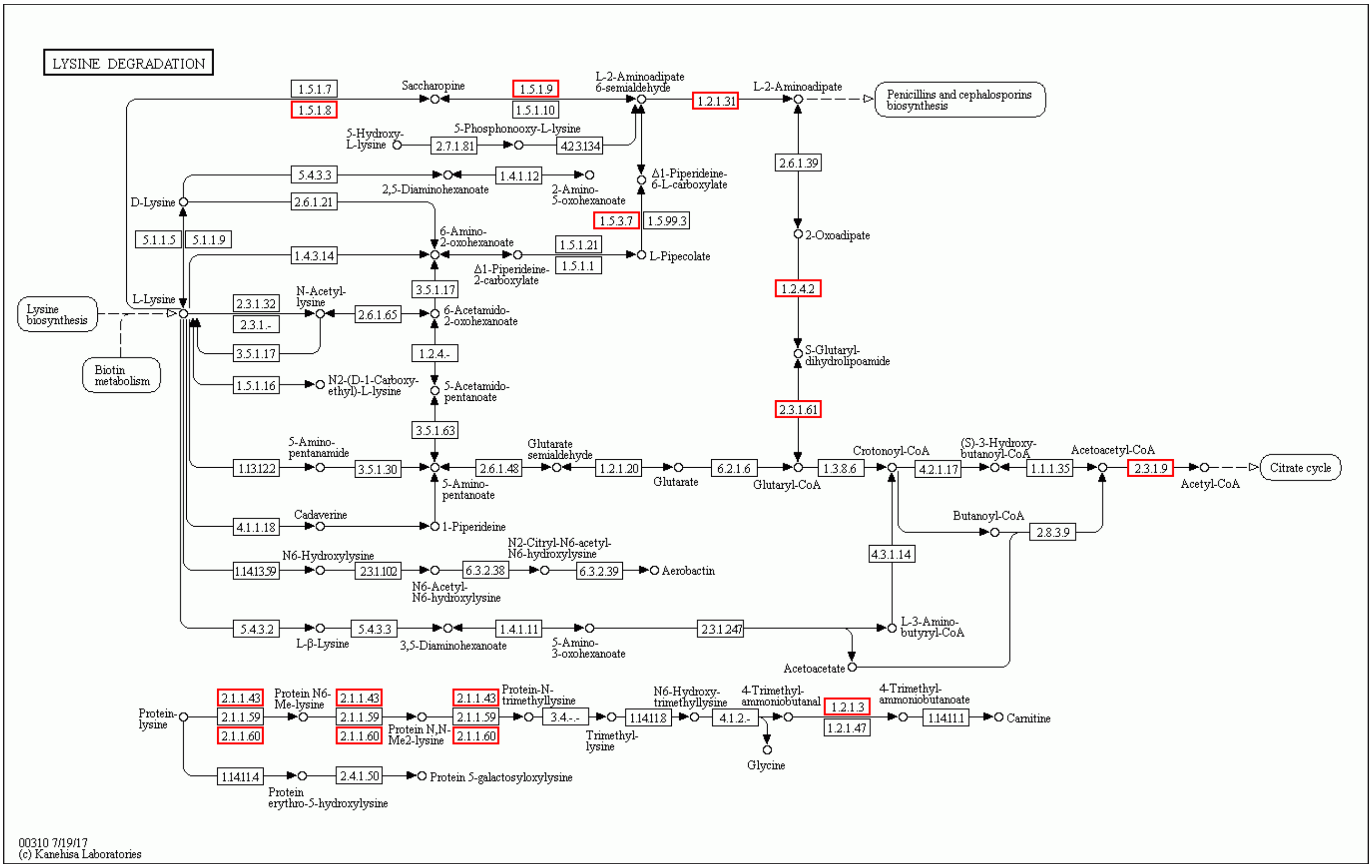
LYSINE DEGRADATION
1.5.1.7
Saccharopine
1.5.1.9
L-2-Aminoadipate 6-semialdehyde
L-2-Aminoadipate
Penicillins and cephalosporins biosynthesis
1.5.1.8
1.5.1.10
1.2.1.31
5-Hydroxy-L-lysine
5-Phosphonooxy-L-lysine
2.7.1.81
4.2.3.134
5.4.3.3
1.4.1.12
Δ1-Piperideine-6-L-carboxylate
2.6.1.39
D-Lysine
2,5-Diaminohexanoate
2-Amino-5-oxohexanoate
2.6.1.21
6-Amino-2-oxohexanoate
1.5.3.7
1.5.99.3
2-Oxoadipate
5.1.1.5
5.1.1.9
1.5.1.21
1.4.3.14
1.5.1.1
L-Pipecolate
Δ1-Piperideine-2-carboxylate
3.5.1.17
1.2.4.2
L-Lysine
2.3.1.32
N-Acetyl-lysine
Lysine biosynthesis
2.3.1.-
2.6.1.65
6-Acetamido-2-oxohexanoate
3.5.1.17
1.2.4.-
S-Glutaryl-dihydrolipoamide
Biotin metabolism
1.5.1.16
N2-(D-1-Carboxy-ethyl)-L-lysine
5-Acetamido-pentanoate
3.5.1.63
2.3.1.61
5-Amino-pentanamide
Glutarate semialdehyde
Crotonoyl-CoA
(S)-3-Hydroxy-butanoyl-CoA
Acetoacetyl-CoA
1.13.12.2
3.5.1.30
2.6.1.48
1.2.1.20
6.2.1.6
1.3.8.6
4.2.1.17
1.1.1.35
2.3.1.9
Citrate cycle
5-Amino-pentanoate
Glutarate
Glutaryl-CoA
Acetyl-CoA
Cadaverine
Butanoyl-CoA
4.1.1.18
1-Piperideine
2.8.3.9
N6-Hydroxylysine
N2-Citryl-N6-acetyl-N6-hydroxylysine
4.3.1.14
1.14.13.59
2.3.1.102
6.3.2.38
6.3.2.39
Aerobactin
N6-Acetyl-N6-hydroxylysine
L-3-Amino-butyryl-CoA
5.4.3.2
5.4.3.3
1.4.1.11
2.3.1.247
L-β-Lysine
3,5-Diaminohexanoate
5-Amino-3-oxohexanoate
Acetoacetate
Protein N6-Me-lysine
Protein-N-trimethyllysine
N6-Hydroxy-trimethyllysine
4-Trimethyl-ammoniobutanal
4-Trimethyl-ammoniobutanoate
2.1.1.43
2.1.1.43
2.1.1.43
Protein-lysine
2.1.1.59
2.1.1.59
2.1.1.59
3.4.-.-
1.14.11.8
4.1.2.-
1.2.1.3
1.14.11.1
Carnitine
2.1.1.60
2.1.1.60
2.1.1.60
1.2.1.47
Protein N,N-Me2-lysine
Trimethyl-lysine
Glycine
1.14.11.4
2.4.1.50
Protein 5-galactosyloxylysine
Protein erythro-5-hydroxylysine
00310 7/19/17
(c) Kanehisa Laboratories

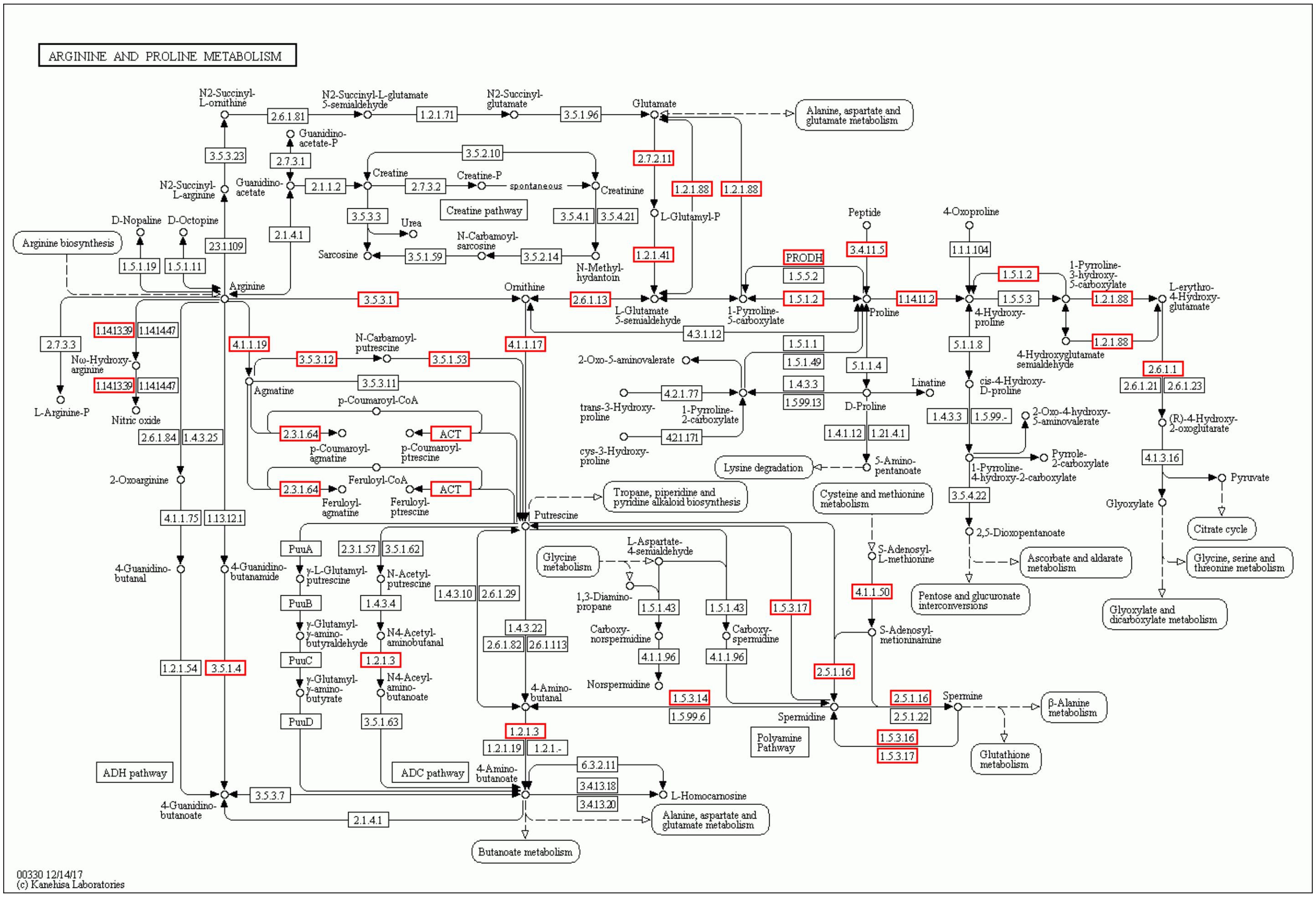

ARGININE AND PROLINE METABOLISM
N2-Succinyl-L-ornithine
2.6.1.81
N2-Succinyl-L-glutamate 5-semialdehyde
1.2.1.71
N2-Succinyl-glutamate
3.5.1.96
Glutamate
Alanine, aspartate and glutamate metabolism
Guanidino-acetate-P
3.5.3.23
2.7.3.1
3.5.2.10
Creatine
Creatine-P
2.7.2.11
N2-Succinyl-L-arginine
Guanidino-acetate
2.1.4.2
2.7.3.2
spontaneous
Creatinine
1.2.1.88
1.2.1.88
Creatine pathway
3.5.3.3
3.5.4.1
3.5.4.21
L-Glutamyl-P
Peptide
4-Oxoproline
D-Nopaline
D-Octopine
Urea
2.1.4.1
N-Carbamoyl-sarcosine
1.1.1.104
Arginine biosynthesis
23.1.109
Sarcosine
3.5.1.59
3.5.2.14
1.2.1.41
3.4.11.5
PRODH
1.5.1.19
1.5.1.11
N-Methyl-hydantoin
1.5.5.2
1.5.1.2
1-Pyrroline-3-hydroxy-5-carboxylate
Arginine
Ornithine
3.5.3.1
2.6.1.13
1.5.1.2
1.14.11.2
1.5.5.3
1.2.1.88
L-erythro-4-Hydroxy-glutamate
L-Glutamate 5-semialdehyde
1-Pyrroline-5-carboxylate
Proline
4-Hydroxy-proline
1.14.13.39
1.14.14.47
4.3.1.12
2.7.3.3
4.1.1.19
N-Carbamoyl-putrescine
4.1.1.17
1.5.1.1
5.1.1.8
1.2.1.88
4-Hydroxyglutamate semialdehyde
Nω-Hydroxy-arginine
3.5.3.12
3.5.1.53
2-Oxo-5-aminovalerate
1.5.1.49
5.1.1.4
2.6.1.1
1.14.13.39
1.14.14.47
3.5.3.11
Agmatine
4.2.1.77
1.4.3.3
Linatine
cis-4-Hydroxy-D-proline
2.6.1.21
2.6.1.23
L-Arginine-P
Nitric oxide
p-Coumaroyl-CoA
trans-3-Hydroxy-proline
1-Pyrroline-2-carboxylate
1.5.99.13
D-Proline
1.4.3.3
1.5.99.-
2-Oxo-4-hydroxy-5-aminovalerate
(R)-4-Hydroxy-2-oxoglutarate
2.3.1.64
ACT
2.6.1.84
1.4.3.25
p-Coumaroyl-agmatine
p-Coumaroyl-ptrescine
4.2.1.171
1.4.1.12
1.21.4.1
cys-3-Hydroxy-proline
Pyrrole-2-carboxylate
4.1.3.16
Lysine degradation
5-Amino-pentanoate
1-Pyrroline-4-hydroxy-2-carboxylate
Pyruvate
2-Oxoarginine
Feruloyl-CoA
2.3.1.64
ACT
Feruloyl-agmatine
Feruloyl-ptrescine
Tropane, piperidine and pyridine alkaloid biosynthesis
Cysteine and methionine metabolism
3.5.4.22
Glyoxylate
4.1.1.75
1.13.12.1
Putrescine
Citrate cycle
2,5-Dioxopentanoate
PuuA
2.3.1.57
3.5.1.62
Glycine metabolism
L-Aspartate-4-semialdehyde
S-Adenosyl-L-methionine
Ascorbate and aldarate metabolism
Glycine, serine and threonine metabolism
4-Guanidino-butanal
4-Guanidino-butanamide
γ-L-Glutamyl-putrescine
N-Acetyl-putrescine
1.4.3.10
2.6.1.29
1,3-Diamino-propane
Pentose and glucuronate interconversions
PuuB
1.4.3.4
1.5.1.43
1.5.1.43
1.5.3.17
4.1.1.50
Glyoxylate and dicarboxylate metabolism
γ-Glutamyl-γ-amino-butyraldehyde
N4-Acetyl-aminobutanal
1.4.3.22
Carboxy-norspermidine
Carboxy-spermidine
S-Adenosyl-methioninamine
2.6.1.82
2.6.1.113
1.2.1.54
3.5.1.4
PuuC
1.2.1.3
4.1.1.96
4.1.1.96
2.5.1.16
γ-Glutamyl-γ-amino-butyrate
N4-Acetyl-amino-butanoate
4-Amino-butanal
Norspermidine
1.5.3.14
2.5.1.16
Spermine
β-Alanine metabolism
1.5.99.6
Spermidine
2.5.1.22
PuuD
3.5.1.63
1.2.1.3
Polyamine Pathway
1.5.3.16
1.5.3.17
Glutathione metabolism
1.2.1.19
1.2.1.-
ADH pathway
ADC pathway
4-Amino-butanoate
6.3.2.11
3.4.13.18
3.4.13.20
L-Homocarnosine
3.5.3.7
4-Guanidino-butanoate
2.1.4.1
Alanine, aspartate and glutamate metabolism
Butanoate metabolism
00330 12/14/17
(c) Kanehisa Laboratories

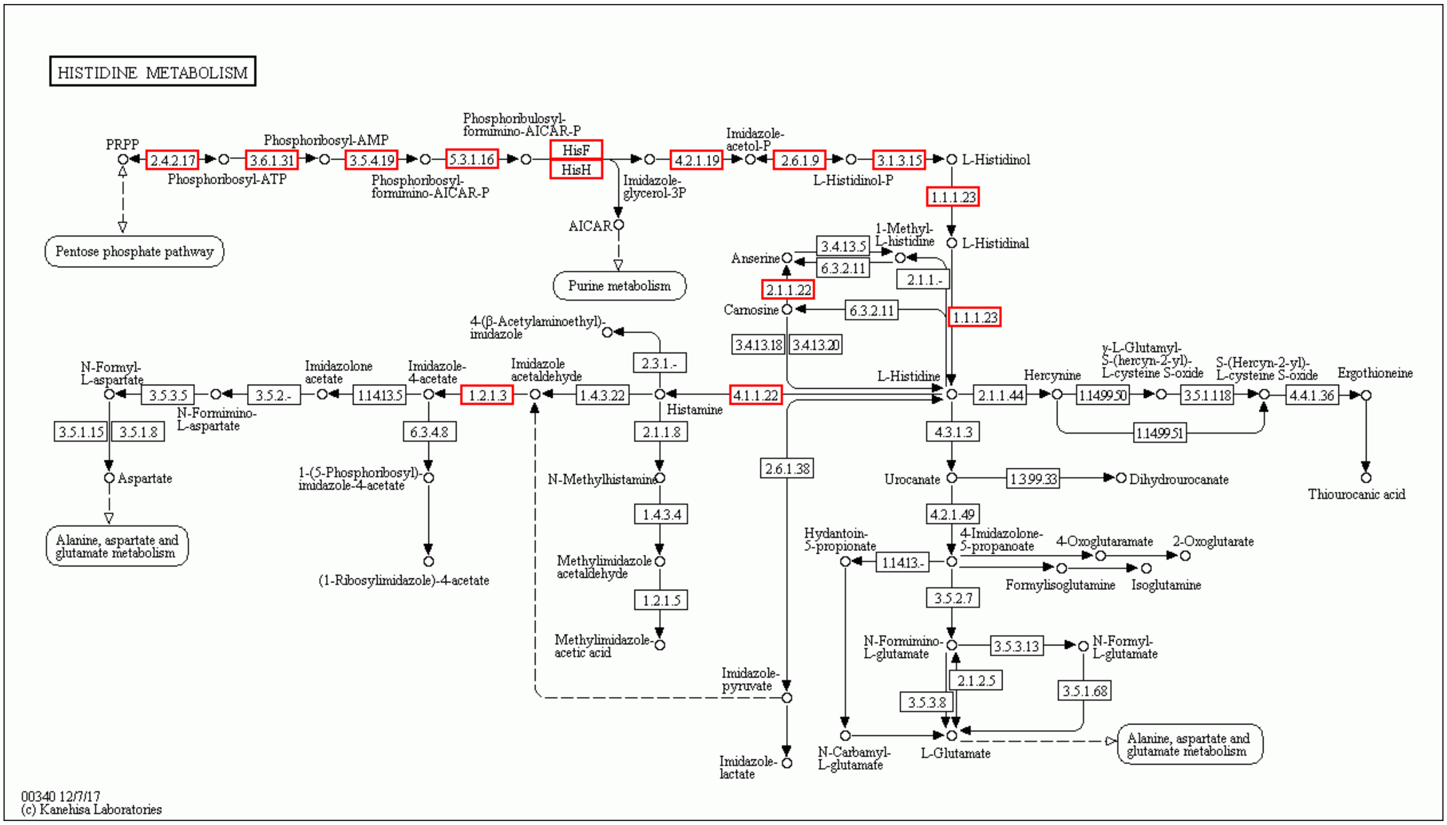

HISTIDINE METABOLISM
PRPP
2.4.2.17
Phosphoribosyl-AMP
3.6.1.31
3.5.4.19
Phosphoribulosyl-formimino-AICAR-P
5.3.1.16
HisF
HisH
Imidazole-acetol-P
4.2.1.19
2.6.1.9
3.1.3.15
L-Histidinol
Phosphoribosyl-ATP
Phosphoribosyl-formimino-AICAR-P
Imidazole-glycerol-3P
L-Histidinol-P
1.1.1.23
AICAR
Pentose phosphate pathway
1-Methyl-L-histidine
3.4.13.5
L-Histidinal
Anserine
6.3.2.11
2.1.1.-
Purine metabolism
2.1.1.22
Carnosine
6.3.2.11
1.1.1.23
4-(β-Acetylaminoethyl)-imidazole
3.4.13.18
3.4.13.20
γ-L-Glutamyl-S-(hercyn-2-yl)-L-cysteine S-oxide
S-(Hercyn-2-yl)-L-cysteine S-oxide
N-Formyl-L-aspartate
Imidazolone acetate
Imidazole-4-acetate
Imidazole acetaldehyde
2.3.1.-
L-Histidine
Hercynine
Ergothioneine
3.5.3.5
3.5.2.-
1.14.13.5
1.2.1.3
1.4.3.22
4.1.1.22
2.1.1.44
1.14.99.50
3.5.1.118
4.4.1.36
N-Formimino-L-aspartate
Histamine
3.5.1.15
3.5.1.8
6.3.4.8
2.1.1.8
4.3.1.3
1.14.99.51
2.6.1.38
Aspartate
1-(5-Phosphoribosyl)-imidazole-4-acetate
N-Methylhistamine
Urocanate
1.3.99.33
Dihydrourocanate
Thiourocanic acid
1.4.3.4
4.2.1.49
Alanine, aspartate and glutamate metabolism
Hydantoin-5-propionate
4-Imidazolone-5-propanoate
4-Oxoglutaramate
2-Oxoglutarate
Methylimidazole acetaldehyde
1.14.13.-
Formylisoglutamine
Isoglutamine
(1-Ribosylimidazole)-4-acetate
1.2.1.5
3.5.2.7
N-Formimino-L-glutamate
3.5.3.13
N-Formyl-L-glutamate
Methylimidazole-acetic acid
2.1.2.5
Imidazole-pyruvate
3.5.1.68
3.5.3.8
N-Carbamyl-L-glutamate
L-Glutamate
Alanine, aspartate and glutamate metabolism
Imidazole-lactate
00340 12/7/17
(c) Kanehisa Laboratories

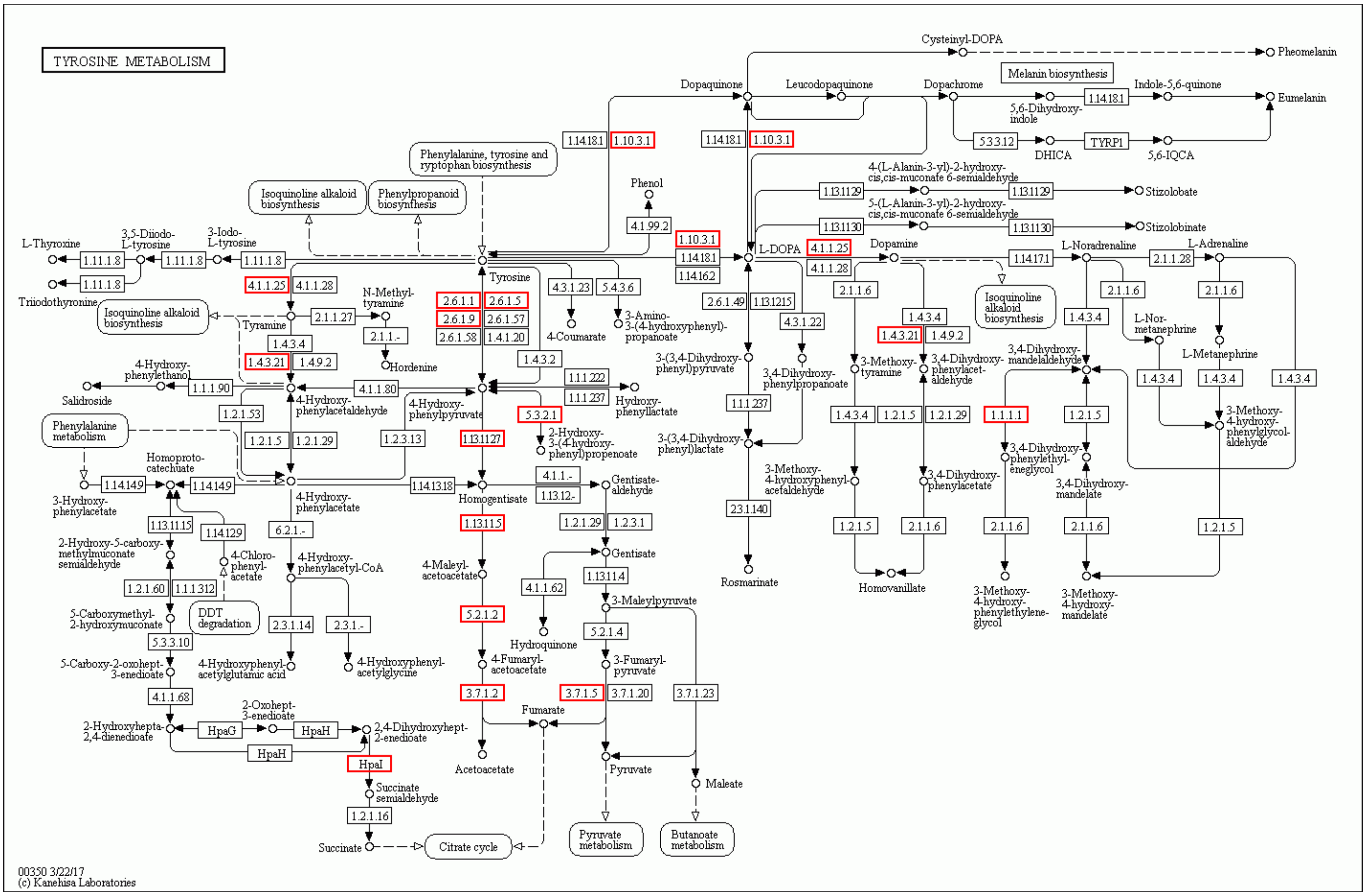

TYROSINE METABOLISM
Cysteinyl-DOPA
Pheomelanin
Melanin biosynthesis
Dopaquinone
Leucodopaquinone
Dopachrome
Indole-5,6-quinone
Eumelanin
5,6-Dihydroxy-indole
DHICA
5,6-IQCA
TYRP1
5.3.3.12
1.14.18.1
1.10.3.1
Phenylalanine, tyrosine and tryptophan biosynthesis
Isoquinoline alkaloid biosynthesis
Phenylpropanoid biosynthesis
Phenol
4.1.99.2
4-(L-Alanin-3-yl)-2-hydroxy-cis,cis-muconate 6-semialdehyde
5-(L-Alanin-3-yl)-2-hydroxy-cis,cis-muconate 6-semialdehyde
1.13.11.29
1.13.11.30
Stizolobate
Stizolobinate
L-Thyroxine
3,5-Diiodo-L-tyrosine
3-Iodo-L-tyrosine
1.11.1.8
Triiodothyronine
L-DOPA
Dopamine
4.1.1.25
4.1.1.28
1.14.17.1
L-Noradrenaline
L-Adrenaline
2.1.1.28
1.14.16.2
Tyrosine
N-Methyl-tyramine
Tyramine
2.1.1.27
Hordenine
2.6.1.1
2.6.1.5
2.6.1.9
2.6.1.57
2.6.1.58
1.4.1.20
4.3.1.23
5.4.3.6
4-Coumarate
3-Amino-3-(4-hydroxyphenyl)-propanoate
2.6.1.49
1.13.12.15
4.3.1.22
2.1.1.6
1.4.3.4
1.4.3.21
1.4.9.2
4-Hydroxy-phenylethanol
Salidroside
1.1.1.90
4.1.1.80
4-Hydroxy-phenylacetaldehyde
4-Hydroxy-phenylpyruvate
1.4.3.2
1.1.1.222
1.1.1.237
Hydroxy-phenyllactate
3-(3,4-Dihydroxy-phenyl)pyruvate
3,4-Dihydroxy-phenylpropanoate
3-Methoxy-tyramine
3,4-Dihydroxy-phenylacet-aldehyde
3,4-Dihydroxy-mandelaldehyde
L-Nor-metanephrine
L-Metanephrine
3-Methoxy-4-hydroxy-phenylglycol-aldehyde
1.2.1.53
1.2.1.5
1.2.1.29
1.2.3.13
5.3.2.1
1.13.11.27
2-Hydroxy-3-(4-hydroxy-phenyl)propenoate
3-(3,4-Dihydroxy-phenyl)lactate
1.1.1.1
3,4-Dihydroxy-phenylethyl-eneglycol
3,4-Dihydroxy-mandelate
3,4-Dihydroxy-phenylacetate
3-Methoxy-4-hydroxyphenyl-acetaldehyde
Phenylalanine metabolism
Homoproto-catechuate
3-Hydroxy-phenylacetate
1.14.14.9
4-Hydroxy-phenylacetate
1.14.13.18
Homogentisate
4.1.1.-
1.13.12.-
Gentisate-aldehyde
2.3.1.140
Rosmarinate
Homovanillate
3-Methoxy-4-hydroxy-phenylethylene-glycol
3-Methoxy-4-hydroxy-mandelate
1.13.11.5
1.2.1.29
1.2.3.1
Gentisate
1.13.11.15
1.14.12.9
6.2.1.-
2-Hydroxy-5-carboxy-methylmuconate semialdehyde
4-Chloro-phenyl-acetate
4-Hydroxy-phenylacetyl-CoA
4-Maleyl-acetoacetate
4.1.1.62
1.13.11.4
3-Maleylpyruvate
1.2.1.60
1.1.1.312
DDT degradation
5-Carboxymethyl-2-hydroxymuconate
5.3.3.10
2.3.1.14
2.3.1.-
5.2.1.2
Hydroquinone
5.2.1.4
5-Carboxy-2-oxohept-3-enedioate
4-Hydroxyphenyl-acetylglutamic acid
4-Hydroxyphenyl-acetylglycine
4-Fumaryl-acetoacetate
3-Fumaryl-pyruvate
4.1.1.68
2-Oxohept-3-enedioate
3.7.1.2
3.7.1.5
3.7.1.20
3.7.1.23
Fumarate
2-Hydroxyhepta-2,4-dienedioate
HpaG
HpaH
2,4-Dihydroxyhept-2-enedioate
HpaI
Acetoacetate
Pyruvate
Maleate
Succinate semialdehyde
1.2.1.16
Succinate
Citrate cycle
Pyruvate metabolism
Butanoate metabolism
00350 3/22/17
(c) Kanehisa Laboratories

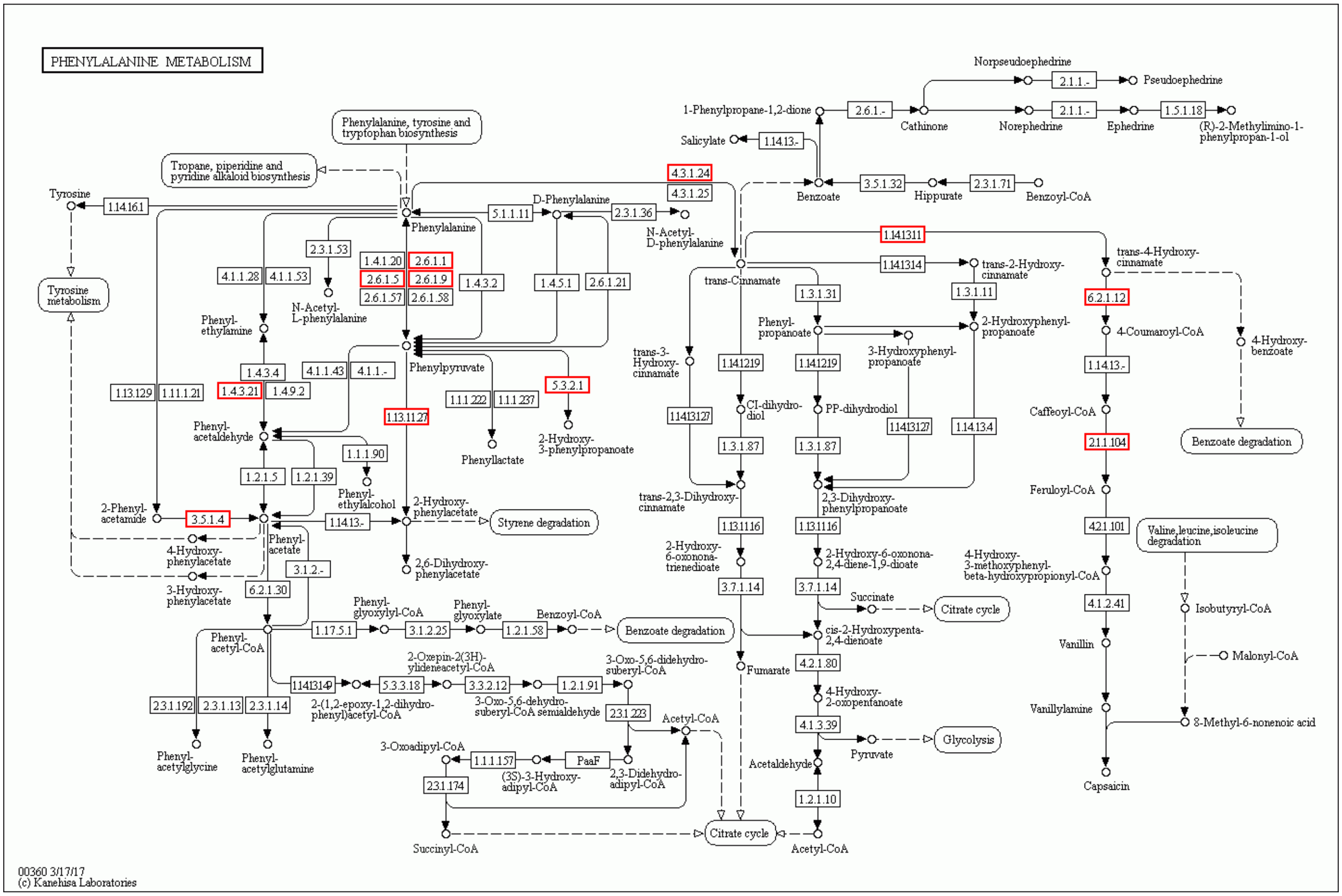

PHENYLALANINE METABOLISM
Norpseudoephedrine
Pseudoephedrine
2.1.1.-
1-Phenylpropane-1,2-dione
2.6.1.-
Cathinone
Norephedrine
Ephedrine
1.5.1.18
(R)-2-Methylimino-1-phenylpropan-1-ol
Salicylate
1.14.13.-
Phenylalanine, tyrosine and tryptophan biosynthesis
Tropane, piperidine and pyridine alkaloid biosynthesis
4.3.1.24
4.3.1.25
Benzoate
3.5.1.32
Hippurate
2.3.1.71
Benzoyl-CoA
Tyrosine
1.14.16.1
Phenylalanine
5.1.1.11
D-Phenylalanine
2.3.1.36
N-Acetyl-D-phenylalanine
1.14.13.11
Tyrosine metabolism
2.3.1.53
4.1.1.28
4.1.1.53
N-Acetyl-L-phenylalanine
1.4.1.20
2.6.1.1
2.6.1.5
2.6.1.9
2.6.1.57
2.6.1.58
1.4.3.2
1.4.5.1
2.6.1.21
trans-Cinnamate
1.14.13.14
trans-2-Hydroxy-cinnamate
trans-4-Hydroxy-cinnamate
6.2.1.12
4-Coumaroyl-CoA
4-Hydroxy-benzoate
Phenyl-ethylamine
1.3.1.31
1.3.1.11
Phenyl-propanoate
2-Hydroxyphenyl-propanoate
3-Hydroxyphenyl-propanoate
1.4.3.4
1.13.12.9
1.11.1.21
1.4.3.21
1.4.9.2
4.1.1.43
4.1.1.-
Phenylpyruvate
trans-3-Hydroxy-cinnamate
1.14.12.19
1.14.13.-
5.3.2.1
1.1.1.222
1.1.1.237
Phenyl-acetaldehyde
1.13.11.27
Cis-dihydro-diol
PP-dihydrodiol
Caffeoyl-CoA
1.14.13.127
1.14.13.4
2.1.1.104
Benzoate degradation
Phenyllactate
2-Hydroxy-3-phenylpropanoate
1.1.1.90
1.2.1.5
1.2.1.39
1.3.1.87
Feruloyl-CoA
2-Phenyl-acetamide
3.5.1.4
Phenyl-ethylalcohol
2-Hydroxy-phenylacetate
Styrene degradation
trans-2,3-Dihydroxy-cinnamate
2,3-Dihydroxy-phenylpropanoate
4.2.1.101
Valine, leucine, isoleucine degradation
4-Hydroxy-phenylacetate
Phenyl-acetate
2,6-Dihydroxy-phenylacetate
1.13.11.16
2-Hydroxy-6-oxonona-trienedioate
2-Hydroxy-6-oxonona-2,4-diene-1,9-dioate
4-Hydroxy-3-methoxyphenyl-beta-hydroxypropionoyl-CoA
3-Hydroxy-phenylacetate
3.1.2.-
6.2.1.30
3.7.1.14
Succinate
Citrate cycle
4.1.2.41
Isobutyryl-CoA
Phenyl-glyoxylyl-CoA
Phenyl-glyoxylate
Benzoyl-CoA
Phenyl-acetyl-CoA
1.17.5.1
3.1.2.25
1.2.1.58
Benzoate degradation
cis-2-Hydroxypenta-2,4-dienoate
Vanillin
Malonyl-CoA
2-Oxepin-2(3H)-ylideneacetyl-CoA
3-Oxo-5,6-didehydro-suberyl-CoA
Fumarate
4.2.1.80
1.14.13.149
5.3.3.18
3.3.2.12
1.2.1.91
2.3.1.192
2.3.1.13
2.3.1.14
2-(1,2-epoxy-1,2-dihydro-phenyl)acetyl-CoA
3-Oxo-5,6-dehydro-suberyl-CoA semialdehyde
2.3.1.223
Acetyl-CoA
4-Hydroxy-2-oxopentanoate
Vanillylamine
8-Methyl-6-nonenoic acid
4.1.3.39
Pyruvate
Glycolysis
3-Oxoadipyl-CoA
1.1.1.157
PaaF
Phenyl-acetylglycine
Phenyl-acetylglutamine
(3S)-3-Hydroxy-adipyl-CoA
2,3-Didehydro-adipyl-CoA
Acetaldehyde
2.3.1.174
1.2.1.10
Capsaicin
Citrate cycle
Succinyl-CoA
Acetyl-CoA
00360 3/17/17
(c) Kanehisa Laboratories

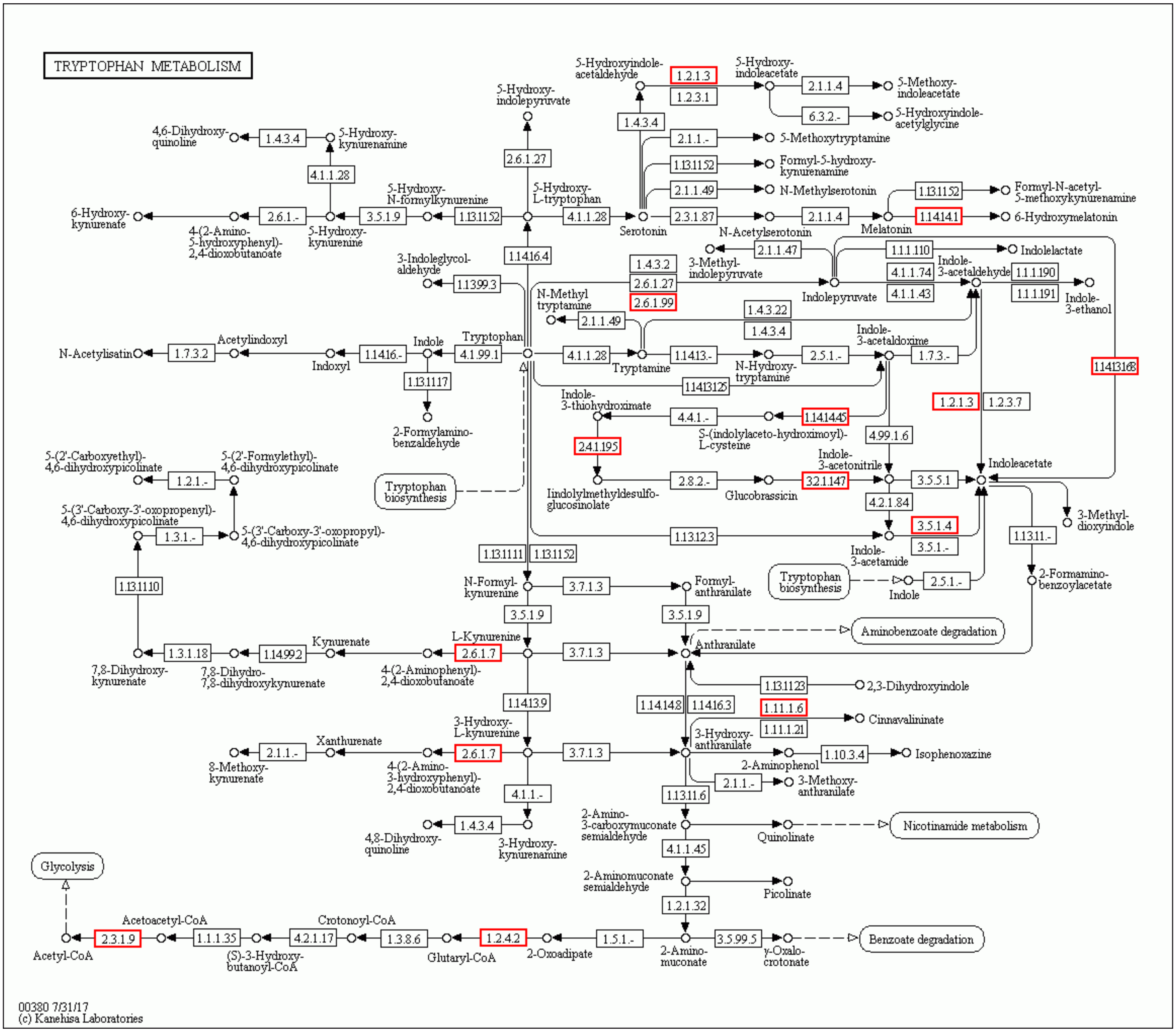
TRYPTOPHAN METABOLISM
5-Hydroxyindole-acetaldehyde
1.2.1.3
1.2.3.1
5-Hydroxy-indoleacetate
2.1.1.4
5-Methoxy-indoleacetate
6.3.2.-
5-Hydroxyindole-acetylglycine
5-Hydroxy-indolepyruvate
1.4.3.4
2.1.1.-
5-Methoxytryptamine
1.13.11.52
Formyl-5-hydroxy-kynurenamine
2.1.1.49
N-Methylserotonin
1.13.11.52
Formyl-N-acetyl-5-methoxykynurenamine
4,6-Dihydroxy-quinoline
1.4.3.4
5-Hydroxy-kynurenamine
2.6.1.27
4.1.1.28
5-Hydroxy-N-formylkynurenine
5-Hydroxy-L-tryptophan
6-Hydroxy-kynurenate
2.6.1.-
3.5.1.9
1.13.11.52
4.1.1.28
Serotonin
2.3.1.87
2.1.1.4
Melatonin
1.14.14.1
6-Hydroxymelatonin
4-(2-Amino-5-hydroxyphenyl)-2,4-dioxobutanoate
5-Hydroxy-kynurenine
N-Acetylserotonin
1.14.16.4
2.1.1.47
1.1.1.110
Indolelactate
3-Indoleglycol-aldehyde
1.4.3.2
3-Methyl-indolepyruvate
4.1.1.74
Indole-3-acetaldehyde
1.1.1.190
1.13.99.3
2.6.1.27
Indolepyruvate
4.1.1.43
1.1.1.191
Indole-3-ethanol
N-Methyl tryptamine
2.6.1.99
1.4.3.22
2.1.1.49
1.4.3.4
Acetylindoxyl
Indole
Tryptophan
Indole-3-acetaldoxime
N-Acetylisatin
1.7.3.2
1.14.16.-
4.1.99.1
4.1.1.28
1.14.13.-
2.5.1.-
1.7.3.-
1.14.13.168
Indoxyl
Tryptamine
N-Hydroxy-tryptamine
1.13.11.17
1.14.13.125
Indole-3-thiohydroximate
1.2.1.3
1.2.3.7
4.4.1.-
1.14.14.45
2-Formylamino-benzaldehyde
S-(indolylacetyl-hydroximoyl)-L-cysteine
4.99.1.6
2.4.1.195
Indole-3-acetonitrile
5-(2'-Carboxyethyl)-4,6-dihydroxypicolinate
5-(2'-Formylethyl)-4,6-dihydroxypicolinate
Indoleacetate
1.2.1.-
Tryptophan biosynthesis
2.8.2.-
3.2.1.147
3.5.5.1
Indolylmethyldesulfo-glucosinolate
Glucobrassicin
4.2.1.84
3-Methyl-dioxyindole
5-(3'-Carboxy-3'-oxopropenyl)-4,6-dihydroxypicolinate
3.5.1.4
1.3.1.-
5-(3'-Carboxy-3'-oxopropyl)-4,6-dihydroxypicolinate
1.13.12.3
1.13.11.-
Indole-3-acetamide
3.5.1.-
1.13.11.11
1.13.11.52
Tryptophan biosynthesis
2.5.1.-
1.13.11.10
N-Formyl-kynurenine
3.7.1.3
Formyl-anthranilate
Indole
2-Formamino-benzoylacetate
3.5.1.9
3.5.1.9
Aminobenzoate degradation
Kynurenate
L-Kynurenine
Anthranilate
1.3.1.18
1.14.99.2
2.6.1.7
3.7.1.3
7,8-Dihydroxy-kynurenate
7,8-Dihydro-7,8-dihydroxykynurenate
4-(2-Aminophenyl)-2,4-dioxobutanoate
1.13.11.23
2,3-Dihydroxyindole
1.14.13.9
1.14.14.8
1.14.16.3
1.11.1.6
Cinnavalinate
3-Hydroxy-L-kynurenine
1.11.1.21
Xanthurenate
3-Hydroxy-anthranilate
2.1.1.-
2.6.1.7
3.7.1.3
2-Aminophenol
1.10.3.4
Isophenoxazine
8-Methoxy-kynurenate
4-(2-Amino-3-hydroxyphenyl)-2,4-dioxobutanoate
2.1.1.-
3-Methoxy-anthranilate
4.1.1.-
1.13.11.6
2-Amino-3-carboxymuconate semialdehyde
Nicotinamide metabolism
1.4.3.4
Quinolinate
4,8-Dihydroxy-quinoline
3-Hydroxy-kynurenamine
4.1.1.45
Glycolysis
2-Aminomuconate semialdehyde
Picolinate
1.2.1.32
Acetoacetyl-CoA
Crotonoyl-CoA
2.3.1.9
1.1.1.35
4.2.1.17
1.3.8.6
1.2.4.2
1.5.1.-
3.5.99.5
Benzoate degradation
Acetyl-CoA
(S)-3-Hydroxy-butanoyl-CoA
Glutaryl-CoA
2-Oxoadipate
2-Amino-muconate
γ-Oxalo-crotonate
00380 7/31/17
(c) Kanehisa Laboratories

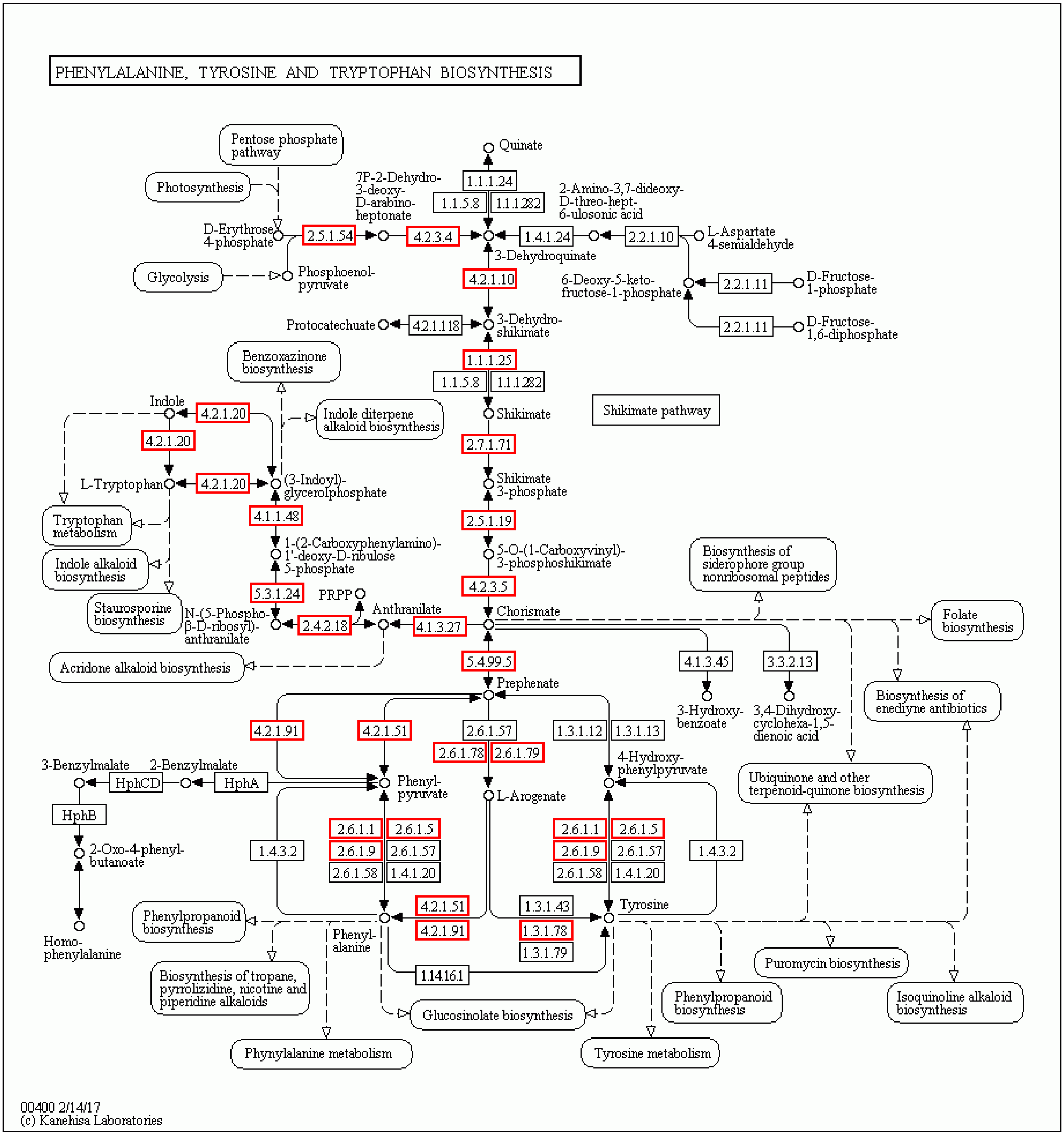
PHENYLALANINE, TYROSINE AND TRYPTOPHAN BIOSYNTHESIS
Pentose phosphate pathway
Photosynthesis
Quinate
1.1.1.24
1.1.5.8
1.1.1.282
7P-2-Dehydro-3-deoxy-D-arabino-heptonate
2-Amino-3,7-dideoxy-D-threo-hept-6-ulosonic acid
D-Erythrose 4-phosphate
2.5.1.54
4.2.3.4
1.4.1.24
2.2.1.10
L-Aspartate 4-semialdehyde
3-Dehydroquinate
Glycolysis
Phosphoenol-pyruvate
4.2.1.10
6-Deoxy-5-keto-fructose-1-phosphate
2.2.1.11
D-Fructose-1-phosphate
Protocatechuate
4.2.1.118
3-Dehydro-shikimate
2.2.1.11
D-Fructose-1,6-diphosphate
Benzoxazinone biosynthesis
1.1.1.25
1.1.5.8
1.1.1.282
Indole
4.2.1.20
Indole diterpene alkaloid biosynthesis
Shikimate
Shikimate pathway
4.2.1.20
2.7.1.71
L-Tryptophan
4.2.1.20
(3-Indoyl)-glycerolphosphate
Shikimate 3-phosphate
Tryptophan metabolism
4.1.1.48
2.5.1.19
1-(2-Carboxyphenylamino)-1'-deoxy-D-ribulose 5-phosphate
5-O-(1-Carboxyvinyl)-3-phosphoshikimate
Indole alkaloid biosynthesis
Biosynthesis of siderophore group nonribosomal peptides
5.3.1.24
PRPP
4.2.3.5
Staurosporine biosynthesis
N-(5-Phospho-β-D-ribosyl)-anthranilate
2.4.2.18
Anthranilate
4.1.3.27
Chorismate
Folate biosynthesis
Acridone alkaloid biosynthesis
5.4.99.5
4.1.3.45
3.3.2.13
Prephenate
Biosynthesis of enediyne antibiotics
3-Hydroxy-benzoate
3,4-Dihydroxy-cyclohexa-1,5-dienoic acid
4.2.1.91
4.2.1.51
2.6.1.57
1.3.1.12
1.3.1.13
2.6.1.78
2.6.1.79
3-Benzylmalate
2-Benzylmalate
4-Hydroxy-phenylpyruvate
HphCD
HphA
Phenyl-pyruvate
L-Arogenate
Ubiquinone and other terpenoid-quinone biosynthesis
HphB
2.6.1.1
2.6.1.5
2.6.1.1
2.6.1.5
2-Oxo-4-phenyl-butanoate
1.4.3.2
2.6.1.9
2.6.1.57
2.6.1.9
2.6.1.57
1.4.3.2
2.6.1.58
1.4.1.20
2.6.1.58
1.4.1.20
Phenylpropanoid biosynthesis
4.2.1.51
1.3.1.43
Tyrosine
Homo-phenylalanine
Phenyl-alanine
4.2.1.91
1.3.1.78
1.3.1.79
Puromycin biosynthesis
Biosynthesis of tropane, pyrrolizidine, nicotine and piperidine alkaloids
1.14.16.1
Phenylpropanoid biosynthesis
Isoquinoline alkaloid biosynthesis
Glucosinolate biosynthesis
Phynylalanine metabolism
Tyrosine metabolism
00400 2/14/17
(c) Kanehisa Laboratories

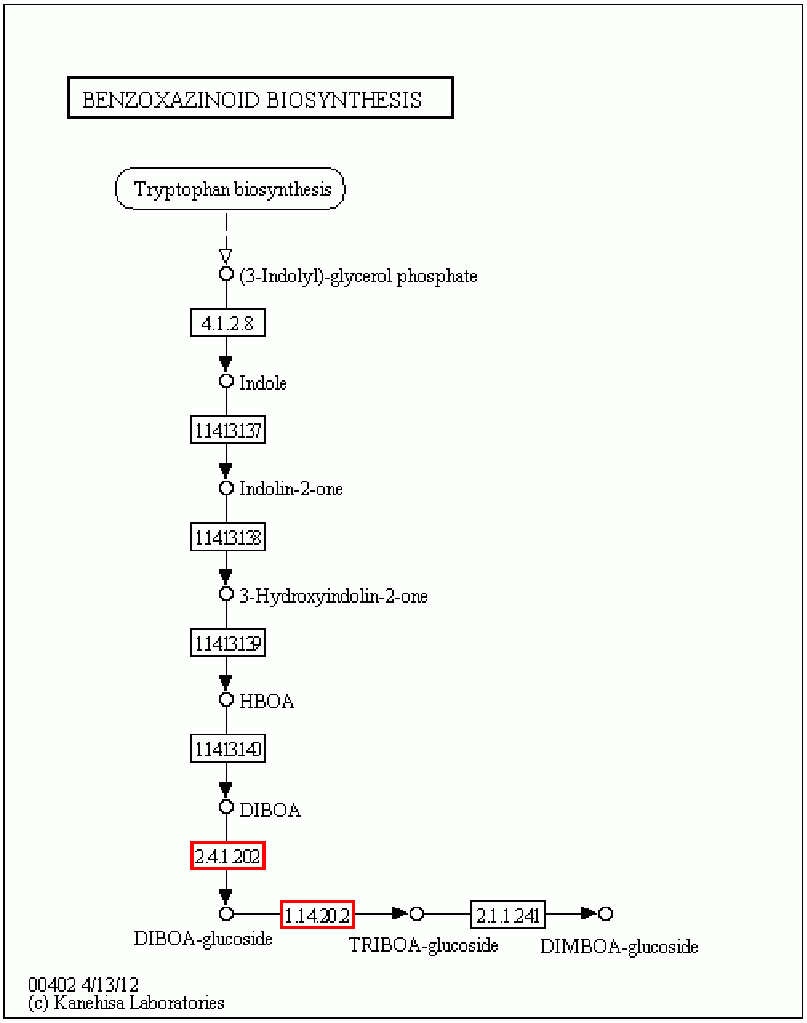
BENZOXAZINOID BIOSYNTHESIS
Tryptophan biosynthesis
(3-Indolyl)-glycerol phosphate
4.1.2.8
Indole
11413137
Indolin-2-one
11413138
3-Hydroxyindolin-2-one
11413139
HBOA
11413140
DIBOA
2.4.1.202
DIBOA-glucoside
1.14.20.2
TRIBOA-glucoside
2.1.1.241
DIMBOA-glucoside
00402 4/13/12
(c) Kanehisa Laboratories

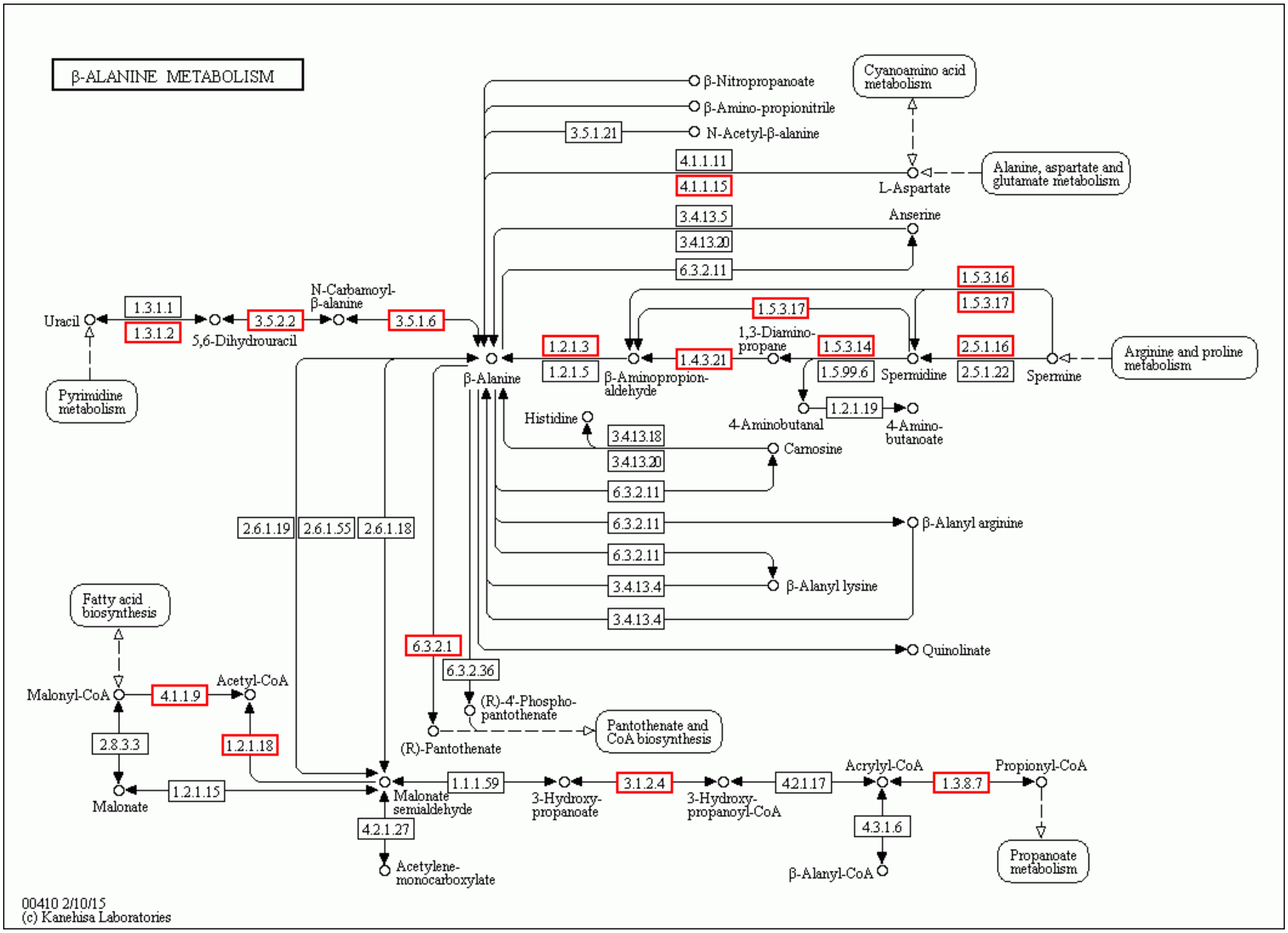
β-ALANINE METABOLISM
β-Nitropropanoate
β-Amino-propionitrile
3.5.1.21
N-Acetyl-β-alanine
Cyanoamino acid metabolism
4.1.1.11
4.1.1.15
L-Aspartate
Alanine, aspartate and glutamate metabolism
3.4.13.5
3.4.13.20
6.3.2.11
Anserine
1.5.3.16
1.5.3.17
1.3.1.1
1.3.1.2
Uracil
5,6-Dihydrouracil
3.5.2.2
N-Carbamoyl-β-alanine
3.5.1.6
1.2.1.3
1.2.1.5
β-Alanine
β-Aminopropion-aldehyde
1.4.3.21
1,3-Diamino-propane
1.5.3.14
1.5.99.6
Spermidine
2.5.1.16
2.5.1.22
Spermine
Arginine and proline metabolism
Pyrimidine metabolism
Histidine
3.4.13.18
3.4.13.20
4-Aminobutanal
1.2.1.19
4-Amino-butanoate
Carnosine
6.3.2.11
6.3.2.11
β-Alanyl arginine
6.3.2.11
3.4.13.4
β-Alanyl lysine
3.4.13.4
2.6.1.19
2.6.1.55
2.6.1.18
Quinolinate
Fatty acid biosynthesis
6.3.2.1
6.3.2.36
Malonyl-CoA
4.1.1.9
Acetyl-CoA
(R)-4'-Phospho-pantothenate
Pantothenate and CoA biosynthesis
2.8.3.3
1.2.1.18
(R)-Pantothenate
Acrylyl-CoA
Propionyl-CoA
1.1.1.59
3.1.2.4
4.2.1.17
1.3.8.7
Malonate
1.2.1.15
Malonate semialdehyde
3-Hydroxy-propanoate
3-Hydroxy-propanoyl-CoA
4.3.1.6
4.2.1.27
β-Alanyl-CoA
Propanoate metabolism
Acetylene-monocarboxylate
00410 2/10/15
(c) Kanehisa Laboratories

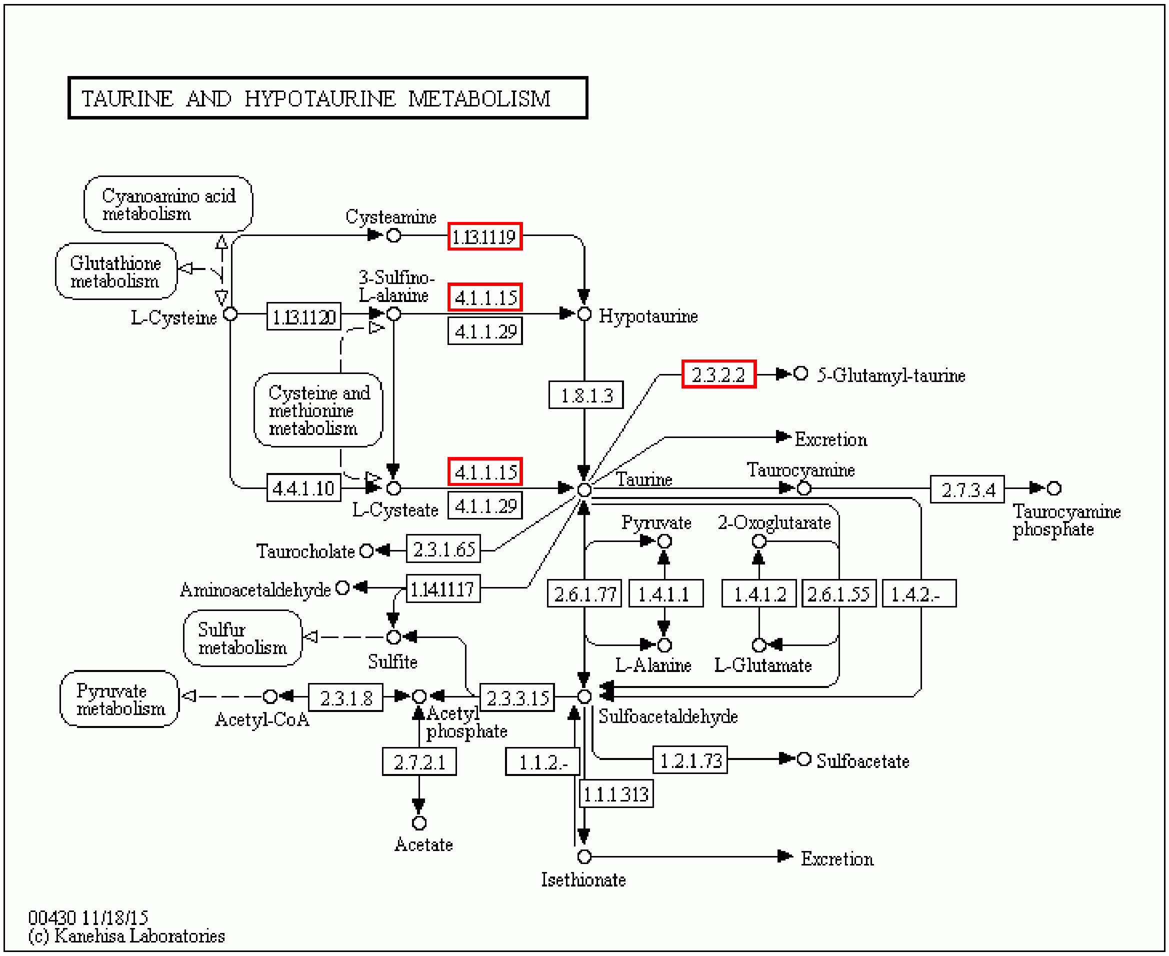

TAURINE AND HYPOTAURINE METABOLISM
Cyanoamino acid metabolism
Glutathione metabolism
Cysteamine
1.13.11.19
3-Sulfino-L-alanine
4.1.1.15
4.1.1.29
Hypotaurine
L-Cysteine
1.13.11.20
Cysteine and methionine metabolism
1.8.1.3
2.3.2.2
5-Glutamyl-taurine
Excretion
4.4.1.10
4.1.1.15
4.1.1.29
L-Cysteate
Taurine
Taurocyamine
2.7.3.4
Taurocyamine phosphate
Taurocholate
2.3.1.65
Pyruvate
2-Oxoglutarate
Aminoacetaldehyde
1.14.11.17
2.6.1.77
1.4.1.1
1.4.1.2
2.6.1.55
1.4.2.-
Sulfur metabolism
Sulfite
L-Alanine
L-Glutamate
Pyruvate metabolism
Acetyl-CoA
2.3.1.8
Acetyl phosphate
2.3.3.15
Sulfoacetaldehyde
2.7.2.1
1.1.2.-
1.2.1.73
Sulfoacetate
1.1.1.313
Acetate
Isethionate
Excretion
00430 11/18/15
(c) Kanehisa Laboratories

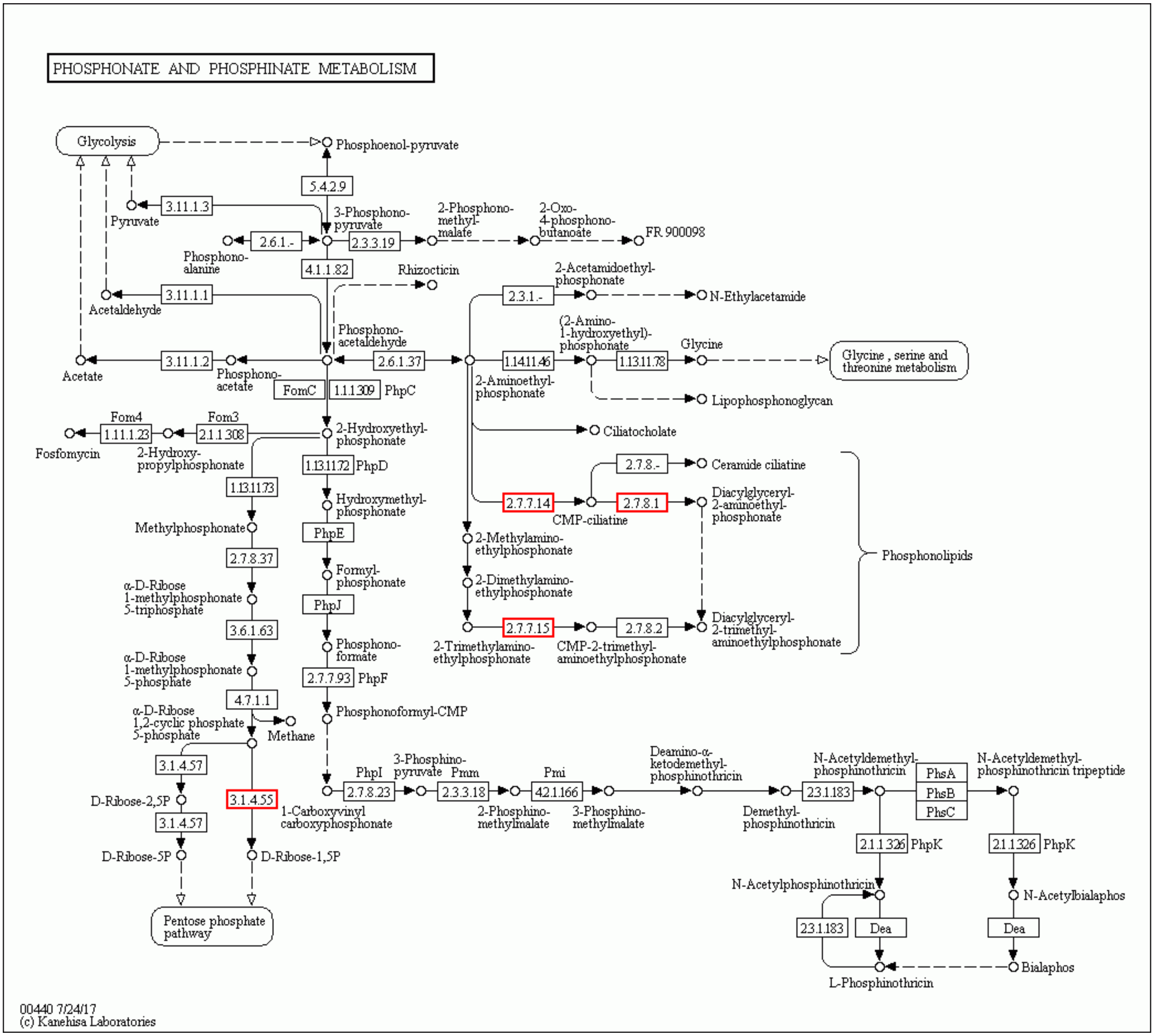
PHOSPHONATE AND PHOSPHINATE METABOLISM
Glycolysis
Phosphoenol-pyruvate
5.4.2.9
3.11.1.3
Pyruvate
3-Phosphono-pyruvate
2.6.1.-
2.3.3.19
2-Phosphono-methyl-malate
2-Oxo-4-phosphono-butanoate
FR 900098
Phosphono-alanine
4.1.1.82
Rhizocticin
3.11.1.1
Acetaldehyde
2.3.1.-
2-Acetamidoethyl-phosphonate
N-Ethylacetamide
Phosphono-acetaldehyde
(2-Amino-1-hydroxyethyl)-phosphonate
Glycine
Glycine , serine and threonine metabolism
3.11.1.2
2.6.1.37
1.14.11.46
1.13.11.78
Acetate
Phosphono-acetate
FomC
1.1.1.309
PhpC
2-Aminoethyl-phosphonate
Lipophosphonoglycan
Fom4
Fom3
1.11.1.23
2.1.1.308
2-Hydroxyethyl-phosphonate
Ciliatocholate
Fosfomycin
2-Hydroxy-propylphosphonate
1.13.11.72
PhpD
2.7.8.-
Ceramide ciliatine
1.13.11.73
Hydroxymethyl-phosphonate
2.7.7.14
2.7.8.1
Diacylglyceryl-2-aminoethyl-phosphonate
CMP-ciliatine
Methylphosphonate
PhpE
2-Methylamino-ethylphosphonate
Phosphonolipids
2.7.8.37
Formyl-phosphonate
α-D-Ribose 1-methylphosphonate 5-triphosphate
2-Dimethylamino-ethylphosphonate
PhpJ
Diacylglyceryl-2-trimethyl-aminoethylphosphonate
3.6.1.63
2.7.7.15
2.7.8.2
Phosphono-formate
2-Trimethylamino-ethylphosphonate
CMP-2-trimethyl-aminoethylphosphonate
α-D-Ribose 1-methylphosphonate 5-phosphate
2.7.7.93
PhpF
4.7.1.1
Phosphonoformyl-CMP
α-D-Ribose 1,2-cyclic phosphate 5-phosphate
Methane
Deamino-α-ketodemethyl-phosphinothricin
N-Acetyldemethyl-phosphinothricin
N-Acetyldemethyl-phosphinothricin tripeptide
3-Phosphino-pyruvate
PhpI
Pmm
Pmi
3.1.4.57
PhsA
PhsB
PhsC
2.7.8.23
2.3.3.18
4.2.1.166
2.3.1.183
D-Ribose-2,5P
3.1.4.55
1-Carboxyvinyl carboxyphosphonate
2-Phosphino-methylmalate
3-Phosphino-methylmalate
Demethyl-phosphinothricin
3.1.4.57
2.1.1.326
PhpK
2.1.1.326
PhpK
D-Ribose-5P
D-Ribose-1,5P
N-Acetylphosphinothricin
N-Acetylbialaphos
Pentose phosphate pathway
2.3.1.183
Dea
Dea
Bialaphos
L-Phosphinothricin
00440 7/24/17
(c) Kanehisa Laboratories

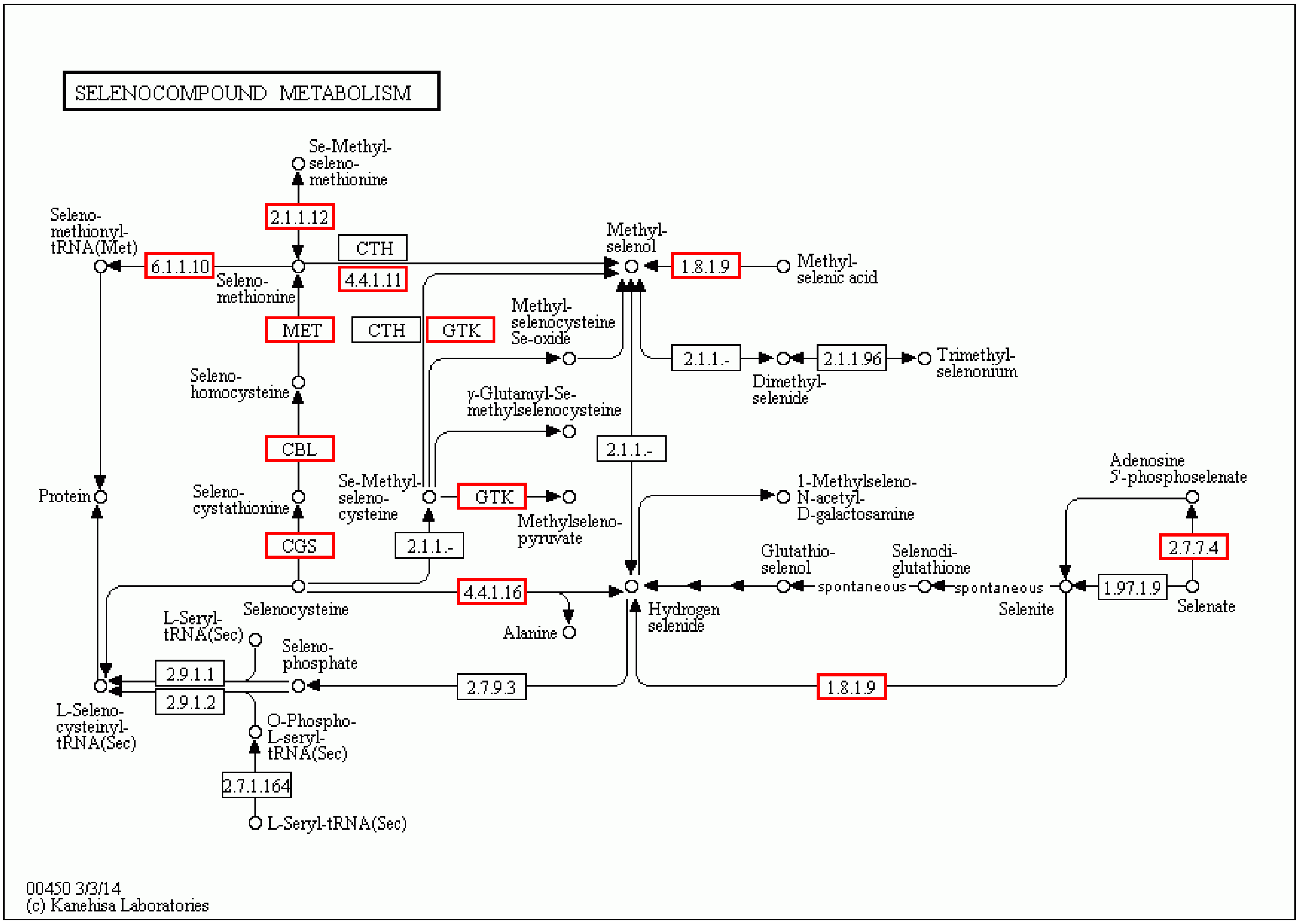
SELENOCOMPOUND METABOLISM
Se-Methyl-selenomethionine
2.1.1.12
Seleno-methionyl-tRNA(Met)
6.1.1.10
CTH
4.4.1.11
Seleno-methionine
Methyl-selenol
1.8.1.9
Methyl-selenic acid
MET
CTH
GTK
Methyl-selenocysteine Se-oxide
2.1.1.-
Dimethyl-selenide
2.1.1.96
Trimethyl-selenonium
Seleno-homocysteine
γ-Glutamyl-Se-methylselenocysteine
CBL
2.1.1.-
Adenosine 5'-phosphoselenate
Protein
Seleno-cystathionine
Se-Methyl-seleno-cysteine
GTK
Methylseleno-pyruvate
1-Methylseleno-N-acetyl-D-galactosamine
2.7.7.4
CGS
2.1.1.-
Glutathio-selenol
Selenodi-glutathione
4.4.1.16
spontaneous
spontaneous
1.97.1.9
Selenocysteine
Hydrogen selenide
Selenite
Selenate
L-Seryl-tRNA(Sec)
Alanine
Seleno-phosphate
2.9.1.1
2.7.9.3
1.8.1.9
2.9.1.2
L-Seleno-cysteinyl-tRNA(Sec)
O-Phospho-L-seryl-tRNA(Sec)
2.7.1.164
L-Seryl-tRNA(Sec)
00450 3/3/14
(c) Kanehisa Laboratories

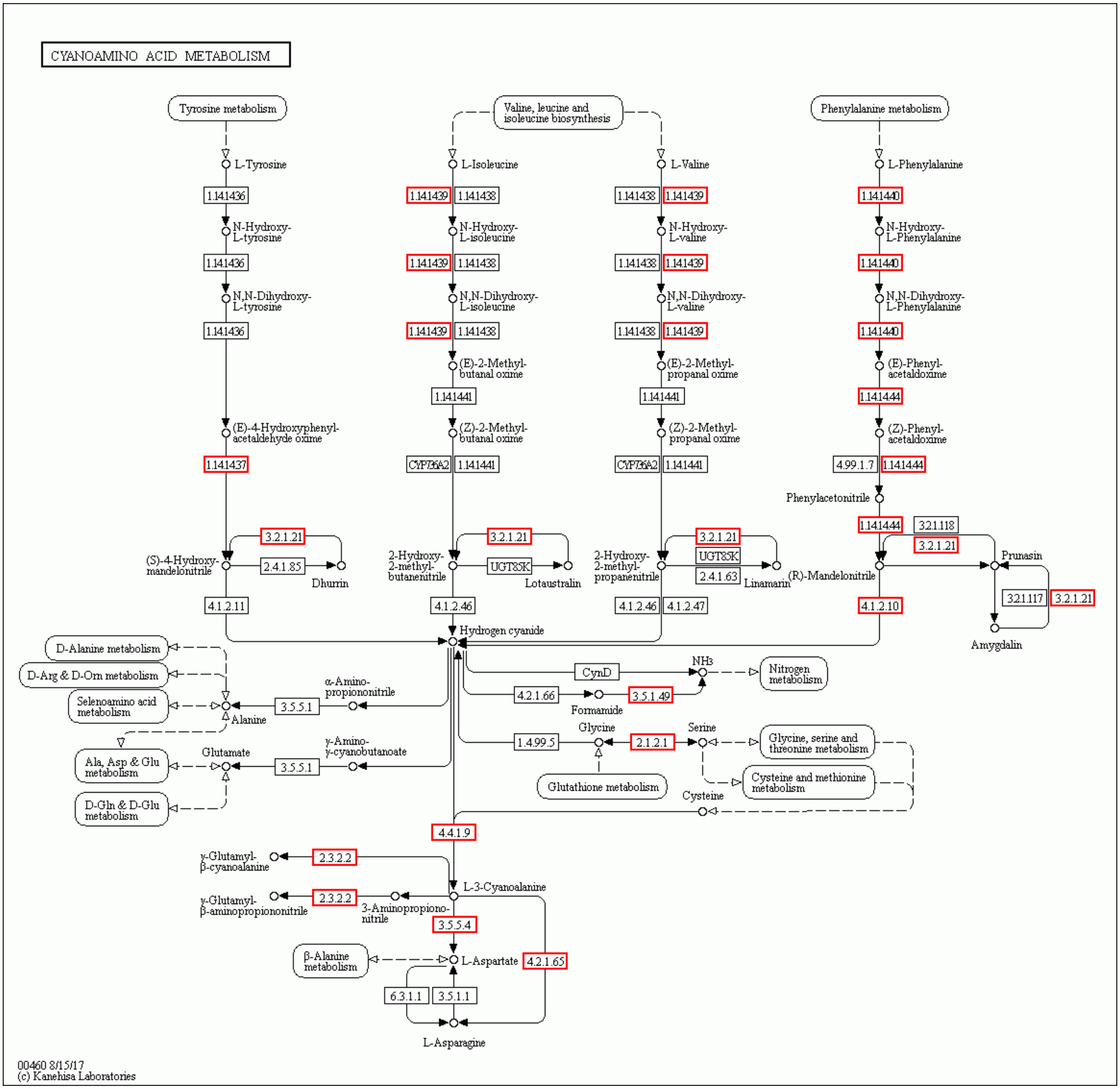
CYANOAMINO ACID METABOLISM
Tyrosine metabolism
Valine, leucine and isoleucine biosynthesis
Phenylalanine metabolism
L-Tyrosine
L-Isoleucine
L-Valine
L-Phenylalanine
1.14.14.36
1.14.14.39
1.14.14.38
1.14.14.40
N-Hydroxy-L-tyrosine
N-Hydroxy-L-isoleucine
N-Hydroxy-L-valine
N-Hydroxy-L-Phenylalanine
N,N-Dihydroxy-L-tyrosine
N,N-Dihydroxy-L-isoleucine
N,N-Dihydroxy-L-valine
N,N-Dihydroxy-L-Phenylalanine
(E)-2-Methyl-butanal oxime
(E)-2-Methyl-propanal oxime
(E)-Phenyl-acetaldoxime
1.14.14.41
1.14.14.44
(E)-4-Hydroxyphenyl-acetaldehyde oxime
(Z)-2-Methyl-butanal oxime
(Z)-2-Methyl-propanal oxime
(Z)-Phenyl-acetaldoxime
1.14.14.37
CYP736A2
4.99.1.7
Phenylacetonitrile
3.2.1.21
3.2.1.118
(S)-4-Hydroxy-mandelonitrile
2.4.1.85
Dhurrin
2-Hydroxy-2-methyl-butanenitrile
UGT85K
Lotaustralin
2-Hydroxy-2-methyl-propanenitrile
2.4.1.63
Linamarin
(R)-Mandelonitrile
Prunasin
3.2.1.117
Amygdalin
4.1.2.11
4.1.2.46
4.1.2.47
4.1.2.10
Hydrogen cyanide
D-Alanine metabolism
D-Arg & D-Orn metabolism
Selenoamino acid metabolism
Alanine
3.5.5.1
α-Amino-propiononitrile
CynD
NH3
Nitrogen metabolism
4.2.1.66
Formamide
3.5.1.49
Glycine
Serine
1.4.99.5
2.1.2.1
Glycine, serine and threonine metabolism
Glutathione metabolism
Cysteine and methionine metabolism
Cysteine
Ala, Asp & Glu metabolism
Glutamate
γ-Amino-γ-cyanobutanoate
D-Gln & D-Glu metabolism
4.4.1.9
γ-Glutamyl-β-cyanoalanine
2.3.2.2
L-3-Cyanoalanine
γ-Glutamyl-β-aminopropiononitrile
3-Aminopropiono-nitrile
3.5.5.4
β-Alanine metabolism
L-Aspartate
4.2.1.65
6.3.1.1
3.5.1.1
L-Asparagine
00460 8/15/17
(c) Kanehisa Laboratories

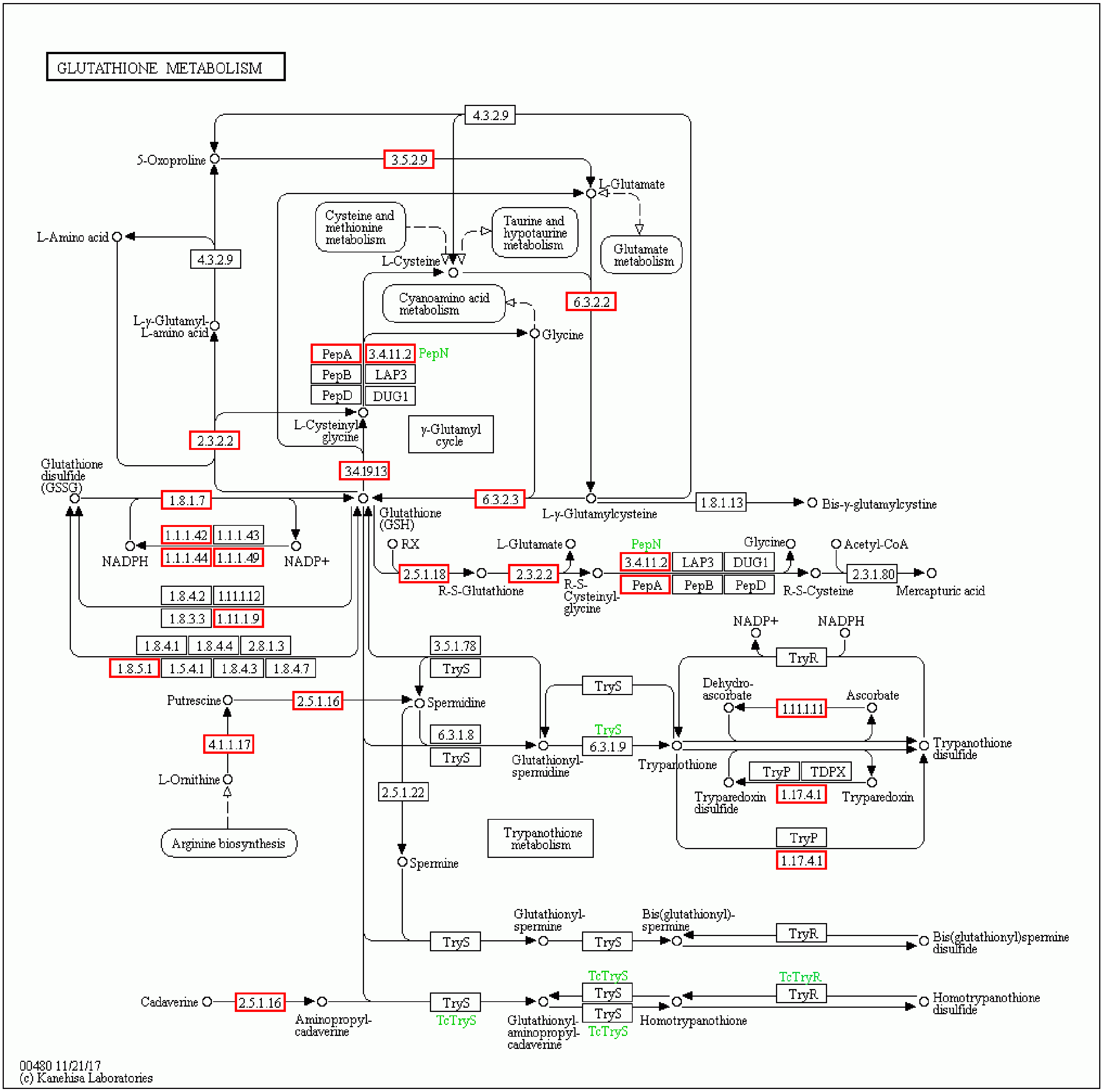
GLUTATHIONE METABOLISM
4.3.2.9
5-Oxoproline
3.5.2.9
L-Glutamate
L-Amino acid
4.3.2.9
Cysteine and methionine metabolism
Taurine and hypotaurine metabolism
Glutamate metabolism
L-Cysteine
Cyanoamino acid metabolism
6.3.2.2
L-γ-Glutamyl-L-amino acid
Glycine
PepA
3.4.11.2
PepN
PepB
LAP3
PepD
DUG1
L-Cysteinyl glycine
γ-Glutamyl cycle
2.3.2.2
3.4.19.13
Glutathione disulfide (GSSG)
1.8.1.7
6.3.2.3
L-γ-Glutamylcysteine
1.8.1.13
Bis-γ-glutamylcystine
Glutathione (GSH)
1.1.1.42
1.1.1.43
1.1.1.44
1.1.1.49
NADPH
NADP+
RX
L-Glutamate
PepN
Glycine
Acetyl-CoA
2.5.1.18
2.3.2.2
3.4.11.2
LAP3
DUG1
2.3.1.80
R-S-Glutathione
R-S-Cysteinyl-glycine
PepA
PepB
PepD
R-S-Cysteine
Mercapturic acid
1.8.4.2
1.11.1.12
1.8.3.3
1.11.1.9
NADP+
NADPH
1.8.4.1
1.8.4.4
2.8.1.3
3.5.1.78
TryR
1.8.5.1
1.5.4.1
1.8.4.3
1.8.4.7
TryS
TryS
Dehydro-ascorbate
Ascorbate
Putrescine
2.5.1.16
Spermidine
1.11.1.11
4.1.1.17
6.3.1.8
TryS
Glutathionyl-spermidine
6.3.1.9
Trypanothione
Trypanothione disulfide
TryS
TryP
TDPX
L-Ornithine
2.5.1.22
Trypanedoxin disulfide
1.17.4.1
Trypanedoxin
Arginine biosynthesis
Trypanothione metabolism
TryP
1.17.4.1
Spermine
Glutathionyl-spermine
Bis(glutathionyl)-spermine
TryR
Bis(glutathionyl)spermine disulfide
TryS
TryS
TcTryS
TcTryR
Cadaverine
2.5.1.16
TryS
TryR
Homotrypanothione disulfide
Aminopropyl-cadaverine
TcTryS
Glutathionyl-aminopropyl-cadaverine
TryS
TcTryS
Homotrypanothione
00480 11/21/17
(c) Kanehisa Laboratories

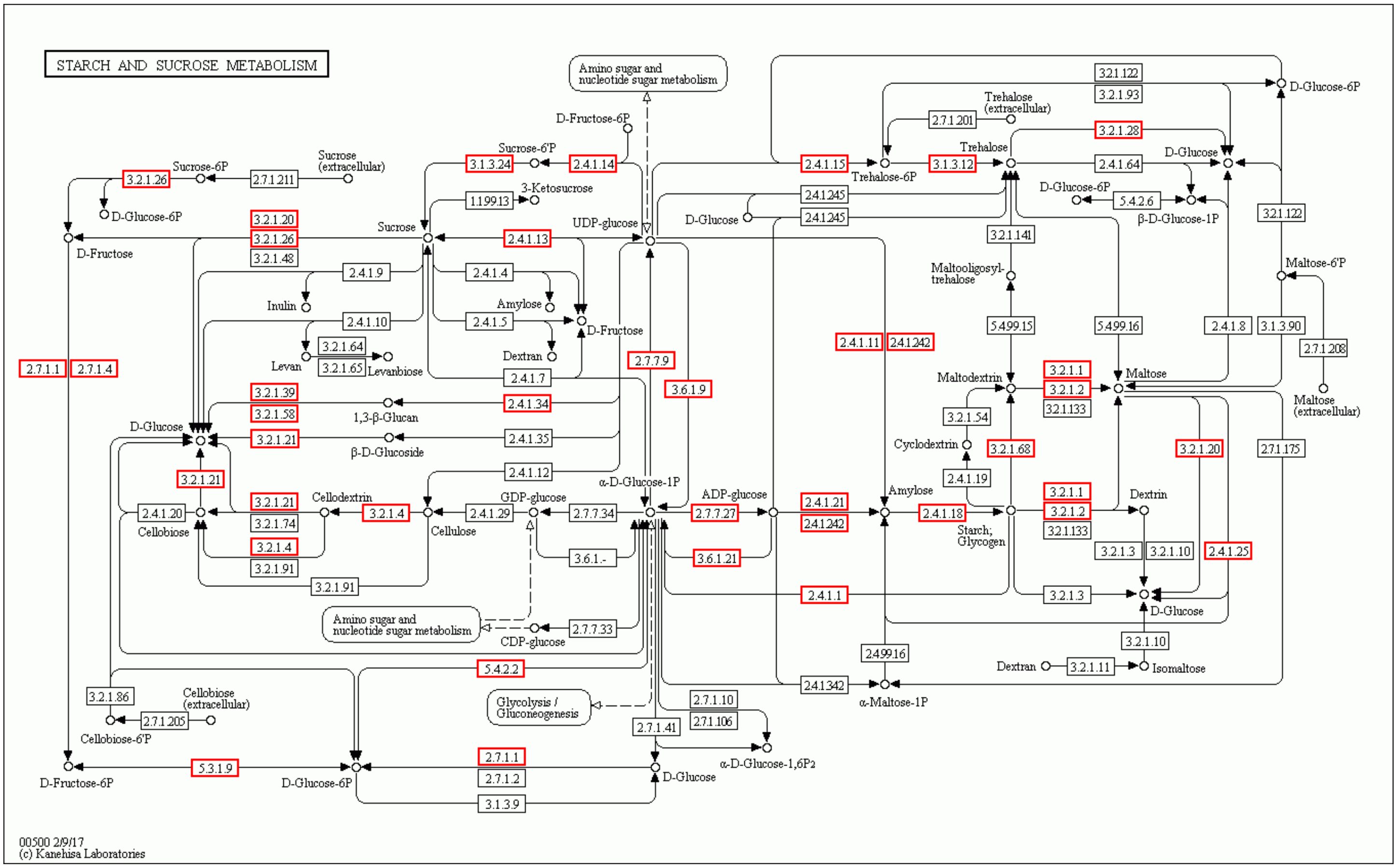
STARCH AND SUCROSE METABOLISM
Amino sugar and nucleotide sugar metabolism
D-Fructose-6P
Sucrose-6'P
3.1.3.24
2.4.1.14
Sucrose-6P
Sucrose (extracellular)
3.2.1.26
2.7.1.211
D-Glucose-6P
3-Ketosucrose
1.1.99.13
D-Fructose
3.2.1.20
3.2.1.26
3.2.1.48
Sucrose
2.4.1.13
UDP-glucose
2.4.1.9
2.4.1.4
Inulin
Amylose
2.4.1.10
2.4.1.5
D-Fructose
3.2.1.64
Levan
3.2.1.65
Levanbiose
Dextran
2.4.1.7
2.7.1.1
2.7.1.4
2.7.7.9
3.6.1.9
3.2.1.39
2.4.1.34
1,3-β-Glucan
3.2.1.58
D-Glucose
3.2.1.21
2.4.1.35
β-D-Glucoside
2.4.1.12
α-D-Glucose-1P
3.2.1.21
Cellodextrin
GDP-glucose
2.4.1.20
3.2.1.21
3.2.1.4
2.4.1.29
2.7.7.34
Cellobiose
3.2.1.74
Cellulose
3.2.1.4
3.6.1.-
3.2.1.91
3.2.1.91
Amino sugar and nucleotide sugar metabolism
2.7.7.33
CDP-glucose
5.4.2.2
3.2.1.86
Cellobiose (extracellular)
2.7.1.205
Cellobiose-6'P
Glycolysis / Gluconeogenesis
2.7.1.10
2.7.1.106
2.7.1.41
α-D-Glucose-1,6P2
2.7.1.1
D-Fructose-6P
5.3.1.9
D-Glucose-6P
2.7.1.2
D-Glucose
3.1.3.9
3.2.1.122
3.2.1.93
D-Glucose-6P
Trehalose (extracellular)
2.7.1.201
3.2.1.28
Trehalose
D-Glucose
2.4.1.15
Trehalose-6P
3.1.3.12
2.4.1.64
2.4.1.245
D-Glucose-6P
5.4.2.6
β-D-Glucose-1P
D-Glucose
2.4.1.245
3.2.1.122
3.2.1.141
Maltooligosyl-trehalose
Maltose-6'P
5.4.99.15
5.4.99.16
2.4.1.8
3.1.3.90
2.7.1.208
2.4.1.11
2.4.1.242
Maltodextrin
3.2.1.1
3.2.1.2
Maltose
Maltose (extracellular)
3.2.1.133
3.2.1.54
Cyclodextrin
3.2.1.68
3.2.1.20
2.7.1.175
2.4.1.19
ADP-glucose
Amylose
3.2.1.1
2.4.1.21
Dextrin
2.7.7.27
2.4.1.242
2.4.1.18
3.2.1.2
Starch; Glycogen
3.2.1.133
3.2.1.3
3.2.1.10
2.4.1.25
3.6.1.21
2.4.1.1
3.2.1.3
D-Glucose
3.2.1.10
2.4.99.16
Dextran
3.2.1.11
Isomaltose
2.4.1.342
α-Maltose-1P
00500 2/9/17
(c) Kanehisa Laboratories

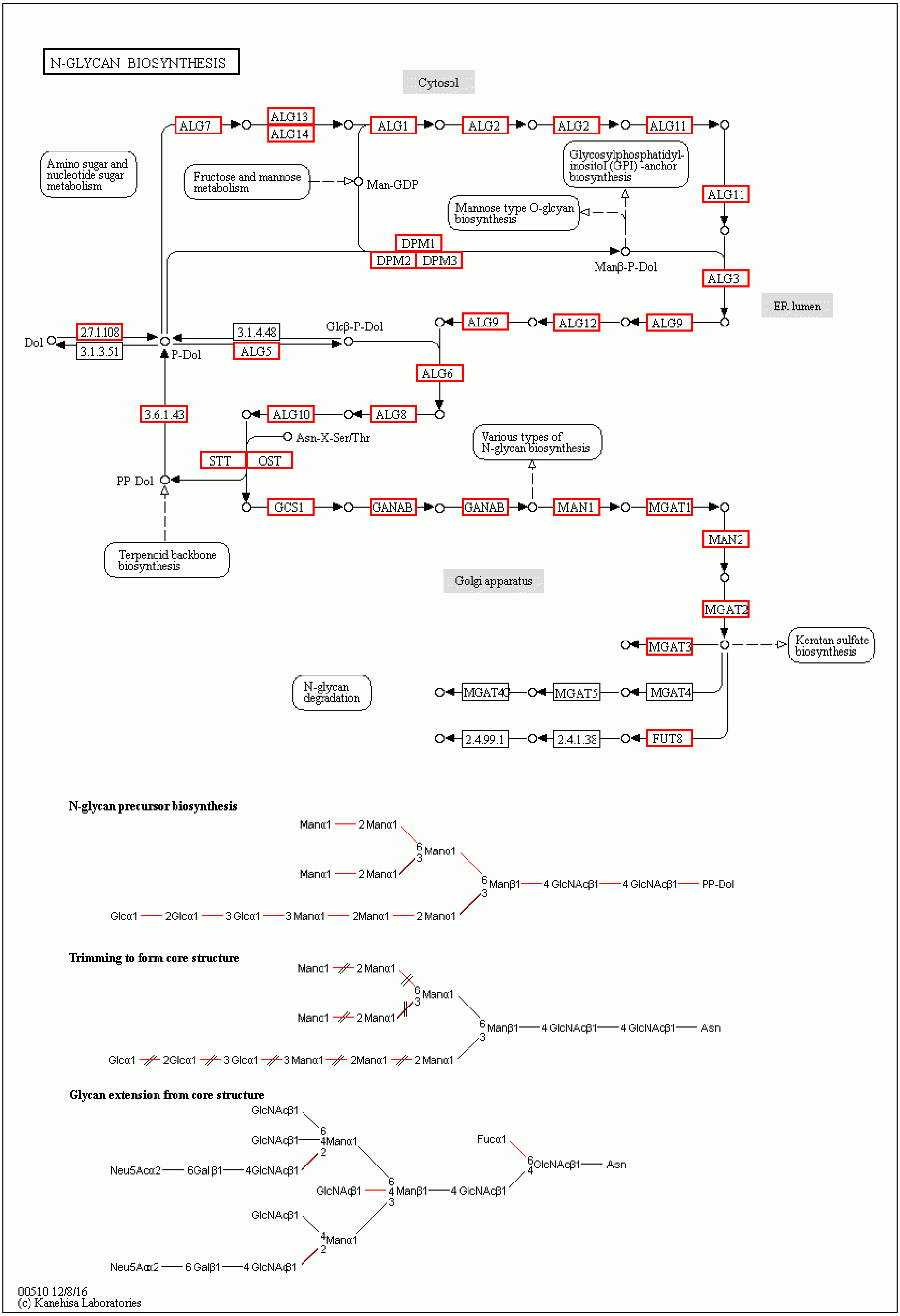
N-GLYCAN BIOSYNTHESIS
Cytosol
ER lumen
Golgi apparatus
Amino sugar and nucleotide sugar metabolism
Fructose and mannose metabolism
Glycosylphosphatidyl-inositol (GPI) -anchor biosynthesis
Mannose type O-glcyan biosynthesis
Various types of N-glycan biosynthesis
Terpenoid backbone biosynthesis
Keratan sulfate biosynthesis
N-glycan degradation
ALG7
ALG13
ALG14
ALG1
ALG2
ALG2
ALG11
ALG11
ALG3
Man-GDP
DPM1
DPM2
DPM3
Manβ-P-Dol
Dol
2.7.1.108
3.1.3.51
P-Dol
3.1.4.48
ALG5
Glcβ-P-Dol
ALG9
ALG12
ALG9
ALG6
3.6.1.43
ALG10
ALG8
Asn-X-Ser/Thr
STT
OST
PP-Dol
GCS1
GANAB
GANAB
MAN1
MGAT1
MAN2
MGAT2
MGAT3
MGAT4C
MGAT5
MGAT4
2.4.99.1
2.4.1.38
FUT8
N-glycan precursor biosynthesis
Manα1 — 2 Manα1
Manα1 — 2 Manα1
6 Manα1 3
6 Manβ1 — 4 GlcNAcβ1 — 4 GlcNAcβ1 — PP-Dol
Glcα1 — 2Glcα1 — 3 Glcα1 — 3 Manα1 — 2Manα1 — 2 Manα1
Trimming to form core structure
Manα1 — 2 Manα1
Manα1 — 2 Manα1
6 Manα1 3
6 Manβ1 — 4 GlcNAcβ1 — 4 GlcNAcβ1 — Asn
Glcα1 — 2Glcα1 — 3 Glcα1 — 3 Manα1 — 2Manα1 — 2 Manα1
Glycan extension from core structure
GlcNAcβ1
GlcNAcβ1 — 6 4Manα1 2
Neu5Acα2 — 6Gal β1 — 4GlcNAcβ1
Fucα1
6 GlcNAcβ1 — Asn 4
GlcNAcβ1 — 6 4 Manβ1 — 4 GlcNAcβ1 3
GlcNAcβ1
4Manα1 2
Neu5Acα2 — 6 Galβ1 — 4 GlcNAcβ1
00510 12/8/16
(c) Kanehisa Laboratories

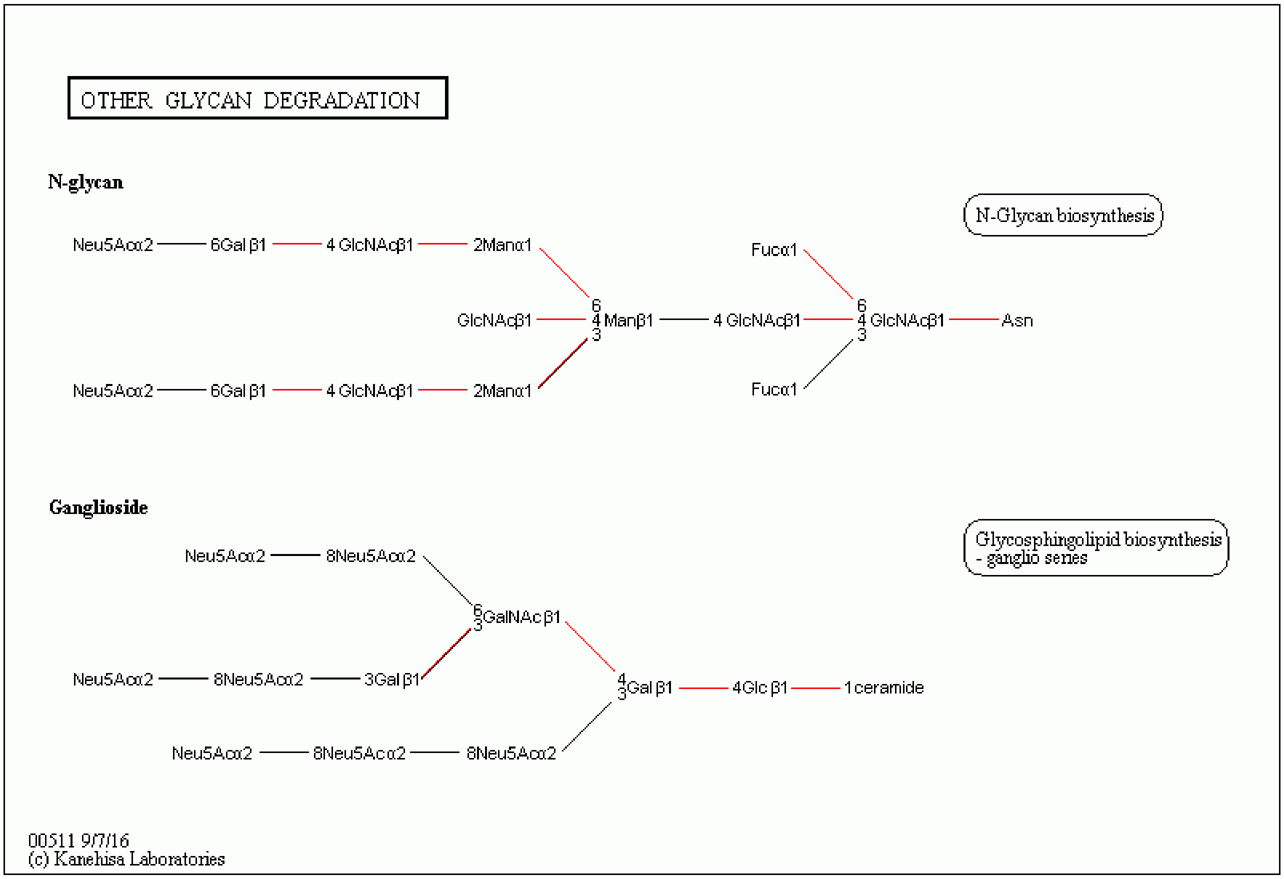
OTHER GLYCAN DEGRADATION
N-glycan
N-Glycan biosynthesis
Neu5Acα2 6Galβ1 4 GlcNAcβ1 2Manα1
GlcNAcβ1 6 4 3 Manβ1 4 GlcNAcβ1 6 4 3 GlcNAcβ1 Asn
Neu5Acα2 6Galβ1 4 GlcNAcβ1 2Manα1
Fucα1
Fucα1
Ganglioside
Glycosphingolipid biosynthesis - ganglio series
Neu5Acα2 8Neu5Acα2
6 3 GalNAcβ1
Neu5Acα2 8Neu5Acα2 3Galβ1
4 3 Galβ1 4Glcβ1 1ceramide
Neu5Acα2 8Neu5Acα2 8Neu5Acα2
00511 9/7/16
(c) Kanehisa Laboratories

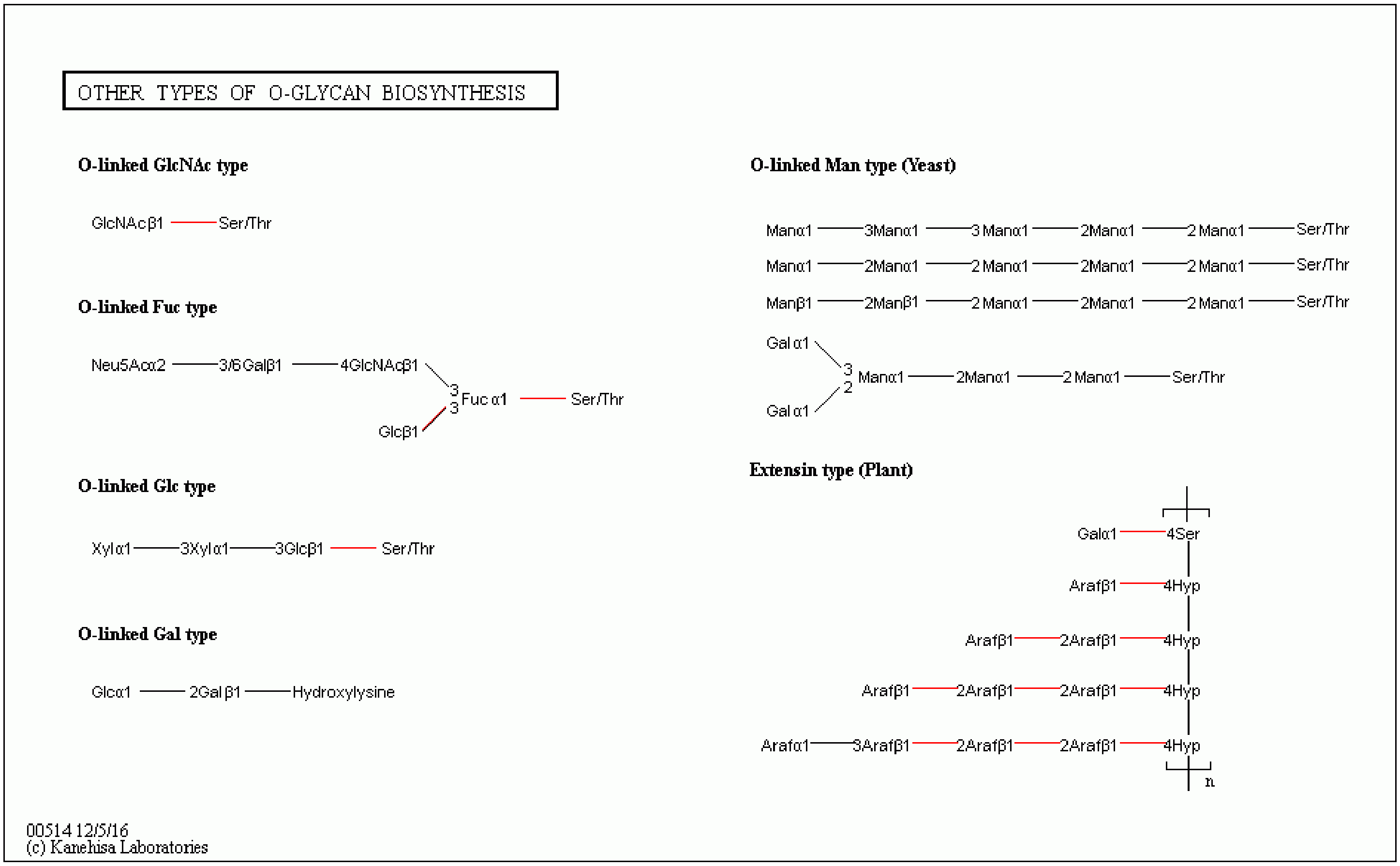
OTHER TYPES OF O-GLYCAN BIOSYNTHESIS
O-linked GlcNAc type
GlcNAcβ1 Ser/Thr
O-linked Fuc type
Neu5Acα2 3/6Galβ1 4GlcNAcβ1 3 3 Fucα1 Ser/Thr
Glcβ1
O-linked Glc type
Xylα1 3Xylα1 3Glcβ1 Ser/Thr
O-linked Gal type
Glcα1 2Galβ1 Hydroxylysine
O-linked Man type (Yeast)
Manα1 3Manα1 3 Manα1 2Manα1 2 Manα1 Ser/Thr
Manα1 2Manα1 2 Manα1 2Manα1 2 Manα1 Ser/Thr
Manβ1 2Manβ1 2 Manα1 2Manα1 2 Manα1 Ser/Thr
Galα1 Galα1 3 2 Manα1 2Manα1 2 Manα1 Ser/Thr
Extensin type (Plant)
Galα1 4Ser
Arafβ1 4Hyp
Arafβ1 2Arafβ1 4Hyp
Arafβ1 2Arafβ1 2Arafβ1 4Hyp
Arafα1 3Arafβ1 2Arafβ1 2Arafβ1 4Hyp
n
00514 12/5/16
(c) Kanehisa Laboratories

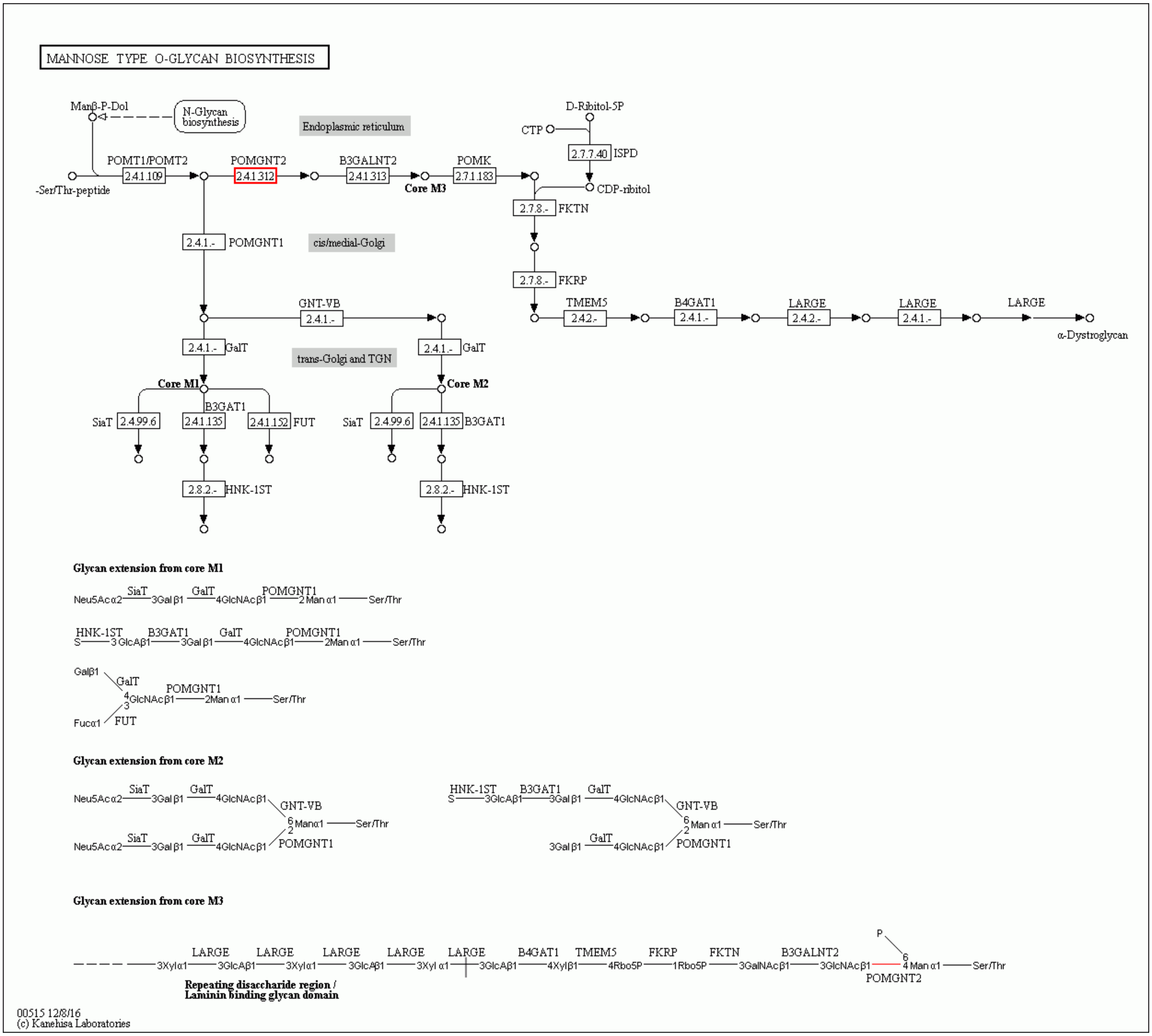
MANNOSE TYPE O-GLYCAN BIOSYNTHESIS
Manβ-P-Dol
N-Glycan biosynthesis
Endoplasmic reticulum
D-Ribitol-5P
CTP
2.7.7.40
ISPD
POMT1/POMT2
2.4.1.109
POMGNT2
2.4.1.312
B3GALNT2
2.4.1.313
POMK
2.7.1.183
CDP-ribitol
-Ser/Thr-peptide
Core M3
2.7.8.-
FKTN
2.4.1.-
POMGNT1
cis/medial-Golgi
2.7.8.-
FKRP
GNT-VB
2.4.1.-
TMEM5
2.4.2.-
B4GAT1
2.4.1.-
LARGE
2.4.2.-
LARGE
2.4.1.-
LARGE
α-Dystroglycan
2.4.1.-
GalT
trans-Golgi and TGN
2.4.1.-
GalT
Core M1
Core M2
B3GAT1
SiaT
2.4.99.6
2.4.1.135
2.4.1.152
FUT
SiaT
2.4.99.6
2.4.1.135
B3GAT1
2.8.2.-
HNK-1ST
2.8.2.-
HNK-1ST
Glycan extension from core M1
Neu5Acα2 SiaT 3Galβ1 GalT 4GlcNAcβ1 POMGNT1 2Manα1 Ser/Thr
S HNK-1ST 3GlcAβ1 B3GAT1 3Galβ1 GalT 4GlcNAcβ1 POMGNT1 2Manα1 Ser/Thr
Galβ1 GalT 4 3 GlcNAcβ1 POMGNT1 2Manα1 Ser/Thr Fucα1 FUT
Glycan extension from core M2
Neu5Acα2 SiaT 3Galβ1 GalT 4GlcNAcβ1 GNT-VB 6 2 Manα1 Ser/Thr
Neu5Acα2 SiaT 3Galβ1 GalT 4GlcNAcβ1 POMGNT1
S HNK-1ST 3GlcAβ1 B3GAT1 3Galβ1 GalT 4GlcNAcβ1 GNT-VB 6 2 Manα1 Ser/Thr
3Galβ1 GalT 4GlcNAcβ1 POMGNT1
Glycan extension from core M3
3Xylα1 LARGE 3GlcAβ1 LARGE 3Xylα1 LARGE 3GlcAβ1 LARGE 3Xylα1 LARGE 3GlcAβ1 B4GAT1 4Xylβ1 TMEM5 4Rbo5P FKRP 1Rbo5P FKTN 3GalNAcβ1 B3GALNT2 3GlcNAcβ1 4 Manα1 Ser/Thr
P 6
POMGNT2
Repeating disaccharide region / Laminin binding glycan domain
00515 12/8/16
(c) Kanehisa Laboratories

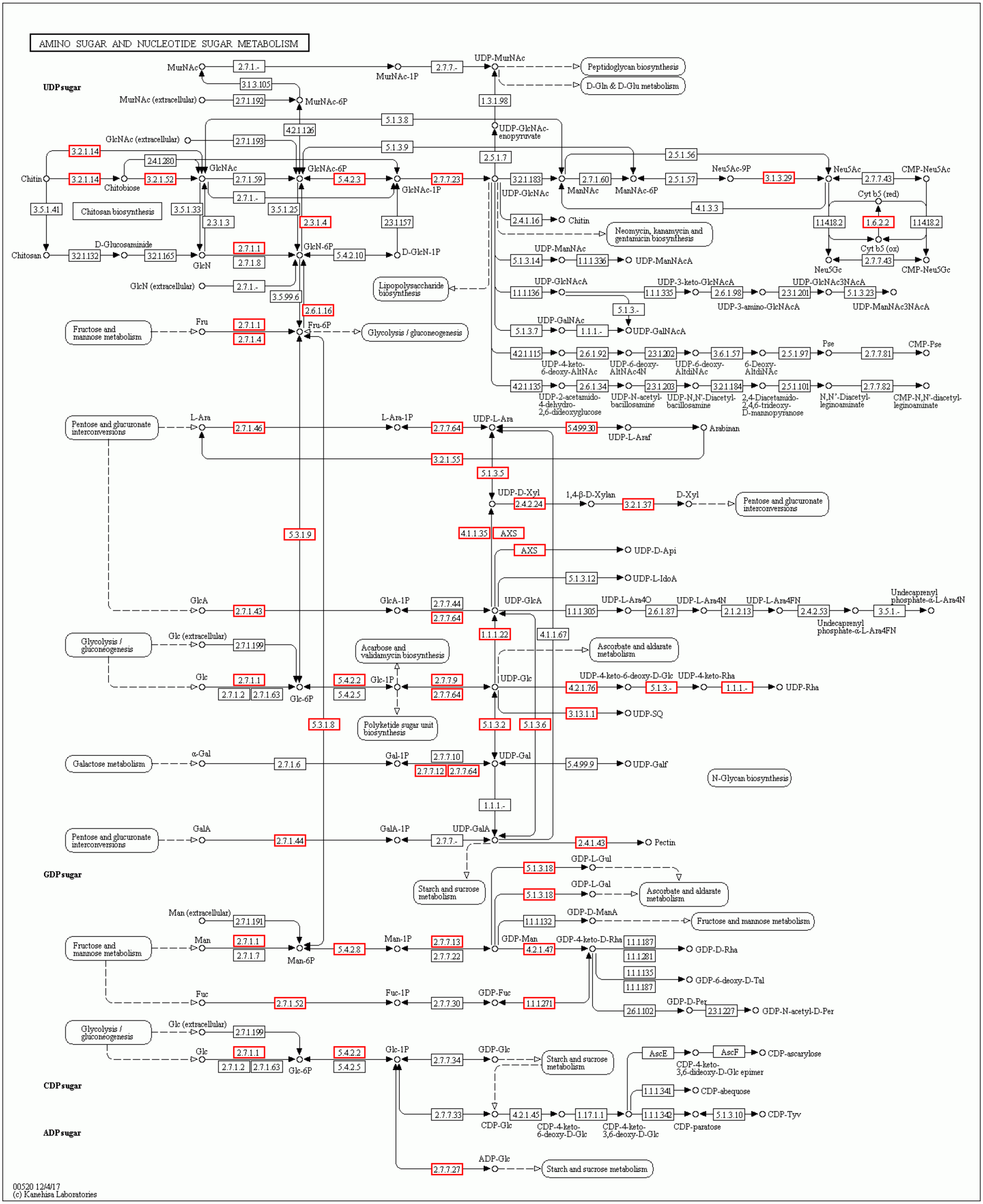
AMINO SUGAR AND NUCLEOTIDE SUGAR METABOLISM
UDP sugar
GDP sugar
CDP sugar
ADP sugar
MurNAc
2.7.1.-
3.1.3.105
MurNAc-1P
2.7.7.-
UDP-MurNAc
Peptidoglycan biosynthesis
D-Gln & D-Glu metabolism
MurNAc (extracellular)
2.7.1.192
MurNAc-6P
1.3.1.98
4.2.1.126
5.1.3.8
UDP-GlcNAc-enopyruvate
GlcNAc (extracellular)
2.7.1.193
5.1.3.9
2.5.1.7
2.5.1.56
3.2.1.14
2.4.1.280
GlcNAc
GlcNAc-6P
Chitin
3.2.1.14
3.2.1.52
2.7.1.59
5.4.2.3
GlcNAc-1P
2.7.7.23
3.2.1.183
2.7.1.60
ManNAc
ManNAc-6P
2.5.1.57
Neu5Ac-9P
3.1.3.29
Neu5Ac
2.7.7.43
CMP-Neu5Ac
Chitobiose
UDP-GlcNAc
2.7.1.-
3.5.1.41
Chitosan biosynthesis
3.5.1.33
3.5.1.25
4.1.3.3
Cyt b5 (red)
2.3.1.3
2.3.1.4
2.3.1.157
2.4.1.16
Chitin
1.14.18.2
1.6.2.2
1.14.18.2
Neomycin, kanamycin and gentamicin biosynthesis
Cyt b5 (ox)
D-Glucosaminide
UDP-ManNAc
Chitosan
3.2.1.132
3.2.1.165
GlcN
2.7.1.1
2.7.1.8
GlcN-6P
5.4.2.10
D-GlcN-1P
5.1.3.14
1.1.1.336
UDP-ManNAcA
Neu5Gc
2.7.7.43
CMP-Neu5Gc
GlcN (extracellular)
2.7.1.-
Lipopolysaccharide biosynthesis
UDP-GlcNAcA
1.1.1.136
UDP-3-keto-GlcNAcA
1.1.1.335
2.6.1.98
UDP-GlcNAc3NAcA
2.3.1.201
5.1.3.23
UDP-3-amino-GlcNAcA
UDP-ManNAc3NAcA
3.5.99.6
2.6.1.16
5.1.3.-
Fructose and mannose metabolism
Fru
2.7.1.1
2.7.1.4
Fru-6P
Glycolysis / gluconeogenesis
UDP-GalNAc
5.1.3.7
1.1.1.-
UDP-GalNAcA
4.2.1.115
2.6.1.92
2.3.1.202
3.6.1.57
2.5.1.97
Pse
2.7.7.81
CMP-Pse
UDP-4-keto-6-deoxy-AltNAc
UDP-6-deoxy-AltNAc4N
UDP-6-deoxy-AltdiNAc
6-Deoxy-AltdiNAc
4.2.1.135
2.6.1.34
2.3.1.203
3.2.1.184
2.5.1.101
2.7.7.82
UDP-2-acetamido-4-dehydro-2,6-dideoxyglucose
UDP-N-acetyl-bacillosamine
UDP-N,N'-Diacetyl-bacillosamine
2,4-Diacetamido-2,4,6-trideoxy-D-mannopyranose
N,N'-Diacetyl-legionaminate
CMP-N,N'-diacetyl-legionaminate
Pentose and glucuronate interconversions
L-Ara
2.7.1.46
L-Ara-1P
2.7.7.64
UDP-L-Ara
5.4.99.30
UDP-L-Araf
Arabinan
3.2.1.55
5.1.3.5
UDP-D-Xyl
2.4.2.24
1,4-β-D-Xylan
3.2.1.37
D-Xyl
Pentose and glucuronate interconversions
5.3.1.9
4.1.1.35
AXS
AXS
UDP-D-Api
5.1.3.12
UDP-L-IdoA
GlcA
2.7.1.43
GlcA-1P
2.7.7.44
2.7.7.64
UDP-GlcA
1.1.1.305
UDP-L-Ara4O
2.6.1.87
UDP-L-Ara4N
2.1.2.13
UDP-L-Ara4FN
2.4.2.53
3.5.1.-
Undecaprenyl phosphate-α-L-Ara4N
Undecaprenyl phosphate-α-L-Ara4FN
1.1.1.22
4.1.1.67
Glycolysis / gluconeogenesis
Glc (extracellular)
2.7.1.199
Acarbose and validamycin biosynthesis
Ascorbate and aldarate metabolism
Glc
2.7.1.1
2.7.1.2
2.7.1.63
Glc-6P
5.4.2.2
5.4.2.5
Glc-1P
2.7.7.9
2.7.7.64
UDP-Glc
UDP-4-keto-6-deoxy-D-Glc
4.2.1.76
5.1.3.-
UDP-4-keto-Rha
1.1.1.-
UDP-Rha
3.13.1.1
UDP-SQ
5.3.1.8
Polyketide sugar unit biosynthesis
5.1.3.2
5.1.3.6
Galactose metabolism
α-Gal
2.7.1.6
Gal-1P
2.7.7.10
2.7.7.12
2.7.7.64
UDP-Gal
5.4.99.9
UDP-Galf
N-Glycan biosynthesis
1.1.1.-
Pentose and glucuronate interconversions
GalA
2.7.1.44
GalA-1P
2.7.7.-
UDP-GalA
2.4.1.43
Pectin
5.1.3.18
GDP-L-Gul
Starch and sucrose metabolism
5.1.3.18
GDP-L-Gal
Ascorbate and aldarate metabolism
Man (extracellular)
2.7.1.191
1.1.1.132
GDP-D-ManA
Fructose and mannose metabolism
Fructose and mannose metabolism
Man
2.7.1.1
2.7.1.7
Man-6P
5.4.2.8
Man-1P
2.7.7.13
2.7.7.22
GDP-Man
4.2.1.47
GDP-4-keto-D-Rha
1.1.1.187
1.1.1.281
GDP-D-Rha
1.1.1.135
1.1.1.187
GDP-6-deoxy-D-Tal
Fuc
2.7.1.52
Fuc-1P
2.7.7.30
GDP-Fuc
1.1.1.271
2.6.1.102
GDP-D-Per
2.3.1.227
GDP-N-acetyl-D-Per
Glycolysis / gluconeogenesis
Glc (extracellular)
2.7.1.199
Glc
2.7.1.1
2.7.1.2
2.7.1.63
Glc-6P
5.4.2.2
5.4.2.5
Glc-1P
2.7.7.34
GDP-Glc
Starch and sucrose metabolism
AscE
AscF
CDP-ascarylose
CDP-4-keto-3,6-dideoxy-D-Glc epimer
1.1.1.341
CDP-abequose
2.7.7.33
CDP-Glc
4.2.1.45
CDP-4-keto-6-deoxy-D-Glc
1.17.1.1
CDP-4-keto-3,6-deoxy-D-Glc
1.1.1.342
CDP-paratose
5.1.3.10
CDP-Tyv
ADP-Glc
2.7.7.27
Starch and sucrose metabolism
00520 12/4/17
(c) Kanehisa Laboratories

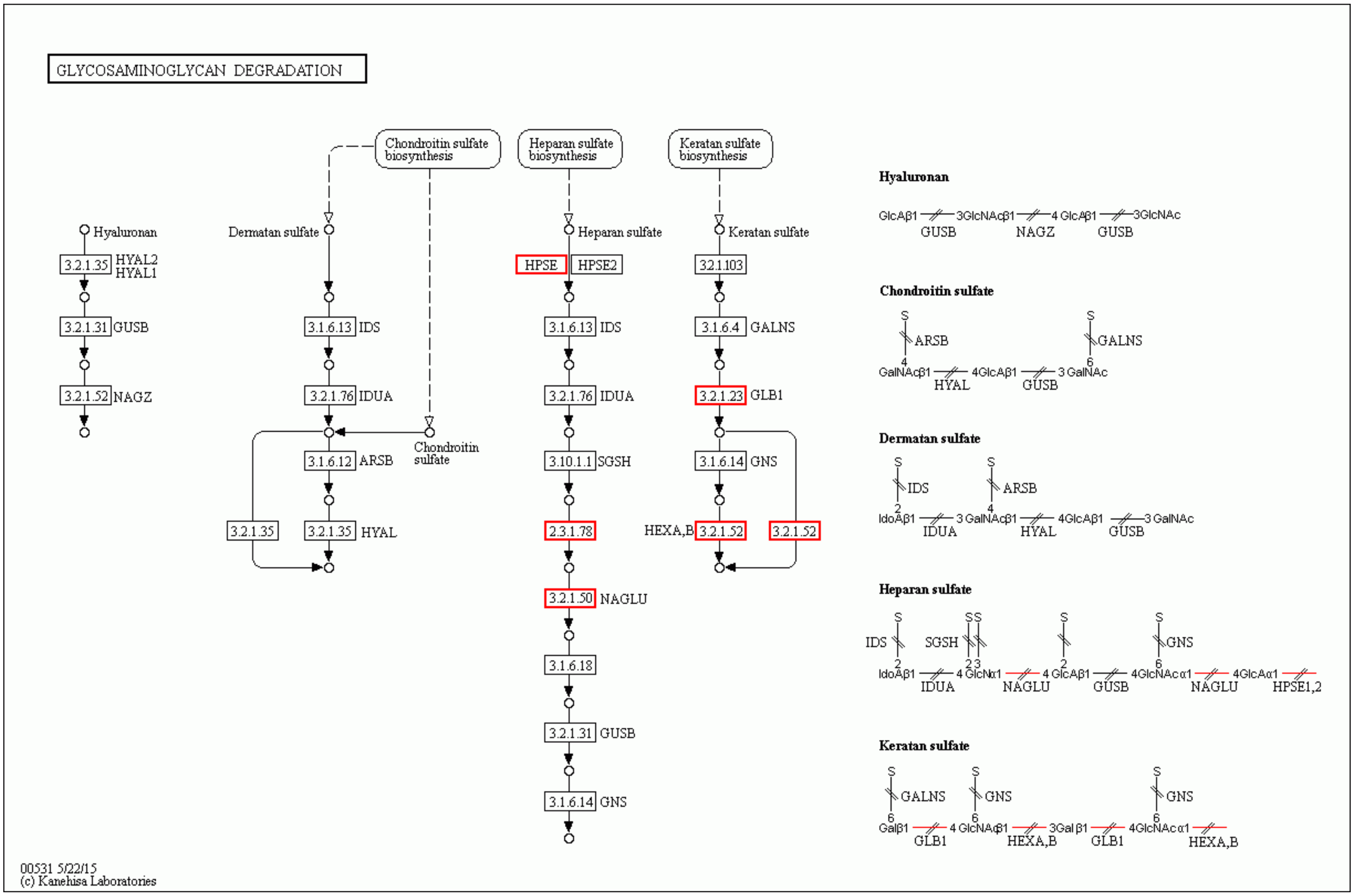
GLYCOSAMINOGLYCAN DEGRADATION
Chondroitin sulfate biosynthesis
Heparan sulfate biosynthesis
Keratan sulfate biosynthesis
Hyaluronan
3.2.1.35 HYAL2 HYAL1
3.2.1.31 GUSB
3.2.1.52 NAGZ
Dermatan sulfate
3.1.6.13 IDS
3.2.1.76 IDUA
Chondroitin sulfate
3.1.6.12 ARSB
3.2.1.35
3.2.1.35 HYAL
Heparan sulfate
HPSE
HPSE2
3.1.6.13 IDS
3.2.1.76 IDUA
3.10.1.1 SGSH
2.3.1.78
3.2.1.50 NAGLU
3.1.6.18
3.2.1.31 GUSB
3.1.6.14 GNS
Keratan sulfate
3.2.1.103
3.1.6.4 GALNS
3.2.1.23 GLB1
3.1.6.14 GNS
HEXA,B 3.2.1.52
3.2.1.52
Hyaluronan
GlcAβ1 3GlcNAcβ1 4 GlcAβ1 3GlcNAc
GUSB NAGZ GUSB
Chondroitin sulfate
S ARSB 4
S GALNS 6
GalNAcβ1 4GlcAβ1 3 GalNAc
HYAL GUSB
Dermatan sulfate
S IDS 2
S ARSB 4
IdoAβ1 3 GalNAcβ1 4GlcAβ1 3 GalNAc
IDUA HYAL GUSB
Heparan sulfate
IDS SGSH GNS
IdoAβ1 4 GlcNα1 4 GlcAβ1 4GlcNAcα1 4GlcAα1
IDUA NAGLU GUSB NAGLU HPSE1,2
Keratan sulfate
GALNS GNS GNS
Galβ1 4 GlcNAcβ1 3Gal β1 4GlcNAcα1
GLB1 HEXA,B GLB1 HEXA,B
00531 5/22/15
(c) Kanehisa Laboratories

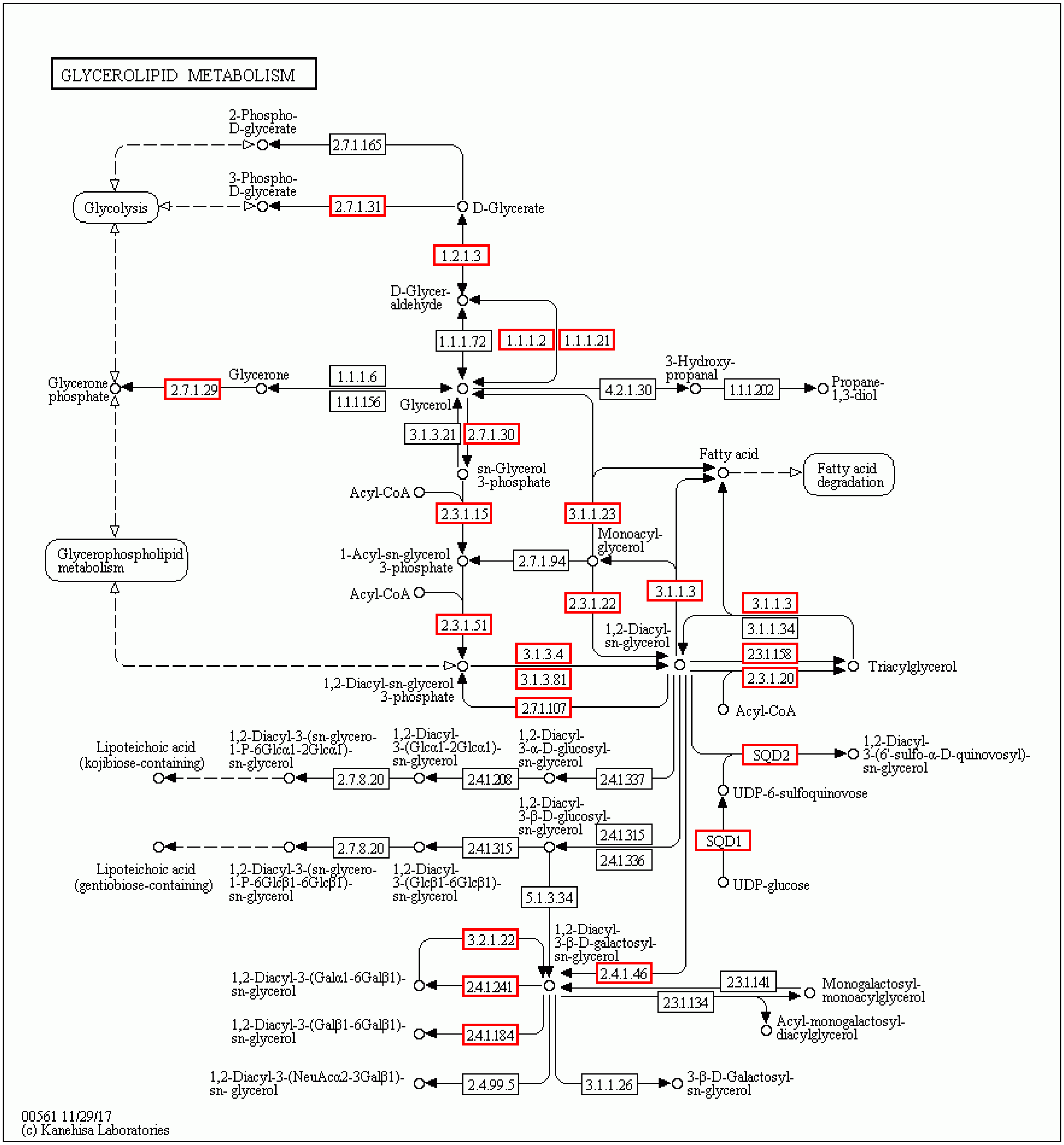
GLYCEROLIPID METABOLISM
2-Phospho-D-glycerate
2.7.1.165
3-Phospho-D-glycerate
2.7.1.31
D-Glycerate
Glycolysis
1.2.1.3
D-Glycer-aldehyde
1.1.1.72
1.1.1.2
1.1.1.21
Glycerone phosphate
2.7.1.29
Glycerone
1.1.1.6
1.1.1.156
Glycerol
4.2.1.30
3-Hydroxy-propanal
1.1.1.202
Propane-1,3-diol
3.1.3.21
2.7.1.30
sn-Glycerol 3-phosphate
Acyl-CoA
2.3.1.15
3.1.1.23
Fatty acid
Fatty acid degradation
Monoacyl-glycerol
Glycerophospholipid metabolism
1-Acyl-sn-glycerol 3-phosphate
2.7.1.94
Acyl-CoA
2.3.1.22
3.1.1.3
3.1.1.3
3.1.1.34
2.3.1.51
1,2-Diacyl-sn-glycerol
3.1.3.4
2.3.1.158
Triacylglycerol
3.1.3.81
2.3.1.20
1,2-Diacyl-sn-glycerol 3-phosphate
2.7.1.107
Acyl-CoA
SQD2
1,2-Diacyl-3-(6'-sulfo-α-D-quinovosyl)-sn-glycerol
Lipoteichoic acid (kojibiose-containing)
1,2-Diacyl-3-(sn-glycero-1-P-6Glcα1-2Glcα1)-sn-glycerol
2.7.8.20
1,2-Diacyl-3-(Glcα1-2Glcα1)-sn-glycerol
2.4.1.208
1,2-Diacyl-3-α-D-glucosyl-sn-glycerol
2.4.1.337
UDP-6-sulfoquinovose
1,2-Diacyl-3-β-D-glucosyl-sn-glycerol
2.4.1.315
SQD1
2.7.8.20
2.4.1.315
2.4.1.336
Lipoteichoic acid (gentiobiose-containing)
1,2-Diacyl-3-(sn-glycero-1-P-6Glcβ1-6Glcβ1)-sn-glycerol
1,2-Diacyl-3-(Glcβ1-6Glcβ1)-sn-glycerol
UDP-glucose
5.1.3.34
3.2.1.22
1,2-Diacyl-3-β-D-galactosyl-sn-glycerol
2.4.1.46
1,2-Diacyl-3-(Galα1-6Galβ1)-sn-glycerol
2.4.1.241
2.3.1.141
Monogalactosyl-monoacylglycerol
2.3.1.134
Acyl-monogalactosyl-diacylglycerol
1,2-Diacyl-3-(Galβ1-6Galβ1)-sn-glycerol
2.4.1.184
1,2-Diacyl-3-(NeuAcα2-3Galβ1)-sn- glycerol
2.4.99.5
3.1.1.26
3-β-D-Galactosyl-sn-glycerol
00561 11/29/17
(c) Kanehisa Laboratories

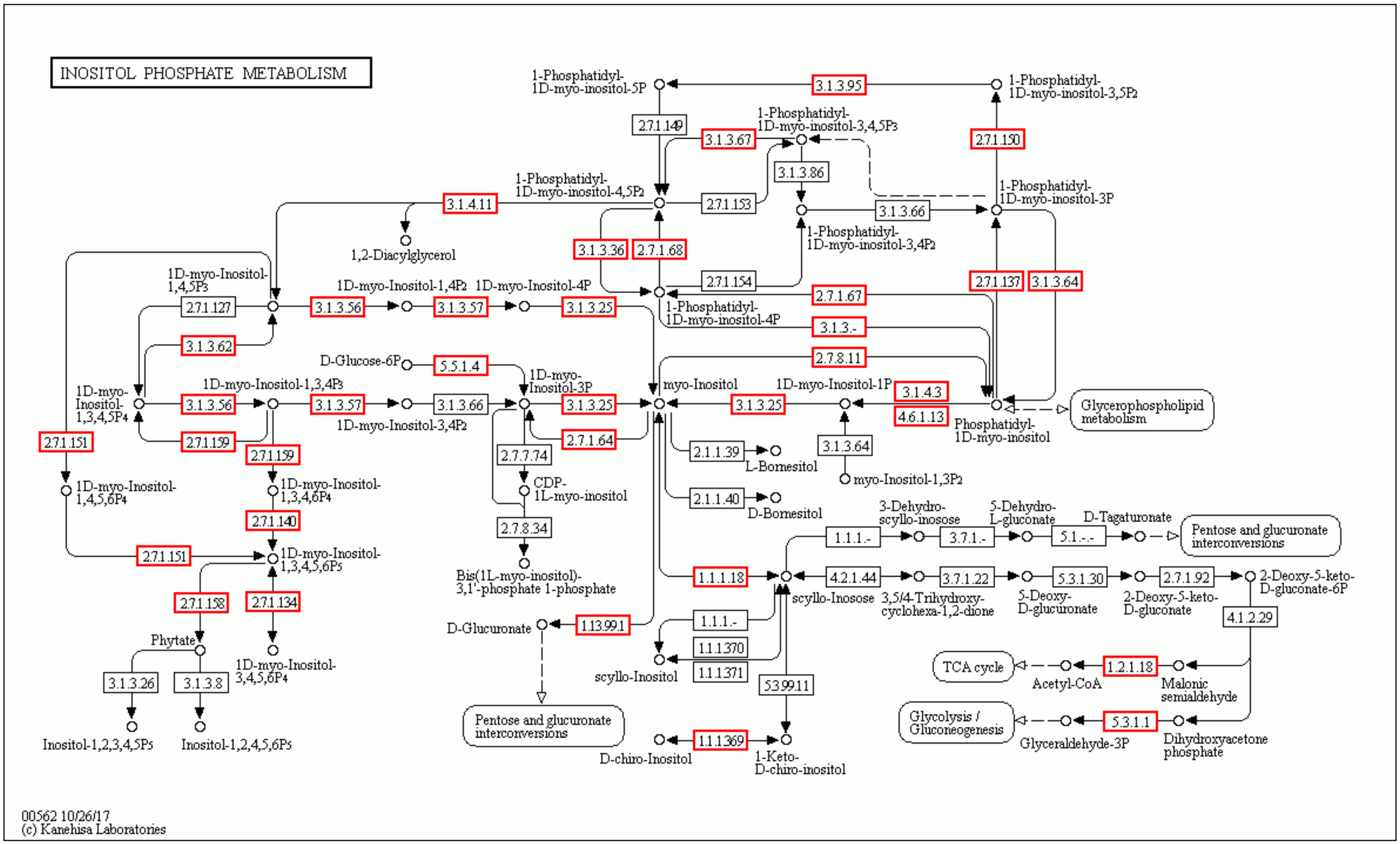
INOSITOL PHOSPHATE METABOLISM
1-Phosphatidyl-1D-myo-inositol-5P
3.1.3.95
1-Phosphatidyl-1D-myo-inositol-3,5P2
2.7.1.149
3.1.3.67
1-Phosphatidyl-1D-myo-inositol-3,4,5P3
2.7.1.150
3.1.3.86
1-Phosphatidyl-1D-myo-inositol-4,5P2
3.1.4.11
2.7.1.153
3.1.3.66
1-Phosphatidyl-1D-myo-inositol-3P
1,2-Diacylglycerol
3.1.3.36
2.7.1.68
1-Phosphatidyl-1D-myo-inositol-3,4P2
1D-myo-Inositol-1,4,5P3
1D-myo-Inositol-1,4P2
1D-myo-Inositol-4P
2.7.1.154
2.7.1.137
3.1.3.64
2.7.1.67
2.7.1.127
3.1.3.56
3.1.3.57
3.1.3.25
1-Phosphatidyl-1D-myo-inositol-4P
3.1.3.-
3.1.3.62
2.7.8.11
D-Glucose-6P
5.5.1.4
1D-myo-Inositol-3P
1D-myo-Inositol-1,3,4,5P4
1D-myo-Inositol-1,3,4P3
myo-Inositol
1D-myo-Inositol-1P
3.1.4.3
3.1.3.56
3.1.3.57
3.1.3.66
3.1.3.25
3.1.3.25
Glycerophospholipid metabolism
4.6.1.13
1D-myo-Inositol-3,4P2
2.7.1.64
Phosphatidyl-1D-myo-inositol
2.7.1.151
2.7.1.159
2.7.1.159
2.7.7.74
2.1.1.39
L-Bornesitol
3.1.3.64
1D-myo-Inositol-1,4,5,6P4
1D-myo-Inositol-1,3,4,6P4
CDP-1L-myo-inositol
myo-Inositol-1,3P2
2.1.1.40
D-Bornesitol
3-Dehydro-scyllo-inosose
5-Dehydro-L-gluconate
D-Tagaturonate
2.7.1.140
2.7.8.34
1.1.1.-
3.7.1.-
5.1.-.-
Pentose and glucuronate interconversions
2.7.1.151
1D-myo-Inositol-1,3,4,5,6P5
Bis(1L-myo-inositol)-3,1'-phosphate 1-phosphate
1.1.1.18
4.2.1.44
3.7.1.22
5.3.1.30
2.7.1.92
2-Deoxy-5-keto-D-gluconate-6P
2.7.1.158
2.7.1.134
scyllo-Inosose
3,5/4-Trihydroxy-cyclohexa-1,2-dione
5-Deoxy-D-glucuronate
2-Deoxy-5-keto-D-gluconate
Phytate
D-Glucuronate
1.13.99.1
1.1.1.-
4.1.2.29
1D-myo-Inositol-3,4,5,6P4
1.1.1.370
TCA cycle
Acetyl-CoA
1.2.1.18
Malonic semialdehyde
3.1.3.26
3.1.3.8
scyllo-Inositol
1.1.1.371
5.3.99.11
Pentose and glucuronate interconversions
Glycolysis / Gluconeogenesis
5.3.1.1
Glyceraldehyde-3P
Dihydroxyacetone phosphate
Inositol-1,2,3,4,5P5
Inositol-1,2,4,5,6P5
D-chiro-Inositol
1.1.1.369
1-Keto-D-chiro-inositol
00562 10/26/17
(c) Kanehisa Laboratories

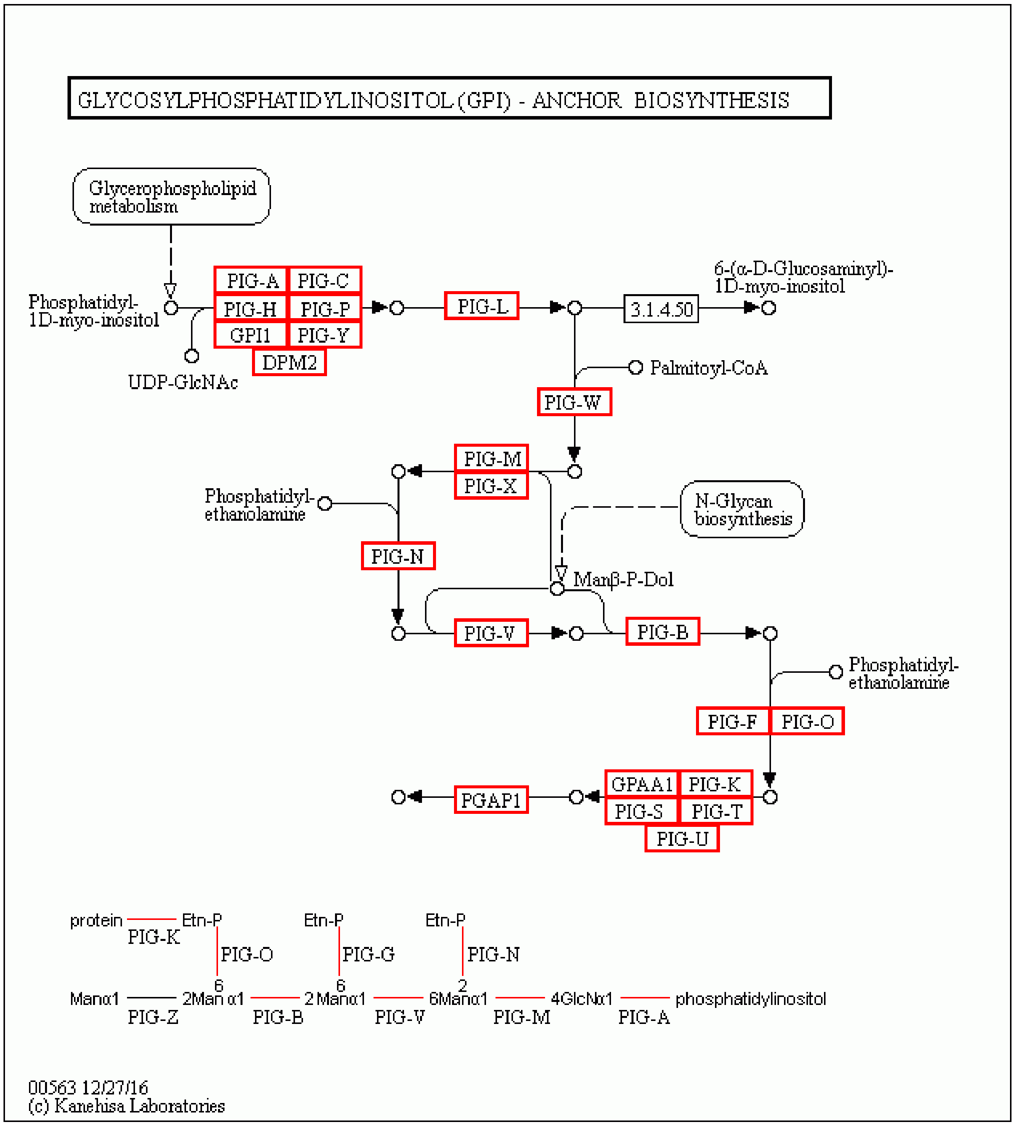

GLYCOSYLPHOSPHATIDYLINOSITOL (GPI) - ANCHOR BIOSYNTHESIS
Glycerophospholipid metabolism
Phosphatidyl-1D-myo-inositol
UDP-GlcNAc
PIG-A
PIG-C
PIG-H
PIG-P
GPI1
PIG-Y
DPM2
PIG-L
3.1.4.50
6-(α-D-Glucosaminyl)-1D-myo-inositol
Palmitoyl-CoA
PIG-W
PIG-M
PIG-X
Phosphatidyl-ethanolamine
N-Glycan biosynthesis
PIG-N
Manβ-P-Dol
PIG-V
PIG-B
Phosphatidyl-ethanolamine
PIG-F
PIG-O
GPAA1
PIG-K
PIG-S
PIG-T
PIG-U
PGAP1
protein
Etn-P
Etn-P
Etn-P
PIG-K
PIG-O
PIG-G
PIG-N
Manα1
2Man α1
2 Manα1
6Manα1
4GlcNα1
phosphatidylinositol
PIG-Z
PIG-B
PIG-V
PIG-M
PIG-A
00563 12/27/16
(c) Kanehisa Laboratories

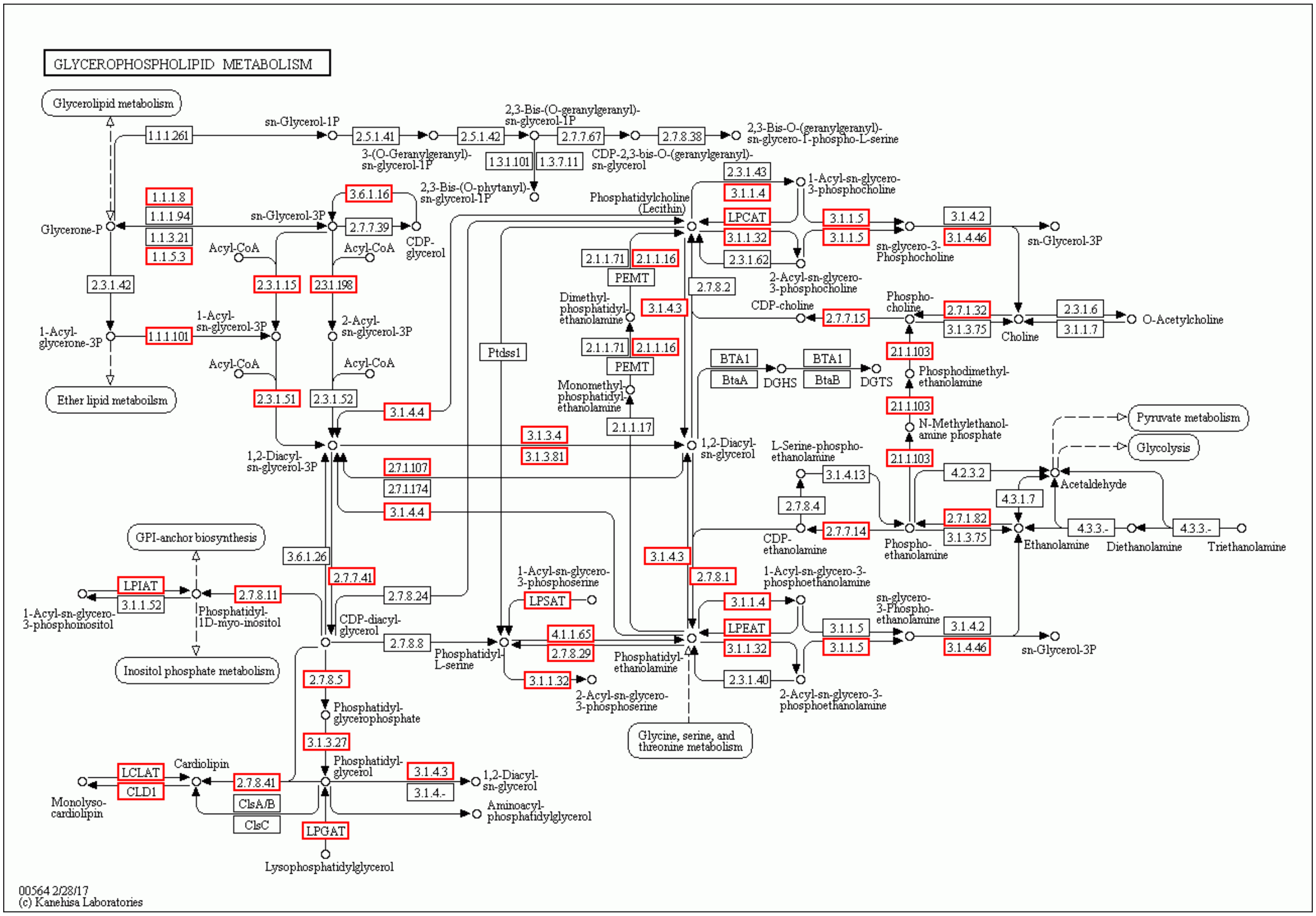
GLYCEROPHOSPHOLIPID METABOLISM
Glycerolipid metabolism
Ether lipid metaboilsm
GPI-anchor biosynthesis
Inositol phosphate metabolism
Glycine, serine, and threonine metabolism
Pyruvate metabolism
Glycolysis
sn-Glycerol-1P
3-(O-Geranylgeranyl)-sn-glycerol-1P
2,3-Bis-(O-geranylgeranyl)-sn-glycerol-1P
CDP-2,3-bis-O-(geranylgeranyl)-sn-glycerol
2,3-Bis-O-(geranylgeranyl)-sn-glycero-1-phospho-L-serine
2,3-Bis-(O-phytanyl)-sn-glycerol-1P
Glycerone-P
sn-Glycerol-3P
CDP-glycerol
Acyl-CoA
1-Acyl-glycerone-3P
1-Acyl-sn-glycerol-3P
2-Acyl-sn-glycerol-3P
1,2-Diacyl-sn-glycerol-3P
Phosphatidylcholine (Lecithin)
1-Acyl-sn-glycero-3-phosphocholine
2-Acyl-sn-glycero-3-phosphocholine
sn-glycero-3-Phosphocholine
sn-Glycerol-3P
Dimethyl-phosphatidyl-ethanolamine
Monomethyl-phosphatidyl-ethanolamine
CDP-choline
Phospho-choline
Choline
O-Acetylcholine
DGHS
DGTS
Phosphodimethyl-ethanolamine
N-Methylethanol-amine phosphate
1,2-Diacyl-sn-glycerol
L-Serine-phospho-ethanolamine
CDP-ethanolamine
Phospho-ethanolamine
Ethanolamine
Diethanolamine
Triethanolamine
Acetaldehyde
1-Acyl-sn-glycero-3-phosphoinositol
Phosphatidyl-1D-myo-inositol
CDP-diacyl-glycerol
Phosphatidyl-L-serine
1-Acyl-sn-glycero-3-phosphoserine
2-Acyl-sn-glycero-3-phosphoserine
Phosphatidyl-ethanolamine
1-Acyl-sn-glycero-3-phosphoethanolamine
2-Acyl-sn-glycero-3-phosphoethanolamine
sn-glycero-3-Phospho-ethanolamine
Phosphatidyl-glycerophosphate
Phosphatidyl-glycerol
Cardiolipin
Monolyso-cardiolipin
Aminoacyl-phosphatidylglycerol
Lysophosphatidylglycerol
1.1.1.261
2.5.1.41
2.5.1.42
2.7.7.67
2.7.8.38
1.3.1.101
1.3.7.11
1.1.1.8
1.1.1.94
1.1.3.21
1.1.5.3
3.6.1.16
2.7.7.39
2.3.1.42
2.3.1.15
2.3.1.198
1.1.1.101
2.3.1.51
2.3.1.52
3.1.4.4
Ptdss1
2.3.1.43
3.1.1.4
LPCAT
3.1.1.32
2.3.1.62
3.1.1.5
3.1.4.2
3.1.4.46
2.1.1.71
2.1.1.16
PEMT
3.1.4.3
2.7.8.2
2.7.7.15
2.7.1.32
3.1.3.75
2.3.1.6
3.1.1.7
BTA1
BtaA
BtaB
2.1.1.103
2.1.1.17
3.1.3.4
3.1.3.81
2.7.1.107
2.7.1.174
3.1.4.13
2.7.8.4
4.2.3.2
4.3.1.7
2.7.7.14
2.7.1.82
4.3.3.-
3.6.1.26
2.7.7.41
2.7.8.24
2.7.8.1
LPIAT
3.1.1.52
2.7.8.11
LPSAT
4.1.1.65
2.7.8.29
2.7.8.8
LPEAT
2.3.1.40
2.7.8.5
3.1.3.27
3.1.4.-
LCLAT
CLD1
2.7.8.41
ClsA/B
ClsC
LPGAT
00564 2/28/17
(c) Kanehisa Laboratories

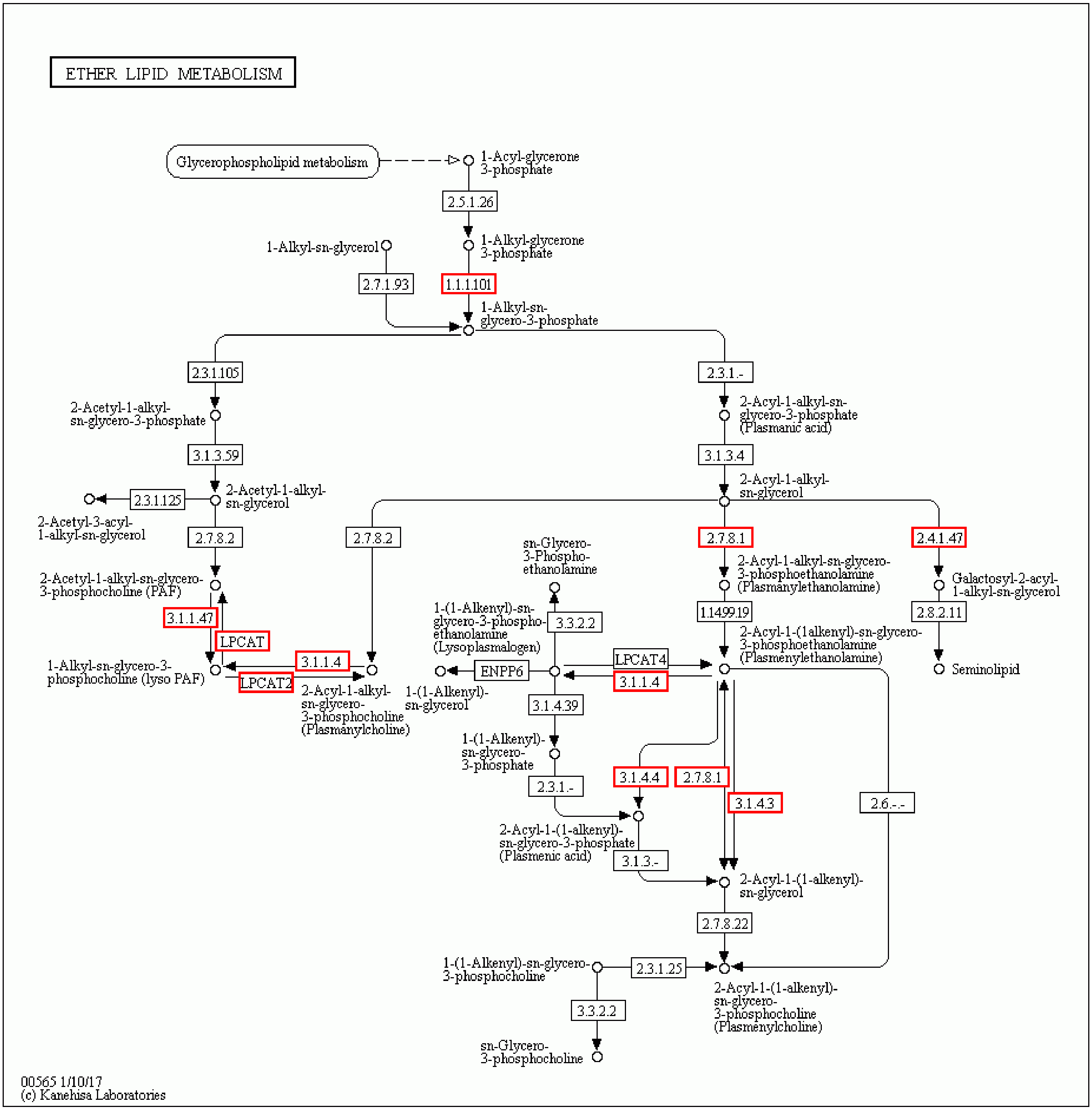
ETHER LIPID METABOLISM
Glycerophospholipid metabolism
1-Acyl-glycerone 3-phosphate
2.5.1.26
1-Alkyl-glycerone 3-phosphate
1-Alkyl-sn-glycerol
2.7.1.93
1.1.1.101
1-Alkyl-sn-glycero-3-phosphate
2.3.1.105
2.3.1.-
2-Acetyl-1-alkyl-sn-glycero-3-phosphate
2-Acyl-1-alkyl-sn-glycero-3-phosphate (Plasmanic acid)
3.1.3.59
3.1.3.4
2.3.1.125
2-Acetyl-1-alkyl-sn-glycerol
2-Acyl-1-alkyl-sn-glycerol
2-Acetyl-3-acyl-1-alkyl-sn-glycerol
2.7.8.2
2.7.8.2
2.7.8.1
2.4.1.47
sn-Glycero-3-Phospho-ethanolamine
2-Acyl-1-alkyl-sn-glycero-3-phosphoethanolamine (Plasmanylethanolamine)
Galactosyl-2-acyl-1-alkyl-sn-glycerol
2-Acetyl-1-alkyl-sn-glycero-3-phosphocholine (PAF)
1.14.99.19
2.8.2.11
3.1.1.47
1-(1-Alkenyl)-sn-glycero-3-phospho-ethanolamine (Lysoplasmalogen)
3.3.2.2
2-Acyl-1-(1alkenyl)-sn-glycero-3-phosphoethanolamine (Plasmenylethanolamine)
LPCAT
LPCAT4
3.1.1.4
ENPP6
3.1.1.4
Seminolipid
1-Alkyl-sn-glycero-3-phosphocholine (lyso PAF)
LPCAT2
2-Acyl-1-alkyl-sn-glycero-3-phosphocholine (Plasmanylcholine)
1-(1-Alkenyl)-sn-glycerol
3.1.4.39
1-(1-Alkenyl)-sn-glycero-3-phosphate
3.1.4.4
2.7.8.1
3.1.4.3
2.3.1.-
2.6.-.-
2-Acyl-1-(1-alkenyl)-sn-glycero-3-phosphate (Plasmenic acid)
3.1.3.-
2-Acyl-1-(1-alkenyl)-sn-glycerol
2.7.8.22
1-(1-Alkenyl)-sn-glycero-3-phosphocholine
2.3.1.25
2-Acyl-1-(1-alkenyl)-sn-glycero-3-phosphocholine (Plasmenylcholine)
3.3.2.2
sn-Glycero-3-phosphocholine
00565 1/10/17
(c) Kanehisa Laboratories

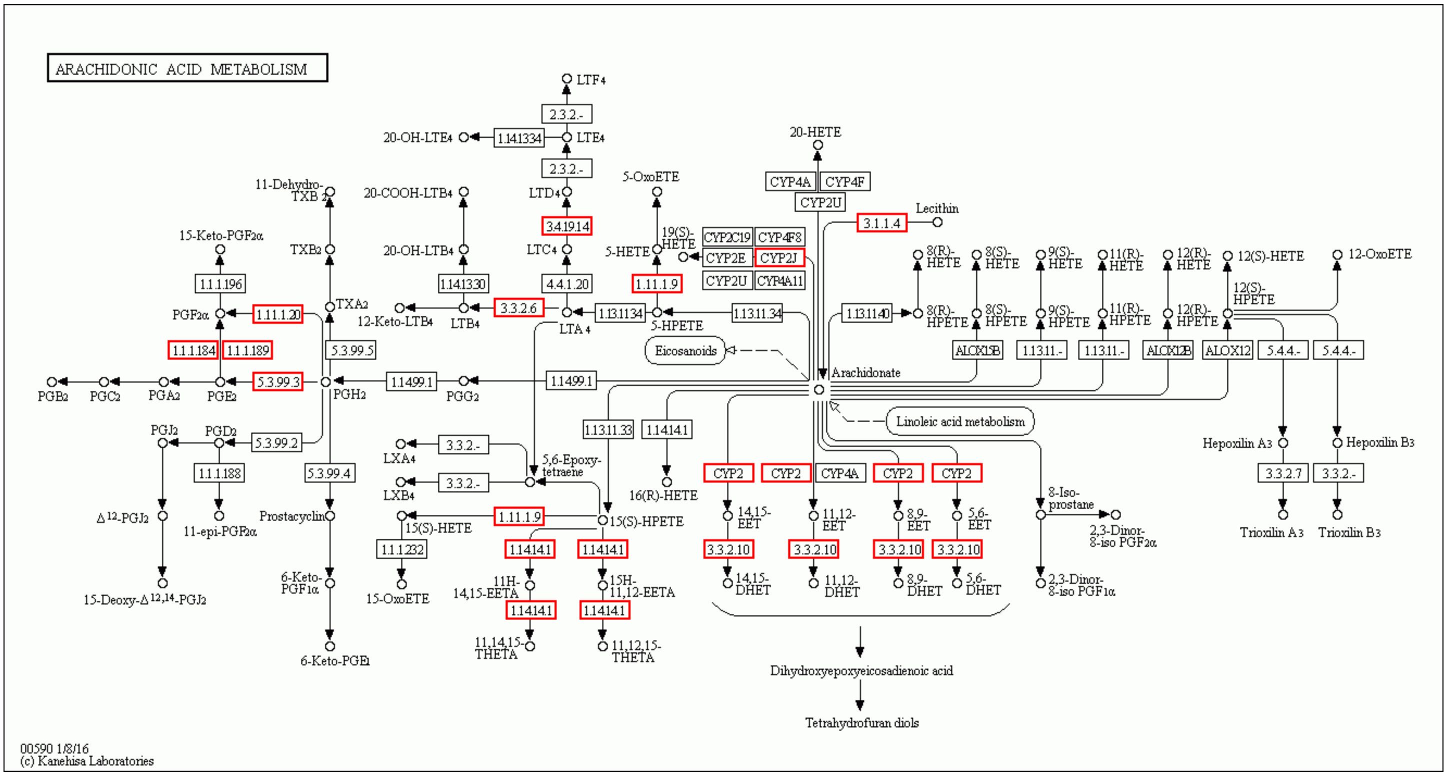

ARACHIDONIC ACID METABOLISM
LTF4
2.3.2.-
20-OH-LTE4
1.14.13.34
LTE4
2.3.2.-
20-HETE
CYP4A
CYP4F
CYP2U
11-Dehydro-TXB2
20-COOH-LTB4
LTD4
5-OxoETE
3.1.1.4
Lecithin
3.4.19.14
15-Keto-PGF2α
TXB2
20-OH-LTB4
LTC4
5-HETE
19(S)-HETE
CYP2C19
CYP4F8
CYP2E
CYP2J
CYP2U
CYP4A11
1.1.1.196
1.14.13.30
4.4.1.20
1.11.1.9
8(R)-HETE
8(S)-HETE
9(S)-HETE
11(R)-HETE
12(R)-HETE
12(S)-HETE
12-OxoETE
12(S)-HPETE
TXA2
PGF2α
1.11.1.20
12-Keto-LTB4
3.3.2.6
LTB4
LTA4
1.13.11.34
5-HPETE
1.13.11.34
1.13.11.40
8(R)-HPETE
8(S)-HPETE
9(S)-HPETE
11(R)-HPETE
12(R)-HPETE
1.1.1.184
1.1.1.189
5.3.99.5
Eicosanoids
ALOX15B
1.13.11.-
1.13.11.-
ALOX12B
ALOX12
5.4.4.-
5.4.4.-
PGB2
PGC2
PGA2
PGE2
5.3.99.3
PGH2
1.14.99.1
PGG2
1.14.99.1
Arachidonate
Linoleic acid metabolism
1.13.11.33
1.14.14.1
PGJ2
PGD2
5.3.99.2
3.3.2.-
LXA4
5,6-Epoxy-tetraene
Hepoxilin A3
Hepoxilin B3
1.1.1.188
5.3.99.4
3.3.2.-
LXB4
CYP2
CYP2
CYP4A
CYP2
CYP2
3.3.2.7
3.3.2.-
16(R)-HETE
8-Iso-prostane
Δ12-PGJ2
11-epi-PGF2α
Prostacyclin
1.11.1.9
15(S)-HETE
15(S)-HPETE
14,15-EET
11,12-EET
8,9-EET
5,6-EET
Trioxilin A3
Trioxilin B3
1.1.1.232
1.14.14.1
1.14.14.1
3.3.2.10
3.3.2.10
3.3.2.10
3.3.2.10
2,3-Dinor-8-iso PGF2α
6-Keto-PGF1α
11H-14,15-EETA
15H-11,12-EETA
14,15-DHET
11,12-DHET
8,9-DHET
5,6-DHET
2,3-Dinor-8-iso PGF1α
15-Deoxy-Δ12,14-PGJ2
15-OxoETE
1.14.14.1
1.14.14.1
11,14,15-THETA
11,12,15-THETA
6-Keto-PGE1
Dihydroxyepoxyeicosadienoic acid
Tetrahydrofuran diols
00590 1/8/16
(c) Kanehisa Laboratories

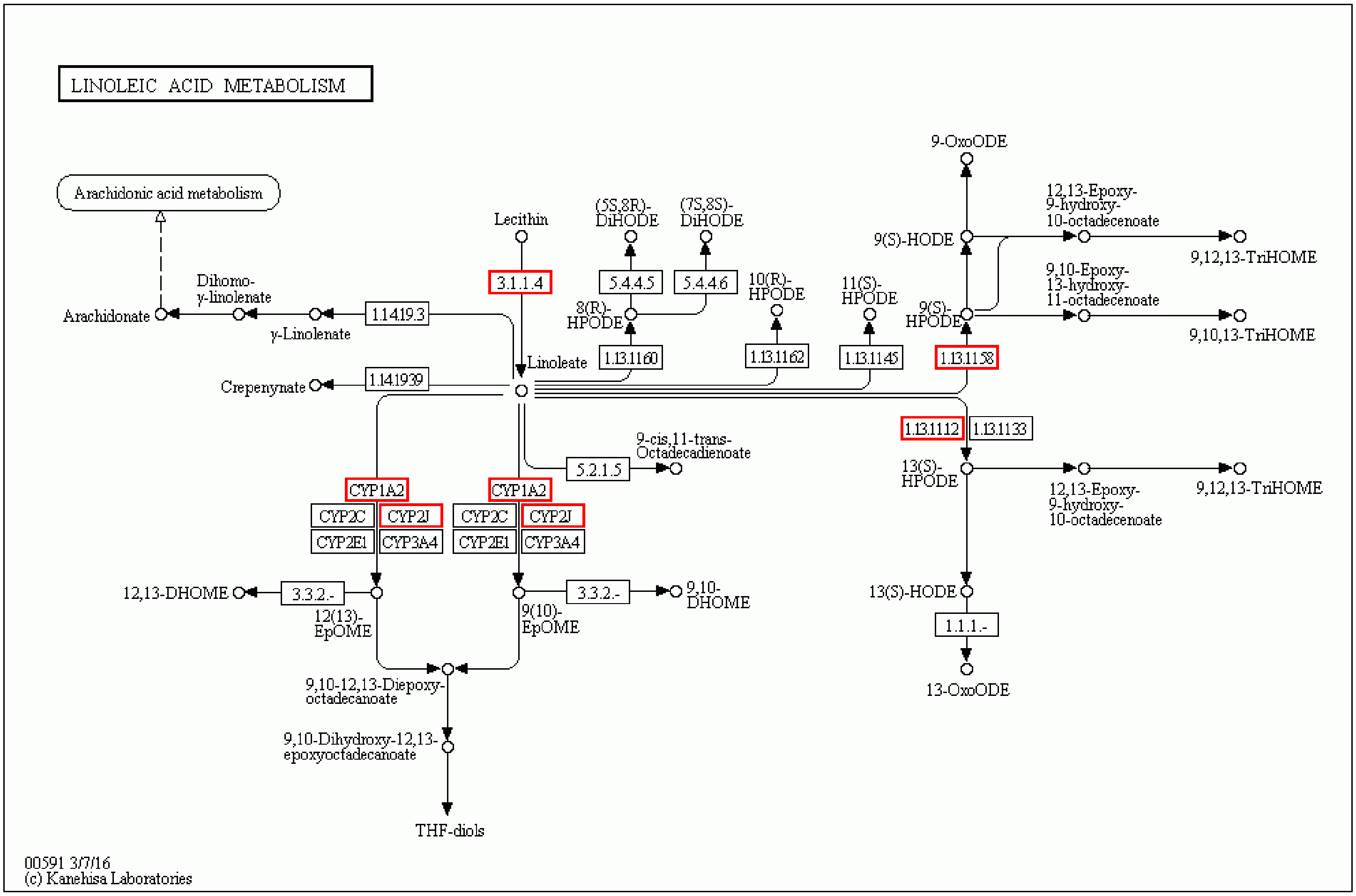

LINOLEIC ACID METABOLISM
9-OxoODE
Arachidonic acid metabolism
12,13-Epoxy-9-hydroxy-10-octadecenoate
Lecithin
(9S,8R)-DiHODE
(7S,8S)-DiHODE
9(S)-HODE
9,12,13-TriHOME
3.1.1.4
5.4.4.5
5.4.4.6
9,10-Epoxy-13-hydroxy-11-octadecenoate
Dihomo-γ-linolenate
8(R)-HPODE
10(R)-HPODE
11(S)-HPODE
9(S)-HPODE
Arachidonate
1.14.19.3
γ-Linolenate
9,10,13-TriHOME
Linoleate
1.13.11.60
1.13.11.62
1.13.11.45
1.13.11.58
Crepenynate
1.14.19.39
1.13.11.12
1.13.11.33
9-cis,11-trans-Octadecadienoate
5.2.1.5
13(S)-HPODE
12,13-Epoxy-9-hydroxy-10-octadecenoate
9,12,13-TriHOME
CYP1A2
CYP1A2
CYP2C
CYP2J
CYP2C
CYP2J
CYP2E1
CYP3A4
CYP2E1
CYP3A4
12,13-DHOME
3.3.2.-
3.3.2.-
9,10-DHOME
13(S)-HODE
12(13)-EpOME
9(10)-EpOME
1.1.1.-
9,10-12,13-Diepoxy-octadecanoate
13-OxoODE
9,10-Dihydroxy-12,13-epoxyoctadecanoate
THF-diols
00591 3/7/16
(c) Kanehisa Laboratories

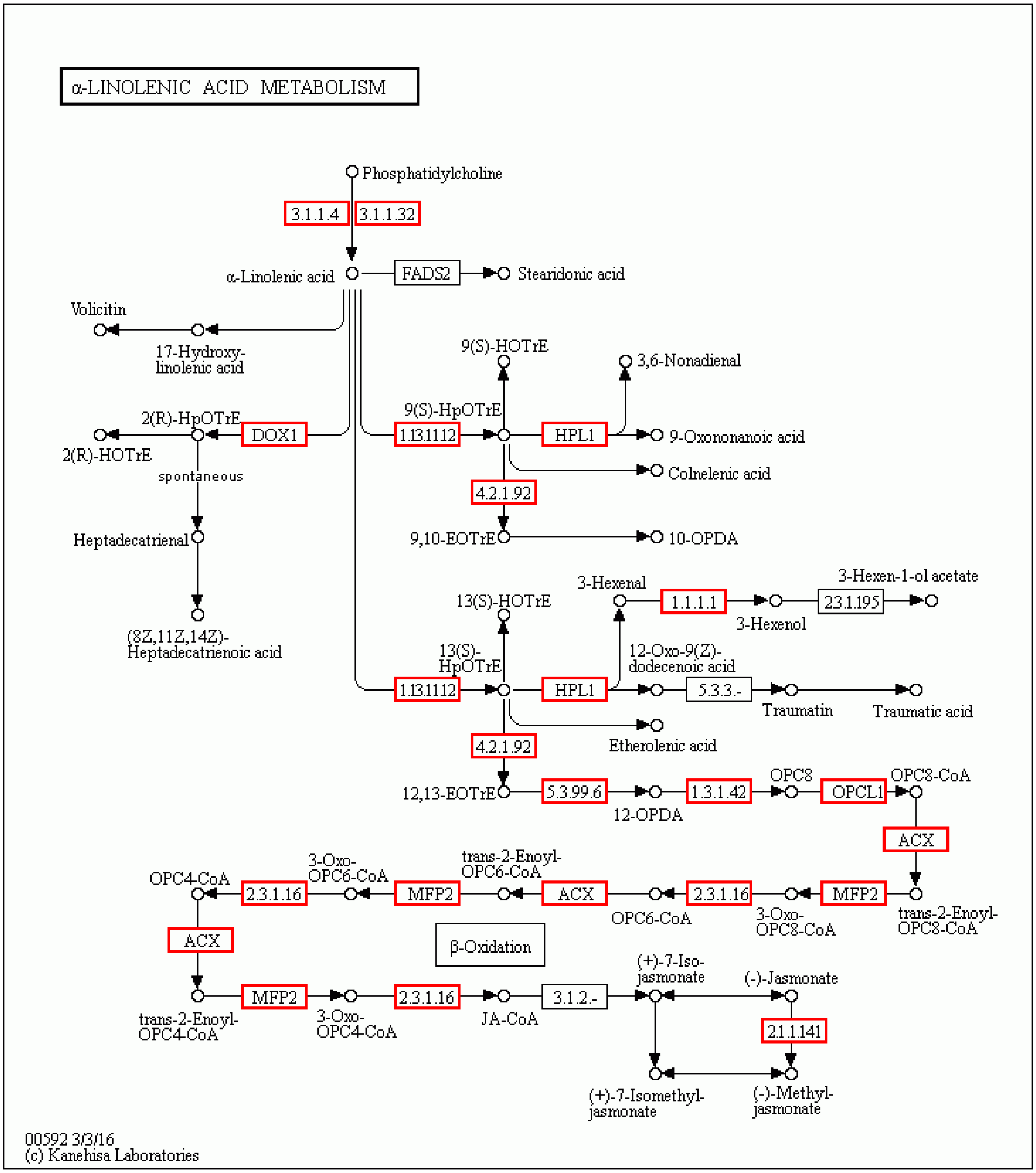
α-LINOLENIC ACID METABOLISM
Phosphatidylcholine
3.1.1.4
3.1.1.32
α-Linolenic acid
FADS2
Stearidonic acid
Volicitin
17-Hydroxy-linolenic acid
9(S)-HOTrE
3,6-Nonadienal
2(R)-HpOTrE
DOX1
2(R)-HOTrE
9(S)-HpOTrE
1.13.11.12
HPL1
9-Oxononanoic acid
Colnelenic acid
spontaneous
4.2.1.92
Heptadecatrienal
9,10-EOTrE
10-OPDA
3-Hexenal
1.1.1.1
3-Hexen-1-ol acetate
2.3.1.195
3-Hexenol
13(S)-HOTrE
(8Z,11Z,14Z)-Heptadecatrienoic acid
13(S)-HpOTrE
12-Oxo-9(Z)-dodecenoic acid
1.13.11.12
HPL1
5.3.3.-
Traumatin
Traumatic acid
Etherolenic acid
4.2.1.92
12,13-EOTrE
5.3.99.6
12-OPDA
1.3.1.42
OPC8
OPCL1
OPC8-CoA
ACX
OPC4-CoA
2.3.1.16
3-Oxo-OPC6-CoA
MFP2
trans-2-Enoyl-OPC6-CoA
ACX
OPC6-CoA
2.3.1.16
3-Oxo-OPC8-CoA
MFP2
trans-2-Enoyl-OPC8-CoA
ACX
β-Oxidation
(+)-7-Iso-jasmonate
(-)-Jasmonate
MFP2
2.3.1.16
3.1.2.-
trans-2-Enoyl-OPC4-CoA
3-Oxo-OPC4-CoA
JA-CoA
2.1.1.141
(+)-7-Isomethyl-jasmonate
(-)-Methyl-jasmonate
00592 3/3/16
(c) Kanehisa Laboratories

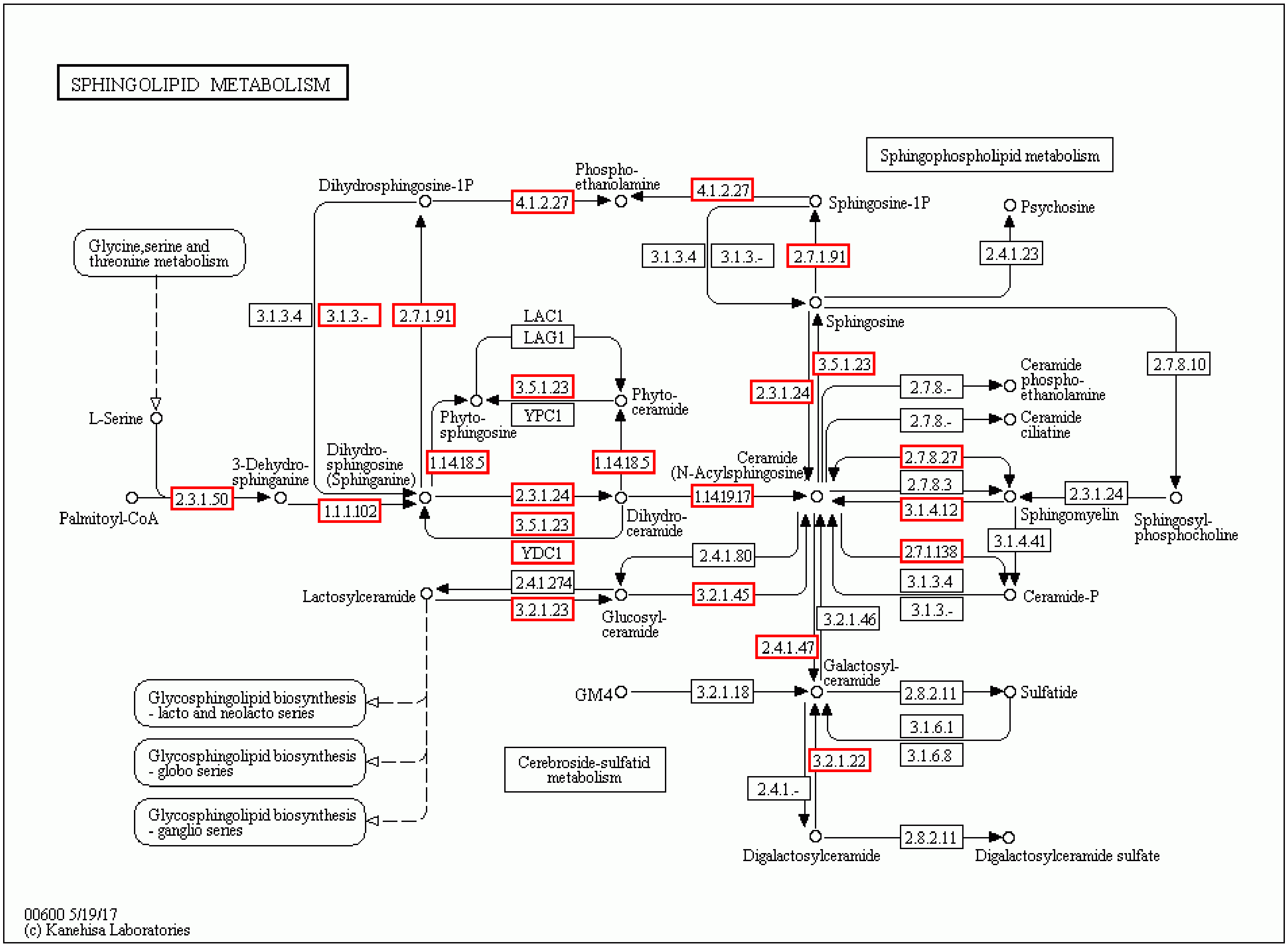

SPHINGOLIPID METABOLISM
Sphingophospholipid metabolism
Glycine,serine and threonine metabolism
Dihydrosphingosine-1P
Phospho-ethanolamine
4.1.2.27
4.1.2.27
Sphingosine-1P
Psychosine
3.1.3.4
3.1.3.-
2.7.1.91
2.4.1.23
3.1.3.4
3.1.3.-
2.7.1.91
LAC1
LAG1
Sphingosine
3.5.1.23
2.3.1.24
2.7.8.10
3.5.1.23
YPC1
Phyto-ceramide
Phyto-sphingosine
2.7.8.-
Ceramide phospho-ethanolamine
2.7.8.-
Ceramide ciliatine
L-Serine
3-Dehydro-sphinganine
Dihydro-sphingosine (Sphinganine)
1.14.18.5
1.14.18.5
Ceramide (N-Acylsphingosine)
2.7.8.27
2.7.8.3
3.1.4.12
Palmitoyl-CoA
2.3.1.50
1.1.1.102
2.3.1.24
3.5.1.23
YDC1
Dihydro-ceramide
1.14.19.17
2.3.1.24
Sphingomyelin
Sphingosyl-phosphocholine
3.1.4.41
2.7.1.138
2.4.1.80
Lactosylceramide
2.4.1.274
3.2.1.23
3.2.1.45
Glucosyl-ceramide
3.1.3.4
3.1.3.-
Ceramide-P
3.2.1.46
2.4.1.47
Galactosyl-ceramide
GM4
3.2.1.18
2.8.2.11
Sulfatide
3.1.6.1
3.1.6.8
3.2.1.22
Glycosphingolipid biosynthesis - lacto and neolacto series
Glycosphingolipid biosynthesis - globo series
Glycosphingolipid biosynthesis - ganglio series
Cerebroside-sulfatid metabolism
2.4.1.-
Digalactosylceramide
2.8.2.11
Digalactosylceramide sulfate
00600 5/19/17
(c) Kanehisa Laboratories

GLYCOSPHINGOLIPID BIOSYNTHESIS - LACTO AND NEOLACTO SERIES

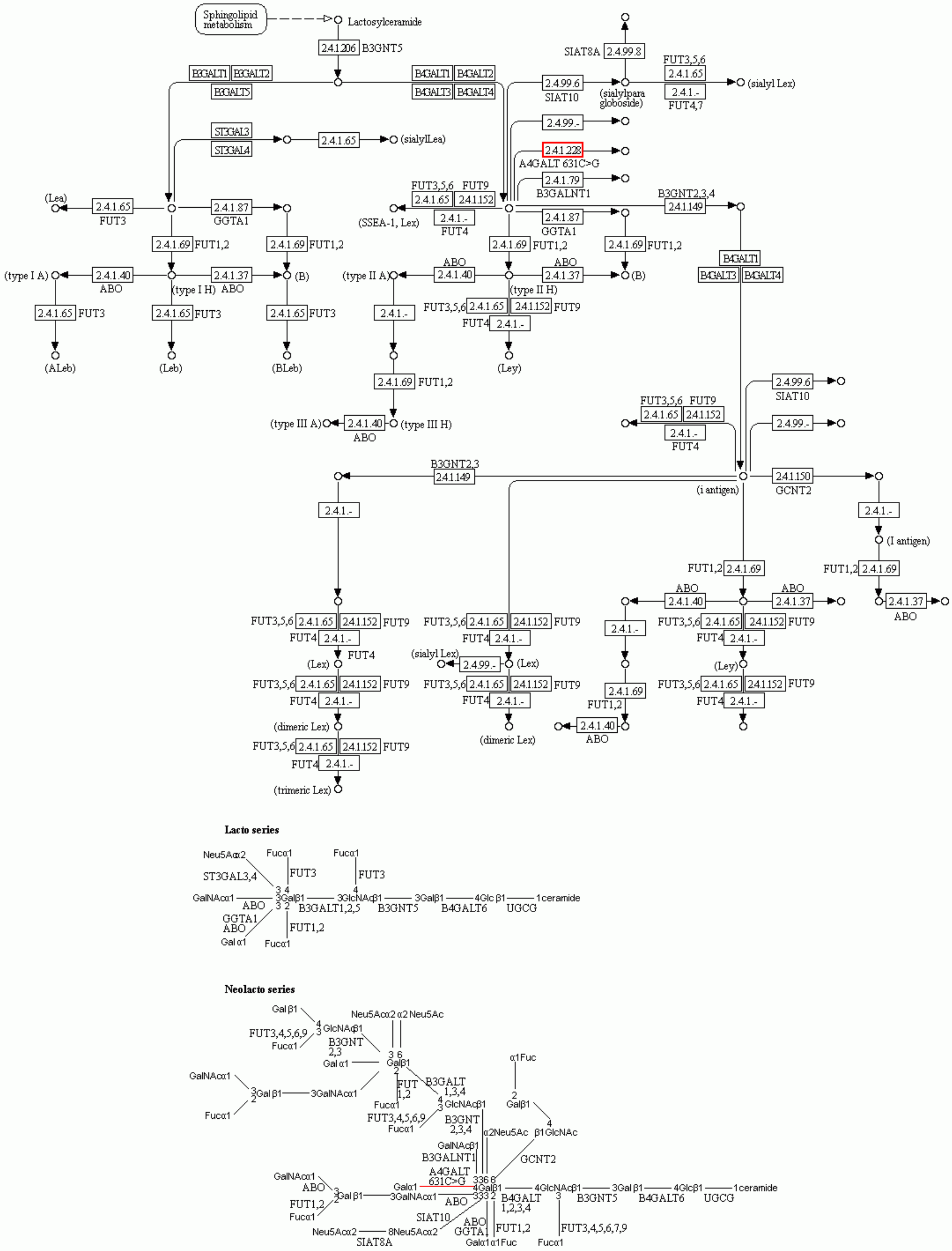

00601 4/11/17
(c) Kanehisa Laboratories

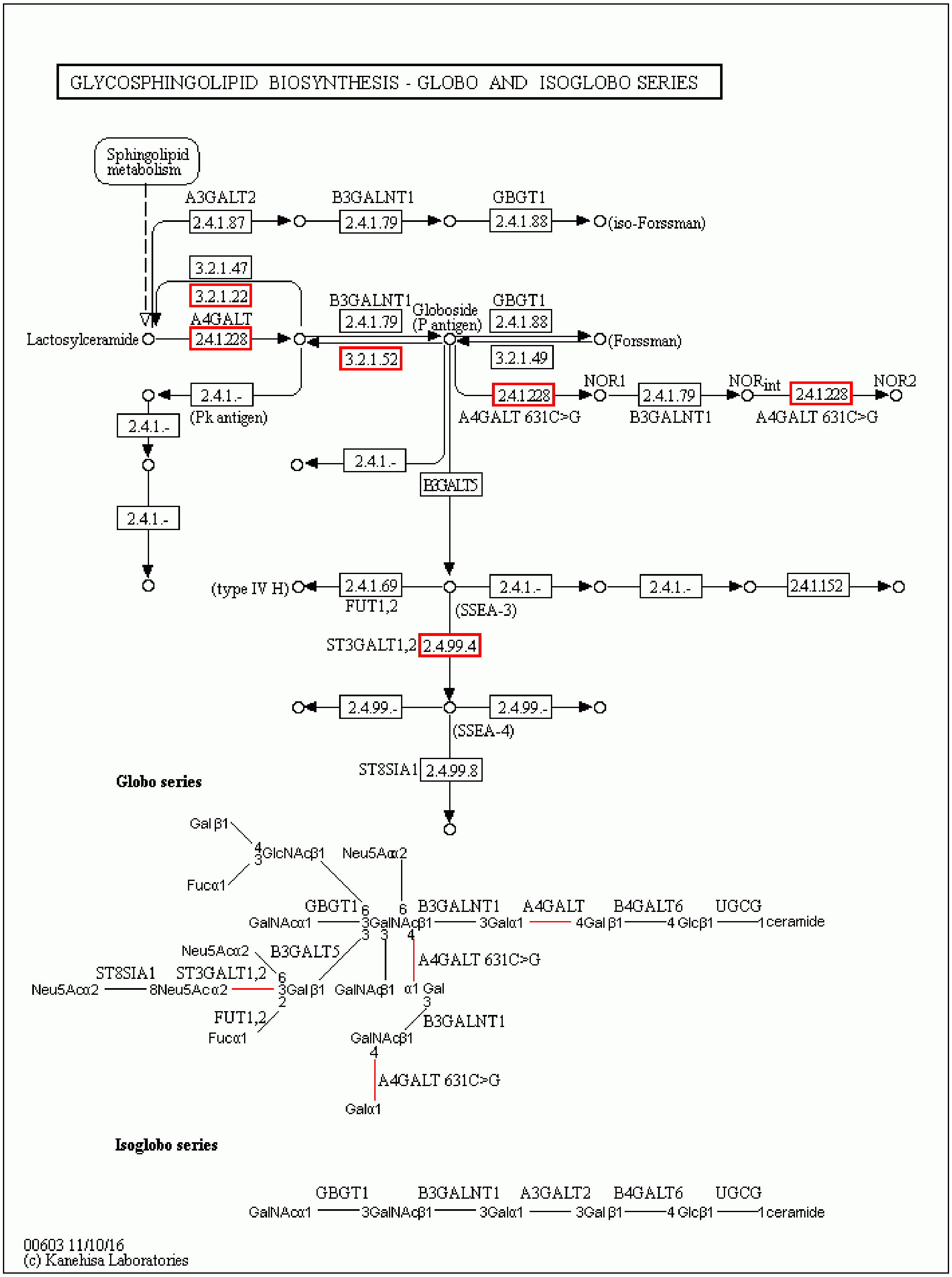
GLYCOSPHINGOLIPID BIOSYNTHESIS - GLOBO AND ISOGLOBO SERIES
Sphingolipid metabolism
A3GALT2
2.4.1.87
B3GALNT1
2.4.1.79
GBGT1
2.4.1.88
(iso-Forssman)
3.2.1.47
3.2.1.22
A4GALT
Lactosylceramide
2.4.1.228
B3GALNT1
2.4.1.79
Globoside
(P antigen)
GBGT1
2.4.1.88
(Forssman)
3.2.1.52
3.2.1.49
NOR1
NORint
NOR2
2.4.1.228
2.4.1.79
2.4.1.228
A4GALT 631C>G
B3GALNT1
A4GALT 631C>G
2.4.1.-
(Pk antigen)
2.4.1.-
2.4.1.-
B3GALT5
2.4.1.-
(type IV H)
2.4.1.69
FUT1,2
2.4.1.-
2.4.1.-
2.4.1.152
(SSEA-3)
ST3GALT1,2
2.4.99.4
2.4.99.-
2.4.99.-
(SSEA-4)
ST8SIA1
2.4.99.8
Globo series
Galβ1
4
3
GlcNAcβ1
Neu5Acα2
Fucα1
GBGT1
6
6
B3GALNT1
A4GALT
B4GALT6
UGCG
GalNAcα1
3GalNAcβ1
3Galα1
4Galβ1
4 Glcβ1
1ceramide
3
3
4
Neu5Acα2
B3GALT5
A4GALT 631C>G
ST8SIA1
ST3GALT1,2
6
Neu5Acα2
8Neu5Acα2
3Galβ1
GalNAcβ1
α1 Gal
2
3
FUT1,2
B3GALNT1
Fucα1
GalNAcβ1
4
A4GALT 631C>G
Galα1
Isoglobo series
GBGT1
B3GALNT1
A3GALT2
B4GALT6
UGCG
GalNAcα1
3GalNAcβ1
3Galα1
3Galβ1
4 Glcβ1
1ceramide
00603 11/10/16
(c) Kanehisa Laboratories

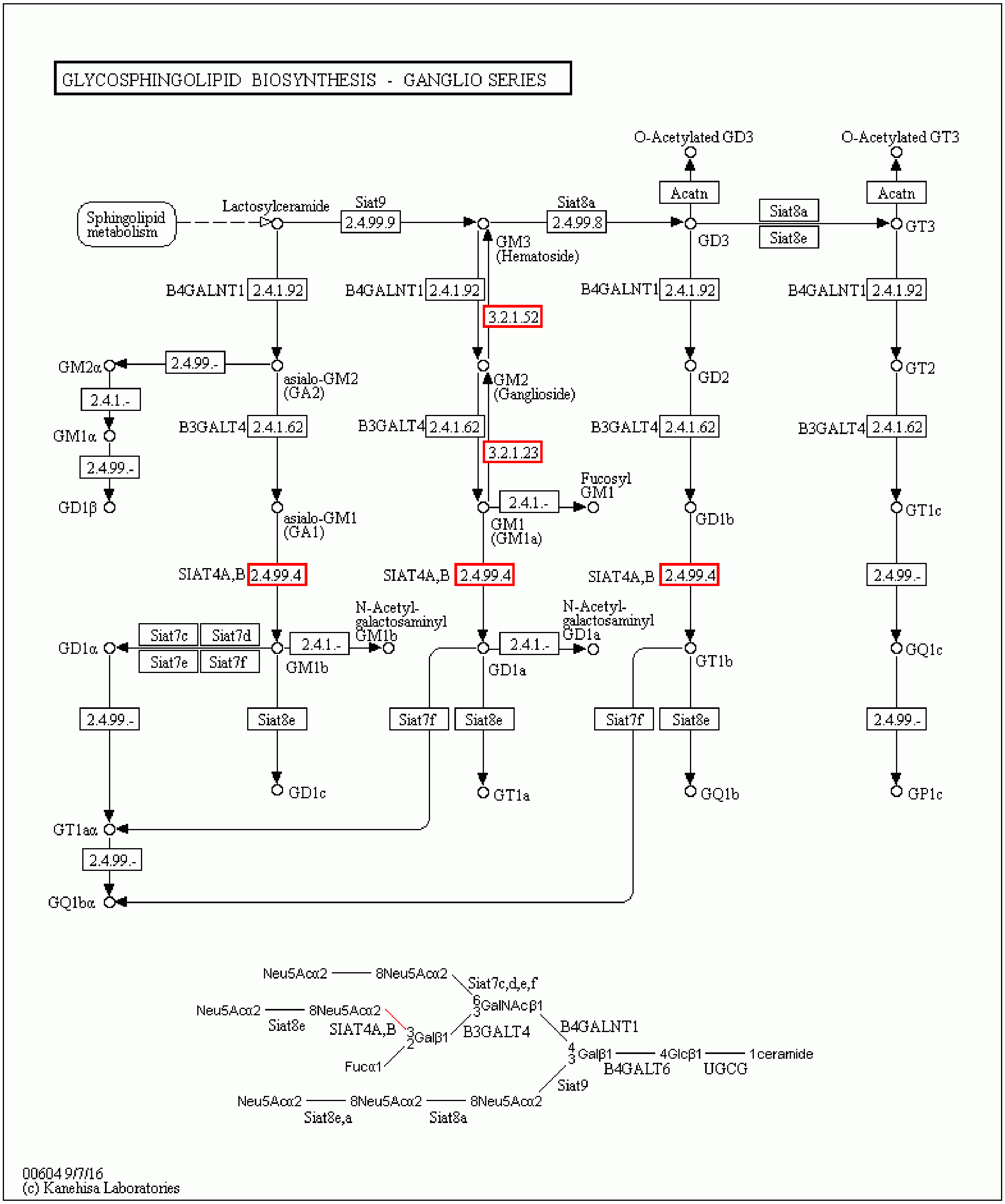
GLYCOSPHINGOLIPID BIOSYNTHESIS - GANGLIO SERIES
O-Acetylated GD3
O-Acetylated GT3
Acatn
Acatn
Sphingolipid metabolism
Lactosylceramide
Siat9
2.4.99.9
Siat8a
2.4.99.8
Siat8a
Siat8e
GM3 (Hematoside)
GD3
GT3
B4GALNT1 2.4.1.92
B4GALNT1 2.4.1.92
B4GALNT1 2.4.1.92
B4GALNT1 2.4.1.92
3.2.1.52
GM2α
2.4.99.-
asialo-GM2 (GA2)
GM2 (Ganglioside)
GD2
GT2
2.4.1.-
GM1α
B3GALT4 2.4.1.62
B3GALT4 2.4.1.62
B3GALT4 2.4.1.62
B3GALT4 2.4.1.62
3.2.1.23
2.4.99.-
Fucosyl GM1
2.4.1.-
GD1β
asialo-GM1 (GA1)
GM1 (GM1a)
GD1b
GT1c
SIAT4A,B 2.4.99.4
SIAT4A,B 2.4.99.4
SIAT4A,B 2.4.99.4
2.4.99.-
N-Acetyl-galactosaminyl GM1b
N-Acetyl-galactosaminyl GD1a
Siat7c
Siat7d
Siat7e
Siat7f
GD1α
2.4.1.-
2.4.1.-
GM1b
GD1a
GT1b
GQ1c
2.4.99.-
Siat8e
Siat7f
Siat8e
Siat7f
Siat8e
2.4.99.-
GD1c
GT1a
GQ1b
GP1c
GT1aα
2.4.99.-
GQ1bα
Neu5Acα2 — 8Neu5Acα2
Siat7c,d,e,f
6 3 GalNAcβ1
Neu5Acα2 — 8Neu5Acα2
Siat8e
SIAT4A,B
3 2 Galβ1
B3GALT4
B4GALNT1
Fucα1
4 3 Galβ1 — 4Glcβ1 — 1ceramide
B4GALT6
UGCG
Siat9
Neu5Acα2 — 8Neu5Acα2 — 8Neu5Acα2
Siat8e,a
Siat8a
00604 9/7/16
(c) Kanehisa Laboratories

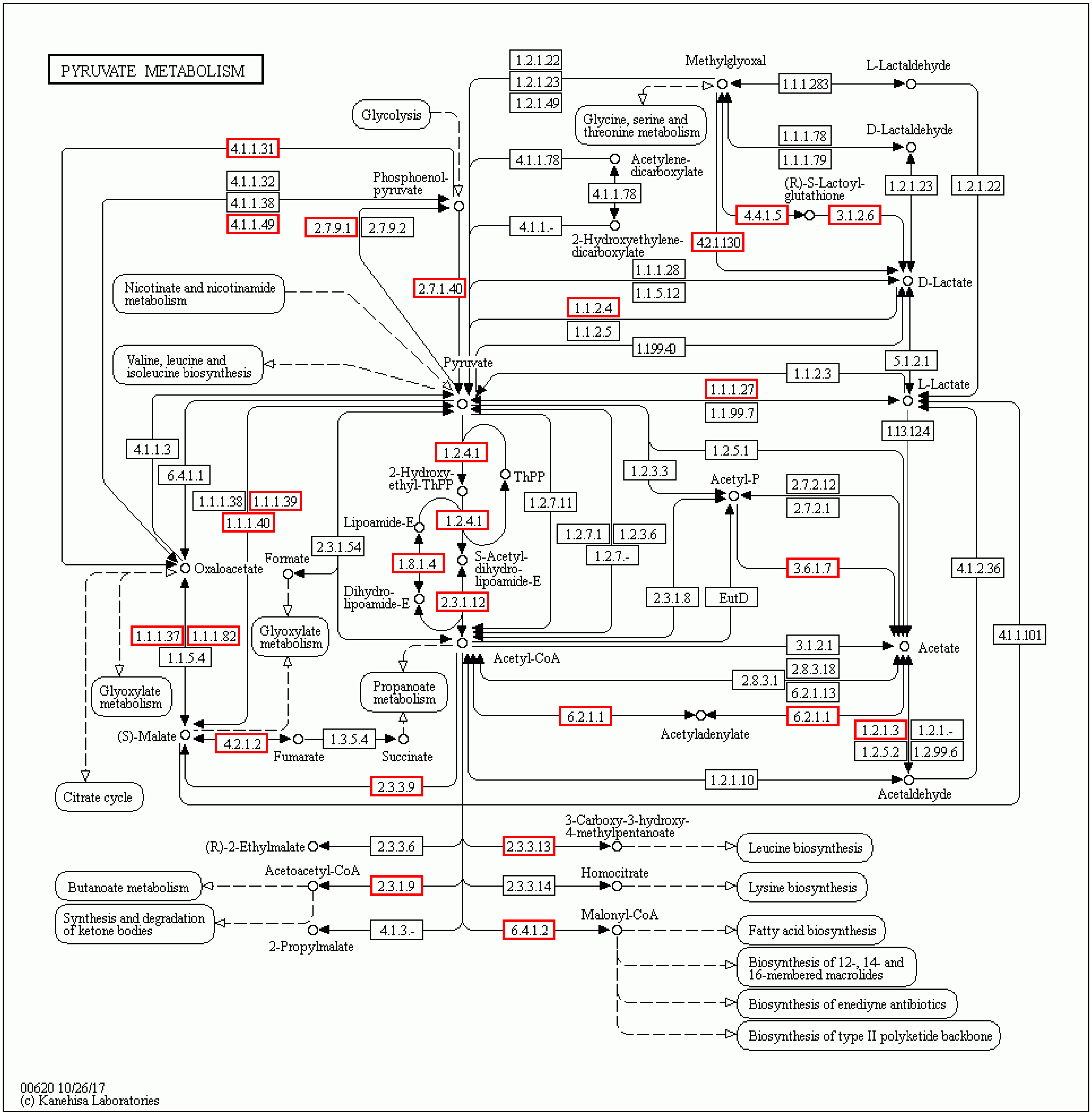

PYRUVATE METABOLISM
Glycolysis
Phosphoenol-pyruvate
Pyruvate
Methylglyoxal
L-Lactaldehyde
D-Lactaldehyde
(R)-S-Lactoyl-glutathione
D-Lactate
L-Lactate
Acetylene-dicarboxylate
2-Hydroxyethylene-dicarboxylate
Glycine, serine and threonine metabolism
Nicotinate and nicotinamide metabolism
Valine, leucine and isoleucine biosynthesis
Oxaloacetate
Formate
Glyoxylate metabolism
Glyoxylate metabolism
(S)-Malate
Fumarate
Succinate
Citrate cycle
Propanoate metabolism
2-Hydroxy-ethyl-ThPP
ThPP
Lipoamide-E
S-Acetyl-dihydro-lipoamide-E
Dihydro-lipoamide-E
Acetyl-CoA
Acetyl-P
Acetate
Acetyladenylate
Acetaldehyde
(R)-2-Ethylmalate
3-Carboxy-3-hydroxy-4-methylpentanoate
Leucine biosynthesis
Acetoacetyl-CoA
Homocitrate
Lysine biosynthesis
Butanoate metabolism
Synthesis and degradation of ketone bodies
2-Propylmalate
Malonyl-CoA
Fatty acid biosynthesis
Biosynthesis of 12-, 14- and 16-membered macrolides
Biosynthesis of enediyne antibiotics
Biosynthesis of type II polyketide backbone
1.2.1.22
1.2.1.23
1.2.1.49
1.1.1.283
1.1.1.78
1.1.1.79
4.1.1.31
4.1.1.32
4.1.1.38
4.1.1.49
2.7.9.1
2.7.9.2
4.1.1.78
4.1.1.78
4.1.1.-
4.4.1.5
3.1.2.6
1.2.1.23
1.2.1.22
4.2.1.130
1.1.1.28
1.1.5.12
2.7.1.40
1.1.2.4
1.1.2.5
1.1.99.40
5.1.2.1
1.1.2.3
1.1.1.27
1.1.99.7
1.13.12.4
4.1.1.3
6.4.1.1
1.2.4.1
1.2.5.1
1.2.3.3
2.7.2.12
2.7.2.1
1.1.1.38
1.1.1.39
1.1.1.40
1.2.7.11
1.2.4.1
2.3.1.54
1.2.7.1
1.2.3.6
1.2.7.-
1.8.1.4
3.6.1.7
4.1.2.36
2.3.1.12
2.3.1.8
EutD
1.1.1.37
1.1.1.82
1.1.5.4
3.1.2.1
4.1.1.101
2.8.3.18
2.8.3.1
6.2.1.13
6.2.1.1
6.2.1.1
4.2.1.2
1.3.5.4
1.2.1.3
1.2.1.-
1.2.5.2
1.2.99.6
2.3.3.9
1.2.1.10
2.3.3.6
2.3.3.13
2.3.1.9
2.3.3.14
4.1.3.-
6.4.1.2
00620 10/26/17
(c) Kanehisa Laboratories

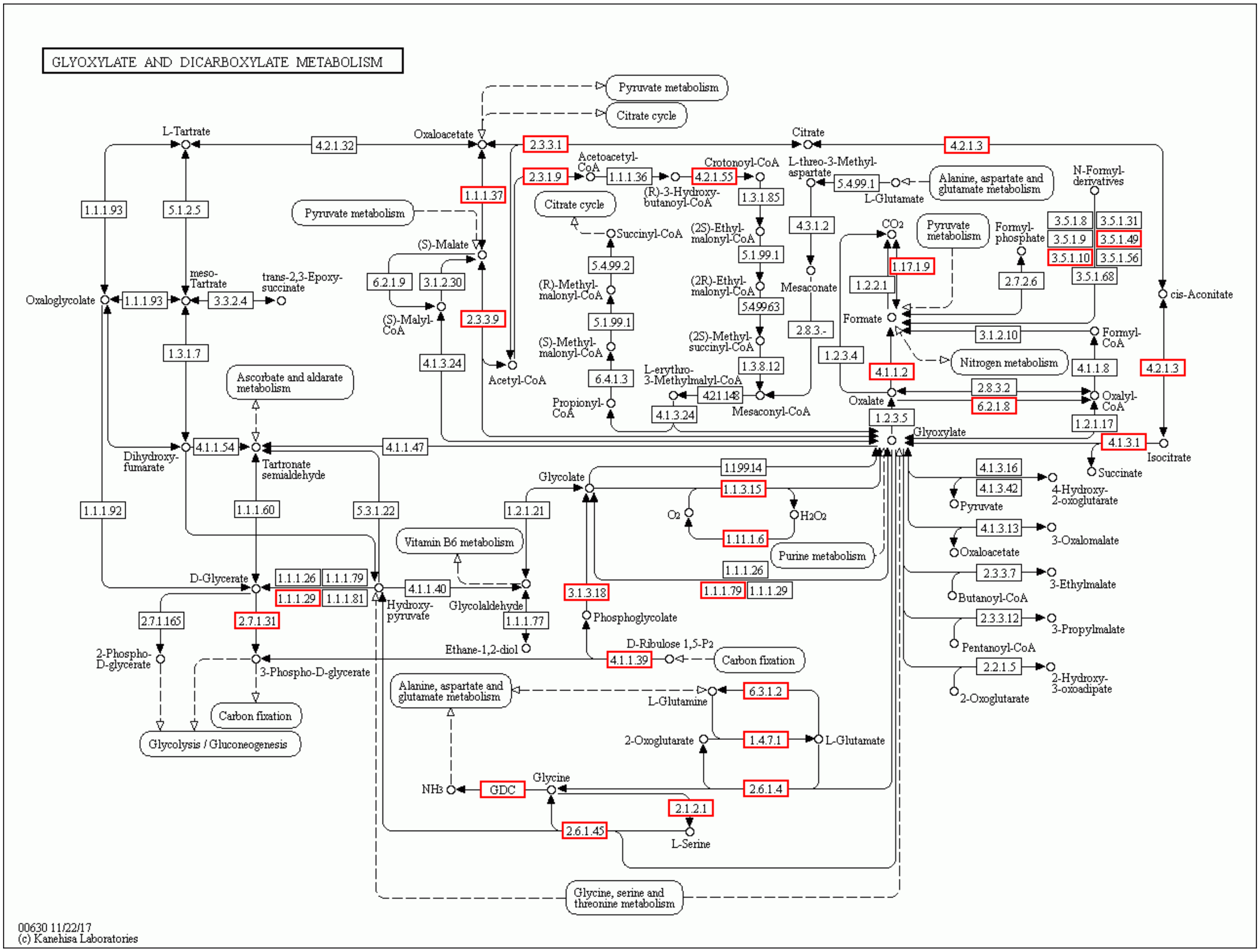
GLYOXYLATE AND DICARBOXYLATE METABOLISM
Pyruvate metabolism
Citrate cycle
L-Tartrate
4.2.1.32
Oxaloacetate
2.3.3.1
Citrate
4.2.1.3
Acetoacetyl-CoA
Crotonoyl-CoA
L-threo-3-Methyl-aspartate
2.3.1.9
1.1.1.36
4.2.1.55
5.4.99.1
Alanine, aspartate and glutamate metabolism
N-Formyl-derivatives
1.1.1.93
5.1.2.5
1.1.1.37
Pyruvate metabolism
Citrate cycle
(R)-3-Hydroxy-butanoyl-CoA
1.3.1.85
L-Glutamate
(S)-Malate
Succinyl-CoA
(2S)-Ethyl-malonyl-CoA
4.3.1.2
CO2
Pyruvate metabolism
Formyl-phosphate
3.5.1.8
3.5.1.31
3.5.1.9
3.5.1.49
3.5.1.10
3.5.1.56
3.5.1.68
meso-Tartrate
trans-2,3-Epoxy-succinate
6.2.1.9
3.1.2.30
5.4.99.2
5.1.99.1
1.17.1.9
Oxaloglycolate
1.1.1.93
3.3.2.4
(R)-Methyl-malonyl-CoA
(2R)-Ethyl-malonyl-CoA
Mesaconate
1.2.2.1
2.7.2.6
cis-Aconitate
(S)-Malyl-CoA
2.3.3.9
5.1.99.1
5.4.99.63
Formate
1.3.1.7
(S)-Methyl-malonyl-CoA
(2S)-Methyl-succinyl-CoA
2.8.3.-
3.1.2.10
Formyl-CoA
4.1.3.24
Acetyl-CoA
1.2.3.4
Nitrogen metabolism
Ascorbate and aldarate metabolism
6.4.1.3
L-erythro-3-Methylmalyl-CoA
1.3.8.12
4.1.1.2
4.1.1.8
4.2.1.3
Propionyl-CoA
4.2.1.148
Mesaconyl-CoA
4.1.3.24
2.8.3.2
Oxalate
6.2.1.8
Oxalyl-CoA
1.2.3.5
1.2.1.17
Glyoxylate
4.1.3.1
Isocitrate
4.1.1.54
4.1.1.47
Dihydroxy-fumarate
Tartronate semialdehyde
1.1.99.14
Succinate
4.1.3.16
4-Hydroxy-2-oxoglutarate
Glycolate
1.1.3.15
4.1.3.42
Pyruvate
1.1.1.92
1.1.1.60
5.3.1.22
1.2.1.21
O2
H2O2
4.1.3.13
3-Oxalomalate
Vitamin B6 metabolism
1.1.1.6
Oxaloacetate
Purine metabolism
1.1.1.26
2.3.3.7
3-Ethylmalate
D-Glycerate
1.1.1.26
1.1.1.79
4.1.1.40
1.1.1.79
1.1.1.29
Butanoyl-CoA
1.1.1.29
1.1.1.81
3.1.3.18
Hydroxy-pyruvate
Glycolaldehyde
2.7.1.165
2.7.1.31
1.1.1.77
Phosphoglycolate
2.3.3.12
3-Propylmalate
Pentanoyl-CoA
2-Phospho-D-glycerate
Ethane-1,2-diol
D-Ribulose 1,5-P2
3-Phospho-D-glycerate
4.1.1.39
Carbon fixation
2.2.1.5
2-Hydroxy-3-oxoadipate
2-Oxoglutarate
Alanine, aspartate and glutamate metabolism
6.3.1.2
L-Glutamine
Carbon fixation
Glycolysis / Gluconeogenesis
2-Oxoglutarate
1.4.7.1
L-Glutamate
Glycine
NH3
GDC
2.6.1.4
2.1.2.1
2.6.1.45
L-Serine
Glycine, serine and threonine metabolism
00630 11/22/17
(c) Kanehisa Laboratories

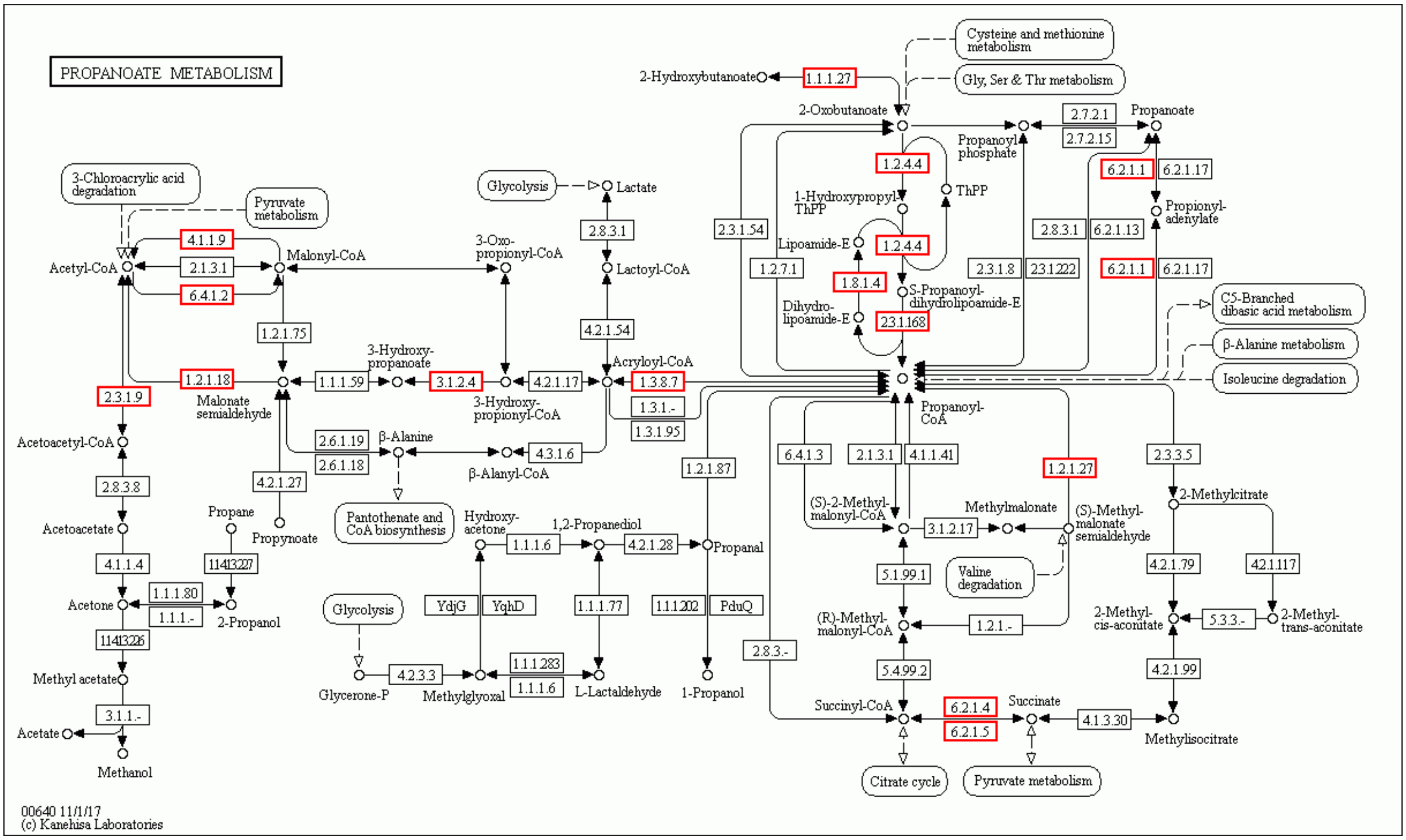

PROPANOATE METABOLISM
Cysteine and methionine metabolism
Gly, Ser & Thr metabolism
2-Hydroxybutanoate
1.1.1.27
2-Oxobutanoate
Propanoyl phosphate
2.7.2.1
2.7.2.15
Propanoate
1.2.4.4
ThPP
6.2.1.1
6.2.1.17
1-Hydroxypropyl-ThPP
Propionyl-adenylate
Lipoamide-E
2.3.1.54
1.2.7.1
1.8.1.4
2.3.1.8
2.3.1.222
2.8.3.1
6.2.1.13
S-Propanoyl-dihydrolipoamide-E
Dihydro-lipoamide-E
2.3.1.168
C5-Branched dibasic acid metabolism
β-Alanine metabolism
Isoleucine degradation
3-Chloroacrylic acid degradation
Pyruvate metabolism
Glycolysis
Lactate
Acetyl-CoA
4.1.1.9
2.1.3.1
6.4.1.2
Malonyl-CoA
3-Oxo-propionyl-CoA
Lactoyl-CoA
1.2.1.75
4.2.1.54
3-Hydroxy-propanoate
Acryloyl-CoA
1.2.1.18
1.1.1.59
3.1.2.4
4.2.1.17
1.3.8.7
2.3.1.9
Malonate semialdehyde
3-Hydroxy-propionyl-CoA
1.3.1.-
1.3.1.95
Propanoyl-CoA
2.6.1.19
2.6.1.18
β-Alanine
4.3.1.6
β-Alanyl-CoA
Acetoacetyl-CoA
2.8.3.8
4.2.1.27
Propane
Propynoate
Pantothenate and CoA biosynthesis
Hydroxy-acetone
1,2-Propanediol
1.1.1.6
4.2.1.28
Propanal
1.2.1.87
6.4.1.3
2.1.3.1
4.1.1.41
1.2.1.27
2.3.3.5
2-Methylcitrate
(S)-2-Methyl-malonyl-CoA
Methylmalonate
3.1.2.17
(S)-Methyl-malonate semialdehyde
Acetoacetate
4.1.1.4
1.14.13.227
Acetone
1.1.1.80
1.1.1.-
2-Propanol
Valine degradation
5.1.99.1
4.2.1.79
4.2.1.117
Glycolysis
YdjG
YqhD
1.1.1.77
1.1.1.202
PduQ
(R)-Methyl-malonyl-CoA
1.2.1.-
2-Methyl-cis-aconitate
5.3.3.-
2-Methyl-trans-aconitate
1.14.13.226
1.1.1.283
2.8.3.-
Methyl acetate
4.2.3.3
1.1.1.6
5.4.99.2
4.2.1.99
Glycerone-P
Methylglyoxal
L-Lactaldehyde
1-Propanol
Succinyl-CoA
6.2.1.4
6.2.1.5
Succinate
4.1.3.30
Methylisocitrate
3.1.1.-
Acetate
Methanol
Citrate cycle
Pyruvate metabolism
00640 11/1/17
(c) Kanehisa Laboratories

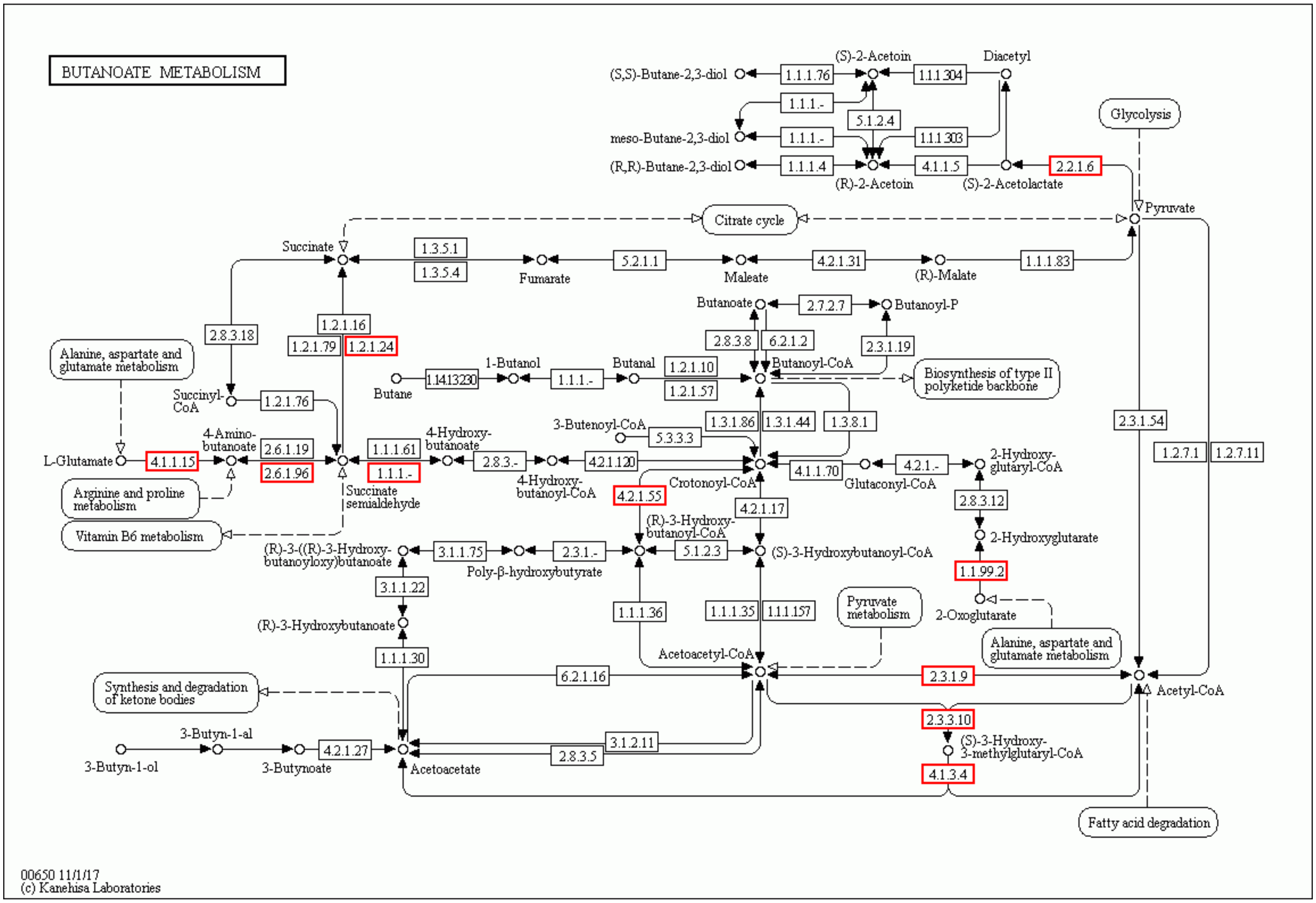

BUTANOATE METABOLISM
(S,S)-Butane-2,3-diol
1.1.1.76
(S)-2-Acetoin
1.1.1.304
Diacetyl
1.1.1.-
5.1.2.4
meso-Butane-2,3-diol
1.1.1.303
Glycolysis
(R,R)-Butane-2,3-diol
1.1.1.4
4.1.1.5
2.2.1.6
(R)-2-Acetoin
(S)-2-Acetolactate
Pyruvate
Citrate cycle
Succinate
1.3.5.1
1.3.5.4
5.2.1.1
4.2.1.31
1.1.1.83
Fumarate
Maleate
(R)-Malate
Butanoate
2.7.2.7
Butanoyl-P
2.8.3.18
1.2.1.16
1.2.1.79
1.2.1.24
2.8.3.8
6.2.1.2
2.3.1.19
Alanine, aspartate and glutamate metabolism
1-Butanol
Butanal
1.2.1.10
Butanoyl-CoA
Biosynthesis of type II polyketide backbone
Butane
1.14.13.230
1.1.1.-
1.2.1.57
Succinyl-CoA
1.2.1.76
3-Butenoyl-CoA
5.3.3.3
1.3.1.86
1.3.1.44
1.3.8.1
2.3.1.54
1.2.7.1
1.2.7.11
4-Amino-butanoate
2.6.1.19
1.1.1.61
4-Hydroxy-butanoate
L-Glutamate
4.1.1.15
2.6.1.96
1.1.1.-
2.8.3.-
4.2.1.120
4.1.1.70
Glutaconyl-CoA
4.2.1.-
2-Hydroxy-glutaryl-CoA
Arginine and proline metabolism
Succinate semialdehyde
4-Hydroxy-butanoyl-CoA
4.2.1.55
Crotonoyl-CoA
4.2.1.17
2.8.3.12
Vitamin B6 metabolism
(R)-3-Hydroxy-butanoyl-CoA
(R)-3-((R)-3-Hydroxy-butanoyloxy)butanoate
3.1.1.75
2.3.1.-
5.1.2.3
(S)-3-Hydroxybutanoyl-CoA
2-Hydroxyglutarate
Poly-β-hydroxybutyrate
3.1.1.22
1.1.1.36
1.1.1.35
1.1.1.157
Pyruvate metabolism
1.1.99.2
2-Oxoglutarate
(R)-3-Hydroxybutanoate
Alanine, aspartate and glutamate metabolism
1.1.1.30
Acetoacetyl-CoA
Synthesis and degradation of ketone bodies
6.2.1.16
2.3.1.9
Acetyl-CoA
2.3.3.10
(S)-3-Hydroxy-3-methylglutaryl-CoA
3-Butyn-1-al
4.2.1.27
3.1.2.11
2.8.3.5
3-Butyn-1-ol
3-Butynoate
Acetoacetate
4.1.3.4
Fatty acid degradation
00650 11/1/17
(c) Kanehisa Laboratories

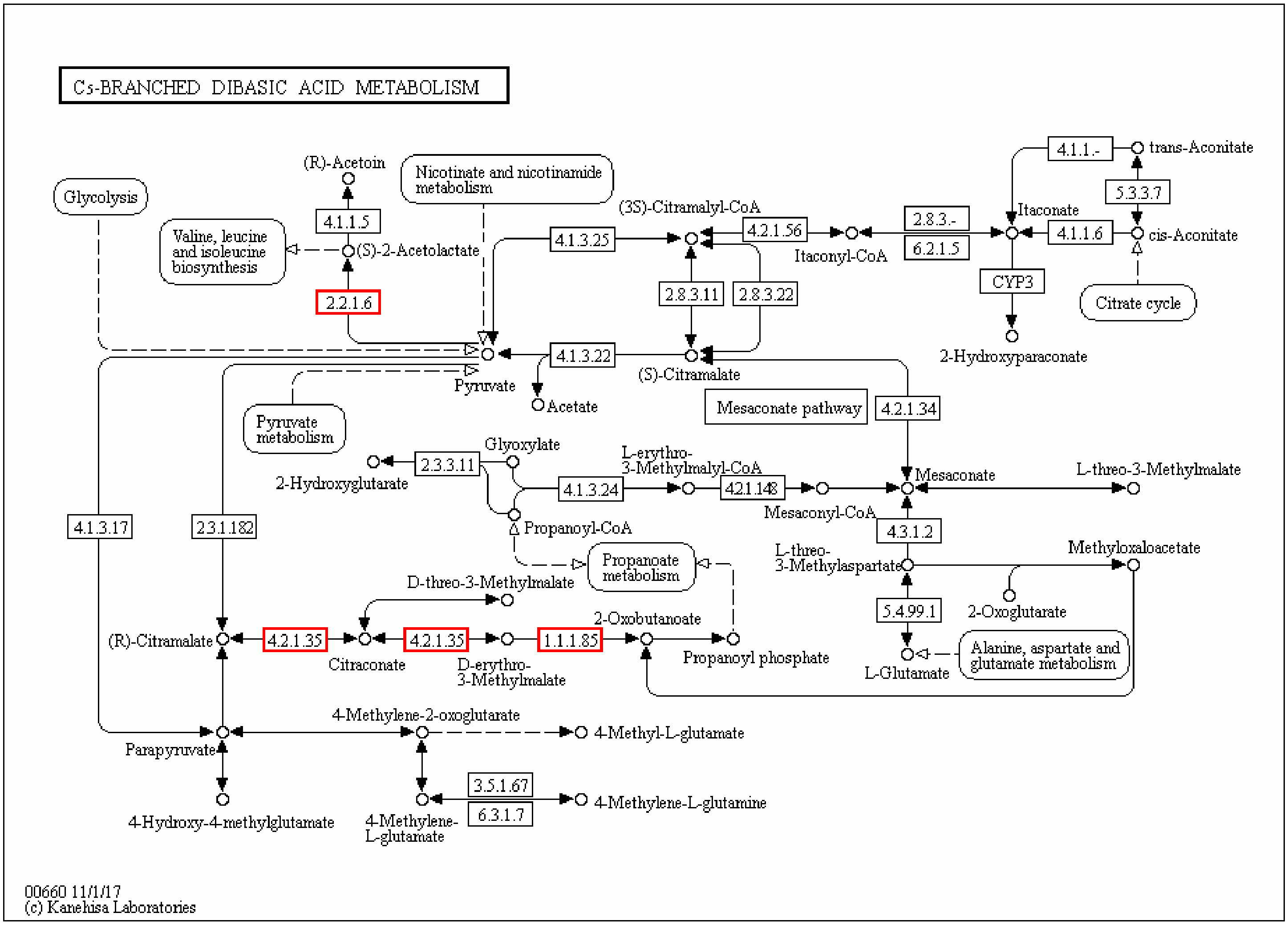
C5-BRANCHED DIBASIC ACID METABOLISM
Glycolysis
(R)-Acetoin
4.1.1.5
Nicotinate and nicotinamide metabolism
Valine, leucine and isoleucine biosynthesis
(S)-2-Acetolactate
2.2.1.6
(3S)-Citramalyl-CoA
4.1.3.25
4.2.1.56
Itaconyl-CoA
2.8.3.-
6.2.1.5
4.1.1.-
trans-Aconitate
5.3.3.7
Itaconate
4.1.1.6
cis-Aconitate
CYP3
Citrate cycle
2-Hydroxyparaconate
2.8.3.11
2.8.3.22
4.1.3.22
Pyruvate
Acetate
(S)-Citramalate
Mesaconate pathway
4.2.1.34
Pyruvate metabolism
Glyoxylate
2.3.3.11
2-Hydroxyglutarate
L-erythro-3-Methylmalyl-CoA
4.1.3.24
4.2.1.148
Mesaconate
L-threo-3-Methylmalate
Mesaconyl-CoA
Propanoyl-CoA
4.3.1.2
4.1.3.17
2.3.1.182
Propanoate metabolism
L-threo-3-Methylaspartate
Methyloxaloacetate
D-threo-3-Methylmalate
2-Oxobutanoate
5.4.99.1
2-Oxoglutarate
(R)-Citramalate
4.2.1.35
Citraconate
4.2.1.35
D-erythro-3-Methylmalate
1.1.1.85
Propanoyl phosphate
L-Glutamate
Alanine, aspartate and glutamate metabolism
4-Methylene-2-oxoglutarate
Parapyruvate
4-Methyl-L-glutamate
3.5.1.67
6.3.1.7
4-Methylene-L-glutamine
4-Hydroxy-4-methylglutamate
4-Methylene-L-glutamate
00660 11/1/17
(c) Kanehisa Laboratories

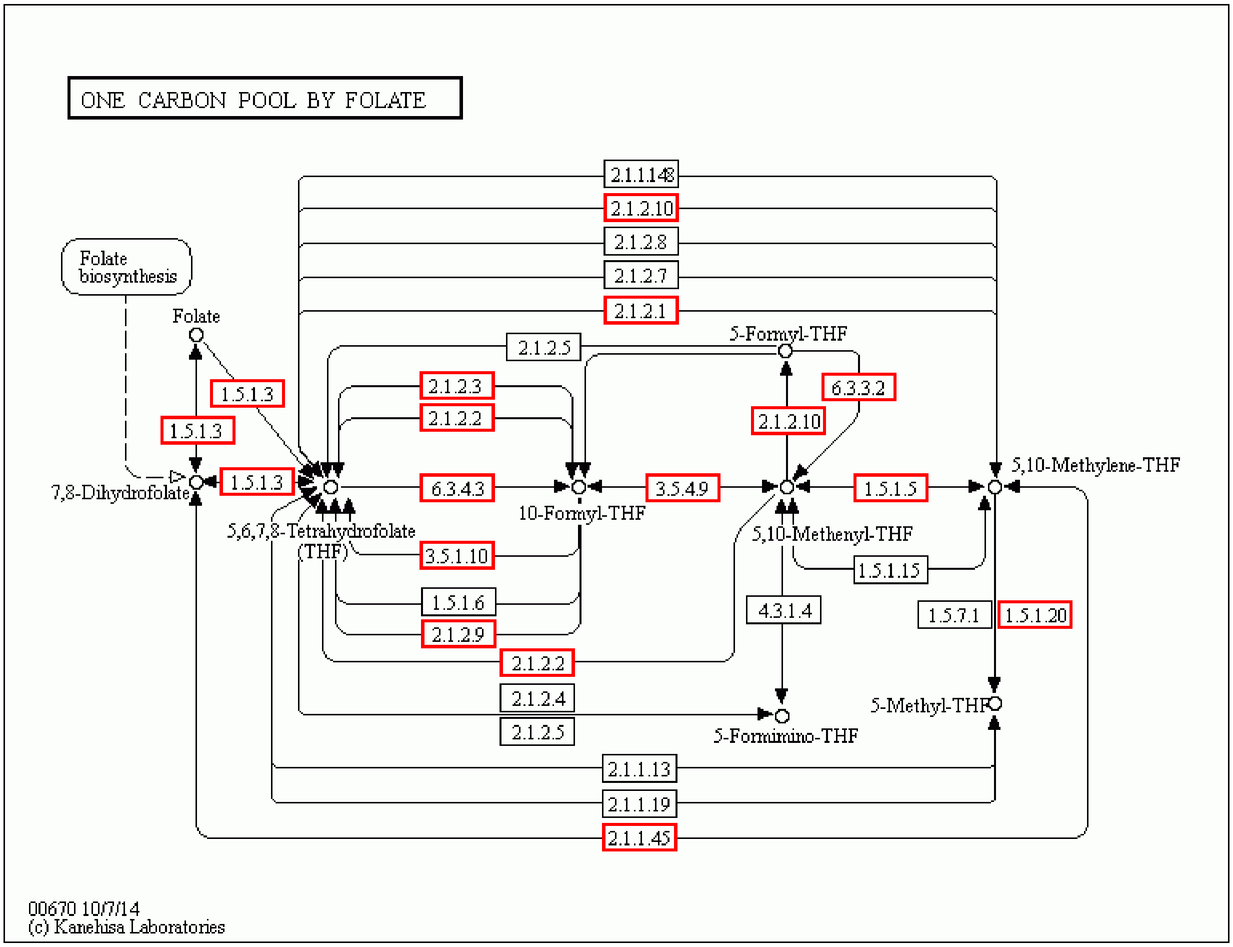
ONE CARBON POOL BY FOLATE
Folate biosynthesis
Folate
7,8-Dihydrofolate
5,6,7,8-Tetrahydrofolate (THF)
10-Formyl-THF
5-Formyl-THF
5,10-Methenyl-THF
5,10-Methylene-THF
5-Formimino-THF
5-Methyl-THF
2.1.1.148
2.1.2.10
2.1.2.8
2.1.2.7
2.1.2.1
2.1.2.5
2.1.2.3
2.1.2.2
1.5.1.3
1.5.1.3
1.5.1.3
6.3.4.3
3.5.4.9
6.3.3.2
2.1.2.10
1.5.1.5
1.5.1.15
3.5.1.10
1.5.1.6
2.1.2.9
2.1.2.2
4.3.1.4
1.5.7.1
1.5.1.20
2.1.2.4
2.1.2.5
2.1.1.13
2.1.1.19
2.1.1.45
00670 10/7/14
(c) Kanehisa Laboratories

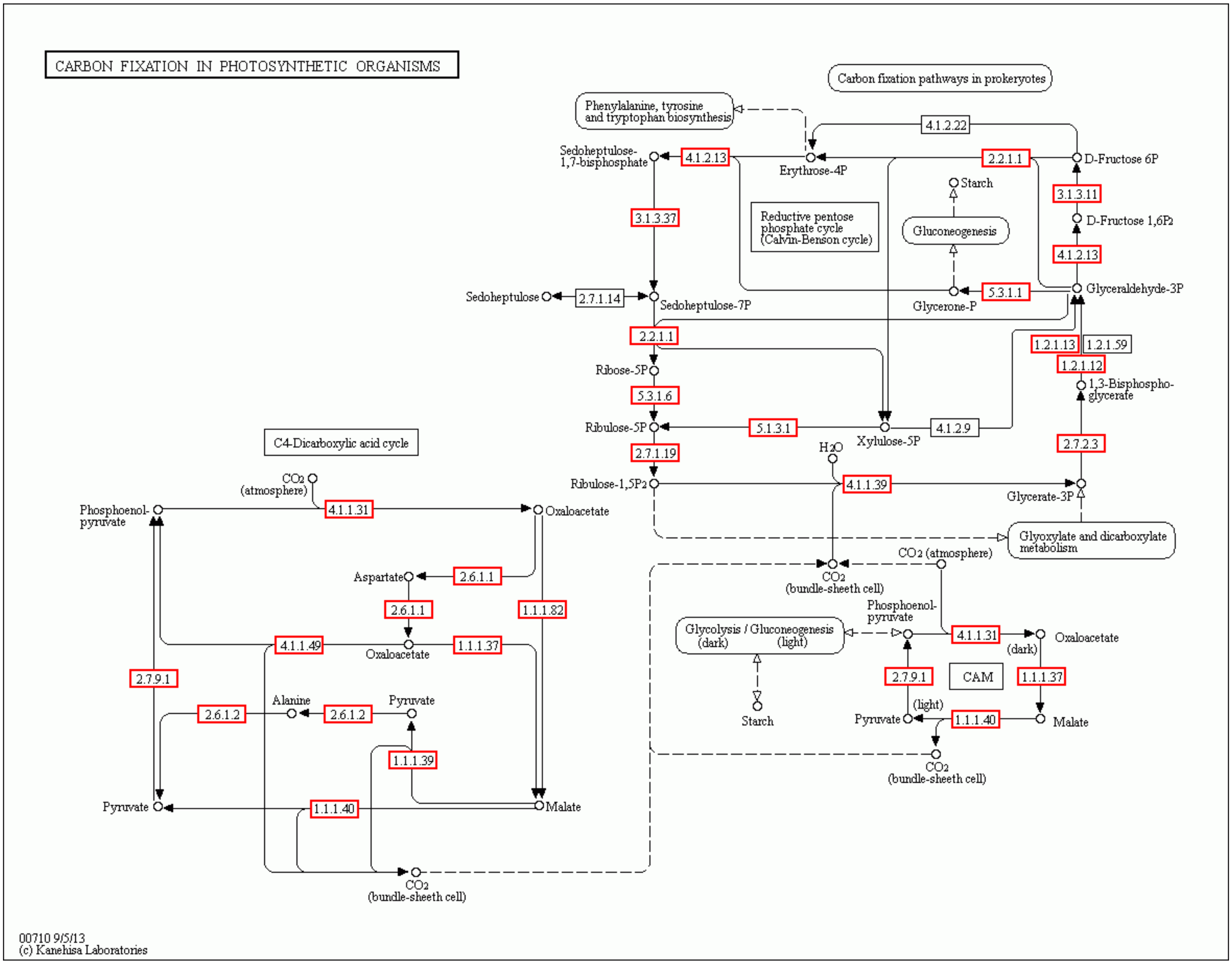
CARBON FIXATION IN PHOTOSYNTHETIC ORGANISMS
Carbon fixation pathways in prokaryotes
Phenylalanine, tyrosine and tryptophan biosynthesis
Sedoheptulose-1,7-bisphosphate
Erythrose-4P
D-Fructose 6P
D-Fructose 1,6P2
Starch
Reductive pentose phosphate cycle (Calvin-Benson cycle)
Gluconeogenesis
Glyceraldehyde-3P
Glycerone-P
Sedoheptulose
Sedoheptulose-7P
Ribose-5P
Ribulose-5P
Xylulose-5P
Ribulose-1,5P2
1,3-Bisphospho-glycerate
Glycerate-3P
Glyoxylate and dicarboxylate metabolism
C4-Dicarboxylic acid cycle
CO2 (atmosphere)
Phosphoenol-pyruvate
Oxaloacetate
Aspartate
Alanine
Pyruvate
Malate
CO2 (bundle-sheeth cell)
Glycolysis / Gluconeogenesis (dark) (light)
CAM
H2O
4.1.2.22 4.1.2.13 2.2.1.1 3.1.3.11 3.1.3.37 5.3.1.1 2.7.1.14 1.2.1.13 1.2.1.59 1.2.1.12 5.3.1.6 5.1.3.1 4.1.2.9 2.7.1.19 4.1.1.39 2.7.2.3 4.1.1.31 2.6.1.1 1.1.1.82 4.1.1.49 1.1.1.37 2.7.9.1 2.6.1.2 1.1.1.39 1.1.1.40
00710 9/5/13
(c) Kanehisa Laboratories

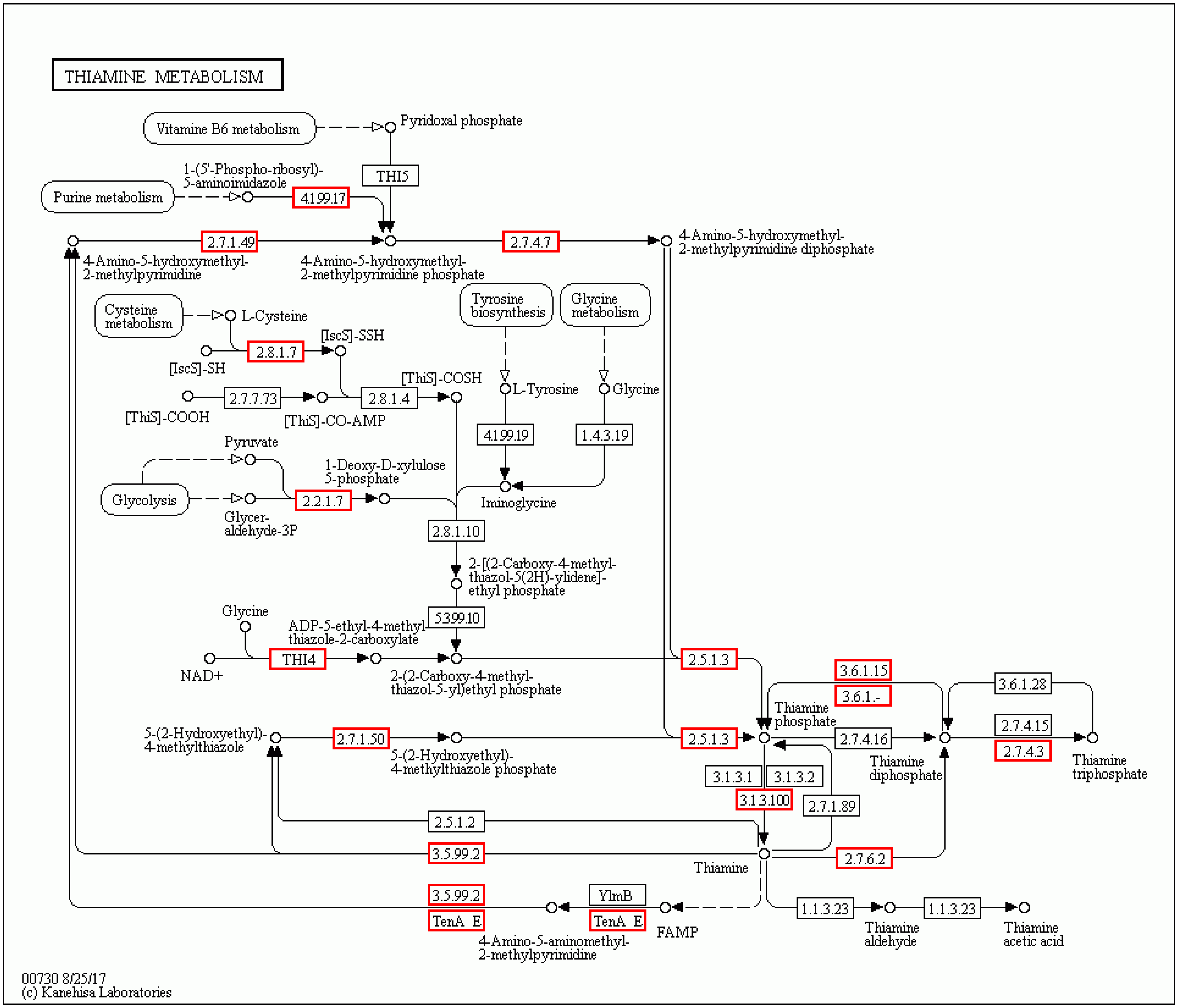
THIAMINE METABOLISM
Vitamine B6 metabolism
Pyridoxal phosphate
THI5
Purine metabolism
1-(5'-Phospho-ribosyl)-5-aminoimidazole
4.1.99.17
2.7.1.49
2.7.4.7
4-Amino-5-hydroxymethyl-2-methylpyrimidine
4-Amino-5-hydroxymethyl-2-methylpyrimidine phosphate
4-Amino-5-hydroxymethyl-2-methylpyrimidine diphosphate
Cysteine metabolism
L-Cysteine
2.8.1.7
[IscS]-SSH
[IscS]-SH
Tyrosine biosynthesis
Glycine metabolism
[ThiS]-COSH
2.7.7.73
2.8.1.4
[ThiS]-COOH
[ThiS]-CO-AMP
L-Tyrosine
Glycine
4.1.99.19
1.4.3.19
Pyruvate
1-Deoxy-D-xylulose 5-phosphate
Glycolysis
2.2.1.7
Glyceraldehyde-3P
Iminoglycine
2.8.1.10
2-[(2-Carboxy-4-methyl-thiazol-5(2H)-ylidene]-ethyl phosphate
Glycine
ADP-5-ethyl-4-methylthiazole-2-carboxylate
5.3.99.10
NAD+
THI4
2-(2-Carboxy-4-methyl-thiazol-5-yl)ethyl phosphate
2.5.1.3
3.6.1.15
3.6.1.-
3.6.1.28
Thiamine phosphate
5-(2-Hydroxyethyl)-4-methylthiazole
2.7.1.50
5-(2-Hydroxyethyl)-4-methylthiazole phosphate
2.5.1.3
2.7.4.16
2.7.4.15
2.7.4.3
Thiamine diphosphate
Thiamine triphosphate
3.1.3.1
3.1.3.2
3.1.3.100
2.7.1.89
2.5.1.2
3.5.99.2
Thiamine
2.7.6.2
3.5.99.2
TenA_E
YlmB
TenA_E
FAMP
4-Amino-5-aminomethyl-2-methylpyrimidine
1.1.3.23
1.1.3.23
Thiamine aldehyde
Thiamine acetic acid
00730 8/25/17
(c) Kanehisa Laboratories

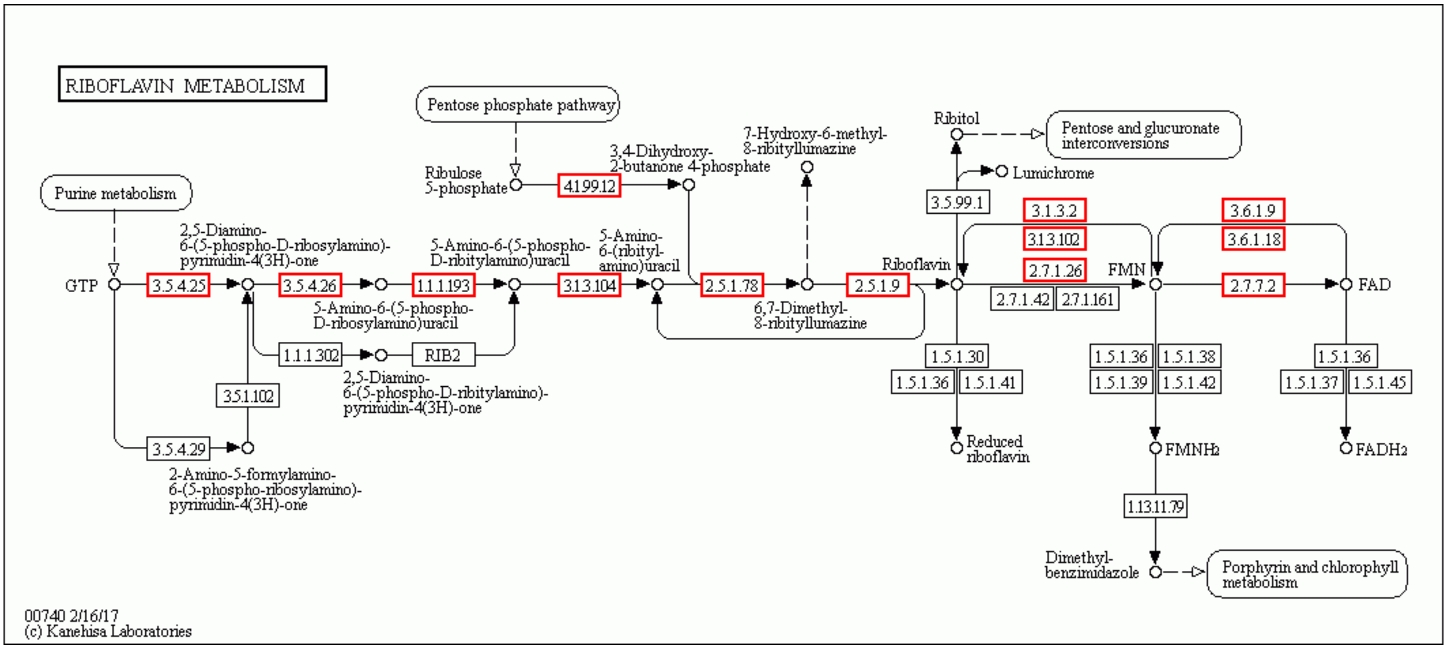
RIBOFLAVIN METABOLISM
Pentose phosphate pathway
Purine metabolism
Ribulose 5-phosphate
4.1.99.12
3,4-Dihydroxy-2-butanone 4-phosphate
7-Hydroxy-6-methyl-8-ribityllumazine
Ribitol
Pentose and glucuronate interconversions
Lumichrome
3.5.99.1
GTP
3.5.4.25
2,5-Diamino-6-(5-phospho-D-ribosylamino)-pyrimidin-4(3H)-one
3.5.4.26
5-Amino-6-(5-phospho-D-ribitylamino)uracil
1.1.1.193
3.1.3.104
5-Amino-6-(ribitylamino)uracil
2.5.1.78
2.5.1.9
Riboflavin
3.1.3.2
3.1.3.102
2.7.1.26
FMN
3.6.1.9
3.6.1.18
2.7.7.2
FAD
5-Amino-6-(5-phospho-D-ribosylamino)uracil
6,7-Dimethyl-8-ribityllumazine
2.7.1.42
2.7.1.161
1.1.1.302
RIB2
3.5.1.102
2,5-Diamino-6-(5-phospho-D-ribitylamino)-pyrimidin-4(3H)-one
1.5.1.30
1.5.1.36
1.5.1.41
1.5.1.36
1.5.1.38
1.5.1.39
1.5.1.42
1.5.1.36
1.5.1.37
1.5.1.45
3.5.4.29
2-Amino-5-formylamino-6-(5-phospho-ribosylamino)-pyrimidin-4(3H)-one
Reduced riboflavin
FMNH2
FADH2
1.13.11.79
Dimethylbenzimidazole
Porphyrin and chlorophyll metabolism
00740 2/16/17
(c) Kanehisa Laboratories

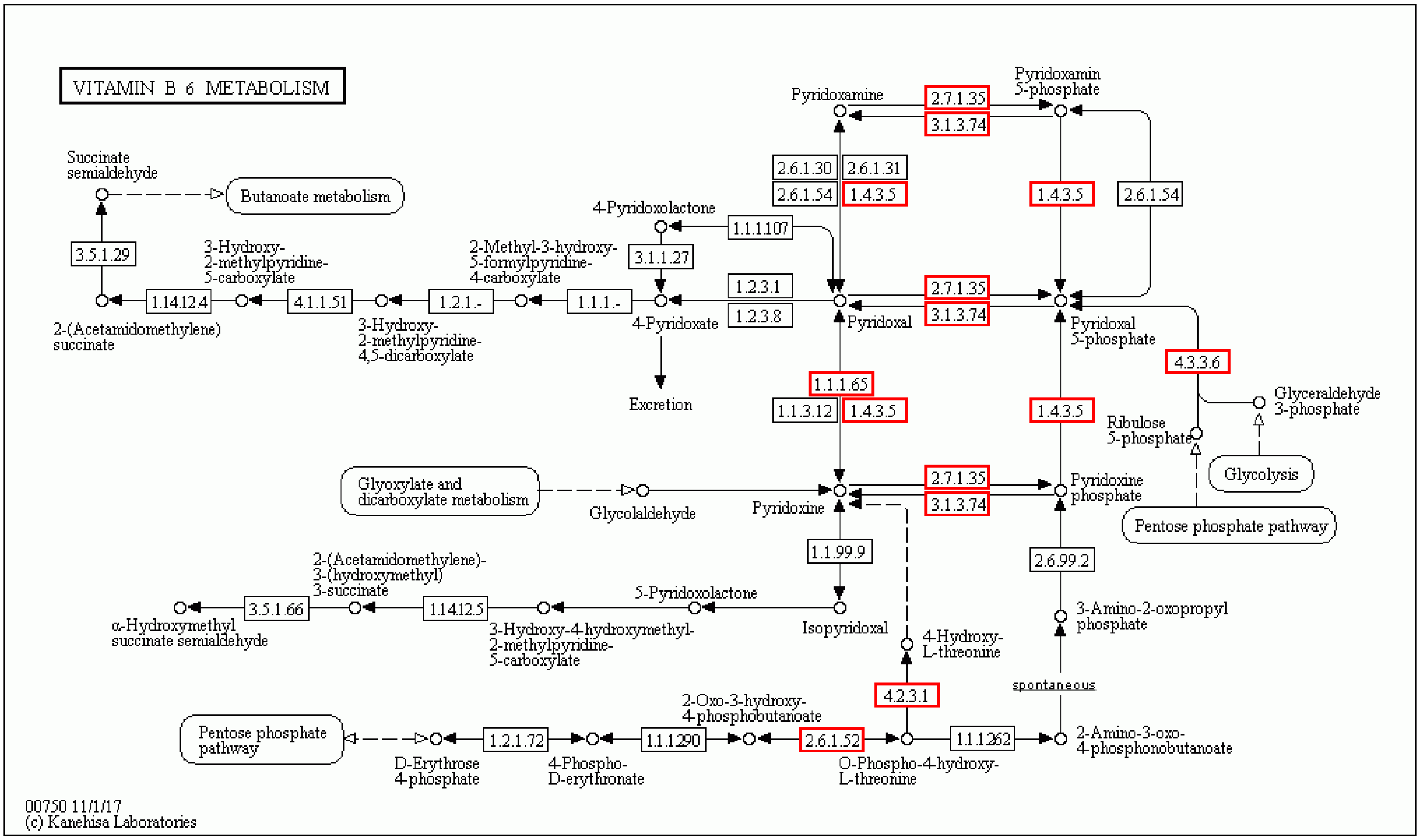
VITAMIN B 6 METABOLISM
Pyridoxamine
2.7.1.35
3.1.3.74
Pyridoxamin 5-phosphate
Succinate semialdehyde
Butanoate metabolism
2.6.1.30
2.6.1.31
2.6.1.54
1.4.3.5
1.4.3.5
2.6.1.54
4-Pyridoxolactone
1.1.1.107
3.5.1.29
3-Hydroxy-2-methylpyridine-5-carboxylate
2-Methyl-3-hydroxy-5-formylpyridine-4-carboxylate
3.1.1.27
1.2.3.1
2.7.1.35
1.14.12.4
4.1.1.51
1.2.1.-
1.1.1.-
1.2.3.8
3.1.3.74
2-(Acetamidomethylene) succinate
3-Hydroxy-2-methylpyridine-4,5-dicarboxylate
4-Pyridoxate
Pyridoxal
Pyridoxal 5-phosphate
4.3.3.6
Excretion
1.1.1.65
1.1.3.12
1.4.3.5
1.4.3.5
Ribulose 5-phosphate
Glyceraldehyde 3-phosphate
Glycolysis
Glyoxylate and dicarboxylate metabolism
Glycolaldehyde
Pyridoxine
2.7.1.35
3.1.3.74
Pyridoxine phosphate
Pentose phosphate pathway
2-(Acetamidomethylene)-3-(hydroxymethyl)-3-succinate
1.1.99.9
2.6.99.2
5-Pyridoxolactone
3.5.1.66
1.14.12.5
Isopyridoxal
3-Amino-2-oxopropyl phosphate
α-Hydroxymethyl succinate semialdehyde
3-Hydroxy-4-hydroxymethyl-2-methylpyridine-5-carboxylate
4-Hydroxy-L-threonine
spontaneous
4.2.3.1
2-Oxo-3-hydroxy-4-phosphobutanoate
Pentose phosphate pathway
1.2.1.72
1.1.1.290
2.6.1.52
1.1.1.262
2-Amino-3-oxo-4-phosphonobutanoate
D-Erythrose 4-phosphate
4-Phospho-D-erythronate
O-Phospho-4-hydroxy-L-threonine
00750 11/1/17
(c) Kanehisa Laboratories

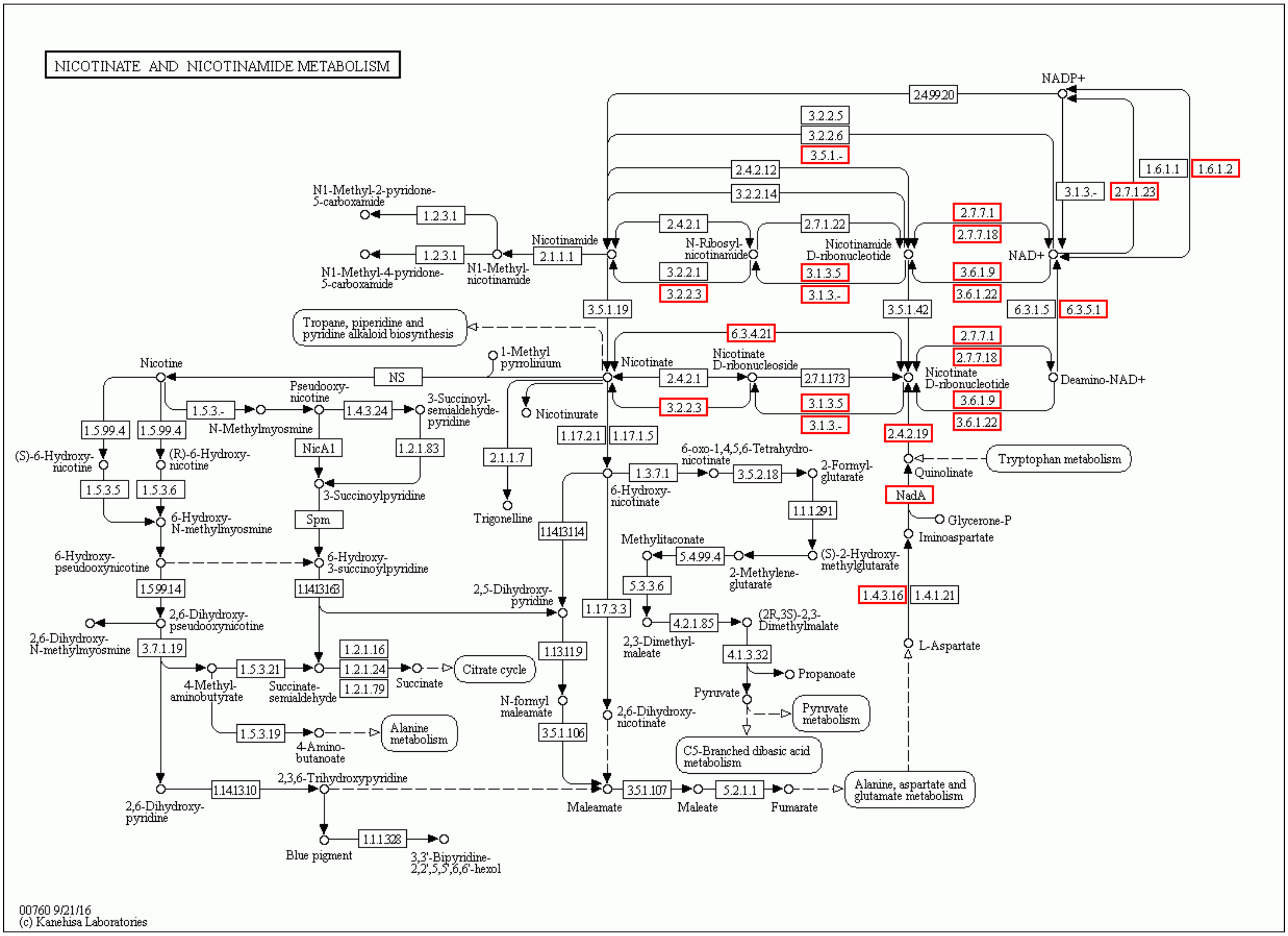
NICOTINATE AND NICOTINAMIDE METABOLISM
NADP+
2.4.99.20
3.2.2.5
3.2.2.6
3.5.1.-
1.6.1.1
1.6.1.2
2.4.2.12
3.1.3.-
2.7.1.23
3.2.2.14
2.7.7.1
2.7.7.18
N1-Methyl-2-pyridone-5-carboxamide
1.2.3.1
2.4.2.1
2.7.1.22
Nicotinamide
N-Ribosyl-nicotinamide
Nicotinamide D-ribonucleotide
NAD+
1.2.3.1
2.1.1.1
N1-Methyl-4-pyridone-5-carboxamide
N1-Methyl-nicotinamide
3.2.2.1
3.1.3.5
3.6.1.9
3.2.2.3
3.1.3.-
3.6.1.22
3.5.1.19
3.5.1.42
6.3.1.5
6.3.5.1
Tropane, piperidine and pyridine alkaloid biosynthesis
6.3.4.21
2.7.7.1
1-Methyl pyrrolinium
2.7.7.18
Nicotine
NS
Nicotinate
Nicotinate D-ribonucleoside
Nicotinate D-ribonucleotide
Deamino-NAD+
Pseudooxy-nicotine
2.4.2.1
2.7.1.173
1.5.3.-
1.4.3.24
3-Succinoyl-semialdehyde-pyridine
3.2.2.3
3.1.3.5
3.6.1.9
1.5.99.4
1.5.99.4
N-Methylmyosmine
Nicotinurate
1.17.2.1
1.17.1.5
3.1.3.-
3.6.1.22
2.4.2.19
(S)-6-Hydroxy-nicotine
(R)-6-Hydroxy-nicotine
NicA1
1.2.1.83
2.1.1.7
6-oxo-1,4,5,6-Tetrahydro-nicotinate
1.3.7.1
3.5.2.18
2-Formyl-glutarate
Quinolinate
Tryptophan metabolism
1.5.3.5
1.5.3.6
3-Succinoylpyridine
6-Hydroxy-nicotinate
NadA
6-Hydroxy-N-methylmyosmine
Spm
Trigonelline
1.14.13.114
1.1.1.291
Glycerone-P
Iminoaspartate
Methylitaconate
5.4.99.4
(S)-2-Hydroxy-methylglutarate
6-Hydroxy-pseudooxynicotine
6-Hydroxy-3-succinoylpyridine
1.5.99.14
1.14.13.163
2,5-Dihydroxy-pyridine
5.3.3.6
2-Methylene-glutarate
1.4.3.16
1.4.1.21
2,6-Dihydroxy-pseudooxynicotine
1.17.3.3
4.2.1.85
(2R,3S)-2,3-Dimethylmalate
2,6-Dihydroxy-N-methylmyosmine
3.7.1.19
1.2.1.16
2,3-Dimethyl-maleate
1.13.11.9
4.1.3.32
L-Aspartate
1.5.3.21
1.2.1.24
Citrate cycle
Propanoate
4-Methyl-aminobutyrate
Succinate-semialdehyde
1.2.1.79
Succinate
Pyruvate
N-formyl maleamate
2,6-Dihydroxy-nicotinate
Pyruvate metabolism
1.5.3.19
Alanine metabolism
4-Amino-butanoate
3.5.1.106
C5-Branched dibasic acid metabolism
2,3,6-Trihydroxypyridine
1.14.13.10
3.5.1.107
5.2.1.1
Alanine, aspartate and glutamate metabolism
2,6-Dihydroxy-pyridine
Maleamate
Maleate
Fumarate
1.1.1.328
Blue pigment
3,3'-Bipyridine-2,2',5,5',6,6'-hexol
00760 9/21/16
(c) Kanehisa Laboratories

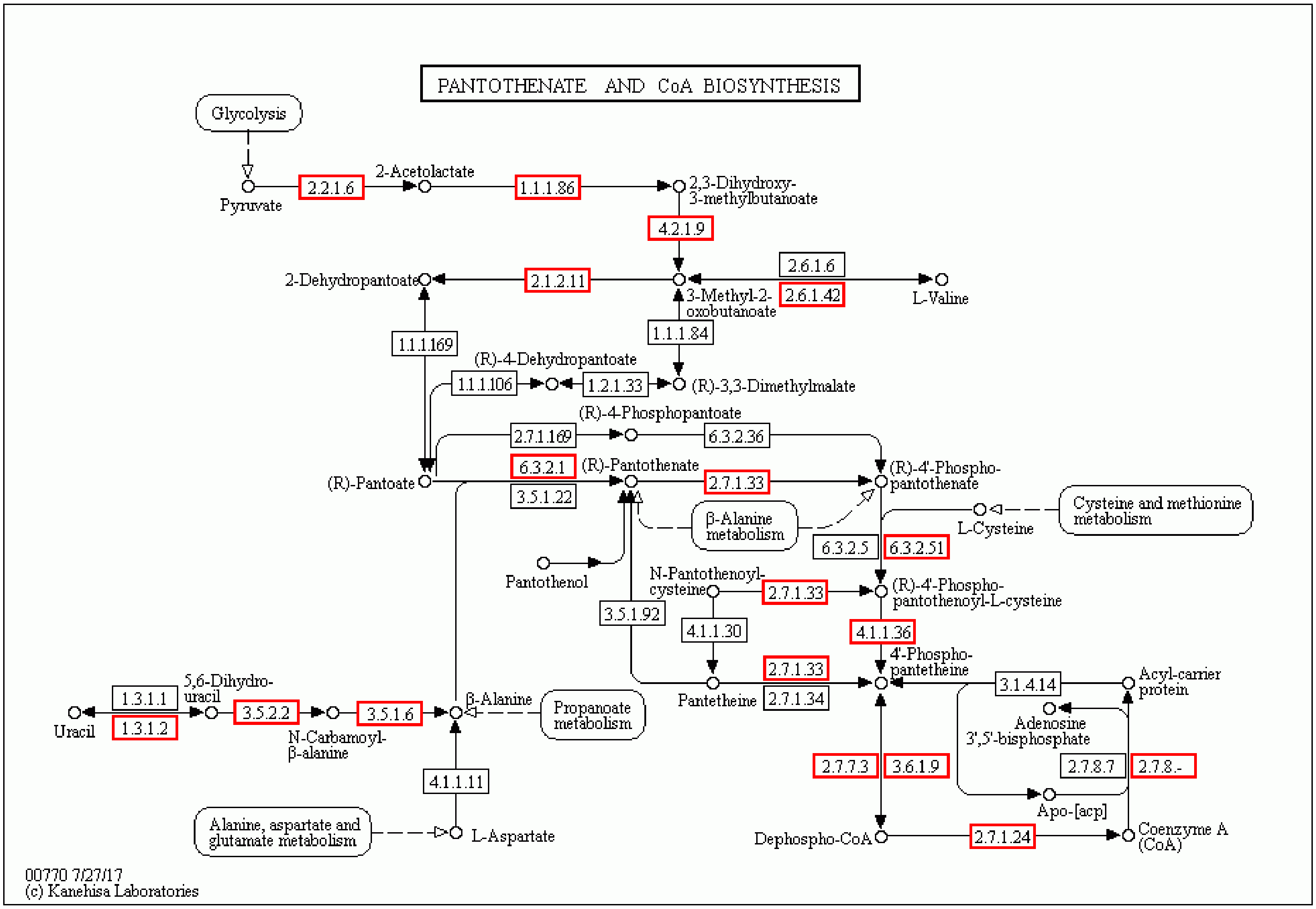
PANTOTHENATE AND CoA BIOSYNTHESIS
Glycolysis
Pyruvate
2.2.1.6
2-Acetolactate
1.1.1.86
2,3-Dihydroxy-
3-methylbutanoate
4.2.1.9
2-Dehydropantoate
2.1.2.11
2.6.1.6
3-Methyl-2-
oxobutanoate
2.6.1.42
L-Valine
1.1.1.169
1.1.1.84
(R)-4-Dehydropantoate
1.1.1.106
1.2.1.33
(R)-3,3-Dimethylmalate
(R)-4-Phosphopantoate
2.7.1.169
6.3.2.36
(R)-Pantoate
6.3.2.1
(R)-Pantothenate
2.7.1.33
(R)-4'-Phospho-
pantothenate
3.5.1.22
β-Alanine
metabolism
L-Cysteine
Cysteine and methionine
metabolism
6.3.2.5
6.3.2.51
Pantothenol
N-Pantothenoyl-
cysteine
2.7.1.33
(R)-4'-Phospho-
pantothenoyl-L-cysteine
3.5.1.92
4.1.1.30
4.1.1.36
4'-Phospho-
pantetheine
2.7.1.33
Pantetheine
2.7.1.34
3.1.4.14
Acyl-carrier
protein
1.3.1.1
5,6-Dihydro-
uracil
Uracil
1.3.1.2
3.5.2.2
N-Carbamoyl-
β-alanine
3.5.1.6
β-Alanine
Propanoate
metabolism
Adenosine
3',5'-bisphosphate
2.7.7.3
3.6.1.9
2.7.8.7
2.7.8.-
Apo-[acp]
4.1.1.11
Alanine, aspartate and
glutamate metabolism
L-Aspartate
Dephospho-CoA
2.7.1.24
Coenzyme A
(CoA)
00770 7/27/17
(c) Kanehisa Laboratories

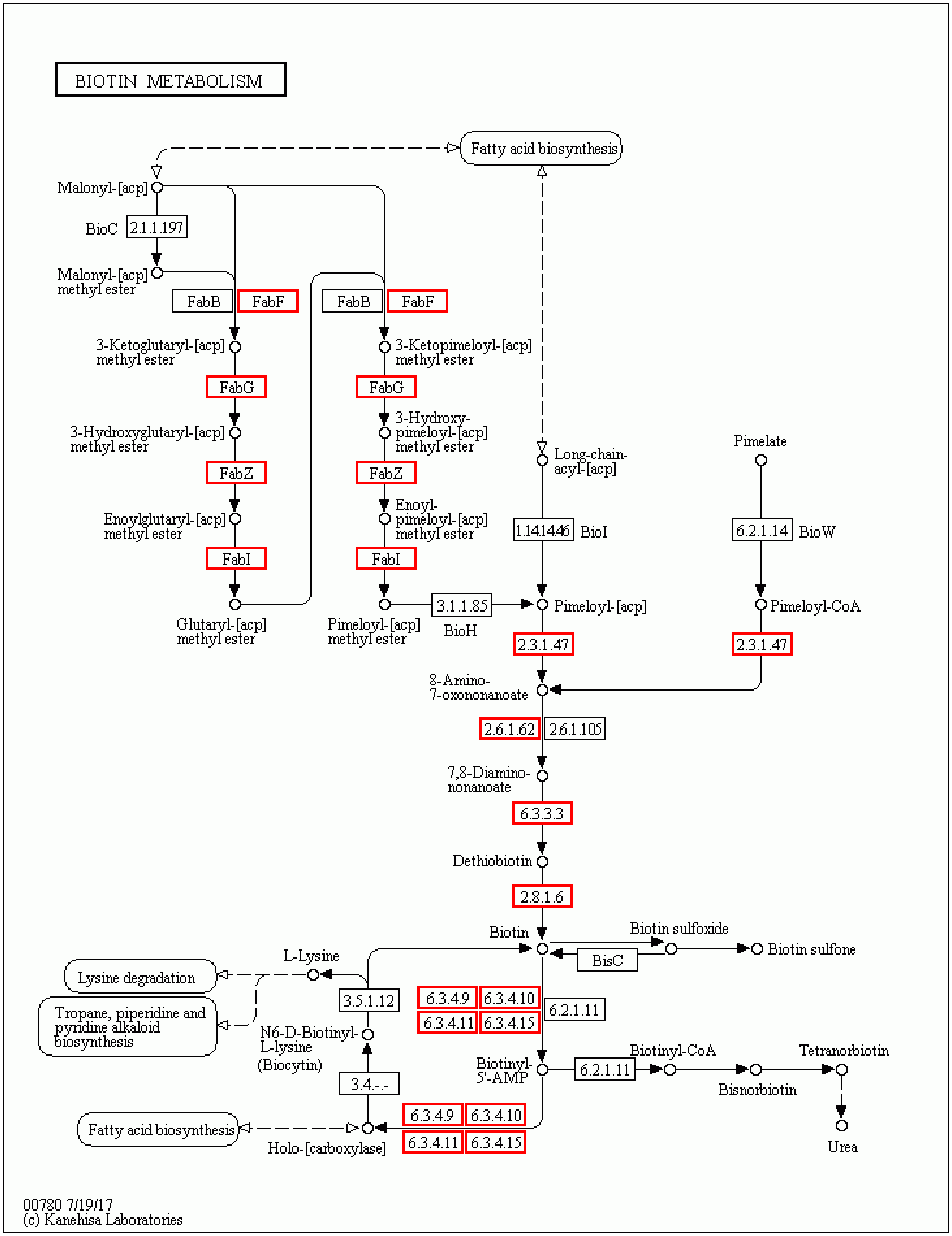
BIOTIN METABOLISM
Fatty acid biosynthesis
Malonyl-[acp]
BioC
2.1.1.197
Malonyl-[acp] methyl ester
FabB
FabF
FabB
FabF
3-Ketoglutaryl-[acp] methyl ester
3-Ketopimeloyl-[acp] methyl ester
FabG
FabG
3-Hydroxyglutaryl-[acp] methyl ester
3-Hydroxy-pimeloyl-[acp] methyl ester
FabZ
FabZ
Enoylglutaryl-[acp] methyl ester
Enoyl-pimeloyl-[acp] methyl ester
FabI
FabI
Glutaryl-[acp] methyl ester
Pimeloyl-[acp] methyl ester
3.1.1.85
BioH
Long-chain-acyl-[acp]
1.14.14.46
BioI
Pimelate
6.2.1.14
BioW
Pimeloyl-[acp]
Pimeloyl-CoA
2.3.1.47
2.3.1.47
8-Amino-7-oxononanoate
2.6.1.62
2.6.1.105
7,8-Diamino-nonanoate
6.3.3.3
Dethiobiotin
2.8.1.6
Biotin
Biotin sulfoxide
Biotin sulfone
BisC
L-Lysine
Lysine degradation
Tropane, piperidine and pyridine alkaloid biosynthesis
3.5.1.12
6.3.4.9
6.3.4.10
6.3.4.11
6.3.4.15
6.2.1.11
N6-D-Biotinyl-L-lysine (Biocytin)
Biotinyl-5'-AMP
6.2.1.11
Biotinyl-CoA
Bisnorbiotin
Tetranorbiotin
3.4.-.-
6.3.4.9
6.3.4.10
6.3.4.11
6.3.4.15
Fatty acid biosynthesis
Holo-[carboxylase]
Urea
00780 7/19/17
(c) Kanehisa Laboratories

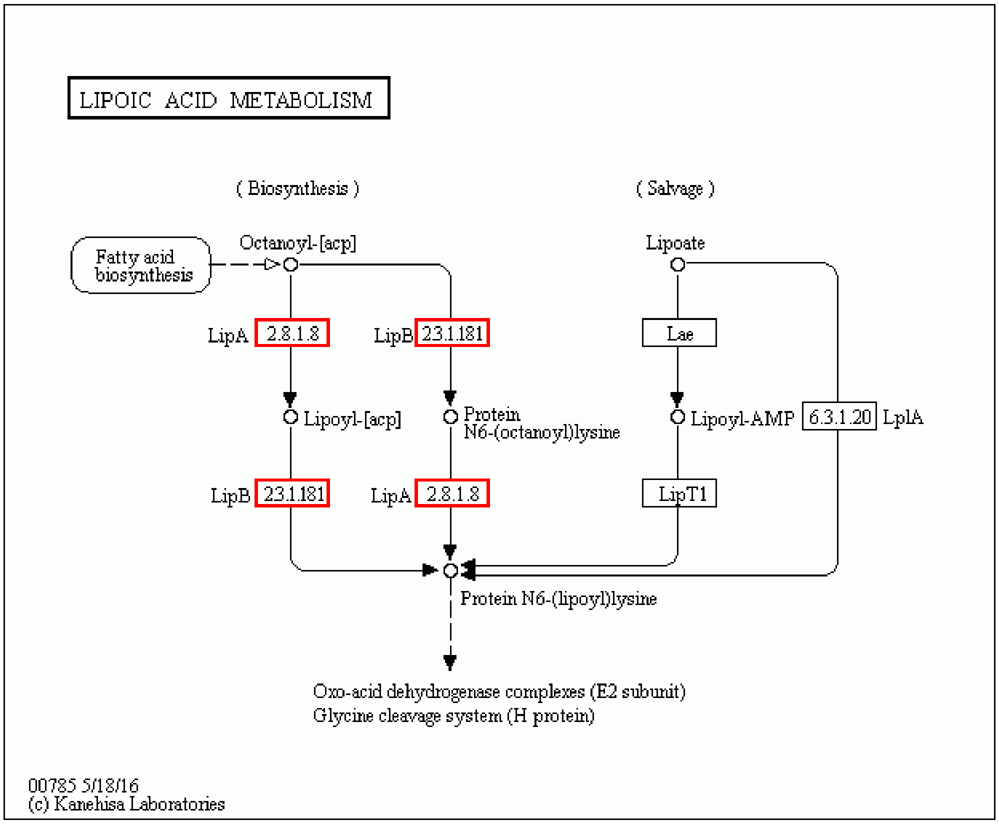
LIPOIC ACID METABOLISM
(Biosynthesis)
(Salvage)
Fatty acid biosynthesis
Octanoyl-[acp]
Lipoate
LipA 2.8.1.8
LipB 2.3.1.181
Lae
Lipoyl-[acp]
Protein N6-(octanoyl)lysine
Lipoyl-AMP
6.3.1.20 LplA
LipB 2.3.1.181
LipA 2.8.1.8
LipT1
Protein N6-(lipoyl)lysine
Oxo-acid dehydrogenase complexes (E2 subunit)
Glycine cleavage system (H protein)
00785 5/18/16
(c) Kanehisa Laboratories

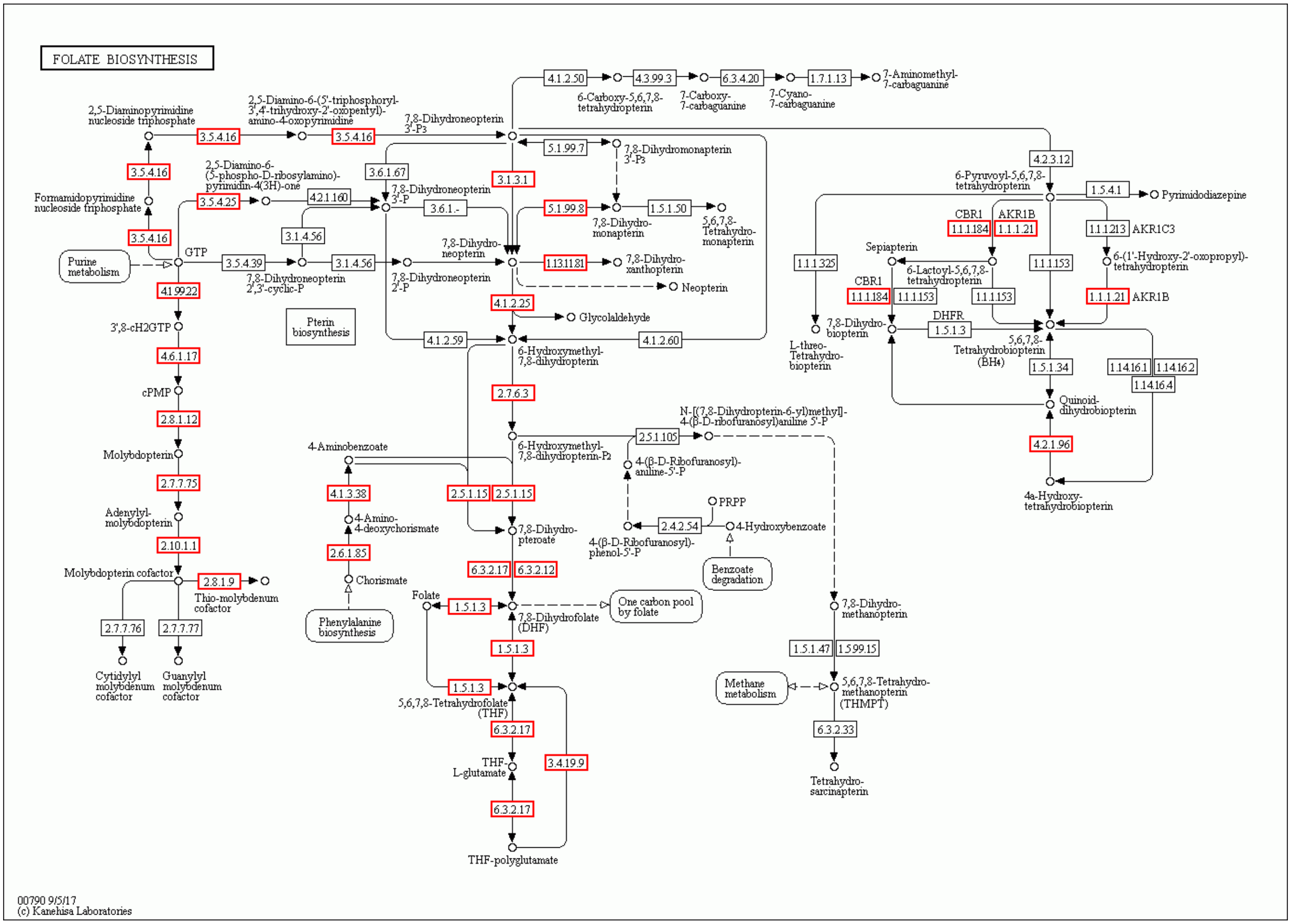
FOLATE BIOSYNTHESIS
2,5-Diaminopyrimidine nucleoside triphosphate
2,5-Diamino-6-(5'-triphosphoryl-3',4'-trihydroxy-2'-oxopentyl)-amino-4-oxopyrimidine
7,8-Dihydroneopterin 3'-P3
3.5.4.16
3.5.4.16
3.5.4.16
3.5.4.16
Formamidopyrimidine nucleoside triphosphate
2,5-Diamino-6-(5-phospho-D-ribosylamino)-pyrimidin-4(3H)-one
3.5.4.25
4.2.1.160
3.6.1.67
7,8-Dihydroneopterin 3'-P
3.6.1.-
3.1.3.1
5.1.99.7
7,8-Dihydromonapterin 3'-P3
5.1.99.8
7,8-Dihydro-monapterin
1.5.1.50
5,6,7,8-Tetrahydro-monapterin
4.1.2.50
6-Carboxy-5,6,7,8-tetrahydropterin
4.3.99.3
7-Carboxy-7-carbaguanine
6.3.4.20
7-Cyano-7-carbaguanine
1.7.1.13
7-Aminomethyl-7-carbaguanine
Purine metabolism
GTP
3.5.4.39
3.1.4.56
3.1.4.56
7,8-Dihydroneopterin 2',3'-cyclic-P
7,8-Dihydroneopterin 2'-P
7,8-Dihydro-neopterin
1.13.11.81
7,8-Dihydro-xanthopterin
Neopterin
4.1.99.22
3',8-cH2GTP
4.6.1.17
cPMP
2.8.1.12
Molybdopterin
2.7.7.75
Adenylyl-molybdopterin
2.10.1.1
Molybdopterin cofactor
2.8.1.9
Thio-molybdenum cofactor
2.7.7.76
2.7.7.77
Cytidylyl molybdenum cofactor
Guanylyl molybdenum cofactor
Pterin biosynthesis
4.1.2.25
Glycolaldehyde
4.1.2.59
4.1.2.60
6-Hydroxymethyl-7,8-dihydropterin
2.7.6.3
6-Hydroxymethyl-7,8-dihydropterin-P2
2.5.1.105
N-[(7,8-Dihydropterin-6-yl)methyl]-4-(β-D-ribofuranosyl)aniline 5'-P
4-(β-D-Ribofuranosyl)-aniline-5'-P
PRPP
2.4.2.54
4-Hydroxybenzoate
4-(β-D-Ribofuranosyl)-phenol-5'-P
Benzoate degradation
4-Aminobenzoate
4.1.3.38
4-Amino-4-deoxychorismate
2.6.1.85
Chorismate
Phenylalanine biosynthesis
2.5.1.15
2.5.1.15
7,8-Dihydro-pteroate
6.3.2.17
6.3.2.12
Folate
1.5.1.3
7,8-Dihydrofolate (DHF)
One carbon pool by folate
1.5.1.3
1.5.1.3
5,6,7,8-Tetrahydrofolate (THF)
6.3.2.17
THF-L-glutamate
3.4.19.9
6.3.2.17
THF-polyglutamate
7,8-Dihydro-methanopterin
1.5.1.47
1.5.99.15
Methane metabolism
5,6,7,8-Tetrahydro-methanopterin (THMPT)
6.3.2.33
Tetrahydro-sarcinapterin
4.2.3.12
6-Pyruvoyl-5,6,7,8-tetrahydropterin
1.5.4.1
Pyrimidodiazepine
CBR1
AKR1B
1.1.1.184
1.1.1.21
1.1.1.213 AKR1C3
6-(1'-Hydroxy-2'-oxopropyl)-tetrahydropterin
Sepiapterin
1.1.1.325
6-Lactoyl-5,6,7,8-tetrahydropterin
1.1.1.153
CBR1
1.1.1.184
1.1.1.153
1.1.1.153
1.1.1.21 AKR1B
7,8-Dihydro-biopterin
DHFR
1.5.1.3
L-threo-Tetrahydro-biopterin
5,6,7,8-Tetrahydrobiopterin (BH4)
1.5.1.34
1.14.16.1
1.14.16.2
1.14.16.4
Quinoid-dihydrobiopterin
4.2.1.96
4a-Hydroxy-tetrahydrobiopterin
00790 9/5/17
(c) Kanehisa Laboratories

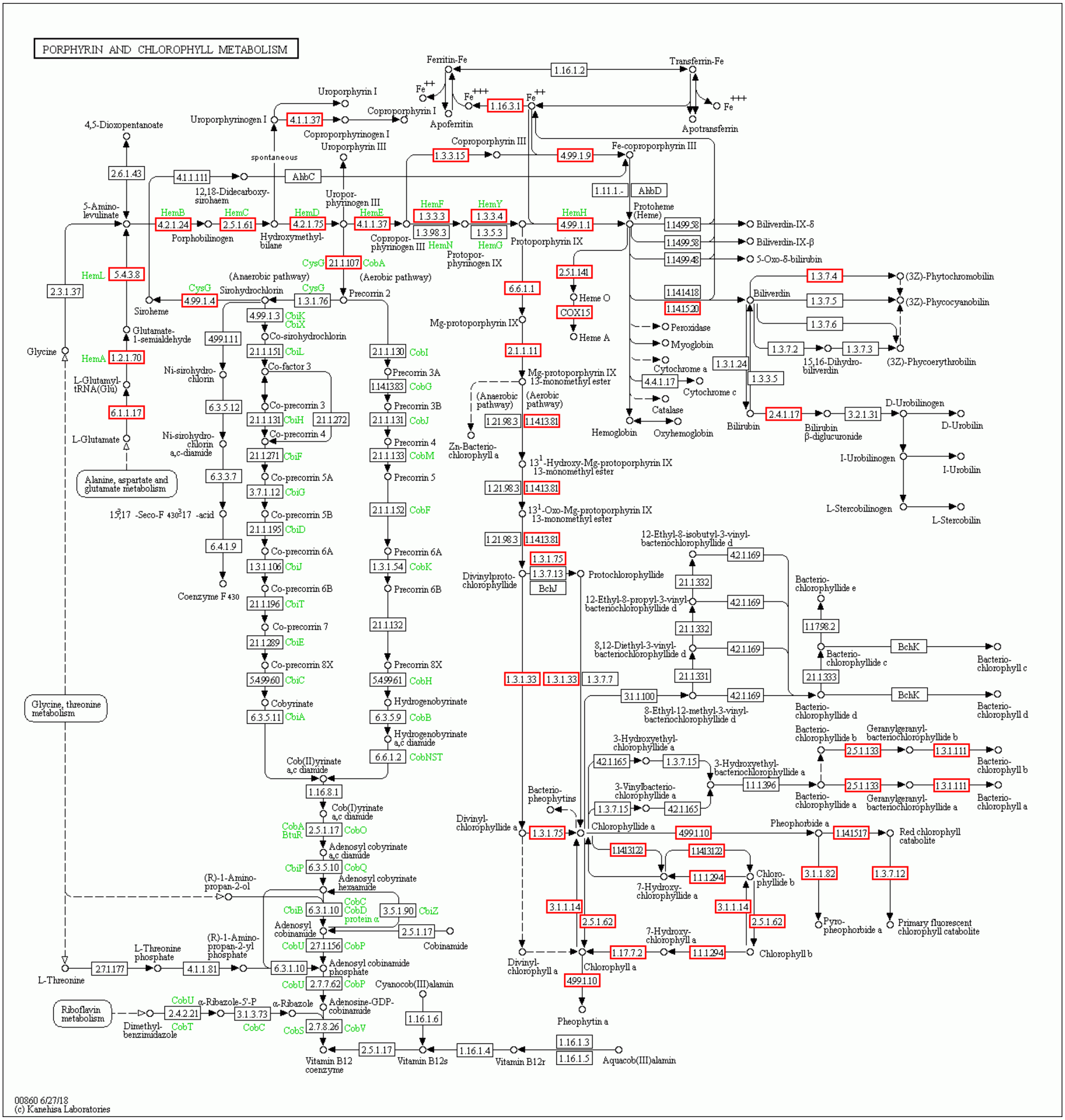
PORPHYRIN AND CHLOROPHYLL METABOLISM
Ferritin-Fe
1.16.1.2
Transferrin-Fe
Fe++
Fe+++
1.16.3.1
Fe++
Fe+++
Apoferritin
Apotransferrin
Uroporphyrin I
Uroporphyrinogen I
4.1.1.37
Coproporphyrin I
Coproporphyrinogen I
Uroporphyrin III
Coproporphyrin III
Fe-coproporphyrin III
1.3.3.15
4.99.1.9
4,5-Dioxopentanoate
2.6.1.43
4.1.1.111
AhbC
spontaneous
12,18-Didecarboxy-siroheam
1.11.1.-
AhbD
Protoheme (Heme)
5-Amino-levulinate
HemB
4.2.1.24
Porphobilinogen
HemC
2.5.1.61
Hydroxymethyl-bilane
HemD
4.2.1.75
Uroporphyrinogen III
HemE
4.1.1.37
Coproporphyrinogen III
HemF
1.3.3.3
1.3.98.3
HemN
HemY
1.3.3.4
1.3.5.3
HemG
Protoporphyrinogen IX
Protoporphyrin IX
HemH
4.99.1.1
1.14.99.58
Biliverdin-IX-δ
1.14.99.58
Biliverdin-IX-β
1.14.99.48
5-Oxo-δ-bilirubin
CysG
2.1.1.107
CobA
HemL
5.4.3.8
(Anaerobic pathway)
(Aerobic pathway)
2.5.1.141
1.3.7.4
(3Z)-Phytochromobilin
2.3.1.37
CysG
4.99.1.4
Sirohydrochlorin
CysG
1.3.1.76
Precorrin 2
6.6.1.1
Heme O
1.14.14.18
1.14.15.20
Biliverdin
1.3.7.5
(3Z)-Phycocyanobilin
Siroheme
4.99.1.3
CbiK
CbiX
COX15
Heme A
1.3.7.6
Glutamate-1-semialdehyde
Co-sirohydrochlorin
Mg-protoporphyrin IX
Peroxidase
Myoglobin
Glycine
HemA
1.2.1.70
4.99.1.11
2.1.1.151
CbiL
2.1.1.130
CobI
2.1.1.11
1.3.7.2
15,16-Dihydro-biliverdin
1.3.7.3
(3Z)-Phycoerythrobilin
Co-factor 3
Precorrin 3A
Cytochrome a
1.3.1.24
L-Glutamyl-tRNA(Glu)
Ni-sirohydro-chlorin
1.14.13.83
CobG
Mg-protoporphyrin IX 13-monomethyl ester
4.4.1.17
Cytochrome c
1.3.3.5
6.3.5.12
Co-precorrin 3
Precorrin 3B
(Anaerobic pathway)
(Aerobic pathway)
Catalase
D-Urobilinogen
6.1.1.17
2.1.1.131
CbiH
2.1.1.272
2.1.1.131
CobJ
1.21.98.3
1.14.13.81
Hemoglobin
Oxyhemoglobin
Bilirubin
2.4.1.17
Bilirubin β-diglucuronide
3.2.1.31
D-Urobilin
L-Glutamate
Ni-sirohydro-chlorin a,c-diamide
Co-precorrin 4
Precorrin 4
Zn-Bacterio-chlorophyll a
2.1.1.271
CbiF
2.1.1.133
CobM
13[1]-Hydroxy-Mg-protoporphyrin IX 13-monomethyl ester
I-Urobilinogen
I-Urobilin
Alanine, aspartate and glutamate metabolism
6.3.3.7
Co-precorrin 5A
Precorrin 5
3.7.1.12
CbiG
1.21.98.3
1.14.13.81
15[1],17[3]-Seco-F430-17[3]-acid
Co-precorrin 5B
13[1]-Oxo-Mg-protoporphyrin IX 13-monomethyl ester
L-Stercobilinogen
L-Stercobilin
2.1.1.195
CbiD
2.1.1.152
CobF
12-Ethyl-8-isobutyl-3-vinyl-bacteriochlorophyllide d
6.4.1.9
Co-precorrin 6A
Precorrin 6A
1.21.98.3
1.14.13.81
4.2.1.169
1.3.1.106
CbiJ
1.3.1.54
CobK
1.3.1.75
Divinylproto-chlorophyllide
1.3.7.13
Protochlorophyllide
BchJ
Coenzyme F430
Co-precorrin 6B
Precorrin 6B
2.1.1.332
Bacterio-chlorophyllide e
2.1.1.196
CbiT
12-Ethyl-8-propyl-3-vinyl-bacteriochlorophyllide d
4.2.1.169
Co-precorrin 7
2.1.1.132
2.1.1.332
1.17.98.2
2.1.1.289
CbiE
8,12-Diethyl-3-vinyl-bacteriochlorophyllide d
4.2.1.169
BchK
Bacterio-chlorophyll c
Bacterio-chlorophyllide c
Co-precorrin 8X
Precorrin 8X
2.1.1.331
2.1.1.333
5.4.99.60
CbiC
5.4.99.61
CobH
1.3.1.33
1.3.1.33
1.3.7.7
Glycine, threonine metabolism
3.1.1.100
4.2.1.169
BchK
Cobyrinate
Hydrogenobyrinate
8-Ethyl-12-methyl-3-vinyl-bacteriochlorophyllide d
Bacterio-chlorophyllide d
Bacterio-chlorophyll d
6.3.5.11
CbiA
6.3.5.9
CobB
Hydrogenobyrinate a,c diamide
3-Hydroxyethyl-chlorophyllide a
Bacterio-chlorophyllide b
Geranylgeranyl-bacteriochlorophyllide b
6.6.1.2
CobNST
4.2.1.165
1.3.7.15
3-Hydroxyethyl-bacteriochlorophyllide a
2.5.1.133
1.3.1.111
Bacterio-chlorophyll b
Cob(II)yrinate a,c diamide
1.16.8.1
3-Vinylbacterio-chlorophyllide a
1.1.1.396
2.5.1.133
1.3.1.111
Bacterio-pheophytins
Bacterio-chlorophyllide a
Geranylgeranyl-bacteriochlorophyllide a
Bacterio-chlorophyll a
Cob(I)yrinate a,c diamide
1.3.7.15
4.2.1.165
CobA
BtuR
2.5.1.17
CobO
Divinyl-chlorophyllide a
1.3.1.75
Chlorophyllide a
4.99.1.10
Pheophorbide a
1.14.15.17
Red chlorophyll catabolite
Adenosyl cobyrinate a,c diamide
1.14.13.122
1.14.13.122
CbiP
6.3.5.10
CobQ
Adenosyl cobyrinate hexaamide
(R)-1-Amino-propan-2-ol
7-Hydroxy-chlorophyllide a
1.1.1.294
Chloro-phyllide b
3.1.1.82
1.3.7.12
CobC
CobD
protein α
CbiB
6.3.1.10
3.5.1.90
CbiZ
3.1.1.14
3.1.1.14
Adenosyl cobinamide
2.5.1.17
2.5.1.62
2.5.1.62
Pyro-pheophorbide a
Primary fluorescent chlorophyll catabolite
L-Threonine phosphate
(R)-1-Amino-propan-2-yl phosphate
CobU
2.7.1.156
CobP
Cobinamide
7-Hydroxy-chlorophyll a
L-Threonine
2.7.1.177
4.1.1.81
6.3.1.10
Adenosyl cobinamide phosphate
Divinyl-chlorophyll a
1.17.7.2
1.1.1.294
Chlorophyll b
Chlorophyll a
CobU
2.7.7.62
CobP
Cyanocob(III)alamin
4.99.1.10
Riboflavin metabolism
CobU
α-Ribazole-5'-P
α-Ribazole
2.4.2.21
3.1.3.73
Adenosine-GDP-cobinamide
1.16.1.6
Pheophytin a
Dimethyl-benzimidazole
CobT
CobC
CobS
2.7.8.26
CobV
1.16.1.3
2.5.1.17
1.16.1.4
1.16.1.5
Vitamin B12 coenzyme
Vitamin B12s
Vitamin B12r
Aquacob(III)alamin
00860 6/27/18
(c) Kanehisa Laboratories

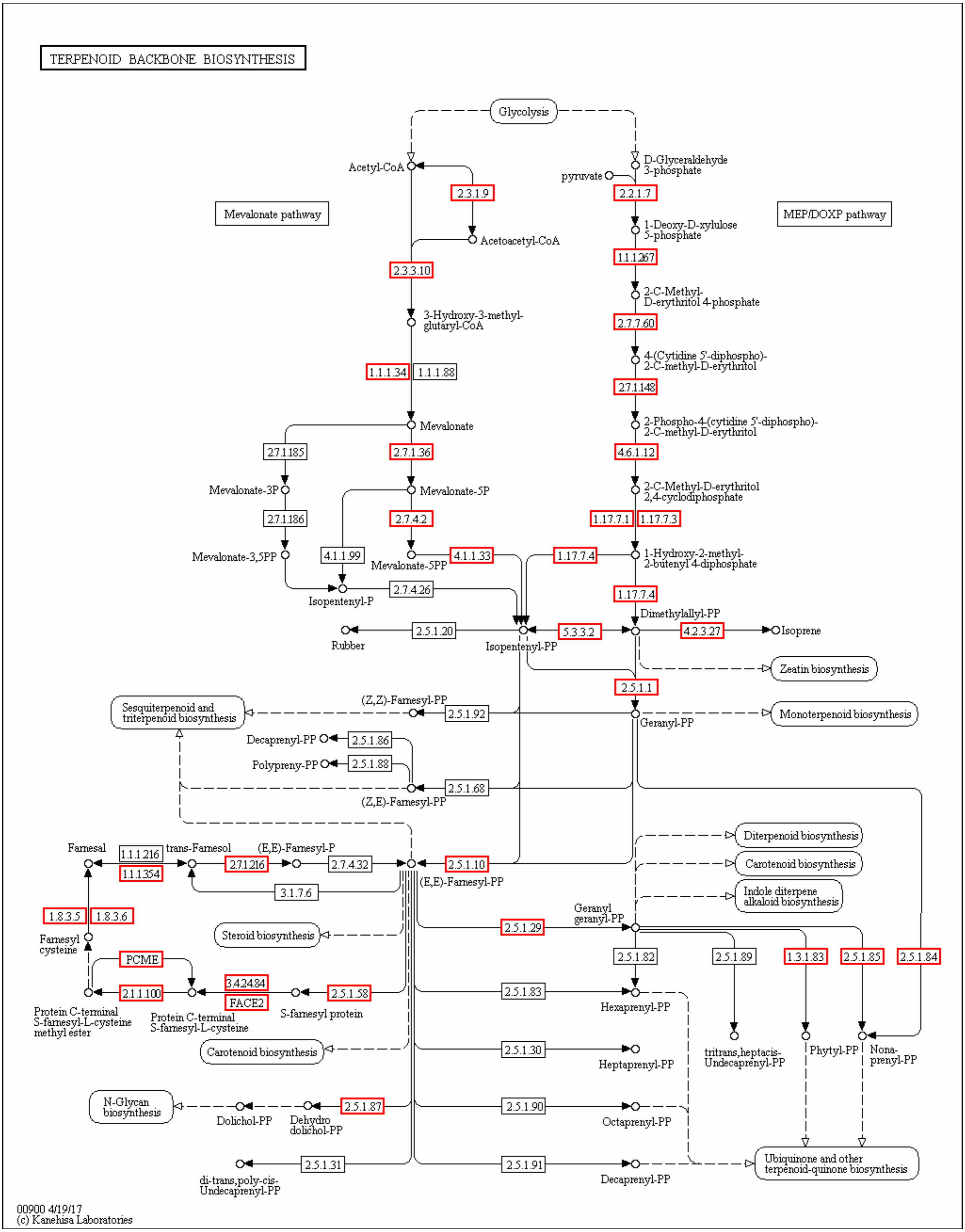
TERPENOID BACKBONE BIOSYNTHESIS
Glycolysis
Acetyl-CoA
D-Glyceraldehyde 3-phosphate
pyruvate
2.3.1.9
2.2.1.7
Mevalonate pathway
MEP/DOXP pathway
Acetoacetyl-CoA
1-Deoxy-D-xylulose 5-phosphate
1.1.1.267
2.3.3.10
2-C-Methyl-D-erythritol 4-phosphate
3-Hydroxy-3-methyl-glutaryl-CoA
2.7.7.60
4-(Cytidine 5'-diphospho)-2-C-methyl-D-erythritol
1.1.1.34
1.1.1.88
2.7.1.148
Mevalonate
2-Phospho-4-(cytidine 5'-diphospho)-2-C-methyl-D-erythritol
2.7.1.185
2.7.1.36
4.6.1.12
Mevalonate-3P
Mevalonate-5P
2-C-Methyl-D-erythritol 2,4-cyclodiphosphate
2.7.1.186
2.7.4.2
1.17.7.1
1.17.7.3
Mevalonate-3,5PP
4.1.1.99
Mevalonate-5PP
4.1.1.33
1.17.7.4
1-Hydroxy-2-methyl-2-butenyl 4-diphosphate
Isopentenyl-P
2.7.4.26
1.17.7.4
Dimethylallyl-PP
Rubber
2.5.1.20
Isopentenyl-PP
5.3.3.2
4.2.3.27
Isoprene
Zeatin biosynthesis
2.5.1.1
(Z,Z)-Farnesyl-PP
Sesquiterpenoid and triterpenoid biosynthesis
2.5.1.92
Geranyl-PP
Monoterpenoid biosynthesis
Decaprenyl-PP
2.5.1.86
Polypreny-PP
2.5.1.88
2.5.1.68
(Z,E)-Farnesyl-PP
Diterpenoid biosynthesis
Carotenoid biosynthesis
Farnesal
1.1.1.216
trans-Farnesol
(E,E)-Farnesyl-P
1.1.1.354
2.7.1.216
2.7.4.32
2.5.1.10
(E,E)-Farnesyl-PP
3.1.7.6
Indole diterpene alkaloid biosynthesis
1.8.3.5
1.8.3.6
Geranyl geranyl-PP
Farnesyl cysteine
Steroid biosynthesis
2.5.1.29
PCME
2.5.1.82
2.5.1.89
1.3.1.83
2.5.1.85
2.5.1.84
3.4.24.84
FACE2
2.1.1.100
2.5.1.58
2.5.1.83
Protein C-terminal S-farnesyl-L-cysteine methyl ester
Protein C-terminal S-farnesyl-L-cysteine
S-farnesyl protein
Hexaprenyl-PP
tritrans,heptacis-Undecaprenyl-PP
Phytyl-PP
Nona-prenyl-PP
Carotenoid biosynthesis
2.5.1.30
Heptaprenyl-PP
N-Glycan biosynthesis
Dolichol-PP
Dehydro dolichol-PP
2.5.1.87
2.5.1.90
Octaprenyl-PP
2.5.1.31
2.5.1.91
di-trans,poly-cis-Undecaprenyl-PP
Decaprenyl-PP
Ubiquinone and other terpenoid-quinone biosynthesis
00900 4/19/17
(c) Kanehisa Laboratories

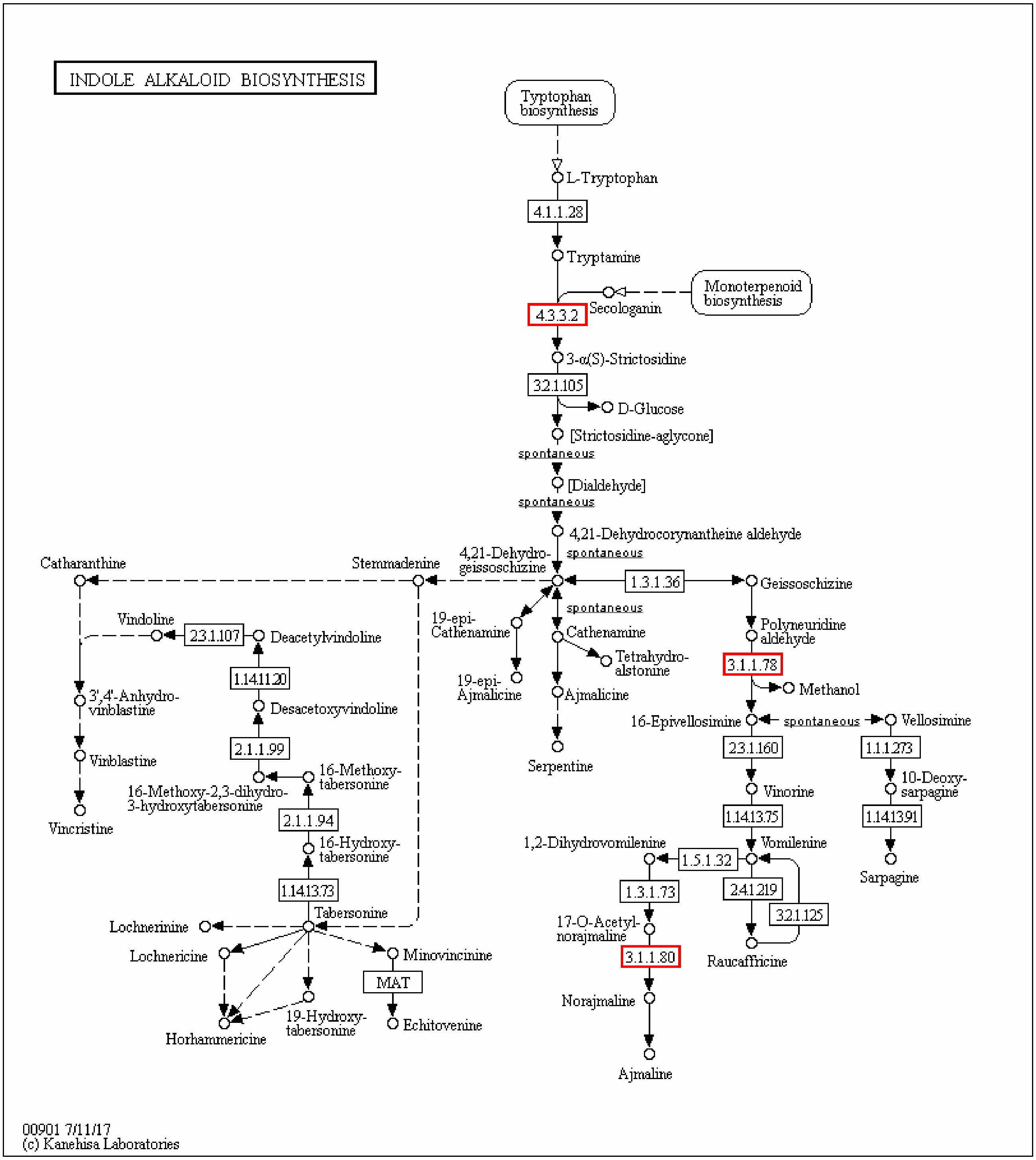

INDOLE ALKALOID BIOSYNTHESIS
Typtophan biosynthesis
L-Tryptophan
4.1.1.28
Tryptamine
Monoterpenoid biosynthesis
Secologanin
4.3.3.2
3-α(S)-Strictosidine
3.2.1.105
D-Glucose
[Strictosidine-aglycone]
spontaneous
[Dialdehyde]
spontaneous
4,21-Dehydrocorynantheine aldehyde
spontaneous
Catharanthine
Stemmadenine
4,21-Dehydro-geissoschizine
1.3.1.36
Geissoschizine
Vindoline
2.3.1.107
Deacetylvindoline
19-epi-Cathenamine
spontaneous
Cathenamine
Polyneuridine aldehyde
1.14.11.20
Tetrahydro-alstonine
3.1.1.78
3',4'-Anhydro-vinblastine
Desacetoxyvindoline
19-epi-Ajmalicine
Ajmalicine
Methanol
16-Epivellosimine
spontaneous
Vellosimine
2.1.1.99
Serpentine
2.3.1.160
1.1.1.273
Vinblastine
16-Methoxy-tabersonine
10-Deoxy-sarpagine
16-Methoxy-2,3-dihydro-3-hydroxytabersonine
Vinorine
Vincristine
2.1.1.94
1.14.13.75
1.14.13.91
16-Hydroxy-tabersonine
1,2-Dihydrovomilenine
Vomilenine
1.5.1.32
Sarpagine
1.14.13.73
1.3.1.73
2.4.1.219
3.2.1.125
Tabersonine
17-O-Acetyl-norajmaline
Lochnerinine
Lochnericine
Minovincinine
3.1.1.80
Raucaffricine
MAT
Norajmaline
19-Hydroxy-tabersonine
Echitovenine
Horhammericine
Ajmaline
00901 7/11/17
(c) Kanehisa Laboratories

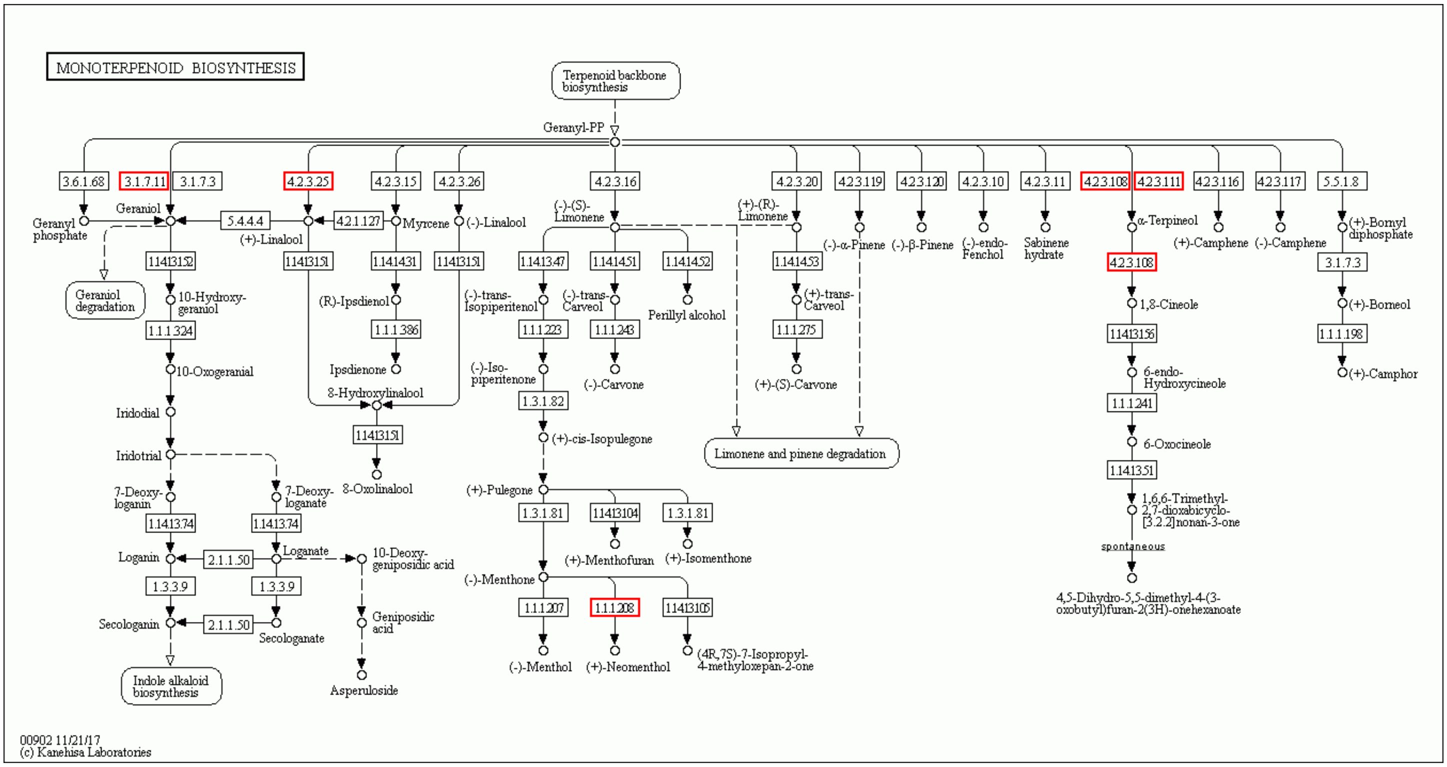
MONOTERPENOID BIOSYNTHESIS
Terpenoid backbone biosynthesis
Geranyl-PP
3.6.1.68
3.1.7.11
3.1.7.3
4.2.3.25
4.2.3.15
4.2.3.26
4.2.3.16
4.2.3.20
4.2.3.119
4.2.3.120
4.2.3.10
4.2.3.11
4.2.3.108
4.2.3.111
4.2.3.116
4.2.3.117
5.5.1.8
Geranyl phosphate
Geraniol
5.4.4.4
(+)-Linalool
4.2.1.127
Myrcene
(-)-Linalool
(-)-(S)-Limonene
(+)-(R)-Limonene
(-)-α-Pinene
(-)-β-Pinene
(-)-endo-Fenchol
Sabinene hydrate
α-Terpineol
(+)-Camphene
(-)-Camphene
(+)-Bornyl diphosphate
1.14.13.152
1.14.13.151
1.14.14.31
1.14.13.151
1.14.13.47
1.14.14.51
1.14.14.52
1.14.14.53
4.2.3.108
3.1.7.3
Geraniol degradation
10-Hydroxy-geraniol
(R)-Ipsdienol
(-)-trans-Isopiperitenol
(-)-trans-Carveol
Perillyl alcohol
(+)-trans-Carveol
1,8-Cineole
(+)-Borneol
1.1.1.324
1.1.1.386
1.1.1.223
1.1.1.243
1.1.1.275
1.14.13.156
1.1.1.198
10-Oxogeranial
Ipsdienone
(-)-Iso-piperitenone
(-)-Carvone
(+)-(S)-Carvone
6-endo-Hydroxycineole
(+)-Camphor
Iridodial
8-Hydroxylinalool
1.3.1.82
1.1.1.241
1.14.13.151
(+)-cis-Isopulegone
Limonene and pinene degradation
6-Oxocineole
Iridotrial
1.14.13.151
7-Deoxy-loganin
7-Deoxy-loganate
8-Oxolinalool
(+)-Pulegone
1,6,6-Trimethyl-2,7-dioxabicyclo-[3.2.2]nonan-3-one
1.14.13.74
1.14.13.74
1.3.1.81
1.14.13.104
1.3.1.81
spontaneous
Loganin
2.1.1.50
Loganate
10-Deoxy-geniposidic acid
(+)-Menthofuran
(+)-Isomenthone
(-)-Menthone
4,5-Dihydro-5,5-dimethyl-4-(3-oxobutyl)furan-2(3H)-onehexanoate
1.3.3.9
1.3.3.9
Geniposidic acid
1.1.1.207
1.1.1.208
1.14.13.105
Secologanin
2.1.1.50
Secologanate
(4R,7S)-7-Isopropyl-4-methyloxepan-2-one
(-)-Menthol
(+)-Neomenthol
Indole alkaloid biosynthesis
Asperuloside
00902 11/21/17
(c) Kanehisa Laboratories

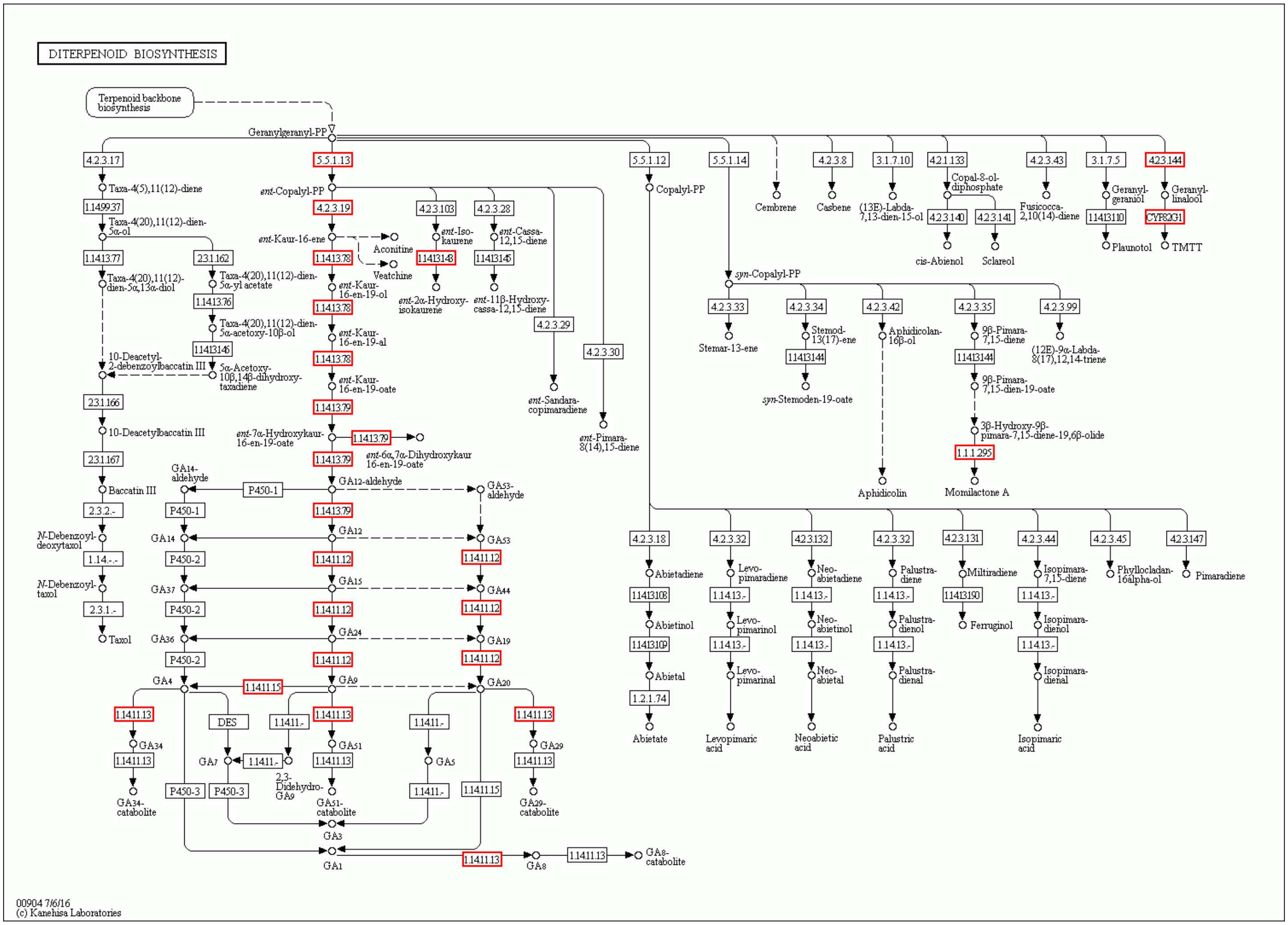
DITERPENOID BIOSYNTHESIS
Terpenoid backbone biosynthesis
Geranylgeranyl-PP
4.2.3.17
5.5.1.13
5.5.1.12
5.5.1.14
4.2.3.8
3.1.7.10
4.2.1.133
4.2.3.43
3.1.7.5
4.2.3.144
Taxa-4(5),11(12)-diene
ent-Copalyl-PP
Copalyl-PP
Cembrene
Casbene
(13E)-Labda-7,13-dien-15-ol
Copal-8-ol-diphosphate
Fusicocca-2,10(14)-diene
Geranyl-geraniol
Geranyl-linalool
1.14.99.37
4.2.3.19
4.2.3.103
4.2.3.28
4.2.3.140
4.2.3.141
1.14.13.110
CYP82G1
Taxa-4(20),11(12)-dien-5α-ol
ent-Kaur-16-ene
Aconitine
ent-Iso-kaurene
ent-Cassa-12,15-diene
cis-Abienol
Sclareol
Plaunotol
TMTT
1.14.13.77
2.3.1.162
1.14.13.78
Veatchine
1.14.13.143
1.14.13.145
syn-Copalyl-PP
Taxa-4(20),11(12)-dien-5α,13α-diol
Taxa-4(20),11(12)-dien-5α-yl acetate
ent-Kaur-16-en-19-ol
ent-2α-Hydroxy-isokaurene
ent-11β-Hydroxy-cassa-12,15-diene
4.2.3.33
4.2.3.34
4.2.3.42
4.2.3.35
4.2.3.99
1.14.13.76
1.14.13.78
4.2.3.29
Stemar-13-ene
Stemod-13(17)-ene
Aphidicolan-16β-ol
9β-Pimara-7,15-diene
(12E)-9α-Labda-8(17),12,14-triene
Taxa-4(20),11(12)-dien-5α-acetoxy-10β-ol
ent-Kaur-16-en-19-al
1.14.13.146
1.14.13.78
4.2.3.30
1.14.13.144
1.14.13.144
10-Deacetyl-2-debenzoylbaccatin III
5α-Acetoxy-10β,14β-dihydroxy-taxadiene
ent-Kaur-16-en-19-oate
syn-Stemoden-19-oate
9β-Pimara-7,15-dien-19-oate
2.3.1.166
1.14.13.79
ent-Sandara-copimaradiene
10-Deacetylbaccatin III
ent-7α-Hydroxykaur-16-en-19-oate
1.14.13.79
ent-Pimara-8(14),15-diene
3β-Hydroxy-9β-pimara-7,15-diene-19,6β-olide
2.3.1.167
GA14-aldehyde
1.14.13.79
ent-6α,7α-Dihydroxykaur-16-en-19-oate
1.1.1.295
Baccatin III
P450-1
GA12-aldehyde
GA53-aldehyde
Aphidicolin
Momilactone A
2.3.2.-
P450-1
1.14.13.79
N-Debenzoyl-deoxytaxol
GA14
GA12
GA53
4.2.3.18
4.2.3.32
4.2.3.132
4.2.3.32
4.2.3.131
4.2.3.44
4.2.3.45
4.2.3.147
1.14.-.-
P450-2
1.14.11.12
1.14.11.12
Abietadiene
Levo-pimaradiene
Neo-abietadiene
Palustra-diene
Miltiradiene
Isopimara-7,15-diene
Phyllocladan-16alpha-ol
Pimaradiene
N-Debenzoyl-taxol
GA37
GA15
GA44
1.14.13.108
1.14.13.-
1.14.13.-
1.14.13.-
1.14.13.190
1.14.13.-
2.3.1.-
P450-2
1.14.11.12
1.14.11.12
Abietinol
Levo-pimarinol
Neo-abietinol
Palustra-dienol
Ferruginol
Isopimara-dienol
Taxol
GA36
GA24
GA19
1.14.13.109
1.14.13.-
1.14.13.-
1.14.13.-
1.14.13.-
P450-2
1.14.11.12
1.14.11.12
Abietal
Levo-pimarinal
Neo-abietal
Palustra-dienal
Isopimara-dienal
GA4
1.14.11.15
GA9
GA20
1.2.1.74
1.14.11.13
DES
1.14.11.-
1.14.11.13
1.14.11.-
1.14.11.13
Abietate
Levopimaric acid
Neoabietic acid
Palustric acid
Isopimaric acid
GA34
GA51
GA5
GA29
1.14.11.13
GA7
1.14.11.-
1.14.11.13
1.14.11.13
2,3-Didehydro-GA9
GA34-catabolite
P450-3
P450-3
GA51-catabolite
1.14.11.-
1.14.11.15
GA29-catabolite
GA3
GA1
1.14.11.13
GA8
1.14.11.13
GA8-catabolite
00904 7/6/16
(c) Kanehisa Laboratories

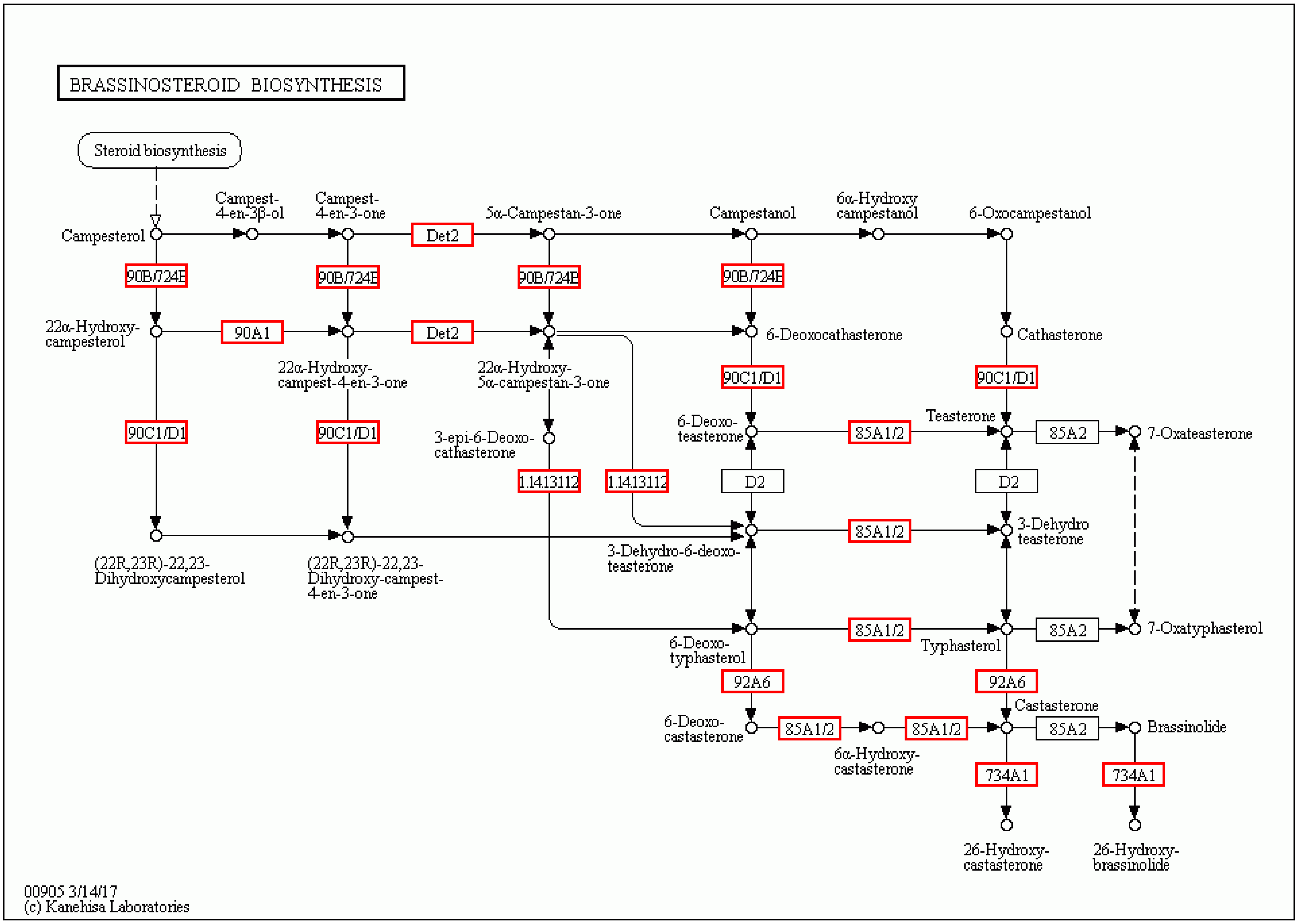

BRASSINOSTEROID BIOSYNTHESIS
Steroid biosynthesis
Campesterol
Campest-4-en-3β-ol
Campest-4-en-3-one
Det2
5α-Campestan-3-one
Campestanol
6α-Hydroxy campestanol
6-Oxocampestanol
90B/724B
22α-Hydroxy-campesterol
90A1
22α-Hydroxy-campest-4-en-3-one
22α-Hydroxy-5α-campestan-3-one
6-Deoxocathasterone
Cathasterone
90C1/D1
3-epi-6-Deoxo-cathasterone
1.14.13.112
6-Deoxo-teasterone
85A1/2
Teasterone
85A2
7-Oxateasterone
D2
(22R,23R)-22,23-Dihydroxycampesterol
(22R,23R)-22,23-Dihydroxy-campest-4-en-3-one
3-Dehydro-6-deoxo-teasterone
3-Dehydro teasterone
6-Deoxo-typhasterol
Typhasterol
7-Oxatyphasterol
92A6
6-Deoxo-castasterone
6α-Hydroxy-castasterone
Castasterone
Brassinolide
734A1
26-Hydroxy-castasterone
26-Hydroxy-brassinolide
00905 3/14/17
(c) Kanehisa Laboratories

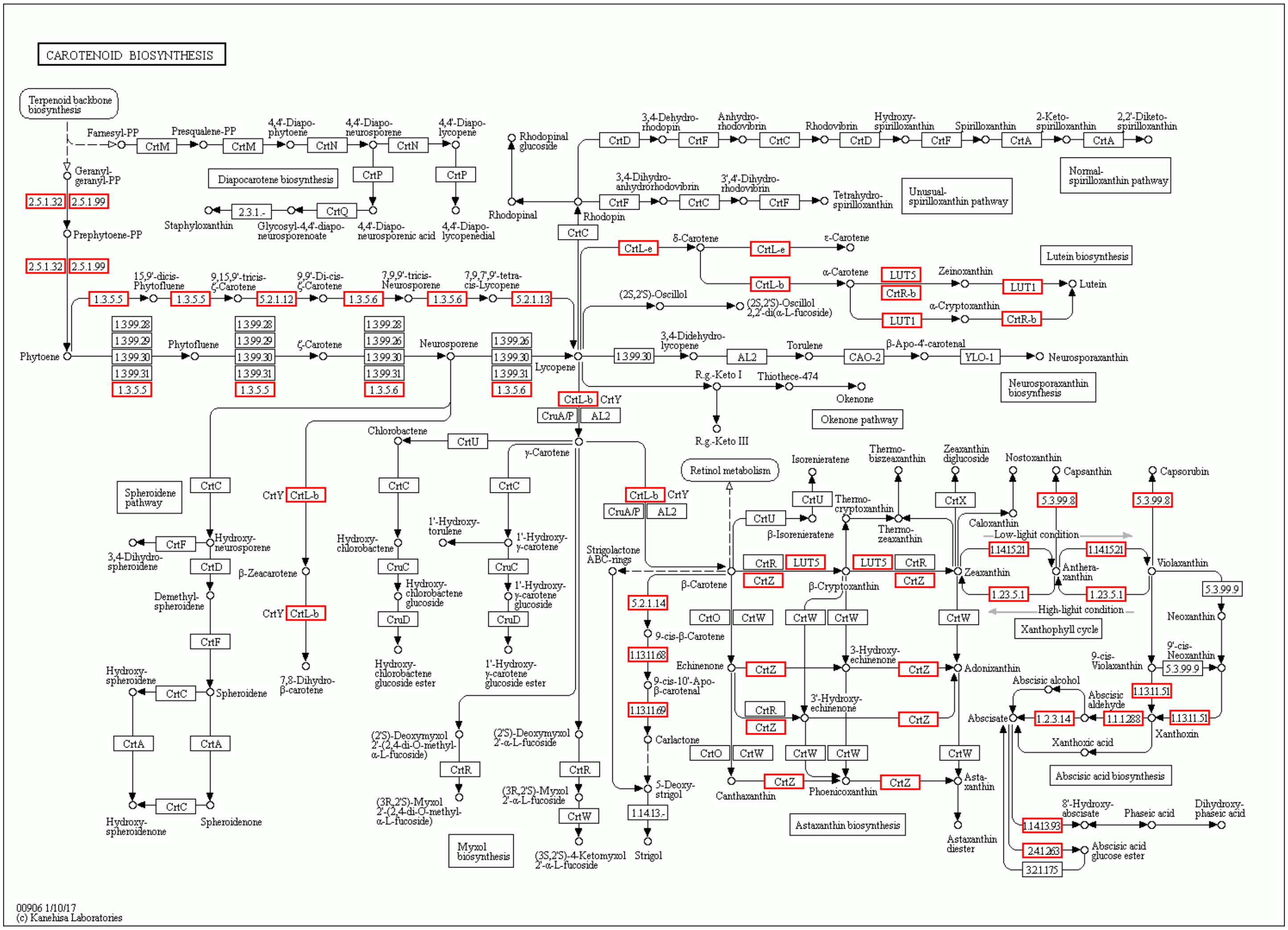
CAROTENOID BIOSYNTHESIS
Terpenoid backbone biosynthesis
Farnesyl-PP
CrtM
Presqualene-PP
CrtM
4,4'-Diapo-phytoene
CrtN
4,4'-Diapo-neurosporene
CrtN
4,4'-Diapo-lycopene
Geranyl-geranyl-PP
Diapocarotene biosynthesis
CrtP
CrtP
2.5.1.32
2.5.1.99
2.3.1.-
CrtQ
Staphyloxanthin
Glycosyl-4,4'-diapo-neurosporenoate
4,4'-Diapo-neurosporenic acid
4,4'-Diapo-lycopenedial
Prephytoene-PP
2.5.1.32
2.5.1.99
Rhodopinal glucoside
Rhodopinal
Rhodopin
CrtC
CrtD
3,4-Dehydro-rhodopin
CrtF
Anhydro-rhodovibrin
CrtC
Rhodovibrin
CrtD
Hydroxy-spirilloxanthin
CrtF
Spirilloxanthin
CrtA
2-Keto-spirilloxanthin
CrtA
2,2'-Diketo-spirilloxanthin
Normal-spirilloxanthin pathway
CrtF
3,4-Dihydro-anhydrorhodovibrin
CrtC
3',4'-Dihydro-rhodovibrin
CrtF
Tetrahydro-spirilloxanthin
Unusual-spirilloxanthin pathway
15,9'-dicis-Phytofluene
9,15,9'-tricis-ζ-Carotene
9,9'-Di-cis-ζ-Carotene
7,9,9'-tricis-Neurosporene
7,9,7',9'-tetra-cis-Lycopene
1.3.5.5
1.3.5.5
5.2.1.12
1.3.5.6
1.3.5.6
5.2.1.13
CrtL-e
δ-Carotene
CrtL-e
ε-Carotene
CrtL-b
α-Carotene
LUT5
CrtR-b
Zeinoxanthin
LUT1
Lutein
LUT1
α-Cryptoxanthin
CrtR-b
Lutein biosynthesis
(2S,2'S)-Oscillol
(2S,2'S)-Oscillol 2,2'-di(α-L-fucoside)
Phytoene
1.3.99.28
1.3.99.29
1.3.99.30
1.3.99.31
1.3.5.5
Phytofluene
ζ-Carotene
Neurosporene
1.3.99.26
1.3.99.30
1.3.99.31
1.3.5.6
Lycopene
1.3.99.30
3,4-Didehydro-lycopene
AL2
Torulene
CAO-2
β-Apo-4'-carotenal
YLO-1
Neurosporaxanthin
Neurosporaxanthin biosynthesis
R.g.-Keto I
Thiothece-474
Okenone
Okenone pathway
R.g.-Keto III
CrtL-b
CrtY
CruA/P
AL2
Chlorobactene
CrtU
γ-Carotene
Spheroidene pathway
CrtC
CrtY
CrtL-b
CrtC
CrtC
CrtF
Hydroxy-neurosporene
3,4-Dihydro-spheroidene
CrtD
β-Zeacarotene
1'-Hydroxy-torulene
Hydroxy-chlorobactene
1'-Hydroxy-γ-carotene
CruC
CruC
Demethyl-spheroidene
CrtY
CrtL-b
Hydroxy-chlorobactene glucoside
1'-Hydroxy-γ-carotene glucoside
CruD
CruD
CrtF
7,8-Dihydro-β-carotene
Hydroxy-chlorobactene glucoside ester
1'-Hydroxy-γ-carotene glucoside ester
Hydroxy-spheroidene
CrtC
Spheroidene
CrtA
CrtA
CrtC
Hydroxy-spheroidenone
Spheroidenone
(2'S)-Deoxymyxol 2'-(2,4-di-O-methyl-α-L-fucoside)
(2'S)-Deoxymyxol 2'-α-L-fucoside
CrtR
CrtR
(3R,2'S)-Myxol 2'-(2,4-di-O-methyl-α-L-fucoside)
(3R,2'S)-Myxol 2'-α-L-fucoside
CrtW
Myxol biosynthesis
(3S,2'S)-4-Ketomyxol 2'-α-L-fucoside
Retinol metabolism
CrtL-b
CrtY
CruA/P
AL2
Strigolactone ABC-rings
β-Carotene
5.2.1.14
9-cis-β-Carotene
1.13.11.68
9-cis-10'-Apo-β-carotenal
1.13.11.69
Carlactone
5-Deoxy-strigol
1.14.13.-
Strigol
Isorenieratene
CrtU
CrtU
β-Isorenieratene
Thermo-biszeaxanthin
Thermo-cryptoxanthin
Thermo-zeaxanthin
Zeaxanthin diglucoside
CrtX
Caloxanthin
Nostoxanthin
CrtR
LUT5
LUT5
CrtR
CrtZ
CrtZ
β-Cryptoxanthin
Zeaxanthin
CrtO
CrtW
CrtW
CrtW
CrtW
Echinenone
CrtZ
3-Hydroxy-echinenone
CrtZ
Adonixanthin
CrtR
CrtZ
3'-Hydroxy-echinenone
CrtZ
CrtO
CrtW
CrtW
CrtW
CrtW
CrtZ
CrtZ
Canthaxanthin
Phoenicoxanthin
Astaxanthin
Astaxanthin biosynthesis
Astaxanthin diester
Capsanthin
Capsorubin
5.3.99.8
5.3.99.8
Low-light condition
1.14.15.21
1.14.15.21
Antheraxanthin
Violaxanthin
1.23.5.1
1.23.5.1
High-light condition
Xanthophyll cycle
5.3.99.9
Neoxanthin
9-cis-Violaxanthin
9'-cis-Neoxanthin
5.3.99.9
Abscisic alcohol
1.13.11.51
Abscisate
1.2.3.14
Abscisic aldehyde
1.1.1.288
1.13.11.51
Xanthoxin
Xanthoxic acid
Abscisic acid biosynthesis
8'-Hydroxy-abscisate
Phaseic acid
Dihydroxy-phaseic acid
1.14.13.93
2.4.1.263
Abscisic acid glucose ester
3.2.1.175
00906 1/10/17
(c) Kanehisa Laboratories

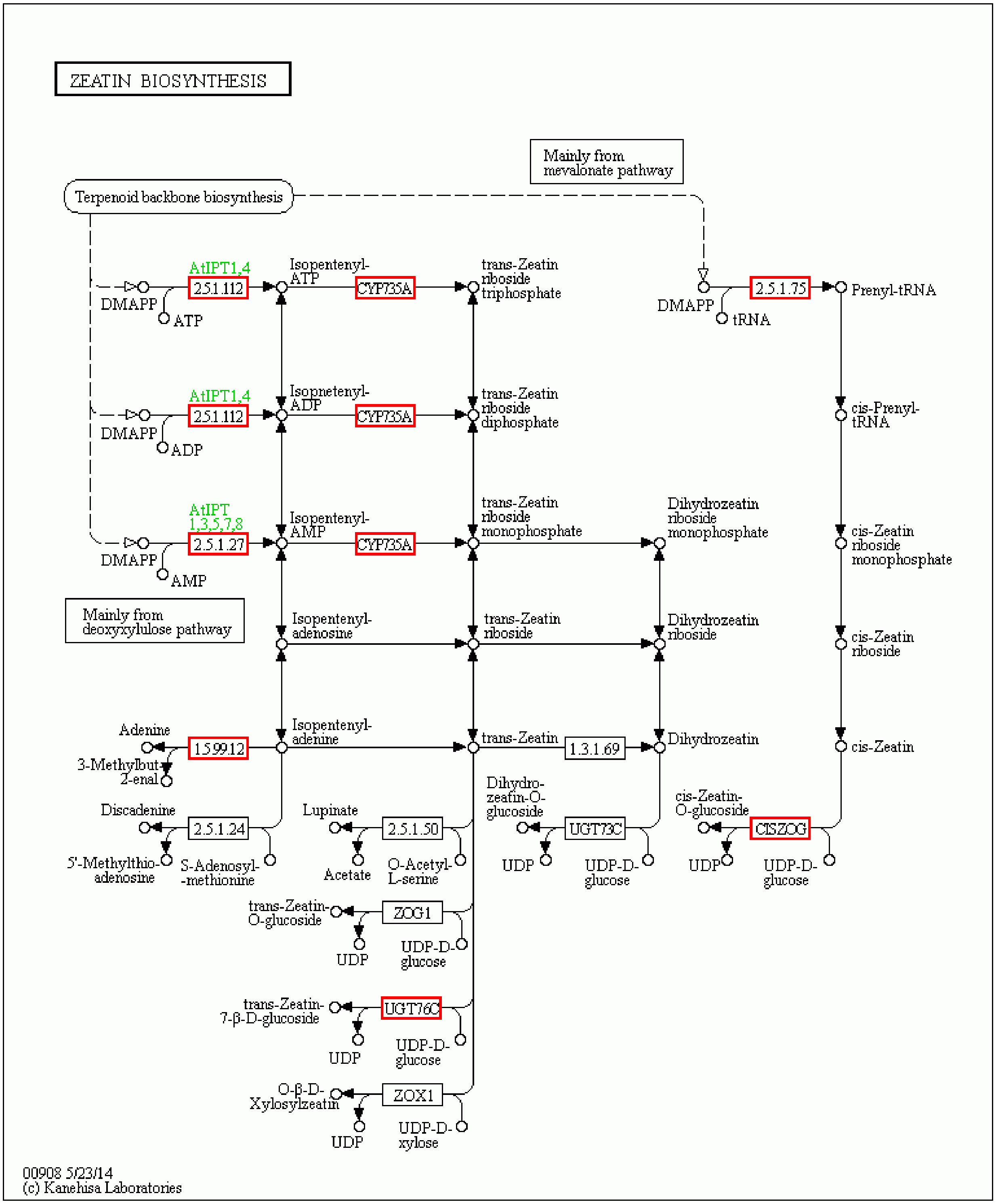

ZEATIN BIOSYNTHESIS
Mainly from mevalonate pathway
Terpenoid backbone biosynthesis
AtIPT1,4
2.5.1.112
DMAPP
ATP
Isopentenyl-ATP
CYP735A
trans-Zeatin riboside triphosphate
DMAPP
2.5.1.75
tRNA
Prenyl-tRNA
AtIPT1,4
2.5.1.112
DMAPP
ADP
Isopnetenyl-ADP
CYP735A
trans-Zeatin riboside diphosphate
cis-Prenyl-tRNA
AtIPT 1,3,5,7,8
2.5.1.27
DMAPP
AMP
Isopentenyl-AMP
CYP735A
trans-Zeatin riboside monophosphate
Dihydrozeatin riboside monophosphate
cis-Zeatin riboside monophosphate
Mainly from deoxyxylulose pathway
Isopentenyl-adenosine
trans-Zeatin riboside
Dihydrozeatin riboside
cis-Zeatin riboside
Adenine
1.5.99.12
3-Methylbut-2-enal
Isopentenyl-adenine
trans-Zeatin
1.3.1.69
Dihydrozeatin
cis-Zeatin
Discadenine
2.5.1.24
5'-Methylthio-adenosine
S-Adenosyl--methionine
Lupinate
2.5.1.50
Acetate
O-Acetyl-L-serine
Dihydro-zeatin-O-glucoside
UGT73C
UDP
UDP-D-glucose
cis-Zeatin-O-glucoside
CISZOG
UDP
UDP-D-glucose
trans-Zeatin-O-glucoside
ZOG1
UDP
UDP-D-glucose
trans-Zeatin-7-β-D-glucoside
UGT76C
UDP
UDP-D-glucose
O-β-D-Xylosylzeatin
ZOX1
UDP
UDP-D-xylose
00908 5/23/14
(c) Kanehisa Laboratories

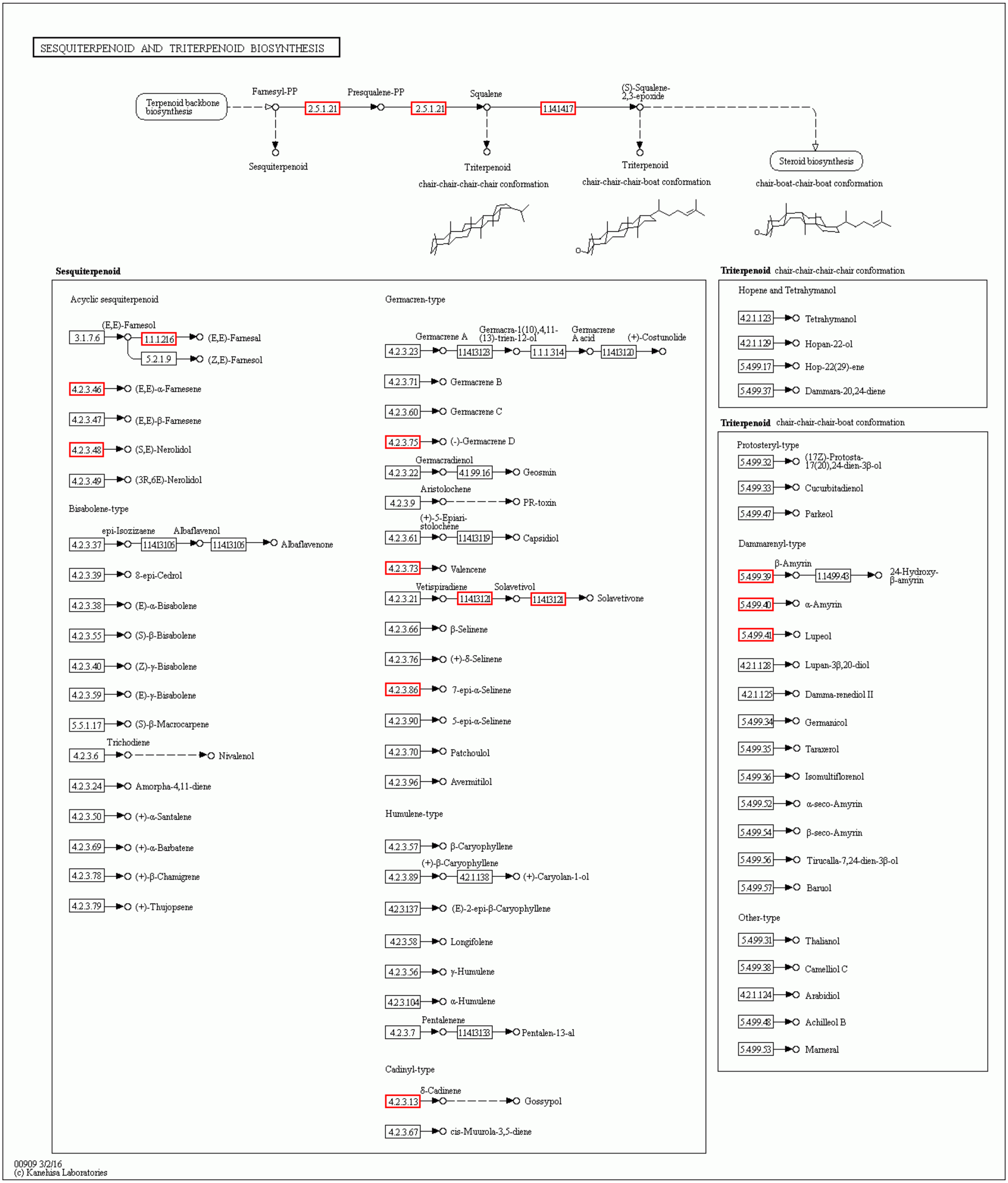

SESQUITERPENOID AND TRITERPENOID BIOSYNTHESIS
Terpenoid backbone biosynthesis
Farnesyl-PP
2.5.1.21
Presqualene-PP
2.5.1.21
Squalene
1.14.14.17
(S)-Squalene-2,3-epoxide
Sesquiterpenoid
Triterpenoid
chair-chair-chair-chair conformation
Triterpenoid
chair-chair-chair-boat conformation
Steroid biosynthesis
chair-boat-chair-boat conformation
Sesquiterpenoid
Acyclic sesquiterpenoid
(E,E)-Farnesol
3.1.7.6
1.1.1.216
(E,E)-Farnesal
5.2.1.9
(Z,E)-Farnesol
4.2.3.46
(E,E)-α-Farnesene
4.2.3.47
(E,E)-β-Farnesene
4.2.3.48
(S,E)-Nerolidol
4.2.3.49
(3R,6E)-Nerolidol
Bisabolene-type
epi-Isozizaene
Albaflavenol
4.2.3.37
1.14.13.106
1.14.13.106
Albaflavenone
4.2.3.39
8-epi-Cedrol
4.2.3.38
(E)-α-Bisabolene
4.2.3.55
(S)-β-Bisabolene
4.2.3.40
(Z)-γ-Bisabolene
4.2.3.59
(E)-γ-Bisabolene
5.5.1.17
(S)-β-Macrocarpene
Trichodiene
4.2.3.6
Nivalenol
4.2.3.24
Amorpha-4,11-diene
4.2.3.50
(+)-α-Santalene
4.2.3.69
(+)-α-Barbatene
4.2.3.78
(+)-β-Chamigrene
4.2.3.79
(+)-Thujopsene
Germacren-type
Germacrene A
Germacra-1(10),4,11-(13)-trien-12-ol
Germacrene A acid
(+)-Costunolide
4.2.3.23
1.14.13.123
1.1.1.314
1.14.13.120
4.2.3.71
Germacrene B
4.2.3.60
Germacrene C
4.2.3.75
(-)-Germacrene D
Germacradienol
4.2.3.22
4.1.99.16
Geosmin
Aristolochene
4.2.3.9
PR-toxin
(+)-5-Epiari-stolochene
4.2.3.61
1.14.13.119
Capsidiol
4.2.3.73
Valencene
Vetispiradiene
Solavetivol
4.2.3.21
1.14.13.121
1.14.13.121
Solavetivone
4.2.3.66
β-Selinene
4.2.3.76
(+)-δ-Selinene
4.2.3.86
7-epi-α-Selinene
4.2.3.90
5-epi-α-Selinene
4.2.3.70
Patchoulol
4.2.3.96
Avermitilol
Humulene-type
4.2.3.57
β-Caryophyllene
(+)-β-Caryophyllene
4.2.3.89
4.2.1.138
(+)-Caryolan-1-ol
4.2.3.137
(E)-2-epi-β-Caryophyllene
4.2.3.58
Longifolene
4.2.3.56
γ-Humulene
4.2.3.104
α-Humulene
Pentalenene
4.2.3.7
1.14.13.133
Pentalen-13-al
Cadinyl-type
δ-Cadinene
4.2.3.13
Gossypol
4.2.3.67
cis-Muurola-3,5-diene
Triterpenoid chair-chair-chair-chair conformation
Hopene and Tetrahymanol
4.2.1.123
Tetrahymanol
4.2.1.129
Hopan-22-ol
5.4.99.17
Hop-22(29)-ene
5.4.99.37
Dammara-20,24-diene
Triterpenoid chair-chair-chair-boat conformation
Protosteryl-type
5.4.99.32
(17Z)-Protosta-17(20),24-dien-3β-ol
5.4.99.33
Cucurbitadienol
5.4.99.47
Parkeol
Dammarenyl-type
β-Amyrin
5.4.99.39
1.14.99.43
24-Hydroxy-β-amyrin
5.4.99.40
α-Amyrin
5.4.99.41
Lupeol
4.2.1.128
Lupan-3β,20-diol
4.2.1.125
Damma-renediol II
5.4.99.34
Germanicol
5.4.99.35
Taraxerol
5.4.99.36
Isomultiflorenol
5.4.99.52
α-seco-Amyrin
5.4.99.54
β-seco-Amyrin
5.4.99.56
Tirucalla-7,24-dien-3β-ol
5.4.99.57
Baruol
Other-type
5.4.99.31
Thalianol
5.4.99.38
Camelliol C
4.2.1.124
Arabidiol
5.4.99.48
Achilleol B
5.4.99.53
Marneral
00909 3/2/16
(c) Kanehisa Laboratories

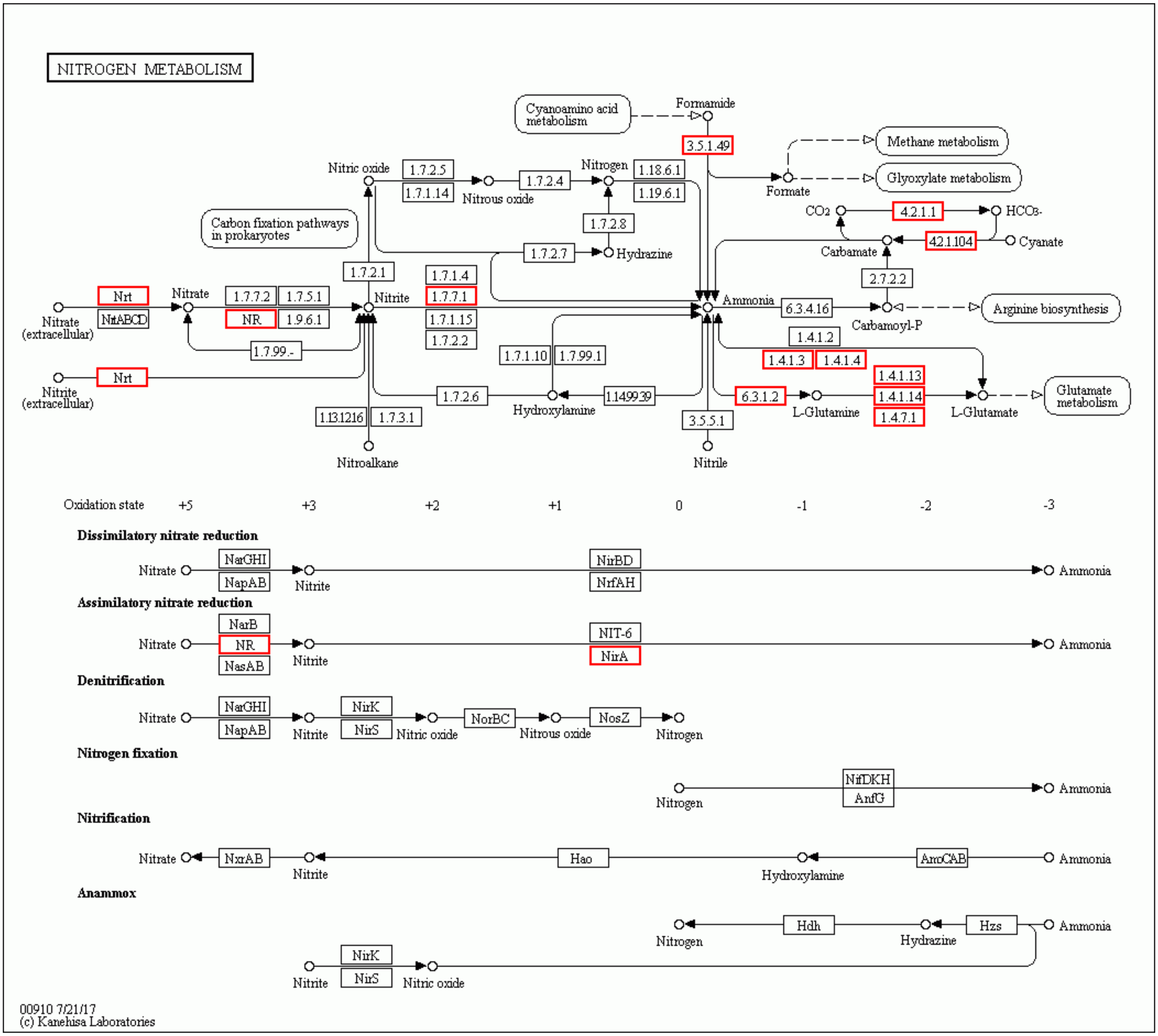

NITROGEN METABOLISM
Cyanoamino acid metabolism
Formamide
3.5.1.49
Methane metabolism
Glyoxylate metabolism
Formate
Nitric oxide
1.7.2.5
1.7.1.14
Nitrous oxide
1.7.2.4
Nitrogen
1.18.6.1
1.19.6.1
1.7.2.8
CO2
4.2.1.1
HCO3-
Carbon fixation pathways in prokaryotes
1.7.2.7
Hydrazine
Carbamate
4.2.1.104
Cyanate
1.7.2.1
1.7.1.4
2.7.2.2
Nrt
Nitrate
1.7.7.2
1.7.5.1
Nitrite
1.7.7.1
Ammonia
6.3.4.16
Carbamoyl-P
Arginine biosynthesis
Nitrate (extracellular)
NrtABCD
NR
1.9.6.1
1.7.1.15
1.4.1.2
1.7.99.-
1.7.2.2
1.7.1.10
1.7.99.1
1.4.1.3
1.4.1.4
Nitrite (extracellular)
Nrt
1.4.1.13
1.7.2.6
Hydroxylamine
1.14.99.39
6.3.1.2
L-Glutamine
1.4.1.14
L-Glutamate
Glutamate metabolism
1.13.12.16
1.7.3.1
3.5.5.1
1.4.7.1
Nitroalkane
Nitrile
Oxidation state
+5
+3
+2
+1
0
-1
-2
-3
Dissimilatory nitrate reduction
Nitrate
NarGHI
NapAB
Nitrite
NirBD
NrfAH
Ammonia
Assimilatory nitrate reduction
Nitrate
NarB
NR
NasAB
Nitrite
NIT-6
NirA
Ammonia
Denitrification
Nitrate
NarGHI
NapAB
Nitrite
NirK
NirS
Nitric oxide
NorBC
Nitrous oxide
NosZ
Nitrogen
Nitrogen fixation
Nitrogen
NifDKH
AnfG
Ammonia
Nitrification
Nitrate
NxrAB
Nitrite
Hao
Hydroxylamine
AmoCAB
Ammonia
Anammox
Nitrogen
Hdh
Hydrazine
Hzs
Ammonia
Nitrite
NirK
NirS
Nitric oxide
00910 7/21/17
(c) Kanehisa Laboratories

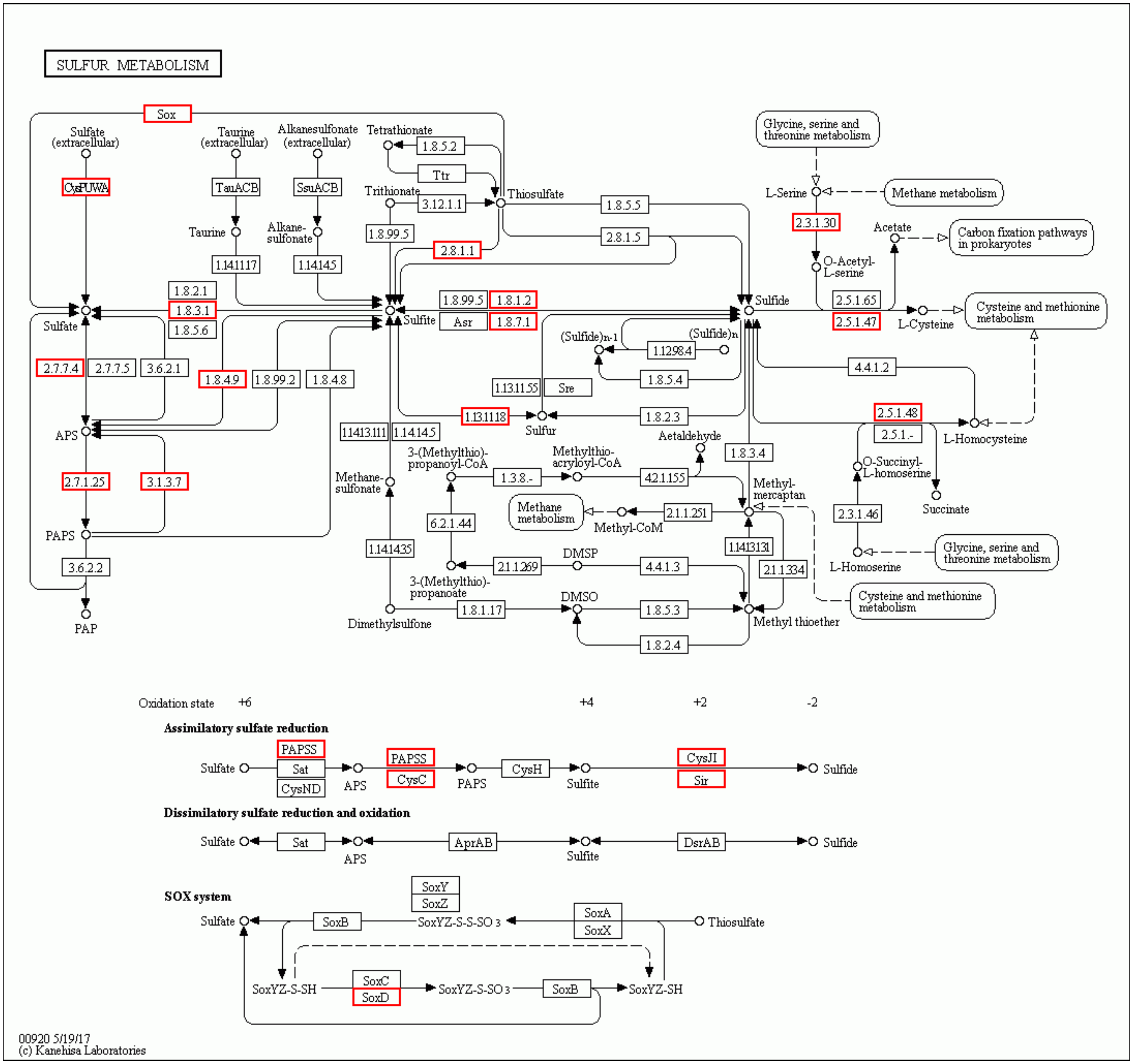
SULFUR METABOLISM
Sox
Sulfate (extracellular)
CysPUWA
Taurine (extracellular)
TauACB
Alkanesulfonate (extracellular)
SsuACB
Tetrathionate
1.8.5.2
Ttr
Trithionate
3.12.1.1
Thiosulfate
1.8.5.5
2.8.1.5
1.8.99.5
2.8.1.1
Taurine
Alkane-sulfonate
1.14.11.17
1.14.14.5
1.8.2.1
1.8.3.1
1.8.5.6
Sulfate
1.8.99.5
1.8.1.2
Asr
1.8.7.1
Sulfite
Sulfide
2.7.7.4
2.7.7.5
3.6.2.1
1.8.4.9
1.8.99.2
1.8.4.8
(Sulfide)n-1
(Sulfide)n
1.12.98.4
1.8.5.4
1.13.11.55
Sre
1.13.11.18
Sulfur
1.8.2.3
APS
1.14.13.111
1.14.14.5
3-(Methylthio)-propanoyl-CoA
Methylthio-acryloyl-CoA
Aetaldehyde
1.8.3.4
1.3.8.-
4.2.1.155
Methane-sulfonate
Methyl-mercaptan
2.7.1.25
3.1.3.7
6.2.1.44
Methane metabolism
2.1.1.251
Methyl-CoM
PAPS
1.14.14.35
DMSP
4.4.1.3
1.14.13.131
2.1.1.269
2.1.1.334
3.6.2.2
3-(Methylthio)-propanoate
PAP
Dimethylsulfone
1.8.1.17
DMSO
1.8.5.3
Methyl thioether
1.8.2.4
Glycine, serine and threonine metabolism
L-Serine
Methane metabolism
2.3.1.30
Acetate
Carbon fixation pathways in prokaryotes
O-Acetyl-L-serine
2.5.1.65
2.5.1.47
L-Cysteine
Cysteine and methionine metabolism
4.4.1.2
2.5.1.48
2.5.1.-
L-Homocysteine
O-Succinyl-L-homoserine
Succinate
2.3.1.46
L-Homoserine
Glycine, serine and threonine metabolism
Cysteine and methionine metabolism
Oxidation state +6 +4 +2 -2
Assimilatory sulfate reduction
Sulfate
PAPSS
Sat
CysND
APS
PAPSS
CysC
PAPS
CysH
Sulfite
CysJI
Sir
Sulfide
Dissimilatory sulfate reduction and oxidation
Sulfate
Sat
APS
AprAB
Sulfite
DsrAB
Sulfide
SOX system
Sulfate
SoxB
SoxY
SoxZ
SoxYZ-S-S-SO3
SoxA
SoxX
Thiosulfate
SoxYZ-S-SH
SoxC
SoxD
SoxYZ-S-SO3
SoxB
SoxYZ-SH
00920 5/19/17
(c) Kanehisa Laboratories

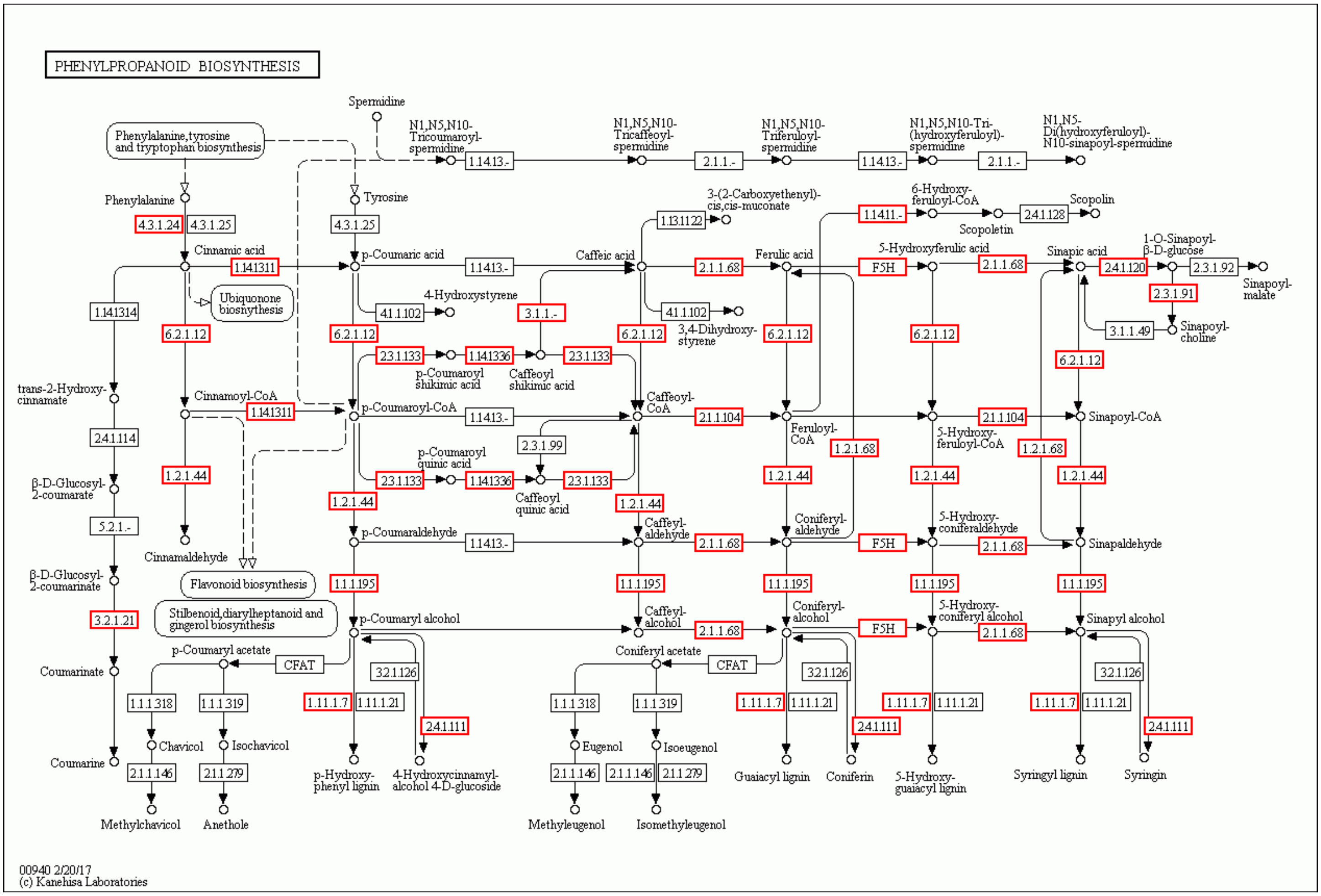
PHENYLPROPANOID BIOSYNTHESIS
Spermidine
Phenylalanine,tyrosine and tryptophan biosynthesis
N1,N5,N10-Tricoumaroyl-spermidine
N1,N5,N10-Tricaffeoyl-spermidine
N1,N5,N10-Triferuloyl-spermidine
N1,N5,N10-Tri-(hydroxyferuloyl)-spermidine
N1,N5-Di(hydroxyferuloyl)-N10-sinapoyl-spermidine
Phenylalanine
Tyrosine
3-(2-Carboxyethenyl)-cis,cis-muconate
6-Hydroxy-feruloyl-CoA
Scopoletin
Scopolin
Cinnamic acid
p-Coumaric acid
Caffeic acid
Ferulic acid
5-Hydroxyferulic acid
Sinapic acid
1-O-Sinapoyl-β-D-glucose
Sinapoyl-malate
Ubiquonone biosynthesis
4-Hydroxystyrene
3,4-Dihydroxy-styrene
Sinapoyl-choline
trans-2-Hydroxy-cinnamate
p-Coumaroyl shikimic acid
Caffeoyl shikimic acid
Cinnamoyl-CoA
p-Coumaroyl-CoA
Caffeoyl-CoA
Feruloyl-CoA
5-Hydroxy-feruloyl-CoA
Sinapoyl-CoA
β-D-Glucosyl-2-coumarate
p-Coumaroyl quinic acid
Caffeoyl quinic acid
p-Coumaraldehyde
Caffeyl-aldehyde
Coniferyl-aldehyde
5-Hydroxy-coniferaldehyde
Sinapaldehyde
Cinnamaldehyde
β-D-Glucosyl-2-coumarinate
Flavonoid biosynthesis
Stilbenoid,diarylheptanoid and gingerol biosynthesis
p-Coumaryl alcohol
Caffeyl-alcohol
Coniferyl-alcohol
5-Hydroxy-coniferyl alcohol
Sinapyl alcohol
p-Coumaryl acetate
Coniferyl acetate
Coumarinate
Chavicol
Isochavicol
Eugenol
Isoeugenol
Coumarine
p-Hydroxy-phenyl lignin
4-Hydroxycinnamyl-alcohol 4-D-glucoside
Guaiacyl lignin
Coniferin
5-Hydroxy-guaiacyl lignin
Syringyl lignin
Syringin
Methylchavicol
Anethole
Methyleugenol
Isomethyleugenol
4.3.1.24
4.3.1.25
1.14.13.11
1.14.13.14
6.2.1.12
1.2.1.44
2.4.1.114
5.2.1.-
3.2.1.21
1.14.13.-
2.1.1.-
1.13.11.22
1.14.11.-
2.4.1.128
2.1.1.68
2.4.1.120
2.3.1.92
2.3.1.91
3.1.1.49
F5H
3.1.1.-
4.1.1.102
2.3.1.133
1.14.13.36
2.1.1.104
2.3.1.99
1.1.1.195
CFAT
3.2.1.126
1.11.1.7
1.11.1.21
2.4.1.111
1.1.1.318
1.1.1.319
2.1.1.146
2.1.1.279
00940 2/20/17
(c) Kanehisa Laboratories

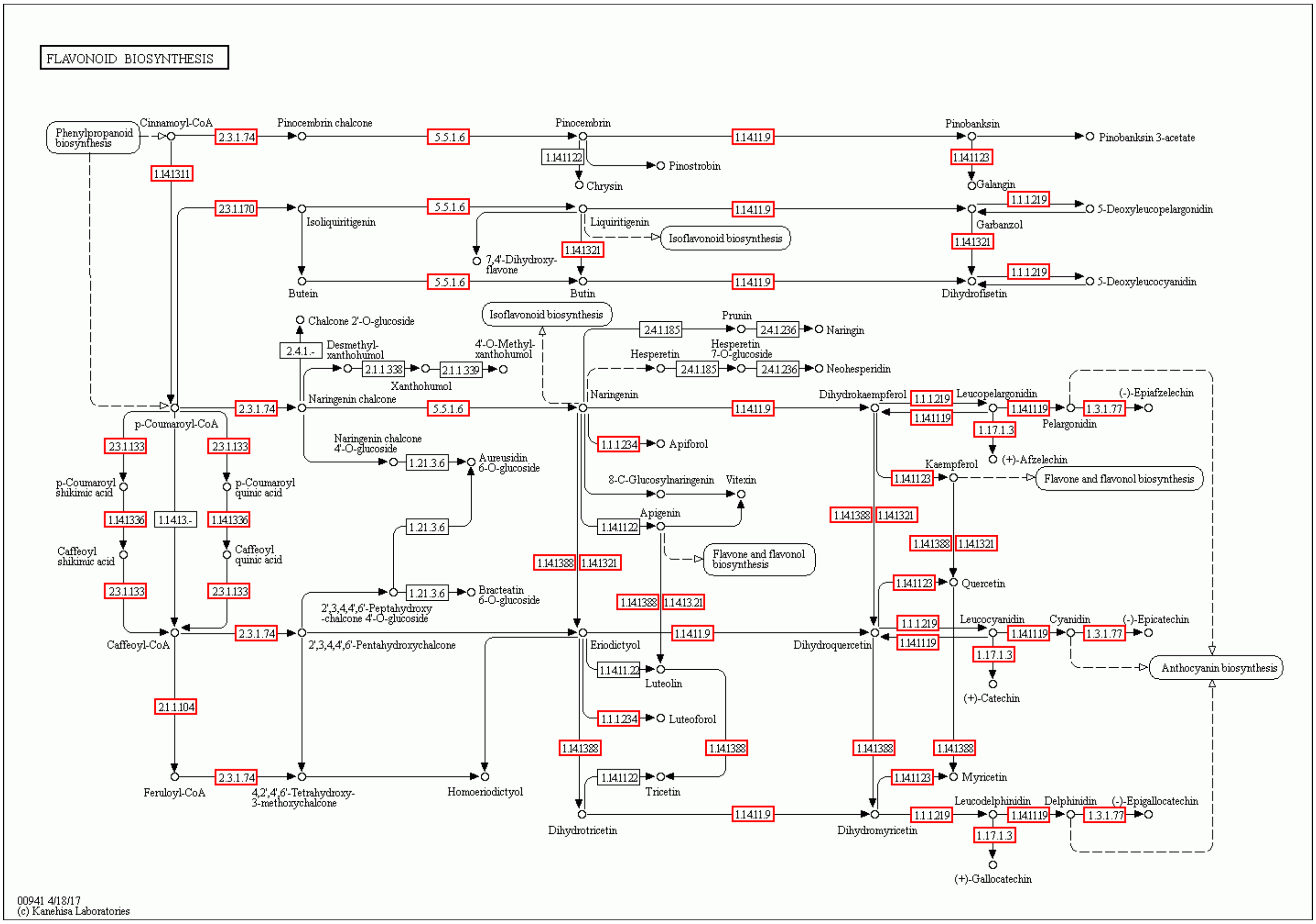
FLAVONOID BIOSYNTHESIS
Phenylpropanoid biosynthesis
Cinnamoyl-CoA
2.3.1.74
Pinocembrin chalcone
5.5.1.6
Pinocembrin
1.14.11.9
Pinobanksin
Pinobanksin 3-acetate
1.14.11.22
Pinostrobin
Chrysin
1.14.11.23
Galangin
1.14.13.11
2.3.1.170
Isoliquiritigenin
Liquiritigenin
1.1.1.219
5-Deoxyleucopelargonidin
Garbanzol
Isoflavonoid biosynthesis
7,4'-Dihydroxy-flavone
1.14.13.21
Butein
Butin
Dihydrofisetin
5-Deoxyleucocyanidin
Chalcone 2'-O-glucoside
Prunin
2.4.1.185
2.4.1.236
Naringin
2.4.1.-
Desmethyl-xanthohumol
4'-O-Methyl-xanthohumol
2.1.1.338
2.1.1.339
Xanthohumol
Hesperetin
Hesperetin 7-O-glucoside
Neohesperidin
Naringenin chalcone
Naringenin
Dihydrokaempferol
Leucopelargonidin
1.14.11.19
Pelargonidin
1.3.1.77
(-)-Epiafzelechin
1.17.1.3
(+)-Afzelechin
p-Coumaroyl-CoA
2.3.1.133
Naringenin chalcone 4'-O-glucoside
1.21.3.6
Aureusidin 6-O-glucoside
1.1.1.234
Apiforol
Kaempferol
Flavone and flavonol biosynthesis
p-Coumaroyl shikimic acid
p-Coumaroyl quinic acid
8-C-Glucosylnaringenin
Vitexin
Apigenin
1.14.13.36
1.14.13.-
1.14.13.88
Caffeoyl shikimic acid
Caffeoyl quinic acid
Quercetin
Bracteatin 6-O-glucoside
2',3,4,4',6'-Peptahydroxy-chalcone 4'-O-glucoside
Caffeoyl-CoA
2',3,4,4',6'-Pentahydroxychalcone
Eriodictyol
Dihydroquercetin
Leucocyanidin
Cyanidin
(-)-Epicatechin
Luteolin
Anthocyanin biosynthesis
(+)-Catechin
2.1.1.104
Luteoforol
Myricetin
Feruloyl-CoA
4,2',4',6'-Tetrahydroxy-3-methoxychalcone
Homoeriodictyol
Tricetin
Leucodelphinidin
Delphinidin
(-)-Epigallocatechin
Dihydrotricetin
Dihydromyricetin
(+)-Gallocatechin
00941 4/18/17
(c) Kanehisa Laboratories

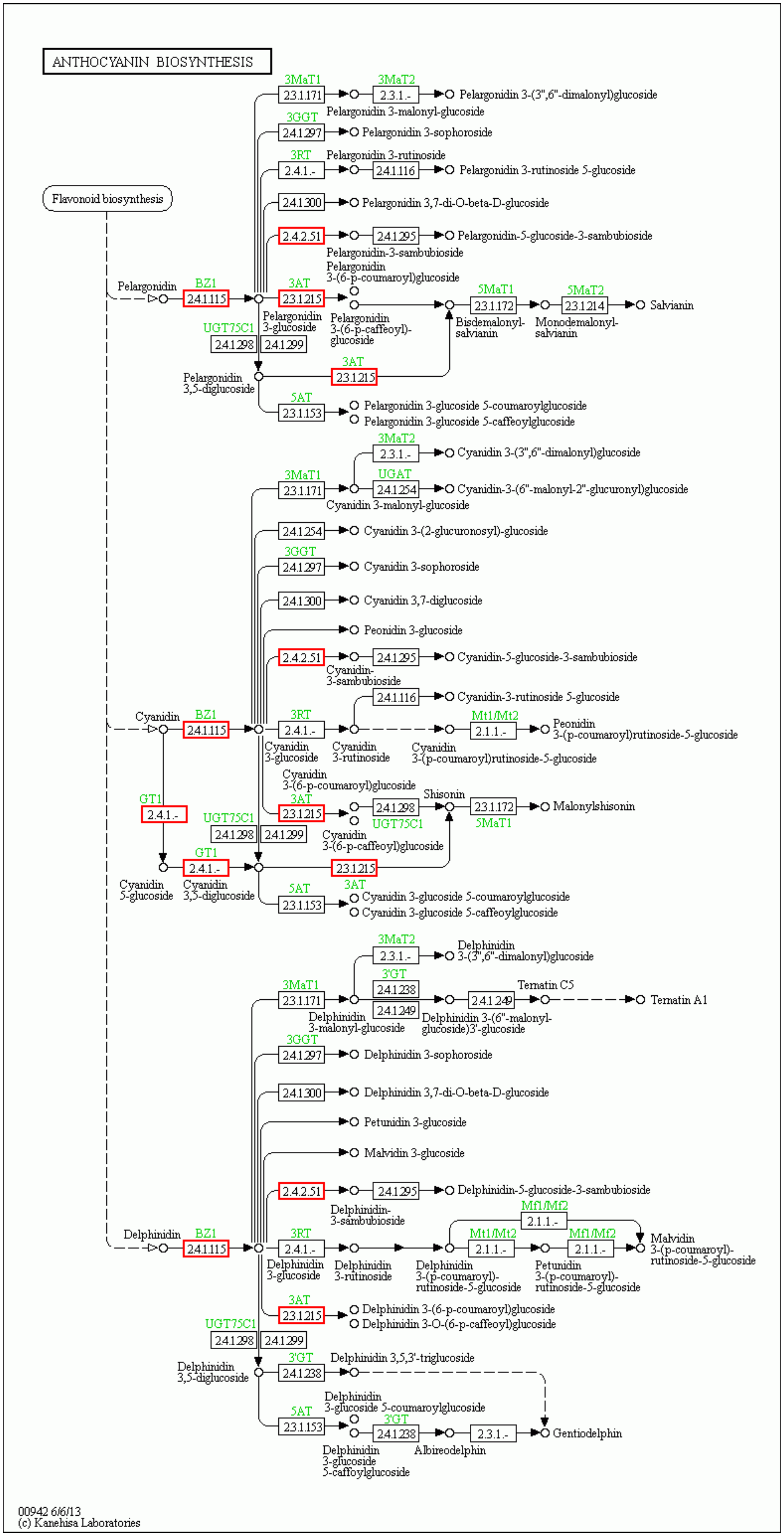
ANTHOCYANIN BIOSYNTHESIS
Flavonoid biosynthesis
Pelargonidin
BZ1
2.4.1.115
Pelargonidin 3-glucoside
3MaT1
2.3.1.171
3MaT2
2.3.1.-
Pelargonidin 3-(3'',6''-dimalonyl)glucoside
Pelargonidin 3-malonyl-glucoside
3GGT
2.4.1.297
Pelargonidin 3-sophoroside
3RT
2.4.1.-
Pelargonidin 3-rutinoside
2.4.1.116
Pelargonidin 3-rutinoside 5-glucoside
2.4.1.300
Pelargonidin 3,7-di-O-beta-D-glucoside
2.4.2.51
2.4.1.295
Pelargonidin-5-glucoside-3-sambubioside
Pelargonidin-3-sambubioside
Pelargonidin 3-(6-p-coumaroyl)glucoside
3AT
2.3.1.215
Pelargonidin 3-(6-p-caffeoyl)-glucoside
5MaT1
2.3.1.172
5MaT2
2.3.1.214
Salvianin
Bisdemalonyl-salvianin
Monodemalonyl-salvianin
UGT75C1
2.4.1.298
2.4.1.299
Pelargonidin 3,5-diglucoside
5AT
2.3.1.153
Pelargonidin 3-glucoside 5-coumaroylglucoside
Pelargonidin 3-glucoside 5-caffeoylglucoside
Cyanidin 3-(3'',6''-dimalonyl)glucoside
UGAT
2.4.1.254
Cyanidin-3-(6''-malonyl-2''-glucuronyl)glucoside
Cyanidin 3-malonyl-glucoside
Cyanidin 3-(2-glucuronosyl)-glucoside
Cyanidin 3-sophoroside
Cyanidin 3,7-diglucoside
Peonidin 3-glucoside
Cyanidin-5-glucoside-3-sambubioside
Cyanidin-3-sambubioside
Cyanidin-3-rutinoside 5-glucoside
Cyanidin
Mt1/Mt2
2.1.1.-
Peonidin 3-(p-coumaroyl)rutinoside-5-glucoside
Cyanidin 3-glucoside
Cyanidin 3-rutinoside
Cyanidin 3-(p-coumaroyl)rutinoside-5-glucoside
Cyanidin 3-(6-p-coumaroyl)glucoside
Shisonin
Malonylshisonin
Cyanidin 3-(6-p-caffeoyl)glucoside
GT1
2.4.1.-
Cyanidin 5-glucoside
Cyanidin 3,5-diglucoside
Cyanidin 3-glucoside 5-coumaroylglucoside
Cyanidin 3-glucoside 5-caffeoylglucoside
Delphinidin 3-(3'',6''-dimalonyl)glucoside
3'GT
2.4.1.238
2.4.1.249
Ternatin C5
Ternatin A1
Delphinidin 3-malonyl-glucoside
Delphinidin 3-(6''-malonyl-glucoside)3'-glucoside
Delphinidin 3-sophoroside
Delphinidin 3,7-di-O-beta-D-glucoside
Petunidin 3-glucoside
Malvidin 3-glucoside
Delphinidin-5-glucoside-3-sambubioside
Delphinidin-3-sambubioside
Mf1/Mf2
2.1.1.-
Delphinidin
Malvidin 3-(p-coumaroyl)-rutinoside-5-glucoside
Delphinidin 3-glucoside
Delphinidin 3-rutinoside
Delphinidin 3-(p-coumaroyl)-rutinoside-5-glucoside
Petunidin 3-(p-coumaroyl)-rutinoside-5-glucoside
Delphinidin 3-(6-p-coumaroyl)glucoside
Delphinidin 3-O-(6-p-caffeoyl)glucoside
Delphinidin 3,5-diglucoside
Delphinidin 3,5,3'-triglucoside
Delphinidin 3-glucoside 5-coumaroylglucoside
Gentiodelphin
Albireodelphin
2.3.1.-
Delphinidin 3-glucoside 5-caffoylglucoside
00942 6/6/13
(c) Kanehisa Laboratories

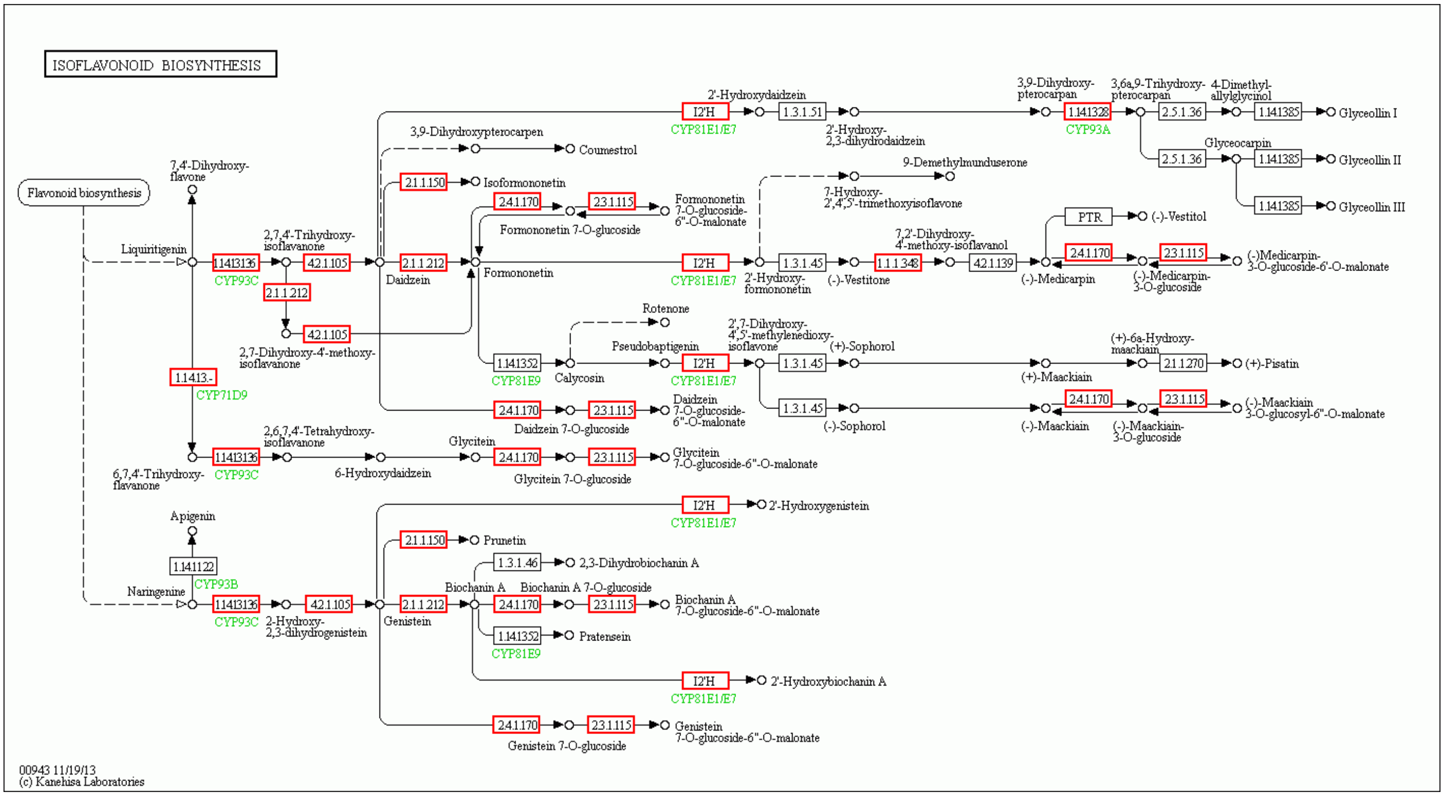
ISOFLAVONOID BIOSYNTHESIS
Flavonoid biosynthesis
7,4'-Dihydroxy-flavone
Liquiritigenin
1.14.13.136
CYP93C
2,7,4'-Trihydroxy-isoflavanone
4.2.1.105
2.1.1.212
2,7-Dihydroxy-4'-methoxy-isoflavanone
1.14.13.-
CYP71D9
6,7,4'-Trihydroxy-flavanone
2,6,7,4'-Tetrahydroxy-isoflavanone
6-Hydroxydaidzein
Glycitein
2.4.1.170
2.3.1.115
Glycitein 7-O-glucoside
Glycitein 7-O-glucoside-6"-O-malonate
Daidzein
2.1.1.150
Isoformononetin
3,9-Dihydroxypterocarpen
Coumestrol
Formononetin
Formononetin 7-O-glucoside
Formononetin 7-O-glucoside-6"-O-malonate
Daidzein 7-O-glucoside
Daidzein 7-O-glucoside-6"-O-malonate
I2'H
CYP81E1/E7
2'-Hydroxydaidzein
1.3.1.51
2'-Hydroxy-2,3-dihydrodaidzein
3,9-Dihydroxy-pterocarpan
1.14.13.28
CYP93A
3,6a,9-Trihydroxy-pterocarpan
2.5.1.36
4-Dimethyl-allylglycinol
1.14.13.85
Glyceollin I
Glyceocarpin
Glyceollin II
Glyceollin III
9-Demethylmunduserone
7-Hydroxy-2',4',5'-trimethoxyisoflavone
2'-Hydroxy-formononetin
1.3.1.45
(-)-Vestitone
1.1.1.348
7,2'-Dihydroxy-4'-methoxy-isoflavanol
4.2.1.139
(-)-Medicarpin
PTR
(-)-Vestitol
(-)-Medicarpin-3-O-glucoside
(-)Medicarpin-3-O-glucoside-6'-O-malonate
Rotenone
1.14.13.52
CYP81E9
Calycosin
Pseudobaptigenin
2',7-Dihydroxy-4',5'-methylenedioxy-isoflavone
(+)-Sophorol
(+)-Maackiain
(+)-6a-Hydroxy-maackiain
2.1.1.270
(+)-Pisatin
(-)-Sophorol
(-)-Maackiain
(-)-Maackiain-3-O-glucoside
(-)-Maackiain-3-O-glucosyl-6"-O-malonate
2'-Hydroxygenistein
Apigenin
1.14.11.22
CYP93B
Naringenine
2-Hydroxy-2,3-dihydrogenistein
Genistein
Prunetin
1.3.1.46
2,3-Dihydrobiochanin A
Biochanin A
Biochanin A 7-O-glucoside
Biochanin A 7-O-glucoside-6"-O-malonate
Pratensein
2'-Hydroxybiochanin A
Genistein 7-O-glucoside
Genistein 7-O-glucoside-6"-O-malonate
00943 11/19/13
(c) Kanehisa Laboratories

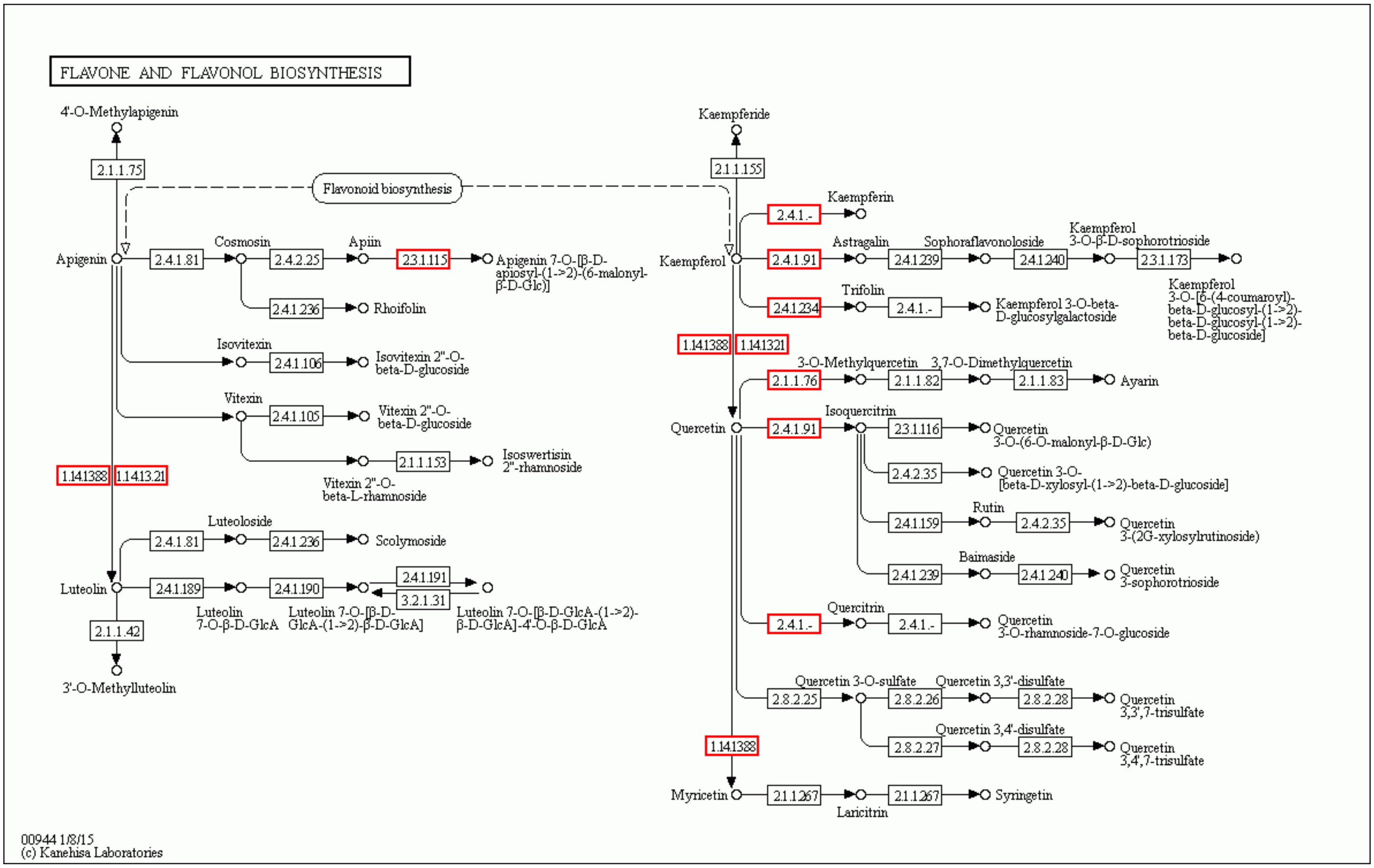
FLAVONE AND FLAVONOL BIOSYNTHESIS
4'-O-Methylapigenin
2.1.1.75
Flavonoid biosynthesis
Apigenin
2.4.1.81
Cosmosin
2.4.2.25
Apiin
2.3.1.115
Apigenin 7-O-[β-D-apiosyl-(1->2)-(6-malonyl-β-D-Glc)]
2.4.1.236
Rhoifolin
Isovitexin
2.4.1.106
Isovitexin 2"-O-beta-D-glucoside
Vitexin
2.4.1.105
Vitexin 2"-O-beta-D-glucoside
2.1.1.153
Isoswertisin 2"-rhamnoside
Vitexin 2"-O-beta-L-rhamnoside
1.14.13.88
1.14.13.21
Luteoloside
Scolymoside
Luteolin
2.4.1.189
2.4.1.190
2.4.1.191
3.2.1.31
Luteolin 7-O-β-D-GlcA
Luteolin 7-O-[β-D-GlcA-(1->2)-β-D-GlcA]
Luteolin 7-O-[β-D-GlcA-(1->2)-β-D-GlcA]-4'-O-β-D-GlcA
2.1.1.42
3'-O-Methylluteolin
Kaempferide
2.1.1.155
Kaempferin
2.4.1.-
Kaempferol
2.4.1.91
Astragalin
2.4.1.239
Sophoraflavonoloside
2.4.1.240
Kaempferol 3-O-β-D-sophorotrioside
2.3.1.173
Kaempferol 3-O-[6-(4-coumaroyl)-beta-D-glucosyl-(1->2)-beta-D-glucosyl-(1->2)-beta-D-glucoside]
2.4.1.234
Trifolin
Kaempferol 3-O-beta-D-glucosylgalactoside
3-O-Methylquercetin
2.1.1.76
2.1.1.82
3,7-O-Dimethylquercetin
2.1.1.83
Ayarin
Quercetin
Isoquercitrin
2.3.1.116
Quercetin 3-O-(6-O-malonyl-β-D-Glc)
2.4.2.35
Quercetin 3-O-[beta-D-xylosyl-(1->2)-beta-D-glucoside]
2.4.1.159
Rutin
Quercetin 3-(2G-xylosylrutinoside)
Baimaside
Quercetin 3-sophorotrioside
Quercitrin
Quercetin 3-O-rhamnoside-7-O-glucoside
Quercetin 3-O-sulfate
2.8.2.25
2.8.2.26
Quercetin 3,3'-disulfate
2.8.2.28
Quercetin 3,3',7-trisulfate
Quercetin 3,4'-disulfate
2.8.2.27
Quercetin 3,4',7-trisulfate
Myricetin
2.1.1.267
Laricitrin
Syringetin
00944 1/8/15
(c) Kanehisa Laboratories

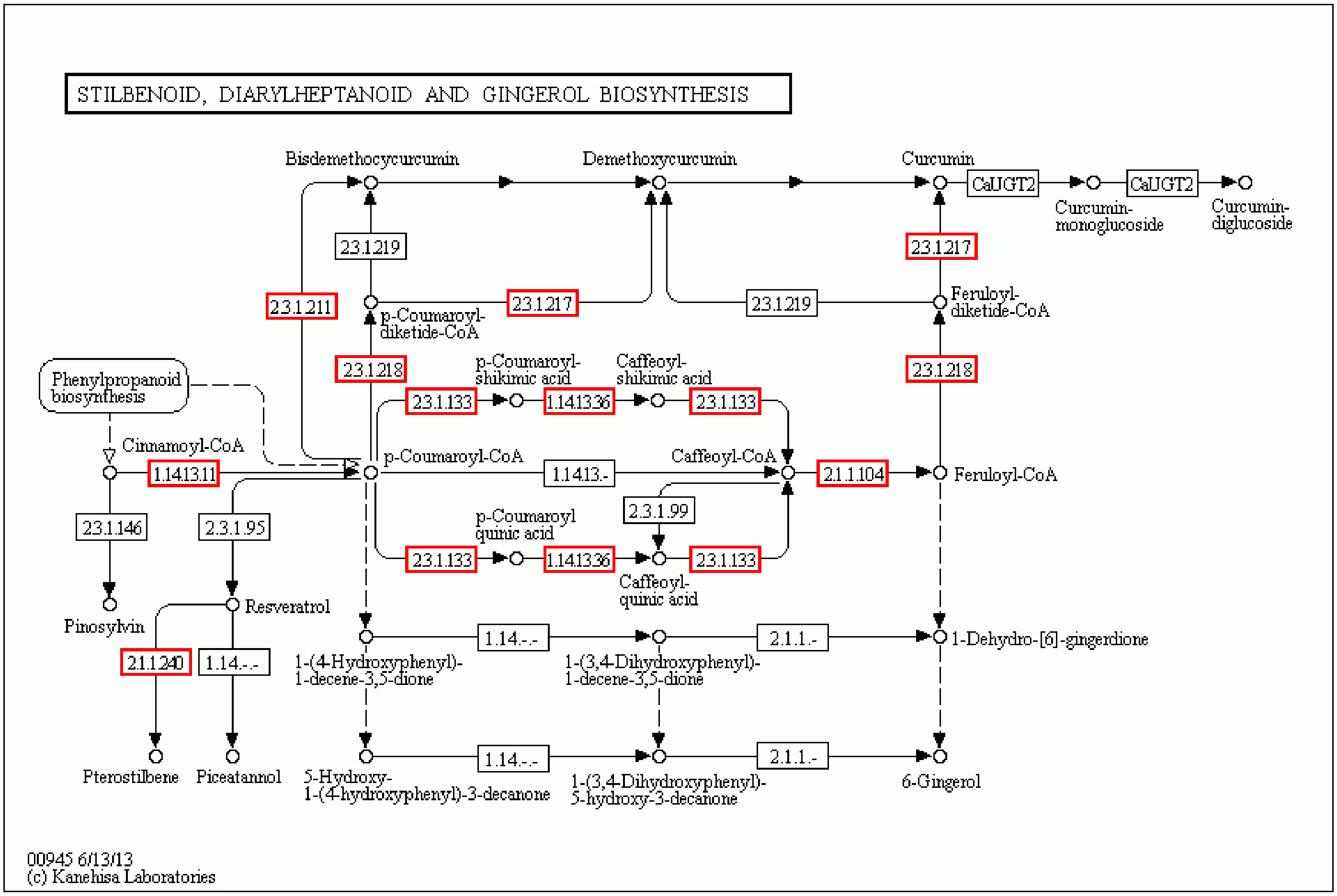
STILBENOID, DIARYLHEPTANOID AND GINGEROL BIOSYNTHESIS
Bisdemethocycurcumin
Demethoxycurcumin
Curcumin
CaUGT2
CaUGT2
Curcumin-monoglucoside
Curcumin-diglucoside
2.3.1.219
2.3.1.217
2.3.1.211
2.3.1.217
2.3.1.219
Feruloyl-diketide-CoA
p-Coumaroyl-diketide-CoA
2.3.1.218
2.3.1.218
Phenylpropanoid biosynthesis
p-Coumaroyl-shikimic acid
Caffeoyl-shikimic acid
2.3.1.133
1.14.13.36
2.3.1.133
Cinnamoyl-CoA
p-Coumaroyl-CoA
Caffeoyl-CoA
1.14.13.11
1.14.13.-
2.1.1.104
Feruloyl-CoA
2.3.1.99
2.3.1.146
2.3.1.95
p-Coumaroyl quinic acid
2.3.1.133
1.14.13.36
2.3.1.133
Caffeoyl-quinic acid
Pinosylvin
Resveratrol
1.14.-.-
2.1.1.-
1-Dehydro-[6]-gingerdione
2.1.1.240
1.14.-.-
1-(4-Hydroxyphenyl)-1-decene-3,5-dione
1-(3,4-Dihydroxyphenyl)-1-decene-3,5-dione
1.14.-.-
2.1.1.-
Pterostilbene
Piceatannol
5-Hydroxy-1-(4-hydroxyphenyl)-3-decanone
1-(3,4-Dihydroxyphenyl)-5-hydroxy-3-decanone
6-Gingerol
00945 6/13/13
(c) Kanehisa Laboratories

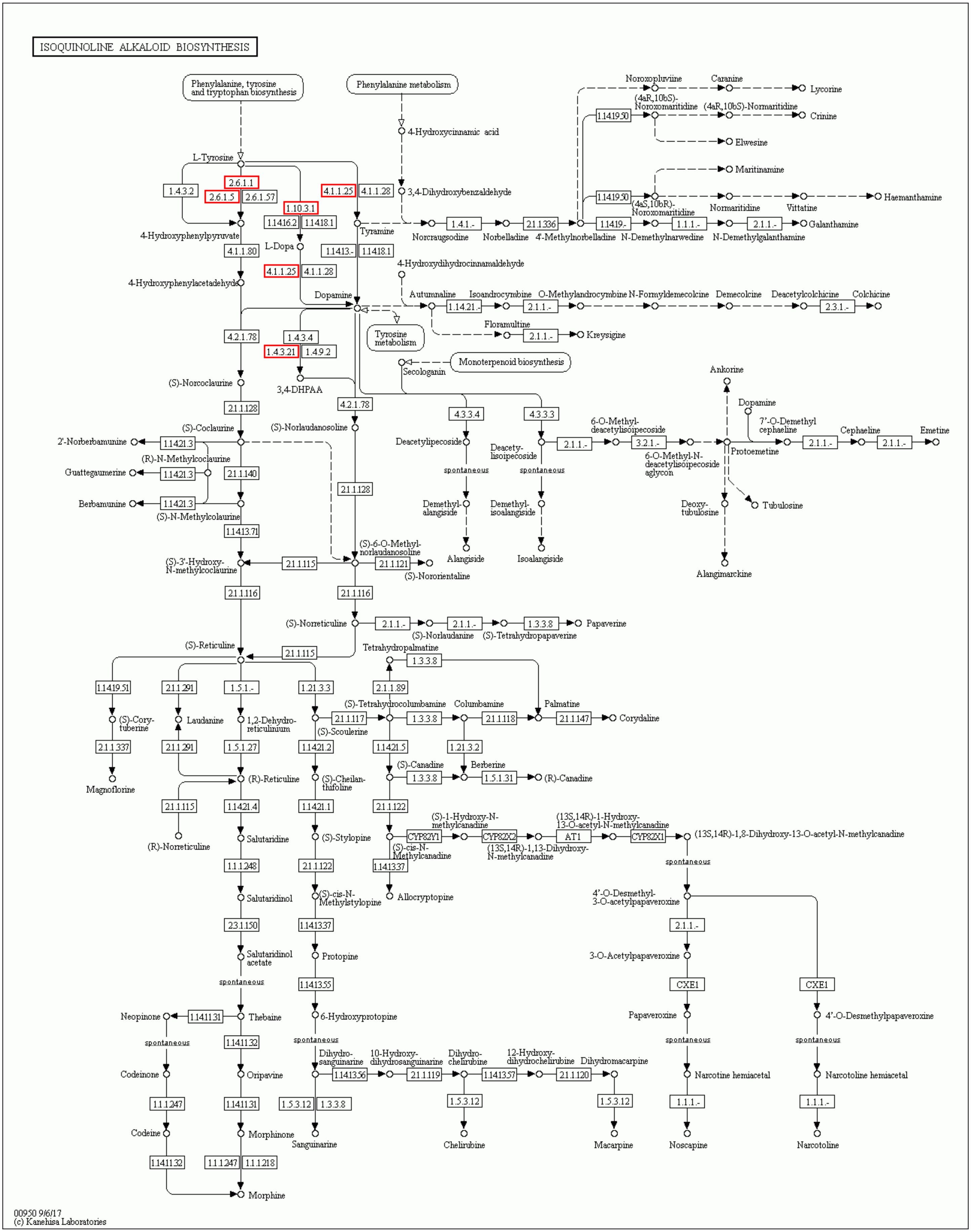
ISOQUINOLINE ALKALOID BIOSYNTHESIS
Phenylalanine, tyrosine and tryptophan biosynthesis
Phenylalanine metabolism
4-Hydroxycinnamic acid
3,4-Dihydroxybenzaldehyde
L-Tyrosine
4-Hydroxyphenylpyruvate
4-Hydroxyphenylacetaldehyde
L-Dopa
Tyramine
Dopamine
Tyrosine metabolism
3,4-DHPAA
Monoterpenoid biosynthesis
Secologanin
4-Hydroxydihydrocinnamaldehyde
Norcraugsodine
Norbelladine
4'-Methylnorbelladine
N-Demethylnarwedine
N-Demethylgalanthamine
Galanthamine
Noroxopluviine
Caranine
Lycorine
(4aR,10bS)-Noroxomaritidine
(4aR,10bS)-Normaritidine
Crinine
Elwesine
Maritinamine
(4aS,10bR)-Noroxomaritidine
Normaritidine
Vittatine
Haemanthamine
Autumnaline
Isoandrocymbine
O-Methylandrocymbine
N-Formyldemecolcine
Demecolcine
Deacetylcolchicine
Colchicine
Floramultine
Kreysigine
Ankorine
Dopamine
7'-O-Demethyl cephaeline
Cephaeline
Emetine
6-O-Methyl-deacetylisoipecoside
6-O-Methyl-N-deacetylisoipecoside aglycon
Protoemetine
Deacetylipecoside
Deacetyl-isoipecoside
spontaneous
Demethyl-alangiside
Demethyl-isoalangiside
Alangiside
Isoalangiside
Deoxy-tubulosine
Tubulosine
Alangimarckine
(S)-Norcoclaurine
(S)-Coclaurine
2'-Norberbamunine
(R)-N-Methylcoclaurine
Guattegaumerine
Berbamunine
(S)-N-Methylcoclaurine
(S)-3'-Hydroxy-N-methylcoclaurine
(S)-Norlaudanosoline
(S)-6-O-Methyl-norlaudanosoline
(S)-Nororientaline
(S)-Norreticuline
(S)-Norlaudanine
(S)-Tetrahydropapaverine
Papaverine
(S)-Reticuline
Tetrahydropalmatine
(S)-Tetrahydrocolumbamine
Columbamine
Palmatine
Corydaline
(S)-Cory-tuberine
Magnoflorine
Laudanine
1,2-Dehydro-reticulinium
(R)-Reticuline
(R)-Norreticuline
(S)-Scoulerine
(S)-Cheilan-thifoline
(S)-Stylopine
(S)-cis-N-Methylstylopine
Protopine
6-Hydroxyprotopine
(S)-Canadine
Berberine
(R)-Canadine
(S)-1-Hydroxy-N-methylcanadine
(S)-cis-N-Methylcanadine
Allocryptopine
(13S,14R)-1,13-Dihydroxy-N-methylcanadine
(13S,14R)-1-Hydroxy-13-O-acetyl-N-methylcanadine
(13S,14R)-1,8-Dihydroxy-13-O-acetyl-N-methylcanadine
4'-O-Desmethyl-3-O-acetylpapaveroxine
3-O-Acetylpapaveroxine
Papaveroxine
4'-O-Desmethylpapaveroxine
Narcotine hemiacetal
Narcotoline hemiacetal
Noscapine
Narcotoline
Salutaridine
Salutaridinol
Salutaridinol acetate
Thebaine
Neopinone
Codeinone
Codeine
Oripavine
Morphinone
Morphine
Dihydro-sanguinarine
10-Hydroxy-dihydrosanguinarine
Dihydro-chelirubine
12-Hydroxy-dihydrochelirubine
Dihydromacarpine
Sanguinarine
Chelirubine
Macarpine
2.6.1.1
2.6.1.5
2.6.1.57
1.4.3.2
1.10.3.1
1.14.16.2
1.14.18.1
4.1.1.25
4.1.1.28
4.1.1.80
1.14.13.-
4.2.1.78
1.4.3.4
1.4.3.21
1.4.9.2
1.4.1.-
2.1.1.336
1.14.19.50
1.14.19.-
1.1.1.-
2.1.1.-
1.14.21.-
2.3.1.-
4.3.3.4
4.3.3.3
3.2.1.-
2.1.1.128
1.14.21.3
2.1.1.140
1.14.13.71
2.1.1.115
2.1.1.121
2.1.1.116
1.3.3.8
1.14.19.51
2.1.1.291
1.5.1.-
1.21.3.3
2.1.1.89
2.1.1.117
2.1.1.118
2.1.1.147
2.1.1.337
1.5.1.27
1.14.21.2
1.14.21.5
1.21.3.2
1.5.1.31
1.14.21.4
1.14.21.1
2.1.1.122
CYP82Y1
CYP82X2
AT1
CYP82X1
1.1.1.248
1.14.13.37
2.3.1.150
1.14.13.55
CXE1
1.14.11.31
1.14.11.32
1.1.1.247
1.1.1.218
1.14.13.56
2.1.1.119
1.14.13.57
2.1.1.120
1.5.3.12
00950 9/6/17
(c) Kanehisa Laboratories

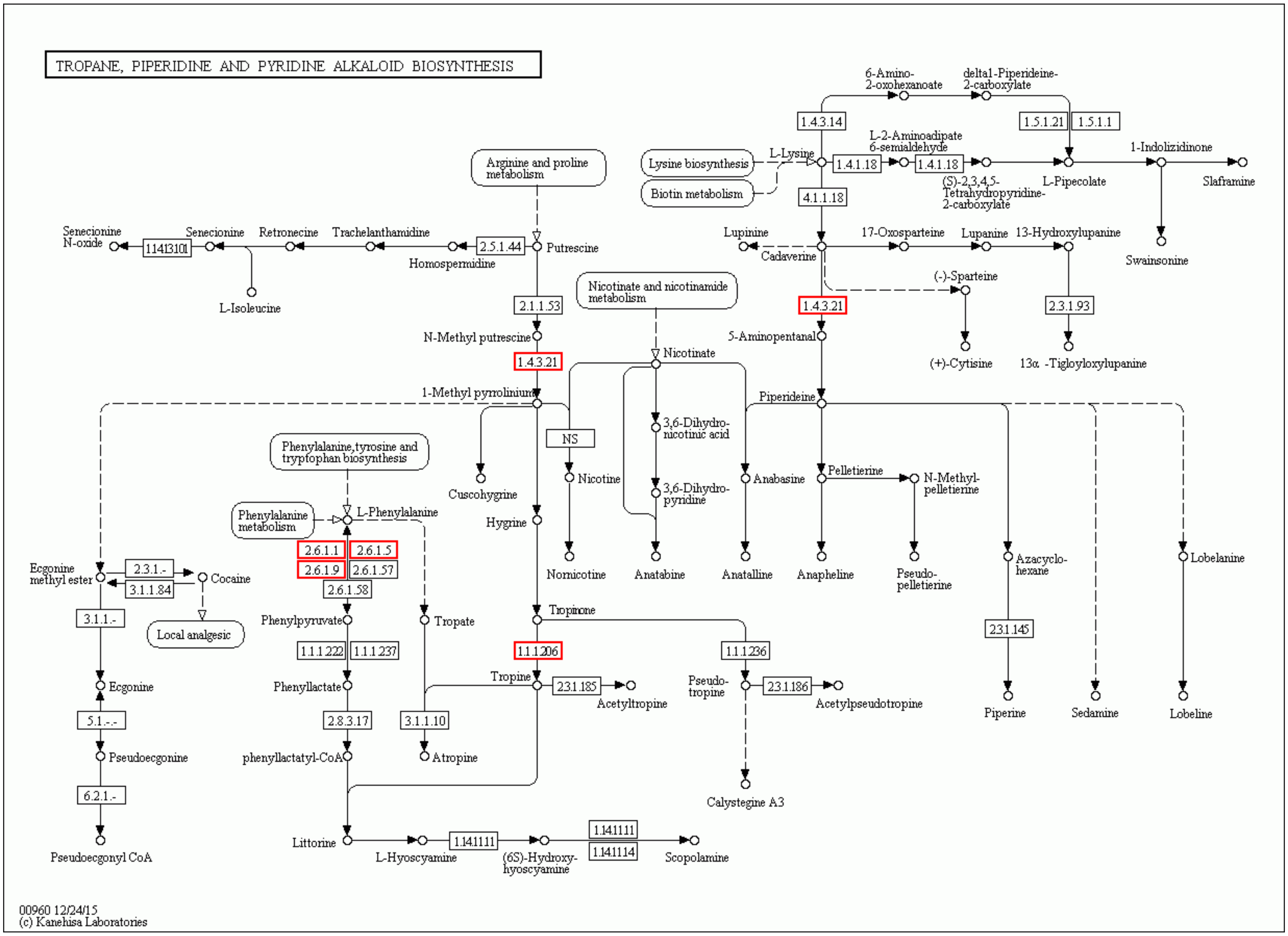

TROPANE, PIPERIDINE AND PYRIDINE ALKALOID BIOSYNTHESIS
6-Amino-2-oxohexanoate
delta1-Piperideine-2-carboxylate
1.4.3.14
1.5.1.21
1.5.1.1
L-2-Aminoadipate 6-semialdehyde
Arginine and proline metabolism
Lysine biosynthesis
L-Lysine
1.4.1.18
1.4.1.18
1-Indolizidinone
Slaframine
Biotin metabolism
4.1.1.18
(S)-2,3,4,5-Tetrahydropyridine-2-carboxylate
L-Pipecolate
Swainsonine
Senecionine N-oxide
1.14.13.101
Senecionine
Retronecine
Trachelanthamidine
2.5.1.44
Putrescine
Homospermidine
L-Isoleucine
Lupinine
Cadaverine
17-Oxosparteine
Lupanine
13-Hydroxylupanine
(-)-Sparteine
Nicotinate and nicotinamide metabolism
2.1.1.53
1.4.3.21
2.3.1.93
N-Methyl putrescine
5-Aminopentanal
Nicotinate
1.4.3.21
(+)-Cytisine
13α -Tigloyloxylupanine
1-Methyl pyrrolinium
Piperideine
3,6-Dihydro-nicotinic acid
NS
Phenylalanine,tyrosine and tryptophan biosynthesis
Cuscohygrine
Nicotine
Anabasine
Pelletierine
N-Methyl-pelletierine
3,6-Dihydro-pyridine
Phenylalanine metabolism
L-Phenylalanine
Hygrine
2.6.1.1
2.6.1.5
2.6.1.9
2.6.1.57
Nornicotine
Anatabine
Anatalline
Anapheline
Pseudo-pelletierine
Azacyclo-hexane
Lobelanine
Ecgonine methyl ester
2.3.1.-
3.1.1.84
Cocaine
2.6.1.58
Tropinone
3.1.1.-
Local analgesic
Phenylpyruvate
Tropate
2.3.1.145
1.1.1.222
1.1.1.237
1.1.1.206
1.1.1.236
Ecgonine
Phenyllactate
Tropine
2.3.1.185
Pseudo-tropine
2.3.1.186
Acetyltropine
Acetylpseudotropine
Piperine
Sedamine
Lobeline
5.1.-.-
2.8.3.17
3.1.1.10
Pseudoecgonine
phenyllactatyl-CoA
Atropine
6.2.1.-
Calystegine A3
1.14.11.11
Littorine
1.14.11.11
L-Hyoscyamine
1.14.11.14
(6S)-Hydroxy-hyoscyamine
Scopolamine
Pseudoecgonyl CoA
00960 12/24/15
(c) Kanehisa Laboratories

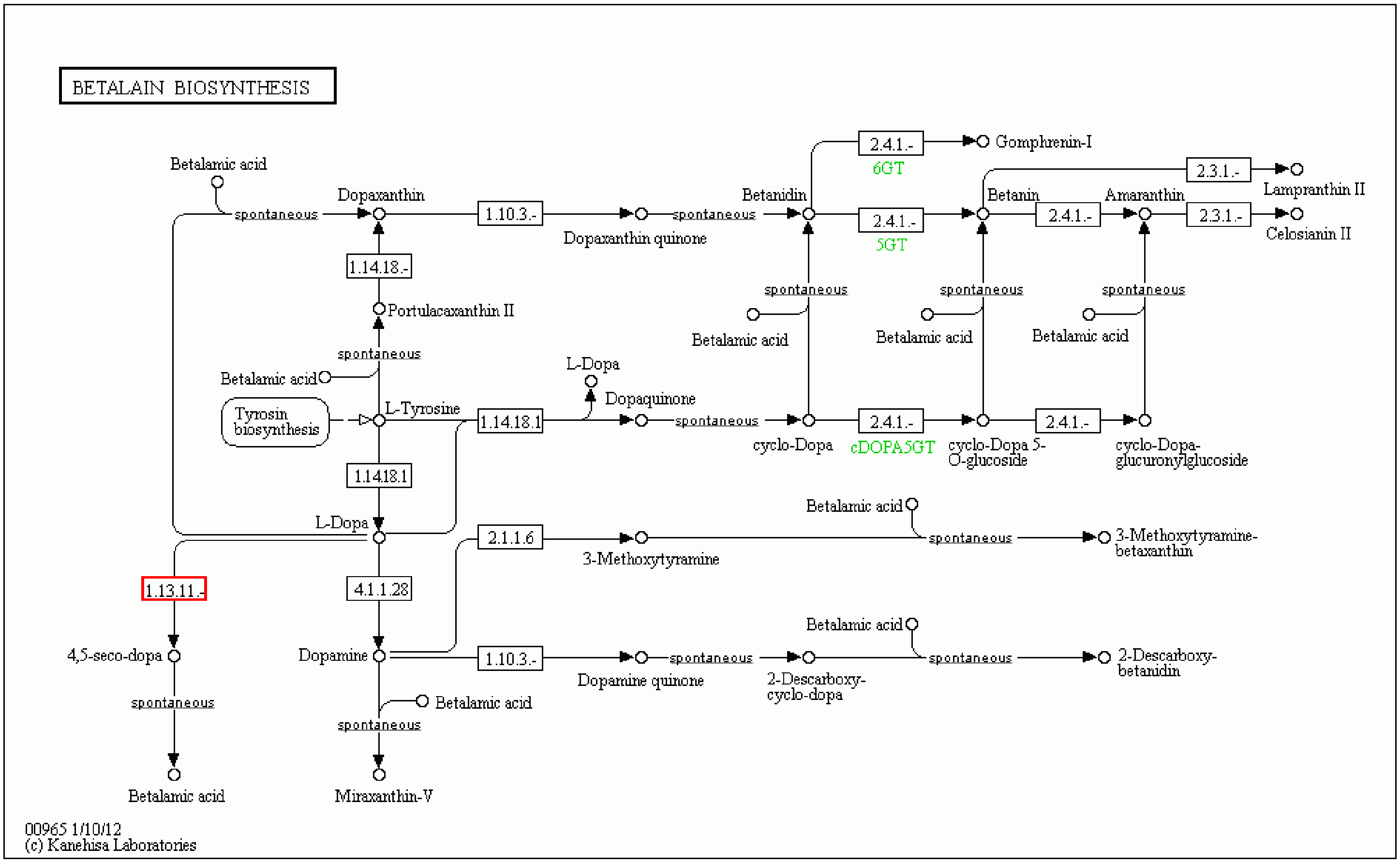
BETALAIN BIOSYNTHESIS
Betalamic acid
spontaneous
Dopaxanthin
1.10.3.-
Dopaxanthin quinone
spontaneous
Betanidin
2.4.1.-
6GT
Gomphrenin-I
2.4.1.-
5GT
Betanin
2.4.1.-
Amaranthin
2.3.1.-
Lampranthin II
2.3.1.-
Celosianin II
1.14.18.-
Portulacaxanthin II
spontaneous
Betalamic acid
Tyrosin biosynthesis
L-Tyrosine
1.14.18.1
L-Dopa
Dopaquinone
spontaneous
cyclo-Dopa
2.4.1.-
cDOPA5GT
cyclo-Dopa 5-O-glucoside
2.4.1.-
cyclo-Dopa-glucuronylglucoside
spontaneous
Betalamic acid
spontaneous
Betalamic acid
spontaneous
Betalamic acid
1.14.18.1
L-Dopa
2.1.1.6
3-Methoxytyramine
Betalamic acid
spontaneous
3-Methoxytyramine-betaxanthin
1.13.11.-
4.1.1.28
4,5-seco-dopa
Dopamine
1.10.3.-
Dopamine quinone
spontaneous
2-Descarboxy-cyclo-dopa
Betalamic acid
spontaneous
2-Descarboxy-betanidin
spontaneous
Betalamic acid
spontaneous
Betalamic acid
Miraxanthin-V
00965 1/10/12
(c) Kanehisa Laboratories

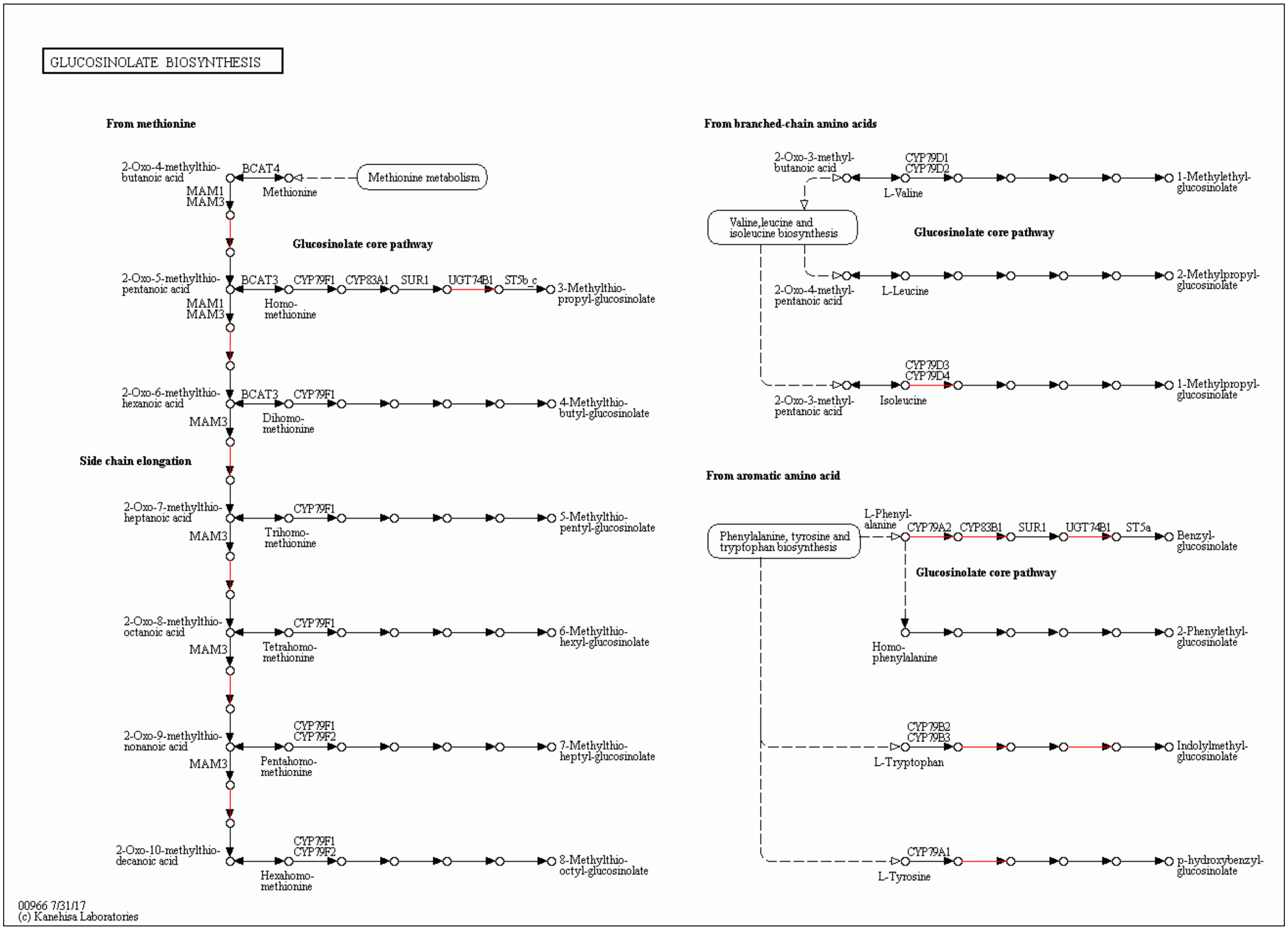

GLUCOSINOLATE BIOSYNTHESIS
From methionine
2-Oxo-4-methylthio-butanoic acid
BCAT4
Methionine
Methionine metabolism
MAM1
MAM3
Glucosinolate core pathway
2-Oxo-5-methylthio-pentanoic acid
BCAT3
CYP79F1
CYP83A1
SUR1
UGT74B1
ST5b_c
3-Methylthio-propyl-glucosinolate
Homo-methionine
2-Oxo-6-methylthio-hexanoic acid
Dihomo-methionine
4-Methylthio-butyl-glucosinolate
Side chain elongation
2-Oxo-7-methylthio-heptanoic acid
Trihomo-methionine
5-Methylthio-pentyl-glucosinolate
2-Oxo-8-methylthio-octanoic acid
Tetrahomo-methionine
6-Methylthio-hexyl-glucosinolate
2-Oxo-9-methylthio-nonanoic acid
CYP79F2
Pentahomo-methionine
7-Methylthio-heptyl-glucosinolate
2-Oxo-10-methylthio-decanoic acid
Hexahomo-methionine
8-Methylthio-octyl-glucosinolate
From branched-chain amino acids
2-Oxo-3-methyl-butanoic acid
CYP79D1
CYP79D2
L-Valine
1-Methylethyl-glucosinolate
Valine,leucine and isoleucine biosynthesis
2-Oxo-4-methyl-pentanoic acid
L-Leucine
2-Methylpropyl-glucosinolate
CYP79D3
CYP79D4
2-Oxo-3-methyl-pentanoic acid
Isoleucine
1-Methylpropyl-glucosinolate
From aromatic amino acid
Phenylalanine, tyrosine and tryptophan biosynthesis
L-Phenyl-alanine
CYP79A2
CYP83B1
SUR1
UGT74B1
ST5a
Benzyl-glucosinolate
Homo-phenylalanine
2-Phenylethyl-glucosinolate
CYP79B2
CYP79B3
L-Tryptophan
Indolylmethyl-glucosinolate
CYP79A1
L-Tyrosine
p-hydroxybenzyl-glucosinolate
00966 7/31/17
(c) Kanehisa Laboratories

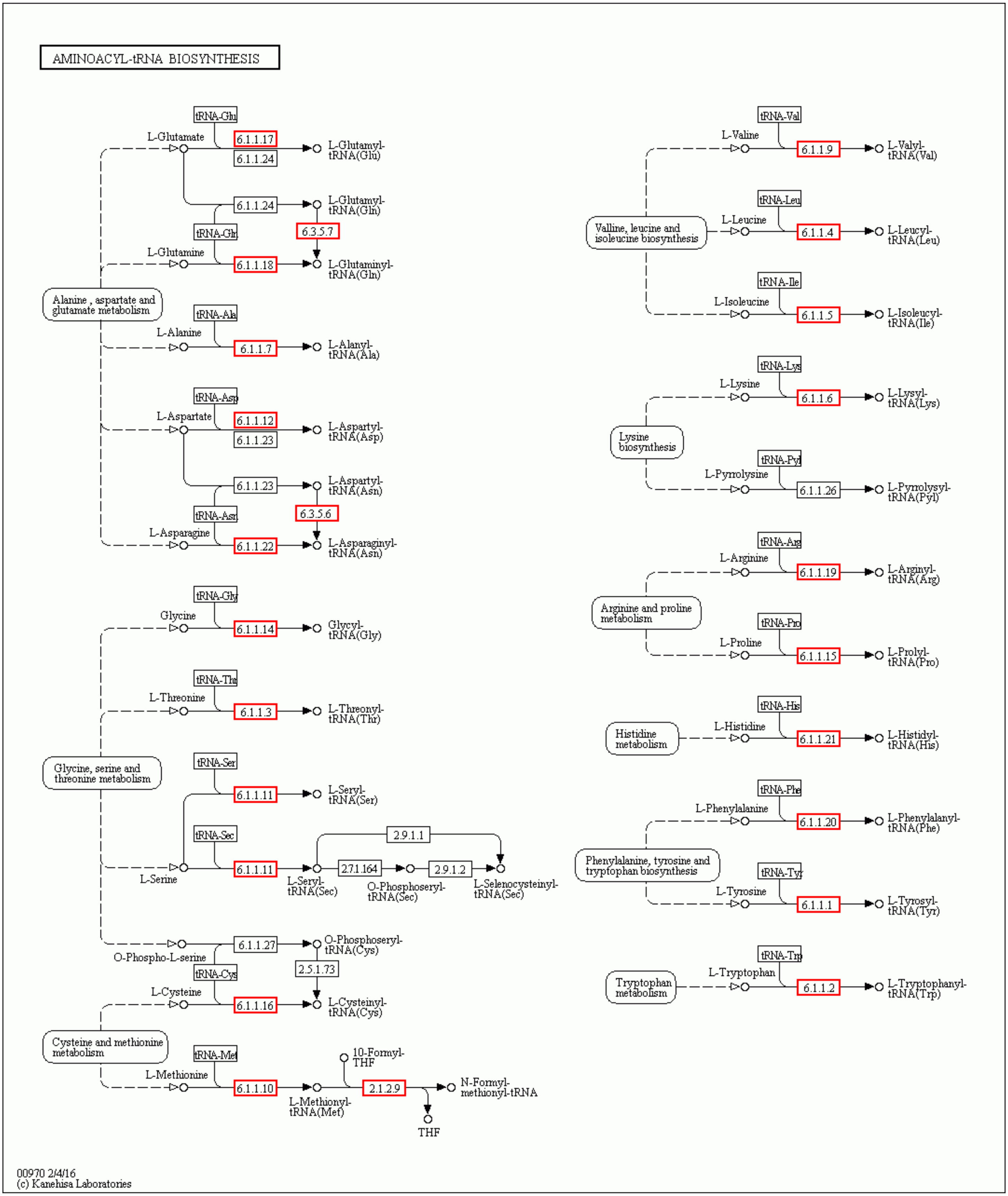
AMINOACYL-tRNA BIOSYNTHESIS
tRNA-Glu
L-Glutamate
6.1.1.17
6.1.1.24
L-Glutamyl-tRNA(Glu)
L-Glutamyl-tRNA(Gln)
6.3.5.7
tRNA-Gln
L-Glutamine
6.1.1.18
L-Glutaminyl-tRNA(Gln)
Alanine , aspartate and glutamate metabolism
tRNA-Ala
L-Alanine
6.1.1.7
L-Alanyl-tRNA(Ala)
tRNA-Asp
L-Aspartate
6.1.1.12
6.1.1.23
L-Aspartyl-tRNA(Asp)
L-Aspartyl-tRNA(Asn)
6.3.5.6
tRNA-Asn
L-Asparagine
6.1.1.22
L-Asparaginyl-tRNA(Asn)
tRNA-Gly
Glycine
6.1.1.14
Glycyl-tRNA(Gly)
tRNA-Thr
L-Threonine
6.1.1.3
L-Threonyl-tRNA(Thr)
Glycine, serine and threonine metabolism
tRNA-Ser
6.1.1.11
L-Seryl-tRNA(Ser)
tRNA-Sec
2.9.1.1
L-Serine
L-Seryl-tRNA(Sec)
2.7.1.164
O-Phosphoseryl-tRNA(Sec)
2.9.1.2
L-Selenocysteinyl-tRNA(Sec)
O-Phospho-L-serine
6.1.1.27
O-Phosphoseryl-tRNA(Cys)
tRNA-Cys
2.5.1.73
L-Cysteine
6.1.1.16
L-Cysteinyl-tRNA(Cys)
Cysteine and methionine metabolism
tRNA-Met
L-Methionine
6.1.1.10
L-Methionyl-tRNA(Met)
10-Formyl-THF
2.1.2.9
N-Formyl-methionyl-tRNA
THF
tRNA-Val
L-Valine
6.1.1.9
L-Valyl-tRNA(Val)
Valline, leucine and isoleucine biosynthesis
tRNA-Leu
L-Leucine
6.1.1.4
L-Leucyl-tRNA(Leu)
tRNA-Ile
L-Isoleucine
6.1.1.5
L-Isoleucyl-tRNA(Ile)
tRNA-Lys
L-Lysine
6.1.1.6
L-Lysyl-tRNA(Lys)
Lysine biosynthesis
tRNA-Pyl
L-Pyrrolysine
6.1.1.26
L-Pyrrolysyl-tRNA(Pyl)
tRNA-Arg
L-Arginine
6.1.1.19
L-Arginyl-tRNA(Arg)
Arginine and proline metabolism
tRNA-Pro
L-Proline
6.1.1.15
L-Prolyl-tRNA(Pro)
tRNA-His
Histidine metabolism
L-Histidine
6.1.1.21
L-Histidyl-tRNA(His)
tRNA-Phe
L-Phenylalanine
6.1.1.20
L-Phenylalanyl-tRNA(Phe)
Phenylalanine, tyrosine and tryptophan biosynthesis
tRNA-Tyr
L-Tyrosine
6.1.1.1
L-Tyrosyl-tRNA(Tyr)
tRNA-Trp
Tryptophan metabolism
L-Tryptophan
6.1.1.2
L-Tryptophanyl-tRNA(Trp)
00970 2/4/16
(c) Kanehisa Laboratories

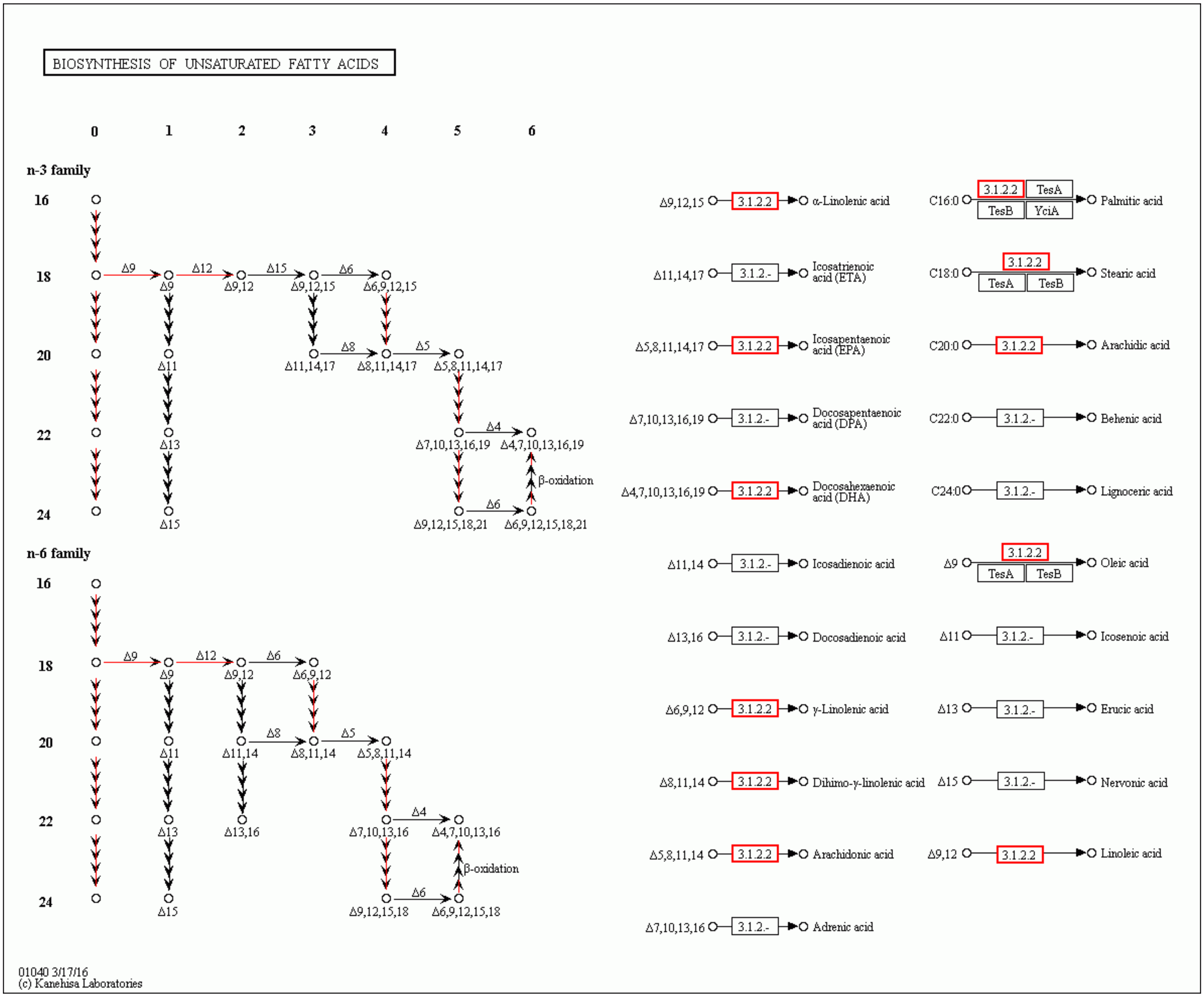
BIOSYNTHESIS OF UNSATURATED FATTY ACIDS
n-3 family
n-6 family
β-oxidation
α-Linolenic acid
Icosatrienoic acid (ETA)
Icosapentaenoic acid (EPA)
Docosapentaenoic acid (DPA)
Docosahexaenoic acid (DHA)
Icosadienoic acid
Docosadienoic acid
γ-Linolenic acid
Dihimo-γ-linolenic acid
Arachidonic acid
Adrenic acid
Palmitic acid
Stearic acid
Arachidic acid
Behenic acid
Lignoceric acid
Oleic acid
Icosenoic acid
Erucic acid
Nervonic acid
Linoleic acid
3.1.2.2
3.1.2.-
TesA
TesB
YciA
01040 3/17/16
(c) Kanehisa Laboratories

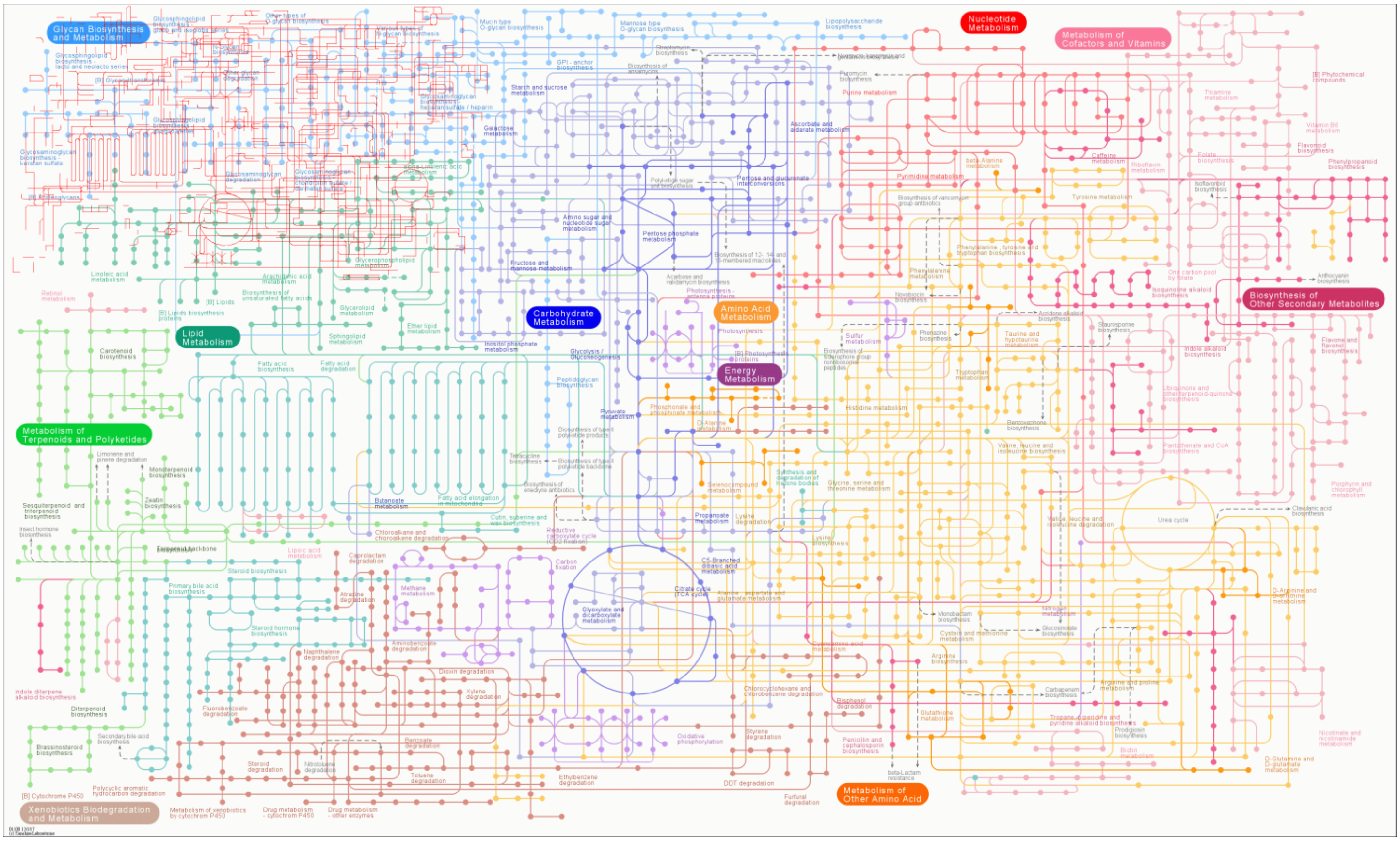
Glycan Biosynthesis and Metabolism
Nucleotide Metabolism
Metabolism of Cofactors and Vitamins
Carbohydrate Metabolism
Amino Acid Metabolism
Energy Metabolism
Lipid Metabolism
Biosynthesis of Other Secondary Metabolites
Metabolism of Terpenoids and Polyketides
Xenobiotics Biodegradation and Metabolism
Metabolism of Other Amino Acid
Purine metabolism
Pyrimidine metabolism
Pentose phosphate metabolism
Glycolysis / Gluconeogenesis
Citrate cycle (TCA cycle)
Urea cycle
Starch and sucrose metabolism
Carotenoid biosynthesis

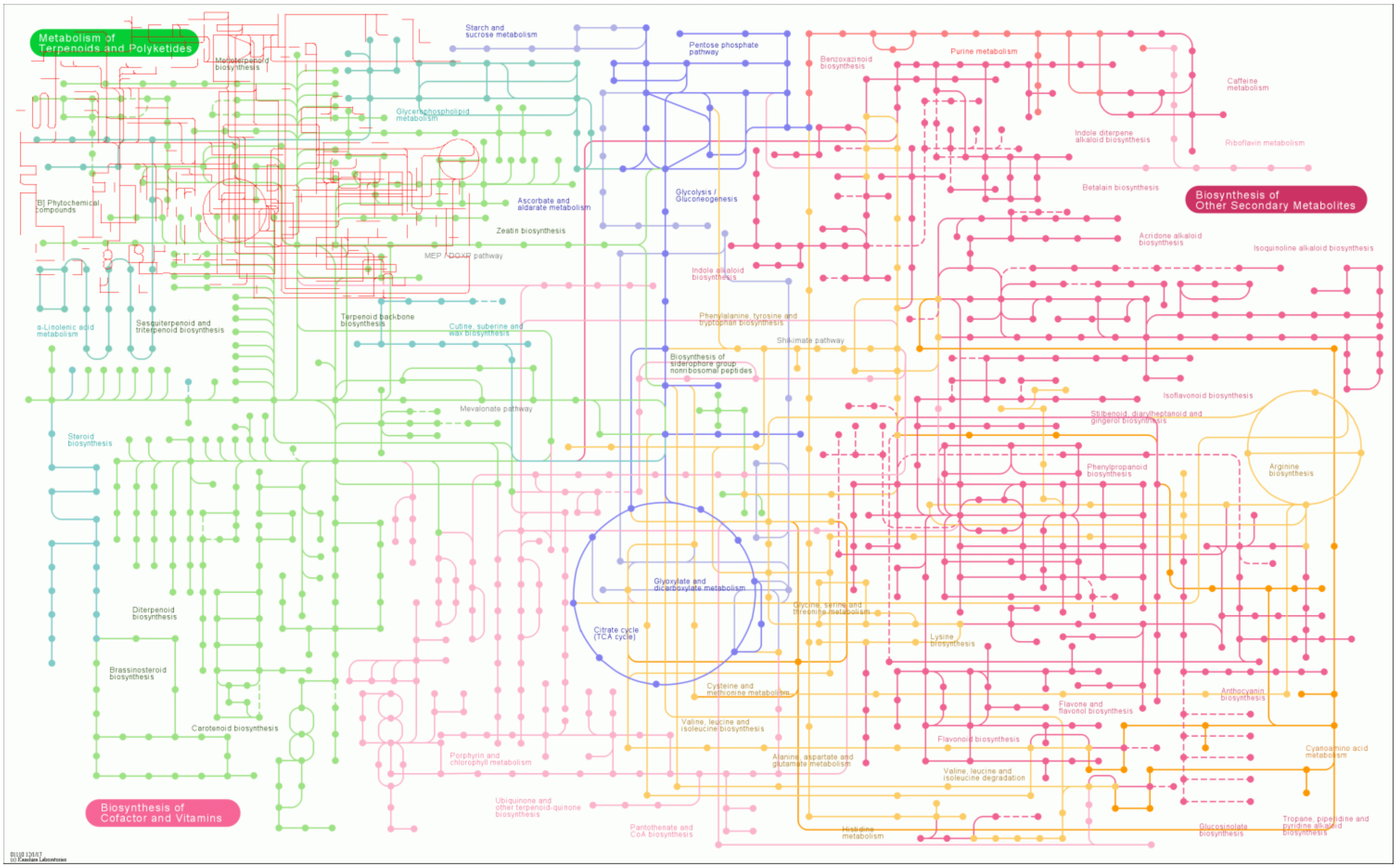
Metabolism of Terpenoids and Polyketides
Starch and sucrose metabolism
Pentose phosphate pathway
Purine metabolism
Benzoxazinoid biosynthesis
Caffeine metabolism
Indole diterpene alkaloid biosynthesis
Riboflavin metabolism
Betalain biosynthesis
Biosynthesis of Other Secondary Metabolites
Acridone alkaloid biosynthesis
Isoquinoline alkaloid biosynthesis
Glycerophospholipid metabolism
Ascorbate and aldarate metabolism
Zeatin biosynthesis
MEP / DOXP pathway
Glycolysis / Gluconeogenesis
Indole alkaloid biosynthesis
Phenylalanine, tyrosine and tryptophan biosynthesis
Shikimate pathway
Terpenoid backbone biosynthesis
Cutine, suberine and wax biosynthesis
Sesquiterpenoid and triterpenoid biosynthesis
Monoterpenoid biosynthesis
[B] Phytochemical compounds
a-Linolenic acid metabolism
Mevalonate pathway
Steroid biosynthesis
Isoflavonoid biosynthesis
Stilbenoid, diarylheptanoid and gingerol biosynthesis
Phenylpropanoid biosynthesis
Arginine biosynthesis
Glyoxylate and dicarboxylate metabolism
Citrate cycle (TCA cycle)
Glycine, serine and threonine metabolism
Lysine biosynthesis
Diterpenoid biosynthesis
Brassinosteroid biosynthesis
Carotenoid biosynthesis
Cysteine and methionine metabolism
Valine, leucine and isoleucine biosynthesis
Anthocyanin biosynthesis
Flavone and flavonol biosynthesis
Flavonoid biosynthesis
Porphyrin and chlorophyll metabolism
Alanine, aspartate and glutamate metabolism
Valine, leucine and isoleucine degradation
Cyanoamino acid metabolism
Ubiquinone and other terpenoid-quinone biosynthesis
Biosynthesis of Cofactor and Vitamins
Pantothenate and CoA biosynthesis
Histidine metabolism
Glucosinolate biosynthesis
Tropane, piperidine and pyridine alkaloid biosynthesis

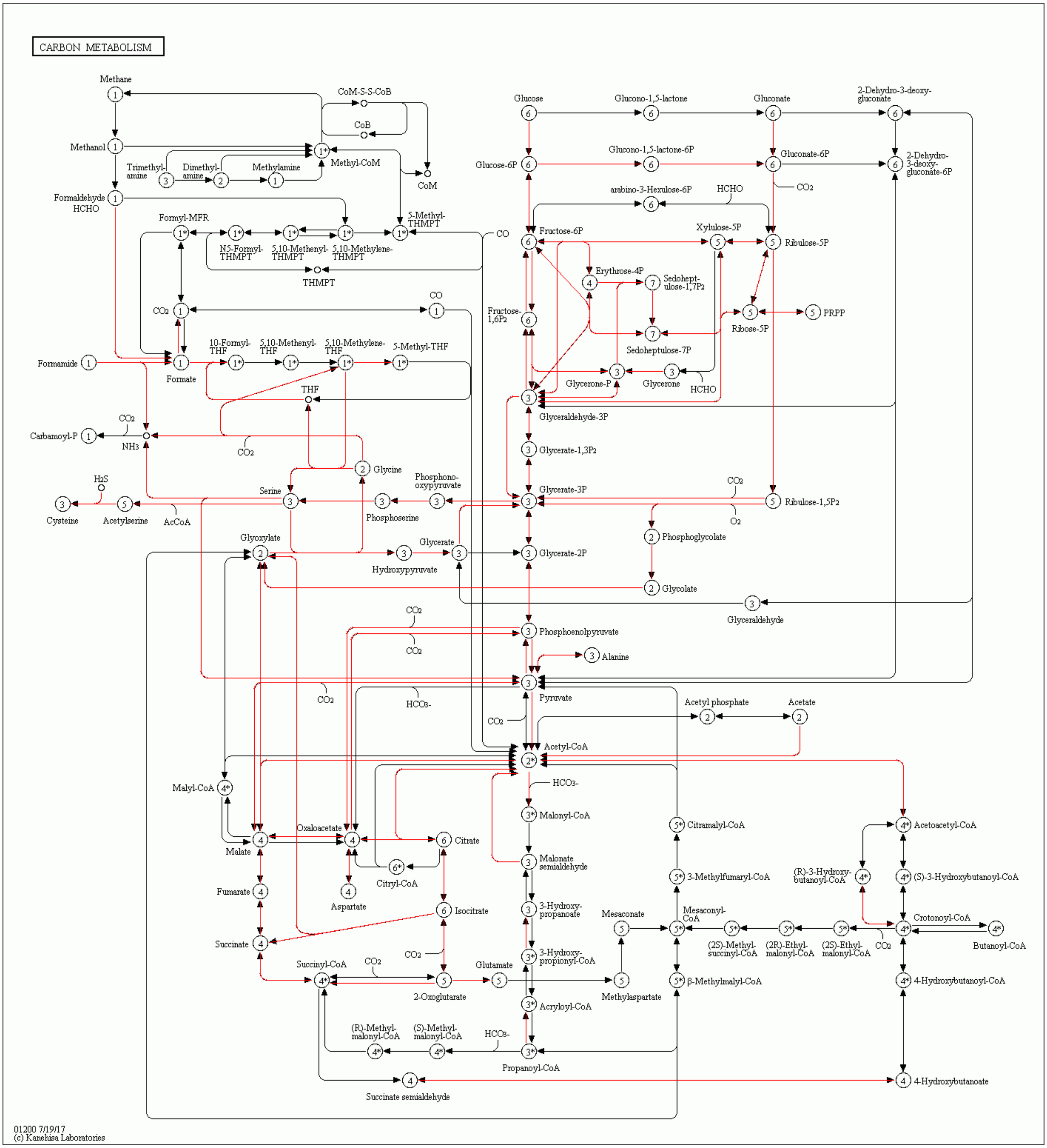
CARBON METABOLISM
Methane
CoM-S-S-CoB
CoB
Methanol
Methyl-CoM
CoM
Trimethyl-amine
Dimethyl-amine
Methylamine
Formaldehyde
HCHO
Formyl-MFR
N5-Formyl-THMPT
5,10-Methenyl-THMPT
5,10-Methylene-THMPT
5-Methyl-THMPT
CO
THMPT
CO_2
10-Formyl-THF
5,10-Methenyl-THF
5,10-Methylene-THF
5-Methyl-THF
Formamide
Formate
THF
Carbamoyl-P
NH_3
Glycine
Phosphono-oxypyruvate
H_2S
Serine
Phosphoserine
Cysteine
Acetylserine
AcCoA
Glyoxylate
Glycerate
Hydroxypyruvate
Glucose
Glucono-1,5-lactone
Gluconate
2-Dehydro-3-deoxy-gluconate
Glucose-6P
Glucono-1,5-lactone-6P
Gluconate-6P
2-Dehydro-3-deoxy-gluconate-6P
arabino-3-Hexulose-6P
Xylulose-5P
Ribulose-5P
Fructose-6P
Erythrose-4P
Sedohept-ulose-1,7P_2
Fructose-1,6P_2
Ribose-5P
PRPP
Sedoheptulose-7P
Glycerone-P
Glycerone
Glyceraldehyde-3P
Glycerate-1,3P_2
Glycerate-3P
Ribulose-1,5P_2
O_2
Phosphoglycolate
Glycerate-2P
Glycolate
Glyceraldehyde
Phosphoenolpyruvate
Alanine
Pyruvate
HCO_3^-
Acetyl phosphate
Acetate
Acetyl-CoA
Malyl-CoA
Malonyl-CoA
Citramalyl-CoA
Acetoacetyl-CoA
Oxaloacetate
Malate
Citrate
Citryl-CoA
Malonate semialdehyde
3-Methylfumaryl-CoA
(R)-3-Hydroxy-butanoyl-CoA
(S)-3-Hydroxybutanoyl-CoA
Fumarate
Aspartate
Isocitrate
3-Hydroxy-propanoate
Mesaconate
Mesaconyl-CoA
Crotonoyl-CoA
Butanoyl-CoA
Succinate
3-Hydroxy-propionyl-CoA
(2S)-Methyl-succinyl-CoA
(2R)-Ethyl-malonyl-CoA
(2S)-Ethyl-malonyl-CoA
Succinyl-CoA
Glutamate
2-Oxoglutarate
Methylaspartate
β-Methylmalyl-CoA
4-Hydroxybutanoyl-CoA
Acryloyl-CoA
(R)-Methyl-malonyl-CoA
(S)-Methyl-malonyl-CoA
Propanoyl-CoA
Succinate semialdehyde
4-Hydroxybutanoate
01200 7/19/17
(c) Kanehisa Laboratories

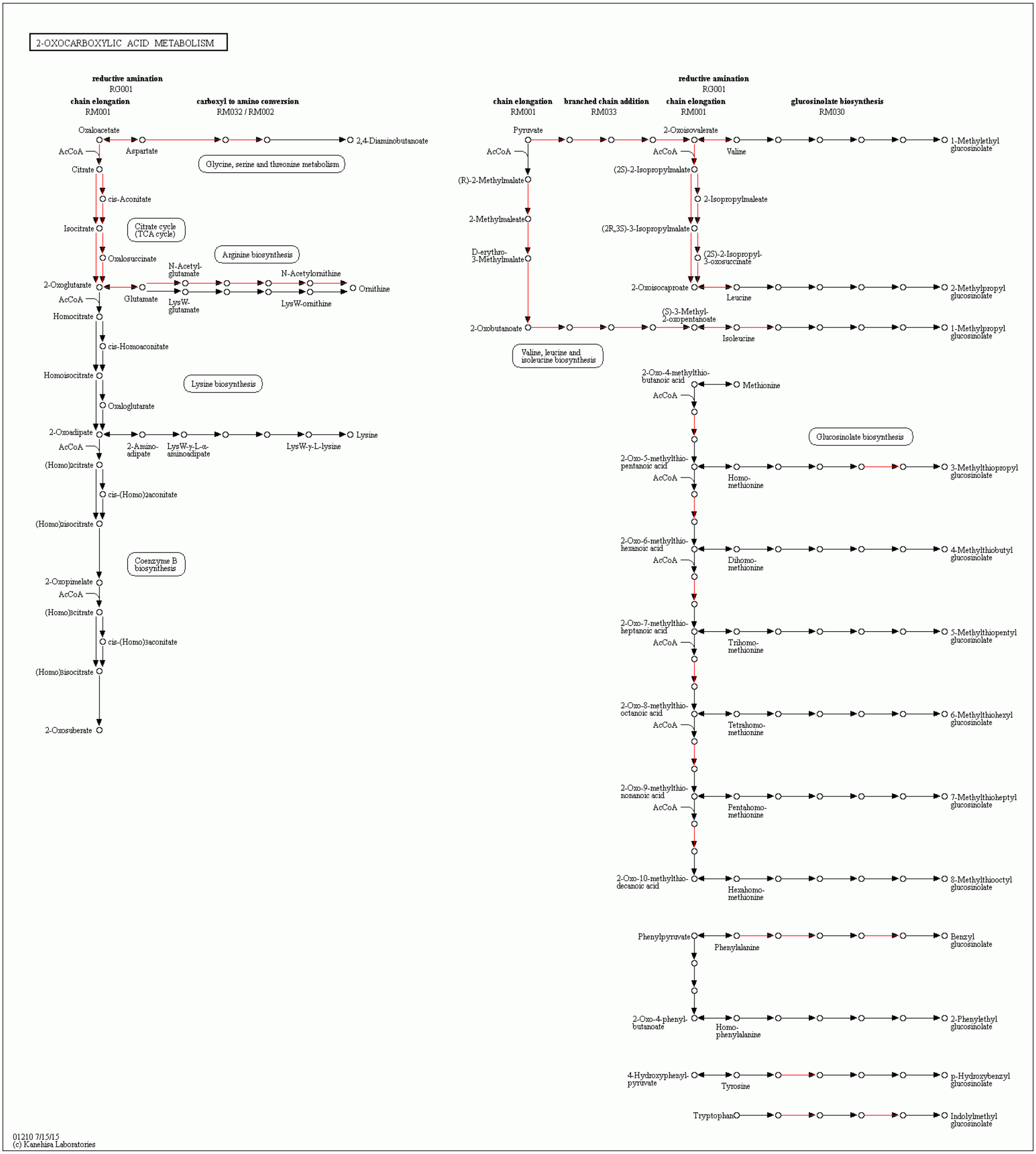
2-OXOCARBOXYLIC ACID METABOLISM
reductive amination
RG001
chain elongation
RM001
carboxyl to amino conversion
RM032 / RM002
Oxaloacetate
Aspartate
2,4-Diaminobutanoate
AcCoA
Citrate
Glycine, serine and threonine metabolism
cis-Aconitate
Isocitrate
Citrate cycle (TCA cycle)
Oxalosuccinate
Arginine biosynthesis
N-Acetyl-glutamate
N-Acetylornithine
2-Oxoglutarate
Glutamate
LysW-glutamate
LysW-ornithine
Ornithine
Homocitrate
cis-Homoaconitate
Homoisocitrate
Lysine biosynthesis
Oxaloglutarate
2-Oxoadipate
2-Amino-adipate
LysW-γ-L-α-aminoadipate
LysW-γ-L-lysine
Lysine
(Homo)2citrate
cis-(Homo)2aconitate
(Homo)2isocitrate
Coenzyme B biosynthesis
2-Oxopimelate
(Homo)3citrate
cis-(Homo)3aconitate
(Homo)3isocitrate
2-Oxosuberate
chain elongation
RM001
branched chain addition
RM033
chain elongation
RM001
glucosinolate biosynthesis
RM030
Pyruvate
2-Oxoisovalerate
Valine
1-Methylethyl glucosinolate
(R)-2-Methylmalate
(2S)-2-Isopropylmalate
2-Isopropylmaleate
2-Methylmaleate
(2R,3S)-3-Isopropylmalate
D-erythro-3-Methylmalate
(2S)-2-Isopropyl-3-oxosuccinate
2-Oxoisocaproate
Leucine
2-Methylpropyl glucosinolate
(S)-3-Methyl-2-oxopentanoate
2-Oxobutanoate
Isoleucine
1-Methylpropyl glucosinolate
Valine, leucine and isoleucine biosynthesis
2-Oxo-4-methylthio-butanoic acid
Methionine
Glucosinolate biosynthesis
2-Oxo-5-methylthio-pentanoic acid
Homo-methionine
3-Methylthiopropyl glucosinolate
2-Oxo-6-methylthio-hexanoic acid
Dihomo-methionine
4-Methylthiobutyl glucosinolate
2-Oxo-7-methylthio-heptanoic acid
Trihomo-methionine
5-Methylthiopentyl glucosinolate
2-Oxo-8-methylthio-octanoic acid
Tetrahomo-methionine
6-Methylthiohexyl glucosinolate
2-Oxo-9-methylthio-nonanoic acid
Pentahomo-methionine
7-Methylthioheptyl glucosinolate
2-Oxo-10-methylthio-decanoic acid
Hexahomo-methionine
8-Methylthiooctyl glucosinolate
Phenylpyruvate
Phenylalanine
Benzyl glucosinolate
2-Oxo-4-phenyl-butanoate
Homo-phenylalanine
2-Phenylethyl glucosinolate
4-Hydroxyphenyl-pyruvate
Tyrosine
p-Hydroxybenzyl glucosinolate
Tryptophan
Indolylmethyl glucosinolate
01210 7/15/15
(c) Kanehisa Laboratories

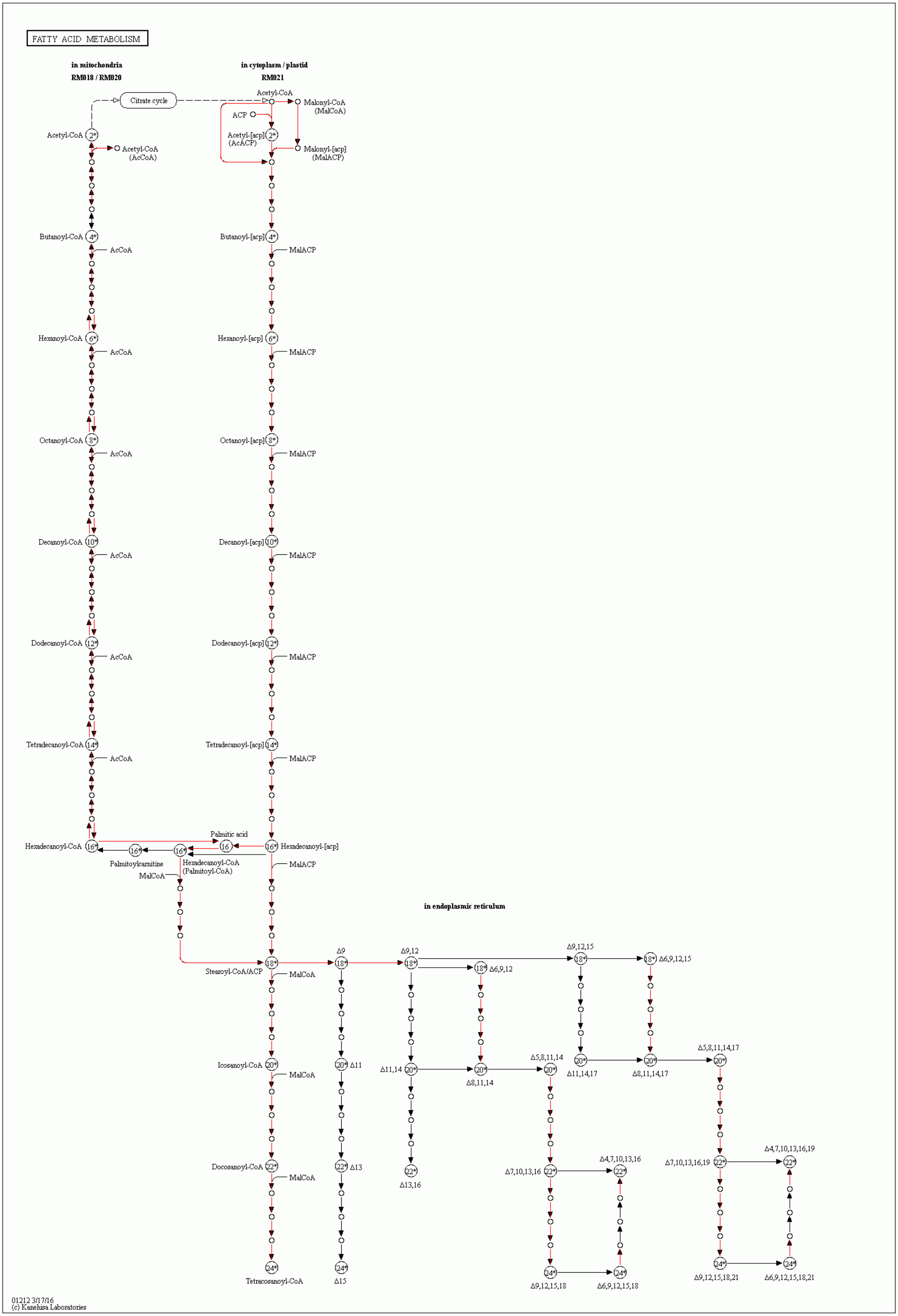
FATTY ACID METABOLISM
in mitochondria
RM018 / RM020
in cytoplasm / plastid
RM021
Citrate cycle
Acetyl-CoA
Malonyl-CoA
(MalCoA)
ACP
Acetyl-[acp]
(AcACP)
Malonyl-[acp]
(MalACP)
Acetyl-CoA
(AcCoA)
Butanoyl-CoA
Butanoyl-[acp]
Hexanoyl-CoA
Hexanoyl-[acp]
Octanoyl-CoA
Octanoyl-[acp]
Decanoyl-CoA
Decanoyl-[acp]
Dodecanoyl-CoA
Dodecanoyl-[acp]
Tetradecanoyl-CoA
Tetradecanoyl-[acp]
Hexadecanoyl-CoA
Hexadecanoyl-[acp]
AcCoA
MalACP
Palmitic acid
Palmitoylcarnitine
Hexadecanoyl-CoA
(Palmitoyl-CoA)
MalCoA
in endoplasmic reticulum
Stearoyl-CoA/ACP
Icosanoyl-CoA
Docosanoyl-CoA
Tetracosanoyl-CoA
Δ9
Δ9,12
Δ6,9,12
Δ9,12,15
Δ6,9,12,15
Δ11
Δ11,14
Δ8,11,14
Δ5,8,11,14
Δ11,14,17
Δ8,11,14,17
Δ5,8,11,14,17
Δ13
Δ13,16
Δ7,10,13,16
Δ4,7,10,13,16
Δ7,10,13,16,19
Δ4,7,10,13,16,19
Δ15
Δ9,12,15,18
Δ6,9,12,15,18
Δ9,12,15,18,21
Δ6,9,12,15,18,21
01212 3/17/16
(c) Kanehisa Laboratories

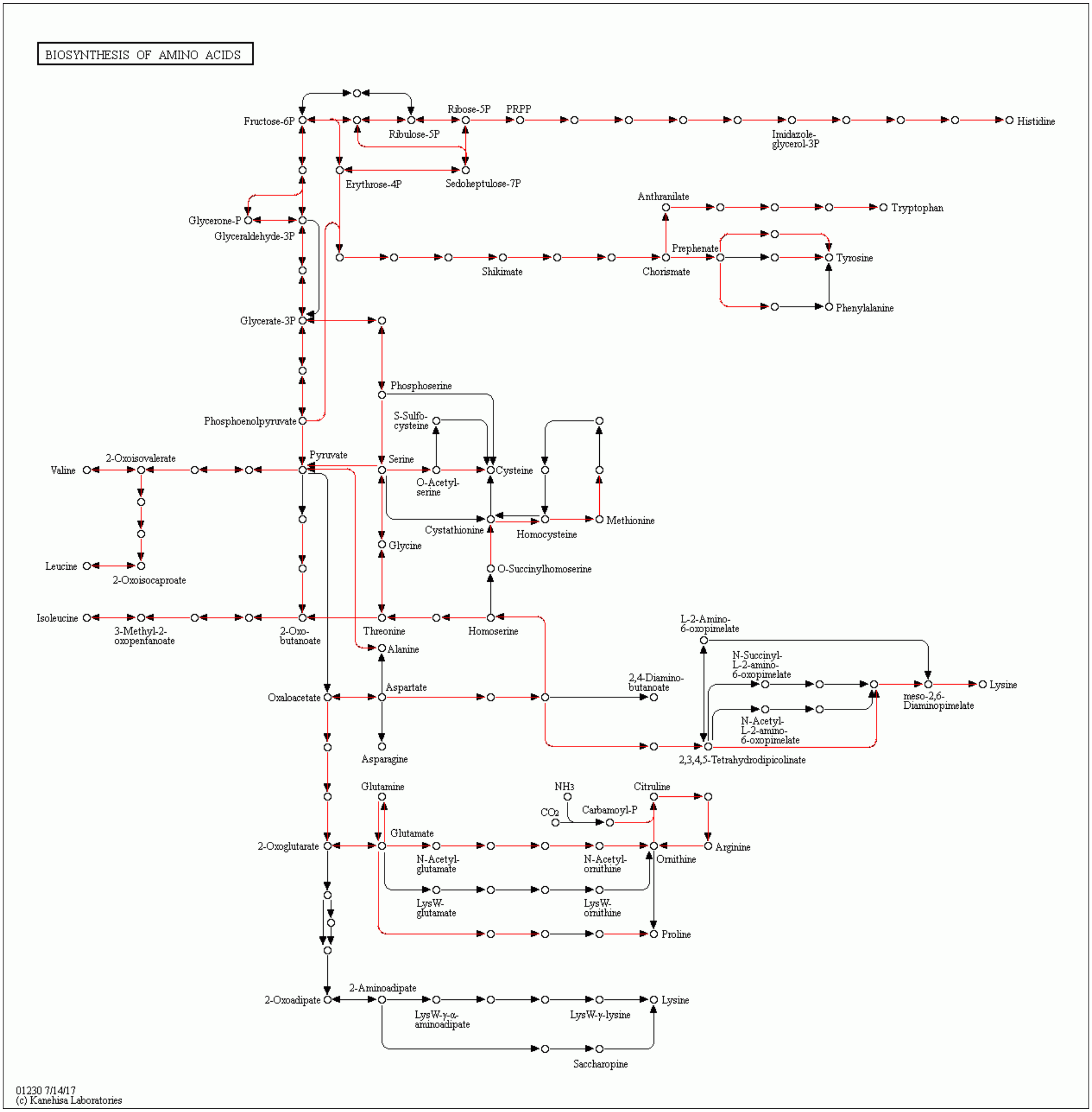
BIOSYNTHESIS OF AMINO ACIDS
Fructose-6P
Ribose-5P
PRPP
Ribulose-5P
Histidine
Imidazole-glycerol-3P
Erythrose-4P
Sedoheptulose-7P
Anthranilate
Tryptophan
Glycerone-P
Glyceraldehyde-3P
Prephenate
Tyrosine
Shikimate
Chorismate
Phenylalanine
Glycerate-3P
Phosphoserine
S-Sulfo-cysteine
Phosphoenolpyruvate
2-Oxoisovalerate
Pyruvate
Serine
Valine
O-Acetyl-serine
Cysteine
Methionine
Cystathionine
Homocysteine
Glycine
Leucine
2-Oxoisocaproate
O-Succinylhomoserine
Isoleucine
3-Methyl-2-oxopentanoate
2-Oxo-butanoate
Threonine
Homoserine
L-2-Amino-6-oxopimelate
Alanine
N-Succinyl-L-2-amino-6-oxopimelate
2,4-Diamino-butanoate
Aspartate
Oxaloacetate
Lysine
meso-2,6-Diaminopimelate
N-Acetyl-L-2-amino-6-oxopimelate
Asparagine
2,3,4,5-Tetrahydrodipicolinate
Glutamine
NH3
Citruline
CO2
Carbamoyl-P
Glutamate
2-Oxoglutarate
Arginine
N-Acetyl-glutamate
N-Acetyl-ornithine
Ornithine
LysW-glutamate
LysW-ornithine
Proline
2-Aminoadipate
2-Oxoadipate
Lysine
LysW-γ-α-aminoadipate
LysW-γ-lysine
Saccharopine
01230 7/14/17
(c) Kanehisa Laboratories

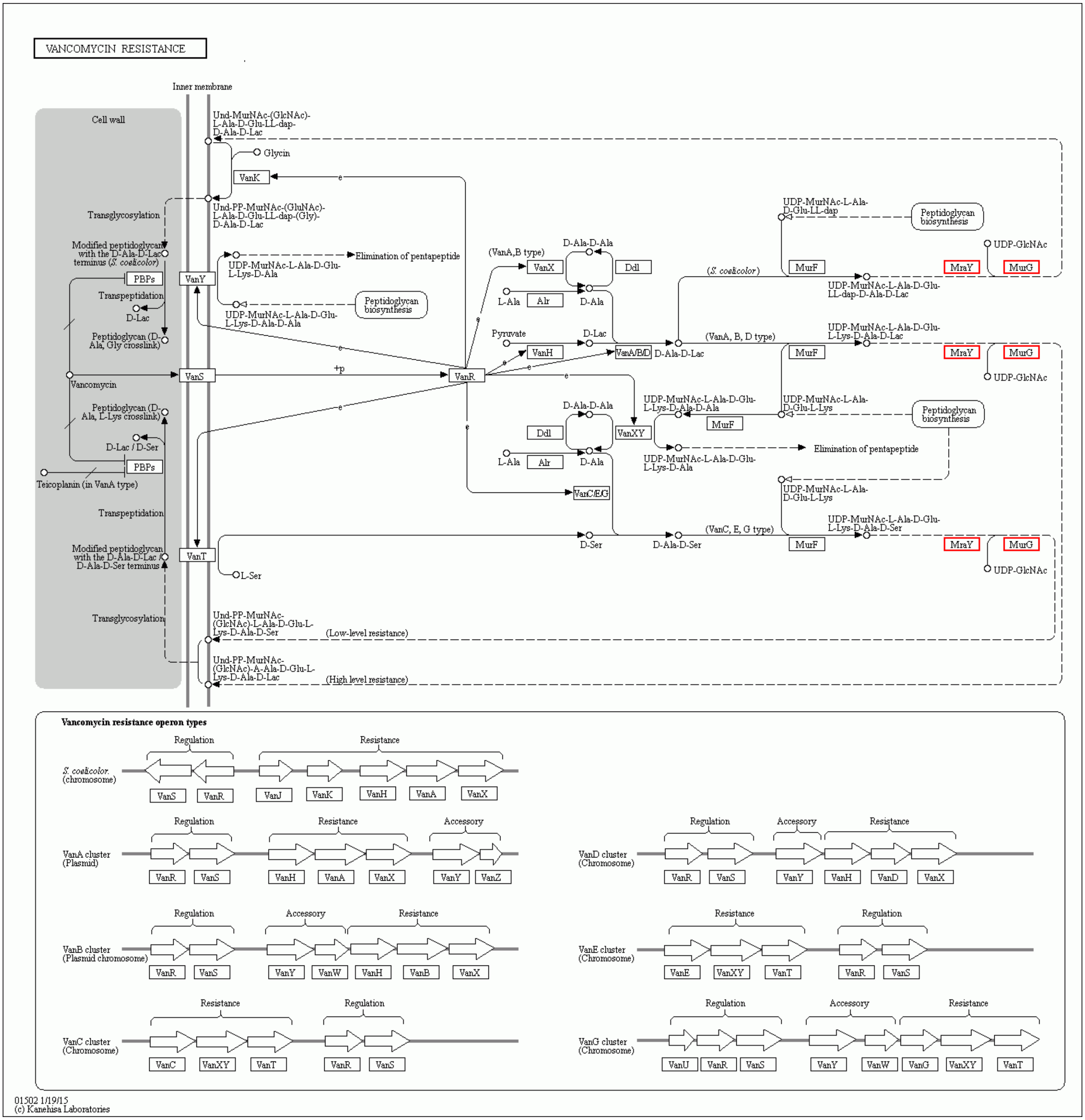
VANCOMYCIN RESISTANCE
Inner membrane
Cell wall
Und-MurNAc-(GlcNAc)-L-Ala-D-Glu-LL-dap-D-Ala-D-Lac
Glycin
VanK
Und-PP-MurNAc-(GluNAc)-L-Ala-D-Glu-LL-dap-(Gly)-D-Ala-D-Lac
Transglycosylation
Modified peptidoglycan with the D-Ala-D-Lac terminus (S. coelicolor)
PBPs
VanY
Transpeptidation
D-Lac
Peptidoglycan (D-Ala, Gly crosslink)
UDP-MurNAc-L-Ala-D-Glu-L-Lys-D-Ala
Elimination of pentapeptide
UDP-MurNAc-L-Ala-D-Glu-L-Lys-D-Ala-D-Ala
Peptidoglycan biosynthesis
(VanA,B type)
D-Ala-D-Ala
VanX
Ddl
L-Ala
Alr
D-Ala
Pyruvate
D-Lac
VanH
VanA/B/D
D-Ala-D-Lac
Vancomycin
VanS
+p
VanR
UDP-MurNAc-L-Ala-D-Glu-LL-dap
(S. coelicolor)
MurF
MraY
MurG
UDP-GlcNAc
UDP-MurNAc-L-Ala-D-Glu-LL-dap-D-Ala-D-Lac
(VanA, B, D type)
UDP-MurNAc-L-Ala-D-Glu-L-Lys-D-Ala-D-Lac
Peptidoglycan (D-Ala, L-Lys crosslink)
D-Lac / D-Ser
Teicoplanin (in VanA type)
VanXY
UDP-MurNAc-L-Ala-D-Glu-L-Lys-D-Ala-D-Ala
UDP-MurNAc-L-Ala-D-Glu-L-Lys
UDP-MurNAc-L-Ala-D-Glu-L-Lys-D-Ala
VanC/E/G
UDP-MurNAc-L-Ala-D-Glu-L-Lys
Modified peptidoglycan with the D-Ala-D-Lac / D-Ala-D-Ser terminus
VanT
D-Ser
D-Ala-D-Ser
(VanC, E, G type)
UDP-MurNAc-L-Ala-D-Glu-L-Lys-D-Ala-D-Ser
L-Ser
Und-PP-MurNAc-(GlcNAc)-L-Ala-D-Glu-L-Lys-D-Ala-D-Ser
(Low-level resistance)
Und-PP-MurNAc-(GlcNAc)-A-Ala-D-Glu-L-Lys-D-Ala-D-Lac
(High level resistance)
Vancomycin resistance operon types
Regulation
Resistance
Accessory
S. coelicolor. (chromosome)
VanS VanR VanJ VanK VanH VanA VanX
VanA cluster (Plasmid)
VanR VanS VanH VanA VanX VanY VanZ
VanD cluster (Chromosome)
VanR VanS VanY VanH VanD VanX
VanB cluster (Plasmid chromosome)
VanR VanS VanY VanW VanH VanB VanX
VanE cluster (Chromosome)
VanE VanXY VanT VanR VanS
VanC cluster (Chromosome)
VanC VanXY VanT VanR VanS
VanG cluster (Chromosome)
VanU VanR VanS VanY VanW VanG VanXY VanT
01502 1/19/15
(c) Kanehisa Laboratories

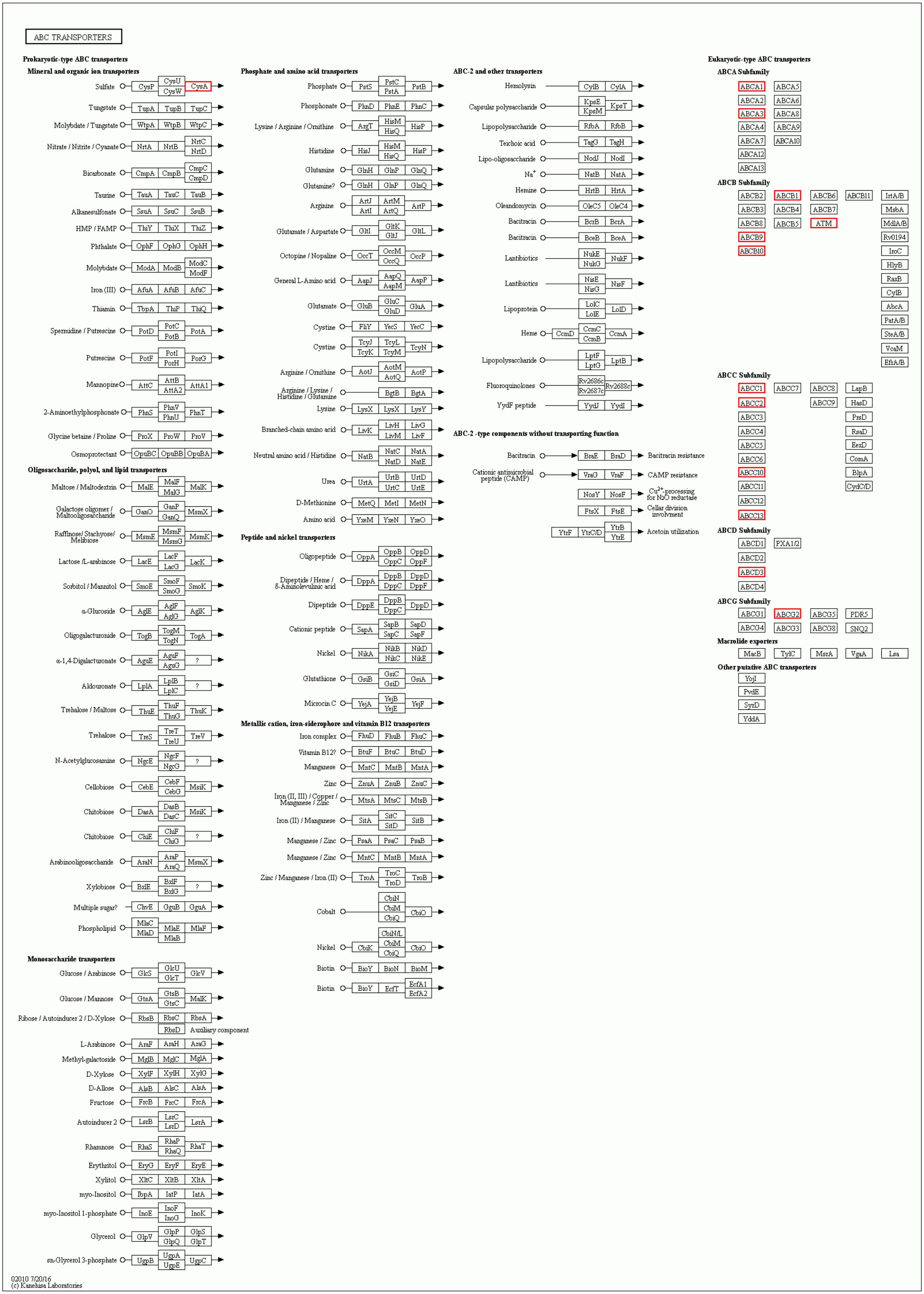
ABC TRANSPORTERS
Prokaryotic-type ABC transporters
Mineral and organic ion transporters
Sulfate CysP CysU CysW CysA
Tungstate TupA TupB TupC
Molybdate / Tungstate WtpA WtpB WtpC
Nitrate / Nitrite / Cyanate NrtA NrtB NrtC NrtD
Bicarbonate CmpA CmpB CmpC CmpD
Taurine TauA TauC TauB
Alkanesulfonate SsuA SsuC SsuB
HMP / FAMP ThiY ThiX ThiZ
Phthalate OphF OphG OphH
Molybdate ModA ModB ModC ModF
Iron (III) AfuA AfuB AfuC
Thiamin TbpA ThiP ThiQ
Spermidine / Putrescine PotD PotC PotB PotA
Putrescine PotF PotI PotH PotG
Mannopine AttC AttB AttA2 AttA1
2-Aminoethylphosphonate PhnS PhnV PhnU PhnT
Glycine betaine / Proline ProX ProW ProV
Osmoprotectant OpuBC OpuBB OpuBA
Oligosaccharide, polyol, and lipid transporters
Maltose / Maltodextrin MalE MalF MalG MalK
Galactose oligomer / Maltooligosaccharide GanO GanP GanQ MsmX
Raffinose/ Stachyose/ Melibiose MsmE MsmF MsmG MsmK
Lactose /L-arabinose LacE LacF LacG LacK
Sorbitol / Mannitol SmoE SmoF SmoG SmoK
α-Glucoside AglE AglF AglG AglK
Oligogalacturonide TogB TogM TogN TogA
α-1,4-Digalacturonate AguE AguF AguG ?
Aldouronate LplA LplB LplC ?
Trehalose / Maltose ThuE ThuF ThuG ThuK
Trehalose TreS TreT TreU TreV
N-Acetylglucosamine NgcE NgcF NgcG ?
Cellobiose CebE CebF CebG MsiK
Chitobiose DasA DasB DasC MsiK
Chitobiose ChiE ChiF ChiG ?
Arabinooligosaccharide AraN AraP AraQ MsmX
Xylobiose BxlE BxlF BxlG ?
Multiple sugar? ChvE GguB GguA
Phospholipid MlaC MlaD MlaE MlaB MlaF
Monosaccharide transporters
Glucose / Arabinose GlcS GlcU GlcT GlcV
Glucose / Mannose GtsA GtsB GtsC MalK
Ribose / Autoinducer 2 / D-Xylose RbsB RbsC RbsA RbsD Auxiliary component
L-Arabinose AraF AraH AraG
Methyl-galactoside MglB MglC MglA
D-Xylose XylF XylH XylG
D-Allose AlsB AlsC AlsA
Fructose FrcB FrcC FrcA
Autoinducer 2 LsrB LsrC LsrD LsrA
Rhamnose RhaS RhaP RhaQ RhaT
Erythritol EryG EryF EryE
Xylitol XltC XltB XltA
myo-Inositol IbpA IatP IatA
myo-Inositol 1-phosphate InoE InoF InoG InoK
Glycerol GlpV GlpP GlpQ GlpS GlpT
sn-Glycerol 3-phosphate UgpB UgpA UgpE UgpC
Phosphate and amino acid transporters
Phosphate PstS PstC PstA PstB
Phosphonate PhnD PhnE PhnC
Lysine / Arginine / Ornithine ArgT HisM HisQ HisP
Histidine HisJ HisM HisQ HisP
Glutamine GlnH GlnP GlnQ
Glutamine? GlnH GlnP GlnQ
Arginine ArtJ ArtI ArtM ArtQ ArtP
Glutamate / Aspartate GltI GltK GltJ GltL
Octopine / Nopaline OccT OccM OccQ OccP
General L-Amino acid AapJ AapQ AapM AapP
Glutamate GluB GluC GluD GluA
Cystine FliY YecS YecC
Cystine TcyJ TcyK TcyL TcyM TcyN
Arginine / Ornithine AotJ AotM AotQ AotP
Arginine / Lysine / Histidine / Glutamine BgtB BgtA
Lysine LysX LysX LysY
Branched-chain amino acid LivK LivH LivM LivG LivF
Neutral amino acid / Histidine NatB NatC NatD NatA NatE
Urea UrtA UrtB UrtC UrtD UrtE
D-Methionine MetQ MetI MetN
Amino acid YxeM YxeN YxeO
Peptide and nickel transporters
Oligopeptide OppA OppB OppC OppD OppF
Dipeptide / Heme / δ-Aminolevulinic acid DppA DppB DppC DppD DppF
Dipeptide DppE DppB DppC DppD
Cationic peptide SapA SapB SapC SapD SapF
Nickel NikA NikB NikC NikD NikE
Glutathione GsiB GsiC GsiD GsiA
Microcin C YejA YejB YejE YejF
Metallic cation, iron-siderophore and vitamin B12 transporters
Iron complex FhuD FhuB FhuC
Vitamin B12? BtuF BtuC BtuD
Manganese MntC MntB MntA
Zinc ZnuA ZnuB ZnuC
Iron (II, III) / Copper / Manganese / Zinc MtsA MtsC MtsB
Iron (II) / Manganese SitA SitC SitD SitB
Manganese / Zinc PsaA PsaC PsaB
Manganese / Zinc MntC MntB MntA
Zinc / Manganese / Iron (II) TroA TroC TroD TroB
Cobalt CbiN CbiM CbiQ CbiO
Nickel NikK CbiN/L CbiM CbiQ CbiO
Biotin BioY BioN BioM
Biotin BioY EcfT EcfA1 EcfA2
ABC-2 and other transporters
Hemolysin CylB CylA
Capsular polysaccharide KpsE KpsM KpsT
Lipopolysaccharide RfbA RfbB
Teichoic acid TagG TagH
Lipo-oligosaccharide NodJ NodI
Na+ NatB NatA
Hemine HrtB HrtA
Oleandomycin OleC5 OleC4
Bacitracin BcrB BcrA
Bacitracin BceB BceA
Lantibiotics NukE NukG NukF
Lantibiotics NisE NisG NisF
Lipoprotein LolC LolE LolD
Heme CcmD CcmC CcmB CcmA
Lipopolysaccharide LptF LptG LptB
Fluoroquinolones Rv2686c Rv2687c Rv2688c
YydF peptide YydJ YydI
ABC-2 -type components without transporting function
Bacitracin BraE BraD Bacitracin resistance
Cationic antimicrobial peptide (CAMP) VraG VraF CAMP resistance
NosY NosF Cu2+-processing for N2O reductase
FtsX FtsE Cellar division involvment
YtrF YtrC/D YtrB YtrE Acetoin utilization
Eukaryotic-type ABC transporters
ABCA Subfamily
ABCA1 ABCA5
ABCA2 ABCA6
ABCA3 ABCA8
ABCA4 ABCA9
ABCA7 ABCA10
ABCA12
ABCA13
ABCB Subfamily
ABCB2 ABCB1 ABCB6 ABCB11 IrtA/B
ABCB3 ABCB4 ABCB7 MsbA
ABCB8 ABCB5 ATM MdlA/B
ABCB9 Rv0194
ABCB10 IroC
HlyB
RaxB
CylB
AbcA
PatA/B
SteA/B
VcaM
EfrA/B
ABCC Subfamily
ABCC1 ABCC7 ABCC8 LapB
ABCC2 ABCC9 HasD
ABCC3 PrsD
ABCC4 RsaD
ABCC5 EexD
ABCC6 ComA
ABCC10 BlpA
ABCC11 CydC/D
ABCC12
ABCC13
ABCD Subfamily
ABCD1 PXA1/2
ABCD2
ABCD3
ABCD4
ABCG Subfamily
ABCG1 ABCG2 ABCG5 PDR5
ABCG4 ABCG3 ABCG8 SNQ2
Macrolide exporters
MacB TylC MsrA VgaA Lsa
Other putative ABC transporters
YojI
PvdE
SyrD
YddA
02010 7/20/16
(c) Kanehisa Laboratories

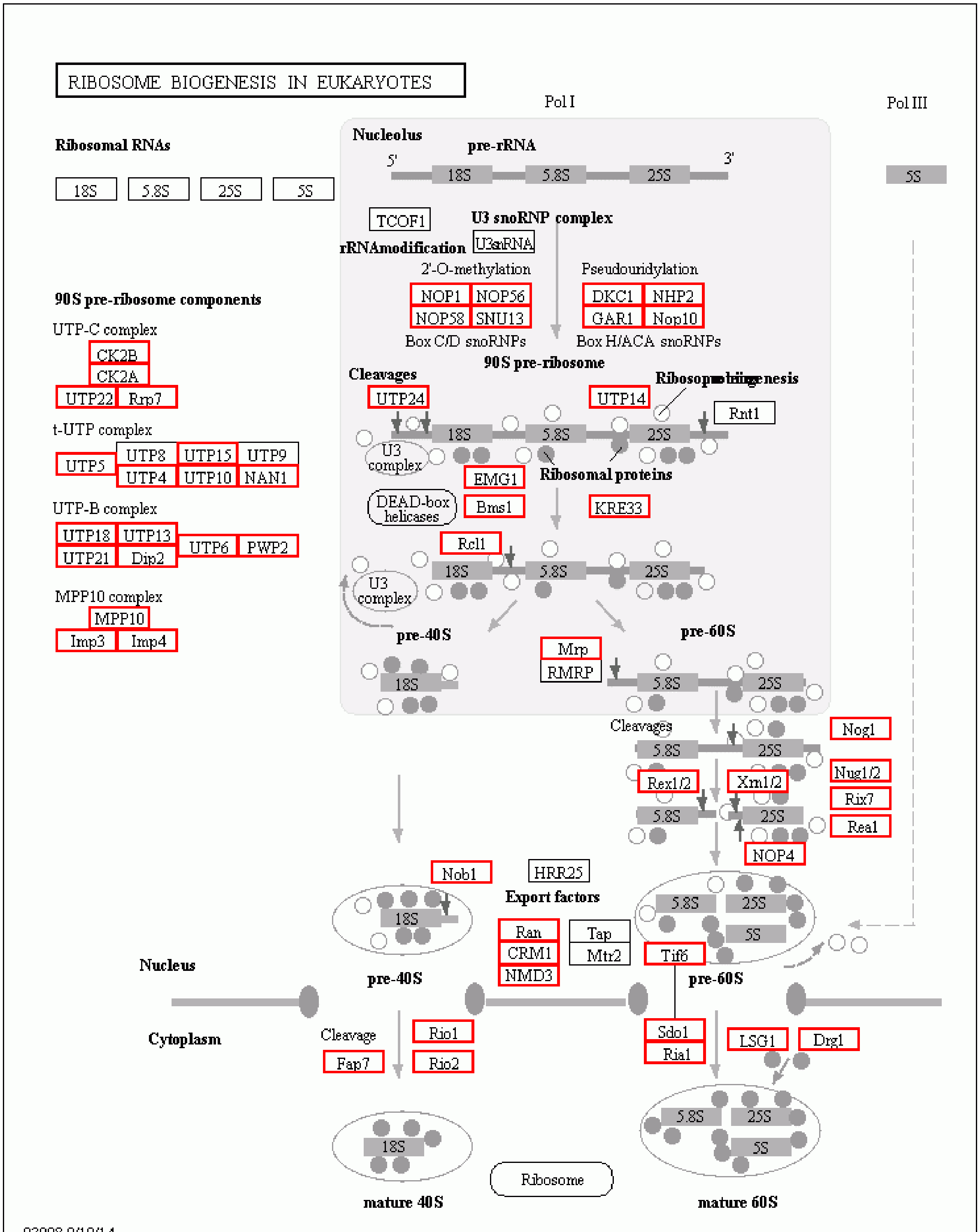

03008 9/10/14
(c) Kanehisa Laboratories

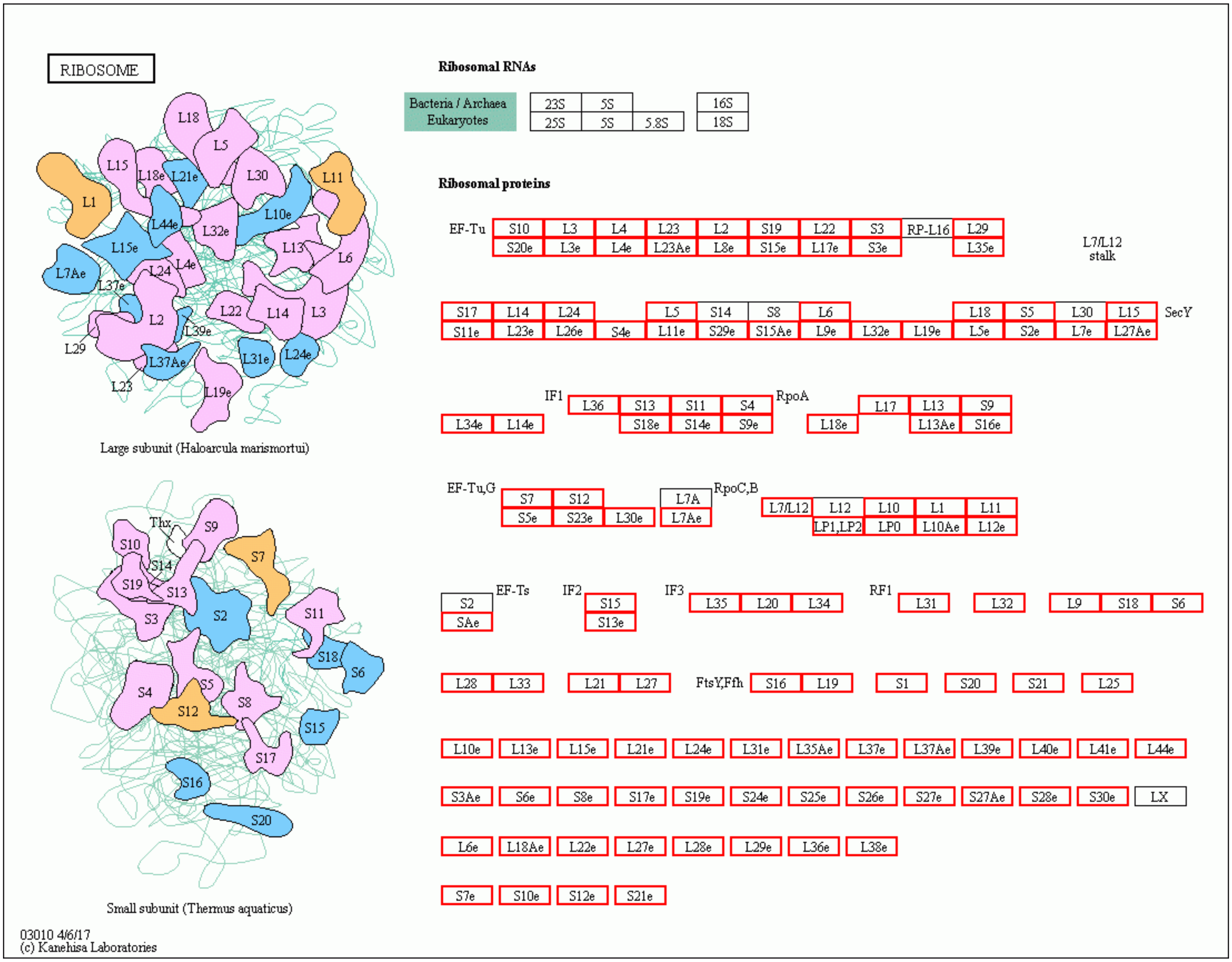
RIBOSOME
Ribosomal RNAs
Bacteria / Archaea
Eukaryotes
23S
5S
16S
25S
5S
5.8S
18S
Ribosomal proteins
EF-Tu
S10 L3 L4 L23 L2 S19 L22 S3 RP-L16 L29
S20e L3e L4e L23Ae L8e S15e L17e S3e L35e
L7/L12 stalk
S17 L14 L24 L5 S14 S8 L6 L18 S5 L30 L15
SecY
S11e L23e L26e S4e L11e S29e S15Ae L9e L32e L19e L5e S2e L7e L27Ae
IF1
RpoA
L36 S13 S11 S4 L17 L13 S9
L34e L14e S18e S14e S9e L18e L13Ae S16e
EF-Tu,G
RpoC,B
S7 S12 L7A
S5e S23e L30e L7Ae
L7/L12 L12 L10 L1 L11
LP1,LP2 LP0 L10Ae L12e
EF-Ts
IF2
IF3
RF1
S2 S15 L35 L20 L34 L31 L32 L9 S18 S6
SAe S13e
L28 L33 L21 L27
FtsY,Ffh
S16 L19 S1 S20 S21 L25
L10e L13e L15e L21e L24e L31e L35Ae L37e L37Ae L39e L40e L41e L44e
S3Ae S6e S8e S17e S19e S24e S25e S26e S27e S27Ae S28e S30e LX
L6e L18Ae L22e L27e L28e L29e L36e L38e
S7e S10e S12e S21e
L18 L5 L15 L18e L21e L30 L11 L1 L44e L32e L10e L15e L13 L6 L7Ae L4e L24 L37e L22 L14 L3 L2 L39e L29 L37Ae L31e L24e L23 L19e
Large subunit (Haloarcula marismortui)
Thx S9 S10 S14 S7 S19 S13 S2 S3 S11 S18 S6 S4 S5 S8 S12 S15 S17 S16 S20
Small subunit (Thermus aquaticus)
03010 4/6/17
(c) Kanehisa Laboratories

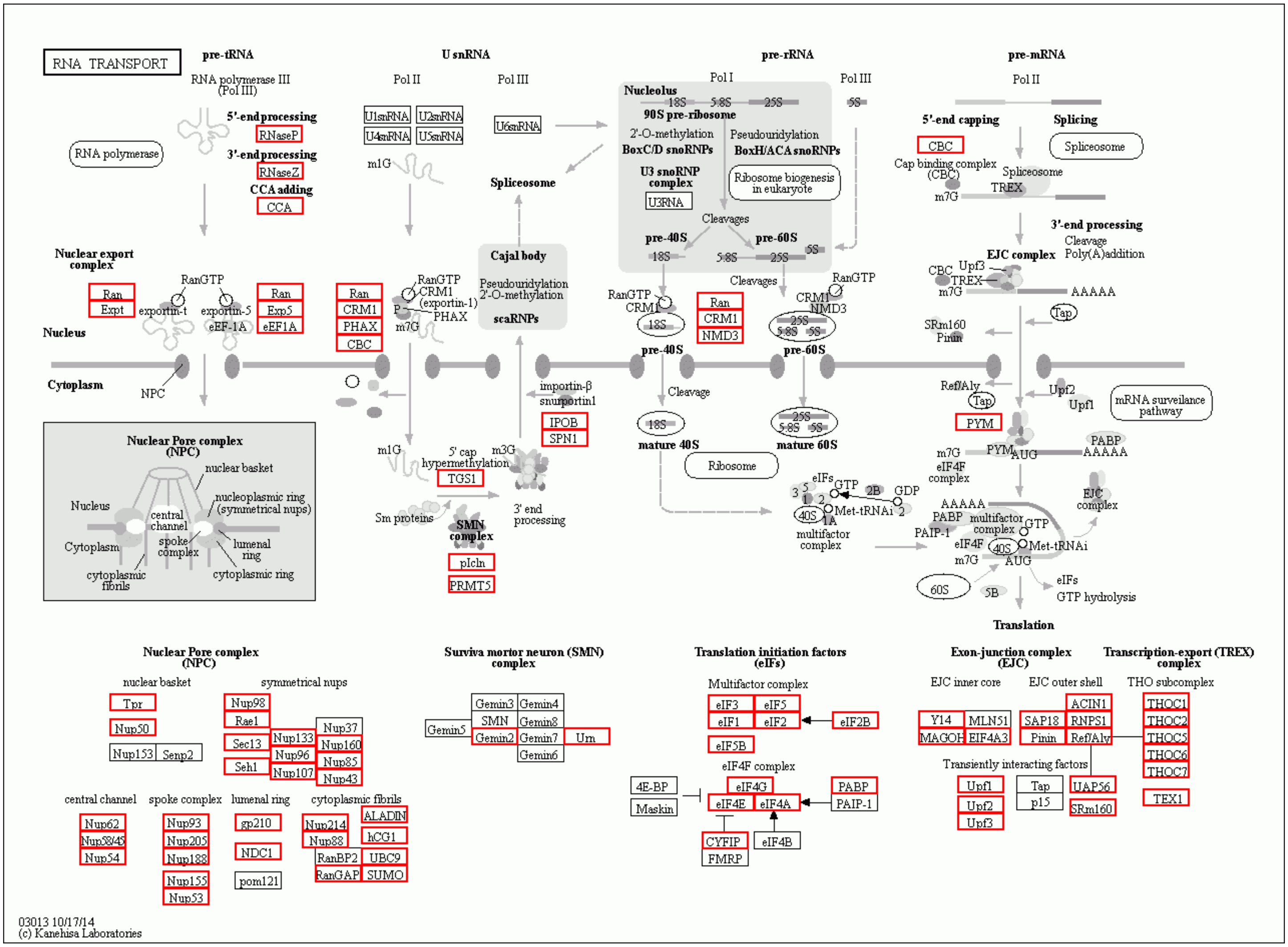
RNA TRANSPORT
pre-tRNA
RNA polymerase III (Pol III)
5'-end processing
RNaseP
3'-end processing
RNaseZ
CCA adding
CCA
RNA polymerase
U snRNA
Pol II
Pol III
U1snRNA
U2snRNA
U4snRNA
U5snRNA
U6snRNA
m1G
Spliceosome
pre-rRNA
Nucleolus
Pol I
18S
5.8S
25S
5S
90S pre-ribosome
2'-O-methylation
BoxC/D snoRNPs
Pseudouridylation
BoxH/ACA snoRNPs
U3 snoRNP complex
U3RNA
Ribosome biogenesis in eukaryote
Cleavages
pre-40S
pre-60S
pre-mRNA
5'-end capping
CBC
Cap binding complex (CBC)
Splicing
Spliceosome
TREX
m7G
3'-end processing
Cleavage
Poly(A)addition
EJC complex
Upf3
AAAAA
Tap
SRm160
Pinin
Nuclear export complex
Ran
Expt
RanGTP
exportin-t
exportin-5
eEF-1A
Exp5
eEF1A
CRM1
PHAX
(exportin-1)
P
Cajal body
Pseudouridylation
2'-O-methylation
scaRNPs
NMD3
Nucleus
Cytoplasm
NPC
importin-β
snurportin1
IPOB
SPN1
m3G
5' cap hypermethylation
TGS1
3' end processing
Sm proteins
SMN complex
pICln
PRMT5
Cleavage
mature 40S
mature 60S
Ribosome
Ref/Aly
PYM
Upf2
Upf1
mRNA surveilance pathway
AUG
PABP
eIF4F complex
eIFs
GTP
GDP
2B
Met-tRNAi
40S
multifactor complex
PAIP-1
EJC complex
60S
5B
GTP hydrolysis
Translation
Nuclear Pore complex (NPC)
nuclear basket
nucleoplasmic ring (symmetrical nups)
central channel
spoke complex
lumenal ring
cytoplasmic fibrils
cytoplasmic ring
Nuclear Pore complex (NPC)
nuclear basket
symmetrical nups
Tpr
Nup50
Nup153
Senp2
Nup98
Rae1
Sec13
Seh1
Nup133
Nup96
Nup107
Nup37
Nup160
Nup85
Nup43
central channel
spoke complex
lumenal ring
cytoplasmic fibrils
Nup62
Nup58/45
Nup54
Nup93
Nup205
Nup188
Nup155
Nup53
gp210
NDC1
pom121
Nup214
Nup88
ALADIN
hCG1
RanBP2
UBC9
RanGAP
SUMO
Surviva mortor neuron (SMN) complex
Gemin3
Gemin4
SMN
Gemin8
Gemin5
Gemin2
Gemin7
Urn
Gemin6
Translation initiation factors (eIFs)
Multifactor complex
eIF3
eIF5
eIF1
eIF2
eIF2B
eIF5B
eIF4F complex
4E-BP
Maskin
eIF4G
eIF4E
eIF4A
PABP
PAIP-1
CYFIP
FMRP
eIF4B
Exon-junction complex (EJC)
EJC inner core
EJC outer shell
Y14
MAGOH
MLN51
EIF4A3
SAP18
Pinin
ACIN1
RNPS1
Ref/Aly
Transiently interacting factors
Upf1
Upf2
Upf3
Tap
p15
UAP56
SRm160
Transcription-export (TREX) complex
THO subcomplex
THOC1
THOC2
THOC5
THOC6
THOC7
TEX1
03013 10/17/14
(c) Kanehisa Laboratories

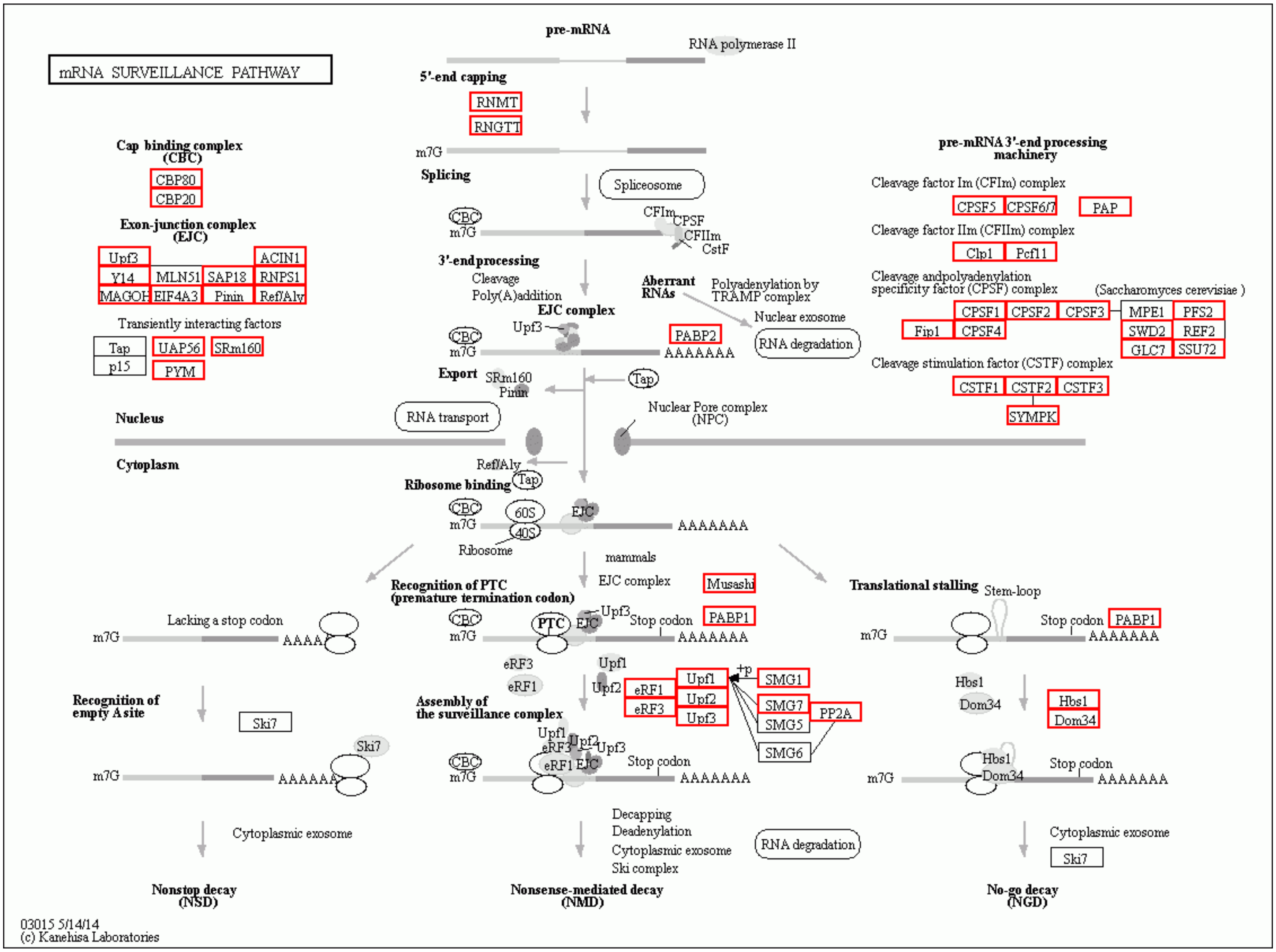

mRNA SURVEILLANCE PATHWAY
pre-mRNA
RNA polymerase II
5'-end capping
RNMT
RNGTT
m7G
Splicing
Spliceosome
Cap binding complex (CBC)
CBP80
CBP20
Exon-junction complex (EJC)
Upf3
ACIN1
Y14
MLN51
SAP18
RNPS1
MAGOH
EIF4A3
Pinin
Ref/Aly
Transiently interacting factors
Tap
UAP56
SRm160
p15
PYM
CBC
CFIm
CPSF
CFIIm
CstF
3'-end processing
Cleavage
Poly(A)addition
EJC complex
Upf3
PABP2
AAAAAAA
Aberrant RNAs
Polyadenylation by TRAMP complex
Nuclear exosome
RNA degradation
Export
SRm160
Pinin
Tap
RNA transport
Nuclear Pore complex (NPC)
Nucleus
Cytoplasm
Ref/Aly
Ribosome binding
60S
40S
EJC
Ribosome
pre-mRNA 3'-end processing machinery
Cleavage factor Im (CFIm) complex
CPSF5
CPSF6/7
PAP
Cleavage factor IIm (CFIIm) complex
Clp1
Pcf11
Cleavage and polyadenylation specificity factor (CPSF) complex
(Saccharomyces cerevisiae)
CPSF1
CPSF2
CPSF3
MPE1
PFS2
Fip1
CPSF4
SWD2
REF2
GLC7
SSU72
Cleavage stimulation factor (CSTF) complex
CSTF1
CSTF2
CSTF3
SYMPK
mammals
EJC complex
Musashi
Recognition of PTC (premature termination codon)
Translational stalling
Stem-loop
Lacking a stop codon
AAAA
PTC
Upf3
Stop codon
PABP1
eRF3
Upf1
eRF1
Upf2
+p
SMG1
SMG7
SMG5
PP2A
SMG6
Recognition of empty A site
Ski7
Assembly of the surveillance complex
Hbs1
Dom34
Decapping
Deadenylation
Cytoplasmic exosome
Ski complex
Nonstop decay (NSD)
Nonsense-mediated decay (NMD)
No-go decay (NGD)
03015 5/14/14
(c) Kanehisa Laboratories

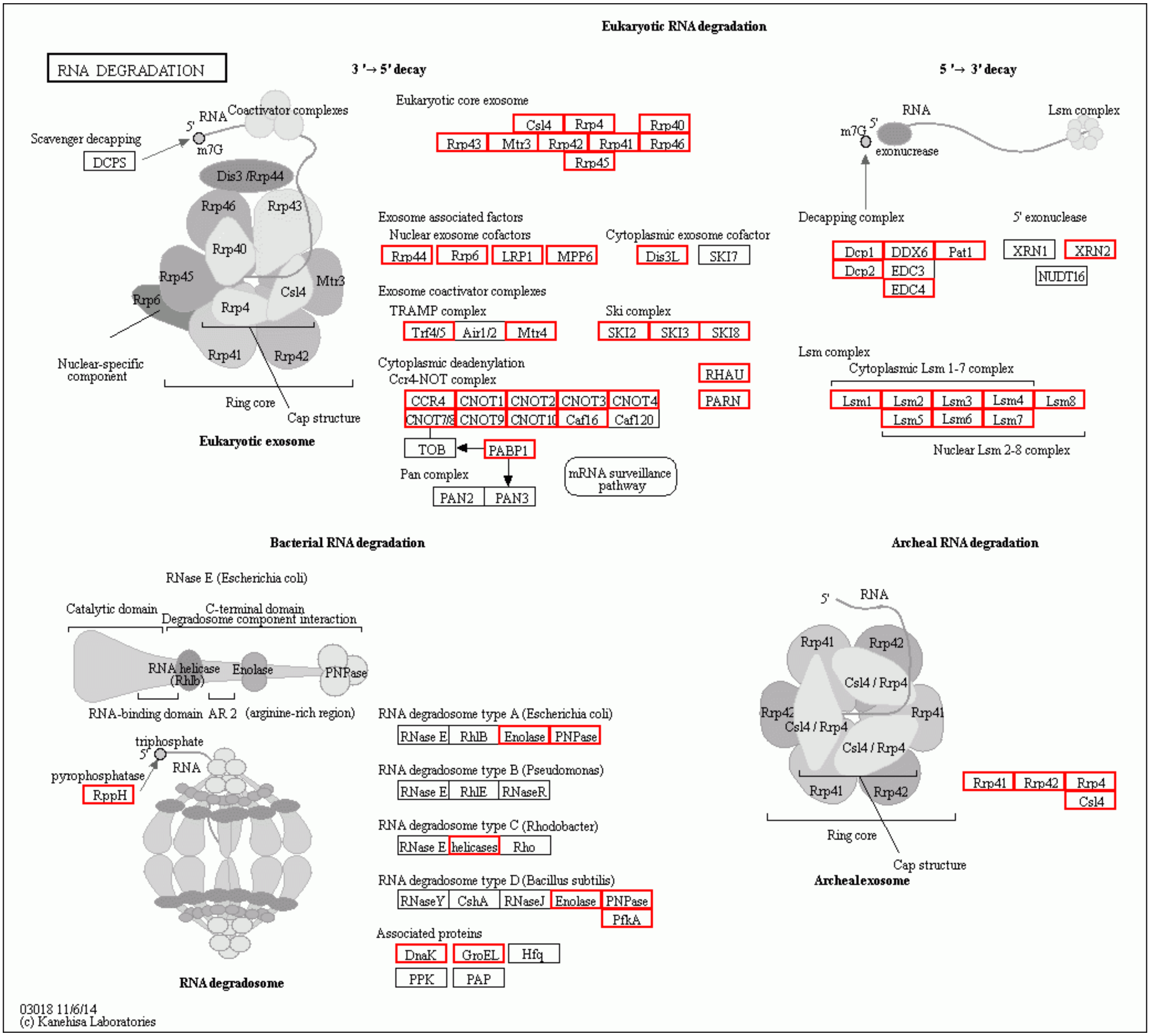

RNA DEGRADATION
Eukaryotic RNA degradation
3'→ 5' decay
5'→ 3' decay
Scavenger decapping
DCPS
RNA
Coactivator complexes
5'
m7G
Dis3 /Rrp44
Rrp46
Rrp43
Rrp40
Rrp45
Mtr3
Csl4
Rrp6
Rrp4
Rrp41
Rrp42
Nuclear-specific component
Ring core
Cap structure
Eukaryotic exosome
Eukaryotic core exosome
Csl4
Rrp4
Rrp40
Rrp43
Mtr3
Rrp42
Rrp41
Rrp46
Rrp45
Exosome associated factors
Nuclear exosome cofactors
Rrp44
Rrp6
LRP1
MPP6
Cytoplasmic exosome cofactor
Dis3L
SKI7
Exosome coactivator complexes
TRAMP complex
Trf4/5
Air1/2
Mtr4
Ski complex
SKI2
SKI3
SKI8
Cytoplasmic deadenylation
Ccr4-NOT complex
CCR4
CNOT1
CNOT2
CNOT3
CNOT4
CNOT7/8
CNOT9
CNOT10
Caf16
Caf120
RHAU
PARN
TOB
PABP1
Pan complex
PAN2
PAN3
mRNA surveillance pathway
RNA
Lsm complex
m7G
5'
exonucrease
Decapping complex
5' exonuclease
Dcp1
DDX6
Pat1
Dcp2
EDC3
EDC4
XRN1
XRN2
NUDT16
Lsm complex
Cytoplasmic Lsm 1-7 complex
Lsm1
Lsm2
Lsm3
Lsm4
Lsm8
Lsm5
Lsm6
Lsm7
Nuclear Lsm 2-8 complex
Bacterial RNA degradation
RNase E (Escherichia coli)
Catalytic domain
C-terminal domain
Degradosome component interaction
RNA helicase (Rhlb)
Enolase
PNPase
RNA-binding domain
AR 2
(arginine-rich region)
triphosphate
5'
pyrophosphatase
RNA
RppH
RNA degradosome
RNA degradosome type A (Escherichia coli)
RNase E
RhlB
Enolase
PNPase
RNA degradosome type B (Pseudomonas)
RNase E
RhlE
RNaseR
RNA degradosome type C (Rhodobacter)
RNase E
helicases
Rho
RNA degradosome type D (Bacillus subtilis)
RNaseY
CshA
RNaseJ
Enolase
PNPase
PfkA
Associated proteins
DnaK
GroEL
Hfq
PPK
PAP
Archeal RNA degradation
5'
RNA
Rrp41
Rrp42
Csl4 / Rrp4
Rrp42
Rrp41
Csl4 / Rrp4
Csl4 / Rrp4
Rrp41
Rrp42
Ring core
Cap structure
Archeal exosome
Rrp41
Rrp42
Rrp4
Csl4
03018 11/6/14
(c) Kanehisa Laboratories

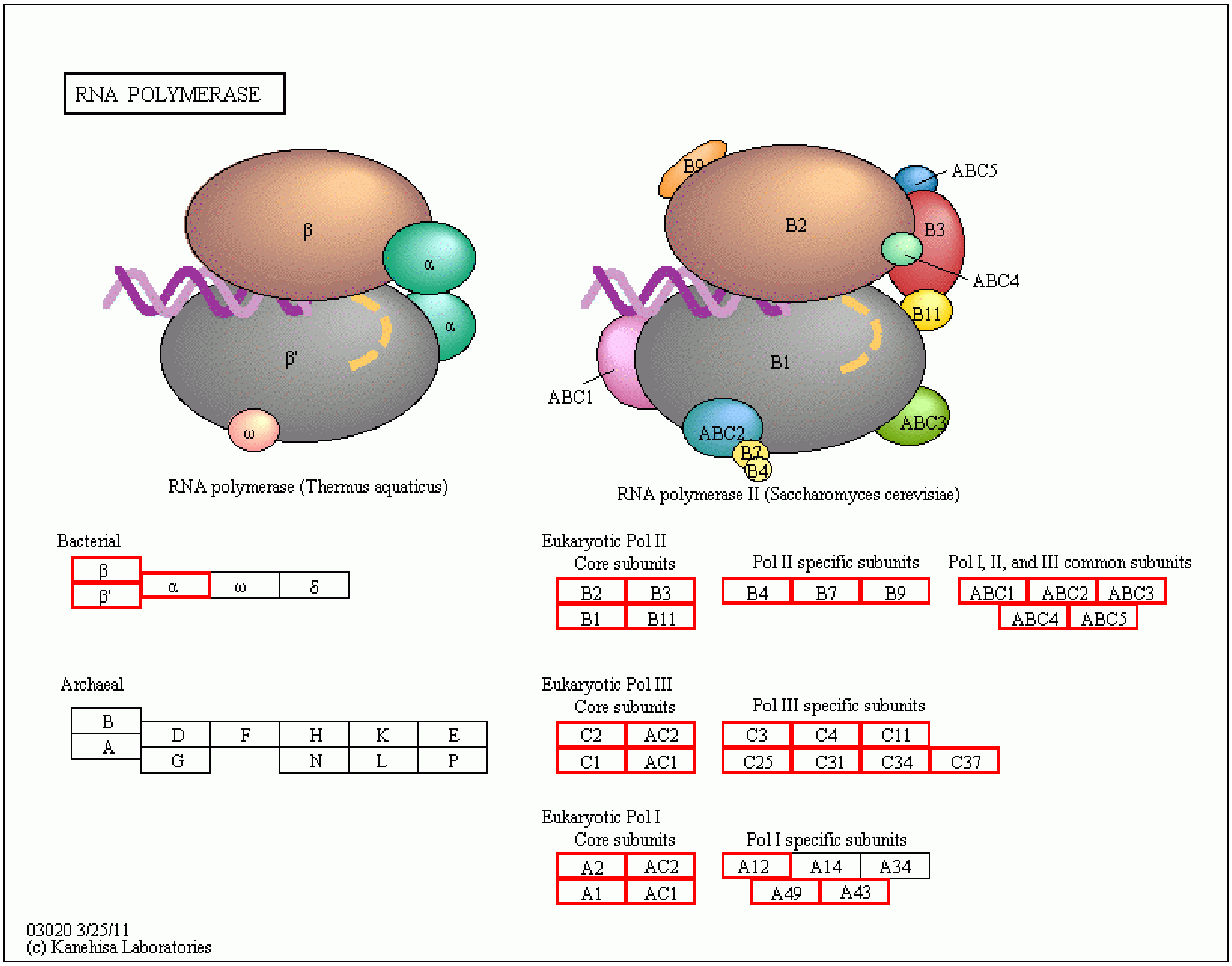
RNA POLYMERASE
β
α
α
β'
ω
RNA polymerase (Thermus aquaticus)
B9
ABC5
B2
B3
ABC4
B11
B1
ABC1
ABC2
ABC3
B7
B4
RNA polymerase II (Saccharomyces cerevisiae)
Bacterial
β
β'
α
ω
δ
Archaeal
B
A
D
G
F
H
N
K
L
E
P
Eukaryotic Pol II
Core subunits
B2 B3
B1 B11
Pol II specific subunits
B4 B7 B9
Pol I, II, and III common subunits
ABC1 ABC2 ABC3
ABC4 ABC5
Eukaryotic Pol III
Core subunits
C2 AC2
C1 AC1
Pol III specific subunits
C3 C4 C11
C25 C31 C34 C37
Eukaryotic Pol I
Core subunits
A2 AC2
A1 AC1
Pol I specific subunits
A12 A14 A34
A49 A43
03020 3/25/11
(c) Kanehisa Laboratories

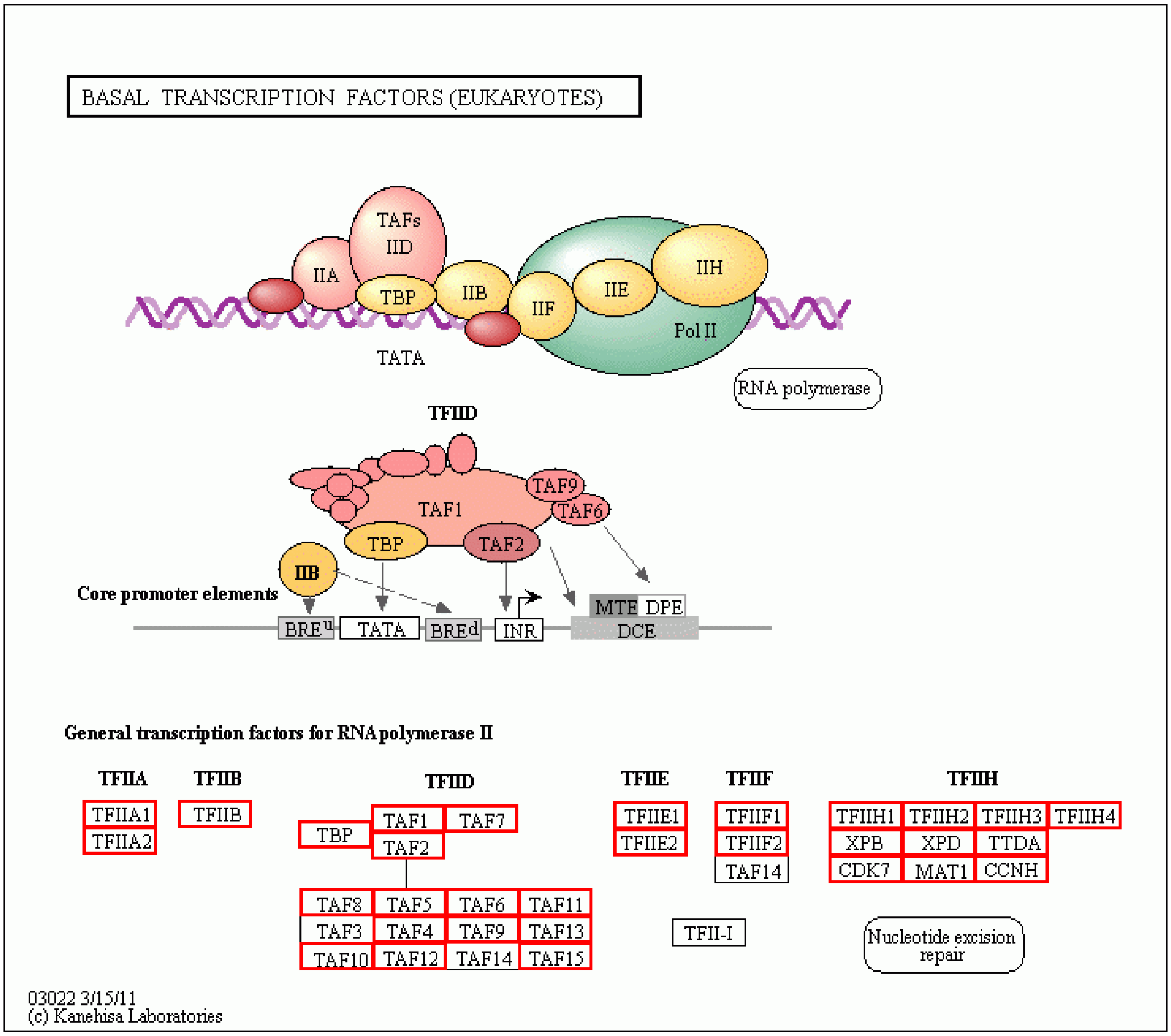
BASAL TRANSCRIPTION FACTORS (EUKARYOTES)
TAFs
IID
IIA
TBP
IIB
IIF
IIE
IIH
Pol II
TATA
RNA polymerase
TFIID
TAF9
TAF6
TAF1
TBP
TAF2
IIB
Core promoter elements
BREu
TATA
BREd
INR
MTE
DPE
DCE
General transcription factors for RNA polymerase II
TFIIA
TFIIA1
TFIIA2
TFIIB
TFIIB
TFIID
TBP
TAF1
TAF7
TAF2
TAF8
TAF5
TAF6
TAF11
TAF3
TAF4
TAF9
TAF13
TAF10
TAF12
TAF14
TAF15
TFIIE
TFIIE1
TFIIE2
TFIIF
TFIIF1
TFIIF2
TAF14
TFII-I
TFIIH
TFIIH1
TFIIH2
TFIIH3
TFIIH4
XPB
XPD
TTDA
CDK7
MAT1
CCNH
Nucleotide excision repair
03022 3/15/11
(c) Kanehisa Laboratories

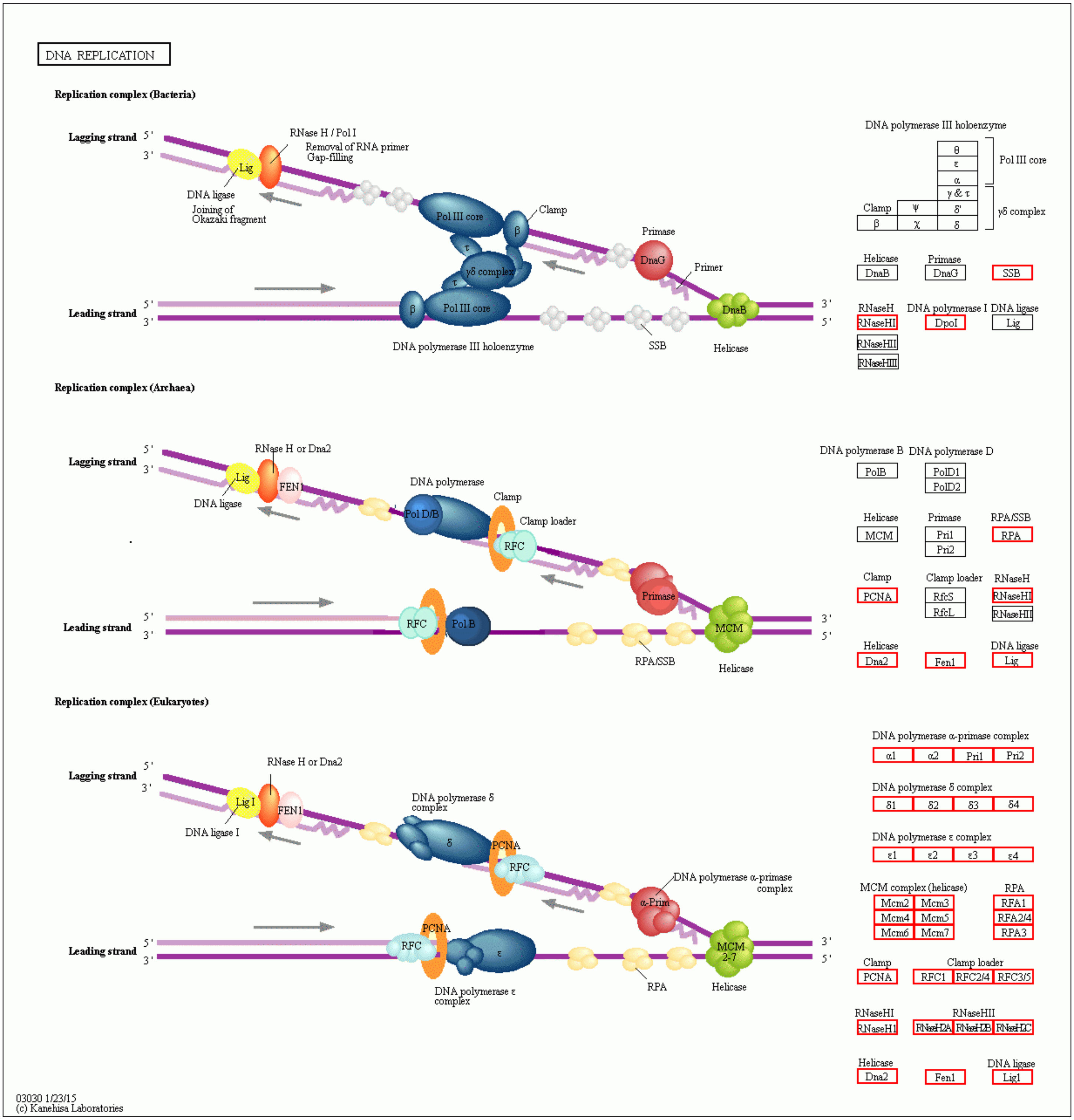
DNA REPLICATION
Replication complex (Bacteria)
Lagging strand
Leading strand
5'
3'
RNase H / Pol I
Removal of RNA primer
Gap-filling
Lig
DNA ligase
Joining of Okazaki fragment
Pol III core
β
τ
γδ complex
Clamp
Primase
DnaG
Primer
DnaB
DNA polymerase III holoenzyme
SSB
Helicase
DNA polymerase III holoenzyme
θ
ε
α
γ & τ
Clamp
ψ
δ'
β
χ
δ
Pol III core
γδ complex
Helicase
DnaB
Primase
DnaG
SSB
RNaseH
RNaseHI
RNaseHII
RNaseHIII
DNA polymerase I
DpoI
DNA ligase
Lig
Replication complex (Archaea)
RNase H or Dna2
FEN1
DNA polymerase
Pol D/B
Clamp
Clamp loader
RFC
Primase
Pol B
MCM
RPA/SSB
DNA polymerase B
PolB
DNA polymerase D
PolD1
PolD2
Helicase
MCM
Primase
Pri1
Pri2
RPA/SSB
RPA
Clamp
PCNA
Clamp loader
RfcS
RfcL
RNaseH
RNaseHI
RNaseHII
Helicase
Dna2
Fen1
DNA ligase
Lig
Replication complex (Eukaryotes)
Lig I
DNA ligase I
DNA polymerase δ complex
δ
PCNA
RFC
DNA polymerase α-primase complex
α-Prim
ε
DNA polymerase ε complex
MCM 2-7
RPA
Helicase
DNA polymerase α-primase complex
α1
α2
Pri1
Pri2
DNA polymerase δ complex
δ1
δ2
δ3
δ4
DNA polymerase ε complex
ε1
ε2
ε3
ε4
MCM complex (helicase)
Mcm2
Mcm3
Mcm4
Mcm5
Mcm6
Mcm7
RPA
RFA1
RFA2/4
RPA3
Clamp
PCNA
Clamp loader
RFC1
RFC2/4
RFC3/5
RNaseHI
RNaseH1
RNaseHII
RNaseH2A
RNaseH2B
RNaseH2C
Helicase
Dna2
Fen1
DNA ligase
Lig1
03030 1/23/15
(c) Kanehisa Laboratories

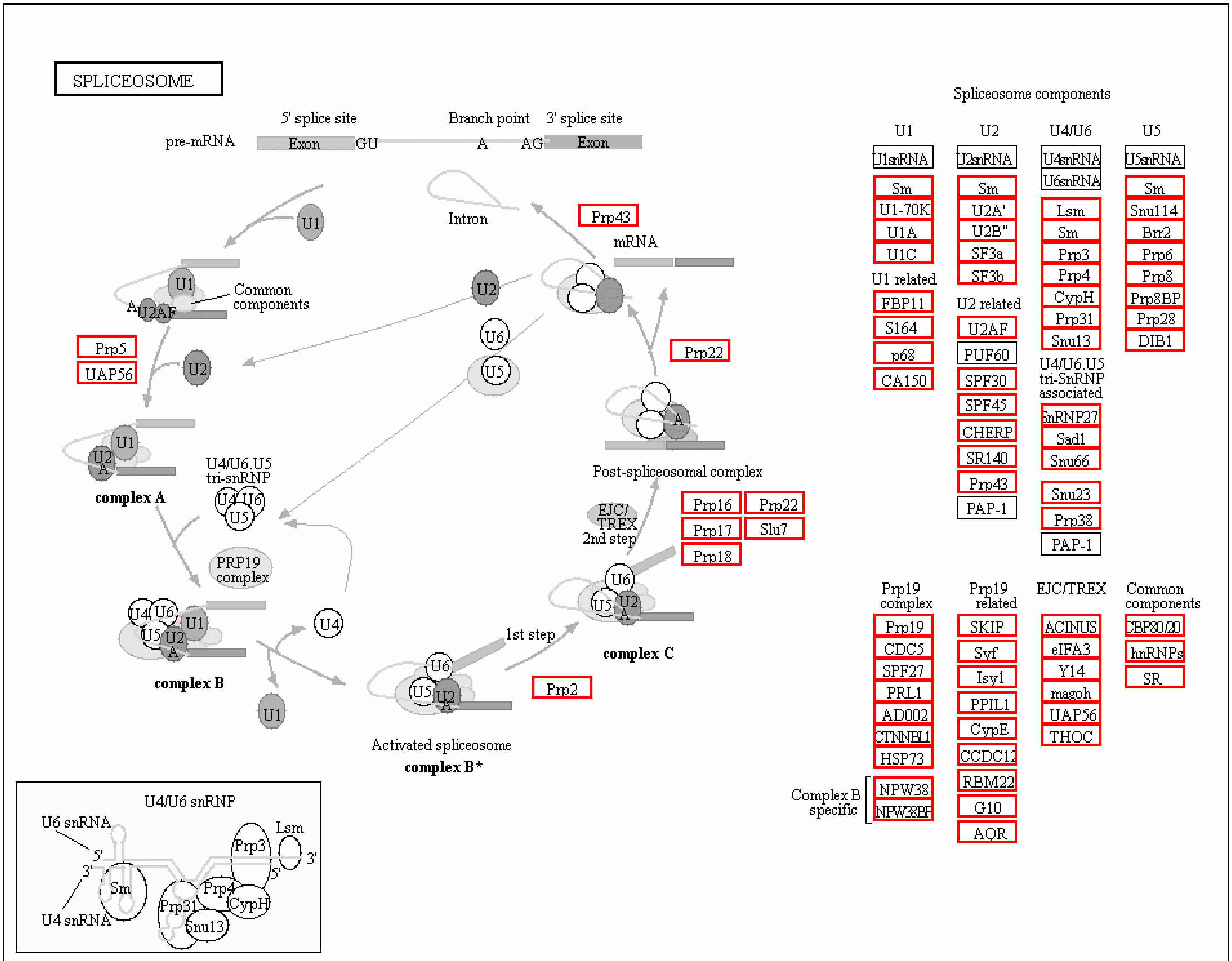

03040 1/11/17
(c) Kanehisa Laboratories

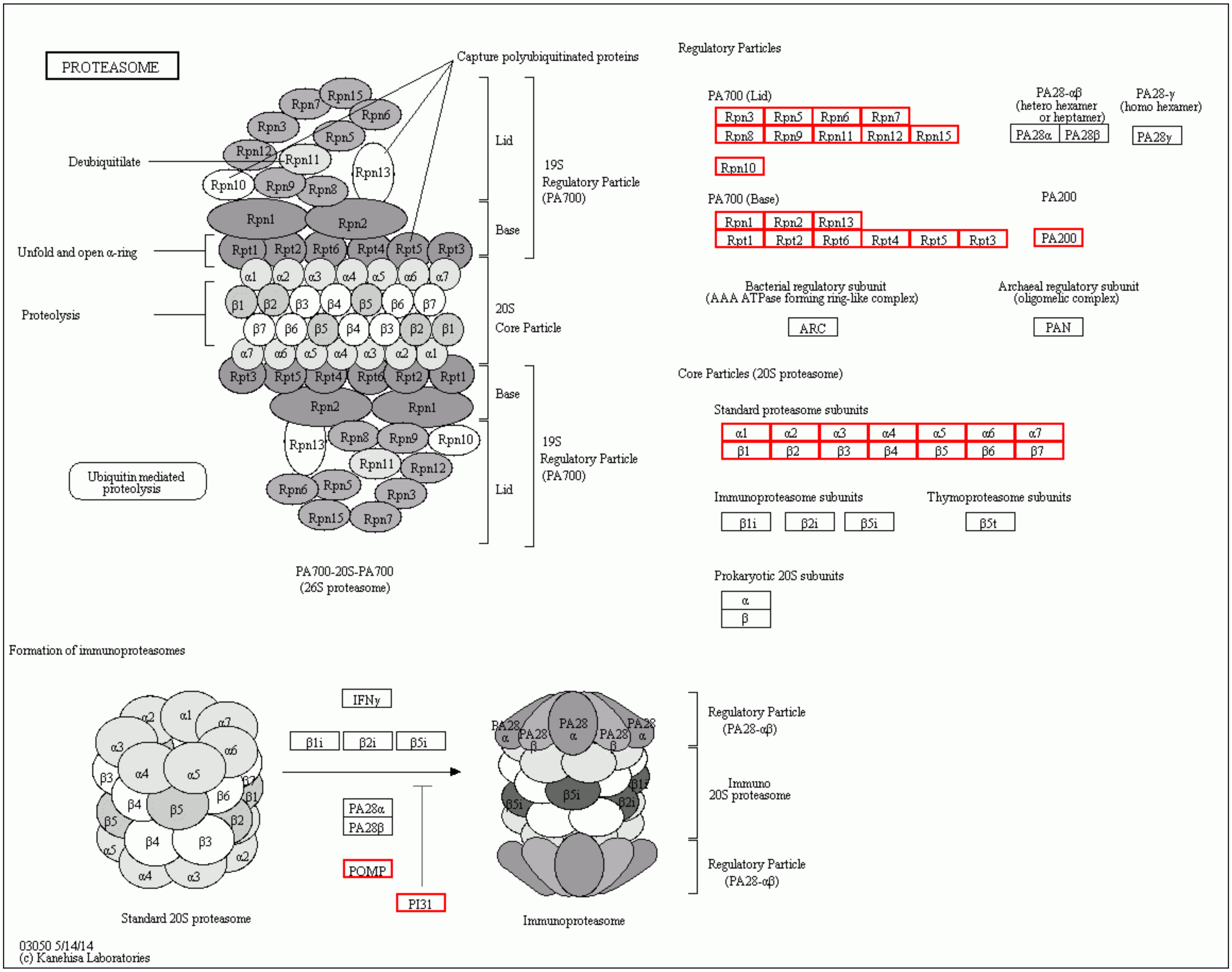
PROTEASOME
Capture polyubiquitinated proteins
Deubiquitilate
Unfold and open α-ring
Proteolysis
Ubiquitin mediated proteolysis
Rpn15
Rpn7
Rpn6
Rpn3
Rpn5
Rpn12
Rpn11
Rpn13
Rpn10
Rpn9
Rpn8
Rpn1
Rpn2
Rpt1
Rpt2
Rpt6
Rpt4
Rpt5
Rpt3
α1 α2 α3 α4 α5 α6 α7
β1 β2 β3 β4 β5 β6 β7
Lid
Base
19S Regulatory Particle (PA700)
20S Core Particle
PA700-20S-PA700 (26S proteasome)
Regulatory Particles
PA700 (Lid)
Rpn3 Rpn5 Rpn6 Rpn7
Rpn8 Rpn9 Rpn11 Rpn12 Rpn15
Rpn10
PA700 (Base)
Rpn1 Rpn2 Rpn13
Rpt1 Rpt2 Rpt6 Rpt4 Rpt5 Rpt3
PA28-αβ (hetero hexamer or heptamer)
PA28-γ (homo hexamer)
PA28α PA28β
PA28γ
PA200
Bacterial regulatory subunit (AAA ATPase forming ring-like complex)
ARC
Archaeal regulatory subunit (oligomelic complex)
PAN
Core Particles (20S proteasome)
Standard proteasome subunits
α1 α2 α3 α4 α5 α6 α7
β1 β2 β3 β4 β5 β6 β7
Immunoproteasome subunits
β1i β2i β5i
Thymoproteasome subunits
β5t
Prokaryotic 20S subunits
α
β
Formation of immunoproteasomes
IFNγ
β1i β2i β5i
PA28α
PA28β
POMP
PI31
Standard 20S proteasome
Immunoproteasome
PA28 α
PA28 β
β5i β1i β2i
Regulatory Particle (PA28-αβ)
Immuno 20S proteasome
Regulatory Particle (PA28-αβ)
03050 5/14/14
(c) Kanehisa Laboratories

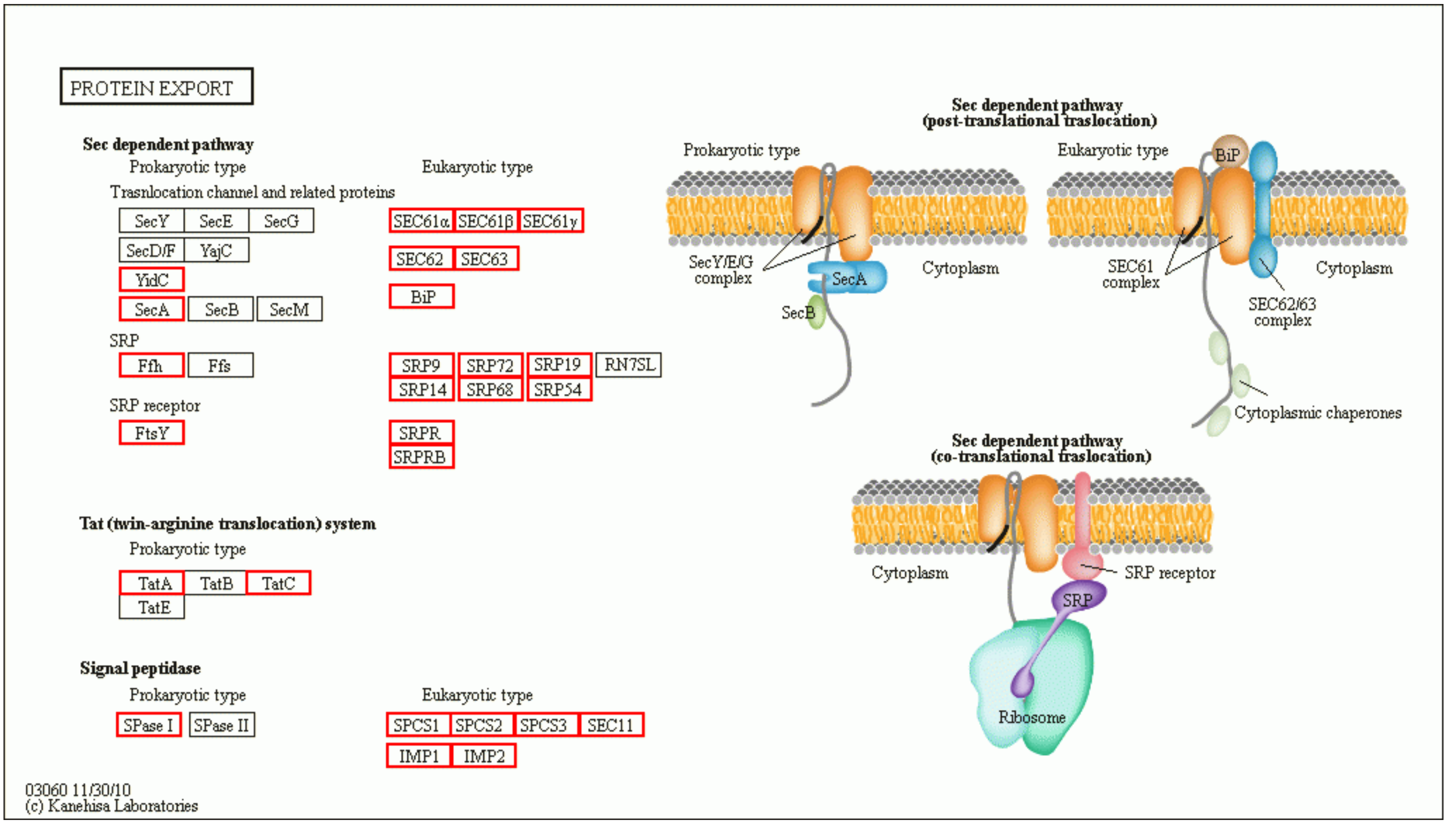
PROTEIN EXPORT
Sec dependent pathway
Prokaryotic type
Eukaryotic type
Trasnlocation channel and related proteins
SecY
SecE
SecG
SecD/F
YajC
YidC
SecA
SecB
SecM
SEC61α
SEC61β
SEC61γ
SEC62
SEC63
BiP
SRP
Ffh
Ffs
SRP9
SRP72
SRP19
RN7SL
SRP14
SRP68
SRP54
SRP receptor
FtsY
SRPR
SRPRB
Tat (twin-arginine translocation) system
Prokaryotic type
TatA
TatB
TatC
TatE
Signal peptidase
Prokaryotic type
Eukaryotic type
SPase I
SPase II
SPCS1
SPCS2
SPCS3
SEC11
IMP1
IMP2
Sec dependent pathway
(post-translational traslocation)
Prokaryotic type
Eukaryotic type
BiP
SecY/E/G
complex
SecA
SecB
Cytoplasm
SEC61
complex
Cytoplasm
SEC62/63
complex
Cytoplasmic chaperones
Sec dependent pathway
(co-translational traslocation)
Cytoplasm
SRP receptor
SRP
Ribosome
03060 11/30/10
(c) Kanehisa Laboratories

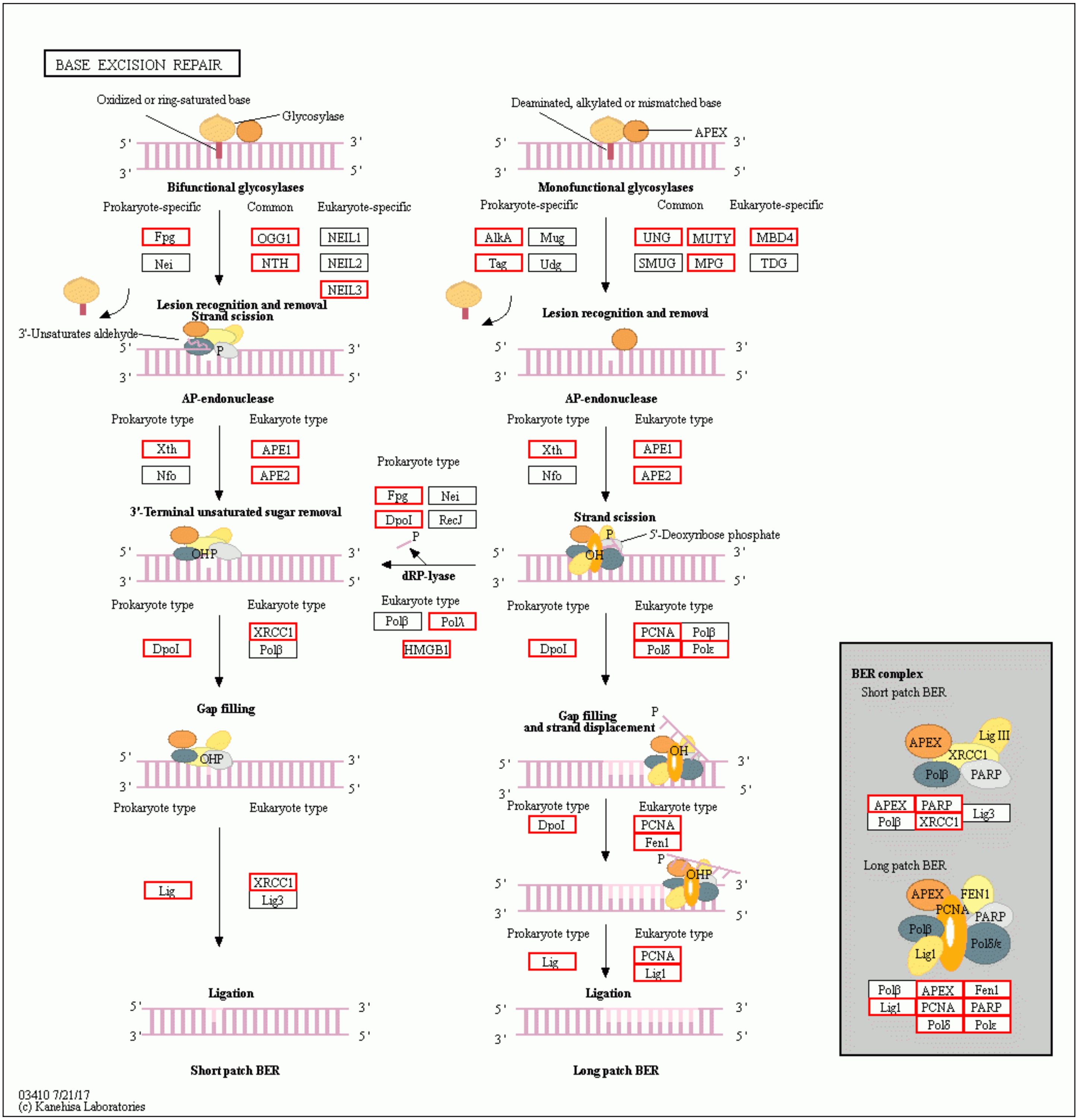
BASE EXCISION REPAIR
Oxidized or ring-saturated base
Glycosylase
Deaminated, alkylated or mismatched base
APEX
Bifunctional glycosylases
Prokaryote-specific
Common
Eukaryote-specific
Fpg
Nei
OGG1
NTH
NEIL1
NEIL2
NEIL3
Monofunctional glycosylases
AlkA
Mug
Tag
Udg
UNG
MUTY
SMUG
MPG
MBD4
TDG
Lesion recognition and removal
Strand scission
3'-Unsaturates aldehyde
AP-endonuclease
Prokaryote type
Eukaryote type
Xth
Nfo
APE1
APE2
3'-Terminal unsaturated sugar removal
OHP
Nei
RecJ
DpoI
dRP-lyase
Polβ
Polλ
HMGB1
Strand scission
5'-Deoxyribose phosphate
OH
XRCC1
PCNA
Polδ
Polε
Gap filling
Gap filling
and strand displacement
Fen1
Lig
Lig3
Lig1
Ligation
Short patch BER
Long patch BER
BER complex
Short patch BER
Lig III
XRCC1
PARP
Long patch BER
FEN1
Polδ/ε
03410 7/21/17
(c) Kanehisa Laboratories

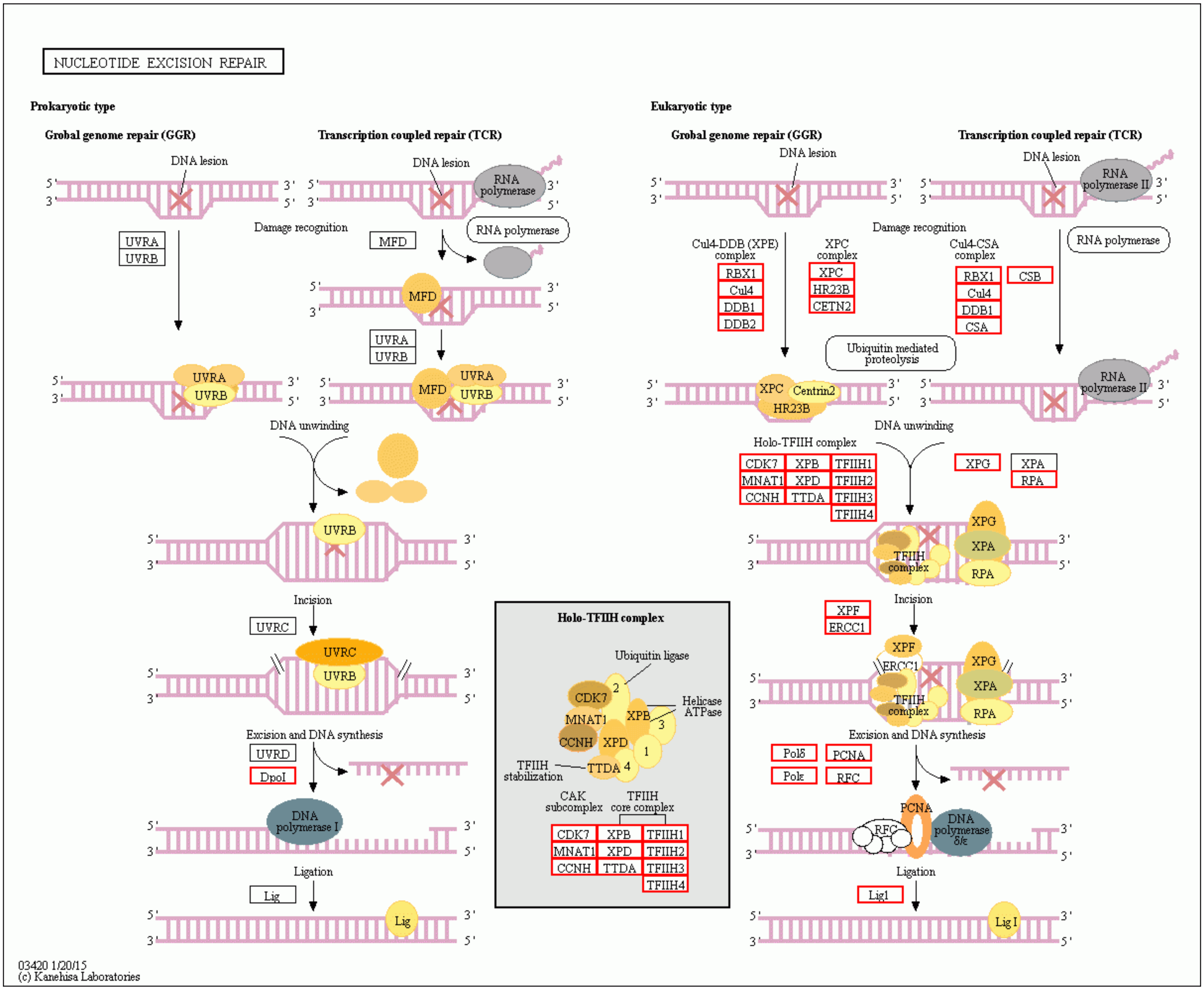
NUCLEOTIDE EXCISION REPAIR
Prokaryotic type
Grobal genome repair (GGR)
Transcription coupled repair (TCR)
DNA lesion
UVRA
UVRB
Damage recognition
MFD
RNA polymerase
UVRC
DNA unwinding
Incision
Excision and DNA synthesis
UVRD
DpoI
DNA polymerase I
Ligation
Lig
Eukaryotic type
Cul4-DDB (XPE) complex
RBX1
Cul4
DDB1
DDB2
XPC complex
XPC
HR23B
CETN2
Cul4-CSA complex
CSA
CSB
RNA polymerase II
Ubiquitin mediated proteolysis
Centrin2
Holo-TFIIH complex
CDK7
MNAT1
CCNH
XPB
XPD
TTDA
TFIIH1
TFIIH2
TFIIH3
TFIIH4
XPG
XPA
RPA
TFIIH complex
XPF
ERCC1
Polδ
Polε
PCNA
RFC
DNA polymerase δ/ε
LigI
Lig I
Ubiquitin ligase
Helicase ATPase
TFIIH stabilization
CAK subcomplex
TFIIH core complex
03420 1/20/15
(c) Kanehisa Laboratories

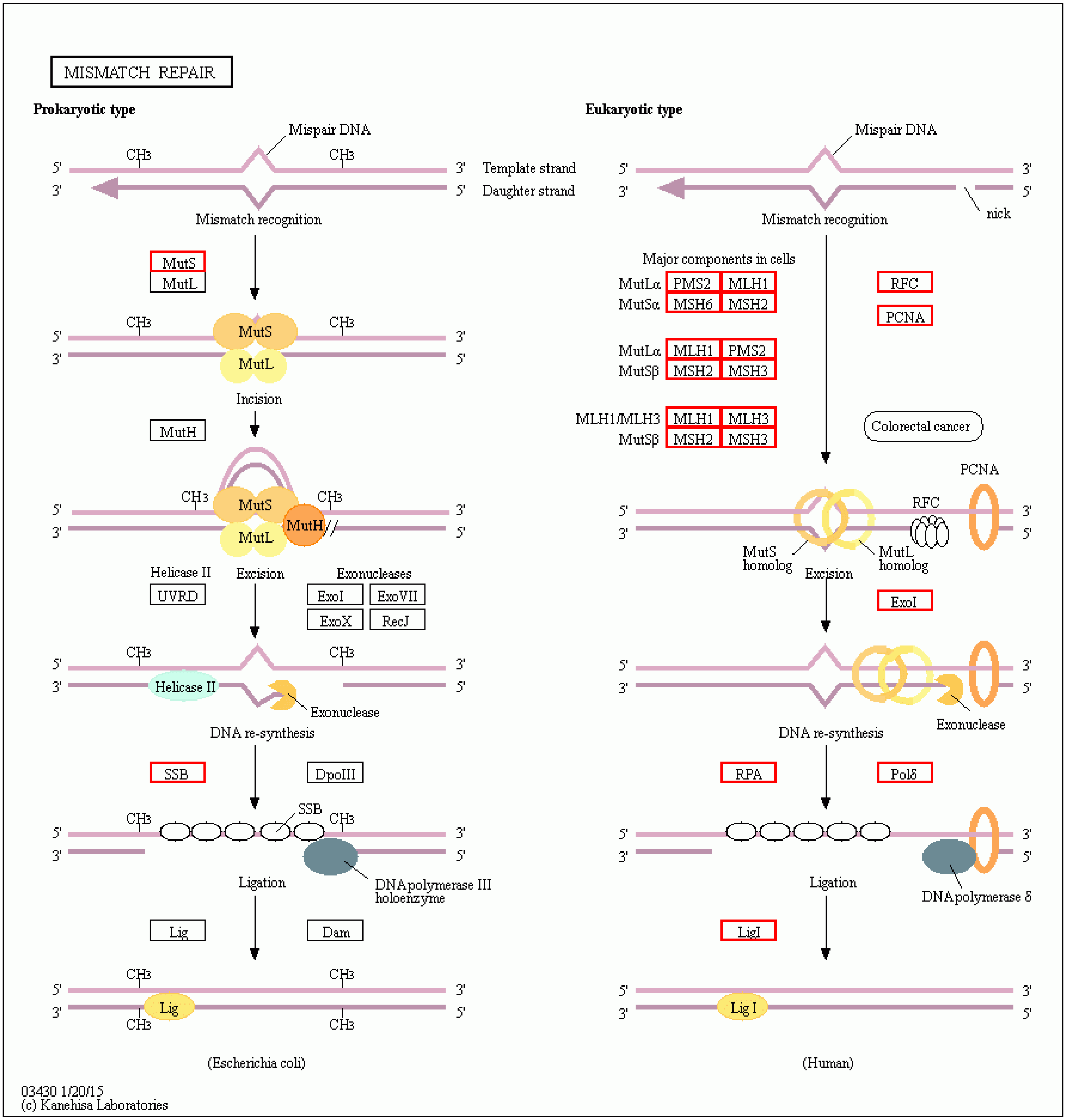

MISMATCH REPAIR
Prokaryotic type
Eukaryotic type
Mispair DNA
CH3
5'
3'
Template strand
Daughter strand
Mismatch recognition
nick
MutS
MutL
Major components in cells
MutLα PMS2 MLH1
MutSα MSH6 MSH2
MutLα MLH1 PMS2
MutSβ MSH2 MSH3
MLH1/MLH3 MLH1 MLH3
MutSβ MSH2 MSH3
RFC
PCNA
Colorectal cancer
Incision
MutH
MutS homolog
MutL homolog
Helicase II
UVRD
Excision
Exonucleases
ExoI
ExoVII
ExoX
RecJ
Exonuclease
DNA re-synthesis
SSB
DpoIII
RPA
Polδ
Ligation
DNApolymerase III holoenzyme
DNApolymerase δ
Lig
Dam
LigI
Lig I
(Escherichia coli)
(Human)
03430 1/20/15
(c) Kanehisa Laboratories

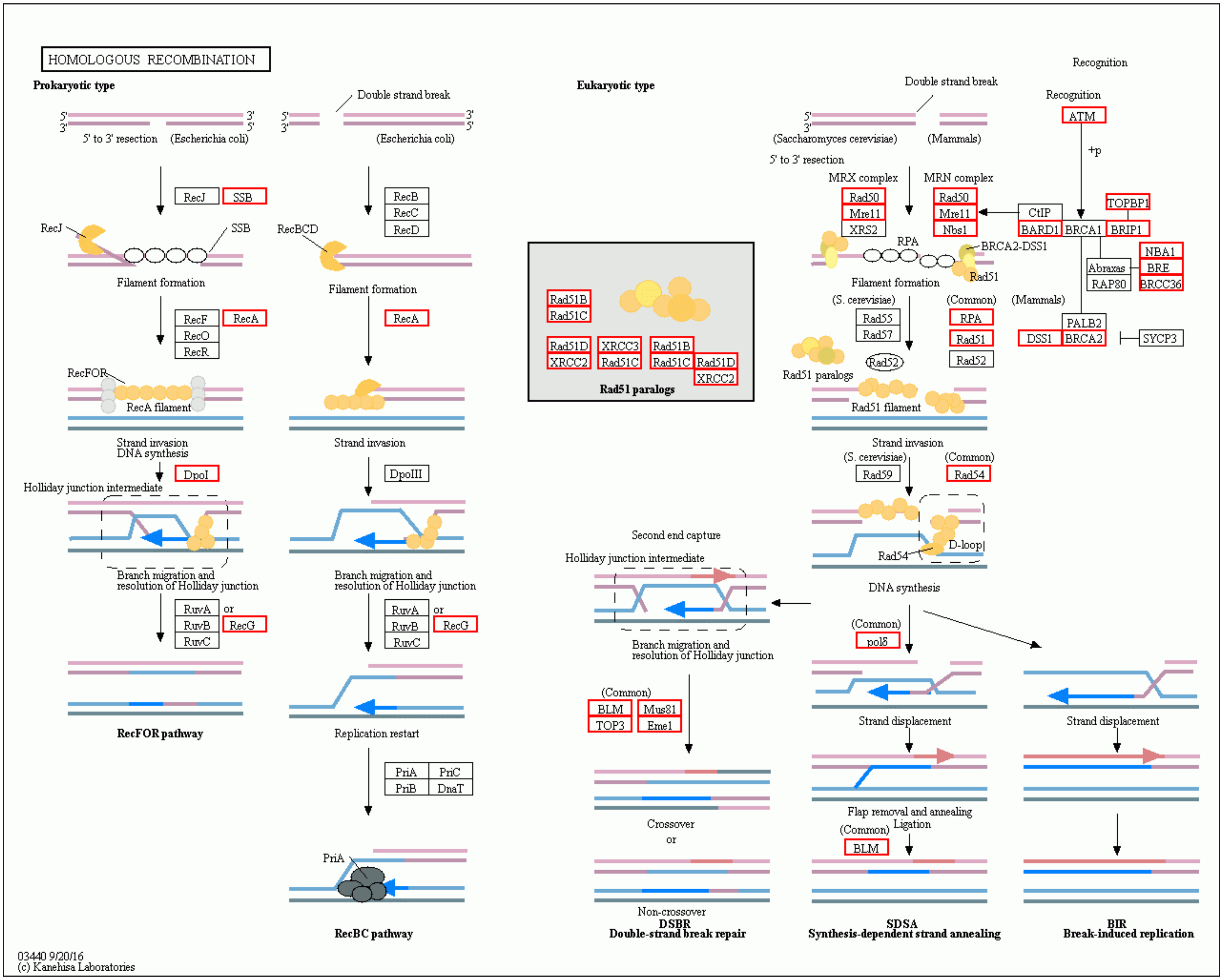
HOMOLOGOUS RECOMBINATION
Prokaryotic type
Double strand break
5' to 3' resection
(Escherichia coli)
RecJ
SSB
RecB
RecC
RecD
RecBCD
Filament formation
RecF
RecO
RecR
RecA
RecFOR
RecA filament
Strand invasion
DNA synthesis
DpoI
DpoIII
Holliday junction intermediate
Branch migration and resolution of Holliday junction
RuvA
RuvB
RuvC
or
RecG
RecFOR pathway
Replication restart
PriA
PriB
PriC
DnaT
RecBC pathway
Eukaryotic type
Rad51B
Rad51C
Rad51D
XRCC2
XRCC3
Rad51 paralogs
(Saccharomyces cerevisiae)
(Mammals)
MRX complex
MRN complex
Rad50
Mre11
XRS2
Nbs1
CtIP
Recognition
ATM
+p
TOPBP1
BARD1
BRCA1
BRIP1
BRCA2-DSS1
RPA
Rad51
Abraxas
RAP80
NBA1
BRE
BRCC36
(S. cerevisiae)
(Common)
Rad55
Rad57
Rad52
PALB2
DSS1
BRCA2
SYCP3
Rad51 filament
Rad59
Rad54
D-loop
Second end capture
DNA synthesis
polδ
BLM
TOP3
Mus81
Eme1
Crossover
Non-crossover
DSBR
Double-strand break repair
Strand displacement
Flap removal and annealing
Ligation
SDSA
Synthesis-dependent strand annealing
BIR
Break-induced replication
03440 9/20/16
(c) Kanehisa Laboratories

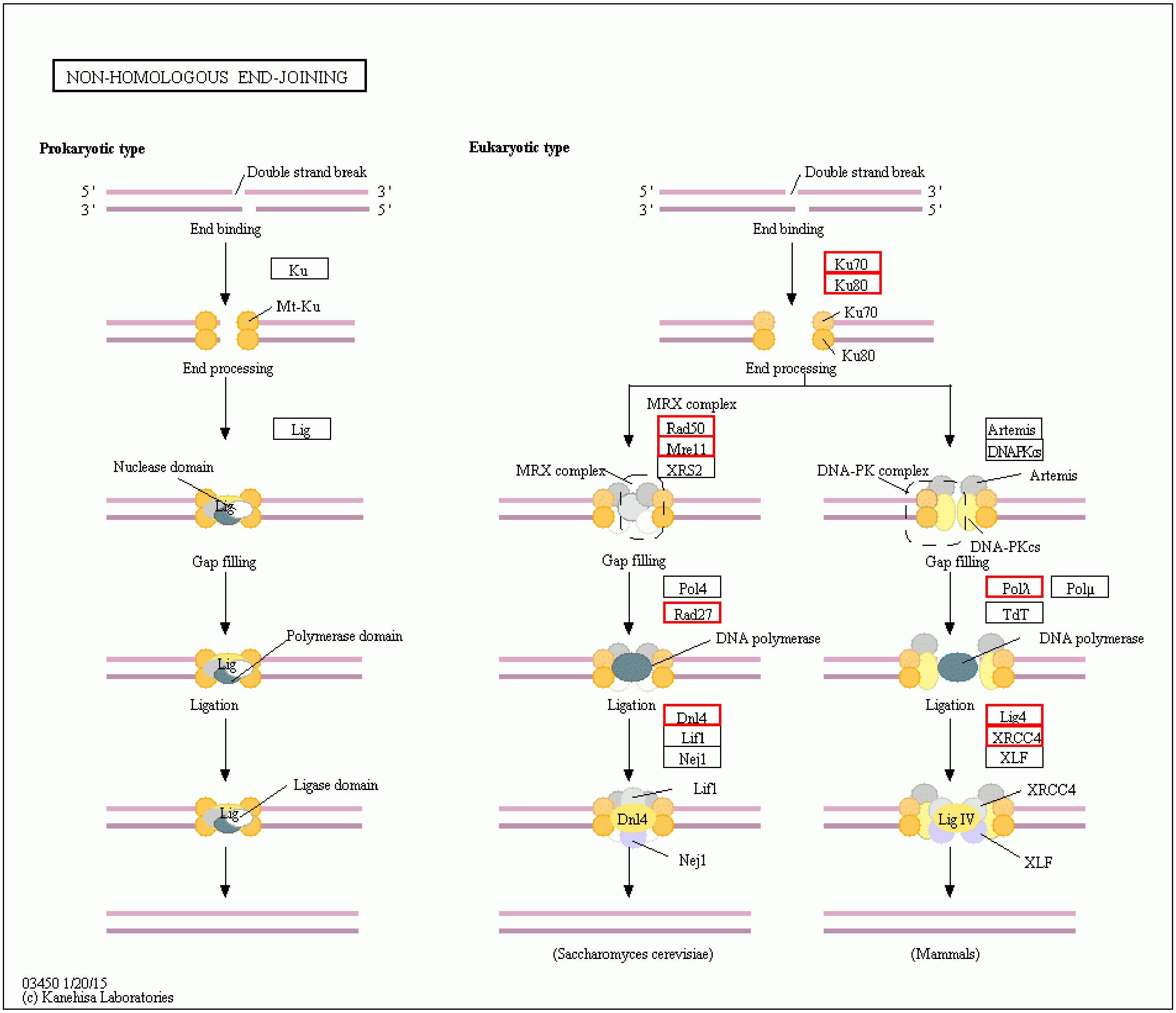
NON-HOMOLOGOUS END-JOINING
Prokaryotic type
Eukaryotic type
Double strand break
5'
3'
End binding
Ku
Mt-Ku
End processing
Lig
Nuclease domain
Gap filling
Polymerase domain
Ligation
Ligase domain
Ku70
Ku80
MRX complex
Rad50
Mre11
XRS2
Artemis
DNAPKcs
DNA-PK complex
DNA-PKcs
Pol4
Rad27
DNA polymerase
Polλ
Polμ
TdT
Dnl4
Lif1
Nej1
Lig4
XRCC4
XLF
Lig IV
(Saccharomyces cerevisiae)
(Mammals)
03450 1/20/15
(c) Kanehisa Laboratories

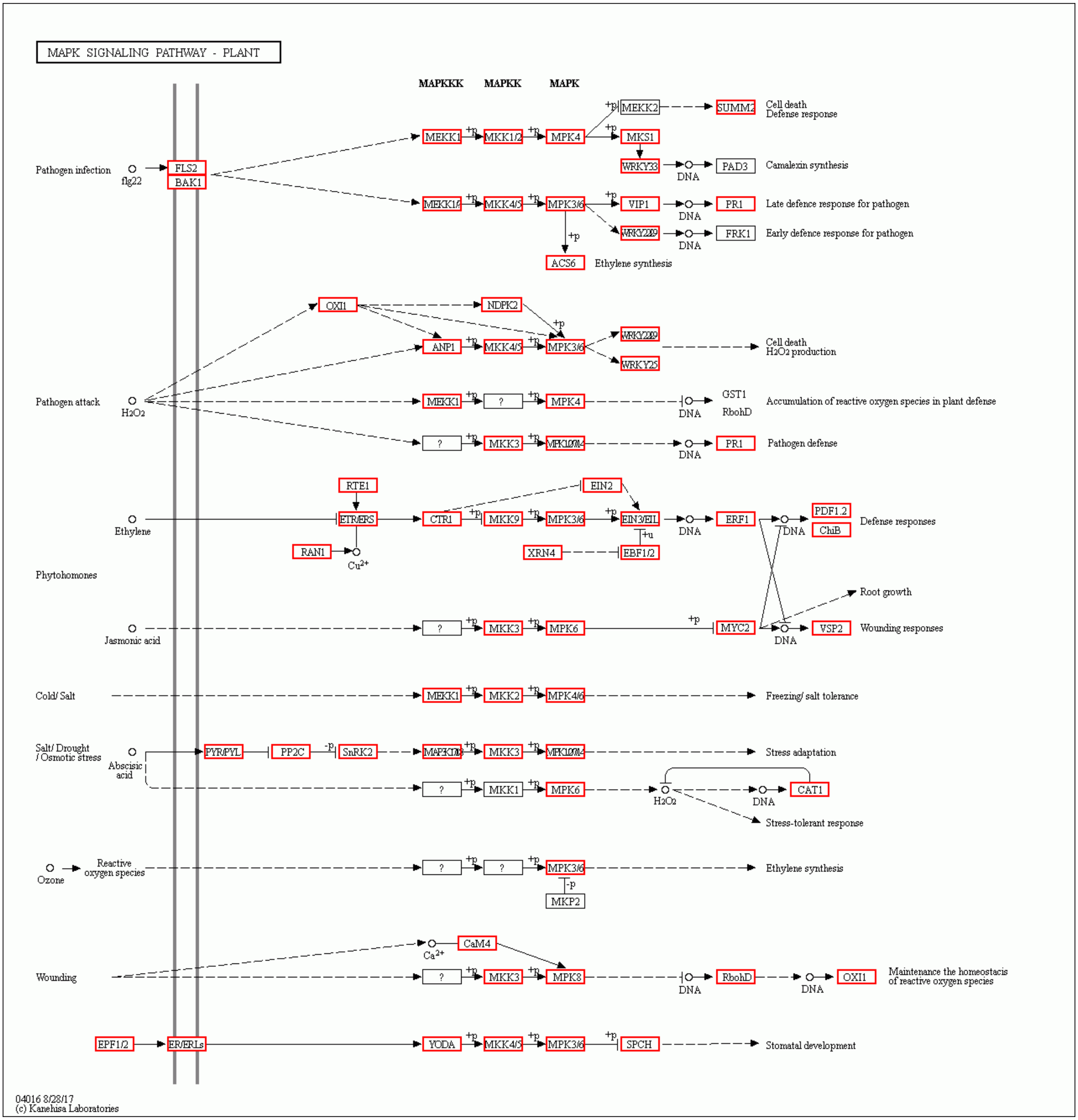
MAPK SIGNALING PATHWAY - PLANT
MAPKKK
MAPKK
MAPK
Pathogen infection
flg22
FLS2
BAK1
MEKK1
MKK1/2
MPK4
MEKK2
SUMM2
Cell death
Defense response
MKS1
WRKY33
DNA
PAD3
Camalexin synthesis
MEKK1/4
MKK4/5
MPK3/6
VIP1
PR1
Late defence response for pathogen
WRKY22/29
FRK1
Early defence response for pathogen
ACS6
Ethylene synthesis
OXI1
NDPK2
ANP1
WRKY22/29
WRKY25
Cell death
H2O2 production
Pathogen attack
H2O2
?
GST1
RbohD
Accumulation of reactive oxygen species in plant defense
MKK3
MPK1/2/7/14
Pathogen defense
RTE1
EIN2
Ethylene
ETR/ERS
CTR1
MKK9
EIN3/EIL
ERF1
PDF1.2
ChiB
Defense responses
RAN1
Cu2+
XRN4
EBF1/2
Phytohomones
Root growth
Jasmonic acid
MPK6
MYC2
VSP2
Wounding responses
Cold/ Salt
MKK2
MPK4/6
Freezing/ salt tolerance
Salt/ Drought
/ Osmotic stress
Abscisic acid
PYR/PYL
PP2C
SnRK2
MAP3K17/18
Stress adaptation
MKK1
CAT1
Stress-tolerant response
Ozone
Reactive oxygen species
MKP2
CaM4
Ca2+
Wounding
MPK8
OXI1
Maintenance the homeostacis of reactive oxygen species
EPF1/2
ER/ERLs
YODA
SPCH
Stomatal development
04016 8/28/17
(c) Kanehisa Laboratories

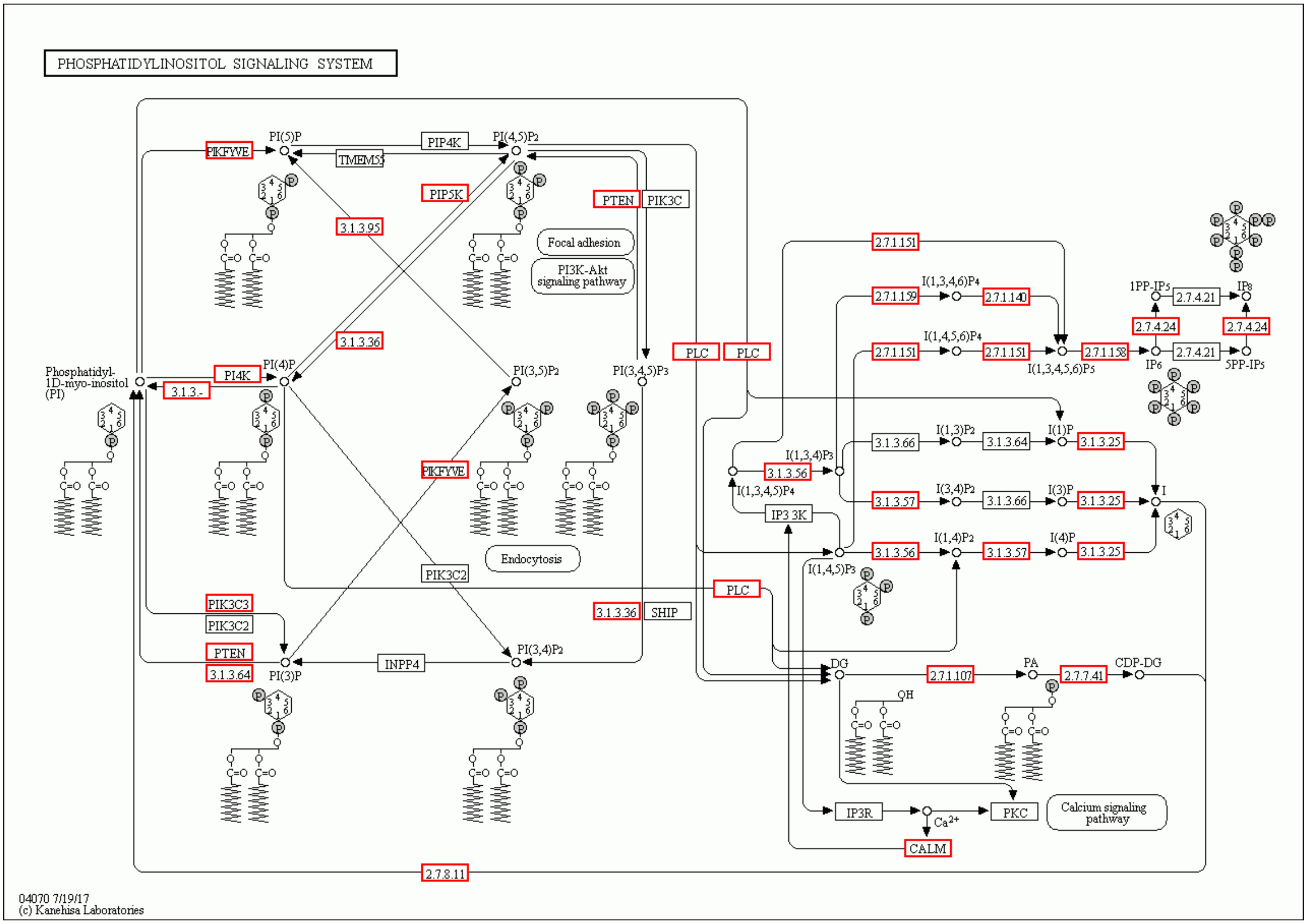

PHOSPHATIDYLINOSITOL SIGNALING SYSTEM
PI(5)P
PIKFYVE
PIP4K
PI(4,5)P2
TMEM55
PIP5K
PTEN
PIK3C
3.1.3.95
Focal adhesion
PI3K-Akt signaling pathway
3.1.3.36
Phosphatidyl-1D-myo-inositol (PI)
PI4K
PI(4)P
3.1.3.-
PI(3,5)P2
PI(3,4,5)P3
PLC
PLC
PIKFYVE
Endocytosis
PIK3C2
PIK3C3
PIK3C2
PTEN
3.1.3.64
PI(3)P
INPP4
PI(3,4)P2
3.1.3.36
SHIP
PLC
2.7.1.151
I(1,3,4,6)P4
2.7.1.159
2.7.1.140
I(1,4,5,6)P4
2.7.1.151
2.7.1.151
2.7.1.158
I(1,3,4,5,6)P5
1PP-IP5
2.7.4.21
IP8
2.7.4.24
2.7.4.24
IP6
2.7.4.21
5PP-IP5
I(1,3,4)P3
3.1.3.56
I(1,3,4,5)P4
3.1.3.66
I(1,3)P2
3.1.3.64
I(1)P
3.1.3.25
I
3.1.3.57
I(3,4)P2
3.1.3.66
I(3)P
3.1.3.25
IP3 3K
I(1,4,5)P3
3.1.3.56
I(1,4)P2
3.1.3.57
I(4)P
3.1.3.25
DG
2.7.1.107
PA
2.7.7.41
CDP-DG
OH
IP3R
Ca2+
PKC
Calcium signaling pathway
CALM
2.7.8.11
04070 7/19/17
(c) Kanehisa Laboratories

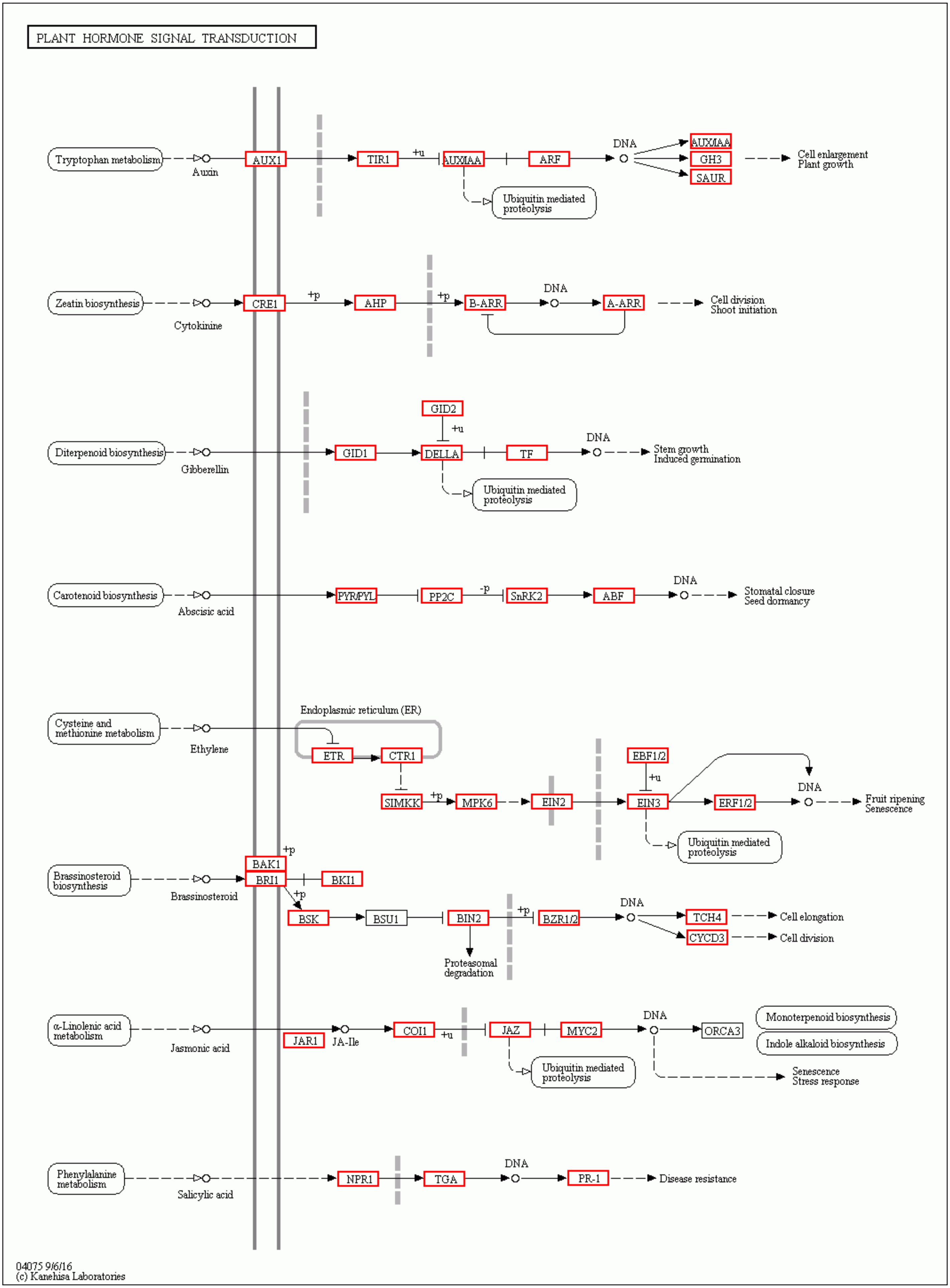
PLANT HORMONE SIGNAL TRANSDUCTION
Tryptophan metabolism
Auxin
AUX1
TIR1
+u
AUX/IAA
ARF
DNA
AUX/IAA
GH3
SAUR
Cell enlargement
Plant growth
Ubiquitin mediated proteolysis
Zeatin biosynthesis
Cytokinine
CRE1
+p
AHP
+p
B-ARR
DNA
A-ARR
Cell division
Shoot initiation
GID2
+u
Diterpenoid biosynthesis
Gibberellin
GID1
DELLA
TF
DNA
Stem growth
Induced germination
Ubiquitin mediated proteolysis
Carotenoid biosynthesis
Abscisic acid
PYR/PYL
PP2C
-p
SnRK2
ABF
DNA
Stomatal closure
Seed dormancy
Endoplasmic reticulum (ER)
Cysteine and methionine metabolism
Ethylene
ETR
CTR1
SIMKK
+p
MPK6
EIN2
EBF1/2
+u
EIN3
ERF1/2
DNA
Fruit ripening
Senescence
Ubiquitin mediated proteolysis
+p
BAK1
Brassinosteroid biosynthesis
Brassinosteroid
BRI1
BKI1
+p
BSK
BSU1
BIN2
+p
BZR1/2
DNA
TCH4
Cell elongation
CYCD3
Cell division
Proteasomal degradation
α-Linolenic acid metabolism
Jasmonic acid
JAR1
JA-Ile
COI1
+u
JAZ
MYC2
DNA
ORCA3
Monoterpenoid biosynthesis
Indole alkaloid biosynthesis
Ubiquitin mediated proteolysis
Senescence
Stress response
Phenylalanine metabolism
Salicylic acid
NPR1
TGA
DNA
PR-1
Disease resistance
04075 9/6/16
(c) Kanehisa Laboratories

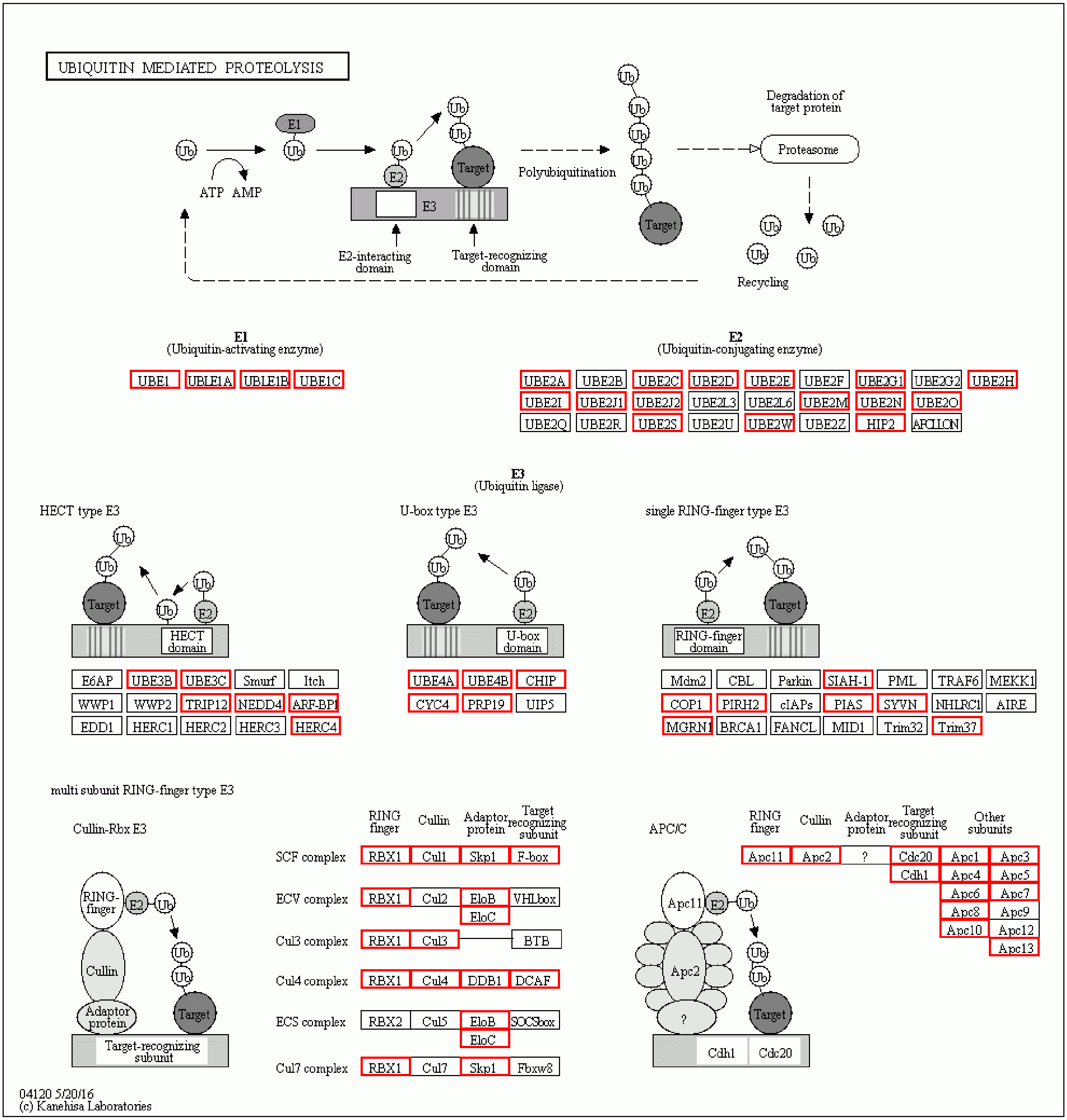
UBIQUITIN MEDIATED PROTEOLYSIS
E1
Ub
ATP AMP
E2
E3
Target
E2-interacting domain
Target-recognizing domain
Polyubiquitination
Degradation of target protein
Proteasome
Recycling
E1
(Ubiquitin-activating enzyme)
UBE1
UBLE1A
UBLE1B
UBE1C
E2
(Ubiquitin-conjugating enzyme)
UBE2A
UBE2B
UBE2C
UBE2D
UBE2E
UBE2F
UBE2G1
UBE2G2
UBE2H
UBE2I
UBE2J1
UBE2J2
UBE2L3
UBE2L6
UBE2M
UBE2N
UBE2O
UBE2Q
UBE2R
UBE2S
UBE2U
UBE2W
UBE2Z
HIP2
APOLLON
E3
(Ubiquitin ligase)
HECT type E3
HECT domain
E6AP
UBE3B
UBE3C
Smurf
Itch
WWP1
WWP2
TRIP12
NEDD4
ARF-BP1
EDD1
HERC1
HERC2
HERC3
HERC4
U-box type E3
U-box domain
UBE4A
UBE4B
CHIP
CYC4
PRP19
UIP5
single RING-finger type E3
RING-finger domain
Mdm2
CBL
Parkin
SIAH-1
PML
TRAF6
MEKK1
COP1
PIRH2
cIAPs
PIAS
SYVN
NHLRC1
AIRE
MGRN1
BRCA1
FANCL
MID1
Trim32
Trim37
multi subunit RING-finger type E3
Cullin-Rbx E3
RING-finger
Cullin
Adaptor protein
Target-recognizing subunit
RING finger
Cullin
Adaptor protein
Target recognizing subunit
SCF complex
RBX1
Cul1
Skp1
F-box
ECV complex
RBX1
Cul2
EloB
VHLbox
EloC
Cul3 complex
RBX1
Cul3
BTB
Cul4 complex
RBX1
Cul4
DDB1
DCAF
ECS complex
RBX2
Cul5
EloB
SOCSbox
EloC
Cul7 complex
RBX1
Cul7
Skp1
Fbxw8
APC/C
Apc11
Apc2
?
Cdh1
Cdc20
RING finger
Cullin
Adaptor protein
Target recognizing subunit
Other subunits
Apc11
Apc2
?
Cdc20
Cdh1
Apc1
Apc3
Apc4
Apc5
Apc6
Apc7
Apc8
Apc9
Apc10
Apc12
Apc13
04120 5/20/16
(c) Kanehisa Laboratories

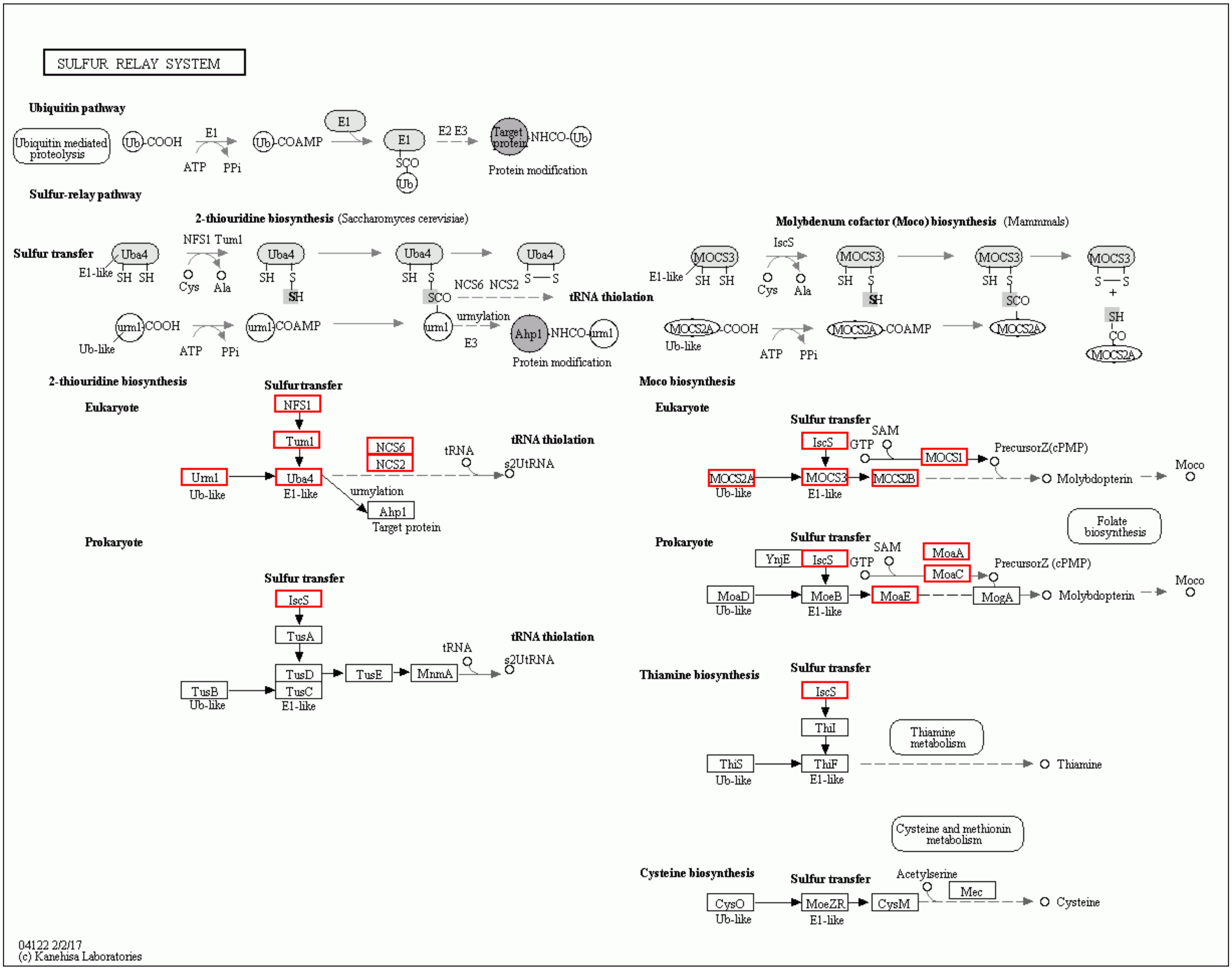
SULFUR RELAY SYSTEM
Ubiquitin pathway
Ubiquitin mediated proteolysis
Protein modification
Sulfur-relay pathway
2-thiouridine biosynthesis (Saccharomyces cerevisiae)
Sulfur transfer
tRNA thiolation
Molybdenum cofactor (Moco) biosynthesis (Mammals)
2-thiouridine biosynthesis
Eukaryote
Prokaryote
Sulfur transfer
NFS1
Tum1
Urm1
Uba4
NCS6
NCS2
Ahp1
Target protein
IscS
TusA
TusB
TusD
TusC
TusE
MnmA
Moco biosynthesis
MOCS2A
MOCS3
MOCS2B
MOCS1
PrecursorZ(cPMP)
Molybdopterin
Moco
Folate biosynthesis
YnjE
MoaA
MoaC
MoaD
MoeB
MoaE
MogA
Thiamine biosynthesis
ThiI
ThiS
ThiF
Thiamine metabolism
Thiamine
Cysteine and methionin metabolism
Cysteine biosynthesis
Acetylserine
Mec
CysO
MoeZR
CysM
Cysteine
04122 2/2/17
(c) Kanehisa Laboratories

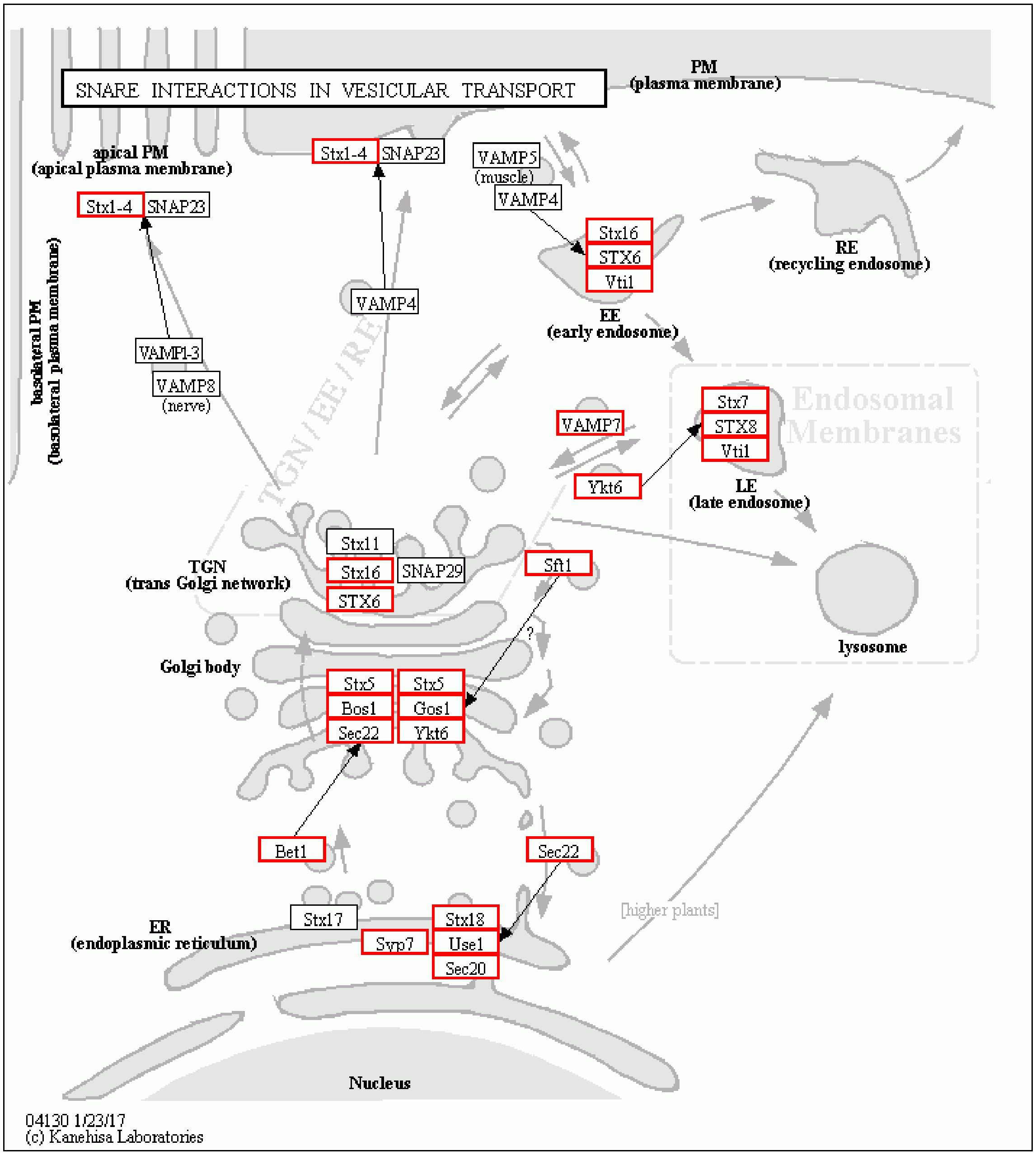
SNARE INTERACTIONS IN VESICULAR TRANSPORT
PM
(plasma membrane)
apical PM
(apical plasma membrane)
basolateral PM
(basolateral plasma membrane)
Stx1-4
SNAP23
Stx1-4
SNAP23
VAMP5
(muscle)
VAMP4
Stx16
STX6
Vti1
EE
(early endosome)
RE
(recycling endosome)
VAMP4
VAMP1-3
VAMP8
(nerve)
TGN / EE / RE
Endosomal Membranes
VAMP7
Ykt6
Stx7
STX8
Vti1
LE
(late endosome)
Stx11
Stx16
STX6
SNAP29
TGN
(trans Golgi network)
Sft1
?
lysosome
Golgi body
Stx5
Bos1
Sec22
Stx5
Gos1
Ykt6
Bet1
Sec22
[higher plants]
Stx17
Syp7
Stx18
Use1
Sec20
ER
(endoplasmic reticulum)
Nucleus
04130 1/23/17
(c) Kanehisa Laboratories

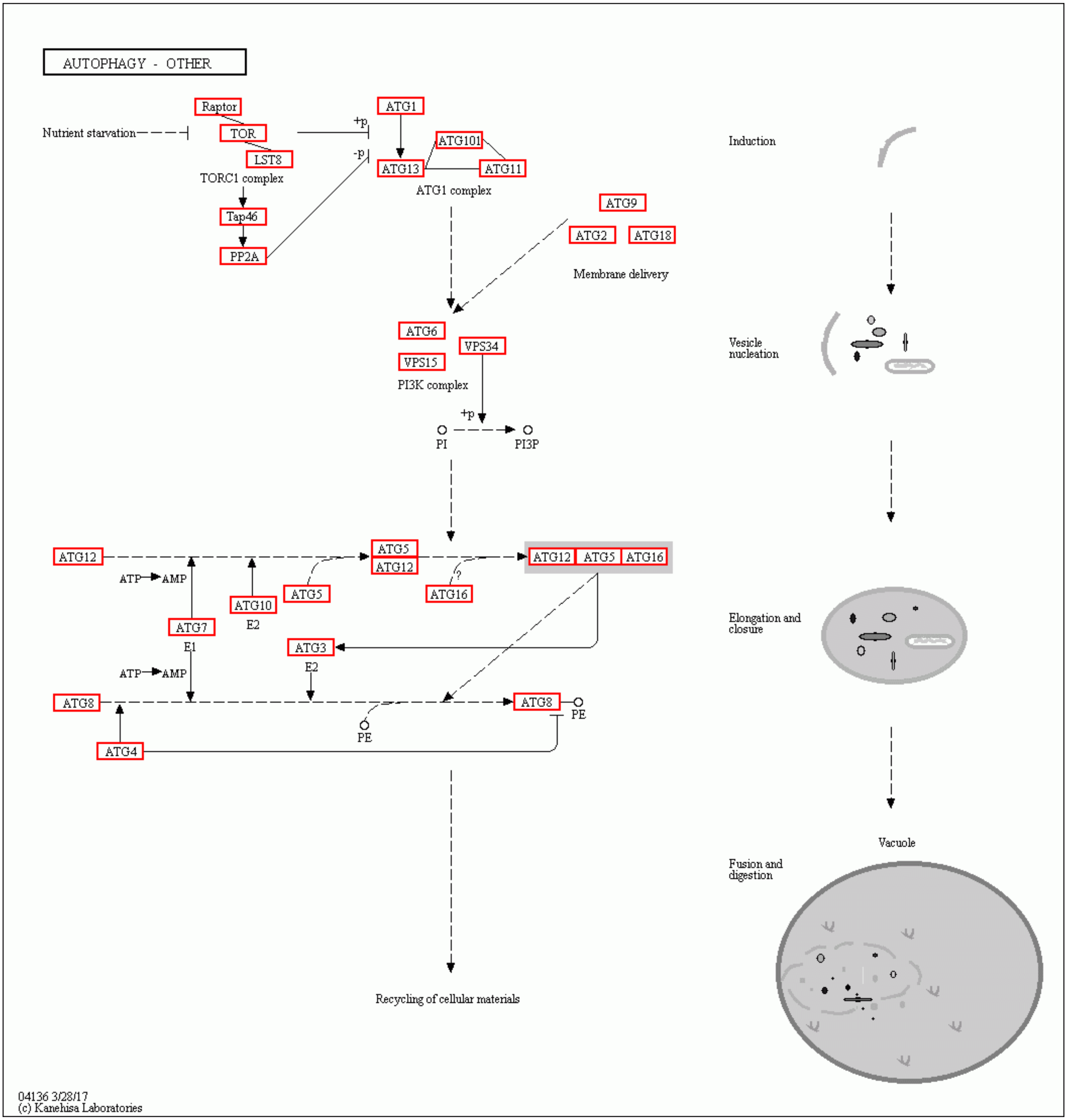
AUTOPHAGY - OTHER
Raptor
TOR
LST8
TORC1 complex
Nutrient starvation
Tap46
PP2A
+p
-p
ATG1
ATG13
ATG101
ATG11
ATG1 complex
ATG9
ATG2
ATG18
Membrane delivery
ATG6
VPS34
VPS15
PI3K complex
+p
PI
PI3P
ATG12
ATP
AMP
ATG10
E2
ATG7
E1
ATG5
ATG12
ATG5
ATG16
?
ATG12
ATG5
ATG16
ATG3
E2
ATP
AMP
ATG8
ATG4
PE
ATG8
PE
Recycling of cellular materials
Induction
Vesicle nucleation
Elongation and closure
Vacuole
Fusion and digestion
04136 3/28/17
(c) Kanehisa Laboratories

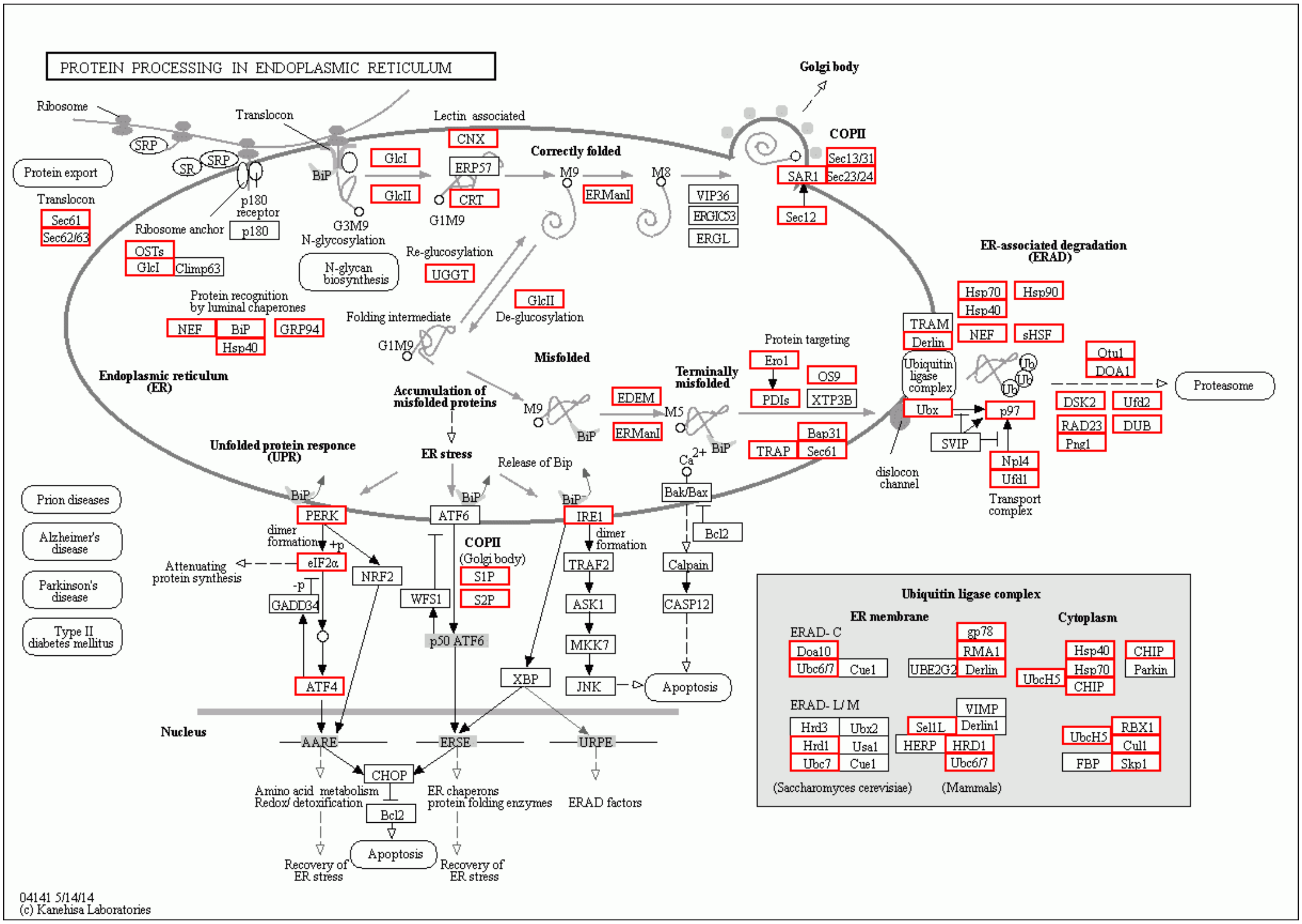
PROTEIN PROCESSING IN ENDOPLASMIC RETICULUM
Ribosome
Translocon
SRP
SR
SRP
Protein export
Translocon
Sec61
Sec62/63
p180 receptor
Ribosome anchor
p180
OSTs
GlcI
Climp63
BiP
GlcI
GlcII
G3M9
N-glycosylation
N-glycan biosynthesis
Lectin associated
CNX
ERP57
CRT
G1M9
Correctly folded
M9
M8
ERManI
VIP36
ERGIC53
ERGL
Golgi body
COPII
Sec13/31
SAR1
Sec23/24
Sec12
Re-glucosylation
UGGT
GlcII
De-glucosylation
Folding intermediate
G1M9
Protein recognition by luminal chaperones
NEF
BiP
GRP94
Hsp40
Endoplasmic reticulum (ER)
Misfolded
Accumulation of misfolded proteins
M9
EDEM
M5
ERManI
BiP
Terminally misfolded
Protein targeting
Ero1
PDIs
OS9
XTP3B
Bap31
TRAP
Sec61
Ca 2+
BiP
ER stress
Release of Bip
Unfolded protein responce (UPR)
ER-associated degradation (ERAD)
Hsp70
Hsp90
Hsp40
NEF
sHSF
TRAM
Derlin
Ubiquitin ligase complex
Ub
Ub
Ub
Ubx
p97
SVIP
Npl4
Ufd1
Transport complex
dislocon channel
Otu1
DOA1
DSK2
Ufd2
RAD23
DUB
Png1
Proteasome
Prion diseases
Alzheimer's disease
Parkinson's disease
Type II diabetes mellitus
BiP
PERK
dimer formation
+p
eIF2α
Attenuating protein synthesis
-p
GADD34
NRF2
ATF4
BiP
ATF6
COPII (Golgi body)
S1P
S2P
WFS1
p50 ATF6
BiP
IRE1
dimer formation
TRAF2
ASK1
MKK7
JNK
XBP
Apoptosis
Bak/Bax
Bcl2
Calpain
CASP12
Nucleus
AARE
ERSE
URPE
CHOP
Amino acid metabolism
Redox/ detoxification
Bcl2
Apoptosis
ER chaperons
protein folding enzymes
ERAD factors
Recovery of ER stress
Recovery of ER stress
Ubiquitin ligase complex
ER membrane
Cytoplasm
ERAD- C
Doa10
Ubc6/7
Cue1
gp78
RMA1
UBE2G2
Derlin
Hsp40
CHIP
Hsp70
Parkin
UbcH5
CHIP
ERAD- L/ M
Hrd3
Ubx2
Hrd1
Usa1
Ubc7
Cue1
VIMP
Sel1L
Derlin1
HERP
HRD1
Ubc6/7
UbcH5
RBX1
Cul1
FBP
Skp1
(Saccharomyces cerevisiae)
(Mammals)
04141 5/14/14
(c) Kanehisa Laboratories

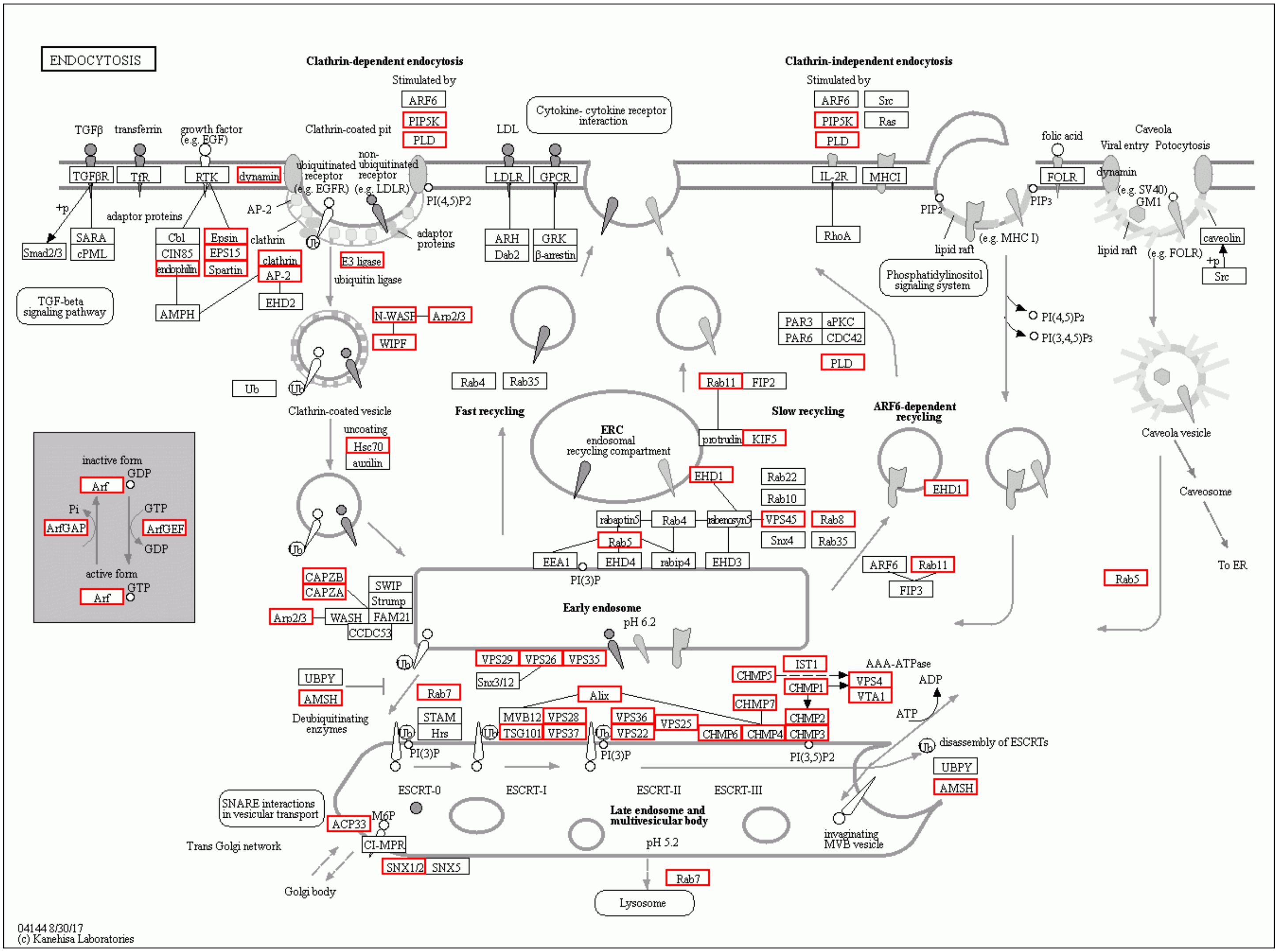
ENDOCYTOSIS
Clathrin-dependent endocytosis
Stimulated by
ARF6
PIP5K
PLD
Clathrin-independent endocytosis
Cytokine- cytokine receptor interaction
Src
Ras
TGFβ
transferrin
growth factor (e.g. EGF)
Clathrin-coated pit
LDL
folic acid
Caveola
Viral entry
Potocytosis
TGFβR
TfR
RTK
dynamin
non-ubiquitinated receptor (e.g. LDLR)
ubiquitinated receptor (e.g. EGFR)
LDLR
GPCR
IL-2R
MHCI
FOLR
(e.g. SV40)
GM1
AP-2
PI(4,5)P2
+p
adaptor proteins
SARA
cPML
Smad2/3
Cbl
CIN85
endophilin
Epsin
EPS15
Spartin
clathrin
E3 ligase
ubiquitin ligase
ARH
Dab2
GRK
β-arrestin
RhoA
PIP2
PIP3
lipid raft
(e.g. MHC I)
(e.g. FOLR)
caveolin
TGF-beta signaling pathway
AMPH
EHD2
Phosphatidylinositol signaling system
PI(4,5)P2
PI(3,4,5)P3
N-WASP
Arp2/3
WIPF
PAR3
aPKC
PAR6
CDC42
Ub
Rab4
Rab35
Rab11
FIP2
Clathrin-coated vesicle
uncoating
Hsc70
auxilin
Fast recycling
ERC
endosomal recycling compartment
Slow recycling
ARF6-dependent recycling
Caveola vesicle
Caveosome
To ER
inactive form
GDP
Arf
Pi
GTP
ArfGAP
ArfGEF
active form
protrudin
KIF5
EHD1
Rab22
Rab10
rabaptin5
Rab4
rabenosyn5
VPS45
Rab8
Rab5
Snx4
Rab35
EEA1
EHD4
rabip4
EHD3
ARF6
Rab11
FIP3
PI(3)P
CAPZB
CAPZA
SWIP
Strump
Arp2/3
WASH
FAM21
CCDC53
Early endosome
pH 6.2
VPS29
VPS26
VPS35
Snx3/12
IST1
CHMP5
CHMP1
VPS4
VTA1
AAA-ATPase
ADP
ATP
UBPY
AMSH
Rab7
Alix
CHMP7
Deubiquitinating enzymes
STAM
Hrs
MVB12
VPS28
TSG101
VPS37
VPS36
VPS22
VPS25
CHMP6
CHMP4
CHMP2
CHMP3
PI(3,5)P2
disassembly of ESCRTs
ESCRT-0
ESCRT-I
ESCRT-II
ESCRT-III
SNARE interactions in vesicular transport
M6P
ACP33
Late endosome and multivesicular body
pH 5.2
invaginating MVB vesicle
Trans Golgi network
CI-MPR
SNX1/2
SNX5
Golgi body
Lysosome
04144 8/30/17
(c) Kanehisa Laboratories

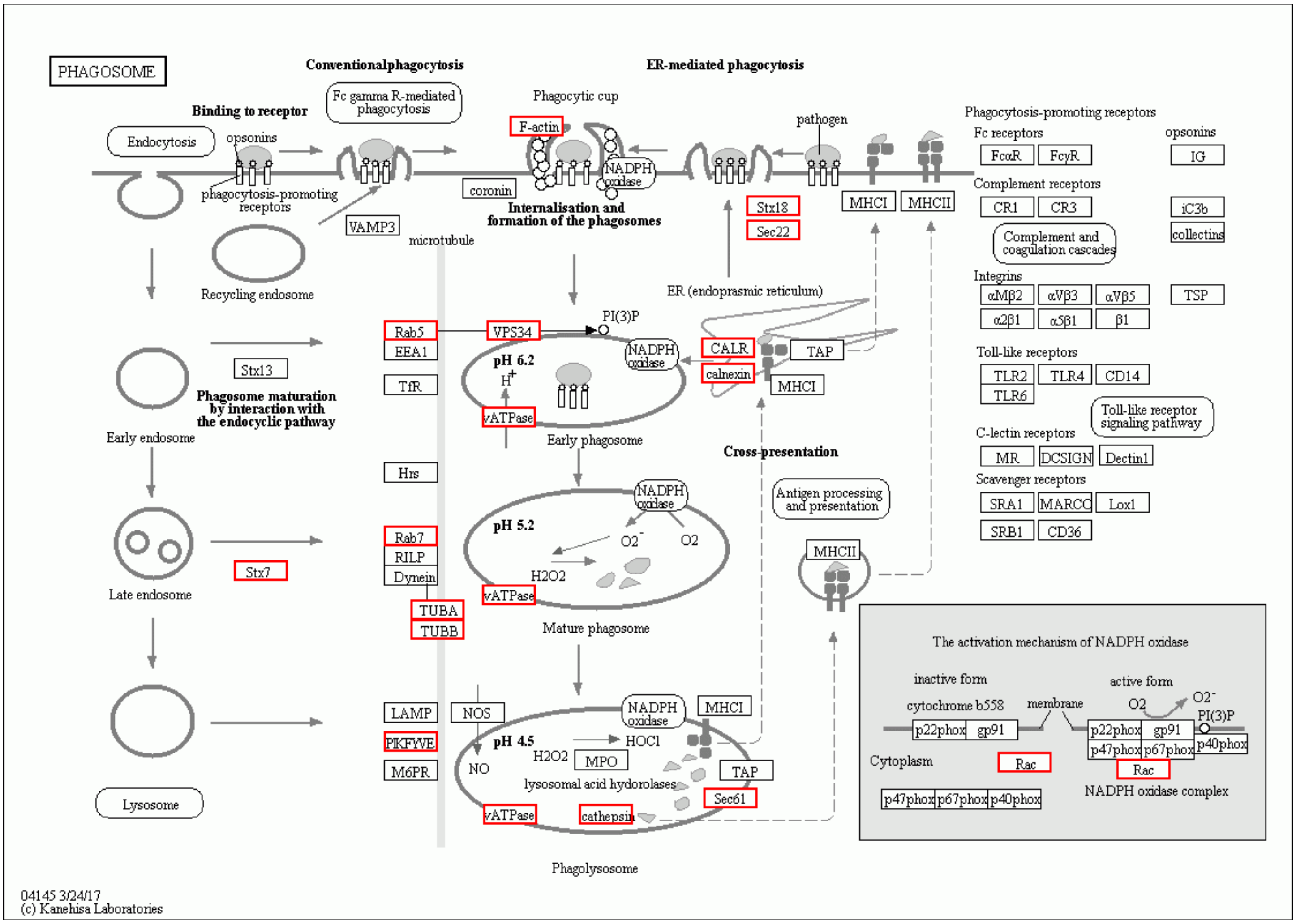
PHAGOSOME
Conventionalphagocytosis
ER-mediated phagocytosis
Binding to receptor
Fc gamma R-mediated phagocytosis
Phagocytic cup
pathogen
Phagocytosis-promoting receptors
Endocytosis
opsonins
F-actin
NADPH oxidase
phagocytosis-promoting receptors
coronin
Internalisation and formation of the phagosomes
Stx18
Sec22
MHCI
MHCII
VAMP3
microtubule
Recycling endosome
ER (endoprasmic reticulum)
Rab5
VPS34
PI(3)P
EEA1
TfR
pH 6.2
H+
CALR
calnexin
TAP
MHCI
vATPase
Early phagosome
Stx13
Phagosome maturation by interaction with the endocyclic pathway
Early endosome
Cross-presentation
Hrs
pH 5.2
O2-
O2
H2O2
Antigen processing and presentation
Rab7
RILP
Dynein
TUBA
TUBB
Stx7
Late endosome
Mature phagosome
LAMP
NOS
PIKFYVE
M6PR
pH 4.5
HOCl
H2O2
MPO
NO
lysosomal acid hydrolases
TAP
Sec61
cathepsin
Lysosome
Phagolysosome
Fc receptors
FcαR
FcγR
IG
Complement receptors
CR1
CR3
iC3b
collectins
Complement and coagulation cascades
Integrins
αMβ2
αVβ3
αVβ5
TSP
α2β1
α5β1
β1
Toll-like receptors
TLR2
TLR4
CD14
TLR6
Toll-like receptor signaling pathway
C-lectin receptors
MR
DCSIGN
Dectin1
Scavenger receptors
SRA1
MARCO
Lox1
SRB1
CD36
The activation mechanism of NADPH oxidase
inactive form
active form
cytochrome b558
membrane
O2
O2-
PI(3)P
p22phox
gp91
p47phox
p67phox
p40phox
Cytoplasm
Rac
NADPH oxidase complex
04145 3/24/17
(c) Kanehisa Laboratories

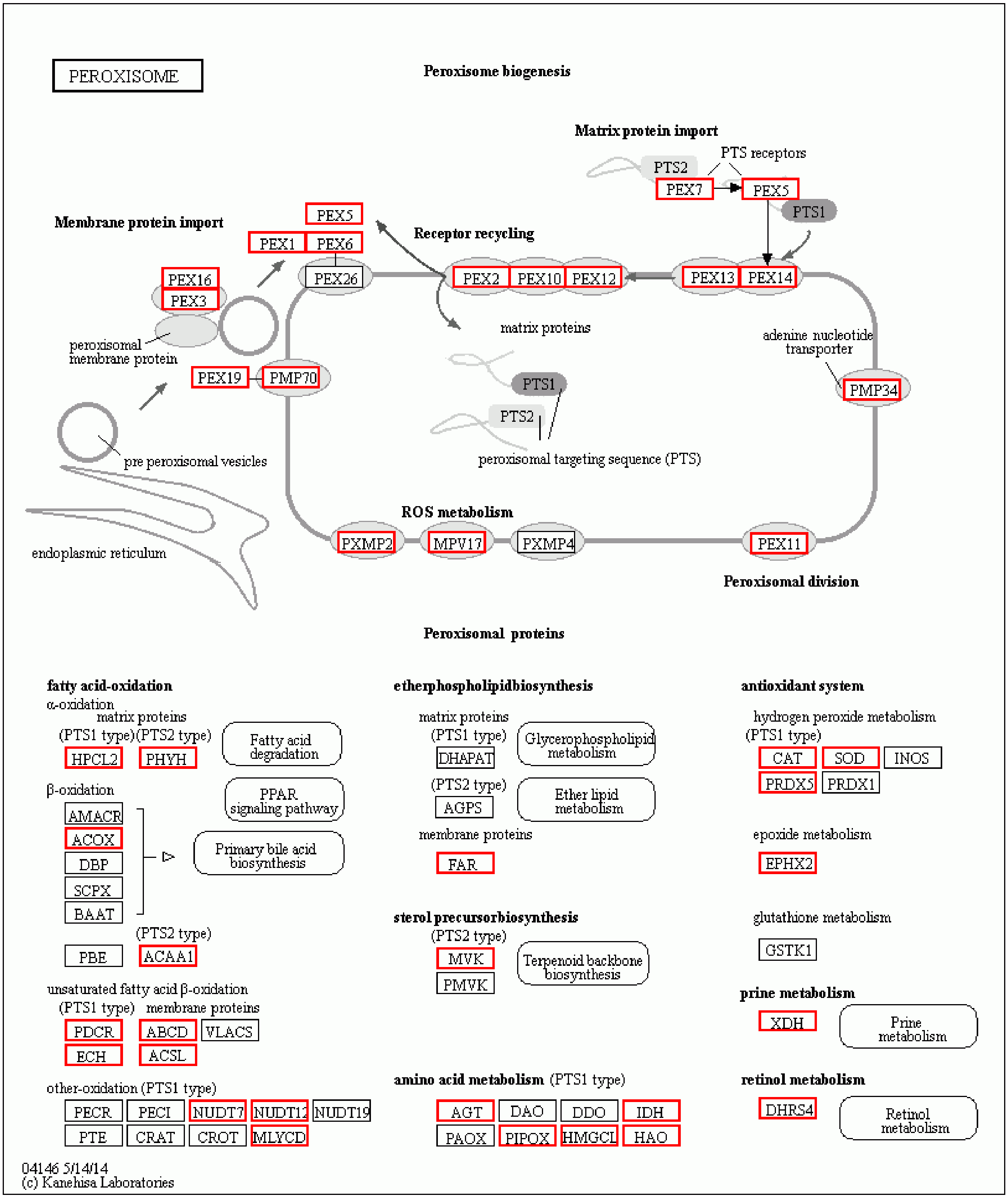
PEROXISOME
Peroxisome biogenesis
Matrix protein import
PTS receptors
PTS2
PEX7
PEX5
PTS1
Membrane protein import
PEX5
PEX1
PEX6
Receptor recycling
PEX16
PEX3
PEX26
PEX2
PEX10
PEX12
PEX13
PEX14
matrix proteins
peroxisomal membrane protein
adenine nucleotide transporter
PEX19
PMP70
PTS1
PMP34
PTS2
pre peroxisomal vesicles
peroxisomal targeting sequence (PTS)
ROS metabolism
endoplasmic reticulum
PXMP2
MPV17
PXMP4
PEX11
Peroxisomal division
Peroxisomal proteins
fatty acid-oxidation
α-oxidation
matrix proteins
(PTS1 type)(PTS2 type)
HPCL2
PHYH
Fatty acid degradation
β-oxidation
PPAR signaling pathway
AMACR
ACOX
DBP
SCPX
BAAT
Primary bile acid biosynthesis
(PTS2 type)
PBE
ACAA1
unsaturated fatty acid β-oxidation
(PTS1 type) membrane proteins
PDCR
ABCD
VLACS
ECH
ACSL
other-oxidation (PTS1 type)
PECR
PECI
NUDT7
NUDT12
NUDT19
PTE
CRAT
CROT
MLYCD
etherphospholipidbiosynthesis
matrix proteins
(PTS1 type)
DHAPAT
Glycerophospholipid metabolism
(PTS2 type)
AGPS
Ether lipid metabolism
membrane proteins
FAR
sterol precursorbiosynthesis
(PTS2 type)
MVK
PMVK
Terpenoid backbone biosynthesis
amino acid metabolism (PTS1 type)
AGT
DAO
DDO
IDH
PAOX
PIPOX
HMGCL
HAO
antioxidant system
hydrogen peroxide metabolism
(PTS1 type)
CAT
SOD
INOS
PRDX5
PRDX1
epoxide metabolism
EPHX2
glutathione metabolism
GSTK1
prine metabolism
XDH
Prine metabolism
retinol metabolism
DHRS4
Retinol metabolism
04146 5/14/14
(c) Kanehisa Laboratories

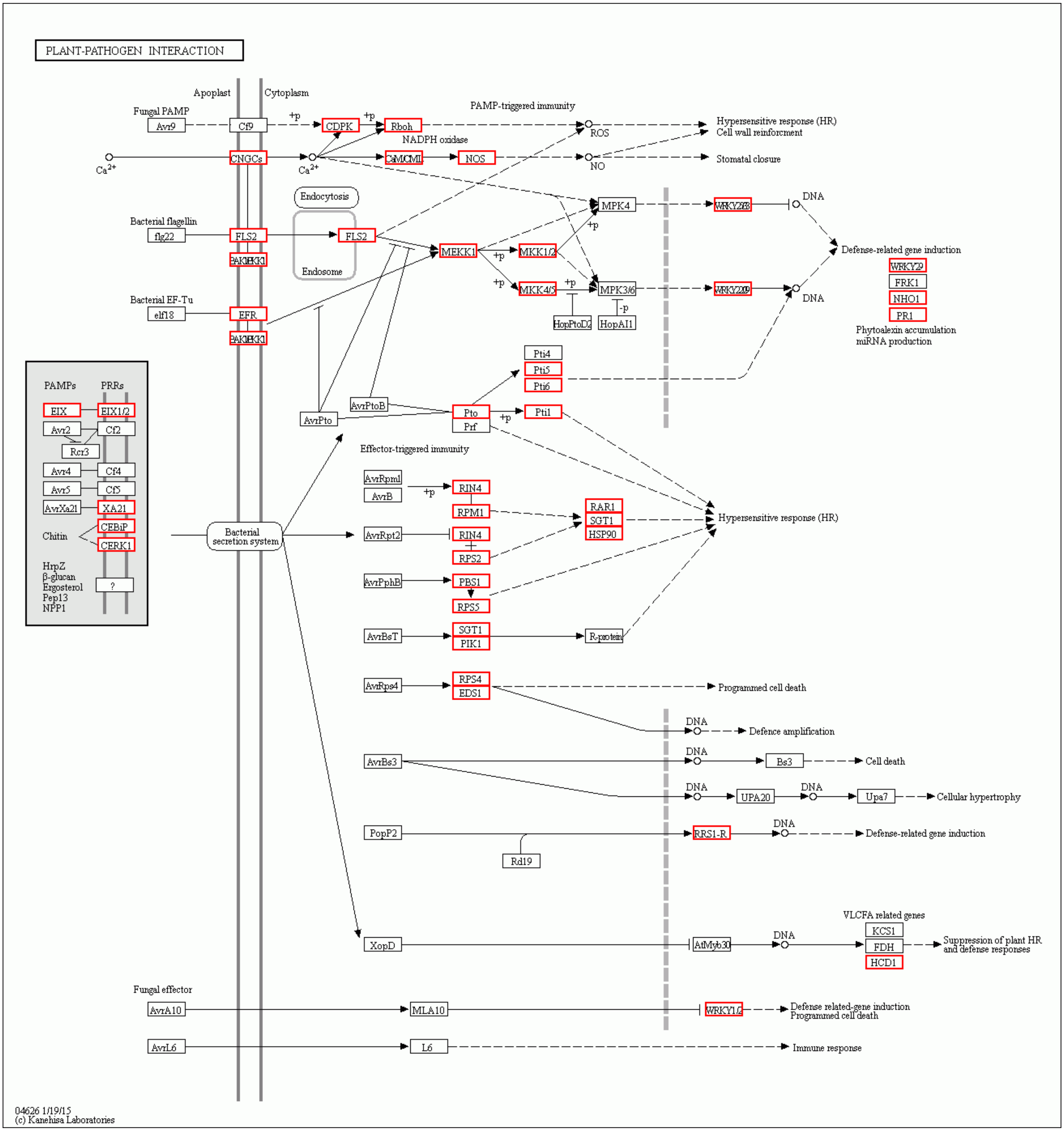

04626 1/19/15
(c) Kanehisa Laboratories

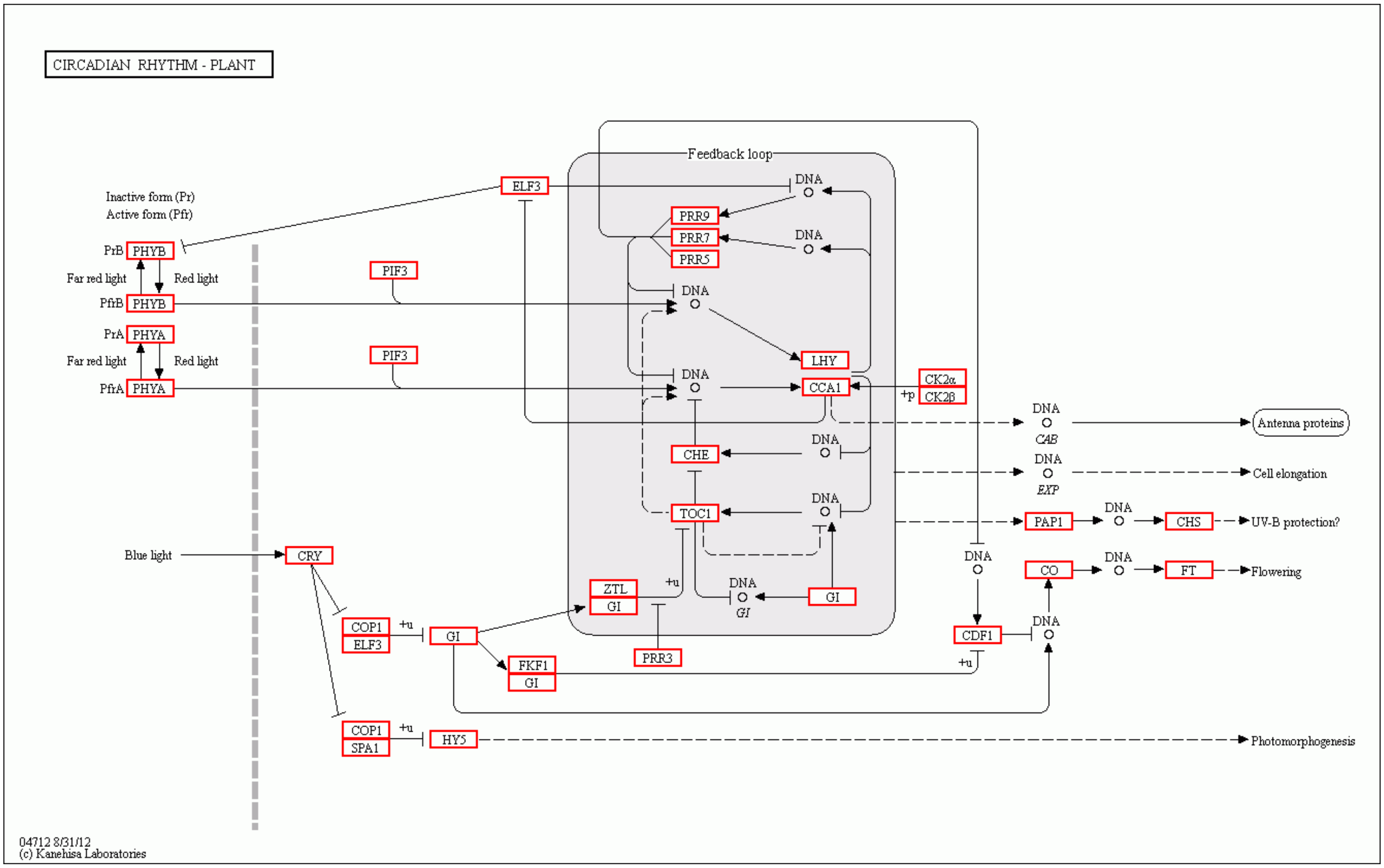
CIRCADIAN RHYTHM - PLANT
Feedback loop
Inactive form (Pr)
Active form (Pfr)
PrB
PHYB
Far red light
Red light
PfrB
PrA
PHYA
PfrA
PIF3
ELF3
DNA
PRR9
PRR7
PRR5
LHY
CCA1
CK2α
CK2β
+p
CHE
TOC1
ZTL
GI
+u
PRR3
Blue light
CRY
COP1
ELF3
FKF1
SPA1
HY5
CDF1
CO
FT
PAP1
CHS
CAB
EXP
Antenna proteins
Cell elongation
UV-B protection?
Flowering
Photomorphogenesis
04712 8/31/12
(c) Kanehisa Laboratories

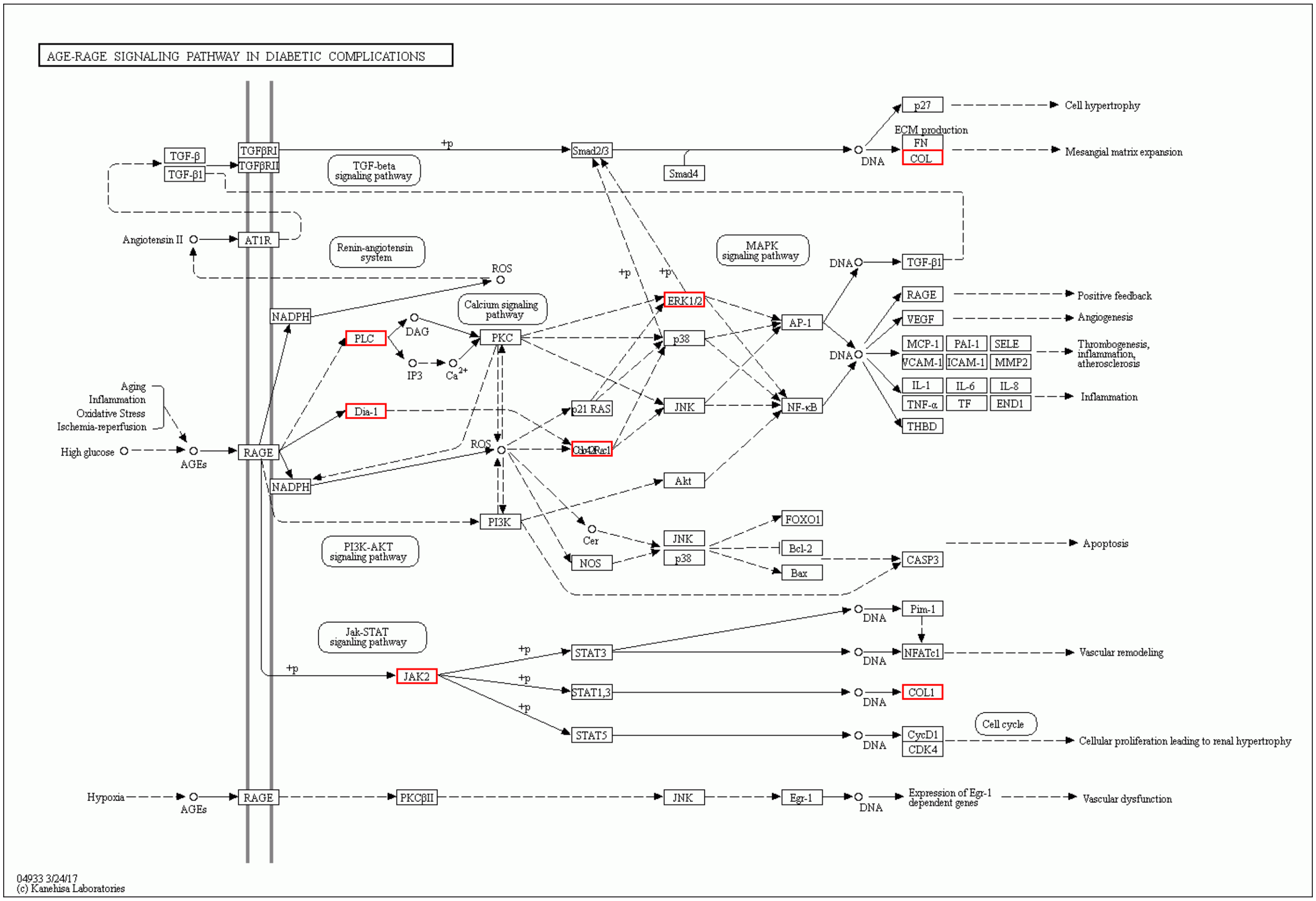
AGE-RAGE SIGNALING PATHWAY IN DIABETIC COMPLICATIONS
TGF-β
TGF-β1
TGFβRI
TGFβRII
TGF-beta signaling pathway
+p
Smad2/3
Smad4
DNA
p27
ECM production
FN
COL
Cell hypertrophy
Mesangial matrix expansion
Angiotensin II
AT1R
Renin-angiotensin system
MAPK signaling pathway
TGF-β1
ROS
NADPH
Calcium signaling pathway
PLC
DAG
IP3
Ca2+
PKC
ERK1/2
p38
AP-1
RAGE
VEGF
MCP-1
PAI-1
SELE
VCAM-1
ICAM-1
MMP2
IL-1
IL-6
IL-8
TNF-α
TF
END1
THBD
Positive feedback
Angiogenesis
Thrombogenesis, inflammation, atherosclerosis
Inflammation
Aging
Inflammation
Oxidative Stress
Ischemia-reperfusion
High glucose
AGEs
Dia-1
p21 RAS
JNK
NF-κB
Cdc42/Rac1
Akt
PI3K
PI3K-AKT signaling pathway
Cer
NOS
FOXO1
Bcl-2
Bax
CASP3
Apoptosis
Jak-STAT siganling pathway
JAK2
STAT3
STAT1,3
STAT5
Pim-1
NFATc1
COL1
CycD1
CDK4
Cell cycle
Vascular remodeling
Cellular proliferation leading to renal hypertrophy
Hypoxia
PKCβII
Egr-1
Expression of Egr-1 dependent genes
Vascular dysfunction
04933 3/24/17
(c) Kanehisa Laboratories

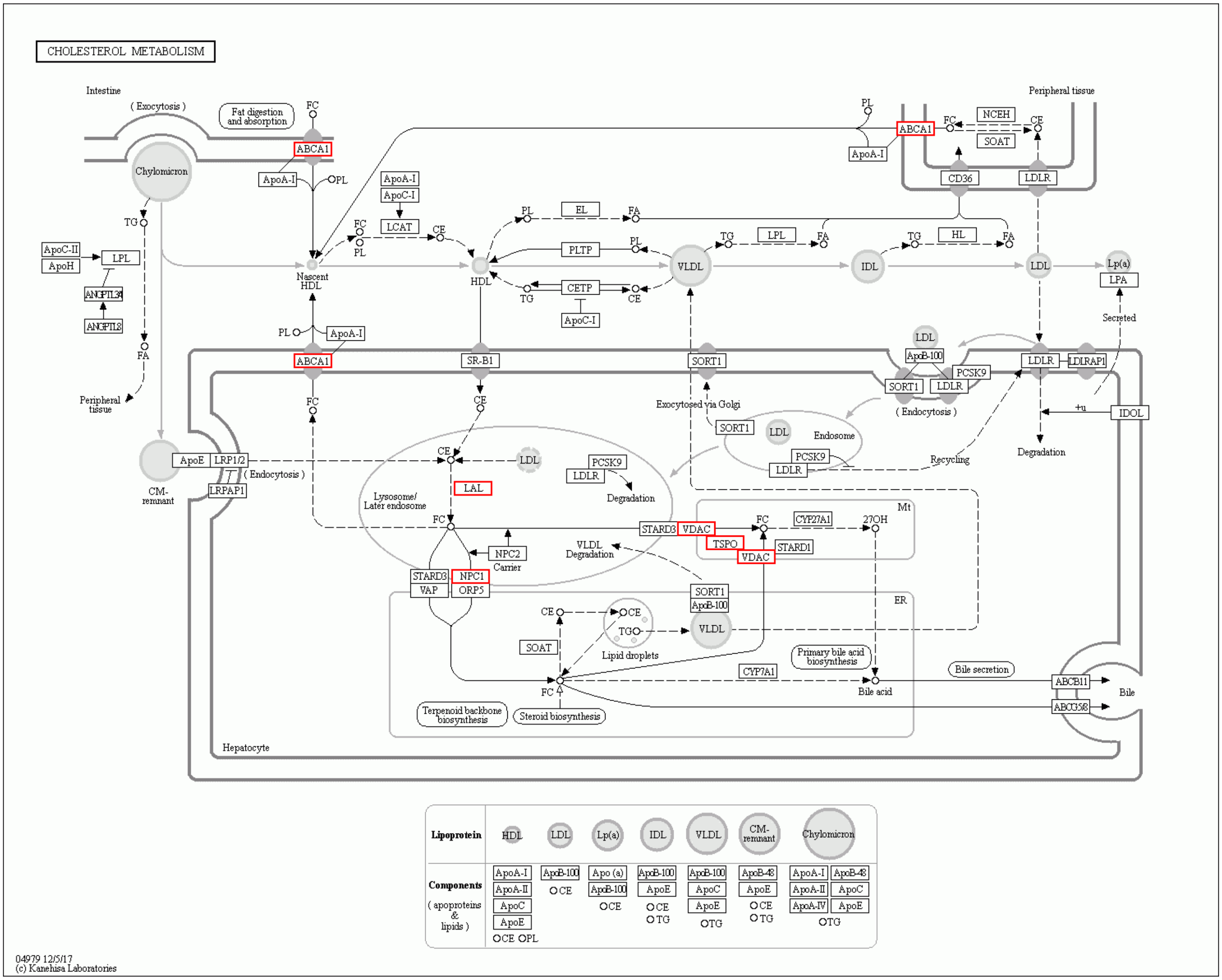
CHOLESTEROL METABOLISM
Intestine
(Exocytosis)
Fat digestion and absorption
FC
ABCA1
Chylomicron
ApoA-I
PL
ApoA-I
ApoC-I
LCAT
CE
TG
ApoC-II
ApoH
LPL
ANGPTL3/4
ANGPTL8
FA
Peripheral tissue
Nascent HDL
HDL
EL
PLTP
CETP
ApoC-I
VLDL
LPL
IDL
HL
LDL
Lp(a)
LPA
Secreted
Peripheral tissue
NCEH
SOAT
CD36
LDLR
SR-B1
SORT1
ApoB-100
PCSK9
LDLRAP1
(Endocytosis)
IDOL
+u
Degradation
Exocytosed via Golgi
Endosome
Recycling
ApoE
LRP1/2
LRPAP1
(Endocytosis)
CM-remnant
Lysosome/ Later endosome
LAL
NPC2
Carrier
STARD3
VAP
NPC1
ORP5
VLDL Degradation
VDAC
TSPO
STARD1
CYP27A1
27OH
Mt
ER
Lipid droplets
Primary bile acid biosynthesis
CYP7A1
Bile acid
Bile secretion
ABCB11
ABCG5/8
Bile
Terpenoid backbone biosynthesis
Steroid biosynthesis
Hepatocyte
Lipoprotein
Chylomicron
Components
(apoproteins & lipids)
ApoA-I
ApoA-II
ApoC
ApoE
CE
PL
ApoB-100
Apo (a)
ApoB-48
ApoA-IV
04979 12/5/17
(c) Kanehisa Laboratories
