# Supplementary material for: De novo transcriptome assembly of Dalbergia sissoo Roxb. (Fabaceae) under Botryodiplodia theobromae-induced dieback disease
Source: Sci Rep. 2023 Nov 22;13:20503. doi: 10.1038/s41598-023-45982-8 (PMC10665356; doi:10.1038/s41598-023-45982-8)
Supplement: Supplementary file 3 — Supplementary Information 3. [file 41598_2023_45982_MOESM3_ESM.pdf]

Supplementary File S3: Datasets of DAGs (Directed Acyclic Graphs) of the GO processes in identified DEGs of all samples.

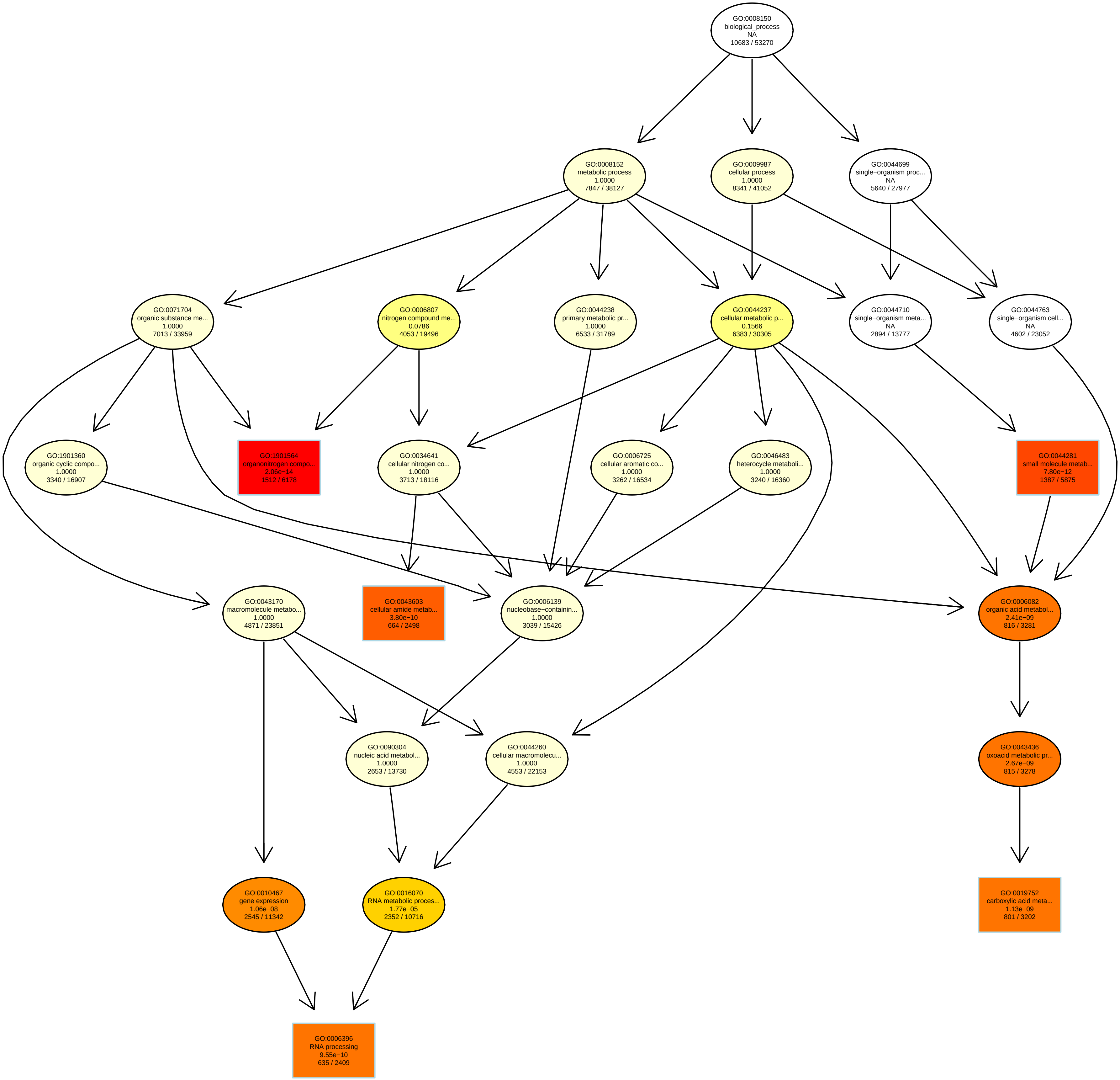
GO:0008150 biological_process NA 10683 / 53270
GO:0008152 metabolic process 1.0000 7847 / 38127
GO:0009987 cellular process 1.0000 8341 / 41052
GO:0044699 single−organism proc... NA 5640 / 27977
GO:0071704 organic substance me... 1.0000 7013 / 33959
GO:0006807 nitrogen compound me... 0.0786 4053 / 19496
GO:0044238 primary metabolic pr... 1.0000 6533 / 31789
GO:0044237 cellular metabolic p... 0.1566 6383 / 30305
GO:0044710 single−organism meta... NA 2894 / 13777
GO:0044763 single−organism cell... NA 4602 / 23052
GO:1901360 organic cyclic compo... 1.0000 3340 / 16907
GO:1901564 organonitrogen compo... 2.06e−14 1512 / 6178
GO:0034641 cellular nitrogen co... 1.0000 3713 / 18116
GO:0006725 cellular aromatic co... 1.0000 3262 / 16534
GO:0046483 heterocycle metaboli... 1.0000 3240 / 16360
GO:0044281 small molecule metab... 7.80e−12 1387 / 5875
GO:0043170 macromolecule metabo... 1.0000 4871 / 23851
GO:0043603 cellular amide metab... 3.80e−10 664 / 2498
GO:0006139 nucleobase−containin... 1.0000 3039 / 15426
GO:0006082 organic acid metabol... 2.41e−09 816 / 3281
GO:0090304 nucleic acid metabol... 1.0000 2653 / 13730
GO:0044260 cellular macromolecu... 1.0000 4553 / 22153
GO:0043436 oxoacid metabolic pr... 2.67e−09 815 / 3278
GO:0010467 gene expression 1.06e−08 2545 / 11342
GO:0016070 RNA metabolic proces... 1.77e−05 2352 / 10716
GO:0019752 carboxylic acid meta... 1.13e−09 801 / 3202
GO:0006396 RNA processing 9.55e−10 635 / 2409

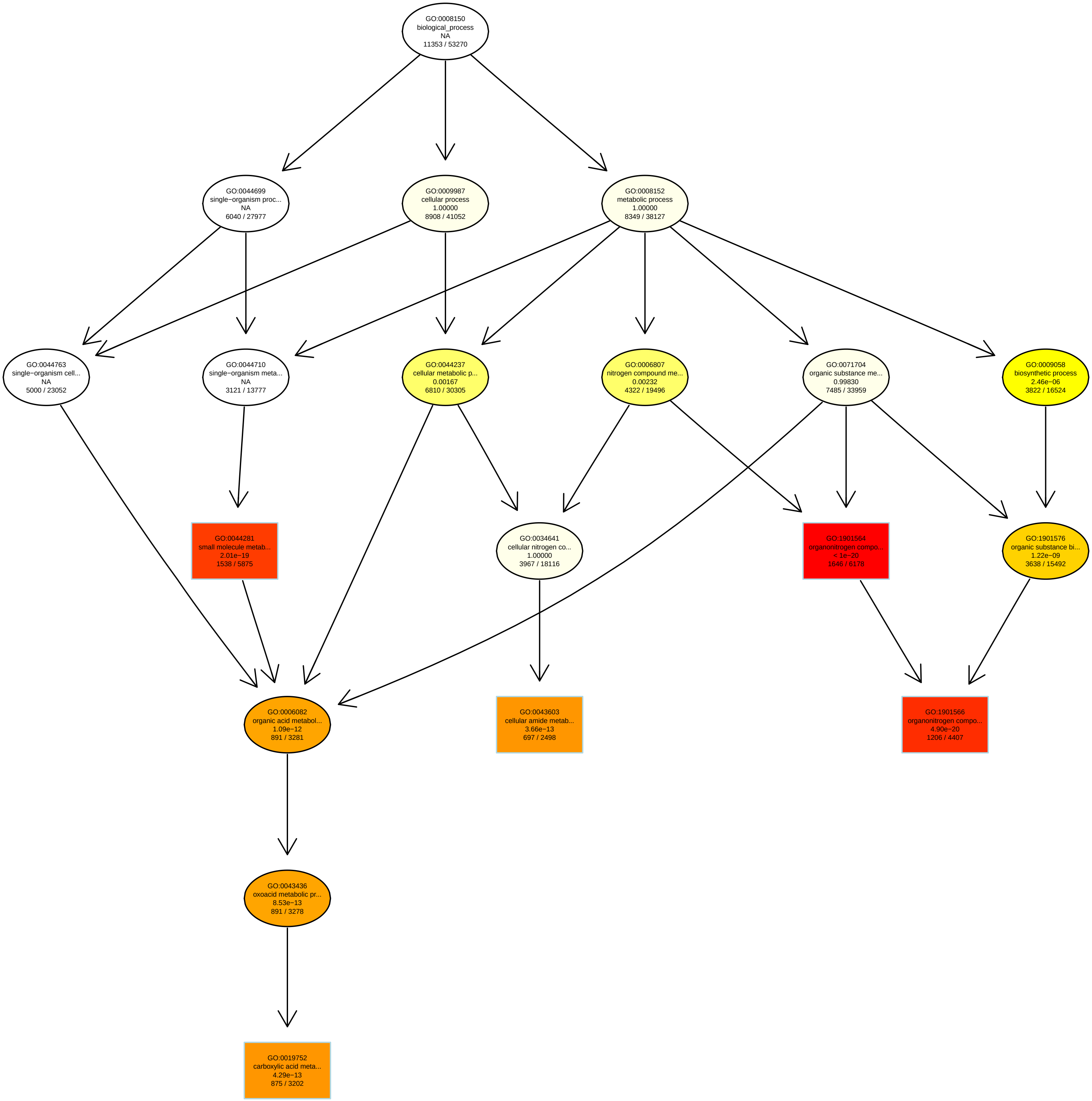
GO:0008150
biological_process
NA
11353 / 53270
GO:0044699
single−organism proc...
NA
6040 / 27977
GO:0009987
cellular process
1.00000
8908 / 41052
GO:0008152
metabolic process
1.00000
8349 / 38127
GO:0044763
single−organism cell...
NA
5000 / 23052
GO:0044710
single−organism meta...
NA
3121 / 13777
GO:0044237
cellular metabolic p...
0.00167
6810 / 30305
GO:0006807
nitrogen compound me...
0.00232
4322 / 19496
GO:0071704
organic substance me...
0.99830
7485 / 33959
GO:0009058
biosynthetic process
2.46e−06
3822 / 16524
GO:0044281
small molecule metab...
2.01e−19
1538 / 5875
GO:0034641
cellular nitrogen co...
1.00000
3967 / 18116
GO:1901564
organonitrogen compo...
< 1e−20
1646 / 6178
GO:1901576
organic substance bi...
1.22e−09
3638 / 15492
GO:0006082
organic acid metabol...
1.09e−12
891 / 3281
GO:0043603
cellular amide metab...
3.66e−13
697 / 2498
GO:1901566
organonitrogen compo...
4.90e−20
1206 / 4407
GO:0043436
oxoacid metabolic pr...
8.53e−13
891 / 3278
GO:0019752
carboxylic acid meta...
4.29e−13
875 / 3202

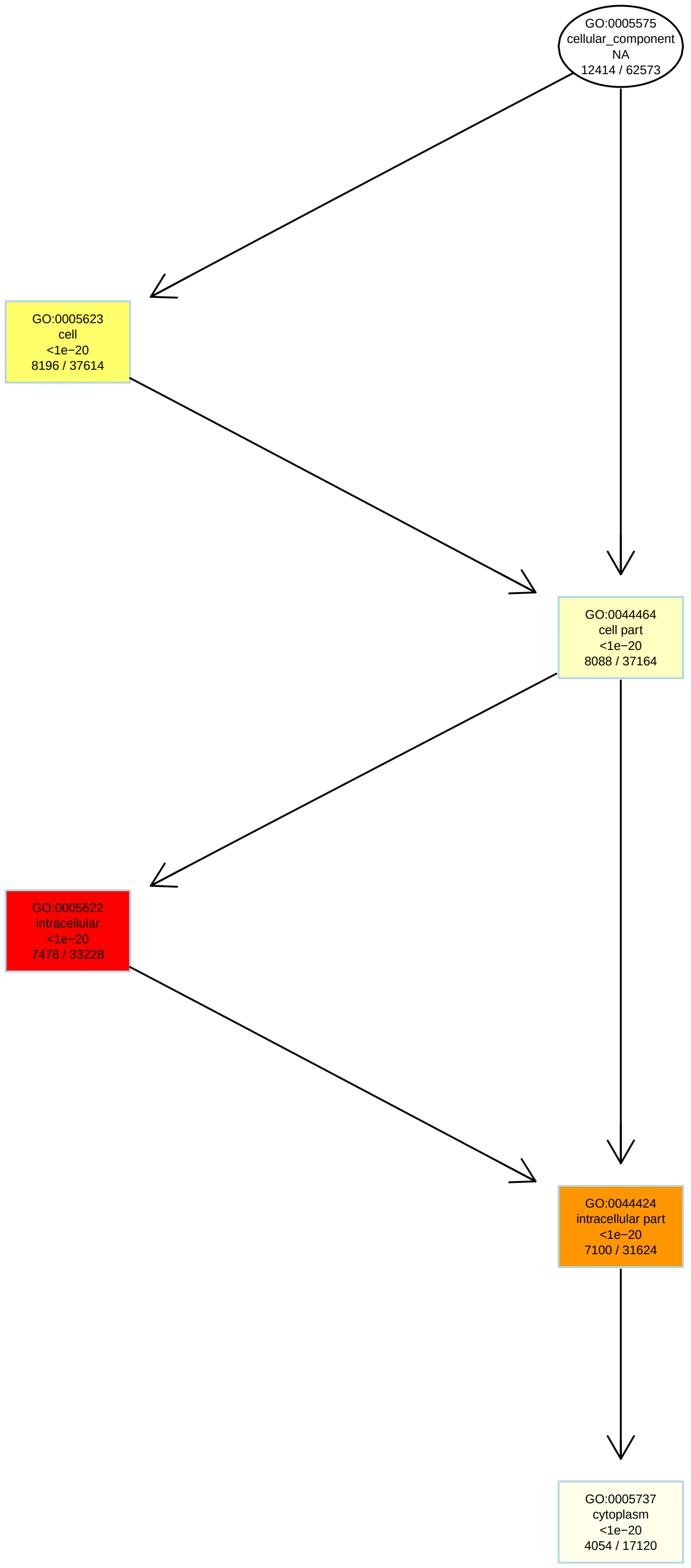
GO:0005575
cellular_component
NA
12414 / 62573
GO:0005623
cell
<1e−20
8196 / 37614
GO:0044464
cell part
<1e−20
8088 / 37164
GO:0005622
intracellular
<1e−20
7478 / 33228
GO:0044424
intracellular part
<1e−20
7100 / 31624
GO:0005737
cytoplasm
<1e−20
4054 / 17120

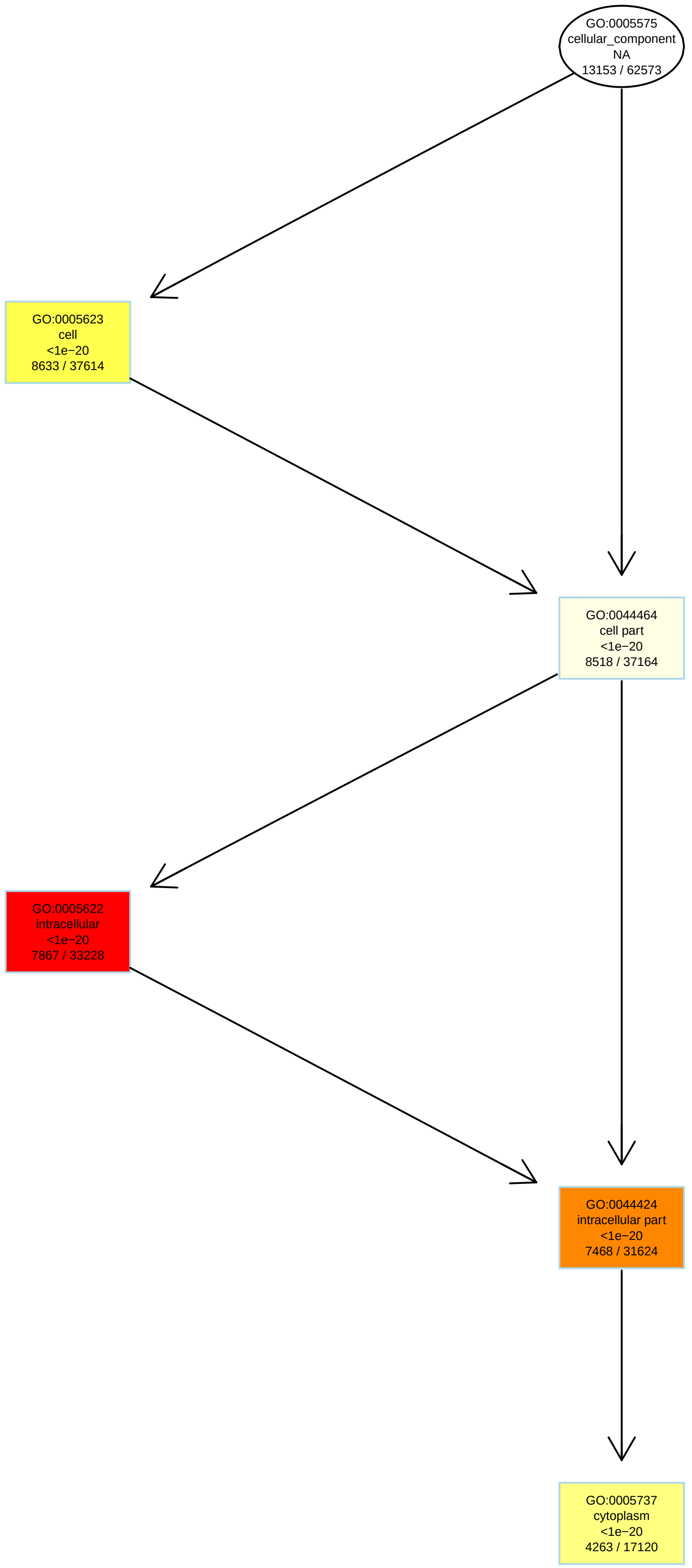
GO:0005575
cellular_component
NA
13153 / 62573
GO:0005623
cell
<1e−20
8633 / 37614
GO:0044464
cell part
<1e−20
8518 / 37164
GO:0005622
intracellular
<1e−20
7867 / 33228
GO:0044424
intracellular part
<1e−20
7468 / 31624
GO:0005737
cytoplasm
<1e−20
4263 / 17120

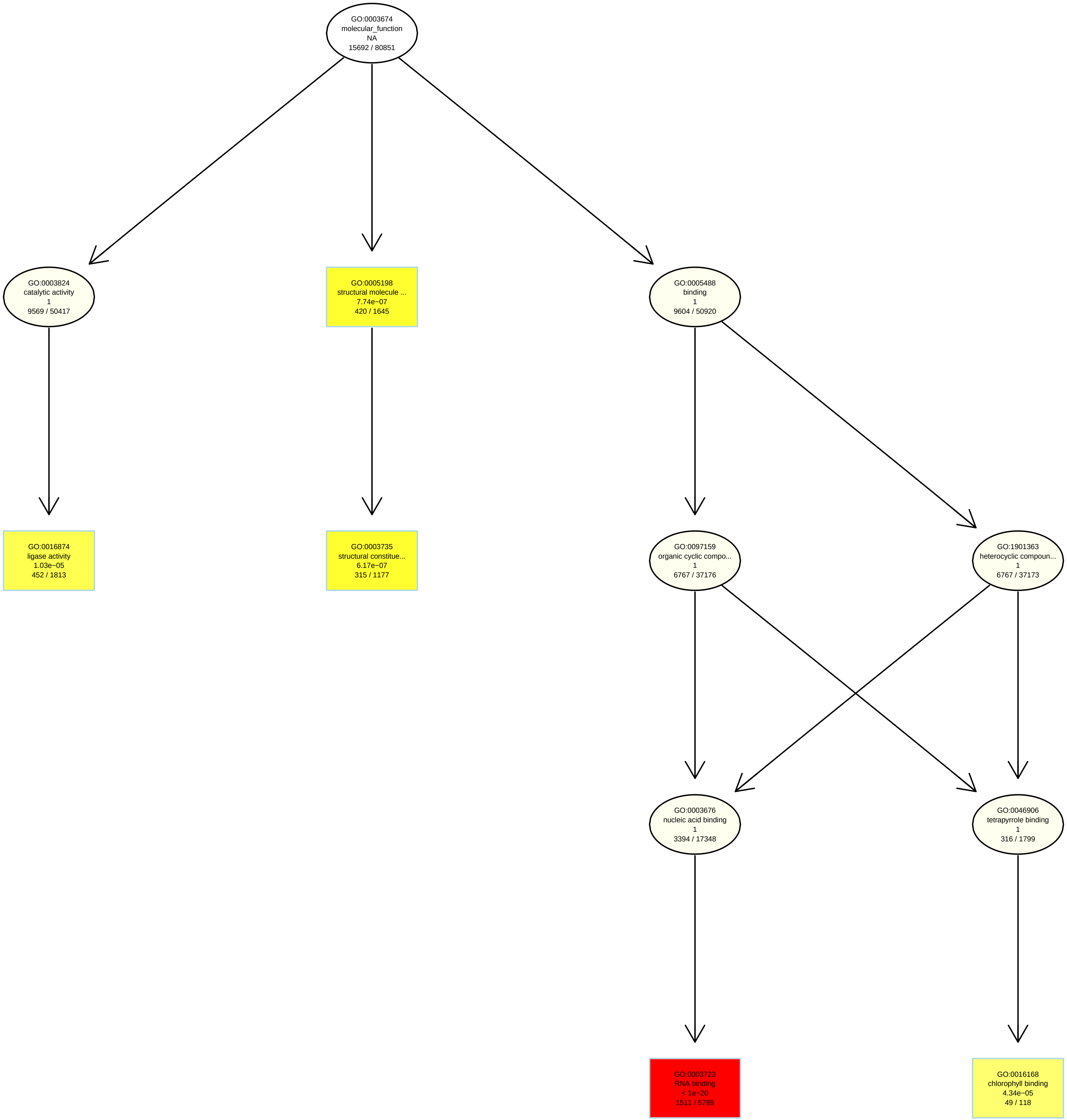

GO:0003674
molecular_function
NA
15692 / 80851
GO:0003824
catalytic activity
1
9569 / 50417
GO:0005198
structural molecule ...
7.74e−07
420 / 1645
GO:0005488
binding
1
9604 / 50920
GO:0016874
ligase activity
1.03e−05
452 / 1813
GO:0003735
structural constitue...
6.17e−07
315 / 1177
GO:0097159
organic cyclic compo...
1
6767 / 37176
GO:1901363
heterocyclic compoun...
1
6767 / 37173
GO:0003676
nucleic acid binding
1
3394 / 17348
GO:0046906
tetrapyrrole binding
1
316 / 1799
GO:0003723
RNA binding
< 1e−20
1511 / 5789
GO:0016168
chlorophyll binding
4.34e−05
49 / 118

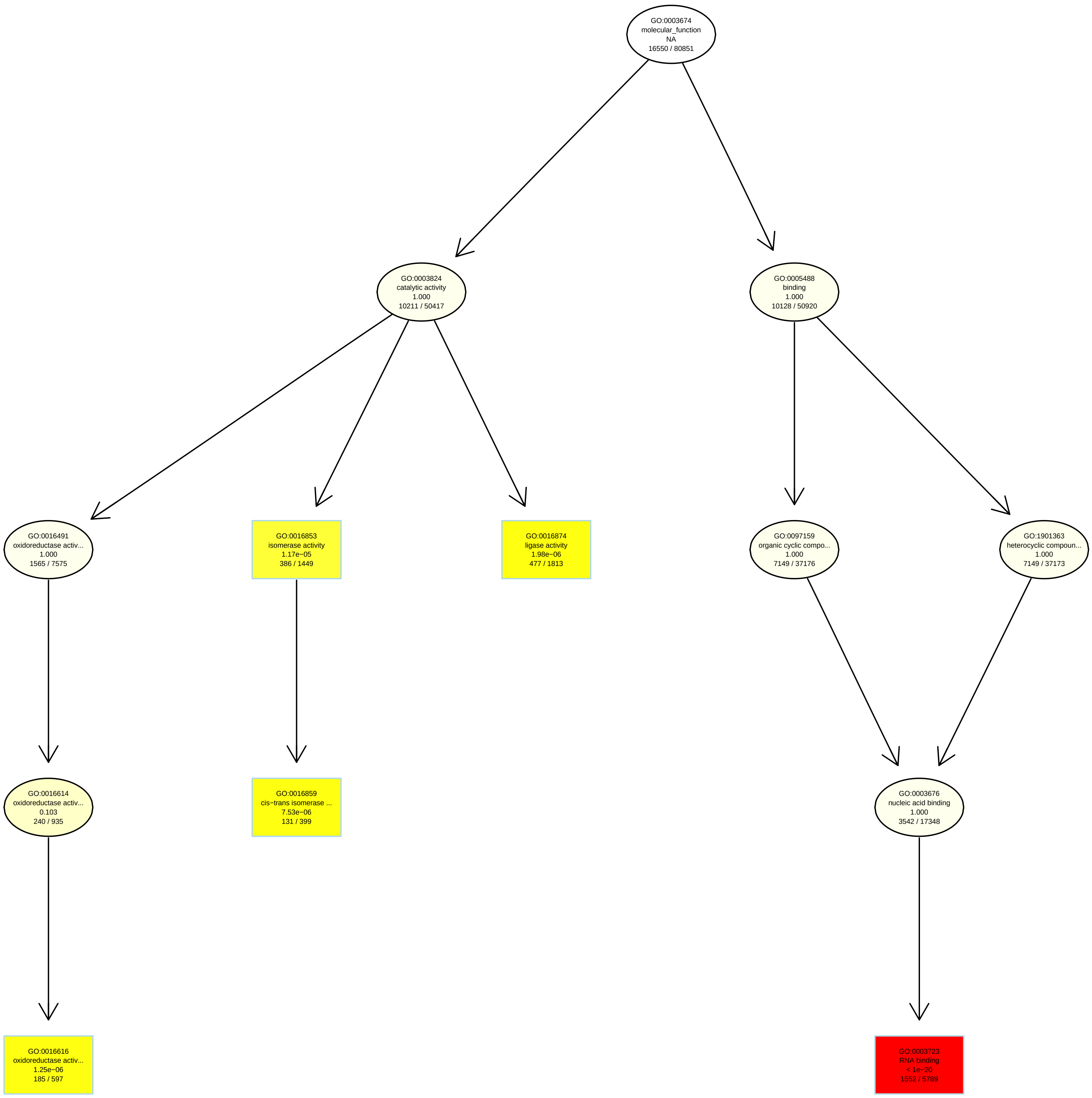
GO:0003674
molecular_function
NA
16550 / 80851
GO:0003824
catalytic activity
1.000
10211 / 50417
GO:0005488
binding
1.000
10128 / 50920
GO:0016491
oxidoreductase activ...
1.000
1565 / 7575
GO:0016853
isomerase activity
1.17e−05
386 / 1449
GO:0016874
ligase activity
1.98e−06
477 / 1813
GO:0097159
organic cyclic compo...
1.000
7149 / 37176
GO:1901363
heterocyclic compoun...
1.000
7149 / 37173
GO:0016614
oxidoreductase activ...
0.103
240 / 935
GO:0016859
cis−trans isomerase ...
7.53e−06
131 / 399
GO:0003676
nucleic acid binding
1.000
3542 / 17348
GO:0016616
oxidoreductase activ...
1.25e−06
185 / 597
GO:0003723
RNA binding
< 1e−20
1552 / 5789
