# Supplementary material for: De novo transcriptome assembly of Dalbergia sissoo Roxb. (Fabaceae) under Botryodiplodia theobromae-induced dieback disease
Source: Sci Rep. 2023 Nov 22;13:20503. doi: 10.1038/s41598-023-45982-8 (PMC10665356; doi:10.1038/s41598-023-45982-8)
Supplement: Supplementary file 4 — Supplementary Information 4. [file 41598_2023_45982_MOESM4_ESM.pdf]

Supplementary File S4: Datasets of KEGG pathway maps in the identified DEGs of all samples.

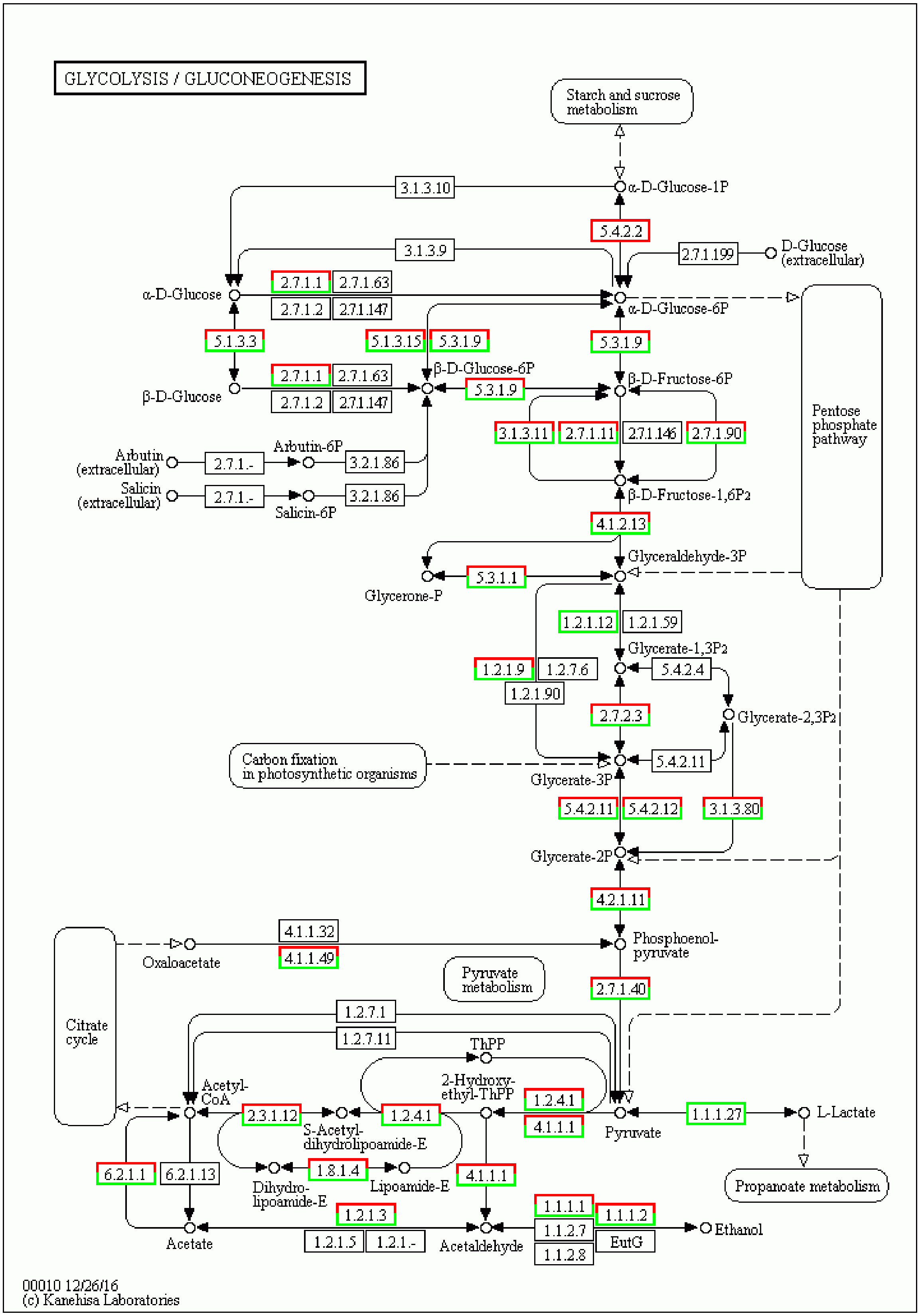
GLYCOLYSIS / GLUCONEOGENESIS
Starch and sucrose metabolism
α-D-Glucose-1P
3.1.3.10
5.4.2.2
3.1.3.9
2.7.1.199
D-Glucose (extracellular)
α-D-Glucose
2.7.1.1
2.7.1.63
2.7.1.2
2.7.1.147
α-D-Glucose-6P
5.1.3.3
5.1.3.15
5.3.1.9
5.3.1.9
β-D-Glucose
β-D-Glucose-6P
β-D-Fructose-6P
2.7.1.1
2.7.1.63
2.7.1.2
2.7.1.147
5.3.1.9
3.1.3.11
2.7.1.11
2.7.1.146
2.7.1.90
Pentose phosphate pathway
Arbutin (extracellular)
2.7.1.-
Arbutin-6P
3.2.1.86
Salicin (extracellular)
2.7.1.-
Salicin-6P
3.2.1.86
β-D-Fructose-1,6P2
4.1.2.13
Glyceraldehyde-3P
Glycerone-P
5.3.1.1
1.2.1.12
1.2.1.59
Glycerate-1,3P2
1.2.1.9
1.2.7.6
5.4.2.4
1.2.1.90
2.7.2.3
Glycerate-2,3P2
Carbon fixation in photosynthetic organisms
5.4.2.11
Glycerate-3P
5.4.2.11
5.4.2.12
3.1.3.80
Glycerate-2P
4.2.1.11
4.1.1.32
4.1.1.49
Oxaloacetate
Phosphoenol-pyruvate
Pyruvate metabolism
2.7.1.40
Citrate cycle
1.2.7.1
1.2.7.11
ThPP
Acetyl-CoA
2-Hydroxy-ethyl-ThPP
2.3.1.12
1.2.4.1
1.2.4.1
4.1.1.1
Pyruvate
1.1.1.27
L-Lactate
S-Acetyl-dihydrolipoamide-E
6.2.1.1
6.2.1.13
Dihydro-lipoamide-E
1.8.1.4
Lipoamide-E
4.1.1.1
Propanoate metabolism
1.2.1.3
1.1.1.1
1.1.1.2
1.2.1.5
1.2.1.-
1.1.2.7
EutG
Ethanol
Acetate
Acetaldehyde
1.1.2.8
00010 12/26/16
(c) Kanehisa Laboratories

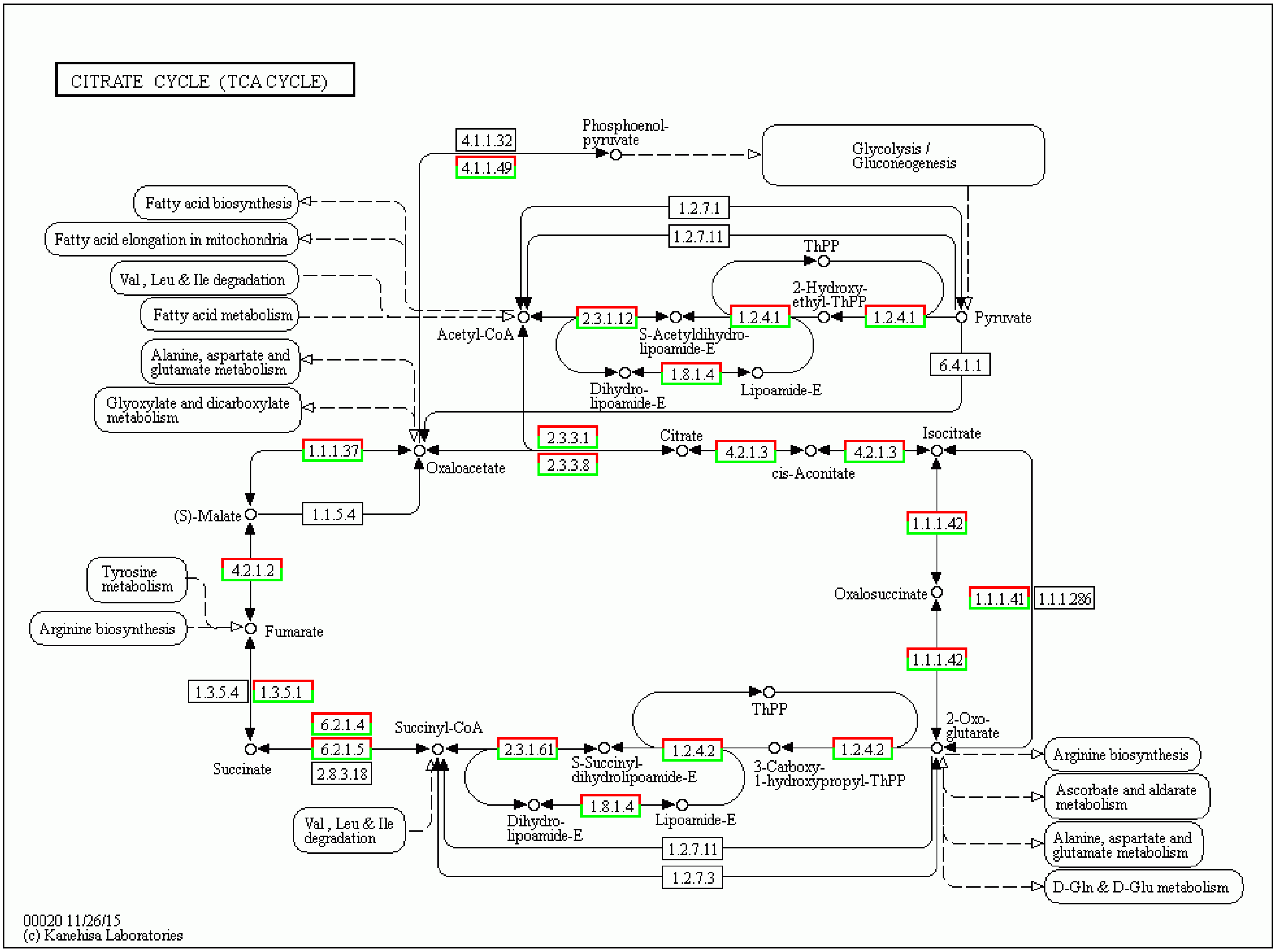
CITRATE CYCLE (TCA CYCLE)
4.1.1.32
4.1.1.49
Phosphoenol-
pyruvate
Glycolysis /
Gluconeogenesis
Fatty acid biosynthesis
Fatty acid elongation in mitochondria
Val, Leu & Ile degradation
Fatty acid metabolism
Alanine, aspartate and
glutamate metabolism
Glyoxylate and dicarboxylate
metabolism
1.2.7.1
1.2.7.11
ThPP
2-Hydroxy-
ethyl-ThPP
2.3.1.12
1.2.4.1
1.2.4.1
Pyruvate
Acetyl-CoA
S-Acetyldihydro-
lipoamide-E
1.8.1.4
Dihydro-
lipoamide-E
Lipoamide-E
6.4.1.1
2.3.3.1
2.3.3.8
Citrate
4.2.1.3
4.2.1.3
Isocitrate
cis-Aconitate
1.1.1.37
Oxaloacetate
(S)-Malate
1.1.5.4
1.1.1.42
4.2.1.2
Tyrosine
metabolism
Arginine biosynthesis
Fumarate
Oxalosuccinate
1.1.1.41
1.1.1.286
1.1.1.42
1.3.5.4
1.3.5.1
6.2.1.4
6.2.1.5
2.8.3.18
Succinate
Succinyl-CoA
2.3.1.61
1.2.4.2
1.2.4.2
ThPP
2-Oxo-
glutarate
S-Succinyl-
dihydrolipoamide-E
3-Carboxy-
1-hydroxypropyl-ThPP
Arginine biosynthesis
Ascorbate and aldarate
metabolism
Alanine, aspartate and
glutamate metabolism
D-Gln & D-Glu metabolism
1.8.1.4
Dihydro-
lipoamide-E
Lipoamide-E
Val, Leu & Ile
degradation
1.2.7.11
1.2.7.3
00020 11/26/15
(c) Kanehisa Laboratories

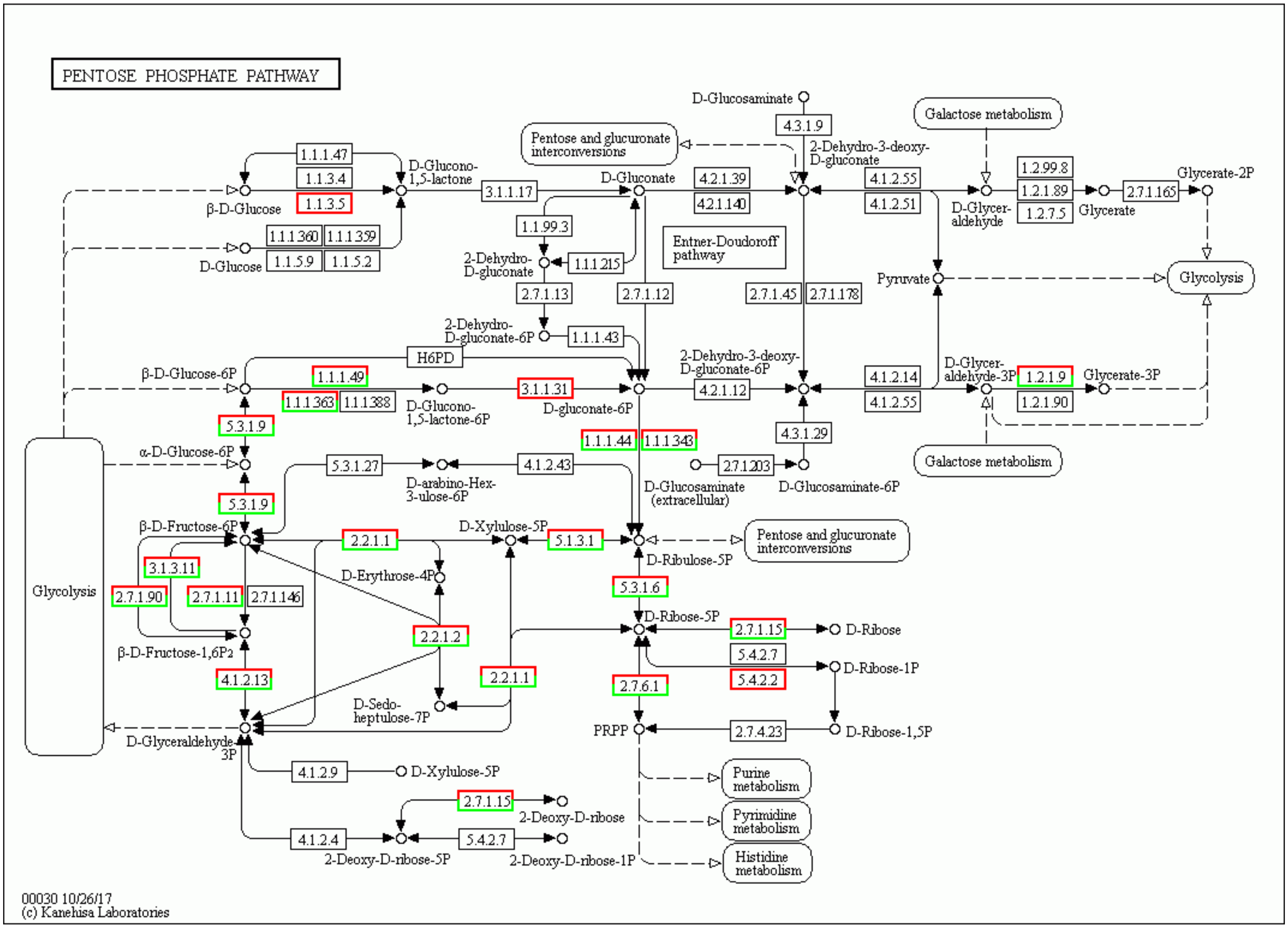
PENTOSE PHOSPHATE PATHWAY
D-Glucosaminate
4.3.1.9
Galactose metabolism
Pentose and glucuronate interconversions
2-Dehydro-3-deoxy-D-gluconate
1.1.1.47
1.1.3.4
1.1.3.5
D-Glucono-1,5-lactone
3.1.1.17
D-Gluconate
4.2.1.39
4.2.1.140
4.1.2.55
4.1.2.51
1.2.99.8
1.2.1.89
1.2.7.5
2.7.1.165
Glycerate-2P
Glycerate
D-Glyceraldehyde
β-D-Glucose
1.1.1.360
1.1.1.359
D-Glucose
1.1.5.9
1.1.5.2
1.1.99.3
2-Dehydro-D-gluconate
1.1.1.215
Entner-Doudoroff pathway
Pyruvate
Glycolysis
2.7.1.13
2.7.1.12
2.7.1.45
2.7.1.178
2-Dehydro-D-gluconate-6P
1.1.1.43
H6PD
β-D-Glucose-6P
1.1.1.49
1.1.1.363
1.1.1.388
D-Glucono-1,5-lactone-6P
3.1.1.31
D-gluconate-6P
2-Dehydro-3-deoxy-D-gluconate-6P
4.2.1.12
4.1.2.14
4.1.2.55
D-Glyceraldehyde-3P
1.2.1.9
1.2.1.90
Glycerate-3P
5.3.1.9
1.1.1.44
1.1.1.343
4.3.1.29
α-D-Glucose-6P
5.3.1.27
D-arabino-Hex-3-ulose-6P
4.1.2.43
2.7.1.203
D-Glucosaminate (extracellular)
D-Glucosamine-6P
5.3.1.9
β-D-Fructose-6P
2.2.1.1
D-Xylulose-5P
5.1.3.1
Pentose and glucuronate interconversions
D-Ribulose-5P
3.1.3.11
D-Erythrose-4P
5.3.1.6
Glycolysis
2.7.1.90
2.7.1.11
2.7.1.146
D-Ribose-5P
2.7.1.15
D-Ribose
2.2.1.2
5.4.2.7
β-D-Fructose-1,6P2
4.1.2.13
2.2.1.1
5.4.2.2
D-Ribose-1P
2.7.6.1
D-Sedo-heptulose-7P
PRPP
2.7.4.23
D-Ribose-1,5P
D-Glyceraldehyde-3P
4.1.2.9
D-Xylulose-5P
Purine metabolism
2.7.1.15
2-Deoxy-D-ribose
Pyrimidine metabolism
4.1.2.4
5.4.2.7
2-Deoxy-D-ribose-5P
2-Deoxy-D-ribose-1P
Histidine metabolism
00030 10/26/17
(c) Kanehisa Laboratories

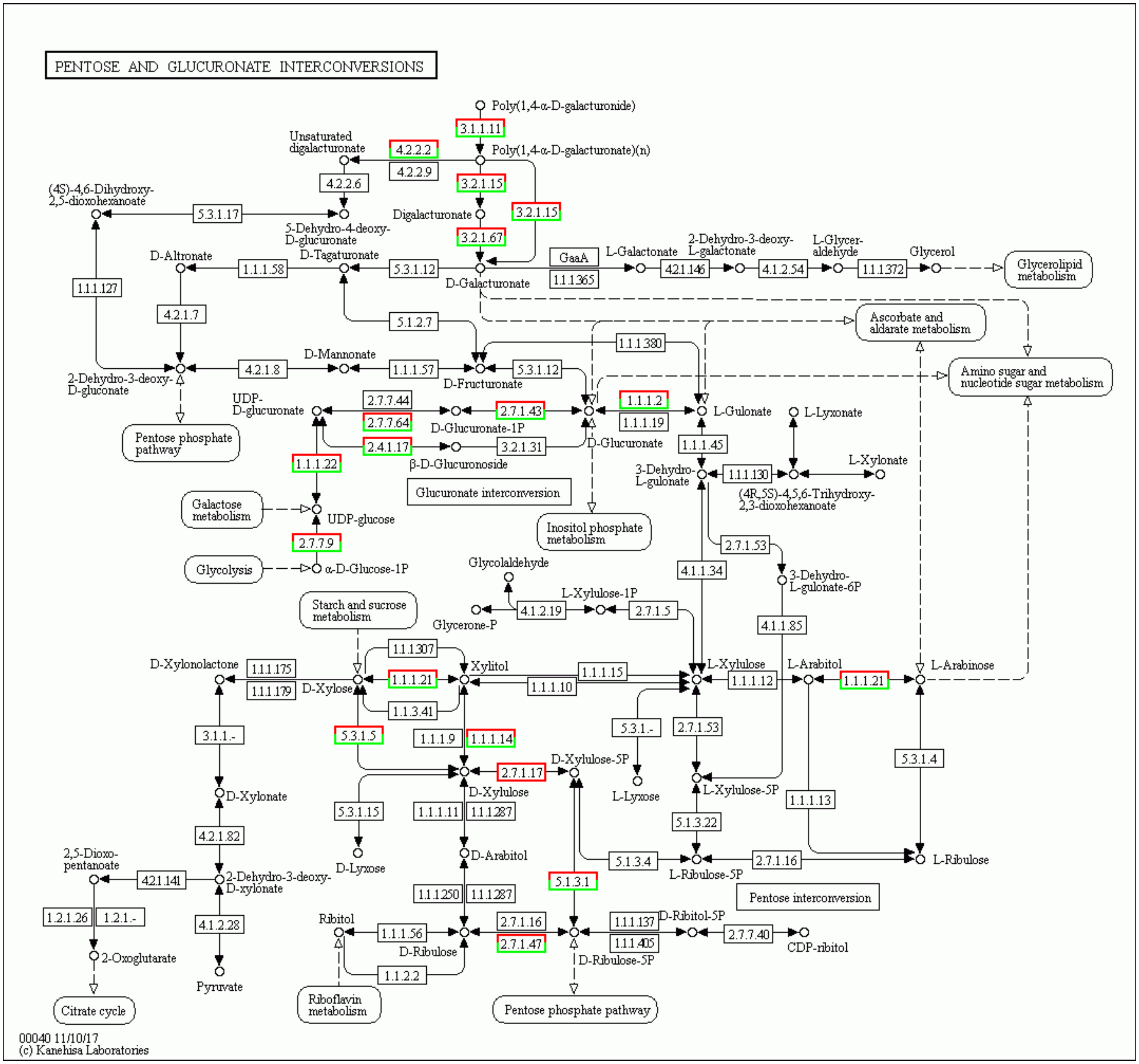
PENTOSE AND GLUCURONATE INTERCONVERSIONS
Poly(1,4-α-D-galacturonide)
3.1.1.11
Poly(1,4-α-D-galacturonate)(n)
Unsaturated digalacturonate
4.2.2.2
4.2.2.9
4.2.2.6
3.2.1.15
3.2.1.15
Digalacturonate
3.2.1.67
(4S)-4,6-Dihydroxy-2,5-dioxohexanoate
5.3.1.17
5-Dehydro-4-deoxy-D-glucuronate
D-Altronate
1.1.1.58
D-Tagaturonate
5.3.1.12
D-Galacturonate
GaaA
1.1.1365
L-Galactonate
4.2.1.146
2-Dehydro-3-deoxy-L-galactonate
4.1.2.54
L-Glyceraldehyde
1.1.1372
Glycerol
Glycerolipid metabolism
1.1.1.127
4.2.1.7
5.1.2.7
Ascorbate and aldarate metabolism
1.1.1380
Amino sugar and nucleotide sugar metabolism
2-Dehydro-3-deoxy-D-gluconate
4.2.1.8
D-Mannonate
1.1.1.57
D-Fructuronate
5.3.1.12
Pentose phosphate pathway
UDP-D-glucuronate
2.7.7.44
2.7.7.64
2.7.1.43
D-Glucuronate-1P
1.1.1.2
1.1.1.19
L-Gulonate
L-Lyxonate
2.4.1.17
3.2.1.31
β-D-Glucuronoside
D-Glucuronate
1.1.1.45
L-Xylonate
1.1.1.22
3-Dehydro-L-gulonate
1.1.1.130
(4R,5S)-4,5,6-Trihydroxy-2,3-dioxohexanoate
Glucuronate interconversion
Galactose metabolism
UDP-glucose
Inositol phosphate metabolism
2.7.7.9
2.7.1.53
Glycolysis
α-D-Glucose-1P
Glycolaldehyde
4.1.1.34
3-Dehydro-L-gulonate-6P
L-Xylulose-1P
4.1.2.19
2.7.1.5
Starch and sucrose metabolism
Glycerone-P
4.1.1.85
1.1.1.307
D-Xylonolactone
1.1.1.175
1.1.1.179
D-Xylose
1.1.1.21
Xylitol
1.1.1.15
1.1.1.10
L-Xylulose
1.1.1.12
L-Arabitol
1.1.1.21
L-Arabinose
1.1.3.41
3.1.1.-
5.3.1.5
1.1.1.9
1.1.1.14
5.3.1.-
2.7.1.53
5.3.1.4
D-Xylulose-5P
2.7.1.17
D-Xylulose
L-Lyxose
L-Xylulose-5P
1.1.1.13
D-Xylonate
5.3.1.15
1.1.1.11
1.1.1.287
5.1.3.22
4.2.1.82
D-Lyxose
D-Arabitol
5.1.3.4
2.7.1.16
L-Ribulose
L-Ribulose-5P
2,5-Dioxopentanoate
4.2.1.141
2-Dehydro-3-deoxy-D-xylonate
5.1.3.1
1.1.1.250
1.1.1.287
Pentose interconversion
1.2.1.26
1.2.1.-
4.1.2.28
Ribitol
1.1.1.56
2.7.1.16
1.1.1.137
D-Ribitol-5P
2.7.7.40
CDP-ribitol
D-Ribulose
2.7.1.47
1.1.1.405
2-Oxoglutarate
D-Ribulose-5P
Pyruvate
1.1.2.2
Citrate cycle
Riboflavin metabolism
Pentose phosphate pathway
00040 11/10/17
(c) Kanehisa Laboratories

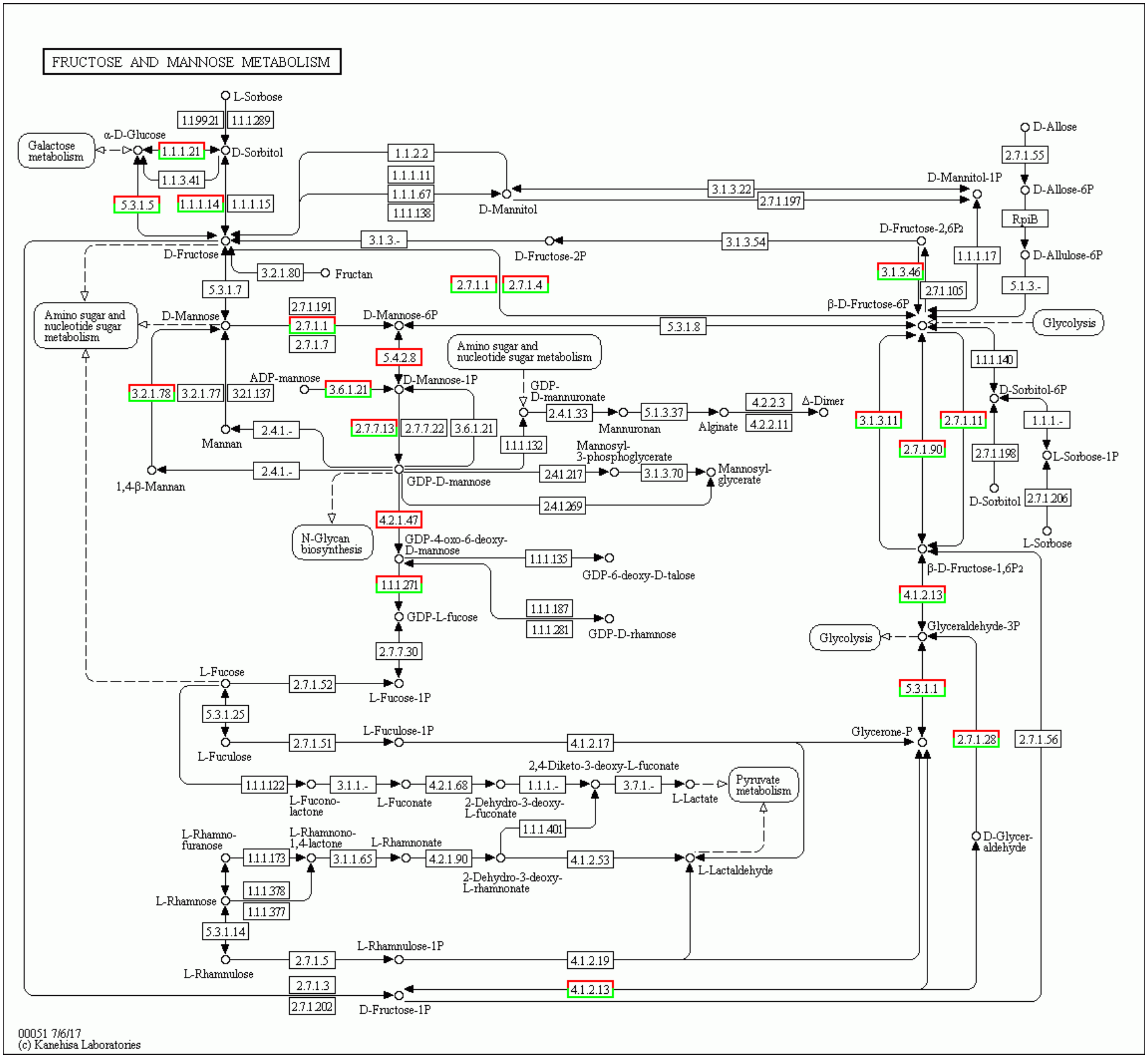
FRUCTOSE AND MANNOSE METABOLISM
L-Sorbose
1.1.99.21
1.1.1.289
α-D-Glucose
Galactose metabolism
1.1.1.21
D-Sorbitol
1.1.3.41
5.3.1.5
1.1.1.14
1.1.1.15
1.1.2.2
1.1.1.11
1.1.1.67
1.1.1.138
D-Mannitol
3.1.3.22
2.7.1.197
D-Mannitol-1P
D-Allose
2.7.1.55
D-Allose-6P
RpiB
D-Allulose-6P
5.1.3.-
D-Fructose
3.1.3.-
D-Fructose-2P
3.1.3.54
D-Fructose-2,6P2
3.1.3.46
2.7.1.105
1.1.1.17
3.2.1.80
Fructan
5.3.1.7
2.7.1.1
2.7.1.4
β-D-Fructose-6P
Glycolysis
Amino sugar and nucleotide sugar metabolism
D-Mannose
2.7.1.191
2.7.1.1
2.7.1.7
D-Mannose-6P
5.3.1.8
5.4.2.8
Amino sugar and nucleotide sugar metabolism
ADP-mannose
3.6.1.21
D-Mannose-1P
3.2.1.78
3.2.1.77
3.2.1.137
GDP-D-mannuronate
2.4.1.33
Mannuronan
5.1.3.37
Alginate
4.2.2.3
4.2.2.11
Δ-Dimer
3.1.3.11
2.7.1.11
1.1.1.140
D-Sorbitol-6P
1.1.1.-
2.4.1.-
Mannan
2.7.7.13
2.7.7.22
3.6.1.21
1.1.1.132
Mannosyl-3-phosphoglycerate
2.7.1.90
2.7.1.198
L-Sorbose-1P
1,4-β-Mannan
2.4.1.-
GDP-D-mannose
2.4.1.217
3.1.3.70
Mannosyl-glycerate
2.4.1.269
D-Sorbitol
2.7.1.206
L-Sorbose
4.2.1.47
N-Glycan biosynthesis
GDP-4-oxo-6-deoxy-D-mannose
1.1.1.135
GDP-6-deoxy-D-talose
β-D-Fructose-1,6P2
1.1.1.271
1.1.1.187
1.1.1.281
GDP-D-rhamnose
4.1.2.13
GDP-L-fucose
Glycolysis
Glyceraldehyde-3P
2.7.7.30
L-Fucose
2.7.1.52
L-Fucose-1P
5.3.1.1
5.3.1.25
L-Fuculose-1P
Glycerone-P
2.7.1.28
2.7.1.56
L-Fuculose
2.7.1.51
4.1.2.17
2,4-Diketo-3-deoxy-L-fuconate
1.1.1.122
L-Fucono-lactone
3.1.1.-
L-Fuconate
4.2.1.68
2-Dehydro-3-deoxy-L-fuconate
1.1.1.-
3.7.1.-
L-Lactate
Pyruvate metabolism
1.1.1.401
L-Rhamno-furanose
L-Rhamnono-1,4-lactone
L-Rhamnonate
1.1.1.173
3.1.1.65
4.2.1.90
2-Dehydro-3-deoxy-L-rhamnonate
4.1.2.53
L-Lactaldehyde
D-Glycer-aldehyde
1.1.1.378
L-Rhamnose
1.1.1.377
5.3.1.14
L-Rhamnulose-1P
L-Rhamnulose
2.7.1.5
4.1.2.19
2.7.1.3
2.7.1.202
D-Fructose-1P
4.1.2.13
00051 7/6/17
(c) Kanehisa Laboratories

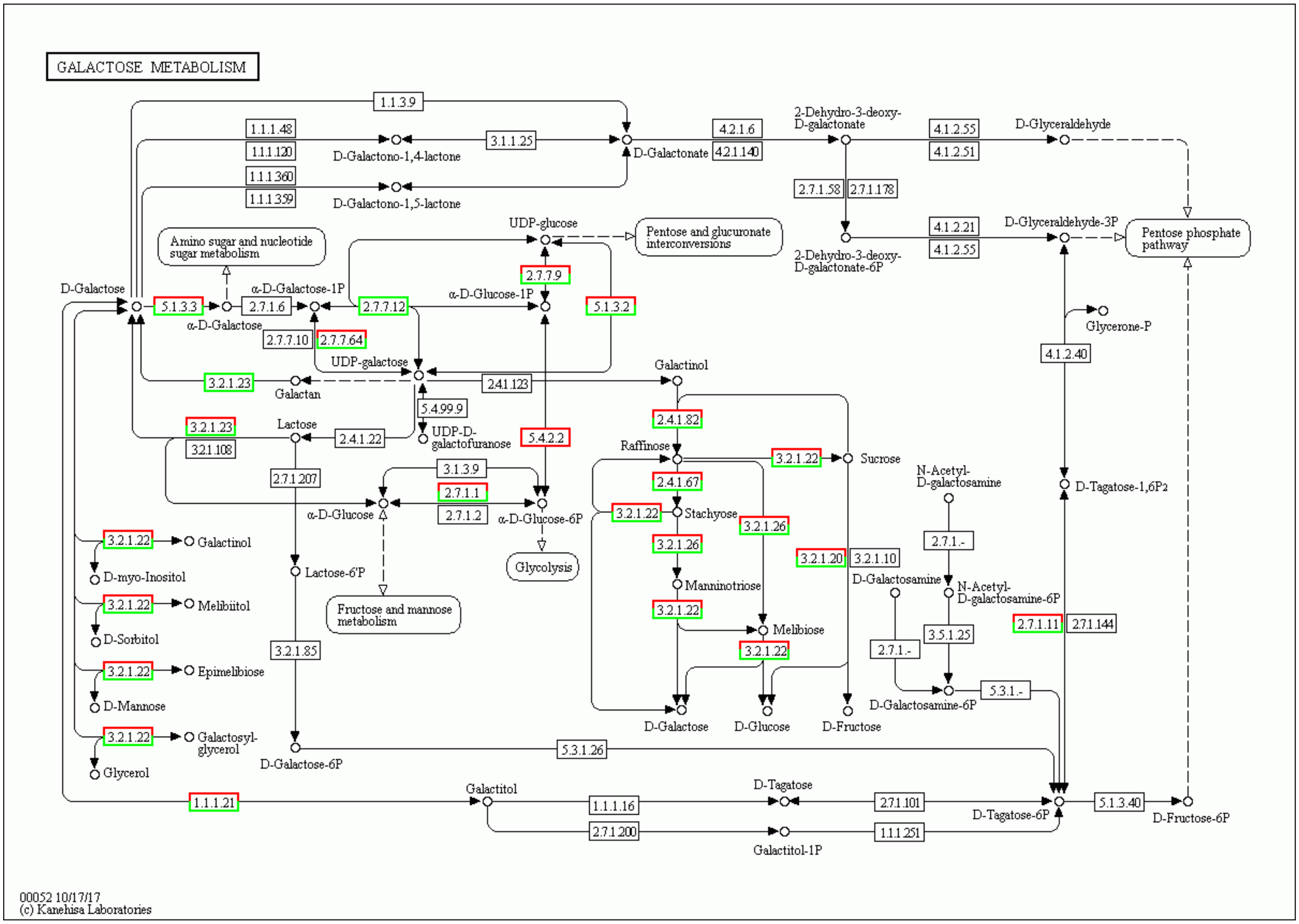
GALACTOSE METABOLISM
1.1.3.9
1.1.1.48
1.1.1.120
3.1.1.25
D-Galactono-1,4-lactone
1.1.1.360
1.1.1.359
D-Galactono-1,5-lactone
4.2.1.6
4.2.1.140
D-Galactonate
2-Dehydro-3-deoxy-
D-galactonate
4.1.2.55
4.1.2.51
D-Glyceraldehyde
2.7.1.58
2.7.1.178
4.1.2.21
4.1.2.55
D-Glyceraldehyde-3P
2-Dehydro-3-deoxy-
D-galactonate-6P
Pentose phosphate pathway
Amino sugar and nucleotide sugar metabolism
UDP-glucose
Pentose and glucuronate interconversions
2.7.7.9
D-Galactose
5.1.3.3
α-D-Galactose
α-D-Galactose-1P
2.7.1.6
2.7.7.12
α-D-Glucose-1P
5.1.3.2
2.7.7.10
2.7.7.64
UDP-galactose
Glycerone-P
4.1.2.40
3.2.1.23
Galactan
2.4.1.123
Galactinol
5.4.99.9
UDP-D-galactofuranose
5.4.2.2
2.4.1.82
3.2.1.23
3.2.1.108
Lactose
2.4.1.22
Raffinose
3.2.1.22
Sucrose
2.7.1.207
3.1.3.9
2.7.1.1
2.7.1.2
α-D-Glucose
α-D-Glucose-6P
2.4.1.67
3.2.1.22
Stachyose
3.2.1.26
N-Acetyl-
D-galactosamine
D-Tagatose-1,6P2
3.2.1.22
Galactinol
D-myo-Inositol
Lactose-6'P
Glycolysis
3.2.1.26
3.2.1.20
3.2.1.10
2.7.1.-
D-Galactosamine
N-Acetyl-
D-galactosamine-6P
3.2.1.22
Melibiitol
D-Sorbitol
Fructose and mannose metabolism
Manninotriose
3.2.1.22
Melibiose
2.7.1.11
2.7.1.144
3.5.1.25
3.2.1.85
3.2.1.22
2.7.1.-
3.2.1.22
Epimelibiose
D-Mannose
5.3.1.-
D-Galactosamine-6P
D-Galactose
D-Glucose
D-Fructose
3.2.1.22
Galactosyl-
glycerol
Glycerol
D-Galactose-6P
5.3.1.26
1.1.1.21
Galactitol
1.1.1.16
D-Tagatose
2.7.1.101
D-Tagatose-6P
5.1.3.40
D-Fructose-6P
2.7.1.200
Galactitol-1P
1.1.1.251
00052 10/17/17
(c) Kanehisa Laboratories

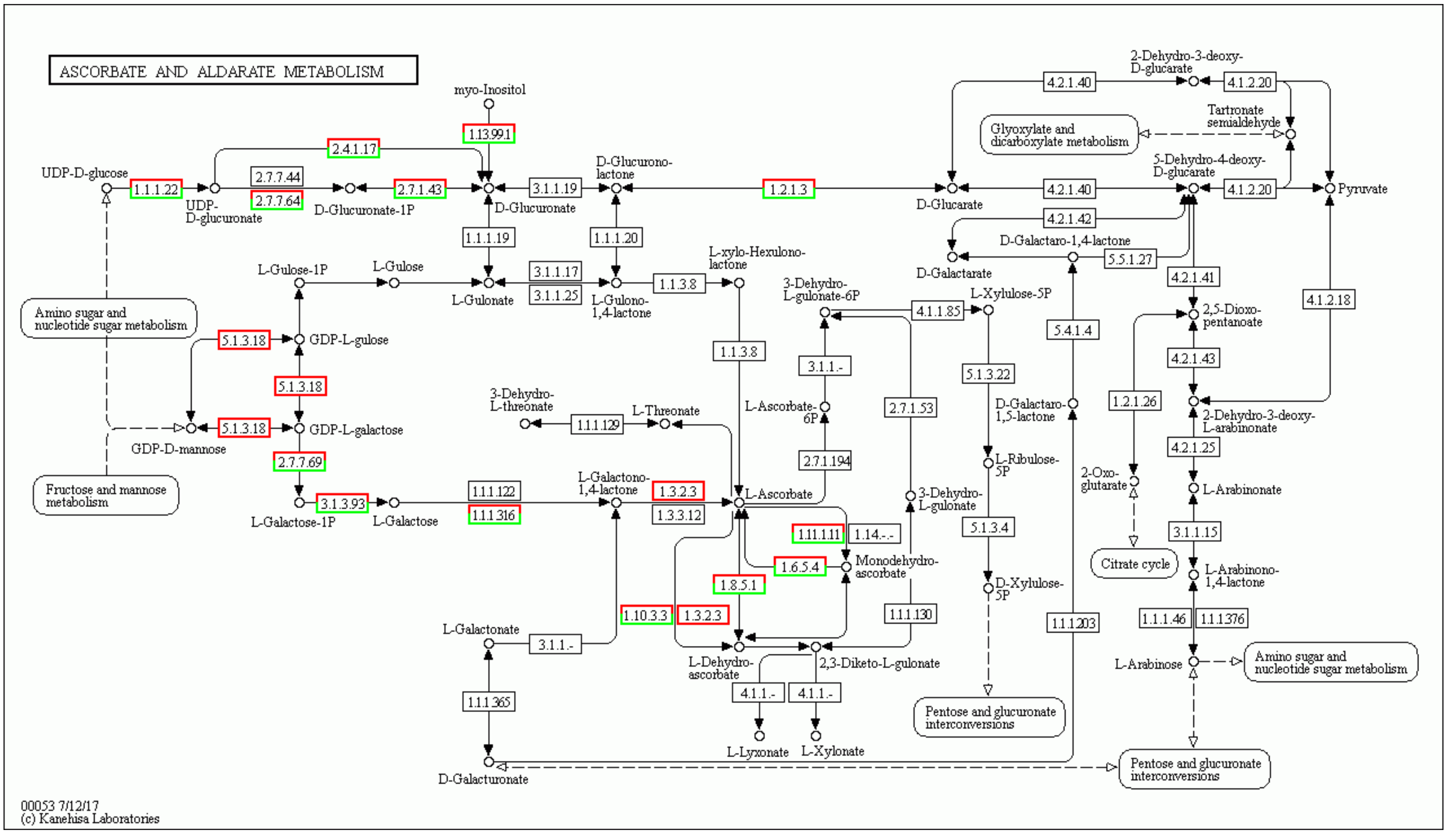
ASCORBATE AND ALDARATE METABOLISM
myo-Inositol
1.13.99.1
2.4.1.17
UDP-D-glucose
1.1.1.22
UDP-
D-glucuronate
2.7.7.44
2.7.7.64
D-Glucuronate-1P
2.7.1.43
D-Glucuronate
3.1.1.19
D-Glucurono-
lactone
1.2.1.3
D-Glucarate
4.2.1.40
2-Dehydro-3-deoxy-
D-glucarate
4.2.1.40
4.1.2.20
Tartronate
semialdehyde
Glyoxylate and dicarboxylate metabolism
5-Dehydro-4-deoxy-
D-glucarate
4.1.2.20
Pyruvate
4.2.1.42
D-Galactarate
D-Galactaro-1,4-lactone
5.5.1.27
1.1.1.19
1.1.1.20
L-Gulose-1P
L-Gulose
L-Gulonate
3.1.1.17
3.1.1.25
L-Gulono-
1,4-lactone
1.1.3.8
L-xylo-Hexulono-
lactone
3-Dehydro-
L-gulonate-6P
4.1.1.85
L-Xylulose-5P
4.2.1.41
2,5-Dioxo-
pentanoate
4.1.2.18
Amino sugar and nucleotide sugar metabolism
5.1.3.18
GDP-L-gulose
1.1.3.8
5.4.1.4
4.2.1.43
3.1.1.-
5.1.3.22
5.1.3.18
3-Dehydro-
L-threonate
L-Threonate
1.1.1.129
L-Ascorbate-
6P
2.7.1.53
D-Galactaro-
1,5-lactone
1.2.1.26
2-Dehydro-3-deoxy-
L-arabinonate
GDP-D-mannose
5.1.3.18
GDP-L-galactose
2.7.1.194
L-Ribulose-
5P
4.2.1.25
2.7.7.69
Fructose and mannose metabolism
L-Galactono-
1,4-lactone
1.3.2.3
L-Ascorbate
3-Dehydro-
L-gulonate
2-Oxo-
glutarate
L-Arabinonate
L-Galactose-1P
3.1.3.93
L-Galactose
1.1.1.122
1.1.1.316
1.3.3.12
1.11.1.11
1.14.-.-
5.1.3.4
3.1.1.15
1.6.5.4
Monodehydro-
ascorbate
Citrate cycle
L-Arabinono-
1,4-lactone
1.8.5.1
D-Xylulose-
5P
1.10.3.3
1.3.2.3
1.1.1.130
1.1.1.203
1.1.1.46
1.1.1.376
L-Galactonate
3.1.1.-
L-Dehydro-
ascorbate
2,3-Diketo-L-gulonate
L-Arabinose
Amino sugar and nucleotide sugar metabolism
1.1.1.365
4.1.1.-
4.1.1.-
Pentose and glucuronate interconversions
L-Lyxonate
L-Xylonate
D-Galacturonate
Pentose and glucuronate interconversions
00053 7/12/17
(c) Kanehisa Laboratories

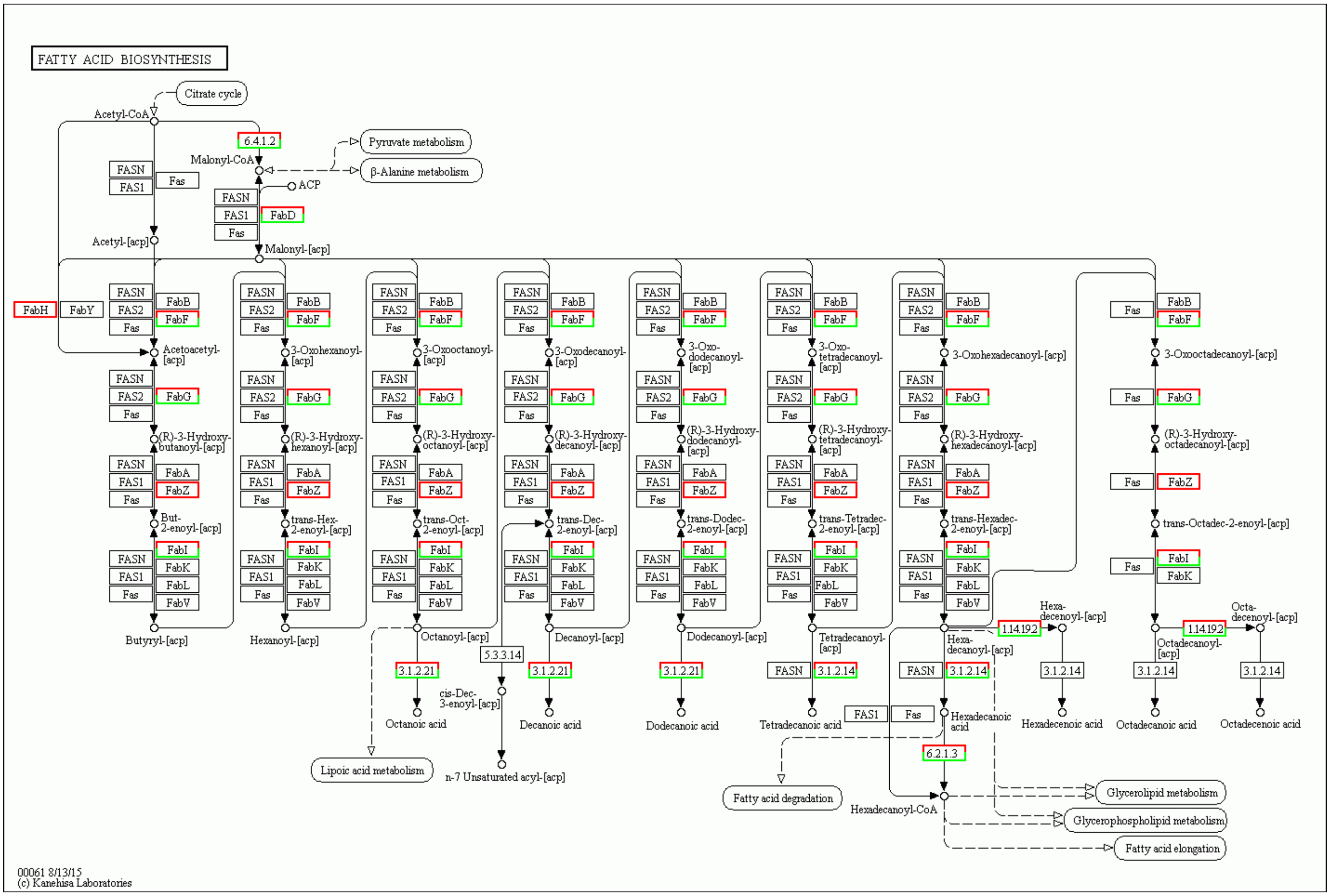
FATTY ACID BIOSYNTHESIS
Citrate cycle
Acetyl-CoA
6.4.1.2
Malonyl-CoA
Pyruvate metabolism
β-Alanine metabolism
ACP
FASN
FAS1
Fas
FabD
Acetyl-[acp]
Malonyl-[acp]
FabH
FabY
FAS2
FabB
FabF
FabG
FabA
FabZ
FabI
FabK
FabL
FabV
Acetoacetyl-[acp]
(R)-3-Hydroxy-butanoyl-[acp]
But-2-enoyl-[acp]
Butyryl-[acp]
3-Oxohexanoyl-[acp]
(R)-3-Hydroxy-hexanoyl-[acp]
trans-Hex-2-enoyl-[acp]
Hexanoyl-[acp]
3-Oxooctanoyl-[acp]
(R)-3-Hydroxy-octanoyl-[acp]
trans-Oct-2-enoyl-[acp]
Octanoyl-[acp]
3-Oxodecanoyl-[acp]
(R)-3-Hydroxy-decanoyl-[acp]
trans-Dec-2-enoyl-[acp]
Decanoyl-[acp]
3-Oxo-dodecanoyl-[acp]
(R)-3-Hydroxy-dodecanoyl-[acp]
trans-Dodec-2-enoyl-[acp]
Dodecanoyl-[acp]
3-Oxo-tetradecanoyl-[acp]
(R)-3-Hydroxy-tetradecanoyl-[acp]
trans-Tetradec-2-enoyl-[acp]
Tetradecanoyl-[acp]
3-Oxohexadecanoyl-[acp]
(R)-3-Hydroxy-hexadecanoyl-[acp]
trans-Hexadec-2-enoyl-[acp]
Hexa-decanoyl-[acp]
3-Oxooctadecanoyl-[acp]
(R)-3-Hydroxy-octadecanoyl-[acp]
trans-Octadec-2-enoyl-[acp]
Octadecanoyl-[acp]
Hexa-decenoyl-[acp]
Octa-decenoyl-[acp]
1.14.19.2
3.1.2.21
3.1.2.14
5.3.3.14
cis-Dec-3-enoyl-[acp]
n-7 Unsaturated acyl-[acp]
Lipoic acid metabolism
Octanoic acid
Decanoic acid
Dodecanoic acid
Tetradecanoic acid
Hexadecanoic acid
Hexadecenoic acid
Octadecanoic acid
Octadecenoic acid
6.2.1.3
Fatty acid degradation
Hexadecanoyl-CoA
Glycerolipid metabolism
Glycerophospholipid metabolism
Fatty acid elongation
00061 8/13/15
(c) Kanehisa Laboratories

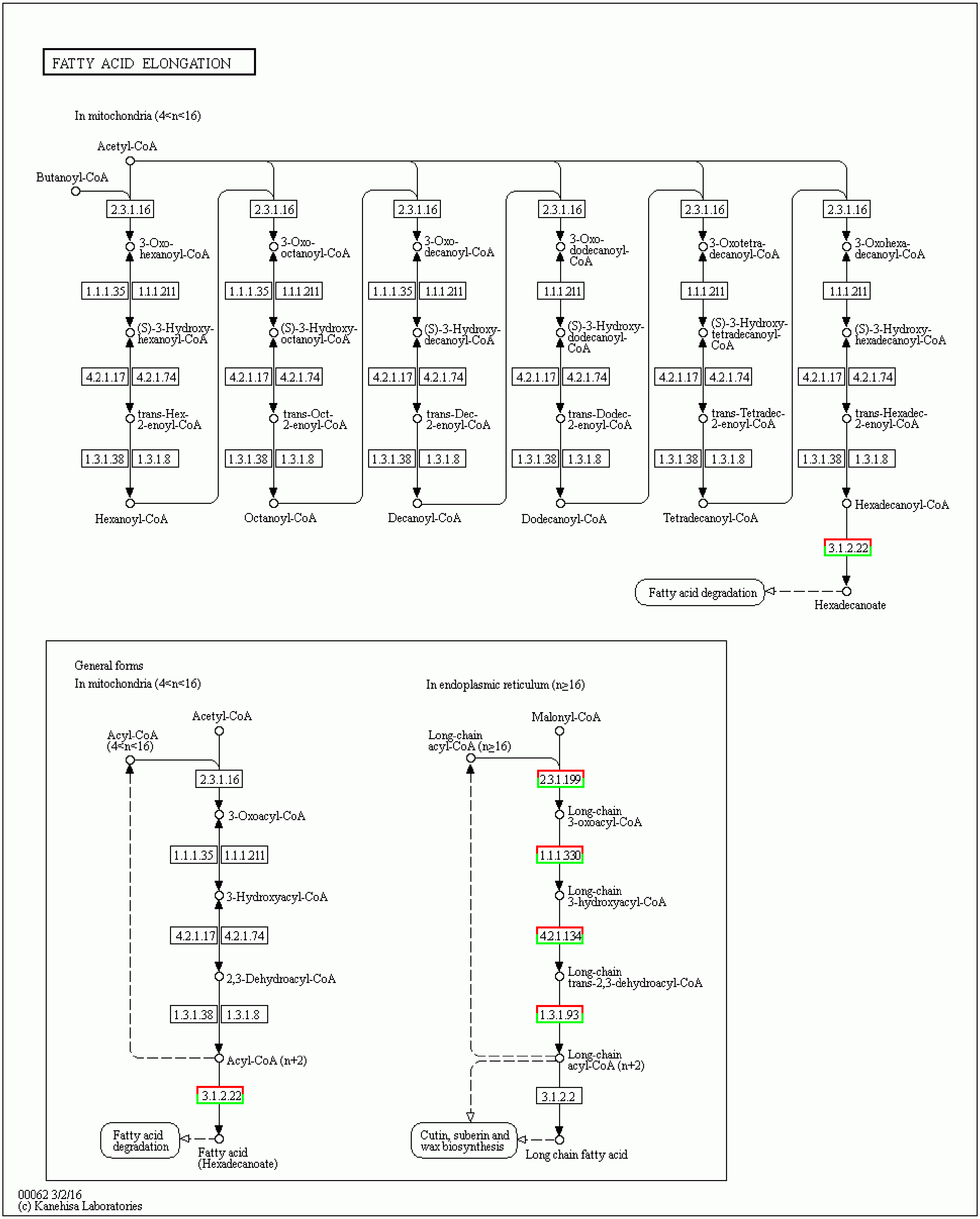
FATTY ACID ELONGATION
In mitochondria (4<n<16)
Acetyl-CoA
Butanoyl-CoA
2.3.1.16
3-Oxo-hexanoyl-CoA
1.1.1.35
1.1.1.211
(S)-3-Hydroxy-hexanoyl-CoA
4.2.1.17
4.2.1.74
trans-Hex-2-enoyl-CoA
1.3.1.38
1.3.1.8
Hexanoyl-CoA
3-Oxo-octanoyl-CoA
(S)-3-Hydroxy-octanoyl-CoA
trans-Oct-2-enoyl-CoA
Octanoyl-CoA
3-Oxo-decanoyl-CoA
(S)-3-Hydroxy-decanoyl-CoA
trans-Dec-2-enoyl-CoA
Decanoyl-CoA
3-Oxo-dodecanoyl-CoA
(S)-3-Hydroxy-dodecanoyl-CoA
trans-Dodec-2-enoyl-CoA
Dodecanoyl-CoA
3-Oxotetra-decanoyl-CoA
(S)-3-Hydroxy-tetradecanoyl-CoA
trans-Tetradec-2-enoyl-CoA
Tetradecanoyl-CoA
3-Oxohexa-decanoyl-CoA
(S)-3-Hydroxy-hexadecanoyl-CoA
trans-Hexadec-2-enoyl-CoA
Hexadecanoyl-CoA
3.1.2.22
Fatty acid degradation
Hexadecanoate
General forms
In mitochondria (4<n<16)
Acetyl-CoA
Acyl-CoA (4<n<16)
3-Oxoacyl-CoA
3-Hydroxyacyl-CoA
2,3-Dehydroacyl-CoA
Acyl-CoA (n+2)
Fatty acid degradation
Fatty acid (Hexadecanoate)
In endoplasmic reticulum (n≥16)
Malonyl-CoA
Long-chain acyl-CoA (n≥16)
2.3.1.199
Long-chain 3-oxoacyl-CoA
1.1.1.330
Long-chain 3-hydroxyacyl-CoA
4.2.1.134
Long-chain trans-2,3-dehydroacyl-CoA
1.3.1.93
Long-chain acyl-CoA (n+2)
3.1.2.2
Cutin, suberin and wax biosynthesis
Long chain fatty acid
00062 3/2/16
(c) Kanehisa Laboratories

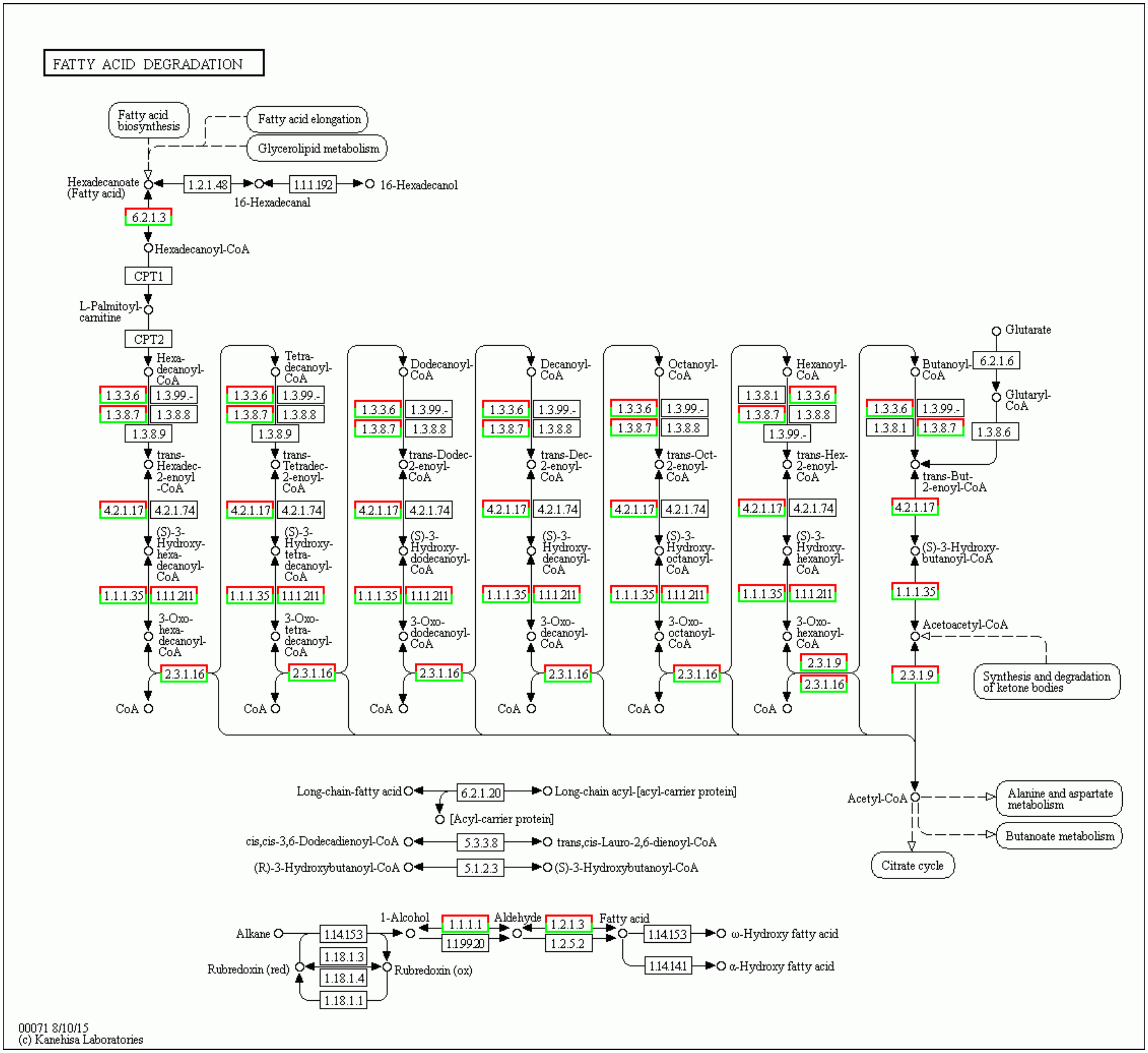
FATTY ACID DEGRADATION
Fatty acid biosynthesis
Fatty acid elongation
Glycerolipid metabolism
Hexadecanoate (Fatty acid)
1.2.1.48
1.1.1.192
16-Hexadecanol
16-Hexadecanal
6.2.1.3
Hexadecanoyl-CoA
CPT1
L-Palmitoyl-carnitine
CPT2
Hexa-decanoyl-CoA
Tetra-decanoyl-CoA
Dodecanoyl-CoA
Decanoyl-CoA
Octanoyl-CoA
Hexanoyl-CoA
Butanoyl-CoA
Glutarate
6.2.1.6
Glutaryl-CoA
1.3.8.6
1.3.3.6
1.3.99.-
1.3.8.7
1.3.8.8
1.3.8.9
1.3.8.1
trans-Hexadec-2-enoyl-CoA
trans-Tetradec-2-enoyl-CoA
trans-Dodec-2-enoyl-CoA
trans-Dec-2-enoyl-CoA
trans-Oct-2-enoyl-CoA
trans-Hex-2-enoyl-CoA
trans-But-2-enoyl-CoA
4.2.1.17
4.2.1.74
(S)-3-Hydroxy-hexa-decanoyl-CoA
(S)-3-Hydroxy-tetra-decanoyl-CoA
(S)-3-Hydroxy-dodecanoyl-CoA
(S)-3-Hydroxy-decanoyl-CoA
(S)-3-Hydroxy-octanoyl-CoA
(S)-3-Hydroxy-hexanoyl-CoA
(S)-3-Hydroxy-butanoyl-CoA
1.1.1.35
1.1.1.211
3-Oxo-hexa-decanoyl-CoA
3-Oxo-tetra-decanoyl-CoA
3-Oxo-dodecanoyl-CoA
3-Oxo-decanoyl-CoA
3-Oxo-octanoyl-CoA
3-Oxo-hexanoyl-CoA
Acetoacetyl-CoA
2.3.1.16
2.3.1.9
CoA
Synthesis and degradation of ketone bodies
Acetyl-CoA
Alanine and aspartate metabolism
Butanoate metabolism
Citrate cycle
Long-chain-fatty acid
6.2.1.20
Long-chain acyl-[acyl-carrier protein]
[Acyl-carrier protein]
cis,cis-3,6-Dodecadienoyl-CoA
5.3.3.8
trans,cis-Lauro-2,6-dienoyl-CoA
(R)-3-Hydroxybutanoyl-CoA
5.1.2.3
(S)-3-Hydroxybutanoyl-CoA
Alkane
1.14.15.3
1-Alcohol
1.1.1.1
1.1.99.20
Aldehyde
1.2.1.3
1.2.5.2
Fatty acid
ω-Hydroxy fatty acid
1.14.14.1
α-Hydroxy fatty acid
1.18.1.3
1.18.1.4
1.18.1.1
Rubredoxin (red)
Rubredoxin (ox)
00071 8/10/15
(c) Kanehisa Laboratories

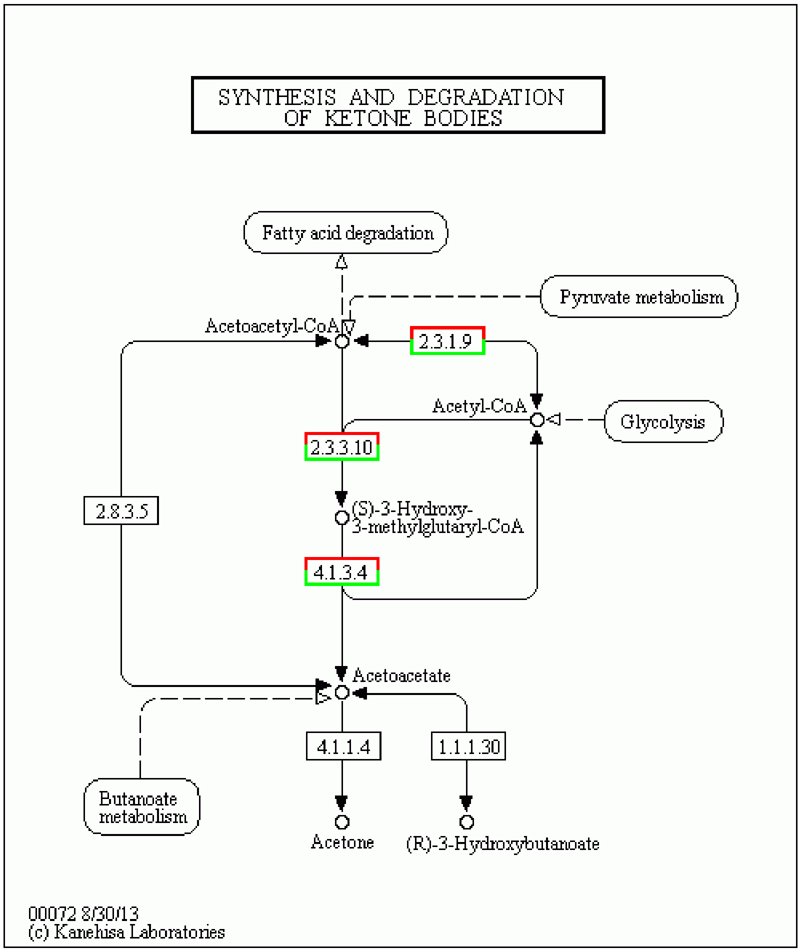
SYNTHESIS AND DEGRADATION OF KETONE BODIES
Fatty acid degradation
Pyruvate metabolism
Acetoacetyl-CoA
2.3.1.9
Acetyl-CoA
Glycolysis
2.3.3.10
2.8.3.5
(S)-3-Hydroxy-3-methylglutaryl-CoA
4.1.3.4
Acetoacetate
4.1.1.4
1.1.1.30
Butanoate metabolism
Acetone
(R)-3-Hydroxybutanoate
00072 8/30/13
(c) Kanehisa Laboratories

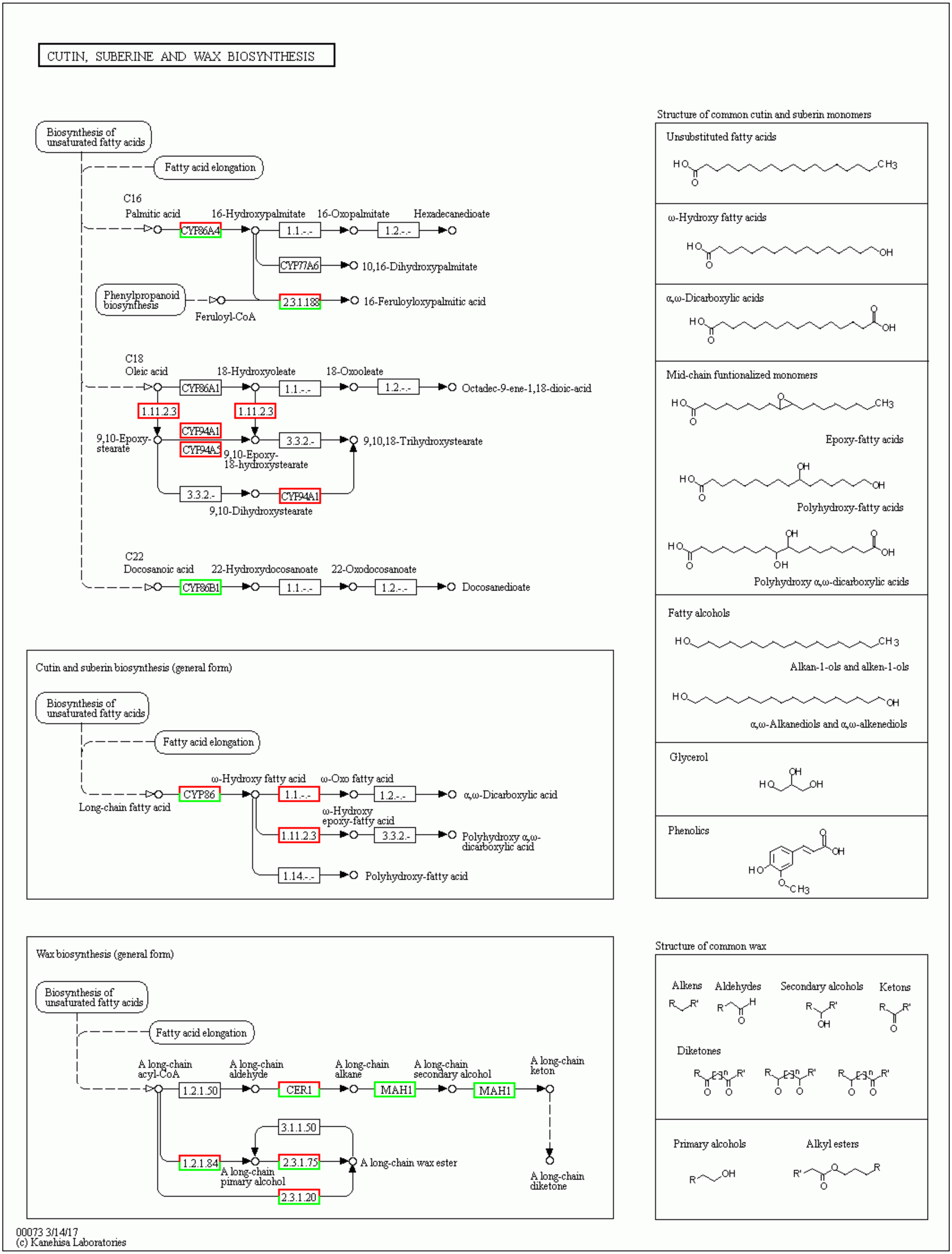
CUTIN, SUBERINE AND WAX BIOSYNTHESIS
Biosynthesis of unsaturated fatty acids
Fatty acid elongation
C16
Palmitic acid
16-Hydroxypalmitate
16-Oxopalmitate
Hexadecanedioate
CYP86A4
1.1.-.-
1.2.-.-
CYP77A6
10,16-Dihydroxypalmitate
Phenylpropanoid biosynthesis
Feruloyl-CoA
2.3.1.188
16-Feruloyloxypalmitic acid
C18
Oleic acid
18-Hydroxyoleate
18-Oxooleate
CYP86A1
Octadec-9-ene-1,18-dioic-acid
1.11.2.3
CYP94A1
CYP94A5
9,10-Epoxy-stearate
3.3.2.-
9,10,18-Trihydroxystearate
9,10-Epoxy-18-hydroxystearate
9,10-Dihydroxystearate
C22
Docosanoic acid
22-Hydroxydocosanoate
22-Oxodocosanoate
CYP86B1
Docosanedioate
Cutin and suberin biosynthesis (general form)
Long-chain fatty acid
CYP86
ω-Hydroxy fatty acid
ω-Oxo fatty acid
α,ω-Dicarboxylic acid
ω-Hydroxy epoxy-fatty acid
Polyhydroxy α,ω-dicarboxylic acid
1.14.-.-
Polyhydroxy-fatty acid
Wax biosynthesis (general form)
A long-chain acyl-CoA
A long-chain aldehyde
A long-chain alkane
A long-chain secondary alcohol
A long-chain keton
1.2.1.50
CER1
MAH1
3.1.1.50
1.2.1.84
2.3.1.75
A long-chain wax ester
A long-chain pimary alcohol
2.3.1.20
A long-chain diketone
Structure of common cutin and suberin monomers
Unsubstituted fatty acids
ω-Hydroxy fatty acids
α,ω-Dicarboxylic acids
Mid-chain funtionalized monomers
Epoxy-fatty acids
Polyhydroxy-fatty acids
Polyhydroxy α,ω-dicarboxylic acids
Fatty alcohols
Alkan-1-ols and alken-1-ols
α,ω-Alkanediols and α,ω-alkenediols
Glycerol
Phenolics
Structure of common wax
Alkens
Aldehydes
Secondary alcohols
Ketons
Diketones
Primary alcohols
Alkyl esters
00073 3/14/17
(c) Kanehisa Laboratories

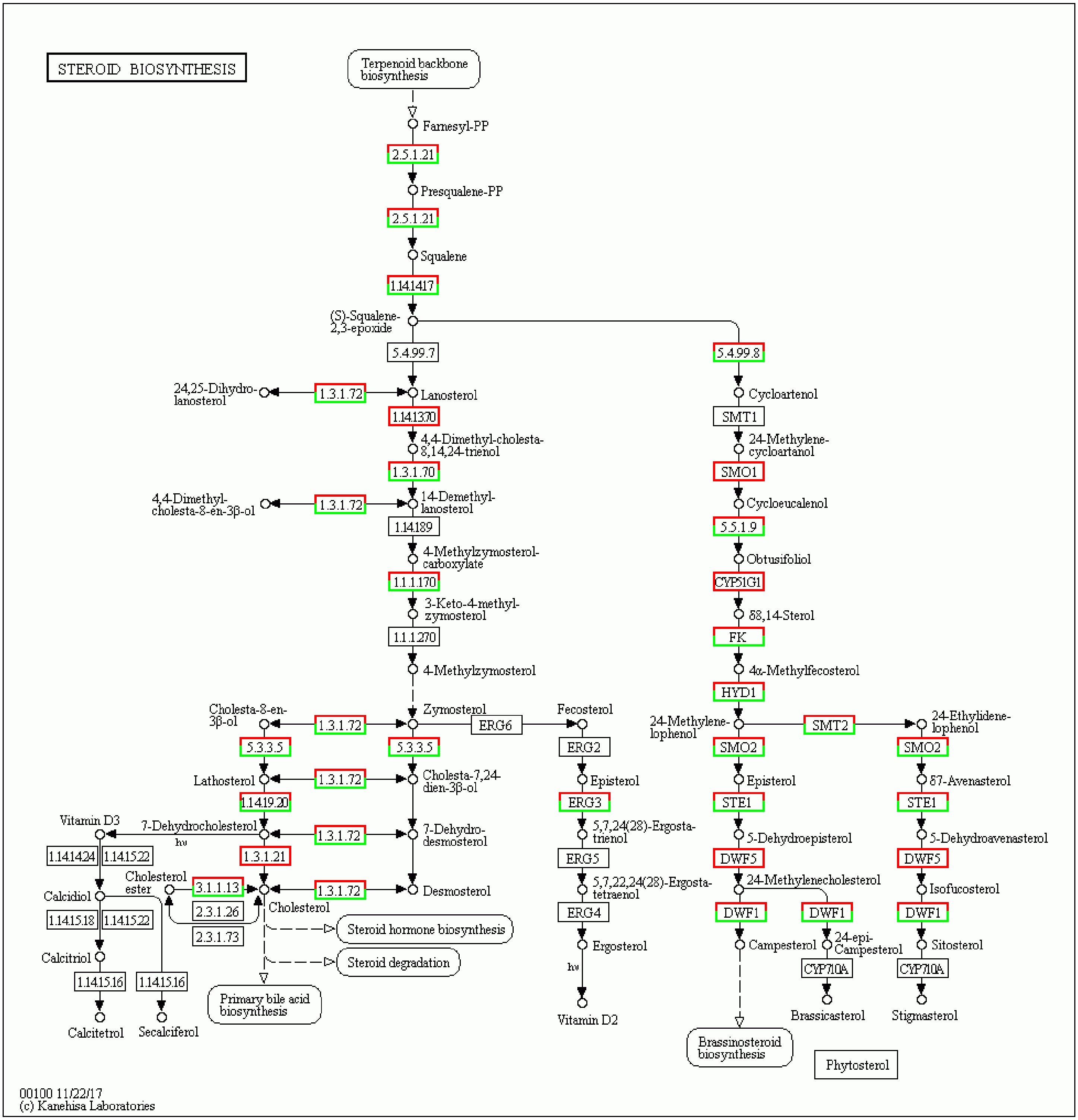
STEROID BIOSYNTHESIS
Terpenoid backbone biosynthesis
Farnesyl-PP
2.5.1.21
Presqualene-PP
2.5.1.21
Squalene
1.14.14.17
(S)-Squalene-2,3-epoxide
5.4.99.7
5.4.99.8
24,25-Dihydro-lanosterol
1.3.1.72
Lanosterol
Cycloartenol
1.14.13.70
SMT1
4,4-Dimethyl-cholesta-8,14,24-trienol
24-Methylene-cycloartanol
1.3.1.70
SMO1
4,4-Dimethyl-cholesta-8-en-3β-ol
1.3.1.72
14-Demethyl-lanosterol
Cycloeucalenol
1.14.18.9
5.5.1.9
4-Methylzymosterol-carboxylate
Obtusifoliol
1.1.1.170
CYP51G1
3-Keto-4-methyl-zymosterol
δ8,14-Sterol
1.1.1.270
FK
4-Methylzymosterol
4α-Methylfecosterol
HYD1
Cholesta-8-en-3β-ol
1.3.1.72
Zymosterol
ERG6
Fecosterol
24-Methylene-lophenol
SMT2
24-Ethylidene-lophenol
5.3.3.5
5.3.3.5
ERG2
SMO2
SMO2
Lathosterol
1.3.1.72
Cholesta-7,24-dien-3β-ol
Episterol
Episterol
δ7-Avenasterol
1.14.19.20
ERG3
STE1
STE1
Vitamin D3
7-Dehydrocholesterol
1.3.1.72
7-Dehydro-desmosterol
5,7,24(28)-Ergosta-trienol
5-Dehydroepisterol
5-Dehydroavenasterol
hν
1.14.14.24
1.14.15.22
1.3.1.21
ERG5
DWF5
DWF5
Cholesterol ester
Calcidiol
3.1.1.13
1.3.1.72
Desmosterol
5,7,22,24(28)-Ergosta-tetraenol
24-Methylenecholesterol
Isofucosterol
2.3.1.26
Cholesterol
ERG4
DWF1
DWF1
DWF1
1.14.15.18
1.14.15.22
2.3.1.73
Steroid hormone biosynthesis
Campesterol
24-epi-Campesterol
Sitosterol
Calcitriol
Ergosterol
Steroid degradation
CYP710A
CYP710A
1.14.15.16
1.14.15.16
hν
Primary bile acid biosynthesis
Calcitetrol
Secalciferol
Vitamin D2
Brassicasterol
Stigmasterol
Brassinosteroid biosynthesis
Phytosterol
00100 11/22/17
(c) Kanehisa Laboratories

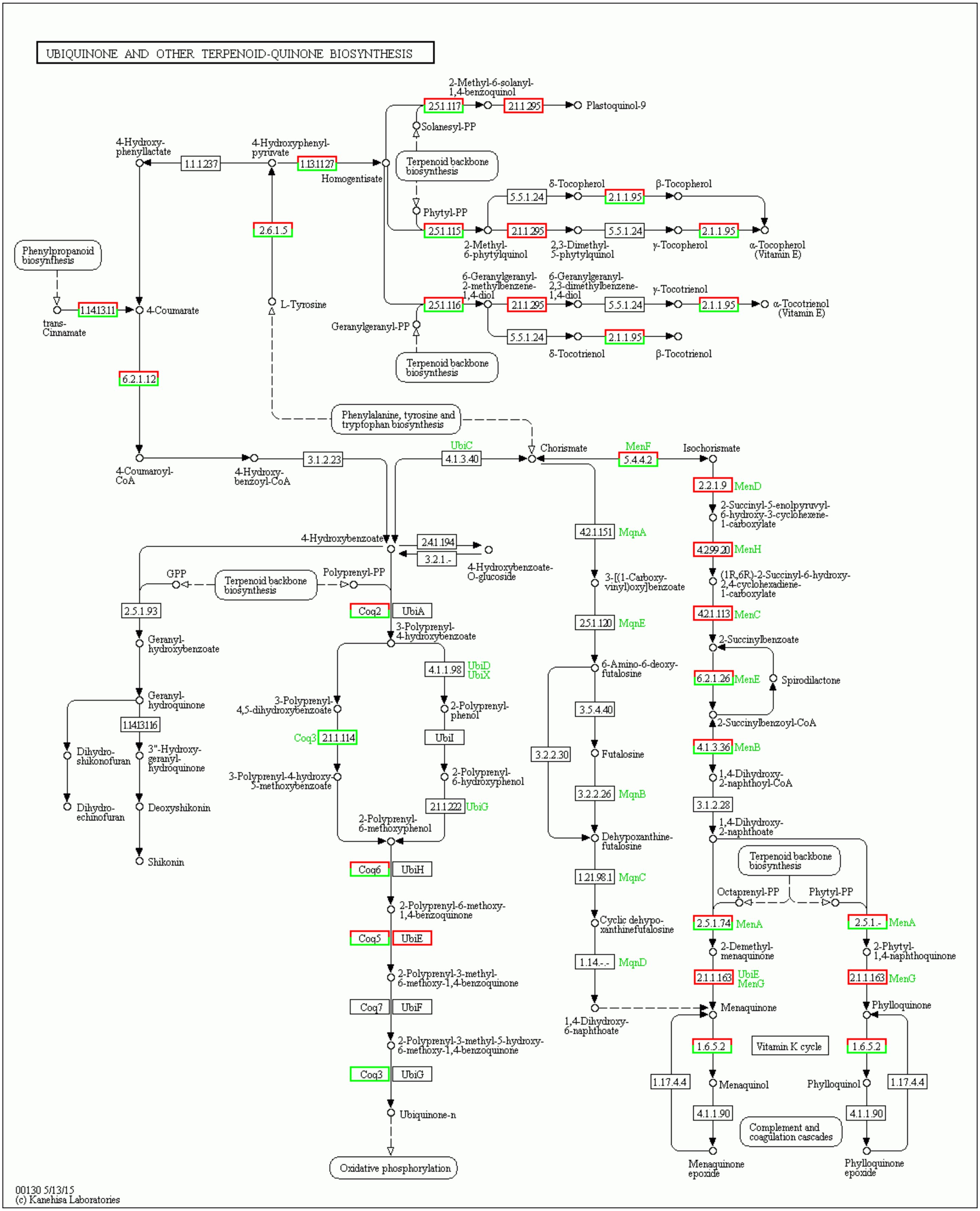
UBIQUINONE AND OTHER TERPENOID-QUINONE BIOSYNTHESIS
2-Methyl-6-solanyl-1,4-benzoquinol
2.5.1.117
2.1.1.295
Plastoquinol-9
Solanesyl-PP
4-Hydroxy-phenyllactate
1.1.1.237
4-Hydroxyphenyl-pyruvate
1.13.11.27
Homogentisate
Terpenoid backbone biosynthesis
δ-Tocopherol
β-Tocopherol
5.5.1.24
2.1.1.95
Phytyl-PP
2.6.1.5
2.5.1.115
2.1.1.295
5.5.1.24
2.1.1.95
2-Methyl-6-phytylquinol
2,3-Dimethyl-5-phytylquinol
γ-Tocopherol
α-Tocopherol (Vitamin E)
Phenylpropanoid biosynthesis
6-Geranylgeranyl-2-methylbenzene-1,4-diol
6-Geranylgeranyl-2,3-dimethylbenzene-1,4-diol
γ-Tocotrienol
trans-Cinnamate
1.14.13.11
4-Coumarate
L-Tyrosine
2.5.1.116
2.1.1.295
5.5.1.24
2.1.1.95
α-Tocotrienol (Vitamin E)
Geranylgeranyl-PP
5.5.1.24
2.1.1.95
δ-Tocotrienol
β-Tocotrienol
Terpenoid backbone biosynthesis
6.2.1.12
Phenylalanine, tyrosine and tryptophan biosynthesis
UbiC
MenF
Chorismate
Isochorismate
4.1.3.40
5.4.4.2
4-Coumaroyl-CoA
4-Hydroxy-benzoyl-CoA
3.1.2.23
2.2.1.9
MenD
2-Succinyl-5-enolpyruvyl-6-hydroxy-3-cyclohexene-1-carboxylate
4-Hydroxybenzoate
2.4.1.194
3.2.1.-
4-Hydroxybenzoate-O-glucoside
4.2.1.151
MqnA
4.2.99.20
MenH
GPP
Terpenoid backbone biosynthesis
Polyprenyl-PP
3-[(1-Carboxy-vinyl)oxy]benzoate
(1R,6R)-2-Succinyl-6-hydroxy-2,4-cyclohexadiene-1-carboxylate
2.5.1.93
Coq2
UbiA
4.2.1.113
MenC
Geranyl-hydroxybenzoate
3-Polyprenyl-4-hydroxybenzoate
2.5.1.120
MqnE
2-Succinylbenzoate
4.1.1.98
UbiD
UbiX
6-Amino-6-deoxy-futalosine
6.2.1.26
MenE
Spirodilactone
Geranyl-hydroquinone
3-Polyprenyl-4,5-dihydroxybenzoate
2-Polyprenyl-phenol
3.5.4.40
2-Succinylbenzoyl-CoA
1.14.13.116
Coq3
2.1.1.114
UbiI
3.2.2.30
Futalosine
4.1.3.36
MenB
Dihydro-shikonofuran
3"-Hydroxy-geranyl-hydroquinone
3-Polyprenyl-4-hydroxy-5-methoxybenzoate
2-Polyprenyl-6-hydroxyphenol
1,4-Dihydroxy-2-naphthoyl-CoA
3.2.2.26
MqnB
3.1.2.28
Dihydro-echinofuran
Deoxyshikonin
2.1.1.222
UbiG
1,4-Dihydroxy-2-naphthoate
2-Polyprenyl-6-methoxyphenol
Dehypoxanthine-futalosine
Terpenoid backbone biosynthesis
Shikonin
Coq6
UbiH
1.21.98.1
MqnC
Octaprenyl-PP
Phytyl-PP
2-Polyprenyl-6-methoxy-1,4-benzoquinone
Cyclic dehypo-xanthinefutalosine
2.5.1.74
MenA
2.5.1.-
MenA
Coq5
UbiE
2-Demethyl-menaquinone
2-Phytyl-1,4-naphthoquinone
2-Polyprenyl-3-methyl-6-methoxy-1,4-benzoquinone
1.14.-.-
MqnD
2.1.1.163
UbiE
MenG
2.1.1.163
MenG
Coq7
UbiF
Menaquinone
Phylloquinone
1,4-Dihydroxy-6-naphthoate
2-Polyprenyl-3-methyl-5-hydroxy-6-methoxy-1,4-benzoquinone
1.6.5.2
Vitamin K cycle
1.6.5.2
Coq3
UbiG
1.17.4.4
Menaquinol
Phylloquinol
1.17.4.4
Ubiquinone-n
4.1.1.90
4.1.1.90
Complement and coagulation cascades
Oxidative phosphorylation
Menaquinone epoxide
Phylloquinone epoxide
00130 5/13/15
(c) Kanehisa Laboratories

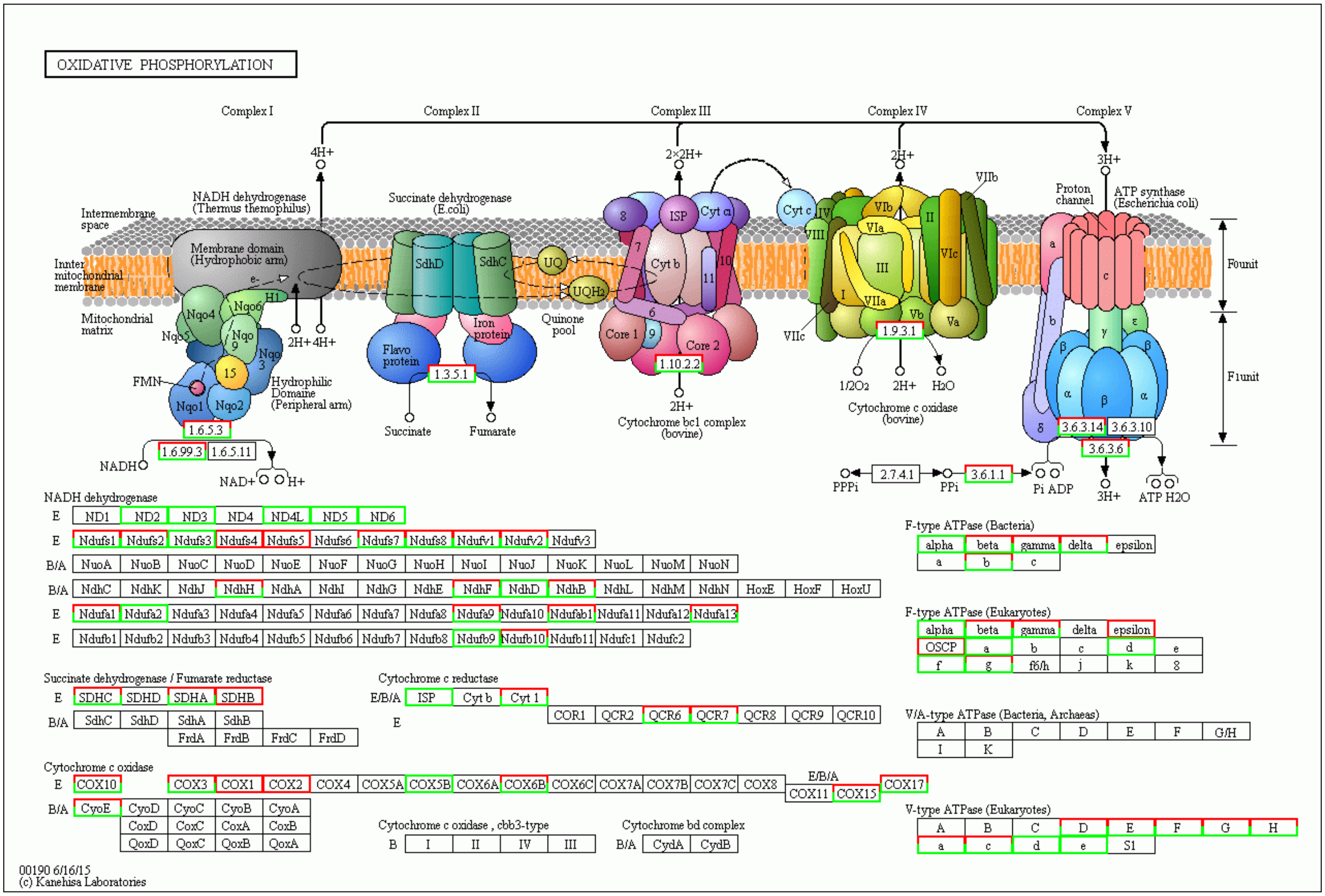
OXIDATIVE PHOSPHORYLATION
Complex I
Complex II
Complex III
Complex IV
Complex V
NADH dehydrogenase (Thermus themophilus)
Succinate dehydrogenase (E.coli)
ATP synthase (Escherichia coli)
Cytochrome bc1 complex (bovine)
Cytochrome c oxidase (bovine)
NADH dehydrogenase
ND1 ND2 ND3 ND4 ND4L ND5 ND6
Ndufs1 Ndufs2 Ndufs3 Ndufs4 Ndufs5 Ndufs6 Ndufs7 Ndufs8 Ndufv1 Ndufv2 Ndufv3
NuoA NuoB NuoC NuoD NuoE NuoF NuoG NuoH NuoI NuoJ NuoK NuoL NuoM NuoN
NdhC NdhK NdhJ NdhH NdhA NdhI NdhG NdhE NdhF NdhD NdhB NdhL NdhM NdhN HoxE HoxF HoxU
Ndufa1 Ndufa2 Ndufa3 Ndufa4 Ndufa5 Ndufa6 Ndufa7 Ndufa8 Ndufa9 Ndufa10 Ndufab1 Ndufa11 Ndufa12 Ndufa13
Ndufb1 Ndufb2 Ndufb3 Ndufb4 Ndufb5 Ndufb6 Ndufb7 Ndufb8 Ndufb9 Ndufb10 Ndufb11 Ndufc1 Ndufc2
Succinate dehydrogenase / Fumarate reductase
SDHC SDHD SDHA SDHB
SdhC SdhD SdhA SdhB FrdA FrdB FrdC FrdD
Cytochrome c reductase
ISP Cyt b Cyt 1
COR1 QCR2 QCR6 QCR7 QCR8 QCR9 QCR10
Cytochrome c oxidase
COX10 COX3 COX1 COX2 COX4 COX5A COX5B COX6A COX6B COX6C COX7A COX7B COX7C COX8 COX11 COX15 COX17
CyoE CyoD CyoC CyoB CyoA CoxD CoxC CoxA CoxB QoxD QoxC QoxB QoxA
Cytochrome c oxidase, cbb3-type
I II IV III
Cytochrome bd complex
CydA CydB
F-type ATPase (Bacteria)
alpha beta gamma delta epsilon a b c
F-type ATPase (Eukaryotes)
alpha beta gamma delta epsilon OSCP a b c d e f g f6/h j k 8
V/A-type ATPase (Bacteria, Archaeas)
A B C D E F G/H I K
V-type ATPase (Eukaryotes)
A B C D E F G H a c d e S1
1.6.5.3 1.6.99.3 1.6.5.11 1.3.5.1 1.10.2.2 1.9.3.1 3.6.3.14 3.6.3.10 3.6.3.6 2.7.4.1 3.6.1.1
00190 6/16/15
(c) Kanehisa Laboratories

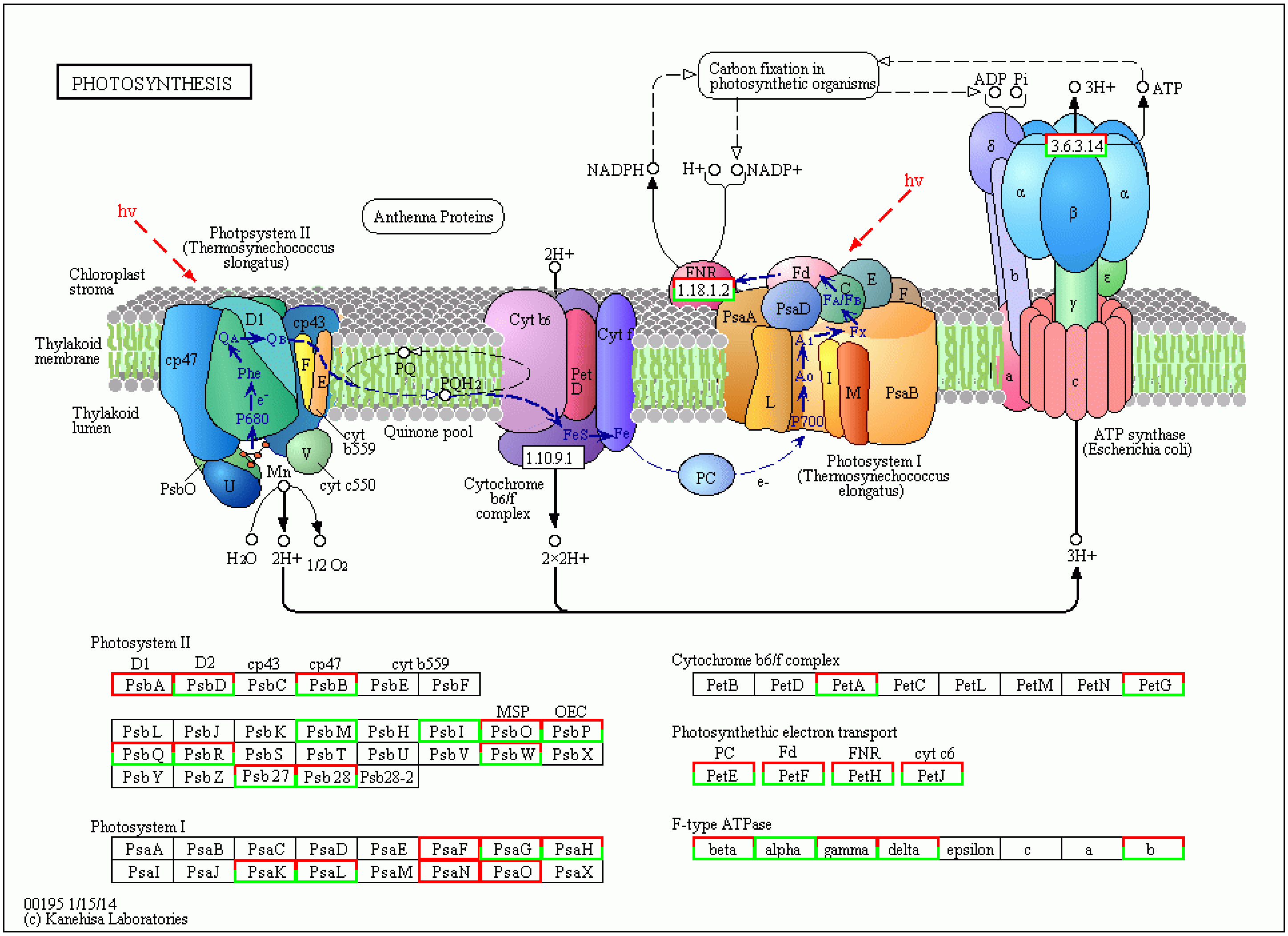
PHOTOSYNTHESIS
Carbon fixation in photosynthetic organisms
Anthenna Proteins
NADPH
H+
NADP+
ADP Pi
3H+
ATP
3.6.3.14
hv
Photpsystem II (Thermosynechococcus slongatus)
Chloroplast stroma
Thylakoid membrane
Thylakoid lumen
D1
cp43
cp47
QA
QB
Phe
P680
F
E
V
U
PsbO
Mn
H2O
2H+
1/2 O2
cyt b559
cyt c550
PQ
PQH2
Quinone pool
2H+
Cyt b6
Pet D
Cyt f
FeS
Fe
1.10.9.1
Cytochrome b6/f complex
2×2H+
PC
e-
FNR
1.18.1.2
Fd
PsaA
PsaD
C
E
F
FA/FB
Fx
A1
A0
P700
L
I
M
PsaB
Photosystem I (Thermosynechococcus elongatus)
δ
α
β
b
ε
γ
a
c
ATP synthase (Escherichia coli)
3H+
Photosystem II
D1 D2 cp43 cp47 cyt b559
PsbA PsbD PsbC PsbB PsbE PsbF
MSP OEC
PsbL PsbJ PsbK PsbM PsbH PsbI PsbO PsbP
PsbQ PsbR PsbS PsbT PsbU PsbV PsbW PsbX
PsbY PsbZ Psb27 Psb28 Psb28-2
Photosystem I
PsaA PsaB PsaC PsaD PsaE PsaF PsaG PsaH
PsaI PsaJ PsaK PsaL PsaM PsaN PsaO PsaX
Cytochrome b6/f complex
PetB PetD PetA PetC PetL PetM PetN PetG
Photosynthethic electron transport
PC Fd FNR cyt c6
PetE PetF PetH PetJ
F-type ATPase
beta alpha gamma delta epsilon c a b
00195 1/15/14
(c) Kanehisa Laboratories

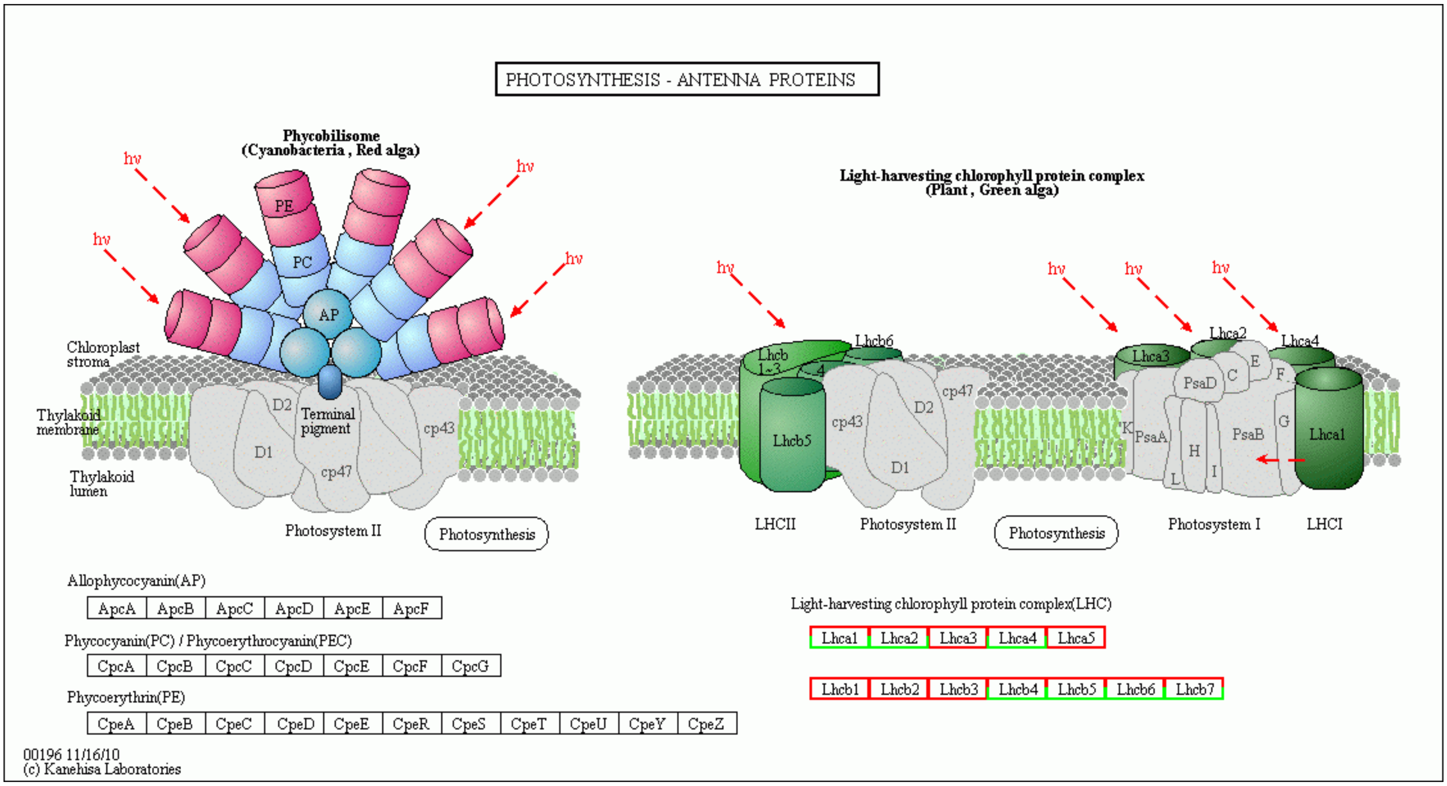
PHOTOSYNTHESIS - ANTENNA PROTEINS
Phycobilisome (Cyanobacteria , Red alga)
hv
PE
PC
AP
Chloroplast stroma
Thylakoid membrane
Thylakoid lumen
D2
D1
Terminal pigment
cp43
cp47
Photosystem II
Photosynthesis
Light-harvesting chlorophyll protein complex (Plant , Green alga)
Lhcb6
Lhcb 1~3
Lhcb5
cp43
cp47
D2
D1
LHCII
Photosystem II
Photosynthesis
Lhca3
Lhca2
Lhca4
PsaD
C
E
F
K
PsaA
H
L
I
PsaB
G
Lhca1
Photosystem I
LHCI
Allophycocyanin(AP)
ApcA ApcB ApcC ApcD ApcE ApcF
Phycocyanin(PC) / Phycoerythrocyanin(PEC)
CpcA CpcB CpcC CpcD CpcE CpcF CpcG
Phycoerythrin(PE)
CpeA CpeB CpeC CpeD CpeE CpeR CpeS CpeT CpeU CpeY CpeZ
Light-harvesting chlorophyll protein complex(LHC)
Lhca1 Lhca2 Lhca3 Lhca4 Lhca5
Lhcb1 Lhcb2 Lhcb3 Lhcb4 Lhcb5 Lhcb6 Lhcb7
00196 11/16/10
(c) Kanehisa Laboratories

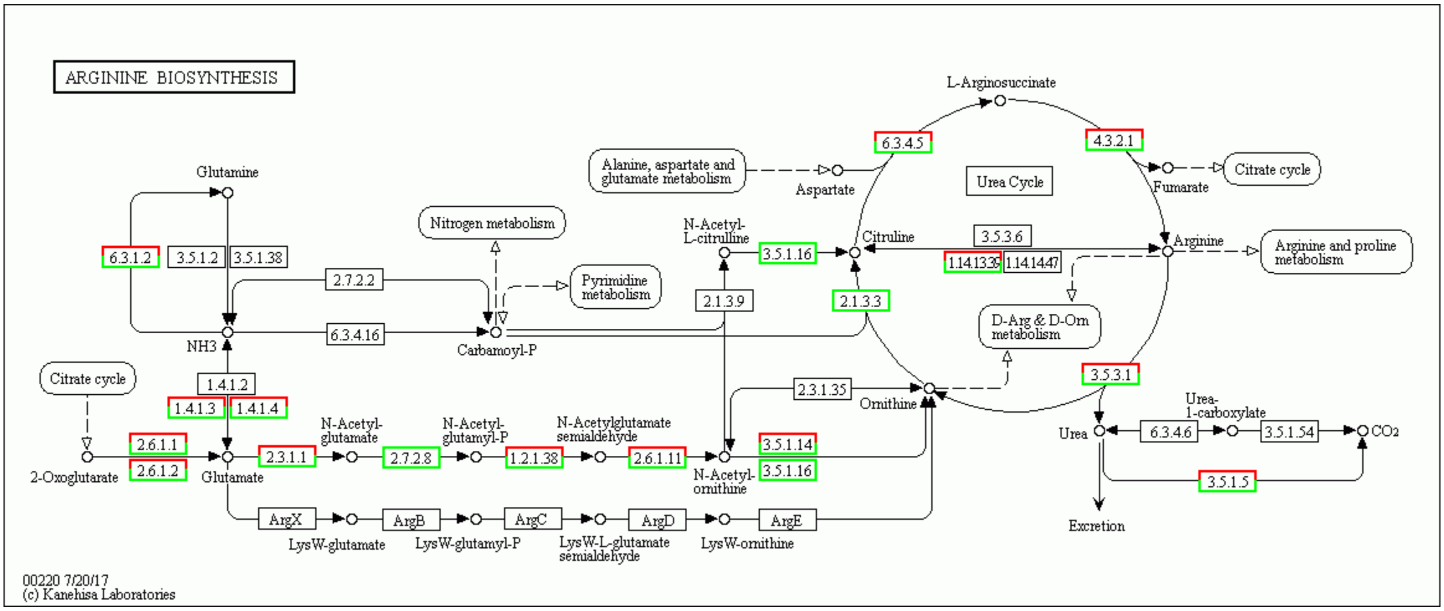
ARGININE BIOSYNTHESIS
L-Arginosuccinate
6.3.4.5
4.3.2.1
Alanine, aspartate and glutamate metabolism
Aspartate
Urea Cycle
Citrate cycle
Fumarate
Glutamine
Nitrogen metabolism
N-Acetyl-L-citrulline
Citruline
3.5.3.6
Arginine
Arginine and proline metabolism
6.3.1.2
3.5.1.2
3.5.1.38
3.5.1.16
1.14.13.39
1.14.14.47
2.7.2.2
Pyrimidine metabolism
2.1.3.9
2.1.3.3
D-Arg & D-Orn metabolism
NH3
6.3.4.16
Carbamoyl-P
Citrate cycle
1.4.1.2
1.4.1.3
1.4.1.4
2.3.1.35
Ornithine
3.5.3.1
Urea-1-carboxylate
N-Acetyl-glutamate
N-Acetyl-glutamyl-P
N-Acetylglutamate semialdehyde
3.5.1.14
Urea
6.3.4.6
3.5.1.54
CO2
2.6.1.1
2.3.1.1
2.7.2.8
1.2.1.38
2.6.1.11
N-Acetyl-ornithine
3.5.1.16
2.6.1.2
2-Oxoglutarate
Glutamate
3.5.1.5
ArgX
ArgB
ArgC
ArgD
ArgE
Excretion
LysW-glutamate
LysW-glutamyl-P
LysW-L-glutamate semialdehyde
LysW-ornithine
00220 7/20/17
(c) Kanehisa Laboratories

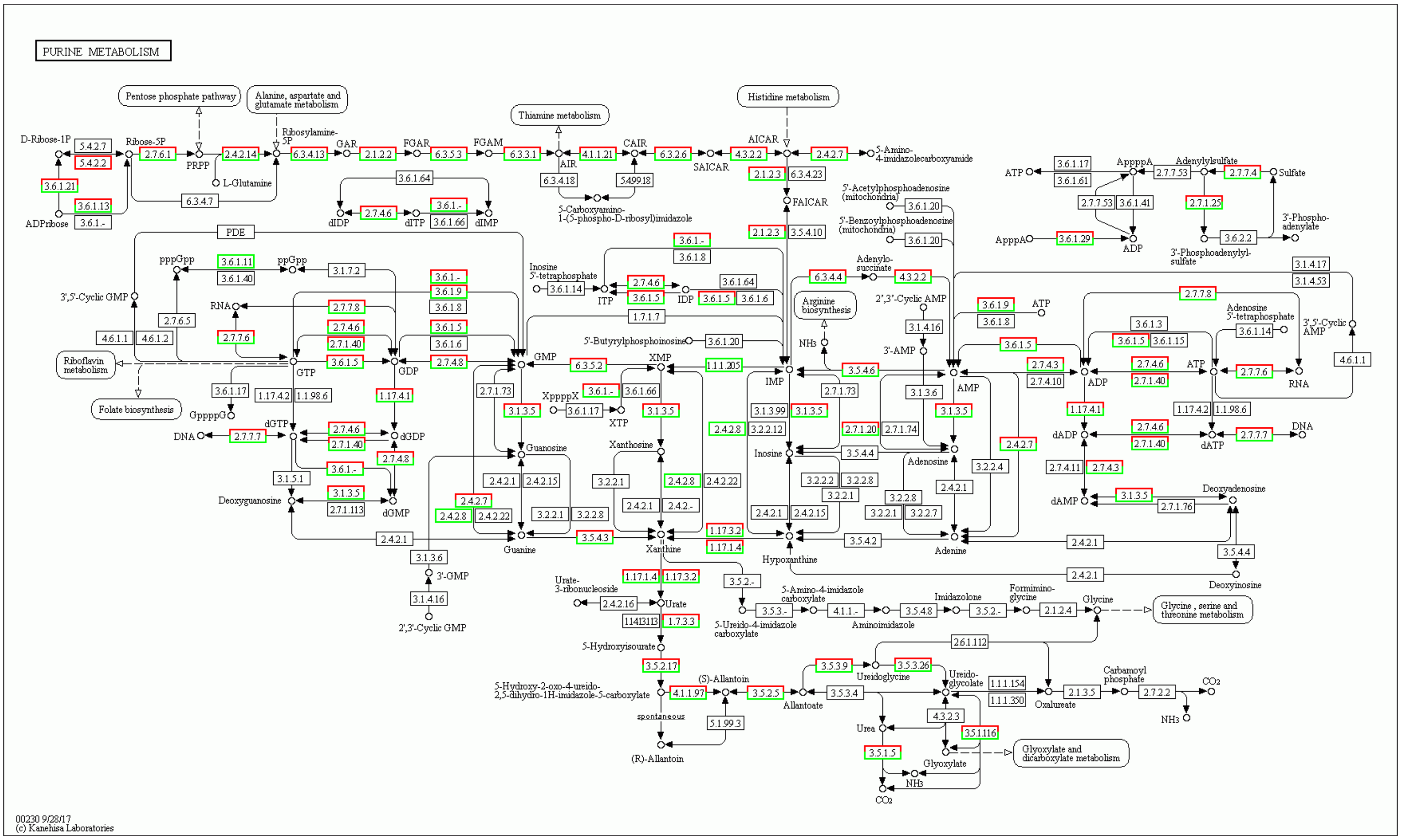
PURINE METABOLISM
Pentose phosphate pathway
Alanine, aspartate and glutamate metabolism
Thiamine metabolism
Histidine metabolism
D-Ribose-1P
Ribose-5P
Ribosylamine-5P
GAR
FGAR
FGAM
CAIR
AICAR
5-Amino-4-imidazolecarboxyamide
PRPP
L-Glutamine
AIR
SAICAR
FAICAR
ADPribose
5-Carboxyamino-1-(5-phospho-D-ribosyl)imidazole
dIDP
dITP
dIMP
ATP
ApppA
Adenylylsulfate
Sulfate
3'-Phospho-adenylylsulfate
3'-Phospho-adenylate
PDE
ppGpp
pppGpp
3',5'-Cyclic GMP
RNA
Riboflavin metabolism
Folate biosynthesis
GTP
GDP
GMP
GppppG
dGTP
DNA
dGDP
dGMP
Deoxyguanosine
Guanosine
Guanine
Inosine 5'-tetraphosphate
ITP
IDP
IMP
Inosine
Hypoxanthine
XMP
XTP
XppppX
Xanthosine
Xanthine
5'-Butyrylphosphoinosine
5'-Acetylphosphoadenosine (mitochondria)
5'-Benzoylphosphoadenosine (mitochondria)
Adenylo-succinate
Arginine biosynthesis
2',3'-Cyclic AMP
3'-AMP
NH3
AMP
Adenosine
Adenine
ADP
dADP
dAMP
dATP
Adenosine 5'-tetraphosphate
3',5'-Cyclic AMP
Deoxyadenosine
Deoxyinosine
3'-GMP
2',3'-Cyclic GMP
Urate-3-ribonucleoside
Urate
5-Hydroxyisourate
5-Amino-4-imidazole carboxylate
5-Ureido-4-imidazole carboxylate
Imidazolone
Aminoimidazole
Formimino-glycine
Glycine
Glycine, serine and threonine metabolism
5-Hydroxy-2-oxo-4-ureido-2,5-dihydro-1H-imidazole-5-carboxylate
(S)-Allantoin
Allantoate
Ureidoglycine
Ureido-glycolate
Oxalureate
Carbamoyl phosphate
CO2
NH3
Urea
Glyoxylate
Glyoxylate and dicarboxylate metabolism
spontaneous
(R)-Allantoin
CO2
00230 9/28/17
(c) Kanehisa Laboratories

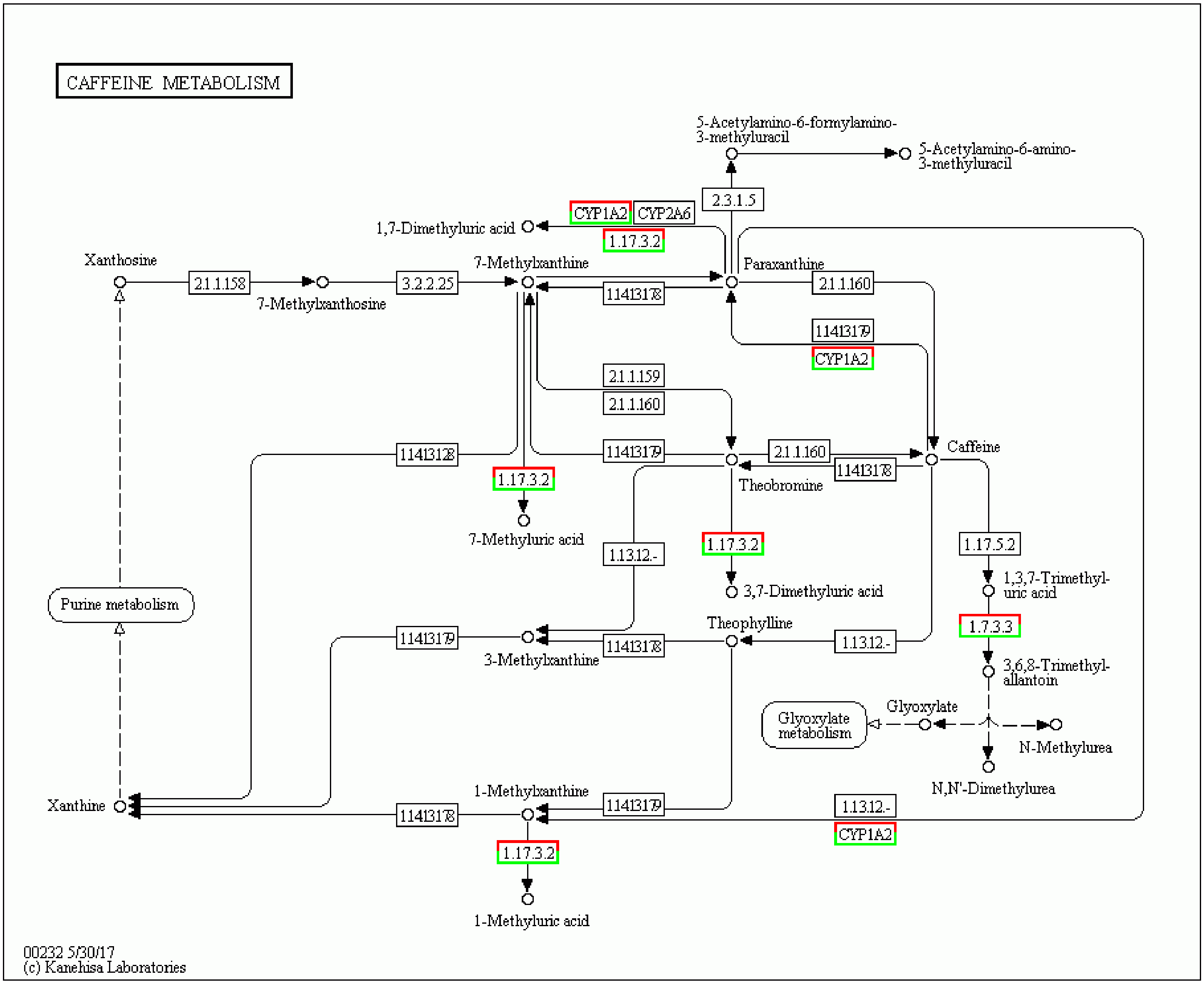
CAFFEINE METABOLISM
5-Acetylamino-6-formylamino-3-methyluracil
5-Acetylamino-6-amino-3-methyluracil
2.3.1.5
CYP1A2
CYP2A6
1,7-Dimethyluric acid
1.17.3.2
Xanthosine
2.1.1.158
7-Methylxanthosine
3.2.2.25
7-Methylxanthine
1.14.13.178
Paraxanthine
2.1.1.160
1.14.13.179
CYP1A2
2.1.1.159
2.1.1.160
1.14.13.128
1.14.13.179
2.1.1.160
Caffeine
1.14.13.178
Theobromine
1.17.3.2
7-Methyluric acid
1.13.12.-
1.17.3.2
3,7-Dimethyluric acid
1.17.5.2
1,3,7-Trimethyluric acid
Purine metabolism
Theophylline
1.7.3.3
1.14.13.179
3-Methylxanthine
1.14.13.178
1.13.12.-
3,6,8-Trimethylallantoin
Glyoxylate
Glyoxylate metabolism
N-Methylurea
N,N'-Dimethylurea
1-Methylxanthine
Xanthine
1.14.13.178
1.14.13.179
1.13.12.-
CYP1A2
1.17.3.2
1-Methyluric acid
00232 5/30/17
(c) Kanehisa Laboratories

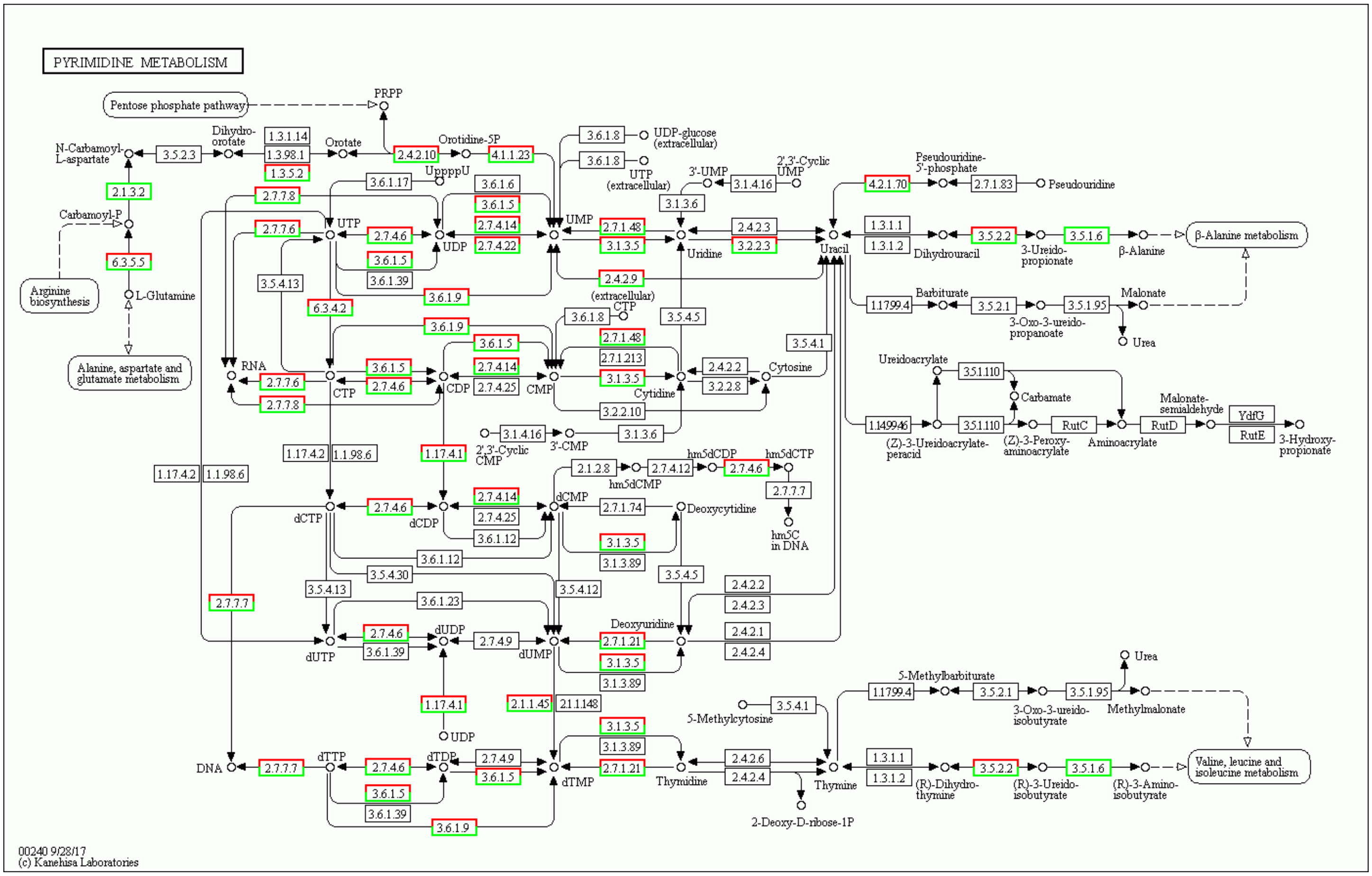
PYRIMIDINE METABOLISM
Pentose phosphate pathway
PRPP
N-Carbamoyl-L-aspartate
3.5.2.3
Dihydro-orotate
1.3.1.14
1.3.98.1
1.3.5.2
Orotate
2.4.2.10
Orotidine-5P
4.1.1.23
2.1.3.2
Carbamoyl-P
6.3.5.5
L-Glutamine
Arginine biosynthesis
Alanine, aspartate and glutamate metabolism
3.6.1.17
UppppU
2.7.7.8
2.7.7.6
UTP
2.7.4.6
UDP
3.6.1.6
3.6.1.5
2.7.4.14
2.7.4.22
3.6.1.39
3.6.1.9
3.5.4.13
6.3.4.2
UMP
3.6.1.8
UDP-glucose (extracellular)
UTP (extracellular)
2.7.1.48
3.1.3.5
2.4.2.9
(extracellular)
3'-UMP
3.1.4.16
2',3'-Cyclic UMP
3.1.3.6
2.4.2.3
3.2.2.3
Uridine
Uracil
4.2.1.70
Pseudouridine-5'-phosphate
2.7.1.83
Pseudouridine
1.3.1.1
1.3.1.2
Dihydrouracil
3.5.2.2
3-Ureido-propionate
3.5.1.6
β-Alanine
β-Alanine metabolism
1.1.99.4
Barbiturate
3.5.2.1
3-Oxo-3-ureido-propanoate
3.5.1.95
Malonate
Urea
Ureidoacrylate
3.5.1.110
Carbamate
1.14.99.46
(Z)-3-Ureidoacrylate-peracid
(Z)-3-Peroxy-aminoacrylate
RutC
Aminoacrylate
RutD
Malonate-semialdehyde
YdfG
RutE
3-Hydroxy-propionate
RNA
CTP
CDP
2.7.4.25
CMP
2.7.1.213
3.5.4.5
3.5.4.1
Cytosine
2.4.2.2
3.2.2.8
Cytidine
3.2.2.10
3.1.4.16
2',3'-Cyclic CMP
3'-CMP
3.1.3.6
1.17.4.2
1.1.98.6
1.17.4.1
2.1.2.8
hm5dCMP
2.7.4.12
hm5dCDP
hm5dCTP
2.7.7.7
hm5C in DNA
dCTP
dCDP
dCMP
3.6.1.12
2.7.1.74
Deoxycytidine
3.1.3.89
3.5.4.30
3.5.4.13
3.6.1.23
3.5.4.12
dUTP
dUDP
2.7.4.9
dUMP
Deoxyuridine
2.7.1.21
2.4.2.1
2.4.2.4
2.1.1.45
2.1.1.148
UDP
5-Methylcytosine
5-Methylbarbiturate
3-Oxo-3-ureido-isobutyrate
Methylmalonate
DNA
dTTP
dTDP
dTMP
Thymidine
2.4.2.6
Thymine
2-Deoxy-D-ribose-1P
(R)-Dihydro-thymine
(R)-3-Ureido-isobutyrate
(R)-3-Amino-isobutyrate
Valine, leucine and isoleucine metabolism
00240 9/28/17
(c) Kanehisa Laboratories

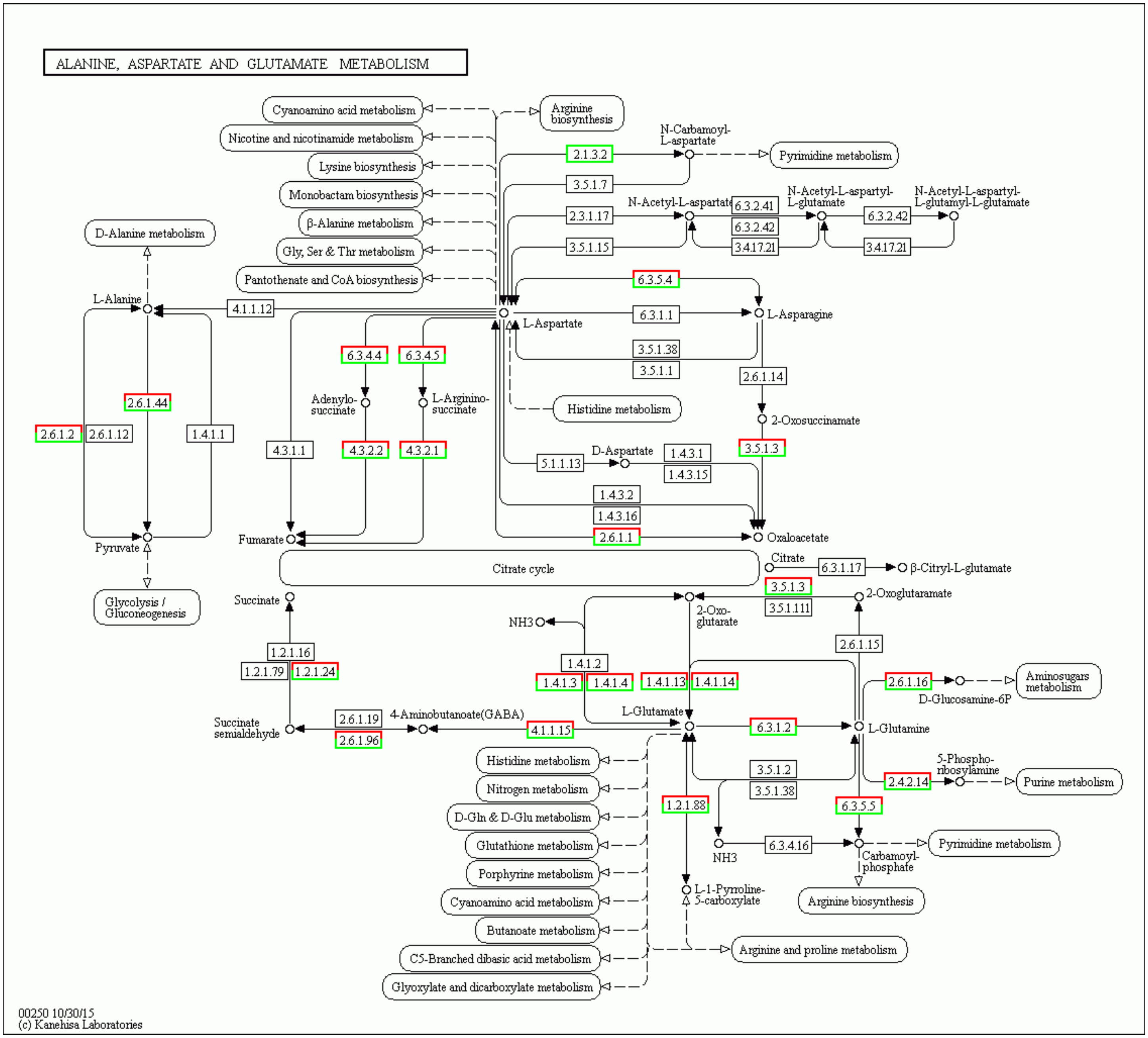
ALANINE, ASPARTATE AND GLUTAMATE METABOLISM
Cyanoamino acid metabolism
Arginine biosynthesis
Nicotine and nicotinamide metabolism
Lysine biosynthesis
Monobactam biosynthesis
β-Alanine metabolism
Gly, Ser & Thr metabolism
Pantothenate and CoA biosynthesis
D-Alanine metabolism
N-Carbamoyl-L-aspartate
Pyrimidine metabolism
2.1.3.2
3.5.1.7
N-Acetyl-L-aspartate
N-Acetyl-L-aspartyl-L-glutamate
N-Acetyl-L-aspartyl-L-glutamyl-L-glutamate
2.3.1.17
3.5.1.15
6.3.2.41
6.3.2.42
3.4.17.21
6.3.2.42
3.4.17.21
6.3.5.4
L-Alanine
4.1.1.12
L-Aspartate
6.3.1.1
L-Asparagine
3.5.1.38
3.5.1.1
2.6.1.14
6.3.4.4
6.3.4.5
Adenylo-succinate
L-Arginino-succinate
Histidine metabolism
2-Oxosuccinamate
2.6.1.44
2.6.1.2
2.6.1.12
1.4.1.1
4.3.1.1
4.3.2.2
4.3.2.1
3.5.1.3
D-Aspartate
5.1.1.13
1.4.3.1
1.4.3.15
1.4.3.2
1.4.3.16
2.6.1.1
Pyruvate
Fumarate
Oxaloacetate
Citrate cycle
Citrate
6.3.1.17
β-Citryl-L-glutamate
3.5.1.3
3.5.1.111
2-Oxoglutaramate
Glycolysis / Gluconeogenesis
Succinate
NH3
2-Oxo-glutarate
1.4.1.2
2.6.1.15
1.2.1.16
1.2.1.79
1.2.1.24
1.4.1.3
1.4.1.4
1.4.1.13
1.4.1.14
2.6.1.16
Aminosugars metabolism
D-Glucosamine-6P
L-Glutamate
Succinate semialdehyde
2.6.1.19
2.6.1.96
4-Aminobutanoate(GABA)
4.1.1.15
6.3.1.2
L-Glutamine
Histidine metabolism
Nitrogen metabolism
D-Gln & D-Glu metabolism
Glutathione metabolism
Porphyrine metabolism
Cyanoamino acid metabolism
Butanoate metabolism
C5-Branched dibasic acid metabolism
Glyoxylate and dicarboxylate metabolism
3.5.1.2
3.5.1.38
5-Phospho-ribosylamine
2.4.2.14
Purine metabolism
1.2.1.88
6.3.5.5
NH3
6.3.4.16
Carbamoyl-phosphate
Pyrimidine metabolism
L-1-Pyrroline-5-carboxylate
Arginine biosynthesis
Arginine and proline metabolism
00250 10/30/15
(c) Kanehisa Laboratories

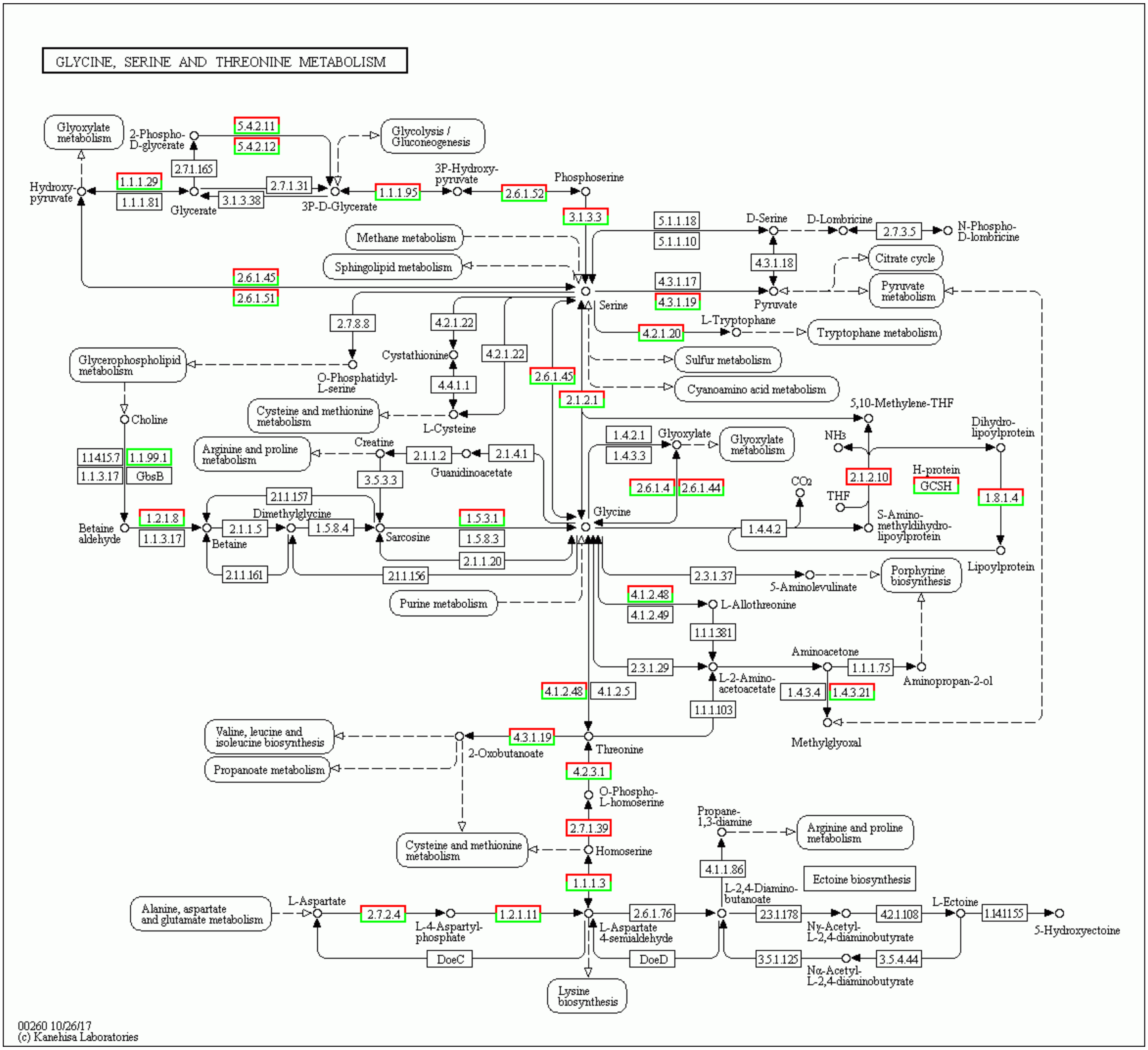
GLYCINE, SERINE AND THREONINE METABOLISM
Glyoxylate metabolism
2-Phospho-D-glycerate
5.4.2.11
5.4.2.12
Glycolysis / Gluconeogenesis
2.7.1.165
1.1.1.29
1.1.1.81
Hydroxy-pyruvate
Glycerate
2.7.1.31
3.1.3.38
3P-D-Glycerate
1.1.1.95
3P-Hydroxy-pyruvate
2.6.1.52
Phosphoserine
3.1.3.3
5.1.1.18
5.1.1.10
D-Serine
D-Lombricine
2.7.3.5
N-Phospho-D-lombricine
4.3.1.18
Citrate cycle
Methane metabolism
Sphingolipid metabolism
2.6.1.45
2.6.1.51
4.3.1.17
4.3.1.19
Pyruvate
Pyruvate metabolism
Serine
2.7.8.8
4.2.1.22
4.2.1.22
4.2.1.20
L-Tryptophan
Tryptophan metabolism
Cystathionine
Glycerophospholipid metabolism
O-Phosphatidyl-L-serine
2.6.1.45
Sulfur metabolism
4.4.1.1
2.1.2.1
Cyanoamino acid metabolism
Cysteine and methionine metabolism
L-Cysteine
5,10-Methylene-THF
Choline
Creatine
Arginine and proline metabolism
2.1.1.2
2.1.4.1
Guanidinoacetate
1.4.2.1
1.4.3.3
Glyoxylate
Glyoxylate metabolism
NH3
Dihydro-lipoylprotein
1.1.4.15.7
1.1.99.1
1.1.3.17
GbsB
3.5.3.3
2.6.1.4
2.6.1.44
2.1.2.10
H-protein
GCSH
1.8.1.4
CO2
THF
2.1.1.157
Dimethylglycine
Betaine aldehyde
1.2.1.8
1.1.3.17
2.1.1.5
Betaine
1.5.8.4
Sarcosine
1.5.3.1
1.5.8.3
Glycine
1.4.4.2
S-Amino-methyldihydro-lipoylprotein
Lipoylprotein
2.1.1.20
2.1.1.161
2.1.1.156
2.3.1.37
5-Aminolevulinate
Porphyrine biosynthesis
Purine metabolism
4.1.2.48
4.1.2.49
L-Allothreonine
1.1.1.381
Aminoacetone
2.3.1.29
1.1.1.75
Aminopropan-2-ol
L-2-Amino-acetoacetate
4.1.2.48
4.1.2.5
1.4.3.4
1.4.3.21
1.1.1.103
Methylglyoxal
Valine, leucine and isoleucine biosynthesis
4.3.1.19
2-Oxobutanoate
Threonine
Propanoate metabolism
4.2.3.1
O-Phospho-L-homoserine
2.7.1.39
Propane-1,3-diamine
Arginine and proline metabolism
Cysteine and methionine metabolism
Homoserine
4.1.1.86
Ectoine biosynthesis
1.1.1.3
L-2,4-Diamino-butanoate
Alanine, aspartate and glutamate metabolism
L-Aspartate
2.7.2.4
1.2.1.11
2.6.1.76
2.3.1.178
4.2.1.108
L-Ectoine
1.14.11.55
5-Hydroxyectoine
L-4-Aspartyl-phosphate
L-Aspartate 4-semialdehyde
Nγ-Acetyl-L-2,4-diaminobutyrate
DoeC
DoeD
3.5.1.125
3.5.4.44
Nα-Acetyl-L-2,4-diaminobutyrate
Lysine biosynthesis
00260 10/26/17
(c) Kanehisa Laboratories

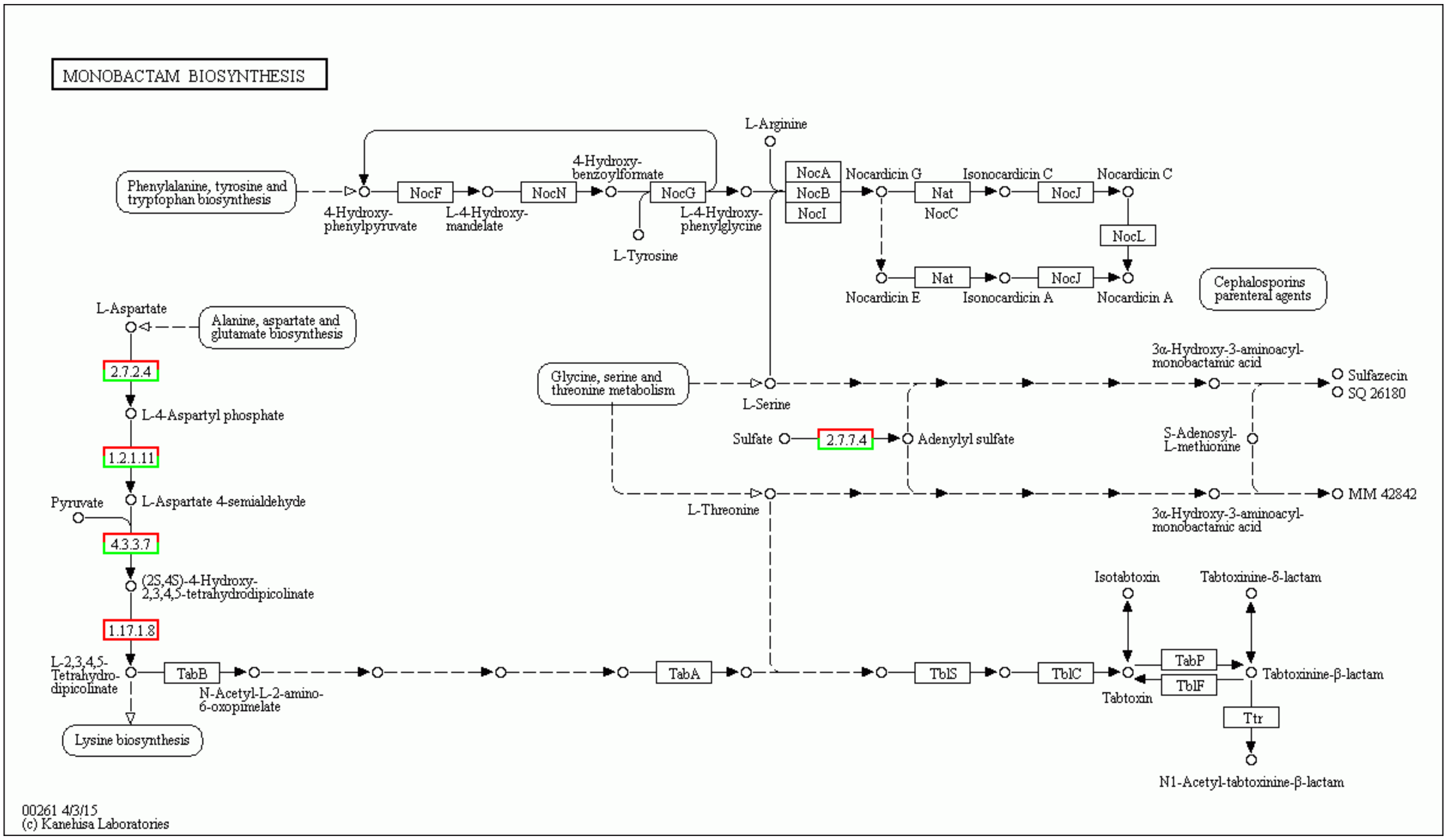
MONOBACTAM BIOSYNTHESIS
Phenylalanine, tyrosine and tryptophan biosynthesis
4-Hydroxy-phenylpyruvate
NocF
L-4-Hydroxy-mandelate
NocN
4-Hydroxy-benzoylformate
NocG
L-Tyrosine
L-4-Hydroxy-phenylglycine
L-Arginine
NocA
NocB
NocI
Nocardicin G
Nat
NocC
Isonocardicin C
NocJ
Nocardicin C
NocL
Nocardicin E
Nat
Isonocardicin A
NocJ
Nocardicin A
Cephalosporins parenteral agents
L-Aspartate
Alanine, aspartate and glutamate biosynthesis
2.7.2.4
L-4-Aspartyl phosphate
1.2.1.11
Pyruvate
L-Aspartate 4-semialdehyde
4.3.3.7
(2S,4S)-4-Hydroxy-2,3,4,5-tetrahydrodipicolinate
1.17.1.8
L-2,3,4,5-Tetrahydro-dipicolinate
TabB
N-Acetyl-L-2-amino-6-oxopimelate
Lysine biosynthesis
Glycine, serine and threonine metabolism
L-Serine
Sulfate
2.7.7.4
Adenylyl sulfate
L-Threonine
3α-Hydroxy-3-aminoacyl-monobactamic acid
Sulfazecin
SQ 26180
S-Adenosyl-L-methionine
3α-Hydroxy-3-aminoacyl-monobactamic acid
MM 42842
TabA
TblS
TblC
Isotabtoxin
Tabtoxinine-δ-lactam
TabP
TblF
Tabtoxin
Tabtoxinine-β-lactam
Ttr
N1-Acetyl-tabtoxinine-β-lactam
00261 4/3/15
(c) Kanehisa Laboratories

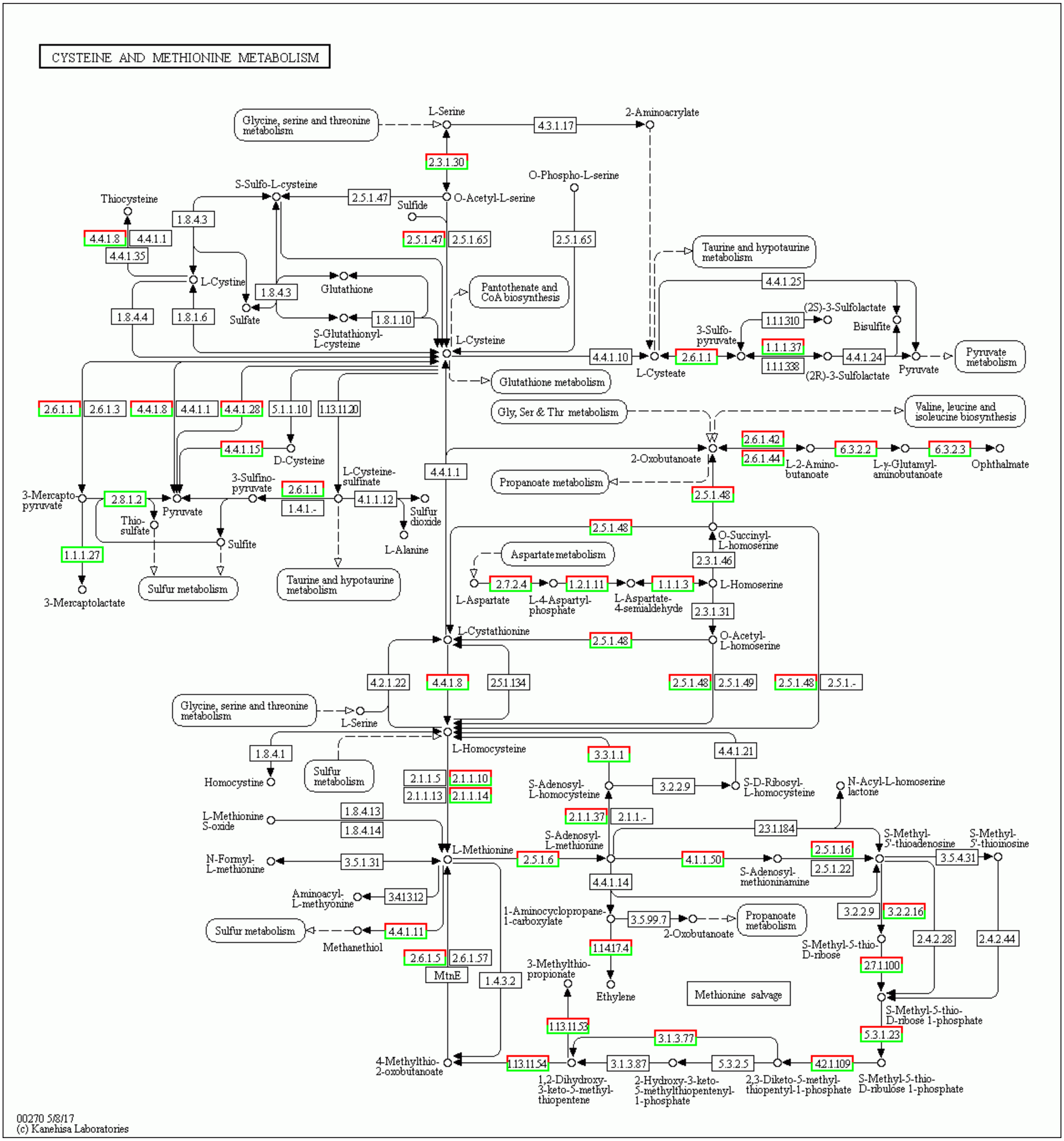
CYSTEINE AND METHIONINE METABOLISM
Glycine, serine and threonine metabolism
L-Serine
4.3.1.17
2-Aminoacrylate
2.3.1.30
O-Phospho-L-serine
S-Sulfo-L-cysteine
2.5.1.47
O-Acetyl-L-serine
Thiocysteine
1.8.4.3
Sulfide
4.4.1.8
4.4.1.1
4.4.1.35
2.5.1.47
2.5.1.65
2.5.1.65
Taurine and hypotaurine metabolism
L-Cystine
1.8.4.3
Glutathione
Pantothenate and CoA biosynthesis
4.4.1.25
Sulfate
1.8.4.4
1.8.1.6
1.8.1.10
S-Glutathionyl-L-cysteine
L-Cysteine
(2S)-3-Sulfolactate
3-Sulfo-pyruvate
1.1.1.310
Bisulfite
1.1.1.37
4.4.1.10
2.6.1.1
4.4.1.24
Pyruvate metabolism
L-Cysteate
1.1.1.338
(2R)-3-Sulfolactate
Pyruvate
Glutathione metabolism
2.6.1.1
2.6.1.3
4.4.1.8
4.4.1.1
4.4.1.28
5.1.1.10
1.13.11.20
Gly, Ser & Thr metabolism
Valine, leucine and isoleucine biosynthesis
2.6.1.42
4.4.1.15
D-Cysteine
2-Oxobutanoate
6.3.2.2
6.3.2.3
2.6.1.44
L-2-Amino-butanoate
L-γ-Glutamyl-aminobutanoate
Ophthalmate
L-Cysteine-sulfinate
4.4.1.1
3-Sulfino-pyruvate
Propanoate metabolism
2.6.1.1
3-Mercapto-pyruvate
2.8.1.2
Pyruvate
4.1.1.12
Sulfur dioxide
2.5.1.48
1.4.1.-
Thio-sulfate
2.5.1.48
O-Succinyl-L-homoserine
Sulfite
L-Alanine
1.1.1.27
Aspartate metabolism
2.3.1.46
Sulfur metabolism
Taurine and hypotaurine metabolism
2.7.2.4
1.2.1.11
1.1.1.3
L-Homoserine
3-Mercaptolactate
L-Aspartate
L-4-Aspartyl-phosphate
L-Aspartate-4-semialdehyde
2.3.1.31
L-Cystathionine
2.5.1.48
O-Acetyl-L-homoserine
4.2.1.22
4.4.1.8
2.5.1.134
2.5.1.48
2.5.1.49
2.5.1.48
2.5.1.-
Glycine, serine and threonine metabolism
L-Serine
L-Homocysteine
1.8.4.1
4.4.1.21
3.3.1.1
Sulfur metabolism
Homocysteine
2.1.1.5
2.1.1.10
S-Adenosyl-L-homocysteine
3.2.2.9
S-D-Ribosyl-L-homocysteine
N-Acyl-L-homoserine lactone
2.1.1.13
2.1.1.14
L-Methionine S-oxide
1.8.4.13
2.1.1.37
2.1.1.-
1.8.4.14
2.3.1.184
S-Adenosyl-L-methionine
S-Methyl-5'-thioadenosine
S-Methyl-5'-thioinosine
L-Methionine
2.5.1.16
N-Formyl-L-methionine
3.5.1.31
2.5.1.6
4.1.1.50
S-Adenosyl-methioninamine
3.5.4.31
2.5.1.22
4.4.1.14
Aminoacyl-L-methyonine
3.4.13.12
1-Aminocyclopropane-1-carboxylate
3.5.99.7
Propanoate metabolism
3.2.2.9
3.2.2.16
Sulfur metabolism
4.4.1.11
2-Oxobutanoate
S-Methyl-5-thio-D-ribose
2.4.2.28
2.4.2.44
Methanethiol
1.14.17.4
2.6.1.5
2.6.1.57
3-Methylthio-propionate
2.7.1.100
MtnE
1.4.3.2
Ethylene
Methionine salvage
S-Methyl-5-thio-D-ribose 1-phosphate
1.13.11.53
5.3.1.23
3.1.3.77
4-Methylthio-2-oxobutanoate
1.13.11.54
3.1.3.87
5.3.2.5
4.2.1.109
1,2-Dihydroxy-3-keto-5-methyl-thiopentene
2-Hydroxy-3-keto-5-methylthiopentenyl-1-phosphate
2,3-Diketo-5-methyl-thiopentyl-1-phosphate
S-Methyl-5-thio-D-ribulose 1-phosphate
00270 5/8/17
(c) Kanehisa Laboratories

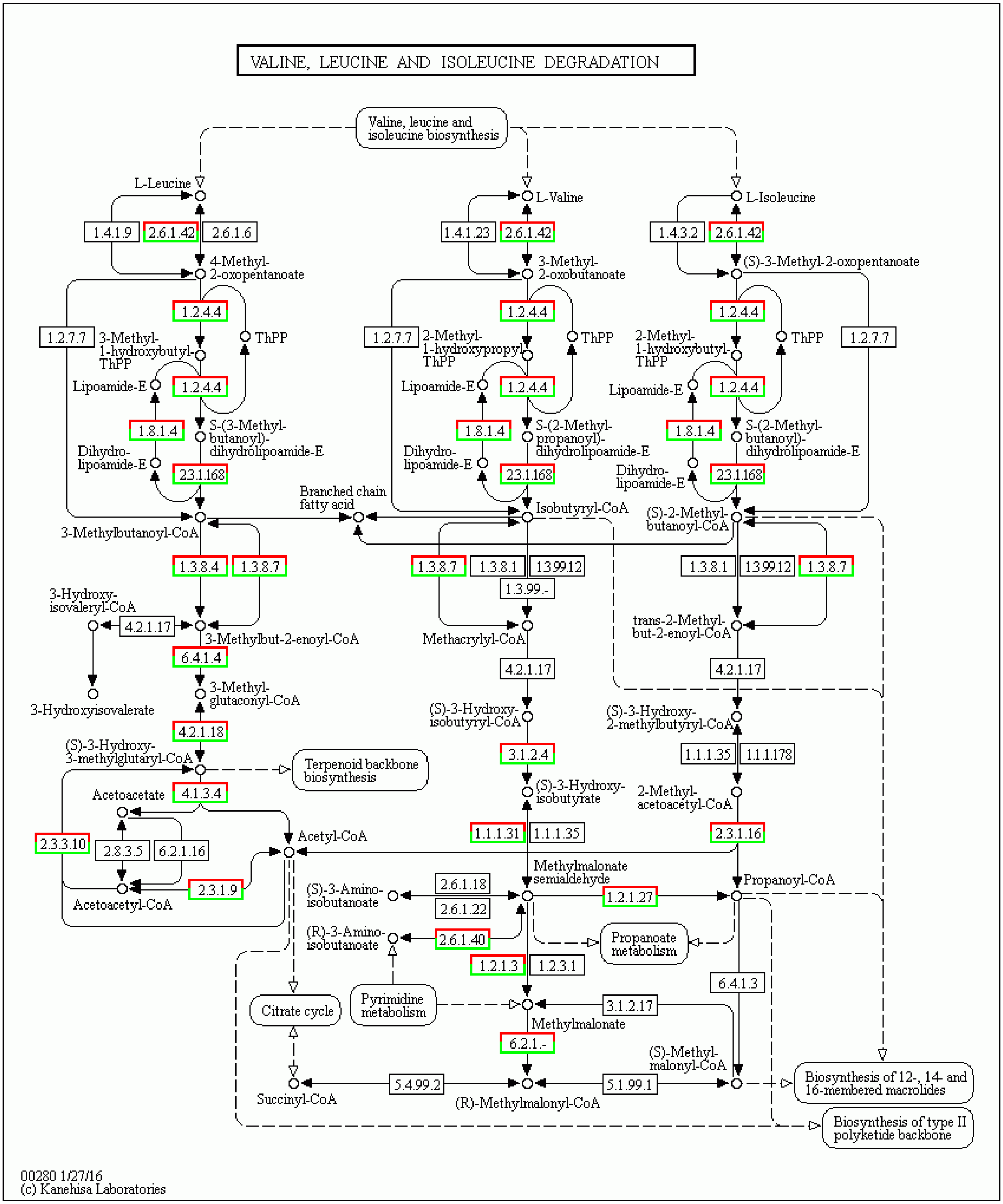
VALINE, LEUCINE AND ISOLEUCINE DEGRADATION
Valine, leucine and isoleucine biosynthesis
L-Leucine
L-Valine
L-Isoleucine
1.4.1.9
2.6.1.42
2.6.1.6
1.4.1.23
2.6.1.42
1.4.3.2
2.6.1.42
4-Methyl-2-oxopentanoate
3-Methyl-2-oxobutanoate
(S)-3-Methyl-2-oxopentanoate
1.2.4.4
1.2.7.7
3-Methyl-1-hydroxybutyl-ThPP
ThPP
2-Methyl-1-hydroxypropyl-ThPP
2-Methyl-1-hydroxybutyl-ThPP
Lipoamide-E
1.8.1.4
S-(3-Methylbutanoyl)-dihydrolipoamide-E
S-(2-Methylpropanoyl)-dihydrolipoamide-E
S-(2-Methylbutanoyl)-dihydrolipoamide-E
Dihydrolipoamide-E
2.3.1.168
Branched chain fatty acid
3-Methylbutanoyl-CoA
Isobutyryl-CoA
(S)-2-Methylbutanoyl-CoA
1.3.8.4
1.3.8.7
1.3.8.1
1.3.99.12
1.3.99.-
3-Hydroxy-isovaleryl-CoA
4.2.1.17
3-Methylbut-2-enoyl-CoA
Methacrylyl-CoA
trans-2-Methyl-but-2-enoyl-CoA
6.4.1.4
3-Hydroxyisovalerate
3-Methyl-glutaconyl-CoA
4.2.1.18
(S)-3-Hydroxy-isobutyryl-CoA
(S)-3-Hydroxy-2-methylbutyryl-CoA
(S)-3-Hydroxy-3-methylglutaryl-CoA
Terpenoid backbone biosynthesis
3.1.2.4
1.1.1.35
1.1.1.178
Acetoacetate
4.1.3.4
(S)-3-Hydroxy-isobutyrate
2-Methyl-acetoacetyl-CoA
2.3.3.10
2.8.3.5
6.2.1.16
Acetyl-CoA
1.1.1.31
2.3.1.16
2.3.1.9
Acetoacetyl-CoA
Methylmalonate semialdehyde
Propanoyl-CoA
(S)-3-Amino-isobutanoate
2.6.1.18
2.6.1.22
1.2.1.27
(R)-3-Amino-isobutanoate
2.6.1.40
Propanoate metabolism
1.2.1.3
1.2.3.1
6.4.1.3
Citrate cycle
Pyrimidine metabolism
3.1.2.17
Methylmalonate
6.2.1.-
(S)-Methyl-malonyl-CoA
Biosynthesis of 12-, 14- and 16-membered macrolides
Succinyl-CoA
5.4.99.2
(R)-Methylmalonyl-CoA
5.1.99.1
Biosynthesis of type II polyketide backbone
00280 1/27/16
(c) Kanehisa Laboratories

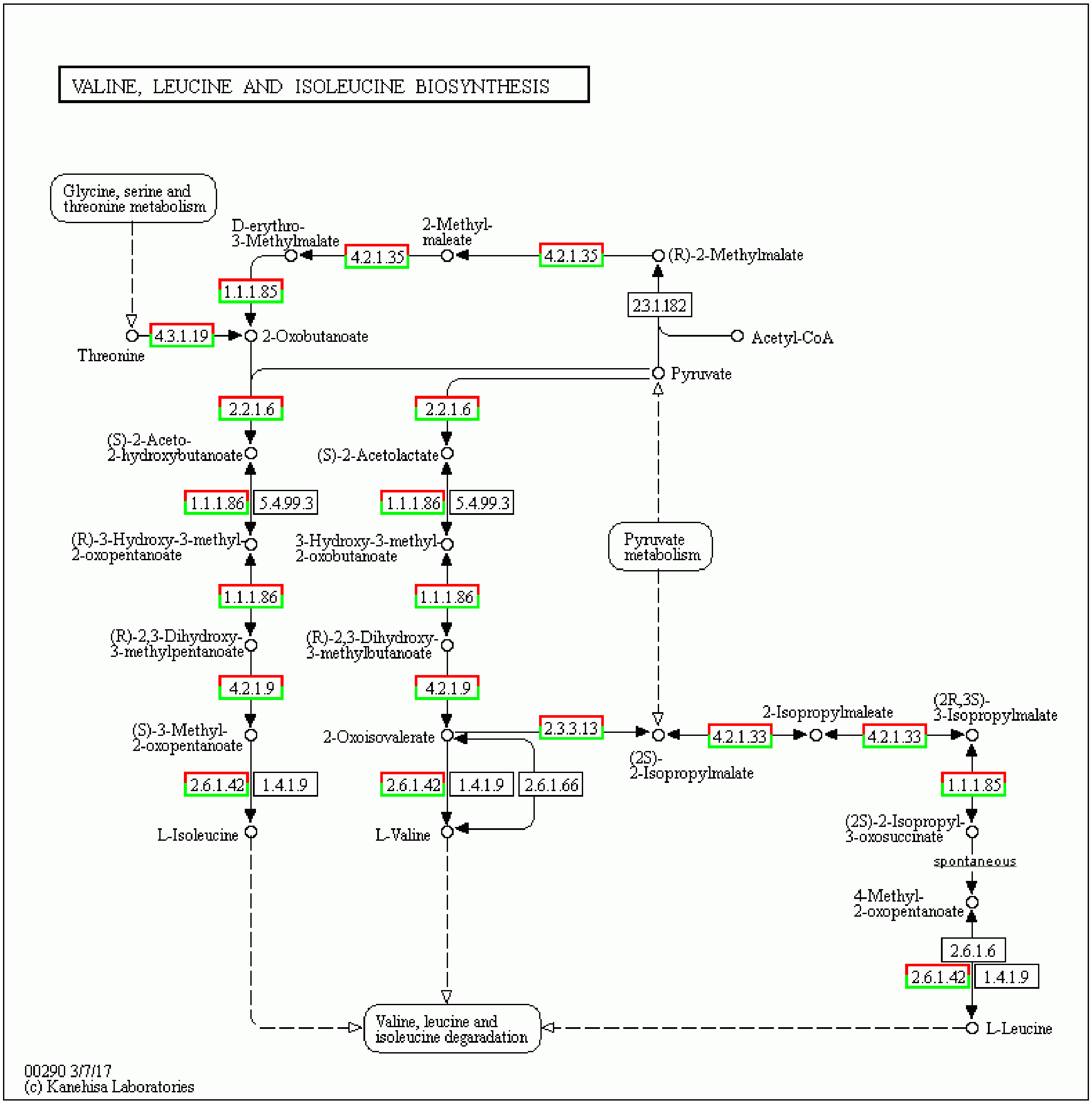
VALINE, LEUCINE AND ISOLEUCINE BIOSYNTHESIS
Glycine, serine and threonine metabolism
D-erythro-3-Methylmalate
2-Methyl-maleate
4.2.1.35
4.2.1.35
(R)-2-Methylmalate
1.1.1.85
2.3.1.182
Threonine
4.3.1.19
2-Oxobutanoate
Acetyl-CoA
Pyruvate
2.2.1.6
2.2.1.6
(S)-2-Aceto-2-hydroxybutanoate
(S)-2-Acetolactate
1.1.1.86
5.4.99.3
1.1.1.86
5.4.99.3
(R)-3-Hydroxy-3-methyl-2-oxopentanoate
3-Hydroxy-3-methyl-2-oxobutanoate
Pyruvate metabolism
1.1.1.86
1.1.1.86
(R)-2,3-Dihydroxy-3-methylpentanoate
(R)-2,3-Dihydroxy-3-methylbutanoate
4.2.1.9
4.2.1.9
(S)-3-Methyl-2-oxopentanoate
2-Oxoisovalerate
2.3.3.13
(2S)-2-Isopropylmalate
2-Isopropylmaleate
4.2.1.33
4.2.1.33
(2R,3S)-3-Isopropylmalate
2.6.1.42
1.4.1.9
2.6.1.42
1.4.1.9
2.6.1.66
1.1.1.85
L-Isoleucine
L-Valine
(2S)-2-Isopropyl-3-oxosuccinate
spontaneous
4-Methyl-2-oxopentanoate
2.6.1.6
2.6.1.42
1.4.1.9
Valine, leucine and isoleucine degaradation
L-Leucine
00290 3/7/17
(c) Kanehisa Laboratories

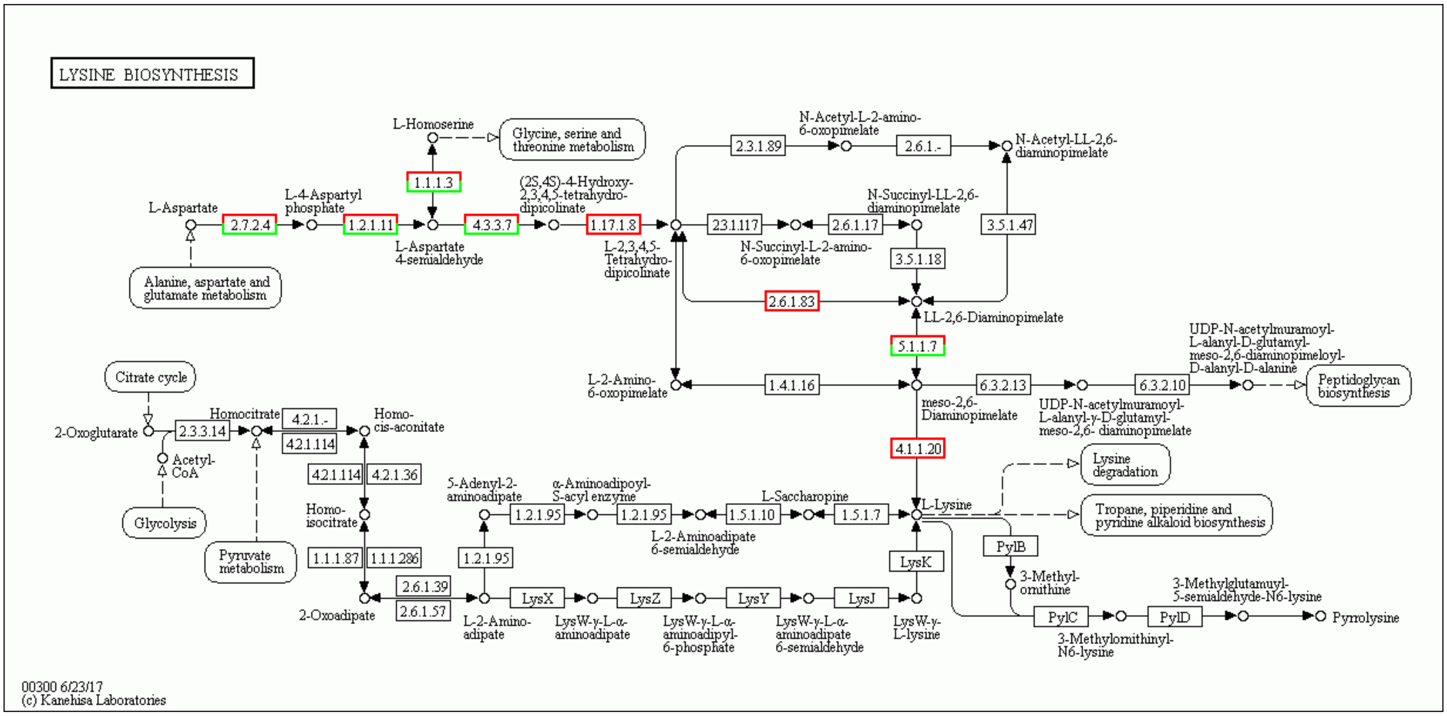
LYSINE BIOSYNTHESIS
L-Homoserine
Glycine, serine and threonine metabolism
1.1.1.3
L-Aspartate
2.7.2.4
L-4-Aspartyl phosphate
1.2.1.11
L-Aspartate 4-semialdehyde
4.3.3.7
(2S,4S)-4-Hydroxy-2,3,4,5-tetrahydro-dipicolinate
1.17.1.8
L-2,3,4,5-Tetrahydro-dipicolinate
Alanine, aspartate and glutamate metabolism
2.3.1.89
N-Acetyl-L-2-amino-6-oxopimelate
2.6.1.-
N-Acetyl-LL-2,6-diaminopimelate
2.3.1.117
2.6.1.17
N-Succinyl-LL-2,6-diaminopimelate
N-Succinyl-L-2-amino-6-oxopimelate
3.5.1.18
3.5.1.47
2.6.1.83
LL-2,6-Diaminopimelate
5.1.1.7
UDP-N-acetylmuramoyl-L-alanyl-D-glutamyl-meso-2,6-diaminopimeloyl-D-alanyl-D-alanine
L-2-Amino-6-oxopimelate
1.4.1.16
6.3.2.13
6.3.2.10
Peptidoglycan biosynthesis
meso-2,6-Diaminopimelate
UDP-N-acetylmuramoyl-L-alanyl-γ-D-glutamyl-meso-2,6- diaminopimelate
Citrate cycle
2-Oxoglutarate
2.3.3.14
Homocitrate
4.2.1.-
4.2.1.114
Homo-cis-aconitate
Acetyl-CoA
4.2.1.114
4.2.1.36
Glycolysis
Homo-isocitrate
Pyruvate metabolism
1.1.1.87
1.1.1.286
2-Oxoadipate
2.6.1.39
2.6.1.57
5-Adenyl-2-aminoadipate
α-Aminoadipoyl-S-acyl enzyme
1.2.1.95
1.2.1.95
L-Saccharopine
1.5.1.10
1.5.1.7
L-2-Aminoadipate 6-semialdehyde
4.1.1.20
L-Lysine
Lysine degradation
Tropane, piperidine and pyridine alkaloid biosynthesis
PylB
3-Methyl-ornithine
LysK
LysX
LysZ
LysY
LysJ
L-2-Amino-adipate
LysW-γ-L-α-aminoadipate
LysW-γ-L-α-aminoadipyl-6-phosphate
LysW-γ-L-α-aminoadipate 6-semialdehyde
LysW-γ-L-lysine
PylC
PylD
3-Methylglutamuyl-5-semialdehyde-N6-lysine
3-Methylornithinyl-N6-lysine
Pyrrolysine
00300 6/23/17
(c) Kanehisa Laboratories

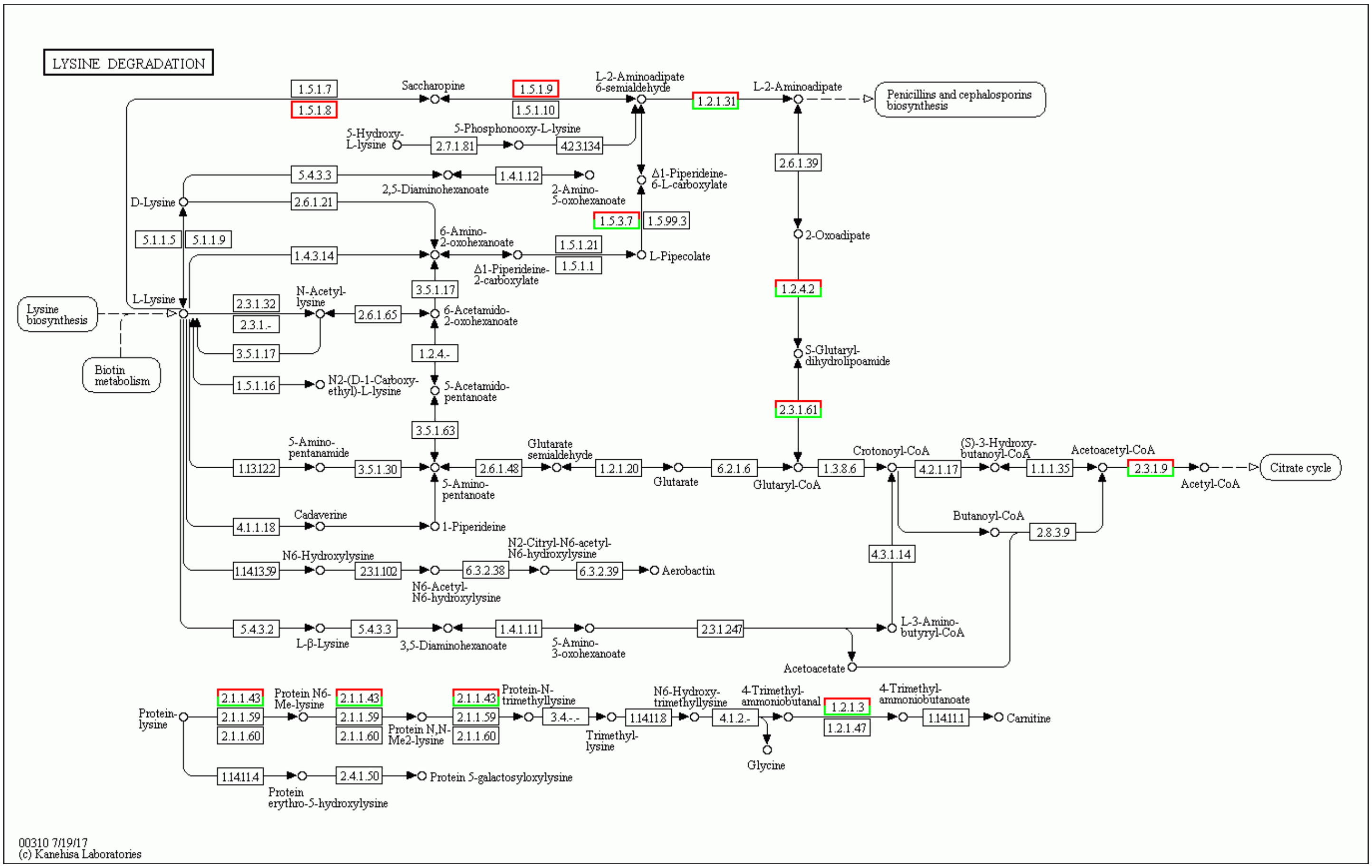
LYSINE DEGRADATION
1.5.1.7
1.5.1.8
Saccharopine
1.5.1.9
1.5.1.10
L-2-Aminoadipate 6-semialdehyde
1.2.1.31
L-2-Aminoadipate
Penicillins and cephalosporins biosynthesis
5-Hydroxy-L-lysine
2.7.1.81
5-Phosphonooxy-L-lysine
4.2.3.134
5.4.3.3
2,5-Diaminohexanoate
1.4.1.12
2-Amino-5-oxohexanoate
Δ1-Piperideine-6-L-carboxylate
2.6.1.39
D-Lysine
2.6.1.21
1.5.3.7
1.5.99.3
2-Oxoadipate
5.1.1.5
5.1.1.9
1.4.3.14
6-Amino-2-oxohexanoate
1.5.1.21
1.5.1.1
Δ1-Piperideine-2-carboxylate
L-Pipecolate
Lysine biosynthesis
L-Lysine
2.3.1.32
N-Acetyl-lysine
2.6.1.65
3.5.1.17
6-Acetamido-2-oxohexanoate
1.2.4.2
2.3.1.-
Biotin metabolism
3.5.1.17
1.2.4.-
S-Glutaryl-dihydrolipoamide
1.5.1.16
N2-(D-1-Carboxy-ethyl)-L-lysine
5-Acetamido-pentanoate
3.5.1.63
2.3.1.61
5-Amino-pentanamide
1.13.12.2
3.5.1.30
Glutarate semialdehyde
2.6.1.48
1.2.1.20
Glutarate
6.2.1.6
Glutaryl-CoA
Crotonoyl-CoA
1.3.8.6
(S)-3-Hydroxy-butanoyl-CoA
4.2.1.17
1.1.1.35
Acetoacetyl-CoA
2.3.1.9
Acetyl-CoA
Citrate cycle
5-Amino-pentanoate
Cadaverine
4.1.1.18
1-Piperideine
Butanoyl-CoA
2.8.3.9
N6-Hydroxylysine
N2-Citryl-N6-acetyl-N6-hydroxylysine
1.14.13.59
2.3.1.102
6.3.2.38
6.3.2.39
Aerobactin
N6-Acetyl-N6-hydroxylysine
4.3.1.14
5.4.3.2
L-β-Lysine
5.4.3.3
3,5-Diaminohexanoate
1.4.1.11
5-Amino-3-oxohexanoate
2.3.1.247
L-3-Amino-butyryl-CoA
Acetoacetate
Protein-lysine
2.1.1.43
2.1.1.59
2.1.1.60
Protein N6-Me-lysine
2.1.1.43
2.1.1.59
2.1.1.60
Protein N,N-Me2-lysine
2.1.1.43
2.1.1.59
2.1.1.60
Protein-N-trimethyllysine
3.4.-.-
Trimethyl-lysine
1.14.11.8
N6-Hydroxy-trimethyllysine
4.1.2.-
4-Trimethyl-ammoniobutanal
1.2.1.3
1.2.1.47
4-Trimethyl-ammoniobutanoate
1.14.11.1
Carnitine
Glycine
1.14.11.4
Protein erythro-5-hydroxylysine
2.4.1.50
Protein 5-galactosyloxylysine
00310 7/19/17
(c) Kanehisa Laboratories

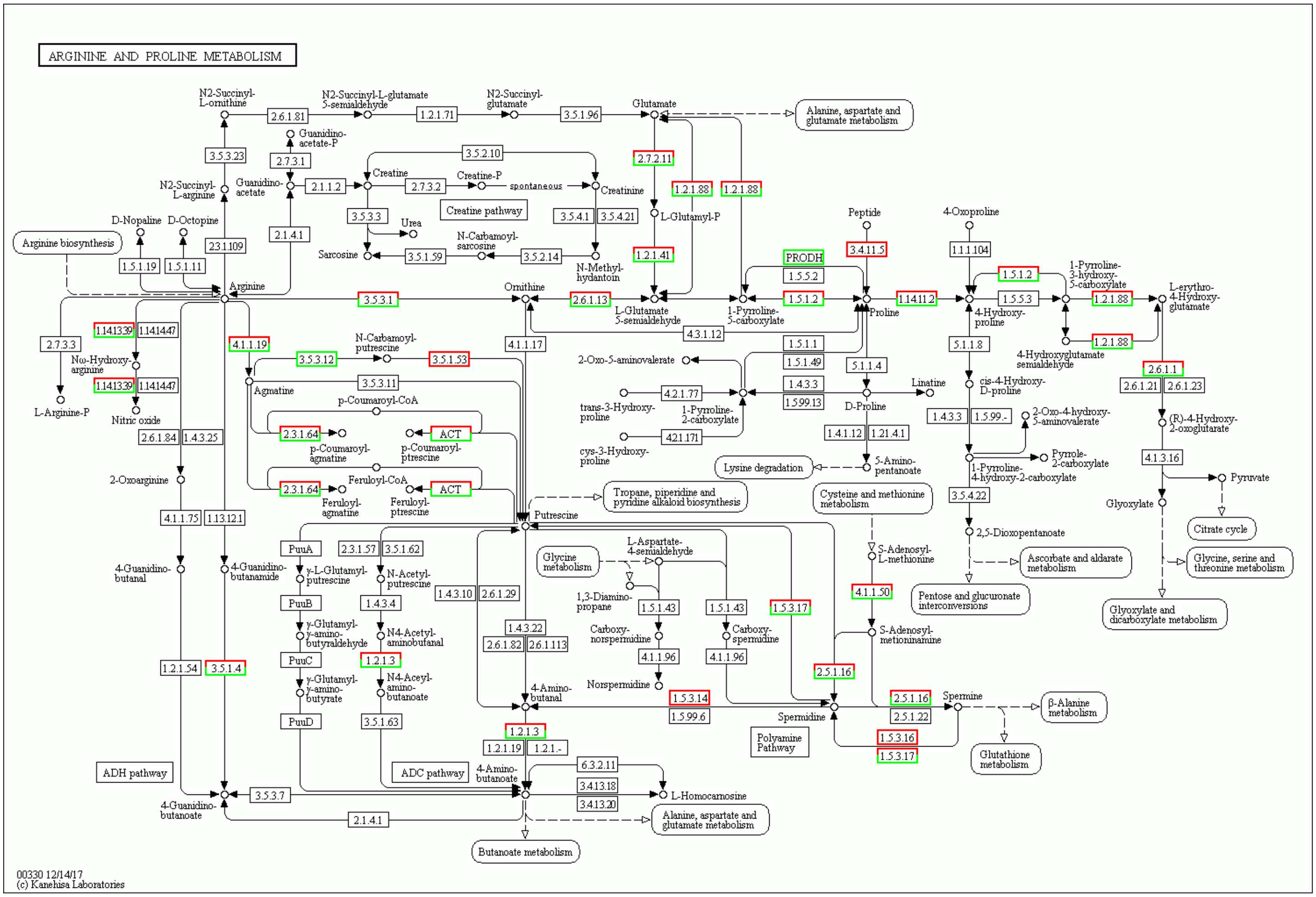
ARGININE AND PROLINE METABOLISM
N2-Succinyl-L-ornithine
2.6.1.81
N2-Succinyl-L-glutamate 5-semialdehyde
1.2.1.71
N2-Succinyl-glutamate
3.5.1.96
Glutamate
Alanine, aspartate and glutamate metabolism
Guanidino-acetate-P
3.5.3.23
2.7.3.1
3.5.2.10
2.7.2.11
N2-Succinyl-L-arginine
Guanidino-acetate
2.1.1.2
Creatine
2.7.3.2
Creatine-P
spontaneous
Creatinine
1.2.1.88
1.2.1.88
Creatine pathway
3.5.3.3
Urea
3.5.4.1
3.5.4.21
L-Glutamyl-P
D-Nopaline
D-Octopine
2.1.4.1
N-Carbamoyl-sarcosine
Peptide
4-Oxoproline
Arginine biosynthesis
23.1.109
Sarcosine
3.5.1.59
3.5.2.14
N-Methyl-hydantoin
1.2.1.41
PRODH
3.4.11.5
1.1.1.104
1.5.1.19
1.5.1.11
Arginine
1.5.5.2
1.5.1.2
1-Pyrroline-3-hydroxy-5-carboxylate
3.5.3.1
Ornithine
2.6.1.13
L-Glutamate 5-semialdehyde
1-Pyrroline-5-carboxylate
1.5.1.2
1.14.11.2
Proline
4-Hydroxy-proline
1.5.5.3
1.2.1.88
L-erythro-4-Hydroxy-glutamate
1.14.13.39
1.14.14.47
4.3.1.12
2.7.3.3
Nω-Hydroxy-arginine
4.1.1.19
N-Carbamoyl-putrescine
4.1.1.17
1.5.1.1
5.1.1.8
4-Hydroxyglutamate semialdehyde
1.2.1.88
3.5.1.2
3.5.1.53
2-Oxo-5-aminovalerate
1.5.1.49
5.1.1.4
2.6.1.1
1.14.13.39
1.14.14.47
3.5.3.11
Agmatine
p-Coumaroyl-CoA
4.2.1.77
1.4.3.3
Linatine
2.6.1.21
2.6.1.23
L-Arginine-P
Nitric oxide
trans-3-Hydroxy-proline
1-Pyrroline-2-carboxylate
1.5.99.13
D-Proline
cis-4-Hydroxy-D-proline
2-Oxo-4-hydroxy-5-aminovalerate
2.3.1.64
ACT
1.4.3.3
1.5.99.-
(R)-4-Hydroxy-2-oxoglutarate
2.6.1.84
1.4.3.25
p-Coumaroyl-agmatine
p-Coumaroyl-ptrescine
4.2.1.171
1.4.1.12
1.21.4.1
cys-3-Hydroxy-proline
Pyrrole-2-carboxylate
4.1.3.16
Lysine degradation
5-Amino-pentanoate
1-Pyrroline-4-hydroxy-2-carboxylate
2-Oxoarginine
Feruloyl-CoA
2.3.1.64
ACT
Pyruvate
Feruloyl-agmatine
Feruloyl-ptrescine
Tropane, piperidine and pyridine alkaloid biosynthesis
Cysteine and methionine metabolism
3.5.4.22
Glyoxylate
4.1.1.75
1.13.12.1
Putrescine
Citrate cycle
2,5-Dioxopentanoate
PuuA
2.3.1.57
3.5.1.62
L-Aspartate-4-semialdehyde
S-Adenosyl-L-methionine
Ascorbate and aldarate metabolism
Glycine, serine and threonine metabolism
4-Guanidino-butanal
4-Guanidino-butanamide
γ-L-Glutamyl-putrescine
N-Acetyl-putrescine
Glycine metabolism
1,3-Diamino-propane
4.1.1.50
Pentose and glucuronate interconversions
PuuB
1.4.3.4
1.4.3.10
2.6.1.29
1.5.1.43
1.5.1.43
1.5.3.17
Glyoxylate and dicarboxylate metabolism
γ-Glutamyl-γ-amino-butyraldehyde
N4-Acetyl-aminobutanal
1.4.3.22
Carboxy-norspermidine
Carboxy-spermidine
S-Adenosyl-methioninamine
2.6.1.82
2.6.1.113
1.2.1.54
3.5.1.4
PuuC
1.2.1.3
4.1.1.96
4.1.1.96
2.5.1.16
γ-Glutamyl-γ-amino-butyrate
N4-Acetyl-amino-butanoate
Norspermidine
4-Amino-butanal
1.5.3.14
2.5.1.16
Spermine
β-Alanine metabolism
PuuD
3.5.1.63
1.5.99.6
Spermidine
2.5.1.22
1.2.1.3
Polyamine Pathway
1.5.3.16
1.2.1.19
1.2.1.-
1.5.3.17
Glutathione metabolism
ADH pathway
ADC pathway
4-Amino-butanoate
6.3.2.11
3.5.3.7
3.4.13.18
L-Homocarnosine
3.4.13.20
4-Guanidino-butanoate
2.1.4.1
Alanine, aspartate and glutamate metabolism
Butanoate metabolism
00330 12/14/17
(c) Kanehisa Laboratories

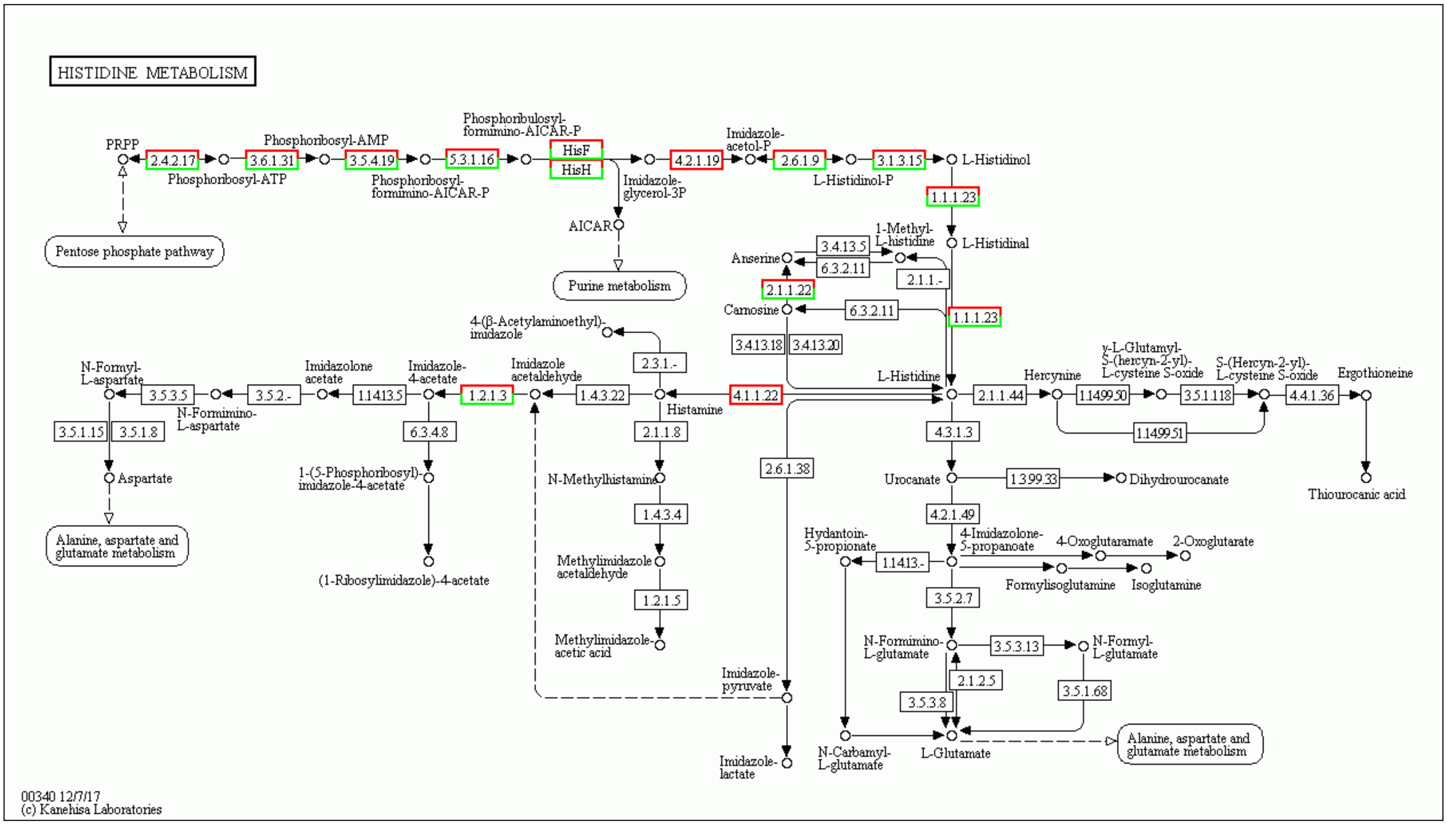
HISTIDINE METABOLISM
Phosphoribulosyl-formimino-AICAR-P
PRPP
2.4.2.17
Phosphoribosyl-AMP
3.6.1.31
3.5.4.19
5.3.1.16
HisF
HisH
4.2.1.19
Imidazole-acetol-P
2.6.1.9
3.1.3.15
L-Histidinol
Phosphoribosyl-ATP
Phosphoribosyl-formimino-AICAR-P
Imidazole-glycerol-3P
L-Histidinol-P
1.1.1.23
AICAR
Pentose phosphate pathway
1-Methyl-L-histidine
3.4.13.5
L-Histidinal
Anserine
6.3.2.11
2.1.1.-
Purine metabolism
2.1.1.22
Carnosine
6.3.2.11
1.1.1.23
4-(β-Acetylaminoethyl)-imidazole
3.4.13.18
3.4.13.20
γ-L-Glutamyl-S-(hercyn-2-yl)-L-cysteine S-oxide
S-(Hercyn-2-yl)-L-cysteine S-oxide
2.3.1.-
N-Formyl-L-aspartate
Imidazolone acetate
Imidazole-4-acetate
Imidazole acetaldehyde
L-Histidine
Hercynine
Ergothioneine
3.5.3.5
3.5.2.-
1.14.13.5
1.2.1.3
1.4.3.22
4.1.1.22
2.1.1.44
1.14.99.50
3.5.1.118
4.4.1.36
N-Formimino-L-aspartate
Histamine
1.14.99.51
3.5.1.15
3.5.1.8
6.3.4.8
2.1.1.8
4.3.1.3
Aspartate
1-(5-Phosphoribosyl)-imidazole-4-acetate
N-Methylhistamine
2.6.1.38
Urocanate
1.3.99.33
Dihydrourocanate
Thiourocanic acid
1.4.3.4
4.2.1.49
Alanine, aspartate and glutamate metabolism
Hydantoin-5-propionate
4-Imidazolone-5-propanoate
4-Oxoglutaramate
2-Oxoglutarate
(1-Ribosylimidazole)-4-acetate
Methylimidazole acetaldehyde
1.14.13.-
Formylisoglutamine
Isoglutamine
1.2.1.5
3.5.2.7
Methylimidazole-acetic acid
N-Formimino-L-glutamate
3.5.3.13
N-Formyl-L-glutamate
Imidazole-pyruvate
2.1.2.5
3.5.1.68
3.5.3.8
L-Glutamate
Alanine, aspartate and glutamate metabolism
Imidazole-lactate
N-Carbamyl-L-glutamate
00340 12/7/17
(c) Kanehisa Laboratories

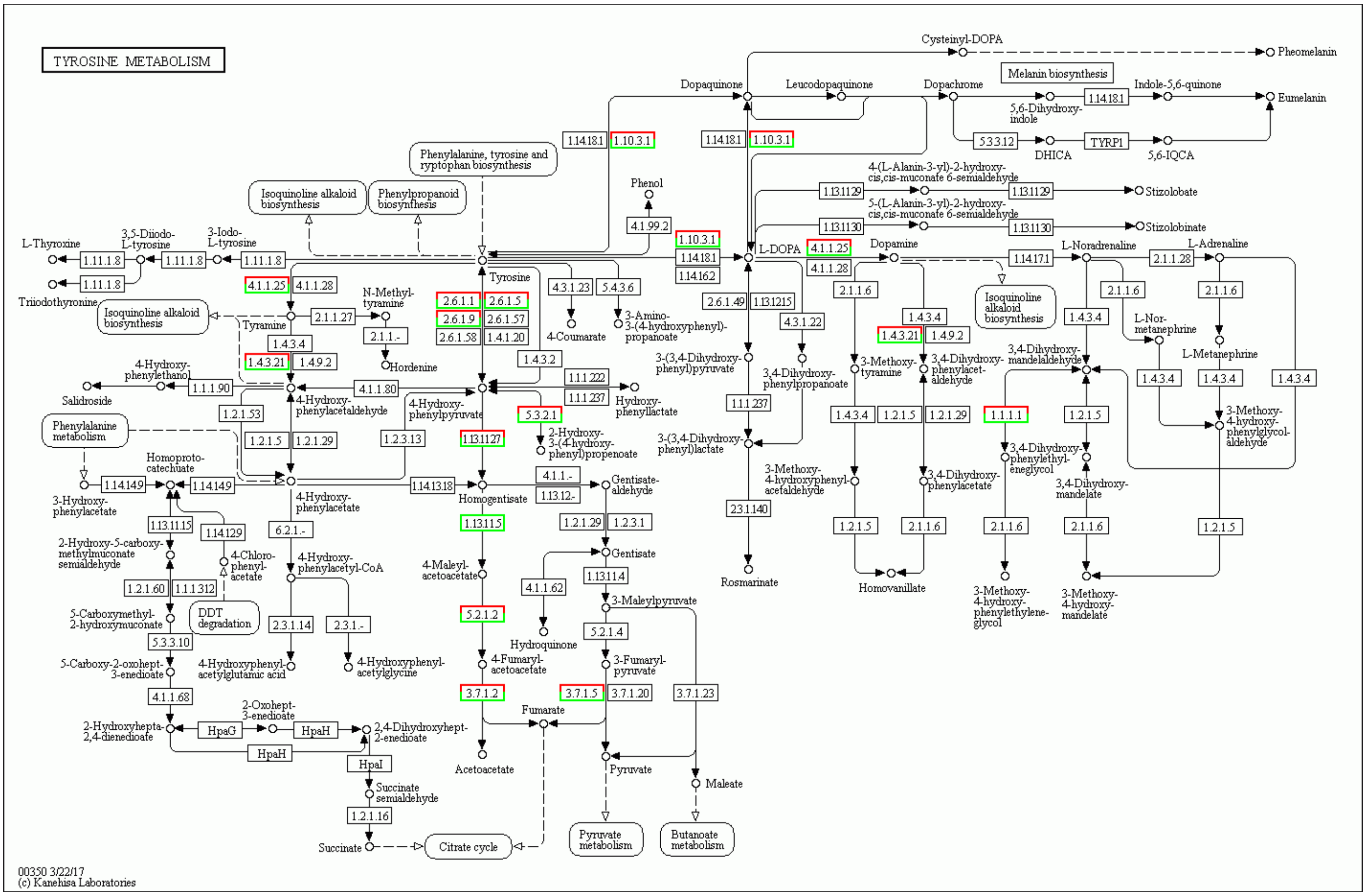
TYROSINE METABOLISM
Cysteinyl-DOPA
Pheomelanin
Melanin biosynthesis
Dopaquinone
Leucodopaquinone
Dopachrome
1.14.18.1
Indole-5,6-quinone
Eumelanin
5,6-Dihydroxy-indole
5.3.3.12
DHICA
TYRP1
5,6-IQCA
1.14.18.1
1.10.3.1
1.14.18.1
1.10.3.1
Phenylalanine, tyrosine and tryptophan biosynthesis
Isoquinoline alkaloid biosynthesis
Phenylpropanoid biosynthesis
Phenol
4.1.99.2
1.10.3.1
1.14.18.1
1.14.16.2
4(L-Alanin-3-yl)-2-hydroxy-cis,cis-muconate 6-semialdehyde
1.13.11.29
1.13.11.29
Stizolobate
5-(L-Alanin-3-yl)-2-hydroxy-cis,cis-muconate 6-semialdehyde
1.13.11.30
1.13.11.30
Stizolobinate
L-DOPA
4.1.1.25
4.1.1.28
Dopamine
1.14.17.1
L-Noradrenaline
2.1.1.28
L-Adrenaline
L-Thyroxine
3,5-Diiodo-L-tyrosine
3-Iodo-L-tyrosine
1.11.1.8
1.11.1.8
1.11.1.8
1.11.1.8
Triiodothyronine
Tyrosine
4.1.1.25
4.1.1.28
N-Methyl-tyramine
2.6.1.1
2.6.1.5
2.6.1.9
2.6.1.57
2.6.1.58
1.4.1.20
4.3.1.23
5.4.3.6
2.6.1.49
1.13.12.15
Isoquinoline alkaloid biosynthesis
Tyramine
2.1.1.27
2.1.1.-
4-Coumarate
3-Amino-3-(4-hydroxyphenyl)-propanoate
4.3.1.22
2.1.1.6
1.4.3.4
Isoquinoline alkaloid biosynthesis
2.1.1.6
2.1.1.6
1.4.3.4
1.4.3.4
1.4.3.21
1.4.9.2
Hordenine
1.4.3.2
1.4.3.21
1.4.9.2
L-Nor-metanephrine
L-Metanephrine
4-Hydroxy-phenylethanol
1.1.1.222
1.1.1.237
3-(3,4-Dihydroxy-phenyl)pyruvate
3,4-Dihydroxy-phenylpropanoate
3-Methoxy-tyramine
3,4-Dihydroxy-phenylacet-aldehyde
3,4-Dihydroxy-mandelaldehyde
Salidroside
1.1.1.90
4.1.1.80
4-Hydroxy-phenylacetaldehyde
4-Hydroxy-phenylpyruvate
Hydroxy-phenyllactate
1.1.1.237
1.4.3.4
1.4.3.4
1.4.3.4
1.2.1.53
Phenylalanine metabolism
5.3.2.1
1.4.3.4
1.2.1.5
1.2.1.29
1.1.1.1
1.2.1.5
3-Methoxy-4-hydroxy-phenylglycol-aldehyde
1.2.1.5
1.2.1.29
1.2.3.13
1.13.11.27
2-Hydroxy-3-(4-hydroxy-phenyl)propanoate
3-(3,4-Dihydroxy-phenyl)lactate
3,4-Dihydroxy-phenylethyl-eneglycol
Homoproto-catechuate
3-Methoxy-4-hydroxyphenyl-acetaldehyde
3,4-Dihydroxy-phenylacetate
3,4-Dihydroxy-mandelate
1.14.14.9
1.14.14.9
1.14.13.18
4.1.1.-
Gentisate-aldehyde
3-Hydroxy-phenylacetate
4-Hydroxy-phenylacetate
Homogentisate
1.13.12.-
1.14.13.140
1.13.11.15
1.14.14.9
6.2.1.-
1.13.11.5
1.2.1.29
1.2.3.1
1.2.1.5
2.1.1.6
2.1.1.6
2.1.1.6
1.2.1.5
2-Hydroxy-5-carboxy-methylmuconate semialdehyde
4-Chloro-phenyl-acetate
4-Hydroxy-phenylacetyl-CoA
Gentisate
Rosmarinate
4-Maleyl-acetoacetate
4.1.1.62
1.13.11.4
Homovanillate
3-Methoxy-4-hydroxy-phenylethylene-glycol
3-Methoxy-4-hydroxy-mandelate
1.2.1.60
1.1.1.312
DDT degradation
3-Maleylpyruvate
5-Carboxymethyl-2-hydroxymuconate
5.2.1.2
2.3.1.14
2.3.1.-
Hydroquinone
5.2.1.4
5.3.3.10
5-Carboxy-2-oxohept-3-enedioate
4-Hydroxyphenyl-acetylglutamic acid
4-Hydroxyphenyl-acetylglycine
4-Fumaryl-acetoacetate
3-Fumaryl-pyruvate
4.1.1.68
3.7.1.2
3.7.1.5
3.7.1.20
3.7.1.23
2-Oxohept-3-enedioate
Fumarate
2-Hydroxyhepta-2,4-dienedioate
HpaG
HpaH
2,4-Dihydroxyhept-2-enedioate
HpaH
HpaI
Acetoacetate
Pyruvate
Maleate
Succinate semialdehyde
1.2.1.16
Succinate
Citrate cycle
Pyruvate metabolism
Butanoate metabolism
00350 3/22/17
(c) Kanehisa Laboratories

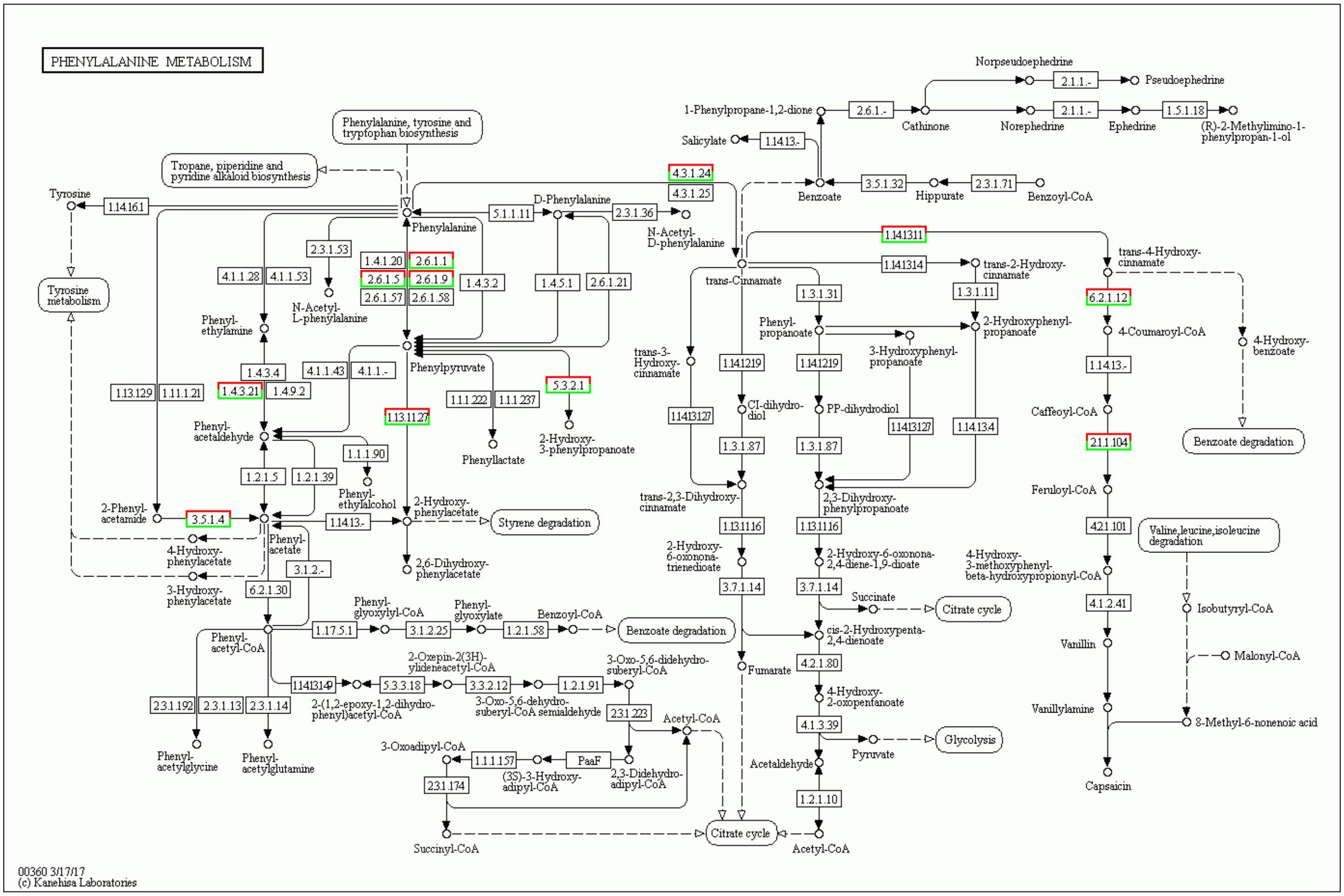
PHENYLALANINE METABOLISM
Norpseudoephedrine
2.1.1.-
Pseudoephedrine
1-Phenylpropane-1,2-dione
2.6.1.-
2.1.1.-
1.5.1.18
Cathinone
Norephedrine
Ephedrine
(R)-2-Methylimino-1-phenylpropan-1-ol
Phenylalanine, tyrosine and tryptophan biosynthesis
Salicylate
1.14.13.-
Tropane, piperidine and pyridine alkaloid biosynthesis
4.3.1.24
4.3.1.25
3.5.1.32
2.3.1.71
Benzoate
Hippurate
Benzoyl-CoA
Tyrosine
1.14.16.1
D-Phenylalanine
5.1.1.11
2.3.1.36
Phenylalanine
N-Acetyl-D-phenylalanine
1.14.13.11
trans-4-Hydroxy-cinnamate
1.14.13.14
trans-2-Hydroxy-cinnamate
trans-Cinnamate
2.3.1.53
1.4.1.20
2.6.1.1
Tyrosine metabolism
4.1.1.28
4.1.1.53
2.6.1.5
2.6.1.9
1.4.3.2
1.4.5.1
2.6.1.21
1.3.1.31
1.3.1.11
6.2.1.12
2.6.1.57
2.6.1.58
N-Acetyl-L-phenylalanine
Phenyl-ethylamine
Phenyl-propanoate
2-Hydroxyphenyl-propanoate
4-Coumaroyl-CoA
4-Hydroxy-benzoate
3-Hydroxyphenyl-propanoate
trans-3-Hydroxy-cinnamate
Phenylpyruvate
1.4.3.4
4.1.1.43
4.1.1.-
1.14.12.19
1.14.12.19
1.14.13.-
1.13.12.9
1.11.1.21
1.4.3.21
1.4.9.2
5.3.2.1
1.1.1.222
1.1.1.237
CI-dihydro-diol
PP-dihydrodiol
Caffeoyl-CoA
1.13.11.27
1.14.13.127
1.14.13.127
1.14.13.4
Phenyl-acetaldehyde
2-Hydroxy-3-phenylpropanoate
Phenyllactate
2.1.1.104
Benzoate degradation
1.1.1.90
1.3.1.87
1.3.1.87
1.2.1.5
1.2.1.39
Phenyl-ethylalcohol
2,3-Dihydroxy-phenylpropanoate
Feruloyl-CoA
trans-2,3-Dihydroxy-cinnamate
2-Phenyl-acetamide
3.5.1.4
1.14.13.-
2-Hydroxy-phenylacetate
Styrene degradation
1.13.11.16
1.13.11.16
4.2.1.101
Phenyl-acetate
Valine, leucine, isoleucine degradation
4-Hydroxy-phenylacetate
2-Hydroxy-6-oxonona-trienedioate
2-Hydroxy-6-oxonona-2,4-diene-1,9-dioate
4-Hydroxy-3-methoxyphenyl-beta-hydroxypropionyl-CoA
3.1.2.-
2,6-Dihydroxy-phenylacetate
3-Hydroxy-phenylacetate
6.2.1.30
3.7.1.14
3.7.1.14
4.1.2.41
Succinate
Citrate cycle
Isobutyryl-CoA
Phenyl-glyoxylyl-CoA
Phenyl-glyoxylate
Benzoyl-CoA
Phenyl-acetyl-CoA
1.17.5.1
3.1.2.25
1.2.1.58
Benzoate degradation
cis-2-Hydroxypenta-2,4-dienoate
Vanillin
Malonyl-CoA
2-Oxepin-2(3H)-ylideneacetyl-CoA
3-Oxo-5,6-didehydro-suberyl-CoA
Fumarate
4.2.1.80
1.14.13.149
5.3.3.18
3.3.2.12
1.2.1.91
4-Hydroxy-2-oxopentanoate
2.3.1.192
2.3.1.13
2.3.1.14
2-(1,2-epoxy-1,2-dihydro-phenyl)acetyl-CoA
3-Oxo-5,6-dehydro-suberyl-CoA semialdehyde
2.3.1.223
Acetyl-CoA
Vanillylamine
8-Methyl-6-nonenoic acid
4.1.3.39
Pyruvate
Glycolysis
3-Oxoadipyl-CoA
1.1.1.157
PaaF
Phenyl-acetylglycine
Phenyl-acetylglutamine
(3S)-3-Hydroxy-adipyl-CoA
2,3-Didehydro-adipyl-CoA
Acetaldehyde
2.3.1.174
Capsaicin
1.2.1.10
Succinyl-CoA
Citrate cycle
Acetyl-CoA
00360 3/17/17
(c) Kanehisa Laboratories

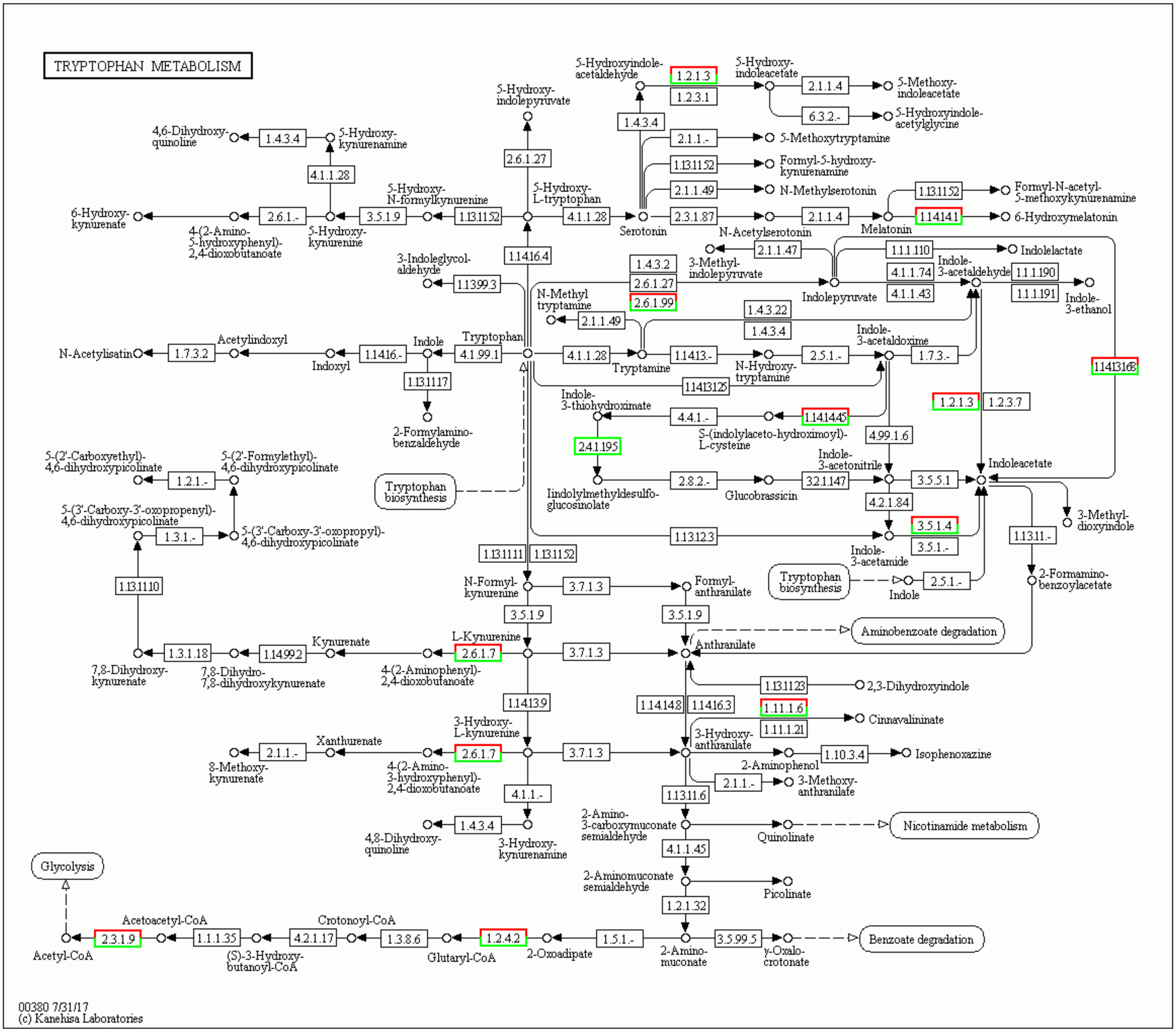

TRYPTOPHAN METABOLISM
5-Hydroxyindole-acetaldehyde
1.2.1.3
1.2.3.1
5-Hydroxy-indoleacetate
2.1.1.4
5-Methoxy-indoleacetate
6.3.2.-
5-Hydroxyindole-acetylglycine
5-Hydroxy-indolepyruvate
1.4.3.4
2.1.1.-
5-Methoxytryptamine
1.13.11.52
Formyl-5-hydroxy-kynurenamine
2.1.1.49
N-Methylserotonin
4,6-Dihydroxy-quinoline
1.4.3.4
5-Hydroxy-kynurenamine
2.6.1.27
4.1.1.28
5-Hydroxy-N-formylkynurenine
5-Hydroxy-L-tryptophan
1.13.11.52
Formyl-N-acetyl-5-methoxykynurenamine
6-Hydroxy-kynurenate
2.6.1.-
3.5.1.9
1.13.11.52
4.1.1.28
2.3.1.87
2.1.1.4
1.14.14.1
6-Hydroxymelatonin
4-(2-Amino-5-hydroxyphenyl)-2,4-dioxobutanoate
5-Hydroxy-kynurenine
Serotonin
N-Acetylserotonin
Melatonin
1.14.16.4
2.1.1.47
1.1.1.110
Indolelactate
3-Indoleglycol-aldehyde
1.4.3.2
3-Methyl-indolepyruvate
4.1.1.74
Indole-3-acetaldehyde
1.1.1.190
1.13.99.3
2.6.1.27
4.1.1.43
N-Methyl tryptamine
2.6.1.99
Indolepyruvate
1.1.1.191
Indole-3-ethanol
1.4.3.22
2.1.1.49
1.4.3.4
Acetylindoxyl
Indole
Tryptophan
Indole-3-acetaldoxime
N-Acetylisatin
1.7.3.2
1.14.16.-
4.1.99.1
4.1.1.28
1.14.13.-
2.5.1.-
1.7.3.-
1.14.13.168
Indoxyl
Tryptamine
N-Hydroxy-tryptamine
1.13.11.17
1.14.13.125
Indole-3-thiohydroximate
1.2.1.3
1.2.3.7
2-Formylamino-benzaldehyde
4.4.1.-
1.14.14.45
2.4.1.195
S-(indolylaceto-hydroximoyl)-L-cysteine
4.99.1.6
5-(2'-Carboxyethyl)-4,6-dihydroxypicolinate
5-(2'-Formylethyl)-4,6-dihydroxypicolinate
Indole-3-acetonitrile
Indoleacetate
1.2.1.-
Tryptophan biosynthesis
2.8.2.-
3.2.1.147
3.5.5.1
Indolylmethyldesulfo-glucosinolate
Glucobrassicin
4.2.1.84
3-Methyl-dioxyindole
5-(3'-Carboxy-3'-oxopropenyl)-4,6-dihydroxypicolinate
1.3.1.-
5-(3'-Carboxy-3'-oxopropyl)-4,6-dihydroxypicolinate
1.13.12.3
3.5.1.4
1.13.11.-
3.5.1.-
Indole-3-acetamide
1.13.11.11
1.13.11.52
1.13.11.10
N-Formyl-kynurenine
3.7.1.3
Formyl-anthranilate
Tryptophan biosynthesis
Indole
2.5.1.-
2-Formamino-benzoylacetate
3.5.1.9
3.5.1.9
Aminobenzoate degradation
Kynurenate
L-Kynurenine
Anthranilate
1.3.1.18
1.14.99.2
2.6.1.7
3.7.1.3
7,8-Dihydroxy-kynurenate
7,8-Dihydro-7,8-dihydroxykynurenate
4-(2-Aminophenyl)-2,4-dioxobutanoate
1.13.11.23
2,3-Dihydroxyindole
1.14.13.9
1.14.14.8
1.14.16.3
1.11.1.6
Cinnavalinate
3-Hydroxy-L-kynurenine
1.11.1.21
Xanthurenate
3-Hydroxy-anthranilate
2.1.1.-
2.6.1.7
3.7.1.3
2-Aminophenol
1.10.3.4
Isophenoxazine
8-Methoxy-kynurenate
4-(2-Amino-3-hydroxyphenyl)-2,4-dioxobutanoate
2.1.1.-
3-Methoxy-anthranilate
4.1.1.-
1.13.11.6
2-Amino-3-carboxymuconate semialdehyde
Quinolinate
Nicotinamide metabolism
4,8-Dihydroxy-quinoline
1.4.3.4
3-Hydroxy-kynurenamine
4.1.1.45
Glycolysis
2-Aminomuconate semialdehyde
Picolinate
1.2.1.32
Acetoacetyl-CoA
Crotonoyl-CoA
Acetyl-CoA
2.3.1.9
1.1.1.35
4.2.1.17
1.3.8.6
1.2.4.2
1.5.1.-
3.5.99.5
Benzoate degradation
(S)-3-Hydroxy-butanoyl-CoA
Glutaryl-CoA
2-Oxoadipate
2-Amino-muconate
γ-Oxalo-crotonate
00380 7/31/17
(c) Kanehisa Laboratories

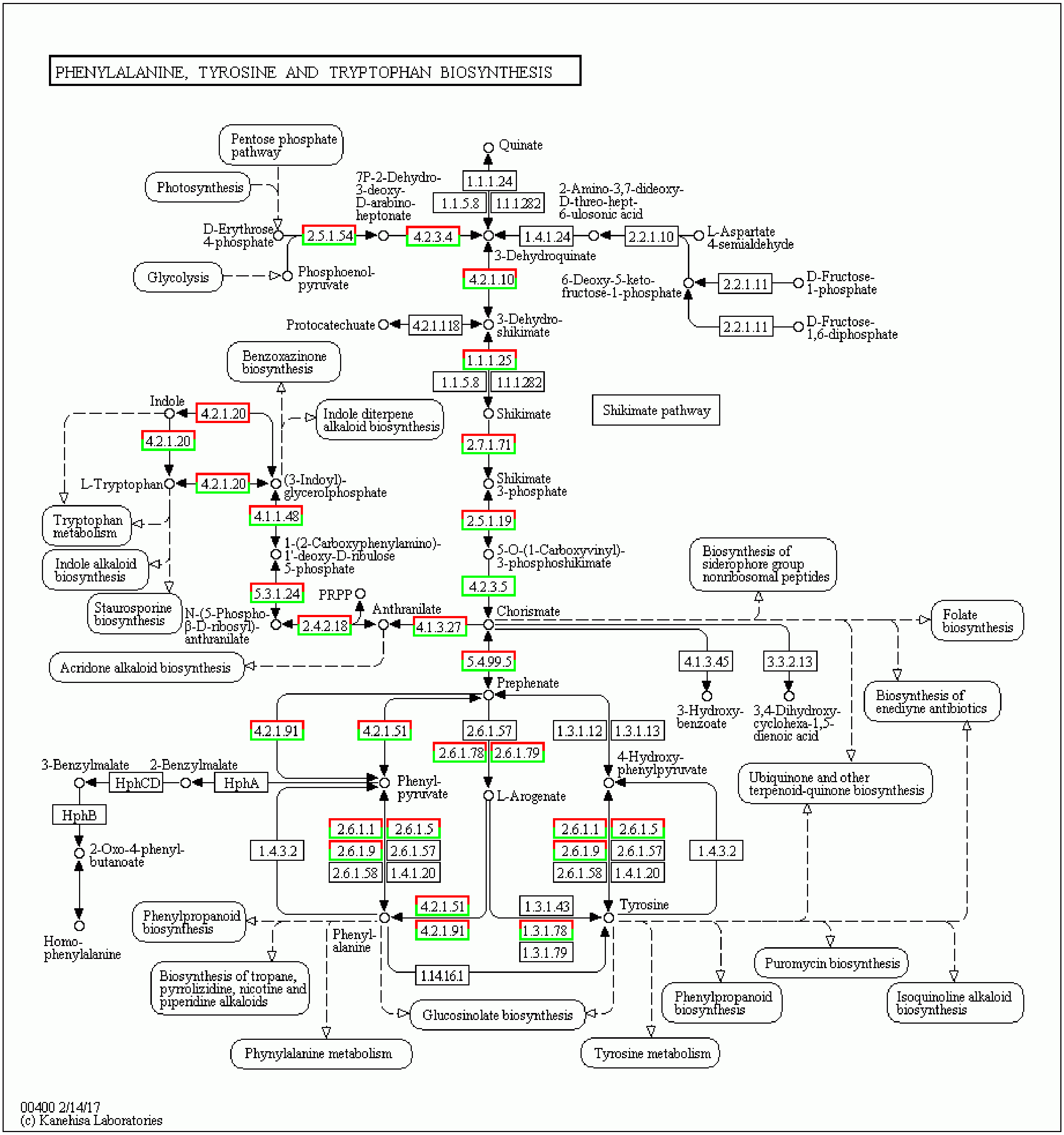
PHENYLALANINE, TYROSINE AND TRYPTOPHAN BIOSYNTHESIS
Pentose phosphate pathway
Photosynthesis
Glycolysis
D-Erythrose 4-phosphate
Phosphoenol-pyruvate
2.5.1.54
7P-2-Dehydro-3-deoxy-D-arabino-heptonate
4.2.3.4
Quinate
1.1.1.24
1.1.5.8
1.1.1.282
2-Amino-3,7-dideoxy-D-threo-hept-6-ulosonic acid
1.4.1.24
2.2.1.10
L-Aspartate 4-semialdehyde
3-Dehydroquinate
4.2.1.10
6-Deoxy-5-keto-fructose-1-phosphate
2.2.1.11
D-Fructose-1-phosphate
2.2.1.11
D-Fructose-1,6-diphosphate
Protocatechuate
4.2.1.118
3-Dehydro-shikimate
1.1.1.25
1.1.5.8
1.1.1.282
Shikimate
Shikimate pathway
2.7.1.71
Shikimate 3-phosphate
2.5.1.19
5-O-(1-Carboxyvinyl)-3-phosphoshikimate
4.2.3.5
Chorismate
Benzoxazinone biosynthesis
Indole
4.2.1.20
4.2.1.20
Indole diterpene alkaloid biosynthesis
L-Tryptophan
4.2.1.20
(3-Indoyl)-glycerolphosphate
Tryptophan metabolism
4.1.1.48
1-(2-Carboxyphenylamino)-1'-deoxy-D-ribulose 5-phosphate
Indole alkaloid biosynthesis
5.3.1.24
PRPP
Staurosporine biosynthesis
N-(5-Phospho-β-D-ribosyl)-anthranilate
2.4.2.18
Anthranilate
4.1.3.27
Acridone alkaloid biosynthesis
Biosynthesis of siderophore group nonribosomal peptides
Folate biosynthesis
4.1.3.45
3.3.2.13
5.4.99.5
Prephenate
3-Hydroxy-benzoate
3,4-Dihydroxy-cyclohexa-1,5-dienoic acid
Biosynthesis of enediyne antibiotics
4.2.1.91
4.2.1.51
2.6.1.57
2.6.1.78
2.6.1.79
1.3.1.12
1.3.1.13
3-Benzylmalate
2-Benzylmalate
HphCD
HphA
Phenyl-pyruvate
L-Arogenate
4-Hydroxy-phenylpyruvate
Ubiquinone and other terpenoid-quinone biosynthesis
HphB
2-Oxo-4-phenyl-butanoate
2.6.1.1
2.6.1.5
2.6.1.9
2.6.1.57
2.6.1.58
1.4.1.20
1.4.3.2
2.6.1.1
2.6.1.5
2.6.1.9
2.6.1.57
2.6.1.58
1.4.1.20
1.4.3.2
Homo-phenylalanine
Phenylpropanoid biosynthesis
4.2.1.51
4.2.1.91
1.3.1.43
1.3.1.78
1.3.1.79
Tyrosine
Phenyl-alanine
1.14.16.1
Biosynthesis of tropane, pyrrolizidine, nicotine and piperidine alkaloids
Glucosinolate biosynthesis
Puromycin biosynthesis
Phenylpropanoid biosynthesis
Isoquinoline alkaloid biosynthesis
Phynylalanine metabolism
Tyrosine metabolism
00400 2/14/17
(c) Kanehisa Laboratories

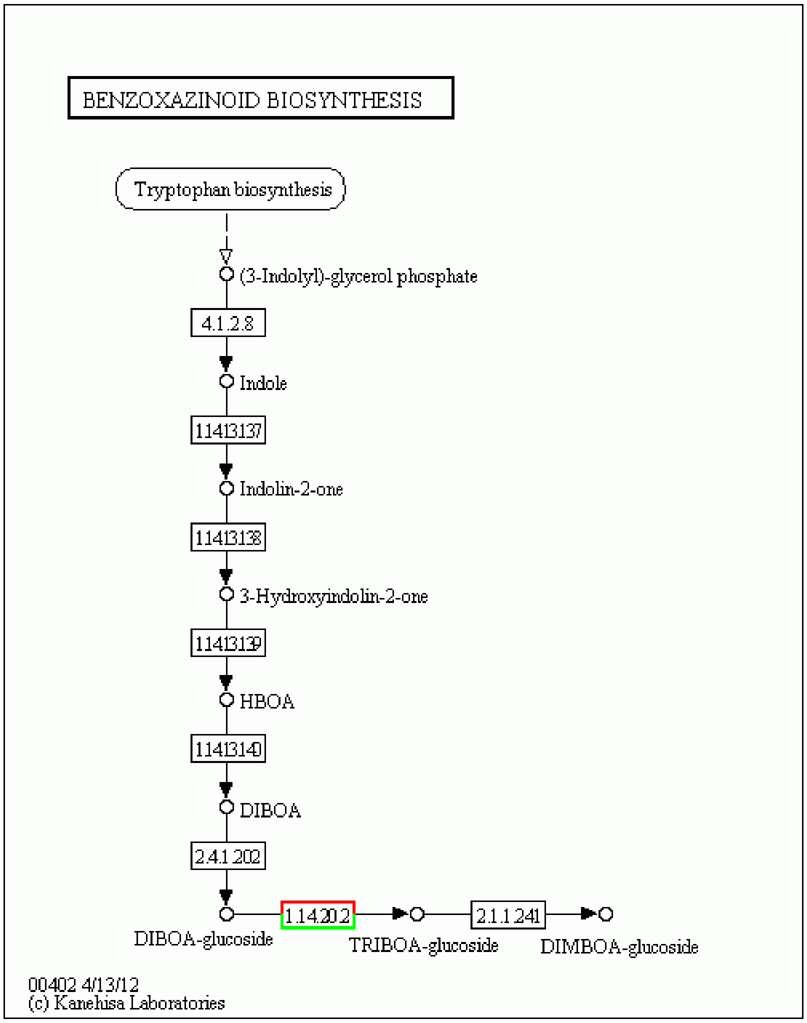

BENZOXAZINOID BIOSYNTHESIS
Tryptophan biosynthesis
(3-Indolyl)-glycerol phosphate
4.1.2.8
Indole
1.14.13.137
Indolin-2-one
1.14.13.138
3-Hydroxyindolin-2-one
1.14.13.139
HBOA
1.14.13.140
DIBOA
2.4.1.202
DIBOA-glucoside
1.14.20.2
TRIBOA-glucoside
2.1.1.241
DIMBOA-glucoside
00402 4/13/12
(c) Kanehisa Laboratories

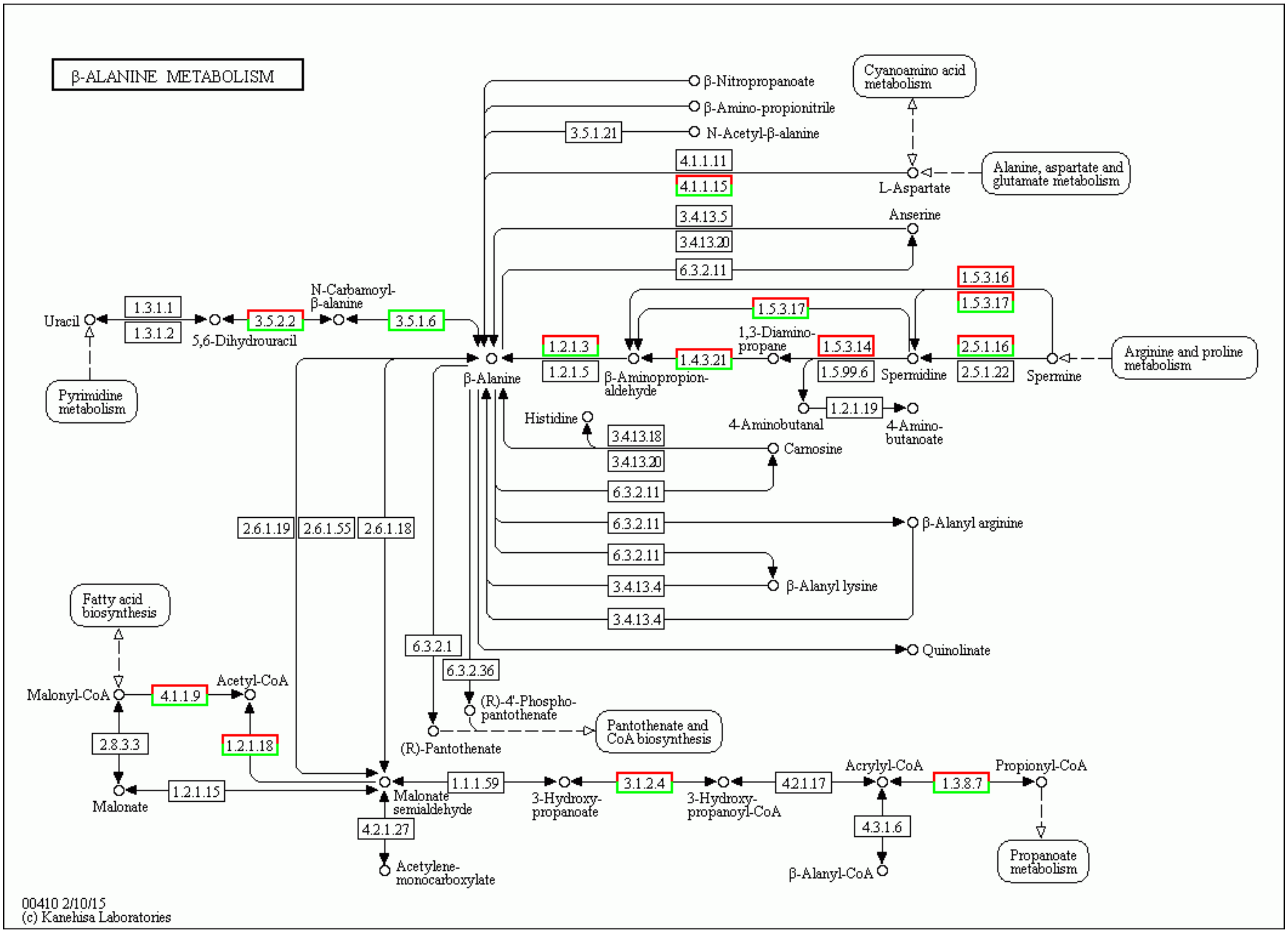
β-ALANINE METABOLISM
β-Nitropropanoate
β-Amino-propionitrile
N-Acetyl-β-alanine
Cyanoamino acid metabolism
L-Aspartate
Alanine, aspartate and glutamate metabolism
Anserine
Uracil
5,6-Dihydrouracil
N-Carbamoyl-β-alanine
Pyrimidine metabolism
β-Alanine
β-Aminopropion-aldehyde
1,3-Diamino-propane
Spermidine
Spermine
Arginine and proline metabolism
4-Aminobutanal
4-Amino-butanoate
Histidine
Carnosine
β-Alanyl arginine
β-Alanyl lysine
Quinolinate
Fatty acid biosynthesis
Malonyl-CoA
Acetyl-CoA
Malonate
(R)-4'-Phospho-pantothenate
(R)-Pantothenate
Pantothenate and CoA biosynthesis
Malonate semialdehyde
3-Hydroxy-propanoate
3-Hydroxy-propanoyl-CoA
Acrylyl-CoA
Propionyl-CoA
Propanoate metabolism
β-Alanyl-CoA
Acetylene-monocarboxylate
3.5.1.21
4.1.1.11
4.1.1.15
3.4.13.5
3.4.13.20
6.3.2.11
1.3.1.1
1.3.1.2
3.5.2.2
3.5.1.6
1.2.1.3
1.2.1.5
1.4.3.21
1.5.3.14
1.5.99.6
1.5.3.16
1.5.3.17
2.5.1.16
2.5.1.22
1.2.1.19
3.4.13.18
3.4.13.4
2.6.1.19
2.6.1.55
2.6.1.18
6.3.2.1
6.3.2.36
4.1.1.9
2.8.3.3
1.2.1.18
1.2.1.15
1.1.1.59
3.1.2.4
4.2.1.17
1.3.8.7
4.3.1.6
4.2.1.27
00410 2/10/15
(c) Kanehisa Laboratories

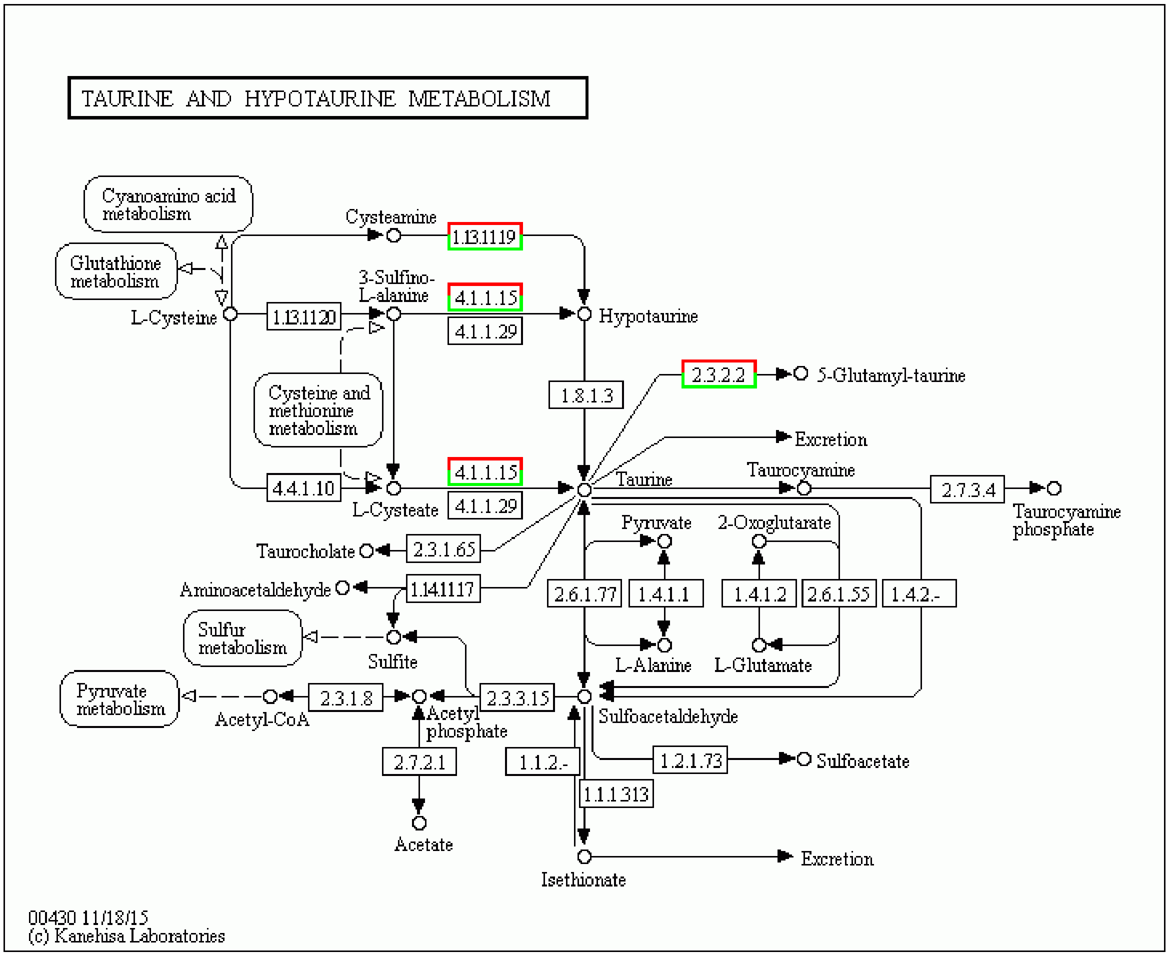
TAURINE AND HYPOTAURINE METABOLISM
Cyanoamino acid metabolism
Glutathione metabolism
Cysteamine
1.13.11.19
3-Sulfino-L-alanine
4.1.1.15
Hypotaurine
L-Cysteine
1.13.11.20
4.1.1.29
2.3.2.2
5-Glutamyl-taurine
1.8.1.3
Cysteine and methionine metabolism
Excretion
4.1.1.15
Taurocyamine
Taurine
4.4.1.10
2.7.3.4
Taurocyamine phosphate
L-Cysteate
4.1.1.29
Pyruvate
2-Oxoglutarate
Taurocholate
2.3.1.65
Aminoacetaldehyde
1.14.11.17
2.6.1.77
1.4.1.1
1.4.1.2
2.6.1.55
1.4.2.-
Sulfur metabolism
Sulfite
L-Alanine
L-Glutamate
Pyruvate metabolism
2.3.1.8
2.3.3.15
Acetyl-CoA
Acetyl phosphate
Sulfoacetaldehyde
2.7.2.1
1.1.2.-
1.2.1.73
Sulfoacetate
1.1.1.313
Acetate
Excretion
Isethionate
00430 11/18/15
(c) Kanehisa Laboratories

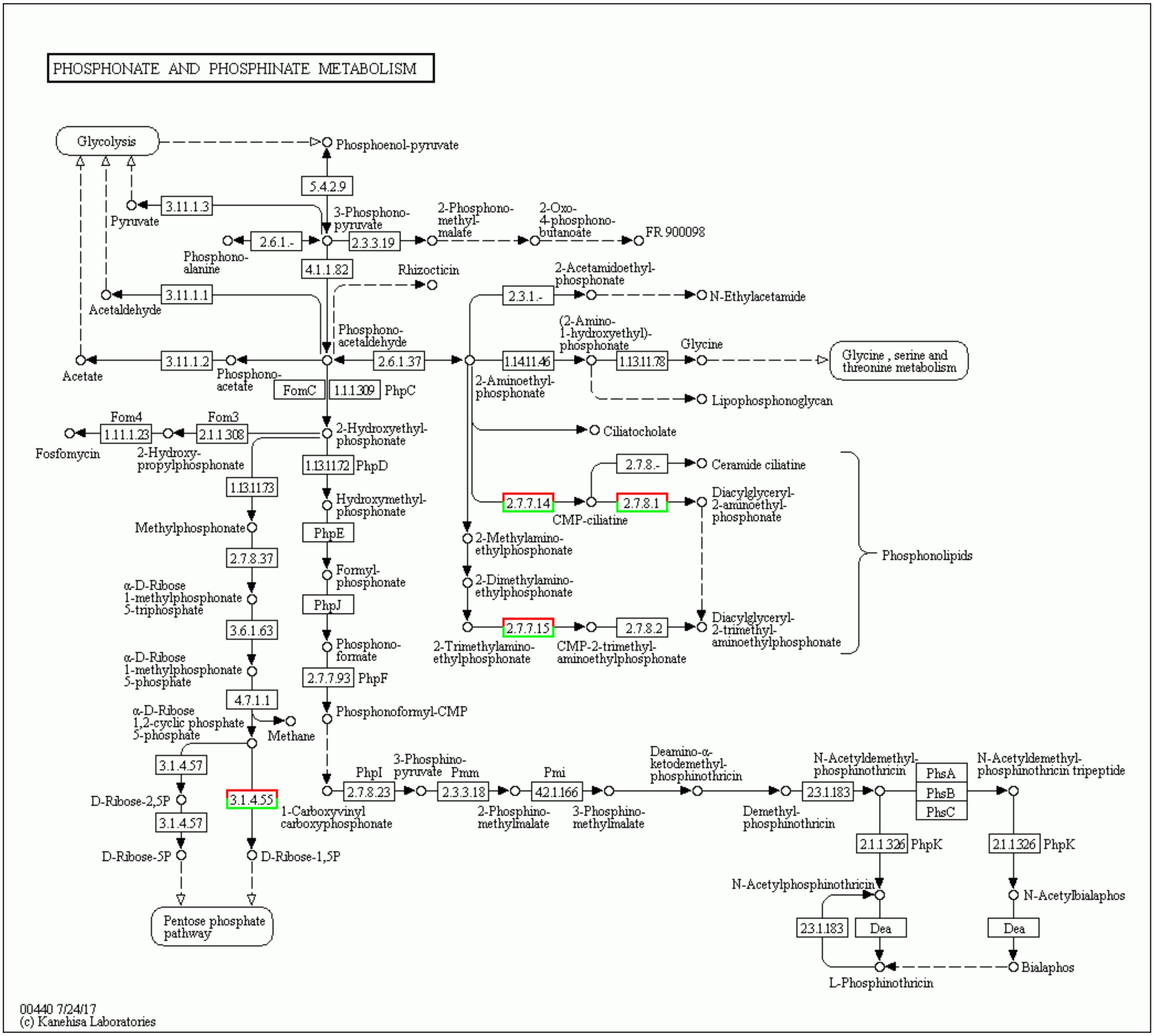
PHOSPHONATE AND PHOSPHINATE METABOLISM
Glycolysis
Phosphoenol-pyruvate
5.4.2.9
3.11.1.3
Pyruvate
3-Phosphono-pyruvate
2-Phosphono-methyl-malate
2-Oxo-4-phosphono-butanoate
FR 900098
2.6.1.-
2.3.3.19
Phosphono-alanine
4.1.1.82
Rhizocticin
3.11.1.1
Acetaldehyde
2-Acetamidoethyl-phosphonate
2.3.1.-
N-Ethylacetamide
Phosphono-acetaldehyde
(2-Amino-1-hydroxyethyl)-phosphonate
Glycine
Glycine, serine and threonine metabolism
3.11.1.2
2.6.1.37
1.14.11.46
1.13.11.78
Acetate
Phosphono-acetate
FomC
1.1.1.309
PhpC
2-Aminoethyl-phosphonate
Lipophosphonoglycan
Fom4
Fom3
1.11.1.23
2.1.1.308
2-Hydroxyethyl-phosphonate
Ciliatocholate
Fosfomycin
2-Hydroxy-propylphosphonate
1.13.11.72
PhpD
2.7.8.-
Ceramide ciliatine
1.13.11.73
Hydroxymethyl-phosphonate
2.7.7.14
2.7.8.1
Diacylglyceryl-2-aminoethyl-phosphonate
CMP-ciliatine
Methylphosphonate
PhpE
2-Methylamino-ethylphosphonate
Phosphonolipids
2.7.8.37
Formyl-phosphonate
α-D-Ribose 1-methylphosphonate 5-triphosphate
2-Dimethylamino-ethylphosphonate
PhpJ
Diacylglyceryl-2-trimethyl-aminoethylphosphonate
3.6.1.63
2.7.7.15
2.7.8.2
Phosphono-formate
2-Trimethylamino-ethylphosphonate
CMP-2-trimethyl-aminoethylphosphonate
α-D-Ribose 1-methylphosphonate 5-phosphate
2.7.7.93
PhpF
4.7.1.1
Phosphonoformyl-CMP
α-D-Ribose 1,2-cyclic phosphate 5-phosphate
Methane
Deamino-α-ketodemethyl-phosphinothricin
N-Acetyldemethyl-phosphinothricin
N-Acetyldemethyl-phosphinothricin tripeptide
3-Phosphino-pyruvate
PhpI
Pmm
Pmi
PhsA
PhsB
PhsC
3.1.4.57
2.7.8.23
2.3.3.18
4.2.1.166
2.3.1.183
D-Ribose-2,5P
3.1.4.55
1-Carboxyvinyl carboxyphosphonate
2-Phosphino-methylmalate
3-Phosphino-methylmalate
Demethyl-phosphinothricin
3.1.4.57
2.1.1.326
PhpK
2.1.1.326
PhpK
D-Ribose-5P
D-Ribose-1,5P
N-Acetylphosphinothricin
N-Acetylbialaphos
2.3.1.183
Dea
Dea
Pentose phosphate pathway
L-Phosphinothricin
Bialaphos
00440 7/24/17
(c) Kanehisa Laboratories

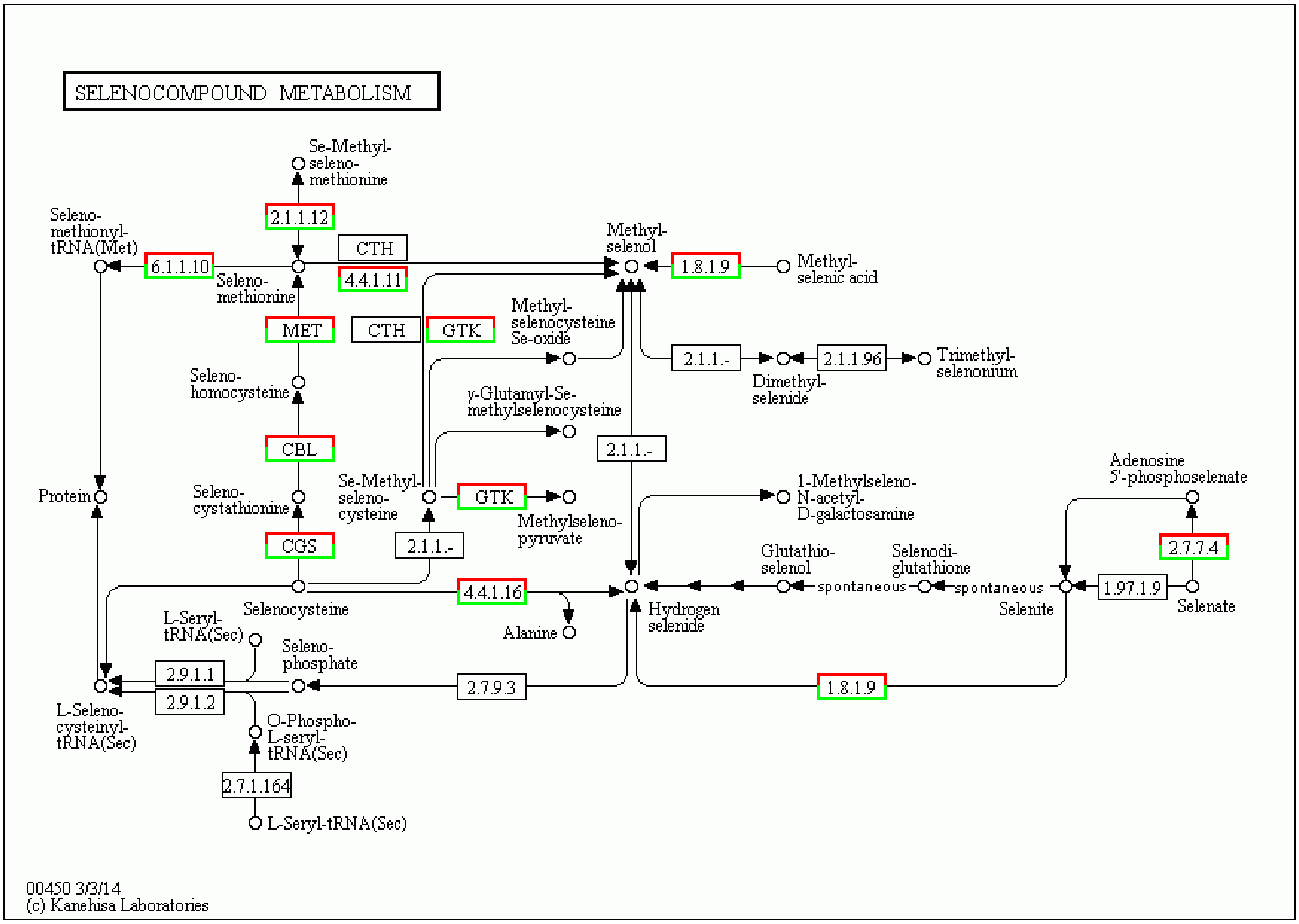
SELENOCOMPOUND METABOLISM
Se-Methyl-seleno-methionine
2.1.1.12
Seleno-methionyl-tRNA(Met)
6.1.1.10
Seleno-methionine
CTH
4.4.1.11
Methyl-selenol
1.8.1.9
Methyl-selenic acid
MET
CTH
GTK
Methyl-selenocysteine Se-oxide
2.1.1.-
Dimethyl-selenide
2.1.1.96
Trimethyl-selenonium
Seleno-homocysteine
γ-Glutamyl-Se-methylselenocysteine
CBL
2.1.1.-
Protein
Seleno-cystathionine
Se-Methyl-seleno-cysteine
GTK
Methylseleno-pyruvate
1-Methylseleno-N-acetyl-D-galactosamine
Adenosine 5'-phosphoselenate
CGS
2.1.1.-
2.7.7.4
Glutathio-selenol
Selenodi-glutathione
spontaneous
spontaneous
1.97.1.9
Selenocysteine
4.4.1.16
Hydrogen selenide
Selenite
Selenate
Alanine
L-Seryl-tRNA(Sec)
Seleno-phosphate
2.9.1.1
2.9.1.2
2.7.9.3
1.8.1.9
L-Seleno-cysteinyl-tRNA(Sec)
O-Phospho-L-seryl-tRNA(Sec)
2.7.1.164
L-Seryl-tRNA(Sec)
00450 3/3/14
(c) Kanehisa Laboratories

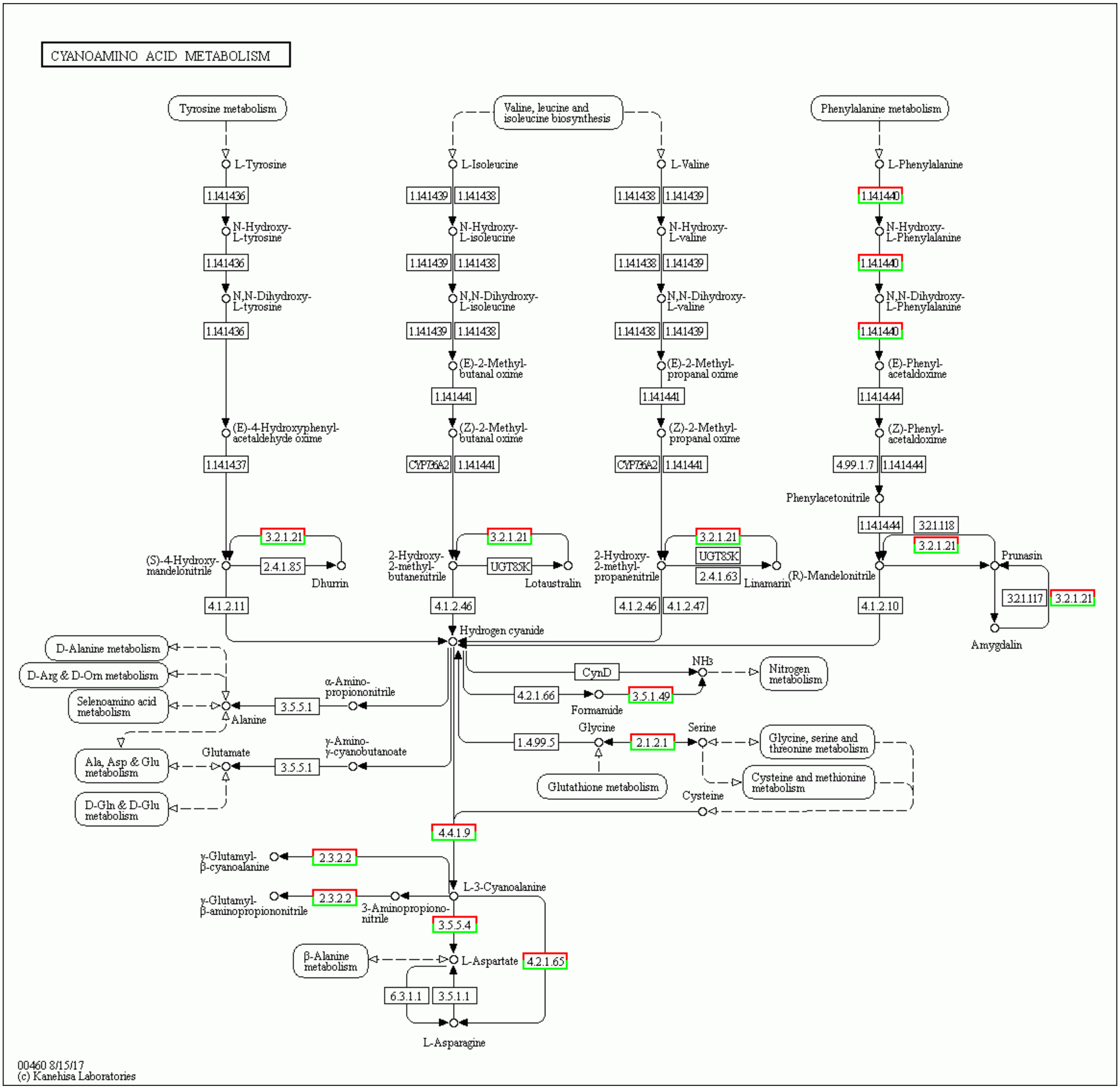
CYANOAMINO ACID METABOLISM
Tyrosine metabolism
Valine, leucine and isoleucine biosynthesis
Phenylalanine metabolism
L-Tyrosine
L-Isoleucine
L-Valine
L-Phenylalanine
1.14.14.36
1.14.14.39
1.14.14.38
1.14.14.40
N-Hydroxy-L-tyrosine
N-Hydroxy-L-isoleucine
N-Hydroxy-L-valine
N-Hydroxy-L-Phenylalanine
N,N-Dihydroxy-L-tyrosine
N,N-Dihydroxy-L-isoleucine
N,N-Dihydroxy-L-valine
N,N-Dihydroxy-L-Phenylalanine
(E)-2-Methyl-butanal oxime
(E)-2-Methyl-propanal oxime
(E)-Phenyl-acetaldoxime
1.14.14.41
1.14.14.44
(E)-4-Hydroxyphenyl-acetaldehyde oxime
(Z)-2-Methyl-butanal oxime
(Z)-2-Methyl-propanal oxime
(Z)-Phenyl-acetaldoxime
1.14.14.37
CYP736A2
4.99.1.7
Phenylacetonitrile
3.2.1.21
3.2.1.118
3.2.1.117
(S)-4-Hydroxy-mandelonitrile
2.4.1.85
Dhurrin
2-Hydroxy-2-methyl-butanenitrile
UGT85K
Lotaustralin
2-Hydroxy-2-methyl-propanenitrile
2.4.1.63
Linamarin
(R)-Mandelonitrile
Prunasin
Amygdalin
4.1.2.11
4.1.2.46
4.1.2.47
4.1.2.10
Hydrogen cyanide
D-Alanine metabolism
D-Arg & D-Orn metabolism
Selenoamino acid metabolism
Alanine
3.5.5.1
α-Amino-propiononitrile
CynD
NH3
Nitrogen metabolism
4.2.1.66
3.5.1.49
Formamide
Glycine
Serine
1.4.99.5
2.1.2.1
Glycine, serine and threonine metabolism
Cysteine and methionine metabolism
Glutathione metabolism
Cysteine
Ala, Asp & Glu metabolism
Glutamate
γ-Amino-γ-cyanobutanoate
D-Gln & D-Glu metabolism
4.4.1.9
γ-Glutamyl-β-cyanoalanine
2.3.2.2
L-3-Cyanoalanine
γ-Glutamyl-β-aminopropiononitrile
3-Aminopropiono-nitrile
3.5.5.4
β-Alanine metabolism
L-Aspartate
4.2.1.65
6.3.1.1
3.5.1.1
L-Asparagine
00460 8/15/17
(c) Kanehisa Laboratories

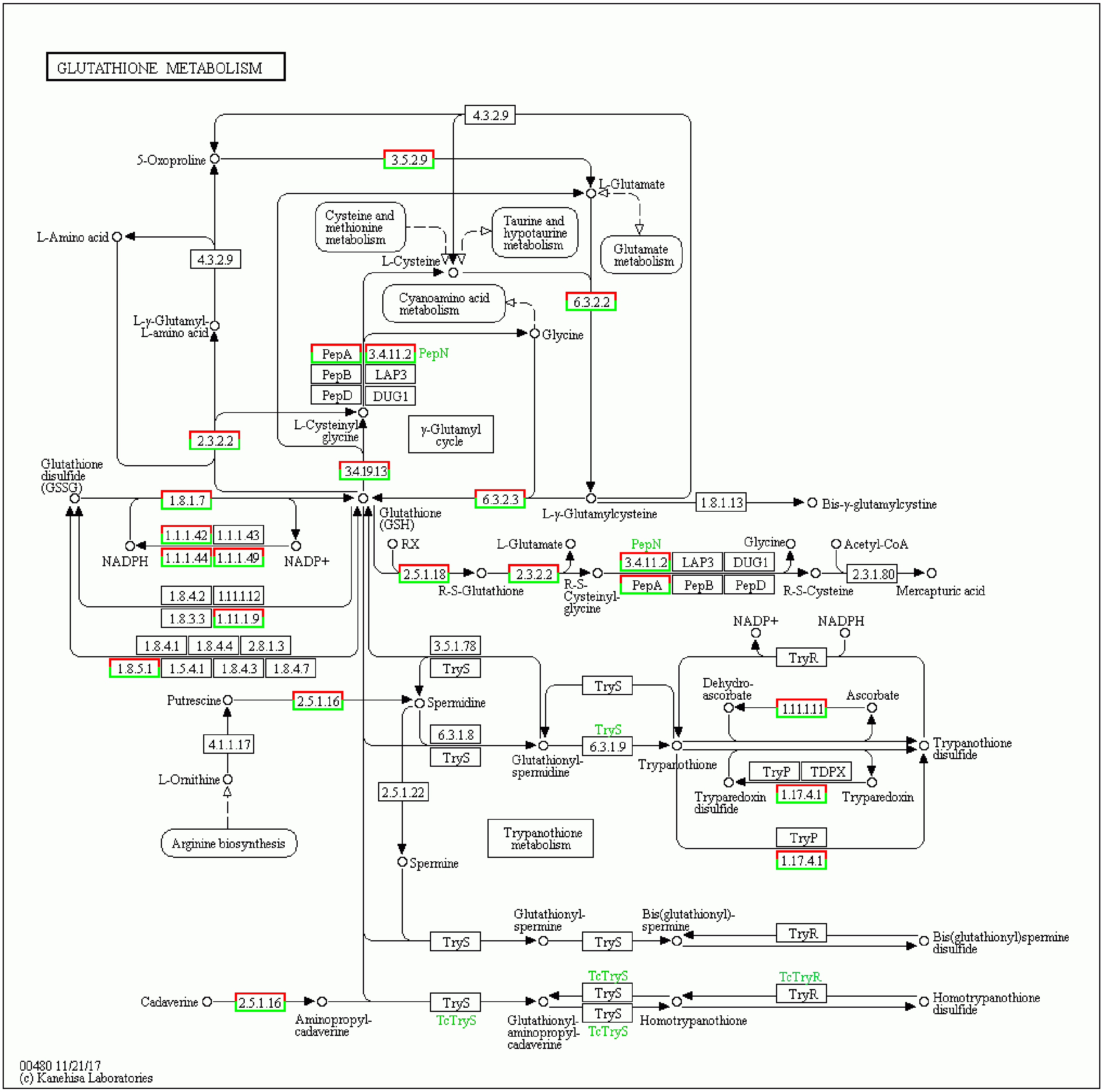
GLUTATHIONE METABOLISM
4.3.2.9
5-Oxoproline
3.5.2.9
L-Glutamate
Cysteine and methionine metabolism
Taurine and hypotaurine metabolism
Glutamate metabolism
L-Amino acid
4.3.2.9
L-Cysteine
Cyanoamino acid metabolism
6.3.2.2
L-γ-Glutamyl-L-amino acid
Glycine
PepA
3.4.11.2
PepN
PepB
LAP3
PepD
DUG1
L-Cysteinyl-glycine
γ-Glutamyl cycle
2.3.2.2
Glutathione disulfide (GSSG)
3.4.19.13
6.3.2.3
1.8.1.7
Glutathione (GSH)
L-γ-Glutamylcysteine
1.8.1.13
Bis-γ-glutamylcystine
1.1.1.42
1.1.1.43
NADPH
NADP+
1.1.1.44
1.1.1.49
RX
L-Glutamate
Glycine
Acetyl-CoA
PepN
3.4.11.2
LAP3
DUG1
2.5.1.18
2.3.2.2
PepA
PepB
PepD
2.3.1.80
R-S-Glutathione
R-S-Cysteinyl-glycine
R-S-Cysteine
Mercapturic acid
1.8.4.2
1.11.1.12
1.8.3.3
1.11.1.9
NADP+
NADPH
1.8.4.1
1.8.4.4
2.8.1.3
3.5.1.78
TryR
1.8.5.1
1.5.4.1
1.8.4.3
1.8.4.7
TryS
TryS
Dehydro-ascorbate
Ascorbate
Putrescine
2.5.1.16
Spermidine
1.11.1.11
TryS
6.3.1.8
4.1.1.17
6.3.1.9
Trypanothione disulfide
TryS
Glutathionyl-spermidine
Trypanothione
L-Ornithine
TryP
TDPX
2.5.1.22
1.17.4.1
Trypanoredoxin disulfide
Trypanoredoxin
Arginine biosynthesis
Trypanothione metabolism
TryP
1.17.4.1
Spermine
Glutathionyl-spermine
Bis(glutathionyl)-spermine
TryS
TryS
TryR
Bis(glutathionyl)spermine disulfide
TcTryS
TcTryR
Cadaverine
2.5.1.16
TryS
TryS
TryR
Homotrypanothione disulfide
TryS
Aminopropyl-cadaverine
TcTryS
Glutathionyl-aminopropyl-cadaverine
TcTryS
Homotrypanothione
00480 11/21/17
(c) Kanehisa Laboratories

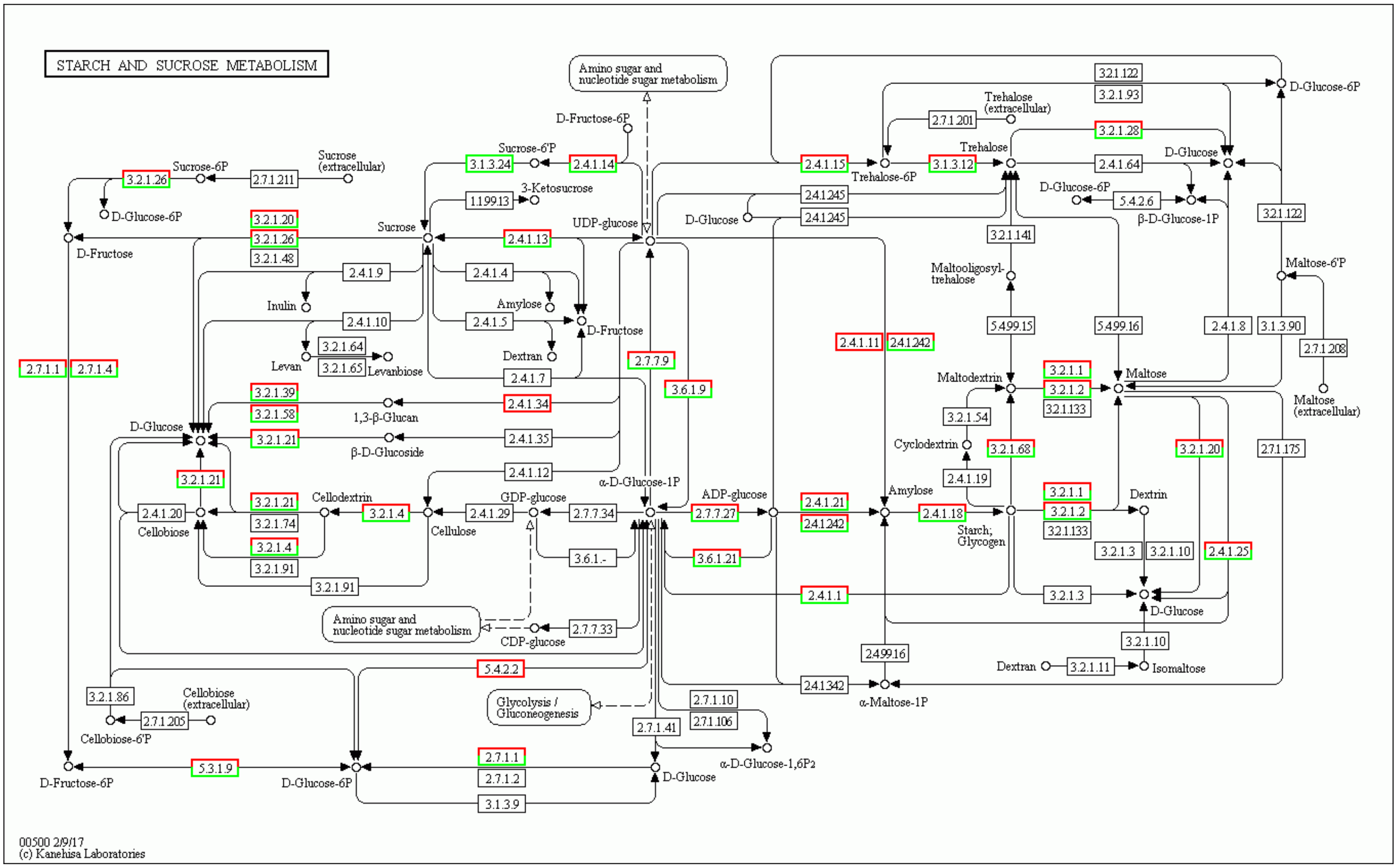
STARCH AND SUCROSE METABOLISM
Amino sugar and nucleotide sugar metabolism
D-Fructose-6P
Sucrose-6'P
Sucrose-6P
Sucrose (extracellular)
3-Ketosucrose
Sucrose
UDP-glucose
D-Glucose-6P
D-Fructose
Inulin
Amylose
Levan
Levanbiose
Dextran
1,3-β-Glucan
β-D-Glucoside
D-Glucose
Cellodextrin
GDP-glucose
Cellulose
Cellobiose
α-D-Glucose-1P
ADP-glucose
CDP-glucose
Glycolysis / Gluconeogenesis
Cellobiose (extracellular)
Cellobiose-6'P
D-Fructose-6P
D-Glucose-6P
D-Glucose
α-D-Glucose-1,6P2
Trehalose (extracellular)
Trehalose
Trehalose-6P
D-Glucose-6P
β-D-Glucose-1P
Maltooligosyl-trehalose
Maltose-6'P
Maltodextrin
Maltose
Maltose (extracellular)
Cyclodextrin
Amylose
Starch; Glycogen
Dextrin
D-Glucose
Dextran
Isomaltose
α-Maltose-1P
3.2.1.26
2.7.1.211
3.2.1.20
3.2.1.26
3.2.1.48
3.1.3.24
2.4.1.14
1.1.99.13
2.4.1.13
2.4.1.9
2.4.1.4
2.4.1.10
2.4.1.5
3.2.1.64
3.2.1.65
2.4.1.7
2.7.1.1
2.7.1.4
3.2.1.39
3.2.1.58
2.4.1.34
3.2.1.21
2.4.1.35
2.4.1.12
3.2.1.21
2.4.1.20
3.2.1.21
3.2.1.74
3.2.1.4
3.2.1.91
3.2.1.4
2.4.1.29
2.7.7.34
3.6.1.-
2.7.7.33
2.7.7.9
3.6.1.9
2.7.7.27
3.6.1.21
5.4.2.2
3.2.1.86
2.7.1.205
5.3.1.9
2.7.1.1
2.7.1.2
3.1.3.9
2.7.1.41
2.7.1.10
2.7.1.106
2.4.1.15
3.1.3.12
2.7.1.201
3.2.1.122
3.2.1.93
3.2.1.28
2.4.1.64
2.4.1.245
2.4.1.245
5.4.2.6
3.2.1.122
3.2.1.141
5.4.99.15
5.4.99.16
2.4.1.8
3.1.3.90
2.7.1.208
2.4.1.11
2.4.1.242
3.2.1.1
3.2.1.2
3.2.1.133
3.2.1.54
3.2.1.68
3.2.1.20
2.7.1.175
2.4.1.19
2.4.1.21
2.4.1.242
2.4.1.18
3.2.1.1
3.2.1.2
3.2.1.133
3.2.1.3
3.2.1.10
2.4.1.25
2.4.1.1
3.2.1.3
3.2.1.10
2.4.99.16
3.2.1.11
2.4.1.342
00500 2/9/17
(c) Kanehisa Laboratories

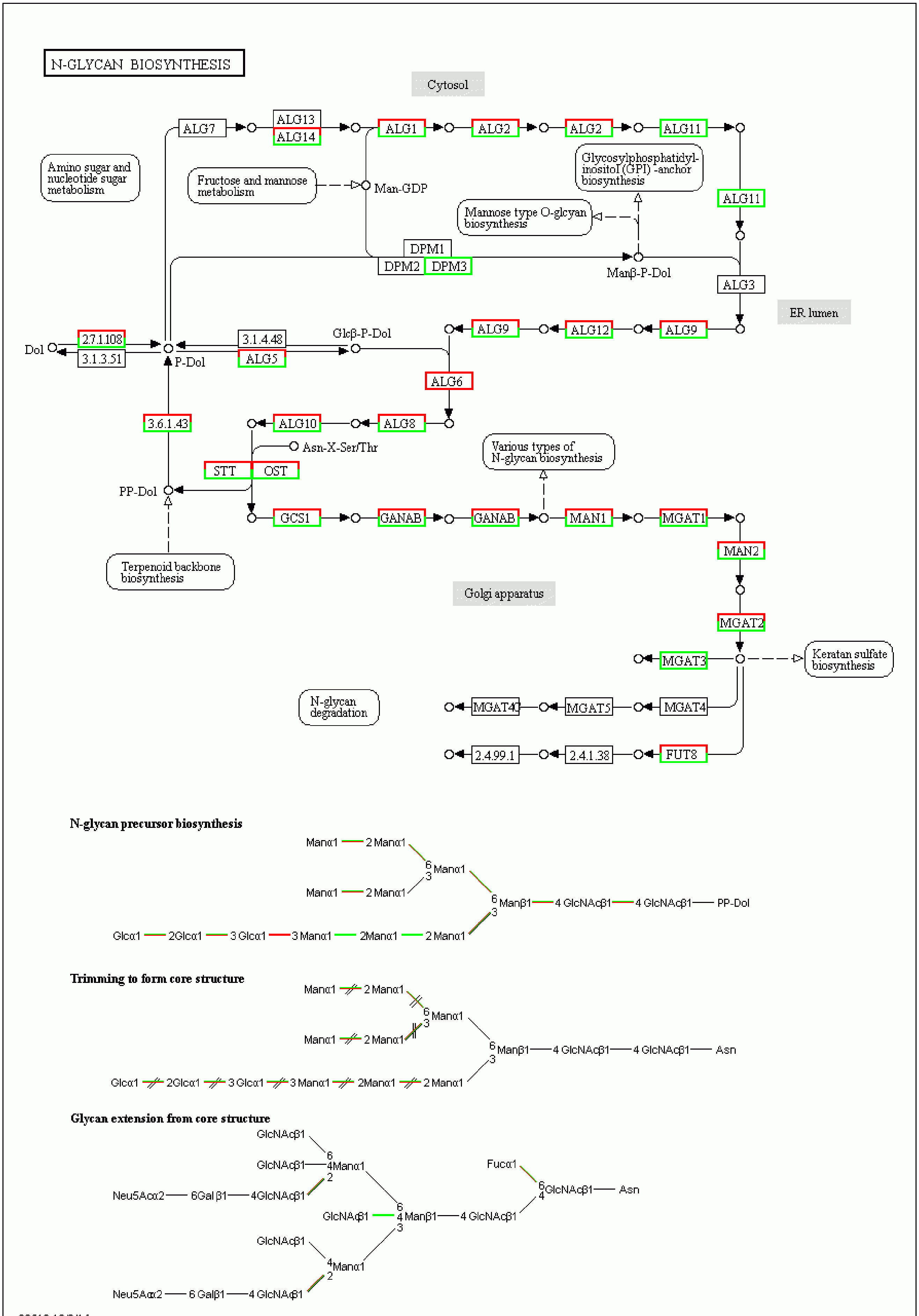

00510 12/8/16

(c) Kanehisa Laboratories

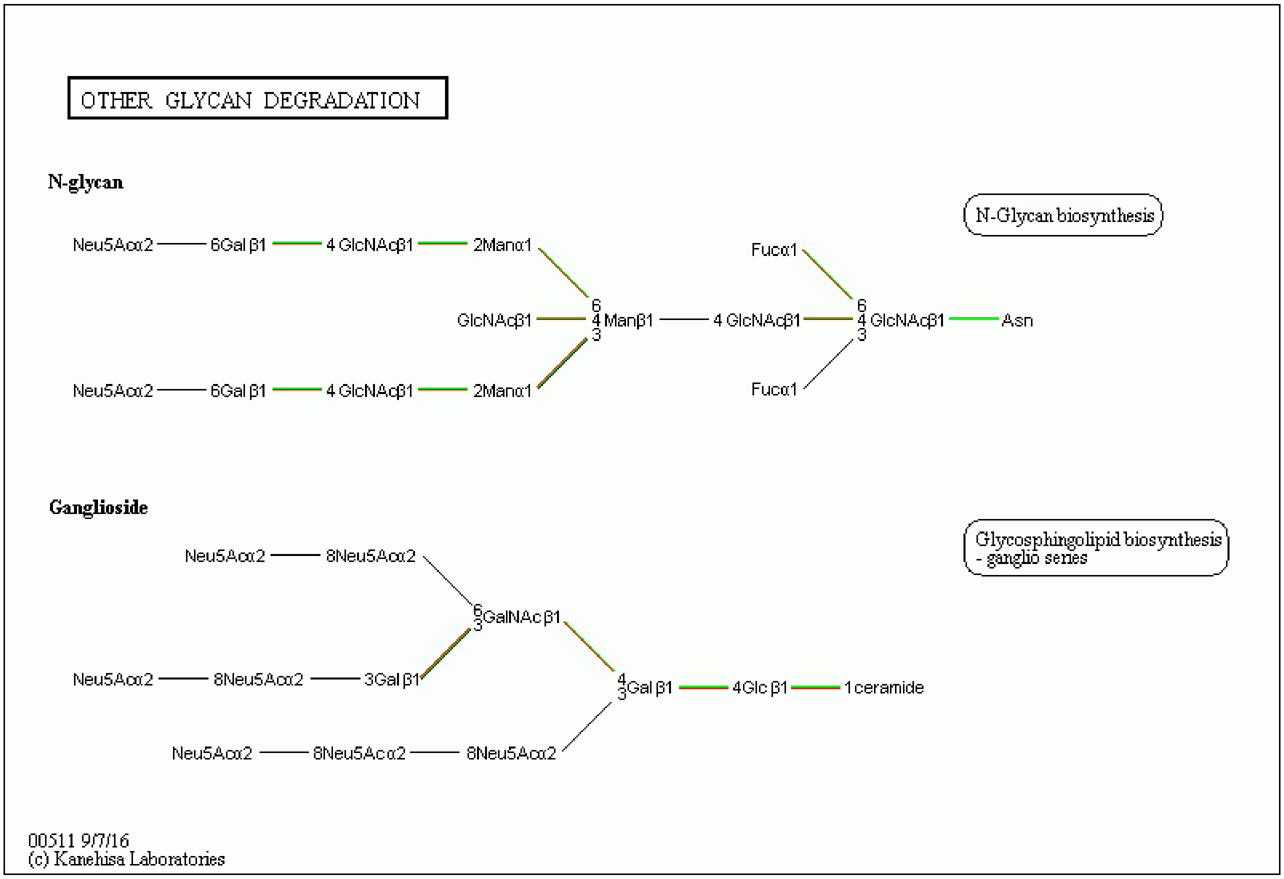
OTHER GLYCAN DEGRADATION
N-glycan
N-Glycan biosynthesis
Neu5Acα2 — 6Gal β1 — 4 GlcNAcβ1 — 2Manα1
GlcNAcβ1 — 6 4 3 Manβ1 — 4 GlcNAcβ1 — 6 4 3 GlcNAcβ1 — Asn
Neu5Acα2 — 6Gal β1 — 4 GlcNAcβ1 — 2Manα1
Fucα1
Fucα1
Ganglioside
Glycosphingolipid biosynthesis - ganglio series
Neu5Acα2 — 8Neu5Acα2
6 3 GalNAc β1
Neu5Acα2 — 8Neu5Acα2 — 3Gal β1
4 3 Gal β1 — 4Glc β1 — 1ceramide
Neu5Acα2 — 8Neu5Acα2 — 8Neu5Acα2
00511 9/7/16
(c) Kanehisa Laboratories

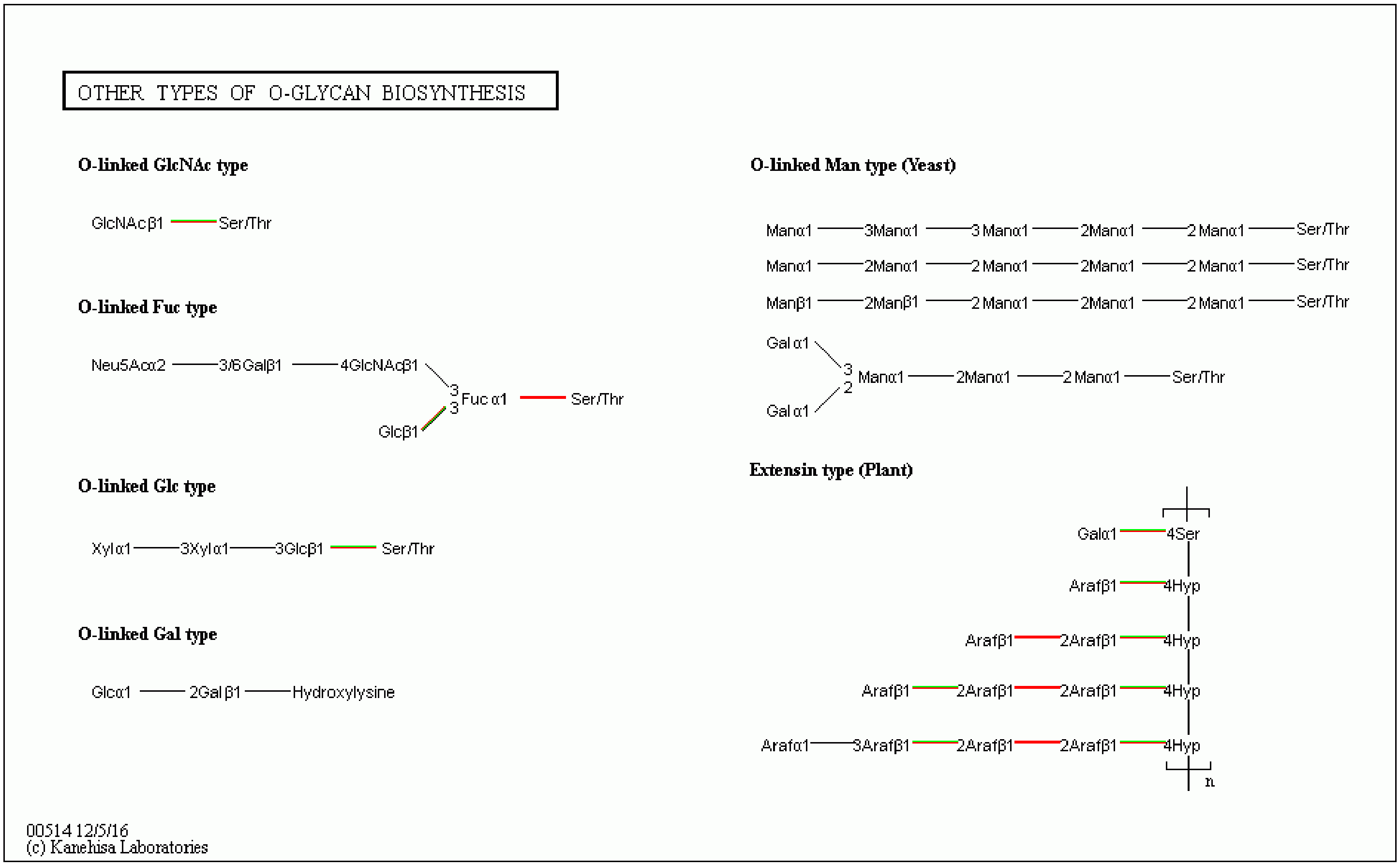
OTHER TYPES OF O-GLYCAN BIOSYNTHESIS
O-linked GlcNAc type
GlcNAcβ1 — Ser/Thr
O-linked Fuc type
Neu5Acα2 — 3/6Galβ1 — 4GlcNAcβ1
3 3 Fuc α1 — Ser/Thr
Glcβ1
O-linked Glc type
Xylα1 — 3Xylα1 — 3Glcβ1 — Ser/Thr
O-linked Gal type
Glcα1 — 2Gal β1 — Hydroxylysine
O-linked Man type (Yeast)
Manα1 — 3Manα1 — 3 Manα1 — 2Manα1 — 2 Manα1 — Ser/Thr
Manα1 — 2Manα1 — 2 Manα1 — 2Manα1 — 2 Manα1 — Ser/Thr
Manβ1 — 2Manβ1 — 2 Manα1 — 2Manα1 — 2 Manα1 — Ser/Thr
Gal α1
3 2 Manα1 — 2Manα1 — 2 Manα1 — Ser/Thr
Gal α1
Extensin type (Plant)
Galα1 — 4Ser
Arafβ1 — 4Hyp
Arafβ1 — 2Arafβ1 — 4Hyp
Arafβ1 — 2Arafβ1 — 2Arafβ1 — 4Hyp
Arafα1 — 3Arafβ1 — 2Arafβ1 — 2Arafβ1 — 4Hyp
n
00514 12/5/16
(c) Kanehisa Laboratories

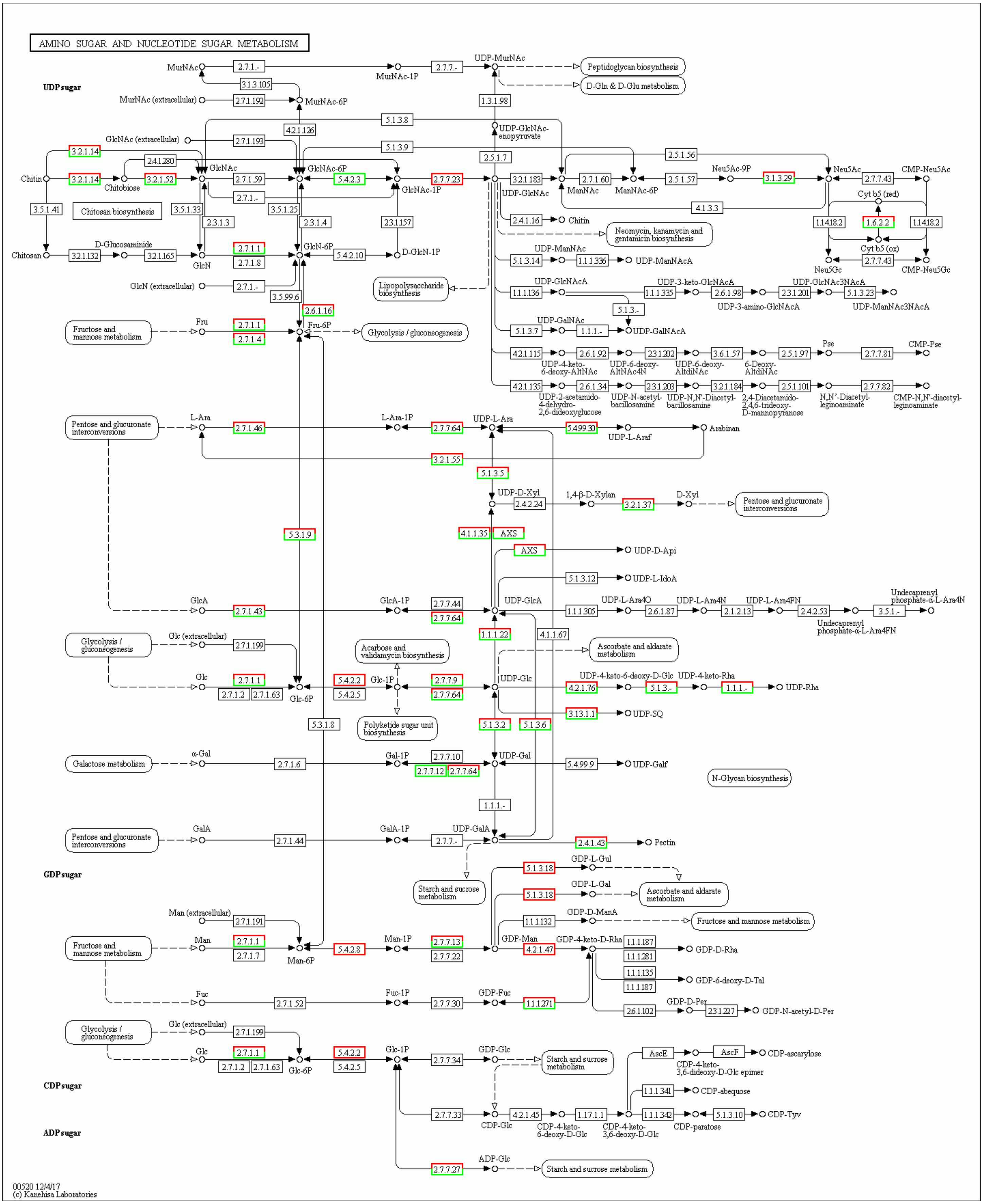
AMINO SUGAR AND NUCLEOTIDE SUGAR METABOLISM
UDP sugar
GDP sugar
CDP sugar
ADP sugar
MurNAc
2.7.1.-
3.1.3.105
MurNAc-1P
2.7.7.-
UDP-MurNAc
Peptidoglycan biosynthesis
D-Gln & D-Glu metabolism
MurNAc (extracellular)
2.7.1.192
MurNAc-6P
1.3.1.98
4.2.1.126
5.1.3.8
UDP-GlcNAc-enopyruvate
GlcNAc (extracellular)
2.7.1.193
3.2.1.14
2.4.1.280
5.1.3.9
2.5.1.7
2.5.1.56
Chitin
Chitobiose
3.2.1.52
GlcNAc
2.7.1.59
GlcNAc-6P
5.4.2.3
GlcNAc-1P
2.7.7.23
3.2.1.183
UDP-GlcNAc
2.7.1.60
ManNAc
ManNAc-6P
2.5.1.57
Neu5Ac-9P
3.1.3.29
Neu5Ac
2.7.7.43
CMP-Neu5Ac
Chitosan biosynthesis
3.5.1.41
3.5.1.33
2.3.1.3
3.5.1.25
2.3.1.4
2.3.1.157
4.1.3.3
Cyt b5 (red)
1.14.18.2
1.6.2.2
Cyt b5 (ox)
2.4.1.16
Chitin
Neomycin, kanamycin and gentamicin biosynthesis
Neu5Gc
CMP-Neu5Gc
D-Glucosaminide
Chitosan
3.2.1.132
3.2.1.165
GlcN
2.7.1.1
2.7.1.8
GlcN-6P
5.4.2.10
D-GlcN-1P
UDP-ManNAc
5.1.3.14
1.1.1.336
UDP-ManNAcA
Lipopolysaccharide biosynthesis
UDP-GlcNAcA
1.1.1.136
UDP-3-keto-GlcNAcA
1.1.1.335
2.6.1.98
UDP-GlcNAc3NAcA
2.3.1.201
5.1.3.23
UDP-3-amino-GlcNAcA
UDP-ManNAc3NAcA
GlcN (extracellular)
3.5.99.6
2.6.1.16
5.1.3.-
Fructose and mannose metabolism
Fru
2.7.1.4
Fru-6P
Glycolysis / gluconeogenesis
UDP-GalNAc
5.1.3.7
1.1.1.-
UDP-GalNAcA
4.2.1.115
2.6.1.92
2.3.1.202
3.6.1.57
2.5.1.97
Pse
2.7.7.81
CMP-Pse
UDP-4-keto-6-deoxy-AltNAc
UDP-6-deoxy-AltNAc4N
UDP-6-deoxy-AltdiNAc
6-Deoxy-AltdiNAc
4.2.1.135
2.6.1.34
2.3.1.203
3.2.1.184
2.5.1.101
2.7.7.82
UDP-2-acetamido-4-dehydro-2,6-dideoxyglucose
UDP-N-acetyl-bacillosamine
UDP-N,N'-Diacetyl-bacillosamine
2,4-Diacetamido-2,4,6-trideoxy-D-mannopyranose
N,N'-Diacetyl-legionaminate
CMP-N,N'-diacetyl-legionaminate
Pentose and glucuronate interconversions
L-Ara
2.7.1.46
L-Ara-1P
2.7.7.64
UDP-L-Ara
5.4.99.30
UDP-L-Araf
Arabinan
3.2.1.55
5.1.3.5
UDP-D-Xyl
2.4.2.24
1,4-β-D-Xylan
3.2.1.37
D-Xyl
Pentose and glucuronate interconversions
5.3.1.9
4.1.1.35
AXS
AXS
UDP-D-Api
5.1.3.12
UDP-L-IdoA
GlcA
2.7.1.43
GlcA-1P
2.7.7.44
2.7.7.64
UDP-GlcA
1.1.1.305
UDP-L-Ara4O
2.6.1.87
UDP-L-Ara4N
2.1.2.13
UDP-L-Ara4FN
2.4.2.53
Undecaprenyl phosphate-α-L-Ara4N
3.5.1.-
Undecaprenyl phosphate-α-L-Ara4FN
1.1.1.22
4.1.1.67
Glycolysis / gluconeogenesis
Glc (extracellular)
2.7.1.199
Acarbose and validamycin biosynthesis
Ascorbate and aldarate metabolism
Glc
2.7.1.1
2.7.1.2
2.7.1.63
Glc-6P
5.4.2.2
5.4.2.5
Glc-1P
2.7.7.9
2.7.7.64
UDP-Glc
UDP-4-keto-6-deoxy-D-Glc
4.2.1.76
5.1.3.-
UDP-4-keto-Rha
1.1.1.-
UDP-Rha
3.13.1.1
UDP-SQ
5.3.1.8
Polyketide sugar unit biosynthesis
5.1.3.2
5.1.3.6
Galactose metabolism
α-Gal
2.7.1.6
Gal-1P
2.7.7.10
2.7.7.12
2.7.7.64
UDP-Gal
5.4.99.9
UDP-Galf
N-Glycan biosynthesis
1.1.1.-
Pentose and glucuronate interconversions
GalA
2.7.1.44
GalA-1P
2.7.7.-
UDP-GalA
2.4.1.43
Pectin
GDP-L-Gul
5.1.3.18
Starch and sucrose metabolism
GDP-L-Gal
5.1.3.18
Ascorbate and aldarate metabolism
GDP-D-ManA
1.1.1.132
Fructose and mannose metabolism
Man (extracellular)
2.7.1.191
Fructose and mannose metabolism
Man
2.7.1.1
2.7.1.7
Man-6P
5.4.2.8
Man-1P
2.7.7.13
2.7.7.22
GDP-Man
4.2.1.47
GDP-4-keto-D-Rha
1.1.1.187
1.1.1.281
GDP-D-Rha
1.1.1.135
1.1.1.187
GDP-6-deoxy-D-Tal
Fuc
2.7.1.52
Fuc-1P
2.7.7.30
GDP-Fuc
1.1.1.271
2.6.1.102
GDP-D-Per
2.3.1.227
GDP-N-acetyl-D-Per
Glycolysis / gluconeogenesis
Glc (extracellular)
2.7.1.199
Glc
2.7.1.1
2.7.1.2
2.7.1.63
Glc-6P
5.4.2.2
5.4.2.5
Glc-1P
2.7.7.34
GDP-Glc
Starch and sucrose metabolism
AscE
AscF
CDP-ascarylose
CDP-4-keto-3,6-dideoxy-D-Glc epimer
1.1.1.341
CDP-abequose
2.7.7.33
CDP-Glc
4.2.1.45
CDP-4-keto-6-deoxy-D-Glc
1.17.1.1
CDP-4-keto-3,6-deoxy-D-Glc
1.1.1.342
CDP-paratose
5.1.3.10
CDP-Tyv
ADP-Glc
2.7.7.27
Starch and sucrose metabolism
00520 12/4/17
(c) Kanehisa Laboratories

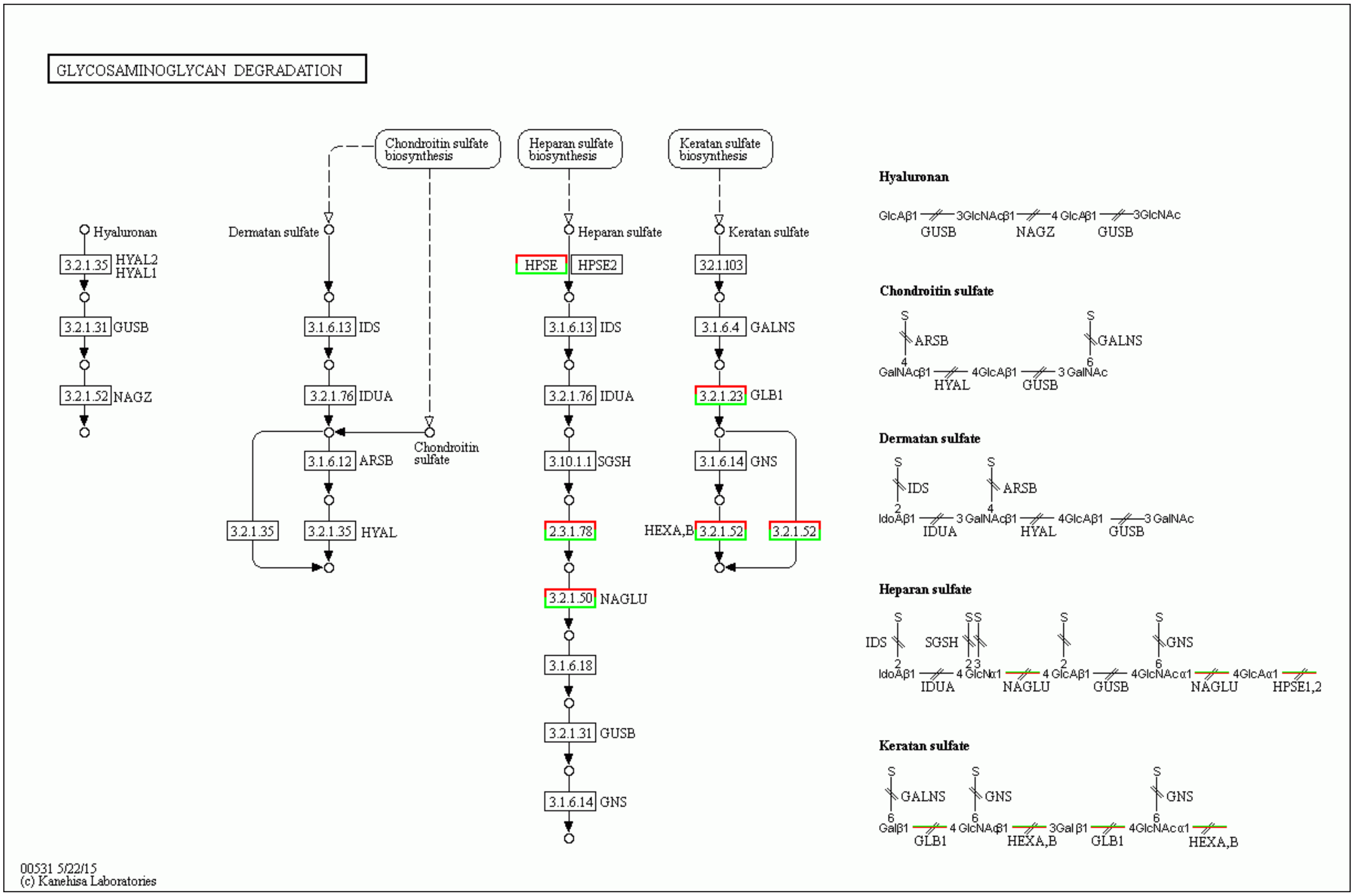

GLYCOSAMINOGLYCAN DEGRADATION
Chondroitin sulfate biosynthesis
Heparan sulfate biosynthesis
Keratan sulfate biosynthesis
Hyaluronan
3.2.1.35 HYAL2 HYAL1
3.2.1.31 GUSB
3.2.1.52 NAGZ
Dermatan sulfate
3.1.6.13 IDS
3.2.1.76 IDUA
Chondroitin sulfate
3.1.6.12 ARSB
3.2.1.35
3.2.1.35 HYAL
Heparan sulfate
HPSE
HPSE2
3.1.6.13 IDS
3.2.1.76 IDUA
3.10.1.1 SGSH
2.3.1.78
3.2.1.50 NAGLU
3.1.6.18
3.2.1.31 GUSB
3.1.6.14 GNS
Keratan sulfate
3.2.1.103
3.1.6.4 GALNS
3.2.1.23 GLB1
3.1.6.14 GNS
HEXA,B 3.2.1.52
3.2.1.52
Hyaluronan
GlcAβ1 3GlcNAcβ1 4 GlcAβ1 3GlcNAc
GUSB NAGZ GUSB
Chondroitin sulfate
ARSB GALNS
GalNAcβ1 4GlcAβ1 3 GalNAc
HYAL GUSB
Dermatan sulfate
IDS ARSB
IdoAβ1 3 GalNAcβ1 4GlcAβ1 3 GalNAc
IDUA HYAL GUSB
Heparan sulfate
IDS SGSH GNS
IdoAβ1 4 GlcNα1 4 GlcAβ1 4GlcNAcα1 4GlcAα1
IDUA NAGLU GUSB NAGLU HPSE1,2
Keratan sulfate
GALNS GNS GNS
Galβ1 4 GlcNAcβ1 3Galβ1 4GlcNAcα1
GLB1 HEXA,B GLB1 HEXA,B
00531 5/22/15
(c) Kanehisa Laboratories

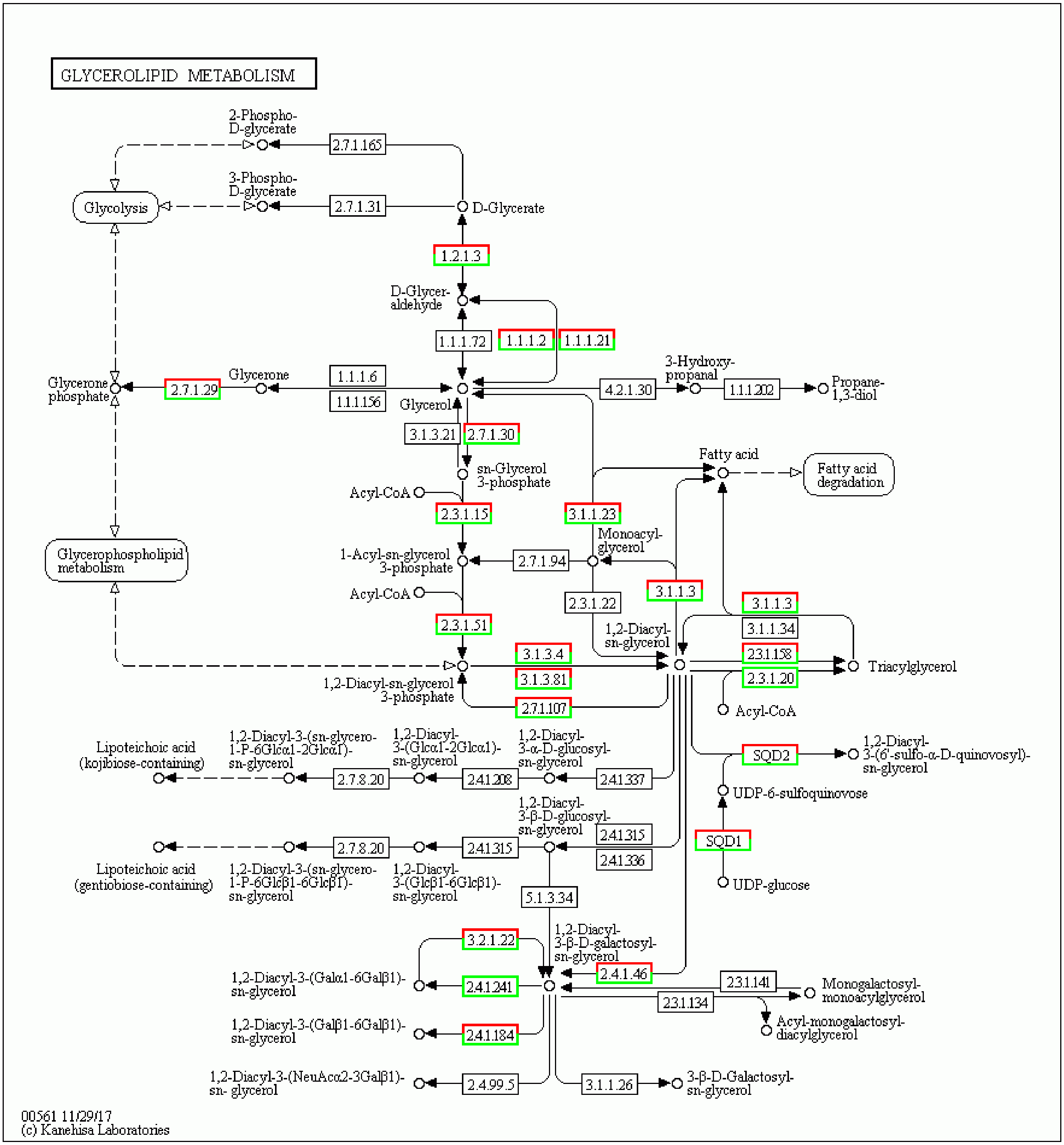
GLYCEROLIPID METABOLISM
2-Phospho-D-glycerate
2.7.1.165
3-Phospho-D-glycerate
2.7.1.31
Glycolysis
D-Glycerate
1.2.1.3
D-Glycer-aldehyde
1.1.1.72
1.1.1.2
1.1.1.21
Glycerone phosphate
2.7.1.29
Glycerone
1.1.1.6
1.1.1.156
Glycerol
4.2.1.30
3-Hydroxy-propanal
1.1.1.202
Propane-1,3-diol
3.1.3.21
2.7.1.30
sn-Glycerol 3-phosphate
Acyl-CoA
2.3.1.15
Fatty acid
Fatty acid degradation
3.1.1.23
Monoacyl-glycerol
Glycerophospholipid metabolism
1-Acyl-sn-glycerol 3-phosphate
2.7.1.94
Acyl-CoA
2.3.1.22
3.1.1.3
3.1.1.3
2.3.1.51
1,2-Diacyl-sn-glycerol
3.1.1.34
3.1.3.4
2.3.1.158
Triacylglycerol
3.1.3.81
2.3.1.20
1,2-Diacyl-sn-glycerol 3-phosphate
2.7.1.107
Acyl-CoA
Lipoteichoic acid (kojibiose-containing)
1,2-Diacyl-3-(sn-glycero-1-P-6Glcα1-2Glcα1)-sn-glycerol
2.7.8.20
1,2-Diacyl-3-(Glcα1-2Glcα1)-sn-glycerol
2.4.1.208
1,2-Diacyl-3-α-D-glucosyl-sn-glycerol
2.4.1.337
SQD2
1,2-Diacyl-3-(6'-sulfo-α-D-quinovosyl)-sn-glycerol
UDP-6-sulfoquinovose
1,2-Diacyl-3-β-D-glucosyl-sn-glycerol
2.4.1.315
2.7.8.20
2.4.1.315
2.4.1.336
SQD1
Lipoteichoic acid (gentiobiose-containing)
1,2-Diacyl-3-(sn-glycero-1-P-6Glcβ1-6Glcβ1)-sn-glycerol
1,2-Diacyl-3-(Glcβ1-6Glcβ1)-sn-glycerol
UDP-glucose
5.1.3.34
3.2.1.22
1,2-Diacyl-3-β-D-galactosyl-sn-glycerol
2.4.1.46
1,2-Diacyl-3-(Galα1-6Galβ1)-sn-glycerol
2.4.1.241
2.3.1.141
2.3.1.134
Monogalactosyl-monoacylglycerol
Acyl-monogalactosyl-diacylglycerol
1,2-Diacyl-3-(Galβ1-6Galβ1)-sn-glycerol
2.4.1.184
1,2-Diacyl-3-(NeuAcα2-3Galβ1)-sn- glycerol
2.4.99.5
3.1.1.26
3-β-D-Galactosyl-sn-glycerol
00561 11/29/17
(c) Kanehisa Laboratories

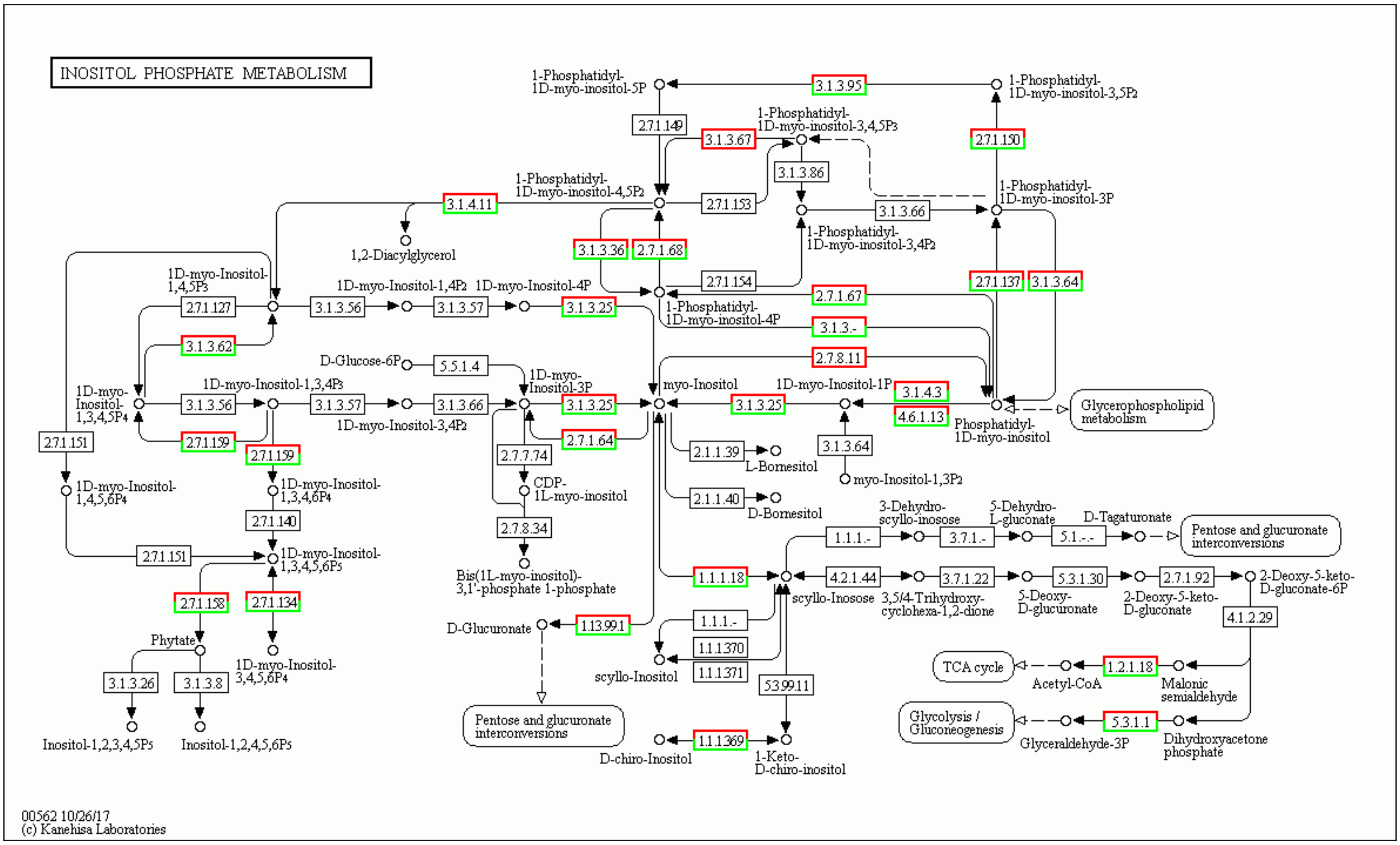
INOSITOL PHOSPHATE METABOLISM
1-Phosphatidyl-1D-myo-inositol-5P
1-Phosphatidyl-1D-myo-inositol-3,5P2
1-Phosphatidyl-1D-myo-inositol-3,4,5P3
1-Phosphatidyl-1D-myo-inositol-4,5P2
1-Phosphatidyl-1D-myo-inositol-3P
1-Phosphatidyl-1D-myo-inositol-3,4P2
1-Phosphatidyl-1D-myo-inositol-4P
1,2-Diacylglycerol
1D-myo-Inositol-1,4,5P3
1D-myo-Inositol-1,4P2
1D-myo-Inositol-4P
D-Glucose-6P
1D-myo-Inositol-3P
myo-Inositol
1D-myo-Inositol-1P
Phosphatidyl-1D-myo-inositol
Glycerophospholipid metabolism
1D-myo-Inositol-1,3,4,5P4
1D-myo-Inositol-1,3,4P3
1D-myo-Inositol-3,4P2
CDP-1L-myo-inositol
L-Bornesitol
D-Bornesitol
myo-Inositol-1,3P2
1D-myo-Inositol-1,4,5,6P4
1D-myo-Inositol-1,3,4,6P4
1D-myo-Inositol-1,3,4,5,6P5
Bis(1L-myo-inositol)-3,1'-phosphate 1-phosphate
3-Dehydro-scyllo-inosose
5-Dehydro-L-gluconate
D-Tagaturonate
Pentose and glucuronate interconversions
scyllo-Inosose
3,5/4-Trihydroxy-cyclohexa-1,2-dione
5-Deoxy-D-glucuronate
2-Deoxy-5-keto-D-gluconate
2-Deoxy-5-keto-D-gluconate-6P
D-Glucuronate
Phytate
1D-myo-Inositol-3,4,5,6P4
scyllo-Inositol
TCA cycle
Acetyl-CoA
Malonic semialdehyde
Glycolysis / Gluconeogenesis
Glyceraldehyde-3P
Dihydroxyacetone phosphate
Inositol-1,2,3,4,5P5
Inositol-1,2,4,5,6P5
D-chiro-Inositol
1-Keto-D-chiro-inositol
3.1.3.95
2.7.1.149
3.1.3.67
3.1.3.86
2.7.1.150
3.1.4.11
2.7.1.153
3.1.3.66
3.1.3.36
2.7.1.68
2.7.1.154
2.7.1.67
2.7.1.137
3.1.3.64
2.7.1.127
3.1.3.56
3.1.3.57
3.1.3.25
3.1.3.-
3.1.3.62
2.7.8.11
5.5.1.4
3.1.4.3
4.6.1.13
2.7.1.159
2.7.7.74
2.7.1.64
2.1.1.39
2.1.1.40
2.7.1.151
2.7.1.140
2.7.8.34
1.1.1.-
3.7.1.-
5.1.-.-
1.1.1.18
4.2.1.44
3.7.1.22
5.3.1.30
2.7.1.92
4.1.2.29
2.7.1.158
2.7.1.134
1.13.99.1
1.1.1.370
1.1.1.371
5.3.99.11
1.2.1.18
3.1.3.26
3.1.3.8
5.3.1.1
1.1.1.369
00562 10/26/17
(c) Kanehisa Laboratories

GLYCOSYLPHOSPHATIDYLINOSITOL (GPI) - ANCHOR BIOSYNTHESIS

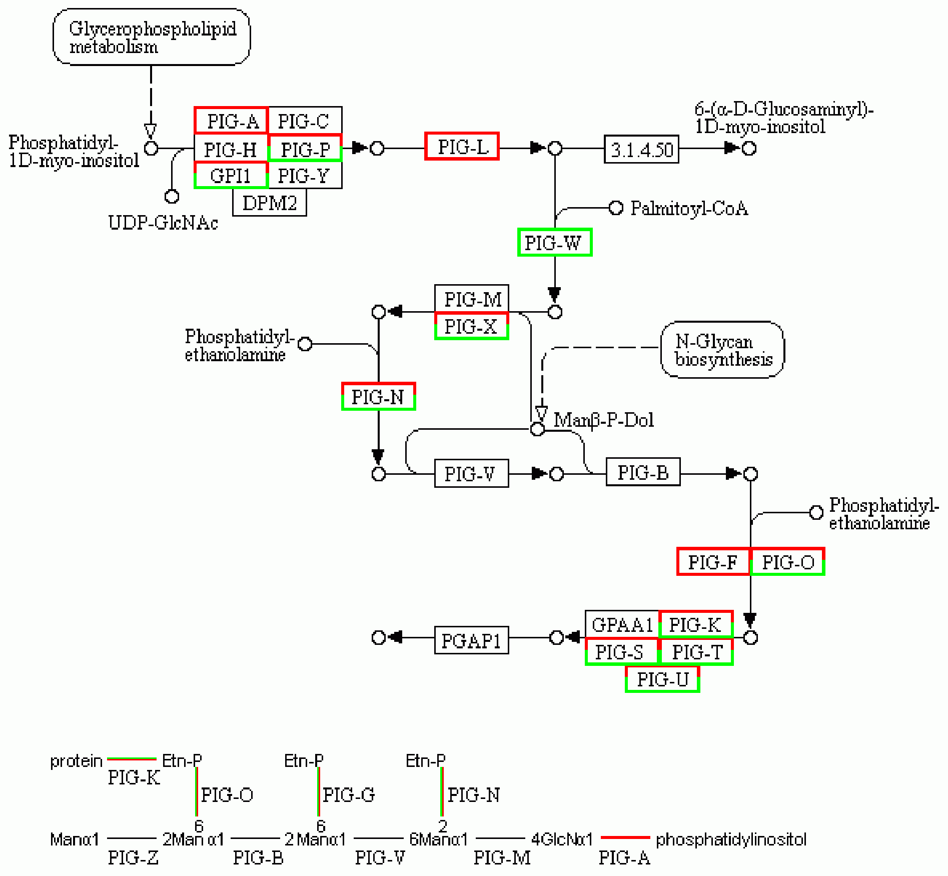

00563 12/27/16
(c) Kanehisa Laboratories

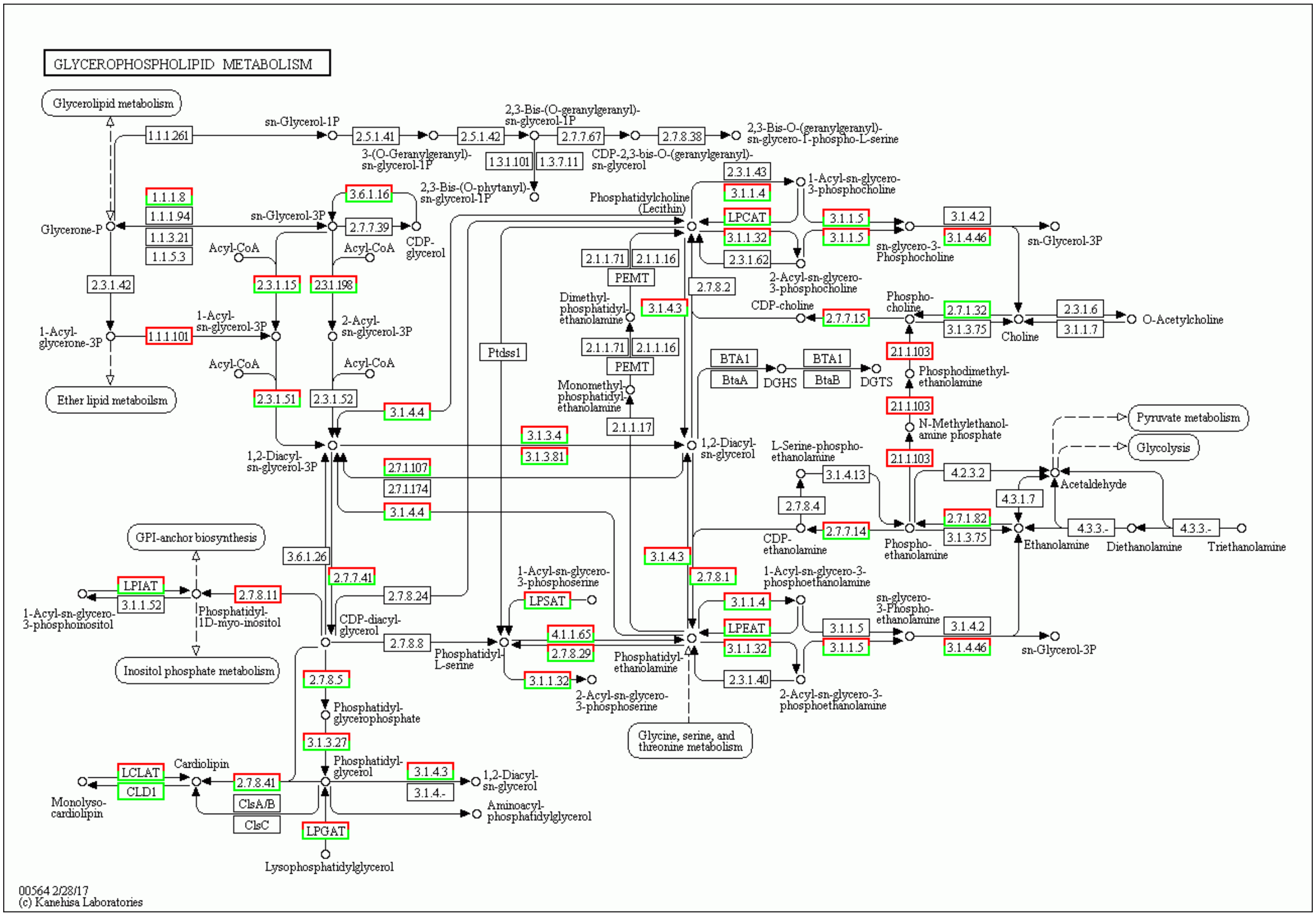
GLYCEROPHOSPHOLIPID METABOLISM
Glycerolipid metabolism
1.1.1.261
sn-Glycerol-1P
2.5.1.41
3-(O-Geranylgeranyl)-sn-glycerol-1P
2.5.1.42
2,3-Bis-(O-geranylgeranyl)-sn-glycerol-1P
1.3.1.101
1.3.7.11
2.7.7.67
CDP-2,3-bis-O-(geranylgeranyl)-sn-glycerol
2.7.8.38
2,3-Bis-O-(geranylgeranyl)-sn-glycero-1-phospho-L-serine
2,3-Bis-(O-phytanyl)-sn-glycerol-1P
1.1.1.8
1.1.1.94
1.1.3.21
1.1.5.3
Glycerone-P
3.6.1.16
sn-Glycerol-3P
2.7.7.39
CDP-glycerol
Acyl-CoA
2.3.1.15
2.3.1.198
2.3.1.42
1-Acyl-glycerone-3P
1.1.1.101
1-Acyl-sn-glycerol-3P
2-Acyl-sn-glycerol-3P
Ether lipid metaboilsm
2.3.1.51
2.3.1.52
3.1.4.4
1,2-Diacyl-sn-glycerol-3P
2.7.1.107
2.7.1.174
3.1.3.4
3.1.3.81
Ptdss1
Phosphatidylcholine (Lecithin)
2.3.1.43
3.1.1.4
LPCAT
3.1.1.32
2.3.1.62
1-Acyl-sn-glycero-3-phosphocholine
2-Acyl-sn-glycero-3-phosphocholine
3.1.1.5
sn-glycero-3-Phosphocholine
3.1.4.2
3.1.4.46
sn-Glycerol-3P
2.1.1.71
2.1.1.16
PEMT
Dimethyl-phosphatidyl-ethanolamine
3.1.4.3
2.7.8.2
CDP-choline
2.7.7.15
Phospho-choline
2.7.1.32
3.1.3.75
Choline
2.3.1.6
3.1.1.7
O-Acetylcholine
Monomethyl-phosphatidyl-ethanolamine
2.1.1.17
BTA1
BtaA
DGHS
BTA1
BtaB
DGTS
2.1.1.103
Phosphodimethyl-ethanolamine
N-Methylethanol-amine phosphate
1,2-Diacyl-sn-glycerol
L-Serine-phospho-ethanolamine
3.1.4.13
Pyruvate metabolism
Glycolysis
4.2.3.2
Acetaldehyde
4.3.1.7
2.7.8.4
CDP-ethanolamine
2.7.7.14
Phospho-ethanolamine
2.7.1.82
3.1.3.75
Ethanolamine
4.3.3.-
Diethanolamine
Triethanolamine
GPI-anchor biosynthesis
3.6.1.26
LPIAT
3.1.1.52
1-Acyl-sn-glycero-3-phosphoinositol
2.7.8.11
Phosphatidyl-1D-myo-inositol
Inositol phosphate metabolism
2.7.7.41
2.7.8.24
CDP-diacyl-glycerol
2.7.8.8
1-Acyl-sn-glycero-3-phosphoserine
LPSAT
4.1.1.65
2.7.8.29
Phosphatidyl-L-serine
3.1.1.32
2-Acyl-sn-glycero-3-phosphoserine
3.1.4.3
2.7.8.1
Phosphatidyl-ethanolamine
1-Acyl-sn-glycero-3-phosphoethanolamine
3.1.1.4
LPEAT
3.1.1.32
2.3.1.40
2-Acyl-sn-glycero-3-phosphoethanolamine
sn-glycero-3-Phospho-ethanolamine
3.1.1.5
3.1.4.2
3.1.4.46
sn-Glycerol-3P
Glycine, serine, and threonine metabolism
2.7.8.5
Phosphatidyl-glycerophosphate
3.1.3.27
Phosphatidyl-glycerol
3.1.4.3
3.1.4.-
1,2-Diacyl-sn-glycerol
Aminoacyl-phosphatidylglycerol
LCLAT
CLD1
Cardiolipin
Monolyso-cardiolipin
2.7.8.41
ClsA/B
ClsC
LPGAT
Lysophosphatidylglycerol
00564 2/28/17
(c) Kanehisa Laboratories

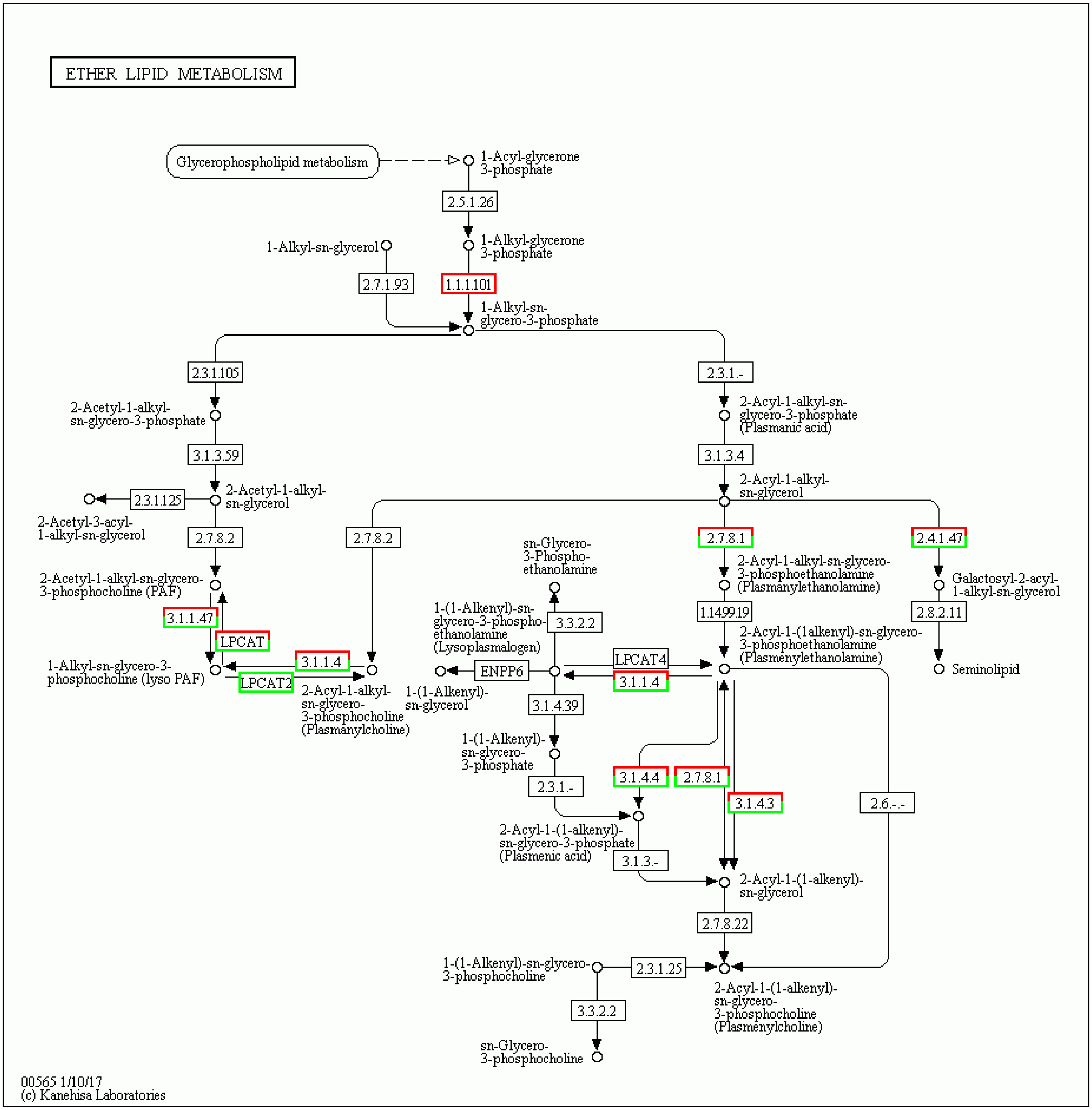
ETHER LIPID METABOLISM
Glycerophospholipid metabolism
1-Acyl-glycerone 3-phosphate
2.5.1.26
1-Alkyl-sn-glycerol
1-Alkyl-glycerone 3-phosphate
2.7.1.93
1.1.1.101
1-Alkyl-sn-glycero-3-phosphate
2.3.1.105
2.3.1.-
2-Acetyl-1-alkyl-sn-glycero-3-phosphate
2-Acyl-1-alkyl-sn-glycero-3-phosphate (Plasmanic acid)
3.1.3.59
3.1.3.4
2.3.1.125
2-Acetyl-1-alkyl-sn-glycerol
2-Acyl-1-alkyl-sn-glycerol
2-Acetyl-3-acyl-1-alkyl-sn-glycerol
2.7.8.2
2.7.8.2
2.7.8.1
2.4.1.47
sn-Glycero-3-Phospho-ethanolamine
2-Acyl-1-alkyl-sn-glycero-3-phosphoethanolamine (Plasmanylethanolamine)
2-Acetyl-1-alkyl-sn-glycero-3-phosphocholine (PAF)
Galactosyl-2-acyl-1-alkyl-sn-glycerol
1-(1-Alkenyl)-sn-glycero-3-phospho-ethanolamine (Lysoplasmalogen)
3.3.2.2
1.14.99.19
2.8.2.11
3.1.1.47
LPCAT
2-Acyl-1-(1alkenyl)-sn-glycero-3-phosphoethanolamine (Plasmenylethanolamine)
3.1.1.4
LPCAT4
1-Alkyl-sn-glycero-3-phosphocholine (lyso PAF)
ENPP6
3.1.1.4
Seminolipid
LPCAT2
2-Acyl-1-alkyl-sn-glycero-3-phosphocholine (Plasmanylcholine)
1-(1-Alkenyl)-sn-glycerol
3.1.4.39
1-(1-Alkenyl)-sn-glycero-3-phosphate
2.3.1.-
3.1.4.4
2.7.8.1
3.1.4.3
2.6.-.-
2-Acyl-1-(1-alkenyl)-sn-glycero-3-phosphate (Plasmenic acid)
3.1.3.-
2-Acyl-1-(1-alkenyl)-sn-glycerol
2.7.8.22
1-(1-Alkenyl)-sn-glycero-3-phosphocholine
2.3.1.25
2-Acyl-1-(1-alkenyl)-sn-glycero-3-phosphocholine (Plasmenylcholine)
3.3.2.2
sn-Glycero-3-phosphocholine
00565 1/10/17
(c) Kanehisa Laboratories

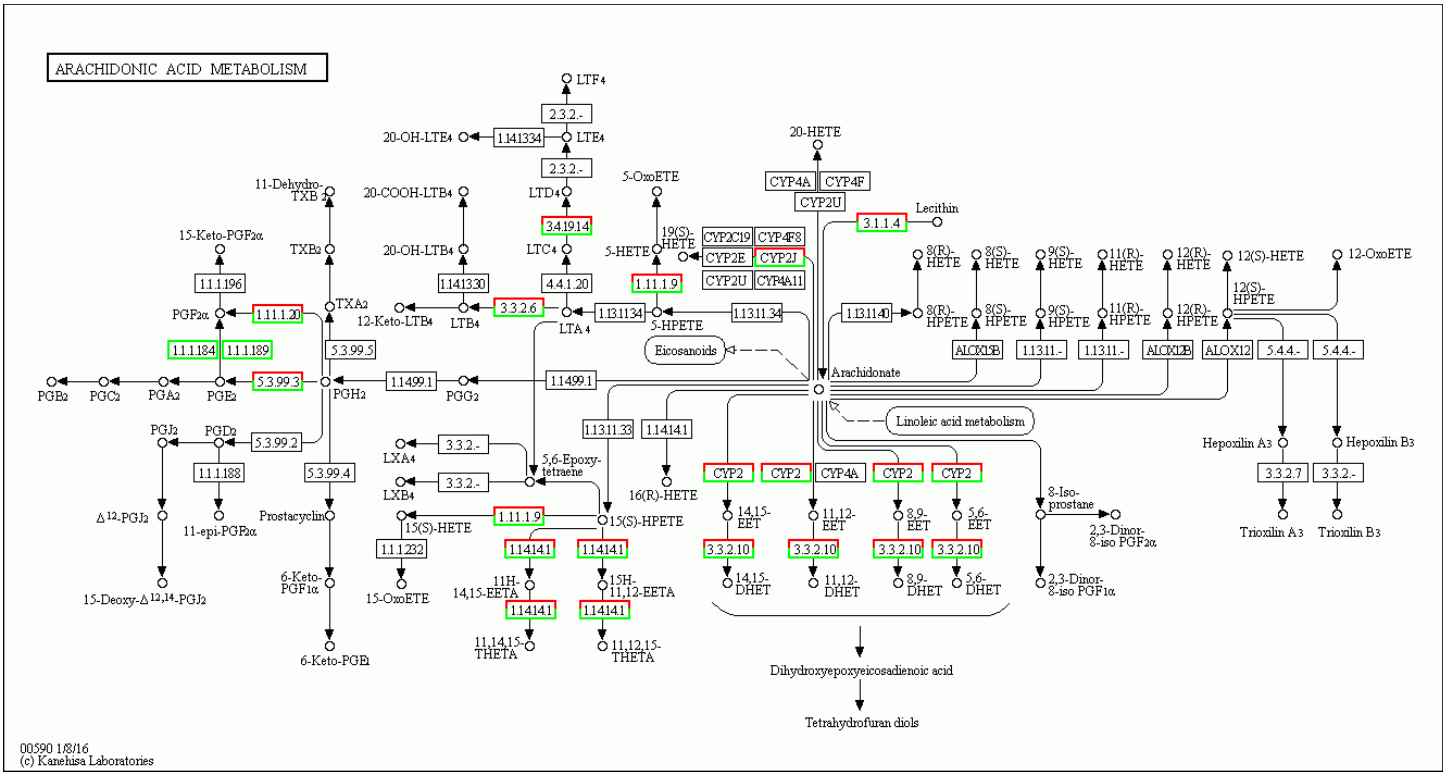
ARACHIDONIC ACID METABOLISM
LTF4
2.3.2.-
20-OH-LTE4
1.14.13.34
LTE4
20-HETE
CYP4A
CYP4F
CYP2U
Lecithin
3.1.1.4
5-OxoETE
LTD4
3.4.19.14
LTC4
11-Dehydro-TXB2
20-COOH-LTB4
20-OH-LTB4
15-Keto-PGF2α
TXB2
1.1.1.196
1.14.13.30
4.4.1.20
5-HETE
19(S)-HETE
CYP2C19
CYP4F8
CYP2E
CYP2J
CYP2U
CYP4A11
1.11.1.9
PGF2α
1.11.1.20
TXA2
12-Keto-LTB4
LTB4
3.3.2.6
LTA4
1.13.11.34
5-HPETE
Eicosanoids
1.13.11.40
8(R)-HETE
8(S)-HETE
9(S)-HETE
11(R)-HETE
12(R)-HETE
12(S)-HETE
12-OxoETE
12(S)-HPETE
8(R)-HPETE
8(S)-HPETE
9(S)-HPETE
11(R)-HPETE
12(R)-HPETE
ALOX15B
1.13.11.-
ALOX12B
ALOX12
5.4.4.-
1.1.1.184
1.1.1.189
5.3.99.5
PGB2
PGC2
PGA2
PGE2
5.3.99.3
PGH2
1.14.99.1
PGG2
Arachidonate
Linoleic acid metabolism
PGJ2
PGD2
5.3.99.2
1.1.1.188
5.3.99.4
1.13.11.33
1.14.14.1
LXA4
3.3.2.-
LXB4
5,6-Epoxy-tetraene
16(R)-HETE
CYP2
CYP4A
Hepoxilin A3
Hepoxilin B3
3.3.2.7
Δ12-PGJ2
11-epi-PGF2α
Prostacyclin
15(S)-HETE
15(S)-HPETE
14,15-EET
11,12-EET
8,9-EET
5,6-EET
8-Iso-prostane
2,3-Dinor-8-iso PGF2α
Trioxilin A3
Trioxilin B3
1.1.1.232
3.3.2.10
15-Deoxy-Δ12,14-PGJ2
6-Keto-PGF1α
15-OxoETE
11H-14,15-EETA
15H-11,12-EETA
14,15-DHET
11,12-DHET
8,9-DHET
5,6-DHET
2,3-Dinor-8-iso PGF1α
11,14,15-THETA
11,12,15-THETA
6-Keto-PGE1
Dihydroxyepoxyeicosadienoic acid
Tetrahydrofuran diols
00590 1/8/16
(c) Kanehisa Laboratories

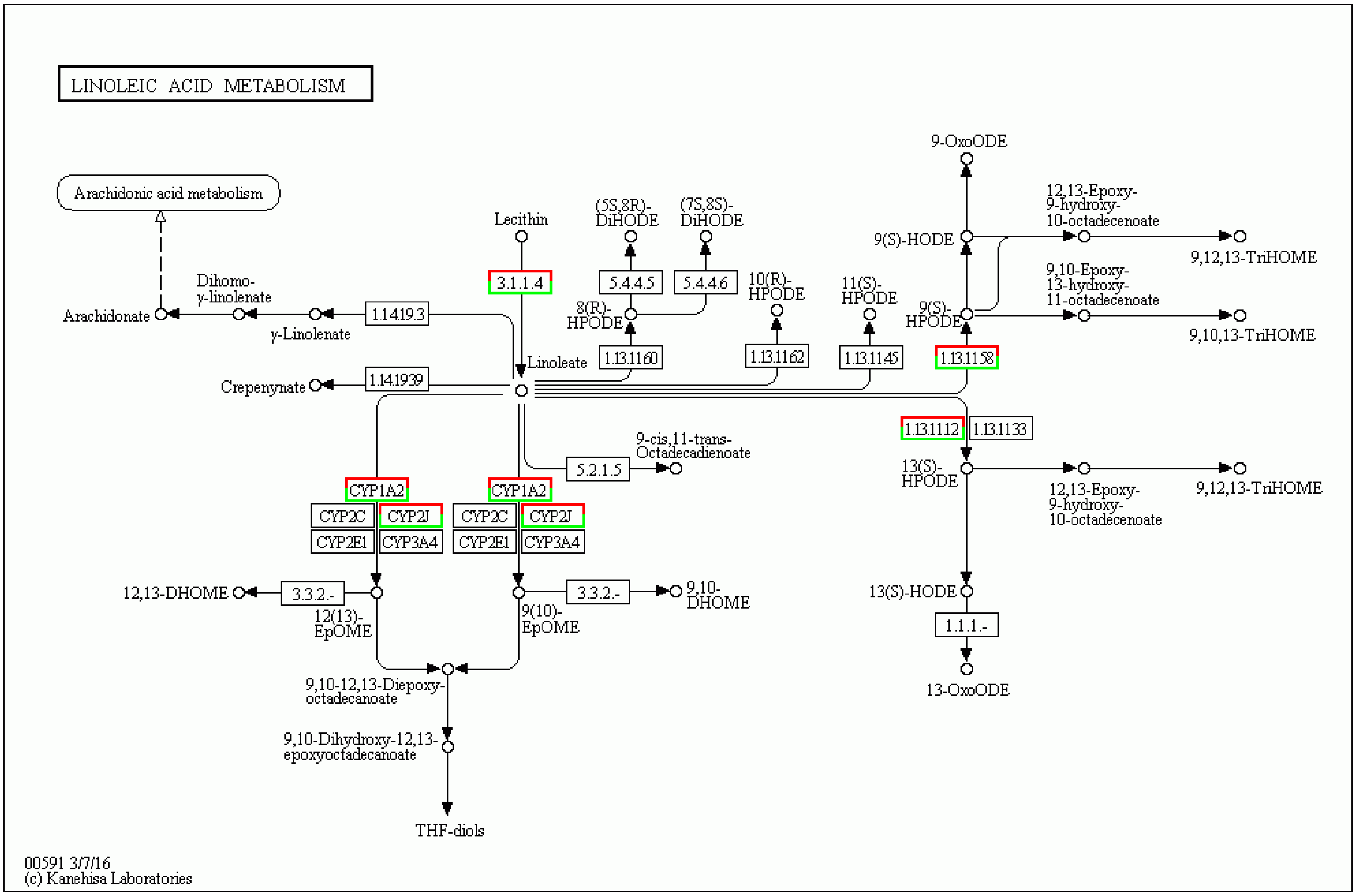
LINOLEIC ACID METABOLISM
9-OxoODE
Arachidonic acid metabolism
Lecithin
(5S,8R)-DiHODE
(7S,8S)-DiHODE
12,13-Epoxy-9-hydroxy-10-octadecenoate
9(S)-HODE
9,12,13-TriHOME
3.1.1.4
5.4.4.5
5.4.4.6
10(R)-HPODE
11(S)-HPODE
9,10-Epoxy-13-hydroxy-11-octadecenoate
Dihomo-γ-linolenate
8(R)-HPODE
9(S)-HPODE
9,10,13-TriHOME
Arachidonate
1.14.19.3
γ-Linolenate
Linoleate
1.13.11.60
1.13.11.62
1.13.11.45
1.13.11.58
Crepenynate
1.14.19.39
1.13.11.12
1.13.11.33
9-cis,11-trans-Octadecadienoate
5.2.1.5
13(S)-HPODE
12,13-Epoxy-9-hydroxy-10-octadecenoate
9,12,13-TriHOME
CYP1A2
CYP2C
CYP2J
CYP2E1
CYP3A4
12,13-DHOME
3.3.2.-
12(13)-EpOME
9(10)-EpOME
9,10-DHOME
13(S)-HODE
1.1.1.-
9,10-12,13-Diepoxy-octadecanoate
13-OxoODE
9,10-Dihydroxy-12,13-epoxyoctadecanoate
THF-diols
00591 3/7/16
(c) Kanehisa Laboratories

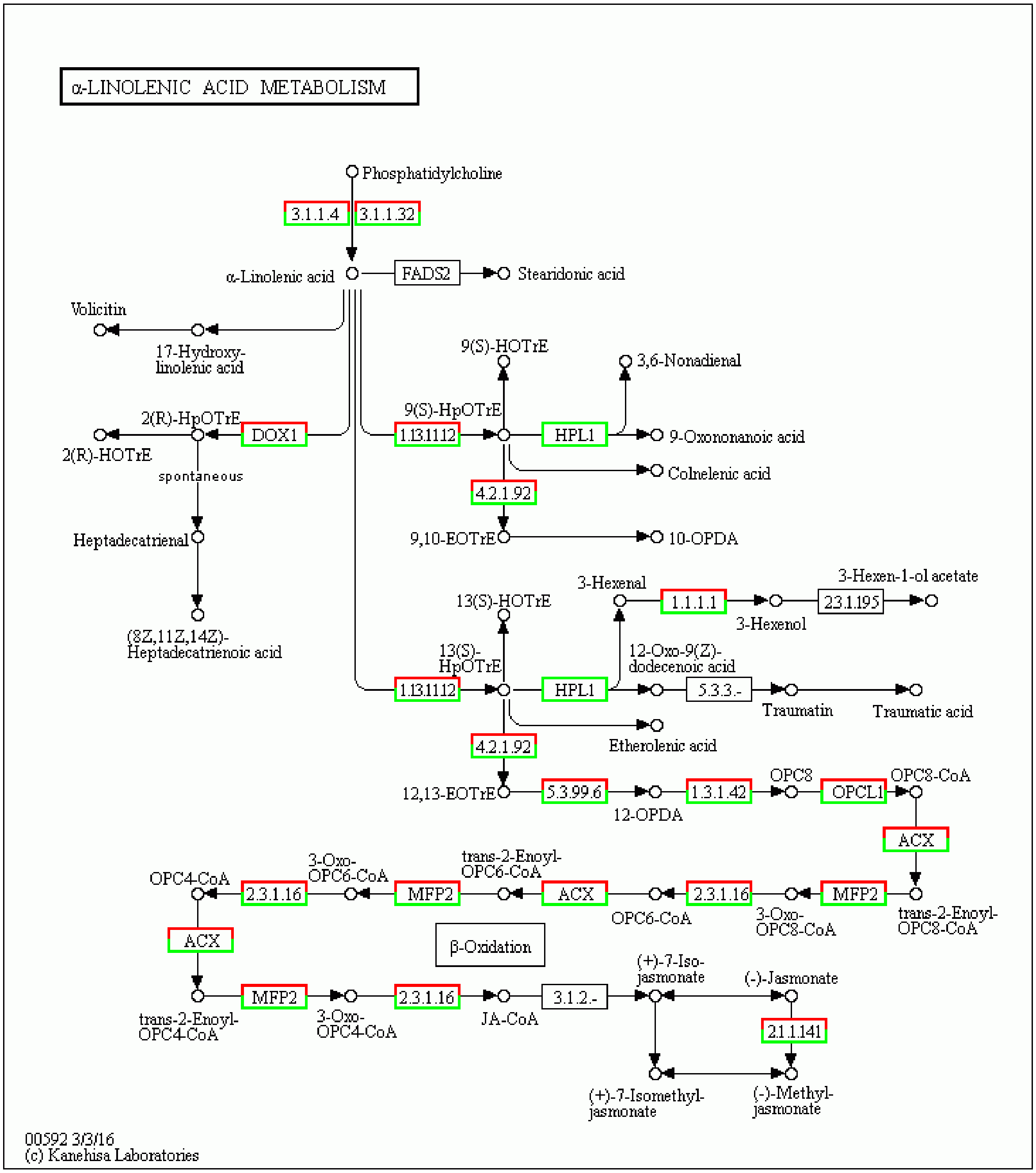

α-LINOLENIC ACID METABOLISM
Phosphatidylcholine
3.1.1.4
3.1.1.32
α-Linolenic acid
FADS2
Stearidonic acid
Volicitin
17-Hydroxy-linolenic acid
9(S)-HOTrE
3,6-Nonadienal
9(S)-HpOTrE
2(R)-HpOTrE
DOX1
2(R)-HOTrE
1.13.11.12
HPL1
9-Oxononanoic acid
Colnelenic acid
spontaneous
4.2.1.92
Heptadecatrienal
9,10-EOTrE
10-OPDA
3-Hexenal
1.1.1.1
3-Hexen-1-ol acetate
2.3.1.195
13(S)-HOTrE
3-Hexenol
(8Z,11Z,14Z)-Heptadecatrienoic acid
13(S)-HpOTrE
12-Oxo-9(Z)-dodecenoic acid
1.13.11.12
HPL1
5.3.3.-
Traumatin
Traumatic acid
4.2.1.92
Etherolenic acid
OPC8
OPC8-CoA
12,13-EOTrE
5.3.99.6
1.3.1.42
OPCL1
12-OPDA
ACX
3-Oxo-OPC6-CoA
trans-2-Enoyl-OPC6-CoA
OPC4-CoA
2.3.1.16
MFP2
ACX
2.3.1.16
MFP2
OPC6-CoA
3-Oxo-OPC8-CoA
trans-2-Enoyl-OPC8-CoA
ACX
β-Oxidation
(+)-7-Iso-jasmonate
(-)-Jasmonate
MFP2
2.3.1.16
3.1.2.-
2.1.1.141
trans-2-Enoyl-OPC4-CoA
3-Oxo-OPC4-CoA
JA-CoA
(+)-7-Isomethyl-jasmonate
(-)-Methyl-jasmonate
00592 3/3/16
(c) Kanehisa Laboratories

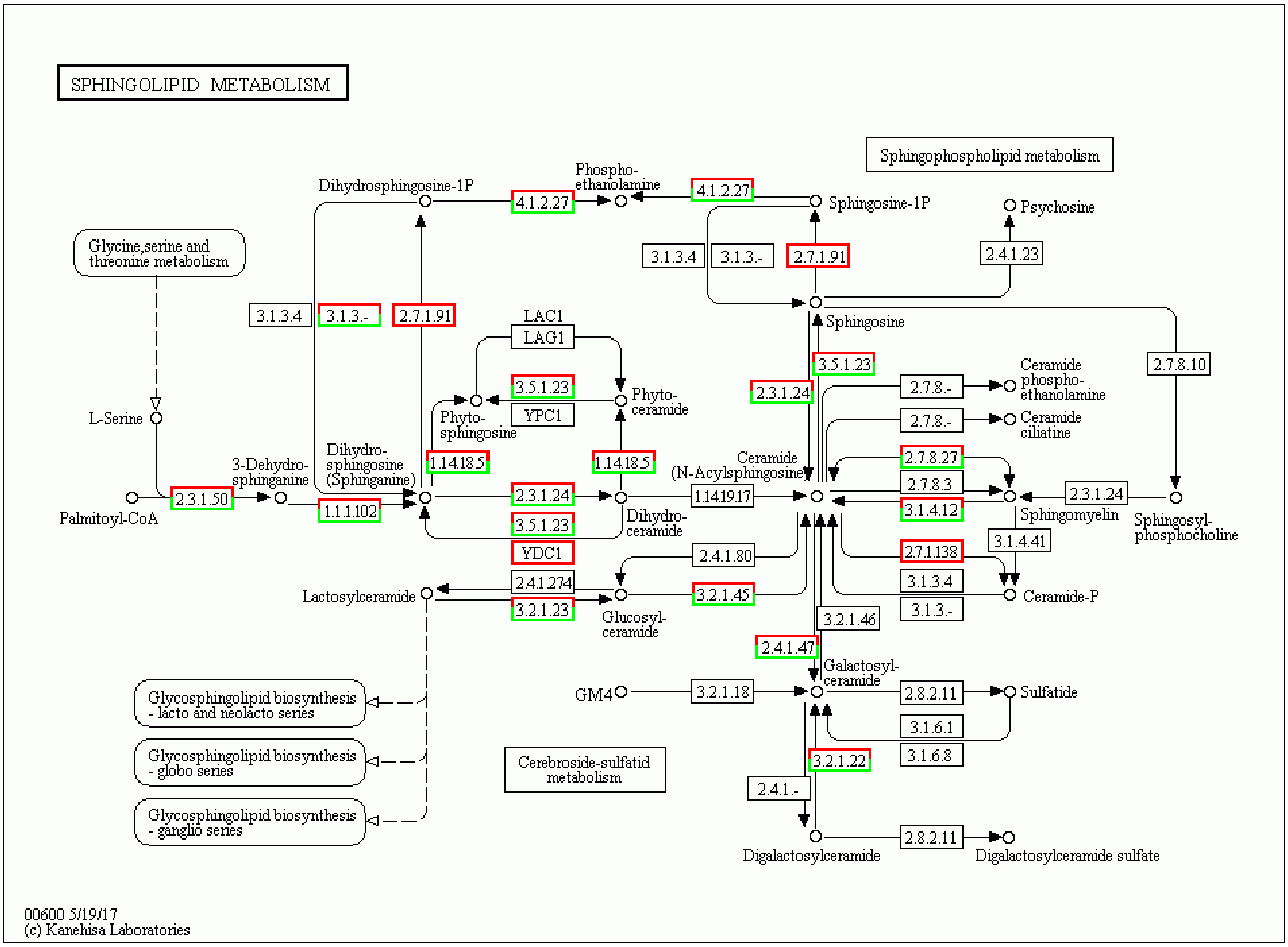
SPHINGOLIPID METABOLISM
Sphingophospholipid metabolism
Dihydrosphingosine-1P
Phospho-ethanolamine
4.1.2.27
4.1.2.27
Sphingosine-1P
Psychosine
Glycine,serine and threonine metabolism
3.1.3.4
3.1.3.-
2.7.1.91
2.4.1.23
3.1.3.4
3.1.3.-
2.7.1.91
Sphingosine
LAC1
LAG1
3.5.1.23
2.7.8.10
3.5.1.23
2.3.1.24
2.7.8.-
Ceramide phospho-ethanolamine
Phyto-ceramide
YPC1
Phyto-sphingosine
2.7.8.-
Ceramide ciliatine
L-Serine
Dihydro-sphingosine (Sphinganine)
3-Dehydro-sphinganine
1.14.18.5
1.14.18.5
Ceramide (N-Acylsphingosine)
2.7.8.27
2.7.8.3
2.3.1.24
Palmitoyl-CoA
2.3.1.50
1.1.1.102
2.3.1.24
1.14.19.17
3.1.4.12
Sphingomyelin
Sphingosyl-phosphocholine
3.5.1.23
Dihydro-ceramide
3.1.4.41
YDC1
2.4.1.80
2.7.1.138
Lactosylceramide
2.4.1.274
3.2.1.23
3.2.1.45
3.1.3.4
Ceramide-P
Glucosyl-ceramide
3.2.1.46
3.1.3.-
2.4.1.47
Galactosyl-ceramide
Glycosphingolipid biosynthesis - lacto and neolacto series
GM4
3.2.1.18
2.8.2.11
Sulfatide
3.1.6.1
Glycosphingolipid biosynthesis - globo series
Cerebroside-sulfatid metabolism
3.2.1.22
3.1.6.8
2.4.1.-
Glycosphingolipid biosynthesis - ganglio series
Digalactosylceramide
2.8.2.11
Digalactosylceramide sulfate
00600 5/19/17
(c) Kanehisa Laboratories

GLYCOSPHINGOLIPID BIOSYNTHESIS - LACTO AND NEOLACTO SERIES

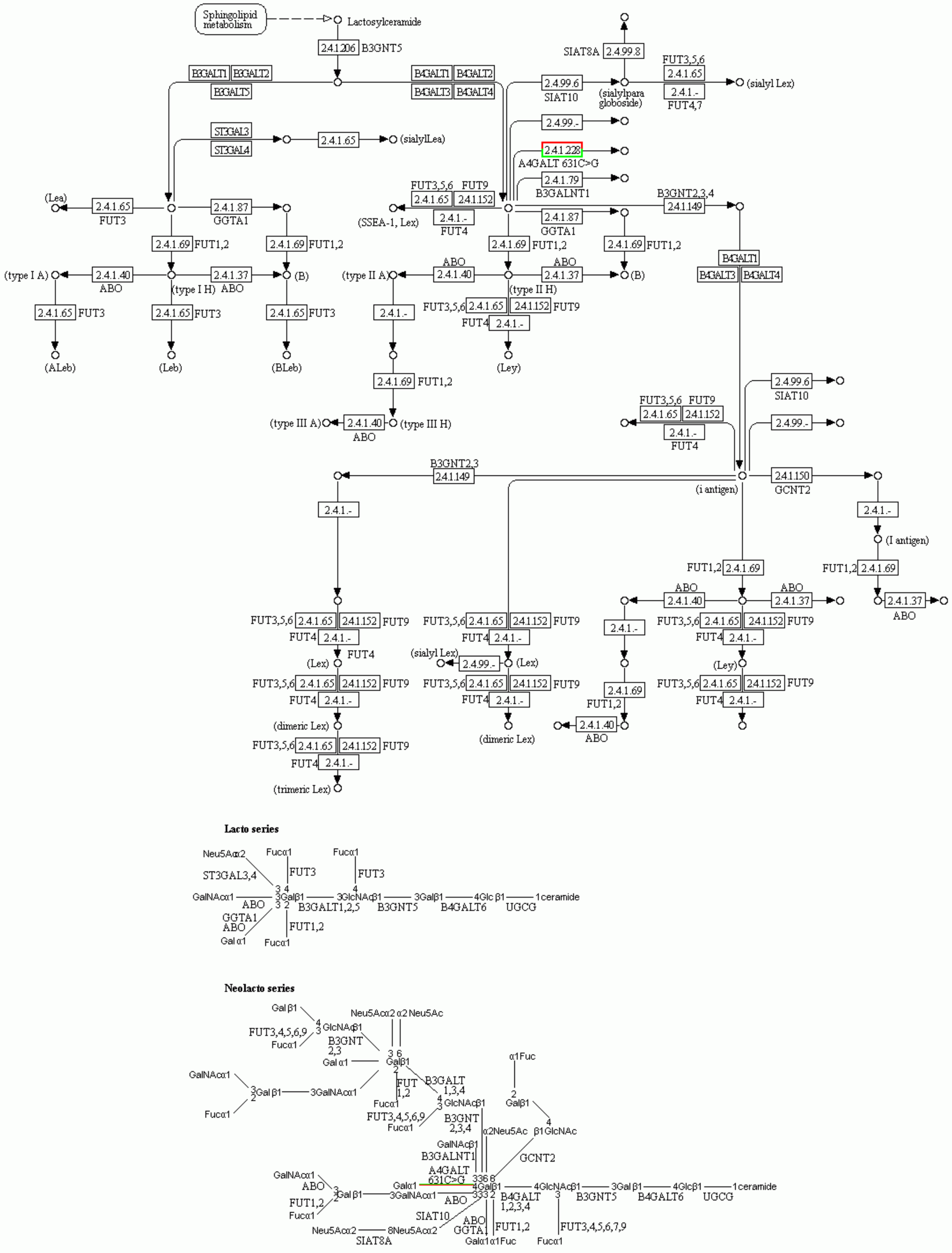

00601 4/11/17
(c) Kanehisa Laboratories

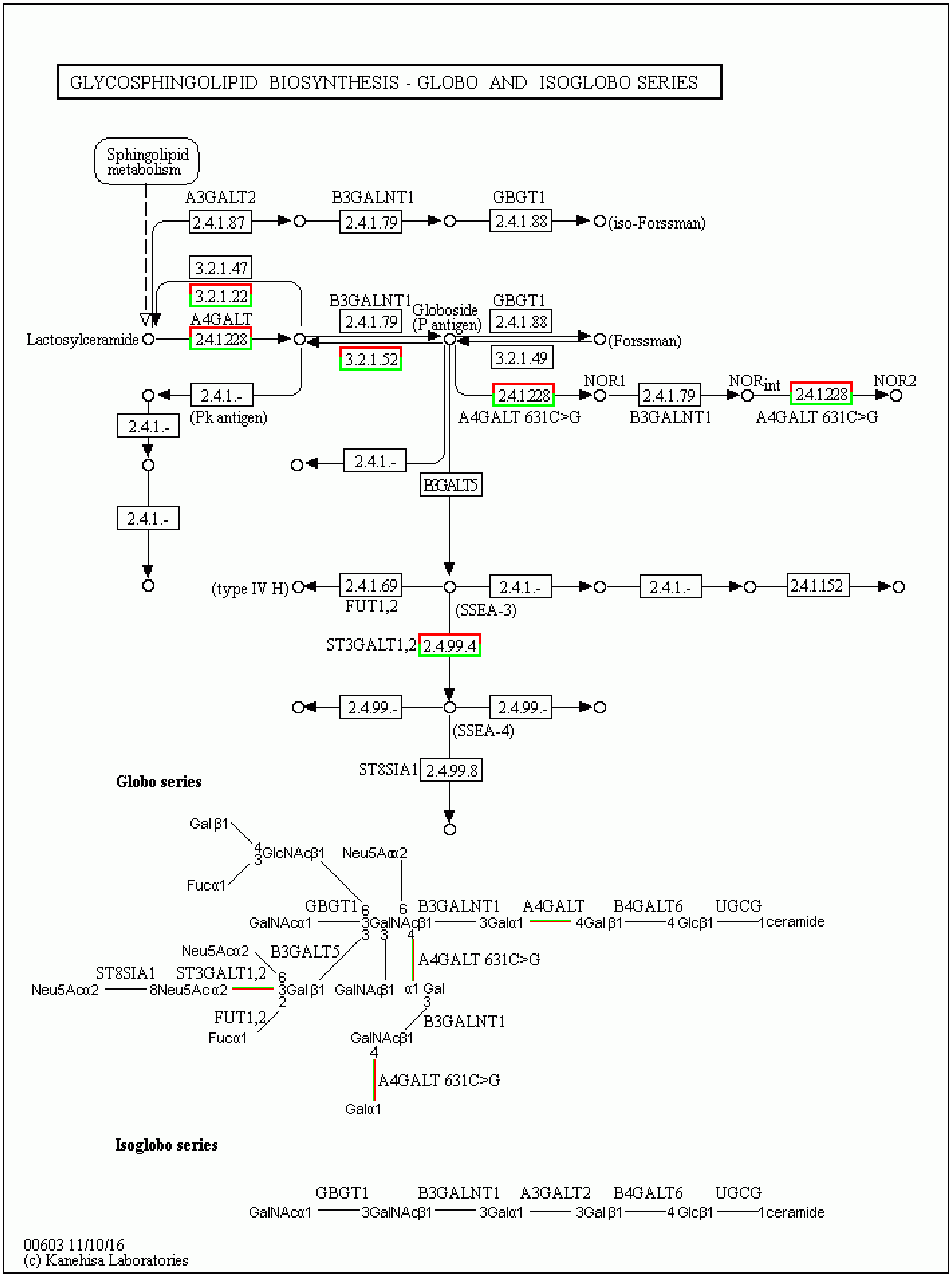
GLYCOSPHINGOLIPID BIOSYNTHESIS - GLOBO AND ISOGLOBO SERIES
Sphingolipid metabolism
A3GALT2
2.4.1.87
B3GALNT1
2.4.1.79
GBGT1
2.4.1.88
(iso-Forssman)
3.2.1.47
3.2.1.22
A4GALT
2.4.1.228
Lactosylceramide
B3GALNT1
2.4.1.79
Globoside (P antigen)
GBGT1
2.4.1.88
(Forssman)
3.2.1.52
3.2.1.49
NOR1
NORint
NOR2
2.4.1.228
2.4.1.79
2.4.1.228
A4GALT 631C>G
B3GALNT1
A4GALT 631C>G
2.4.1.-
(Pk antigen)
2.4.1.-
2.4.1.-
2.4.1.-
B3GALT5
(type IV H)
2.4.1.69
FUT1,2
2.4.1.-
2.4.1.-
2.4.1.152
(SSEA-3)
ST3GALT1,2
2.4.99.4
2.4.99.-
2.4.99.-
(SSEA-4)
ST8SIA1
2.4.99.8
Globo series
Galβ1
GlcNAcβ1
Fucα1
Neu5Acα2
GBGT1
B3GALNT1
A4GALT
B4GALT6
UGCG
GalNAcα1
3GalNAcβ1
3Galα1
4Galβ1
4Glcβ1
1ceramide
Neu5Acα2
B3GALT5
A4GALT 631C>G
ST8SIA1
ST3GALT1,2
Neu5Acα2
8Neu5Acα2
3Galβ1
GalNAcβ1
α1 Gal
FUT1,2
Fucα1
B3GALNT1
GalNAcβ1
A4GALT 631C>G
Galα1
Isoglobo series
GBGT1
B3GALNT1
A3GALT2
B4GALT6
UGCG
GalNAcα1
3GalNAcβ1
3Galα1
3Galβ1
4Glcβ1
1ceramide
00603 11/10/16
(c) Kanehisa Laboratories

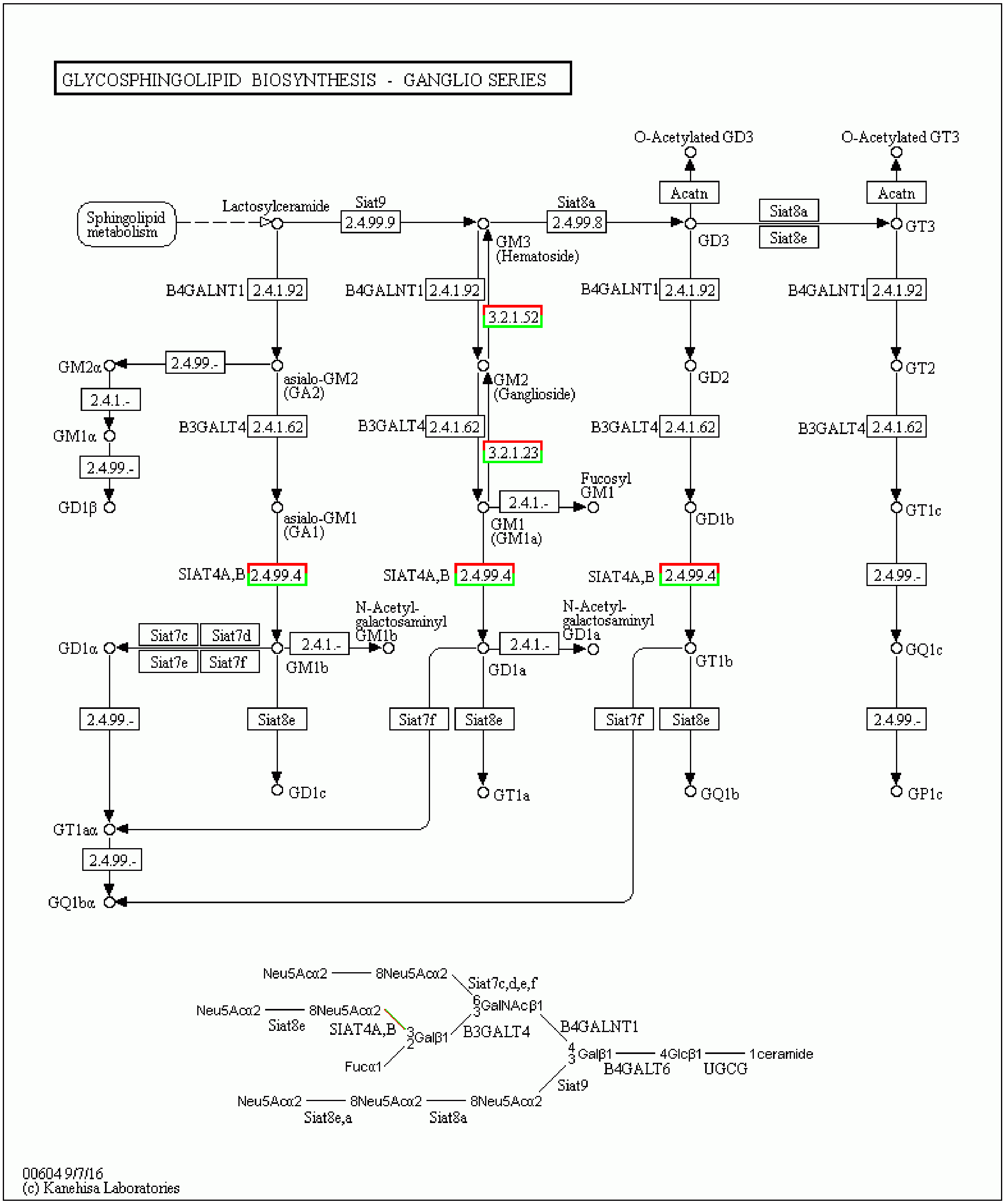
GLYCOSPHINGOLIPID BIOSYNTHESIS - GANGLIO SERIES
Sphingolipid metabolism
Lactosylceramide
Siat9
2.4.99.9
Siat8a
2.4.99.8
O-Acetylated GD3
O-Acetylated GT3
Acatn
Siat8a
Siat8e
GM3 (Hematoside)
GD3
GT3
B4GALNT1
2.4.1.92
3.2.1.52
GM2α
2.4.99.-
asialo-GM2 (GA2)
GM2 (Ganglioside)
GD2
GT2
2.4.1.-
GM1α
B3GALT4
2.4.1.62
3.2.1.23
Fucosyl GM1
GD1β
asialo-GM1 (GA1)
GM1 (GM1a)
GD1b
GT1c
SIAT4A,B
2.4.99.4
N-Acetyl-galactosaminyl GM1b
N-Acetyl-galactosaminyl GD1a
Siat7c
Siat7d
Siat7e
Siat7f
GD1α
GM1b
GD1a
GT1b
GQ1c
Siat8e
GD1c
GT1a
GQ1b
GP1c
GT1aα
GQ1bα
Neu5Acα2
8Neu5Acα2
Siat7c,d,e,f
6 3 GalNAcβ1
Siat8e
SIAT4A,B
3 2 Galβ1
B3GALT4
B4GALNT1
Fucα1
4 3 Galβ1
4Glcβ1
1ceramide
B4GALT6
UGCG
Siat9
Siat8e,a
Siat8a
00604 9/7/16
(c) Kanehisa Laboratories

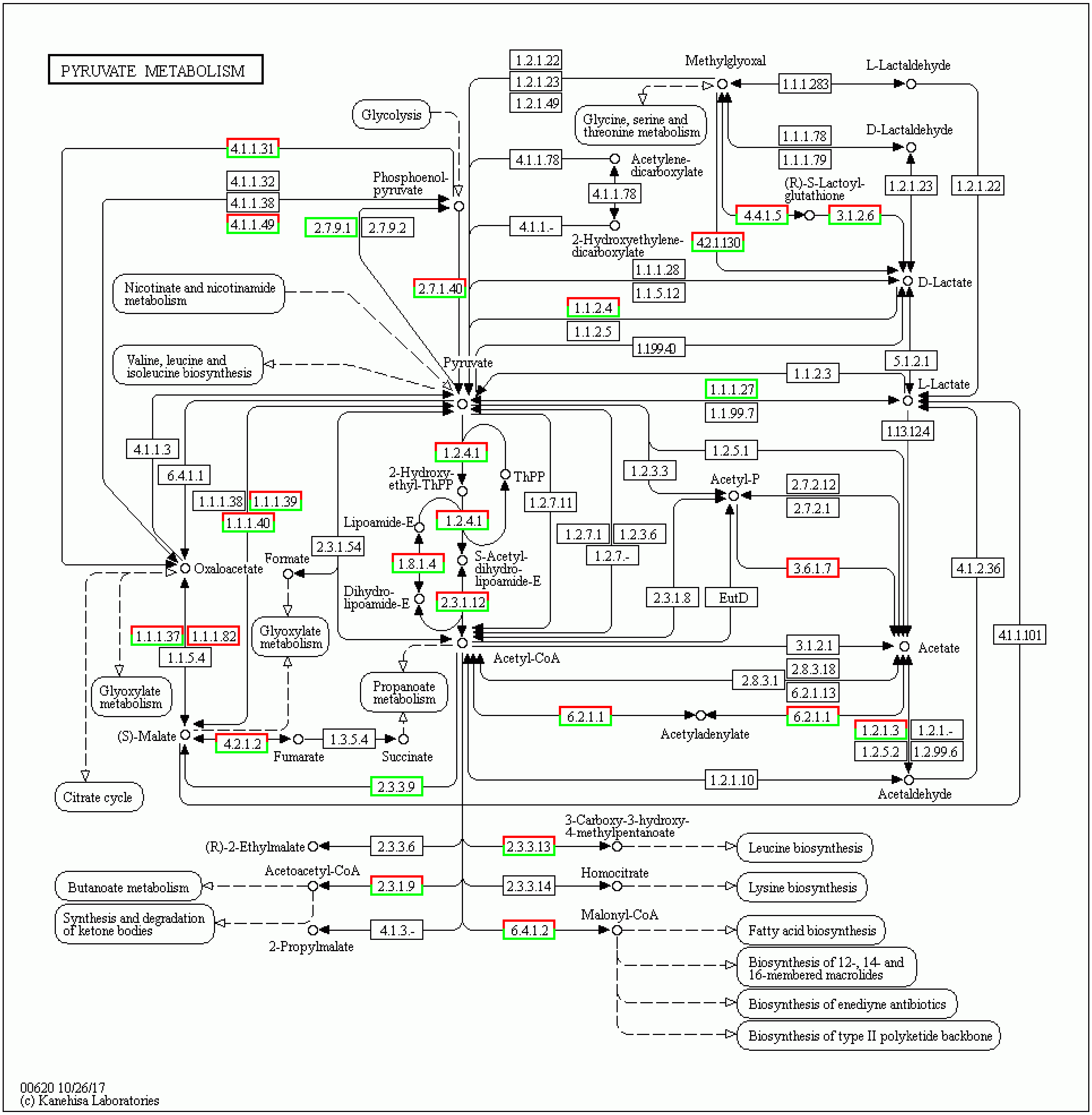

PYRUVATE METABOLISM
Glycolysis
Phosphoenol-pyruvate
4.1.1.31
4.1.1.32
4.1.1.38
4.1.1.49
2.7.9.1
2.7.9.2
1.2.1.22
1.2.1.23
1.2.1.49
Methylglyoxal
L-Lactaldehyde
1.1.1.283
Glycine, serine and threonine metabolism
1.1.1.78
1.1.1.79
D-Lactaldehyde
4.1.1.78
Acetylene-dicarboxylate
4.1.1.78
4.1.1.-
2-Hydroxyethylene-dicarboxylate
(R)-S-Lactoyl-glutathione
1.2.1.23
1.2.1.22
4.4.1.5
3.1.2.6
4.2.1.130
1.1.1.28
1.1.5.12
D-Lactate
Nicotinate and nicotinamide metabolism
2.7.1.40
1.1.2.4
1.1.2.5
1.1.99.40
Valine, leucine and isoleucine biosynthesis
Pyruvate
5.1.2.1
1.1.2.3
1.1.1.27
L-Lactate
1.1.99.7
1.13.12.4
4.1.1.3
6.4.1.1
1.2.4.1
1.2.5.1
1.2.3.3
2-Hydroxy-ethyl-ThPP
ThPP
Acetyl-P
2.7.2.12
2.7.2.1
1.1.1.38
1.1.1.39
1.1.1.40
1.2.7.11
Lipoamide-E
1.2.4.1
1.2.7.1
1.2.3.6
1.2.7.-
2.3.1.54
Formate
1.8.1.4
S-Acetyl-dihydro-lipoamide-E
3.6.1.7
4.1.2.36
Oxaloacetate
Dihydro-lipoamide-E
2.3.1.12
2.3.1.8
EutD
Glyoxylate metabolism
1.1.1.37
1.1.1.82
1.1.5.4
3.1.2.1
Acetate
4.1.1.101
Acetyl-CoA
2.8.3.18
2.8.3.1
6.2.1.13
Glyoxylate metabolism
Propanoate metabolism
6.2.1.1
Acetyladenylate
6.2.1.1
1.2.1.3
1.2.1.-
1.2.5.2
1.2.99.6
(S)-Malate
4.2.1.2
Fumarate
1.3.5.4
Succinate
1.2.1.10
Acetaldehyde
2.3.3.9
Citrate cycle
(R)-2-Ethylmalate
2.3.3.6
2.3.3.13
3-Carboxy-3-hydroxy-4-methylpentanoate
Leucine biosynthesis
Acetoacetyl-CoA
Butanoate metabolism
2.3.1.9
2.3.3.14
Homocitrate
Lysine biosynthesis
Synthesis and degradation of ketone bodies
4.1.3.-
2-Propylmalate
6.4.1.2
Malonyl-CoA
Fatty acid biosynthesis
Biosynthesis of 12-, 14- and 16-membered macrolides
Biosynthesis of enediyne antibiotics
Biosynthesis of type II polyketide backbone
00620 10/26/17
(c) Kanehisa Laboratories

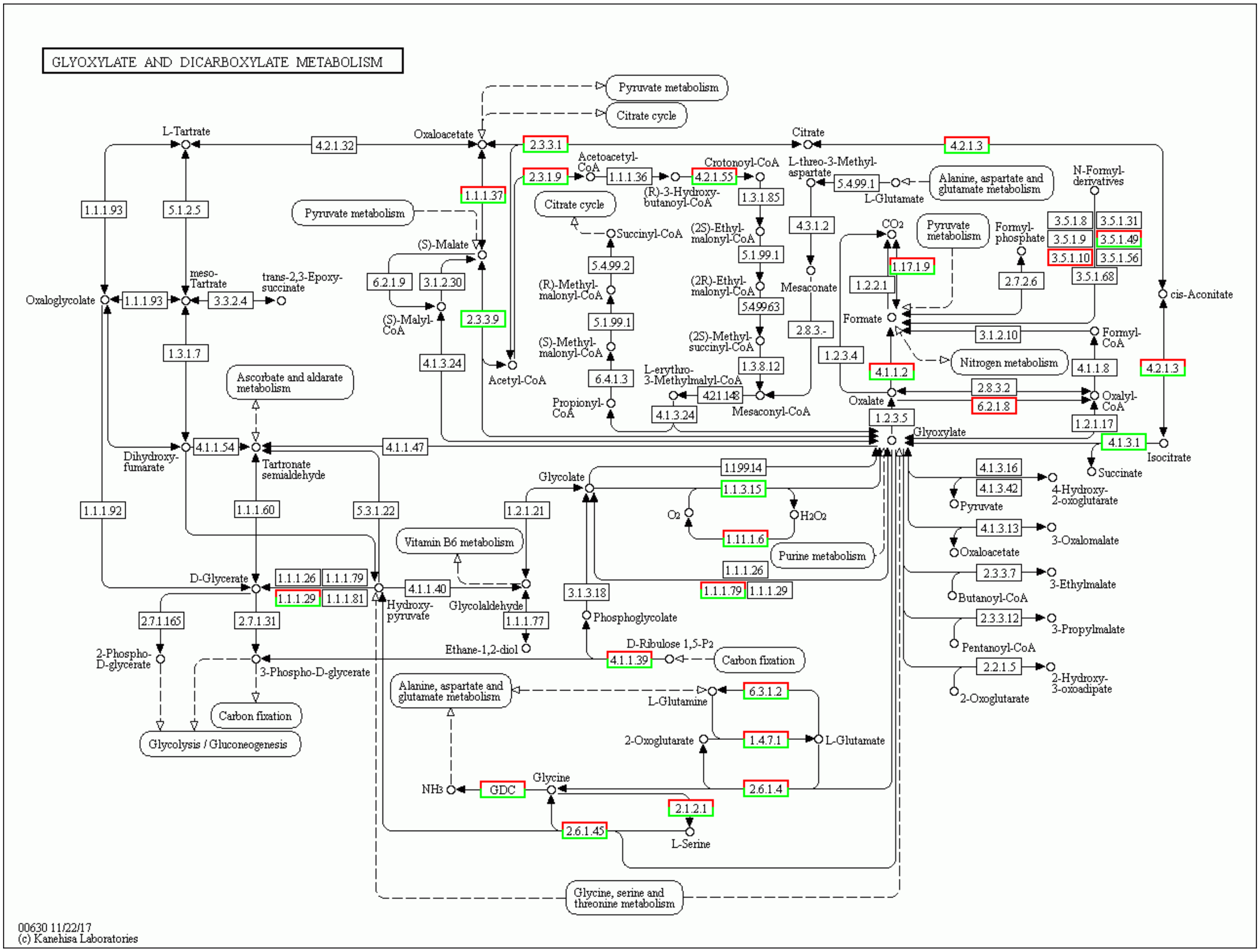
GLYOXYLATE AND DICARBOXYLATE METABOLISM
Pyruvate metabolism
Citrate cycle
L-Tartrate
4.2.1.32
Oxaloacetate
2.3.3.1
Citrate
4.2.1.3
Acetoacetyl-CoA
Crotonoyl-CoA
L-threo-3-Methyl-aspartate
N-Formyl-derivatives
2.3.1.9
1.1.1.36
4.2.1.55
5.4.99.1
Alanine, aspartate and glutamate metabolism
1.1.1.37
(R)-3-Hydroxy-butanoyl-CoA
L-Glutamate
1.1.1.93
5.1.2.5
Pyruvate metabolism
Citrate cycle
1.3.1.85
4.3.1.2
CO2
Pyruvate metabolism
Formyl-phosphate
3.5.1.8
3.5.1.31
3.5.1.9
3.5.1.49
3.5.1.10
3.5.1.56
3.5.1.68
(S)-Malate
Succinyl-CoA
(2S)-Ethyl-malonyl-CoA
5.1.99.1
1.17.1.9
meso-Tartrate
trans-2,3-Epoxy-succinate
6.2.1.9
3.1.2.30
5.4.99.2
(R)-Methyl-malonyl-CoA
(2R)-Ethyl-malonyl-CoA
Mesaconate
1.2.2.1
2.7.2.6
Oxaloglycolate
1.1.1.93
3.3.2.4
cis-Aconitate
5.4.99.63
(S)-Malyl-CoA
2.3.3.9
5.1.99.1
Formate
2.8.3.-
(2S)-Methyl-succinyl-CoA
3.1.2.10
Formyl-CoA
(S)-Methyl-malonyl-CoA
1.3.1.7
1.2.3.4
Nitrogen metabolism
4.1.3.24
1.3.8.12
L-erythro-3-Methylmalyl-CoA
4.1.1.2
4.1.1.8
4.2.1.3
Ascorbate and aldarate metabolism
Acetyl-CoA
6.4.1.3
4.2.1.148
2.8.3.2
Propionyl-CoA
Mesaconyl-CoA
Oxalate
Oxalyl-CoA
6.2.1.8
4.1.3.24
1.2.3.5
1.2.1.17
4.1.1.54
4.1.1.47
Glyoxylate
4.1.3.1
Dihydroxy-fumarate
Tartronate semialdehyde
Isocitrate
1.1.99.14
4.1.3.16
Succinate
Glycolate
4.1.3.42
4.1.3.15
4-Hydroxy-2-oxoglutarate
Pyruvate
1.1.1.92
1.1.1.60
5.3.1.22
1.2.1.21
O2
H2O2
4.1.3.13
1.1.1.6
3-Oxalomalate
Vitamin B6 metabolism
Purine metabolism
Oxaloacetate
1.1.1.26
2.3.3.7
D-Glycerate
1.1.1.26
1.1.1.79
4.1.1.40
3.1.3.18
3-Ethylmalate
1.1.1.29
1.1.1.81
1.1.1.79
1.1.1.29
Butanoyl-CoA
Hydroxy-pyruvate
Glycolaldehyde
2.7.1.165
2.7.1.31
1.1.1.77
Phosphoglycolate
2.3.3.12
3-Propylmalate
D-Ribulose 1,5-P2
Pentanoyl-CoA
2-Phospho-D-glycerate
Ethane-1,2-diol
4.1.1.39
Carbon fixation
3-Phospho-D-glycerate
2.2.1.5
2-Hydroxy-3-oxoadipate
Alanine, aspartate and glutamate metabolism
6.3.1.2
2-Oxoglutarate
L-Glutamine
Carbon fixation
Glycolysis / Gluconeogenesis
2-Oxoglutarate
1.4.7.1
L-Glutamate
Glycine
NH3
GDC
2.6.1.4
2.1.2.1
2.6.1.45
L-Serine
Glycine, serine and threonine metabolism
00630 11/22/17
(c) Kanehisa Laboratories

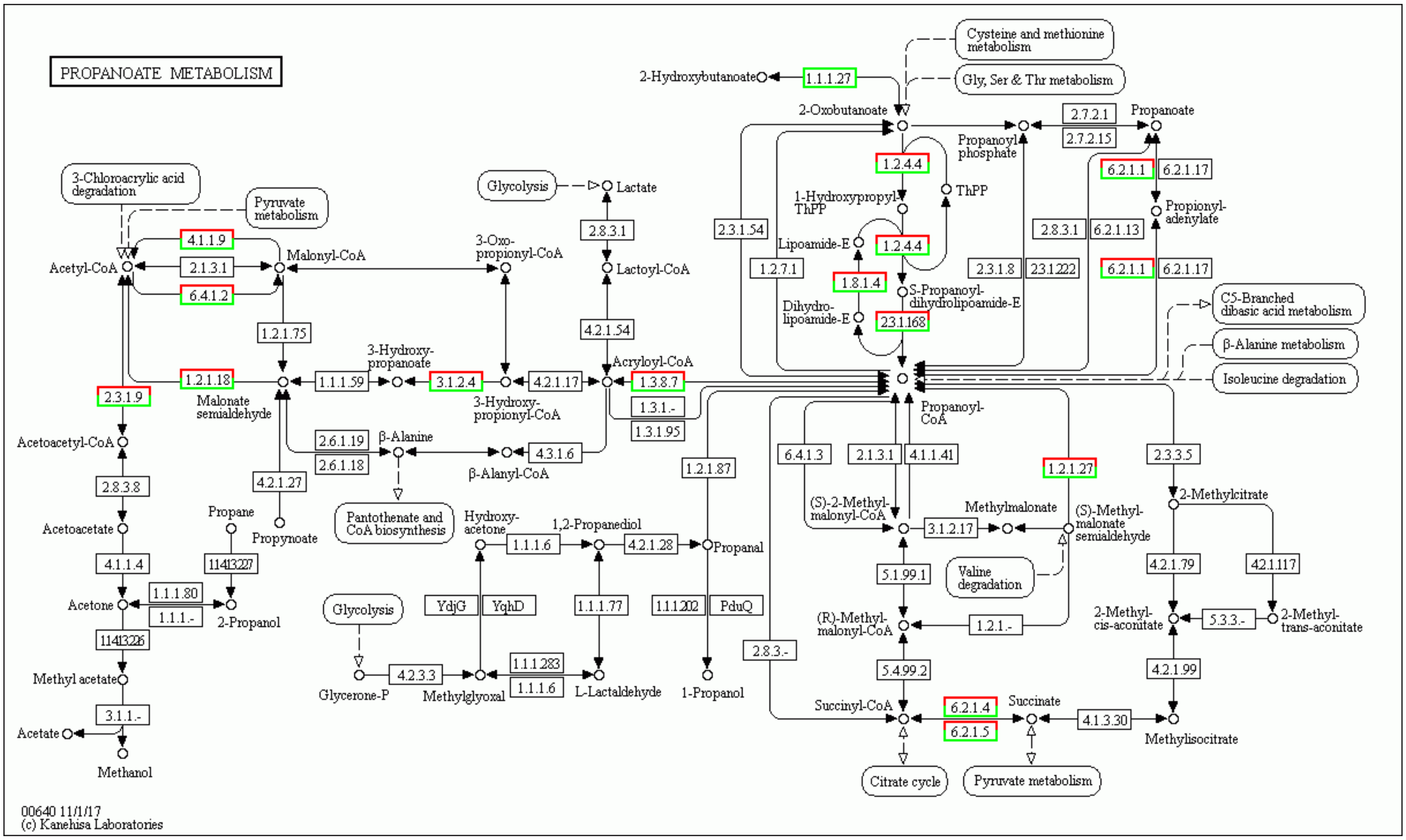
PROPANOATE METABOLISM
2-Hydroxybutanoate
1.1.1.27
Cysteine and methionine metabolism
Gly, Ser & Thr metabolism
2-Oxobutanoate
2.7.2.1
2.7.2.15
Propanoate
Propanoyl phosphate
1.2.4.4
6.2.1.1
6.2.1.17
1-Hydroxypropyl-ThPP
ThPP
Propionyl-adenylate
3-Chloroacrylic acid degradation
Pyruvate metabolism
Glycolysis
Lactate
2.8.3.1
2.3.1.54
Lipoamide-E
1.2.4.4
2.8.3.1
6.2.1.13
4.1.1.9
3-Oxo-propionyl-CoA
Acetyl-CoA
2.1.3.1
Malonyl-CoA
Lactoyl-CoA
1.2.7.1
1.8.1.4
2.3.1.8
2.3.1.222
6.2.1.1
6.2.1.17
6.4.1.2
S-Propanoyl-dihydrolipoamide-E
C5-Branched dibasic acid metabolism
Dihydro-lipoamide-E
2.3.1.168
1.2.1.75
4.2.1.54
β-Alanine metabolism
3-Hydroxy-propanoate
Acryloyl-CoA
1.2.1.18
1.1.1.59
3.1.2.4
4.2.1.17
1.3.8.7
Isoleucine degradation
2.3.1.9
Malonate semialdehyde
3-Hydroxy-propionyl-CoA
1.3.1.-
Propanoyl-CoA
1.3.1.95
Acetoacetyl-CoA
2.6.1.19
β-Alanine
2.6.1.18
4.3.1.6
β-Alanyl-CoA
1.2.1.87
6.4.1.3
2.1.3.1
4.1.1.41
1.2.1.27
2.3.3.5
2.8.3.8
4.2.1.27
2-Methylcitrate
Propane
(S)-2-Methyl-malonyl-CoA
Methylmalonate
(S)-Methyl-malonate semialdehyde
Acetoacetate
Propynoate
Pantothenate and CoA biosynthesis
Hydroxy-acetone
1,2-Propanediol
1.1.1.6
4.2.1.28
Propanal
3.1.2.17
4.1.1.4
1.1.1.27
4.2.1.79
4.2.1.117
5.1.99.1
Valine degradation
1.1.1.80
Acetone
1.1.1.-
2-Propanol
Glycolysis
YdjG
YqhD
1.1.1.77
1.1.1.202
PduQ
(R)-Methyl-malonyl-CoA
1.2.1.-
2-Methyl-cis-aconitate
5.3.3.-
2-Methyl-trans-aconitate
1.14.13.226
2.8.3.-
Methyl acetate
4.2.3.3
1.1.1.283
5.4.99.2
4.2.1.99
Glycerone-P
Methylglyoxal
1.1.1.6
L-Lactaldehyde
1-Propanol
Succinyl-CoA
6.2.1.4
Succinate
4.1.3.30
3.1.1.-
6.2.1.5
Methylisocitrate
Acetate
Methanol
Citrate cycle
Pyruvate metabolism
00640 11/1/17
(c) Kanehisa Laboratories

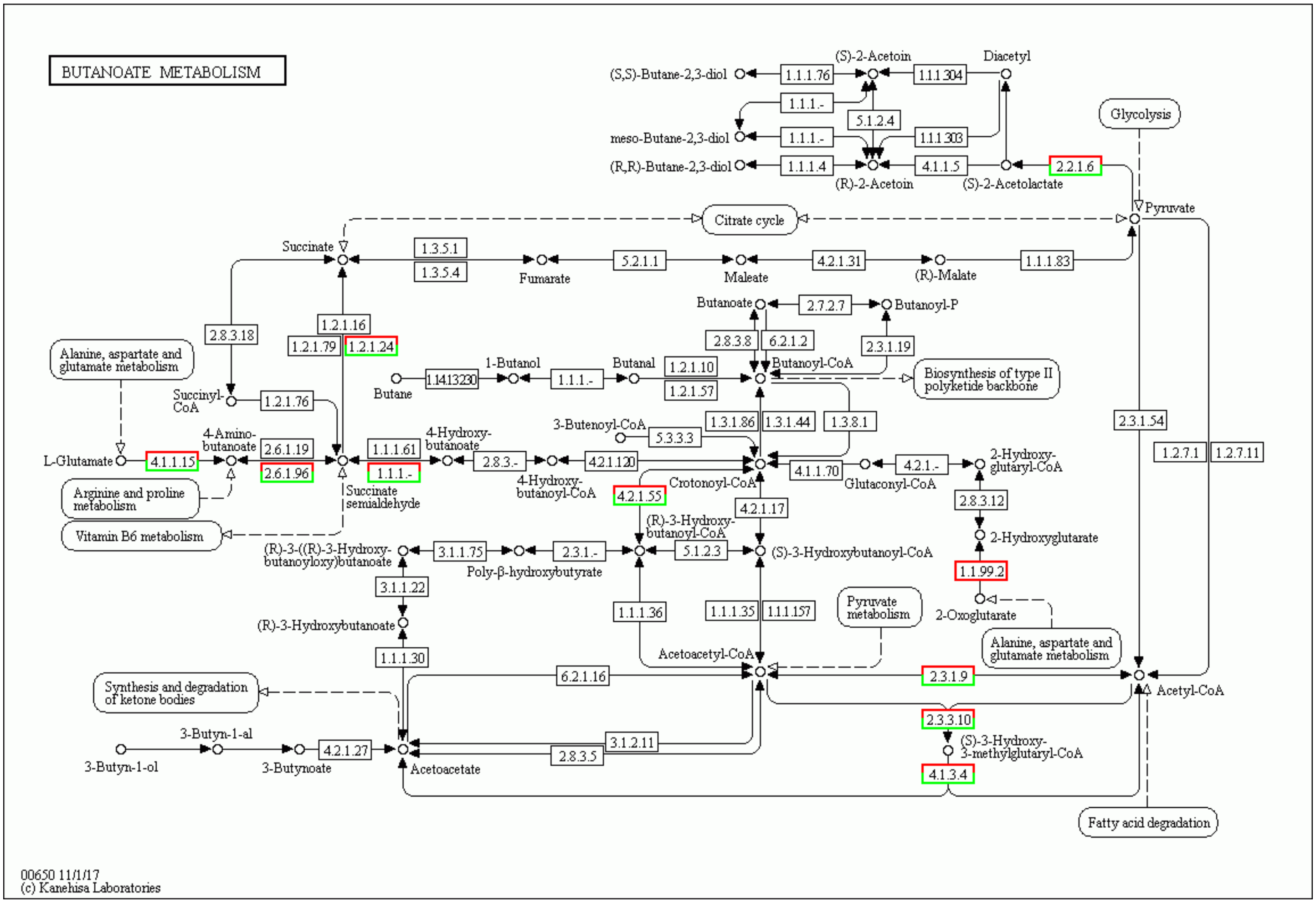
BUTANOATE METABOLISM
(S)-2-Acetoin
Diacetyl
(S,S)-Butane-2,3-diol
1.1.1.76
1.1.1.304
1.1.1.-
5.1.2.4
Glycolysis
meso-Butane-2,3-diol
1.1.1.-
1.1.1.303
(R,R)-Butane-2,3-diol
1.1.1.4
4.1.1.5
2.2.1.6
(R)-2-Acetoin
(S)-2-Acetolactate
Pyruvate
Citrate cycle
Succinate
1.3.5.1
1.3.5.4
Fumarate
5.2.1.1
Maleate
4.2.1.31
(R)-Malate
1.1.1.83
Butanoate
2.7.2.7
Butanoyl-P
2.8.3.18
1.2.1.16
1.2.1.79
1.2.1.24
2.8.3.8
6.2.1.2
2.3.1.19
Alanine, aspartate and glutamate metabolism
1-Butanol
Butanal
1.2.1.10
Butanoyl-CoA
Biosynthesis of type II polyketide backbone
Butane
1.14.13.230
1.1.1.-
1.2.1.57
Succinyl-CoA
1.2.1.76
3-Butenoyl-CoA
1.3.1.86
1.3.1.44
1.3.8.1
2.3.1.54
1.2.7.1
1.2.7.11
4-Amino-butanoate
2.6.1.19
1.1.1.61
4-Hydroxy-butanoate
5.3.3.3
L-Glutamate
4.1.1.15
2.6.1.96
1.1.1.-
2.8.3.-
4.2.1.120
4.1.1.70
4.2.1.-
2-Hydroxy-glutaryl-CoA
Arginine and proline metabolism
Succinate semialdehyde
4-Hydroxy-butanoyl-CoA
4.2.1.55
Crotonoyl-CoA
Glutaconyl-CoA
2.8.3.12
4.2.1.17
Vitamin B6 metabolism
(R)-3-Hydroxy-butanoyl-CoA
2-Hydroxyglutarate
(R)-3-((R)-3-Hydroxy-butanoyloxy)butanoate
3.1.1.75
2.3.1.-
5.1.2.3
(S)-3-Hydroxybutanoyl-CoA
Poly-β-hydroxybutyrate
1.1.99.2
3.1.1.22
1.1.1.36
1.1.1.35
1.1.1.157
Pyruvate metabolism
2-Oxoglutarate
(R)-3-Hydroxybutanoate
Alanine, aspartate and glutamate metabolism
1.1.1.30
Acetoacetyl-CoA
6.2.1.16
2.3.1.9
Acetyl-CoA
Synthesis and degradation of ketone bodies
2.3.3.10
(S)-3-Hydroxy-3-methylglutaryl-CoA
3.1.2.11
3-Butyn-1-al
4.2.1.27
2.8.3.5
4.1.3.4
3-Butyn-1-ol
3-Butynoate
Acetoacetate
Fatty acid degradation
00650 11/1/17
(c) Kanehisa Laboratories

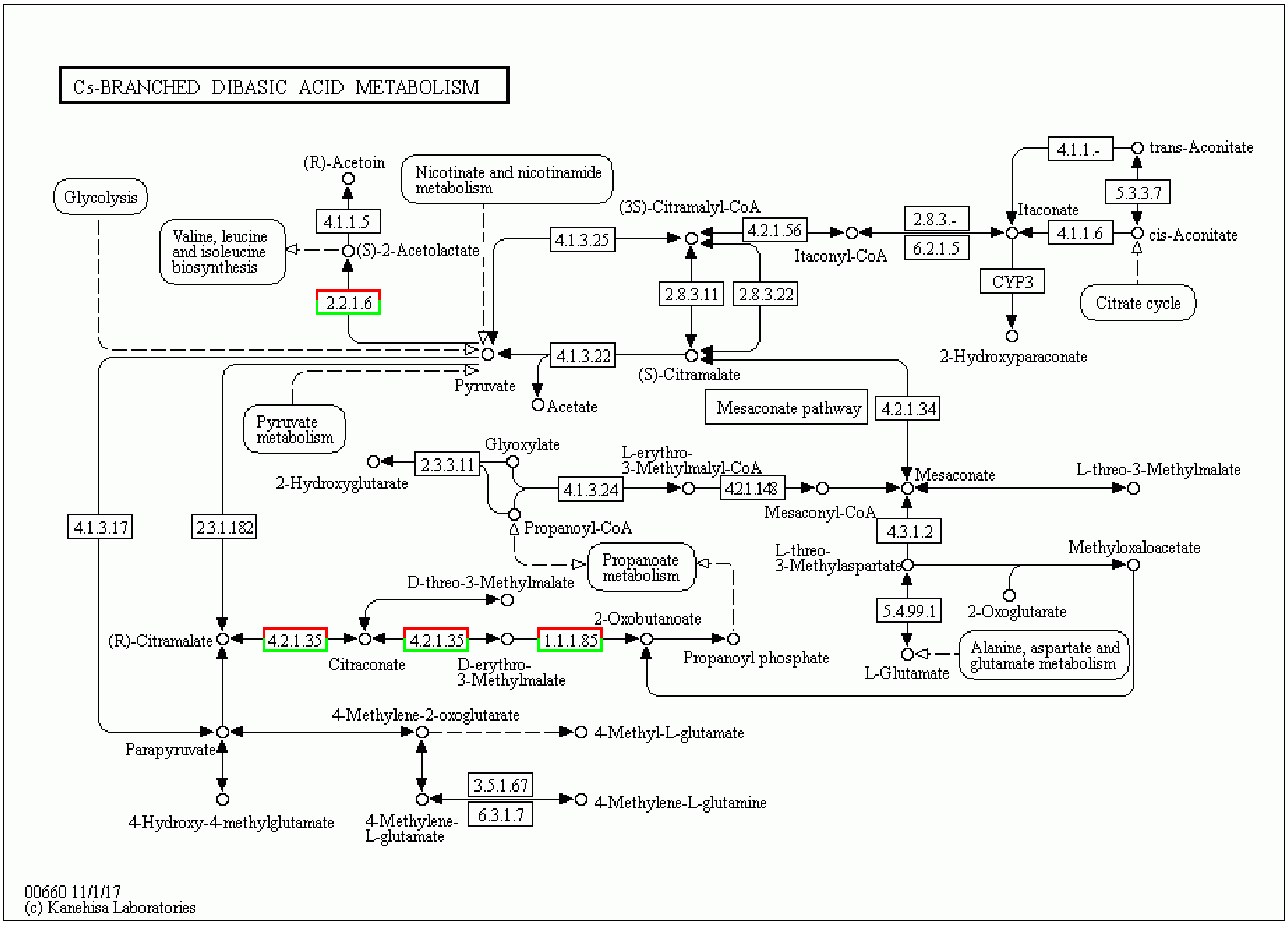
C5-BRANCHED DIBASIC ACID METABOLISM
Glycolysis
(R)-Acetoin
4.1.1.5
Valine, leucine and isoleucine biosynthesis
(S)-2-Acetolactate
2.2.1.6
Nicotinate and nicotinamide metabolism
4.1.3.25
(3S)-Citramalyl-CoA
4.2.1.56
Itaconyl-CoA
2.8.3.-
6.2.1.5
2.8.3.11
2.8.3.22
4.1.1.-
trans-Aconitate
5.3.3.7
Itaconate
4.1.1.6
cis-Aconitate
CYP3
Citrate cycle
2-Hydroxyparaconate
Pyruvate
4.1.3.22
(S)-Citramalate
Acetate
Pyruvate metabolism
Mesaconate pathway
4.2.1.34
Glyoxylate
2.3.3.11
2-Hydroxyglutarate
L-erythro-3-Methylmalyl-CoA
4.1.3.24
4.2.1.148
Mesaconate
L-threo-3-Methylmalate
Mesaconyl-CoA
Propanoyl-CoA
4.3.1.2
L-threo-3-Methylaspartate
Methyloxaloacetate
4.1.3.17
2.3.1.182
Propanoate metabolism
D-threo-3-Methylmalate
2-Oxobutanoate
5.4.99.1
2-Oxoglutarate
(R)-Citramalate
4.2.1.35
Citraconate
4.2.1.35
D-erythro-3-Methylmalate
1.1.1.85
Propanoyl phosphate
L-Glutamate
Alanine, aspartate and glutamate metabolism
4-Methylene-2-oxoglutarate
Parapyruvate
4-Methyl-L-glutamate
3.5.1.67
6.3.1.7
4-Methylene-L-glutamine
4-Hydroxy-4-methylglutamate
4-Methylene-L-glutamate
00660 11/1/17
(c) Kanehisa Laboratories

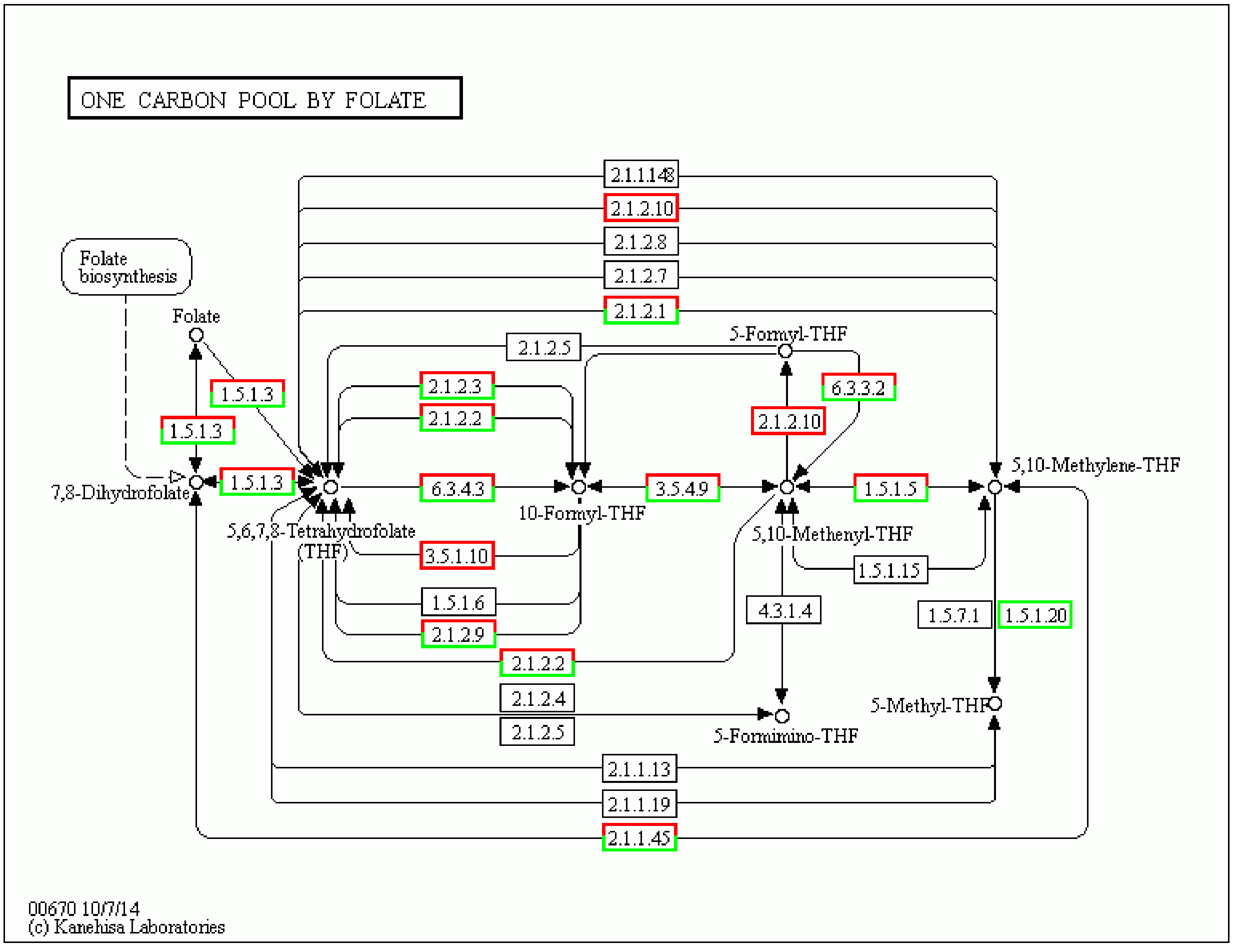
ONE CARBON POOL BY FOLATE
2.1.1.148
2.1.2.10
2.1.2.8
2.1.2.7
2.1.2.1
Folate biosynthesis
Folate
2.1.2.5
5-Formyl-THF
1.5.1.3
2.1.2.3
2.1.2.2
6.3.3.2
1.5.1.3
2.1.2.10
5,10-Methylene-THF
7,8-Dihydrofolate
1.5.1.3
6.3.4.3
3.5.4.9
1.5.1.5
5,6,7,8-Tetrahydrofolate (THF)
10-Formyl-THF
5,10-Methenyl-THF
3.5.1.10
1.5.1.15
1.5.1.6
4.3.1.4
1.5.7.1
1.5.1.20
2.1.2.9
2.1.2.2
2.1.2.4
5-Methyl-THF
2.1.2.5
5-Formimino-THF
2.1.1.13
2.1.1.19
2.1.1.45
00670 10/7/14
(c) Kanehisa Laboratories

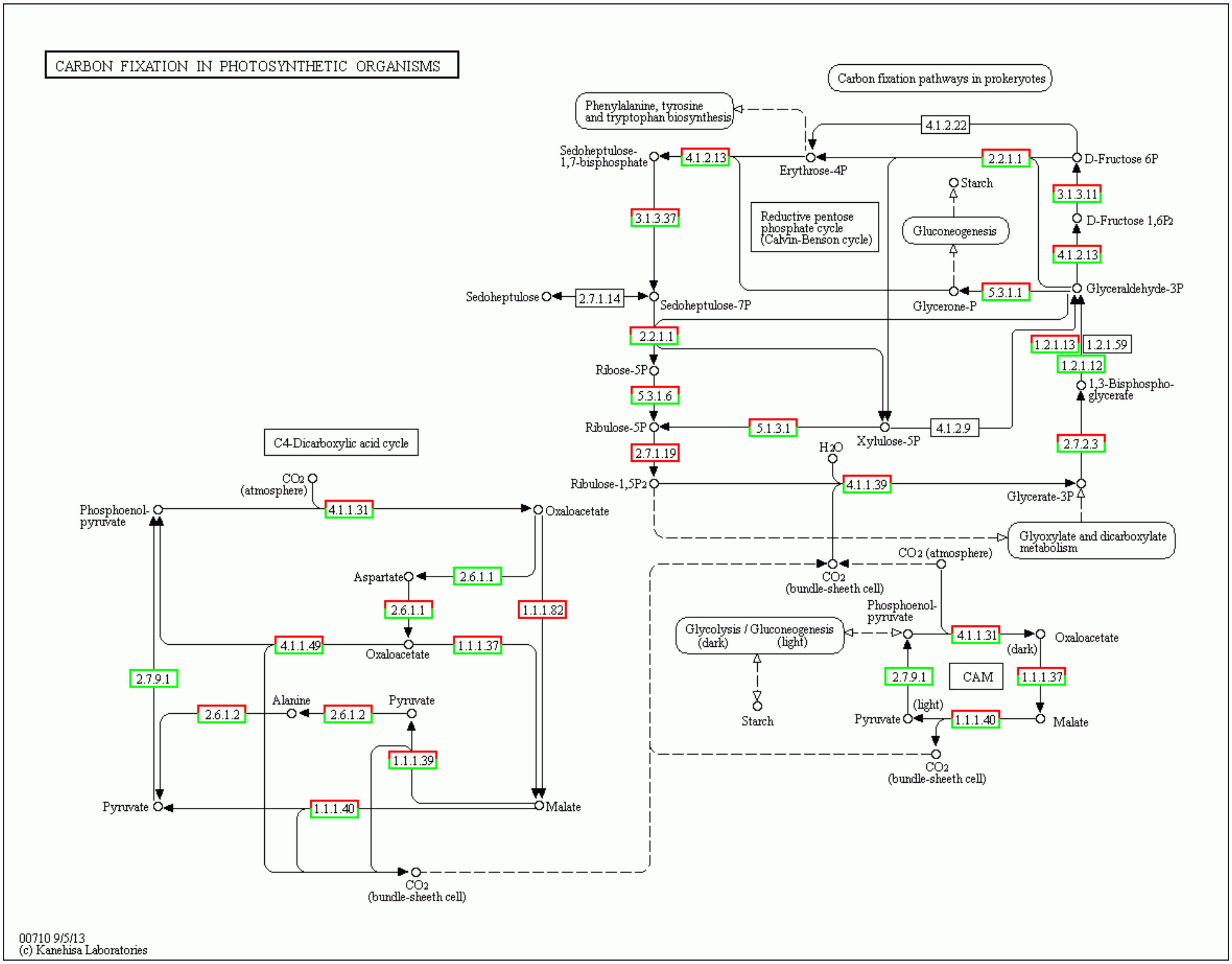
CARBON FIXATION IN PHOTOSYNTHETIC ORGANISMS
Carbon fixation pathways in prokaryotes
Phenylalanine, tyrosine and tryptophan biosynthesis
4.1.2.22
Sedoheptulose-1,7-bisphosphate
4.1.2.13
Erythrose-4P
2.2.1.1
D-Fructose 6P
3.1.3.37
Starch
3.1.3.11
Reductive pentose phosphate cycle (Calvin-Benson cycle)
Gluconeogenesis
D-Fructose 1,6P2
4.1.2.13
Sedoheptulose
2.7.1.14
Sedoheptulose-7P
Glycerone-P
5.3.1.1
Glyceraldehyde-3P
2.2.1.1
1.2.1.13
1.2.1.59
1.2.1.12
Ribose-5P
1,3-Bisphospho-glycerate
5.3.1.6
Ribulose-5P
5.1.3.1
Xylulose-5P
4.1.2.9
C4-Dicarboxylic acid cycle
2.7.1.19
H2O
2.7.2.3
CO2 (atmosphere)
Ribulose-1,5P2
4.1.1.39
Glycerate-3P
Phosphoenol-pyruvate
4.1.1.31
Oxaloacetate
Glyoxylate and dicarboxylate metabolism
CO2 (atmosphere)
Aspartate
2.6.1.1
CO2 (bundle-sheeth cell)
2.6.1.1
1.1.1.82
Phosphoenol-pyruvate
Glycolysis / Gluconeogenesis (dark) (light)
4.1.1.31
Oxaloacetate
4.1.1.49
Oxaloacetate
1.1.1.37
(dark)
2.7.9.1
2.7.9.1
CAM
1.1.1.37
Alanine
Pyruvate
2.6.1.2
2.6.1.2
Starch
(light)
Pyruvate
1.1.1.40
Malate
1.1.1.39
CO2 (bundle-sheeth cell)
Pyruvate
1.1.1.40
Malate
CO2 (bundle-sheeth cell)
00710 9/5/13
(c) Kanehisa Laboratories

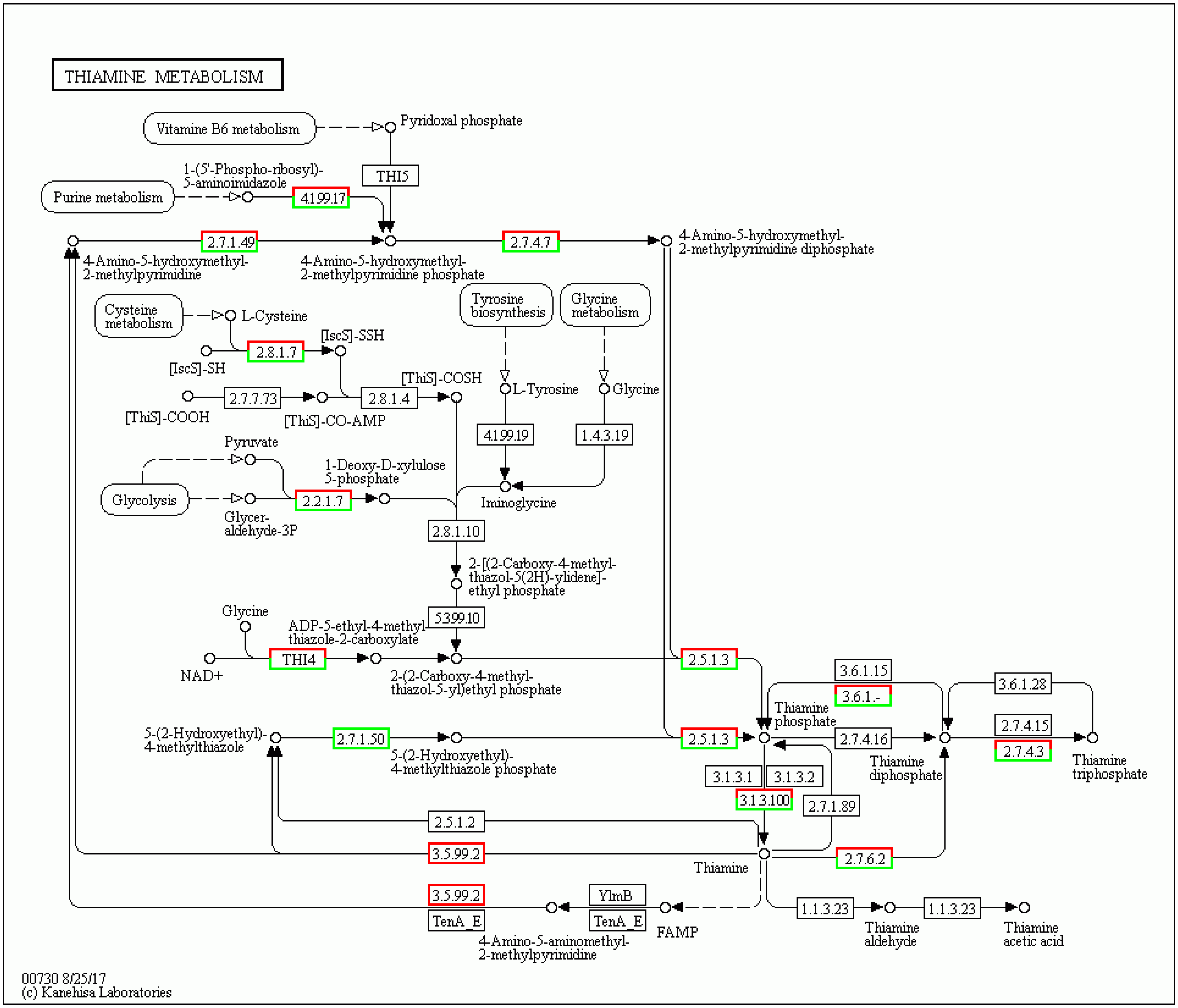
THIAMINE METABOLISM
Vitamine B6 metabolism
Pyridoxal phosphate
THI5
1-(5'-Phospho-ribosyl)-5-aminoimidazole
Purine metabolism
4.1.99.17
2.7.1.49
2.7.4.7
4-Amino-5-hydroxymethyl-2-methylpyrimidine
4-Amino-5-hydroxymethyl-2-methylpyrimidine phosphate
4-Amino-5-hydroxymethyl-2-methylpyrimidine diphosphate
Cysteine metabolism
L-Cysteine
Tyrosine biosynthesis
Glycine metabolism
2.8.1.7
[IscS]-SSH
[IscS]-SH
[ThiS]-COSH
2.7.7.73
2.8.1.4
[ThiS]-COOH
[ThiS]-CO-AMP
L-Tyrosine
Glycine
4.1.99.19
1.4.3.19
Pyruvate
1-Deoxy-D-xylulose 5-phosphate
Glycolysis
2.2.1.7
Iminoglycine
Glycer-aldehyde-3P
2.8.1.10
2-[(2-Carboxy-4-methyl-thiazol-5(2H)-ylidene]-ethyl phosphate
Glycine
5.3.99.10
ADP-5-ethyl-4-methylthiazole-2-carboxylate
NAD+
THI4
2.5.1.3
2-(2-Carboxy-4-methyl-thiazol-5-yl)ethyl phosphate
3.6.1.15
3.6.1.-
3.6.1.28
Thiamine phosphate
2.7.4.15
5-(2-Hydroxyethyl)-4-methylthiazole
2.7.1.50
2.5.1.3
2.7.4.16
Thiamine triphosphate
2.7.4.3
5-(2-Hydroxyethyl)-4-methylthiazole phosphate
Thiamine diphosphate
3.1.3.1
3.1.3.2
3.1.3.100
2.7.1.89
2.5.1.2
3.5.99.2
2.7.6.2
Thiamine
3.5.99.2
YlmB
TenA_E
TenA_E
1.1.3.23
1.1.3.23
4-Amino-5-aminomethyl-2-methylpyrimidine
FAMP
Thiamine aldehyde
Thiamine acetic acid
00730 8/25/17
(c) Kanehisa Laboratories

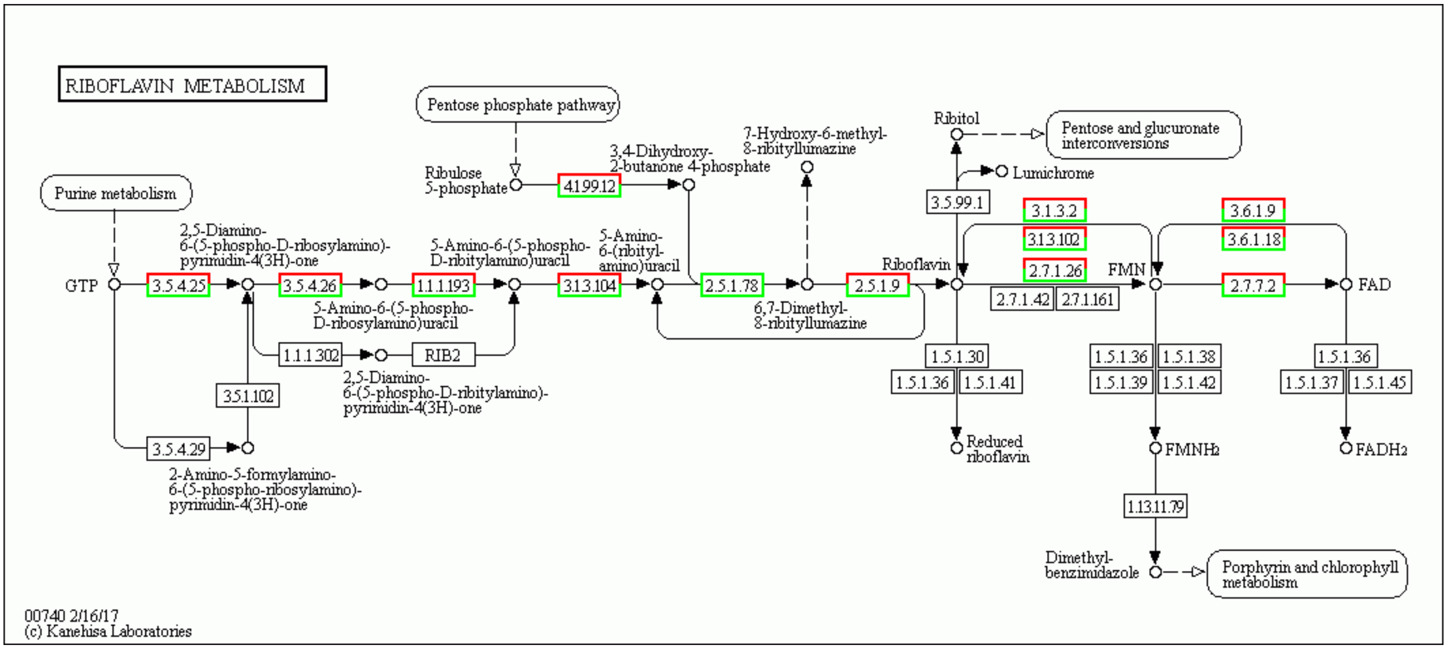
RIBOFLAVIN METABOLISM
Pentose phosphate pathway
Ribitol
Pentose and glucuronate interconversions
Lumichrome
7-Hydroxy-6-methyl-8-ribityllumazine
3,4-Dihydroxy-2-butanone 4-phosphate
Ribulose 5-phosphate
4.1.99.12
Purine metabolism
3.5.99.1
3.1.3.2
3.6.1.9
2,5-Diamino-6-(5-phospho-D-ribosylamino)-pyrimidin-4(3H)-one
5-Amino-6-(5-phospho-D-ribitylamino)uracil
5-Amino-6-(ribityl-amino)uracil
3.1.3.102
3.6.1.18
2.7.1.26
Riboflavin
FMN
GTP
3.5.4.25
3.5.4.26
1.1.1.193
3.1.3.104
2.5.1.78
2.5.1.9
2.7.7.2
FAD
5-Amino-6-(5-phospho-D-ribosylamino)uracil
6,7-Dimethyl-8-ribityllumazine
2.7.1.42
2.7.1.161
1.1.1.302
RIB2
1.5.1.30
1.5.1.36
1.5.1.38
1.5.1.36
2,5-Diamino-6-(5-phospho-D-ribitylamino)-pyrimidin-4(3H)-one
1.5.1.36
1.5.1.41
1.5.1.39
1.5.1.42
1.5.1.37
1.5.1.45
3.5.1.102
3.5.4.29
Reduced riboflavin
FMNH2
FADH2
2-Amino-5-formylamino-6-(5-phospho-ribosylamino)-pyrimidin-4(3H)-one
1.13.11.79
Dimethyl-benzimidazole
Porphyrin and chlorophyll metabolism
00740 2/16/17
(c) Kanehisa Laboratories

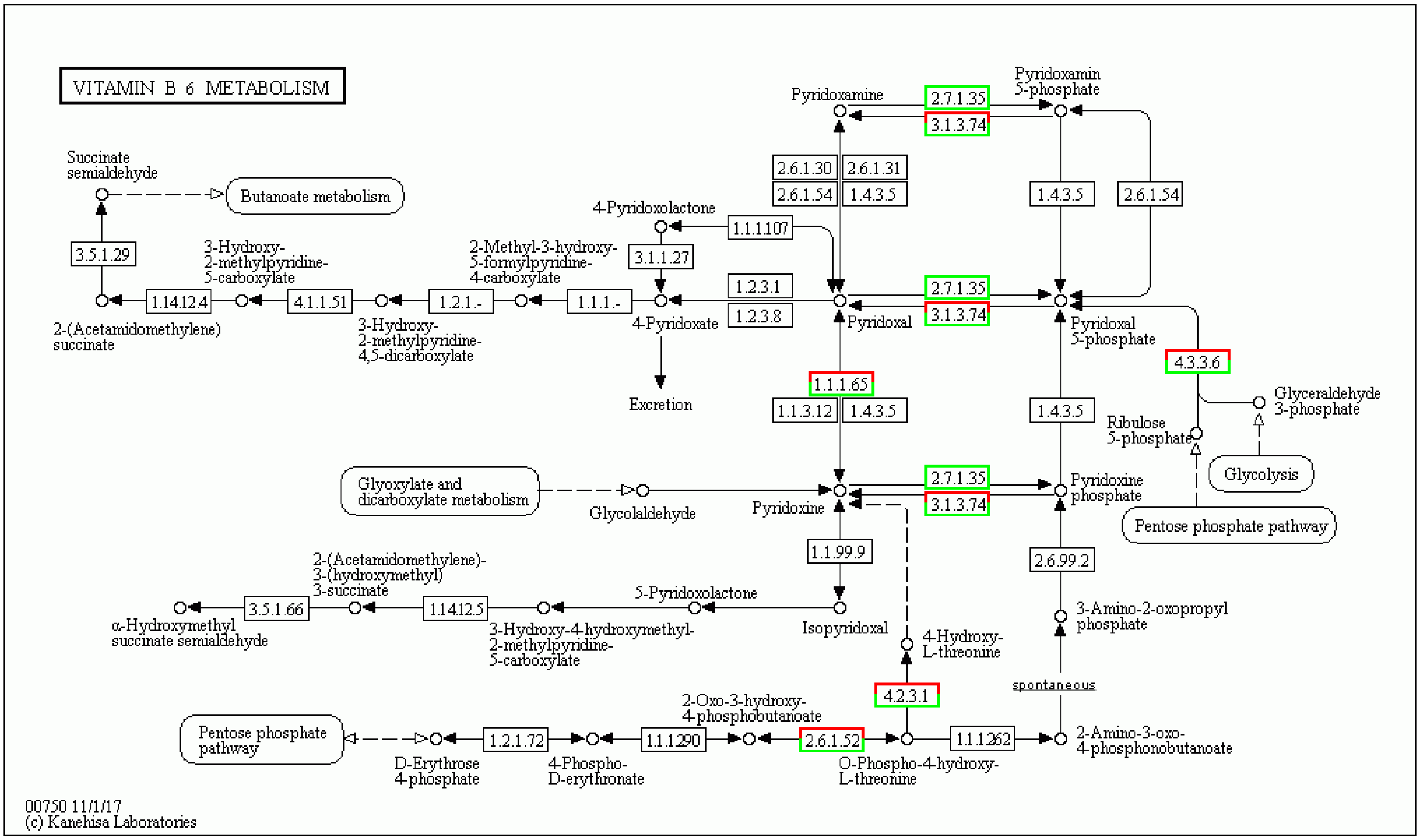
VITAMIN B 6 METABOLISM
Succinate semialdehyde
Butanoate metabolism
3.5.1.29
3-Hydroxy-2-methylpyridine-5-carboxylate
2-Methyl-3-hydroxy-5-formylpyridine-4-carboxylate
4-Pyridoxolactone
1.1.1.107
3.1.1.27
1.14.12.4
4.1.1.51
1.2.1.-
1.1.1.-
2-(Acetamidomethylene) succinate
3-Hydroxy-2-methylpyridine-4,5-dicarboxylate
4-Pyridoxate
Excretion
1.2.3.1
1.2.3.8
Pyridoxamine
2.7.1.35
3.1.3.74
Pyridoxamin 5-phosphate
2.6.1.30
2.6.1.31
2.6.1.54
1.4.3.5
2.6.1.54
Pyridoxal
Pyridoxal 5-phosphate
4.3.3.6
1.1.1.65
1.1.3.12
Ribulose 5-phosphate
Glyceraldehyde 3-phosphate
Glycolysis
Glyoxylate and dicarboxylate metabolism
Glycolaldehyde
Pyridoxine
Pyridoxine phosphate
Pentose phosphate pathway
2-(Acetamidomethylene)-3-(hydroxymethyl) 3-succinate
3.5.1.66
1.14.12.5
5-Pyridoxolactone
1.1.99.9
2.6.99.2
α-Hydroxymethyl succinate semialdehyde
3-Hydroxy-4-hydroxymethyl-2-methylpyridine-5-carboxylate
Isopyridoxal
4-Hydroxy-L-threonine
3-Amino-2-oxopropyl phosphate
spontaneous
4.2.3.1
2-Oxo-3-hydroxy-4-phosphobutanoate
Pentose phosphate pathway
1.2.1.72
1.1.1.290
2.6.1.52
1.1.1.262
D-Erythrose 4-phosphate
4-Phospho-D-erythronate
O-Phospho-4-hydroxy-L-threonine
2-Amino-3-oxo-4-phosphonobutanoate
00750 11/1/17
(c) Kanehisa Laboratories

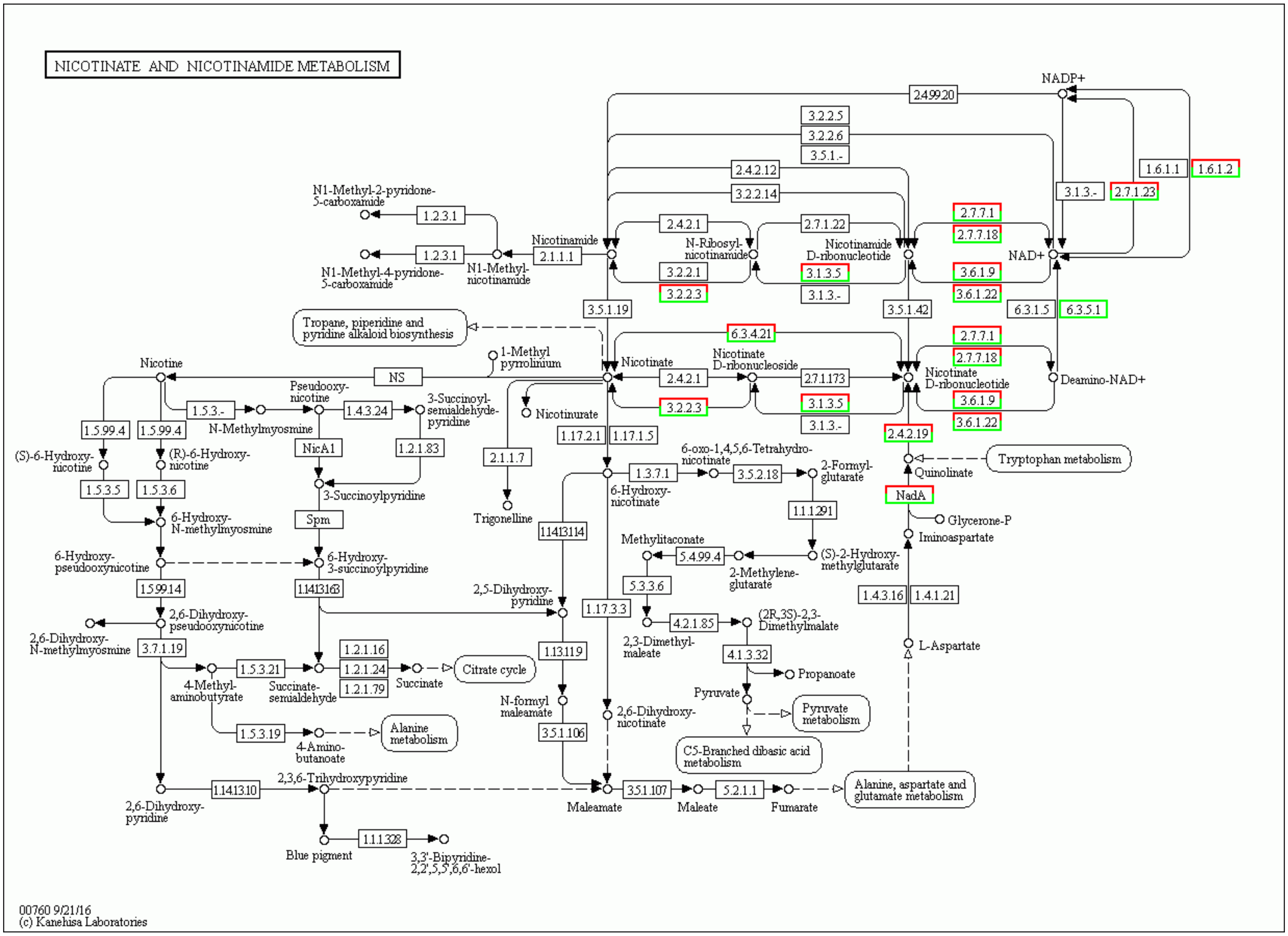
NICOTINATE AND NICOTINAMIDE METABOLISM
NADP+
2.4.99.20
3.2.2.5
3.2.2.6
3.5.1.-
2.4.2.12
3.2.2.14
1.6.1.1
1.6.1.2
3.1.3.-
2.7.1.23
N1-Methyl-2-pyridone-5-carboxamide
1.2.3.1
1.2.3.1
N1-Methyl-4-pyridone-5-carboxamide
N1-Methyl-nicotinamide
2.1.1.1
Nicotinamide
2.4.2.1
N-Ribosyl-nicotinamide
2.7.1.22
Nicotinamide D-ribonucleotide
2.7.7.1
2.7.7.18
NAD+
3.2.2.1
3.2.2.3
3.1.3.5
3.1.3.-
3.6.1.9
3.6.1.22
3.5.1.19
3.5.1.42
6.3.1.5
6.3.5.1
Tropane, piperidine and pyridine alkaloid biosynthesis
1-Methyl pyrrolinium
6.3.4.21
2.7.7.1
2.7.7.18
Nicotine
NS
Nicotinate
Nicotinate D-ribonucleoside
2.4.2.1
2.7.1.173
Nicotinate D-ribonucleotide
Deamino-NAD+
Pseudooxy-nicotine
3-Succinoyl-semialdehyde-pyridine
Nicotinurate
3.2.2.3
3.1.3.5
3.6.1.9
1.5.3.-
1.4.3.24
3.1.3.-
3.6.1.22
1.5.99.4
1.5.99.4
N-Methylmyosmine
NicA1
1.2.1.83
1.17.2.1
1.17.1.5
2.4.2.19
(S)-6-Hydroxy-nicotine
(R)-6-Hydroxy-nicotine
2.1.1.7
6-oxo-1,4,5,6-Tetrahydro-nicotinate
Tryptophan metabolism
1.3.7.1
3.5.2.18
2-Formyl-glutarate
Quinolinate
1.5.3.5
1.5.3.6
3-Succinoylpyridine
Trigonelline
6-Hydroxy-nicotinate
NadA
Glycerone-P
6-Hydroxy-N-methylmyosmine
Spm
1.14.13.114
1.1.1.291
Iminoaspartate
Methylitaconate
6-Hydroxy-pseudooxynicotine
6-Hydroxy-3-succinoylpyridine
5.4.99.4
(S)-2-Hydroxy-methylglutarate
2-Methylene-glutarate
5.3.3.6
1.5.99.14
1.14.13.163
2,5-Dihydroxy-pyridine
1.17.3.3
1.4.3.16
1.4.1.21
2,6-Dihydroxy-pseudooxynicotine
4.2.1.85
(2R,3S)-2,3-Dimethylmalate
2,6-Dihydroxy-N-methylmyosmine
3.7.1.19
1.2.1.16
2,3-Dimethyl-maleate
1.13.11.9
4.1.3.32
L-Aspartate
1.5.3.21
1.2.1.24
Citrate cycle
Propanoate
4-Methyl-aminobutyrate
Succinate-semialdehyde
1.2.1.79
Succinate
Pyruvate
N-formyl maleamate
2,6-Dihydroxy-nicotinate
Pyruvate metabolism
1.5.3.19
Alanine metabolism
4-Amino-butanoate
3.5.1.106
C5-Branched dibasic acid metabolism
2,3,6-Trihydroxypyridine
1.14.13.10
3.5.1.107
5.2.1.1
Alanine, aspartate and glutamate metabolism
2,6-Dihydroxy-pyridine
Maleamate
Maleate
Fumarate
1.1.13.28
Blue pigment
3,3'-Bipyridine-2,2',5,5',6,6'-hexol
00760 9/21/16
(c) Kanehisa Laboratories

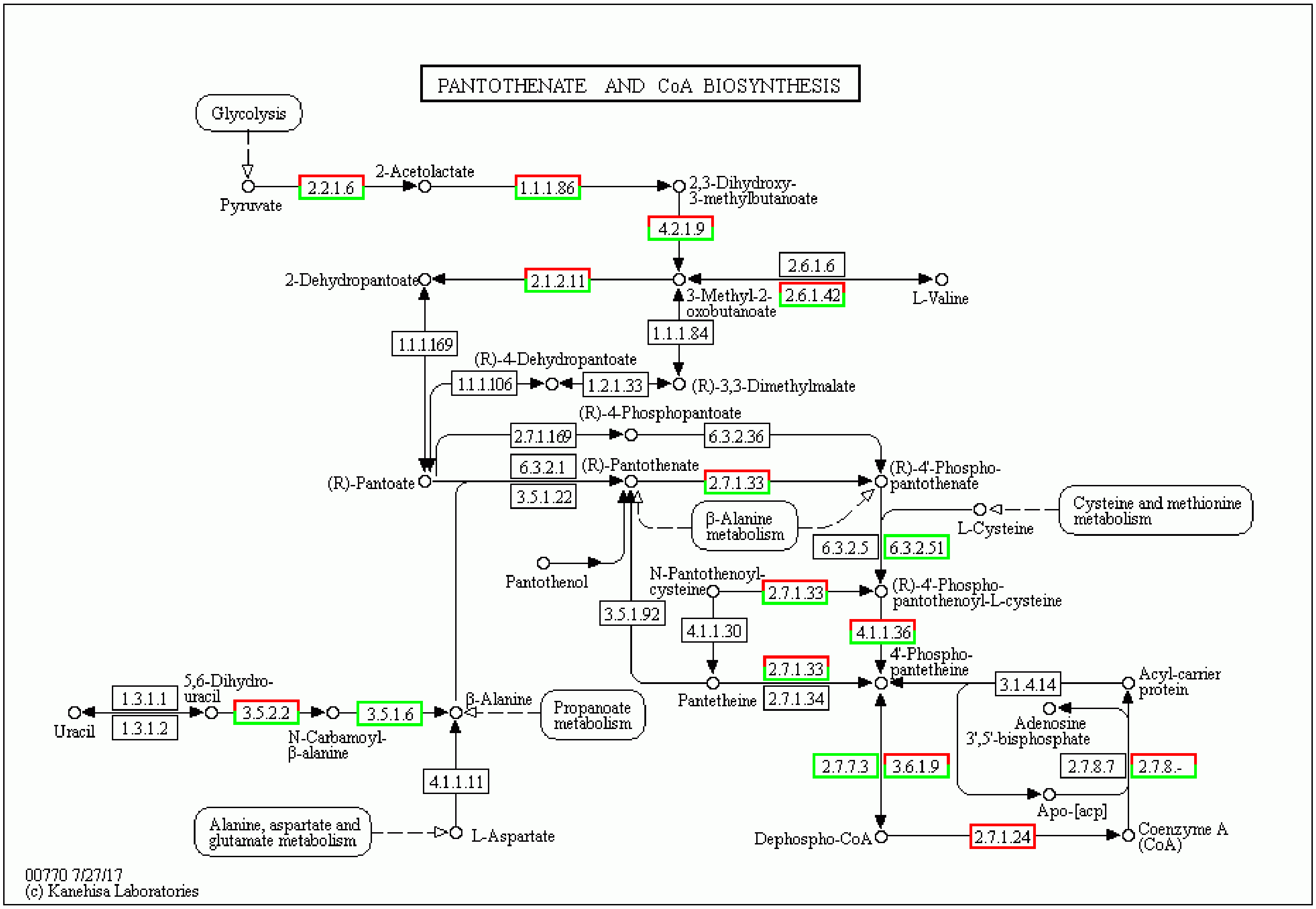
PANTOTHENATE AND CoA BIOSYNTHESIS
Glycolysis
Pyruvate
2.2.1.6
2-Acetolactate
1.1.1.86
2,3-Dihydroxy-3-methylbutanoate
4.2.1.9
2.6.1.6
2-Dehydropantoate
2.1.2.11
3-Methyl-2-oxobutanoate
2.6.1.42
L-Valine
1.1.1.169
1.1.1.84
(R)-4-Dehydropantoate
1.1.1.106
1.2.1.33
(R)-3,3-Dimethylmalate
(R)-4-Phosphopantoate
2.7.1.169
6.3.2.36
(R)-Pantoate
6.3.2.1
(R)-Pantothenate
2.7.1.33
(R)-4'-Phosphopantothenate
3.5.1.22
β-Alanine metabolism
L-Cysteine
Cysteine and methionine metabolism
6.3.2.5
6.3.2.51
Pantothenol
N-Pantothenoyl-cysteine
2.7.1.33
(R)-4'-Phosphopantothenoyl-L-cysteine
3.5.1.92
4.1.1.30
4.1.1.36
4'-Phosphopantetheine
2.7.1.33
Pantetheine
2.7.1.34
3.1.4.14
Acyl-carrier protein
1.3.1.1
5,6-Dihydrouracil
Uracil
1.3.1.2
3.5.2.2
N-Carbamoyl-β-alanine
3.5.1.6
β-Alanine
Propanoate metabolism
Adenosine 3',5'-bisphosphate
2.7.7.3
3.6.1.9
2.7.8.7
2.7.8.-
Apo-[acp]
4.1.1.11
Alanine, aspartate and glutamate metabolism
L-Aspartate
Dephospho-CoA
2.7.1.24
Coenzyme A (CoA)
00770 7/27/17
(c) Kanehisa Laboratories

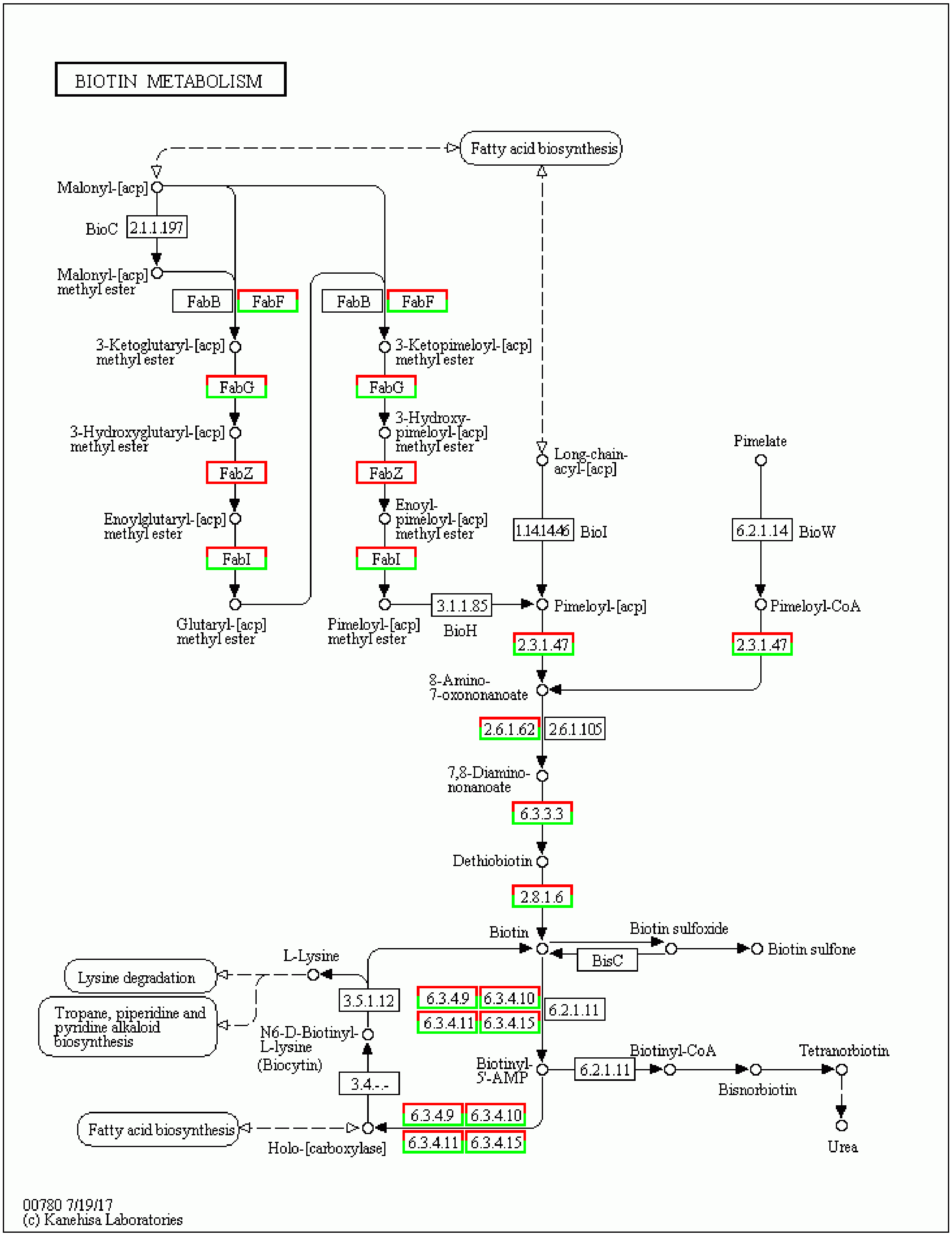
BIOTIN METABOLISM
Fatty acid biosynthesis
Malonyl-[acp]
BioC 2.1.1.197
Malonyl-[acp] methyl ester
FabB
FabF
FabB
FabF
3-Ketoglutaryl-[acp] methyl ester
3-Ketopimeloyl-[acp] methyl ester
FabG
FabG
3-Hydroxyglutaryl-[acp] methyl ester
3-Hydroxy-pimeloyl-[acp] methyl ester
FabZ
FabZ
Enoylglutaryl-[acp] methyl ester
Enoyl-pimeloyl-[acp] methyl ester
FabI
FabI
Glutaryl-[acp] methyl ester
Pimeloyl-[acp] methyl ester
3.1.1.85
BioH
Long-chain-acyl-[acp]
1.14.14.46 BioI
Pimelate
6.2.1.14 BioW
Pimeloyl-[acp]
Pimeloyl-CoA
2.3.1.47
2.3.1.47
8-Amino-7-oxononanoate
2.6.1.62
2.6.1.105
7,8-Diamino-nonanoate
6.3.3.3
Dethiobiotin
2.8.1.6
Biotin
Biotin sulfoxide
Biotin sulfone
BisC
L-Lysine
Lysine degradation
Tropane, piperidine and pyridine alkaloid biosynthesis
3.5.1.12
6.3.4.9
6.3.4.10
6.3.4.11
6.3.4.15
6.2.1.11
N6-D-Biotinyl-L-lysine (Biocytin)
3.4.-.-
Biotinyl-5'-AMP
6.2.1.11
Biotinyl-CoA
Bisnorbiotin
Tetranorbiotin
Urea
6.3.4.9
6.3.4.10
6.3.4.11
6.3.4.15
Fatty acid biosynthesis
Holo-[carboxylase]
00780 7/19/17
(c) Kanehisa Laboratories

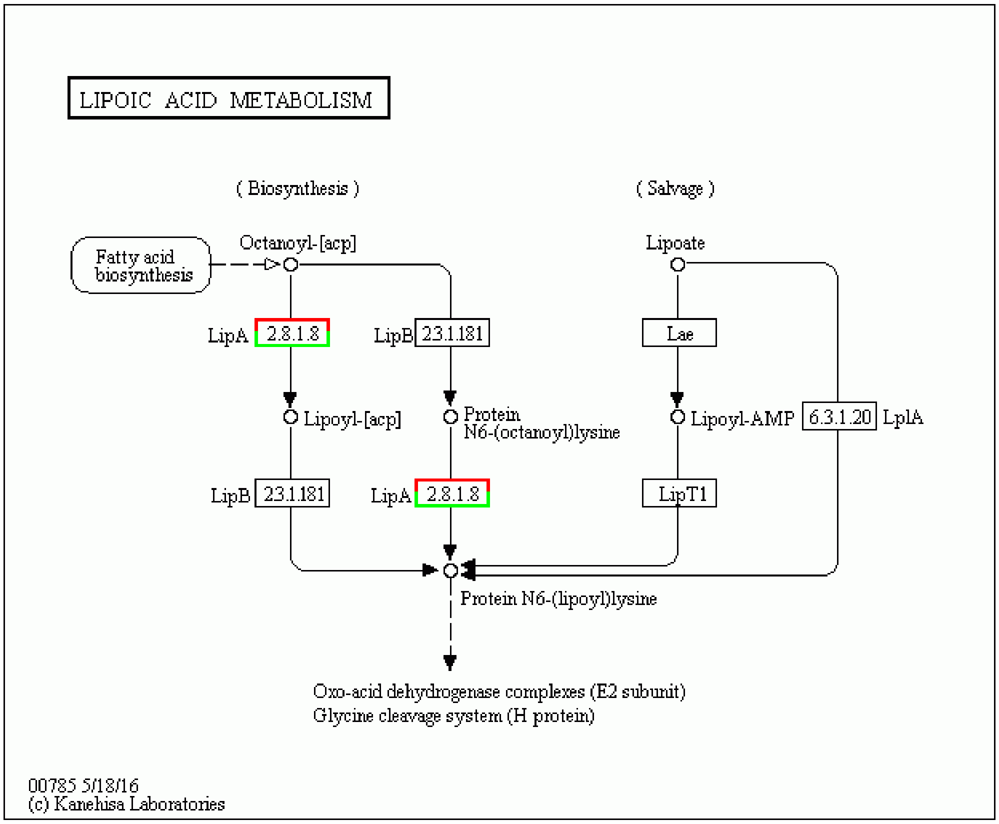
LIPOIC ACID METABOLISM
(Biosynthesis)
(Salvage)
Fatty acid biosynthesis
Octanoyl-[acp]
Lipoate
LipA 2.8.1.8
LipB 2.3.1.181
Lae
Lipoyl-[acp]
Protein N6-(octanoyl)lysine
Lipoyl-AMP
6.3.1.20 LplA
LipB 2.3.1.181
LipA 2.8.1.8
LipT1
Protein N6-(lipoyl)lysine
Oxo-acid dehydrogenase complexes (E2 subunit)
Glycine cleavage system (H protein)
00785 5/18/16
(c) Kanehisa Laboratories

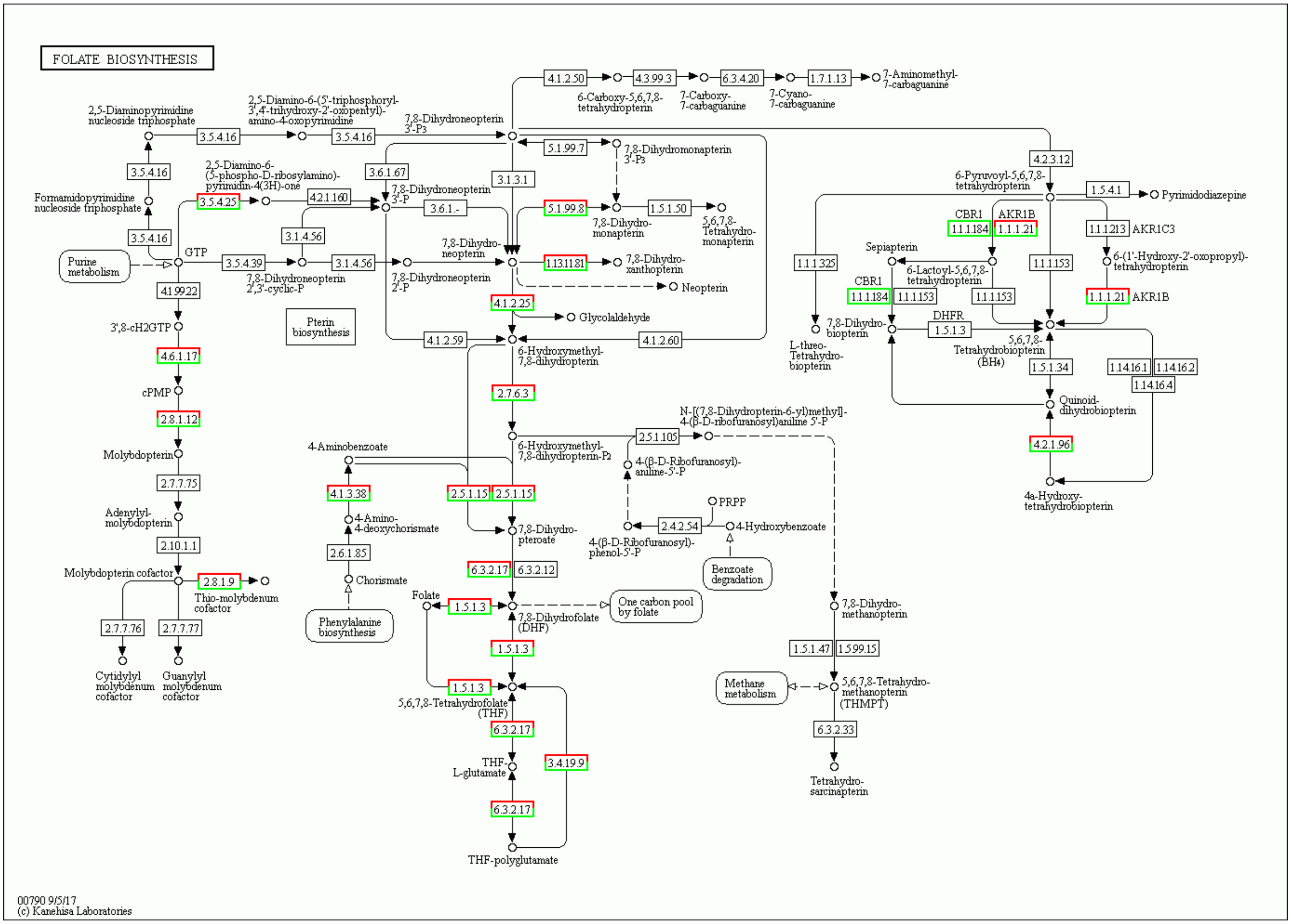
FOLATE BIOSYNTHESIS
2,5-Diaminopyrimidine nucleoside triphosphate
2,5-Diamino-6-(5'-triphosphoryl-3',4'-trihydroxy-2'-oxopentyl)-amino-4-oxopyrimidine
7,8-Dihydroneopterin 3'-P3
3.5.4.16
3.5.4.16
3.5.4.16
3.5.4.16
Formamidopyrimidine nucleoside triphosphate
2,5-Diamino-6-(5-phospho-D-ribosylamino)-pyrimidin-4(3H)-one
3.5.4.25
4.2.1.160
3.6.1.67
7,8-Dihydroneopterin 3'-P
3.6.1.-
3.1.3.1
3.1.4.56
3.1.4.56
Purine metabolism
GTP
3.5.4.39
7,8-Dihydroneopterin 2',3'-cyclic-P
7,8-Dihydroneopterin 2'-P
7,8-Dihydroneopterin
4.1.99.22
3',8-cH2GTP
4.6.1.17
cPMP
2.8.1.12
Molybdopterin
2.7.7.75
Adenylyl-molybdopterin
2.10.1.1
Molybdopterin cofactor
2.8.1.9
Thio-molybdenum cofactor
2.7.7.76
2.7.7.77
Cytidylyl molybdenum cofactor
Guanylyl molybdenum cofactor
Pterin biosynthesis
4.1.2.50
6-Carboxy-5,6,7,8-tetrahydropterin
4.3.99.3
7-Carboxy-7-carbaguanine
6.3.4.20
7-Cyano-7-carbaguanine
1.7.1.13
7-Aminomethyl-7-carbaguanine
5.1.99.7
7,8-Dihydromonapterin 3'-P3
5.1.99.8
7,8-Dihydro-monapterin
1.5.1.50
5,6,7,8-Tetrahydro-monapterin
1.13.11.81
7,8-Dihydro-xanthopterin
Neopterin
4.1.2.25
Glycolaldehyde
4.1.2.59
4.1.2.60
6-Hydroxymethyl-7,8-dihydropterin
2.7.6.3
6-Hydroxymethyl-7,8-dihydropterin-P2
2.5.1.105
N-[(7,8-Dihydropterin-6-yl)methyl]-4-(β-D-ribofuranosyl)aniline 5'-P
4-(β-D-Ribofuranosyl)-aniline-5'-P
PRPP
2.4.2.54
4-Hydroxybenzoate
4-(β-D-Ribofuranosyl)-phenol-5'-P
Benzoate degradation
4-Aminobenzoate
4.1.3.38
4-Amino-4-deoxychorismate
2.6.1.85
Chorismate
Phenylalanine biosynthesis
2.5.1.15
2.5.1.15
7,8-Dihydro-pteroate
6.3.2.17
6.3.2.12
Folate
1.5.1.3
7,8-Dihydrofolate (DHF)
One carbon pool by folate
1.5.1.3
1.5.1.3
5,6,7,8-Tetrahydrofolate (THF)
6.3.2.17
THF-L-glutamate
3.4.19.9
6.3.2.17
THF-polyglutamate
7,8-Dihydro-methanopterin
1.5.1.47
1.5.99.15
Methane metabolism
5,6,7,8-Tetrahydro-methanopterin (THMPT)
6.3.2.33
Tetrahydro-sarcinapterin
4.2.3.12
6-Pyruvoyl-5,6,7,8-tetrahydropterin
1.5.4.1
Pyrimidodiazepine
CBR1
AKR1B
1.1.1.184
1.1.1.21
1.1.1.213 AKR1C3
Sepiapterin
6-Lactoyl-5,6,7,8-tetrahydropterin
6-(1'-Hydroxy-2'-oxopropyl)-tetrahydropterin
1.1.1.325
CBR1
1.1.1.184
1.1.1.153
1.1.1.153
1.1.1.153
1.1.1.21 AKR1B
7,8-Dihydro-biopterin
DHFR
1.5.1.3
L-threo-Tetrahydro-biopterin
5,6,7,8-Tetrahydrobiopterin (BH4)
1.5.1.34
1.14.16.1
1.14.16.2
1.14.16.4
Quinoid-dihydrobiopterin
4.2.1.96
4a-Hydroxy-tetrahydrobiopterin
00790 9/5/17
(c) Kanehisa Laboratories

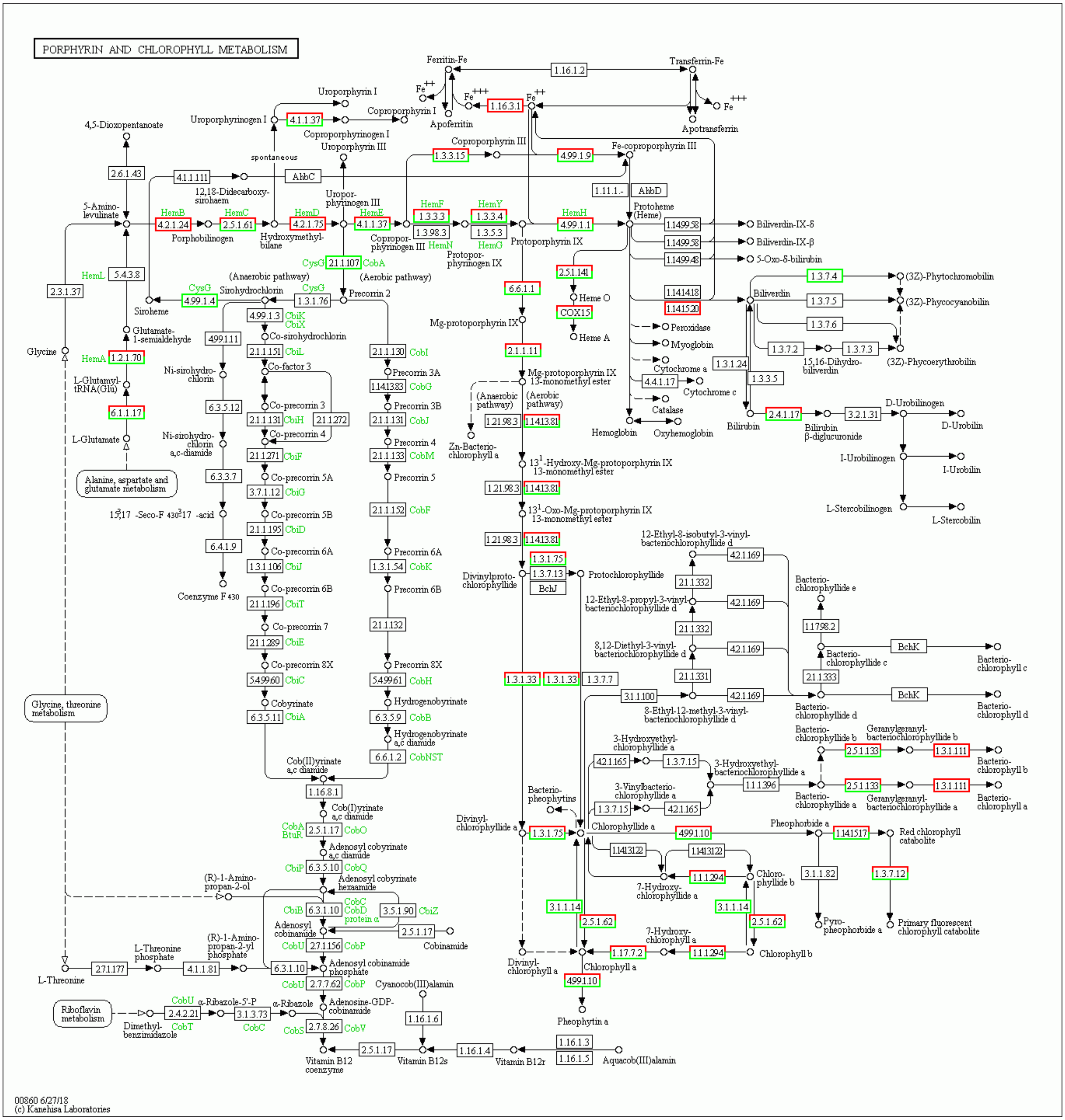
PORPHYRIN AND CHLOROPHYLL METABOLISM
Ferritin-Fe
1.16.1.2
Transferrin-Fe
Fe++
Fe+++
1.16.3.1
Fe++
Fe+++
Apoferritin
Apotransferrin
Uroporphyrin I
Uroporphyrinogen I
4.1.1.37
Coproporphyrin I
Coproporphyrinogen I
Uroporphyrin III
4,5-Dioxopentanoate
2.6.1.43
spontaneous
4.1.1.111
AhbC
12,18-Didecarboxy-siroheam
Coproporphyrin III
1.3.3.15
4.99.1.9
Fe-coproporphyrin III
1.11.1.-
AhbD
Protoheme (Heme)
5-Amino-levulinate
HemB
4.2.1.24
HemC
2.5.1.61
HemD
4.2.1.75
HemE
4.1.1.37
HemF
1.3.3.3
1.3.98.3
HemY
1.3.3.4
1.3.5.3
HemH
4.99.1.1
Porphobilinogen
Hydroxymethyl-bilane
Uropor-phyrinogen III
Copropor-phyrinogen III
HemN
Protopor-phyrinogen IX
HemG
Protoporphyrin IX
1.14.99.58
Biliverdin-IX-δ
1.14.99.58
Biliverdin-IX-β
1.14.99.48
5-Oxo-δ-bilirubin
HemL
5.4.3.8
CysG
2.1.1.107
CobA
(Anaerobic pathway)
(Aerobic pathway)
2.5.1.141
1.3.7.4
(3Z)-Phytochromobilin
2.3.1.37
CysG
4.99.1.4
Sirohydrochlorin
CysG
1.3.1.76
Precorrin 2
6.6.1.1
Heme O
1.14.14.18
Biliverdin
1.3.7.5
(3Z)-Phycocyanobilin
Siroheme
4.99.1.3
CbiK
CbiX
1.14.15.20
COX15
Glutamate-1-semialdehyde
4.99.1.11
Co-sirohydrochlorin
Mg-protoporphyrin IX
Heme A
Peroxidase
1.3.7.6
Glycine
2.1.1.151
CbiL
2.1.1.130
CobI
2.1.1.11
Myoglobin
HemA
1.2.1.70
Ni-sirohydro-chlorin
Co-factor 3
Precorrin 3A
Mg-protoporphyrin IX 13-monomethyl ester
Cytochrome a
1.3.1.24
1.3.7.2
1.3.7.3
15,16-Dihydro-biliverdin
(3Z)-Phycoerythrobilin
L-Glutamyl-tRNA(Glu)
1.14.13.83
CobG
4.4.1.17
Cytochrome c
1.3.3.5
6.3.5.12
Co-precorrin 3
Precorrin 3B
(Anaerobic pathway)
(Aerobic pathway)
Catalase
6.1.1.17
2.1.1.131
CbiH
2.1.1.272
2.1.1.131
CobJ
1.21.98.3
1.14.13.81
2.4.1.17
3.2.1.31
D-Urobilinogen
Hemoglobin
Oxyhemoglobin
Bilirubin
Bilirubin β-diglucuronide
D-Urobilin
L-Glutamate
Ni-sirohydro-chlorin a,c-diamide
Co-precorrin 4
Precorrin 4
Zn-Bacterio-chlorophyll a
2.1.1.271
CbiF
2.1.1.133
CobM
I-Urobilinogen
Alanine, aspartate and glutamate metabolism
6.3.3.7
Co-precorrin 5A
Precorrin 5
13¹-Hydroxy-Mg-protoporphyrin IX 13-monomethyl ester
I-Urobilin
3.7.1.12
CbiG
1.21.98.3
1.14.13.81
L-Stercobilinogen
15¹,17³-Seco-F430-17³-acid
Co-precorrin 5B
2.1.1.152
CobF
13¹-Oxo-Mg-protoporphyrin IX 13-monomethyl ester
L-Stercobilin
2.1.1.195
CbiD
1.21.98.3
1.14.13.81
12-Ethyl-8-isobutyl-3-vinyl-bacteriochlorophyllide d
6.4.1.9
Co-precorrin 6A
Precorrin 6A
4.2.1.169
1.3.1.106
CbiJ
1.3.1.54
CobK
1.3.1.75
Divinylproto-chlorophyllide
1.3.7.13
Protochlorophyllide
BchJ
Bacterio-chlorophyllide e
Co-precorrin 6B
Precorrin 6B
2.1.1.332
Coenzyme F430
2.1.1.196
CbiT
12-Ethyl-8-propyl-3-vinyl-bacteriochlorophyllide d
4.2.1.169
2.1.1.132
2.1.1.332
1.17.98.2
Co-precorrin 7
8,12-Diethyl-3-vinyl-bacteriochlorophyllide d
4.2.1.169
BchK
2.1.1.289
CbiE
Bacterio-chlorophyllide c
Bacterio-chlorophyll c
Co-precorrin 8X
Precorrin 8X
2.1.1.331
2.1.1.333
5.4.99.60
CbiC
5.4.99.61
CobH
1.3.1.33
1.3.1.33
1.3.7.7
3.1.1.100
4.2.1.169
BchK
Glycine, threonine metabolism
Cobyrinate
Hydrogenobyrinate
8-Ethyl-12-methyl-3-vinyl-bacteriochlorophyllide d
Bacterio-chlorophyllide d
Bacterio-chlorophyll d
6.3.5.11
CbiA
6.3.5.9
CobB
Hydrogenobyrinate a,c diamide
3-Hydroxyethyl-chlorophyllide a
Bacterio-chlorophyllide b
Geranylgeranyl-bacteriochlorophyllide b
6.6.1.2
CobNST
4.2.1.165
1.3.7.15
3-Hydroxyethyl-bacteriochlorophyllide a
2.5.1.133
1.3.1.111
Bacterio-chlorophyll b
Cob(II)yrinate a,c diamide
1.1.1.396
1.16.8.1
Bacterio-pheophytins
3-Vinylbacterio-chlorophyllide a
2.5.1.133
1.3.1.111
Cob(I)yrinate a,c diamide
1.3.7.15
4.2.1.165
Bacterio-chlorophyllide a
Geranylgeranyl-bacteriochlorophyllide a
Bacterio-chlorophyll a
CobA
BtuR
2.5.1.17
CobO
Divinyl-chlorophyllide a
1.3.1.75
Chlorophyllide a
4.99.1.10
Pheophorbide a
1.14.15.17
Red chlorophyll catabolite
Adenosyl cobyrinate a,c diamide
1.14.13.122
1.14.13.122
CbiP
6.3.5.10
CobQ
Adenosyl cobyrinate hexaamide
Chloro-phyllide b
(R)-1-Amino-propan-2-ol
1.1.1.294
3.1.1.82
1.3.7.12
7-Hydroxy-chlorophyllide a
CbiB
6.3.1.10
CobC
CobD
protein α
3.5.1.90
CbiZ
3.1.1.14
3.1.1.14
Adenosyl cobinamide
2.5.1.17
Cobinamide
2.5.1.62
2.5.1.62
Pyro-pheophorbide a
Primary fluorescent chlorophyll catabolite
(R)-1-Amino-propan-2-yl phosphate
L-Threonine phosphate
CobU
2.7.1.156
CobP
7-Hydroxy-chlorophyll a
2.7.1.177
4.1.1.81
6.3.1.10
Adenosyl cobinamide phosphate
1.17.7.2
1.1.1.294
Divinyl-chlorophyll a
Chlorophyll a
Chlorophyll b
L-Threonine
CobU
2.7.7.62
CobP
Cyanocob(III)alamin
4.99.1.10
CobU
α-Ribazole-5'-P
α-Ribazole
Adenosine-GDP-cobinamide
Riboflavin metabolism
2.4.2.21
3.1.3.73
1.16.1.6
Pheophytin a
Dimethyl-benzimidazole
CobT
CobC
CobS
2.7.8.26
CobV
1.16.1.3
2.5.1.17
1.16.1.4
Vitamin B12 coenzyme
Vitamin B12s
Vitamin B12r
1.16.1.5
Aquacob(III)alamin
00860 6/27/18
(c) Kanehisa Laboratories

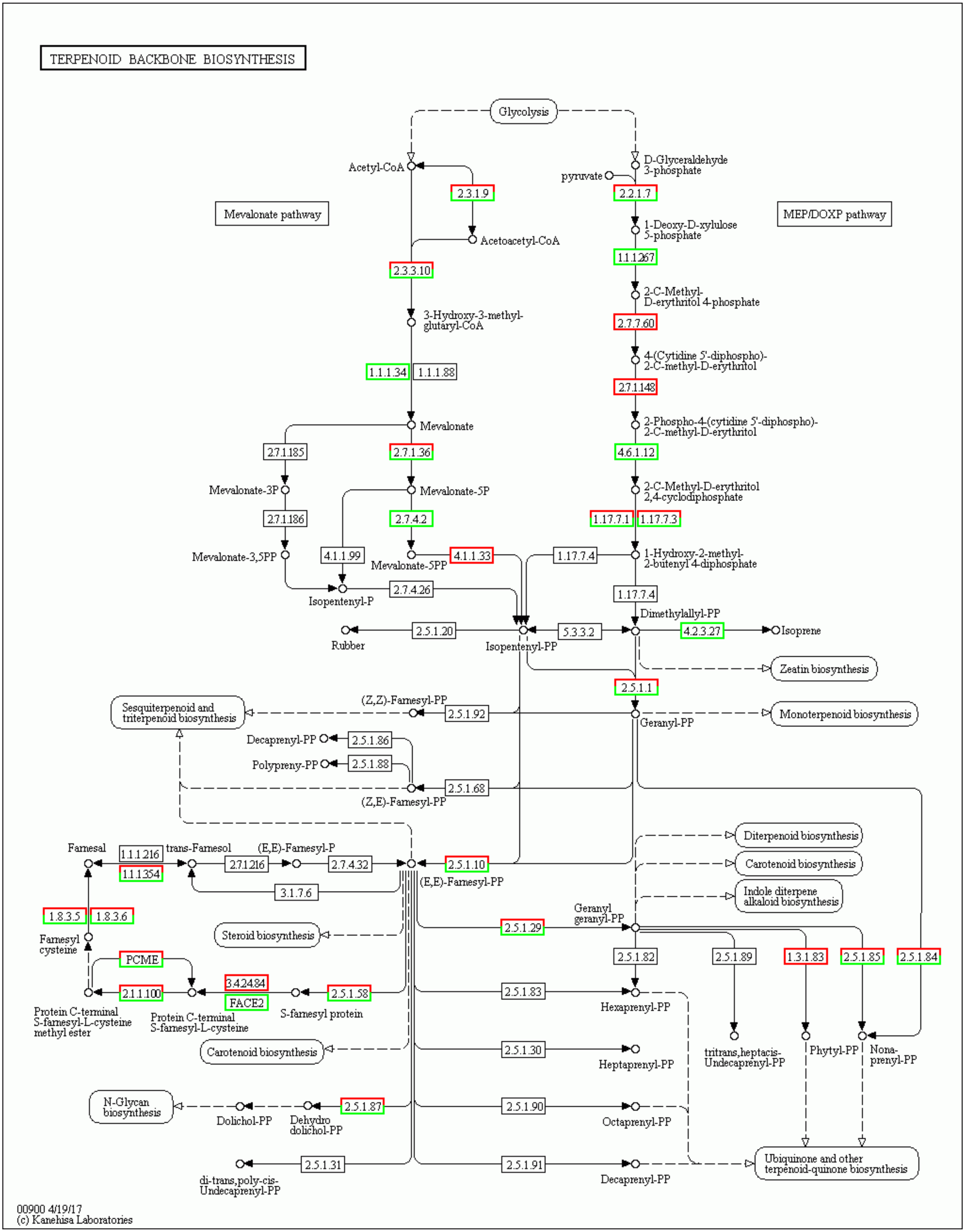
TERPENOID BACKBONE BIOSYNTHESIS
Glycolysis
Acetyl-CoA
D-Glyceraldehyde 3-phosphate
pyruvate
2.3.1.9
2.2.1.7
Mevalonate pathway
MEP/DOXP pathway
Acetoacetyl-CoA
1-Deoxy-D-xylulose 5-phosphate
2.3.3.10
1.1.1.267
2-C-Methyl-D-erythritol 4-phosphate
3-Hydroxy-3-methyl-glutaryl-CoA
2.7.7.60
4-(Cytidine 5'-diphospho)-2-C-methyl-D-erythritol
1.1.1.34
1.1.1.88
2.7.1.148
Mevalonate
2-Phospho-4-(cytidine 5'-diphospho)-2-C-methyl-D-erythritol
2.7.1.185
2.7.1.36
4.6.1.12
Mevalonate-3P
Mevalonate-5P
2-C-Methyl-D-erythritol 2,4-cyclodiphosphate
2.7.1.186
2.7.4.2
1.17.7.1
1.17.7.3
Mevalonate-3,5PP
4.1.1.99
4.1.1.33
1.17.7.4
1-Hydroxy-2-methyl-2-butenyl 4-diphosphate
Mevalonate-5PP
Isopentenyl-P
2.7.4.26
1.17.7.4
Dimethylallyl-PP
Rubber
2.5.1.20
Isopentenyl-PP
5.3.3.2
4.2.3.27
Isoprene
Zeatin biosynthesis
2.5.1.1
(Z,Z)-Farnesyl-PP
Sesquiterpenoid and triterpenoid biosynthesis
2.5.1.92
Geranyl-PP
Monoterpenoid biosynthesis
Decaprenyl-PP
2.5.1.86
Polypreny-PP
2.5.1.88
2.5.1.68
(Z,E)-Farnesyl-PP
Diterpenoid biosynthesis
Carotenoid biosynthesis
Farnesal
1.1.1.216
trans-Farnesol
(E,E)-Farnesyl-P
1.1.1.354
2.7.1.216
2.7.4.32
2.5.1.10
(E,E)-Farnesyl-PP
3.1.7.6
Indole diterpene alkaloid biosynthesis
1.8.3.5
1.8.3.6
Geranyl geranyl-PP
Farnesyl cysteine
Steroid biosynthesis
2.5.1.29
2.5.1.82
2.5.1.89
1.3.1.83
2.5.1.85
2.5.1.84
PCME
3.4.24.84
FACE2
2.1.1.100
2.5.1.58
2.5.1.83
Hexaprenyl-PP
Protein C-terminal S-farnesyl-L-cysteine methyl ester
Protein C-terminal S-farnesyl-L-cysteine
S-farnesyl protein
tritrans,heptacis-Undecaprenyl-PP
Phytyl-PP
Nona-prenyl-PP
Carotenoid biosynthesis
2.5.1.30
Heptaprenyl-PP
N-Glycan biosynthesis
Dolichol-PP
Dehydro dolichol-PP
2.5.1.87
2.5.1.90
Octaprenyl-PP
2.5.1.31
2.5.1.91
Ubiquinone and other terpenoid-quinone biosynthesis
di-trans,poly-cis-Undecaprenyl-PP
Decaprenyl-PP
00900 4/19/17
(c) Kanehisa Laboratories

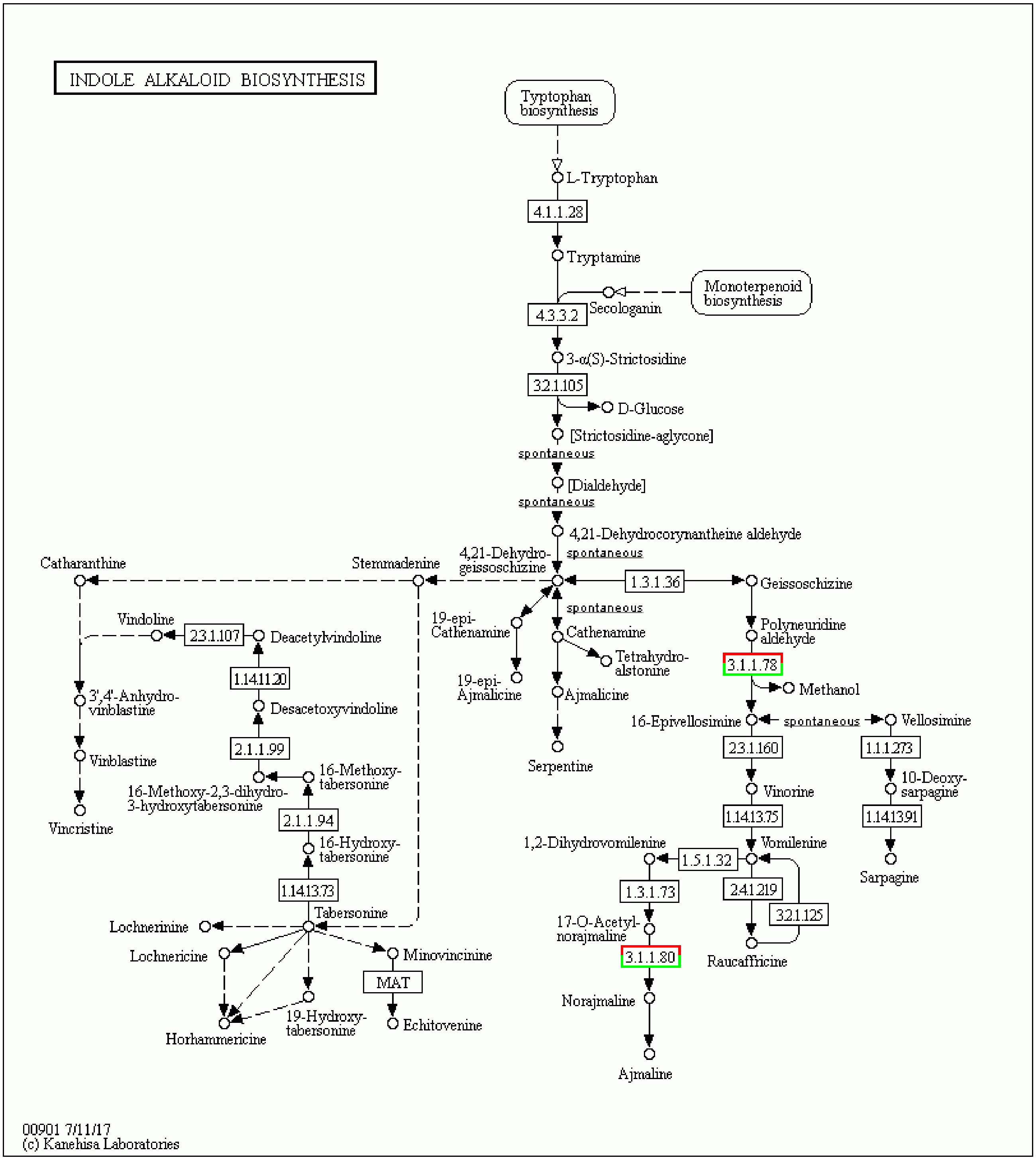
INDOLE ALKALOID BIOSYNTHESIS
Typtophan biosynthesis
L-Tryptophan
4.1.1.28
Tryptamine
Monoterpenoid biosynthesis
Secologanin
4.3.3.2
3-α(S)-Strictosidine
3.2.1.105
D-Glucose
[Strictosidine-aglycone]
spontaneous
[Dialdehyde]
spontaneous
4,21-Dehydrocorynantheine aldehyde
spontaneous
4,21-Dehydro-geissoschizine
1.3.1.36
Geissoschizine
Polyneuridine aldehyde
3.1.1.78
Methanol
16-Epivellosimine
spontaneous
Vellosimine
2.3.1.160
1.1.1.273
Vinorine
10-Deoxy-sarpagine
1.14.13.75
1.14.13.91
Vomilenine
Sarpagine
1,2-Dihydrovomilenine
1.5.1.32
2.4.1.219
3.2.1.125
1.3.1.73
Raucaffricine
17-O-Acetyl-norajmaline
3.1.1.80
Norajmaline
Ajmaline
Catharanthine
Stemmadenine
19-epi-Cathenamine
Cathenamine
Tetrahydro-alstonine
19-epi-Ajmalicine
Ajmalicine
Serpentine
spontaneous
Vindoline
2.3.1.107
Deacetylvindoline
1.14.11.20
Desacetoxyvindoline
2.1.1.99
3',4'-Anhydro-vinblastine
Vinblastine
Vincristine
16-Methoxy-tabersonine
16-Methoxy-2,3-dihydro-3-hydroxytabersonine
2.1.1.94
16-Hydroxy-tabersonine
1.14.13.73
Tabersonine
Lochnerinine
Lochnericine
Minovincinine
MAT
Echitovenine
19-Hydroxy-tabersonine
Horhammericine
00901 7/11/17
(c) Kanehisa Laboratories

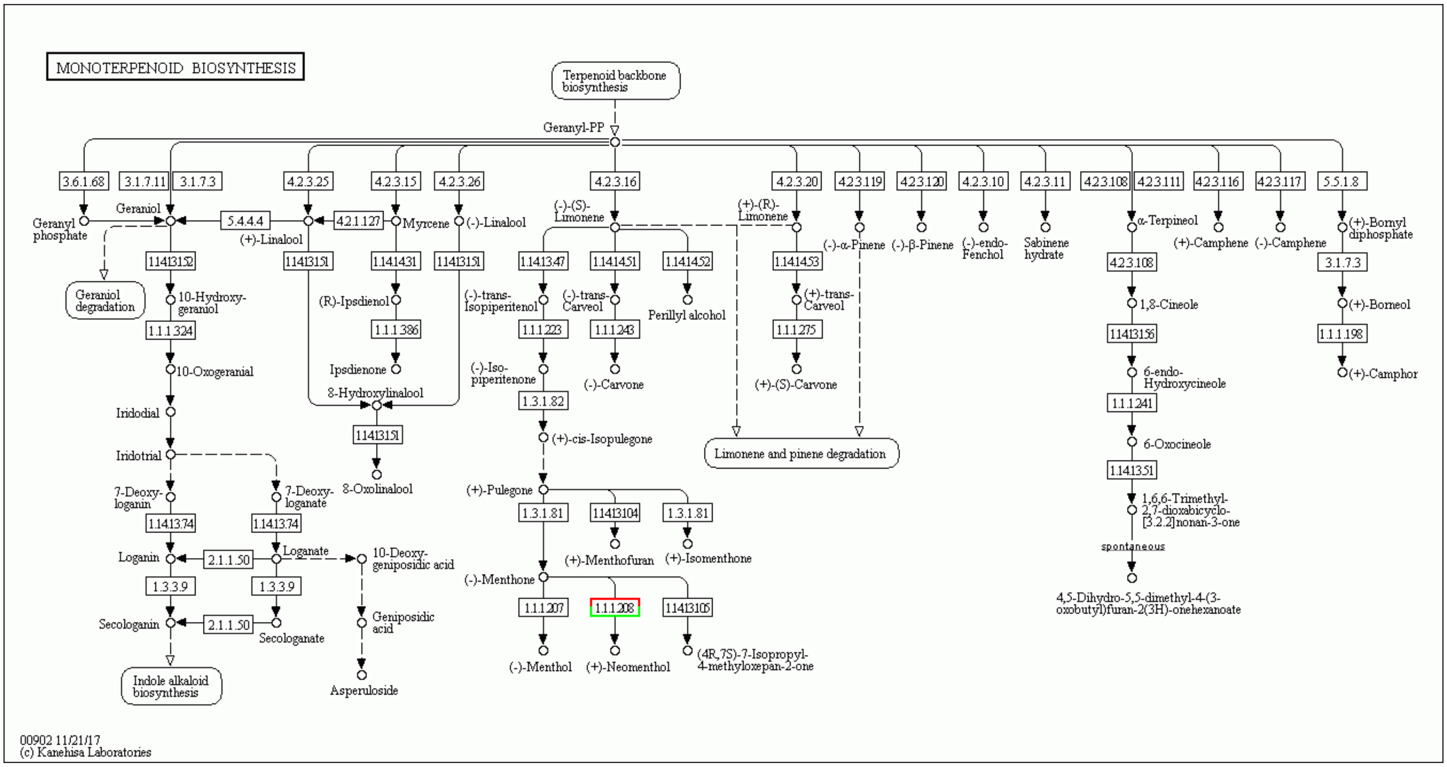

MONOTERPENOID BIOSYNTHESIS
Terpenoid backbone biosynthesis
Geranyl-PP
3.6.1.68
3.1.7.11
3.1.7.3
4.2.3.25
4.2.3.15
4.2.3.26
4.2.3.16
4.2.3.20
4.2.3.119
4.2.3.120
4.2.3.10
4.2.3.11
4.2.3.108
4.2.3.111
4.2.3.116
4.2.3.117
5.5.1.8
Geranyl phosphate
Geraniol
5.4.4.4
(+)-Linalool
4.2.1.127
Myrcene
(-)-Linalool
(-)-(S)-Limonene
(+)-(R)-Limonene
(-)-α-Pinene
(-)-β-Pinene
(-)-endo-Fenchol
Sabinene hydrate
α-Terpineol
(+)-Camphene
(-)-Camphene
(+)-Bornyl diphosphate
Geraniol degradation
1.14.13.152
10-Hydroxy-geraniol
1.1.1.324
10-Oxogeranial
Iridodial
Iridotrial
7-Deoxy-loganin
7-Deoxy-loganate
1.14.13.74
Loganin
2.1.1.50
Loganate
10-Deoxy-geniposidic acid
1.3.3.9
Secologanin
Secologanate
Geniposidic acid
Asperuloside
Indole alkaloid biosynthesis
1.14.13.151
(R)-Ipsdienol
1.1.1.386
Ipsdienone
8-Hydroxylinalool
8-Oxolinalool
1.14.13.47
1.14.14.51
1.14.14.52
1.14.14.53
(-)-trans-Isopiperitenol
(-)-trans-Carveol
Perillyl alcohol
(+)-trans-Carveol
1.1.1.223
1.1.1.243
1.1.1.275
(-)-Iso-piperitenone
(-)-Carvone
(+)-(S)-Carvone
1.3.1.82
(+)-cis-Isopulegone
Limonene and pinene degradation
(+)-Pulegone
1.3.1.81
1.14.13.104
(+)-Menthofuran
(+)-Isomenthone
(-)-Menthone
1.1.1.207
1.1.1.208
1.14.13.105
(-)-Menthol
(+)-Neomenthol
(4R,7S)-7-Isopropyl-4-methyloxepan-2-one
4.2.3.108
1,8-Cineole
1.14.13.156
6-endo-Hydroxycineole
1.1.1.241
6-Oxocineole
1.14.13.51
1,6,6-Trimethyl-2,7-dioxabicyclo-[3.2.2]nonan-3-one
spontaneous
4,5-Dihydro-5,5-dimethyl-4-(3-oxobutyl)furan-2(3H)-onehexanoate
3.1.7.3
(+)-Borneol
1.1.1.198
(+)-Camphor
00902 11/21/17
(c) Kanehisa Laboratories

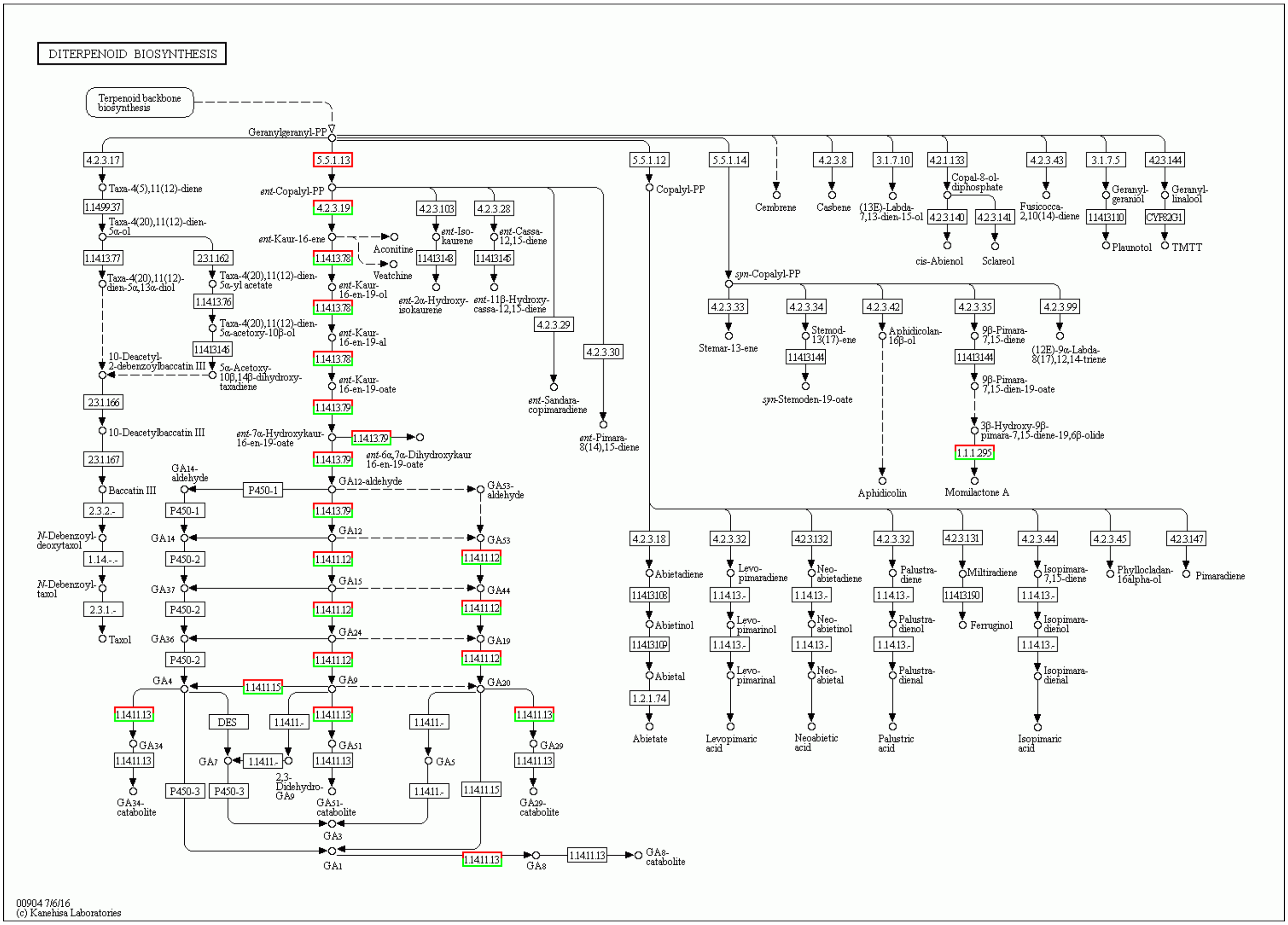

DITERPENOID BIOSYNTHESIS
Terpenoid backbone biosynthesis
Geranylgeranyl-PP
4.2.3.17
Taxa-4(5),11(12)-diene
1.14.99.37
Taxa-4(20),11(12)-dien-5α-ol
1.14.13.77
2.3.1.162
Taxa-4(20),11(12)-dien-5α,13α-diol
Taxa-4(20),11(12)-dien-5α-yl acetate
1.14.13.76
Taxa-4(20),11(12)-dien-5α-acetoxy-10β-ol
1.14.13.146
10-Deacetyl-2-debenzoylbaccatin III
5α-Acetoxy-10β,14β-dihydroxy-taxadiene
2.3.1.166
10-Deacetylbaccatin III
2.3.1.167
Baccatin III
2.3.2.-
N-Debenzoyl-deoxytaxol
1.14.-.-
N-Debenzoyl-taxol
2.3.1.-
Taxol
5.5.1.13
ent-Copalyl-PP
4.2.3.19
4.2.3.103
4.2.3.28
ent-Kaur-16-ene
Aconitine
Veatchine
ent-Iso-kaurene
ent-Cassa-12,15-diene
1.14.13.78
1.14.13.148
1.14.13.145
ent-Kaur-16-en-19-ol
ent-2α-Hydroxy-isokaurene
ent-11β-Hydroxy-cassa-12,15-diene
ent-Kaur-16-en-19-al
4.2.3.29
4.2.3.30
ent-Kaur-16-en-19-oate
1.14.13.79
ent-Sandara-copimaradiene
ent-7α-Hydroxykaur-16-en-19-oate
ent-Pimara-8(14),15-diene
ent-6α,7α-Dihydroxykaur-16-en-19-oate
GA14-aldehyde
P450-1
GA12-aldehyde
GA53-aldehyde
GA14
P450-2
GA12
GA53
1.14.11.12
GA37
GA15
GA44
GA36
GA24
GA19
GA4
1.14.11.15
GA9
GA20
1.14.11.13
DES
1.14.11.-
GA34
GA7
GA51
GA5
GA29
2,3-Didehydro-GA9
P450-3
GA34-catabolite
GA51-catabolite
GA29-catabolite
GA3
GA1
GA8
GA8-catabolite
5.5.1.12
5.5.1.14
Copalyl-PP
syn-Copalyl-PP
4.2.3.8
3.1.7.10
4.2.1.133
4.2.3.43
3.1.7.5
4.2.3.144
Cembrene
Casbene
(13E)-Labda-7,13-dien-15-ol
Copal-8-ol-diphosphate
4.2.3.140
4.2.3.141
cis-Abienol
Sclareol
Fusicocca-2,10(14)-diene
Geranyl-geraniol
Geranyl-linalool
1.14.13.110
CYP82G1
Plaunotol
TMTT
4.2.3.33
4.2.3.34
4.2.3.42
4.2.3.35
4.2.3.99
Stemar-13-ene
Stemod-13(17)-ene
Aphidicolan-16β-ol
9β-Pimara-7,15-diene
(12E)-9α-Labda-8(17),12,14-triene
1.14.13.144
syn-Stemoden-19-oate
9β-Pimara-7,15-dien-19-oate
3β-Hydroxy-9β-pimara-7,15-diene-19,6β-olide
1.1.1.295
Aphidicolin
Momilactone A
4.2.3.18
4.2.3.32
4.2.3.132
4.2.3.131
4.2.3.44
4.2.3.45
4.2.3.147
Abietadiene
Levo-pimaradiene
Neo-abietadiene
Palustra-diene
Miltiradiene
Isopimara-7,15-diene
Phyllocladan-16alpha-ol
Pimaradiene
1.14.13.108
1.14.13.-
1.14.13.190
Abietinol
Levo-pimarinol
Neo-abietinol
Palustra-dienol
Ferruginol
Isopimara-dienol
1.14.13.109
Abietal
Levo-pimarinal
Neo-abietal
Palustra-dienal
Isopimara-dienal
1.2.1.74
Abietate
Levopimaric acid
Neoabietic acid
Palustric acid
Isopimaric acid
00904 7/6/16
(c) Kanehisa Laboratories

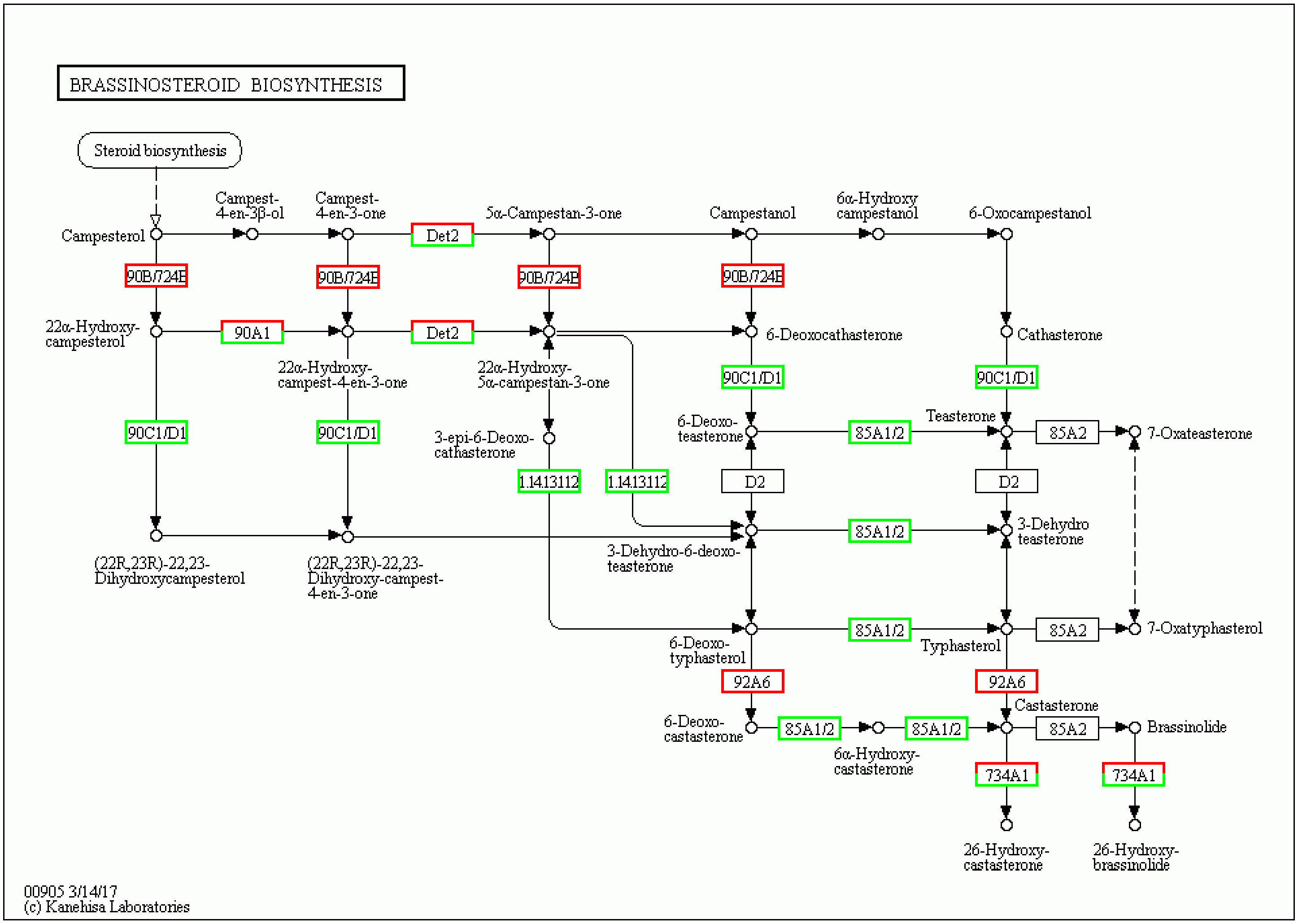
BRASSINOSTEROID BIOSYNTHESIS
Steroid biosynthesis
Campesterol
Campest-4-en-3β-ol
Campest-4-en-3-one
Det2
5α-Campestan-3-one
Campestanol
6α-Hydroxy campestanol
6-Oxocampestanol
90B/724B
90B/724B
90B/724B
90B/724B
22α-Hydroxy-campesterol
90A1
Det2
6-Deoxocathasterone
Cathasterone
22α-Hydroxy-campest-4-en-3-one
22α-Hydroxy-5α-campestan-3-one
90C1/D1
90C1/D1
90C1/D1
90C1/D1
3-epi-6-Deoxo-cathasterone
6-Deoxo-teasterone
85A1/2
Teasterone
85A2
7-Oxateasterone
1.14.13.112
1.14.13.112
D2
D2
85A1/2
3-Dehydro teasterone
(22R,23R)-22,23-Dihydroxycampesterol
(22R,23R)-22,23-Dihydroxy-campest-4-en-3-one
3-Dehydro-6-deoxo-teasterone
85A1/2
85A2
7-Oxatyphasterol
6-Deoxo-typhasterol
Typhasterol
92A6
92A6
Castasterone
6-Deoxo-castasterone
85A1/2
85A1/2
85A2
Brassinolide
6α-Hydroxy-castasterone
734A1
734A1
26-Hydroxy-castasterone
26-Hydroxy-brassinolide
00905 3/14/17
(c) Kanehisa Laboratories

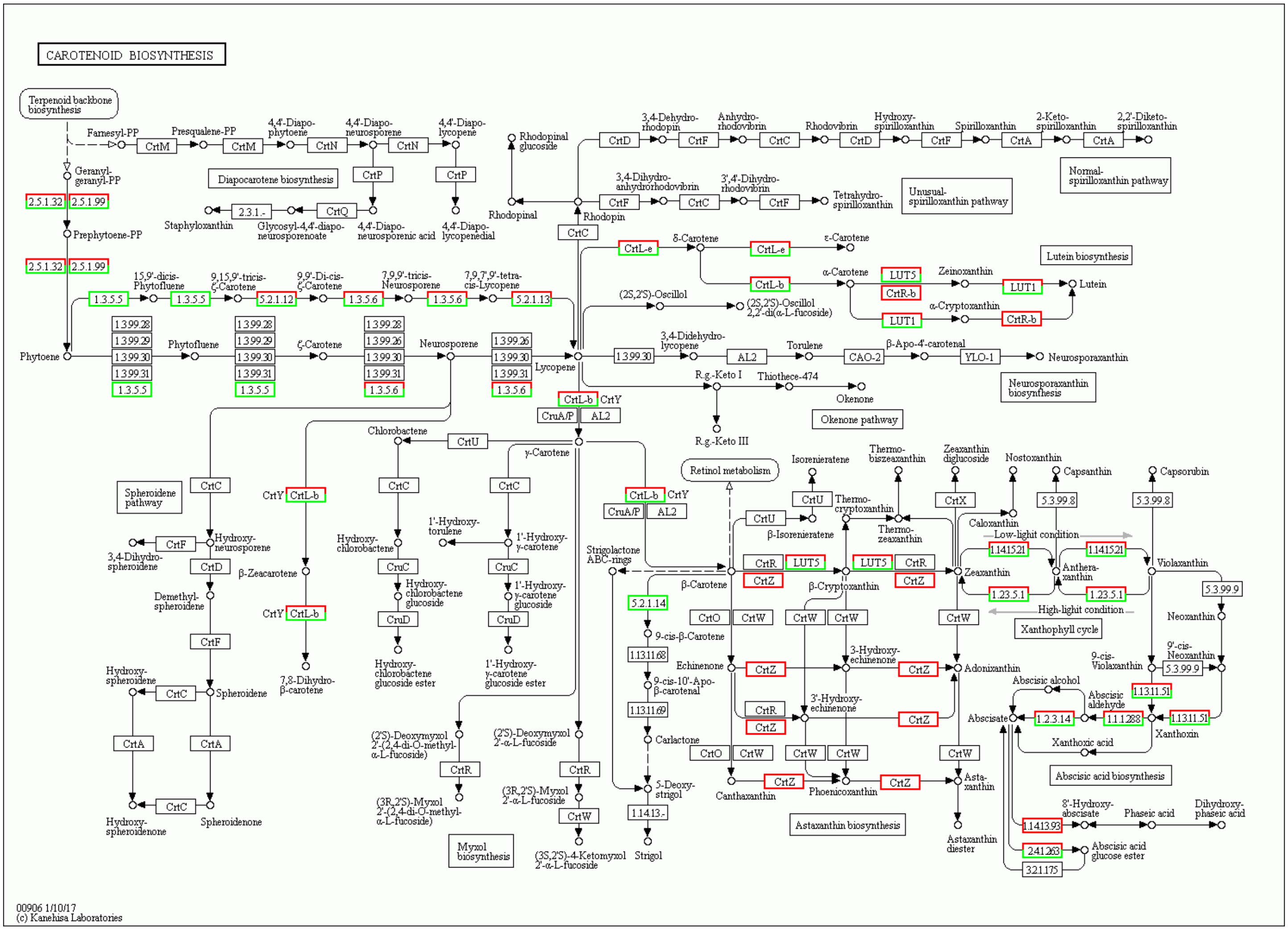
CAROTENOID BIOSYNTHESIS
Terpenoid backbone biosynthesis
Farnesyl-PP
CrtM
Presqualene-PP
4,4'-Diapo-phytoene
CrtN
4,4'-Diapo-neurosporene
4,4'-Diapo-lycopene
CrtP
Diapocarotene biosynthesis
Geranyl-geranyl-PP
2.5.1.32
2.5.1.99
Prephytoene-PP
2.3.1.-
CrtQ
Staphyloxanthin
Glycosyl-4,4'-diapo-neurosporenoate
4,4'-Diapo-neurosporenic acid
4,4'-Diapo-lycopenedial
15,9'-dicis-Phytofluene
9,15,9'-tricis-ζ-Carotene
9,9'-Di-cis-ζ-Carotene
7,9,9'-tricis-Neurosporene
7,9,7',9'-tetra-cis-Lycopene
1.3.5.5
5.2.1.12
1.3.5.6
5.2.1.13
1.3.99.28
1.3.99.29
1.3.99.30
1.3.99.31
1.3.99.26
Phytoene
Phytofluene
ζ-Carotene
Neurosporene
Lycopene
Rhodopinal glucoside
Rhodopinal
Rhodopin
CrtC
CrtD
CrtF
3,4-Dehydro-rhodopin
Anhydro-rhodovibrin
Rhodovibrin
Hydroxy-spirilloxanthin
Spirilloxanthin
2-Keto-spirilloxanthin
2,2'-Diketo-spirilloxanthin
CrtA
Normal-spirilloxanthin pathway
3,4-Dihydro-anhydrorhodovibrin
3',4'-Dihydro-rhodovibrin
Tetrahydro-spirilloxanthin
Unusual-spirilloxanthin pathway
CrtL-e
δ-Carotene
ε-Carotene
Lutein biosynthesis
CrtL-b
α-Carotene
LUT5
CrtR-b
Zeinoxanthin
LUT1
Lutein
α-Cryptoxanthin
(2S,2'S)-Oscillol
(2S,2'S)-Oscillol 2,2'-di(α-L-fucoside)
3,4-Didehydro-lycopene
AL2
Torulene
CAO-2
β-Apo-4'-carotenal
YLO-1
Neurosporaxanthin
Neurosporaxanthin biosynthesis
R.g.-Keto I
Thiothece-474
Okenone
Okenone pathway
R.g.-Keto III
CrtY
CruA/P
Chlorobactene
CrtU
γ-Carotene
Spheroidene pathway
Hydroxy-neurosporene
3,4-Dihydro-spheroidene
Demethyl-spheroidene
Hydroxy-spheroidene
Spheroidene
Hydroxy-spheroidenone
Spheroidenone
β-Zeacarotene
7,8-Dihydro-β-carotene
CruC
CruD
Hydroxy-chlorobactene
Hydroxy-chlorobactene glucoside
Hydroxy-chlorobactene glucoside ester
1'-Hydroxy-torulene
1'-Hydroxy-γ-carotene
1'-Hydroxy-γ-carotene glucoside
1'-Hydroxy-γ-carotene glucoside ester
(2'S)-Deoxymyxol 2'-(2,4-di-O-methyl-α-L-fucoside)
(2'S)-Deoxymyxol 2'-α-L-fucoside
CrtR
(3R,2'S)-Myxol 2'-(2,4-di-O-methyl-α-L-fucoside)
(3R,2'S)-Myxol 2'-α-L-fucoside
CrtW
(3S,2'S)-4-Ketomyxol 2'-α-L-fucoside
Myxol biosynthesis
Retinol metabolism
Strigolactone ABC-rings
β-Carotene
5.2.1.14
9-cis-β-Carotene
1.13.11.68
9-cis-10'-Apo-β-carotenal
1.13.11.69
Carlactone
5-Deoxy-strigol
1.14.13.-
Strigol
Isorenieratene
β-Isorenieratene
Thermo-biszeaxanthin
Thermo-cryptoxanthin
Thermo-zeaxanthin
Zeaxanthin diglucoside
CrtX
Nostoxanthin
Caloxanthin
Capsanthin
Capsorubin
5.3.99.8
Low-light condition
1.14.15.21
Zeaxanthin
Anthera-xanthin
Violaxanthin
1.23.5.1
High-light condition
Xanthophyll cycle
Neoxanthin
5.3.99.9
9-cis-Violaxanthin
9'-cis-Neoxanthin
β-Cryptoxanthin
CrtZ
CrtO
Echinenone
3-Hydroxy-echinenone
3'-Hydroxy-echinenone
Adonixanthin
Canthaxanthin
Phoenicoxanthin
Asta-xanthin
Astaxanthin diester
Astaxanthin biosynthesis
Abscisic alcohol
Abscisic aldehyde
Abscisate
1.2.3.14
1.1.1.288
1.13.11.51
Xanthoxin
Xanthoxic acid
Abscisic acid biosynthesis
8'-Hydroxy-abscisate
1.14.13.93
Phaseic acid
Dihydroxy-phaseic acid
2.4.1.263
Abscisic acid glucose ester
3.2.1.175
00906 1/10/17
(c) Kanehisa Laboratories

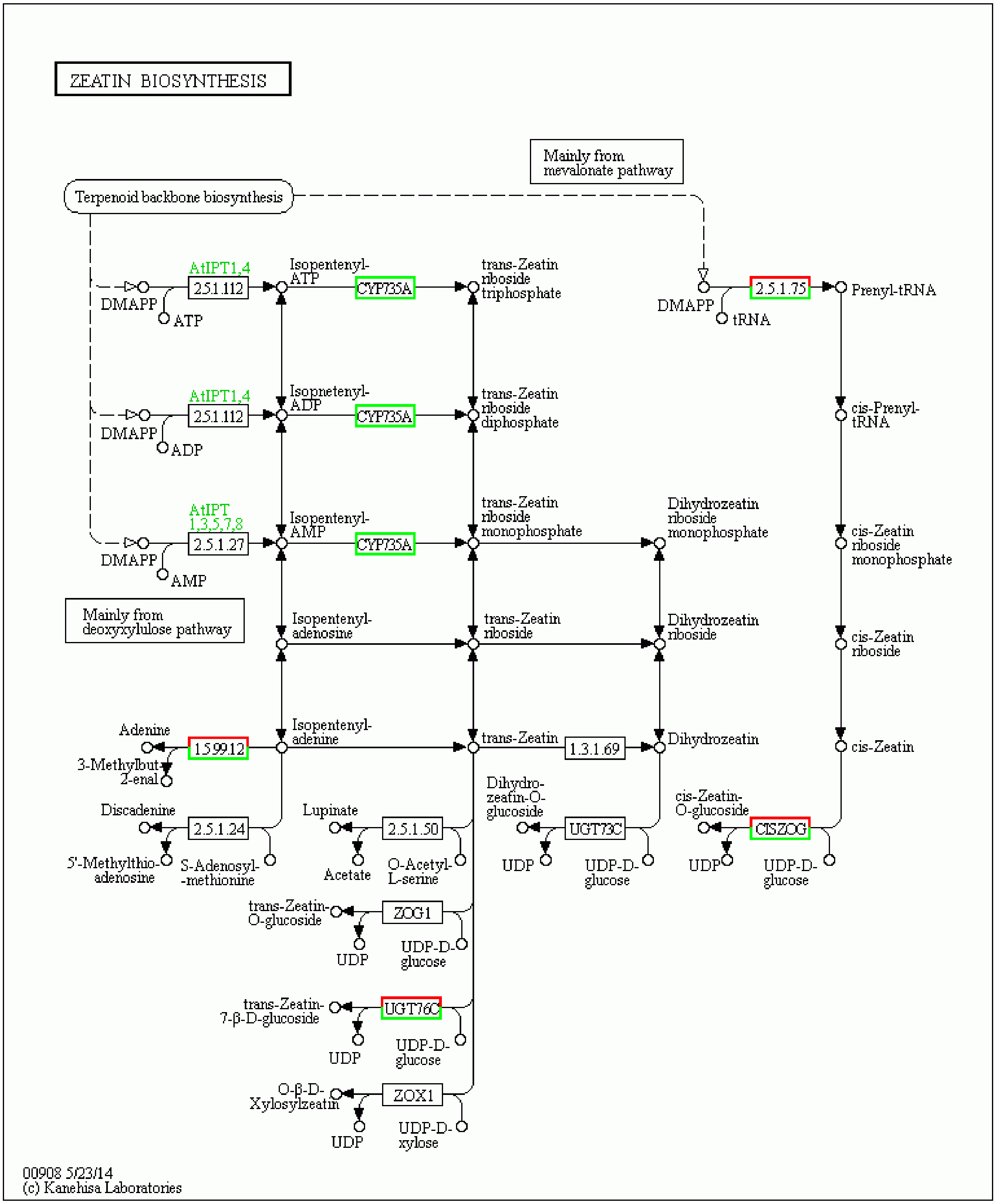

ZEATIN BIOSYNTHESIS
Mainly from mevalonate pathway
Terpenoid backbone biosynthesis
AtIPT1,4
2.5.1.112
DMAPP
ATP
Isopentenyl-ATP
CYP735A
trans-Zeatin riboside triphosphate
DMAPP
tRNA
2.5.1.75
Prenyl-tRNA
AtIPT1,4
2.5.1.112
DMAPP
ADP
Isopnetenyl-ADP
CYP735A
trans-Zeatin riboside diphosphate
cis-Prenyl-tRNA
AtIPT 1,3,5,7,8
2.5.1.27
DMAPP
AMP
Isopentenyl-AMP
CYP735A
trans-Zeatin riboside monophosphate
Dihydrozeatin riboside monophosphate
cis-Zeatin riboside monophosphate
Mainly from deoxyxylulose pathway
Isopentenyl-adenosine
trans-Zeatin riboside
Dihydrozeatin riboside
cis-Zeatin riboside
Adenine
1.5.99.12
3-Methylbut-2-enal
Isopentenyl-adenine
trans-Zeatin
1.3.1.69
Dihydrozeatin
cis-Zeatin
Discadenine
2.5.1.24
5'-Methylthio-adenosine
S-Adenosyl--methionine
Lupinate
2.5.1.50
Acetate
O-Acetyl-L-serine
Dihydro-zeatin-O-glucoside
UGT73C
UDP
UDP-D-glucose
cis-Zeatin-O-glucoside
CISZOG
UDP
UDP-D-glucose
trans-Zeatin-O-glucoside
ZOG1
UDP
UDP-D-glucose
trans-Zeatin-7-β-D-glucoside
UGT76C
UDP
UDP-D-glucose
O-β-D-Xylosylzeatin
ZOX1
UDP
UDP-D-xylose
00908 5/23/14
(c) Kanehisa Laboratories

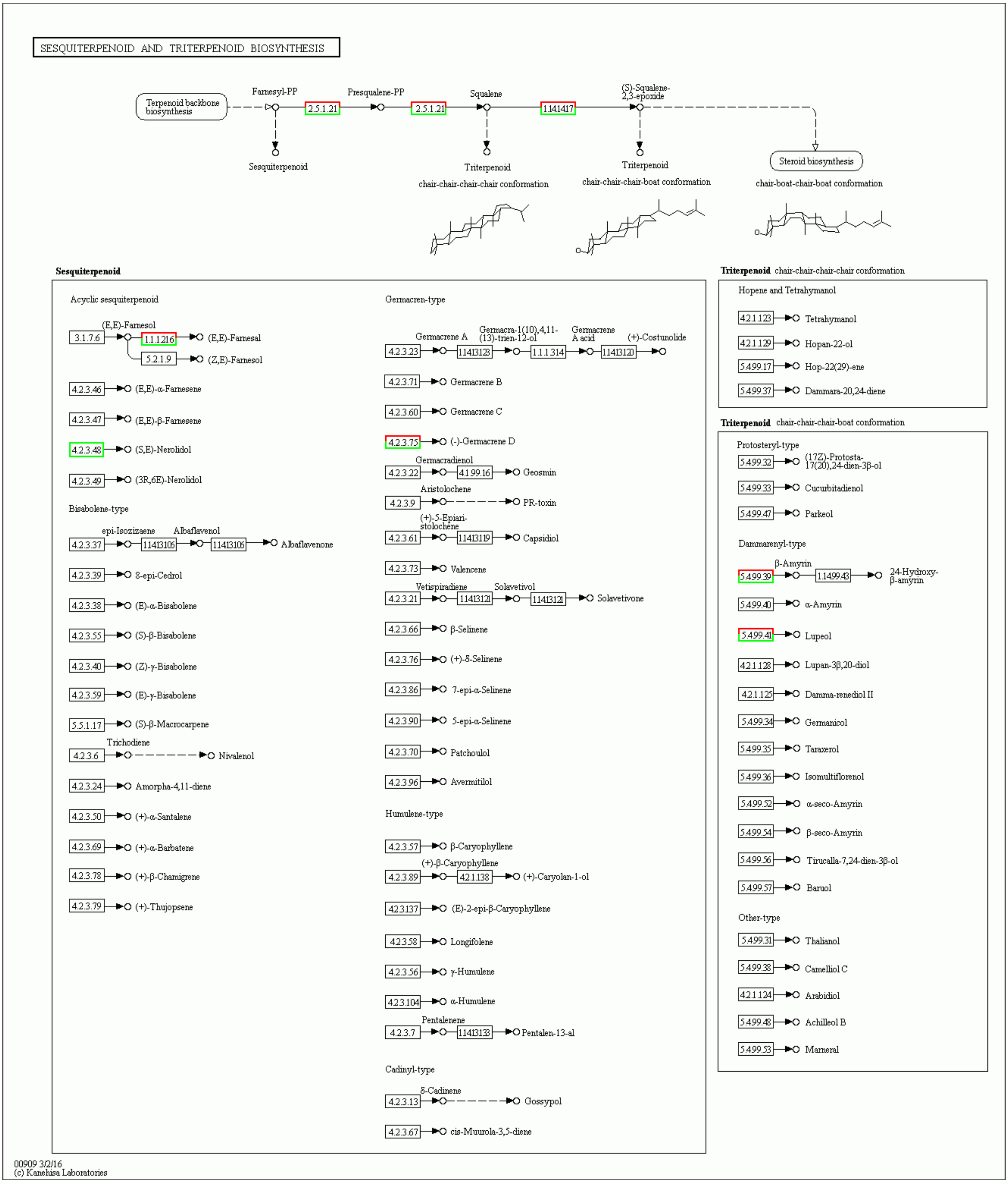
SESQUITERPENOID AND TRITERPENOID BIOSYNTHESIS
Terpenoid backbone biosynthesis
Farnesyl-PP
2.5.1.21
Presqualene-PP
2.5.1.21
Squalene
1.14.14.17
(S)-Squalene-2,3-epoxide
Sesquiterpenoid
Triterpenoid
chair-chair-chair-chair conformation
Triterpenoid
chair-chair-chair-boat conformation
Steroid biosynthesis
chair-boat-chair-boat conformation
Sesquiterpenoid
Acyclic sesquiterpenoid
(E,E)-Farnesol
3.1.7.6
1.1.1.216
(E,E)-Farnesal
5.2.1.9
(Z,E)-Farnesol
4.2.3.46
(E,E)-α-Farnesene
4.2.3.47
(E,E)-β-Farnesene
4.2.3.48
(S,E)-Nerolidol
4.2.3.49
(3R,6E)-Nerolidol
Bisabolene-type
epi-Isozizaene
Albaflavenol
4.2.3.37
11413106
11413106
Albaflavenone
4.2.3.39
8-epi-Cedrol
4.2.3.38
(E)-α-Bisabolene
4.2.3.55
(S)-β-Bisabolene
4.2.3.40
(Z)-γ-Bisabolene
4.2.3.59
(E)-γ-Bisabolene
5.5.1.17
(S)-β-Macrocarpene
Trichodiene
4.2.3.6
Nivalenol
4.2.3.24
Amorpha-4,11-diene
4.2.3.50
(+)-α-Santalene
4.2.3.69
(+)-α-Barbatene
4.2.3.78
(+)-β-Chamigrene
4.2.3.79
(+)-Thujopsene
Germacren-type
Germacrene A
Germacra-1(10),4,11-(13)-trien-12-ol
Germacrene A acid
(+)-Costunolide
4.2.3.23
11413123
1.1.1.314
11413120
4.2.3.71
Germacrene B
4.2.3.60
Germacrene C
4.2.3.75
(-)-Germacrene D
Germacradienol
4.2.3.22
4.1.99.16
Geosmin
Aristolochene
4.2.3.9
PR-toxin
(+)-5-Epiari-stolochene
4.2.3.61
11413119
Capsidiol
4.2.3.73
Valencene
Vetispiradiene
Solavetivol
4.2.3.21
11413121
11413121
Solavetivone
4.2.3.66
β-Selinene
4.2.3.76
(+)-δ-Selinene
4.2.3.86
7-epi-α-Selinene
4.2.3.90
5-epi-α-Selinene
4.2.3.70
Patchoulol
4.2.3.96
Avermitilol
Humulene-type
4.2.3.57
β-Caryophyllene
(+)-β-Caryophyllene
4.2.3.89
4.2.1.138
(+)-Caryolan-1-ol
4.2.3.137
(E)-2-epi-β-Caryophyllene
4.2.3.58
Longifolene
4.2.3.56
γ-Humulene
4.2.3.104
α-Humulene
Pentalenene
4.2.3.7
11413133
Pentalen-13-al
Cadinyl-type
δ-Cadinene
4.2.3.13
Gossypol
4.2.3.67
cis-Muurola-3,5-diene
Triterpenoid chair-chair-chair-chair conformation
Hopene and Tetrahymanol
4.2.1.123
Tetrahymanol
4.2.1.129
Hopan-22-ol
5.4.99.17
Hop-22(29)-ene
5.4.99.37
Dammara-20,24-diene
Triterpenoid chair-chair-chair-boat conformation
Protosteryl-type
5.4.99.32
(17Z)-Protosta-17(20),24-dien-3β-ol
5.4.99.33
Cucurbitadienol
5.4.99.47
Parkeol
Dammarenyl-type
β-Amyrin
5.4.99.39
1.14.99.43
24-Hydroxy-β-amyrin
5.4.99.40
α-Amyrin
5.4.99.41
Lupeol
4.2.1.128
Lupan-3β,20-diol
4.2.1.125
Damma-renediol II
5.4.99.34
Germanicol
5.4.99.35
Taraxerol
5.4.99.36
Isomultiflorenol
5.4.99.52
α-seco-Amyrin
5.4.99.54
β-seco-Amyrin
5.4.99.56
Tirucalla-7,24-dien-3β-ol
5.4.99.57
Baruol
Other-type
5.4.99.31
Thalianol
5.4.99.38
Camelliol C
4.2.1.124
Arabidiol
5.4.99.48
Achilleol B
5.4.99.53
Marneral
00909 3/2/16
(c) Kanehisa Laboratories

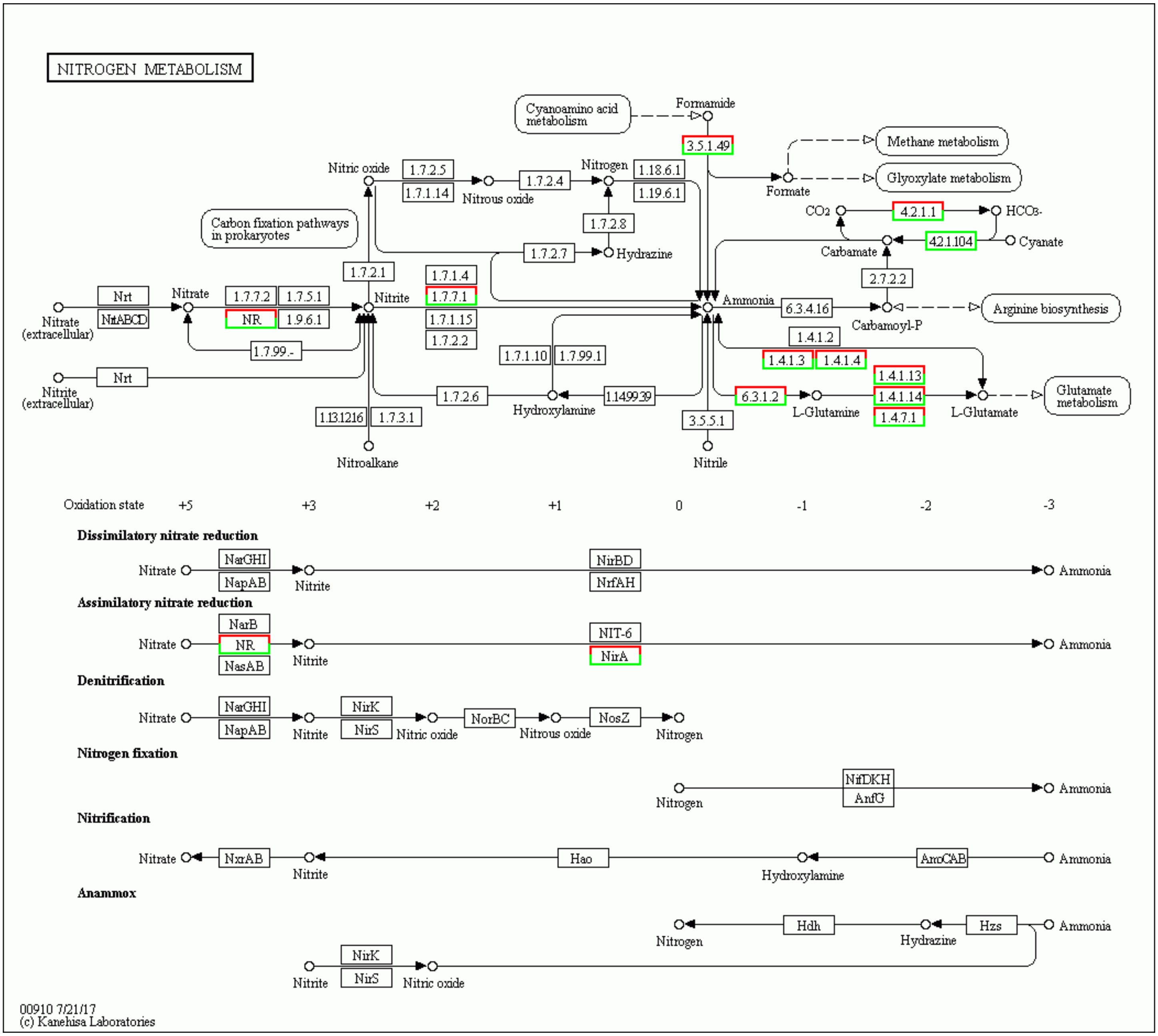
NITROGEN METABOLISM
Cyanoamino acid metabolism
Formamide
3.5.1.49
Methane metabolism
Glyoxylate metabolism
Formate
Nitric oxide
1.7.2.5
1.7.1.14
1.7.2.4
Nitrous oxide
Nitrogen
1.18.6.1
1.19.6.1
1.7.2.8
Hydrazine
1.7.2.7
Carbon fixation pathways in prokaryotes
CO2
4.2.1.1
HCO3-
4.2.1.104
Cyanate
Carbamate
2.7.2.2
1.7.2.1
1.7.1.4
1.7.7.1
1.7.1.15
1.7.2.2
Nitrite
Nitrate
Nrt
NrtABCD
1.7.7.2
1.7.5.1
NR
1.9.6.1
1.7.99.-
Nitrate (extracellular)
Nitrite (extracellular)
Nrt
Ammonia
6.3.4.16
Carbamoyl-P
Arginine biosynthesis
1.4.1.2
1.4.1.3
1.4.1.4
1.4.1.13
1.4.1.14
1.4.7.1
6.3.1.2
L-Glutamine
L-Glutamate
Glutamate metabolism
1.7.1.10
1.7.99.1
1.7.2.6
Hydroxylamine
1.14.99.39
1.13.12.16
1.7.3.1
Nitroalkane
3.5.5.1
Nitrile
Oxidation state +5 +3 +2 +1 0 -1 -2 -3
Dissimilatory nitrate reduction
Nitrate NarGHI NapAB Nitrite NirBD NrfAH Ammonia
Assimilatory nitrate reduction
Nitrate NarB NR NasAB Nitrite NIT-6 NirA Ammonia
Denitrification
Nitrate NarGHI NapAB Nitrite NirK NirS Nitric oxide NorBC Nitrous oxide NosZ Nitrogen
Nitrogen fixation
Nitrogen NifDKH AnfG Ammonia
Nitrification
Nitrate NxrAB Nitrite Hao Hydroxylamine AmoCAB Ammonia
Anammox
Nitrogen Hdh Hydrazine Hzs Ammonia
Nitrite NirK NirS Nitric oxide
00910 7/21/17
(c) Kanehisa Laboratories

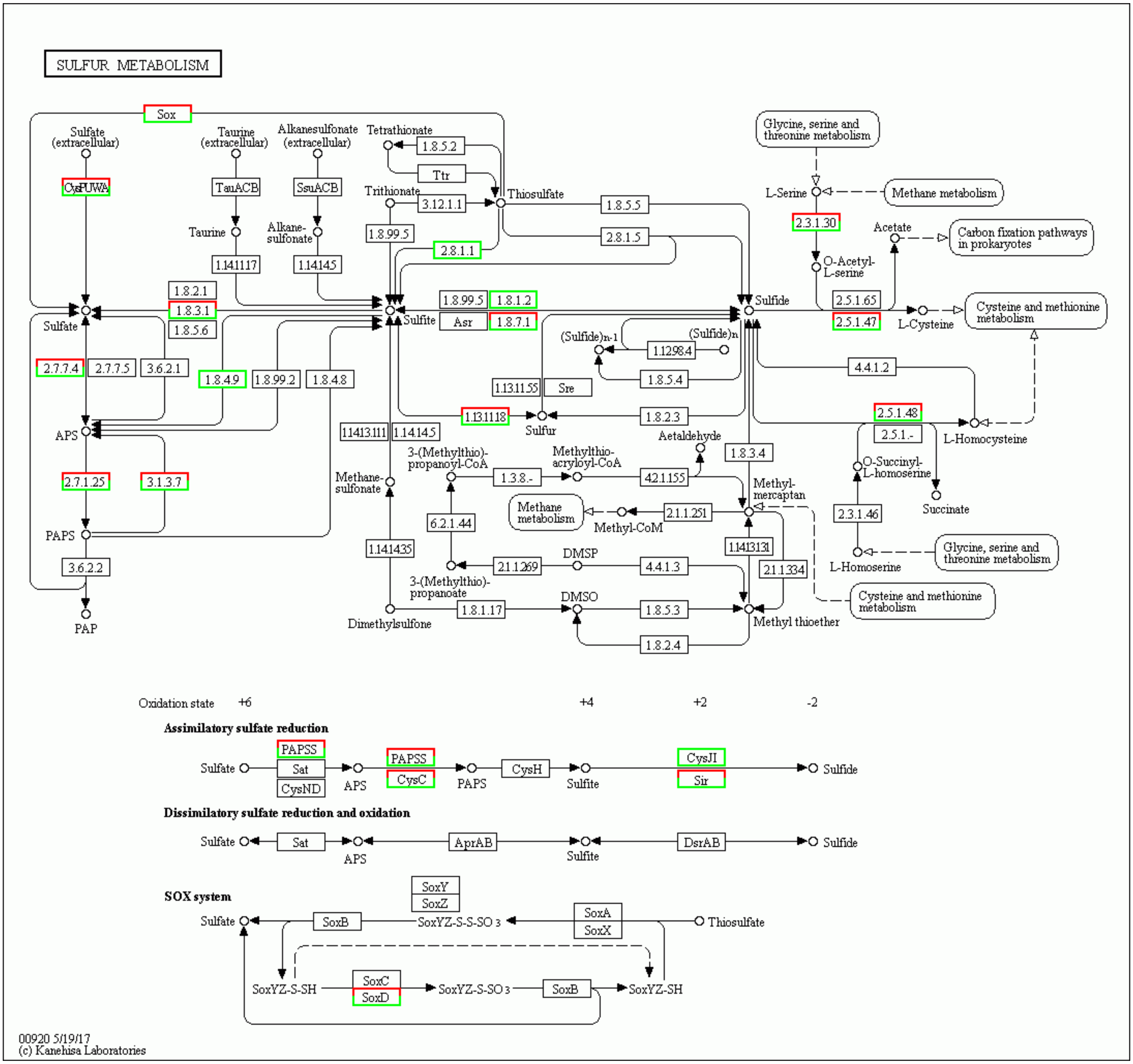
SULFUR METABOLISM
Sox
Sulfate (extracellular)
Taurine (extracellular)
Alkanesulfonate (extracellular)
Tetrathionate
1.8.5.2
Ttr
Trithionate
3.12.1.1
Thiosulfate
1.8.5.5
2.8.1.5
Glycine, serine and threonine metabolism
L-Serine
Methane metabolism
2.3.1.30
Acetate
Carbon fixation pathways in prokaryotes
O-Acetyl-L-serine
CysPUWA
TauACB
SsuACB
Taurine
Alkane-sulfonate
1.8.99.5
2.8.1.1
1.14.11.17
1.14.14.5
1.8.2.1
1.8.3.1
1.8.5.6
Sulfate
Sulfite
1.8.99.5
1.8.1.2
Asr
1.8.7.1
Sulfide
2.5.1.65
2.5.1.47
L-Cysteine
Cysteine and methionine metabolism
(Sulfide)n-1
(Sulfide)n
1.12.98.4
1.8.5.4
4.4.1.2
2.7.7.4
2.7.7.5
3.6.2.1
1.8.4.9
1.8.99.2
1.8.4.8
1.13.11.55
Sre
APS
1.14.13.111
1.14.14.5
1.13.11.18
Sulfur
1.8.2.3
2.5.1.48
2.5.1.-
L-Homocysteine
3-(Methylthio)-propanoyl-CoA
Methylthio-acryloyl-CoA
Aetaldehyde
1.8.3.4
O-Succinyl-L-homoserine
Methane-sulfonate
1.3.8.-
4.2.1.155
Methyl-mercaptan
2.7.1.25
3.1.3.7
Methane metabolism
2.1.1.251
Methyl-CoM
2.3.1.46
Succinate
PAPS
6.2.1.44
1.14.14.35
DMSP
1.14.13.131
2.1.1.269
4.4.1.3
2.1.1.334
L-Homoserine
Glycine, serine and threonine metabolism
3.6.2.2
3-(Methylthio)-propanoate
Cysteine and methionine metabolism
DMSO
Dimethylsulfone
1.8.1.17
1.8.5.3
Methyl thioether
PAP
1.8.2.4
Oxidation state +6 +4 +2 -2
Assimilatory sulfate reduction
Sulfate
PAPSS
Sat
CysND
APS
PAPSS
CysC
PAPS
CysH
Sulfite
CysJI
Sir
Sulfide
Dissimilatory sulfate reduction and oxidation
Sulfate
Sat
APS
AprAB
Sulfite
DsrAB
Sulfide
SOX system
SoxY
SoxZ
Sulfate
SoxB
SoxYZ-S-S-SO3
SoxA
SoxX
Thiosulfate
SoxYZ-S-SH
SoxC
SoxD
SoxYZ-S-SO3
SoxB
SoxYZ-SH
00920 5/19/17
(c) Kanehisa Laboratories

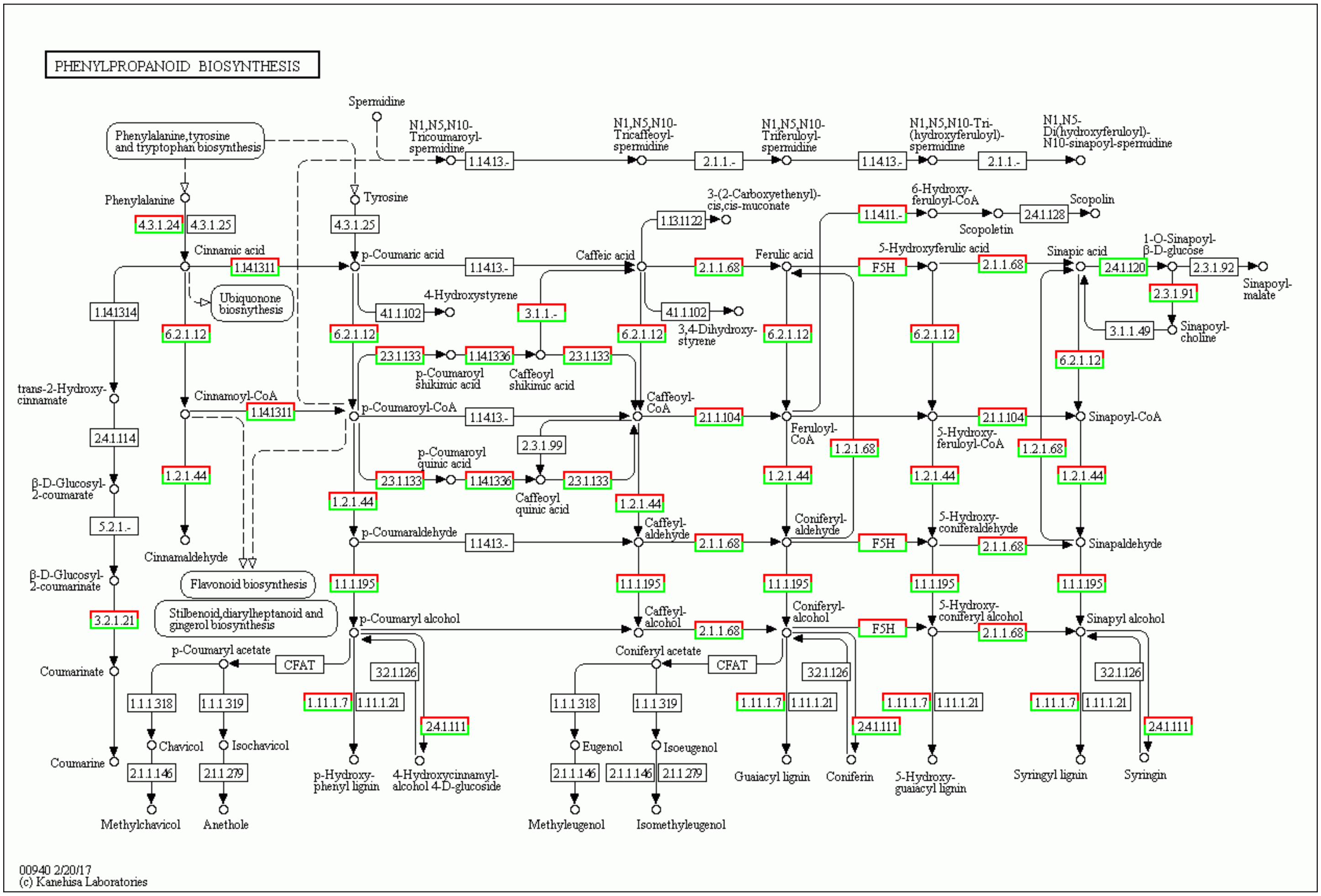
PHENYLPROPANOID BIOSYNTHESIS
Phenylalanine,tyrosine and tryptophan biosynthesis
Spermidine
Phenylalanine
Tyrosine
Cinnamic acid
p-Coumaric acid
Caffeic acid
Ferulic acid
5-Hydroxyferulic acid
Sinapic acid
Ubiquinone biosynthesis
4-Hydroxystyrene
3,4-Dihydroxy-styrene
Cinnamoyl-CoA
p-Coumaroyl-CoA
Caffeoyl-CoA
Feruloyl-CoA
5-Hydroxy-feruloyl-CoA
Sinapoyl-CoA
Cinnamaldehyde
p-Coumaraldehyde
Caffeyl-aldehyde
Coniferyl-aldehyde
5-Hydroxy-coniferaldehyde
Sinapaldehyde
Flavonoid biosynthesis
Stilbenoid,diarylheptanoid and gingerol biosynthesis
p-Coumaryl alcohol
Caffeyl-alcohol
Coniferyl-alcohol
5-Hydroxy-coniferyl alcohol
Sinapyl alcohol
Scopoletin
Scopolin
Sinapoyl-malate
Sinapoyl-choline
Coumarinate
Coumarine
Chavicol
Isochavicol
Methylchavicol
Anethole
Eugenol
Isoeugenol
Methyleugenol
Isomethyleugenol
p-Hydroxy-phenyl lignin
Guaiacyl lignin
Coniferin
5-Hydroxy-guaiacyl lignin
Syringyl lignin
Syringin
00940 2/20/17
(c) Kanehisa Laboratories

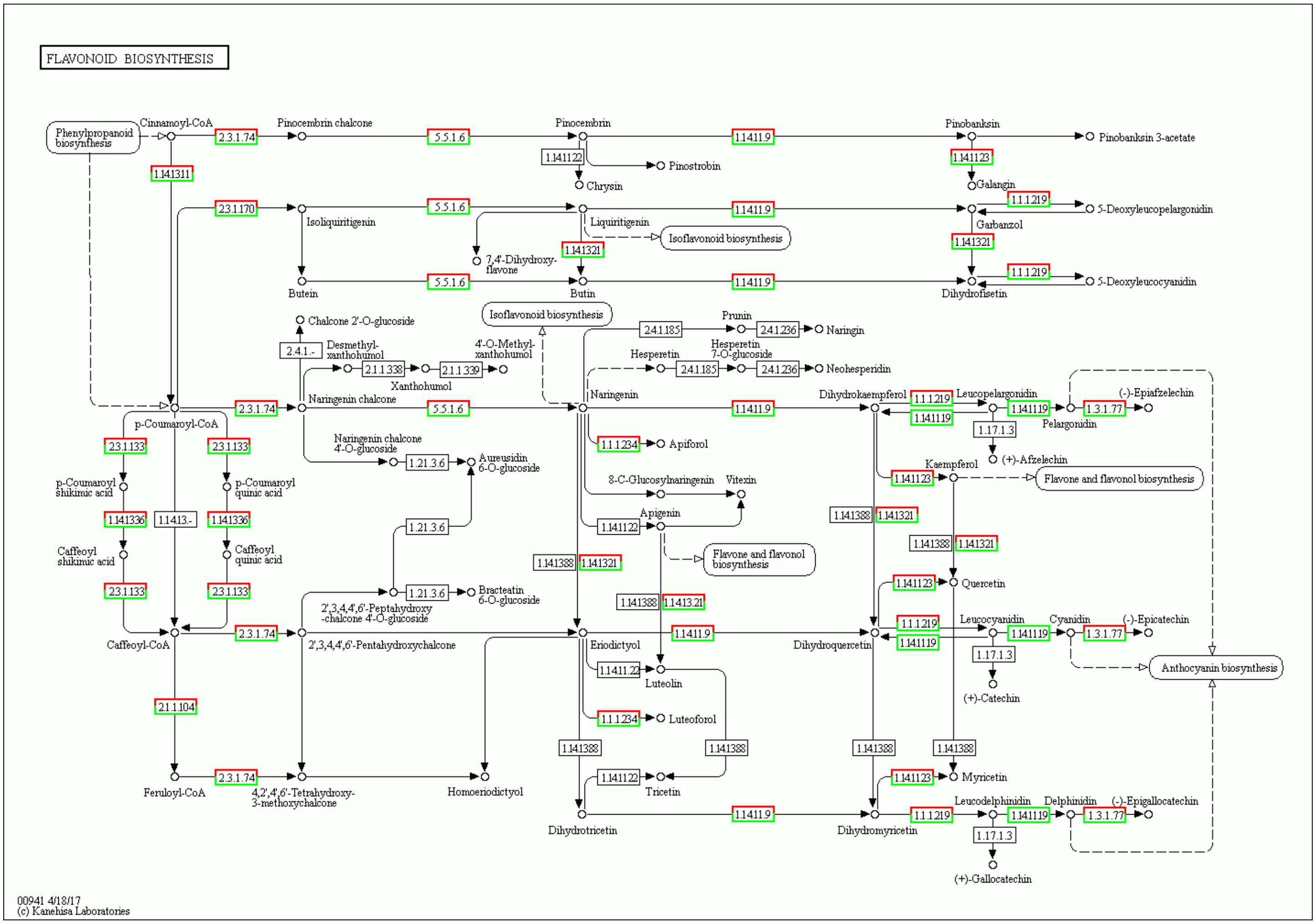
FLAVONOID BIOSYNTHESIS
Phenylpropanoid biosynthesis
Cinnamoyl-CoA
2.3.1.74
Pinocembrin chalcone
5.5.1.6
Pinocembrin
1.14.11.9
Pinobanksin
Pinobanksin 3-acetate
1.14.11.22
Pinostrobin
Chrysin
1.14.13.11
1.14.11.23
Galangin
2.3.1.170
Isoliquiritigenin
Liquiritigenin
1.1.1.219
5-Deoxyleucopelargonidin
Garbanzol
7,4'-Dihydroxy-flavone
1.14.13.21
Isoflavonoid biosynthesis
Butein
Butin
Dihydrofisetin
5-Deoxyleucocyanidin
Chalcone 2'-O-glucoside
2.4.1.-
Desmethyl-xanthohumol
2.1.1.338
Xanthohumol
2.1.1.339
4'-O-Methyl-xanthohumol
2.4.1.185
Prunin
2.4.1.236
Naringin
Hesperetin
Hesperetin 7-O-glucoside
Neohesperidin
Naringenin chalcone
Naringenin
Dihydrokaempferol
Leucopelargonidin
1.14.11.19
1.3.1.77
(-)-Epiafzelechin
Pelargonidin
1.17.1.3
(+)-Afzelechin
p-Coumaroyl-CoA
2.3.1.133
1.1.1.234
Apiforol
Naringenin chalcone 4'-O-glucoside
1.21.3.6
Aureusidin 6-O-glucoside
Kaempferol
Flavone and flavonol biosynthesis
8-C-Glucosylnaringenin
Vitexin
p-Coumaroyl shikimic acid
p-Coumaroyl quinic acid
1.14.13.36
1.14.13.-
Apigenin
1.14.13.88
Caffeoyl shikimic acid
Caffeoyl quinic acid
Bracteatin 6-O-glucoside
Quercetin
2',3,4,4',6'-Peptahydroxy-chalcone 4'-O-glucoside
Caffeoyl-CoA
2',3,4,4',6'-Pentahydroxychalcone
Eriodictyol
Dihydroquercetin
Leucocyanidin
Cyanidin
(-)-Epicatechin
Luteolin
Anthocyanin biosynthesis
(+)-Catechin
2.1.1.104
Luteoforol
Myricetin
Feruloyl-CoA
4,2',4',6'-Tetrahydroxy-3-methoxychalcone
Homoeriodictyol
Tricetin
Dihydrotricetin
Dihydromyricetin
Leucodelphinidin
Delphinidin
(-)-Epigallocatechin
(+)-Gallocatechin
00941 4/18/17
(c) Kanehisa Laboratories

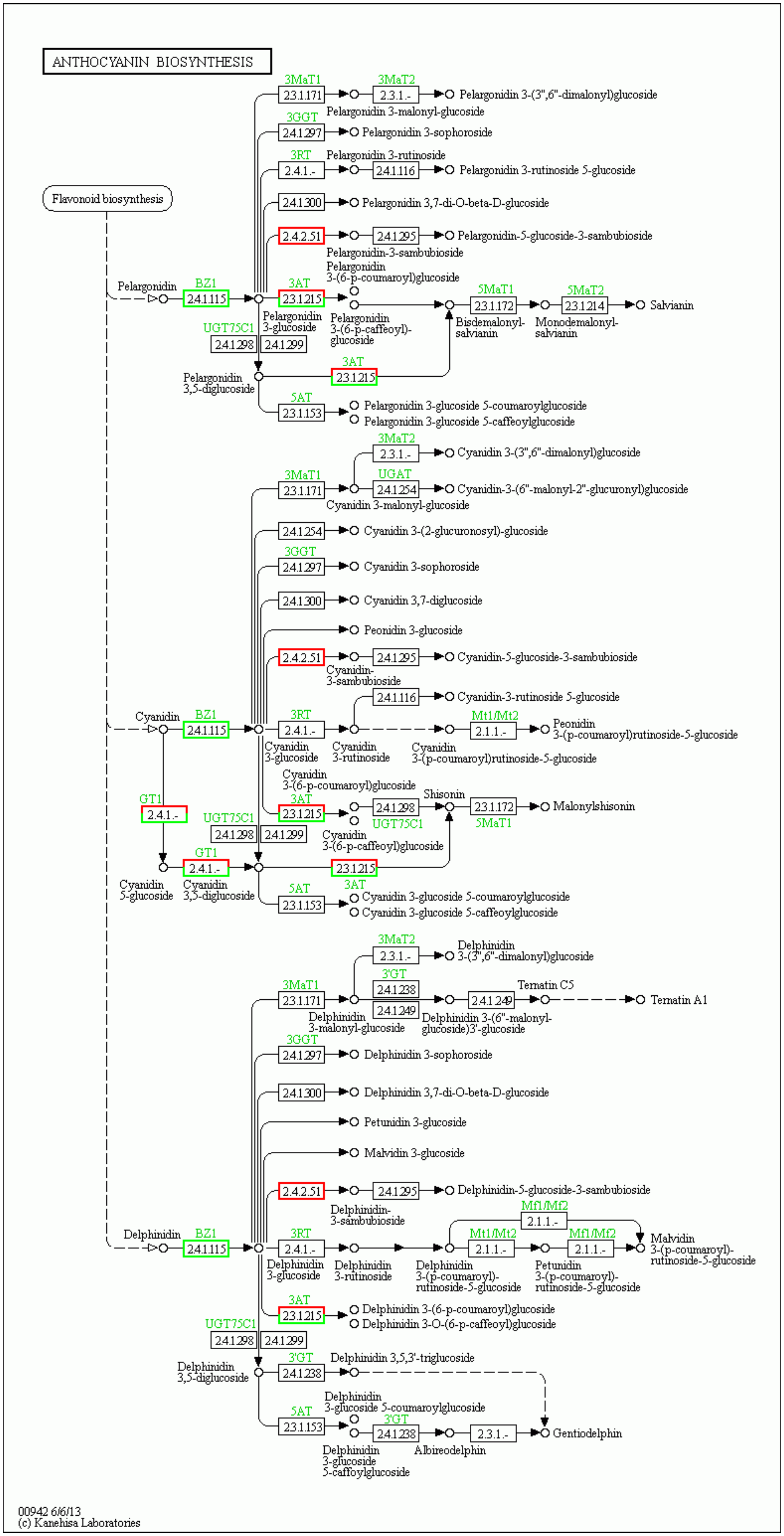
ANTHOCYANIN BIOSYNTHESIS
Flavonoid biosynthesis
3MaT1
2.3.1.171
3MaT2
2.3.1.-
Pelargonidin 3-(3",6"-dimalonyl)glucoside
Pelargonidin 3-malonyl-glucoside
3GGT
2.4.1.297
Pelargonidin 3-sophoroside
3RT
2.4.1.-
Pelargonidin 3-rutinoside
2.4.1.116
Pelargonidin 3-rutinoside 5-glucoside
2.4.1.300
Pelargonidin 3,7-di-O-beta-D-glucoside
2.4.2.51
2.4.1.295
Pelargonidin-5-glucoside-3-sambubioside
Pelargonidin-3-sambubioside
Pelargonidin 3-(6-p-coumaroyl)glucoside
Pelargonidin
BZ1
2.4.1.115
3AT
2.3.1.215
5MaT1
2.3.1.172
5MaT2
2.3.1.214
Salvianin
Pelargonidin 3-glucoside
Pelargonidin 3-(6-p-caffeoyl)-glucoside
Bisdemalonyl-salvianin
Monodemalonyl-salvianin
UGT75C1
2.4.1.298
2.4.1.299
Pelargonidin 3,5-diglucoside
5AT
2.3.1.153
Pelargonidin 3-glucoside 5-coumaroylglucoside
Pelargonidin 3-glucoside 5-caffeoylglucoside
Cyanidin 3-(3",6"-dimalonyl)glucoside
UGAT
2.4.1.254
Cyanidin-3-(6"-malonyl-2"-glucuronyl)glucoside
Cyanidin 3-malonyl-glucoside
Cyanidin 3-(2-glucuronosyl)-glucoside
Cyanidin 3-sophoroside
Cyanidin 3,7-diglucoside
Peonidin 3-glucoside
Cyanidin-5-glucoside-3-sambubioside
Cyanidin-3-sambubioside
Cyanidin-3-rutinoside 5-glucoside
Cyanidin
Mt1/Mt2
2.1.1.-
Peonidin 3-(p-coumaroyl)rutinoside-5-glucoside
Cyanidin 3-glucoside
Cyanidin 3-rutinoside
Cyanidin 3-(p-coumaroyl)rutinoside-5-glucoside
Cyanidin 3-(6-p-coumaroyl)glucoside
Shisonin
Malonylshisonin
GT1
2.4.1.-
Cyanidin 3-(6-p-caffeoyl)glucoside
Cyanidin 5-glucoside
Cyanidin 3,5-diglucoside
Cyanidin 3-glucoside 5-coumaroylglucoside
Cyanidin 3-glucoside 5-caffeoylglucoside
Delphinidin 3-(3",6"-dimalonyl)glucoside
3'GT
2.4.1.238
2.4.1.249
Ternatin C5
Ternatin A1
Delphinidin 3-malonyl-glucoside
Delphinidin 3-(6"-malonyl-glucoside)3'-glucoside
Delphinidin 3-sophoroside
Delphinidin 3,7-di-O-beta-D-glucoside
Petunidin 3-glucoside
Malvidin 3-glucoside
Delphinidin-5-glucoside-3-sambubioside
Delphinidin-3-sambubioside
Mf1/Mf2
Malvidin 3-(p-coumaroyl)-rutinoside-5-glucoside
Delphinidin
Delphinidin 3-glucoside
Delphinidin 3-rutinoside
Delphinidin 3-(p-coumaroyl)-rutinoside-5-glucoside
Petunidin 3-(p-coumaroyl)-rutinoside-5-glucoside
Delphinidin 3-(6-p-coumaroyl)glucoside
Delphinidin 3-O-(6-p-caffeoyl)glucoside
Delphinidin 3,5-diglucoside
Delphinidin 3,5,3'-triglucoside
Delphinidin 3-glucoside 5-coumaroylglucoside
Gentiodelphin
Delphinidin 3-glucoside 5-caffoylglucoside
Albireodelphin
00942 6/6/13
(c) Kanehisa Laboratories

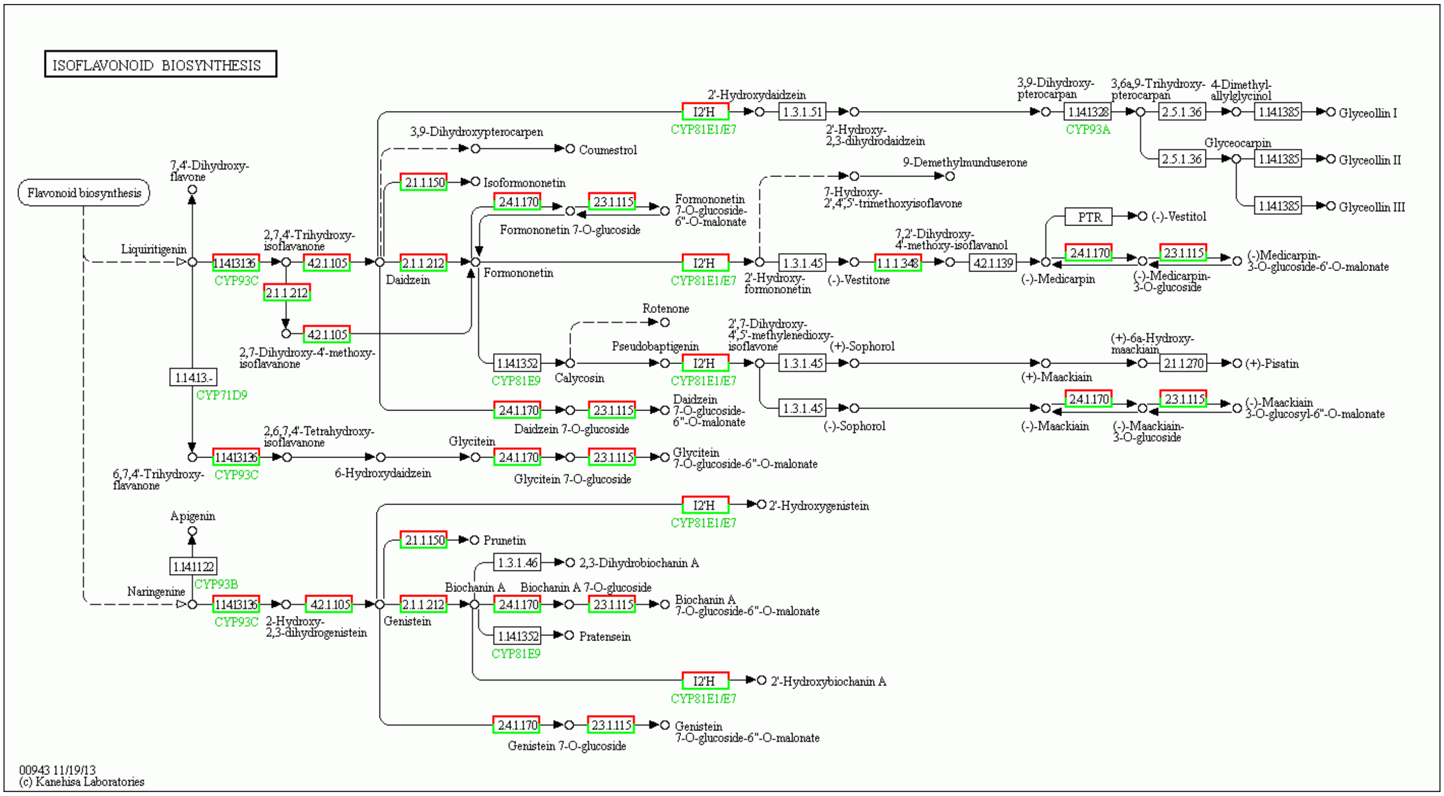
ISOFLAVONOID BIOSYNTHESIS
Flavonoid biosynthesis
7,4'-Dihydroxy-flavone
Liquiritigenin
1.14.13.136
CYP93C
2,7,4'-Trihydroxy-isoflavanone
4.2.1.105
2.1.1.212
2,7-Dihydroxy-4'-methoxy-isoflavanone
1.14.13.-
CYP71D9
Daidzein
2.1.1.150
Isoformononetin
2.4.1.170
2.3.1.115
Formononetin 7-O-glucoside
Formononetin 7-O-glucoside-6"-O-malonate
Formononetin
3,9-Dihydroxypterocarpen
Coumestrol
I2'H
CYP81E1/E7
2'-Hydroxydaidzein
1.3.1.51
2'-Hydroxy-2,3-dihydrodaidzein
3,9-Dihydroxy-pterocarpan
1.14.13.28
CYP93A
3,6a,9-Trihydroxy-pterocarpan
2.5.1.36
4-Dimethyl-allylglycinol
1.14.13.85
Glyceollin I
Glyceocarpin
Glyceollin II
Glyceollin III
9-Demethylmunduserone
7-Hydroxy-2',4',5'-trimethoxyisoflavone
2'-Hydroxy-formononetin
1.3.1.45
(-)-Vestitone
1.1.1.348
7,2'-Dihydroxy-4'-methoxy-isoflavanol
4.2.1.139
(-)-Medicarpin
PTR
(-)-Vestitol
(-)-Medicarpin-3-O-glucoside
(-)Medicarpin-3-O-glucoside-6'-O-malonate
Rotenone
1.14.13.52
CYP81E9
Calycosin
Pseudobaptigenin
2',7-Dihydroxy-4',5'-methylenedioxy-isoflavone
(+)-Sophorol
(+)-Maackiain
(+)-6a-Hydroxy-maackiain
2.1.1.270
(+)-Pisatin
(-)-Sophorol
(-)-Maackiain
(-)-Maackiain-3-O-glucoside
(-)-Maackiain-3-O-glucosyl-6"-O-malonate
Daidzein 7-O-glucoside
Daidzein 7-O-glucoside-6"-O-malonate
2,6,7,4'-Tetrahydroxy-isoflavanone
6,7,4'-Trihydroxy-flavanone
6-Hydroxydaidzein
Glycitein
Glycitein 7-O-glucoside
Glycitein 7-O-glucoside-6"-O-malonate
Apigenin
1.14.11.22
CYP93B
Naringenine
2-Hydroxy-2,3-dihydrogenistein
2'-Hydroxygenistein
Prunetin
1.3.1.46
2,3-Dihydrobiochanin A
Genistein
Biochanin A
Biochanin A 7-O-glucoside
Biochanin A 7-O-glucoside-6"-O-malonate
Pratensein
2'-Hydroxybiochanin A
Genistein 7-O-glucoside
Genistein 7-O-glucoside-6"-O-malonate
00943 11/19/13
(c) Kanehisa Laboratories

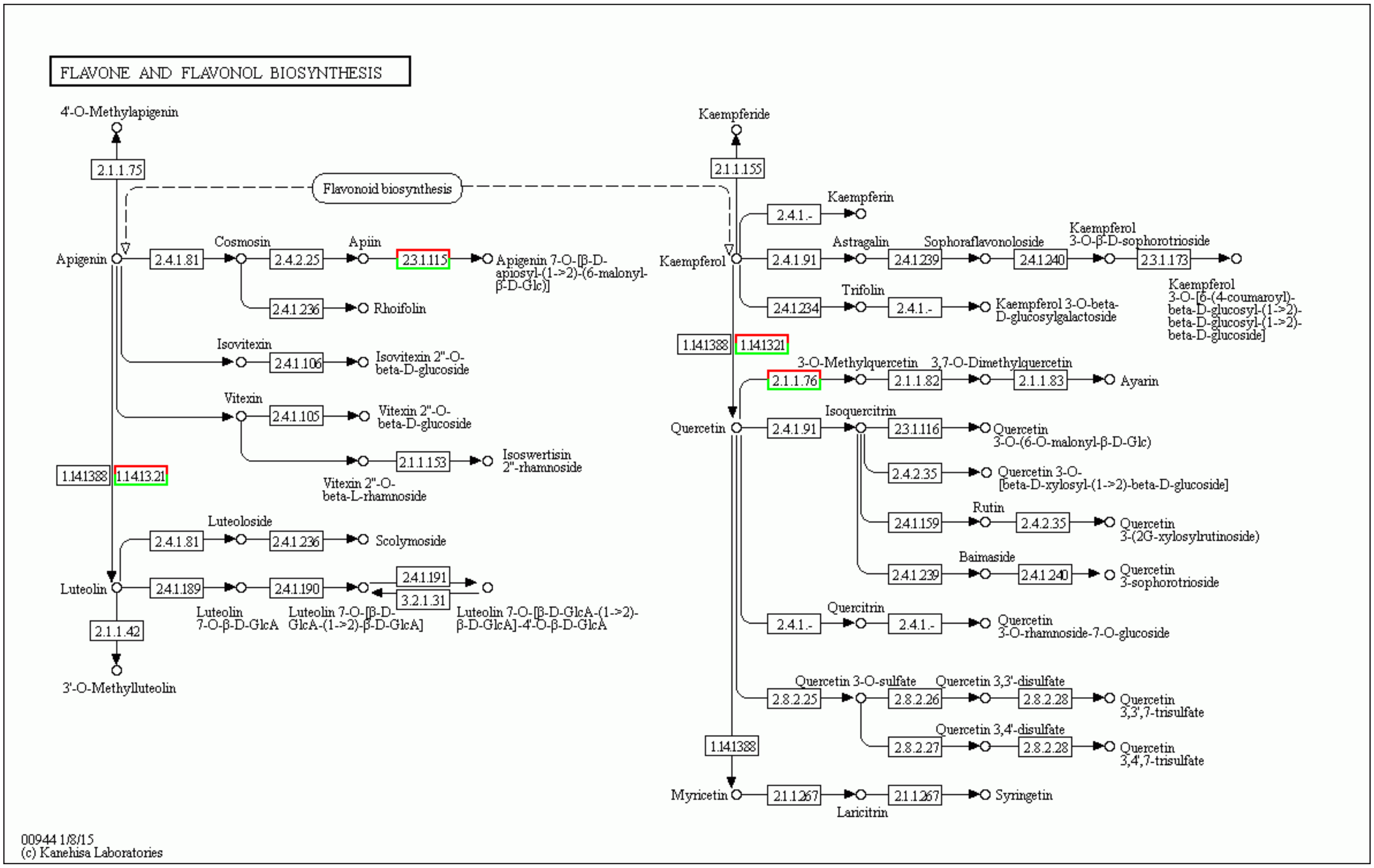
FLAVONE AND FLAVONOL BIOSYNTHESIS
4'-O-Methylapigenin
2.1.1.75
Flavonoid biosynthesis
Apigenin
2.4.1.81
Cosmosin
2.4.2.25
Apiin
2.3.1.115
Apigenin 7-O-[β-D-apiosyl-(1->2)-(6-malonyl-β-D-Glc)]
2.4.1.236
Rhoifolin
Isovitexin
2.4.1.106
Isovitexin 2"-O-beta-D-glucoside
Vitexin
2.4.1.105
Vitexin 2"-O-beta-D-glucoside
2.1.1.153
Isoswertisin 2"-rhamnoside
Vitexin 2"-O-beta-L-rhamnoside
1.14.13.88
1.14.13.21
Luteoloside
2.4.1.81
2.4.1.236
Scolymoside
Luteolin
2.4.1.189
2.4.1.190
2.4.1.191
3.2.1.31
Luteolin 7-O-β-D-GlcA
Luteolin 7-O-[β-D-GlcA-(1->2)-β-D-GlcA]
Luteolin 7-O-[β-D-GlcA-(1->2)-β-D-GlcA]-4'-O-β-D-GlcA
2.1.1.42
3'-O-Methylluteolin
Kaempferide
2.1.1.155
2.4.1.-
Kaempferin
Kaempferol
2.4.1.91
Astragalin
2.4.1.239
Sophoraflavonoloside
2.4.1.240
Kaempferol 3-O-β-D-sophorotrioside
2.3.1.173
Kaempferol 3-O-[6-(4-coumaroyl)-beta-D-glucosyl-(1->2)-beta-D-glucosyl-(1->2)-beta-D-glucoside]
2.4.1.234
Trifolin
Kaempferol 3-O-beta-D-glucosylgalactoside
3-O-Methylquercetin
3,7-O-Dimethylquercetin
2.1.1.76
2.1.1.82
2.1.1.83
Ayarin
Quercetin
Isoquercitrin
2.3.1.116
Quercetin 3-O-(6-O-malonyl-β-D-Glc)
2.4.2.35
Quercetin 3-O-[beta-D-xylosyl-(1->2)-beta-D-glucoside]
Rutin
2.4.1.159
Quercetin 3-(2G-xylosylrutinoside)
Baimaside
Quercetin 3-sophorotrioside
Quercitrin
Quercetin 3-O-rhamnoside-7-O-glucoside
Quercetin 3-O-sulfate
Quercetin 3,3'-disulfate
2.8.2.25
2.8.2.26
2.8.2.28
Quercetin 3,3',7-trisulfate
Quercetin 3,4'-disulfate
2.8.2.27
Quercetin 3,4',7-trisulfate
Myricetin
2.1.1.267
Laricitrin
Syringetin
00944 1/8/15
(c) Kanehisa Laboratories

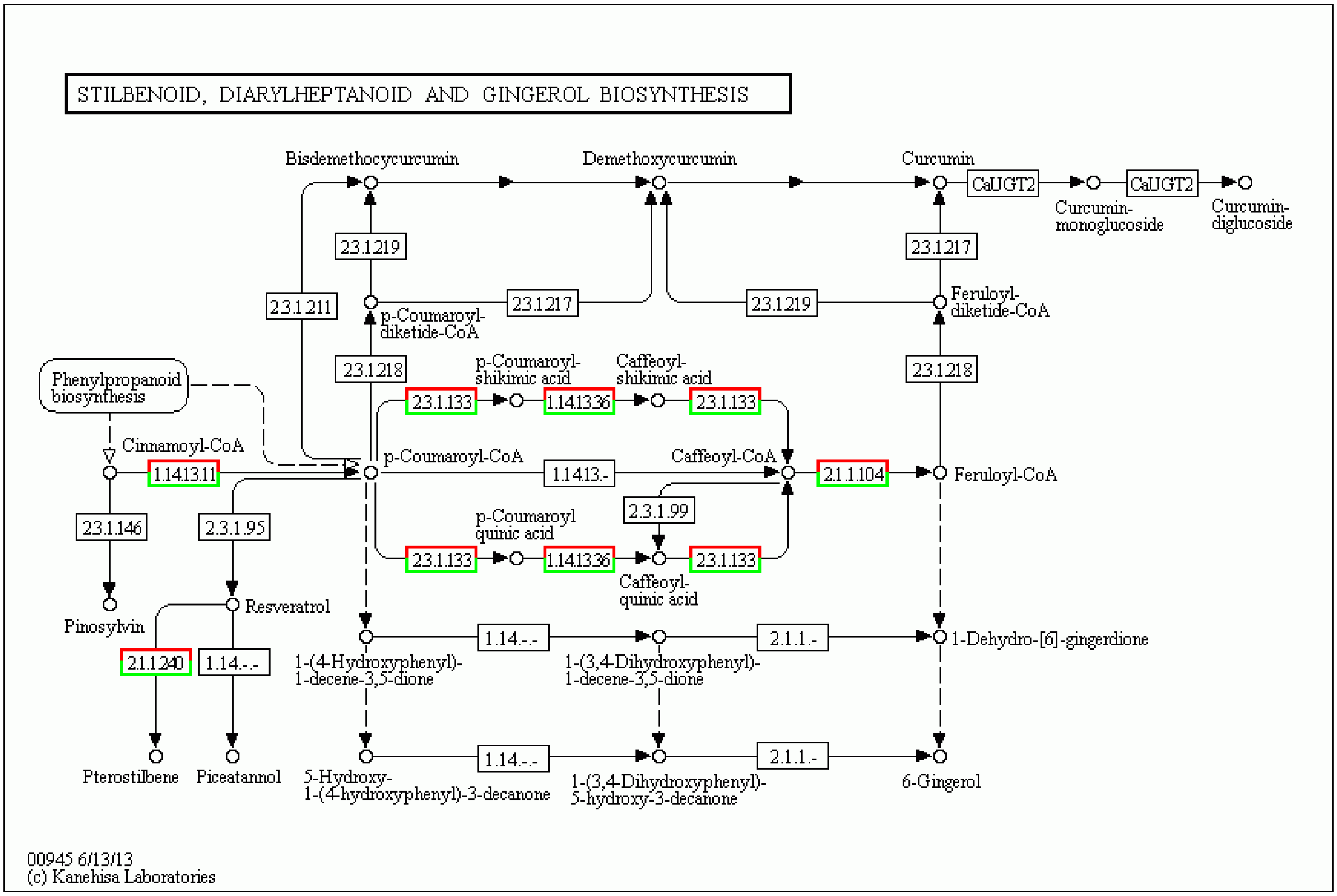
STILBENOID, DIARYLHEPTANOID AND GINGEROL BIOSYNTHESIS
Bisdemethocycurcumin
Demethoxycurcumin
Curcumin
CaUGT2
Curcumin-monoglucoside
CaUGT2
Curcumin-diglucoside
2.3.1.219
2.3.1.217
2.3.1.211
p-Coumaroyl-diketide-CoA
2.3.1.217
2.3.1.219
Feruloyl-diketide-CoA
2.3.1.218
2.3.1.218
Phenylpropanoid biosynthesis
p-Coumaroyl-shikimic acid
Caffeoyl-shikimic acid
2.3.1.133
1.14.13.36
2.3.1.133
Cinnamoyl-CoA
p-Coumaroyl-CoA
Caffeoyl-CoA
1.14.13.11
1.14.13.-
2.1.1.104
Feruloyl-CoA
2.3.1.99
2.3.1.146
2.3.1.95
p-Coumaroyl quinic acid
2.3.1.133
1.14.13.36
2.3.1.133
Caffeoyl-quinic acid
Pinosylvin
Resveratrol
1.14.-.-
2.1.1.-
1-Dehydro-[6]-gingerdione
2.1.1.240
1.14.-.-
1-(4-Hydroxyphenyl)-1-decene-3,5-dione
1-(3,4-Dihydroxyphenyl)-1-decene-3,5-dione
1.14.-.-
2.1.1.-
Pterostilbene
Piceatannol
5-Hydroxy-1-(4-hydroxyphenyl)-3-decanone
1-(3,4-Dihydroxyphenyl)-5-hydroxy-3-decanone
6-Gingerol
00945 6/13/13
(c) Kanehisa Laboratories

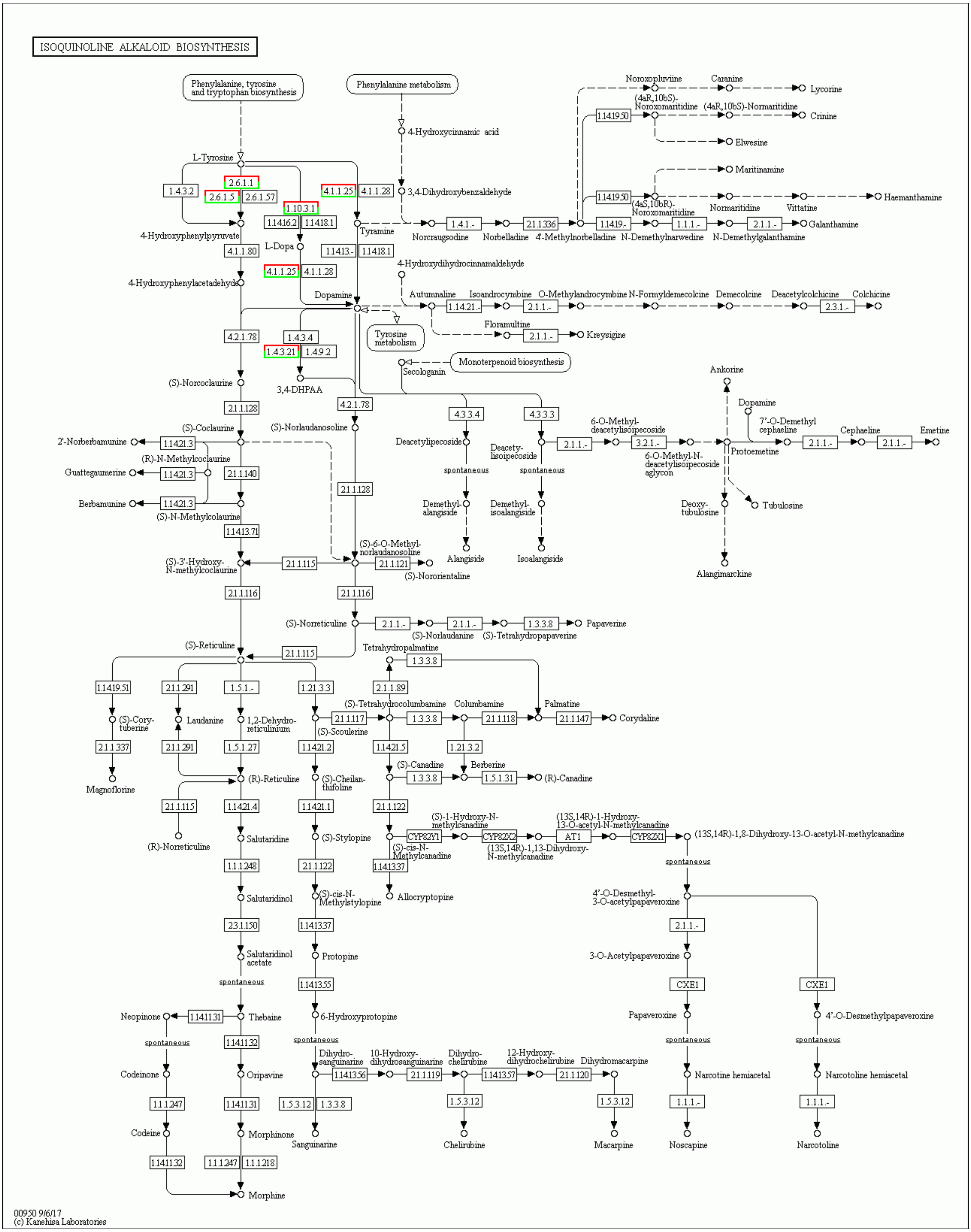
ISOQUINOLINE ALKALOID BIOSYNTHESIS
Phenylalanine, tyrosine and tryptophan biosynthesis
Phenylalanine metabolism
4-Hydroxycinnamic acid
L-Tyrosine
2.6.1.1
1.4.3.2
2.6.1.5
2.6.1.57
1.10.3.1
4.1.1.25
4.1.1.28
3,4-Dihydroxybenzaldehyde
1.14.16.2
1.14.18.1
4-Hydroxyphenylpyruvate
L-Dopa
Tyramine
1.14.13.-
4.1.1.80
4-Hydroxyphenylacetaldehyde
4-Hydroxydihydrocinnamaldehyde
Dopamine
Tyrosine metabolism
4.2.1.78
1.4.3.4
1.4.3.21
1.4.9.2
3,4-DHPAA
(S)-Norcoclaurine
2.1.1.128
(S)-Coclaurine
2'-Norberbamunine
2.1.1.140
(R)-N-Methylcoclaurine
Guattegaumerine
Berbamunine
1.14.21.3
(S)-N-Methylcoclaurine
1.14.13.71
(S)-3'-Hydroxy-N-methylcoclaurine
2.1.1.116
(S)-Reticuline
(S)-Norlaudanosoline
(S)-6-O-Methyl-norlaudanosoline
2.1.1.115
2.1.1.121
(S)-Nororientaline
(S)-Norreticuline
2.1.1.-
(S)-Norlaudanine
(S)-Tetrahydropapaverine
1.3.3.8
Papaverine
Tetrahydropalmatine
2.1.1.89
(S)-Tetrahydrocolumbamine
Columbamine
Palmatine
2.1.1.117
2.1.1.118
2.1.1.147
Corydaline
(S)-Scoulerine
1.21.3.3
1.21.3.2
1.14.21.5
(S)-Canadine
Berberine
1.5.1.31
(R)-Canadine
2.1.1.122
(S)-1-Hydroxy-N-methylcanadine
CYP82Y1
CYP82X2
AT1
CYP82X1
(13S,14R)-1-Hydroxy-13-O-acetyl-N-methylcanadine
(13S,14R)-1,8-Dihydroxy-13-O-acetyl-N-methylcanadine
(S)-cis-N-Methylcanadine
(13S,14R)-1,13-Dihydroxy-N-methylcanadine
1.14.13.37
Allocryptopine
1.14.19.51
(S)-Cory-tuberine
2.1.1.337
Magnoflorine
2.1.1.291
Laudanine
1.5.1.-
1,2-Dehydro-reticulinium
1.5.1.27
(R)-Reticuline
(R)-Norreticuline
1.14.21.4
Salutaridine
1.1.1.248
Salutaridinol
2.3.1.150
Salutaridinol acetate
spontaneous
Thebaine
Neopinone
1.14.11.31
1.14.11.32
Codeinone
Oripavine
1.1.1.247
Codeine
Morphinone
1.1.1.218
Morphine
1.14.21.2
(S)-Cheilan-thifoline
1.14.21.1
(S)-Stylopine
(S)-cis-N-Methylstylopine
Protopine
1.14.13.55
6-Hydroxyprotopine
Dihydro-sanguinarine
10-Hydroxy-dihydrosanguinarine
Dihydro-chelirubine
12-Hydroxy-dihydrochelirubine
Dihydromacarpine
1.14.13.56
2.1.1.119
1.14.13.57
2.1.1.120
1.5.3.12
Sanguinarine
Chelirubine
Macarpine
4'-O-Desmethyl-3-O-acetylpapaveroxine
3-O-Acetylpapaveroxine
CXE1
Papaveroxine
4'-O-Desmethylpapaveroxine
Narcotine hemiacetal
Narcotoline hemiacetal
1.1.1.-
Noscapine
Narcotoline
Noroxopluviine
Caranine
Lycorine
(4aR,10bS)-Noroxomaritidine
(4aS,10bS)-Normaritidine
Crinine
1.14.19.50
Elwesine
Maritinamine
(4aS,10bR)-Noroxomaritidine
Normaritidine
Vittatine
Haemanthamine
1.4.1.-
2.1.1.336
1.14.19.-
Norcraugsodine
Norbelladine
4'-Methylnorbelladine
N-Demethylnarwedine
N-Demethylgalanthamine
Galanthamine
Autumnaline
Isoandrocymbine
O-Methylandrocymbine
N-Formyldemecolcine
Demecolcine
Deacetylcolchicine
Colchicine
1.14.21.-
2.3.1.-
Floramultine
Kreysigine
Monoterpenoid biosynthesis
Secologanin
4.3.3.4
4.3.3.3
Deacetylipecoside
Deacetylisoipecoside
6-O-Methyl-deacetylisoipecoside
3.2.1.-
6-O-Methyl-N-deacetylisoipecoside aglycon
Protoemetine
Ankorine
7'-O-Demethyl cephaeline
Cephaeline
Emetine
Demethyl-alangiside
Demethyl-isoalangiside
Alangiside
Isoalangiside
Deoxy-tubulosine
Tubulosine
Alangimarckine
00950 9/6/17
(c) Kanehisa Laboratories

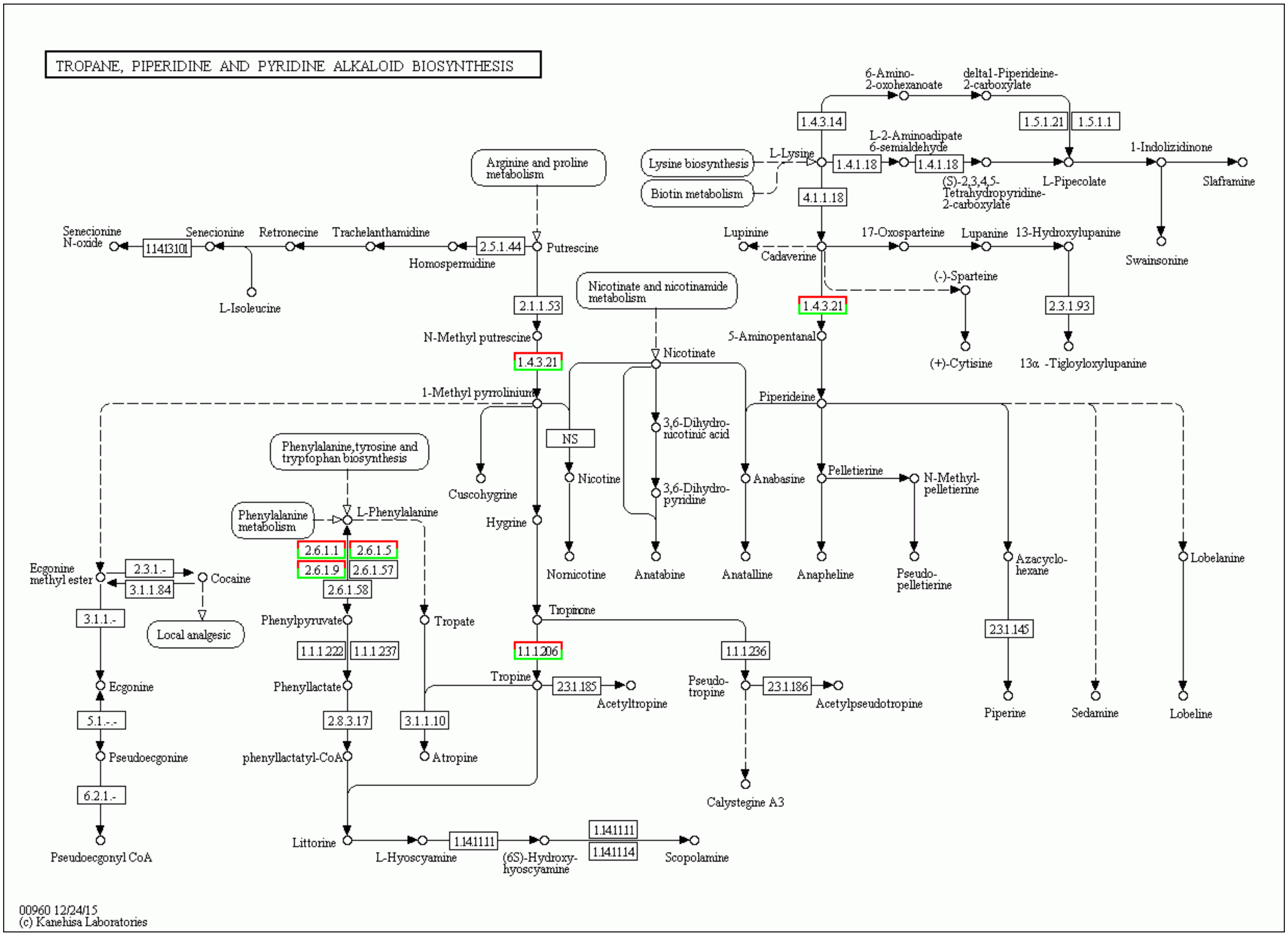
TROPANE, PIPERIDINE AND PYRIDINE ALKALOID BIOSYNTHESIS
6-Amino-2-oxohexanoate
delta1-Piperideine-2-carboxylate
1.4.3.14
1.5.1.21
1.5.1.1
L-2-Aminoadipate 6-semialdehyde
Arginine and proline metabolism
Lysine biosynthesis
L-Lysine
1.4.1.18
1.4.1.18
1-Indolizidinone
Slaframine
Biotin metabolism
(S)-2,3,4,5-Tetrahydropyridine-2-carboxylate
L-Pipecolate
4.1.1.18
Senecionine N-oxide
1.14.13.101
Senecionine
Retronecine
Trachelanthamidine
2.5.1.44
Putrescine
Homospermidine
Lupinine
Cadaverine
17-Oxosparteine
Lupanine
13-Hydroxylupanine
Swainsonine
L-Isoleucine
Nicotinate and nicotinamide metabolism
(-)-Sparteine
2.1.1.53
1.4.3.21
2.3.1.93
N-Methyl putrescine
5-Aminopentanal
1.4.3.21
Nicotinate
(+)-Cytisine
13α -Tigloyloxylupanine
1-Methyl pyrrolinium
Piperideine
3,6-Dihydro-nicotinic acid
NS
Phenylalanine,tyrosine and tryptophan biosynthesis
Cuscohygrine
Nicotine
Anabasine
Pelletierine
N-Methyl-pelletierine
3,6-Dihydro-pyridine
Phenylalanine metabolism
L-Phenylalanine
Hygrine
2.6.1.1
2.6.1.5
2.6.1.9
2.6.1.57
Ecgonine methyl ester
2.3.1.-
3.1.1.84
Cocaine
2.6.1.58
Nornicotine
Anatabine
Anatalline
Anapheline
Pseudo-pelletierine
Azacyclo-hexane
Lobelanine
3.1.1.-
Local analgesic
Phenylpyruvate
Tropate
Tropinone
2.3.1.145
1.1.1.222
1.1.1.237
1.1.1.206
1.1.1.236
Ecgonine
Phenyllactate
Tropine
2.3.1.185
Acetyltropine
Pseudo-tropine
2.3.1.186
Acetylpseudotropine
Piperine
Sedamine
Lobeline
5.1.-.-
2.8.3.17
3.1.1.10
Pseudoecgonine
phenyllactatyl-CoA
Atropine
6.2.1.-
Calystegine A3
1.14.11.11
Littorine
1.14.11.11
L-Hyoscyamine
(6S)-Hydroxy-hyoscyamine
1.14.11.14
Scopolamine
Pseudoecgonyl CoA
00960 12/24/15
(c) Kanehisa Laboratories

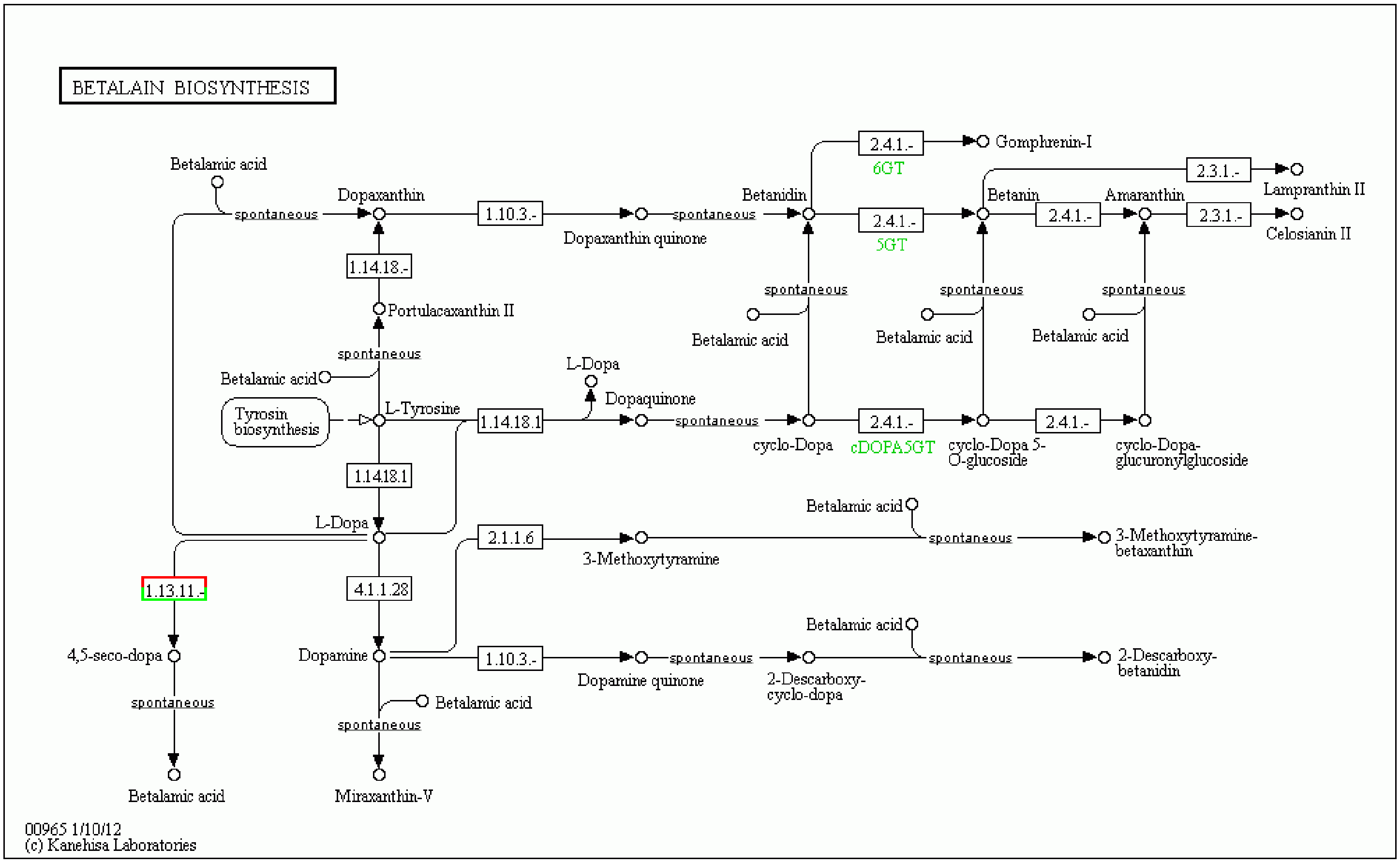
BETALAIN BIOSYNTHESIS
Betalamic acid
spontaneous
Dopaxanthin
1.10.3.-
Dopaxanthin quinone
Betanidin
2.4.1.-
6GT
Gomphrenin-I
2.3.1.-
Lampranthin II
Betanin
5GT
Amaranthin
Celosianin II
1.14.18.-
Portulacaxanthin II
L-Dopa
Dopaquinone
Tyrosin biosynthesis
L-Tyrosine
1.14.18.1
cyclo-Dopa
cDOPA5GT
cyclo-Dopa 5-O-glucoside
cyclo-Dopa-glucuronylglucoside
2.1.1.6
3-Methoxytyramine
3-Methoxytyramine-betaxanthin
1.13.11.-
4.1.1.28
4,5-seco-dopa
Dopamine
Dopamine quinone
2-Descarboxy-cyclo-dopa
2-Descarboxy-betanidin
Miraxanthin-V
00965 1/10/12
(c) Kanehisa Laboratories

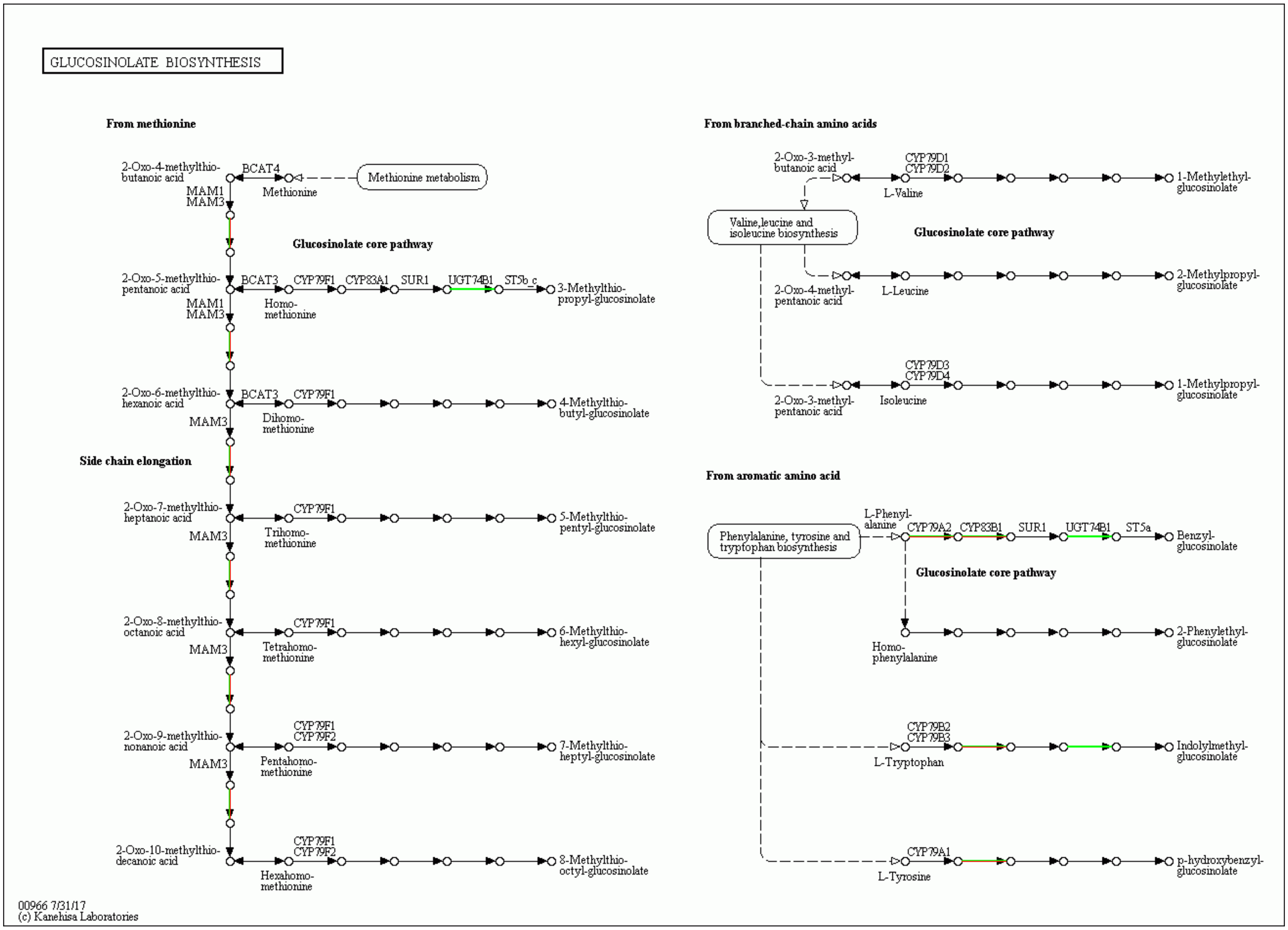
GLUCOSINOLATE BIOSYNTHESIS
From methionine
2-Oxo-4-methylthio-butanoic acid
BCAT4
Methionine
Methionine metabolism
MAM1
MAM3
Glucosinolate core pathway
2-Oxo-5-methylthio-pentanoic acid
BCAT3
CYP79F1
CYP83A1
SUR1
UGT74B1
ST5b_c
Homo-methionine
3-Methylthio-propyl-glucosinolate
2-Oxo-6-methylthio-hexanoic acid
Dihomo-methionine
4-Methylthio-butyl-glucosinolate
Side chain elongation
2-Oxo-7-methylthio-heptanoic acid
Trihomo-methionine
5-Methylthio-pentyl-glucosinolate
2-Oxo-8-methylthio-octanoic acid
Tetrahomo-methionine
6-Methylthio-hexyl-glucosinolate
2-Oxo-9-methylthio-nonanoic acid
CYP79F2
Pentahomo-methionine
7-Methylthio-heptyl-glucosinolate
2-Oxo-10-methylthio-decanoic acid
Hexahomo-methionine
8-Methylthio-octyl-glucosinolate
From branched-chain amino acids
2-Oxo-3-methyl-butanoic acid
CYP79D1
CYP79D2
L-Valine
1-Methylethyl-glucosinolate
Valine, leucine and isoleucine biosynthesis
Glucosinolate core pathway
2-Oxo-4-methyl-pentanoic acid
L-Leucine
2-Methylpropyl-glucosinolate
CYP79D3
CYP79D4
2-Oxo-3-methyl-pentanoic acid
Isoleucine
1-Methylpropyl-glucosinolate
From aromatic amino acid
Phenylalanine, tyrosine and tryptophan biosynthesis
L-Phenyl-alanine
CYP79A2
CYP83B1
SUR1
UGT74B1
ST5a
Benzyl-glucosinolate
Glucosinolate core pathway
Homo-phenylalanine
2-Phenylethyl-glucosinolate
CYP79B2
CYP79B3
L-Tryptophan
Indolylmethyl-glucosinolate
CYP79A1
L-Tyrosine
p-hydroxybenzyl-glucosinolate
00966 7/31/17
(c) Kanehisa Laboratories

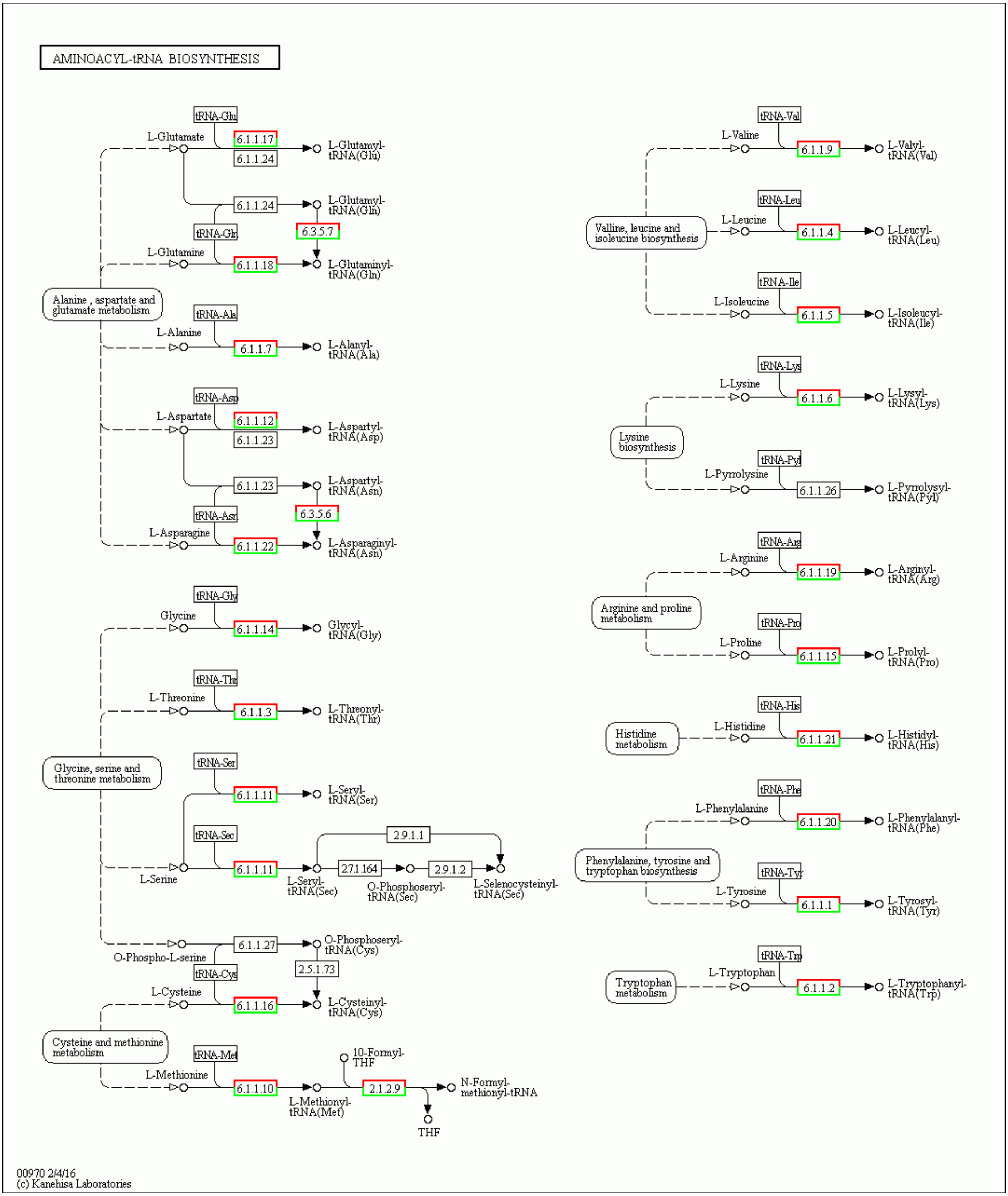
AMINOACYL-tRNA BIOSYNTHESIS
tRNA-Glu
L-Glutamate
6.1.1.17
6.1.1.24
L-Glutamyl-tRNA(Glu)
L-Glutamyl-tRNA(Gln)
6.3.5.7
tRNA-Gln
L-Glutamine
6.1.1.18
L-Glutaminyl-tRNA(Gln)
Alanine , aspartate and glutamate metabolism
tRNA-Ala
L-Alanine
6.1.1.7
L-Alanyl-tRNA(Ala)
tRNA-Asp
L-Aspartate
6.1.1.12
6.1.1.23
L-Aspartyl-tRNA(Asp)
L-Aspartyl-tRNA(Asn)
6.3.5.6
tRNA-Asn
L-Asparagine
6.1.1.22
L-Asparaginyl-tRNA(Asn)
tRNA-Gly
Glycine
6.1.1.14
Glycyl-tRNA(Gly)
tRNA-Thr
L-Threonine
6.1.1.3
L-Threonyl-tRNA(Thr)
Glycine, serine and threonine metabolism
tRNA-Ser
6.1.1.11
L-Seryl-tRNA(Ser)
tRNA-Sec
2.9.1.1
L-Serine
L-Seryl-tRNA(Sec)
2.7.1.164
O-Phosphoseryl-tRNA(Sec)
2.9.1.2
L-Selenocysteinyl-tRNA(Sec)
O-Phospho-L-serine
6.1.1.27
O-Phosphoseryl-tRNA(Cys)
2.5.1.73
tRNA-Cys
L-Cysteine
6.1.1.16
L-Cysteinyl-tRNA(Cys)
Cysteine and methionine metabolism
tRNA-Met
L-Methionine
6.1.1.10
L-Methionyl-tRNA(Met)
10-Formyl-THF
2.1.2.9
N-Formyl-methionyl-tRNA
THF
tRNA-Val
L-Valine
6.1.1.9
L-Valyl-tRNA(Val)
Valline, leucine and isoleucine biosynthesis
tRNA-Leu
L-Leucine
6.1.1.4
L-Leucyl-tRNA(Leu)
tRNA-Ile
L-Isoleucine
6.1.1.5
L-Isoleucyl-tRNA(Ile)
tRNA-Lys
L-Lysine
6.1.1.6
L-Lysyl-tRNA(Lys)
Lysine biosynthesis
tRNA-Pyl
L-Pyrrolysine
6.1.1.26
L-Pyrrolysyl-tRNA(Pyl)
tRNA-Arg
L-Arginine
6.1.1.19
L-Arginyl-tRNA(Arg)
Arginine and proline metabolism
tRNA-Pro
L-Proline
6.1.1.15
L-Prolyl-tRNA(Pro)
tRNA-His
Histidine metabolism
L-Histidine
6.1.1.21
L-Histidyl-tRNA(His)
tRNA-Phe
L-Phenylalanine
6.1.1.20
L-Phenylalanyl-tRNA(Phe)
Phenylalanine, tyrosine and tryptophan biosynthesis
tRNA-Tyr
L-Tyrosine
6.1.1.1
L-Tyrosyl-tRNA(Tyr)
tRNA-Trp
Tryptophan metabolism
L-Tryptophan
6.1.1.2
L-Tryptophanyl-tRNA(Trp)
00970 2/4/16
(c) Kanehisa Laboratories

BIOSYNTHESIS OF UNSATURATED FATTY ACIDS

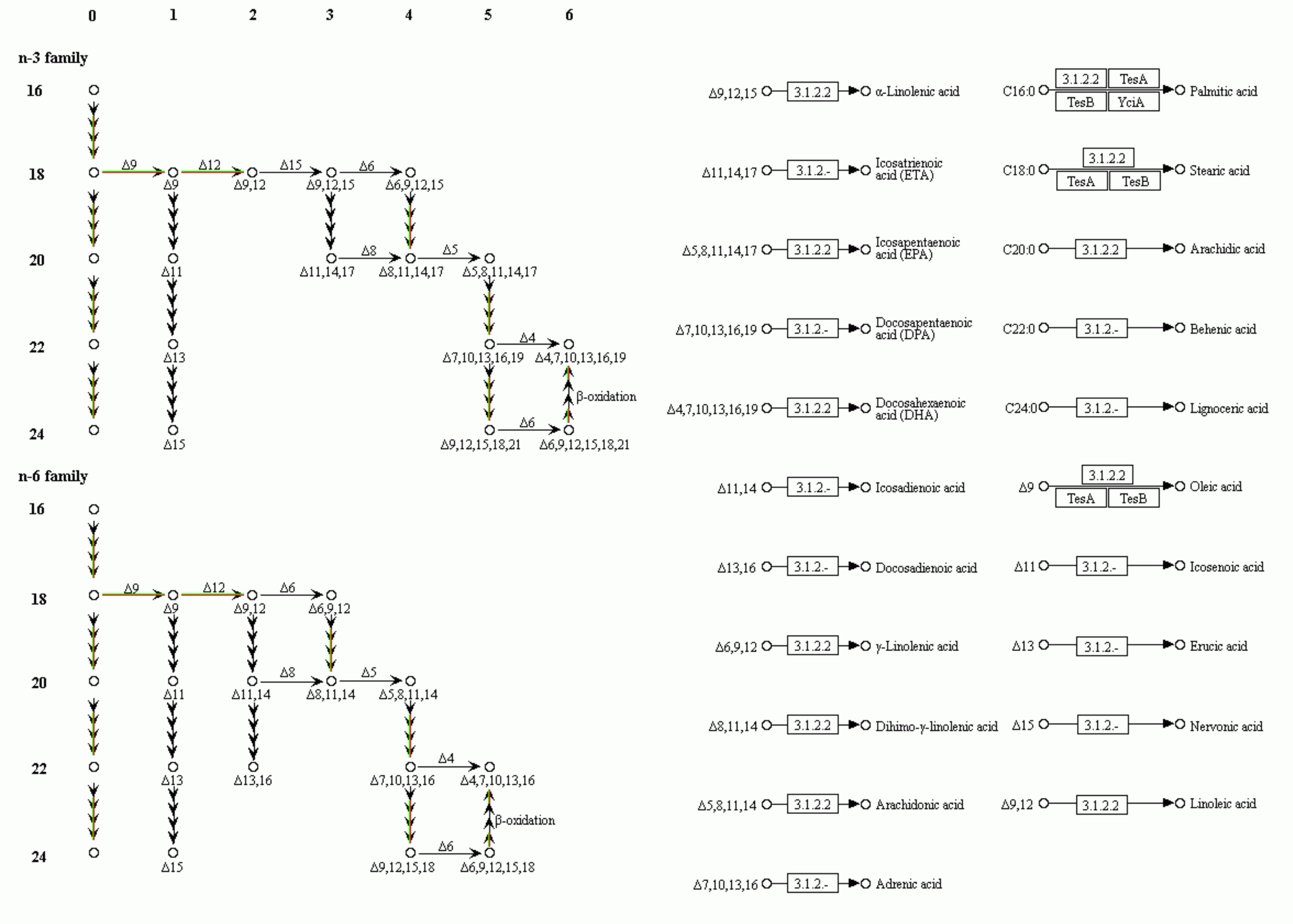

01040 3/17/16

(c) Kanehisa Laboratories

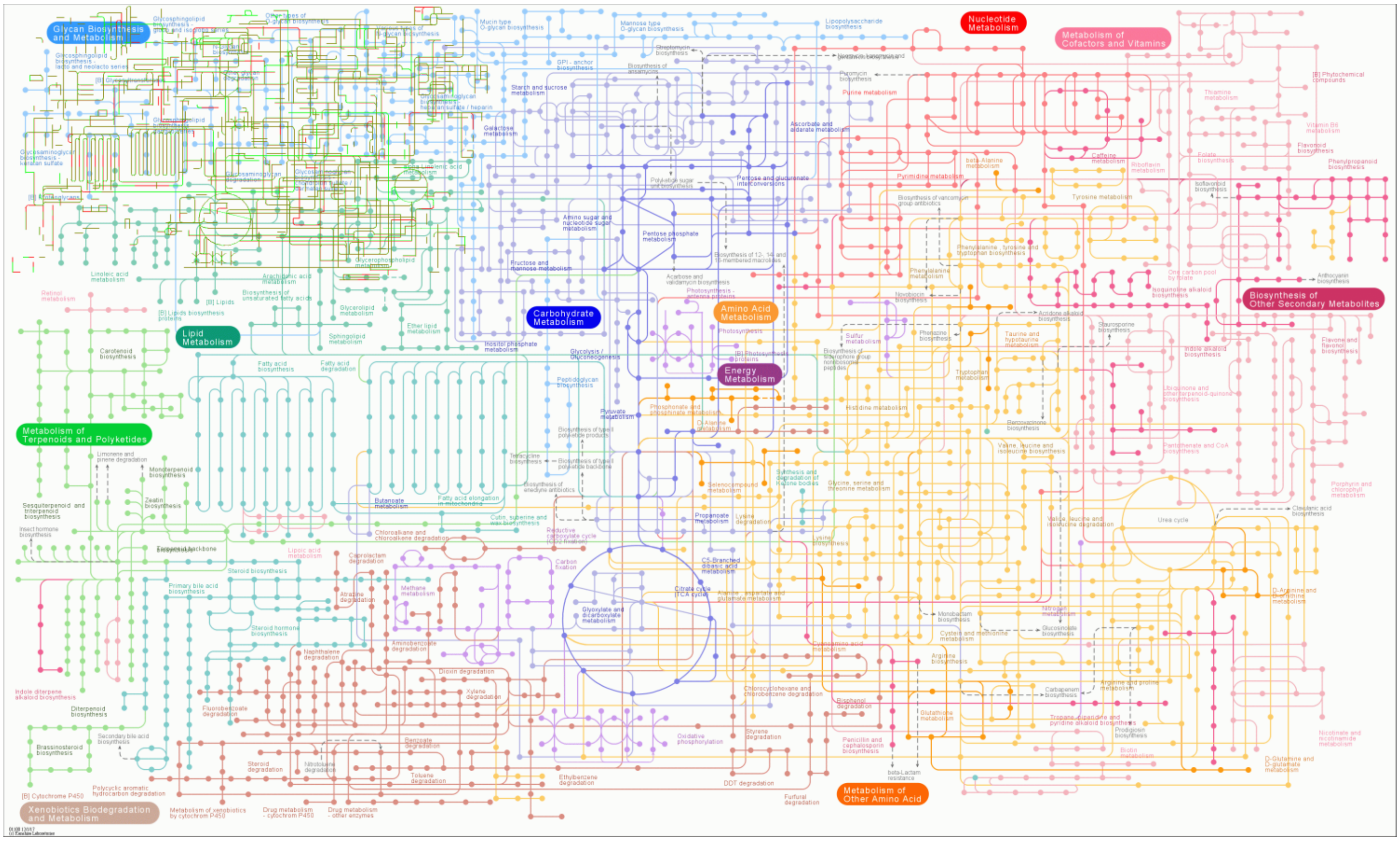
Glycan Biosynthesis and Metabolism
Nucleotide Metabolism
Metabolism of Cofactors and Vitamins
Lipid Metabolism
Carbohydrate Metabolism
Amino Acid Metabolism
Energy Metabolism
Metabolism of Terpenoids and Polyketides
Biosynthesis of Other Secondary Metabolites
Xenobiotics Biodegradation and Metabolism
Metabolism of Other Amino Acid

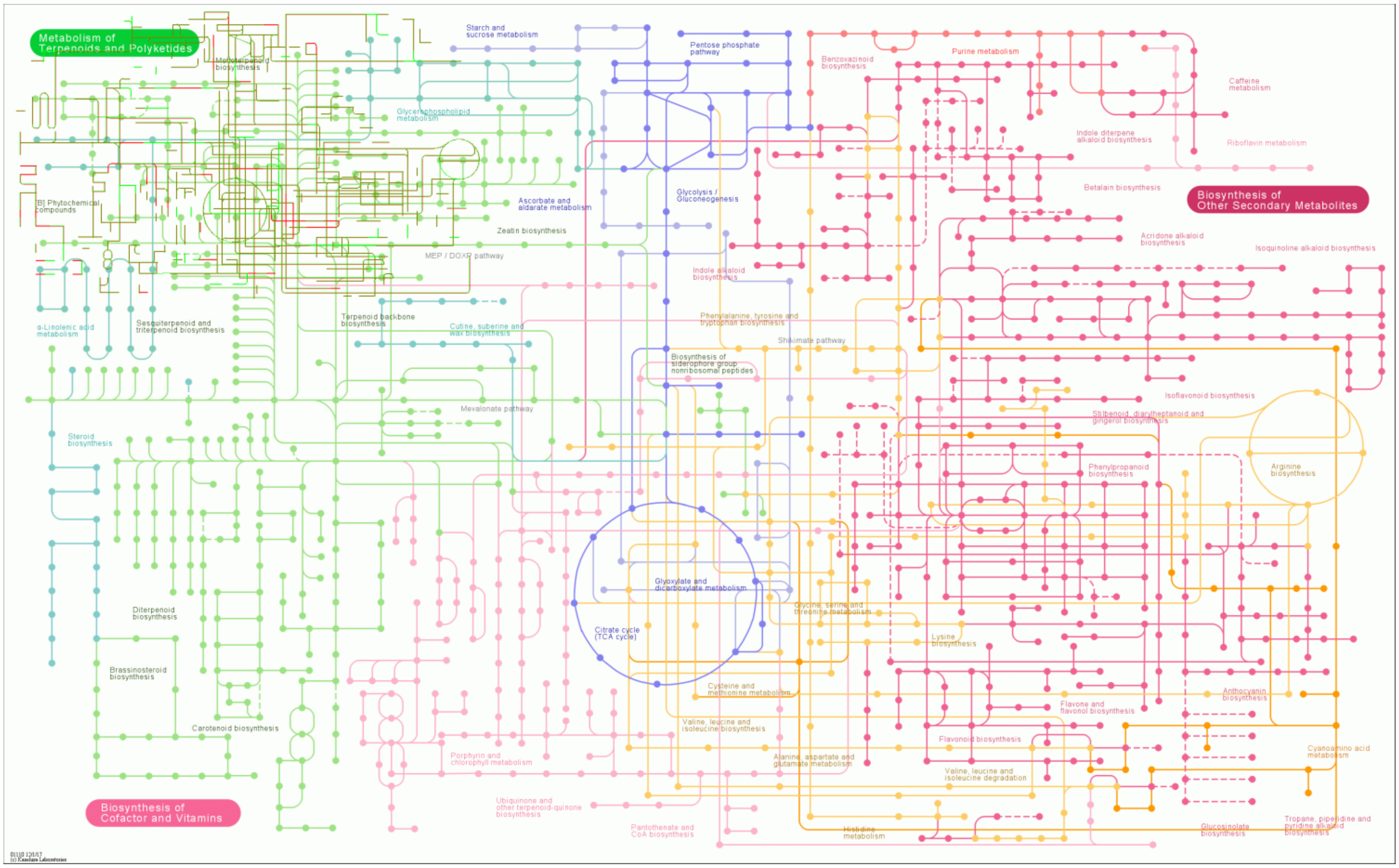
Metabolism of Terpenoids and Polyketides
Starch and sucrose metabolism
Pentose phosphate pathway
Purine metabolism
Caffeine metabolism
Riboflavin metabolism
Biosynthesis of Other Secondary Metabolites
Glycolysis / Gluconeogenesis
Citrate cycle (TCA cycle)
Glyoxylate and dicarboxylate metabolism
Arginine biosynthesis
Steroid biosynthesis
Diterpenoid biosynthesis
Brassinosteroid biosynthesis
Carotenoid biosynthesis
Biosynthesis of Cofactor and Vitamins

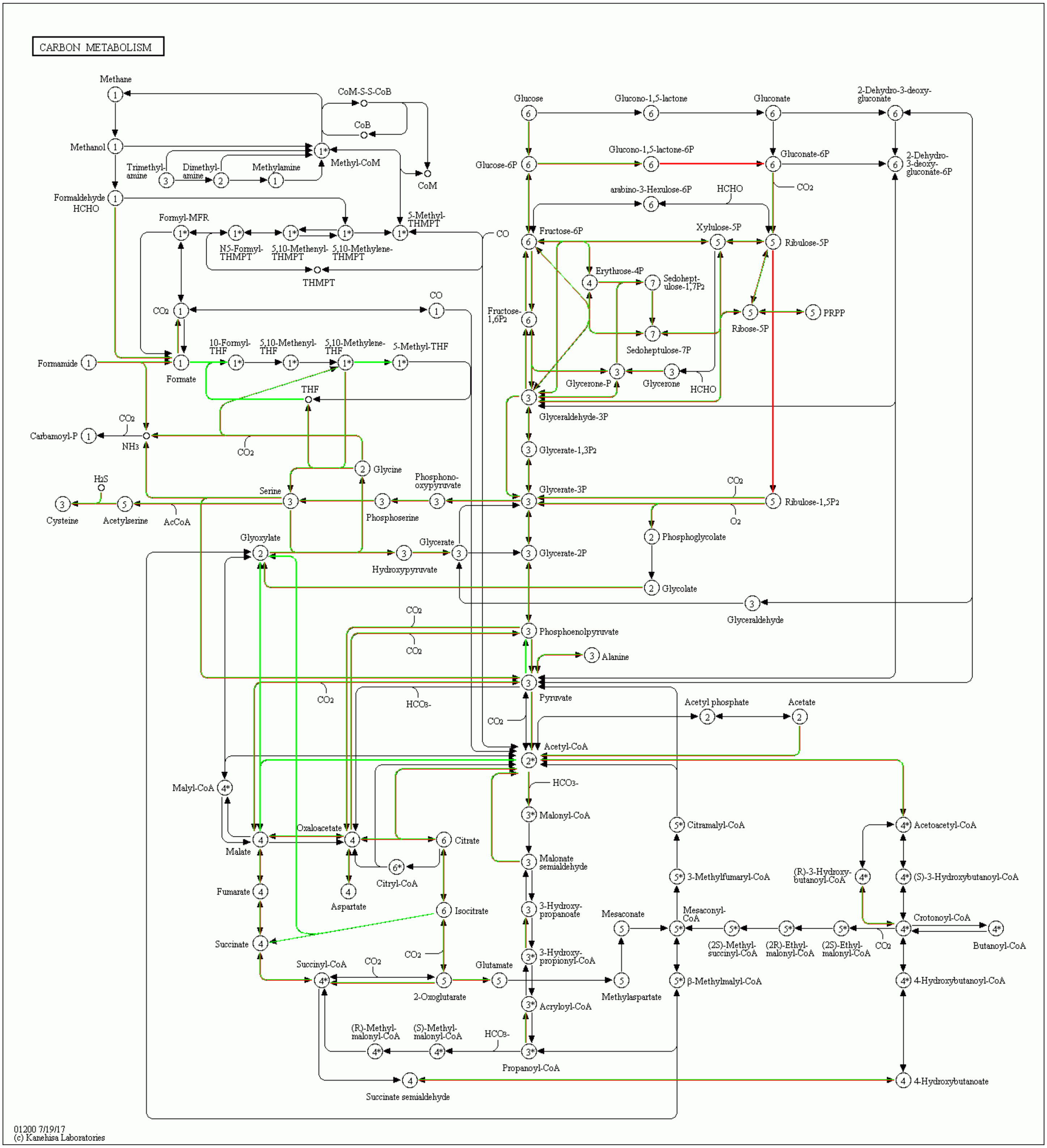
CARBON METABOLISM
Methane
CoM-S-S-CoB
CoB
Methanol
Methyl-CoM
Trimethyl-amine
Dimethyl-amine
Methylamine
CoM
Formaldehyde
HCHO
Formyl-MFR
5-Methyl-THMPT
N5-Formyl-THMPT
5,10-Methenyl-THMPT
5,10-Methylene-THMPT
CO
THMPT
CO2
10-Formyl-THF
5,10-Methenyl-THF
5,10-Methylene-THF
5-Methyl-THF
Formamide
Formate
THF
Carbamoyl-P
NH3
H2S
Cysteine
Acetylserine
AcCoA
Serine
Glycine
Phosphono-oxypyruvate
Phosphoserine
Glyoxylate
Glycerate
Hydroxypyruvate
Glucose
Glucono-1,5-lactone
Gluconate
2-Dehydro-3-deoxy-gluconate
Glucose-6P
Glucono-1,5-lactone-6P
Gluconate-6P
2-Dehydro-3-deoxy-gluconate-6P
arabino-3-Hexulose-6P
HCHO
Xylulose-5P
Fructose-6P
Ribulose-5P
Erythrose-4P
Sedohept-ulose-1,7P2
Fructose-1,6P2
Ribose-5P
PRPP
Sedoheptulose-7P
Glycerone-P
Glycerone
Glyceraldehyde-3P
Glycerate-1,3P2
Glycerate-3P
Ribulose-1,5P2
O2
Phosphoglycolate
Glycerate-2P
Glycolate
Glyceraldehyde
Phosphoenolpyruvate
Alanine
Pyruvate
HCO3-
Acetyl phosphate
Acetate
Acetyl-CoA
Malyl-CoA
Malonyl-CoA
Citramalyl-CoA
Acetoacetyl-CoA
Oxaloacetate
Citrate
Malate
Malonate semialdehyde
3-Methylfumaryl-CoA
(R)-3-Hydroxy-butanoyl-CoA
(S)-3-Hydroxybutanoyl-CoA
Citryl-CoA
Fumarate
Aspartate
Isocitrate
3-Hydroxy-propanoate
Mesaconate
Mesaconyl-CoA
Crotonoyl-CoA
Succinate
(2S)-Methyl-succinyl-CoA
(2R)-Ethyl-malonyl-CoA
(2S)-Ethyl-malonyl-CoA
Butanoyl-CoA
3-Hydroxy-propionyl-CoA
Succinyl-CoA
Glutamate
2-Oxoglutarate
Methylaspartate
β-Methylmalyl-CoA
4-Hydroxybutanoyl-CoA
Acryloyl-CoA
(R)-Methyl-malonyl-CoA
(S)-Methyl-malonyl-CoA
Propanoyl-CoA
Succinate semialdehyde
4-Hydroxybutanoate
01200 7/19/17
(c) Kanehisa Laboratories

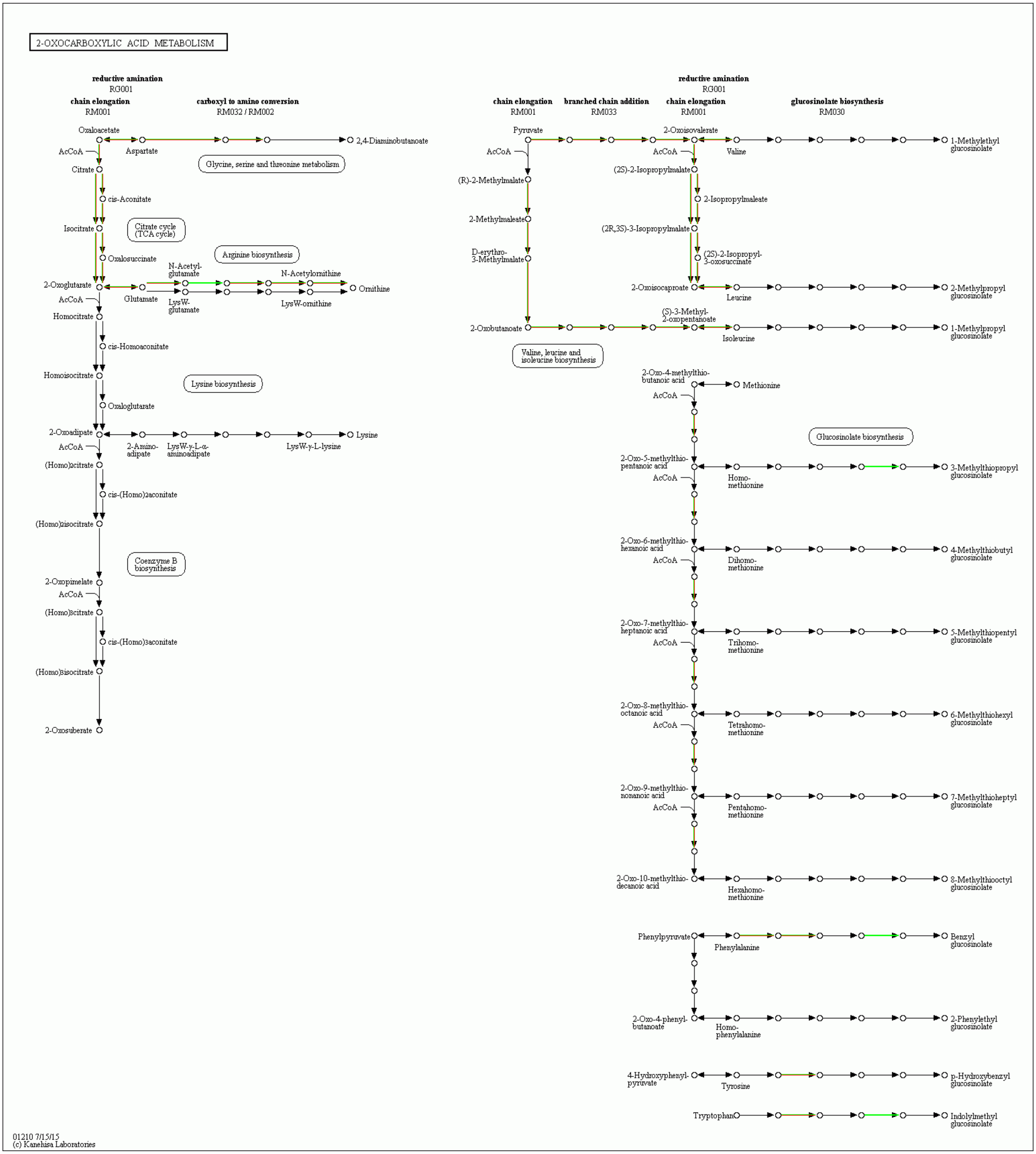
2-OXOCARBOXYLIC ACID METABOLISM
reductive amination
RG001
chain elongation
RM001
carboxyl to amino conversion
RM032 / RM002
Oxaloacetate
Aspartate
2,4-Diaminobutanoate
AcCoA
Citrate
Glycine, serine and threonine metabolism
cis-Aconitate
Isocitrate
Citrate cycle (TCA cycle)
Oxalosuccinate
Arginine biosynthesis
N-Acetyl-glutamate
N-Acetylornithine
2-Oxoglutarate
Glutamate
Ornithine
LysW-glutamate
LysW-ornithine
Homocitrate
cis-Homoaconitate
Homoisocitrate
Lysine biosynthesis
Oxaloglutarate
2-Oxoadipate
Lysine
2-Amino-adipate
LysW-γ-L-α-aminoadipate
LysW-γ-L-lysine
(Homo)2citrate
cis-(Homo)2aconitate
(Homo)2isocitrate
Coenzyme B biosynthesis
2-Oxopimelate
(Homo)3citrate
cis-(Homo)3aconitate
(Homo)3isocitrate
2-Oxosuberate
chain elongation
RM001
branched chain addition
RM033
chain elongation
RM001
glucosinolate biosynthesis
RM030
Pyruvate
2-Oxoisovalerate
Valine
1-Methylethyl glucosinolate
(R)-2-Methylmalate
(2S)-2-Isopropylmalate
2-Isopropylmaleate
2-Methylmaleate
(2R,3S)-3-Isopropylmalate
D-erythro-3-Methylmalate
(2S)-2-Isopropyl-3-oxosuccinate
2-Oxoisocaproate
Leucine
2-Methylpropyl glucosinolate
(S)-3-Methyl-2-oxopentanoate
2-Oxobutanoate
Isoleucine
1-Methylpropyl glucosinolate
Valine, leucine and isoleucine biosynthesis
2-Oxo-4-methylthio-butanoic acid
Methionine
Glucosinolate biosynthesis
2-Oxo-5-methylthio-pentanoic acid
Homo-methionine
3-Methylthiopropyl glucosinolate
2-Oxo-6-methylthio-hexanoic acid
Dihomo-methionine
4-Methylthiobutyl glucosinolate
2-Oxo-7-methylthio-heptanoic acid
Trihomo-methionine
5-Methylthiopentyl glucosinolate
2-Oxo-8-methylthio-octanoic acid
Tetrahomo-methionine
6-Methylthiohexyl glucosinolate
2-Oxo-9-methylthio-nonanoic acid
Pentahomo-methionine
7-Methylthioheptyl glucosinolate
2-Oxo-10-methylthio-decanoic acid
Hexahomo-methionine
8-Methylthiooctyl glucosinolate
Phenylpyruvate
Phenylalanine
Benzyl glucosinolate
2-Oxo-4-phenyl-butanoate
Homo-phenylalanine
2-Phenylethyl glucosinolate
4-Hydroxyphenyl-pyruvate
Tyrosine
p-Hydroxybenzyl glucosinolate
Tryptophan
Indolylmethyl glucosinolate
01210 7/15/15
(c) Kanehisa Laboratories

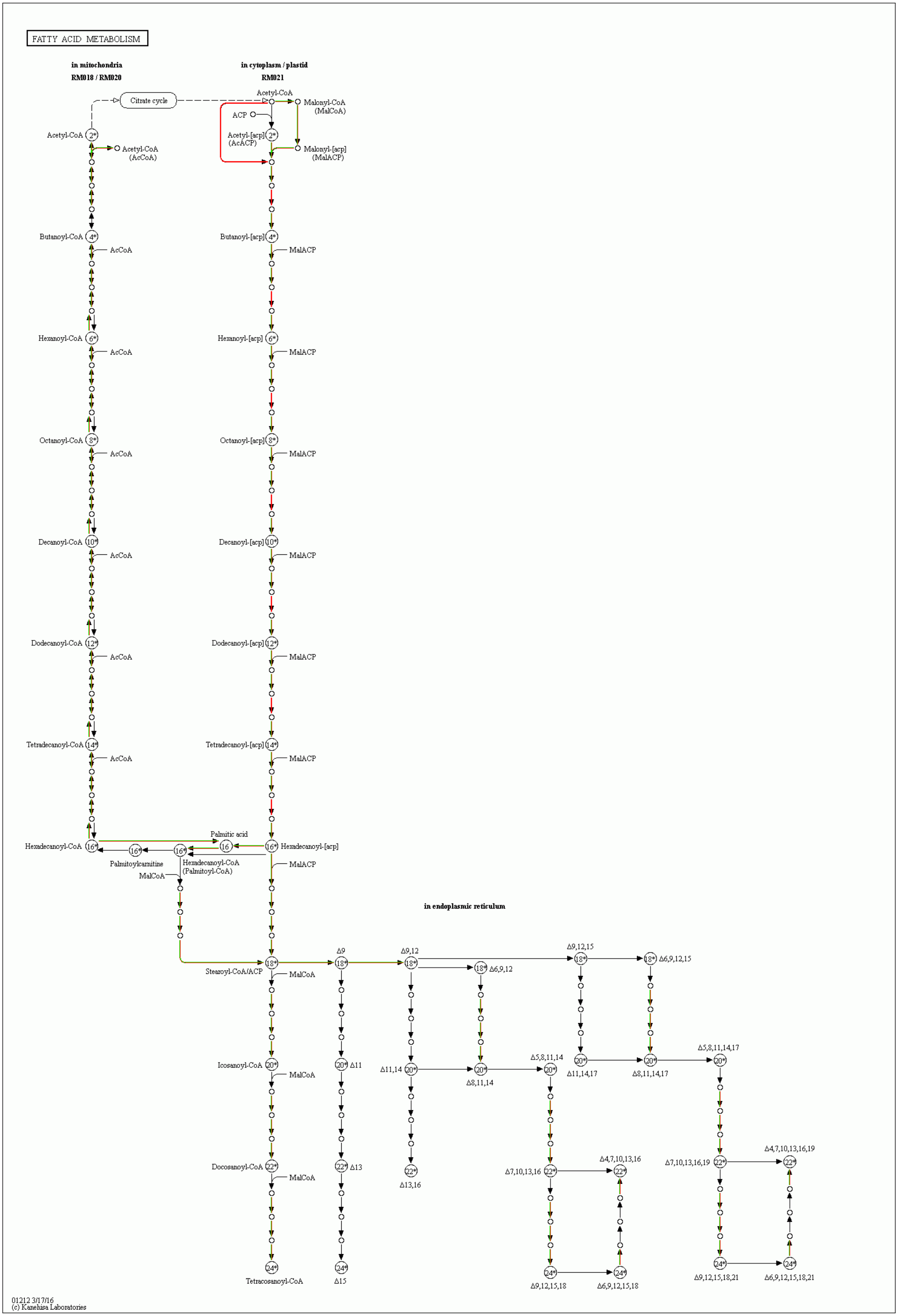
FATTY ACID METABOLISM
in mitochondria
RM018 / RM020
in cytoplasm / plastid
RM021
Citrate cycle
Acetyl-CoA
Malonyl-CoA (MalCoA)
ACP
Acetyl-[acp] (AcACP)
Malonyl-[acp] (MalACP)
Acetyl-CoA (AcCoA)
Butanoyl-CoA
Butanoyl-[acp]
AcCoA
MalACP
Hexanoyl-CoA
Hexanoyl-[acp]
Octanoyl-CoA
Octanoyl-[acp]
Decanoyl-CoA
Decanoyl-[acp]
Dodecanoyl-CoA
Dodecanoyl-[acp]
Tetradecanoyl-CoA
Tetradecanoyl-[acp]
Hexadecanoyl-CoA
Palmitic acid
Hexadecanoyl-[acp]
Palmitoylcarnitine
Hexadecanoyl-CoA (Palmitoyl-CoA)
MalCoA
in endoplasmic reticulum
Stearoyl-CoA/ACP
Icosanoyl-CoA
Docosanoyl-CoA
Tetracosanoyl-CoA
Δ9
Δ9,12
Δ6,9,12
Δ9,12,15
Δ6,9,12,15
Δ11
Δ13
Δ15
Δ11,14
Δ13,16
Δ8,11,14
Δ5,8,11,14
Δ11,14,17
Δ8,11,14,17
Δ5,8,11,14,17
Δ7,10,13,16
Δ4,7,10,13,16
Δ9,12,15,18
Δ6,9,12,15,18
Δ7,10,13,16,19
Δ4,7,10,13,16,19
Δ9,12,15,18,21
Δ6,9,12,15,18,21
01212 3/17/16
(c) Kanehisa Laboratories

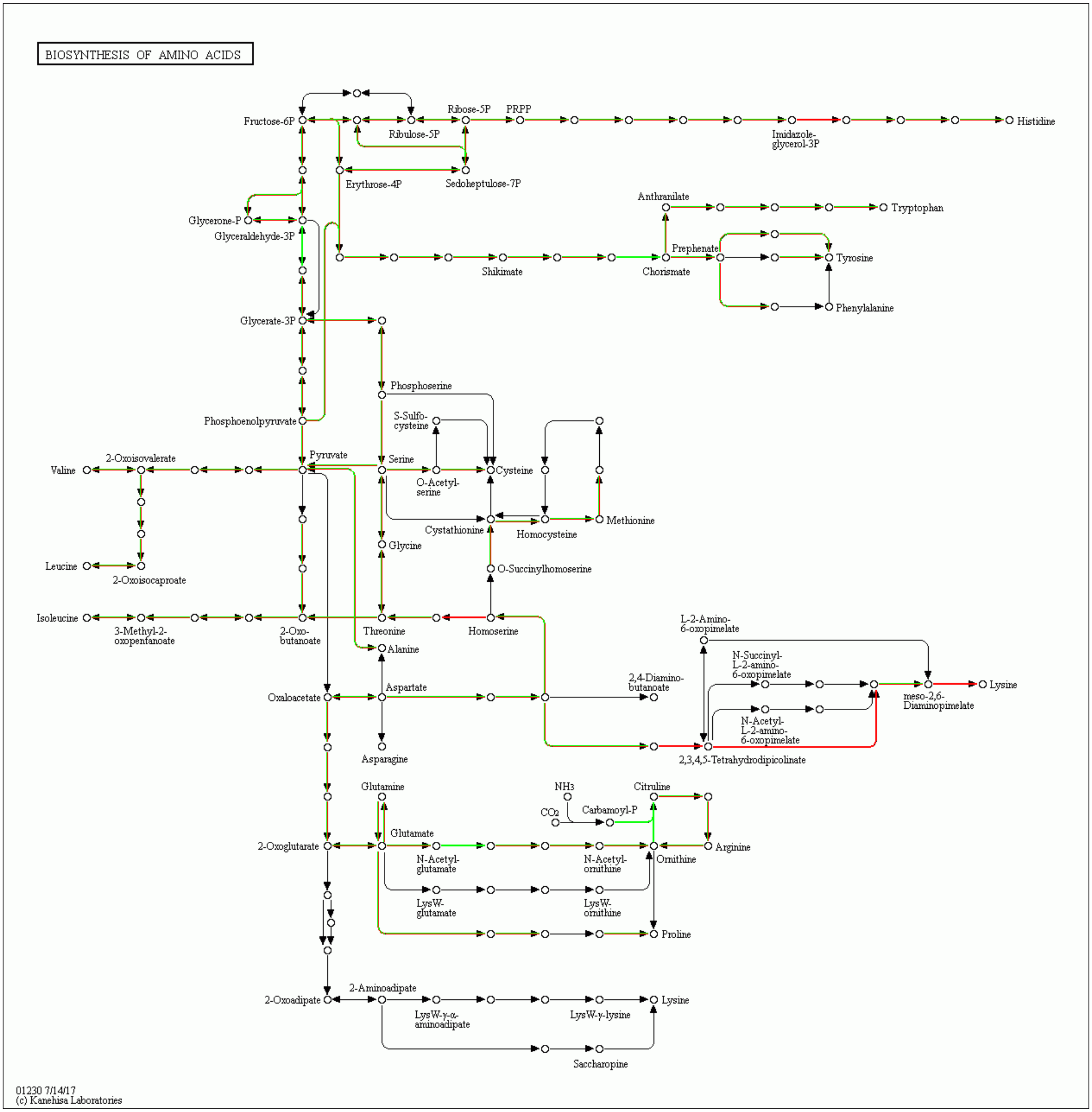
BIOSYNTHESIS OF AMINO ACIDS
Fructose-6P
Ribose-5P
PRPP
Ribulose-5P
Imidazole-glycerol-3P
Histidine
Erythrose-4P
Sedoheptulose-7P
Glycerone-P
Glyceraldehyde-3P
Anthranilate
Tryptophan
Prephenate
Tyrosine
Shikimate
Chorismate
Phenylalanine
Glycerate-3P
Phosphoserine
S-Sulfo-cysteine
Phosphoenolpyruvate
2-Oxoisovalerate
Pyruvate
Serine
Valine
Cysteine
O-Acetyl-serine
Methionine
Cystathionine
Homocysteine
Glycine
Leucine
2-Oxoisocaproate
O-Succinylhomoserine
Isoleucine
3-Methyl-2-oxopentanoate
2-Oxo-butanoate
Threonine
Homoserine
L-2-Amino-6-oxopimelate
Alanine
N-Succinyl-L-2-amino-6-oxopimelate
2,4-Diamino-butanoate
Oxaloacetate
Aspartate
Lysine
meso-2,6-Diaminopimelate
N-Acetyl-L-2-amino-6-oxopimelate
Asparagine
2,3,4,5-Tetrahydrodipicolinate
Glutamine
NH3
Citruline
CO2
Carbamoyl-P
Glutamate
2-Oxoglutarate
Arginine
N-Acetyl-glutamate
N-Acetyl-ornithine
Ornithine
LysW-glutamate
LysW-ornithine
Proline
2-Aminoadipate
2-Oxoadipate
Lysine
LysW-γ-α-aminoadipate
LysW-γ-lysine
Saccharopine
01230 7/14/17
(c) Kanehisa Laboratories

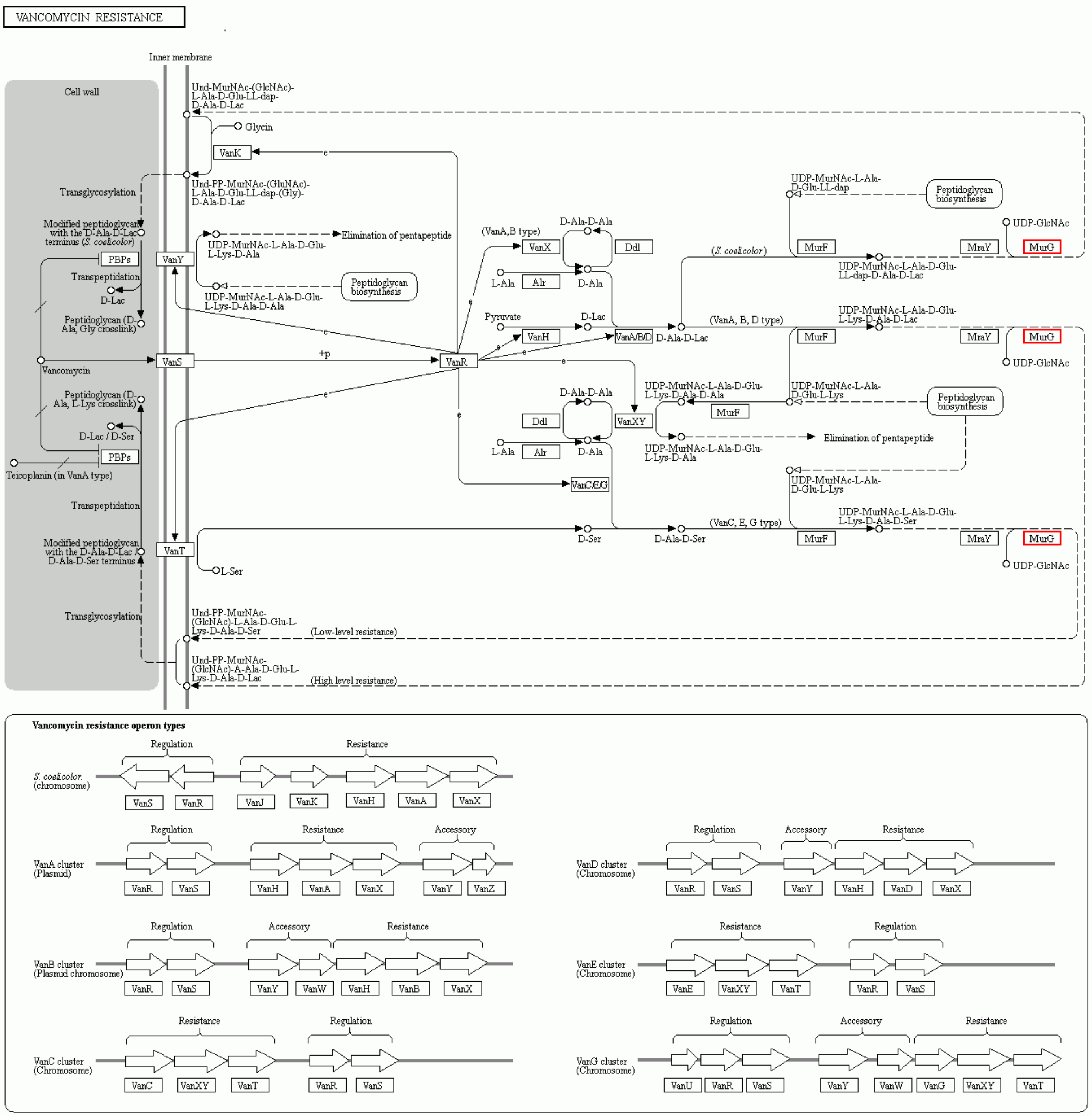

01502 1/19/15
(c) Kanehisa Laboratories

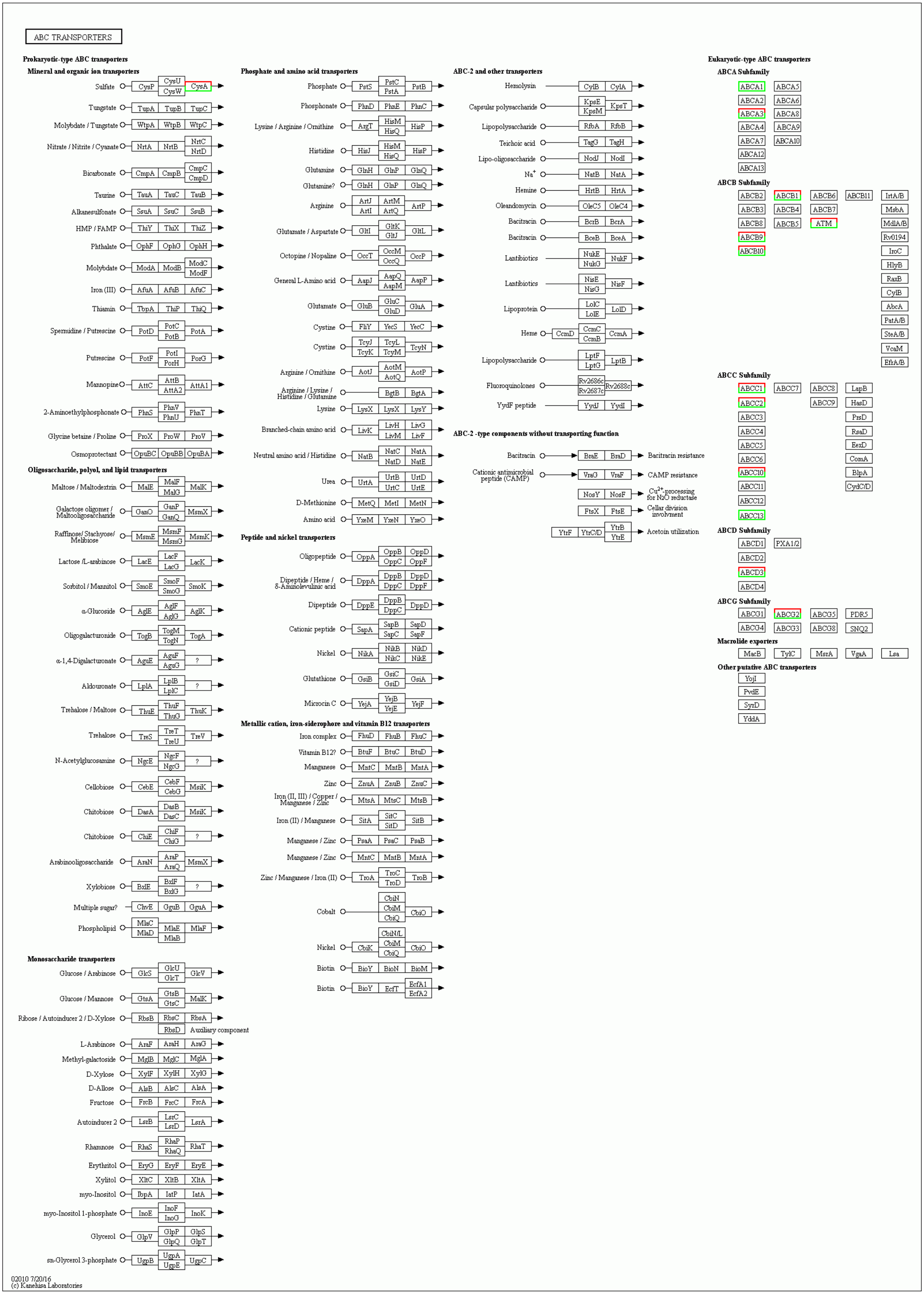
ABC TRANSPORTERS
Prokaryotic-type ABC transporters
Mineral and organic ion transporters
Sulfate CysP CysU CysW CysA
Tungstate TupA TupB TupC
Molybdate / Tungstate WtpA WtpB WtpC
Nitrate / Nitrite / Cyanate NrtA NrtB NrtC NrtD
Bicarbonate CmpA CmpB CmpC CmpD
Taurine TauA TauC TauB
Alkanesulfonate SsuA SsuC SsuB
HMP / FAMP ThiY ThiX ThiZ
Phthalate OphF OphG OphH
Molybdate ModA ModB ModC ModF
Iron (III) AfuA AfuB AfuC
Thiamin TbpA ThiP ThiQ
Spermidine / Putrescine PotD PotC PotB PotA
Putrescine PotF PotI PotH PotG
Mannopine AttC AttB AttA2 AttA1
2-Aminoethylphosphonate PhnS PhnV PhnU PhnT
Glycine betaine / Proline ProX ProW ProV
Osmoprotectant OpuBC OpuBB OpuBA
Oligosaccharide, polyol, and lipid transporters
Maltose / Maltodextrin MalE MalF MalG MalK
Galactose oligomer / Maltooligosaccharide GanO GanP GanQ MsmX
Raffinose/ Stachyose/ Melibiose MsmE MsmF MsmG MsmK
Lactose /L-arabinose LacE LacF LacG LacK
Sorbitol / Mannitol SmoE SmoF SmoG SmoK
α-Glucoside AglE AglF AglG AglK
Oligogalacturonide TogB TogM TogN TogA
α-1,4-Digalacturonate AguE AguF AguG ?
Aldouronate LplA LplB LplC ?
Trehalose / Maltose ThuE ThuF ThuG ThuK
Trehalose TreS TreT TreU TreV
N-Acetylglucosamine NgcE NgcF NgcG ?
Cellobiose CebE CebF CebG MsiK
Chitobiose DasA DasB DasC MsiK
Chitobiose ChiE ChiF ChiG ?
Arabinooligosaccharide AraN AraP AraQ MsmX
Xylobiose BxlE BxlF BxlG ?
Multiple sugar? ChvE GguB GguA
Phospholipid MlaC MlaD MlaE MlaB MlaF
Monosaccharide transporters
Glucose / Arabinose GlcS GlcU GlcT GlcV
Glucose / Mannose GtsA GtsB GtsC MalK
Ribose / Autoinducer 2 / D-Xylose RbsB RbsC RbsA RbsD Auxiliary component
L-Arabinose AraF AraH AraG
Methyl-galactoside MglB MglC MglA
D-Xylose XylF XylH XylG
D-Allose AlsB AlsC AlsA
Fructose FrcB FrcC FrcA
Autoinducer 2 LsrB LsrC LsrD LsrA
Rhamnose RhaS RhaP RhaQ RhaT
Erythritol EryG EryF EryE
Xylitol XltC XltB XltA
myo-Inositol IbpA IatP IatA
myo-Inositol 1-phosphate InoE InoF InoG InoK
Glycerol GlpV GlpP GlpQ GlpS GlpT
sn-Glycerol 3-phosphate UgpB UgpA UgpE UgpC
Phosphate and amino acid transporters
Phosphate PstS PstC PstA PstB
Phosphonate PhnD PhnE PhnC
Lysine / Arginine / Ornithine ArgT HisM HisQ HisP
Histidine HisJ HisM HisQ HisP
Glutamine GlnH GlnP GlnQ
Glutamine? GlnH GlnP GlnQ
Arginine ArtJ ArtI ArtM ArtQ ArtP
Glutamate / Aspartate GltI GltK GltJ GltL
Octopine / Nopaline OccT OccM OccQ OccP
General L-Amino acid AapJ AapQ AapM AapP
Glutamate GluB GluC GluD GluA
Cystine FliY YecS YecC
Cystine TcyJ TcyK TcyL TcyM TcyN
Arginine / Ornithine AotJ AotM AotQ AotP
Arginine / Lysine / Histidine / Glutamine BgtB BgtA
Lysine LysX LysX LysY
Branched-chain amino acid LivK LivH LivM LivG LivF
Neutral amino acid / Histidine NatB NatC NatD NatA NatE
Urea UrtA UrtB UrtC UrtD UrtE
D-Methionine MetQ MetI MetN
Amino acid YxeM YxeN YxeO
Peptide and nickel transporters
Oligopeptide OppA OppB OppC OppD OppF
Dipeptide / Heme / δ-Aminolevulinic acid DppA DppB DppC DppD DppF
Dipeptide DppE DppB DppC DppD
Cationic peptide SapA SapB SapC SapD SapF
Nickel NikA NikB NikC NikD NikE
Glutathione GsiB GsiC GsiD GsiA
Microcin C YejA YejB YejE YejF
Metallic cation, iron-siderophore and vitamin B12 transporters
Iron complex FhuD FhuB FhuC
Vitamin B12? BtuF BtuC BtuD
Manganese MntC MntB MntA
Zinc ZnuA ZnuB ZnuC
Iron (II, III) / Copper / Manganese / Zinc MtsA MtsC MtsB
Iron (II) / Manganese SitA SitC SitD SitB
Manganese / Zinc PsaA PsaC PsaB
Manganese / Zinc MntC MntB MntA
Zinc / Manganese / Iron (II) TroA TroC TroD TroB
Cobalt CbiN CbiM CbiQ CbiO
Nickel CbiK CbiN/L CbiM CbiQ CbiO
Biotin BioY BioN BioM
Biotin BioY EcfT EcfA1 EcfA2
ABC-2 and other transporters
Hemolysin CylB CylA
Capsular polysaccharide KpsE KpsM KpsT
Lipopolysaccharide RfbA RfbB
Teichoic acid TagG TagH
Lipo-oligosaccharide NodJ NodI
Na+ NatB NatA
Hemine HrtB HrtA
Oleandomycin OleC5 OleC4
Bacitracin BcrB BcrA
Bacitracin BceB BceA
Lantibiotics NukE NukG NukF
Lantibiotics NisE NisG NisF
Lipoprotein LolC LolE LolD
Heme CcmD CcmC CcmB CcmA
Lipopolysaccharide LptF LptG LptB
Fluoroquinolones Rv2686c Rv2687c Rv2688c
YydF peptide YydJ YydI
ABC-2 -type components without transporting function
Bacitracin BraE BraD Bacitracin resistance
Cationic antimicrobial peptide (CAMP) VraG VraF CAMP resistance
NosY NosF Cu2+-processing for N2O reductase
FtsX FtsE Cellar division involvment
YtrF YtrC/D YtrB YtrE Acetoin utilization
Eukaryotic-type ABC transporters
ABCA Subfamily
ABCA1 ABCA5
ABCA2 ABCA6
ABCA3 ABCA8
ABCA4 ABCA9
ABCA7 ABCA10
ABCA12
ABCA13
ABCB Subfamily
ABCB2 ABCB1 ABCB6 ABCB11 IrtA/B
ABCB3 ABCB4 ABCB7 MsbA
ABCB8 ABCB5 ATM MdlA/B
ABCB9 Rv0194
ABCB10 IroC
HlyB
RaxB
CylB
AbcA
PatA/B
SteA/B
VcaM
EfrA/B
ABCC Subfamily
ABCC1 ABCC7 ABCC8 LapB
ABCC2 ABCC9 HasD
ABCC3 PrsD
ABCC4 RsaD
ABCC5 EexD
ABCC6 ComA
ABCC10 BlpA
ABCC11 CydC/D
ABCC12
ABCC13
ABCD Subfamily
ABCD1 PXA1/2
ABCD2
ABCD3
ABCD4
ABCG Subfamily
ABCG1 ABCG2 ABCG5 PDR5
ABCG4 ABCG3 ABCG8 SNQ2
Macrolide exporters
MacB TylC MsrA VgaA Lsa
Other putative ABC transporters
YojI
PvdE
SyrD
YddA
02010 7/20/16
(c) Kanehisa Laboratories

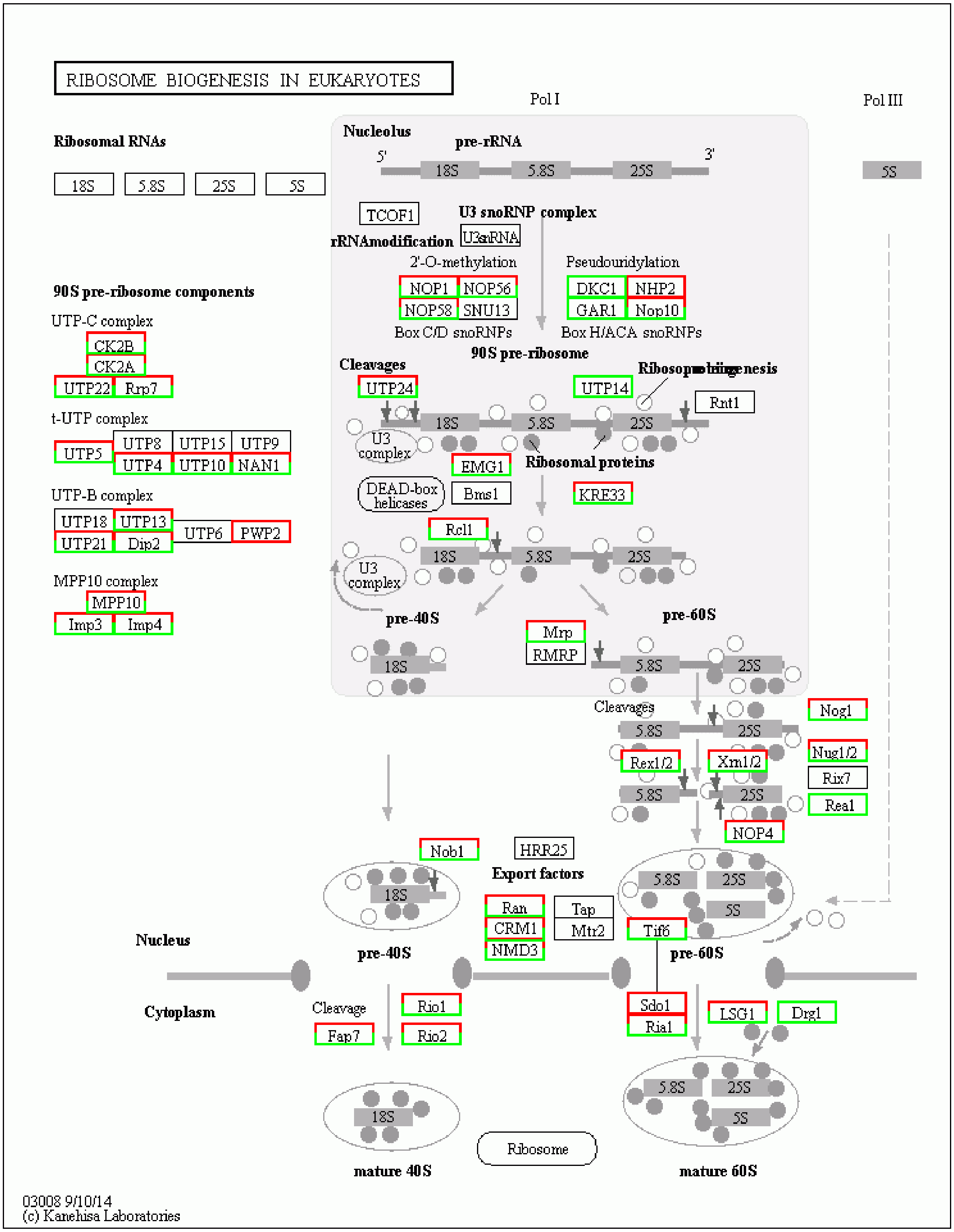
RIBOSOME BIOGENESIS IN EUKARYOTES
Ribosomal RNAs
18S
5.8S
25S
5S
Nucleolus
pre-rRNA
Pol I
Pol III
TCOF1
U3 snoRNP complex
U3snRNA
rRNA modification
2'-O-methylation
Pseudouridylation
NOP1
NOP56
NOP58
SNU13
Box C/D snoRNPs
DKC1
NHP2
GAR1
Nop10
Box H/ACA snoRNPs
90S pre-ribosome components
UTP-C complex
CK2B
CK2A
UTP22
Rrp7
t-UTP complex
UTP5
UTP8
UTP15
UTP9
UTP4
UTP10
NAN1
UTP-B complex
UTP18
UTP13
UTP21
Dip2
UTP6
PWP2
MPP10 complex
MPP10
Imp3
Imp4
Cleavages
UTP24
90S pre-ribosome
UTP14
Rnt1
U3 complex
EMG1
Ribosomal proteins
DEAD-box helicases
Bms1
KRE33
Rcl1
pre-40S
pre-60S
Mrp
RMRP
Nog1
Nug1/2
Rix7
Rea1
Rex1/2
Xrn1/2
NOP4
Nob1
HRR25
Export factors
Ran
CRM1
NMD3
Tap
Mtr2
Tif6
Nucleus
Cytoplasm
Cleavage
Fap7
Rio1
Rio2
Sdo1
Ria1
LSG1
Drg1
mature 40S
Ribosome
mature 60S
03008 9/10/14
(c) Kanehisa Laboratories

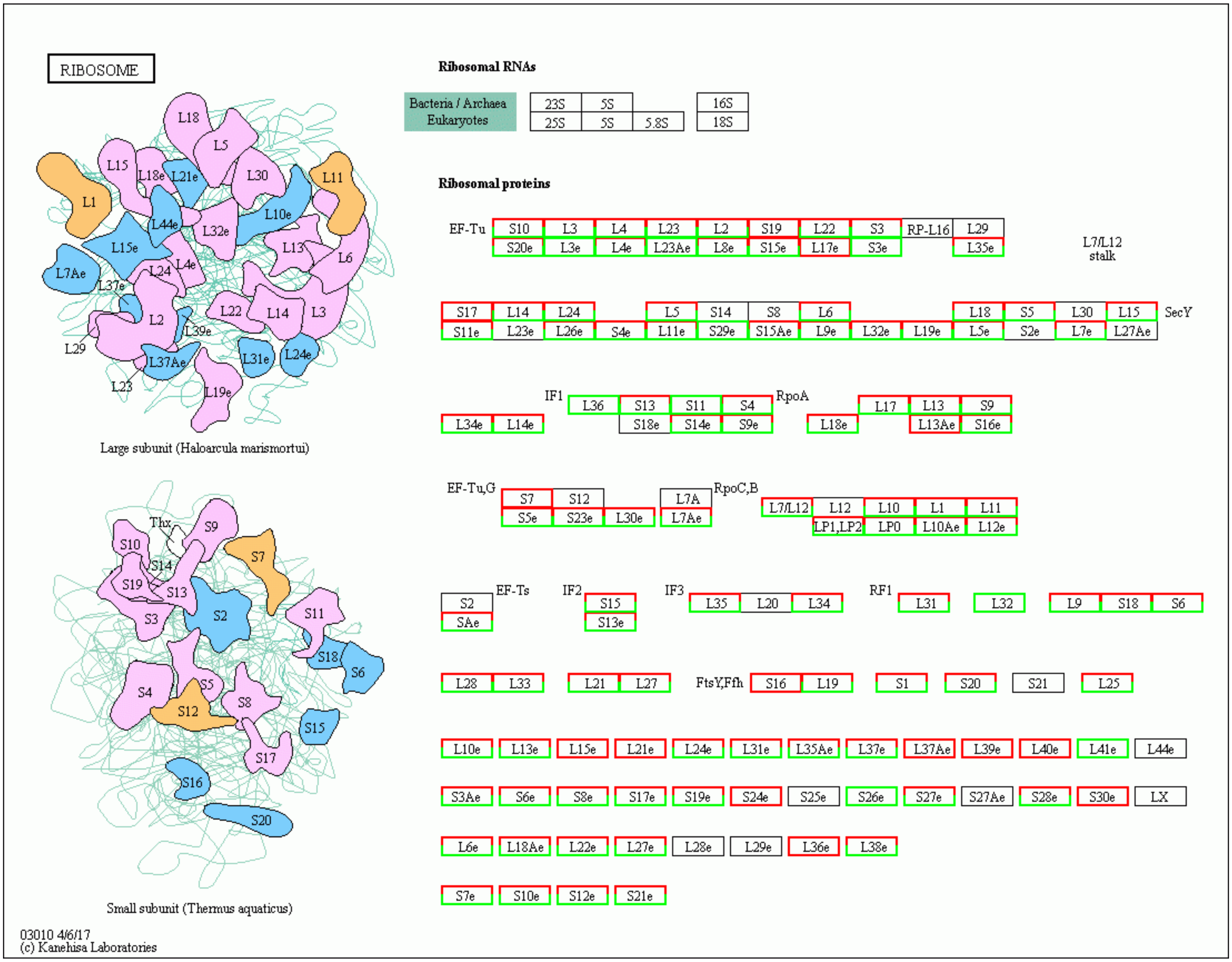
RIBOSOME
Large subunit (Haloarcula marismortui)
L18
L5
L15
L18e
L21e
L30
L11
L1
L44e
L32e
L10e
L15e
L13
L6
L7Ae
L24
L4e
L37e
L2
L22
L14
L3
L39e
L29
L37Ae
L31e
L24e
L23
L19e
Small subunit (Thermus aquaticus)
Thx
S9
S10
S7
S14
S19
S13
S2
S3
S11
S18
S6
S4
S5
S12
S8
S15
S17
S16
S20
Ribosomal RNAs
Bacteria / Archaea
Eukaryotes
23S
5S
16S
25S
5S
5.8S
18S
Ribosomal proteins
EF-Tu
S10 L3 L4 L23 L2 S19 L22 S3 RP-L16 L29
S20e L3e L4e L23Ae L8e S15e L17e S3e L35e
L7/L12
stalk
S17 L14 L24 L5 S14 S8 L6 L18 S5 L30 L15
SecY
S11e L23e L26e S4e L11e S29e S15Ae L9e L32e L19e L5e S2e L7e L27Ae
IF1
L36 S13 S11 S4
RpoA
L17 L13 S9
L34e L14e S18e S14e S9e L18e L13Ae S16e
EF-Tu,G
S7 S12 L7A
RpoC,B
L7/L12 L12 L10 L1 L11
S5e S23e L30e L7Ae LP1,LP2 LP0 L10Ae L12e
EF-Ts
IF2
IF3
RF1
S2 S15 L35 L20 L34 L31 L32 L9 S18 S6
SAe S13e
L28 L33 L21 L27
FtsY,Ffh
S16 L19 S1 S20 S21 L25
L10e L13e L15e L21e L24e L31e L35Ae L37e L37Ae L39e L40e L41e L44e
S3Ae S6e S8e S17e S19e S24e S25e S26e S27e S27Ae S28e S30e LX
L6e L18Ae L22e L27e L28e L29e L36e L38e
S7e S10e S12e S21e
03010 4/6/17
(c) Kanehisa Laboratories

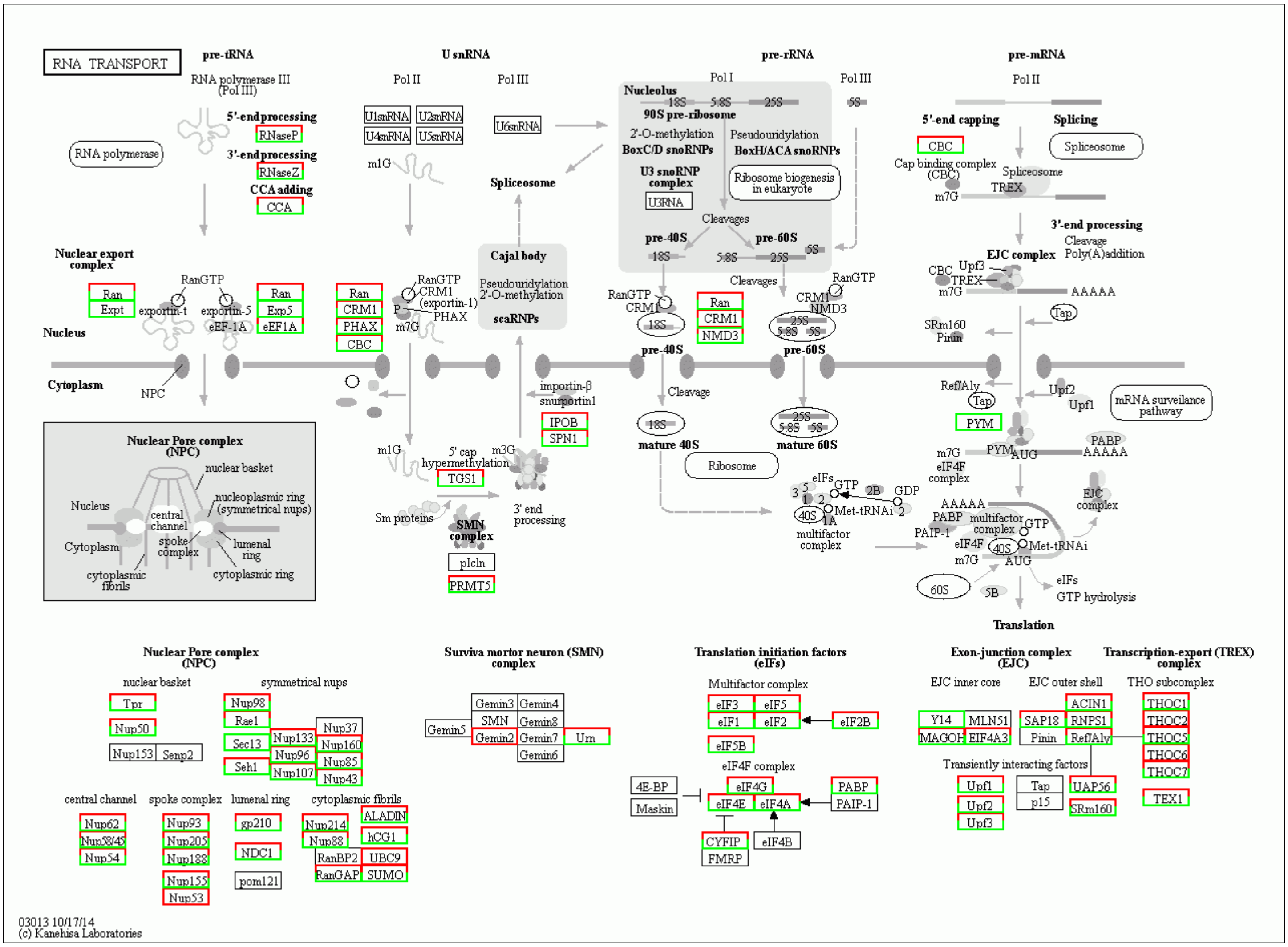
RNA TRANSPORT
pre-tRNA
U snRNA
pre-rRNA
pre-mRNA
Nucleolus
90S pre-ribosome
Cajal body
Spliceosome
Nucleus
Cytoplasm
Nuclear Pore complex (NPC)
Translation
Nuclear Pore complex (NPC)
Surviva mortor neuron (SMN) complex
Translation initiation factors (eIFs)
Exon-junction complex (EJC)
Transcription-export (TREX) complex
nuclear basket
symmetrical nups
Tpr
Nup50
Nup153
Senp2
Nup98
Rae1
Sec13
Seh1
Nup133
Nup96
Nup107
Nup37
Nup160
Nup85
Nup43
central channel
spoke complex
lumenal ring
cytoplasmic fibrils
Nup62
Nup58/45
Nup54
Nup93
Nup205
Nup188
Nup155
Nup53
gp210
NDC1
pom121
Nup214
Nup88
RanBP2
RanGAP
ALADIN
hCG1
UBC9
SUMO
Gemin3
Gemin4
Gemin5
SMN
Gemin8
Gemin2
Gemin7
Urn
Gemin6
Multifactor complex
eIF3
eIF5
eIF1
eIF2
eIF2B
eIF5B
eIF4F complex
4E-BP
Maskin
eIF4G
eIF4E
eIF4A
PABP
PAIP-1
CYFIP
FMRP
eIF4B
EJC inner core
EJC outer shell
THO subcomplex
Y14
MLN51
MAGOH
EIF4A3
SAP18
RNPS1
Pinin
Ref/Aly
ACIN1
THOC1
THOC2
THOC5
THOC6
THOC7
TEX1
Transiently interacting factors
Upf1
Upf2
Upf3
Tap
p15
UAP56
SRm160
03013 10/17/14
(c) Kanehisa Laboratories

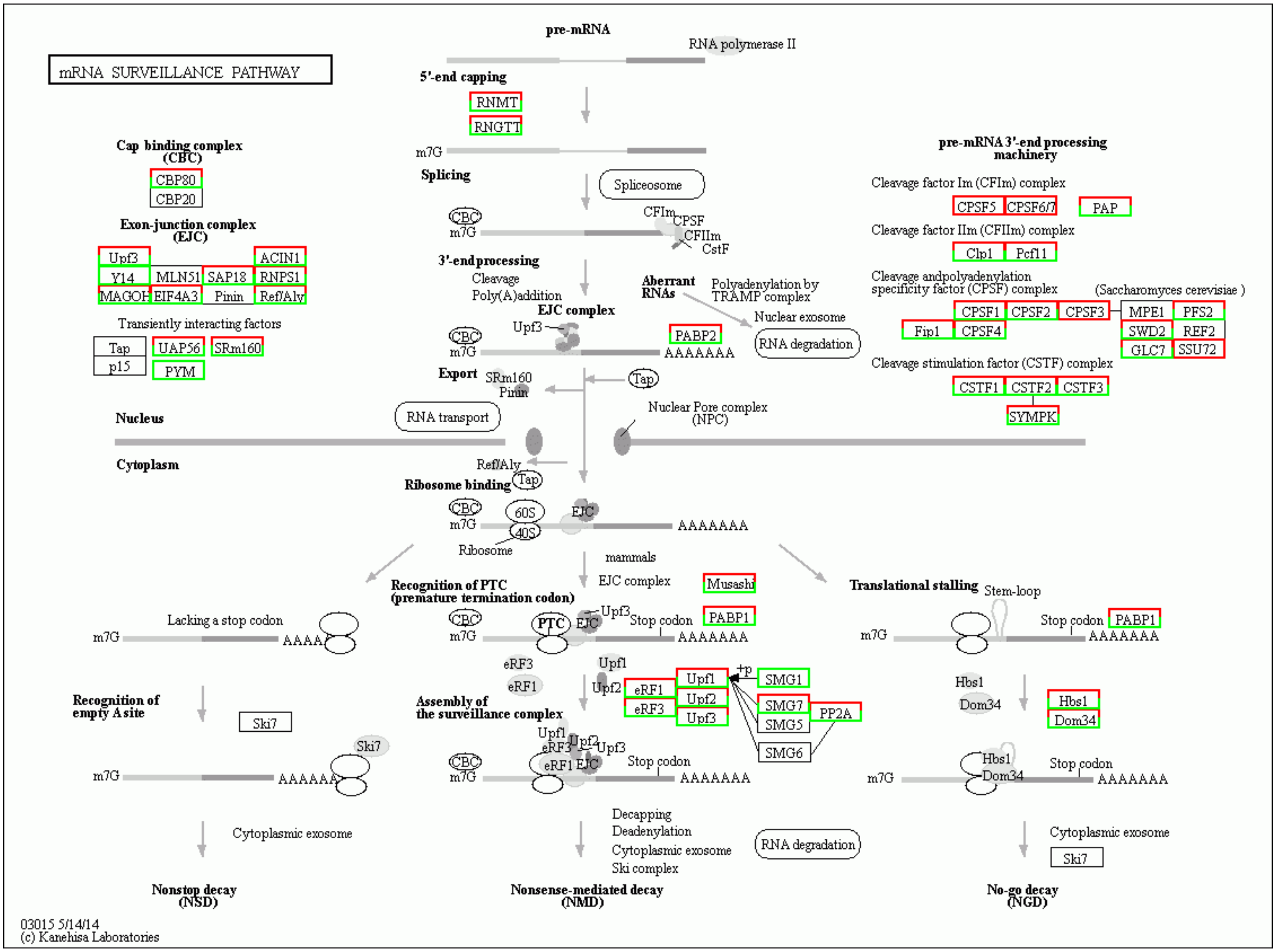
mRNA SURVEILLANCE PATHWAY
pre-mRNA
RNA polymerase II
5'-end capping
RNMT
RNGTT
m7G
Splicing
Spliceosome
Cap binding complex (CBC)
CBP80
CBP20
Exon-junction complex (EJC)
Upf3
ACIN1
Y14
MLN51
SAP18
RNPS1
MAGOH
EIF4A3
Pinin
Ref/Aly
Transiently interacting factors
Tap
UAP56
SRm160
p15
PYM
CBC
CFIm
CPSF
CFIIm
CstF
3'-end processing
Cleavage
Poly(A)addition
EJC complex
Aberrant RNAs
Polyadenylation by TRAMP complex
Nuclear exosome
RNA degradation
PABP2
AAAAAAA
Export
SRm160
Pinin
Tap
RNA transport
Nuclear Pore complex (NPC)
Nucleus
Cytoplasm
Ref/Aly
Ribosome binding
60S
40S
EJC
Ribosome
pre-mRNA 3'-end processing machinery
Cleavage factor Im (CFIm) complex
CPSF5
CPSF6/7
PAP
Cleavage factor IIm (CFIIm) complex
Clp1
Pcf11
Cleavage and polyadenylation specificity factor (CPSF) complex
(Saccharomyces cerevisiae)
CPSF1
CPSF2
CPSF3
MPE1
PFS2
Fip1
CPSF4
SWD2
REF2
GLC7
SSU72
Cleavage stimulation factor (CSTF) complex
CSTF1
CSTF2
CSTF3
SYMPK
mammals
EJC complex
Musashi
Recognition of PTC (premature termination codon)
Translational stalling
Stem-loop
Lacking a stop codon
PTC
Stop codon
PABP1
Recognition of empty A site
Ski7
eRF3
eRF1
Upf1
Upf2
Upf3
+p
SMG1
SMG7
SMG5
PP2A
SMG6
Assembly of the surveillance complex
Hbs1
Dom34
Decapping
Deadenylation
Cytoplasmic exosome
Ski complex
Nonstop decay (NSD)
Nonsense-mediated decay (NMD)
No-go decay (NGD)
03015 5/14/14
(c) Kanehisa Laboratories

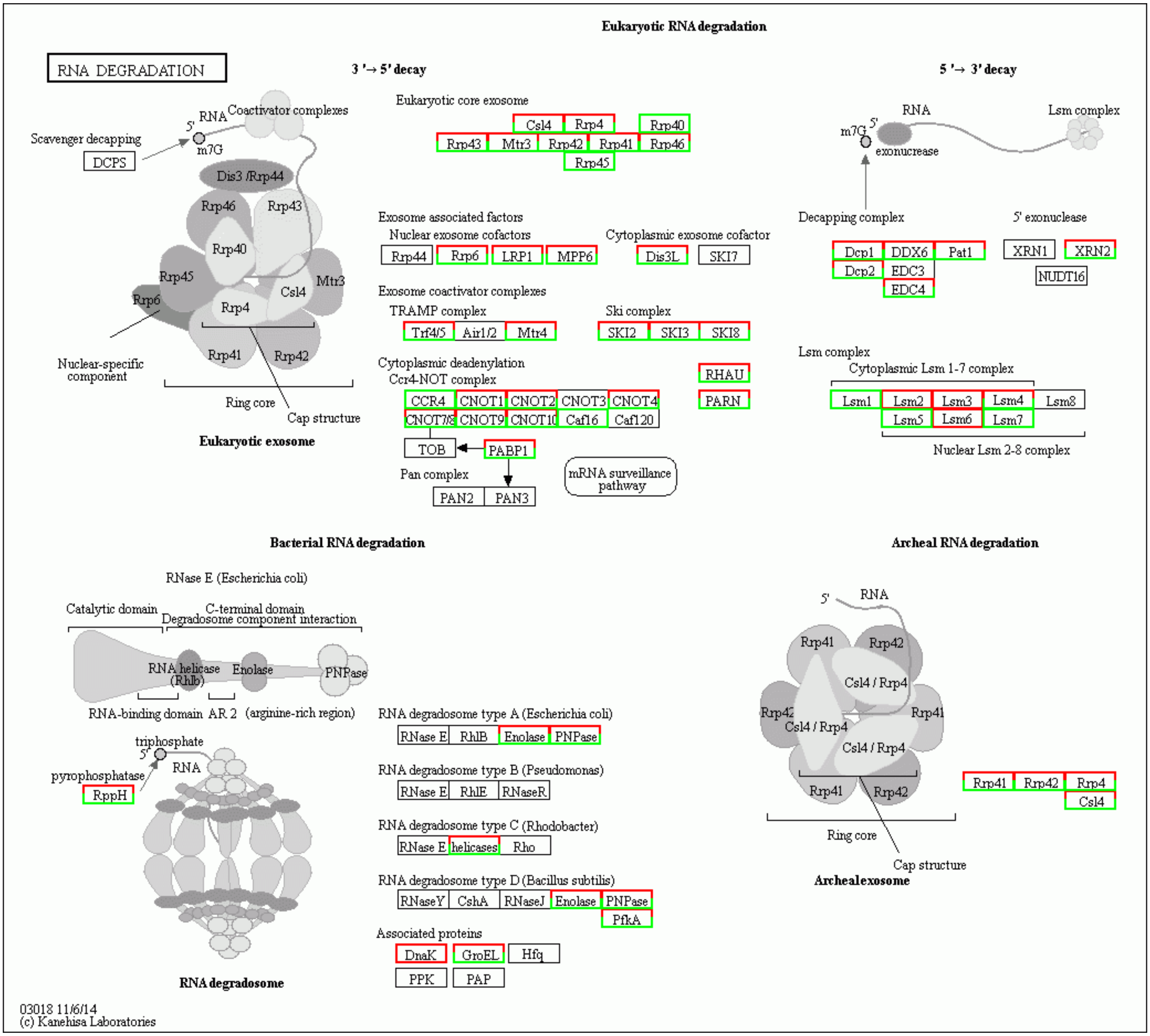
Eukaryotic RNA degradation
RNA DEGRADATION
3 '→ 5' decay
5 '→ 3' decay
Scavenger decapping
DCPS
RNA
Coactivator complexes
5'
m7G
Dis3 /Rrp44
Rrp46
Rrp43
Rrp40
Rrp45
Mtr3
Csl4
Rrp6
Rrp4
Rrp41
Rrp42
Nuclear-specific component
Ring core
Cap structure
Eukaryotic exosome
Eukaryotic core exosome
Csl4
Rrp4
Rrp40
Rrp43
Mtr3
Rrp42
Rrp41
Rrp46
Rrp45
Exosome associated factors
Nuclear exosome cofactors
Rrp44
Rrp6
LRP1
MPP6
Cytoplasmic exosome cofactor
Dis3L
SKI7
Exosome coactivator complexes
TRAMP complex
Trf4/5
Air1/2
Mtr4
Ski complex
SKI2
SKI3
SKI8
Cytoplasmic deadenylation
Ccr4-NOT complex
CCR4
CNOT1
CNOT2
CNOT3
CNOT4
CNOT7/8
CNOT9
CNOT10
Caf16
Caf120
RHAU
PARN
TOB
PABP1
Pan complex
PAN2
PAN3
mRNA surveillance pathway
RNA
Lsm complex
m7G
exonucrease
Decapping complex
5' exonuclease
Dcp1
DDX6
Pat1
Dcp2
EDC3
EDC4
XRN1
XRN2
NUDT16
Lsm complex
Cytoplasmic Lsm 1-7 complex
Lsm1
Lsm2
Lsm3
Lsm4
Lsm8
Lsm5
Lsm6
Lsm7
Nuclear Lsm 2-8 complex
Bacterial RNA degradation
RNase E (Escherichia coli)
Catalytic domain
C-terminal domain
Degradosome component interaction
RNA helicase (Rhlb)
Enolase
PNPase
RNA-binding domain
AR 2
(arginine-rich region)
triphosphate
5'
RNA
pyrophosphatase
RppH
RNA degradosome
RNA degradosome type A (Escherichia coli)
RNase E
RhlB
Enolase
PNPase
RNA degradosome type B (Pseudomonas)
RNase E
RhlE
RNaseR
RNA degradosome type C (Rhodobacter)
RNase E
helicases
Rho
RNA degradosome type D (Bacillus subtilis)
RNaseY
CshA
RNaseJ
Enolase
PNPase
PfkA
Associated proteins
DnaK
GroEL
Hfq
PPK
PAP
Archeal RNA degradation
5'
RNA
Rrp41
Rrp42
Csl4 / Rrp4
Rrp42
Rrp41
Csl4 / Rrp4
Csl4 / Rrp4
Rrp41
Rrp42
Ring core
Cap structure
Archealexosome
Rrp41
Rrp42
Rrp4
Csl4
03018 11/6/14
(c) Kanehisa Laboratories

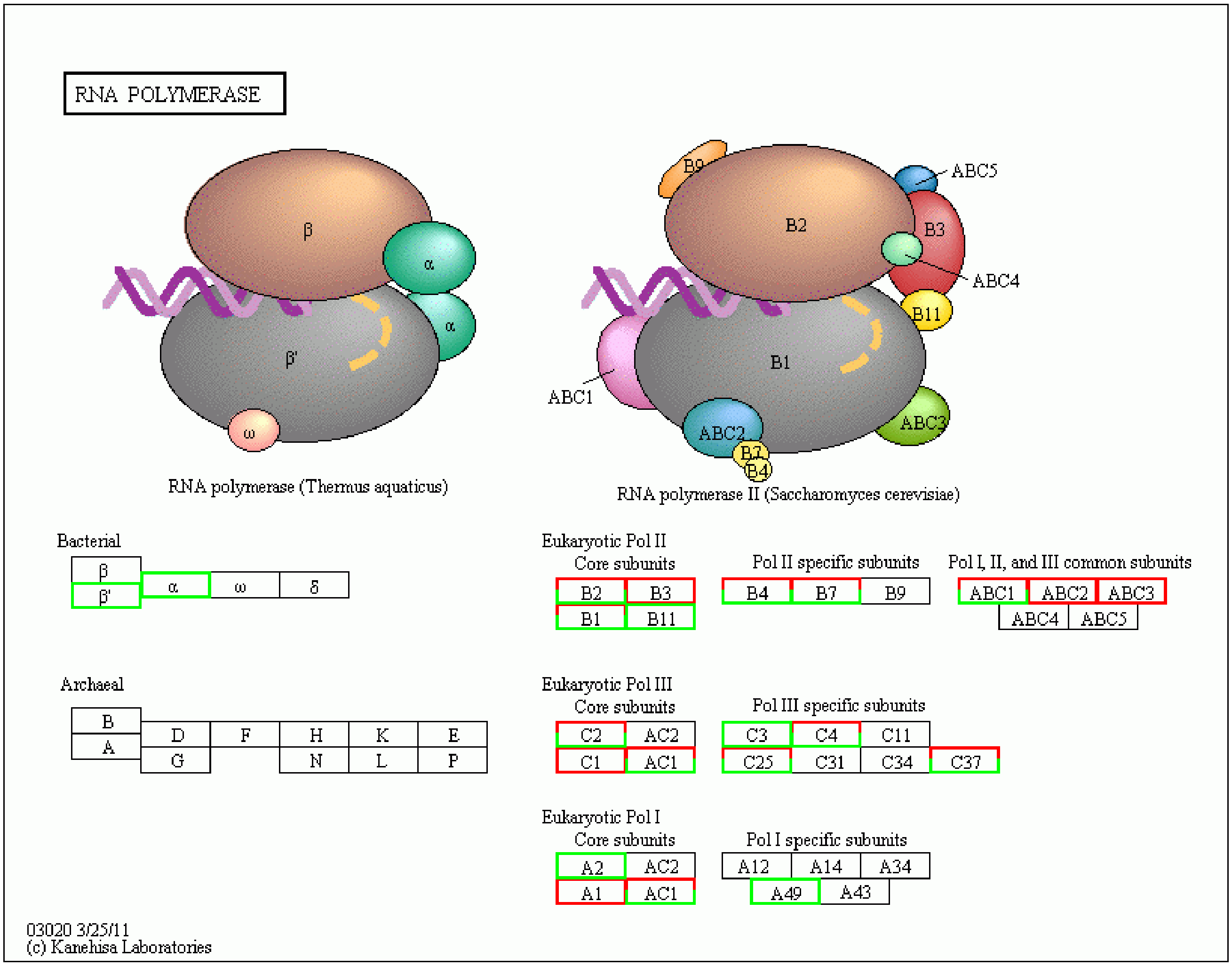
RNA POLYMERASE
β
α
α
β'
ω
RNA polymerase (Thermus aquaticus)
B9
ABC5
B2
B3
ABC4
B11
B1
ABC1
ABC2
ABC3
B7
B4
RNA polymerase II (Saccharomyces cerevisiae)
Bacterial
β
β'
α
ω
δ
Archaeal
B
A
D
G
F
H
N
K
L
E
P
Eukaryotic Pol II
Core subunits
B2
B3
B1
B11
Pol II specific subunits
B4
B7
B9
Pol I, II, and III common subunits
ABC1
ABC2
ABC3
ABC4
ABC5
Eukaryotic Pol III
Core subunits
C2
AC2
C1
AC1
Pol III specific subunits
C3
C4
C11
C25
C31
C34
C37
Eukaryotic Pol I
Core subunits
A2
AC2
A1
AC1
Pol I specific subunits
A12
A14
A34
A49
A43
03020 3/25/11
(c) Kanehisa Laboratories

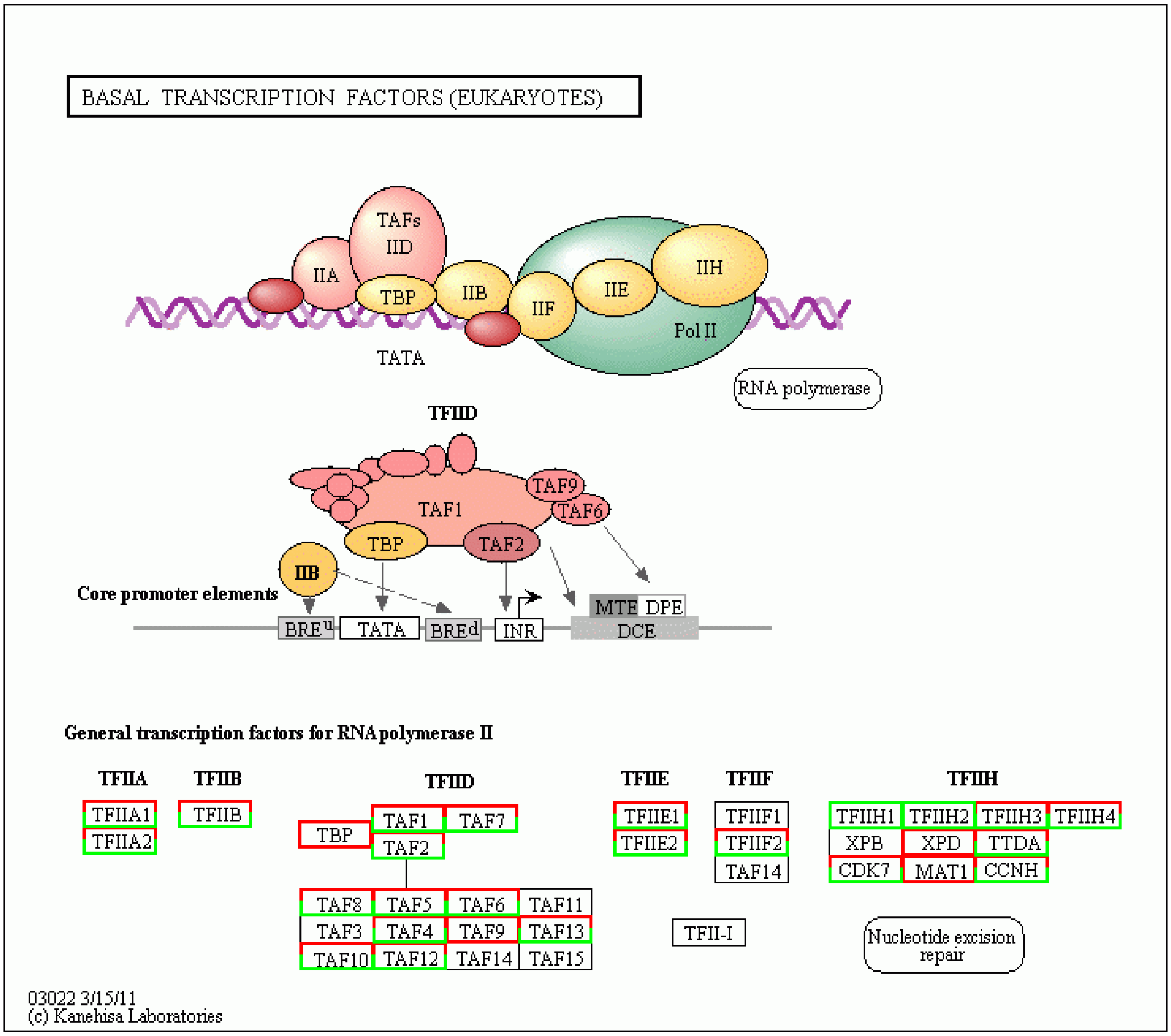

BASAL TRANSCRIPTION FACTORS (EUKARYOTES)
TAFs
IID
IIA
TBP
IIB
IIF
IIE
IIH
Pol II
TATA
RNA polymerase
TFIID
TAF9
TAF6
TAF1
TBP
TAF2
IIB
Core promoter elements
BRE
TATA
BRE
INR
MTE
DPE
DCE
General transcription factors for RNA polymerase II
TFIIA
TFIIB
TFIID
TFIIE
TFIIF
TFIIH
TFIIA1
TFIIA2
TFIIB
TBP
TAF1
TAF7
TAF2
TAF8
TAF5
TAF6
TAF11
TAF3
TAF4
TAF9
TAF13
TAF10
TAF12
TAF14
TAF15
TFIIE1
TFIIE2
TFIIF1
TFIIF2
TAF14
TFII-I
TFIIH1
TFIIH2
TFIIH3
TFIIH4
XPB
XPD
TTDA
CDK7
MAT1
CCNH
Nucleotide excision repair
03022 3/15/11
(c) Kanehisa Laboratories

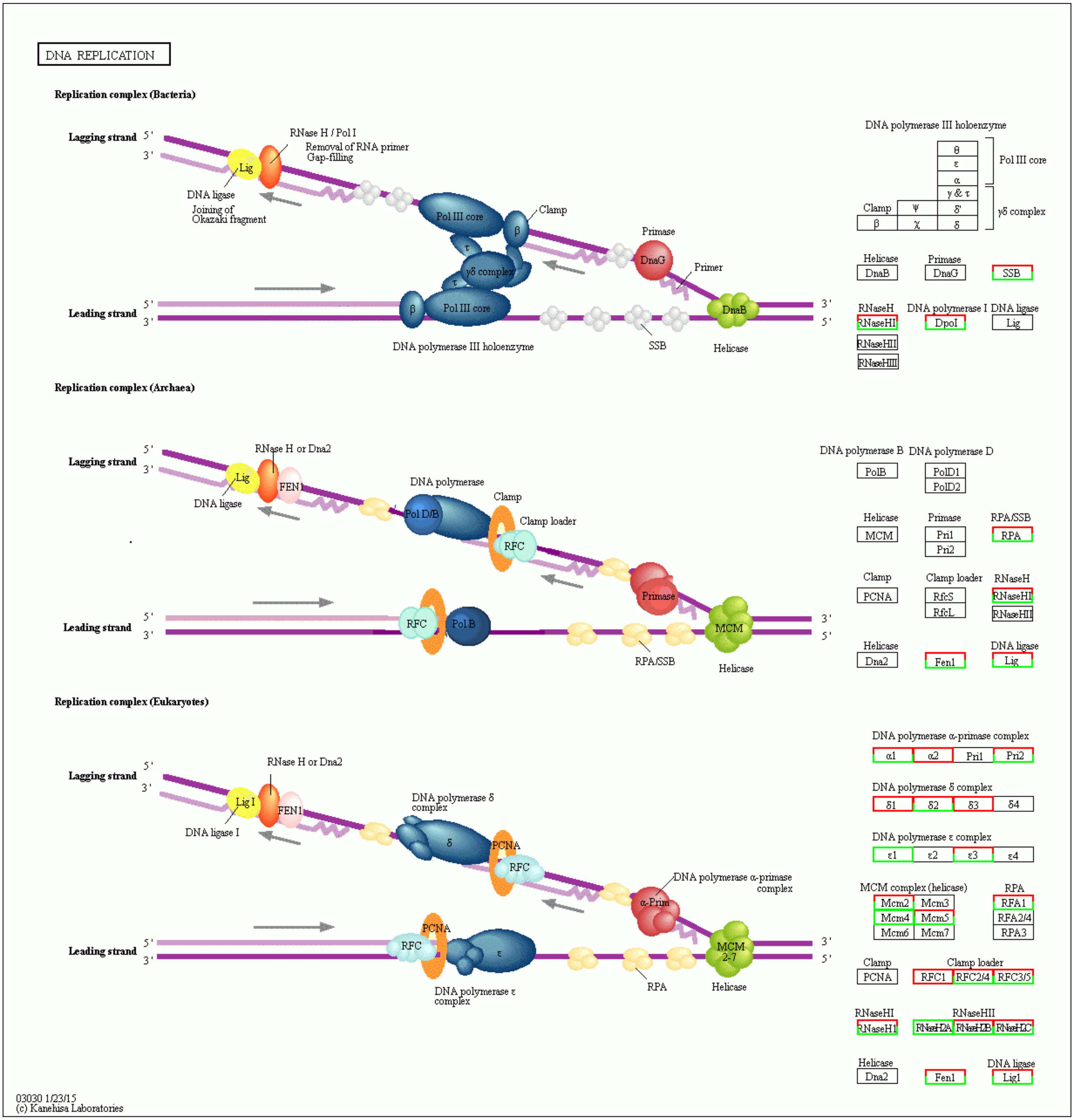
DNA REPLICATION
Replication complex (Bacteria)
Lagging strand
Leading strand
RNase H / Pol I
Removal of RNA primer
Gap-filling
DNA ligase
Joining of Okazaki fragment
Lig
Pol III core
Clamp
β
τ
γδ complex
Primase
DnaG
Primer
DnaB
DNA polymerase III holoenzyme
SSB
Helicase
DNA polymerase III holoenzyme
θ
ε
α
γ & τ
Pol III core
Clamp
ψ
δ'
β
χ
δ
γδ complex
Helicase
DnaB
Primase
DnaG
SSB
RNaseH
RNaseHI
RNaseHII
RNaseHIII
DNA polymerase I
DpoI
DNA ligase
Lig
Replication complex (Archaea)
RNase H or Dna2
FEN1
DNA polymerase
Pol D/B
Clamp loader
RFC
Pol B
MCM
RPA/SSB
DNA polymerase B
PolB
DNA polymerase D
PolD1
PolD2
MCM
Primase
Pri1
Pri2
RPA/SSB
RPA
Clamp
PCNA
Clamp loader
RfcS
RfcL
RNaseH
RNaseHI
RNaseHII
Helicase
Dna2
Fen1
DNA ligase
Lig
Replication complex (Eukaryotes)
Lig I
DNA ligase I
DNA polymerase δ complex
δ
PCNA
DNA polymerase α-primase complex
α-Prim
ε
DNA polymerase ε complex
MCM 2-7
RPA
α1
α2
Pri1
Pri2
DNA polymerase δ complex
δ1
δ2
δ3
δ4
DNA polymerase ε complex
ε1
ε2
ε3
ε4
MCM complex (helicase)
Mcm2
Mcm3
Mcm4
Mcm5
Mcm6
Mcm7
RFA1
RFA2/4
RFA3
RFC1
RFC2/4
RFC3/5
RNaseHI
RNaseH1
RNaseHII
RNaseH2A
RNaseH2B
RNaseH2C
Lig1
03030 1/23/15
(c) Kanehisa Laboratories

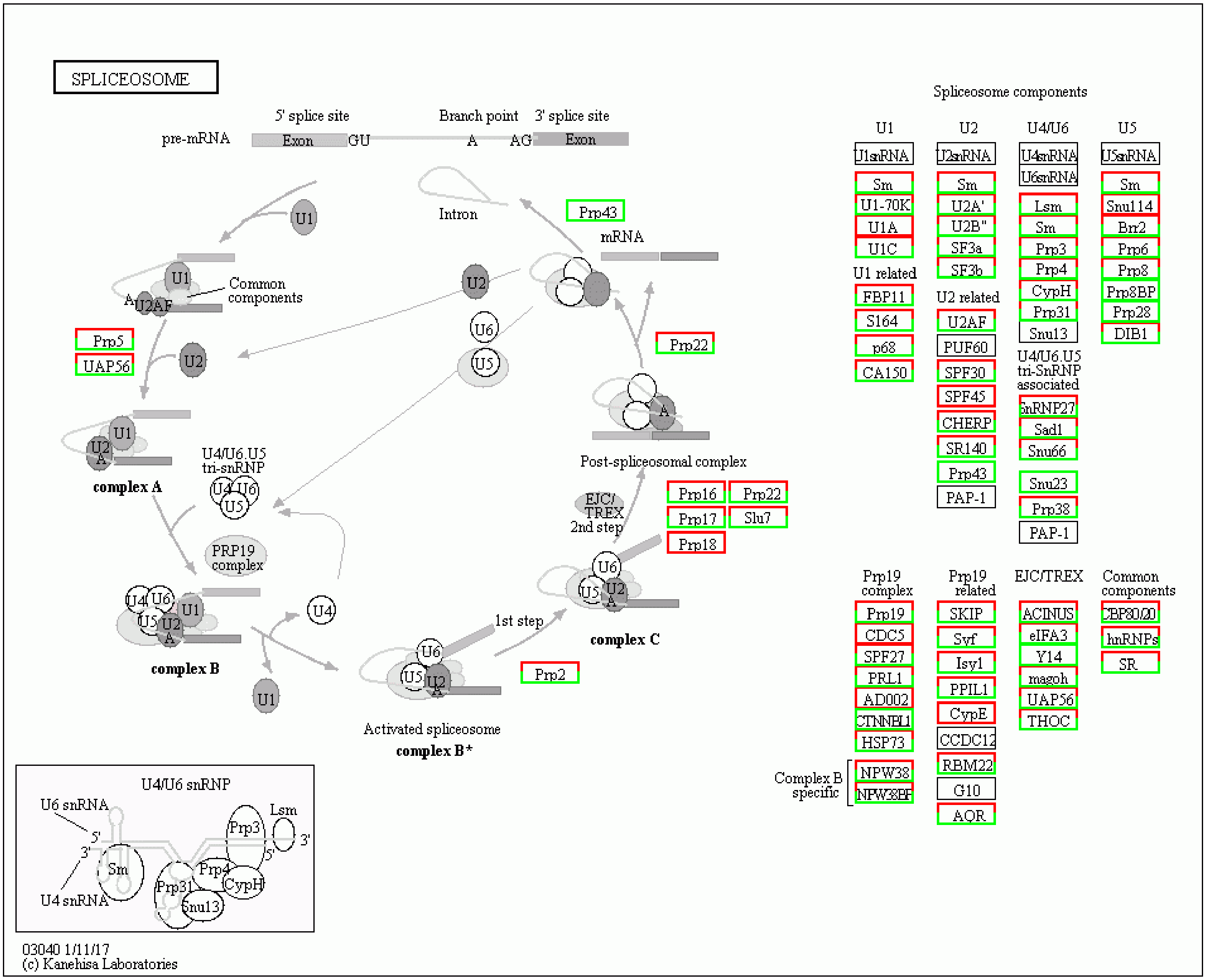
SPLICEOSOME
pre-mRNA
5' splice site
Branch point
3' splice site
Exon
GU
A
AG
Exon
Intron
Prp43
mRNA
U1
U2AF
Common components
Prp5
UAP56
U2
U6
U5
Prp22
complex A
U4/U6.U5 tri-snRNP
PRP19 complex
Post-spliceosomal complex
EJC/TREX
2nd step
Prp16
Prp22
Prp17
Slu7
Prp18
complex C
1st step
complex B
Prp2
Activated spliceosome
complex B*
U4/U6 snRNP
U6 snRNA
U4 snRNA
Sm
Lsm
Prp3
Prp4
Prp31
CypH
Snu13
Spliceosome components
U1
U1snRNA
Sm
U1-70K
U1A
U1C
U1 related
FBP11
S164
p68
CA150
U2
U2snRNA
Sm
U2A'
U2B"
SF3a
SF3b
U2 related
U2AF
PUF60
SPF30
SPF45
CHERP
SR140
Prp43
PAP-1
U4/U6
U4snRNA
U6snRNA
Lsm
Sm
Prp3
Prp4
CypH
Prp31
Snu13
U4/U6.U5 tri-SnRNP associated
snRNP27
Sad1
Snu66
Snu23
Prp38
PAP-1
U5
U5snRNA
Sm
Snu114
Brr2
Prp6
Prp8
Prp8BP
Prp28
DIB1
Prp19 complex
Prp19
CDC5
SPF27
PRL1
AD002
CTNNBL1
HSP73
Complex B specific
NPW38
NPW38BP
Prp19 related
SKIP
Syf
Isy1
PPIL1
CypE
CCDC12
RBM22
G10
AQR
EJC/TREX
ACINUS
eIFA3
Y14
magoh
UAP56
THOC
Common components
CBP80/20
hnRNPs
SR
03040 1/11/17
(c) Kanehisa Laboratories

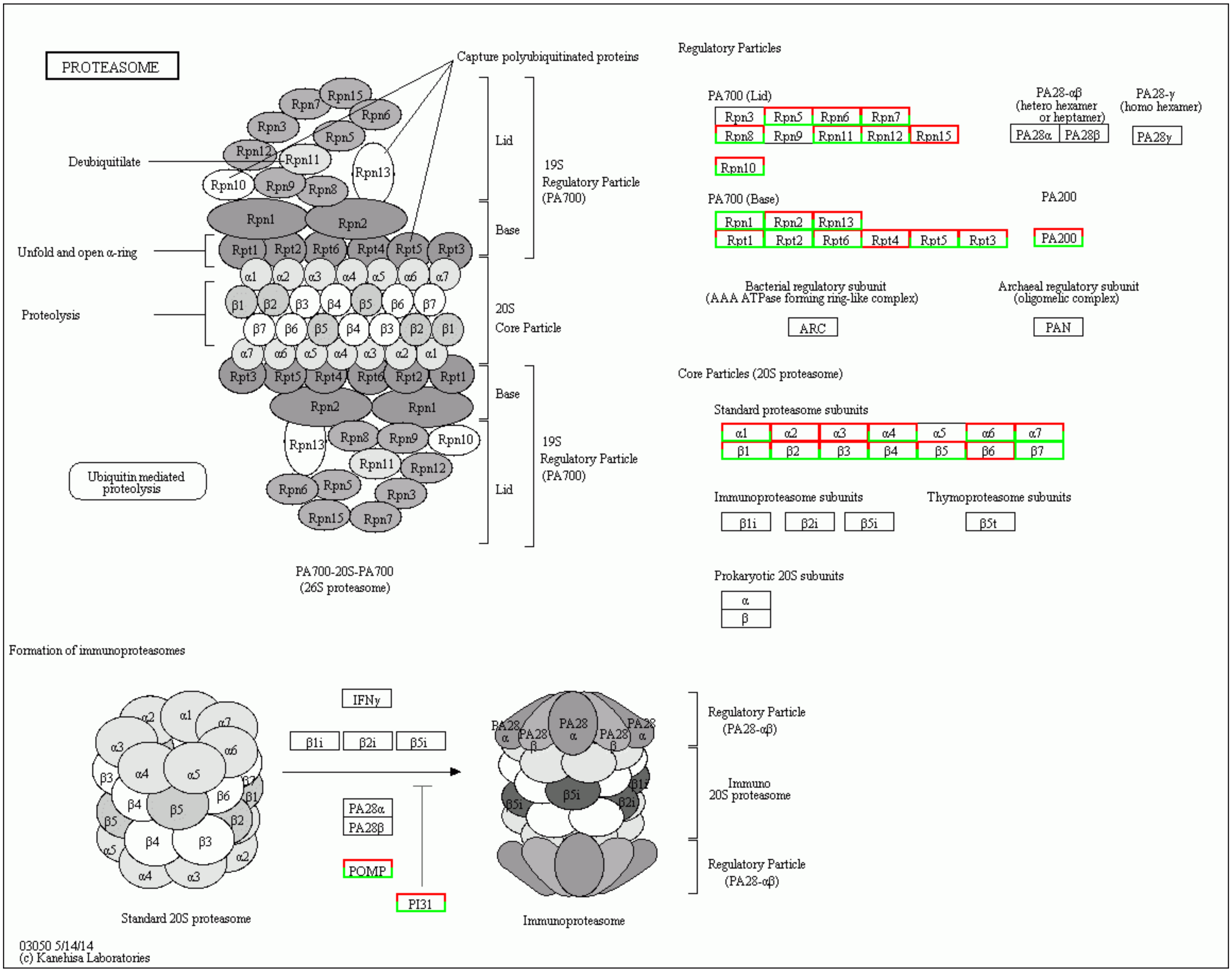
PROTEASOME
Capture polyubiquitinated proteins
Deubiquitilate
Unfold and open α-ring
Proteolysis
Ubiquitin mediated proteolysis
Lid
Base
19S Regulatory Particle (PA700)
20S Core Particle
PA700-20S-PA700 (26S proteasome)
Regulatory Particles
PA700 (Lid)
Rpn3 Rpn5 Rpn6 Rpn7
Rpn8 Rpn9 Rpn11 Rpn12 Rpn15
Rpn10
PA700 (Base)
Rpn1 Rpn2 Rpn13
Rpt1 Rpt2 Rpt6 Rpt4 Rpt5 Rpt3
PA28-αβ (hetero hexamer or heptamer)
PA28α PA28β
PA28-γ (homo hexamer)
PA28γ
PA200
Bacterial regulatory subunit (AAA ATPase forming ring-like complex)
ARC
Archaeal regulatory subunit (oligomelic complex)
PAN
Core Particles (20S proteasome)
Standard proteasome subunits
α1 α2 α3 α4 α5 α6 α7
β1 β2 β3 β4 β5 β6 β7
Immunoproteasome subunits
β1i β2i β5i
Thymoproteasome subunits
β5t
Prokaryotic 20S subunits
α
β
Formation of immunoproteasomes
IFNγ
β1i β2i β5i
PA28α
PA28β
POMP
PI31
Standard 20S proteasome
Immunoproteasome
Regulatory Particle (PA28-αβ)
Immuno 20S proteasome
Regulatory Particle (PA28-αβ)
03050 5/14/14
(c) Kanehisa Laboratories

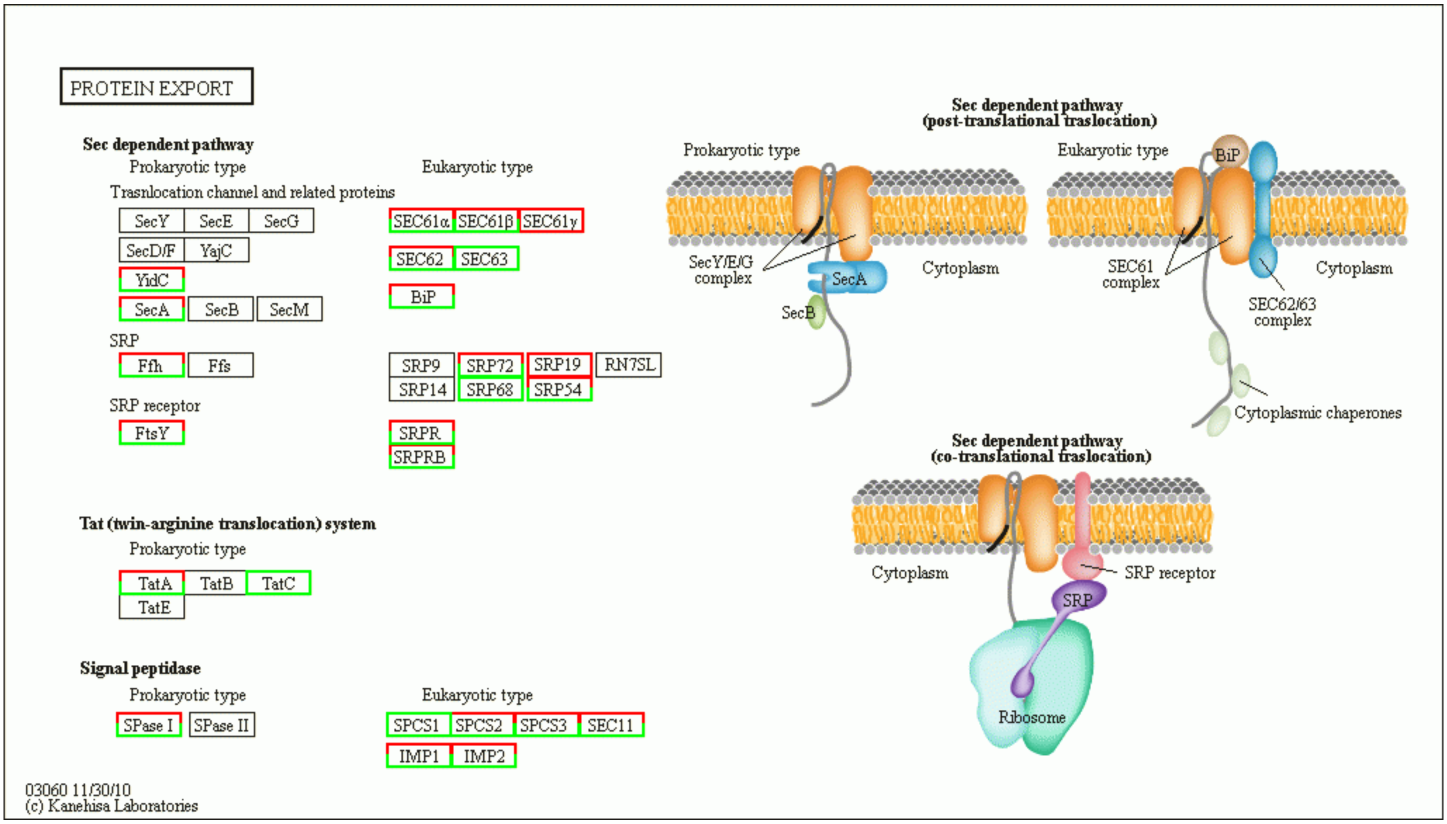

PROTEIN EXPORT
Sec dependent pathway
Prokaryotic type
Eukaryotic type
Trasnlocation channel and related proteins
SecY
SecE
SecG
SecD/F
YajC
YidC
SecA
SecB
SecM
SEC61α
SEC61β
SEC61γ
SEC62
SEC63
BiP
SRP
Ffh
Ffs
SRP9
SRP72
SRP19
RN7SL
SRP14
SRP68
SRP54
SRP receptor
FtsY
SRPR
SRPRB
Tat (twin-arginine translocation) system
Prokaryotic type
TatA
TatB
TatC
TatE
Signal peptidase
Prokaryotic type
Eukaryotic type
SPase I
SPase II
SPCS1
SPCS2
SPCS3
SEC11
IMP1
IMP2
03060 11/30/10
(c) Kanehisa Laboratories
Sec dependent pathway
(post-translational traslocation)
Prokaryotic type
Eukaryotic type
BiP
SecY/E/G
complex
SecA
SecB
Cytoplasm
SEC61
complex
SEC62/63
complex
Cytoplasm
Cytoplasmic chaperones
Sec dependent pathway
(co-translational traslocation)
Cytoplasm
SRP receptor
SRP
Ribosome

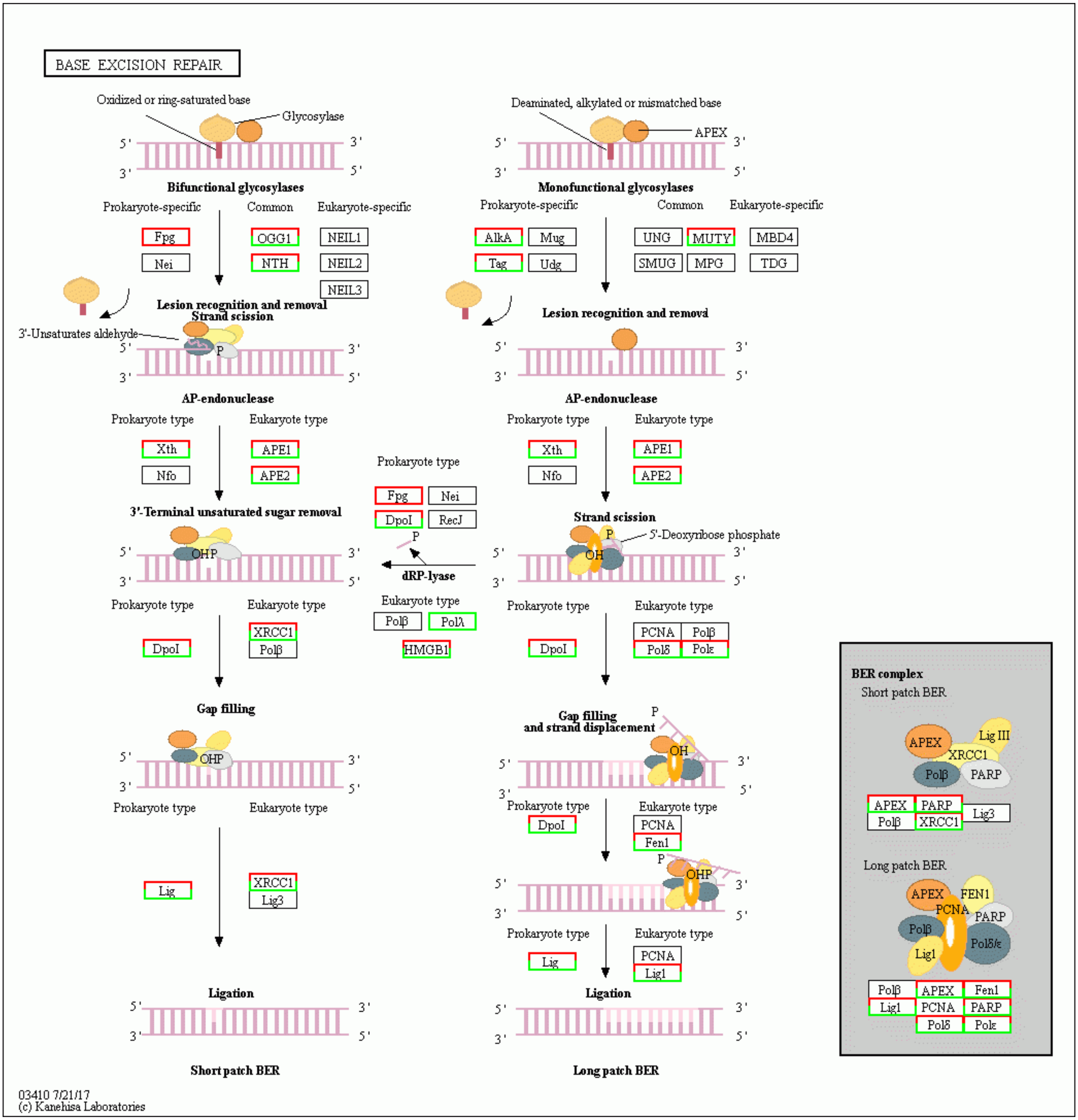
BASE EXCISION REPAIR
Oxidized or ring-saturated base
Glycosylase
Deaminated, alkylated or mismatched base
APEX
Bifunctional glycosylases
Prokaryote-specific
Common
Eukaryote-specific
Fpg
Nei
OGG1
NTH
NEIL1
NEIL2
NEIL3
Monofunctional glycosylases
AlkA
Mug
Tag
Udg
UNG
MUTY
SMUG
MPG
MBD4
TDG
Lesion recognition and removal
Strand scission
3'-Unsaturates aldehyde
AP-endonuclease
Prokaryote type
Eukaryote type
Xth
Nfo
APE1
APE2
3'-Terminal unsaturated sugar removal
Strand scission
5'-Deoxyribose phosphate
dRP-lyase
Nei
DpoI
RecJ
Polβ
Polλ
HMGB1
XRCC1
PCNA
Polδ
Polε
Gap filling
Gap filling
and strand displacement
Fen1
Ligation
Lig
Lig3
Lig1
Short patch BER
Long patch BER
BER complex
APEX
Lig III
XRCC1
PARP
FEN1
Polδ/ε
03410 7/21/17
(c) Kanehisa Laboratories

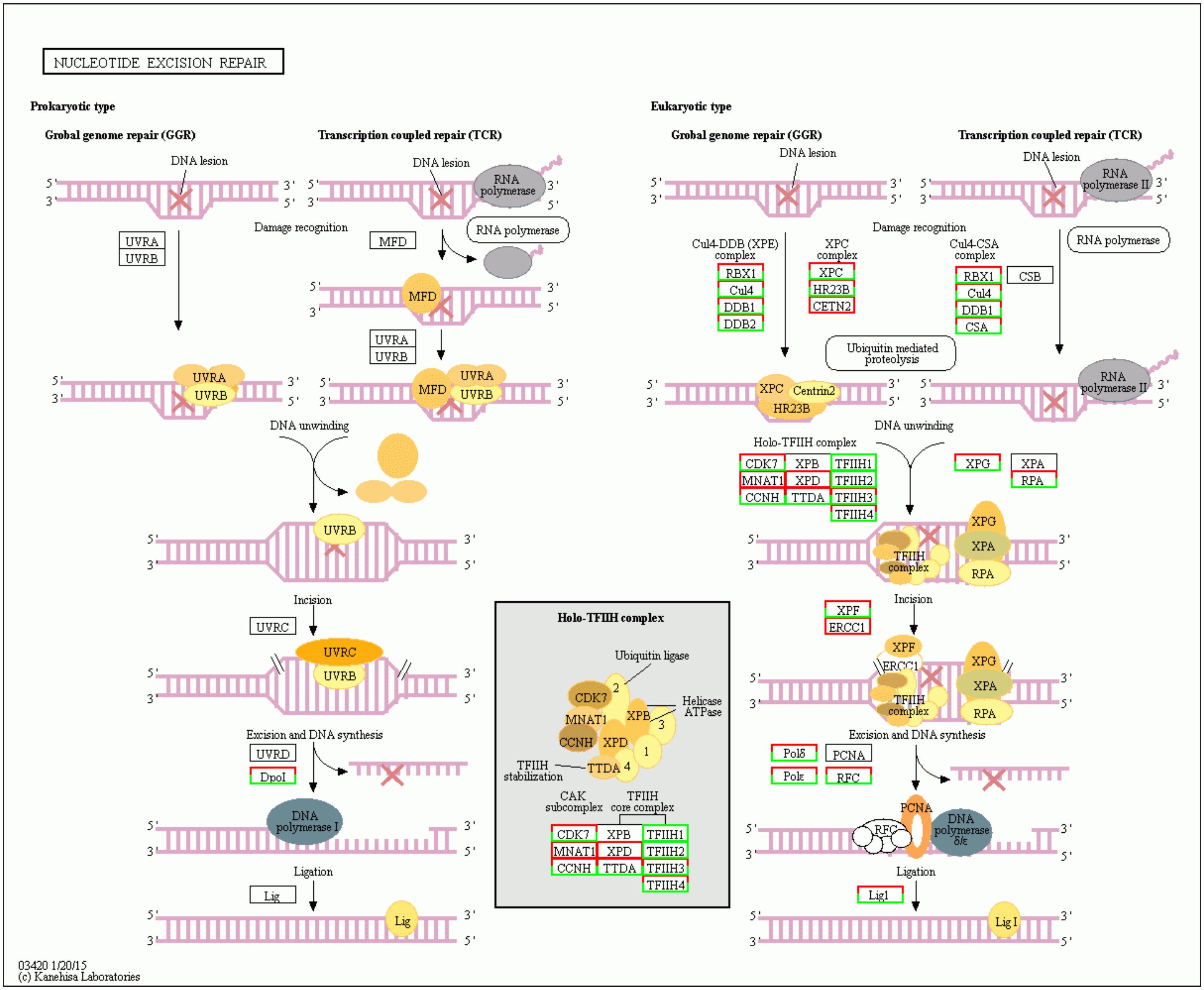
NUCLEOTIDE EXCISION REPAIR
Prokaryotic type
Grobal genome repair (GGR)
Transcription coupled repair (TCR)
Eukaryotic type
Grobal genome repair (GGR)
Transcription coupled repair (TCR)
DNA lesion
RNA polymerase
RNA polymerase II
Damage recognition
UVRA
UVRB
MFD
Cul4-DDB (XPE) complex
RBX1
Cul4
DDB1
DDB2
XPC complex
XPC
HR23B
CETN2
Cul4-CSA complex
CSA
CSB
Ubiquitin mediated proteolysis
Centrin2
DNA unwinding
Holo-TFIIH complex
CDK7
XPB
TFIIH1
MNAT1
XPD
TFIIH2
CCNH
TTDA
TFIIH3
TFIIH4
XPG
XPA
RPA
TFIIH complex
Incision
UVRC
XPF
ERCC1
Ubiquitin ligase
Helicase ATPase
TFIIH stabilization
CAK subcomplex
TFIIH core complex
Excision and DNA synthesis
UVRD
DpoI
DNA polymerase I
Polδ
Polε
PCNA
RFC
DNA polymerase δ/ε
Ligation
Lig
LigI
Lig I
03420 1/20/15
(c) Kanehisa Laboratories

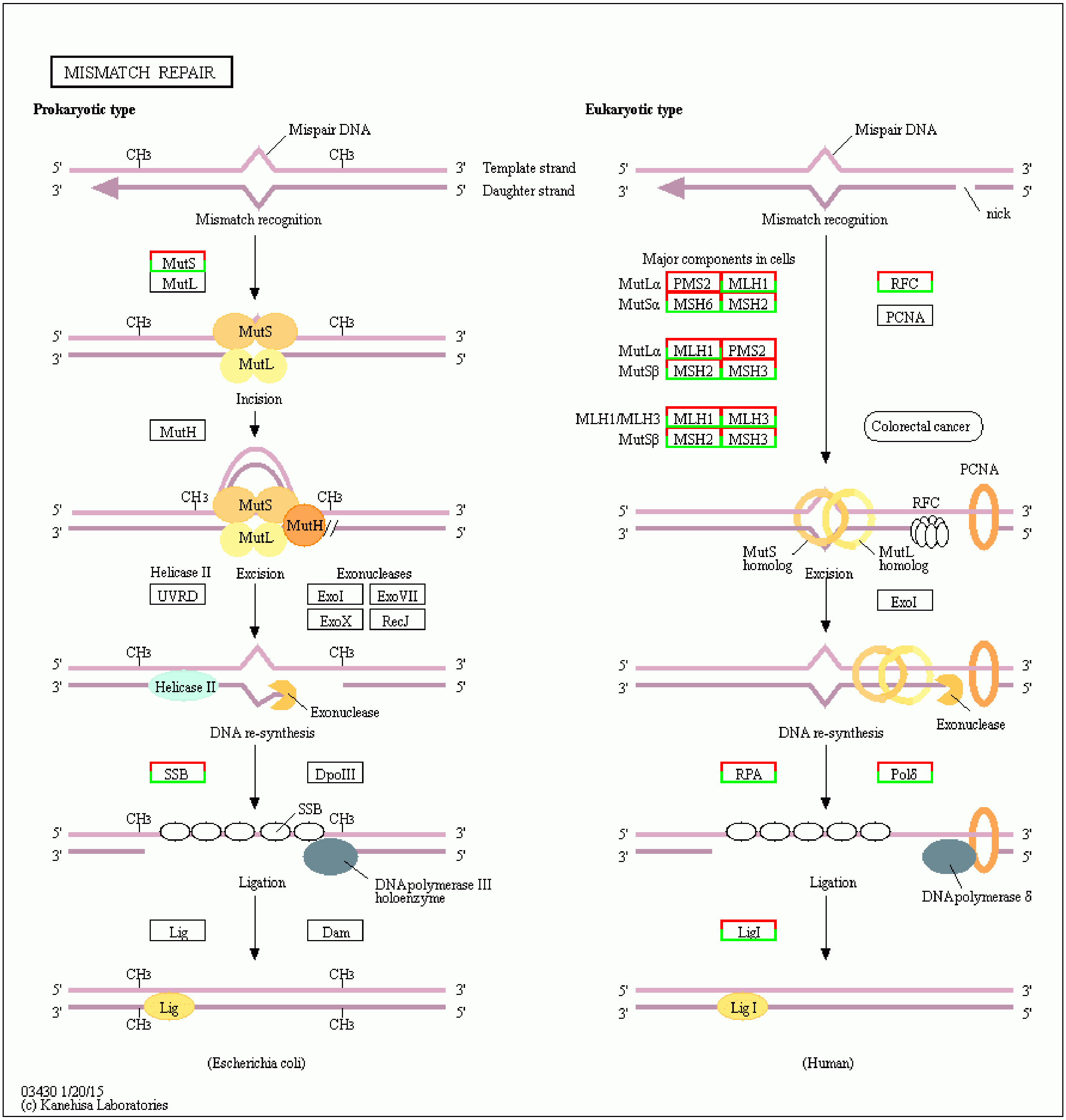
MISMATCH REPAIR
Prokaryotic type
Eukaryotic type
Mispair DNA
CH3
Template strand
Daughter strand
Mismatch recognition
nick
MutS
MutL
Major components in cells
MutLα PMS2 MLH1
MutSα MSH6 MSH2
MutLα MLH1 PMS2
MutSβ MSH2 MSH3
MLH1/MLH3 MLH1 MLH3
MutSβ MSH2 MSH3
RFC
PCNA
Colorectal cancer
Incision
MutH
MutS homolog
MutL homolog
Helicase II
UVRD
Excision
Exonucleases
ExoI
ExoVII
ExoX
RecJ
Exonuclease
DNA re-synthesis
SSB
DpolIII
RPA
Polδ
Ligation
DNApolymerase III holoenzyme
DNApolymerase δ
Lig
Dam
LigI
Lig I
(Escherichia coli)
(Human)
03430 1/20/15
(c) Kanehisa Laboratories

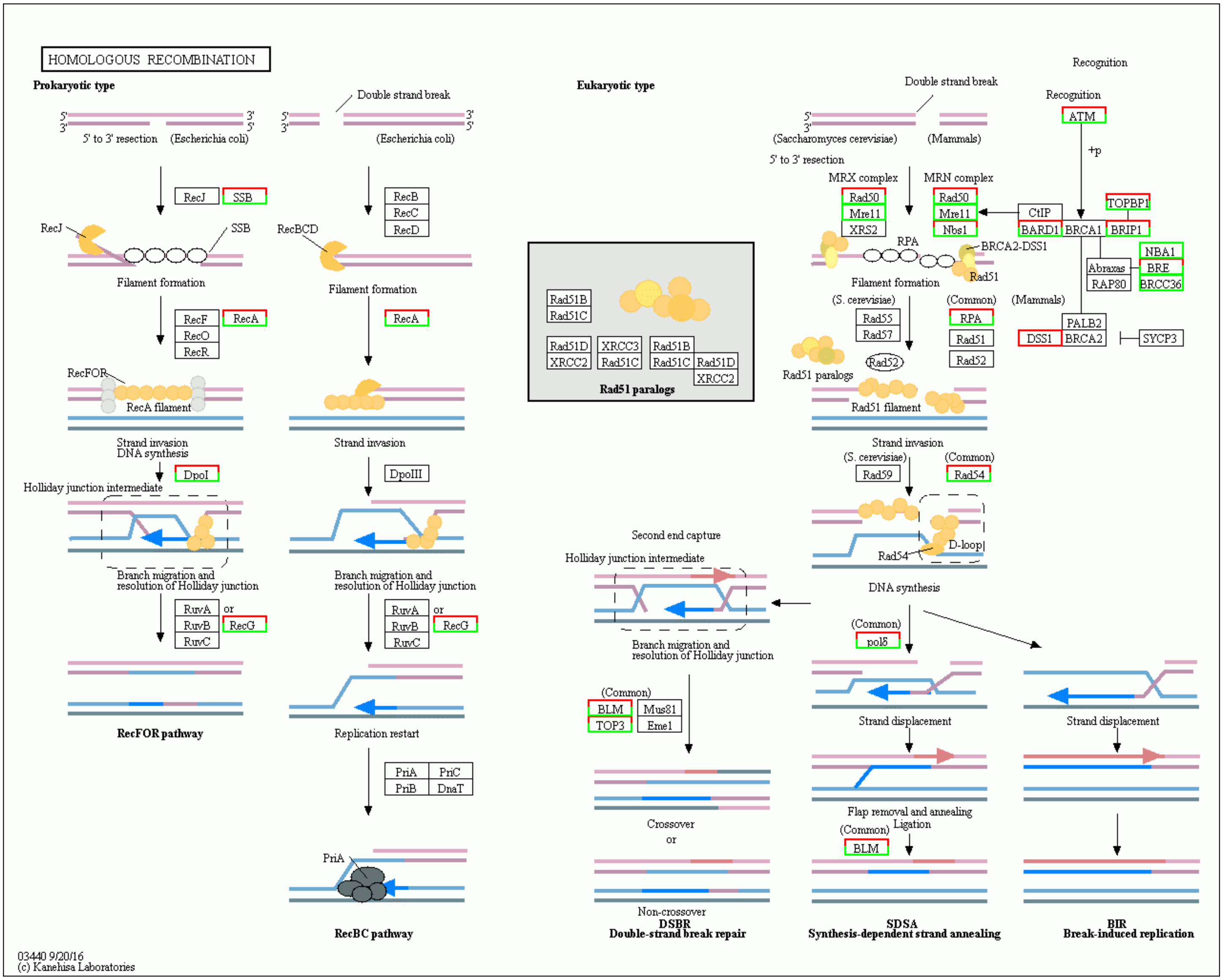

HOMOLOGOUS RECOMBINATION
Prokaryotic type
Double strand break
5' to 3' resection
(Escherichia coli)
(Escherichia coli)
RecJ
SSB
RecB
RecC
RecD
RecJ
SSB
RecBCD
Filament formation
Filament formation
RecF
RecA
RecO
RecR
RecA
RecFOR
RecA filament
Strand invasion
DNA synthesis
Strand invasion
DpoI
DpoIII
Holliday junction intermediate
Branch migration and
resolution of Holliday junction
Branch migration and
resolution of Holliday junction
RuvA
or
RuvB
RecG
RuvC
RuvA
or
RuvB
RecG
RuvC
RecFOR pathway
Replication restart
PriA
PriC
PriB
DnaT
PriA
RecBC pathway
Eukaryotic type
Rad51B
Rad51C
Rad51D
XRCC2
XRCC3
Rad51C
Rad51B
Rad51C
Rad51D
XRCC2
Rad51 paralogs
Double strand break
Recognition
Recognition
ATM
+p
(Saccharomyces cerevisiae)
(Mammals)
5' to 3' resection
MRX complex
MRN complex
Rad50
Mre11
XRS2
Rad50
Mre11
Nbs1
CtIP
TOPBP1
BARD1
BRCA1
BRIP1
RPA
BRCA2-DSS1
Rad51
NBA1
Abraxas
BRE
RAP80
BRCC36
Filament formation
(S. cerevisiae)
(Common)
(Mammals)
Rad55
Rad57
RPA
Rad51
Rad52
PALB2
DSS1
BRCA2
SYCP3
Rad52
Rad51 paralogs
Rad51 filament
Strand invasion
(S. cerevisiae)
(Common)
Rad59
Rad54
Second end capture
Holliday junction intermediate
D-loop
Rad54
DNA synthesis
Branch migration and
resolution of Holliday junction
(Common)
polδ
(Common)
BLM
Mus81
TOP3
Eme1
Strand displacement
Strand displacement
Flap removal and annealing
Ligation
(Common)
BLM
Crossover
or
Non-crossover
DSBR
Double-strand break repair
SDSA
Synthesis-dependent strand annealing
BIR
Break-induced replication
03440 9/20/16
(c) Kanehisa Laboratories

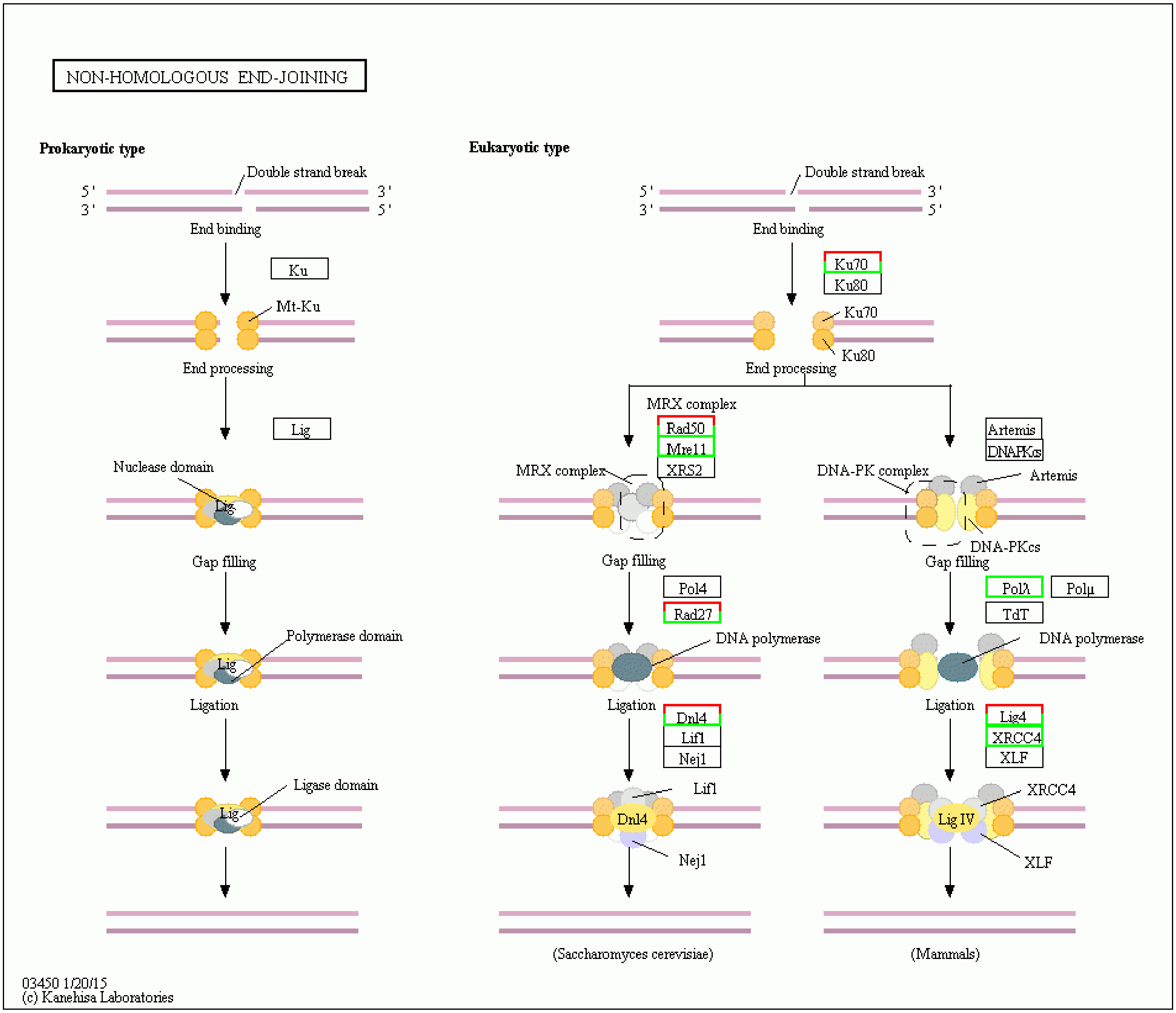
NON-HOMOLOGOUS END-JOINING
Prokaryotic type
Double strand break
5' 3'
3' 5'
End binding
Ku
Mt-Ku
End processing
Lig
Nuclease domain
Lig
Gap filling
Polymerase domain
Lig
Ligation
Ligase domain
Lig
Eukaryotic type
Double strand break
5' 3'
3' 5'
End binding
Ku70
Ku80
Ku70
Ku80
End processing
MRX complex
Rad50
Mre11
XRS2
MRX complex
Artemis
DNAPKcs
DNA-PK complex
Artemis
DNA-PKcs
Gap filling
Pol4
Rad27
DNA polymerase
Gap filling
Polλ
Polμ
TdT
DNA polymerase
Ligation
Dnl4
Lif1
Nej1
Lif1
Dnl4
Nej1
Ligation
Lig4
XRCC4
XLF
XRCC4
Lig IV
XLF
(Saccharomyces cerevisiae)
(Mammals)
03450 1/20/15
(c) Kanehisa Laboratories

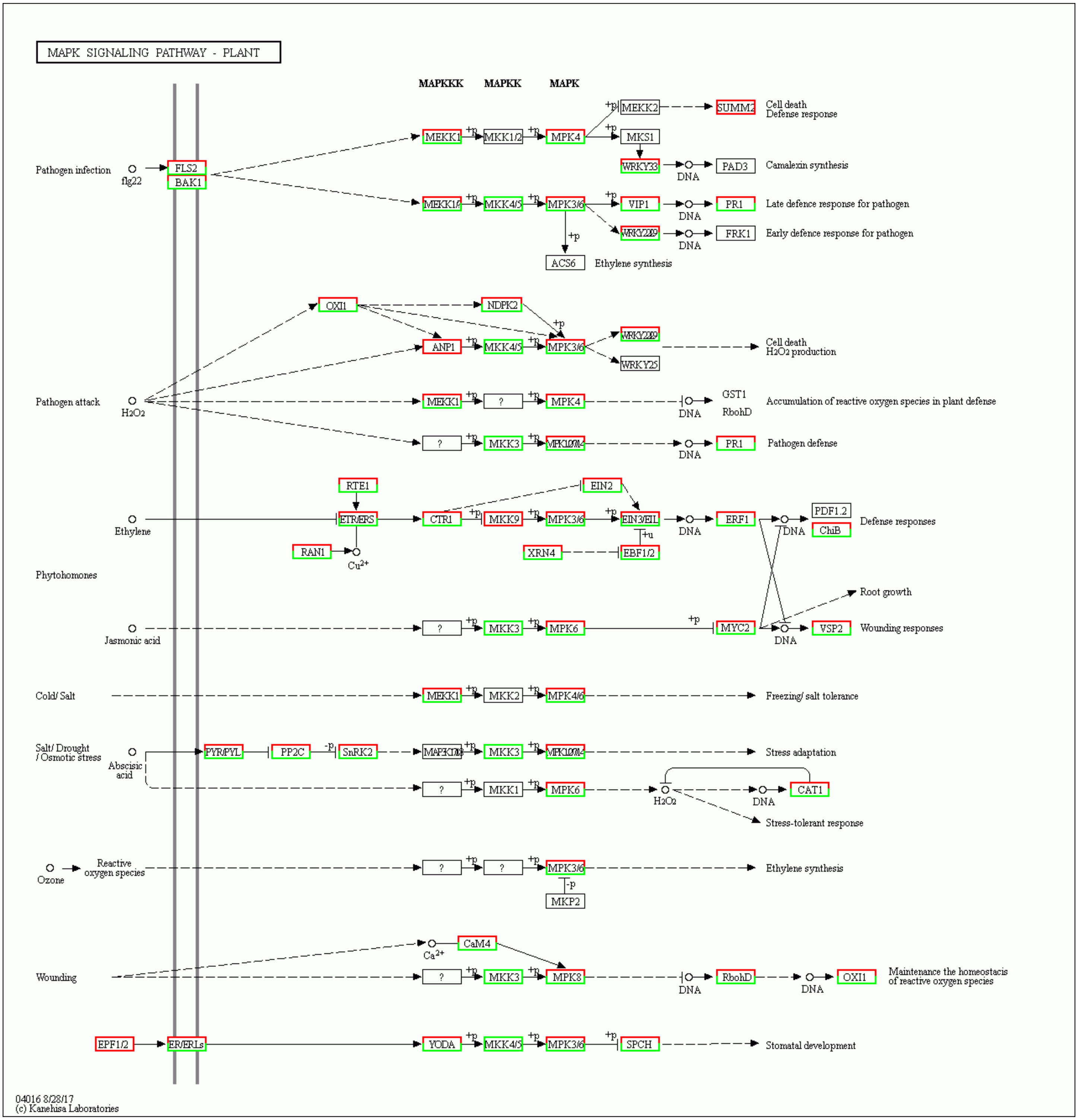
MAPK SIGNALING PATHWAY - PLANT
MAPKKK
MAPKK
MAPK
Pathogen infection
flg22
FLS2
BAK1
MEKK1
MKK1/2
MPK4
MEKK2
SUMM2
Cell death
Defense response
MKS1
WRKY33
PAD3
Camalexin synthesis
DNA
MKK4/5
MPK3/6
VIP1
PR1
Late defence response for pathogen
FRK1
Early defence response for pathogen
ACS6
Ethylene synthesis
OXI1
NDPK2
ANP1
WRKY25
H2O2 production
Pathogen attack
H2O2
GST1
RbohD
Accumulation of reactive oxygen species in plant defense
MKK3
Pathogen defense
RTE1
EIN2
Ethylene
ETR/ERS
CTR1
MKK9
EIN3/EIL
ERF1
PDF1.2
ChiB
Defense responses
RAN1
XRN4
EBF1/2
Phytohomones
Root growth
Jasmonic acid
MPK6
MYC2
VSP2
Wounding responses
Cold/ Salt
MKK2
MPK4/6
Freezing/ salt tolerance
Salt/ Drought / Osmotic stress
Abscisic acid
PYR/PYL
PP2C
SnRK2
Stress adaptation
MKK1
CAT1
Stress-tolerant response
Ozone
Reactive oxygen species
MKP2
Wounding
CaM4
MPK8
OXI1
Maintenance the homeostacis of reactive oxygen species
EPF1/2
ER/ERLs
YODA
SPCH
Stomatal development
04016 8/28/17
(c) Kanehisa Laboratories

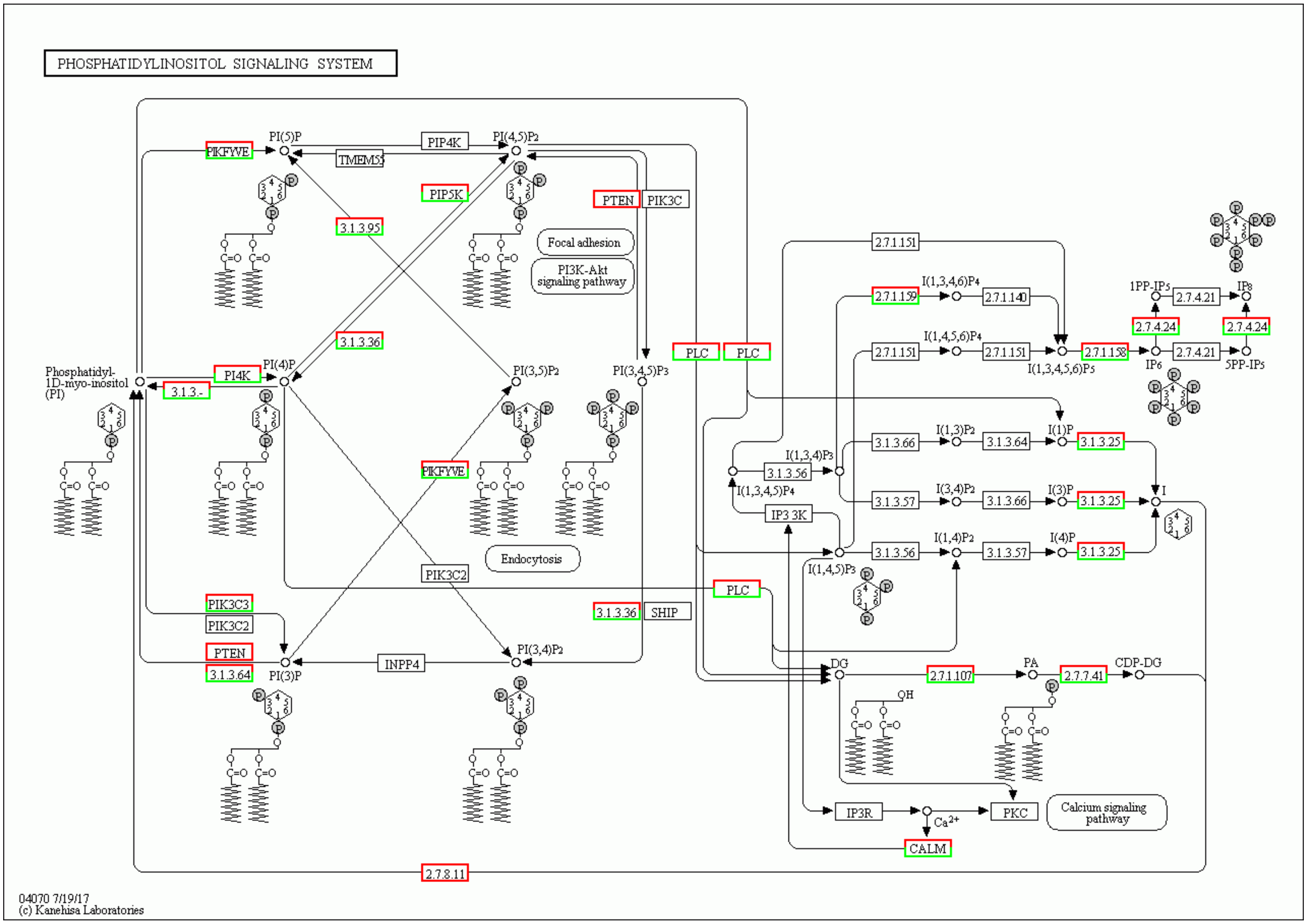
PHOSPHATIDYLINOSITOL SIGNALING SYSTEM
PIKFYVE
PI(5)P
PIP4K
PI(4,5)P2
TMEM55
PIP5K
PTEN
PIK3C
3.1.3.95
Focal adhesion
PI3K-Akt signaling pathway
3.1.3.36
PI4K
PI(4)P
Phosphatidyl-1D-myo-inositol (PI)
3.1.3.-
PI(3,5)P2
PI(3,4,5)P3
PLC
PLC
PIKFYVE
Endocytosis
PIK3C2
PIK3C3
PIK3C2
PTEN
3.1.3.64
PI(3)P
INPP4
PI(3,4)P2
3.1.3.36
SHIP
PLC
2.7.1.151
2.7.1.159
I(1,3,4,6)P4
2.7.1.140
2.7.1.151
I(1,4,5,6)P4
2.7.1.151
I(1,3,4,5,6)P5
2.7.1.158
IP6
1PP-IP5
2.7.4.21
IP8
2.7.4.24
2.7.4.24
2.7.4.21
5PP-IP5
I(1,3,4)P3
3.1.3.56
I(1,3,4,5)P4
IP3 3K
3.1.3.66
I(1,3)P2
3.1.3.64
I(1)P
3.1.3.25
3.1.3.57
I(3,4)P2
3.1.3.66
I(3)P
3.1.3.25
I
I(1,4,5)P3
3.1.3.56
I(1,4)P2
3.1.3.57
I(4)P
3.1.3.25
DG
2.7.1.107
PA
2.7.7.41
CDP-DG
IP3R
Ca2+
PKC
Calcium signaling pathway
CALM
2.7.8.11
04070 7/19/17
(c) Kanehisa Laboratories

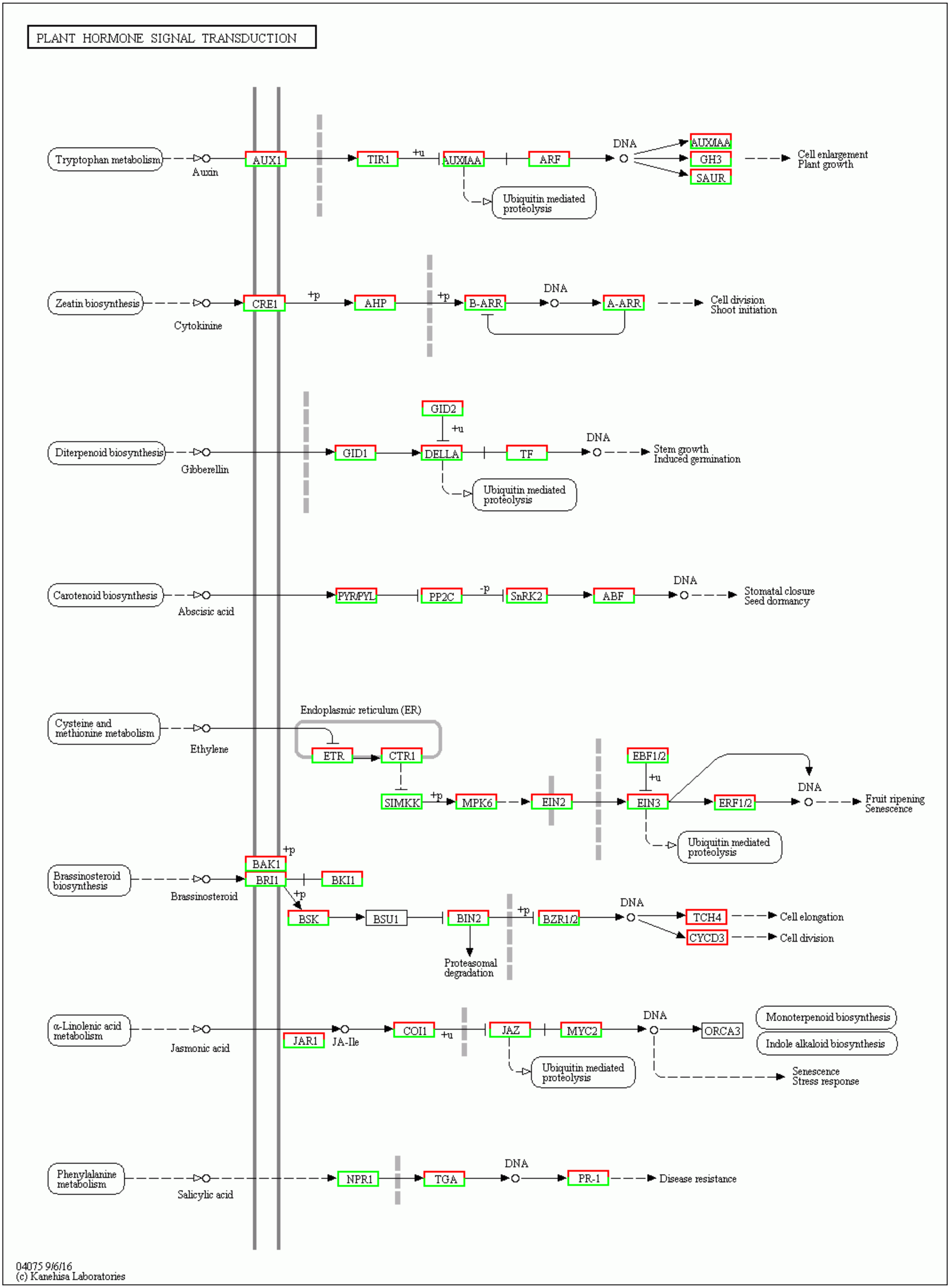
PLANT HORMONE SIGNAL TRANSDUCTION
Tryptophan metabolism
Auxin
AUX1
TIR1
+u
AUX/IAA
ARF
DNA
AUX/IAA
GH3
SAUR
Cell enlargement
Plant growth
Ubiquitin mediated proteolysis
Zeatin biosynthesis
Cytokinine
CRE1
+p
AHP
+p
B-ARR
DNA
A-ARR
Cell division
Shoot initiation
GID2
+u
Diterpenoid biosynthesis
Gibberellin
GID1
DELLA
TF
DNA
Stem growth
Induced germination
Ubiquitin mediated proteolysis
Carotenoid biosynthesis
Abscisic acid
PYR/PYL
PP2C
-p
SnRK2
ABF
DNA
Stomatal closure
Seed dormancy
Endoplasmic reticulum (ER)
Cysteine and methionine metabolism
Ethylene
ETR
CTR1
SIMKK
+p
MPK6
EIN2
EBF1/2
+u
EIN3
ERF1/2
DNA
Fruit ripening
Senescence
Ubiquitin mediated proteolysis
Brassinosteroid biosynthesis
Brassinosteroid
+p
BAK1
BRI1
BKI1
+p
BSK
BSU1
BIN2
+p
BZR1/2
DNA
TCH4
Cell elongation
CYCD3
Cell division
Proteasomal degradation
α-Linolenic acid metabolism
Jasmonic acid
JAR1
JA-Ile
COI1
+u
JAZ
MYC2
DNA
ORCA3
Monoterpenoid biosynthesis
Indole alkaloid biosynthesis
Ubiquitin mediated proteolysis
Senescence
Stress response
Phenylalanine metabolism
Salicylic acid
NPR1
TGA
DNA
PR-1
Disease resistance
04075 9/6/16
(c) Kanehisa Laboratories

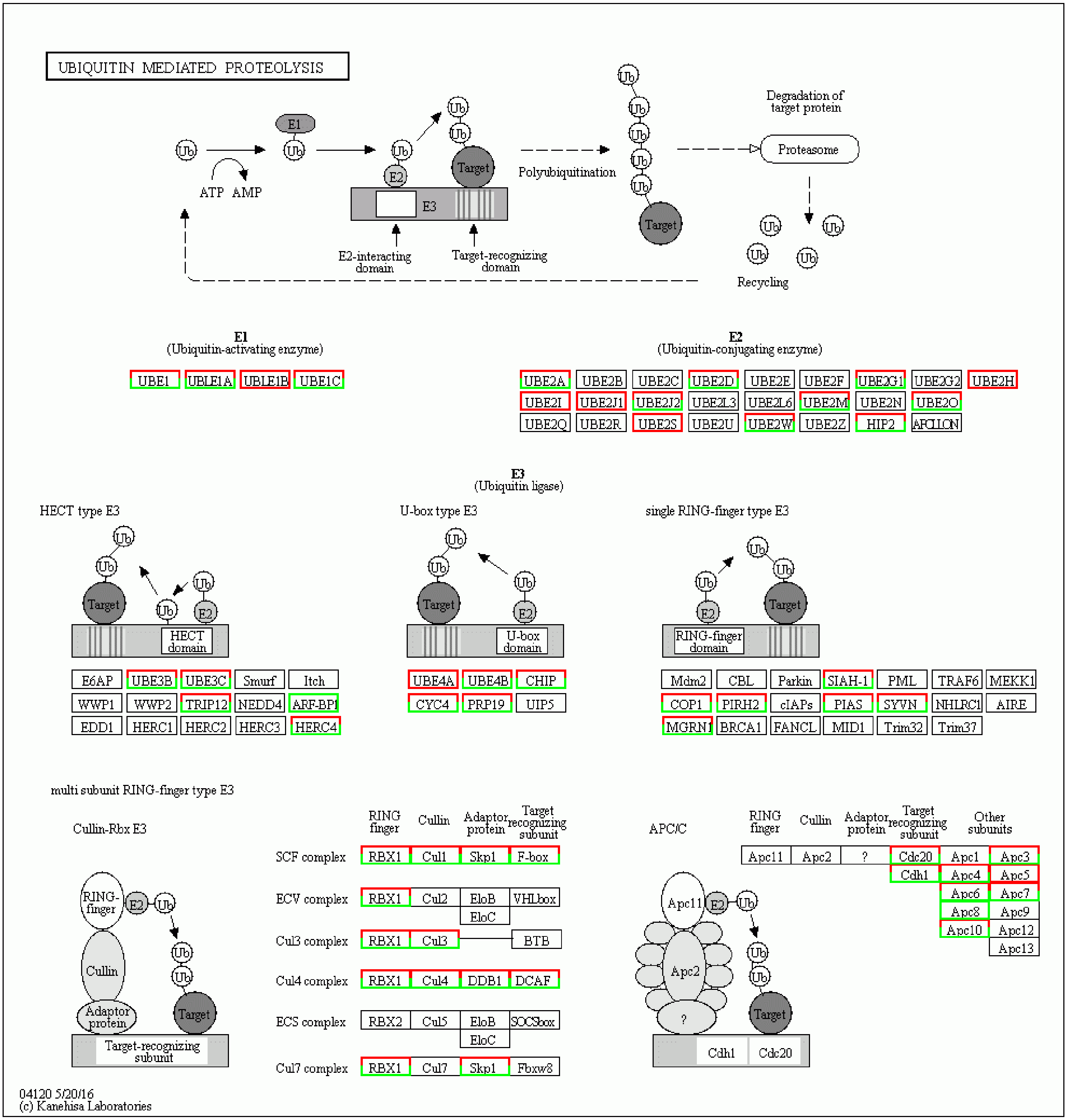
UBIQUITIN MEDIATED PROTEOLYSIS
Ub
E1
E2
E3
Target
ATP AMP
E2-interacting domain
Target-recognizing domain
Polyubiquitination
Degradation of target protein
Proteasome
Recycling
E1
(Ubiquitin-activating enzyme)
UBE1
UBLE1A
UBLE1B
UBE1C
E2
(Ubiquitin-conjugating enzyme)
UBE2A
UBE2B
UBE2C
UBE2D
UBE2E
UBE2F
UBE2G1
UBE2G2
UBE2H
UBE2I
UBE2J1
UBE2J2
UBE2L3
UBE2L6
UBE2M
UBE2N
UBE2O
UBE2Q
UBE2R
UBE2S
UBE2U
UBE2W
UBE2Z
HIP2
APOLLON
E3
(Ubiquitin ligase)
HECT type E3
HECT domain
E6AP
UBE3B
UBE3C
Smurf
Itch
WWP1
WWP2
TRIP12
NEDD4
ARF-BP1
EDD1
HERC1
HERC2
HERC3
HERC4
U-box type E3
U-box domain
UBE4A
UBE4B
CHIP
CYC4
PRP19
UIP5
single RING-finger type E3
RING-finger domain
Mdm2
CBL
Parkin
SIAH-1
PML
TRAF6
MEKK1
COP1
PIRH2
cIAPs
PIAS
SYVN
NHLRC1
AIRE
MGRN1
BRCA1
FANCL
MID1
Trim32
Trim37
multi subunit RING-finger type E3
Cullin-Rbx E3
RING-finger
Cullin
Adaptor protein
Target-recognizing subunit
RING finger
Cullin
Adaptor protein
Target recognizing subunit
SCF complex
RBX1
Cul1
Skp1
F-box
ECV complex
RBX1
Cul2
EloB
VHLbox
EloC
Cul3 complex
RBX1
Cul3
BTB
Cul4 complex
RBX1
Cul4
DDB1
DCAF
ECS complex
RBX2
Cul5
EloB
SOCSbox
EloC
Cul7 complex
RBX1
Cul7
Skp1
Fbxw8
APC/C
Apc11
Apc2
?
Cdh1
Cdc20
RING finger
Cullin
Adaptor protein
Target recognizing subunit
Other subunits
Apc11
Apc2
?
Cdc20
Cdh1
Apc1
Apc3
Apc4
Apc5
Apc6
Apc7
Apc8
Apc9
Apc10
Apc12
Apc13
04120 5/20/16
(c) Kanehisa Laboratories

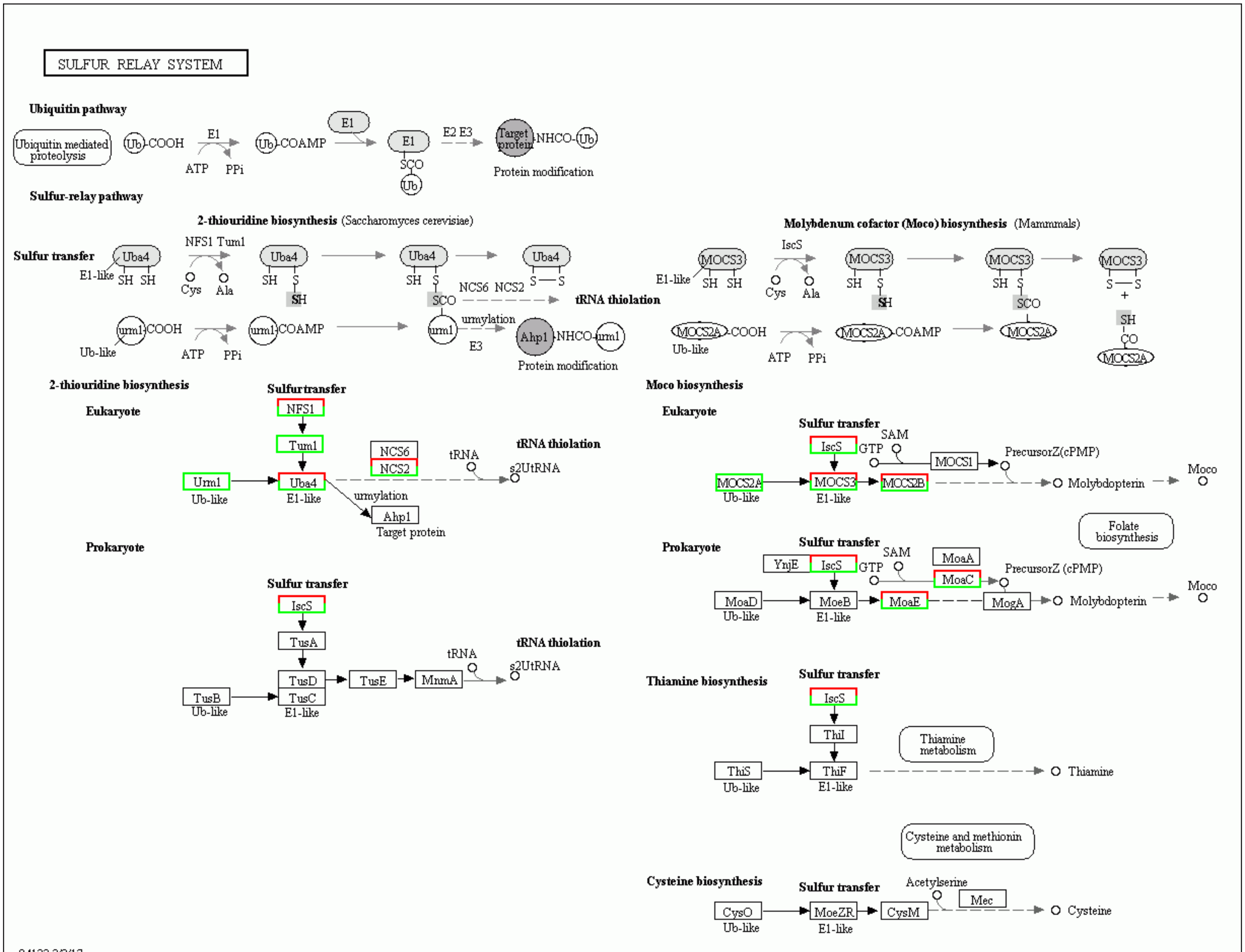

04122 2/2/17
(c) Kanehisa Laboratories

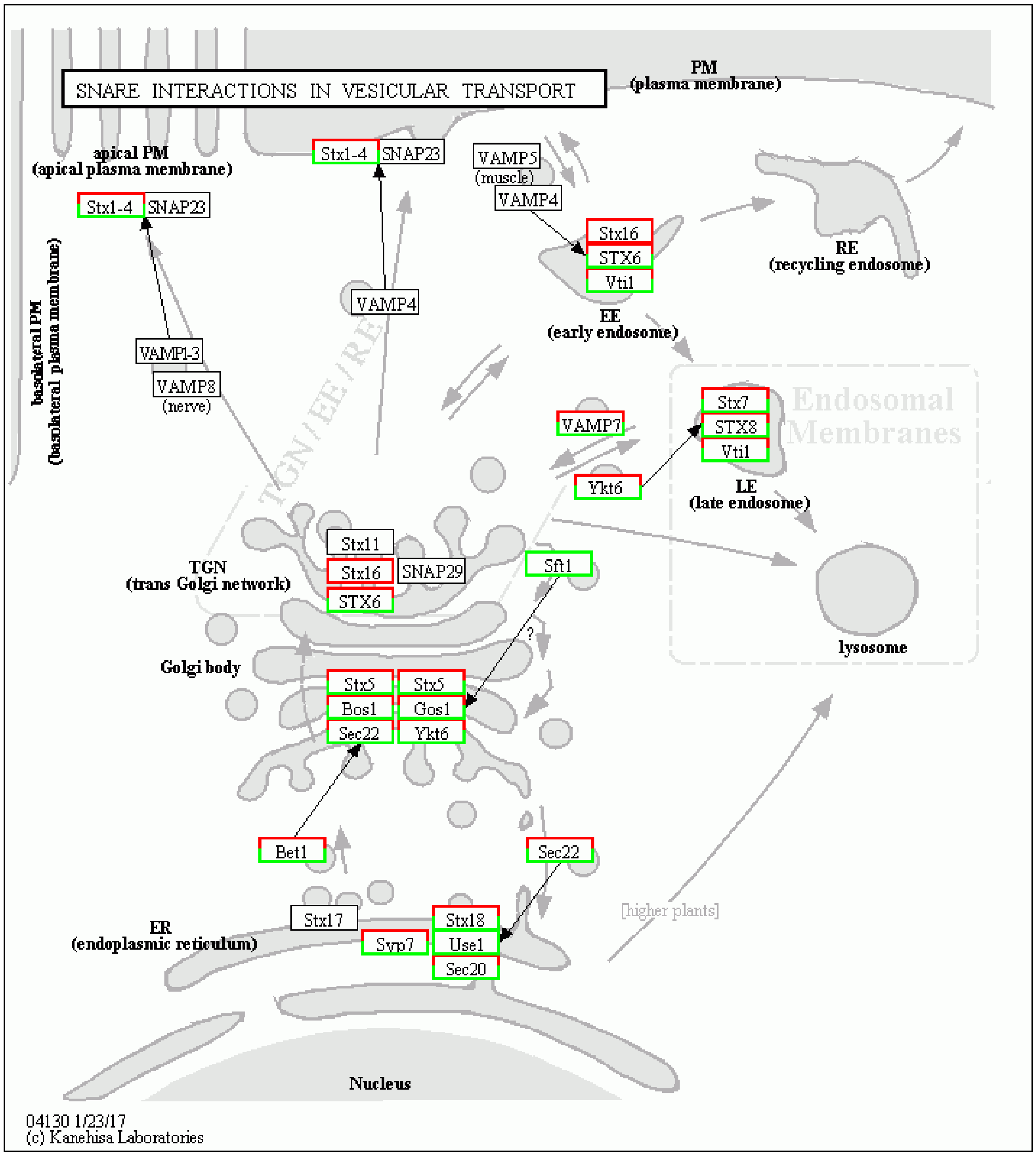

04130 1/23/17
(c) Kanehisa Laboratories

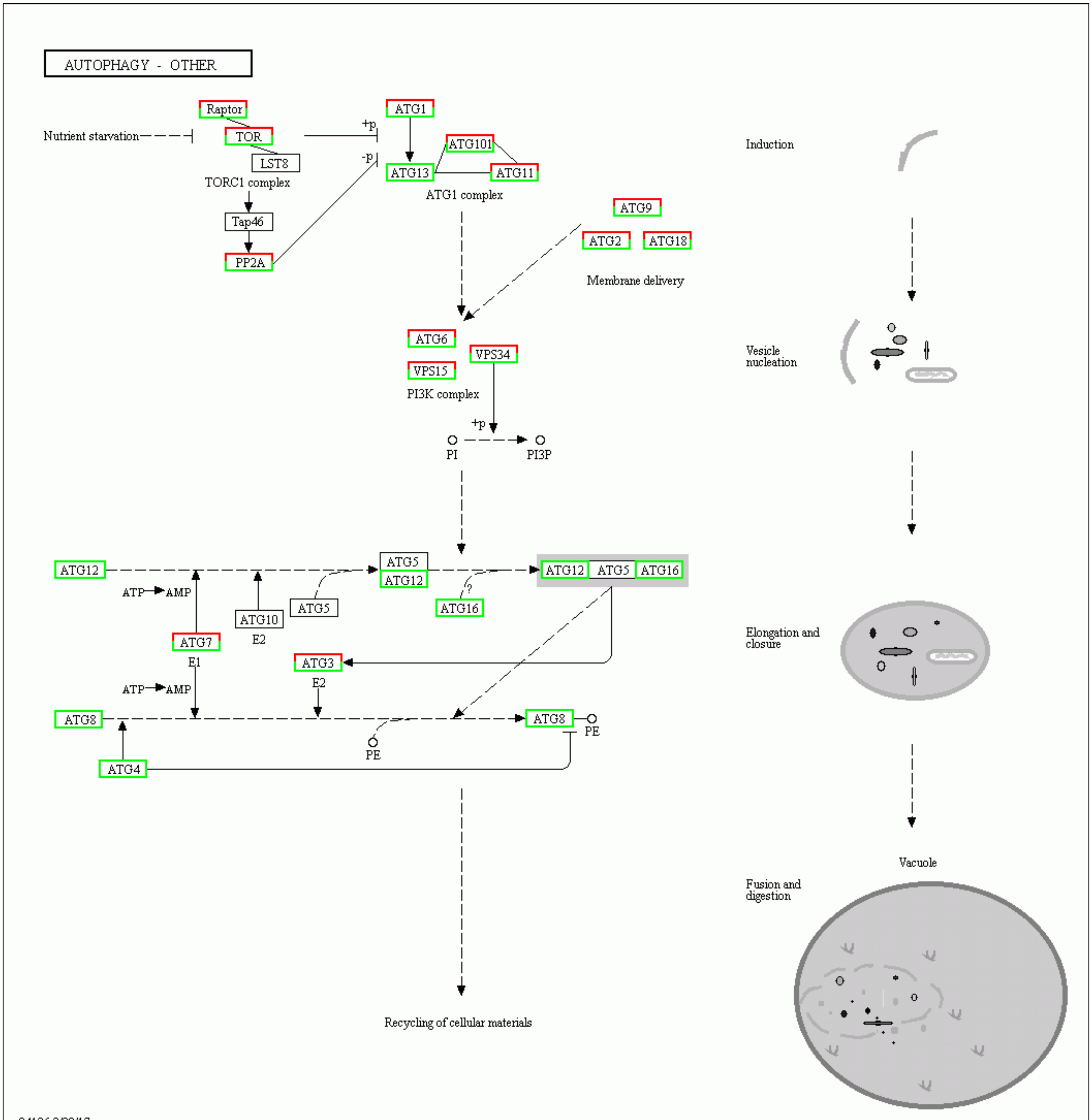

04136 3/28/17
(c) Kanehisa Laboratories

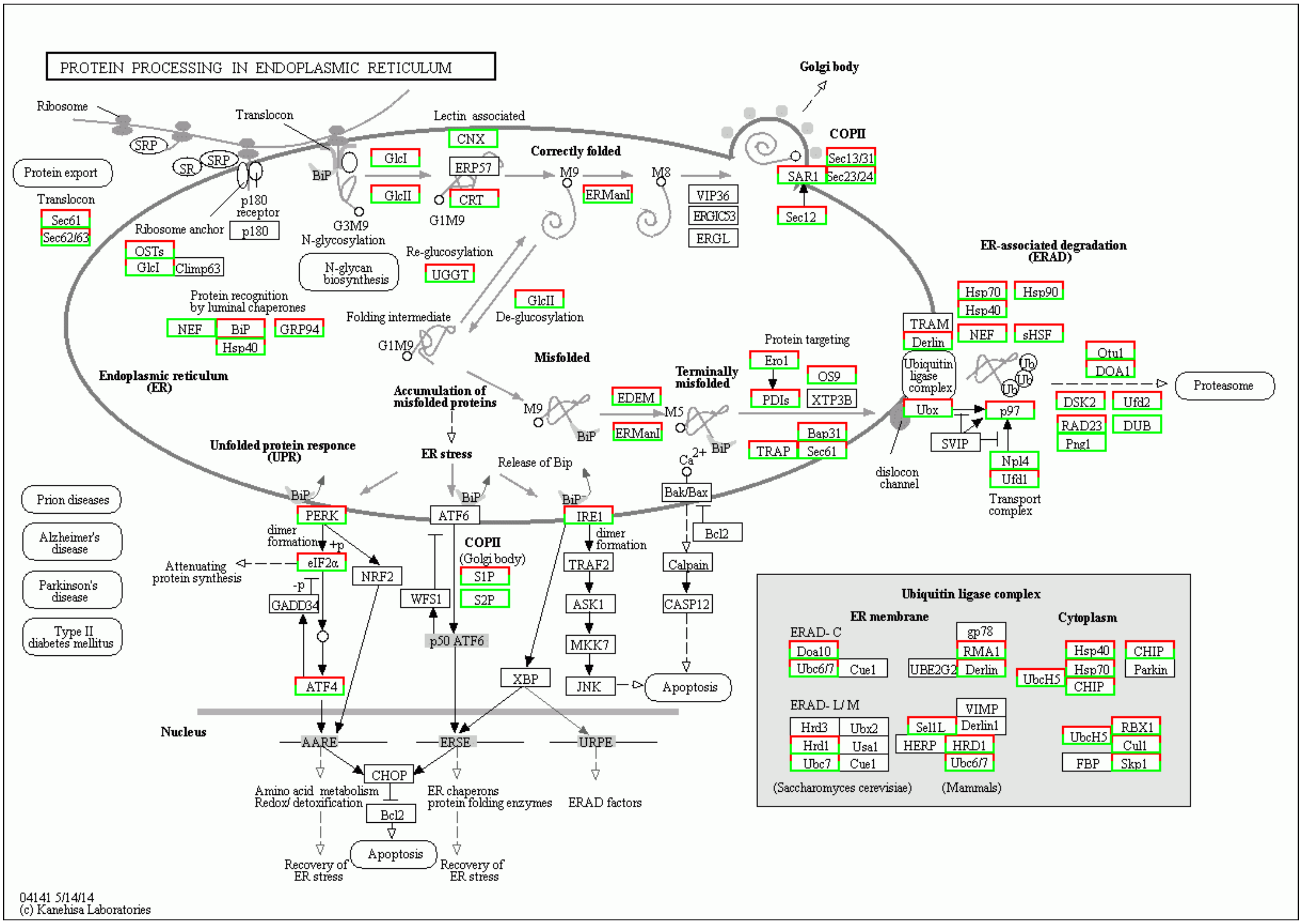

PROTEIN PROCESSING IN ENDOPLASMIC RETICULUM
Ribosome
Translocon
SRP
SR
p180 receptor
Protein export
Sec61
Sec62/63
Ribosome anchor
p180
OSTs
GlcI
Climp63
BiP
G3M9
N-glycosylation
N-glycan biosynthesis
Lectin associated
CNX
GlcI
ERP57
GlcII
CRT
G1M9
Correctly folded
M9
M8
ERManI
VIP36
ERGIC53
ERGL
Golgi body
COPII
Sec13/31
SAR1
Sec23/24
Sec12
Re-glucosylation
UGGT
GlcII
De-glucosylation
Folding intermediate
Protein recognition by luminal chaperones
NEF
BiP
GRP94
Hsp40
Endoplasmic reticulum (ER)
Misfolded
Accumulation of misfolded proteins
EDEM
ERManI
M5
Terminally misfolded
Protein targeting
Ero1
OS9
PDIs
XTP3B
Bap31
TRAP
Sec61
ER stress
Release of Bip
Unfolded protein responce (UPR)
ER-associated degradation (ERAD)
Hsp70
Hsp90
Hsp40
NEF
sHSF
TRAM
Derlin
Ubiquitin ligase complex
Ubx
p97
SVIP
Npl4
Ufd1
Transport complex
dislocon channel
Otu1
DOA1
Proteasome
DSK2
Ufd2
RAD23
DUB
Png1
Prion diseases
Alzheimer's disease
Parkinson's disease
Type II diabetes mellitus
PERK
dimer formation
+p
eIF2α
-p
GADD34
Attenuating protein synthesis
NRF2
ATF4
ATF6
WFS1
COPII (Golgi body)
S1P
S2P
p50 ATF6
IRE1
TRAF2
ASK1
MKK7
JNK
XBP
Ca2+
Bak/Bax
Bcl2
Calpain
CASP12
Apoptosis
Nucleus
AARE
ERSE
URPE
CHOP
Bcl2
Amino acid metabolism
Redox/ detoxification
ER chaperons
protein folding enzymes
ERAD factors
Apoptosis
Recovery of ER stress
Recovery of ER stress
Ubiquitin ligase complex
ER membrane
ERAD- C
Doa10
Ubc6/7
Cue1
gp78
RMA1
UBE2G2
Derlin
ERAD- L/ M
Hrd3
Ubx2
Hrd1
Usa1
Ubc7
Cue1
VIMP
Sel1L
Derlin1
HERP
HRD1
Ubc6/7
(Saccharomyces cerevisiae)
(Mammals)
Cytoplasm
Hsp40
CHIP
UbcH5
Hsp70
Parkin
CHIP
UbcH5
RBX1
Cul1
FBP
Skp1
04141 5/14/14
(c) Kanehisa Laboratories

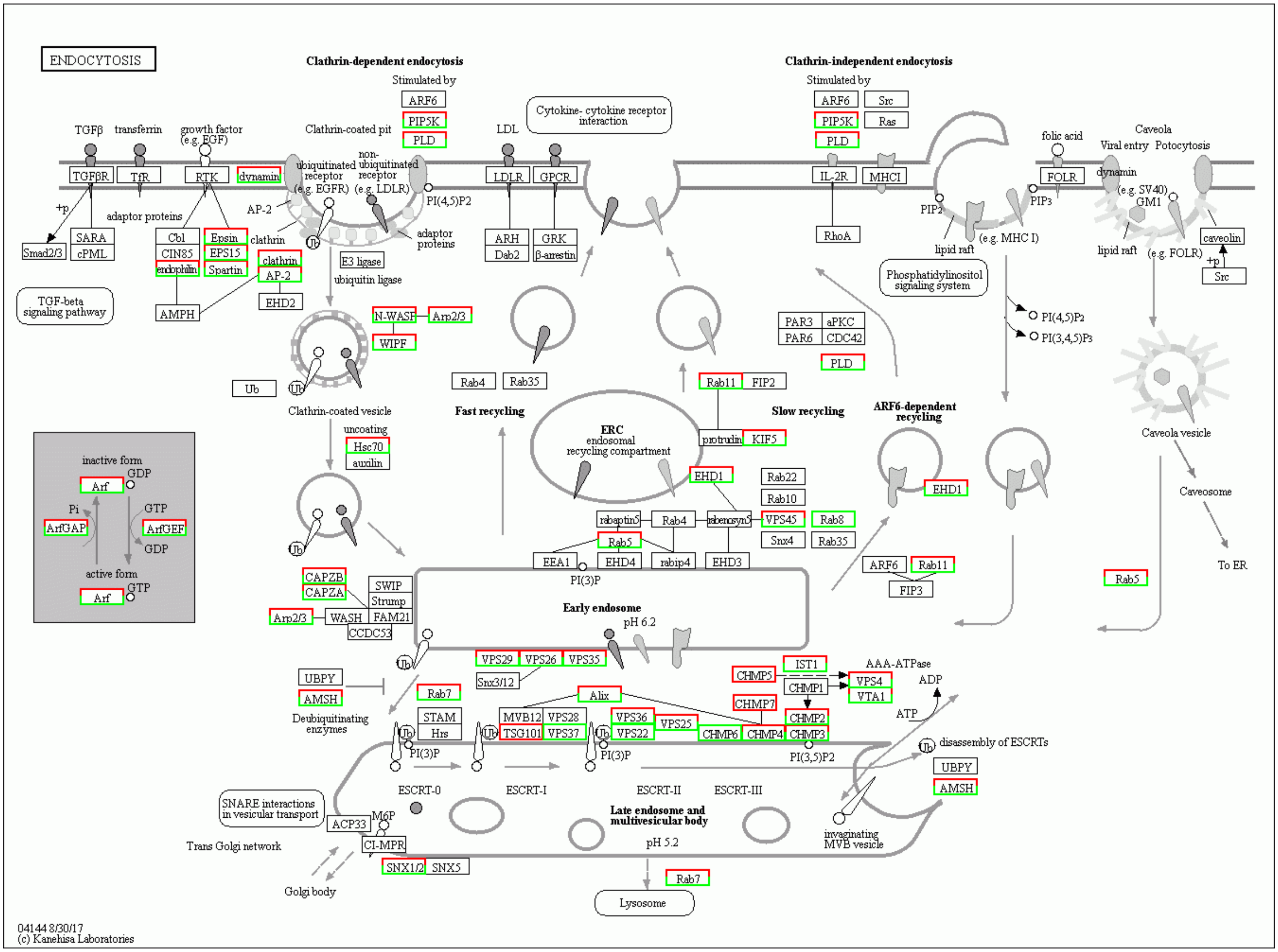
ENDOCYTOSIS
Clathrin-dependent endocytosis
Stimulated by
ARF6
PIP5K
PLD
Cytokine- cytokine receptor interaction
Clathrin-independent endocytosis
Stimulated by
ARF6
Src
PIP5K
Ras
PLD
TGFβ
transferrin
growth factor (e.g. EGF)
Clathrin-coated pit
LDL
folic acid
Caveola
Viral entry
Potocytosis
TGFβR
TfR
RTK
dynamin
ubiquitinated receptor (e.g. EGFR)
non-ubiquitinated receptor (e.g. LDLR)
LDLR
GPCR
IL-2R
MHCI
FOLR
dynamin
(e.g. SV40)
GM1
PI(4,5)P2
AP-2
+p
adaptor proteins
adaptor proteins
PIP2
PIP3
Smad2/3
SARA
cPML
Cbl
CIN85
endophilin
Epsin
EPS15
Spartin
clathrin
clathrin
AP-2
E3 ligase
ubiquitin ligase
ARH
Dab2
GRK
β-arrestin
RhoA
lipid raft
(e.g. MHC I)
lipid raft
(e.g. FOLR)
caveolin
+p
Src
Phosphatidylinositol signaling system
TGF-beta signaling pathway
AMPH
EHD2
N-WASP
Arp2/3
WIPF
PI(4,5)P2
PI(3,4,5)P3
PAR3
aPKC
PAR6
CDC42
PLD
Ub
Ub
Rab4
Rab35
Rab11
FIP2
Clathrin-coated vesicle
uncoating
Hsc70
auxilin
Fast recycling
ERC
endosomal recycling compartment
Slow recycling
ARF6-dependent recycling
Caveola vesicle
inactive form
GDP
Arf
Pi
GTP
ArfGAP
ArfGEF
GDP
active form
GTP
Arf
protrudin
KIF5
EHD1
Rab22
Rab10
EHD1
Caveosome
rabaptin5
Rab4
rabenosyn5
VPS45
Rab8
Snx4
Rab35
Rab5
EEA1
EHD4
rabip4
EHD3
ARF6
Rab11
Rab5
To ER
PI(3)P
FIP3
Ub
CAPZB
CAPZA
SWIP
Strump
Arp2/3
WASH
FAM21
CCDC53
Early endosome
pH 6.2
Ub
VPS29
VPS26
VPS35
UBPY
AMSH
Snx3/12
Rab7
Deubiquitinating enzymes
STAM
Hrs
MVB12
VPS28
TSG101
VPS37
Alix
VPS36
VPS22
VPS25
CHMP5
IST1
CHMP1
CHMP7
AAA-ATPase
VPS4
VTA1
ADP
ATP
CHMP2
CHMP6
CHMP4
CHMP3
Ub
disassembly of ESCRTs
Ub
PI(3)P
Ub
Ub
PI(3)P
PI(3,5)P2
UBPY
AMSH
ESCRT-0
ESCRT-I
ESCRT-II
ESCRT-III
SNARE interactions in vesicular transport
M6P
ACP33
Late endosome and multivesicular body
invaginating MVB vesicle
Trans Golgi network
CI-MPR
pH 5.2
SNX1/2
SNX5
Rab7
Golgi body
Lysosome
04144 8/30/17
(c) Kanehisa Laboratories

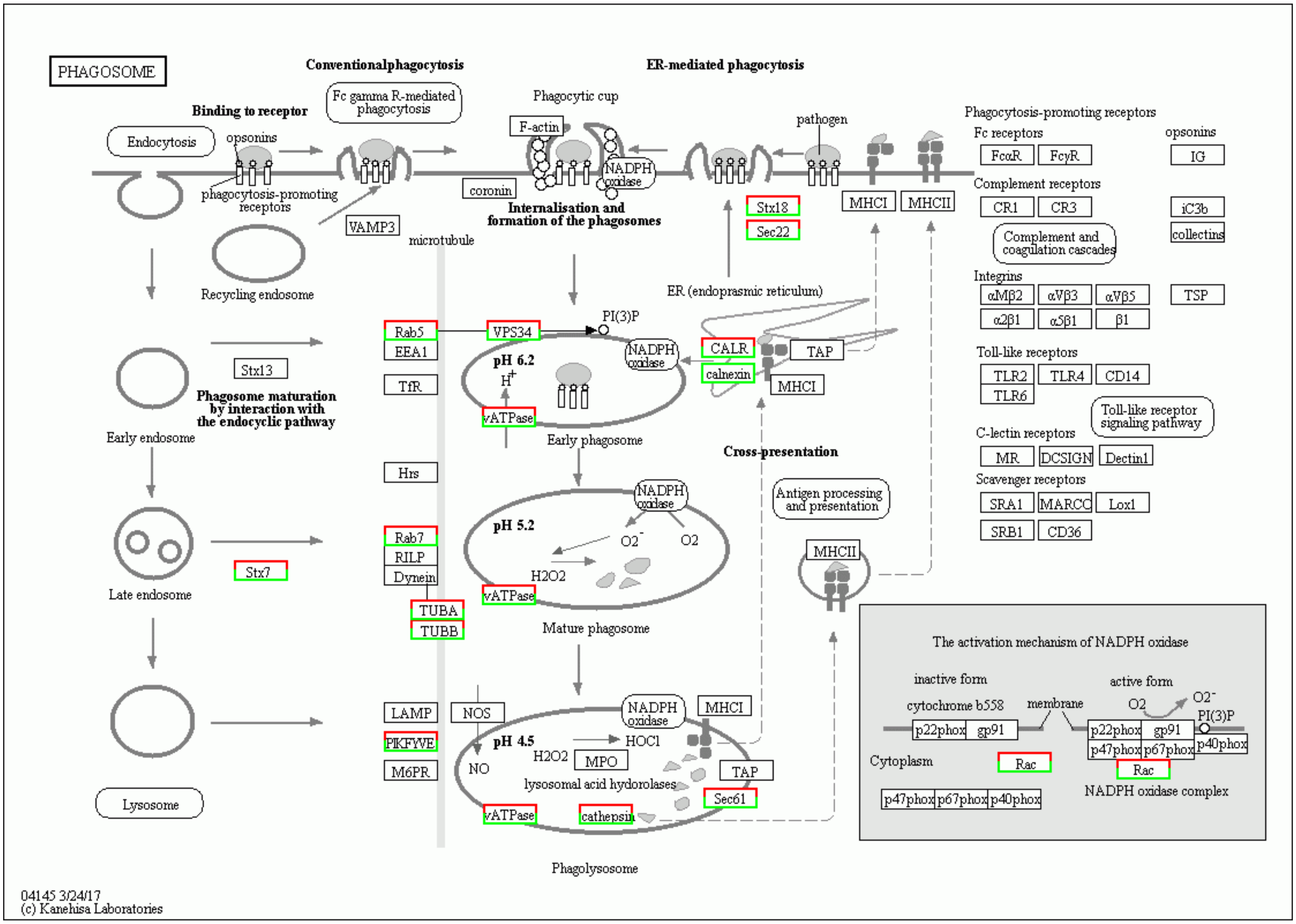
PHAGOSOME
Conventionalphagocytosis
ER-mediated phagocytosis
Binding to receptor
Fc gamma R-mediated phagocytosis
Phagocytic cup
Endocytosis
opsonins
F-actin
pathogen
phagocytosis-promoting receptors
NADPH oxidase
coronin
Stx18
Sec22
MHCI
MHCII
VAMP3
Internalisation and formation of the phagosomes
microtubule
Recycling endosome
ER (endoprasmic reticulum)
Phagocytosis-promoting receptors
Fc receptors
FcαR
FcγR
opsonins
IG
Complement receptors
CR1
CR3
iC3b
Complement and coagulation cascades
collectins
Integrins
αMβ2
αVβ3
αVβ5
TSP
α2β1
α5β1
β1
Toll-like receptors
TLR2
TLR4
CD14
TLR6
Toll-like receptor signaling pathway
C-lectin receptors
MR
DCSIGN
Dectin1
Scavenger receptors
SRA1
MARCO
Lox1
SRB1
CD36
Rab5
VPS34
PI(3)P
EEA1
pH 6.2
CALR
TAP
calnexin
MHCI
Stx13
TfR
Phagosome maturation by interaction with the endocyclic pathway
vATPase
Early endosome
Early phagosome
Cross-presentation
Hrs
Antigen processing and presentation
pH 5.2
Rab7
RILP
Dynein
O2-
O2
H2O2
Stx7
Late endosome
MHCII
TUBA
TUBB
Mature phagosome
The activation mechanism of NADPH oxidase
inactive form
active form
cytochrome b558
membrane
p22phox
gp91
Cytoplasm
Rac
p47phox
p67phox
p40phox
NADPH oxidase complex
LAMP
NOS
pH 4.5
HOCl
PIKFYVE
M6PR
NO
MPO
lysosomal acid hydrolases
Sec61
Lysosome
cathepsin
Phagolysosome
04145 3/24/17
(c) Kanehisa Laboratories

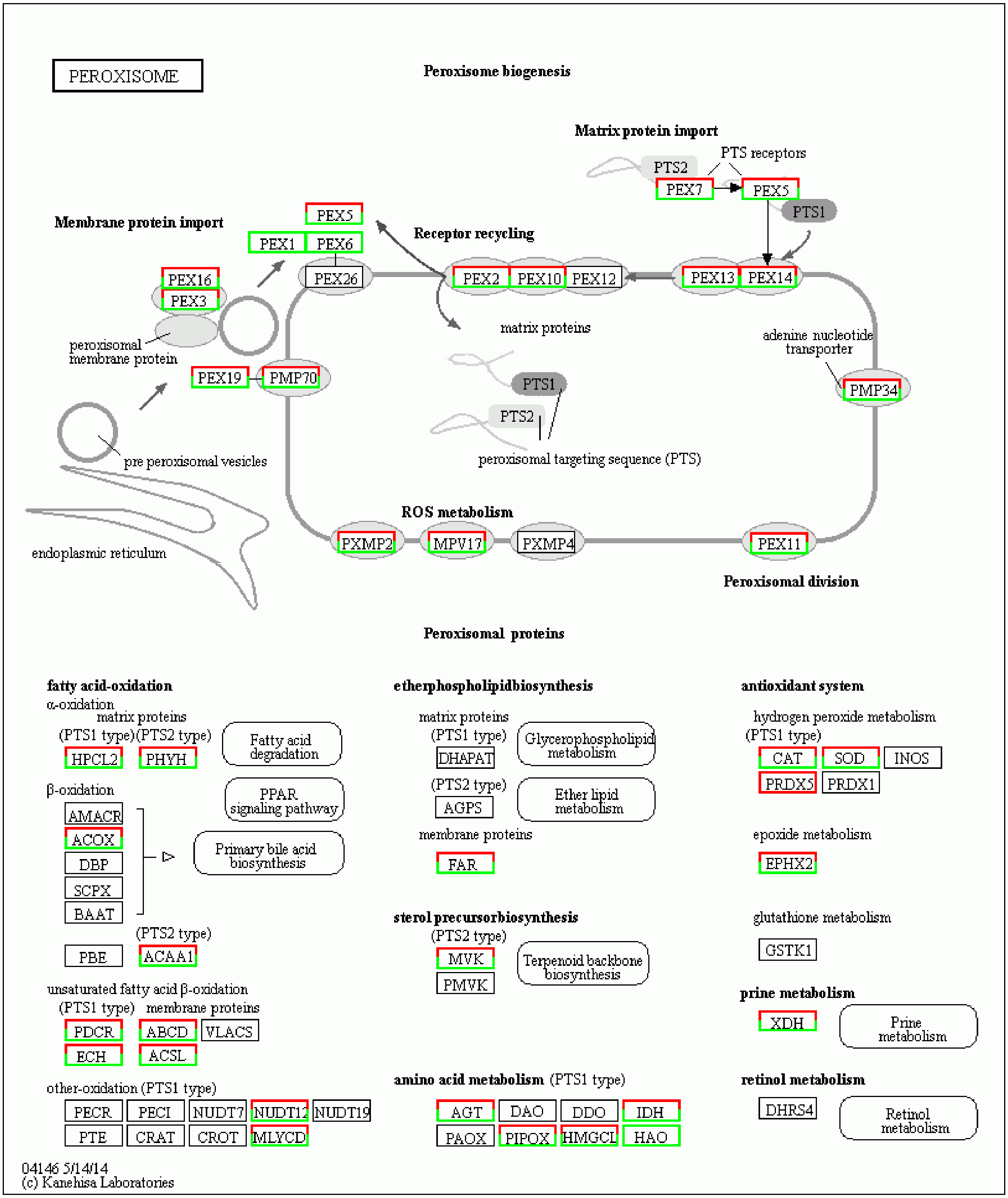

PEROXISOME
Peroxisome biogenesis
Matrix protein import
PTS receptors
PTS2
PEX7
PEX5
PTS1
Membrane protein import
PEX5
PEX1
PEX6
Receptor recycling
PEX16
PEX3
PEX26
PEX2
PEX10
PEX12
PEX13
PEX14
matrix proteins
peroxisomal membrane protein
adenine nucleotide transporter
PEX19
PMP70
PTS1
PMP34
PTS2
pre peroxisomal vesicles
peroxisomal targeting sequence (PTS)
ROS metabolism
endoplasmic reticulum
PXMP2
MPV17
PXMP4
PEX11
Peroxisomal division
Peroxisomal proteins
fatty acid-oxidation
α-oxidation
matrix proteins
(PTS1 type)(PTS2 type)
HPCL2
PHYH
Fatty acid degradation
β-oxidation
PPAR signaling pathway
AMACR
ACOX
DBP
SCPX
BAAT
Primary bile acid biosynthesis
(PTS2 type)
PBE
ACAA1
unsaturated fatty acid β-oxidation
(PTS1 type)
membrane proteins
PDCR
ABCD
VLACS
ECH
ACSL
other-oxidation (PTS1 type)
PECR
PECI
NUDT7
NUDT12
NUDT19
PTE
CRAT
CROT
MLYCD
etherphospholipidbiosynthesis
matrix proteins
(PTS1 type)
DHAPAT
Glycerophospholipid metabolism
(PTS2 type)
AGPS
Ether lipid metabolism
membrane proteins
FAR
sterol precursorbiosynthesis
(PTS2 type)
MVK
PMVK
Terpenoid backbone biosynthesis
amino acid metabolism (PTS1 type)
AGT
DAO
DDO
IDH
PAOX
PIPOX
HMGCL
HAO
antioxidant system
hydrogen peroxide metabolism
(PTS1 type)
CAT
SOD
INOS
PRDX5
PRDX1
epoxide metabolism
EPHX2
glutathione metabolism
GSTK1
prine metabolism
XDH
Prine metabolism
retinol metabolism
DHRS4
Retinol metabolism
04146 5/14/14
(c) Kanehisa Laboratories

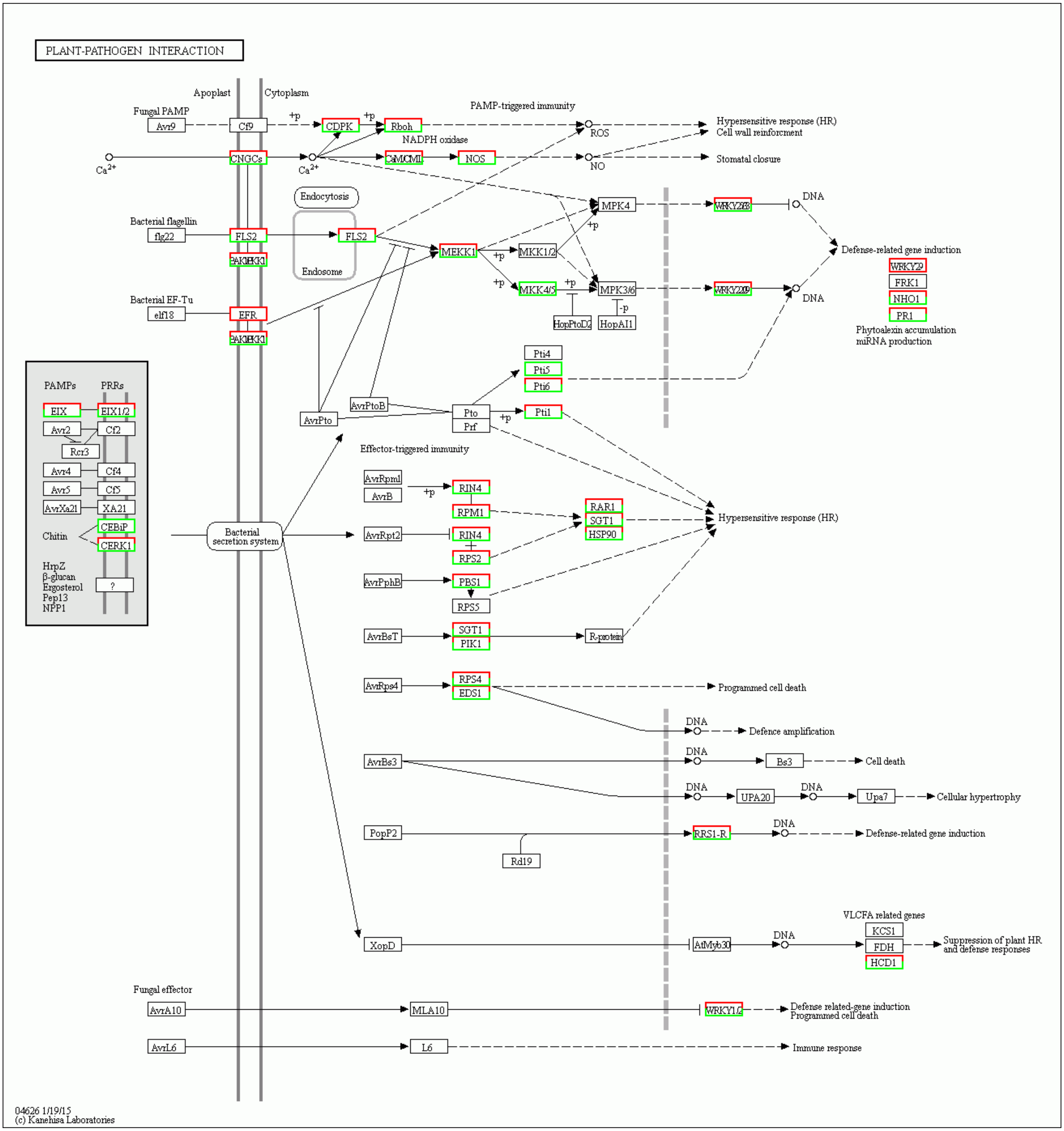
PLANT-PATHOGEN INTERACTION
Apoplast
Cytoplasm
Fungal PAMP
Avr9
Cf9
+p
CDPK
Rboh
PAMP-triggered immunity
ROS
Hypersensitive response (HR)
Cell wall reinforcment
NADPH oxidase
Ca2+
CNGCs
CaM/CML
NOS
NO
Stomatal closure
Endocytosis
Endosome
Bacterial flagellin
flg22
FLS2
BAK1/BKK1
MEKK1
MKK1/2
MPK4
WRKY25/33
DNA
Defense-related gene induction
WRKY29
FRK1
NHO1
PR1
Phytoalexin accumulation
miRNA production
MKK4/5
MPK3/6
WRKY22/29
-p
HopPtoD2
HopAI1
Bacterial EF-Tu
elf18
EFR
PAMPs
PRRs
EIX
EIX1/2
Avr2
Cf2
Rcr3
Avr4
Cf4
Avr5
Cf5
AvrXa21
XA21
CEBiP
CERK1
Chitin
HrpZ
β-glucan
Ergosterol
Pep13
NPP1
?
Pti4
Pti5
Pti6
AvrPtoB
AvrPto
Pto
Prf
Pti1
Effector-triggered immunity
AvrRpm1
AvrB
RIN4
RPM1
RAR1
SGT1
HSP90
Hypersensitive response (HR)
Bacterial secretion system
AvrRpt2
RPS2
AvrPphB
PBS1
RPS5
AvrBsT
PIK1
R-protein
AvrRps4
RPS4
EDS1
Programmed cell death
Defence amplification
AvrBs3
Bs3
Cell death
UPA20
Upa7
Cellular hypertrophy
PopP2
RRS1-R
Rd19
VLCFA related genes
KCS1
FDH
HCD1
XopD
AtMyb30
Suppression of plant HR and defense responses
Fungal effector
AvrA10
MLA10
WRKY1/2
Defense related-gene induction
Programmed cell death
AvrL6
L6
Immune response
04626 1/19/15
(c) Kanehisa Laboratories

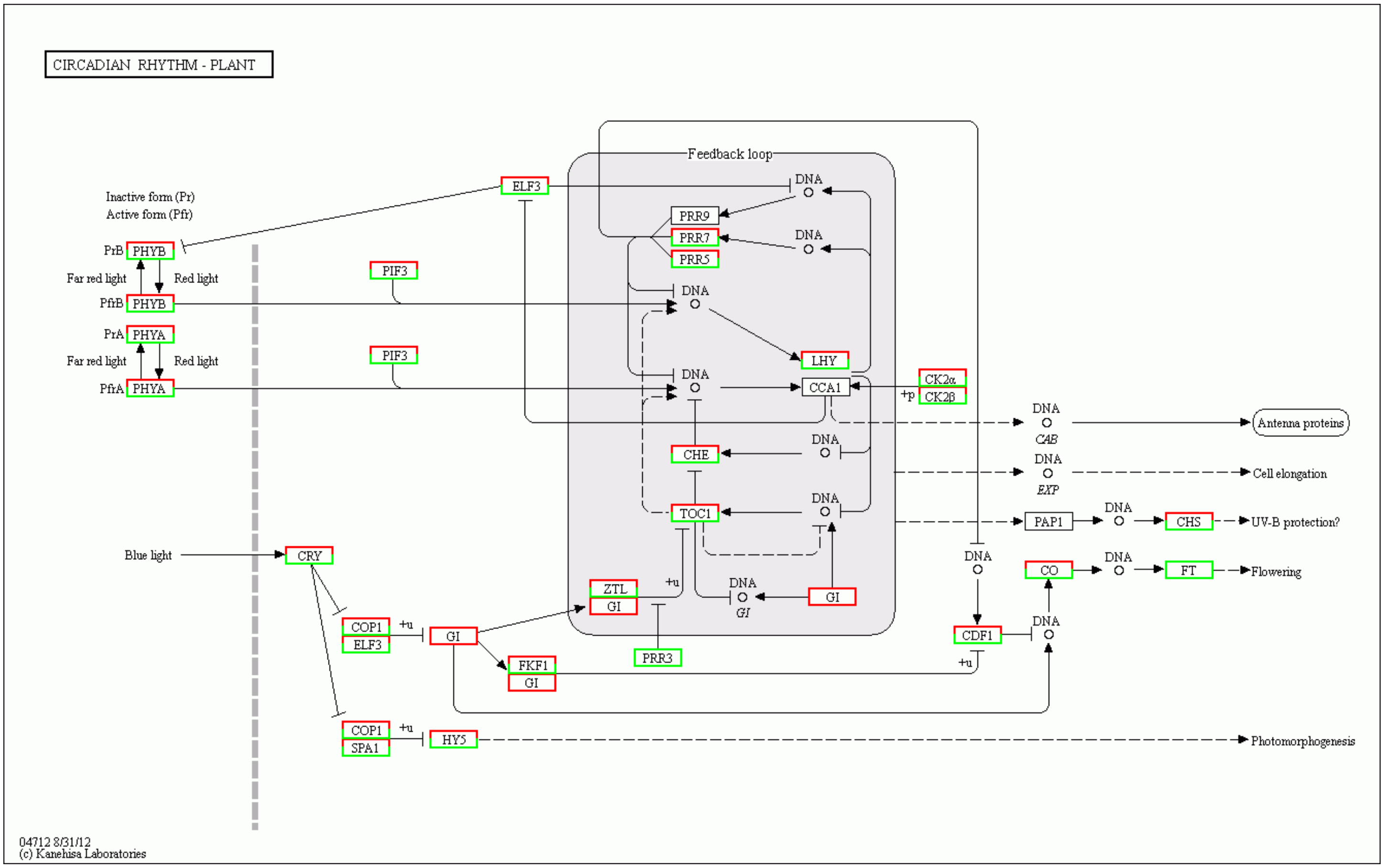

CIRCADIAN RHYTHM - PLANT
Feedback loop
Inactive form (Pr)
Active form (Pfr)
PrB
PHYB
Far red light
Red light
PfrB
PHYB
PrA
PHYA
Far red light
Red light
PfrA
PHYA
PIF3
PIF3
ELF3
DNA
PRR9
PRR7
PRR5
DNA
DNA
LHY
DNA
CCA1
CK2α
+p
CK2β
DNA
CAB
Antenna proteins
DNA
EXP
Cell elongation
CHE
DNA
TOC1
DNA
PAP1
DNA
CHS
UV-B protection?
DNA
CO
DNA
FT
Flowering
Blue light
CRY
ZTL
+u
GI
DNA
GI
GI
COP1
+u
ELF3
GI
FKF1
GI
PRR3
CDF1
DNA
+u
COP1
+u
SPA1
HY5
Photomorphogenesis
04712 8/31/12
(c) Kanehisa Laboratories

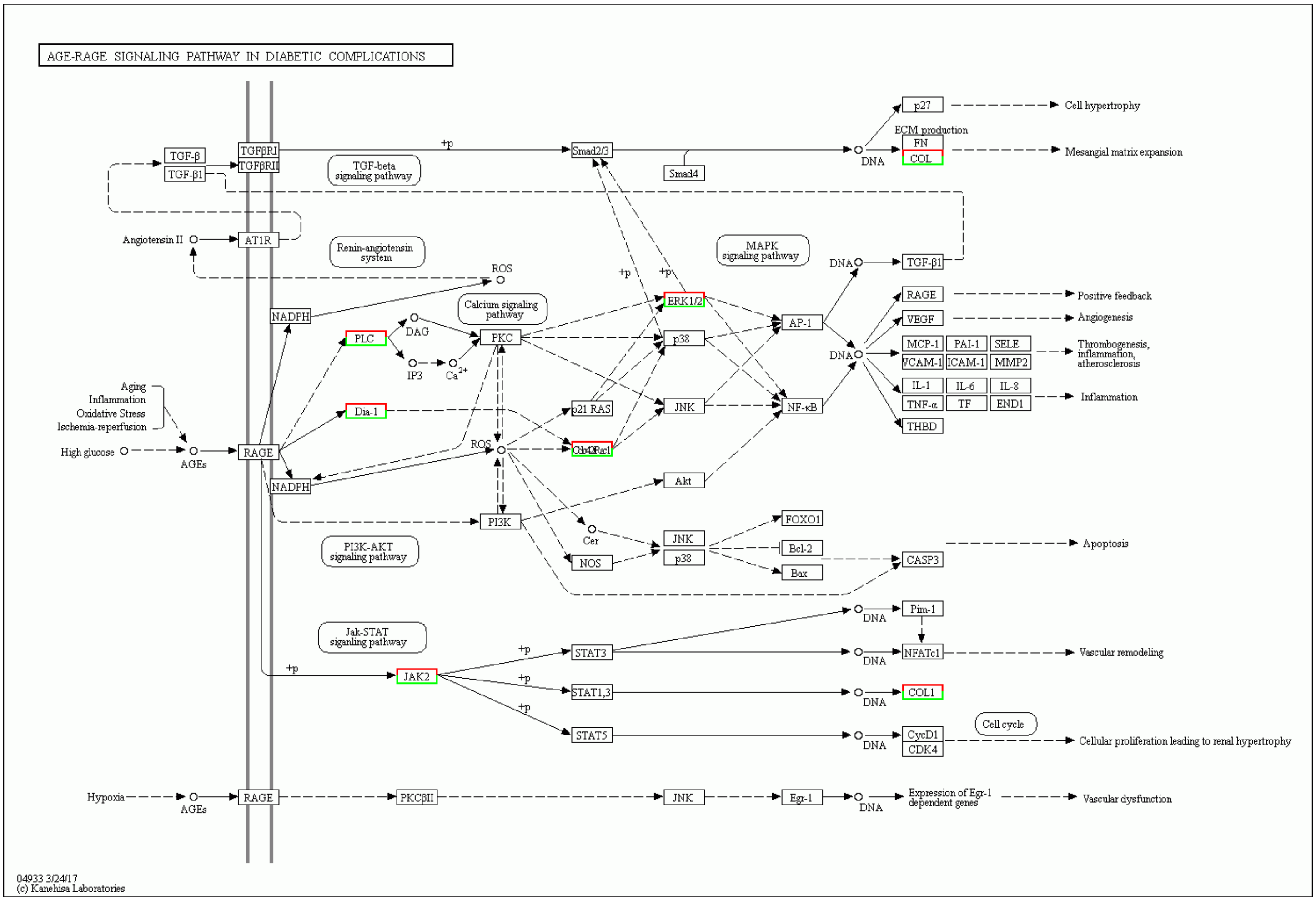

AGE-RAGE SIGNALING PATHWAY IN DIABETIC COMPLICATIONS
TGF-β
TGF-β1
TGFβRI
TGFβRII
+p
TGF-beta signaling pathway
Smad2/3
Smad4
DNA
p27
Cell hypertrophy
ECM production
FN
COL
Mesangial matrix expansion
Angiotensin II
AT1R
Renin-angiotensin system
ROS
MAPK signaling pathway
DNA
TGF-β1
+p
+p
ERK1/2
RAGE
Positive feedback
NADPH
Calcium signaling pathway
AP-1
VEGF
Angiogenesis
PLC
DAG
PKC
p38
DNA
MCP-1
PAI-1
SELE
VCAM-1
ICAM-1
MMP2
Thrombogenesis, inflammation, atherosclerosis
IP3
Ca2+
IL-1
IL-6
IL-8
Aging
Inflammation
Oxidative Stress
Ischemia-reperfusion
Dia-1
p21 RAS
JNK
NF-κB
TNF-α
TF
END1
Inflammation
THBD
High glucose
AGEs
RAGE
ROS
Cdc42/Rac1
NADPH
Akt
PI3K
Cer
JNK
FOXO1
Bcl-2
NOS
p38
Bax
CASP3
Apoptosis
PI3K-AKT signaling pathway
DNA
Pim-1
Jak-STAT signaling pathway
+p
STAT3
DNA
NFATc1
Vascular remodeling
+p
JAK2
+p
STAT1,3
DNA
COL1
+p
STAT5
DNA
CycD1
CDK4
Cell cycle
Cellular proliferation leading to renal hypertrophy
Hypoxia
AGEs
RAGE
PKCβII
JNK
Egr-1
DNA
Expression of Egr-1 dependent genes
Vascular dysfunction
04933 3/24/17
(c) Kanehisa Laboratories

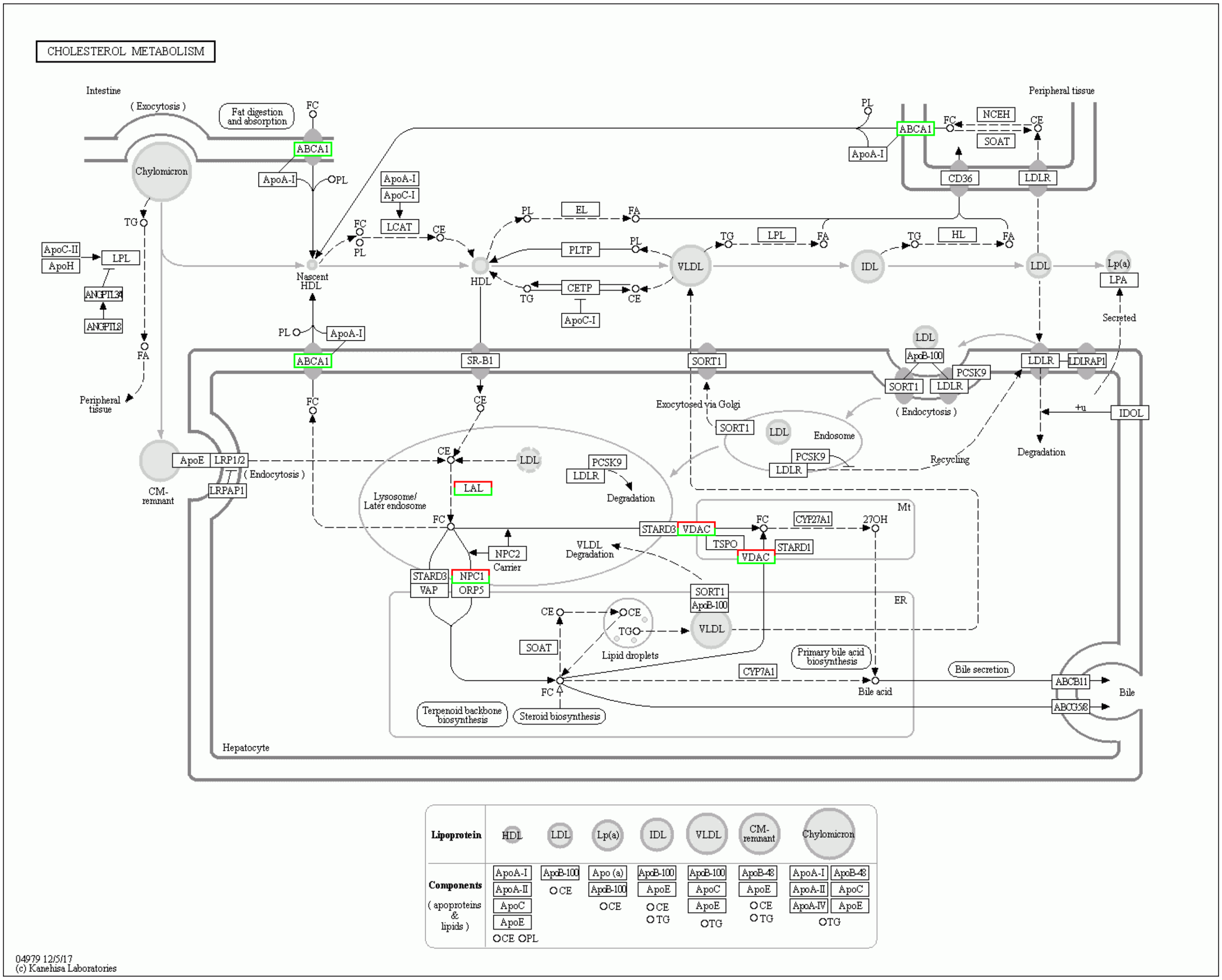
CHOLESTEROL METABOLISM
Intestine
(Exocytosis)
Fat digestion and absorption
Chylomicron
ABCA1
ApoA-I
PL
FC
TG
ApoC-II
ApoH
LPL
ANGPTL3/4
ANGPTL8
FA
Peripheral tissue
ApoA-I
ApoC-I
LCAT
CE
Nascent HDL
HDL
EL
PLTP
CETP
VLDL
IDL
LDL
Lp(a)
LPA
HL
Secreted
NCEH
SOAT
CD36
LDLR
ABCA1
SR-B1
SORT1
ApoB-100
PCSK9
LDLRAP1
(Endocytosis)
IDOL
+u
Degradation
Exocytosed via Golgi
Endosome
Recycling
CM-remnant
ApoE
LRP1/2
LRPAP1
Lysosome/ Later endosome
LAL
NPC2
Carrier
STARD3
NPC1
VAP
ORP5
VLDL Degradation
Mt
VDAC
TSPO
STARD1
CYP27A1
27OH
ER
Lipid droplets
Primary bile acid biosynthesis
CYP7A1
Bile acid
Bile secretion
ABCB11
ABCG5/8
Bile
Terpenoid backbone biosynthesis
Steroid biosynthesis
Hepatocyte
Lipoprotein
Components
(apoproteins & lipids)
ApoA-II
ApoC
Apo (a)
ApoB-48
ApoA-IV
04979 12/5/17
(c) Kanehisa Laboratories

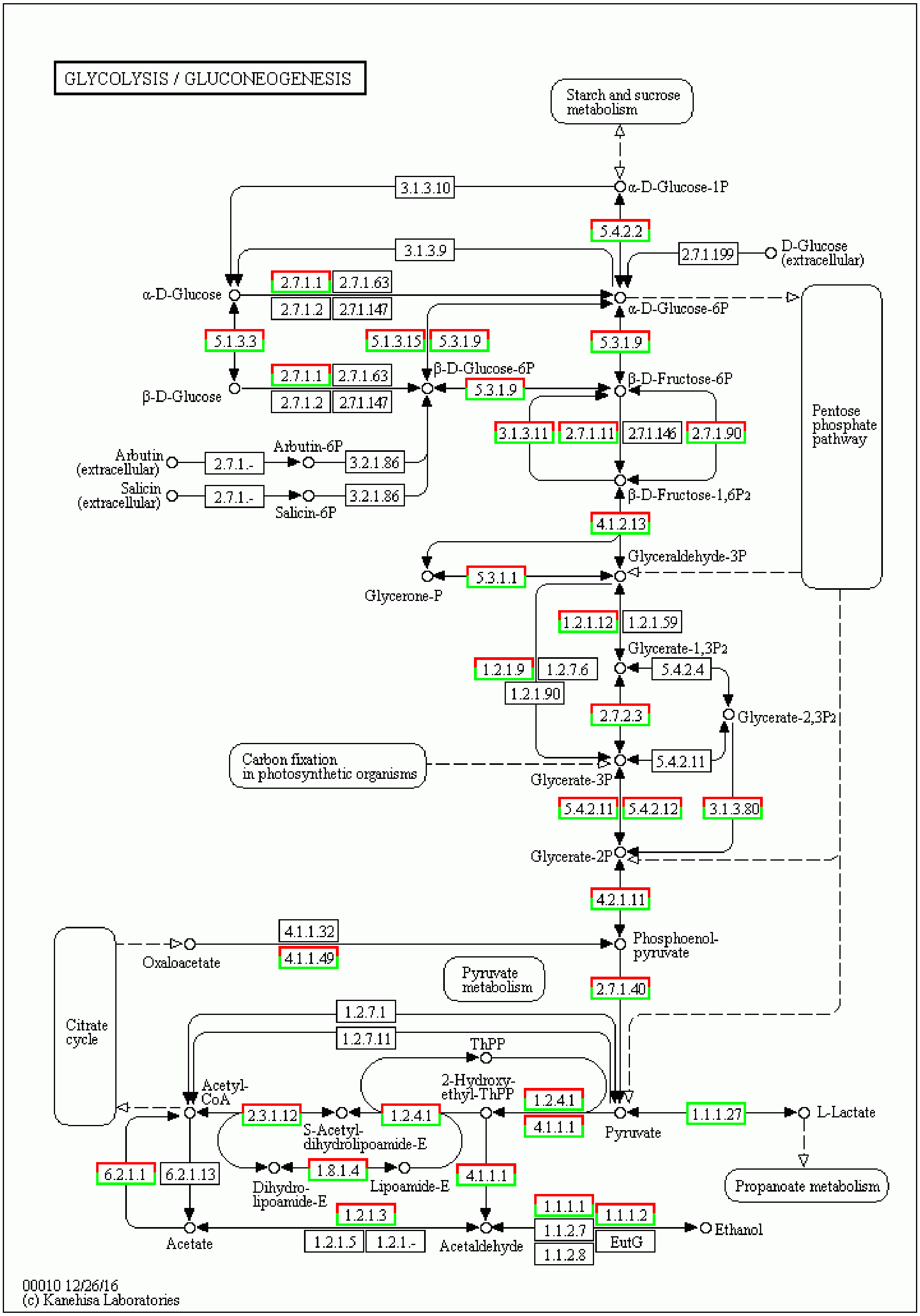
GLYCOLYSIS / GLUCONEOGENESIS
Starch and sucrose metabolism
3.1.3.10
α-D-Glucose-1P
5.4.2.2
3.1.3.9
2.7.1.199
D-Glucose (extracellular)
α-D-Glucose
2.7.1.1
2.7.1.63
2.7.1.2
2.7.1.147
α-D-Glucose-6P
5.1.3.3
5.1.3.15
5.3.1.9
β-D-Glucose-6P
β-D-Fructose-6P
β-D-Glucose
Pentose phosphate pathway
3.1.3.11
2.7.1.11
2.7.1.146
2.7.1.90
Arbutin (extracellular)
2.7.1.-
Arbutin-6P
3.2.1.86
Salicin (extracellular)
Salicin-6P
β-D-Fructose-1,6P2
4.1.2.13
Glyceraldehyde-3P
Glycerone-P
5.3.1.1
1.2.1.12
1.2.1.59
1.2.1.9
1.2.7.6
1.2.1.90
Glycerate-1,3P2
5.4.2.4
2.7.2.3
Glycerate-2,3P2
Carbon fixation in photosynthetic organisms
Glycerate-3P
5.4.2.11
5.4.2.12
3.1.3.80
Glycerate-2P
4.2.1.11
Citrate cycle
Oxaloacetate
4.1.1.32
4.1.1.49
Phosphoenol-pyruvate
Pyruvate metabolism
2.7.1.40
1.2.7.1
1.2.7.11
ThPP
Acetyl-CoA
2.3.1.12
2-Hydroxy-ethyl-ThPP
1.2.4.1
4.1.1.1
Pyruvate
1.1.1.27
L-Lactate
S-Acetyl-dihydrolipoamide-E
Dihydro-lipoamide-E
1.8.1.4
Lipoamide-E
6.2.1.1
6.2.1.13
Propanoate metabolism
1.2.1.3
1.1.1.1
1.1.1.2
Acetate
1.2.1.5
1.2.1.-
Acetaldehyde
1.1.2.7
EutG
1.1.2.8
Ethanol
00010 12/26/16
(c) Kanehisa Laboratories

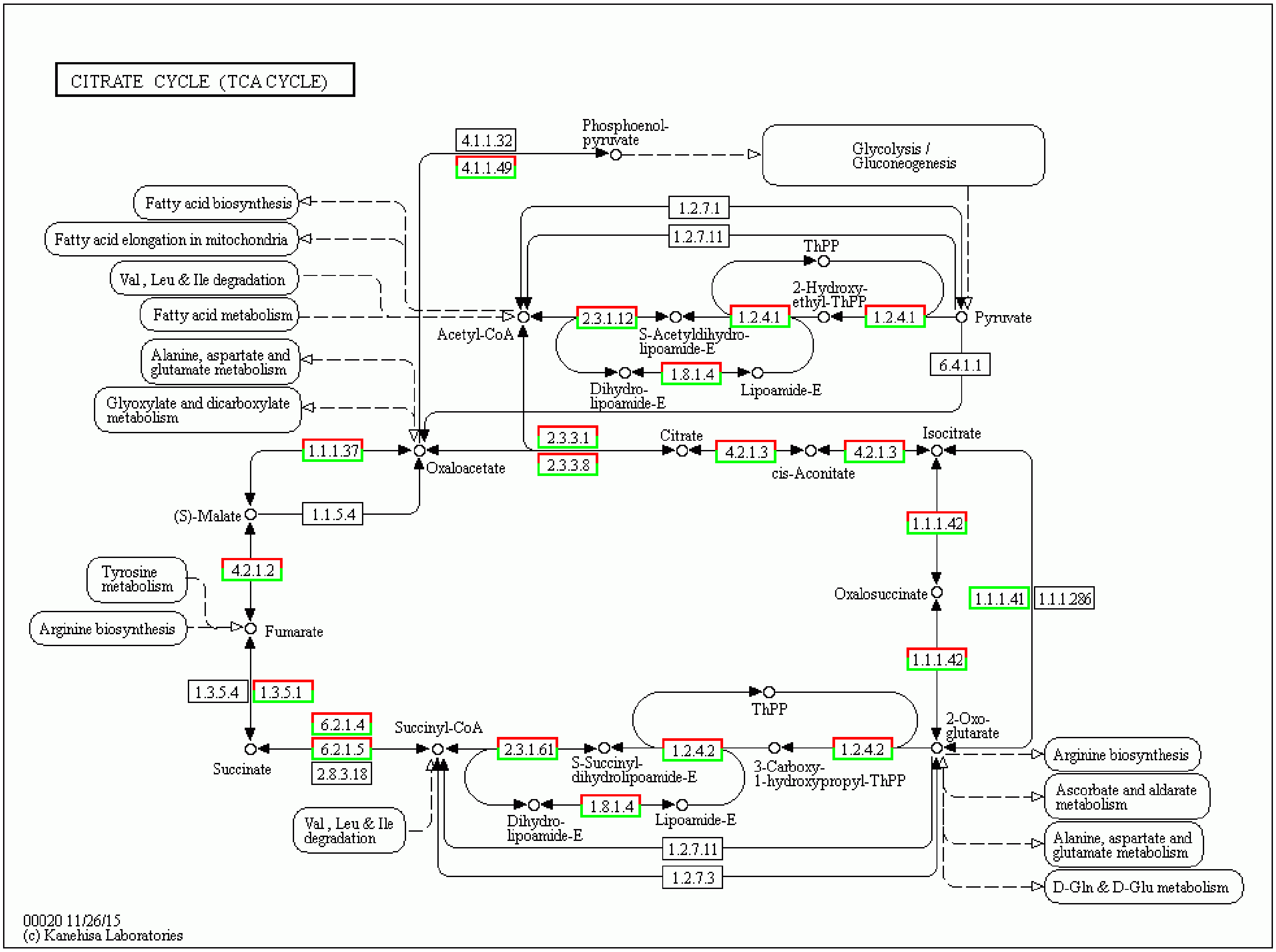
CITRATE CYCLE (TCA CYCLE)
4.1.1.32
4.1.1.49
Phosphoenol-pyruvate
Glycolysis / Gluconeogenesis
Fatty acid biosynthesis
Fatty acid elongation in mitochondria
Val, Leu & Ile degradation
Fatty acid metabolism
Alanine, aspartate and glutamate metabolism
Glyoxylate and dicarboxylate metabolism
1.2.7.1
1.2.7.11
ThPP
2-Hydroxy-ethyl-ThPP
Acetyl-CoA
2.3.1.12
1.2.4.1
1.2.4.1
Pyruvate
S-Acetyldihydro-lipoamide-E
1.8.1.4
Dihydro-lipoamide-E
Lipoamide-E
6.4.1.1
1.1.1.37
Oxaloacetate
2.3.3.1
2.3.3.8
Citrate
4.2.1.3
cis-Aconitate
4.2.1.3
Isocitrate
(S)-Malate
1.1.5.4
1.1.1.42
4.2.1.2
Tyrosine metabolism
Arginine biosynthesis
Fumarate
Oxalosuccinate
1.1.1.41
1.1.1.286
1.1.1.42
1.3.5.4
1.3.5.1
6.2.1.4
6.2.1.5
2.8.3.18
Succinate
Succinyl-CoA
2.3.1.61
S-Succinyl-dihydrolipoamide-E
1.2.4.2
3-Carboxy-1-hydroxypropyl-ThPP
1.2.4.2
2-Oxo-glutarate
Arginine biosynthesis
Ascorbate and aldarate metabolism
Alanine, aspartate and glutamate metabolism
D-Gln & D-Glu metabolism
Val, Leu & Ile degradation
Dihydro-lipoamide-E
1.8.1.4
Lipoamide-E
1.2.7.11
1.2.7.3
00020 11/26/15
(c) Kanehisa Laboratories

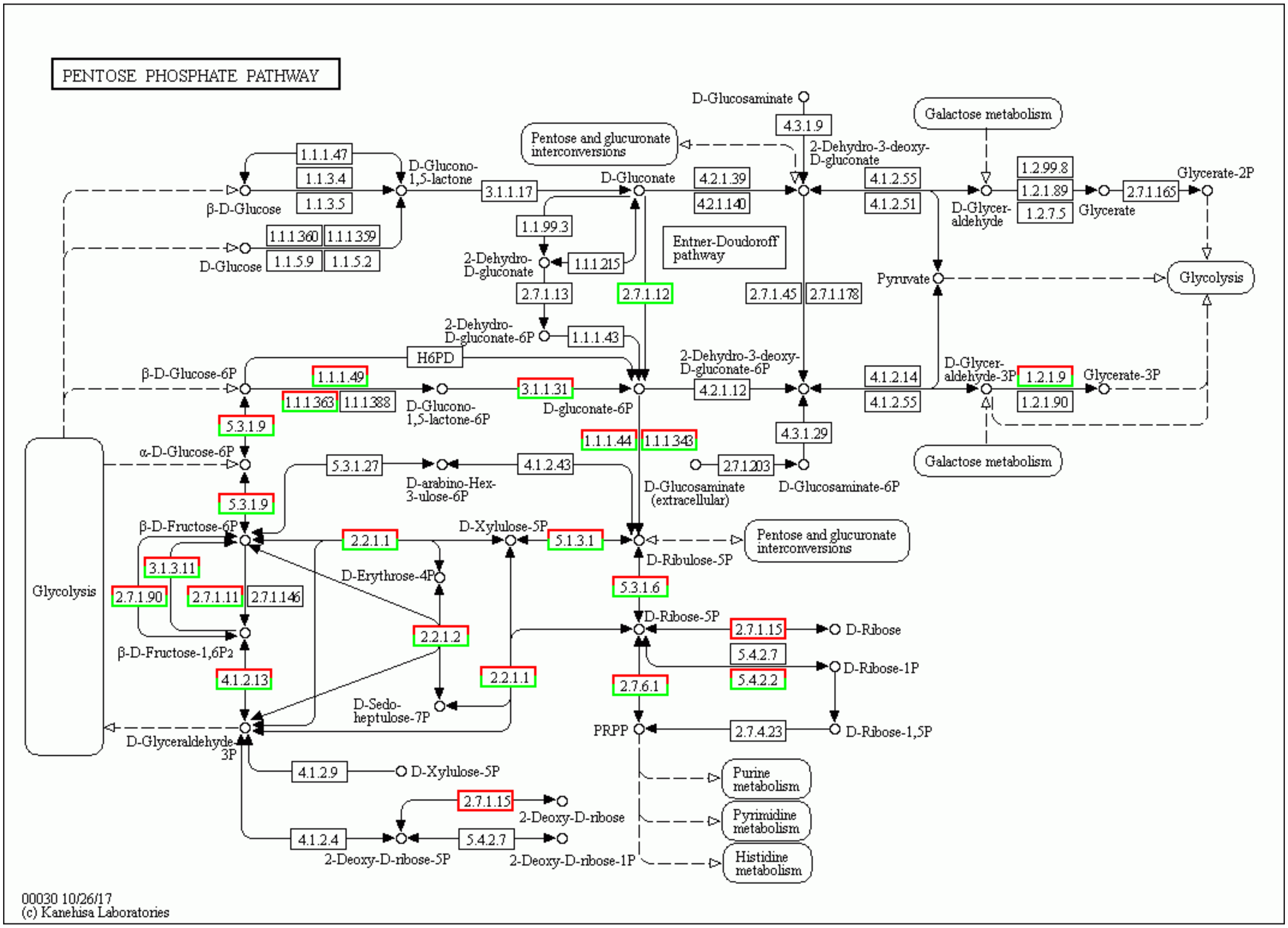
PENTOSE PHOSPHATE PATHWAY
D-Glucosaminate
4.3.1.9
Galactose metabolism
Pentose and glucuronate interconversions
2-Dehydro-3-deoxy-D-gluconate
1.1.1.47
1.1.3.4
1.1.3.5
D-Glucono-1,5-lactone
3.1.1.17
D-Gluconate
4.2.1.39
4.2.1.140
4.1.2.55
4.1.2.51
1.2.99.8
1.2.1.89
1.2.7.5
2.7.1.165
Glycerate-2P
Glycerate
D-Glyceraldehyde
β-D-Glucose
1.1.1.360
1.1.1.359
D-Glucose
1.1.5.9
1.1.5.2
1.1.99.3
2-Dehydro-D-gluconate
1.1.1.215
Entner-Doudoroff pathway
Pyruvate
Glycolysis
2.7.1.13
2.7.1.12
2.7.1.45
2.7.1.178
2-Dehydro-D-gluconate-6P
1.1.1.43
H6PD
β-D-Glucose-6P
1.1.1.49
1.1.1.363
1.1.1.388
D-Glucono-1,5-lactone-6P
3.1.1.31
D-gluconate-6P
2-Dehydro-3-deoxy-D-gluconate-6P
4.2.1.12
4.1.2.14
4.1.2.55
D-Glyceraldehyde-3P
1.2.1.9
1.2.1.90
Glycerate-3P
5.3.1.9
1.1.1.44
1.1.1.343
4.3.1.29
α-D-Glucose-6P
5.3.1.27
4.1.2.43
D-arabino-Hex-3-ulose-6P
2.7.1.203
D-Glucosaminate (extracellular)
D-Glucosaminate-6P
Galactose metabolism
Glycolysis
5.3.1.9
β-D-Fructose-6P
2.2.1.1
D-Xylulose-5P
5.1.3.1
Pentose and glucuronate interconversions
D-Ribulose-5P
3.1.3.11
D-Erythrose-4P
5.3.1.6
2.7.1.90
2.7.1.11
2.7.1.146
D-Ribose-5P
2.7.1.15
D-Ribose
2.2.1.2
5.4.2.7
β-D-Fructose-1,6P2
D-Ribose-1P
4.1.2.13
2.2.1.1
5.4.2.2
2.7.6.1
D-Sedo-heptulose-7P
PRPP
2.7.4.23
D-Ribose-1,5P
D-Glyceraldehyde-3P
4.1.2.9
D-Xylulose-5P
Purine metabolism
2.7.1.15
2-Deoxy-D-ribose
Pyrimidine metabolism
4.1.2.4
5.4.2.7
2-Deoxy-D-ribose-5P
2-Deoxy-D-ribose-1P
Histidine metabolism
00030 10/26/17
(c) Kanehisa Laboratories

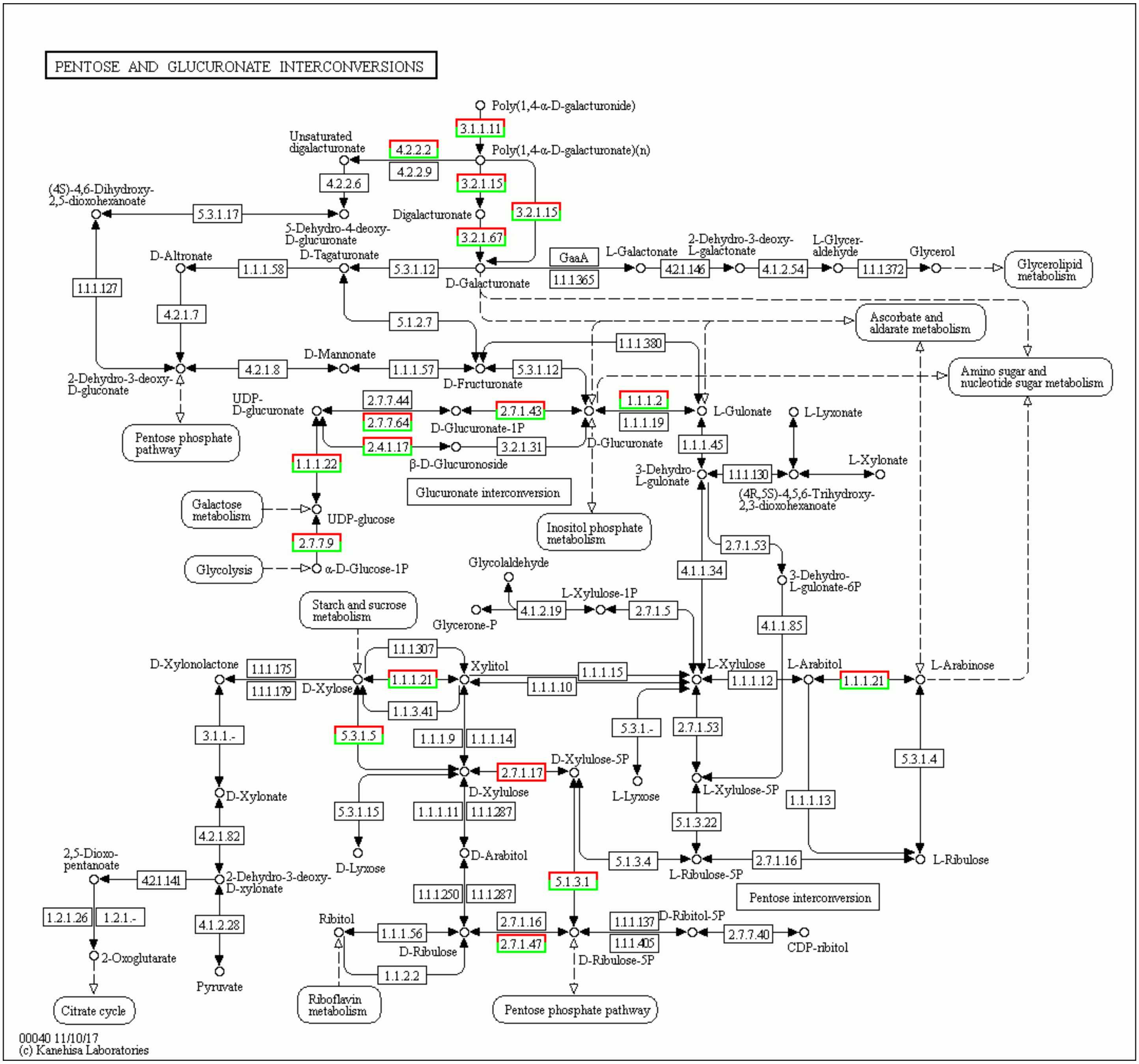
PENTOSE AND GLUCURONATE INTERCONVERSIONS
Poly(1,4-α-D-galacturonide)
3.1.1.11
Poly(1,4-α-D-galacturonate)(n)
Unsaturated digalacturonate
4.2.2.2
4.2.2.9
4.2.2.6
3.2.1.15
3.2.1.15
(4S)-4,6-Dihydroxy-2,5-dioxohexanoate
5.3.1.17
5-Dehydro-4-deoxy-D-glucuronate
Digalacturonate
3.2.1.67
D-Altronate
1.1.1.58
D-Tagaturonate
5.3.1.12
D-Galacturonate
GaaA
1.1.1.365
L-Galactonate
4.2.1.146
2-Dehydro-3-deoxy-L-galactonate
4.1.2.54
L-Glyceraldehyde
1.1.1.372
Glycerol
Glycerolipid metabolism
1.1.1.127
4.2.1.7
5.1.2.7
Ascorbate and aldarate metabolism
1.1.1.380
D-Mannonate
4.2.1.8
1.1.1.57
5.3.1.12
D-Fructuronate
Amino sugar and nucleotide sugar metabolism
2-Dehydro-3-deoxy-D-gluconate
UDP-D-glucuronate
2.7.7.44
2.7.7.64
2.7.1.43
1.1.1.2
1.1.1.19
L-Gulonate
L-Lyxonate
D-Glucuronate-1P
2.4.1.17
3.2.1.31
D-Glucuronate
1.1.1.45
Pentose phosphate pathway
β-D-Glucuronoside
1.1.1.22
L-Xylonate
3-Dehydro-L-gulonate
1.1.1.130
(4R,5S)-4,5,6-Trihydroxy-2,3-dioxohexanoate
Glucuronate interconversion
Galactose metabolism
UDP-glucose
Inositol phosphate metabolism
2.7.1.53
2.7.7.9
Glycolysis
α-D-Glucose-1P
Glycolaldehyde
4.1.1.34
3-Dehydro-L-gulonate-6P
L-Xylulose-1P
4.1.2.19
2.7.1.5
Starch and sucrose metabolism
Glycerone-P
4.1.1.85
1.1.1.307
D-Xylonolactone
1.1.1.175
1.1.1.179
Xylitol
1.1.1.15
L-Xylulose
L-Arabitol
L-Arabinose
D-Xylose
1.1.1.21
1.1.1.10
1.1.1.12
1.1.1.21
1.1.3.41
3.1.1.-
5.3.1.5
1.1.1.9
1.1.1.14
5.3.1.-
2.7.1.53
5.3.1.4
D-Xylulose-5P
2.7.1.17
D-Xylulose
L-Lyxose
L-Xylulose-5P
D-Xylonate
1.1.1.13
5.3.1.15
1.1.1.11
1.1.1.287
5.1.3.22
4.2.1.82
D-Arabitol
2,5-Dioxopentanoate
D-Lyxose
5.1.3.4
2.7.1.16
L-Ribulose
4.2.1.141
2-Dehydro-3-deoxy-D-xylonate
L-Ribulose-5P
5.1.3.1
1.1.1.250
1.1.1.287
Pentose interconversion
1.2.1.26
1.2.1.-
4.1.2.28
Ribitol
2.7.1.16
1.1.1.137
D-Ribitol-5P
1.1.1.56
2.7.1.47
1.1.1.405
2.7.7.40
CDP-ribitol
D-Ribulose
D-Ribulose-5P
2-Oxoglutarate
Pyruvate
1.1.2.2
Citrate cycle
Riboflavin metabolism
Pentose phosphate pathway
00040 11/10/17
(c) Kanehisa Laboratories

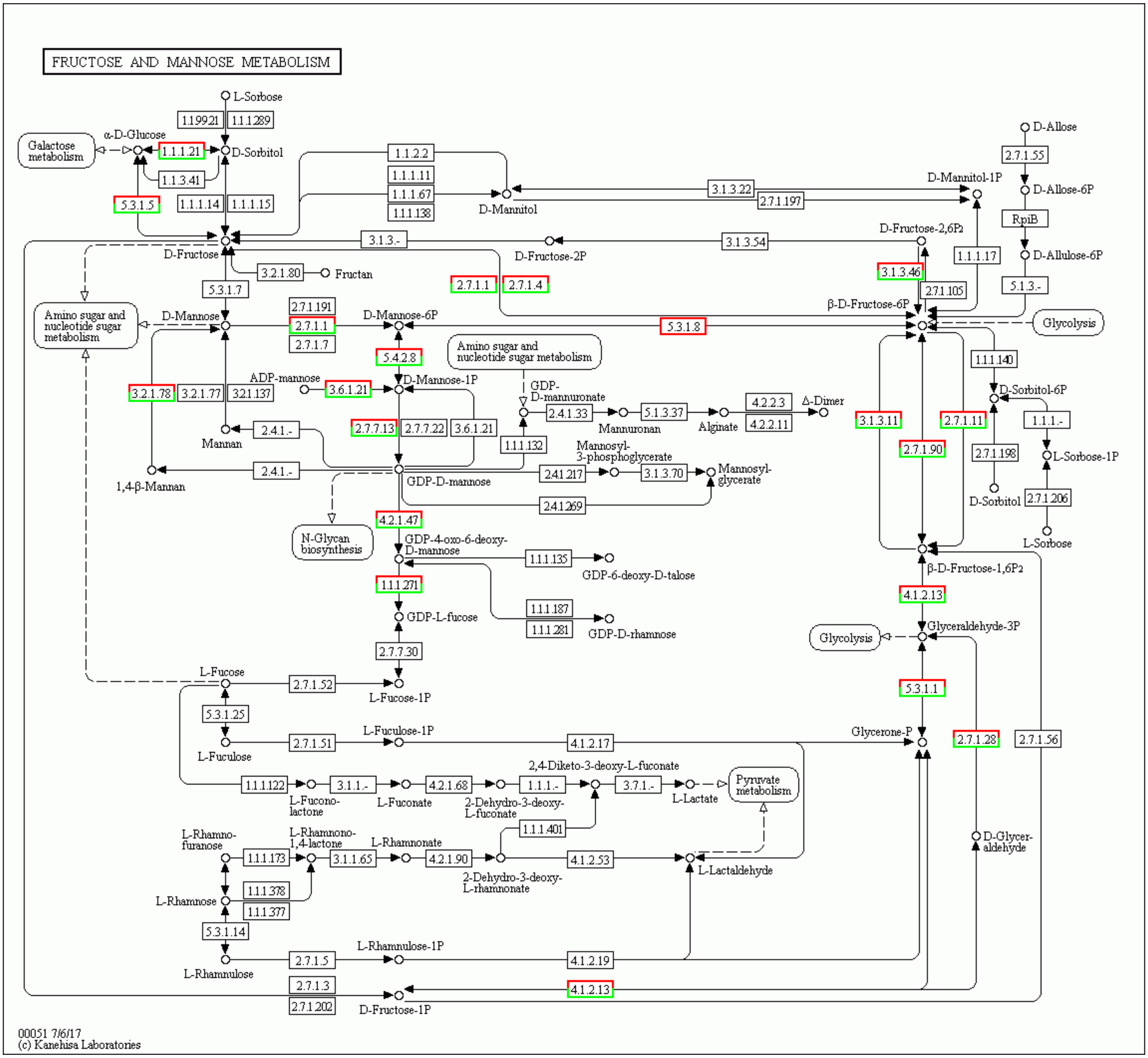
FRUCTOSE AND MANNOSE METABOLISM
L-Sorbose
1.1.99.21
1.1.1.289
α-D-Glucose
Galactose metabolism
1.1.1.21
D-Sorbitol
1.1.3.41
5.3.1.5
1.1.1.14
1.1.1.15
1.1.2.2
1.1.1.11
1.1.1.67
1.1.1.138
D-Mannitol
3.1.3.22
2.7.1.197
D-Mannitol-1P
D-Allose
2.7.1.55
D-Allose-6P
RpiB
D-Allulose-6P
5.1.3.-
D-Fructose
3.1.3.-
D-Fructose-2P
3.1.3.54
D-Fructose-2,6P2
3.1.3.46
2.7.1.105
1.1.1.17
3.2.1.80
Fructan
5.3.1.7
2.7.1.1
2.7.1.4
β-D-Fructose-6P
Glycolysis
Amino sugar and nucleotide sugar metabolism
D-Mannose
2.7.1.191
2.7.1.1
2.7.1.7
D-Mannose-6P
5.3.1.8
5.4.2.8
Amino sugar and nucleotide sugar metabolism
1.1.1.140
D-Sorbitol-6P
3.2.1.78
3.2.1.77
3.2.1.137
ADP-mannose
3.6.1.21
D-Mannose-1P
GDP-D-mannuronate
2.4.1.33
Mannuronan
5.1.3.37
Alginate
4.2.2.3
4.2.2.11
Δ-Dimer
3.1.3.11
2.7.1.11
1.1.1.-
Mannan
2.4.1.-
2.7.7.13
2.7.7.22
3.6.1.21
1.1.1.132
Mannosyl-3-phosphoglycerate
2.7.1.90
2.7.1.198
L-Sorbose-1P
1,4-β-Mannan
2.4.1.-
GDP-D-mannose
2.4.1.217
3.1.3.70
Mannosyl-glycerate
D-Sorbitol
2.7.1.206
2.4.1.269
N-Glycan biosynthesis
4.2.1.47
GDP-4-oxo-6-deoxy-D-mannose
1.1.1.135
GDP-6-deoxy-D-talose
L-Sorbose
β-D-Fructose-1,6P2
1.1.1.271
4.1.2.13
1.1.1.187
1.1.1.281
GDP-L-fucose
GDP-D-rhamnose
Glycolysis
Glyceraldehyde-3P
2.7.7.30
L-Fucose
2.7.1.52
L-Fucose-1P
5.3.1.1
5.3.1.25
L-Fuculose-1P
2.7.1.51
4.1.2.17
Glycerone-P
2.7.1.28
2.7.1.56
L-Fuculose
2,4-Diketo-3-deoxy-L-fuconate
1.1.1.122
3.1.1.-
4.2.1.68
1.1.1.-
3.7.1.-
Pyruvate metabolism
L-Fucono-lactone
L-Fuconate
2-Dehydro-3-deoxy-L-fuconate
L-Lactate
1.1.1.401
L-Rhamno-furanose
L-Rhamnono-1,4-lactone
L-Rhamnonate
1.1.1.173
3.1.1.65
4.2.1.90
4.1.2.53
D-Glycer-aldehyde
2-Dehydro-3-deoxy-L-rhamnonate
L-Lactaldehyde
1.1.1.378
L-Rhamnose
1.1.1.377
5.3.1.14
L-Rhamnulose-1P
2.7.1.5
4.1.2.19
L-Rhamnulose
2.7.1.3
4.1.2.13
2.7.1.202
D-Fructose-1P
00051 7/6/17
(c) Kanehisa Laboratories

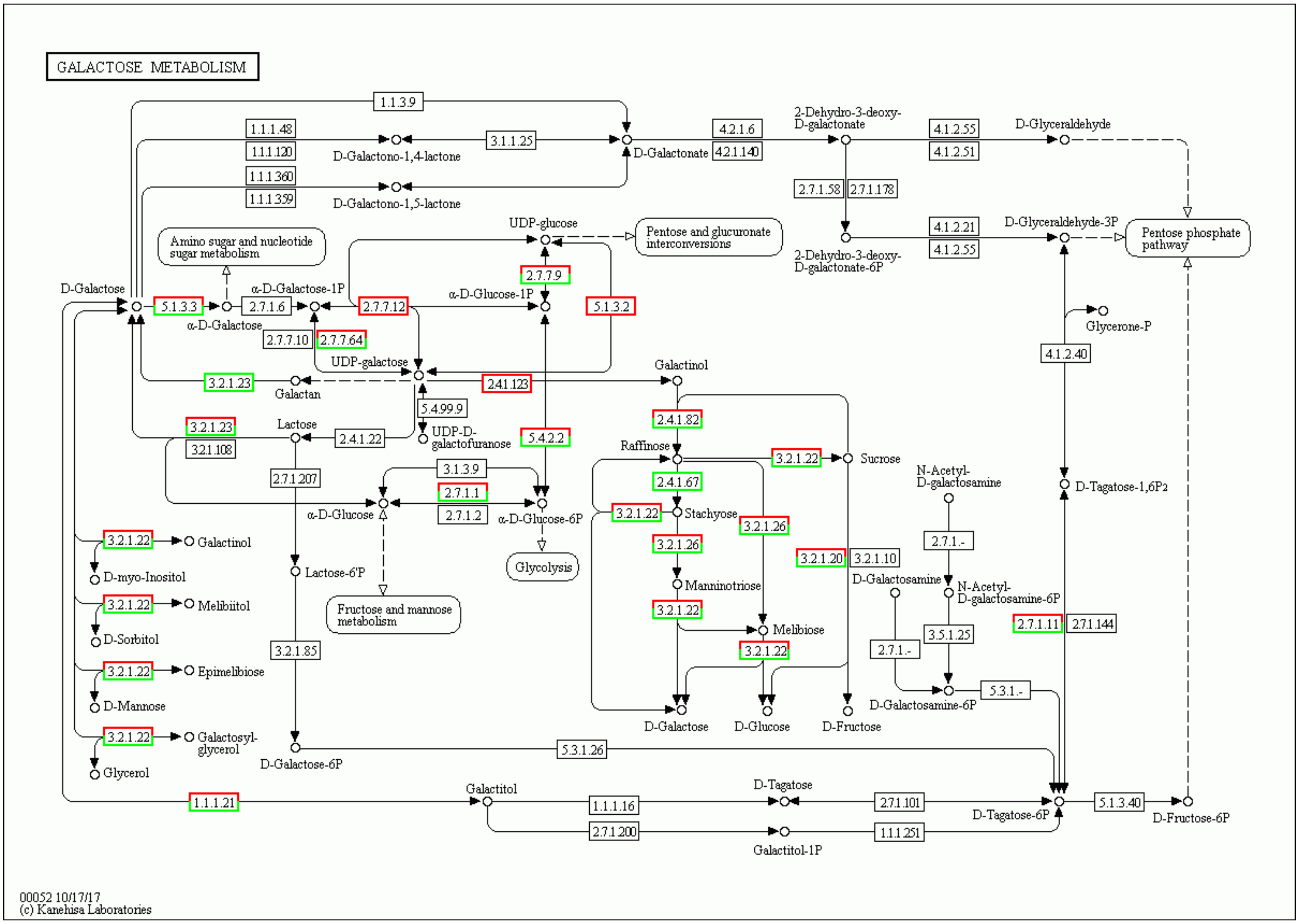
GALACTOSE METABOLISM
1.1.3.9
1.1.1.48
1.1.1.120
3.1.1.25
D-Galactono-1,4-lactone
1.1.1.360
1.1.1.359
D-Galactono-1,5-lactone
4.2.1.6
4.2.1.140
D-Galactonate
2-Dehydro-3-deoxy-D-galactonate
4.1.2.55
4.1.2.51
D-Glyceraldehyde
2.7.1.58
2.7.1.178
2-Dehydro-3-deoxy-D-galactonate-6P
4.1.2.21
4.1.2.55
D-Glyceraldehyde-3P
Pentose phosphate pathway
Amino sugar and nucleotide sugar metabolism
UDP-glucose
Pentose and glucuronate interconversions
2.7.7.9
D-Galactose
5.1.3.3
α-D-Galactose
α-D-Galactose-1P
2.7.1.6
2.7.7.12
α-D-Glucose-1P
5.1.3.2
2.7.7.10
2.7.7.64
UDP-galactose
3.2.1.23
Galactan
2.4.1.123
Galactinol
Glycerone-P
4.1.2.40
5.4.99.9
UDP-D-galactofuranose
3.2.1.23
3.2.1.108
Lactose
2.4.1.22
5.4.2.2
2.4.1.82
Raffinose
3.2.1.22
Sucrose
2.7.1.207
3.1.3.9
2.7.1.1
2.7.1.2
α-D-Glucose
α-D-Glucose-6P
2.4.1.67
N-Acetyl-D-galactosamine
D-Tagatose-1,6P2
3.2.1.22
Stachyose
3.2.1.26
Galactinol
3.2.1.22
D-myo-Inositol
Lactose-6'P
Glycolysis
3.2.1.26
2.7.1.-
3.2.1.20
3.2.1.10
Manninotriose
D-Galactosamine
N-Acetyl-D-galactosamine-6P
3.2.1.22
Melibiitol
Fructose and mannose metabolism
3.2.1.22
Melibiose
2.7.1.11
2.7.1.144
D-Sorbitol
3.5.1.25
3.2.1.22
3.2.1.85
2.7.1.-
3.2.1.22
Epimelibiose
5.3.1.-
D-Mannose
D-Galactose
D-Glucose
D-Fructose
D-Galactosamine-6P
3.2.1.22
Galactosyl-glycerol
5.3.1.26
D-Galactose-6P
Glycerol
1.1.1.21
Galactitol
1.1.1.16
D-Tagatose
2.7.1.101
5.1.3.40
D-Tagatose-6P
D-Fructose-6P
2.7.1.200
1.1.1.251
Galactitol-1P
00052 10/17/17
(c) Kanehisa Laboratories

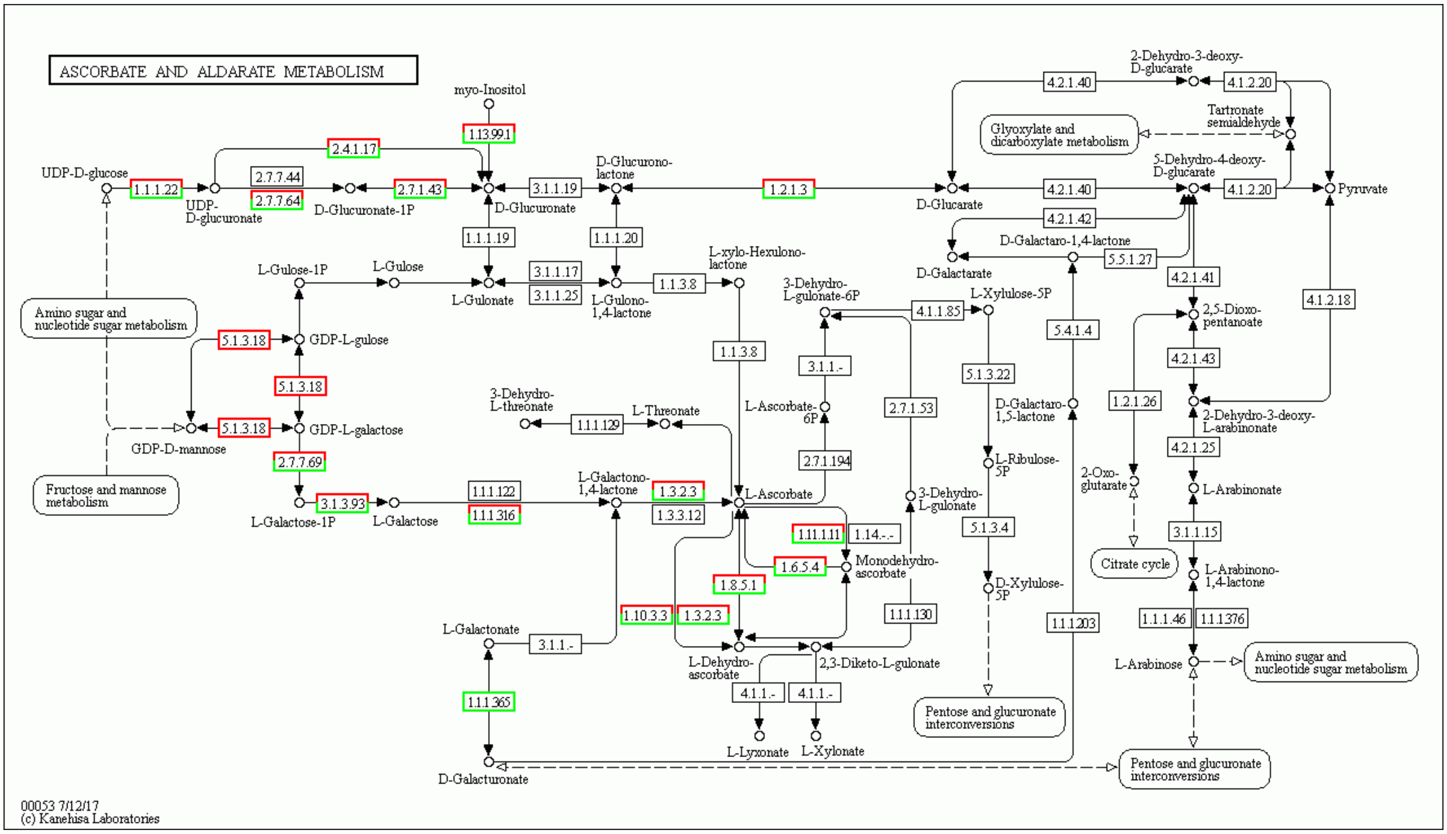
ASCORBATE AND ALDARATE METABOLISM
2-Dehydro-3-deoxy-D-glucarate
4.2.1.40
4.1.2.20
myo-Inositol
Tartronate semialdehyde
Glyoxylate and dicarboxylate metabolism
1.13.99.1
2.4.1.17
5-Dehydro-4-deoxy-D-glucarate
UDP-D-glucose
1.1.1.22
2.7.7.44
D-Glucurono-lactone
3.1.1.19
1.2.1.3
4.2.1.40
4.1.2.20
Pyruvate
UDP-D-glucuronate
2.7.7.64
2.7.1.43
D-Glucuronate-1P
D-Glucuronate
D-Glucarate
4.2.1.42
D-Galactaro-1,4-lactone
1.1.1.19
1.1.1.20
L-xylo-Hexulono-lactone
5.5.1.27
D-Galactarate
L-Gulose-1P
L-Gulose
3.1.1.17
3.1.1.25
1.1.3.8
3-Dehydro-L-gulonate-6P
L-Xylulose-5P
4.2.1.41
L-Gulonate
L-Gulono-1,4-lactone
4.1.1.85
2,5-Dioxo-pentanoate
4.1.2.18
Amino sugar and nucleotide sugar metabolism
5.4.1.4
5.1.3.18
GDP-L-gulose
1.1.3.8
3.1.1.-
4.2.1.43
5.1.3.22
5.1.3.18
3-Dehydro-L-threonate
L-Threonate
L-Ascorbate-6P
2.7.1.53
D-Galactaro-1,5-lactone
1.2.1.26
2-Dehydro-3-deoxy-L-arabinonate
5.1.3.18
GDP-L-galactose
1.1.1.129
GDP-D-mannose
2.7.7.69
2.7.1.194
L-Ribulose-5P
4.2.1.25
Fructose and mannose metabolism
L-Galactono-1,4-lactone
1.3.2.3
3-Dehydro-L-gulonate
2-Oxo-glutarate
L-Arabinonate
3.1.3.93
1.1.1.122
1.1.1.316
1.3.3.12
L-Ascorbate
L-Galactose-1P
L-Galactose
1.11.1.11
1.14.-.-
5.1.3.4
3.1.1.15
1.6.5.4
Monodehydro-ascorbate
Citrate cycle
L-Arabinono-1,4-lactone
1.8.5.1
D-Xylulose-5P
1.10.3.3
1.3.2.3
1.1.1.130
1.1.1.203
1.1.1.46
1.1.1.376
L-Galactonate
3.1.1.-
L-Dehydro-ascorbate
2,3-Diketo-L-gulonate
L-Arabinose
Amino sugar and nucleotide sugar metabolism
4.1.1.-
4.1.1.-
1.1.1.365
Pentose and glucuronate interconversions
L-Lyxonate
L-Xylonate
D-Galacturonate
Pentose and glucuronate interconversions
00053 7/12/17
(c) Kanehisa Laboratories

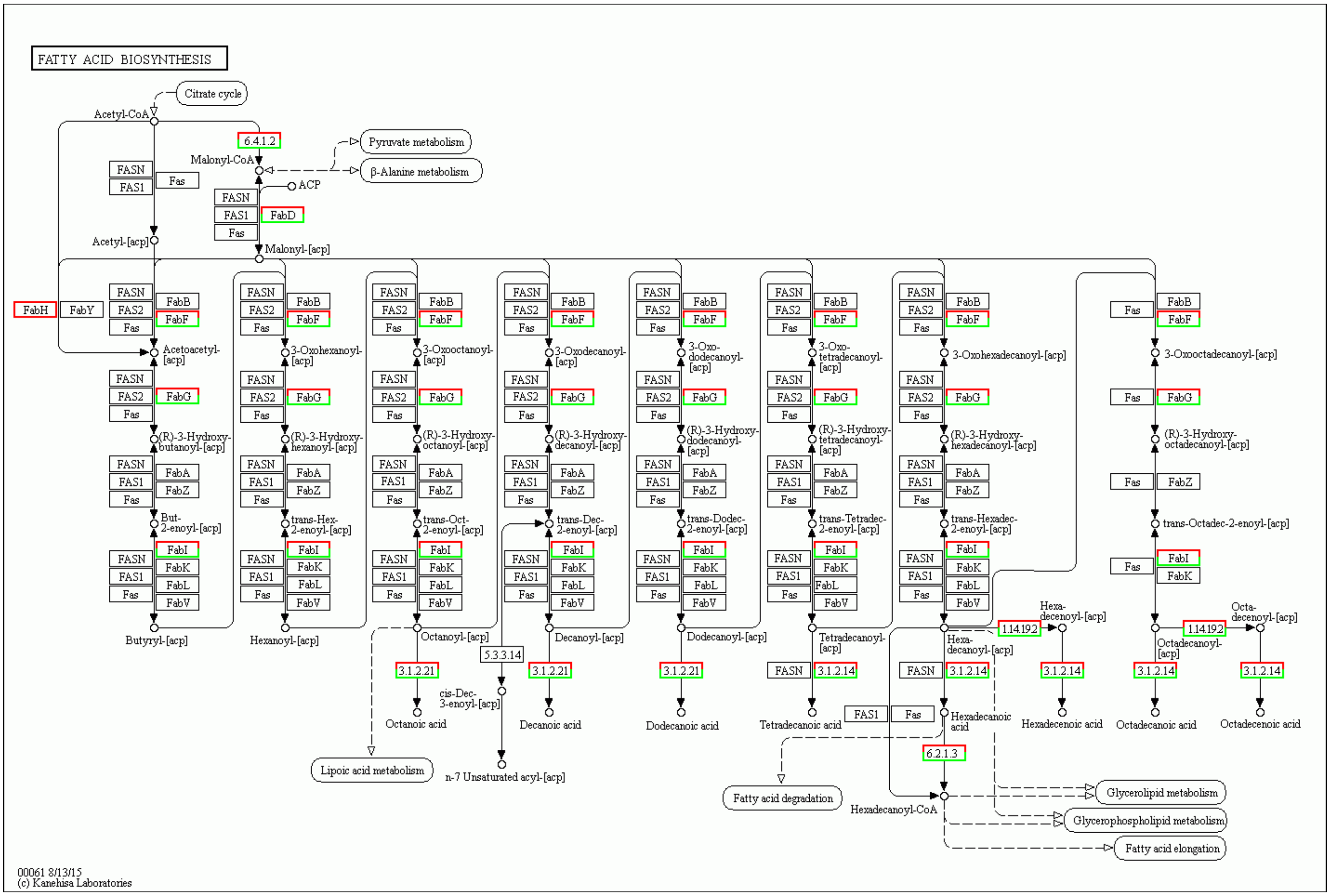
FATTY ACID BIOSYNTHESIS
Citrate cycle
Acetyl-CoA
6.4.1.2
Malonyl-CoA
Pyruvate metabolism
β-Alanine metabolism
ACP
FASN
FAS1
FAS2
Fas
FabD
Acetyl-[acp]
Malonyl-[acp]
FabH
FabY
FabB
FabF
FabG
FabA
FabZ
FabI
FabK
FabL
FabV
Acetoacetyl-[acp]
(R)-3-Hydroxy-butanoyl-[acp]
But-2-enoyl-[acp]
Butyryl-[acp]
3-Oxohexanoyl-[acp]
(R)-3-Hydroxy-hexanoyl-[acp]
trans-Hex-2-enoyl-[acp]
Hexanoyl-[acp]
3-Oxooctanoyl-[acp]
(R)-3-Hydroxy-octanoyl-[acp]
trans-Oct-2-enoyl-[acp]
Octanoyl-[acp]
3-Oxodecanoyl-[acp]
(R)-3-Hydroxy-decanoyl-[acp]
trans-Dec-2-enoyl-[acp]
Decanoyl-[acp]
3-Oxo-dodecanoyl-[acp]
(R)-3-Hydroxy-dodecanoyl-[acp]
trans-Dodec-2-enoyl-[acp]
Dodecanoyl-[acp]
3-Oxo-tetradecanoyl-[acp]
(R)-3-Hydroxy-tetradecanoyl-[acp]
trans-Tetradec-2-enoyl-[acp]
Tetradecanoyl-[acp]
3-Oxohexadecanoyl-[acp]
(R)-3-Hydroxy-hexadecanoyl-[acp]
trans-Hexadec-2-enoyl-[acp]
Hexa-decanoyl-[acp]
3-Oxooctadecanoyl-[acp]
(R)-3-Hydroxy-octadecanoyl-[acp]
trans-Octadec-2-enoyl-[acp]
Octadecanoyl-[acp]
5.3.3.14
cis-Dec-3-enoyl-[acp]
n-7 Unsaturated acyl-[acp]
3.1.2.21
3.1.2.14
1.14.19.2
Hexa-decenoyl-[acp]
Octa-decenoyl-[acp]
Octanoic acid
Decanoic acid
Dodecanoic acid
Tetradecanoic acid
Hexadecanoic acid
Hexadecenoic acid
Octadecanoic acid
Octadecenoic acid
Lipoic acid metabolism
6.2.1.3
Fatty acid degradation
Hexadecanoyl-CoA
Glycerolipid metabolism
Glycerophospholipid metabolism
Fatty acid elongation
00061 8/13/15
(c) Kanehisa Laboratories

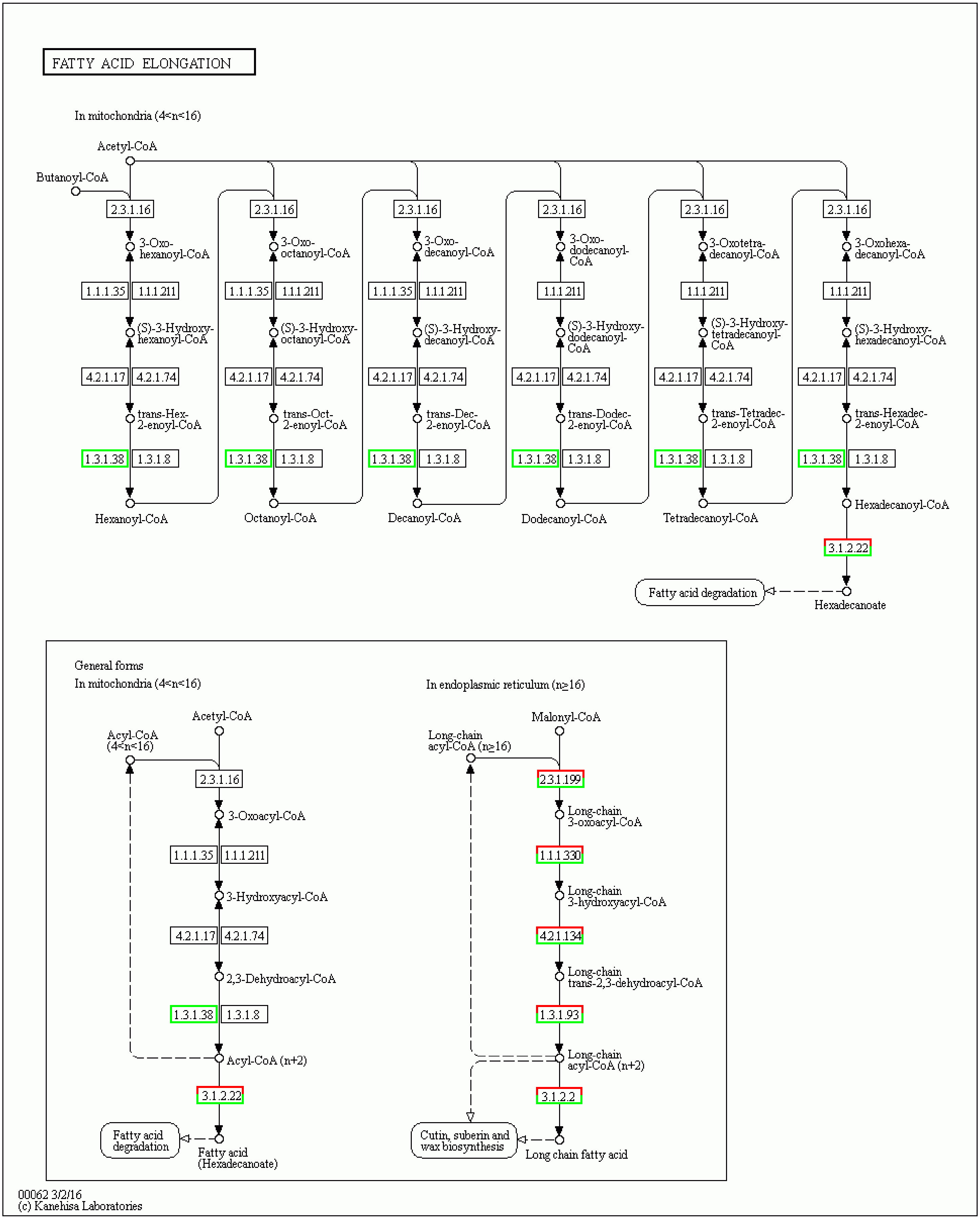
FATTY ACID ELONGATION
In mitochondria (4<n<16)
Acetyl-CoA
Butanoyl-CoA
2.3.1.16
3-Oxo-hexanoyl-CoA
3-Oxo-octanoyl-CoA
3-Oxo-decanoyl-CoA
3-Oxo-dodecanoyl-CoA
3-Oxotetra-decanoyl-CoA
3-Oxohexa-decanoyl-CoA
1.1.1.35
1.1.1.211
(S)-3-Hydroxy-hexanoyl-CoA
(S)-3-Hydroxy-octanoyl-CoA
(S)-3-Hydroxy-decanoyl-CoA
(S)-3-Hydroxy-dodecanoyl-CoA
(S)-3-Hydroxy-tetradecanoyl-CoA
(S)-3-Hydroxy-hexadecanoyl-CoA
4.2.1.17
4.2.1.74
trans-Hex-2-enoyl-CoA
trans-Oct-2-enoyl-CoA
trans-Dec-2-enoyl-CoA
trans-Dodec-2-enoyl-CoA
trans-Tetradec-2-enoyl-CoA
trans-Hexadec-2-enoyl-CoA
1.3.1.38
1.3.1.8
Hexanoyl-CoA
Octanoyl-CoA
Decanoyl-CoA
Dodecanoyl-CoA
Tetradecanoyl-CoA
Hexadecanoyl-CoA
3.1.2.22
Fatty acid degradation
Hexadecanoate
General forms
In mitochondria (4<n<16)
Acetyl-CoA
Acyl-CoA (4<n<16)
2.3.1.16
3-Oxoacyl-CoA
1.1.1.35
1.1.1.211
3-Hydroxyacyl-CoA
4.2.1.17
4.2.1.74
2,3-Dehydroacyl-CoA
1.3.1.38
1.3.1.8
Acyl-CoA (n+2)
3.1.2.22
Fatty acid degradation
Fatty acid (Hexadecanoate)
In endoplasmic reticulum (n≥16)
Malonyl-CoA
Long-chain acyl-CoA (n≥16)
2.3.1.199
Long-chain 3-oxoacyl-CoA
1.1.1.330
Long-chain 3-hydroxyacyl-CoA
4.2.1.134
Long-chain trans-2,3-dehydroacyl-CoA
1.3.1.93
Long-chain acyl-CoA (n+2)
3.1.2.2
Cutin, suberin and wax biosynthesis
Long chain fatty acid
00062 3/2/16
(c) Kanehisa Laboratories

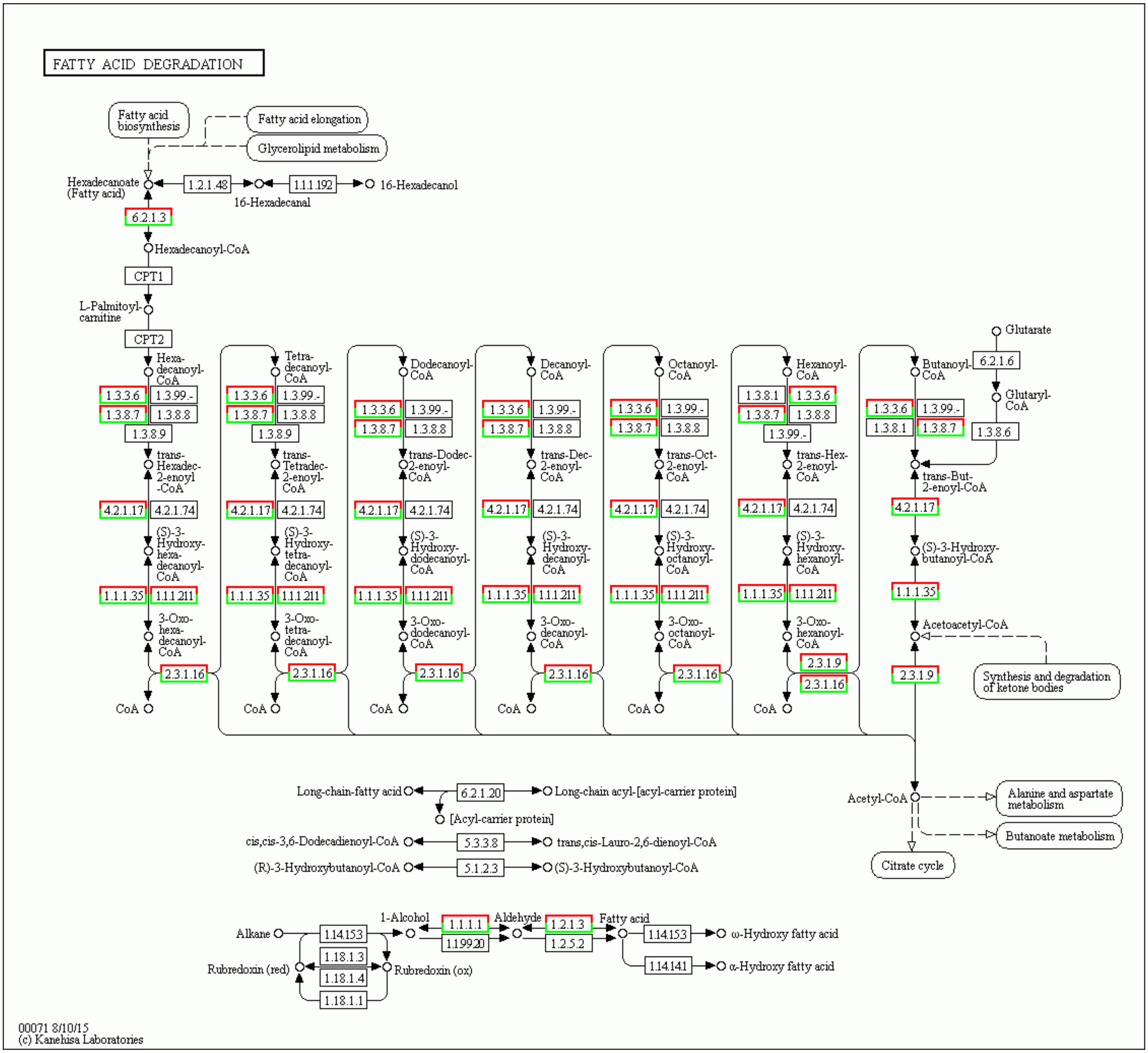
FATTY ACID DEGRADATION
Fatty acid biosynthesis
Fatty acid elongation
Glycerolipid metabolism
Hexadecanoate (Fatty acid)
1.2.1.48
1.1.1.192
16-Hexadecanol
16-Hexadecanal
6.2.1.3
Hexadecanoyl-CoA
CPT1
L-Palmitoyl-carnitine
CPT2
Hexa-decanoyl-CoA
Tetra-decanoyl-CoA
Dodecanoyl-CoA
Decanoyl-CoA
Octanoyl-CoA
Hexanoyl-CoA
Butanoyl-CoA
Glutarate
6.2.1.6
Glutaryl-CoA
1.3.8.6
1.3.3.6
1.3.99.-
1.3.8.7
1.3.8.8
1.3.8.9
1.3.8.1
trans-Hexadec-2-enoyl-CoA
trans-Tetradec-2-enoyl-CoA
trans-Dodec-2-enoyl-CoA
trans-Dec-2-enoyl-CoA
trans-Oct-2-enoyl-CoA
trans-Hex-2-enoyl-CoA
trans-But-2-enoyl-CoA
4.2.1.17
4.2.1.74
(S)-3-Hydroxy-hexa-decanoyl-CoA
(S)-3-Hydroxy-tetra-decanoyl-CoA
(S)-3-Hydroxy-dodecanoyl-CoA
(S)-3-Hydroxy-decanoyl-CoA
(S)-3-Hydroxy-octanoyl-CoA
(S)-3-Hydroxy-hexanoyl-CoA
(S)-3-Hydroxy-butanoyl-CoA
1.1.1.35
1.1.1.211
3-Oxo-hexa-decanoyl-CoA
3-Oxo-tetra-decanoyl-CoA
3-Oxo-dodecanoyl-CoA
3-Oxo-decanoyl-CoA
3-Oxo-octanoyl-CoA
3-Oxo-hexanoyl-CoA
Acetoacetyl-CoA
2.3.1.16
2.3.1.9
CoA
Synthesis and degradation of ketone bodies
Acetyl-CoA
Alanine and aspartate metabolism
Butanoate metabolism
Citrate cycle
Long-chain-fatty acid
6.2.1.20
Long-chain acyl-[acyl-carrier protein]
[Acyl-carrier protein]
cis,cis-3,6-Dodecadienoyl-CoA
5.3.3.8
trans,cis-Lauro-2,6-dienoyl-CoA
(R)-3-Hydroxybutanoyl-CoA
5.1.2.3
(S)-3-Hydroxybutanoyl-CoA
Alkane
1.14.15.3
1-Alcohol
1.1.1.1
1.1.99.20
Aldehyde
1.2.1.3
1.2.5.2
Fatty acid
ω-Hydroxy fatty acid
1.14.14.1
α-Hydroxy fatty acid
1.18.1.3
1.18.1.4
1.18.1.1
Rubredoxin (red)
Rubredoxin (ox)
00071 8/10/15
(c) Kanehisa Laboratories

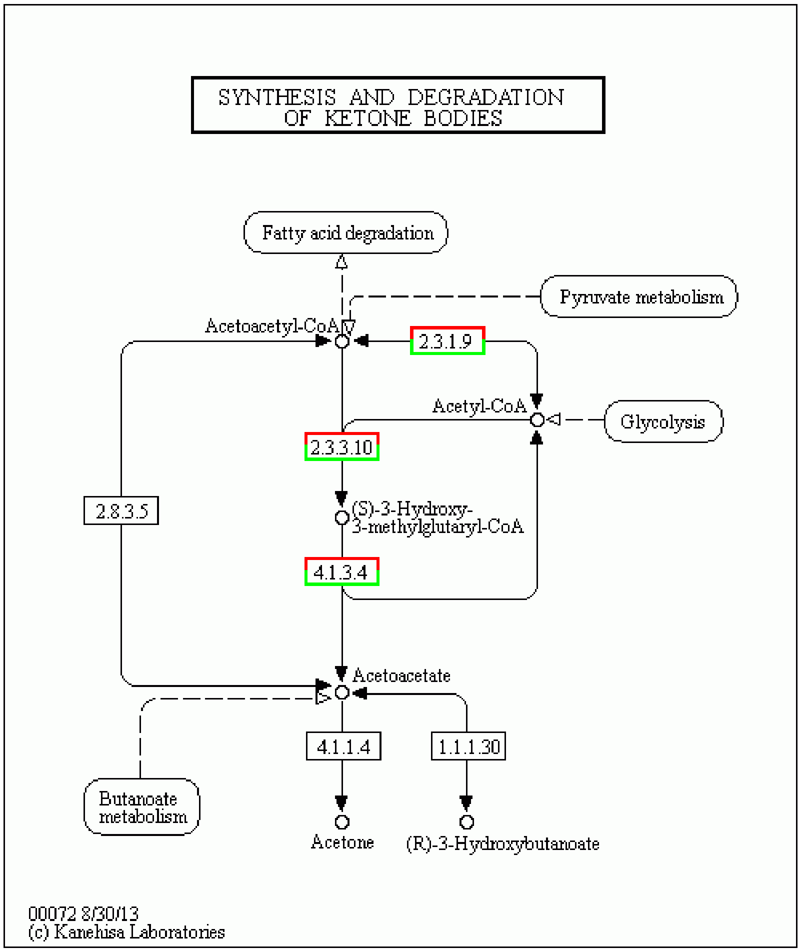
SYNTHESIS AND DEGRADATION OF KETONE BODIES
Fatty acid degradation
Pyruvate metabolism
Acetoacetyl-CoA
2.3.1.9
Acetyl-CoA
Glycolysis
2.3.3.10
2.8.3.5
(S)-3-Hydroxy-
3-methylglutaryl-CoA
4.1.3.4
Acetoacetate
4.1.1.4
1.1.1.30
Butanoate
metabolism
Acetone
(R)-3-Hydroxybutanoate
00072 8/30/13
(c) Kanehisa Laboratories

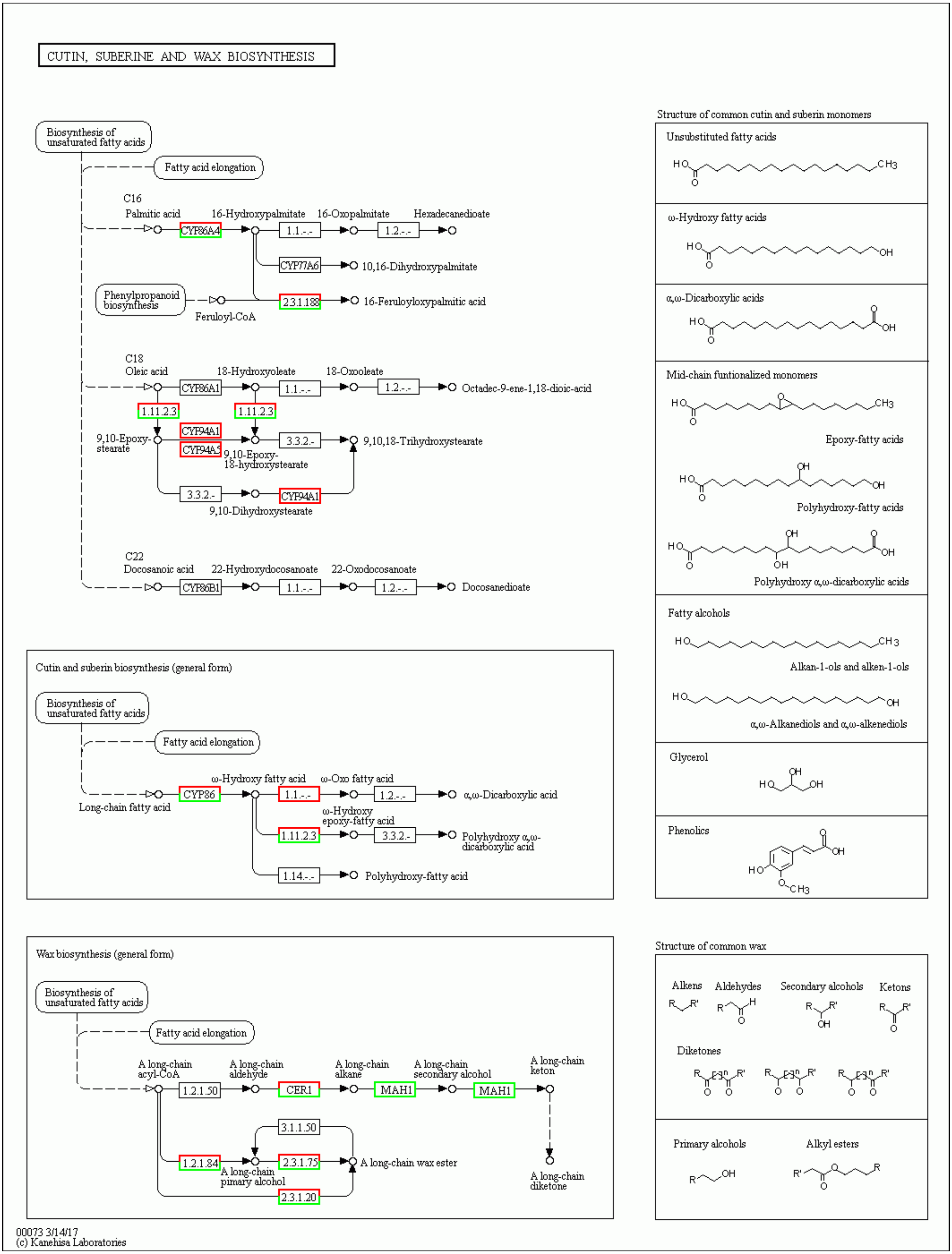
CUTIN, SUBERINE AND WAX BIOSYNTHESIS
Biosynthesis of unsaturated fatty acids
Fatty acid elongation
C16
Palmitic acid
16-Hydroxypalmitate
16-Oxopalmitate
Hexadecanedioate
CYP86A4
1.1.-.-
1.2.-.-
CYP77A6
10,16-Dihydroxypalmitate
Phenylpropanoid biosynthesis
Feruloyl-CoA
2.3.1.188
16-Feruloyloxypalmitic acid
C18
Oleic acid
18-Hydroxyoleate
18-Oxooleate
CYP86A1
Octadec-9-ene-1,18-dioic-acid
1.11.2.3
CYP94A1
CYP94A5
9,10-Epoxy-stearate
3.3.2.-
9,10,18-Trihydroxystearate
9,10-Epoxy-18-hydroxystearate
9,10-Dihydroxystearate
C22
Docosanoic acid
22-Hydroxydocosanoate
22-Oxodocosanoate
CYP86B1
Docosanedioate
Cutin and suberin biosynthesis (general form)
Long-chain fatty acid
CYP86
ω-Hydroxy fatty acid
ω-Oxo fatty acid
α,ω-Dicarboxylic acid
ω-Hydroxy epoxy-fatty acid
Polyhydroxy α,ω-dicarboxylic acid
1.14.-.-
Polyhydroxy-fatty acid
Wax biosynthesis (general form)
A long-chain acyl-CoA
A long-chain aldehyde
A long-chain alkane
A long-chain secondary alcohol
A long-chain keton
1.2.1.50
CER1
MAH1
3.1.1.50
1.2.1.84
2.3.1.75
2.3.1.20
A long-chain pimary alcohol
A long-chain wax ester
A long-chain diketone
Structure of common cutin and suberin monomers
Unsubstituted fatty acids
ω-Hydroxy fatty acids
α,ω-Dicarboxylic acids
Mid-chain funtionalized monomers
Epoxy-fatty acids
Polyhydroxy-fatty acids
Polyhydroxy α,ω-dicarboxylic acids
Fatty alcohols
Alkan-1-ols and alken-1-ols
α,ω-Alkanediols and α,ω-alkenediols
Glycerol
Phenolics
Structure of common wax
Alkens
Aldehydes
Secondary alcohols
Ketons
Diketones
Primary alcohols
Alkyl esters
00073 3/14/17
(c) Kanehisa Laboratories

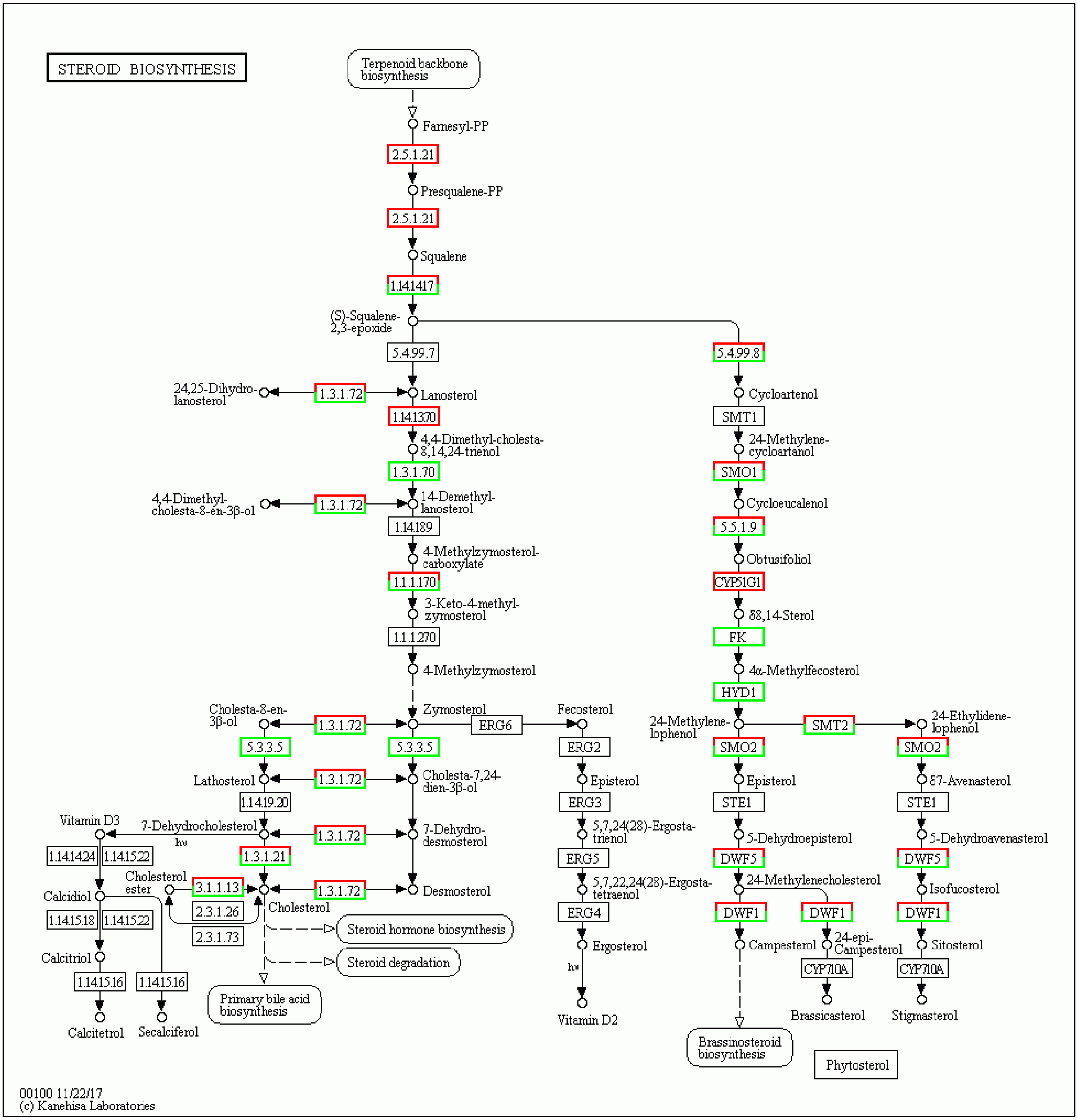
STEROID BIOSYNTHESIS
Terpenoid backbone biosynthesis
Farnesyl-PP
2.5.1.21
Presqualene-PP
2.5.1.21
Squalene
1.14.14.17
(S)-Squalene-2,3-epoxide
5.4.99.7
5.4.99.8
24,25-Dihydro-lanosterol
1.3.1.72
Lanosterol
1.14.13.70
4,4-Dimethyl-cholesta-8,14,24-trienol
1.3.1.70
4,4-Dimethyl-cholesta-8-en-3β-ol
14-Demethyl-lanosterol
1.14.18.9
4-Methylzymosterol-carboxylate
1.1.1.170
3-Keto-4-methyl-zymosterol
1.1.1.270
4-Methylzymosterol
Zymosterol
ERG6
Fecosterol
Cholesta-8-en-3β-ol
5.3.3.5
ERG2
Episterol
Lathosterol
Cholesta-7,24-dien-3β-ol
1.14.19.20
ERG3
5,7,24(28)-Ergosta-trienol
Vitamin D3
7-Dehydrocholesterol
7-Dehydro-desmosterol
hν
1.14.14.24
1.14.15.22
1.3.1.21
ERG5
5,7,22,24(28)-Ergosta-tetraenol
Cholesterol ester
3.1.1.13
Desmosterol
Calcidiol
Cholesterol
2.3.1.26
2.3.1.73
ERG4
1.14.15.18
Steroid hormone biosynthesis
Ergosterol
Calcitriol
Steroid degradation
1.14.15.16
Primary bile acid biosynthesis
Vitamin D2
Calcitetrol
Secalciferol
Cycloartenol
SMT1
24-Methylene-cycloartanol
SMO1
Cycloeucalenol
5.5.1.9
Obtusifoliol
CYP51G1
δ8,14-Sterol
FK
4α-Methylfecosterol
HYD1
24-Methylene-lophenol
SMT2
24-Ethylidene-lophenol
SMO2
Episterol
δ7-Avenasterol
STE1
5-Dehydroepisterol
5-Dehydroavenasterol
DWF5
24-Methylenecholesterol
Isofucosterol
DWF1
Campesterol
24-epi-Campesterol
Sitosterol
CYP710A
Brassicasterol
Stigmasterol
Brassinosteroid biosynthesis
Phytosterol
00100 11/22/17
(c) Kanehisa Laboratories

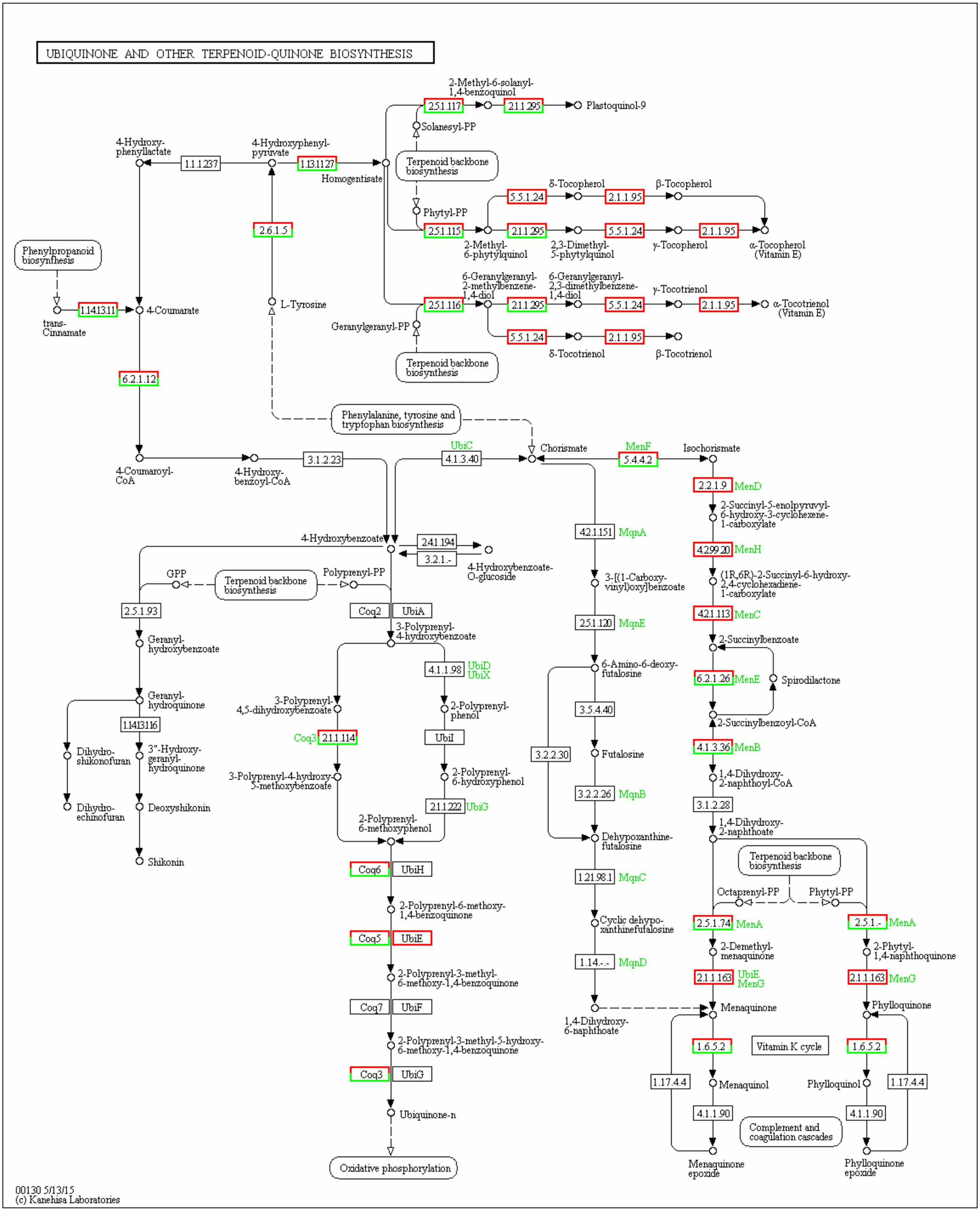

UBIQUINONE AND OTHER TERPENOID-QUINONE BIOSYNTHESIS
2-Methyl-6-solanyl-1,4-benzoquinol
2.5.1.117
2.1.1.295
Plastoquinol-9
Solanesyl-PP
4-Hydroxy-phenyllactate
1.1.1.237
4-Hydroxyphenyl-pyruvate
1.13.11.27
Homogentisate
Terpenoid backbone biosynthesis
δ-Tocopherol
β-Tocopherol
5.5.1.24
2.1.1.95
Phytyl-PP
2.6.1.5
2.5.1.115
2.1.1.295
5.5.1.24
2.1.1.95
2-Methyl-6-phytylquinol
2,3-Dimethyl-5-phytylquinol
γ-Tocopherol
α-Tocopherol (Vitamin E)
Phenylpropanoid biosynthesis
6-Geranylgeranyl-2-methylbenzene-1,4-diol
6-Geranylgeranyl-2,3-dimethylbenzene-1,4-diol
γ-Tocotrienol
1.14.13.11
4-Coumarate
L-Tyrosine
2.5.1.116
2.1.1.295
5.5.1.24
2.1.1.95
α-Tocotrienol (Vitamin E)
trans-Cinnamate
Geranylgeranyl-PP
5.5.1.24
2.1.1.95
δ-Tocotrienol
β-Tocotrienol
Terpenoid backbone biosynthesis
6.2.1.12
Phenylalanine, tyrosine and tryptophan biosynthesis
UbiC
MenF
Chorismate
Isochorismate
4.1.3.40
5.4.4.2
4-Coumaroyl-CoA
4-Hydroxy-benzoyl-CoA
3.1.2.23
2.2.1.9
MenD
2-Succinyl-5-enolpyruvyl-6-hydroxy-3-cyclohexene-1-carboxylate
4.2.1.151
MqnA
4-Hydroxybenzoate
2.4.1.194
3.2.1.-
4.2.99.20
MenH
4-Hydroxybenzoate-O-glucoside
GPP
Terpenoid backbone biosynthesis
Polyprenyl-PP
3-[(1-Carboxy-vinyl)oxy]benzoate
(1R,6R)-2-Succinyl-6-hydroxy-2,4-cyclohexadiene-1-carboxylate
2.5.1.93
Coq2
UbiA
4.2.1.113
MenC
2.5.1.120
MqnE
Geranyl-hydroxybenzoate
3-Polyprenyl-4-hydroxybenzoate
2-Succinylbenzoate
6-Amino-6-deoxy-futalosine
4.1.1.98
UbiD
UbiX
6.2.1.26
MenE
Spirodilactone
Geranyl-hydroquinone
3-Polyprenyl-4,5-dihydroxybenzoate
2-Polyprenyl-phenol
3.5.4.40
2-Succinylbenzoyl-CoA
1.14.13.116
Coq3
2.1.1.114
UbiI
3.2.2.30
Futalosine
4.1.3.36
MenB
Dihydro-shikonofuran
3"-Hydroxy-geranyl-hydroquinone
3-Polyprenyl-4-hydroxy-5-methoxybenzoate
2-Polyprenyl-6-hydroxyphenol
1,4-Dihydroxy-2-naphthoyl-CoA
3.2.2.26
MqnB
2.1.1.222
UbiG
3.1.2.28
Dihydro-echinofuran
Deoxyshikonin
2-Polyprenyl-6-methoxyphenol
Dehypoxanthine-futalosine
1,4-Dihydroxy-2-naphthoate
Shikonin
Coq6
UbiH
Terpenoid backbone biosynthesis
1.21.98.1
MqnC
Octaprenyl-PP
Phytyl-PP
2-Polyprenyl-6-methoxy-1,4-benzoquinone
Cyclic dehypo-xanthinefutalosine
2.5.1.74
MenA
2.5.1.-
MenA
Coq5
UbiE
2-Demethyl-menaquinone
2-Phytyl-1,4-naphthoquinone
1.14.-.-
MqnD
2-Polyprenyl-3-methyl-6-methoxy-1,4-benzoquinone
2.1.1.163
UbiE
MenG
2.1.1.163
MenG
Coq7
UbiF
1,4-Dihydroxy-6-naphthoate
Menaquinone
Phylloquinone
2-Polyprenyl-3-methyl-5-hydroxy-6-methoxy-1,4-benzoquinone
1.6.5.2
Vitamin K cycle
1.6.5.2
Coq3
UbiG
1.17.4.4
Menaquinol
Phylloquinol
1.17.4.4
4.1.1.90
4.1.1.90
Ubiquinone-n
Complement and coagulation cascades
Oxidative phosphorylation
Menaquinone epoxide
Phylloquinone epoxide
00130 5/13/15
(c) Kanehisa Laboratories

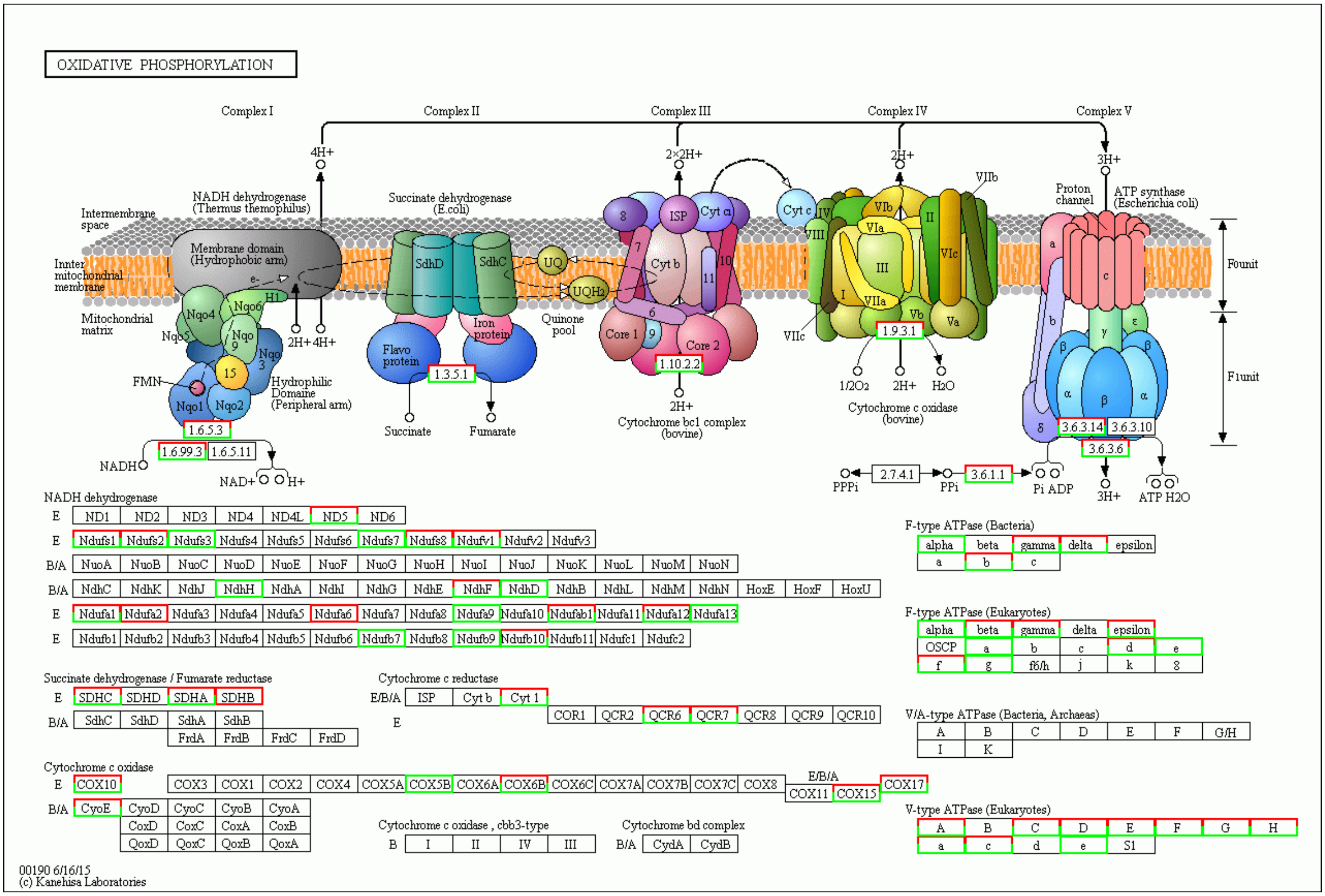
OXIDATIVE PHOSPHORYLATION
Complex I
Complex II
Complex III
Complex IV
Complex V
4H+
2×2H+
2H+
3H+
Intermembrane space
Inner mitochondrial membrane
Mitochondrial matrix
NADH dehydrogenase (Thermus themophilus)
Membrane domain (Hydrophobic arm)
Hydrophilic Domaine (Peripheral arm)
FMN
Nqo4
Nqo6
Nqo5
Nqo9
Nqo3
Nqo1
Nqo2
15
H1
e-
2H+ 4H+
Succinate dehydrogenase (E.coli)
SdhD
SdhC
Flavo protein
Iron protein
UQ
UQH2
Quinone pool
Succinate
Fumarate
8
ISP
Cyt c1
Cyt b
Core 1
Core 2
Cytochrome bc1 complex (bovine)
Cyt c
VIIb
VIb
VIa
VIIa
VIII
VIIc
VIc
Vb
Va
IV
III
II
I
Cytochrome c oxidase (bovine)
1/2O2
H2O
Proton channel
ATP synthase (Escherichia coli)
Founit
F1unit
NADH
NAD+
H+
1.6.5.3
1.6.99.3
1.6.5.11
1.3.5.1
1.10.2.2
1.9.3.1
3.6.3.14
3.6.3.10
3.6.3.6
2.7.4.1
3.6.1.1
PPPi
PPi
Pi ADP
ATP H2O
NADH dehydrogenase
E ND1 ND2 ND3 ND4 ND4L ND5 ND6
E Ndufs1 Ndufs2 Ndufs3 Ndufs4 Ndufs5 Ndufs6 Ndufs7 Ndufs8 Ndufv1 Ndufv2 Ndufv3
B/A NuoA NuoB NuoC NuoD NuoE NuoF NuoG NuoH NuoI NuoJ NuoK NuoL NuoM NuoN
B/A NdhC NdhK NdhJ NdhH NdhA NdhI NdhG NdhE NdhF NdhD NdhB NdhL NdhM NdhN HoxE HoxF HoxU
E Ndufa1 Ndufa2 Ndufa3 Ndufa4 Ndufa5 Ndufa6 Ndufa7 Ndufa8 Ndufa9 Ndufa10 Ndufab1 Ndufa11 Ndufa12 Ndufa13
E Ndufb1 Ndufb2 Ndufb3 Ndufb4 Ndufb5 Ndufb6 Ndufb7 Ndufb8 Ndufb9 Ndufb10 Ndufb11 Ndufc1 Ndufc2
Succinate dehydrogenase / Fumarate reductase
E SDHC SDHD SDHA SDHB
B/A SdhC SdhD SdhA SdhB
FrdA FrdB FrdC FrdD
Cytochrome c reductase
E/B/A ISP Cyt b Cyt 1
E COR1 QCR2 QCR6 QCR7 QCR8 QCR9 QCR10
Cytochrome c oxidase
E COX10 COX3 COX1 COX2 COX4 COX5A COX5B COX6A COX6B COX6C COX7A COX7B COX7C COX8
E/B/A COX11 COX15 COX17
B/A CyoE CyoD CyoC CyoB CyoA
CoxD CoxC CoxA CoxB
QoxD QoxC QoxB QoxA
Cytochrome c oxidase , cbb3-type
B I II IV III
Cytochrome bd complex
B/A CydA CydB
F-type ATPase (Bacteria)
alpha beta gamma delta epsilon
a b c
F-type ATPase (Eukaryotes)
alpha beta gamma delta epsilon
OSCP a b c d e
f g f6/h j k 8
V/A-type ATPase (Bacteria, Archaeas)
A B C D E F G/H
I K
V-type ATPase (Eukaryotes)
A B C D E F G H
a c d e S1
00190 6/16/15
(c) Kanehisa Laboratories

PHOTOSYNTHESIS

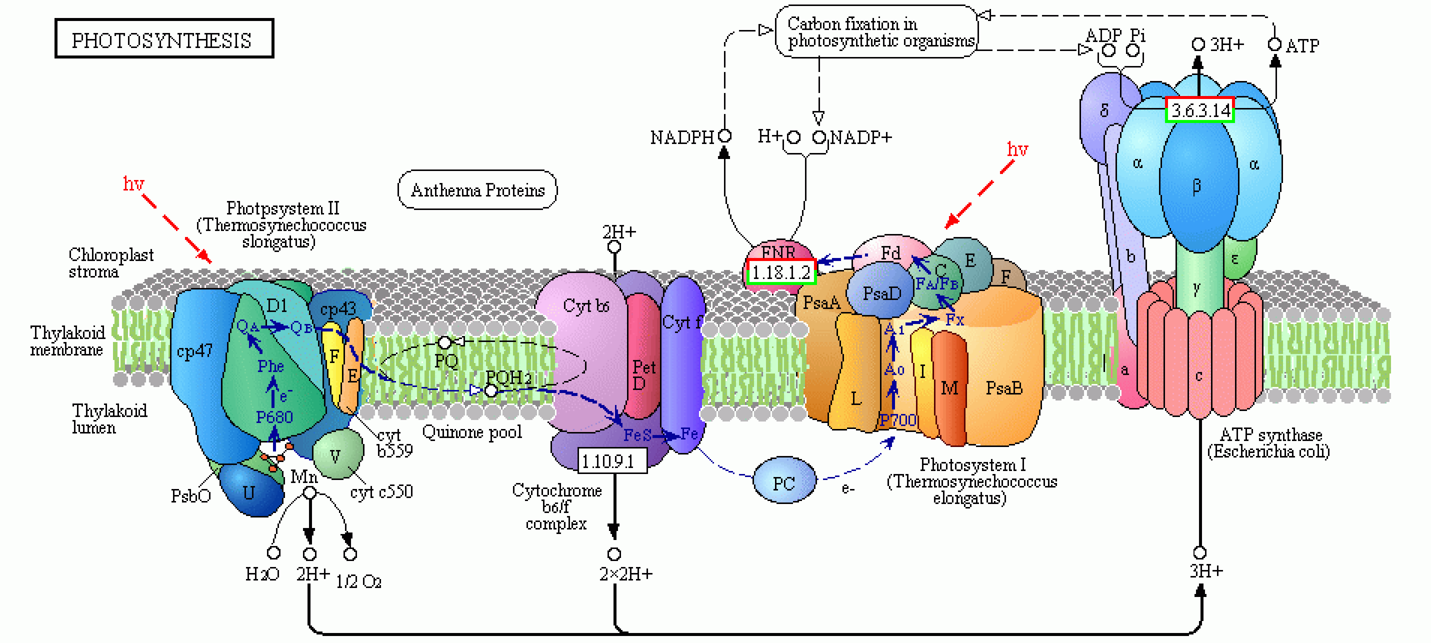

Photosystem II

D1	D2	cp43	cp47	cyt b559	
PsbA	PsbD	PsbC	PsbB	PsbE	PsbF

					MSP	OEC
Psb L	Psb J	Psb K	Psb M	Psb H	Psb I	Psb O	Psb P
Psb Q	Psb R	Psb S	Psb T	Psb U	Psb V	Psb W	Psb X
Psb Y	Psb Z	Psb 27	Psb 28	Psb28-2			

Photosystem I

PsaA	PsaB	PsaC	PsaD	PsaE	PsaF	PsaG	PsaH
PsaI	PsaJ	PsaK	PsaL	PsaM	PsaN	PsaO	PsaX

Cytochrome b6/f complex

PetB	PetD	PetA	PetC	PetL	PetM	PetN	PetG

Photosynthethic electron transport

PC	Fd	FNR	cyt c6
PetE	PetF	PetH	PetJ

F-type ATPase

beta	alpha	gamma	delta	epsilon	c	a	b

00195 1/15/14
(c) Kanehisa Laboratories

PHOTOSYNTHESIS - ANTENNA PROTEINS

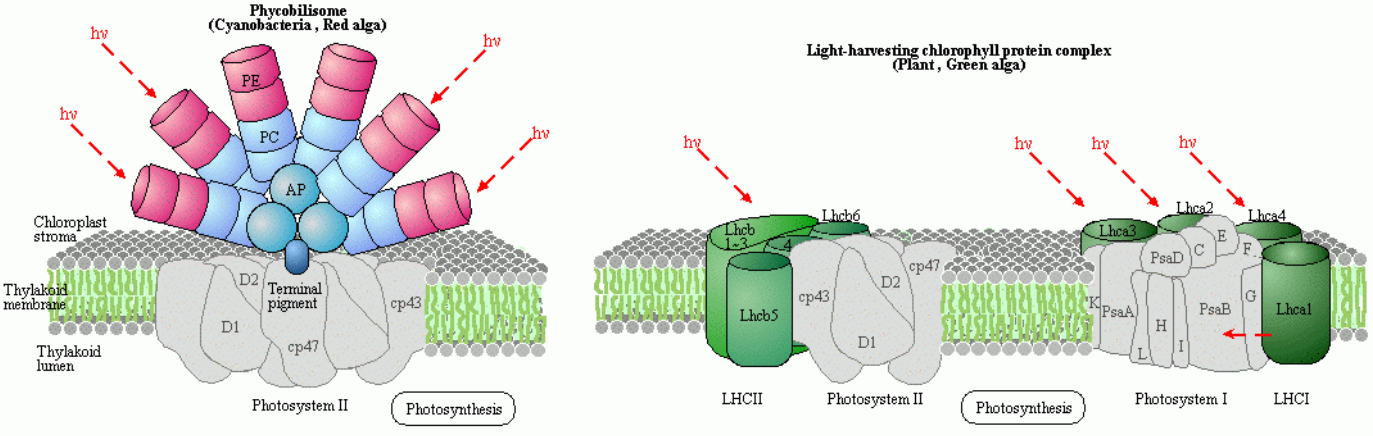

Allophycocyanin(AP)

ApcA	ApcB	ApcC	ApcD	ApcE	ApcF

Phycocyanin(PC) / Phycoerythrocyanin(PEC)

CpcA	CpcB	CpcC	CpcD	CpcE	CpcF	CpcG

Phycoerythrin(PE)

CpeA	CpeB	CpeC	CpeD	CpeE	CpeR	CpeS	CpeT	CpeU	CpeY	CpeZ

Light-harvesting chlorophyll protein complex(LHC)

Lhca1	Lhca2	Lhca3	Lhca4	Lhca5

Lhcb1	Lhcb2	Lhcb3	Lhcb4	Lhcb5	Lhcb6	Lhcb7

00196 11/16/10
(c) Kanehisa Laboratories

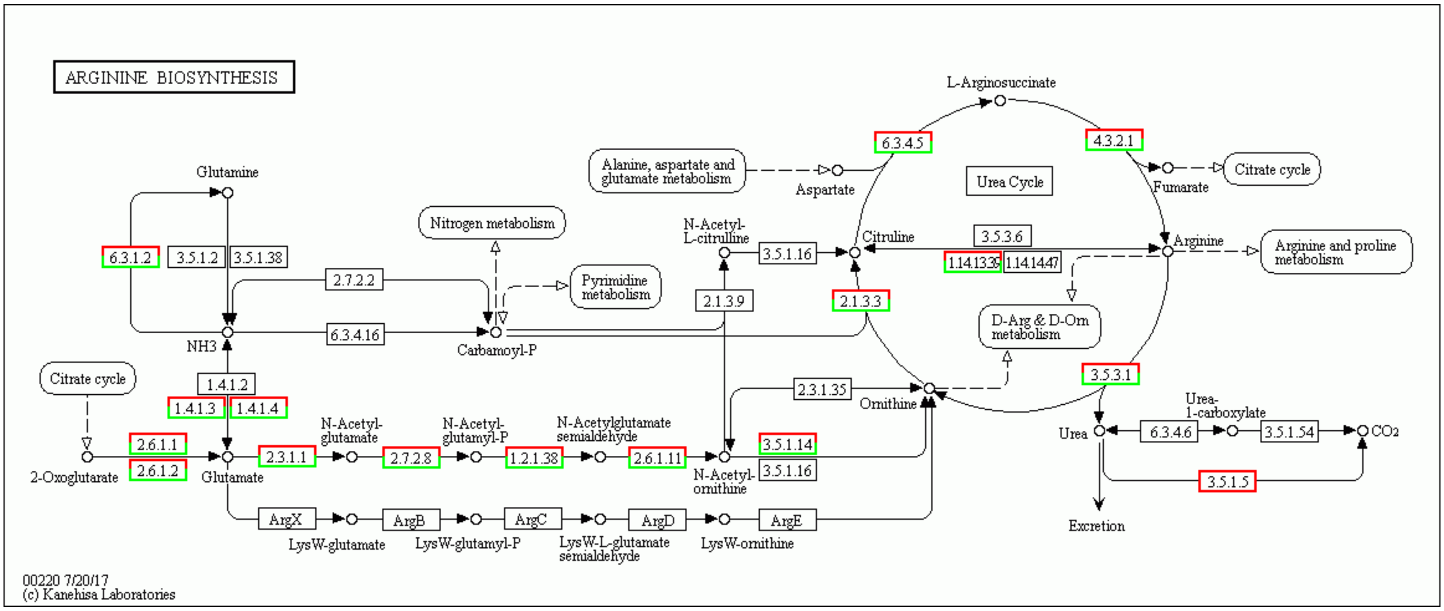

ARGININE BIOSYNTHESIS
L-Arginosuccinate
6.3.4.5
4.3.2.1
Alanine, aspartate and glutamate metabolism
Aspartate
Urea Cycle
Citrate cycle
Fumarate
Glutamine
Nitrogen metabolism
N-Acetyl-L-citrulline
Citrulline
3.5.3.6
Arginine
Arginine and proline metabolism
6.3.1.2
3.5.1.2
3.5.1.38
2.7.2.2
Pyrimidine metabolism
3.5.1.16
1.14.13.39
1.14.14.47
2.1.3.9
2.1.3.3
D-Arg & D-Orn metabolism
NH3
6.3.4.16
Carbamoyl-P
Citrate cycle
1.4.1.2
1.4.1.3
1.4.1.4
2.3.1.35
Ornithine
3.5.3.1
Urea-1-carboxylate
N-Acetyl-glutamate
N-Acetyl-glutamyl-P
N-Acetylglutamate semialdehyde
Urea
6.3.4.6
3.5.1.54
CO2
2.6.1.1
2.3.1.1
2.7.2.8
1.2.1.38
2.6.1.11
3.5.1.14
2.6.1.2
2-Oxoglutarate
Glutamate
N-Acetyl-ornithine
3.5.1.16
3.5.1.5
ArgX
ArgB
ArgC
ArgD
ArgE
Excretion
LysW-glutamate
LysW-glutamyl-P
LysW-L-glutamate semialdehyde
LysW-ornithine
00220 7/20/17
(c) Kanehisa Laboratories

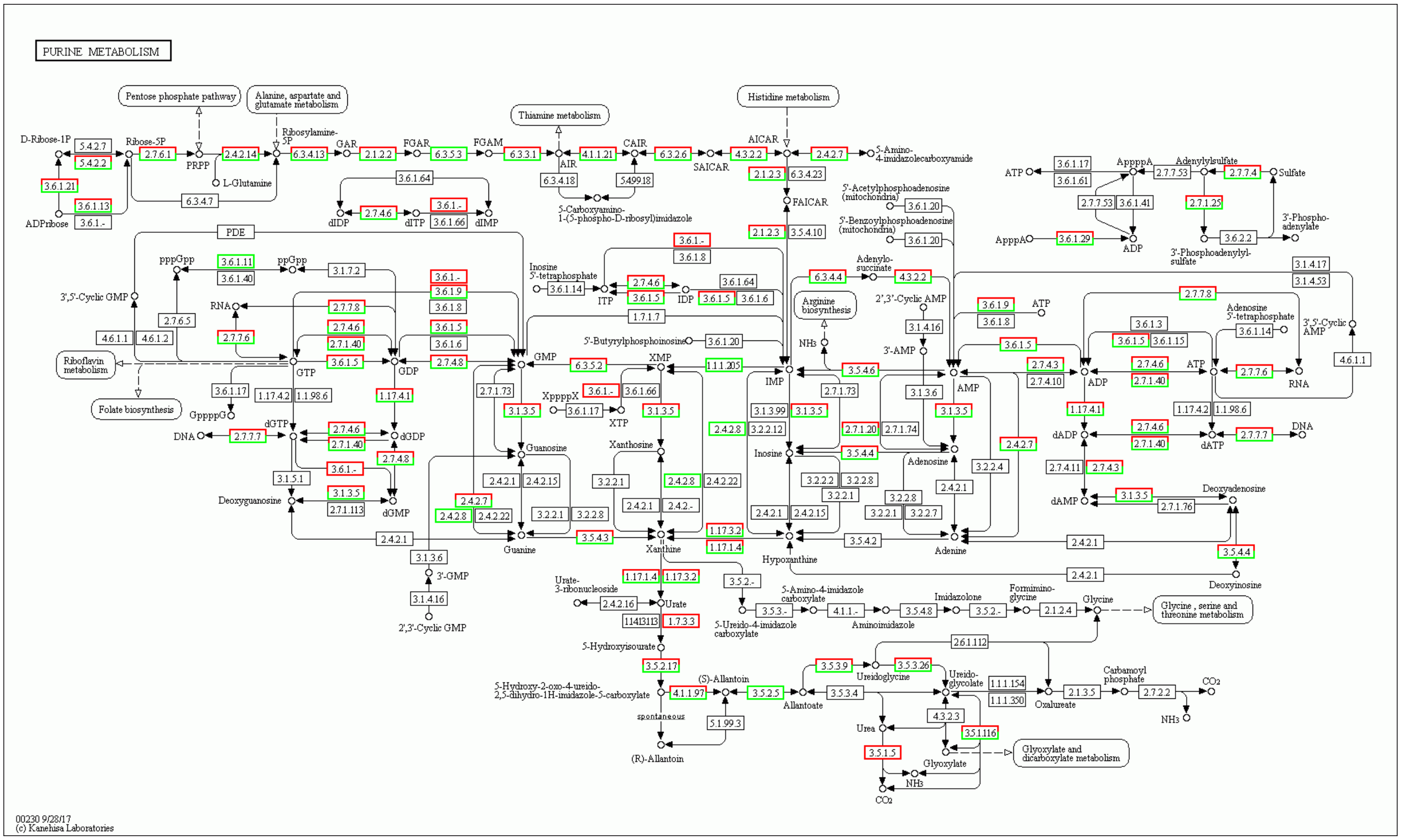

PURINE METABOLISM
Pentose phosphate pathway
Alanine, aspartate and glutamate metabolism
Thiamine metabolism
Histidine metabolism
D-Ribose-1P
Ribose-5P
Ribosylamine-5P
GAR
FGAR
FGAM
AIR
CAIR
SAICAR
AICAR
5-Amino-4-imidazolecarboxyamide
PRPP
L-Glutamine
ADPribose
dIDP
dITP
dIMP
5-Carboxyamino-1-(5-phospho-D-ribosyl)imidazole
FAICAR
PDE
ppGpp
pppGpp
3',5'-Cyclic GMP
RNA
Riboflavin metabolism
Folate biosynthesis
GTP
GDP
GMP
GppppG
dGTP
dGDP
dGMP
DNA
Deoxyguanosine
Guanosine
Guanine
Inosine 5'-tetraphosphate
ITP
IDP
IMP
XTP
XpppppX
XMP
Xanthosine
Xanthine
Inosine
Hypoxanthine
5'-Butyrylphosphoinosine
Arginine biosynthesis
Adenylo-succinate
NH3
2',3'-Cyclic AMP
3'-AMP
AMP
Adenosine
Adenine
5'-Acetylphosphoadenosine (mitochondria)
5'-Benzoylphosphoadenosine (mitochondria)
ATP
ApppA
Adenylylsulfate
Sulfate
AppppA
ADP
3'-Phospho-adenylate
3'-Phosphoadenylyl-sulfate
Adenosine 5'-tetraphosphate
3',5'-Cyclic AMP
dADP
dATP
dAMP
Deoxyadenosine
Deoxyinosine
3'-GMP
2',3'-Cyclic GMP
Urate-3-ribonucleoside
Urate
5-Hydroxyisourate
5-Hydroxy-2-oxo-4-ureido-2,5-dihydro-1H-imidazole-5-carboxylate
spontaneous
(S)-Allantoin
(R)-Allantoin
Allantoate
5-Amino-4-imidazole carboxylate
5-Ureido-4-imidazole carboxylate
Aminoimidazole
Imidazolone
Formimino-glycine
Glycine
Glycine, serine and threonine metabolism
Ureidoglycine
Ureido-glycolate
Oxalureate
Carbamoyl phosphate
CO2
NH3
Urea
Glyoxylate
Glyoxylate and dicarboxylate metabolism
00230 9/28/17
(c) Kanehisa Laboratories

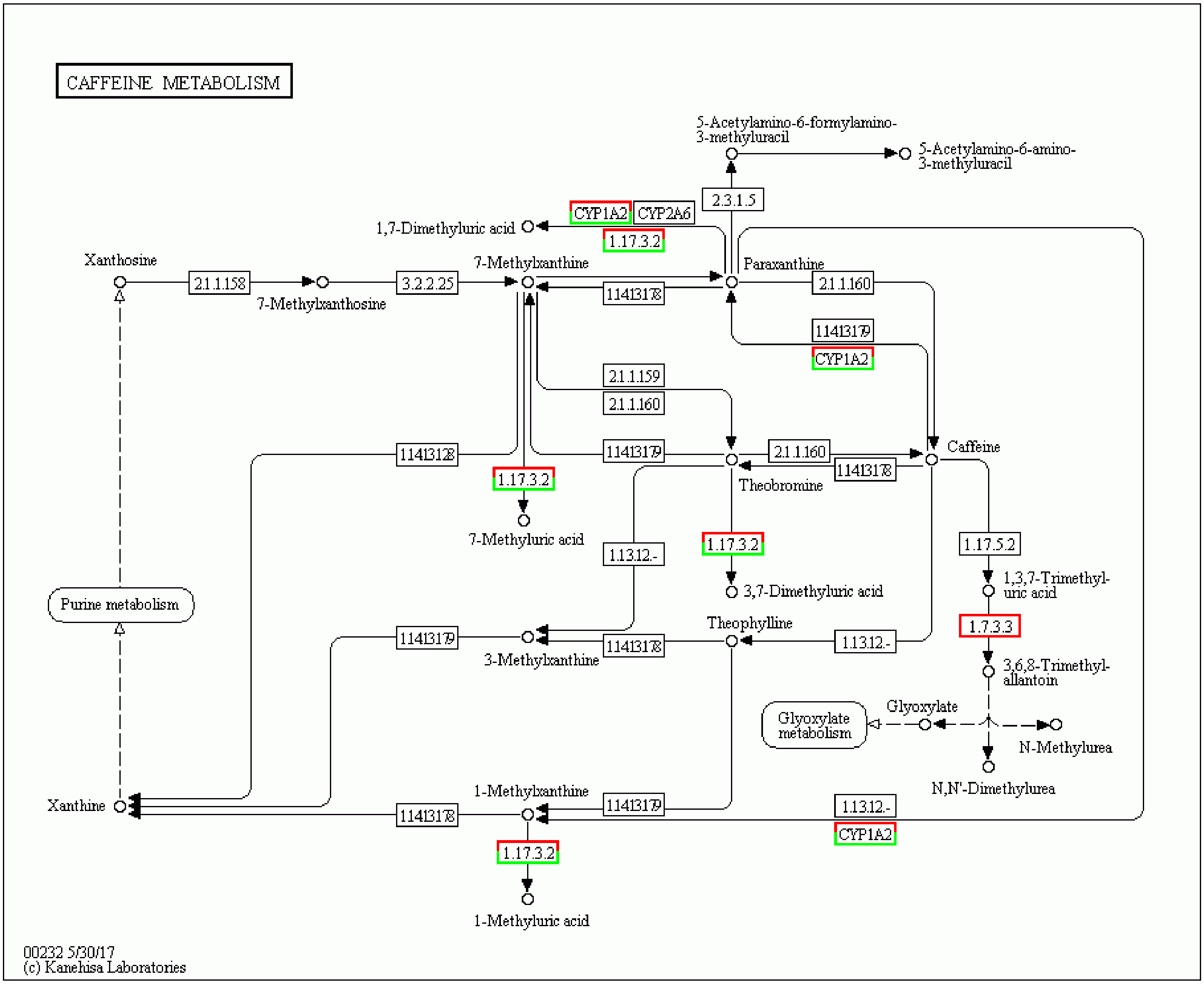
CAFFEINE METABOLISM
5-Acetylamino-6-formylamino-3-methyluracil
5-Acetylamino-6-amino-3-methyluracil
2.3.1.5
CYP1A2
CYP2A6
1.17.3.2
1,7-Dimethyluric acid
Xanthosine
2.1.1.158
7-Methylxanthosine
3.2.2.25
7-Methylxanthine
1.14.13.178
Paraxanthine
2.1.1.160
1.14.13.179
CYP1A2
2.1.1.159
2.1.1.160
1.14.13.128
1.14.13.179
2.1.1.160
Caffeine
1.14.13.178
Theobromine
1.17.3.2
7-Methyluric acid
1.13.12.-
1.17.3.2
3,7-Dimethyluric acid
1.17.5.2
1,3,7-Trimethyl-uric acid
Purine metabolism
1.14.13.179
3-Methylxanthine
1.14.13.178
Theophylline
1.13.12.-
1.7.3.3
3,6,8-Trimethyl-allantoin
Glyoxylate
Glyoxylate metabolism
N-Methylurea
N,N'-Dimethylurea
1-Methylxanthine
Xanthine
1.14.13.178
1.14.13.179
1.13.12.-
CYP1A2
1.17.3.2
1-Methyluric acid
00232 5/30/17
(c) Kanehisa Laboratories

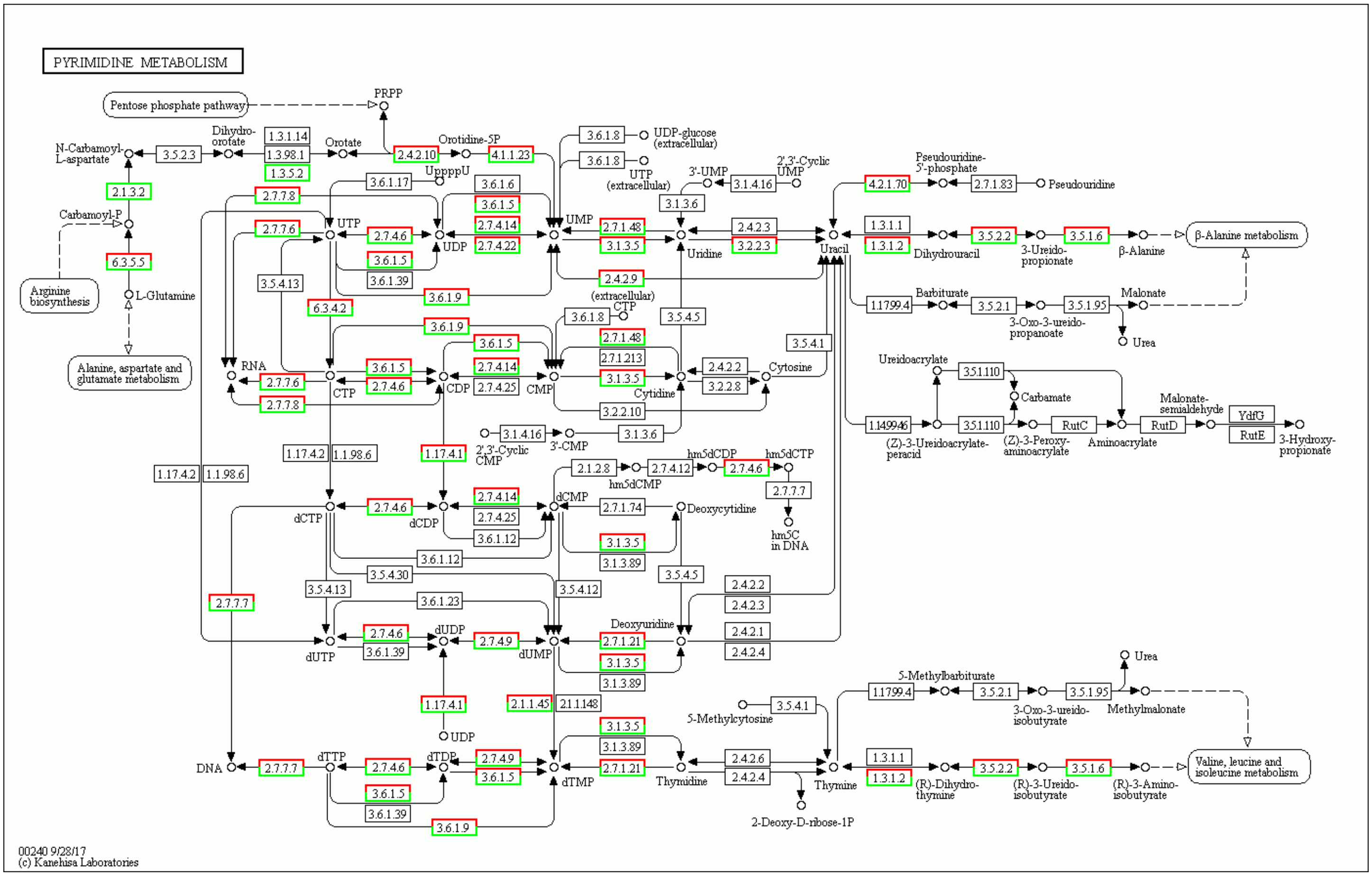
PYRIMIDINE METABOLISM
Pentose phosphate pathway
PRPP
N-Carbamoyl-L-aspartate
3.5.2.3
Dihydro-orotate
1.3.1.14
1.3.98.1
1.3.5.2
Orotate
2.4.2.10
Orotidine-5P
4.1.1.23
2.1.3.2
Carbamoyl-P
6.3.5.5
Arginine biosynthesis
L-Glutamine
Alanine, aspartate and glutamate metabolism
UDP-glucose (extracellular)
UTP (extracellular)
3'-UMP
2',3'-Cyclic UMP
3.1.4.16
3.1.3.6
3.6.1.8
3.6.1.17
UppppU
3.6.1.6
3.6.1.5
2.7.4.14
2.7.4.22
2.7.7.8
2.7.7.6
UTP
2.7.4.6
UDP
UMP
3.6.1.39
3.6.1.9
3.5.4.13
6.3.4.2
2.7.1.48
3.1.3.5
2.4.2.3
3.2.2.3
Uridine
2.4.2.9
(extracellular)
CTP
Pseudouridine-5'-phosphate
4.2.1.70
2.7.1.83
Pseudouridine
1.3.1.1
1.3.1.2
Uracil
Dihydrouracil
3.5.2.2
3-Ureido-propionate
3.5.1.6
β-Alanine
β-Alanine metabolism
1.17.99.4
Barbiturate
3.5.2.1
3-Oxo-3-ureido-propanoate
3.5.1.95
Malonate
Urea
RNA
CTP
CDP
2.7.4.25
CMP
2.7.1.213
Cytidine
2.4.2.2
3.2.2.8
Cytosine
3.5.4.5
3.5.4.1
3.2.2.10
3.1.3.6
3.1.4.16
2',3'-Cyclic CMP
3'-CMP
Ureidoacrylate
3.5.1.110
Carbamate
1.14.99.46
(Z)-3-Ureidoacrylate-peracid
(Z)-3-Peroxy-aminoacrylate
RutC
Aminoacrylate
RutD
Malonate-semialdehyde
YdfG
RutE
3-Hydroxy-propionate
1.17.4.2
1.1.98.6
1.17.4.1
2.1.2.8
hm5dCMP
2.7.4.12
hm5dCDP
hm5dCTP
2.7.7.7
hm5C in DNA
dCTP
dCDP
dCMP
2.7.1.74
Deoxycytidine
3.6.1.12
3.1.3.89
3.5.4.30
3.5.4.12
3.6.1.23
dUTP
dUDP
2.7.4.9
dUMP
2.7.1.21
Deoxyuridine
2.4.2.1
2.4.2.4
2.1.1.45
2.1.1.148
UDP
5-Methylcytosine
5-Methylbarbiturate
3-Oxo-3-ureido-isobutyrate
Methylmalonate
DNA
dTTP
dTDP
dTMP
Thymidine
2.4.2.6
Thymine
2-Deoxy-D-ribose-1P
(R)-Dihydro-thymine
(R)-3-Ureido-isobutyrate
(R)-3-Amino-isobutyrate
Valine, leucine and isoleucine metabolism
00240 9/28/17
(c) Kanehisa Laboratories

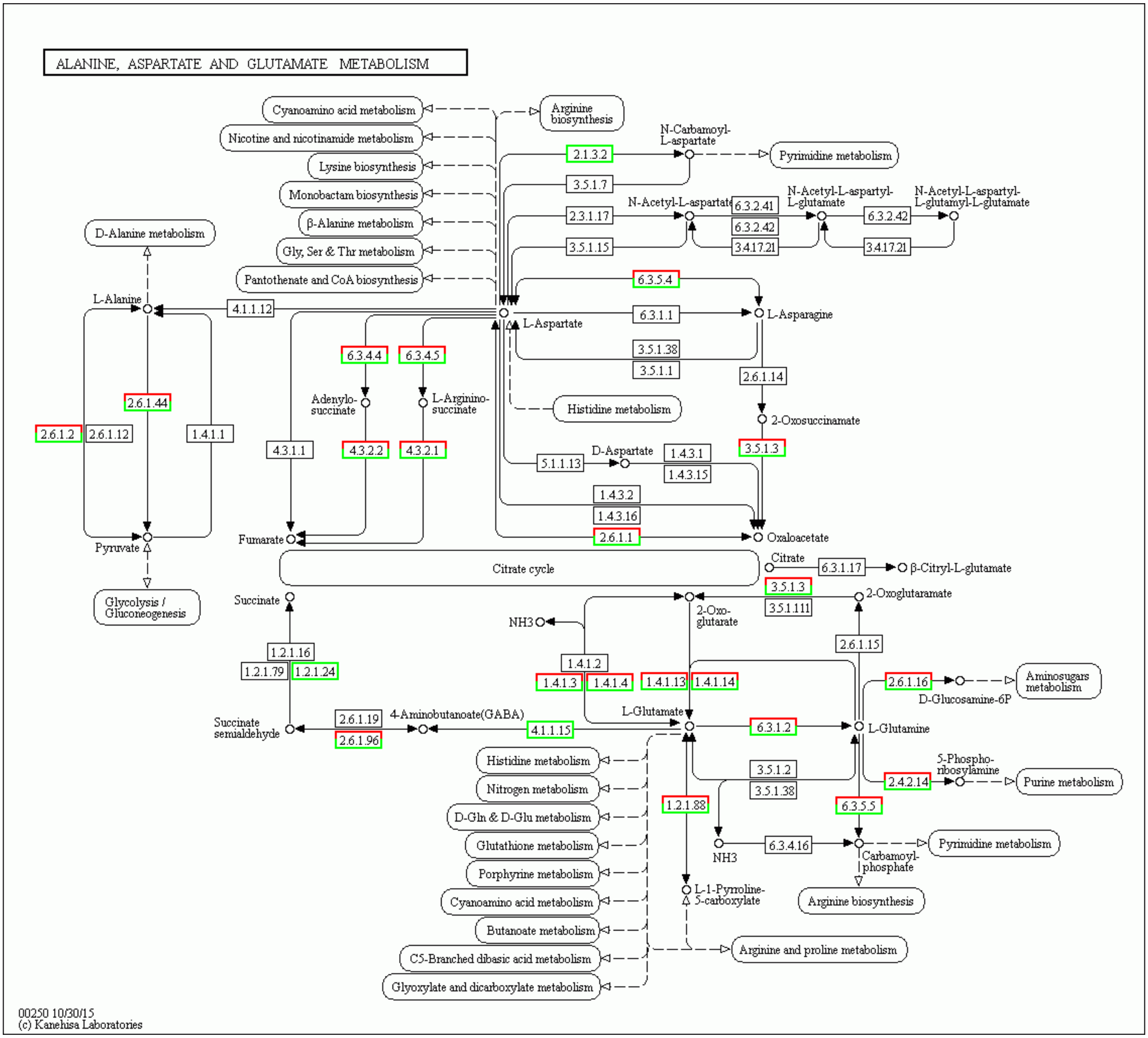

ALANINE, ASPARTATE AND GLUTAMATE METABOLISM
Cyanoamino acid metabolism
Nicotine and nicotinamide metabolism
Lysine biosynthesis
Monobactam biosynthesis
β-Alanine metabolism
Gly, Ser & Thr metabolism
Pantothenate and CoA biosynthesis
Arginine biosynthesis
N-Carbamoyl-L-aspartate
Pyrimidine metabolism
2.1.3.2
3.5.1.7
2.3.1.17
3.5.1.15
N-Acetyl-L-aspartate
6.3.2.41
6.3.2.42
3.4.17.21
N-Acetyl-L-aspartyl-L-glutamate
6.3.2.42
3.4.17.21
N-Acetyl-L-aspartyl-L-glutamyl-L-glutamate
D-Alanine metabolism
L-Alanine
4.1.1.12
6.3.5.4
6.3.1.1
L-Aspartate
L-Asparagine
3.5.1.38
3.5.1.1
6.3.4.4
6.3.4.5
Adenylo-succinate
L-Argininosuccinate
Histidine metabolism
2.6.1.14
2-Oxosuccinamate
2.6.1.44
2.6.1.2
2.6.1.12
1.4.1.1
4.3.1.1
4.3.2.2
4.3.2.1
3.5.1.3
D-Aspartate
1.4.3.1
1.4.3.15
5.1.1.13
1.4.3.2
1.4.3.16
2.6.1.1
Pyruvate
Fumarate
Oxaloacetate
Glycolysis / Gluconeogenesis
Citrate cycle
Citrate
6.3.1.17
β-Citryl-L-glutamate
3.5.1.3
3.5.1.111
2-Oxoglutaramate
Succinate
NH3
2-Oxo-glutarate
2.6.1.15
1.2.1.16
1.4.1.2
1.2.1.79
1.2.1.24
1.4.1.3
1.4.1.4
1.4.1.13
1.4.1.14
2.6.1.16
Aminosugars metabolism
D-Glucosamine-6P
Succinate semialdehyde
2.6.1.19
4-Aminobutanoate(GABA)
L-Glutamate
2.6.1.96
4.1.1.15
6.3.1.2
L-Glutamine
Histidine metabolism
Nitrogen metabolism
D-Gln & D-Glu metabolism
Glutathione metabolism
Porphyrine metabolism
Cyanoamino acid metabolism
Butanoate metabolism
C5-Branched dibasic acid metabolism
Glyoxylate and dicarboxylate metabolism
3.5.1.2
3.5.1.38
5-Phospho-ribosylamine
2.4.2.14
Purine metabolism
1.2.1.88
6.3.5.5
NH3
6.3.4.16
Carbamoyl-phosphate
Pyrimidine metabolism
L-1-Pyrroline-5-carboxylate
Arginine biosynthesis
Arginine and proline metabolism
00250 10/30/15
(c) Kanehisa Laboratories

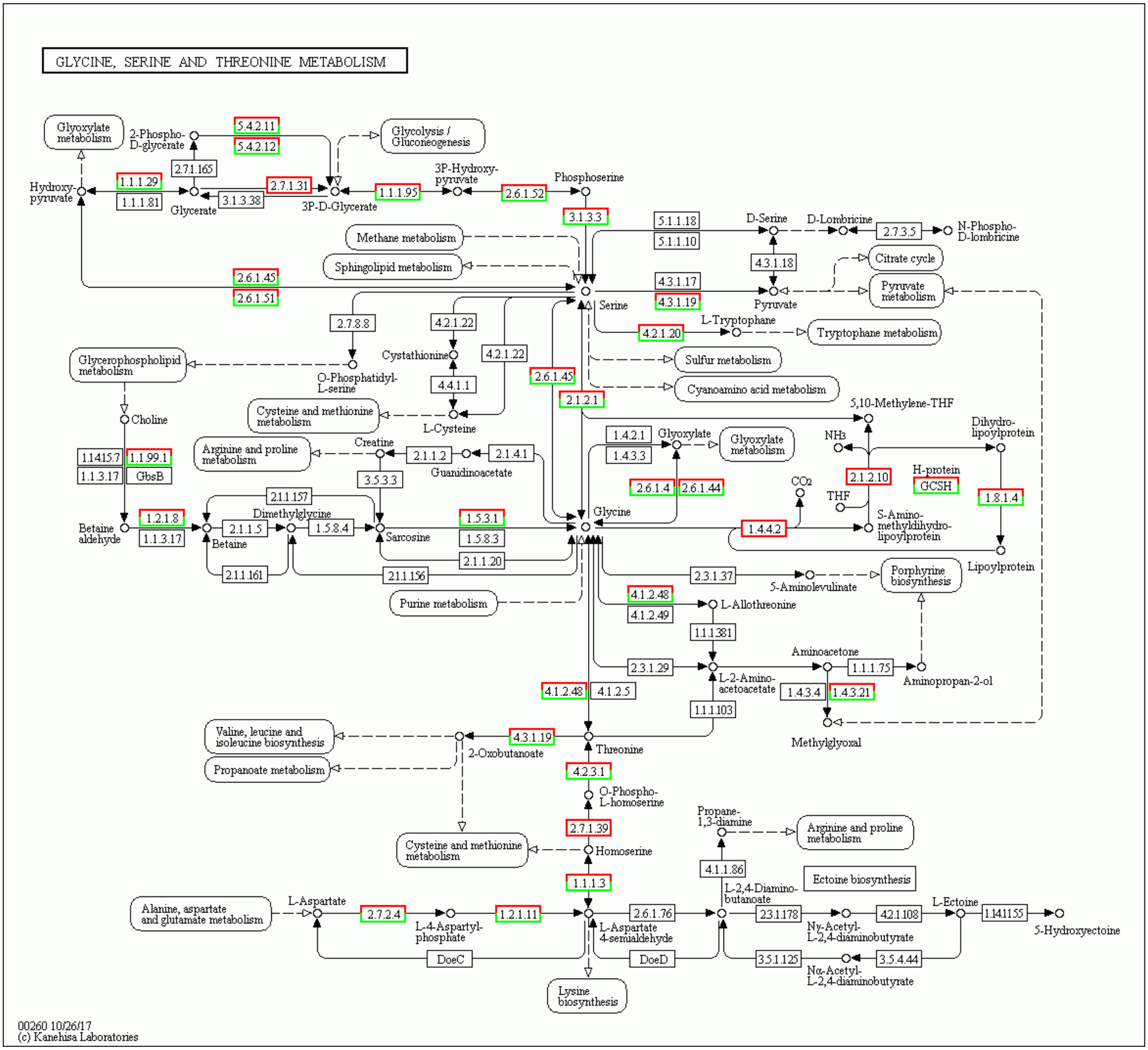
GLYCINE, SERINE AND THREONINE METABOLISM
Glyoxylate metabolism
2-Phospho-D-glycerate
5.4.2.11
5.4.2.12
Glycolysis / Gluconeogenesis
2.7.1.165
1.1.1.29
1.1.1.81
2.7.1.31
3.1.3.38
1.1.1.95
3P-Hydroxy-pyruvate
2.6.1.52
Phosphoserine
Hydroxy-pyruvate
Glycerate
3P-D-Glycerate
3.1.3.3
5.1.1.18
5.1.1.10
D-Serine
D-Lombricine
2.7.3.5
N-Phospho-D-lombricine
Methane metabolism
Sphingolipid metabolism
4.3.1.18
Citrate cycle
2.6.1.45
2.6.1.51
4.3.1.17
4.3.1.19
Serine
Pyruvate
Pyruvate metabolism
2.7.8.8
4.2.1.22
4.2.1.22
4.2.1.20
L-Tryptophane
Tryptophane metabolism
Glycerophospholipid metabolism
O-Phosphatidyl-L-serine
Cystathionine
2.6.1.45
Sulfur metabolism
4.4.1.1
2.1.2.1
Cyanoamino acid metabolism
Choline
Cysteine and methionine metabolism
L-Cysteine
5,10-Methylene-THF
Creatine
Arginine and proline metabolism
2.1.1.2
2.1.4.1
Guanidinoacetate
1.4.2.1
1.4.3.3
Glyoxylate
Glyoxylate metabolism
NH3
Dihydro-lipoylprotein
1.1.4.15.7
1.1.99.1
1.1.3.17
GbsB
3.5.3.3
2.1.2.10
H-protein
GCSH
2.1.1.157
2.6.1.4
2.6.1.44
CO2
THF
1.8.1.4
Dimethylglycine
1.2.1.8
1.5.3.1
Glycine
Betaine aldehyde
2.1.1.5
1.5.8.4
Sarcosine
1.5.8.3
1.4.4.2
S-Amino-methyldihydro-lipoylprotein
1.1.3.17
Betaine
2.1.1.20
Lipoylprotein
2.1.1.161
2.1.1.156
2.3.1.37
5-Aminolevulinate
Porphyrine biosynthesis
Purine metabolism
4.1.2.48
4.1.2.49
L-Allothreonine
1.1.1.381
Aminoacetone
2.3.1.29
1.1.1.75
L-2-Amino-acetoacetate
Aminopropan-2-ol
4.1.2.48
4.1.2.5
1.4.3.4
1.4.3.21
1.1.1.103
Methylglyoxal
Valine, leucine and isoleucine biosynthesis
4.3.1.19
Threonine
2-Oxobutanoate
Propanoate metabolism
4.2.3.1
O-Phospho-L-homoserine
2.7.1.39
Propane-1,3-diamine
Arginine and proline metabolism
Cysteine and methionine metabolism
Homoserine
4.1.1.86
Ectoine biosynthesis
1.1.1.3
L-2,4-Diamino-butanoate
Alanine, aspartate and glutamate metabolism
L-Aspartate
2.7.2.4
1.2.1.11
2.6.1.76
2.3.1.178
4.2.1.108
L-Ectoine
1.14.11.55
5-Hydroxyectoine
L-4-Aspartyl-phosphate
L-Aspartate 4-semialdehyde
Nγ-Acetyl-L-2,4-diaminobutyrate
DoeC
DoeD
3.5.1.125
3.5.4.44
Nα-Acetyl-L-2,4-diaminobutyrate
Lysine biosynthesis
00260 10/26/17
(c) Kanehisa Laboratories

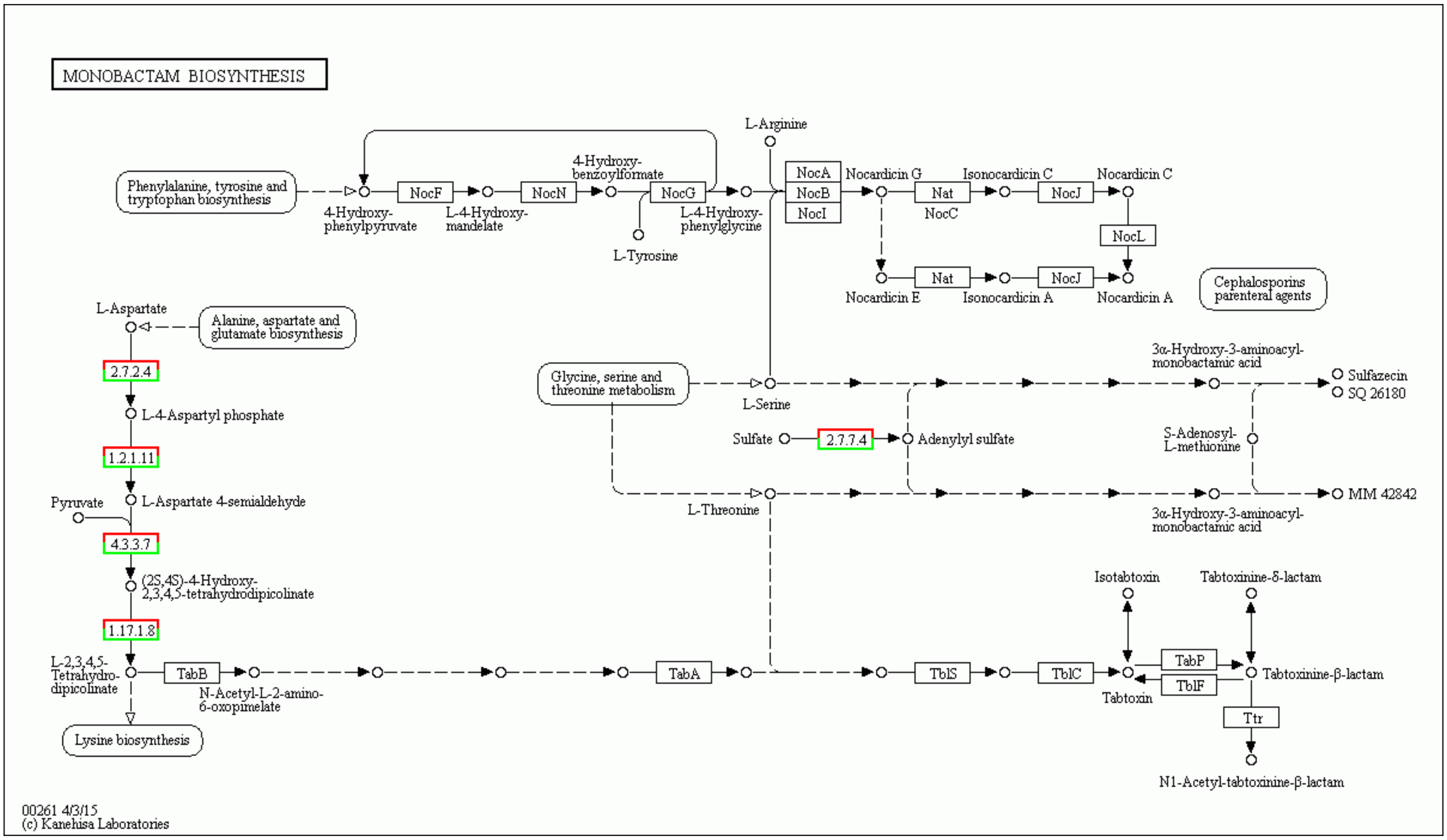
MONOBACTAM BIOSYNTHESIS
Phenylalanine, tyrosine and tryptophan biosynthesis
4-Hydroxy-phenylpyruvate
NocF
L-4-Hydroxy-mandelate
NocN
4-Hydroxy-benzoylformate
NocG
L-Tyrosine
L-4-Hydroxy-phenylglycine
L-Arginine
NocA
NocB
NocI
Nocardicin G
Nat
NocC
Isonocardicin C
NocJ
Nocardicin C
NocL
Nocardicin E
Isonocardicin A
Nocardicin A
Cephalosporins parenteral agents
L-Aspartate
Alanine, aspartate and glutamate biosynthesis
2.7.2.4
L-4-Aspartyl phosphate
1.2.1.11
Pyruvate
L-Aspartate 4-semialdehyde
4.3.3.7
(2S,4S)-4-Hydroxy-2,3,4,5-tetrahydrodipicolinate
1.17.1.8
L-2,3,4,5-Tetrahydro-dipicolinate
TabB
N-Acetyl-L-2-amino-6-oxopimelate
Lysine biosynthesis
Glycine, serine and threonine metabolism
L-Serine
Sulfate
2.7.7.4
Adenylyl sulfate
3α-Hydroxy-3-aminoacyl-monobactamic acid
Sulfazecin
SQ 26180
S-Adenosyl-L-methionine
L-Threonine
3α-Hydroxy-3-aminoacyl-monobactamic acid
MM 42842
TabA
TblS
TblC
Isotabtoxin
Tabtoxinine-δ-lactam
TabP
TblF
Tabtoxin
Tabtoxinine-β-lactam
Ttr
N1-Acetyl-tabtoxinine-β-lactam
00261 4/3/15
(c) Kanehisa Laboratories

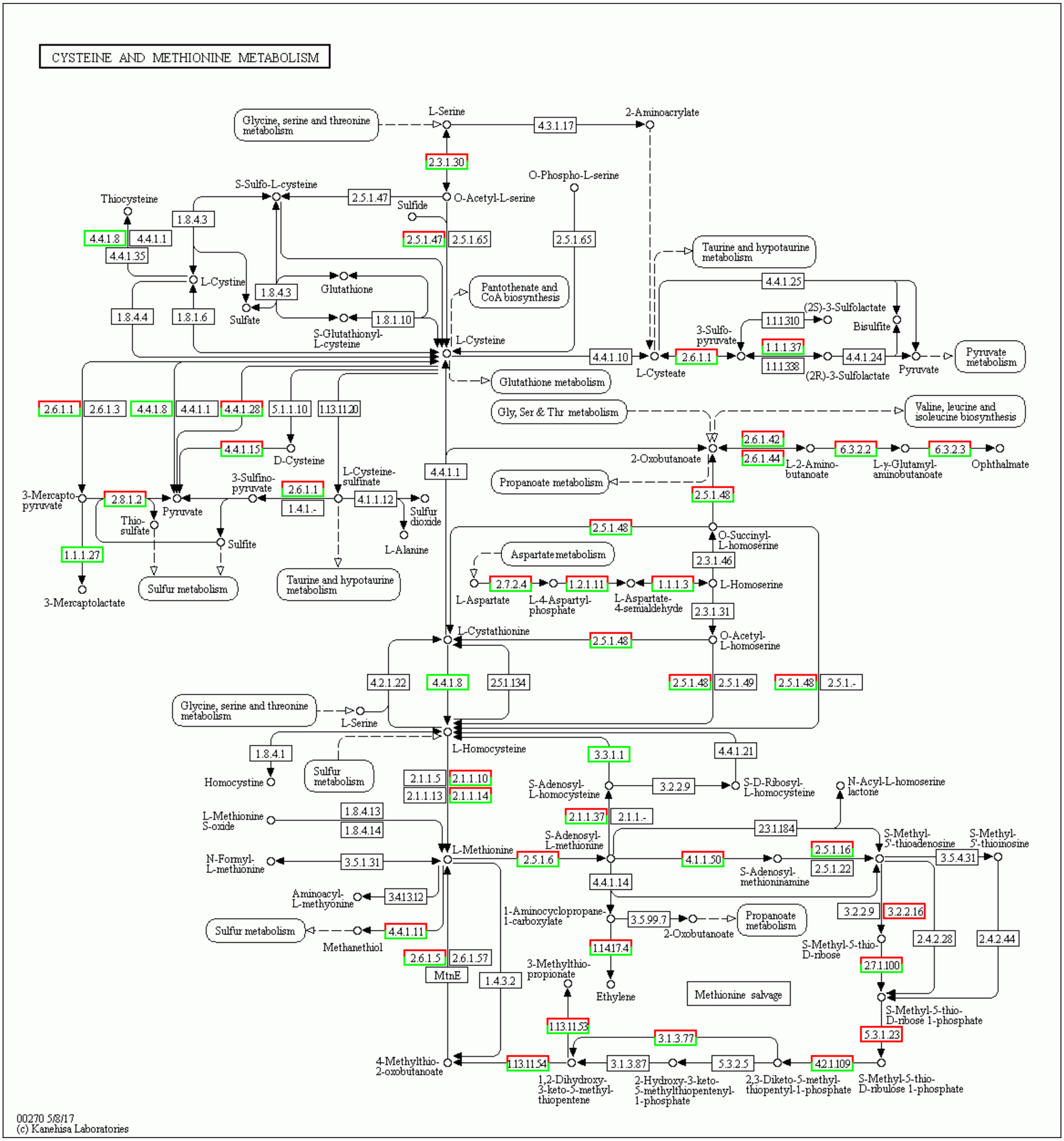
CYSTEINE AND METHIONINE METABOLISM
L-Serine
2-Aminoacrylate
4.3.1.17
Glycine, serine and threonine metabolism
2.3.1.30
O-Phospho-L-serine
S-Sulfo-L-cysteine
2.5.1.47
O-Acetyl-L-serine
Sulfide
Thiocysteine
1.8.4.3
4.4.1.8
4.4.1.1
4.4.1.35
2.5.1.47
2.5.1.65
2.5.1.65
Taurine and hypotaurine metabolism
L-Cystine
Glutathione
1.8.4.3
Pantothenate and CoA biosynthesis
4.4.1.25
Sulfate
1.8.4.4
1.8.1.6
S-Glutathionyl-L-cysteine
1.8.1.10
L-Cysteine
3-Sulfo-pyruvate
1.1.1.310
(2S)-3-Sulfolactate
Bisulfite
1.1.1.37
1.1.1.338
4.4.1.10
2.6.1.1
4.4.1.24
Pyruvate
Pyruvate metabolism
L-Cysteate
(2R)-3-Sulfolactate
Glutathione metabolism
2.6.1.1
2.6.1.3
4.4.1.8
4.4.1.1
4.4.1.28
5.1.1.10
1.13.11.20
Gly, Ser & Thr metabolism
Valine, leucine and isoleucine biosynthesis
4.4.1.15
D-Cysteine
2-Oxobutanoate
2.6.1.42
2.6.1.44
6.3.2.2
6.3.2.3
L-2-Amino-butanoate
L-γ-Glutamyl-aminobutanoate
Ophthalmate
4.4.1.1
L-Cysteine-sulfinate
Propanoate metabolism
3-Sulfino-pyruvate
2.6.1.1
3-Mercapto-pyruvate
2.8.1.2
Pyruvate
1.4.1.-
4.1.1.12
Sulfur dioxide
2.5.1.48
Thio-sulfate
Sulfite
L-Alanine
O-Succinyl-L-homoserine
1.1.1.27
Aspartate metabolism
2.3.1.46
Sulfur metabolism
Taurine and hypotaurine metabolism
2.7.2.4
1.2.1.11
1.1.1.3
L-Homoserine
3-Mercaptolactate
L-Aspartate
L-4-Aspartyl-phosphate
L-Aspartate-4-semialdehyde
2.3.1.31
L-Cystathionine
2.5.1.48
O-Acetyl-L-homoserine
4.2.1.22
4.4.1.8
2.5.1.134
2.5.1.48
2.5.1.49
2.5.1.48
2.5.1.-
Glycine, serine and threonine metabolism
L-Serine
L-Homocysteine
1.8.4.1
Sulfur metabolism
3.3.1.1
4.4.1.21
Homocystine
2.1.1.5
2.1.1.10
2.1.1.13
2.1.1.14
S-Adenosyl-L-homocysteine
3.2.2.9
S-D-Ribosyl-L-homocysteine
N-Acyl-L-homoserine lactone
L-Methionine S-oxide
1.8.4.13
1.8.4.14
2.1.1.37
2.1.1.-
2.3.1.184
N-Formyl-L-methionine
3.5.1.31
L-Methionine
2.5.1.6
S-Adenosyl-L-methionine
4.1.1.50
S-Adenosyl-methioninamine
2.5.1.16
2.5.1.22
S-Methyl-5'-thioadenosine
3.5.4.31
S-Methyl-5'-thioinosine
Aminoacyl-L-methionine
3.4.13.12
4.4.1.14
1-Aminocyclopropane-1-carboxylate
3.5.99.7
2-Oxobutanoate
Propanoate metabolism
3.2.2.9
3.2.2.16
Sulfur metabolism
Methanethiol
4.4.1.11
1.14.17.4
S-Methyl-5-thio-D-ribose
2.4.2.28
2.4.2.44
2.6.1.5
2.6.1.57
3-Methylthio-propionate
2.7.1.100
MtnE
1.4.3.2
Ethylene
Methionine salvage
S-Methyl-5-thio-D-ribose 1-phosphate
1.13.11.53
5.3.1.23
3.1.3.77
4-Methylthio-2-oxobutanoate
1.13.11.54
3.1.3.87
5.3.2.5
4.2.1.109
1,2-Dihydroxy-3-keto-5-methylthiopentene
2-Hydroxy-3-keto-5-methylthiopentenyl-1-phosphate
2,3-Diketo-5-methylthiopentyl-1-phosphate
S-Methyl-5-thio-D-ribulose 1-phosphate
00270 5/8/17
(c) Kanehisa Laboratories

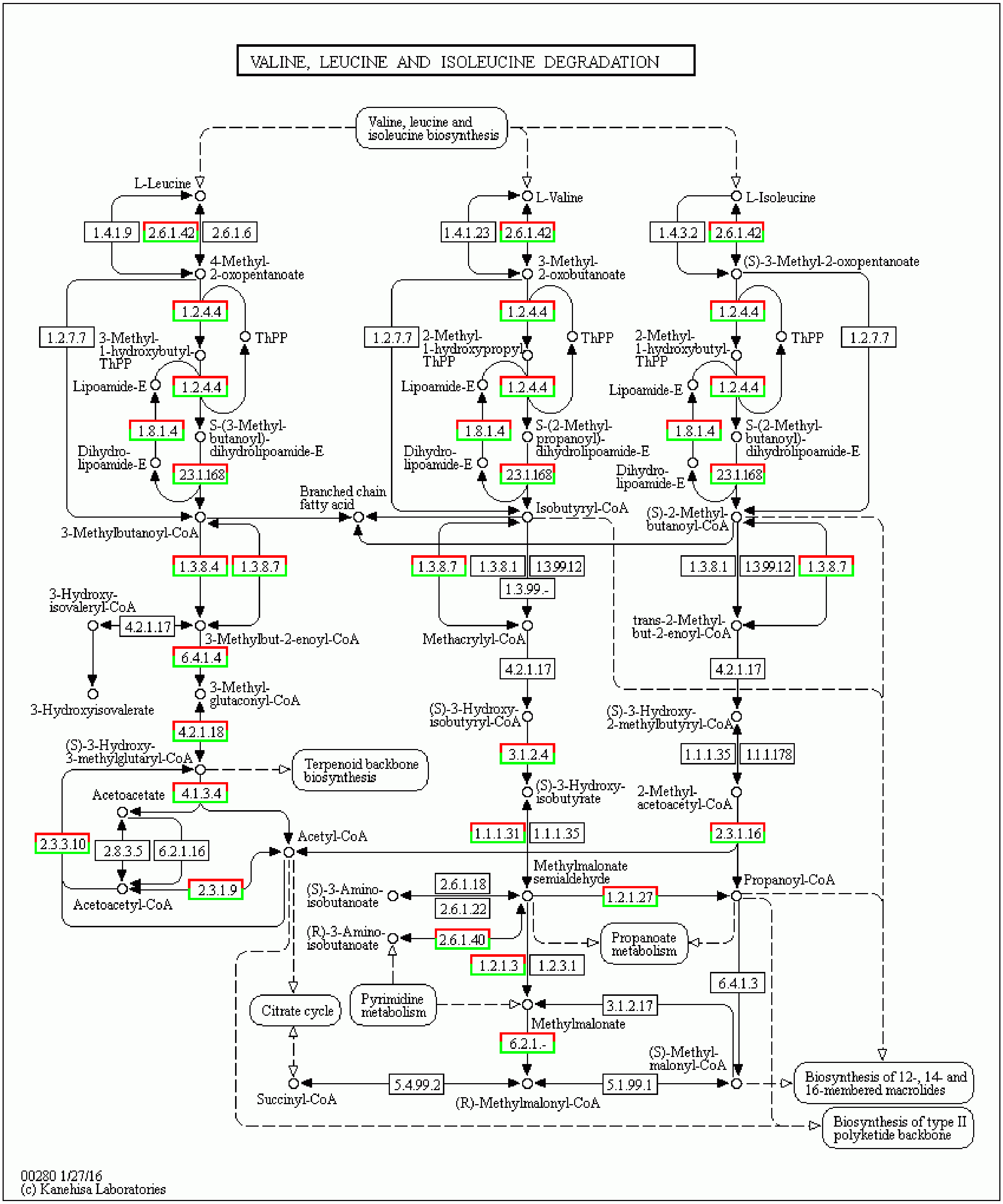
VALINE, LEUCINE AND ISOLEUCINE DEGRADATION
Valine, leucine and isoleucine biosynthesis
L-Leucine
L-Valine
L-Isoleucine
1.4.1.9
2.6.1.42
2.6.1.6
1.4.1.23
1.4.3.2
4-Methyl-2-oxopentanoate
3-Methyl-2-oxobutanoate
(S)-3-Methyl-2-oxopentanoate
1.2.4.4
ThPP
1.2.7.7
3-Methyl-1-hydroxybutyl-ThPP
2-Methyl-1-hydroxypropyl-ThPP
2-Methyl-1-hydroxybutyl-ThPP
Lipoamide-E
1.8.1.4
Dihydro-lipoamide-E
S-(3-Methylbutanoyl)-dihydrolipoamide-E
S-(2-Methylpropanoyl)-dihydrolipoamide-E
S-(2-Methylbutanoyl)-dihydrolipoamide-E
2.3.1.168
Branched chain fatty acid
3-Methylbutanoyl-CoA
Isobutyryl-CoA
(S)-2-Methylbutanoyl-CoA
1.3.8.4
1.3.8.7
1.3.8.1
1.3.99.12
1.3.99.-
3-Hydroxy-isovaleryl-CoA
4.2.1.17
3-Methylbut-2-enoyl-CoA
Methacrylyl-CoA
trans-2-Methyl-but-2-enoyl-CoA
6.4.1.4
3-Hydroxyisovalerate
3-Methyl-glutaconyl-CoA
4.2.1.18
(S)-3-Hydroxy-isobutyryl-CoA
(S)-3-Hydroxy-2-methylbutyryl-CoA
(S)-3-Hydroxy-3-methylglutaryl-CoA
Terpenoid backbone biosynthesis
3.1.2.4
1.1.1.35
1.1.1.178
Acetoacetate
4.1.3.4
(S)-3-Hydroxy-isobutyrate
2-Methyl-acetoacetyl-CoA
2.3.3.10
2.8.3.5
6.2.1.16
Acetyl-CoA
1.1.1.31
2.3.1.16
2.3.1.9
Acetoacetyl-CoA
(S)-3-Amino-isobutanoate
2.6.1.18
2.6.1.22
Methylmalonate semialdehyde
1.2.1.27
Propanoyl-CoA
(R)-3-Amino-isobutanoate
2.6.1.40
Propanoate metabolism
1.2.1.3
1.2.3.1
6.4.1.3
Citrate cycle
Pyrimidine metabolism
3.1.2.17
Methylmalonate
6.2.1.-
(S)-Methyl-malonyl-CoA
Succinyl-CoA
5.4.99.2
(R)-Methylmalonyl-CoA
5.1.99.1
Biosynthesis of 12-, 14- and 16-membered macrolides
Biosynthesis of type II polyketide backbone
00280 1/27/16
(c) Kanehisa Laboratories

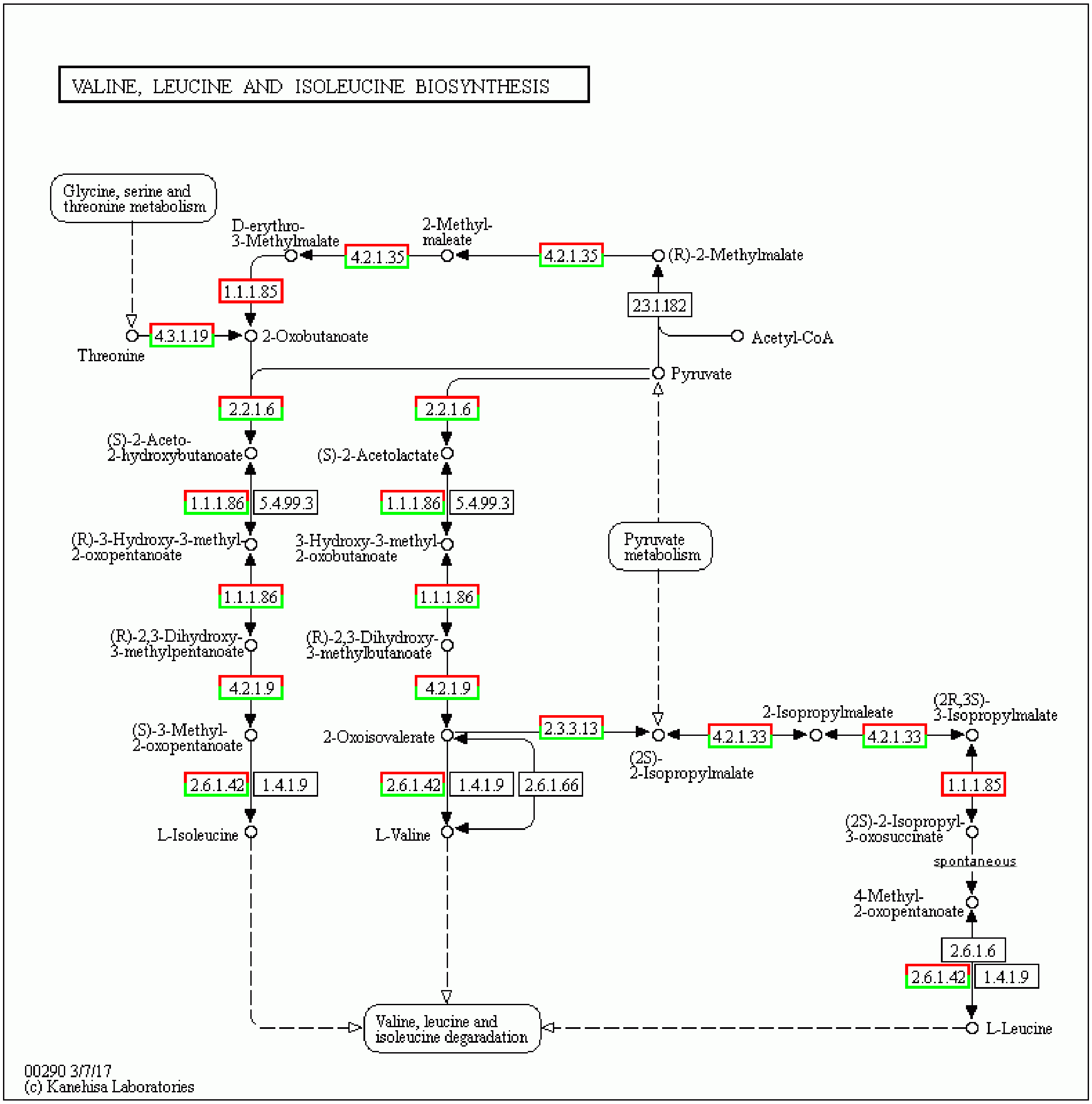
VALINE, LEUCINE AND ISOLEUCINE BIOSYNTHESIS
Glycine, serine and threonine metabolism
D-erythro-3-Methylmalate
2-Methyl-maleate
4.2.1.35
4.2.1.35
(R)-2-Methylmalate
1.1.1.85
2.3.1.182
Threonine
4.3.1.19
2-Oxobutanoate
Acetyl-CoA
Pyruvate
2.2.1.6
2.2.1.6
(S)-2-Aceto-2-hydroxybutanoate
(S)-2-Acetolactate
1.1.1.86
5.4.99.3
1.1.1.86
5.4.99.3
(R)-3-Hydroxy-3-methyl-2-oxopentanoate
3-Hydroxy-3-methyl-2-oxobutanoate
Pyruvate metabolism
1.1.1.86
1.1.1.86
(R)-2,3-Dihydroxy-3-methylpentanoate
(R)-2,3-Dihydroxy-3-methylbutanoate
4.2.1.9
4.2.1.9
(S)-3-Methyl-2-oxopentanoate
2-Oxoisovalerate
2.3.3.13
(2S)-2-Isopropylmalate
4.2.1.33
2-Isopropylmaleate
4.2.1.33
(2R,3S)-3-Isopropylmalate
2.6.1.42
1.4.1.9
2.6.1.42
1.4.1.9
2.6.1.66
1.1.1.85
L-Isoleucine
L-Valine
(2S)-2-Isopropyl-3-oxosuccinate
spontaneous
4-Methyl-2-oxopentanoate
2.6.1.6
2.6.1.42
1.4.1.9
Valine, leucine and isoleucine degaradation
L-Leucine
00290 3/7/17
(c) Kanehisa Laboratories

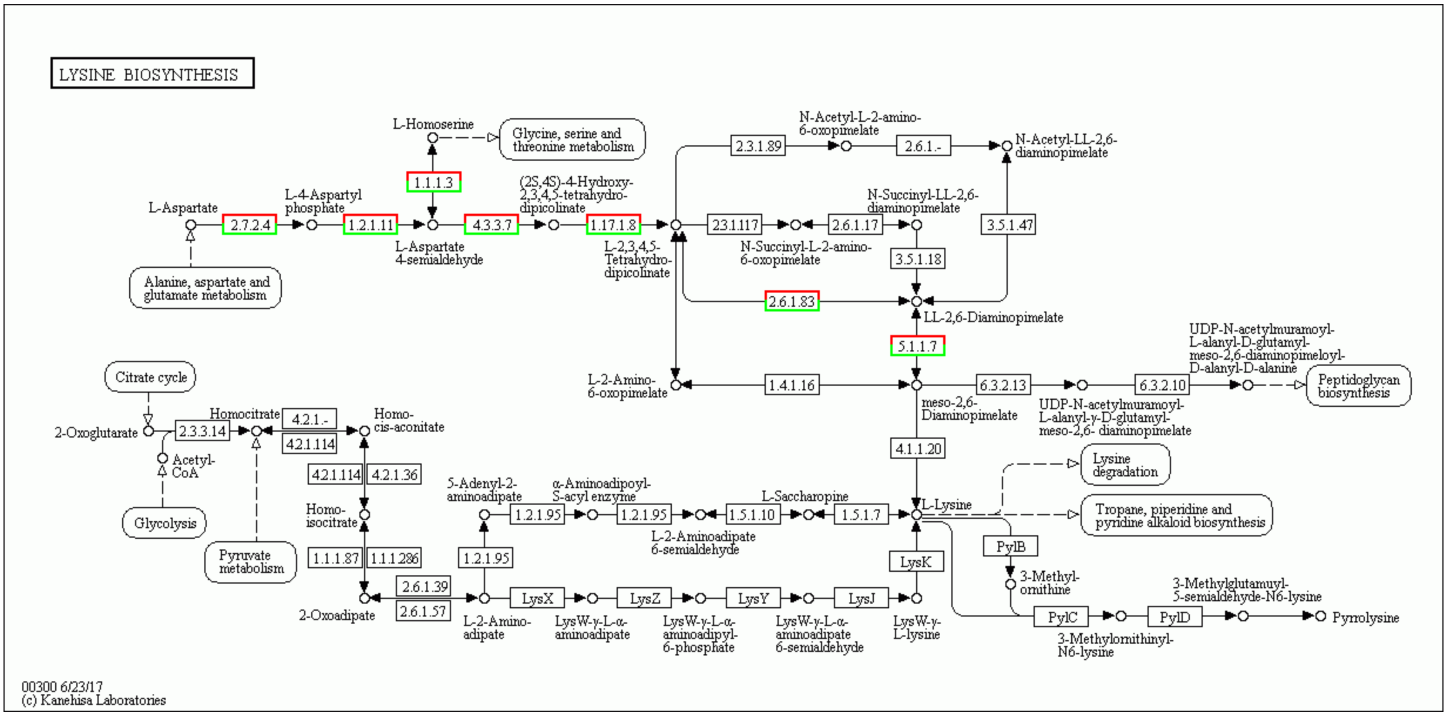
LYSINE BIOSYNTHESIS
L-Homoserine
Glycine, serine and threonine metabolism
N-Acetyl-L-2-amino-6-oxopimelate
2.3.1.89
2.6.1.-
N-Acetyl-LL-2,6-diaminopimelate
1.1.1.3
(2S,4S)-4-Hydroxy-2,3,4,5-tetrahydro-dipicolinate
N-Succinyl-LL-2,6-diaminopimelate
L-Aspartate
L-4-Aspartyl phosphate
2.7.2.4
1.2.1.11
4.3.3.7
1.17.1.8
2.3.1.117
2.6.1.17
3.5.1.47
L-Aspartate 4-semialdehyde
L-2,3,4,5-Tetrahydro-dipicolinate
N-Succinyl-L-2-amino-6-oxopimelate
3.5.1.18
Alanine, aspartate and glutamate metabolism
2.6.1.83
LL-2,6-Diaminopimelate
5.1.1.7
UDP-N-acetylmuramoyl-L-alanyl-D-glutamyl-meso-2,6-diaminopimeloyl-D-alanyl-D-alanine
Citrate cycle
L-2-Amino-6-oxopimelate
1.4.1.16
6.3.2.13
6.3.2.10
Peptidoglycan biosynthesis
meso-2,6-Diaminopimelate
UDP-N-acetylmuramoyl-L-alanyl-γ-D-glutamyl-meso-2,6- diaminopimelate
Homocitrate
4.2.1.-
Homo-cis-aconitate
2-Oxoglutarate
2.3.3.14
4.2.1.114
4.1.1.20
Acetyl-CoA
4.2.1.114
4.2.1.36
Lysine degradation
5-Adenyl-2-aminoadipate
α-Aminoadipoyl-S-acyl enzyme
L-Saccharopine
L-Lysine
Glycolysis
Homo-isocitrate
1.2.1.95
1.2.1.95
1.5.1.10
1.5.1.7
Tropane, piperidine and pyridine alkaloid biosynthesis
L-2-Aminoadipate 6-semialdehyde
PylB
Pyruvate metabolism
1.1.1.87
1.1.1.286
1.2.1.95
LysK
3-Methyl-ornithine
2.6.1.39
3-Methylglutamuyl-5-semialdehyde-N6-lysine
2.6.1.57
LysX
LysZ
LysY
LysJ
PylC
PylD
Pyrrolysine
2-Oxoadipate
L-2-Amino-adipate
LysW-γ-L-α-aminoadipate
LysW-γ-L-α-aminoadipyl-6-phosphate
LysW-γ-L-α-aminoadipate 6-semialdehyde
LysW-γ-L-lysine
3-Methylornithinyl-N6-lysine
00300 6/23/17
(c) Kanehisa Laboratories

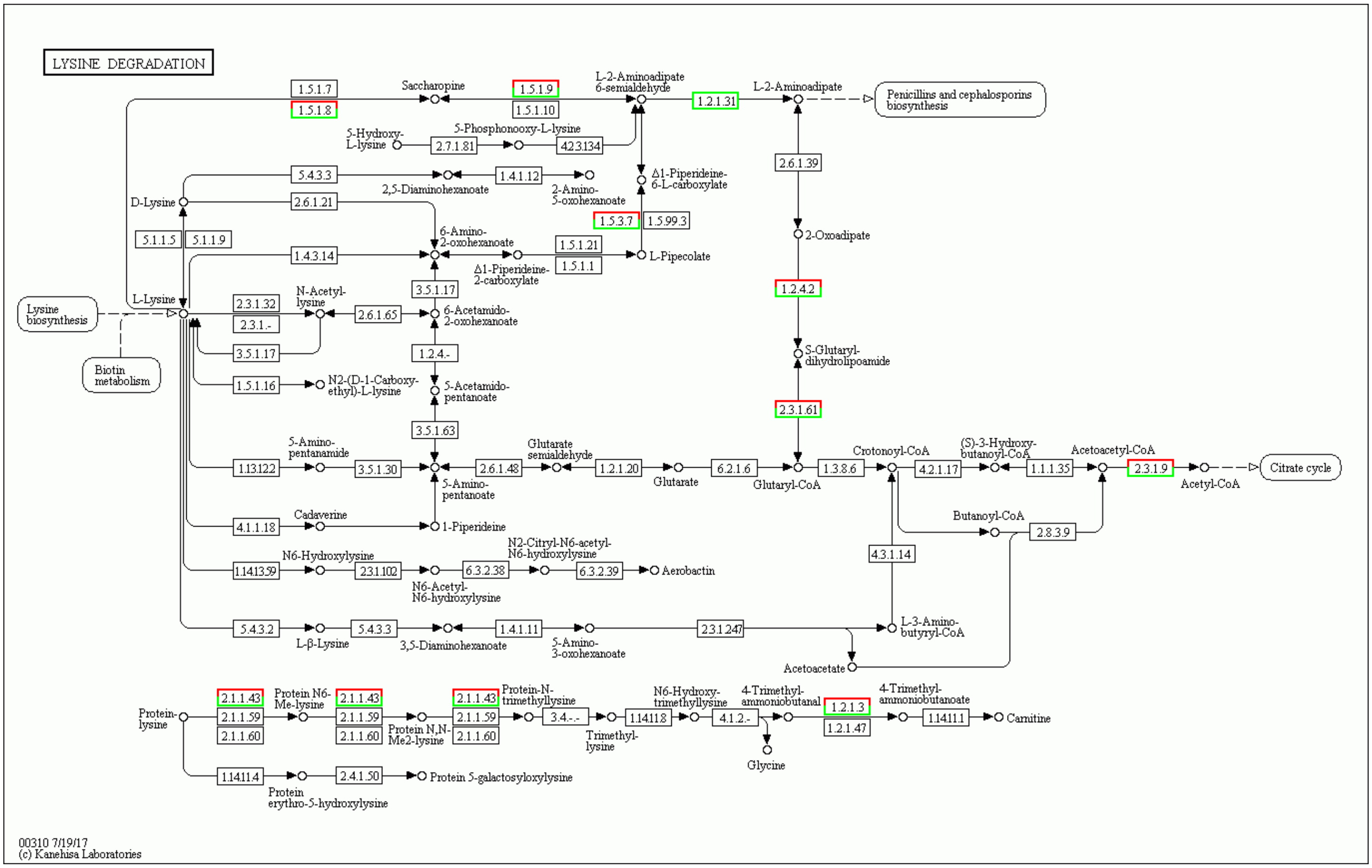
LYSINE DEGRADATION
1.5.1.7
1.5.1.8
Saccharopine
1.5.1.9
1.5.1.10
L-2-Aminoadipate 6-semialdehyde
1.2.1.31
L-2-Aminoadipate
Penicillins and cephalosporins biosynthesis
5-Hydroxy-L-lysine
5-Phosphonooxy-L-lysine
2.7.1.81
4.2.3.134
5.4.3.3
1.4.1.12
Δ1-Piperideine-6-L-carboxylate
2.6.1.39
2,5-Diaminohexanoate
2-Amino-5-oxohexanoate
D-Lysine
2.6.1.21
1.5.3.7
1.5.99.3
2-Oxoadipate
5.1.1.5
5.1.1.9
6-Amino-2-oxohexanoate
1.5.1.21
1.4.3.14
Δ1-Piperideine-2-carboxylate
1.5.1.1
L-Pipecolate
L-Lysine
N-Acetyl-lysine
3.5.1.17
1.2.4.2
Lysine biosynthesis
2.3.1.32
2.6.1.65
6-Acetamido-2-oxohexanoate
2.3.1.-
S-Glutaryl-dihydrolipoamide
3.5.1.17
1.2.4.-
Biotin metabolism
1.5.1.16
N2-(D-1-Carboxy-ethyl)-L-lysine
5-Acetamido-pentanoate
3.5.1.63
2.3.1.61
5-Amino-pentanamide
Glutarate semialdehyde
Crotonoyl-CoA
(S)-3-Hydroxy-butanoyl-CoA
Acetoacetyl-CoA
1.13.12.2
3.5.1.30
2.6.1.48
1.2.1.20
6.2.1.6
1.3.8.6
4.2.1.17
1.1.1.35
2.3.1.9
Citrate cycle
5-Amino-pentanoate
Glutarate
Glutaryl-CoA
Acetyl-CoA
Cadaverine
Butanoyl-CoA
4.1.1.18
1-Piperideine
2.8.3.9
N2-Citryl-N6-acetyl-N6-hydroxylysine
N6-Hydroxylysine
4.3.1.14
1.14.13.59
2.3.1.102
6.3.2.38
6.3.2.39
Aerobactin
N6-Acetyl-N6-hydroxylysine
5.4.3.2
5.4.3.3
1.4.1.11
2.3.1.247
L-3-Amino-butyryl-CoA
L-β-Lysine
3,5-Diaminohexanoate
5-Amino-3-oxohexanoate
Acetoacetate
2.1.1.43
Protein N6-Me-lysine
2.1.1.43
2.1.1.43
Protein-N-trimethyllysine
N6-Hydroxy-trimethyllysine
4-Trimethyl-ammoniobutanal
4-Trimethyl-ammoniobutanoate
Protein-lysine
2.1.1.59
2.1.1.59
2.1.1.59
3.4.-.-
1.14.11.8
4.1.2.-
1.2.1.3
1.14.11.1
Carnitine
2.1.1.60
2.1.1.60
Protein N,N-Me2-lysine
2.1.1.60
Trimethyl-lysine
1.2.1.47
Glycine
1.14.11.4
2.4.1.50
Protein 5-galactosyloxylysine
Protein erythro-5-hydroxylysine
00310 7/19/17
(c) Kanehisa Laboratories

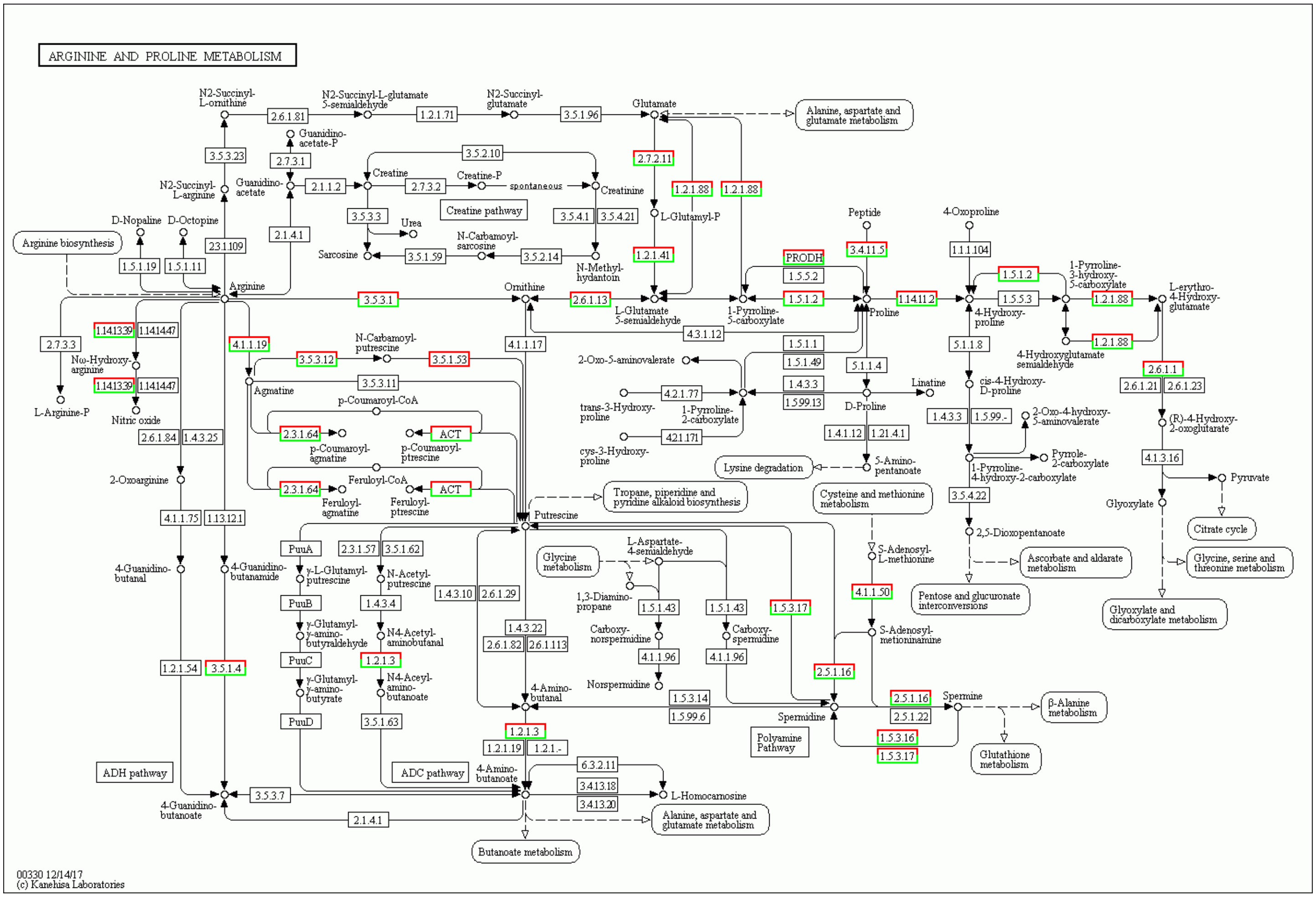
ARGININE AND PROLINE METABOLISM
N2-Succinyl-L-ornithine
2.6.1.81
N2-Succinyl-L-glutamate 5-semialdehyde
1.2.1.71
N2-Succinyl-glutamate
3.5.1.96
Glutamate
Alanine, aspartate and glutamate metabolism
Guanidino-acetate-P
3.5.3.23
2.7.3.1
3.5.2.10
Creatine
Creatine-P
spontaneous
Creatinine
2.7.2.11
N2-Succinyl-L-arginine
Guanidino-acetate
2.1.1.2
2.7.3.2
1.2.1.88
Creatine pathway
3.5.3.3
3.5.4.1
3.5.4.21
L-Glutamyl-P
Peptide
4-Oxoproline
D-Nopaline
D-Octopine
Urea
2.3.1.109
2.1.4.1
N-Carbamoyl-sarcosine
Arginine biosynthesis
Sarcosine
3.5.1.59
3.5.2.14
N-Methyl-hydantoin
1.2.1.41
PRODH
3.4.11.5
1.1.1.104
1.5.1.19
1.5.1.11
1.5.5.2
1-Pyrroline-3-hydroxy-5-carboxylate
Arginine
3.5.3.1
Ornithine
2.6.1.13
L-Glutamate 5-semialdehyde
1-Pyrroline-5-carboxylate
1.5.1.2
Proline
1.14.11.2
4-Hydroxy-proline
1.5.5.3
L-erythro-4-Hydroxy-glutamate
1.14.13.39
1.14.14.47
4.3.1.12
4-Hydroxyglutamate semialdehyde
2.7.3.3
4.1.1.19
N-Carbamoyl-putrescine
4.1.1.17
1.5.1.1
5.1.1.8
2.6.1.1
Nω-Hydroxy-arginine
3.5.3.12
3.5.1.53
2-Oxo-5-aminovalerate
1.5.1.49
5.1.1.4
Agmatine
3.5.3.11
1.4.3.3
Linatine
cis-4-Hydroxy-D-proline
2.6.1.21
2.6.1.23
L-Arginine-P
Nitric oxide
p-Coumaroyl-CoA
trans-3-Hydroxy-proline
4.2.1.77
1-Pyrroline-2-carboxylate
1.5.99.13
D-Proline
2-Oxo-4-hydroxy-5-aminovalerate
(R)-4-Hydroxy-2-oxoglutarate
2.6.1.84
1.4.3.25
2.3.1.64
ACT
p-Coumaroyl-agmatine
p-Coumaroyl-ptrescine
4.2.1.171
1.4.1.12
1.21.4.1
1.4.3.3
1.5.99.-
Pyrrole-2-carboxylate
cys-3-Hydroxy-proline
5-Amino-pentanoate
Lysine degradation
1-Pyrroline-4-hydroxy-2-carboxylate
4.1.3.16
2-Oxoarginine
Feruloyl-CoA
Pyruvate
Feruloyl-agmatine
Feruloyl-ptrescine
Tropane, piperidine and pyridine alkaloid biosynthesis
Cysteine and methionine metabolism
3.5.4.22
Glyoxylate
4.1.1.75
1.13.12.1
Putrescine
2,5-Dioxopentanoate
Citrate cycle
PuuA
2.3.1.57
3.5.1.62
L-Aspartate-4-semialdehyde
Glycine metabolism
S-Adenosyl-L-methionine
Ascorbate and aldarate metabolism
Glycine, serine and threonine metabolism
4-Guanidino-butanal
4-Guanidino-butanamide
γ-L-Glutamyl-putrescine
N-Acetyl-putrescine
1,3-Diamino-propane
Pentose and glucuronate interconversions
PuuB
1.4.3.4
1.4.3.10
2.6.1.29
1.5.1.43
1.5.3.17
4.1.1.50
Glyoxylate and dicarboxylate metabolism
γ-Glutamyl-γ-amino-butyraldehyde
N4-Acetyl-aminobutanal
1.4.3.22
Carboxy-norspermidine
Carboxy-spermidine
S-Adenosyl-metioninamine
2.6.1.82
2.6.1.113
1.2.1.54
3.5.1.4
PuuC
1.2.1.3
4.1.1.96
2.5.1.16
γ-Glutamyl-γ-amino-butyrate
N4-Acetyl-amino-butanoate
Norspermidine
4-Amino-butanal
1.5.3.14
Spermine
β-Alanine metabolism
1.5.99.6
Spermidine
2.5.1.22
PuuD
3.5.1.63
1.5.3.16
Polyamine Pathway
Glutathione metabolism
ADH pathway
1.2.1.19
1.2.1.-
ADC pathway
4-Amino-butanoate
6.3.2.11
3.4.13.18
L-Homocarnosine
3.5.3.7
3.4.13.20
4-Guanidino-butanoate
2.1.4.1
Alanine, aspartate and glutamate metabolism
Butanoate metabolism
00330 12/14/17
(c) Kanehisa Laboratories

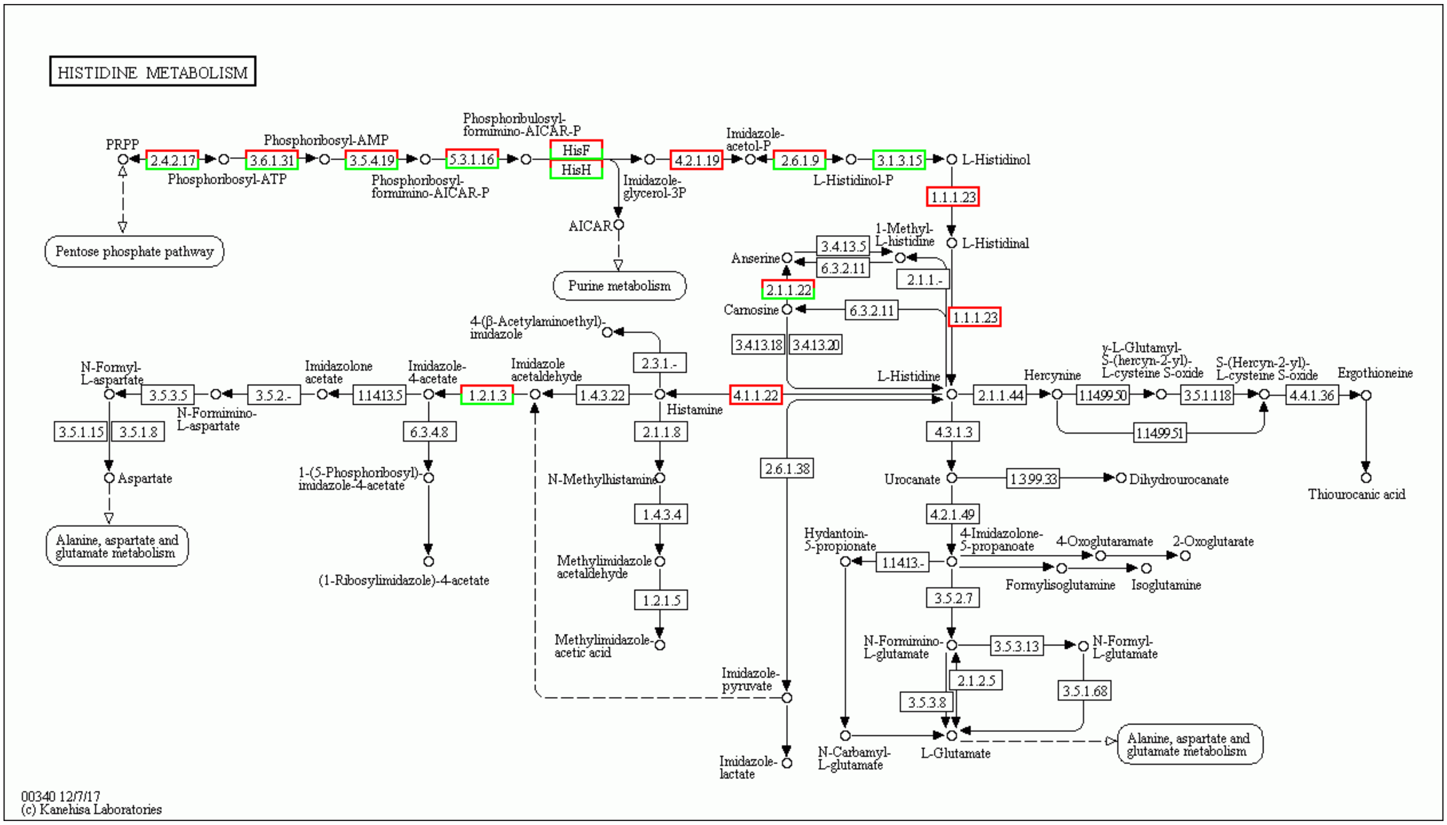
HISTIDINE METABOLISM
Phosphoribulosyl-formimino-AICAR-P
Phosphoribosyl-AMP
Imidazole-acetol-P
PRPP
2.4.2.17
3.6.1.31
3.5.4.19
5.3.1.16
HisF
HisH
4.2.1.19
2.6.1.9
3.1.3.15
L-Histidinol
Phosphoribosyl-ATP
Phosphoribosyl-formimino-AICAR-P
Imidazole-glycerol-3P
L-Histidinol-P
1.1.1.23
AICAR
Pentose phosphate pathway
1-Methyl-L-histidine
3.4.13.5
L-Histidinal
Anserine
6.3.2.11
2.1.1.-
Purine metabolism
2.1.1.22
Carnosine
6.3.2.11
1.1.1.23
4-(β-Acetylaminoethyl)-imidazole
3.4.13.18
3.4.13.20
γ-L-Glutamyl-S-(hercyn-2-yl)-L-cysteine S-oxide
S-(Hercyn-2-yl)-L-cysteine S-oxide
N-Formyl-L-aspartate
Imidazolone acetate
Imidazole-4-acetate
Imidazole acetaldehyde
2.3.1.-
L-Histidine
Hercynine
Ergothioneine
3.5.3.5
3.5.2.-
1.14.13.5
1.2.1.3
1.4.3.22
4.1.1.22
2.1.1.44
1.14.99.50
3.5.1.118
4.4.1.36
N-Formimino-L-aspartate
Histamine
3.5.1.15
3.5.1.8
6.3.4.8
2.1.1.8
4.3.1.3
1.14.99.51
Aspartate
1-(5-Phosphoribosyl)-imidazole-4-acetate
N-Methylhistamine
2.6.1.38
Urocanate
1.3.99.33
Dihydrourocanate
Thiourocanic acid
1.4.3.4
4.2.1.49
Alanine, aspartate and glutamate metabolism
Hydantoin-5-propionate
4-Imidazolone-5-propanoate
4-Oxoglutaramate
2-Oxoglutarate
Methylimidazole acetaldehyde
1.14.13.-
(1-Ribosylimidazole)-4-acetate
Formylisoglutamine
Isoglutamine
1.2.1.5
3.5.2.7
Methylimidazole-acetic acid
N-Formimino-L-glutamate
3.5.3.13
N-Formyl-L-glutamate
Imidazole-pyruvate
2.1.2.5
3.5.1.68
3.5.3.8
N-Carbamyl-L-glutamate
L-Glutamate
Alanine, aspartate and glutamate metabolism
Imidazole-lactate
00340 12/7/17
(c) Kanehisa Laboratories

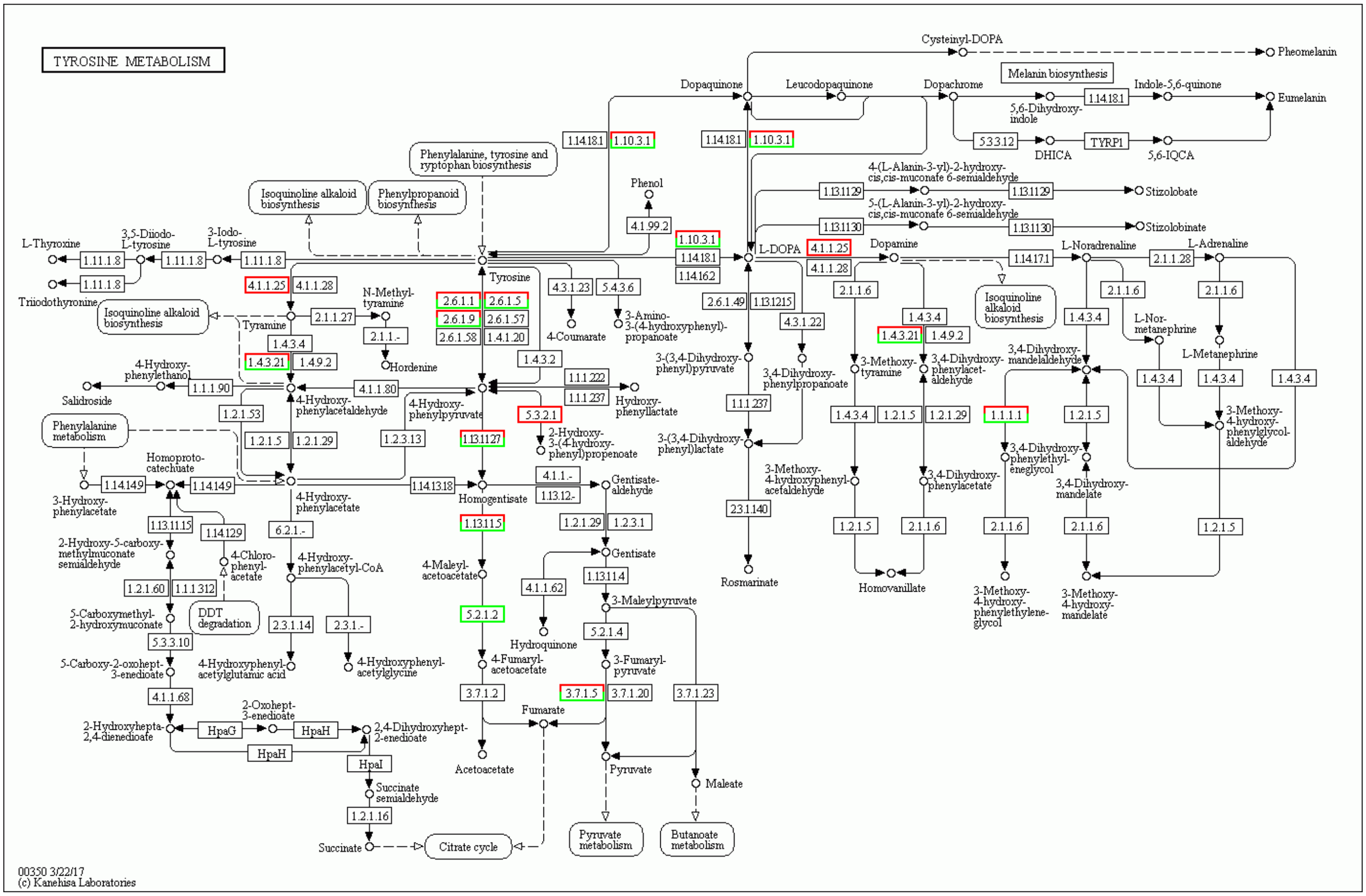
TYROSINE METABOLISM
Cysteinyl-DOPA
Pheomelanin
Melanin biosynthesis
Dopaquinone
Leucodopaquinone
Dopachrome
Indole-5,6-quinone
Eumelanin
5,6-Dihydroxy-indole
DHICA
5,6-IQCA
TYRP1
5.3.3.12
1.14.18.1
1.10.3.1
Phenylalanine, tyrosine and tryptophan biosynthesis
Isoquinoline alkaloid biosynthesis
Phenylpropanoid biosynthesis
Phenol
4.1.99.2
4-(L-Alanin-3-yl)-2-hydroxy-cis,cis-muconate 6-semialdehyde
5-(L-Alanin-3-yl)-2-hydroxy-cis,cis-muconate 6-semialdehyde
1.13.11.29
1.13.11.30
Stizolobate
Stizolobinate
L-DOPA
4.1.1.25
4.1.1.28
Dopamine
1.14.17.1
L-Noradrenaline
L-Adrenaline
2.1.1.28
L-Thyroxine
3,5-Diiodo-L-tyrosine
3-Iodo-L-tyrosine
1.11.1.8
Triiodothyronine
Tyrosine
1.14.16.2
Tyramine
N-Methyl-tyramine
Hordenine
2.1.1.27
2.1.1.-
2.6.1.1
2.6.1.5
2.6.1.9
2.6.1.57
2.6.1.58
1.4.1.20
4.3.1.23
5.4.3.6
4-Coumarate
3-Amino-3-(4-hydroxyphenyl)-propanoate
2.6.1.49
1.13.12.15
4.3.1.22
1.4.3.4
1.4.3.21
1.4.9.2
4-Hydroxy-phenylethanol
Salidroside
1.1.1.90
4.1.1.80
4-Hydroxy-phenylacetaldehyde
4-Hydroxy-phenylpyruvate
1.4.3.2
1.1.1.222
1.1.1.237
Hydroxy-phenyllactate
3-(3,4-Dihydroxy-phenyl)pyruvate
3,4-Dihydroxy-phenylpropanoate
3-Methoxy-tyramine
3,4-Dihydroxy-phenylacet-aldehyde
3,4-Dihydroxy-mandelaldehyde
L-Nor-metanephrine
L-Metanephrine
3-Methoxy-4-hydroxy-phenylglycol-aldehyde
Phenylalanine metabolism
1.2.1.53
1.2.1.5
1.2.1.29
1.2.3.13
1.13.11.27
5.3.2.1
2-Hydroxy-3-(4-hydroxy-phenyl)propenoate
3-(3,4-Dihydroxy-phenyl)lactate
1.1.1.1
2.1.1.6
3,4-Dihydroxy-phenylethyl-eneglycol
Homoproto-catechuate
1.14.14.9
3-Hydroxy-phenylacetate
4-Hydroxy-phenylacetate
1.14.13.18
Homogentisate
4.1.1.-
1.13.12.-
Gentisate-aldehyde
3-Methoxy-4-hydroxyphenyl-acetaldehyde
3,4-Dihydroxy-phenylacetate
3,4-Dihydroxy-mandelate
23.1.140
Rosmarinate
1.13.11.15
1.14.12.9
6.2.1.-
1.13.11.5
1.2.1.29
1.2.3.1
Gentisate
2-Hydroxy-5-carboxy-methylmuconate semialdehyde
4-Chloro-phenyl-acetate
4-Hydroxy-phenylacetyl-CoA
4-Maleyl-acetoacetate
1.13.11.4
4.1.1.62
3-Maleylpyruvate
Homovanillate
3-Methoxy-4-hydroxy-phenylethylene-glycol
3-Methoxy-4-hydroxy-mandelate
1.2.1.60
1.1.1.312
DDT degradation
5-Carboxymethyl-2-hydroxymuconate
2.3.1.14
2.3.1.-
5.2.1.2
Hydroquinone
5.2.1.4
5.3.3.10
5-Carboxy-2-oxohept-3-enedioate
4-Hydroxyphenyl-acetylglutamic acid
4-Hydroxyphenyl-acetylglycine
4-Fumaryl-acetoacetate
3-Fumaryl-pyruvate
3.7.1.2
3.7.1.5
3.7.1.20
3.7.1.23
4.1.1.68
2-Oxohept-3-enedioate
Fumarate
2-Hydroxyhepta-2,4-dienedioate
HpaG
HpaH
2,4-Dihydroxyhept-2-enedioate
HpaI
Acetoacetate
Pyruvate
Maleate
Succinate semialdehyde
1.2.1.16
Succinate
Citrate cycle
Pyruvate metabolism
Butanoate metabolism
00350 3/22/17
(c) Kanehisa Laboratories

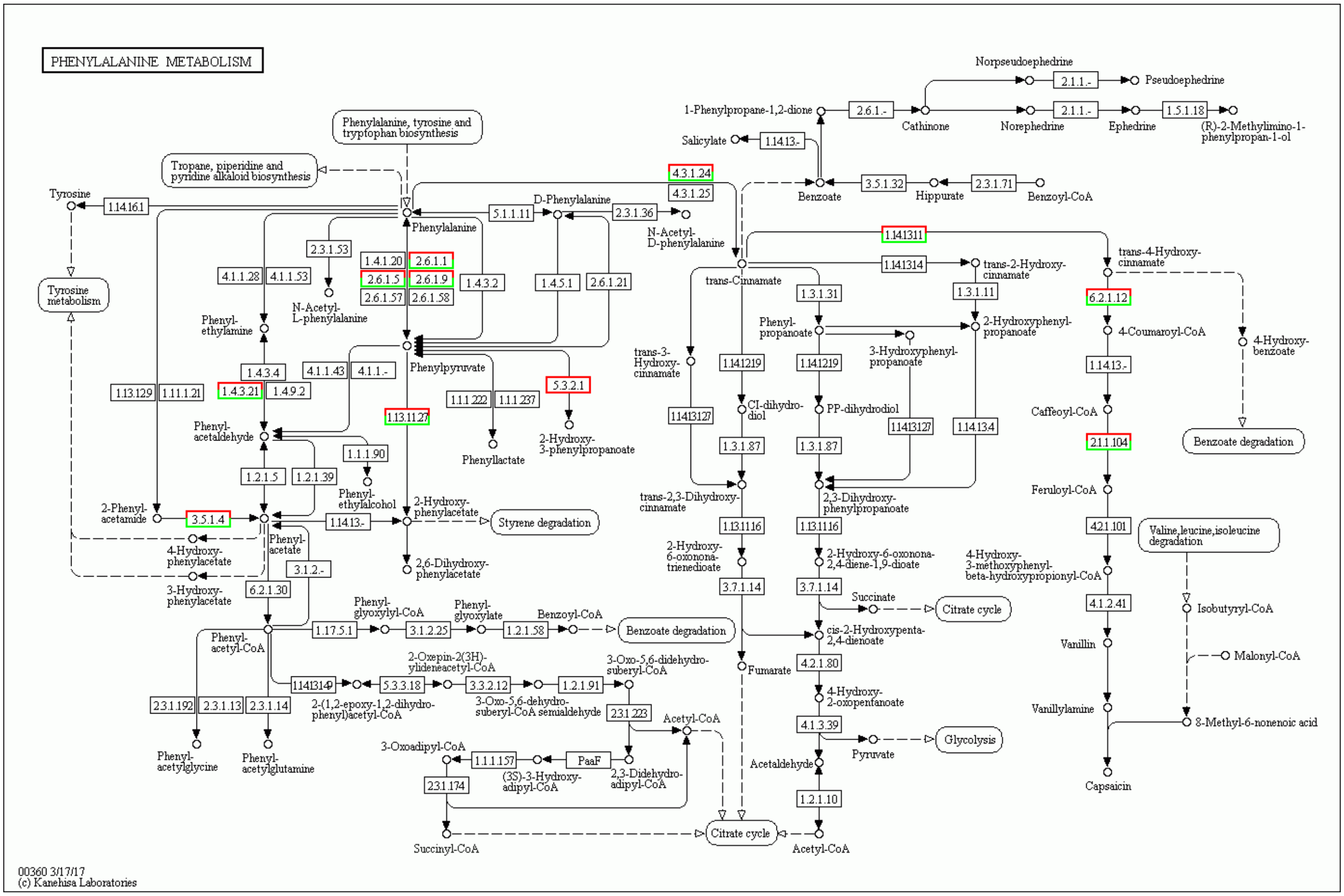
PHENYLALANINE METABOLISM
Norpseudoephedrine
2.1.1.-
Pseudoephedrine
1-Phenylpropane-1,2-dione
2.6.1.-
Cathinone
Norephedrine
Ephedrine
1.5.1.18
(R)-2-Methylimino-1-phenylpropan-1-ol
Phenylalanine, tyrosine and tryptophan biosynthesis
Salicylate
1.14.13.-
Tropane, piperidine and pyridine alkaloid biosynthesis
4.3.1.24
4.3.1.25
Benzoate
3.5.1.32
Hippurate
2.3.1.71
Benzoyl-CoA
Tyrosine
1.14.16.1
D-Phenylalanine
5.1.1.11
2.3.1.36
N-Acetyl-D-phenylalanine
Phenylalanine
1.14.13.11
1.14.13.14
trans-Cinnamate
trans-2-Hydroxy-cinnamate
trans-4-Hydroxy-cinnamate
Tyrosine metabolism
2.3.1.53
4.1.1.28
4.1.1.53
N-Acetyl-L-phenylalanine
1.4.1.20
2.6.1.1
2.6.1.5
2.6.1.9
2.6.1.57
2.6.1.58
1.4.3.2
1.4.5.1
2.6.1.21
1.3.1.31
1.3.1.11
6.2.1.12
Phenyl-ethylamine
Phenyl-propanoate
2-Hydroxyphenyl-propanoate
3-Hydroxyphenyl-propanoate
4-Coumaroyl-CoA
4-Hydroxy-benzoate
trans-3-Hydroxy-cinnamate
Phenylpyruvate
1.4.3.4
4.1.1.43
4.1.1.-
1.13.12.9
1.11.1.21
1.4.3.21
1.4.9.2
5.3.2.1
1.1.1.222
1.1.1.237
1.14.12.19
CI-dihydro-diol
PP-dihydrodiol
1.14.13.-
1.13.11.27
1.14.13.127
Caffeoyl-CoA
Phenyl-acetaldehyde
Phenyllactate
2-Hydroxy-3-phenylpropanoate
1.14.13.4
2.1.1.104
Benzoate degradation
1.1.1.90
1.2.1.5
1.2.1.39
1.3.1.87
Phenyl-ethylalcohol
trans-2,3-Dihydroxy-cinnamate
2,3-Dihydroxy-phenylpropanoate
Feruloyl-CoA
2-Phenyl-acetamide
3.5.1.4
1.14.13.-
2-Hydroxy-phenylacetate
Styrene degradation
1.13.11.16
4.2.1.101
Valine, leucine, isoleucine degradation
4-Hydroxy-phenylacetate
Phenyl-acetate
2,6-Dihydroxy-phenylacetate
2-Hydroxy-6-oxonona-trienedioate
2-Hydroxy-6-oxonona-2,4-diene-1,9-dioate
4-Hydroxy-3-methoxyphenyl-beta-hydroxypropionyl-CoA
3.1.2.-
3-Hydroxy-phenylacetate
6.2.1.30
3.7.1.14
Succinate
Citrate cycle
4.1.2.41
Isobutyryl-CoA
Phenyl-glyoxylyl-CoA
Phenyl-glyoxylate
Benzoyl-CoA
1.17.5.1
3.1.2.25
1.2.1.58
Benzoate degradation
cis-2-Hydroxypenta-2,4-dienoate
Vanillin
Malonyl-CoA
Phenyl-acetyl-CoA
2-Oxepin-2(3H)-ylideneacetyl-CoA
3-Oxo-5,6-didehydro-suberyl-CoA
4.2.1.80
Fumarate
1.14.13.149
5.3.3.18
3.3.2.12
1.2.1.91
4-Hydroxy-2-oxopentanoate
2.3.1.192
2.3.1.13
2.3.1.14
2-(1,2-epoxy-1,2-dihydro-phenyl)acetyl-CoA
3-Oxo-5,6-dehydro-suberyl-CoA semialdehyde
2.3.1.223
Acetyl-CoA
4.1.3.39
Pyruvate
Glycolysis
Vanillylamine
8-Methyl-6-nonenoic acid
3-Oxoadipyl-CoA
1.1.1.157
PaaF
Phenyl-acetylglycine
Phenyl-acetylglutamine
(3S)-3-Hydroxy-adipyl-CoA
2,3-Didehydro-adipyl-CoA
Acetaldehyde
2.3.1.174
1.2.1.10
Capsaicin
Succinyl-CoA
Citrate cycle
Acetyl-CoA
00360 3/17/17
(c) Kanehisa Laboratories

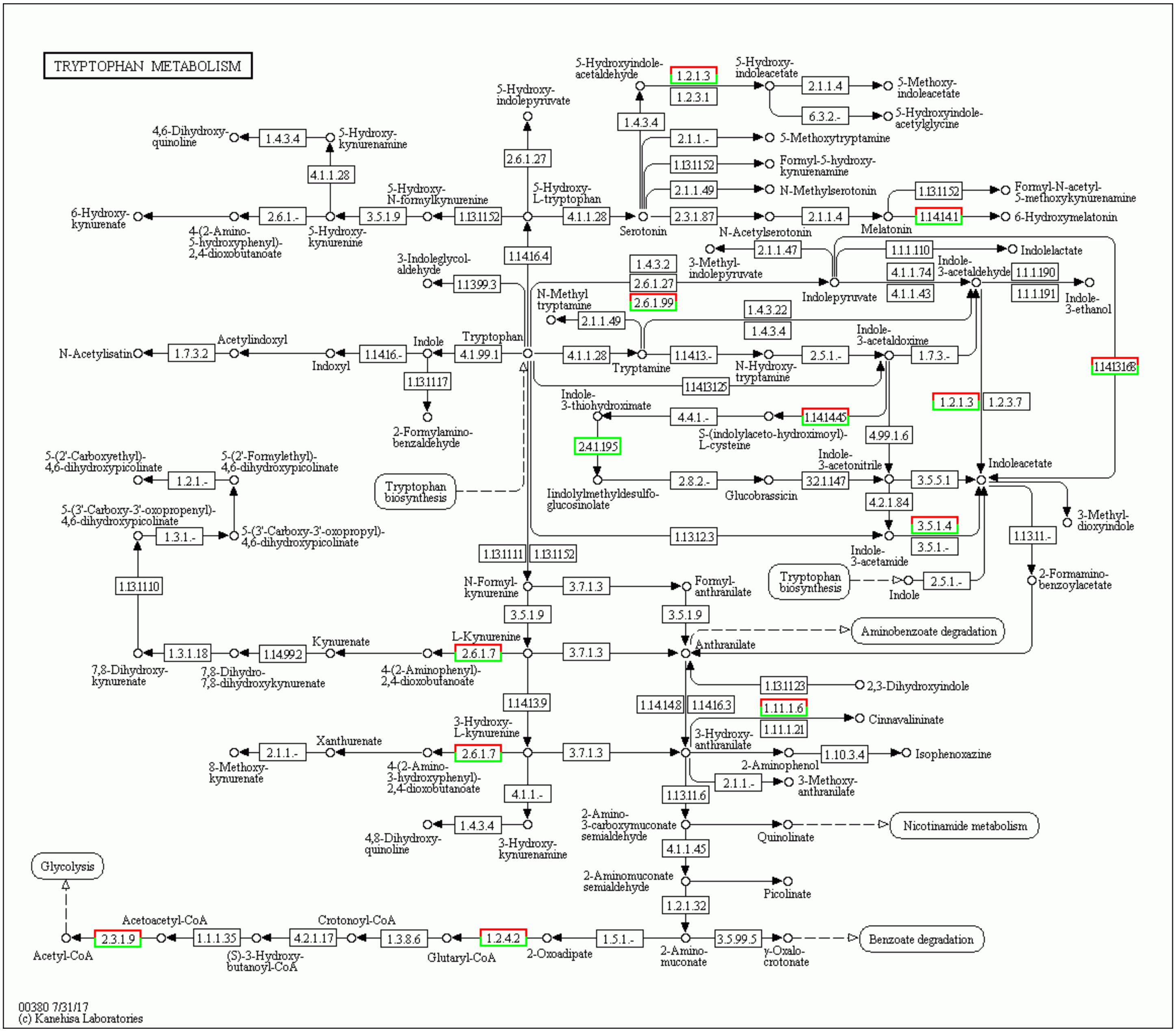
TRYPTOPHAN METABOLISM
5-Hydroxyindole-acetaldehyde
1.2.1.3
1.2.3.1
5-Hydroxy-indoleacetate
2.1.1.4
5-Methoxy-indoleacetate
6.3.2.-
5-Hydroxyindole-acetylglycine
5-Hydroxy-indolepyruvate
1.4.3.4
2.1.1.-
5-Methoxytryptamine
1.13.11.52
Formyl-5-hydroxy-kynurenamine
2.1.1.49
N-Methylserotonin
1.13.11.52
Formyl-N-acetyl-5-methoxykynurenamine
4,6-Dihydroxy-quinoline
1.4.3.4
5-Hydroxy-kynurenamine
4.1.1.28
2.6.1.27
5-Hydroxy-N-formylkynurenine
5-Hydroxy-L-tryptophan
6-Hydroxy-kynurenate
2.6.1.-
4-(2-Amino-5-hydroxyphenyl)-2,4-dioxobutanoate
3.5.1.9
5-Hydroxy-kynurenine
1.13.11.52
4.1.1.28
Serotonin
2.3.1.87
N-Acetylserotonin
2.1.1.4
Melatonin
1.14.14.1
6-Hydroxymelatonin
1.14.16.4
3-Indoleglycol-aldehyde
1.13.99.3
1.4.3.2
2.6.1.27
2.6.1.99
3-Methyl-indolepyruvate
2.1.1.47
1.1.1.110
Indolelactate
Indolepyruvate
4.1.1.74
4.1.1.43
Indole-3-acetaldehyde
1.1.1.190
1.1.1.191
Indole-3-ethanol
N-Methyl tryptamine
2.1.1.49
1.4.3.22
1.4.3.4
N-Acetylisatin
1.7.3.2
Acetylindoxyl
Indoxyl
1.14.16.-
Indole
4.1.99.1
Tryptophan
4.1.1.28
Tryptamine
1.14.13.-
N-Hydroxy-tryptamine
2.5.1.-
Indole-3-acetaldoxime
1.7.3.-
1.13.11.17
1.14.13.125
1.14.13.168
2-Formylamino-benzaldehyde
Indole-3-thiohydroximate
4.4.1.-
1.14.14.45
S-(indolylaceto-hydroximoyl)-L-cysteine
1.2.1.3
1.2.3.7
2.4.1.195
4.99.1.6
5-(2'-Carboxyethyl)-4,6-dihydroxypicolinate
1.2.1.-
5-(2'-Formylethyl)-4,6-dihydroxypicolinate
Tryptophan biosynthesis
Indolylmethyldesulfo-glucosinolate
2.8.2.-
Glucobrassicin
3.2.1.147
Indole-3-acetonitrile
3.5.5.1
Indoleacetate
4.2.1.84
3-Methyl-dioxyindole
5-(3'-Carboxy-3'-oxopropenyl)-4,6-dihydroxypicolinate
1.3.1.-
5-(3'-Carboxy-3'-oxopropyl)-4,6-dihydroxypicolinate
1.13.12.3
3.5.1.4
3.5.1.-
1.13.11.-
Indole-3-acetamide
1.13.11.10
1.13.11.11
1.13.11.52
N-Formyl-kynurenine
3.7.1.3
Formyl-anthranilate
Tryptophan biosynthesis
Indole
2.5.1.-
2-Formamino-benzoylacetate
3.5.1.9
3.5.1.9
Aminobenzoate degradation
Kynurenate
L-Kynurenine
Anthranilate
7,8-Dihydroxy-kynurenate
1.3.1.18
7,8-Dihydro-7,8-dihydroxykynurenate
1.14.99.2
2.6.1.7
4-(2-Aminophenyl)-2,4-dioxobutanoate
3.7.1.3
1.13.11.23
2,3-Dihydroxyindole
1.14.13.9
1.14.14.8
1.14.16.3
1.11.1.6
Cinnavalinate
1.11.1.21
3-Hydroxy-L-kynurenine
Xanthurenate
8-Methoxy-kynurenate
2.1.1.-
2.6.1.7
4-(2-Amino-3-hydroxyphenyl)-2,4-dioxobutanoate
3.7.1.3
3-Hydroxy-anthranilate
2-Aminophenol
1.10.3.4
Isophenoxazine
2.1.1.-
3-Methoxy-anthranilate
4.1.1.-
1.13.11.6
4,8-Dihydroxy-quinoline
1.4.3.4
3-Hydroxy-kynurenamine
2-Amino-3-carboxymuconate semialdehyde
Quinolinate
Nicotinamide metabolism
4.1.1.45
Glycolysis
2-Aminomuconate semialdehyde
Picolinate
1.2.1.32
Acetoacetyl-CoA
Crotonoyl-CoA
Acetyl-CoA
2.3.1.9
1.1.1.35
(S)-3-Hydroxy-butanoyl-CoA
4.2.1.17
1.3.8.6
Glutaryl-CoA
1.2.4.2
2-Oxoadipate
1.5.1.-
2-Amino-muconate
3.5.99.5
γ-Oxalo-crotonate
Benzoate degradation
00380 7/31/17
(c) Kanehisa Laboratories

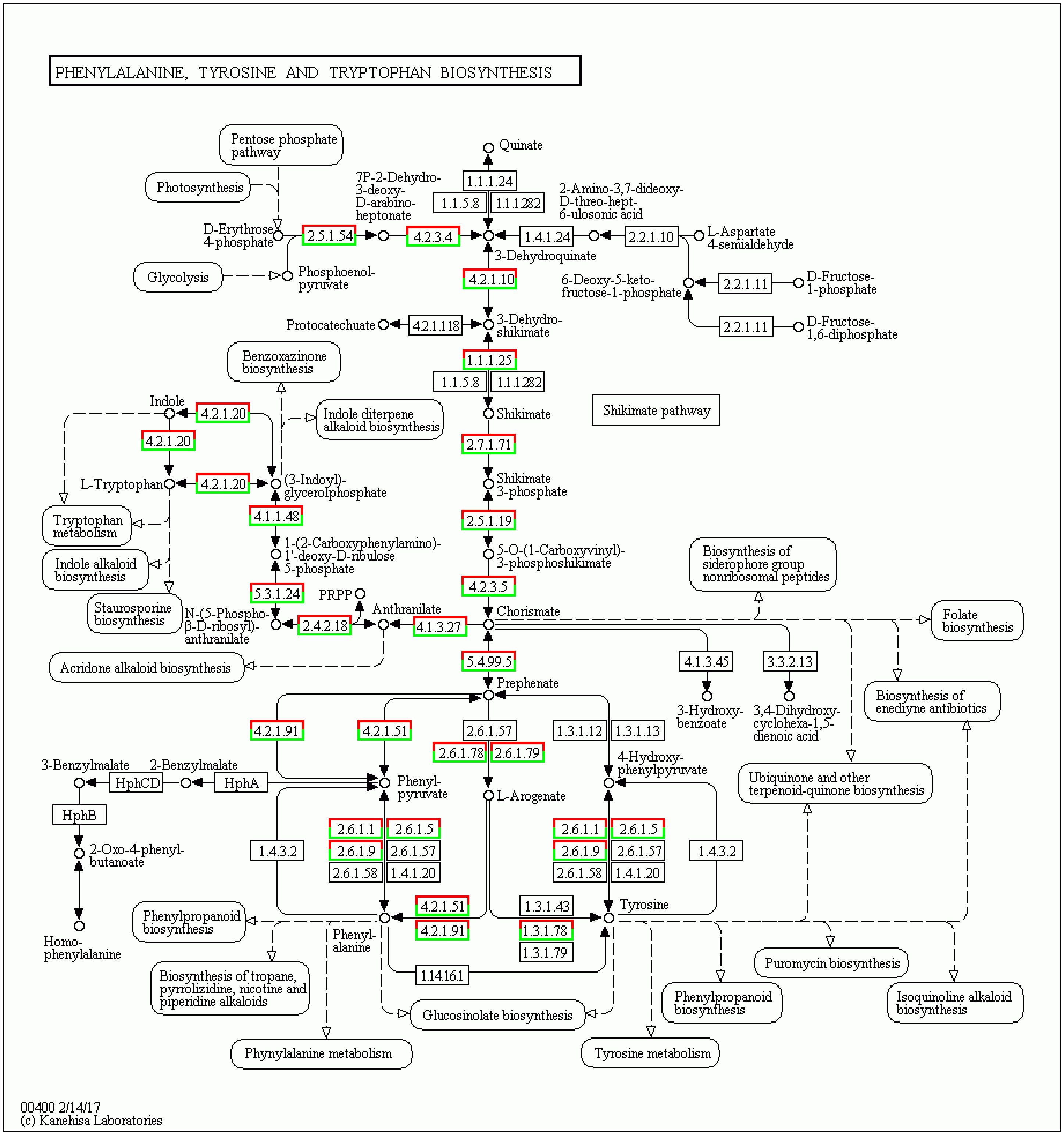
PHENYLALANINE, TYROSINE AND TRYPTOPHAN BIOSYNTHESIS
Pentose phosphate pathway
Photosynthesis
Glycolysis
D-Erythrose 4-phosphate
Phosphoenol-pyruvate
2.5.1.54
7P-2-Dehydro-3-deoxy-D-arabino-heptonate
4.2.3.4
Quinate
1.1.1.24
1.1.5.8
1.1.1.282
2-Amino-3,7-dideoxy-D-threo-hept-6-ulosonic acid
1.4.1.24
2.2.1.10
L-Aspartate 4-semialdehyde
3-Dehydroquinate
4.2.1.10
6-Deoxy-5-keto-fructose-1-phosphate
2.2.1.11
D-Fructose-1-phosphate
D-Fructose-1,6-diphosphate
Protocatechuate
4.2.1.118
3-Dehydro-shikimate
1.1.1.25
Benzoxazinone biosynthesis
Indole
4.2.1.20
Indole diterpene alkaloid biosynthesis
Shikimate
Shikimate pathway
2.7.1.71
L-Tryptophan
(3-Indoyl)-glycerolphosphate
Shikimate 3-phosphate
Tryptophan metabolism
4.1.1.48
2.5.1.19
1-(2-Carboxyphenylamino)-1'-deoxy-D-ribulose 5-phosphate
5-O-(1-Carboxyvinyl)-3-phosphoshikimate
Indole alkaloid biosynthesis
Biosynthesis of siderophore group nonribosomal peptides
5.3.1.24
PRPP
4.2.3.5
Staurosporine biosynthesis
N-(5-Phospho-β-D-ribosyl)-anthranilate
2.4.2.18
Anthranilate
4.1.3.27
Chorismate
Folate biosynthesis
Acridone alkaloid biosynthesis
5.4.99.5
4.1.3.45
3.3.2.13
Prephenate
3-Hydroxy-benzoate
3,4-Dihydroxy-cyclohexa-1,5-dienoic acid
Biosynthesis of enediyne antibiotics
4.2.1.91
4.2.1.51
2.6.1.57
1.3.1.12
1.3.1.13
2.6.1.78
2.6.1.79
3-Benzylmalate
2-Benzylmalate
HphCD
HphA
Phenyl-pyruvate
L-Arogenate
4-Hydroxy-phenylpyruvate
Ubiquinone and other terpenoid-quinone biosynthesis
HphB
2-Oxo-4-phenyl-butanoate
2.6.1.1
2.6.1.5
2.6.1.9
2.6.1.58
1.4.1.20
1.4.3.2
Homo-phenylalanine
Phenylpropanoid biosynthesis
Phenyl-alanine
1.3.1.43
1.3.1.78
1.3.1.79
Tyrosine
1.14.16.1
Puromycin biosynthesis
Biosynthesis of tropane, pyrrolizidine, nicotine and piperidine alkaloids
Glucosinolate biosynthesis
Isoquinoline alkaloid biosynthesis
Phynylalanine metabolism
Tyrosine metabolism
00400 2/14/17
(c) Kanehisa Laboratories

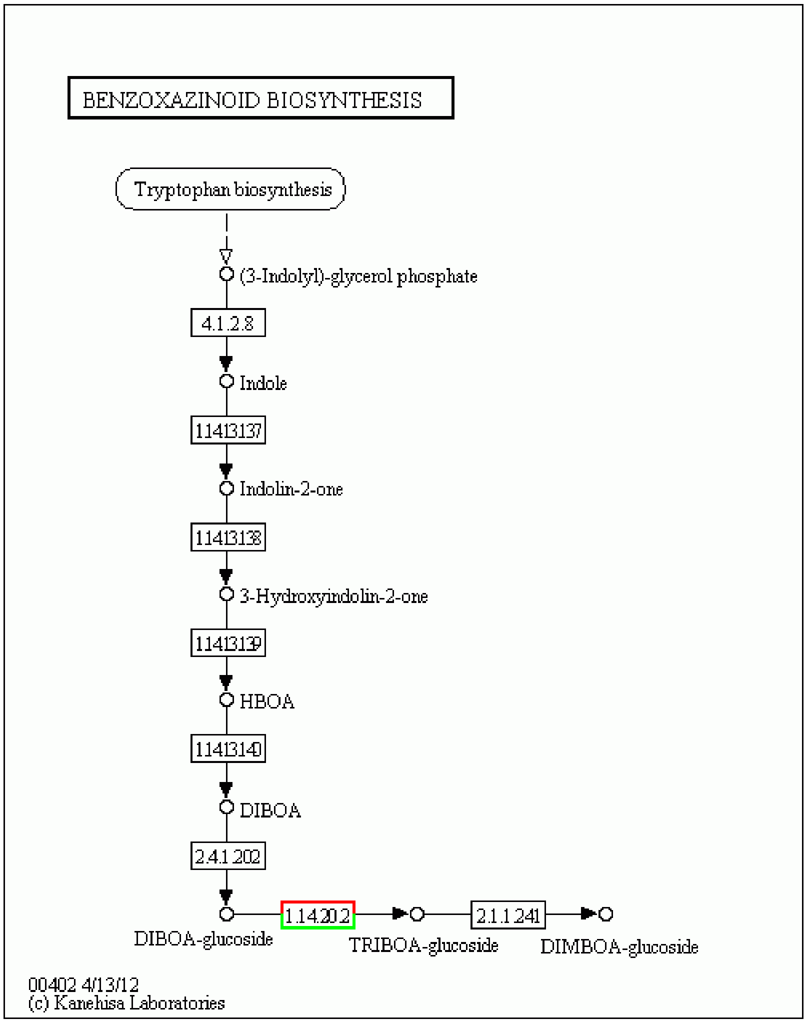
BENZOXAZINOID BIOSYNTHESIS
Tryptophan biosynthesis
(3-Indolyl)-glycerol phosphate
4.1.2.8
Indole
1.14.13.137
Indolin-2-one
1.14.13.138
3-Hydroxyindolin-2-one
1.14.13.139
HBOA
1.14.13.140
DIBOA
2.4.1.202
DIBOA-glucoside
1.14.20.2
TRIBOA-glucoside
2.1.1.241
DIMBOA-glucoside
00402 4/13/12
(c) Kanehisa Laboratories

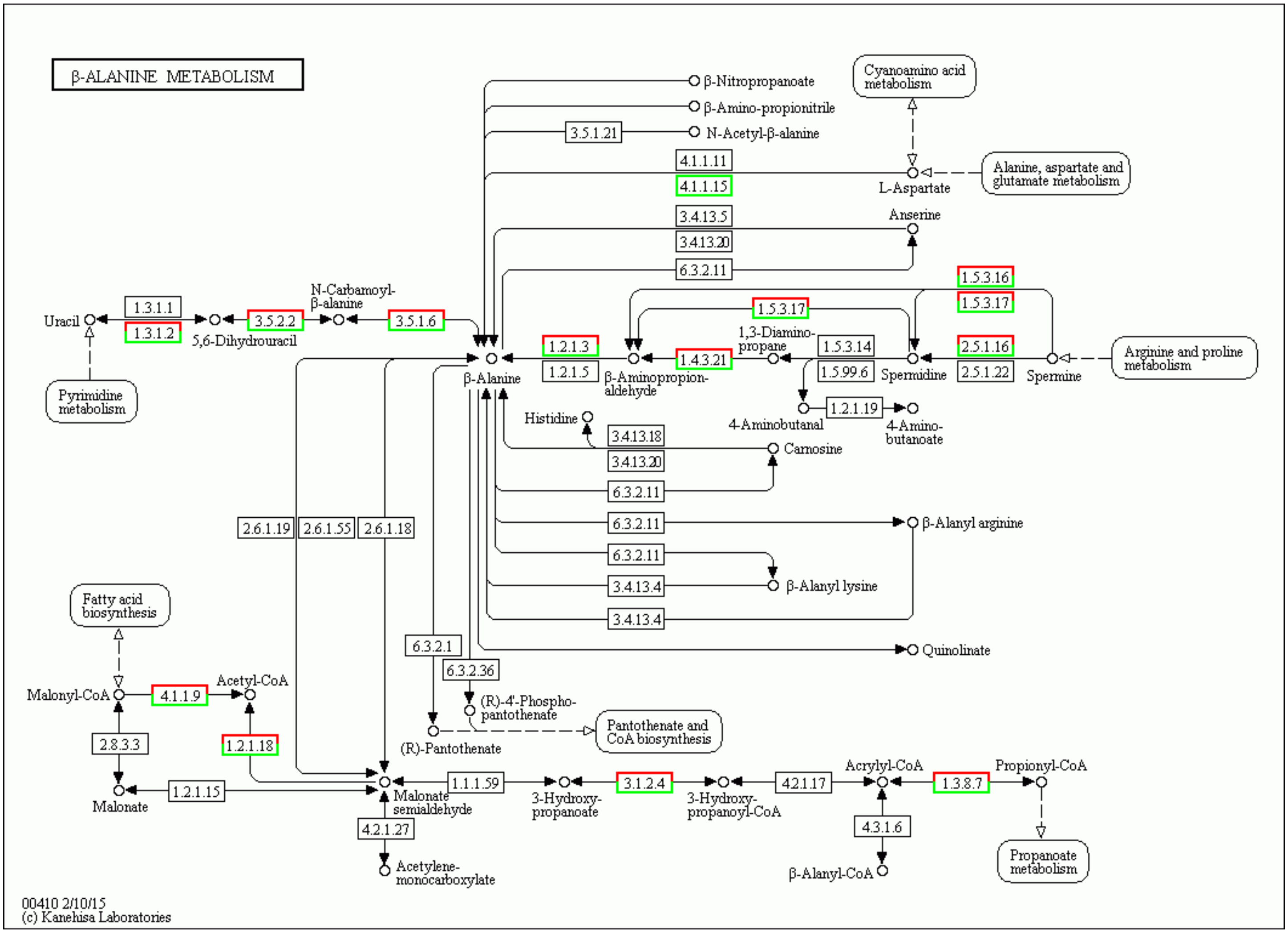
β-ALANINE METABOLISM
β-Nitropropanoate
β-Amino-propionitrile
N-Acetyl-β-alanine
3.5.1.21
Cyanoamino acid metabolism
4.1.1.11
4.1.1.15
L-Aspartate
Alanine, aspartate and glutamate metabolism
3.4.13.5
3.4.13.20
6.3.2.11
Anserine
1.5.3.16
1.5.3.17
N-Carbamoyl-β-alanine
Uracil
1.3.1.1
1.3.1.2
3.5.2.2
3.5.1.6
5,6-Dihydrouracil
1.5.3.17
1,3-Diamino-propane
1.2.1.3
1.2.1.5
1.4.3.21
1.5.3.14
1.5.99.6
2.5.1.16
2.5.1.22
β-Alanine
β-Aminopropion-aldehyde
Spermidine
Spermine
Arginine and proline metabolism
Pyrimidine metabolism
4-Aminobutanal
1.2.1.19
4-Amino-butanoate
Histidine
3.4.13.18
3.4.13.20
Carnosine
6.3.2.11
6.3.2.11
β-Alanyl arginine
2.6.1.19
2.6.1.55
2.6.1.18
6.3.2.11
3.4.13.4
β-Alanyl lysine
3.4.13.4
Fatty acid biosynthesis
6.3.2.1
Quinolinate
6.3.2.36
Malonyl-CoA
4.1.1.9
Acetyl-CoA
(R)-4'-Phospho-pantothenate
Pantothenate and CoA biosynthesis
2.8.3.3
1.2.1.18
(R)-Pantothenate
1.2.1.15
1.1.1.59
3.1.2.4
4.2.1.17
Acrylyl-CoA
1.3.8.7
Propionyl-CoA
Malonate
Malonate semialdehyde
3-Hydroxy-propanoate
3-Hydroxy-propanoyl-CoA
4.2.1.27
4.3.1.6
Acetylene-monocarboxylate
β-Alanyl-CoA
Propanoate metabolism
00410 2/10/15
(c) Kanehisa Laboratories

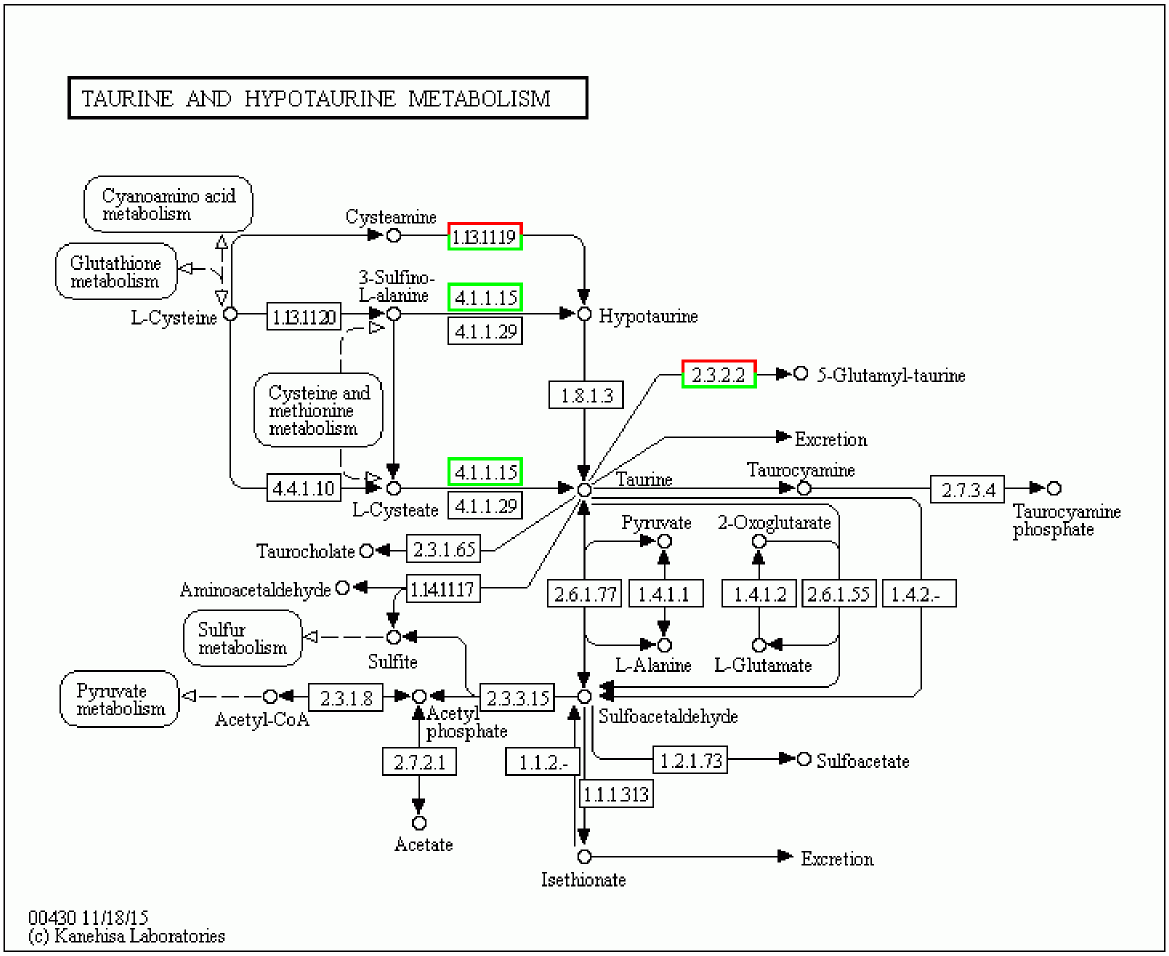
TAURINE AND HYPOTAURINE METABOLISM
Cyanoamino acid metabolism
Glutathione metabolism
Cysteamine
1.13.11.19
3-Sulfino-L-alanine
4.1.1.15
4.1.1.29
L-Cysteine
1.13.11.20
Hypotaurine
2.3.2.2
5-Glutamyl-taurine
1.8.1.3
Cysteine and methionine metabolism
Excretion
4.4.1.10
L-Cysteate
4.1.1.15
4.1.1.29
Taurine
Taurocyamine
2.7.3.4
Taurocyamine phosphate
Taurocholate
2.3.1.65
Pyruvate
2-Oxoglutarate
Aminoacetaldehyde
1.14.11.17
2.6.1.77
1.4.1.1
1.4.1.2
2.6.1.55
1.4.2.-
Sulfur metabolism
Sulfite
L-Alanine
L-Glutamate
Pyruvate metabolism
Acetyl-CoA
2.3.1.8
Acetyl phosphate
2.3.3.15
Sulfoacetaldehyde
2.7.2.1
1.1.2.-
1.2.1.73
Sulfoacetate
1.1.1.313
Acetate
Isethionate
Excretion
00430 11/18/15
(c) Kanehisa Laboratories

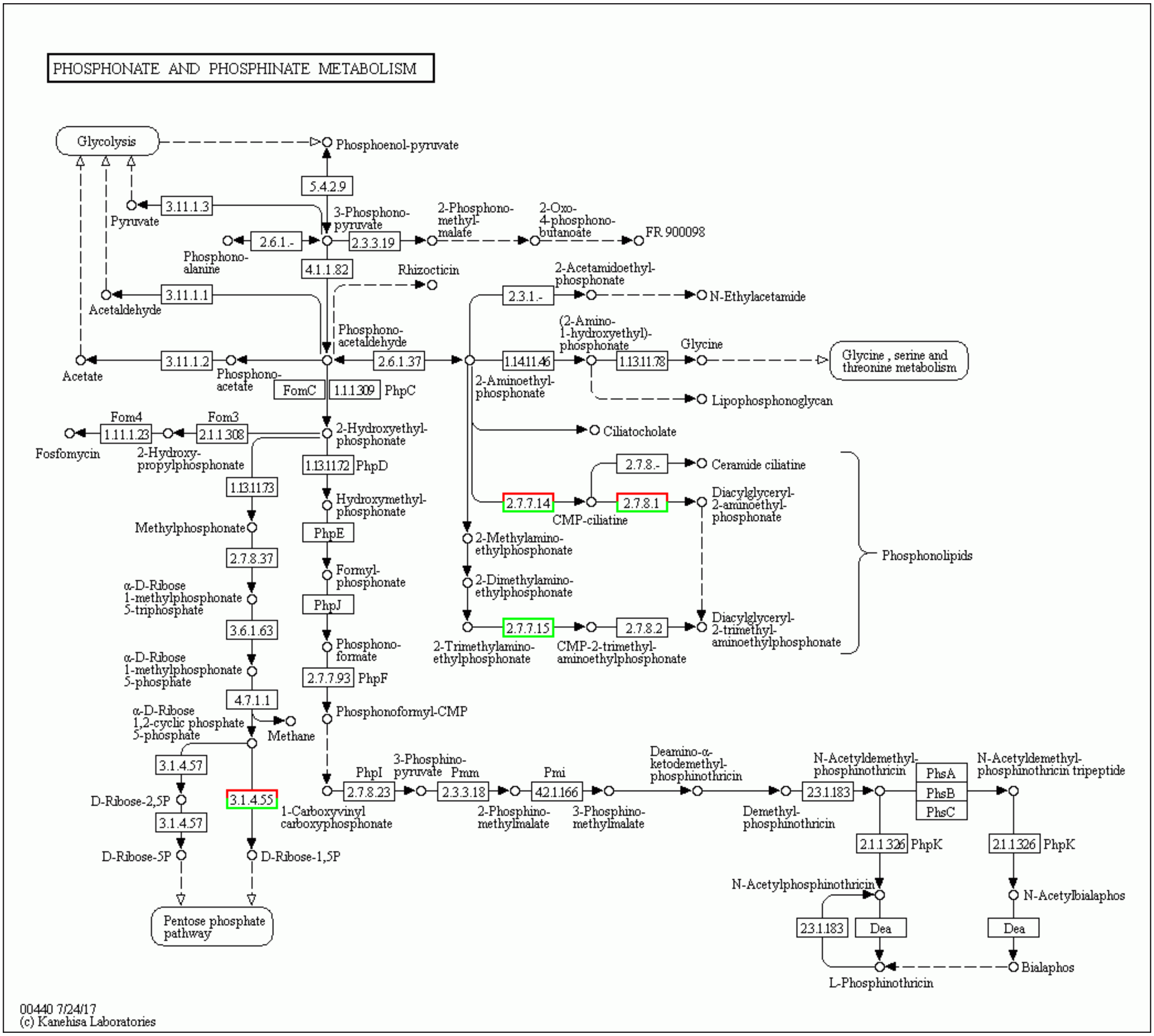
PHOSPHONATE AND PHOSPHINATE METABOLISM
Glycolysis
Phosphoenol-pyruvate
5.4.2.9
3.11.1.3
Pyruvate
3-Phosphono-pyruvate
2-Phosphono-methyl-malate
2-Oxo-4-phosphono-butanoate
FR 900098
2.6.1.-
2.3.3.19
Phosphono-alanine
4.1.1.82
Rhizocticin
3.11.1.1
Acetaldehyde
2-Acetamidoethyl-phosphonate
2.3.1.-
N-Ethylacetamide
Phosphono-acetaldehyde
(2-Amino-1-hydroxyethyl)-phosphonate
Glycine
Glycine, serine and threonine metabolism
3.11.1.2
Acetate
Phosphono-acetate
2.6.1.37
1.14.11.46
1.13.11.78
FomC
1.1.1.309
PhpC
2-Aminoethyl-phosphonate
Lipophosphonoglycan
Fom4
Fom3
1.11.1.23
2.1.1.308
Fosfomycin
2-Hydroxy-propylphosphonate
2-Hydroxyethyl-phosphonate
Ciliatocholate
1.13.11.72
PhpD
2.7.8.-
Ceramide ciliatine
1.13.11.73
Hydroxymethyl-phosphonate
2.7.7.14
2.7.8.1
CMP-ciliatine
Diacylglyceryl-2-aminoethyl-phosphonate
Methylphosphonate
PhpE
2-Methylamino-ethylphosphonate
Phosphonolipids
2.7.8.37
Formyl-phosphonate
2-Dimethylamino-ethylphosphonate
α-D-Ribose 1-methylphosphonate 5-triphosphate
PhpJ
3.6.1.63
Phosphono-formate
2.7.7.15
2.7.8.2
2-Trimethylamino-ethylphosphonate
CMP-2-trimethyl-aminoethylphosphonate
Diacylglyceryl-2-trimethyl-aminoethylphosphonate
α-D-Ribose 1-methylphosphonate 5-phosphate
2.7.7.93
PhpF
4.7.1.1
Phosphonoformyl-CMP
α-D-Ribose 1,2-cyclic phosphate 5-phosphate
Methane
3.1.4.57
PhpI
3-Phosphino-pyruvate
Pmm
Pmi
Deamino-α-ketodemethyl-phosphinothricin
N-Acetyldemethyl-phosphinothricin
N-Acetyldemethyl-phosphinothricin tripeptide
2.7.8.23
2.3.3.18
4.2.1.166
2.3.1.183
PhsA
PhsB
PhsC
D-Ribose-2,5P
3.1.4.55
1-Carboxyvinyl carboxyphosphonate
2-Phosphino-methylmalate
3-Phosphino-methylmalate
Demethyl-phosphinothricin
3.1.4.57
2.1.1.326
PhpK
2.1.1.326
PhpK
D-Ribose-5P
D-Ribose-1,5P
N-Acetylphosphinothricin
N-Acetylbialaphos
2.3.1.183
Dea
Dea
Pentose phosphate pathway
L-Phosphinothricin
Bialaphos
00440 7/24/17
(c) Kanehisa Laboratories

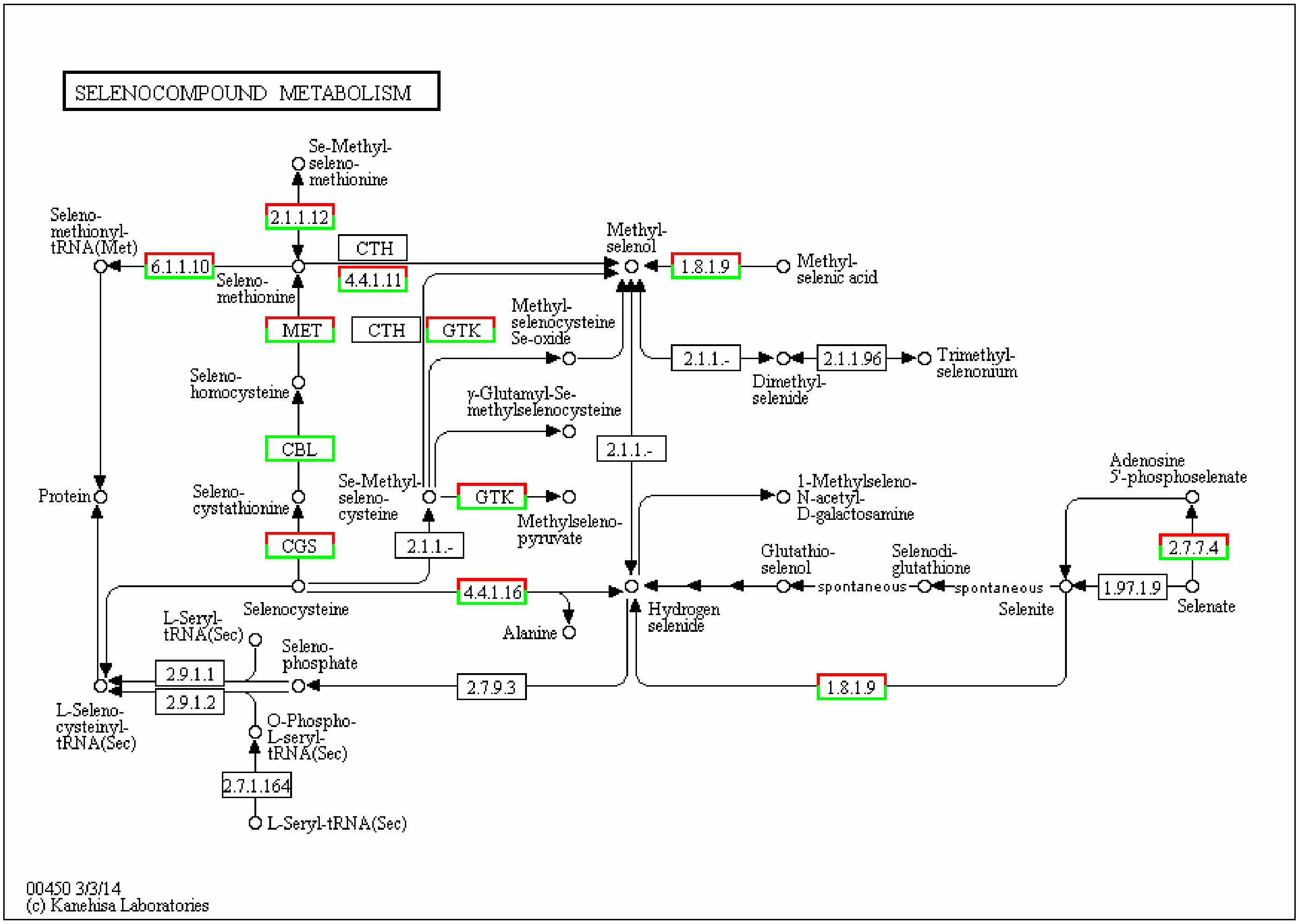
SELENOCOMPOUND METABOLISM
Se-Methyl-
seleno-
methionine
2.1.1.12
Seleno-
methionyl-
tRNA(Met)
6.1.1.10
CTH
4.4.1.11
Seleno-
methionine
Methyl-
selenol
1.8.1.9
Methyl-
selenic acid
MET
CTH
GTK
Methyl-
selenocysteine
Se-oxide
2.1.1.-
Dimethyl-
selenide
2.1.1.96
Trimethyl-
selenonium
Seleno-
homocysteine
γ-Glutamyl-Se-
methylselenocysteine
CBL
2.1.1.-
Adenosine
5'-phosphoselenate
Protein
Seleno-
cystathionine
Se-Methyl-
seleno-
cysteine
GTK
Methylseleno-
pyruvate
1-Methylseleno-
N-acetyl-
D-galactosamine
2.7.7.4
CGS
2.1.1.-
Glutathio-
selenol
Selenodi-
glutathione
spontaneous
spontaneous
1.97.1.9
Selenate
Selenite
4.4.1.16
Selenocysteine
Hydrogen
selenide
Alanine
L-Seryl-
tRNA(Sec)
Seleno-
phosphate
2.9.1.1
2.9.1.2
2.7.9.3
1.8.1.9
L-Seleno-
cysteinyl-
tRNA(Sec)
O-Phospho-
L-seryl-
tRNA(Sec)
2.7.1.164
L-Seryl-tRNA(Sec)
00450 3/3/14
(c) Kanehisa Laboratories

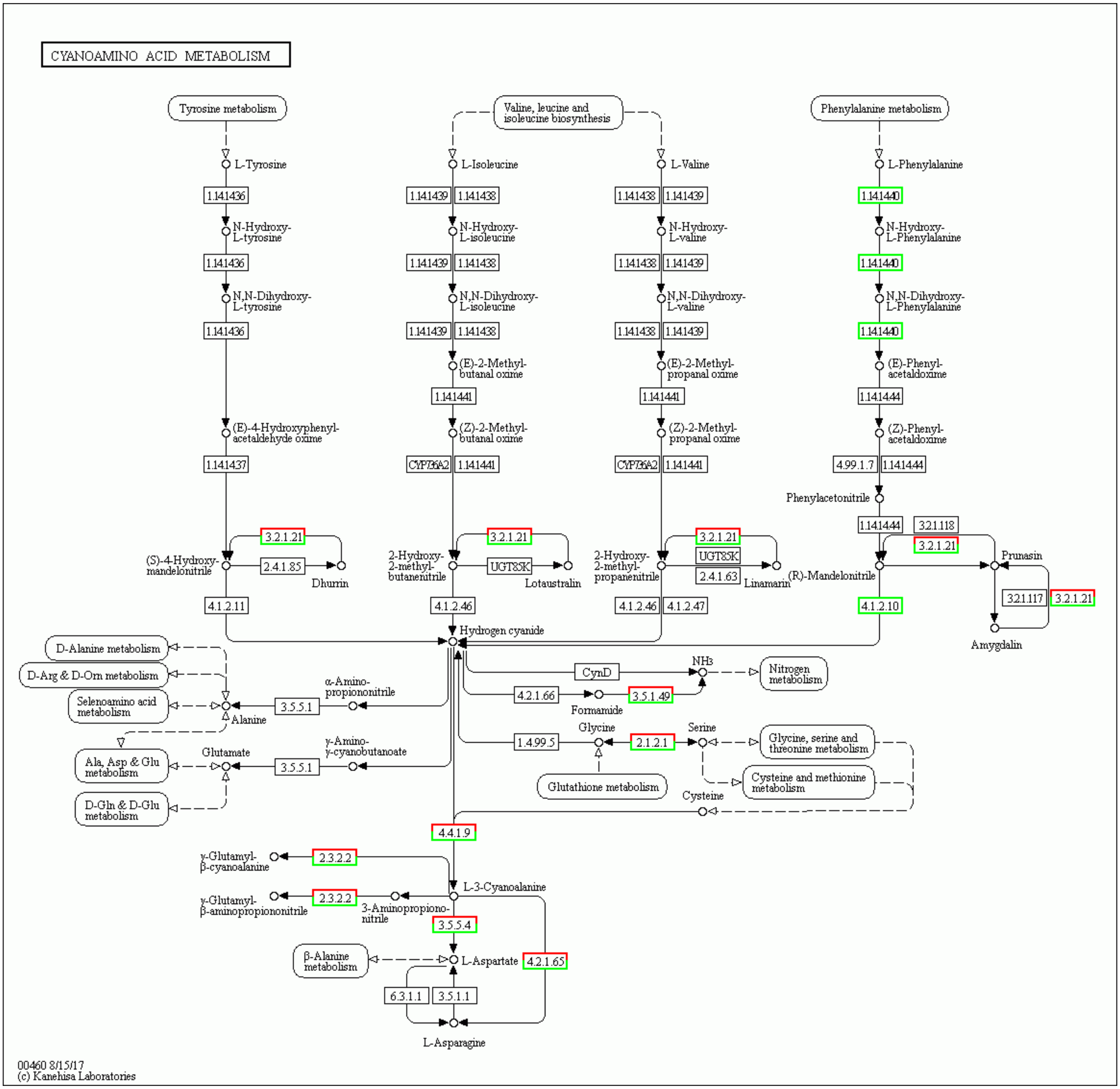
CYANOAMINO ACID METABOLISM
Tyrosine metabolism
Valine, leucine and isoleucine biosynthesis
Phenylalanine metabolism
L-Tyrosine
L-Isoleucine
L-Valine
L-Phenylalanine
N-Hydroxy-L-tyrosine
N-Hydroxy-L-isoleucine
N-Hydroxy-L-valine
N-Hydroxy-L-Phenylalanine
N,N-Dihydroxy-L-tyrosine
N,N-Dihydroxy-L-isoleucine
N,N-Dihydroxy-L-valine
N,N-Dihydroxy-L-Phenylalanine
(E)-2-Methyl-butanal oxime
(E)-2-Methyl-propanal oxime
(E)-Phenyl-acetaldoxime
(E)-4-Hydroxyphenyl-acetaldehyde oxime
(Z)-2-Methyl-butanal oxime
(Z)-2-Methyl-propanal oxime
(Z)-Phenyl-acetaldoxime
Phenylacetonitrile
(S)-4-Hydroxy-mandelonitrile
2-Hydroxy-2-methyl-butanenitrile
2-Hydroxy-2-methyl-propanenitrile
(R)-Mandelonitrile
Dhurrin
Lotaustralin
Linamarin
Prunasin
Amygdalin
Hydrogen cyanide
D-Alanine metabolism
D-Arg & D-Orn metabolism
Selenoamino acid metabolism
Alanine
α-Amino-propiononitrile
Ala, Asp & Glu metabolism
D-Gln & D-Glu metabolism
Glutamate
γ-Amino-γ-cyanobutanoate
NH3
Nitrogen metabolism
Formamide
Glycine
Serine
Glycine, serine and threonine metabolism
Cysteine and methionine metabolism
Glutathione metabolism
Cysteine
γ-Glutamyl-β-cyanoalanine
γ-Glutamyl-β-aminopropiononitrile
L-3-Cyanoalanine
3-Aminopropiono-nitrile
β-Alanine metabolism
L-Aspartate
L-Asparagine
1.14.14.36
1.14.14.39
1.14.14.38
1.14.14.40
1.14.14.41
1.14.14.37
1.14.14.44
CYP736A2
4.99.1.7
3.2.1.21
3.2.1.118
3.2.1.117
2.4.1.85
UGT85K
2.4.1.63
4.1.2.11
4.1.2.46
4.1.2.47
4.1.2.10
CynD
4.2.1.66
3.5.1.49
3.5.5.1
1.4.99.5
2.1.2.1
4.4.1.9
2.3.2.2
3.5.5.4
4.2.1.65
6.3.1.1
3.5.1.1
00460 8/15/17
(c) Kanehisa Laboratories

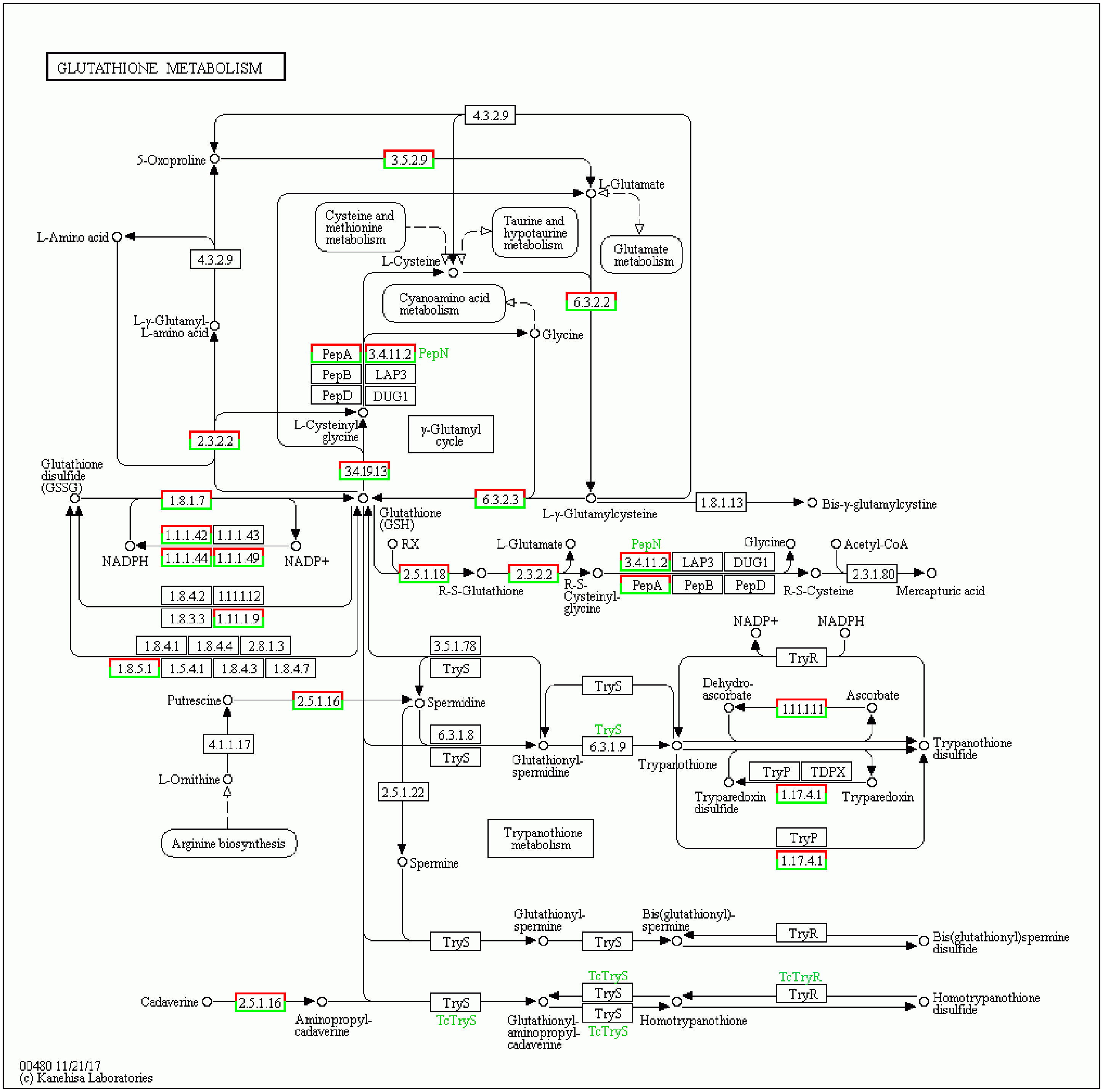
GLUTATHIONE METABOLISM
4.3.2.9
5-Oxoproline
3.5.2.9
L-Glutamate
Cysteine and methionine metabolism
Taurine and hypotaurine metabolism
Glutamate metabolism
L-Amino acid
4.3.2.9
L-Cysteine
Cyanoamino acid metabolism
6.3.2.2
L-γ-Glutamyl-L-amino acid
Glycine
PepA
3.4.11.2
PepN
PepB
LAP3
PepD
DUG1
L-Cysteinyl-glycine
γ-Glutamyl cycle
2.3.2.2
Glutathione disulfide (GSSG)
3.4.19.13
6.3.2.3
1.8.1.7
Glutathione (GSH)
L-γ-Glutamylcysteine
1.8.1.13
Bis-γ-glutamylcystine
1.1.1.42
1.1.1.43
1.1.1.44
1.1.1.49
NADPH
NADP+
RX
L-Glutamate
PepN
Glycine
Acetyl-CoA
2.5.1.18
2.3.2.2
3.4.11.2
LAP3
DUG1
2.3.1.80
PepA
PepB
PepD
R-S-Glutathione
R-S-Cysteinyl-glycine
R-S-Cysteine
Mercapturic acid
1.8.4.2
1.11.1.12
1.8.3.3
1.11.1.9
NADP+
NADPH
1.8.4.1
1.8.4.4
2.8.1.3
3.5.1.78
TryR
1.8.5.1
1.5.4.1
1.8.4.3
1.8.4.7
TryS
TryS
Dehydro-ascorbate
Ascorbate
Putrescine
2.5.1.16
Spermidine
1.11.1.11
TryS
6.3.1.8
6.3.1.9
Trypanothione disulfide
4.1.1.17
TryS
Glutathionyl-spermidine
Trypanothione
TryP
TDPX
L-Ornithine
2.5.1.22
Tryparedoxin disulfide
1.17.4.1
Tryparedoxin
Arginine biosynthesis
Trypanothione metabolism
TryP
1.17.4.1
Spermine
Glutathionyl-spermine
Bis(glutathionyl)-spermine
TryR
TryS
TryS
Bis(glutathionyl)spermine disulfide
TcTryS
TcTryR
Cadaverine
2.5.1.16
TryS
TryS
TryR
TryS
Homotrypanothione disulfide
Aminopropyl-cadaverine
TcTryS
Glutathionyl-aminopropyl-cadaverine
TcTryS
Homotrypanothione
00480 11/21/17
(c) Kanehisa Laboratories

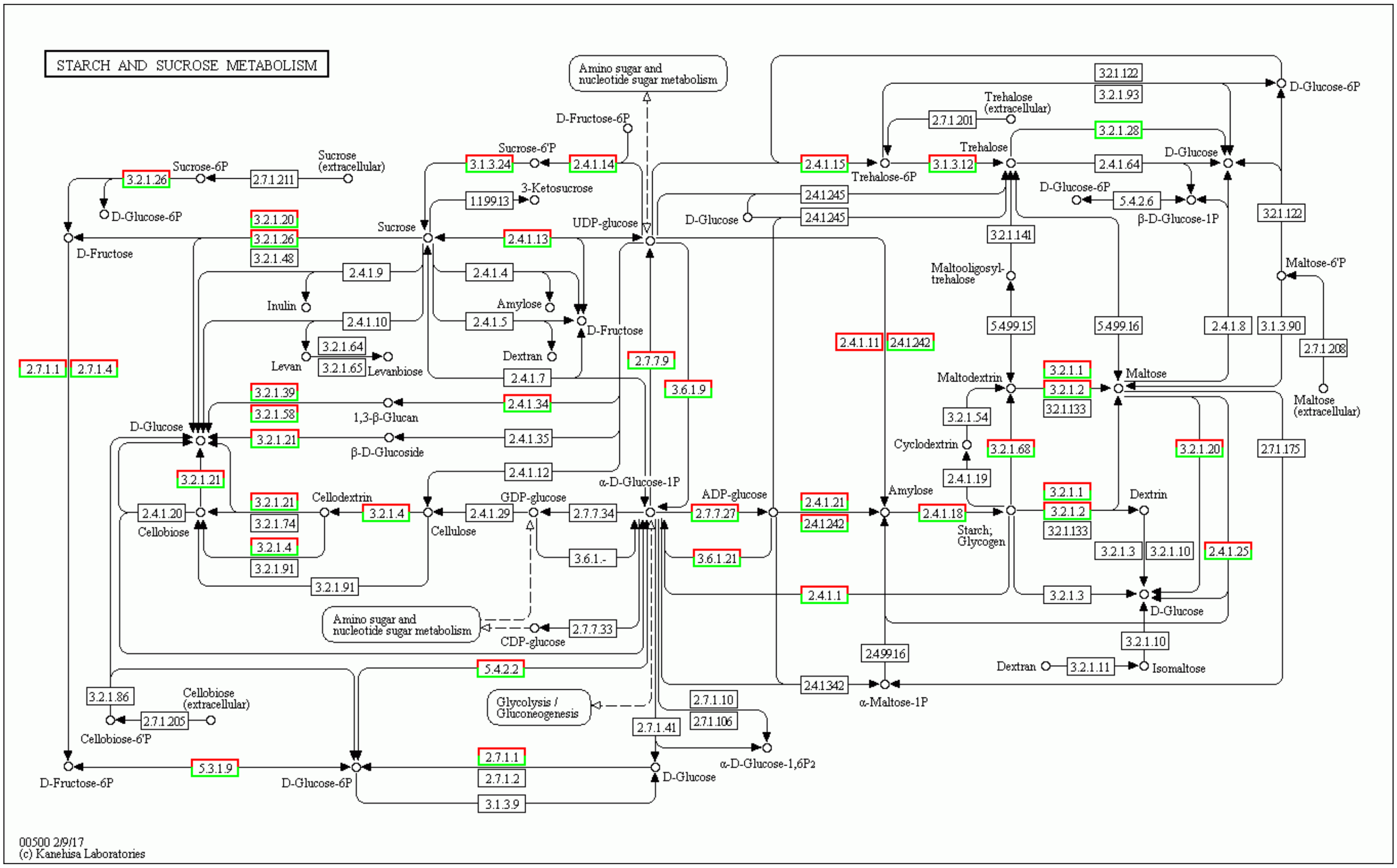
STARCH AND SUCROSE METABOLISM
Amino sugar and nucleotide sugar metabolism
D-Fructose-6P
Sucrose-6'P
3.1.3.24
2.4.1.14
3-Ketosucrose
1.1.99.13
UDP-glucose
Sucrose-6P
Sucrose (extracellular)
2.7.1.211
3.2.1.26
D-Glucose-6P
D-Fructose
3.2.1.20
3.2.1.26
3.2.1.48
Sucrose
2.4.1.13
2.4.1.9
Inulin
2.4.1.4
Amylose
2.4.1.10
2.4.1.5
D-Fructose
Levan
3.2.1.64
3.2.1.65
Levanbiose
Dextran
2.4.1.7
2.7.1.1
2.7.1.4
3.2.1.39
3.2.1.58
2.4.1.34
1,3-β-Glucan
D-Glucose
3.2.1.21
2.4.1.35
β-D-Glucoside
2.4.1.12
3.2.1.21
3.2.1.21
Cellodextrin
3.2.1.74
3.2.1.4
2.4.1.29
GDP-glucose
2.7.7.34
2.4.1.20
Cellobiose
Cellulose
3.2.1.4
3.2.1.91
3.6.1.-
3.2.1.91
Amino sugar and nucleotide sugar metabolism
2.7.7.33
CDP-glucose
2.7.7.9
3.6.1.9
α-D-Glucose-1P
2.7.7.27
ADP-glucose
3.6.1.21
5.4.2.2
Glycolysis / Gluconeogenesis
3.2.1.86
Cellobiose (extracellular)
2.7.1.205
Cellobiose-6'P
2.7.1.10
2.7.1.106
2.7.1.41
α-D-Glucose-1,6P2
D-Fructose-6P
5.3.1.9
D-Glucose-6P
2.7.1.1
2.7.1.2
D-Glucose
3.1.3.9
3.2.1.122
3.2.1.93
D-Glucose-6P
Trehalose (extracellular)
2.7.1.201
3.2.1.28
Trehalose
2.4.1.15
Trehalose-6P
3.1.3.12
2.4.1.64
D-Glucose
D-Glucose
2.4.1.245
2.4.1.245
D-Glucose-6P
5.4.2.6
β-D-Glucose-1P
3.2.1.122
3.2.1.141
Maltooligosyl-trehalose
Maltose-6'P
5.4.99.15
5.4.99.16
2.4.1.8
3.1.3.90
2.7.1.208
2.4.1.11
2.4.1.242
Maltodextrin
3.2.1.1
3.2.1.2
3.2.1.133
Maltose
Maltose (extracellular)
3.2.1.54
Cyclodextrin
3.2.1.68
3.2.1.20
2.7.1.175
2.4.1.19
2.4.1.21
2.4.1.242
Amylose
2.4.1.18
3.2.1.1
3.2.1.2
3.2.1.133
Dextrin
Starch; Glycogen
3.2.1.3
3.2.1.10
2.4.1.25
2.4.1.1
3.2.1.3
D-Glucose
3.2.1.10
2.4.99.16
Dextran
3.2.1.11
Isomaltose
2.4.1.342
α-Maltose-1P
00500 2/9/17
(c) Kanehisa Laboratories

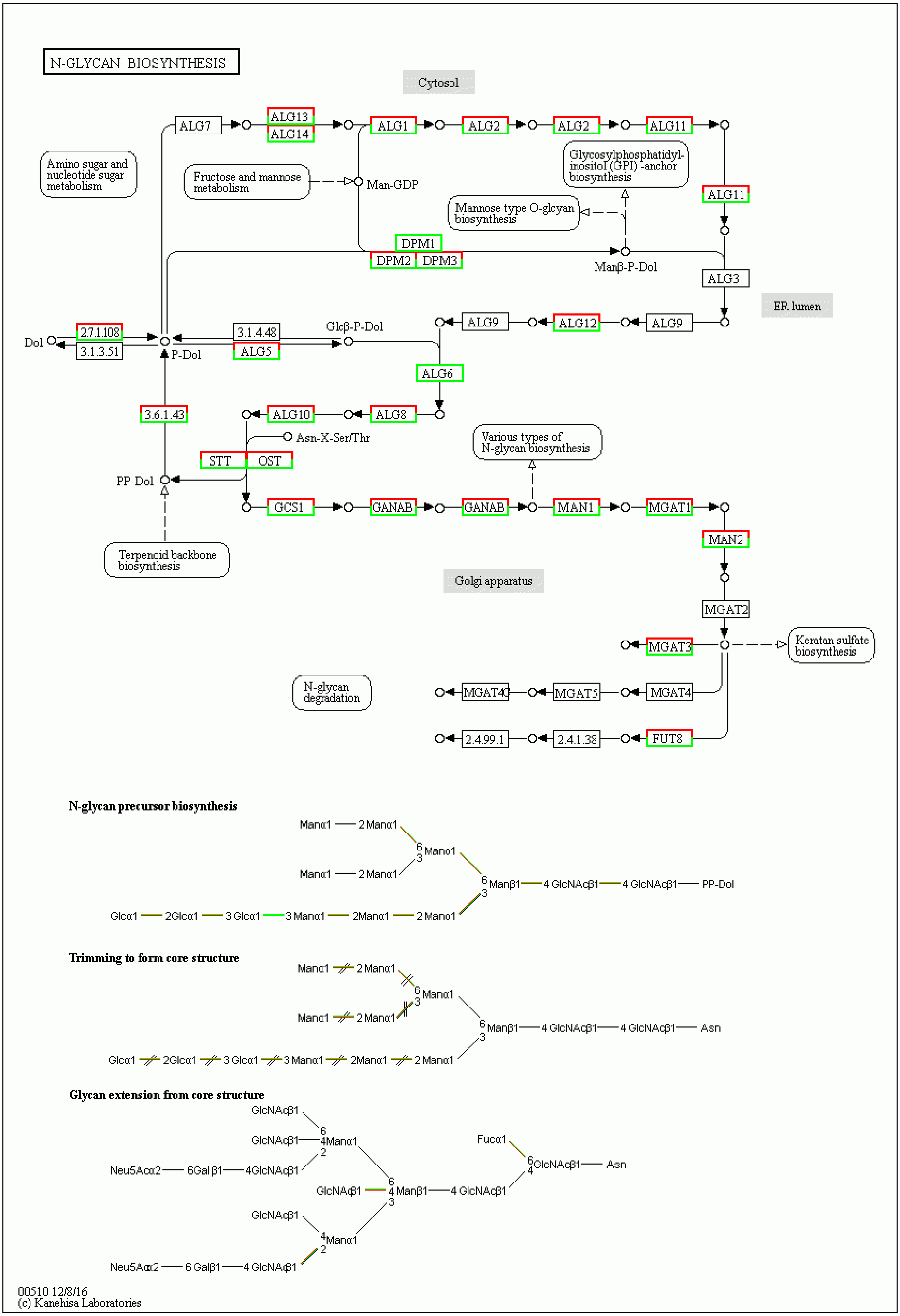
N-GLYCAN BIOSYNTHESIS
Cytosol
ALG7
ALG13
ALG14
ALG1
ALG2
ALG2
ALG11
Amino sugar and nucleotide sugar metabolism
Fructose and mannose metabolism
Man-GDP
Glycosylphosphatidyl-inositol (GPI) -anchor biosynthesis
ALG11
Mannose type O-glcyan biosynthesis
DPM1
DPM2
DPM3
Manβ-P-Dol
ALG3
ER lumen
Dol
2.7.1.108
3.1.3.51
P-Dol
3.1.4.48
ALG5
Glcβ-P-Dol
ALG9
ALG12
ALG9
ALG6
3.6.1.43
ALG10
ALG8
Asn-X-Ser/Thr
Various types of N-glycan biosynthesis
STT
OST
PP-Dol
GCS1
GANAB
GANAB
MAN1
MGAT1
MAN2
Terpenoid backbone biosynthesis
Golgi apparatus
MGAT2
MGAT3
Keratan sulfate biosynthesis
N-glycan degradation
MGAT4C
MGAT5
MGAT4
2.4.99.1
2.4.1.38
FUT8
N-glycan precursor biosynthesis
Manα1 — 2 Manα1
6 Manα1
3
Manα1 — 2 Manα1
6 Manβ1 — 4 GlcNAcβ1 — 4 GlcNAcβ1 — PP-Dol
3
Glcα1 — 2Glcα1 — 3 Glcα1 — 3 Manα1 — 2Manα1 — 2 Manα1
Trimming to form core structure
Manα1 — 2 Manα1
6 Manα1
3
Manα1 — 2 Manα1
6 Manβ1 — 4 GlcNAcβ1 — 4 GlcNAcβ1 — Asn
3
Glcα1 — 2Glcα1 — 3 Glcα1 — 3 Manα1 — 2Manα1 — 2 Manα1
Glycan extension from core structure
GlcNAcβ1
6
GlcNAcβ1 — 4Manα1
2
Neu5Acα2 — 6Gal β1 — 4GlcNAcβ1
Fucα1
6 GlcNAcβ1 — Asn
4
6
GlcNAcβ1 — 4 Manβ1 — 4 GlcNAcβ1
3
GlcNAcβ1
4 Manα1
2
Neu5Acα2 — 6 Galβ1 — 4 GlcNAcβ1
00510 12/8/16
(c) Kanehisa Laboratories

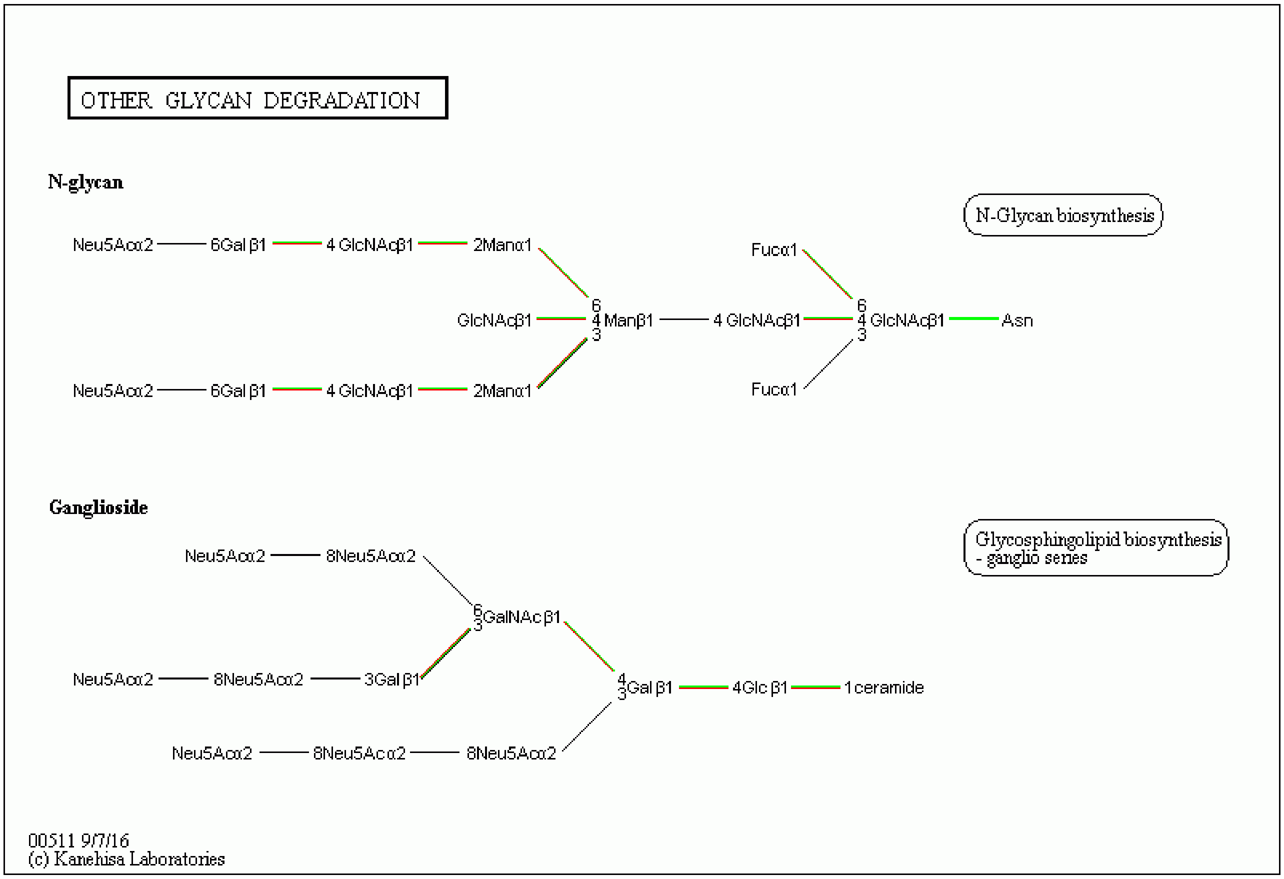
OTHER GLYCAN DEGRADATION
N-glycan
N-Glycan biosynthesis
Neu5Acα2 6Galβ1 4GlcNAcβ1 2Manα1
GlcNAcβ1 6 4 3 Manβ1 4GlcNAcβ1
Neu5Acα2 6Galβ1 4GlcNAcβ1 2Manα1
Fucα1
6 4 3 GlcNAcβ1 Asn
Fucα1
Ganglioside
Glycosphingolipid biosynthesis - ganglio series
Neu5Acα2 8Neu5Acα2
6 3 GalNAcβ1
Neu5Acα2 8Neu5Acα2 3Galβ1
4 3 Galβ1 4Glcβ1 1ceramide
Neu5Acα2 8Neu5Acα2 8Neu5Acα2
00511 9/7/16
(c) Kanehisa Laboratories

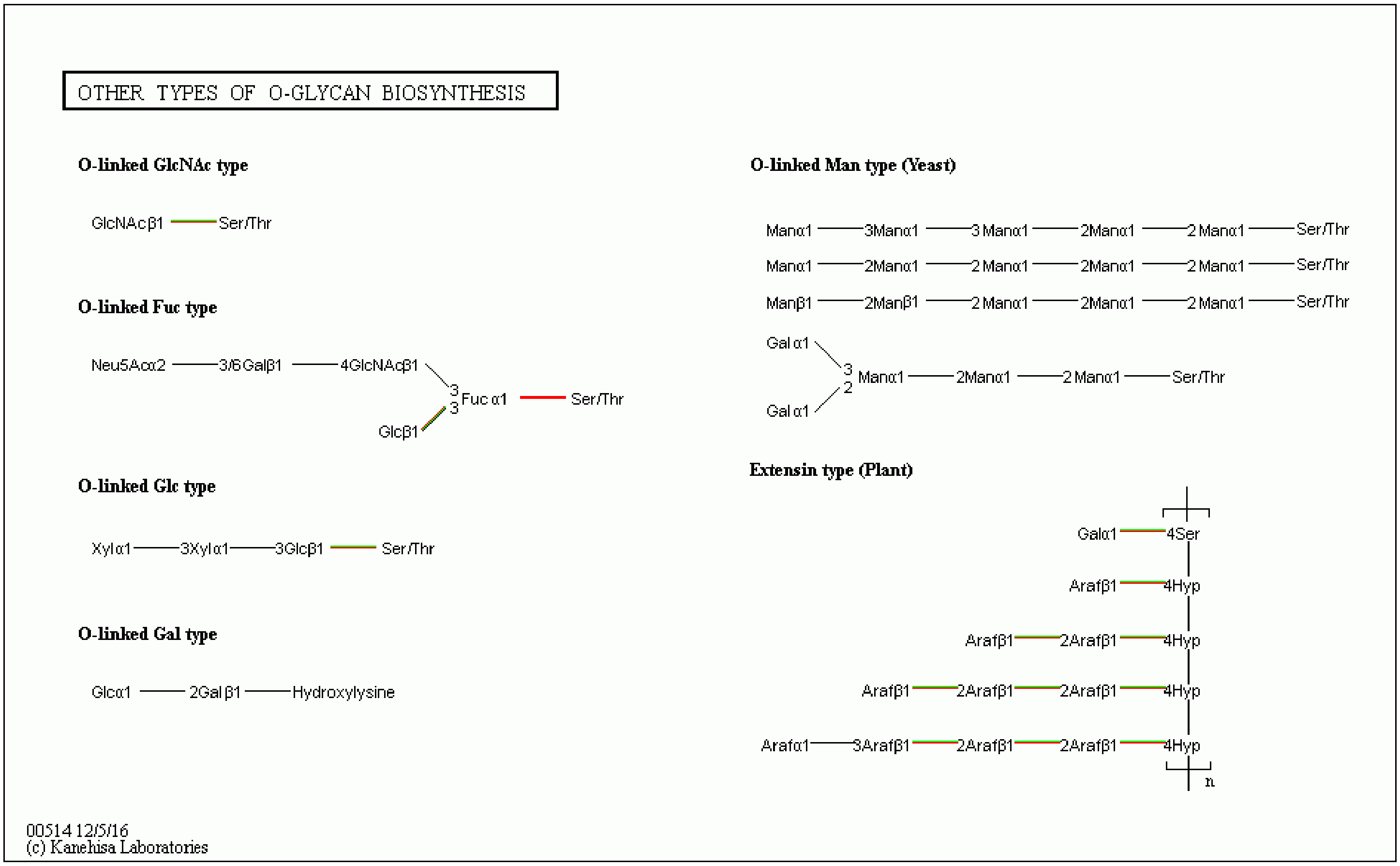
OTHER TYPES OF O-GLYCAN BIOSYNTHESIS
O-linked GlcNAc type
GlcNAcβ1 Ser/Thr
O-linked Fuc type
Neu5Acα2 3/6Galβ1 4GlcNAcβ1
3 3 Fucα1 Ser/Thr
Glcβ1
O-linked Glc type
Xylα1 3Xylα1 3Glcβ1 Ser/Thr
O-linked Gal type
Glcα1 2Galβ1 Hydroxylysine
O-linked Man type (Yeast)
Manα1 3Manα1 3Manα1 2Manα1 2Manα1 Ser/Thr
Manα1 2Manα1 2Manα1 2Manα1 2Manα1 Ser/Thr
Manβ1 2Manβ1 2Manα1 2Manα1 2Manα1 Ser/Thr
Galα1
3 2 Manα1 2Manα1 2Manα1 Ser/Thr
Galα1
Extensin type (Plant)
Galα1 4Ser
Arafβ1 4Hyp
Arafβ1 2Arafβ1 4Hyp
Arafβ1 2Arafβ1 2Arafβ1 4Hyp
Arafα1 3Arafβ1 2Arafβ1 2Arafβ1 4Hyp
n
00514 12/5/16
(c) Kanehisa Laboratories

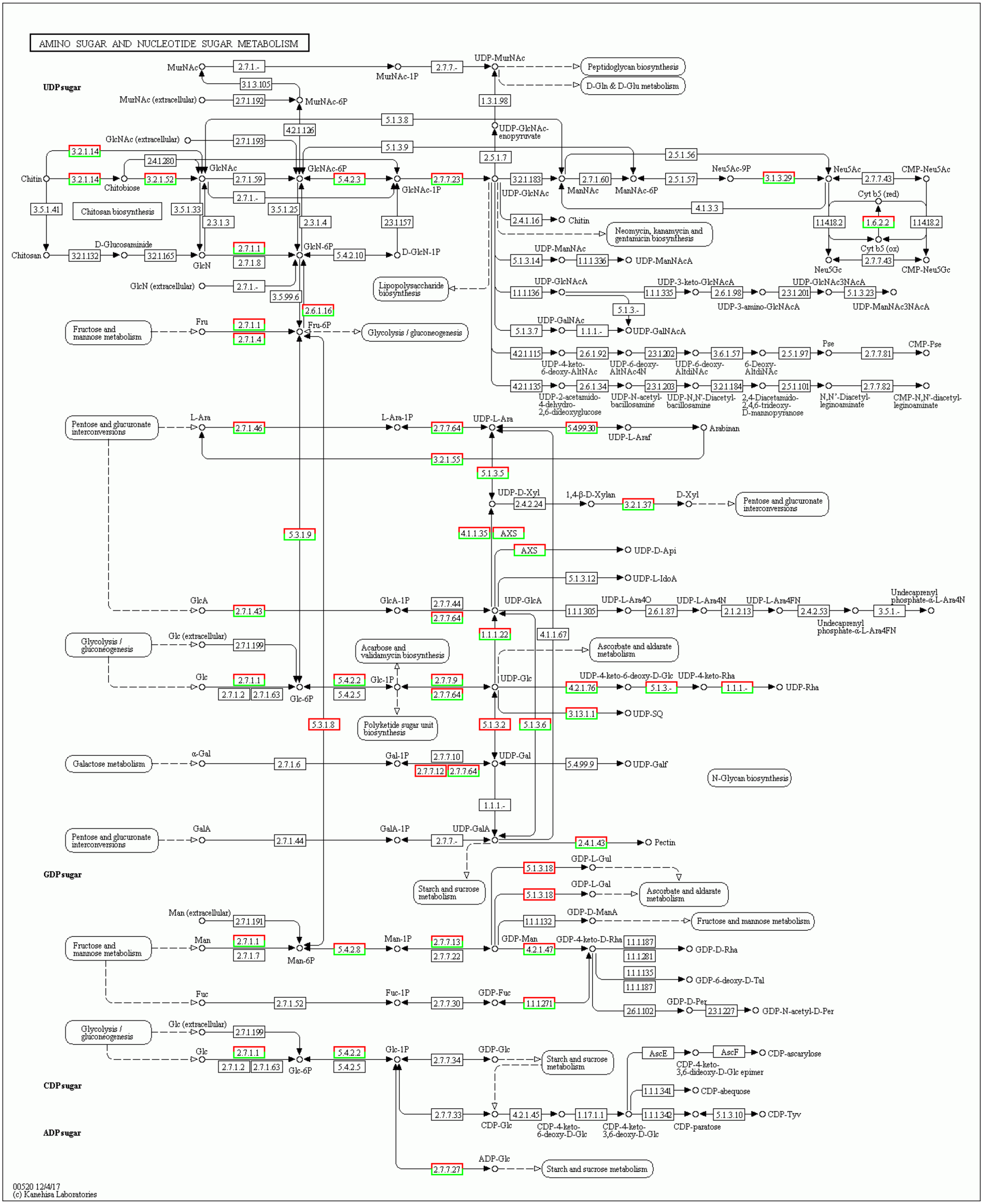
AMINO SUGAR AND NUCLEOTIDE SUGAR METABOLISM
UDP sugar
MurNAc
2.7.1.-
MurNAc-1P
2.7.7.-
UDP-MurNAc
Peptidoglycan biosynthesis
D-Gln & D-Glu metabolism
3.1.3.105
MurNAc (extracellular)
2.7.1.192
MurNAc-6P
1.3.1.98
5.1.3.8
4.2.1.126
UDP-GlcNAc-enopyruvate
GlcNAc (extracellular)
2.7.1.193
5.1.3.9
3.2.1.14
2.4.1.280
2.5.1.7
2.5.1.56
GlcNAc
GlcNAc-6P
Chitin
3.2.1.14
Chitobiose
3.2.1.52
2.7.1.59
5.4.2.3
GlcNAc-1P
2.7.7.23
3.2.1.183
UDP-GlcNAc
2.7.1.60
ManNAc
ManNAc-6P
2.5.1.57
Neu5Ac-9P
3.1.3.29
Neu5Ac
2.7.7.43
CMP-Neu5Ac
2.7.1.-
3.5.1.41
Chitosan biosynthesis
3.5.1.33
3.5.1.25
2.3.1.4
2.3.1.157
4.1.3.3
Cyt b5 (red)
1.14.18.2
1.6.2.2
1.14.18.2
2.3.1.3
2.4.1.16
Chitin
Neomycin, kanamycin and gentamicin biosynthesis
Cyt b5 (ox)
Chitosan
3.2.1.132
D-Glucosaminide
3.2.1.165
GlcN
2.7.1.1
2.7.1.8
GlcN-6P
5.4.2.10
D-GlcN-1P
UDP-ManNAc
5.1.3.14
1.1.1.336
UDP-ManNAcA
Neu5Gc
2.7.7.43
CMP-Neu5Gc
GlcN (extracellular)
2.7.1.-
3.5.99.6
Lipopolysaccharide biosynthesis
UDP-GlcNAcA
1.1.1.136
UDP-3-keto-GlcNAcA
1.1.1.335
2.6.1.98
2.3.1.201
UDP-GlcNAc3NAcA
5.1.3.23
UDP-3-amino-GlcNAcA
UDP-ManNAc3NAcA
2.6.1.16
5.1.3.-
Fructose and mannose metabolism
Fru
2.7.1.1
2.7.1.4
Fru-6P
Glycolysis / gluconeogenesis
UDP-GalNAc
5.1.3.7
1.1.1.-
UDP-GalNAcA
4.2.1.115
2.6.1.92
2.3.1.202
3.6.1.57
2.5.1.97
Pse
2.7.7.81
CMP-Pse
UDP-4-keto-6-deoxy-AltNAc
UDP-6-deoxy-AltNAc4N
UDP-6-deoxy-AltdiNAc
6-Deoxy-AltdiNAc
4.2.1.135
2.6.1.34
2.3.1.203
3.2.1.184
2.5.1.101
2.7.7.82
UDP-2-acetamido-4-dehydro-2,6-dideoxyglucose
UDP-N-acetyl-bacillosamine
UDP-N,N'-Diacetyl-bacillosamine
2,4-Diacetamido-2,4,6-trideoxy-D-mannopyranose
N,N'-Diacetyl-legionaminate
CMP-N,N'-diacetyl-legionaminate
Pentose and glucuronate interconversions
L-Ara
2.7.1.46
L-Ara-1P
2.7.7.64
UDP-L-Ara
5.4.99.30
UDP-L-Araf
Arabinan
3.2.1.55
5.1.3.5
UDP-D-Xyl
2.4.2.24
1,4-β-D-Xylan
3.2.1.37
D-Xyl
Pentose and glucuronate interconversions
5.3.1.9
4.1.1.35
AXS
AXS
UDP-D-Api
5.1.3.12
UDP-L-IdoA
GlcA
2.7.1.43
GlcA-1P
2.7.7.44
2.7.7.64
UDP-GlcA
1.1.1.305
UDP-L-Ara4O
2.6.1.87
UDP-L-Ara4N
2.1.2.13
UDP-L-Ara4FN
2.4.2.53
3.5.1.-
Undecaprenyl phosphate-α-L-Ara4N
Undecaprenyl phosphate-α-L-Ara4FN
1.1.1.22
4.1.1.67
Glycolysis / gluconeogenesis
Glc (extracellular)
2.7.1.199
Acarbose and validamycin biosynthesis
Ascorbate and aldarate metabolism
Glc
2.7.1.1
2.7.1.2
2.7.1.63
Glc-6P
5.4.2.2
5.4.2.5
Glc-1P
2.7.7.9
2.7.7.64
UDP-Glc
UDP-4-keto-6-deoxy-D-Glc
4.2.1.76
5.1.3.-
UDP-4-keto-Rha
1.1.1.-
UDP-Rha
3.13.1.1
UDP-SQ
5.3.1.8
Polyketide sugar unit biosynthesis
5.1.3.2
5.1.3.6
Galactose metabolism
α-Gal
2.7.1.6
Gal-1P
2.7.7.10
2.7.7.12
2.7.7.64
UDP-Gal
5.4.99.9
UDP-Galf
N-Glycan biosynthesis
1.1.1.-
Pentose and glucuronate interconversions
GalA
2.7.1.44
GalA-1P
2.7.7.-
UDP-GalA
2.4.1.43
Pectin
GDP sugar
5.1.3.18
GDP-L-Gul
Starch and sucrose metabolism
5.1.3.18
GDP-L-Gal
Ascorbate and aldarate metabolism
1.1.1.132
GDP-D-ManA
Fructose and mannose metabolism
Man (extracellular)
2.7.1.191
Fructose and mannose metabolism
Man
2.7.1.1
2.7.1.7
Man-6P
5.4.2.8
Man-1P
2.7.7.13
2.7.7.22
GDP-Man
4.2.1.47
GDP-4-keto-D-Rha
1.1.1.187
1.1.1.281
GDP-D-Rha
1.1.1.135
1.1.1.187
GDP-6-deoxy-D-Tal
Fuc
2.7.1.52
Fuc-1P
2.7.7.30
GDP-Fuc
1.1.1.271
GDP-D-Per
2.6.1.102
2.3.1.227
GDP-N-acetyl-D-Per
Glycolysis / gluconeogenesis
Glc (extracellular)
2.7.1.199
Glc
2.7.1.1
2.7.1.2
2.7.1.63
Glc-6P
5.4.2.2
5.4.2.5
Glc-1P
2.7.7.34
GDP-Glc
Starch and sucrose metabolism
AscE
AscF
CDP-ascarylose
CDP-4-keto-3,6-dideoxy-D-Glc epimer
1.1.1.341
CDP-abequose
CDP sugar
2.7.7.33
CDP-Glc
4.2.1.45
CDP-4-keto-6-deoxy-D-Glc
1.17.1.1
CDP-4-keto-3,6-deoxy-D-Glc
1.1.1.342
CDP-paratose
5.1.3.10
CDP-Tyv
ADP sugar
2.7.7.27
ADP-Glc
Starch and sucrose metabolism
00520 12/4/17
(c) Kanehisa Laboratories

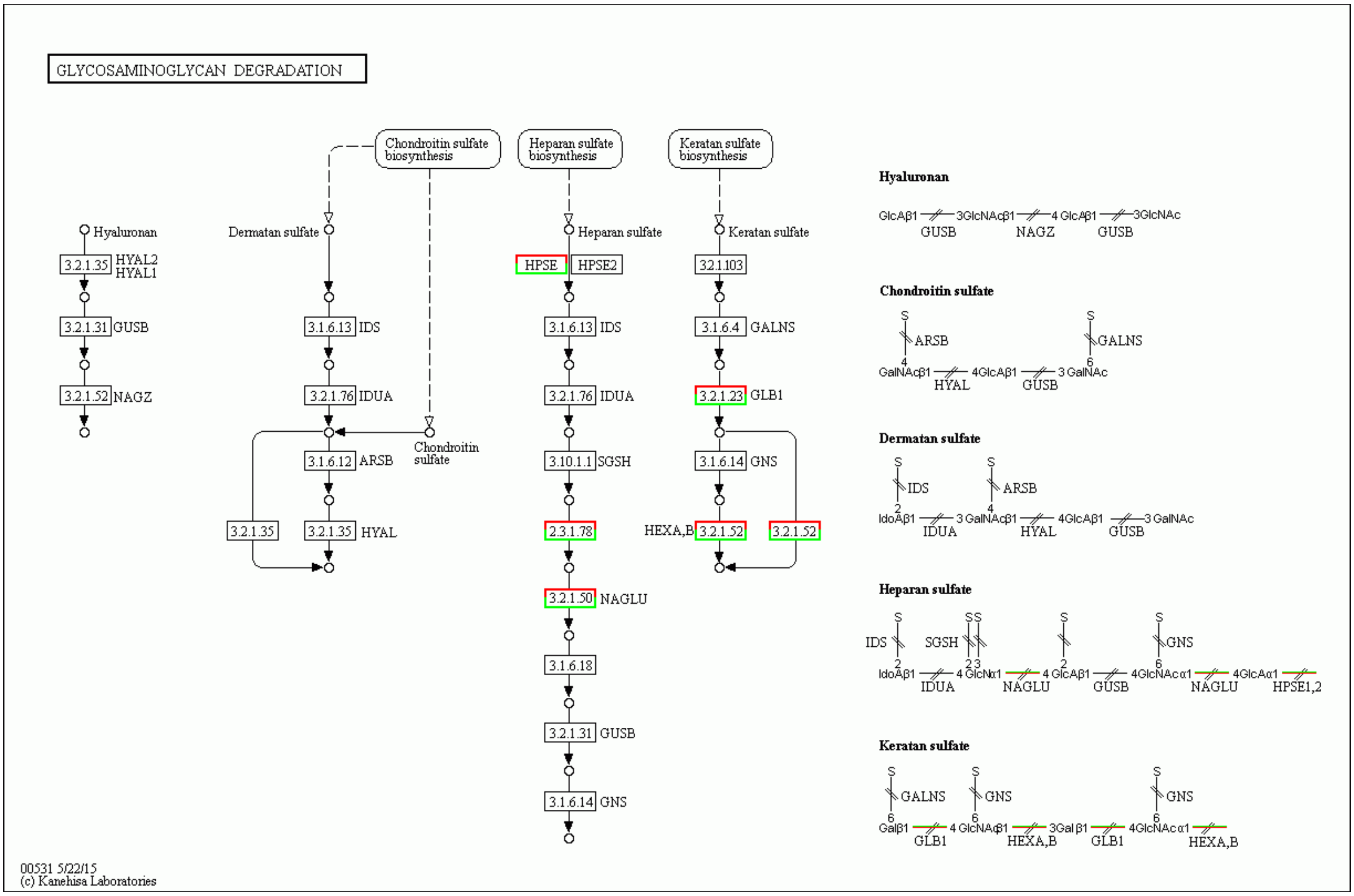
GLYCOSAMINOGLYCAN DEGRADATION
Chondroitin sulfate biosynthesis
Heparan sulfate biosynthesis
Keratan sulfate biosynthesis
Hyaluronan
3.2.1.35 HYAL2 HYAL1
3.2.1.31 GUSB
3.2.1.52 NAGZ
Dermatan sulfate
3.1.6.13 IDS
3.2.1.76 IDUA
3.1.6.12 ARSB
3.2.1.35
3.2.1.35 HYAL
Chondroitin sulfate
Heparan sulfate
HPSE HPSE2
3.1.6.13 IDS
3.2.1.76 IDUA
3.10.1.1 SGSH
2.3.1.78
3.2.1.50 NAGLU
3.1.6.18
3.2.1.31 GUSB
3.1.6.14 GNS
Keratan sulfate
3.2.1.103
3.1.6.4 GALNS
3.2.1.23 GLB1
3.1.6.14 GNS
HEXA,B 3.2.1.52
3.2.1.52
Hyaluronan
GlcAβ1 3GlcNAcβ1 4 GlcAβ1 3GlcNAc
GUSB NAGZ GUSB
Chondroitin sulfate
ARSB GALNS
GalNAcβ1 4GlcAβ1 3 GalNAc
HYAL GUSB
Dermatan sulfate
IDS ARSB
IdoAβ1 3 GalNAcβ1 4GlcAβ1 3 GalNAc
IDUA HYAL GUSB
Heparan sulfate
IDS SGSH GNS
IdoAβ1 4 GlcNα1 4 GlcAβ1 4GlcNAcα1 4GlcAα1
IDUA NAGLU GUSB NAGLU HPSE1,2
Keratan sulfate
GALNS GNS GNS
Galβ1 4 GlcNAcβ1 3Gal β1 4GlcNAcα1
GLB1 HEXA,B GLB1 HEXA,B
00531 5/22/15
(c) Kanehisa Laboratories

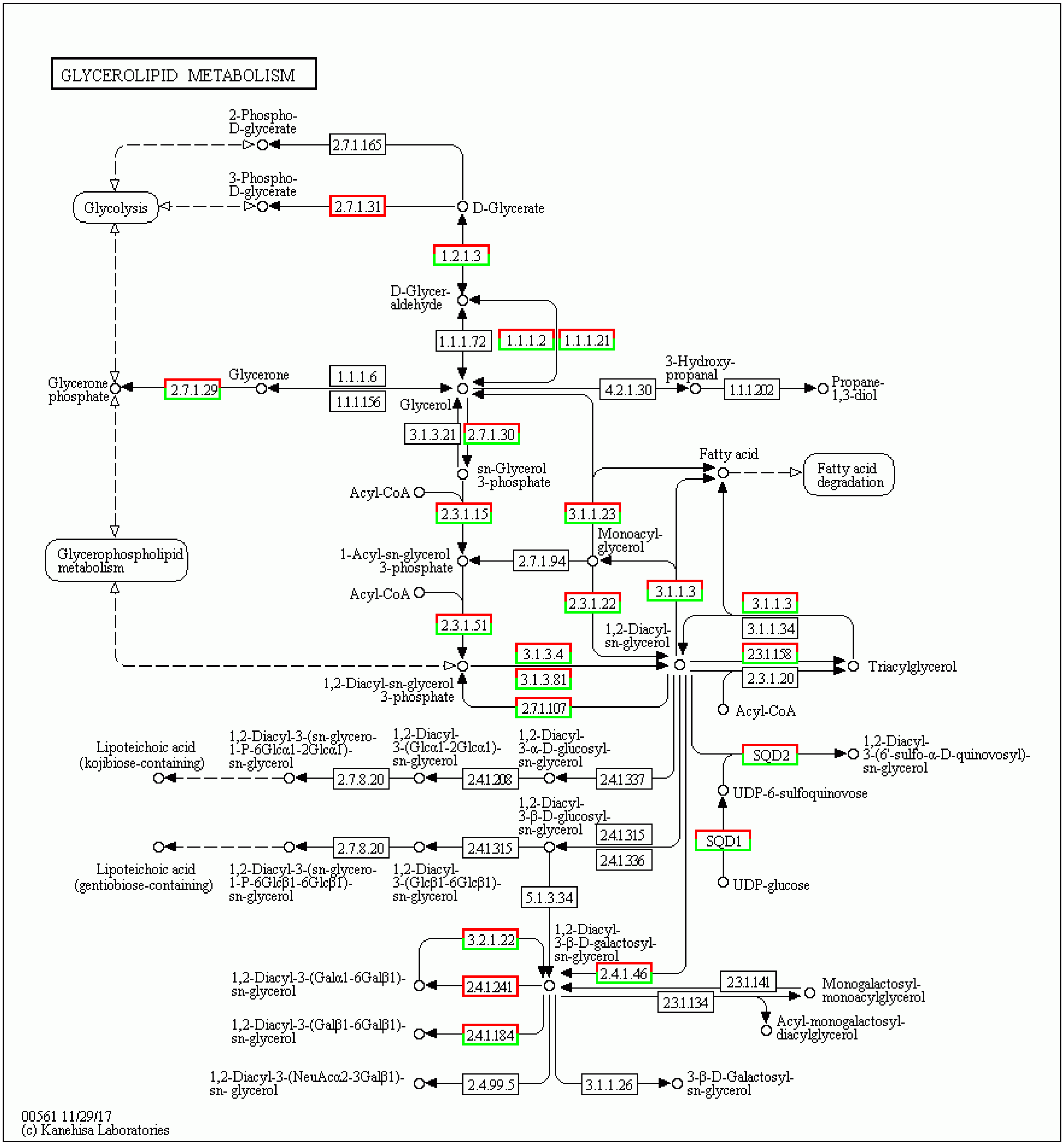
GLYCEROLIPID METABOLISM
2-Phospho-D-glycerate
2.7.1.165
3-Phospho-D-glycerate
2.7.1.31
Glycolysis
D-Glycerate
1.2.1.3
D-Glycer-aldehyde
1.1.1.72
1.1.1.2
1.1.1.21
Glycerone phosphate
2.7.1.29
Glycerone
1.1.1.6
1.1.1.156
Glycerol
4.2.1.30
3-Hydroxy-propanal
1.1.1.202
Propane-1,3-diol
3.1.3.21
2.7.1.30
sn-Glycerol 3-phosphate
Fatty acid
Fatty acid degradation
Acyl-CoA
2.3.1.15
3.1.1.23
Glycerophospholipid metabolism
1-Acyl-sn-glycerol 3-phosphate
2.7.1.94
Monoacyl-glycerol
3.1.1.3
2.3.1.22
2.3.1.51
1,2-Diacyl-sn-glycerol
3.1.1.34
2.3.1.158
2.3.1.20
Triacylglycerol
3.1.3.4
3.1.3.81
2.7.1.107
1,2-Diacyl-sn-glycerol 3-phosphate
Lipoteichoic acid (kojibiose-containing)
1,2-Diacyl-3-(sn-glycero-1-P-6Glcα1-2Glcα1)-sn-glycerol
2.7.8.20
1,2-Diacyl-3-(Glcα1-2Glcα1)-sn-glycerol
2.4.1.208
1,2-Diacyl-3-α-D-glucosyl-sn-glycerol
2.4.1.337
SQD2
1,2-Diacyl-3-(6'-sulfo-α-D-quinovosyl)-sn-glycerol
UDP-6-sulfoquinovose
SQD1
UDP-glucose
1,2-Diacyl-3-β-D-glucosyl-sn-glycerol
2.4.1.315
2.4.1.336
Lipoteichoic acid (gentiobiose-containing)
1,2-Diacyl-3-(sn-glycero-1-P-6Glcβ1-6Glcβ1)-sn-glycerol
1,2-Diacyl-3-(Glcβ1-6Glcβ1)-sn-glycerol
5.1.3.34
3.2.1.22
1,2-Diacyl-3-β-D-galactosyl-sn-glycerol
2.4.1.46
1,2-Diacyl-3-(Galα1-6Galβ1)-sn-glycerol
2.4.1.241
2.3.1.141
2.3.1.134
Monogalactosyl-monoacylglycerol
Acyl-monogalactosyl-diacylglycerol
1,2-Diacyl-3-(Galβ1-6Galβ1)-sn-glycerol
2.4.1.184
1,2-Diacyl-3-(NeuAcα2-3Galβ1)-sn- glycerol
2.4.99.5
3.1.1.26
3-β-D-Galactosyl-sn-glycerol
00561 11/29/17
(c) Kanehisa Laboratories

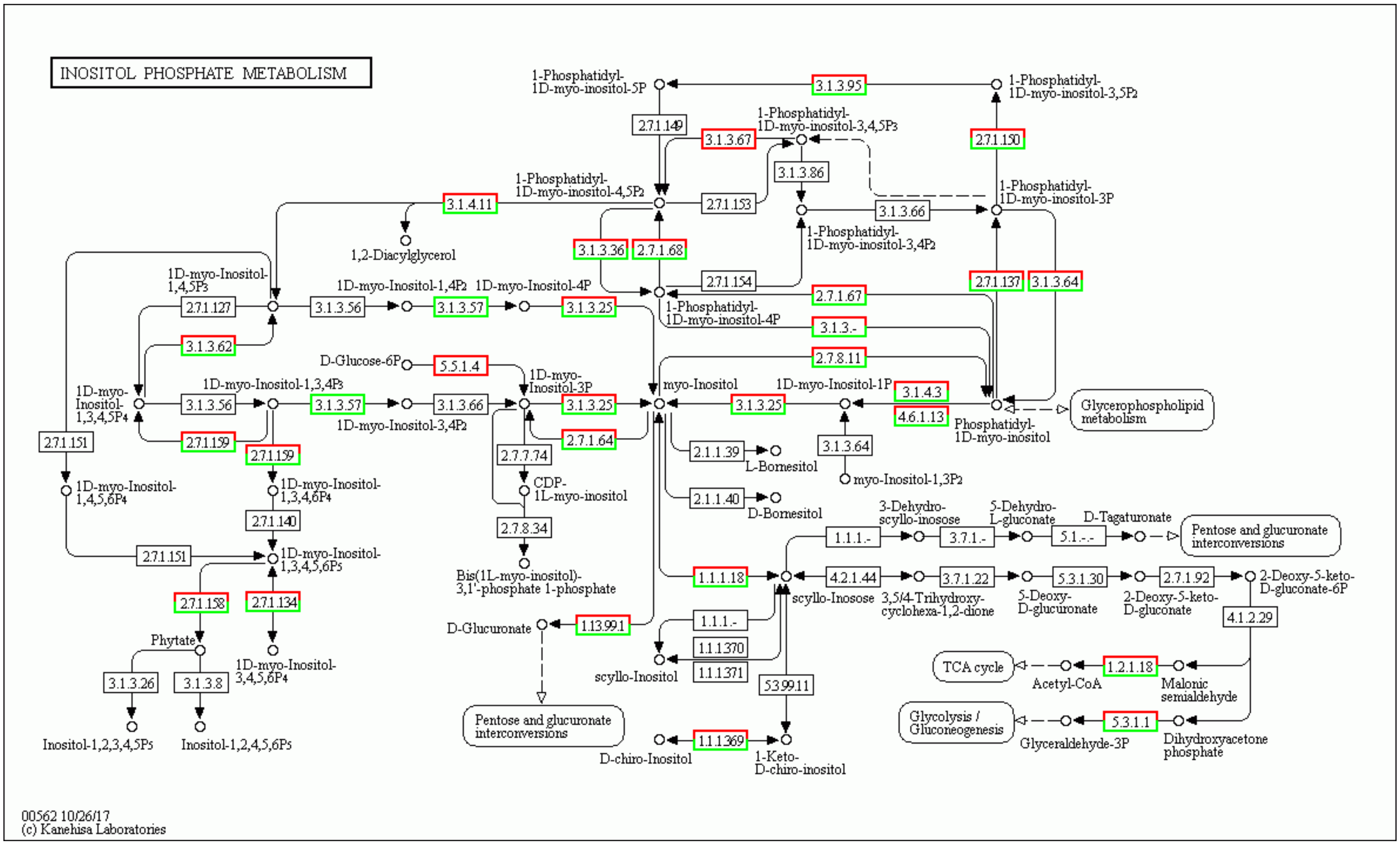
INOSITOL PHOSPHATE METABOLISM
1-Phosphatidyl-1D-myo-inositol-5P
3.1.3.95
1-Phosphatidyl-1D-myo-inositol-3,5P2
2.7.1.149
1-Phosphatidyl-1D-myo-inositol-3,4,5P3
3.1.3.67
2.7.1.150
3.1.3.86
1-Phosphatidyl-1D-myo-inositol-4,5P2
3.1.4.11
2.7.1.153
3.1.3.66
1-Phosphatidyl-1D-myo-inositol-3P
1,2-Diacylglycerol
3.1.3.36
2.7.1.68
1-Phosphatidyl-1D-myo-inositol-3,4P2
1D-myo-Inositol-1,4,5P3
2.7.1.154
2.7.1.137
3.1.3.64
1D-myo-Inositol-1,4P2
1D-myo-Inositol-4P
2.7.1.67
2.7.1.127
3.1.3.56
3.1.3.57
3.1.3.25
1-Phosphatidyl-1D-myo-inositol-4P
3.1.3.-
3.1.3.62
2.7.8.11
D-Glucose-6P
5.5.1.4
1D-myo-Inositol-3P
1D-myo-Inositol-1,3,4,5P4
1D-myo-Inositol-1,3,4P3
myo-Inositol
1D-myo-Inositol-1P
3.1.4.3
Glycerophospholipid metabolism
3.1.3.66
4.6.1.13
Phosphatidyl-1D-myo-inositol
1D-myo-Inositol-3,4P2
2.7.1.64
2.7.1.151
2.7.1.159
2.7.7.74
2.1.1.39
L-Bornesitol
3.1.3.64
myo-Inositol-1,3P2
1D-myo-Inositol-1,4,5,6P4
1D-myo-Inositol-1,3,4,6P4
CDP-1L-myo-inositol
2.1.1.40
D-Bornesitol
3-Dehydro-scyllo-inosose
5-Dehydro-L-gluconate
D-Tagaturonate
2.7.1.140
2.7.8.34
1.1.1.-
3.7.1.-
5.1.-.-
Pentose and glucuronate interconversions
1D-myo-Inositol-1,3,4,5,6P5
Bis(1L-myo-inositol)-3,1'-phosphate 1-phosphate
1.1.1.18
4.2.1.44
3.7.1.22
5.3.1.30
2.7.1.92
2-Deoxy-5-keto-D-gluconate-6P
2.7.1.158
2.7.1.134
scyllo-Inosose
3,5/4-Trihydroxy-cyclohexa-1,2-dione
5-Deoxy-D-glucuronate
2-Deoxy-5-keto-D-gluconate
D-Glucuronate
1.13.99.1
1.1.1.-
4.1.2.29
Phytate
1.1.1.370
1D-myo-Inositol-3,4,5,6P4
scyllo-Inositol
1.1.1.371
TCA cycle
Acetyl-CoA
1.2.1.18
Malonic semialdehyde
3.1.3.26
3.1.3.8
5.3.99.11
Pentose and glucuronate interconversions
Glycolysis / Gluconeogenesis
5.3.1.1
Glyceraldehyde-3P
Dihydroxyacetone phosphate
Inositol-1,2,3,4,5P5
Inositol-1,2,4,5,6P5
D-chiro-Inositol
1.1.1.369
1-Keto-D-chiro-inositol
00562 10/26/17
(c) Kanehisa Laboratories

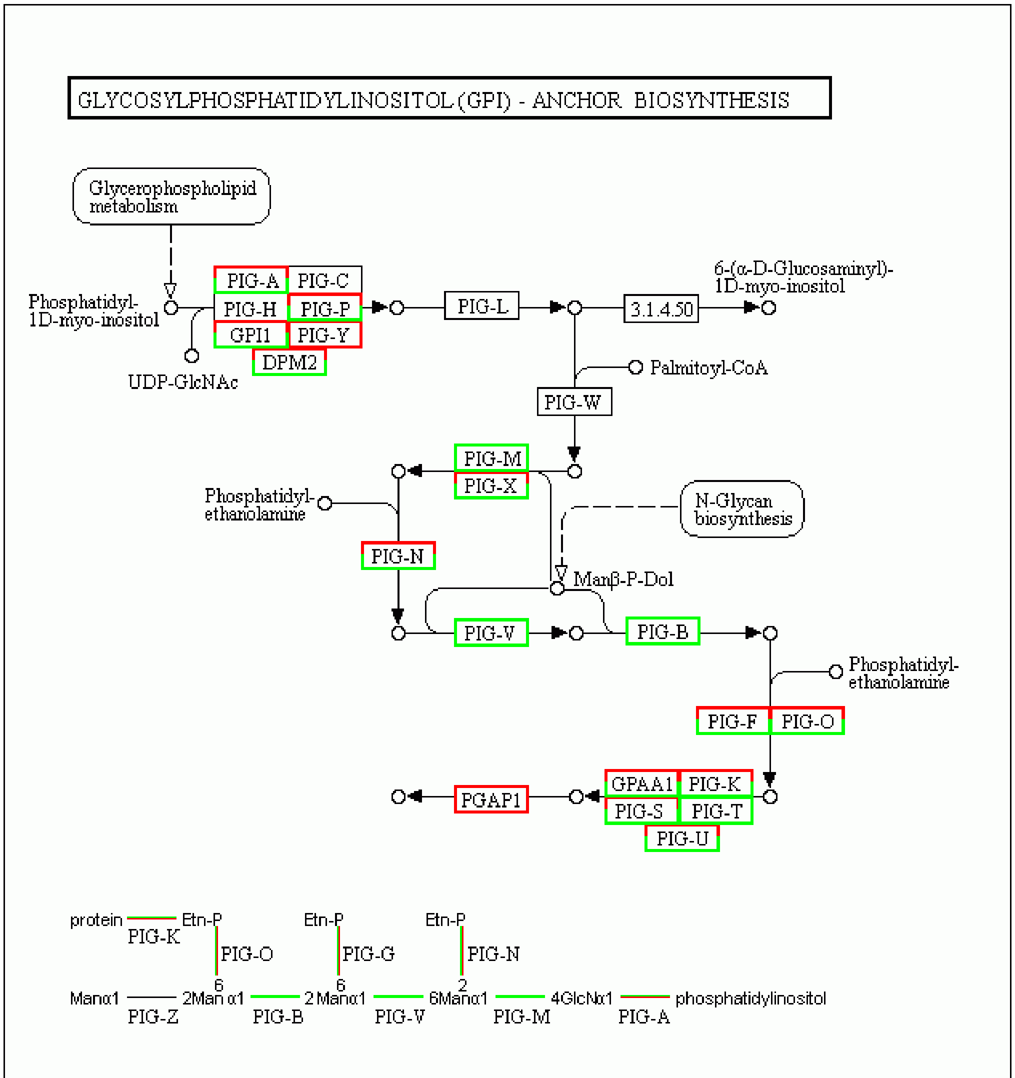

00563 12/27/16
(c) Kanehisa Laboratories

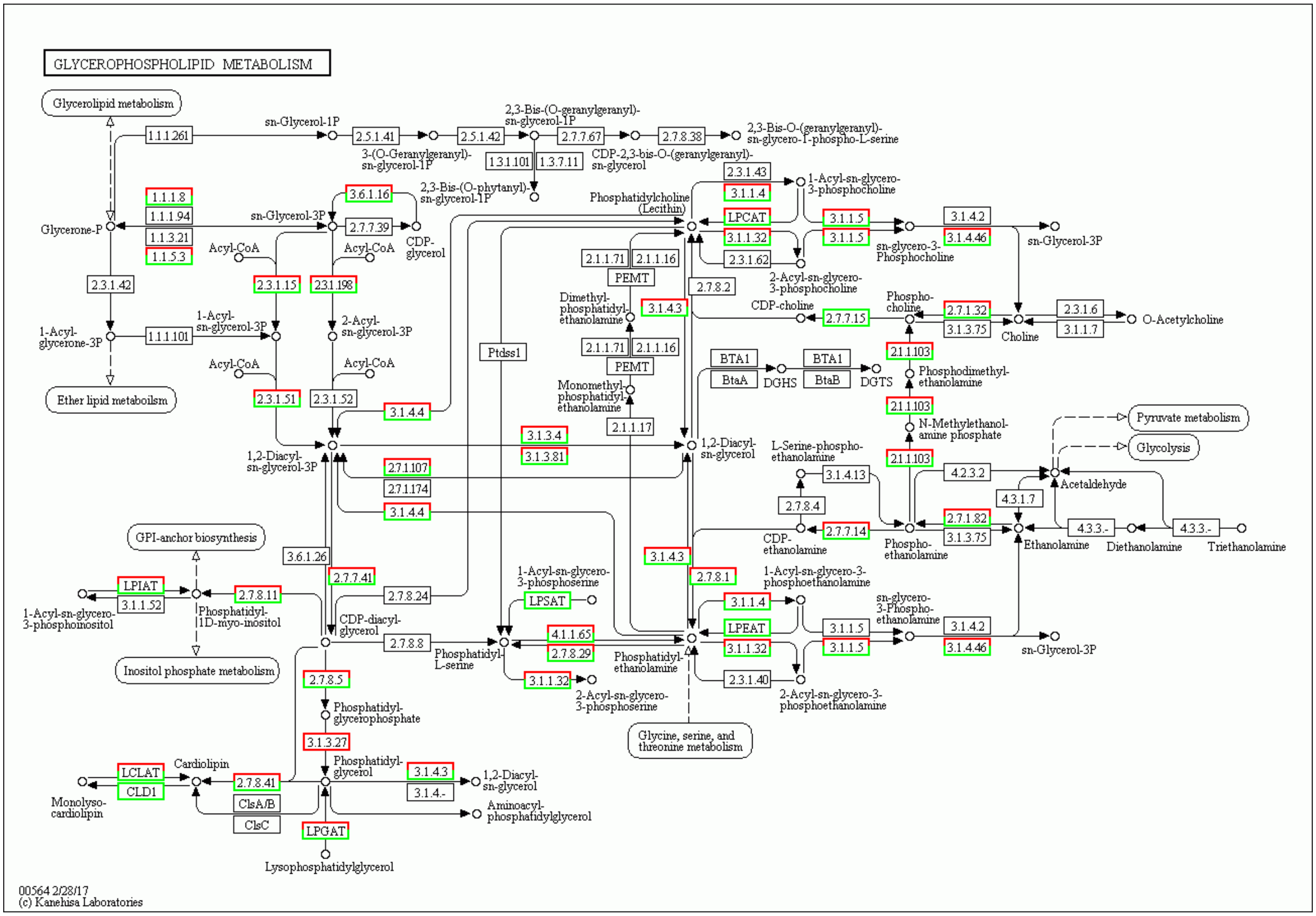
GLYCEROPHOSPHOLIPID METABOLISM
Glycerolipid metabolism
1.1.1.261
sn-Glycerol-1P
2.5.1.41
2.5.1.42
2,3-Bis-(O-geranylgeranyl)-sn-glycerol-1P
2.7.7.67
2.7.8.38
2,3-Bis-O-(geranylgeranyl)-sn-glycero-1-phospho-L-serine
3-(O-Geranylgeranyl)-sn-glycerol-1P
1.3.1.101
1.3.7.11
CDP-2,3-bis-O-(geranylgeranyl)-sn-glycerol
1.1.1.8
1.1.1.94
1.1.3.21
1.1.5.3
Glycerone-P
3.6.1.16
2,3-Bis-(O-phytanyl)-sn-glycerol-1P
sn-Glycerol-3P
2.7.7.39
CDP-glycerol
Acyl-CoA
Acyl-CoA
2.3.1.15
2.3.1.198
2.3.1.42
1-Acyl-glycerone-3P
1.1.1.101
1-Acyl-sn-glycerol-3P
2-Acyl-sn-glycerol-3P
Ether lipid metaboilsm
Acyl-CoA
Acyl-CoA
2.3.1.51
2.3.1.52
3.1.4.4
1,2-Diacyl-sn-glycerol-3P
2.7.1.107
2.7.1.174
3.1.4.4
3.1.3.4
3.1.3.81
Phosphatidylcholine (Lecithin)
2.3.1.43
3.1.1.4
LPCAT
3.1.1.32
2.3.1.62
1-Acyl-sn-glycero-3-phosphocholine
2-Acyl-sn-glycero-3-phosphocholine
3.1.1.5
3.1.1.5
sn-glycero-3-Phosphocholine
3.1.4.2
3.1.4.46
sn-Glycerol-3P
2.1.1.71
2.1.1.16
PEMT
Dimethyl-phosphatidyl-ethanolamine
3.1.4.3
2.7.8.2
CDP-choline
2.7.7.15
Phospho-choline
2.7.1.32
3.1.3.75
Choline
2.3.1.6
3.1.1.7
O-Acetylcholine
Ptdss1
2.1.1.71
2.1.1.16
PEMT
Monomethyl-phosphatidyl-ethanolamine
2.1.1.17
BTA1
BtaA
DGHS
BTA1
BtaB
DGTS
2.1.1.103
Phosphodimethyl-ethanolamine
2.1.1.103
N-Methylethanol-amine phosphate
2.1.1.103
1,2-Diacyl-sn-glycerol
L-Serine-phospho-ethanolamine
3.1.4.13
2.7.8.4
CDP-ethanolamine
2.7.7.14
Phospho-ethanolamine
4.2.3.2
4.3.1.7
2.7.1.82
3.1.3.75
Ethanolamine
Pyruvate metabolism
Glycolysis
Acetaldehyde
4.3.3.-
Diethanolamine
4.3.3.-
Triethanolamine
GPI-anchor biosynthesis
3.6.1.26
LPIAT
3.1.1.52
1-Acyl-sn-glycero-3-phosphoinositol
2.7.8.11
Phosphatidyl-1D-myo-inositol
Inositol phosphate metabolism
2.7.7.41
2.7.8.24
CDP-diacyl-glycerol
2.7.8.8
1-Acyl-sn-glycero-3-phosphoserine
LPSAT
Phosphatidyl-L-serine
4.1.1.65
2.7.8.29
3.1.1.32
2-Acyl-sn-glycero-3-phosphoserine
3.1.4.3
2.7.8.1
Phosphatidyl-ethanolamine
1-Acyl-sn-glycero-3-phosphoethanolamine
3.1.1.4
LPEAT
3.1.1.32
2.3.1.40
2-Acyl-sn-glycero-3-phosphoethanolamine
sn-glycero-3-Phospho-ethanolamine
3.1.1.5
3.1.1.5
3.1.4.2
3.1.4.46
sn-Glycerol-3P
Glycine, serine, and threonine metabolism
2.7.8.5
Phosphatidyl-glycerophosphate
3.1.3.27
Phosphatidyl-glycerol
3.1.4.3
3.1.4.-
1,2-Diacyl-sn-glycerol
Aminoacyl-phosphatidylglycerol
LCLAT
CLD1
Cardiolipin
2.7.8.41
ClsA/B
ClsC
Monolyso-cardiolipin
LPGAT
Lysophosphatidylglycerol
00564 2/28/17
(c) Kanehisa Laboratories

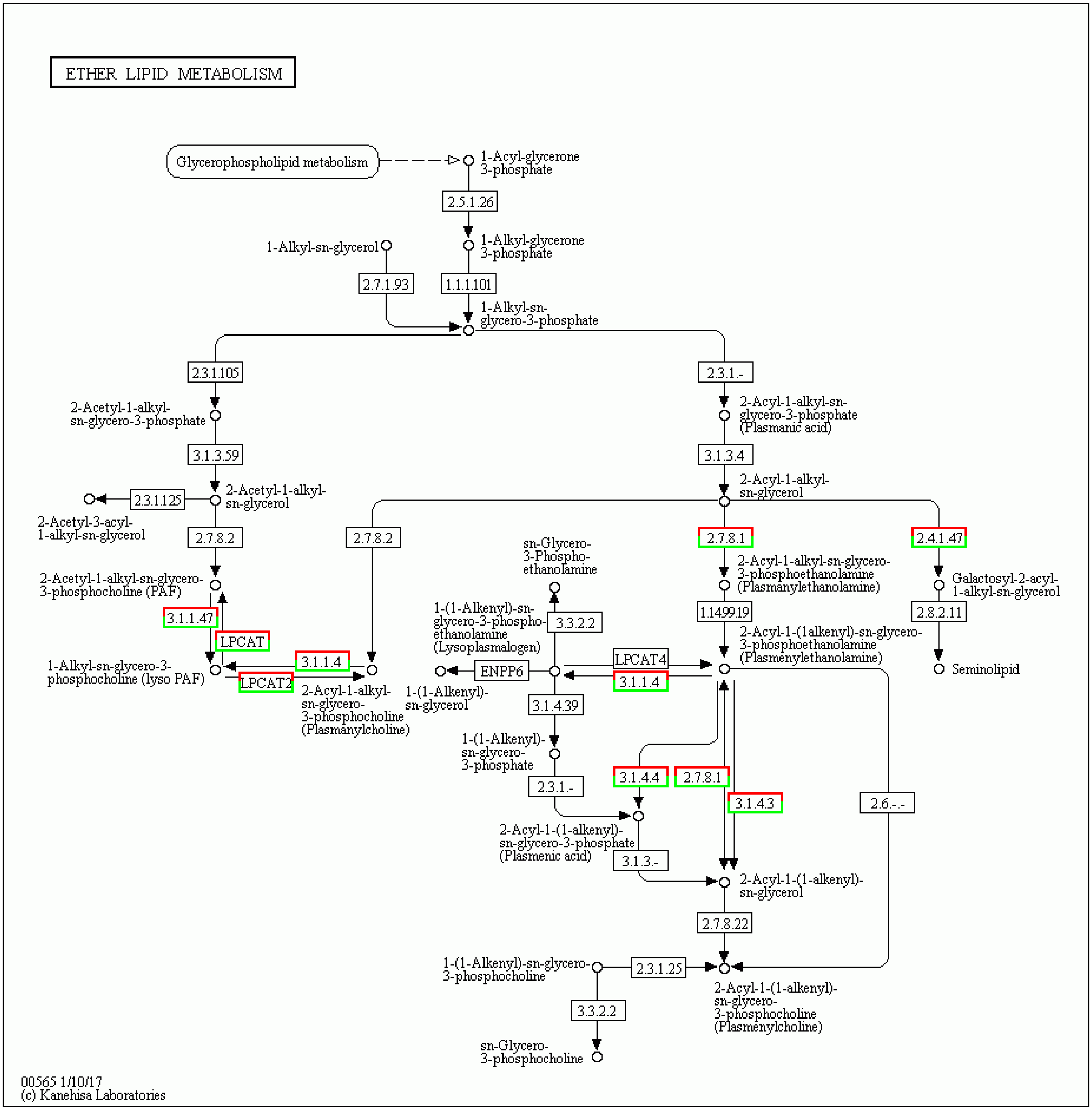
ETHER LIPID METABOLISM
Glycerophospholipid metabolism
1-Acyl-glycerone 3-phosphate
2.5.1.26
1-Alkyl-glycerone 3-phosphate
1-Alkyl-sn-glycerol
2.7.1.93
1.1.1.101
1-Alkyl-sn-glycero-3-phosphate
2.3.1.105
2.3.1.-
2-Acetyl-1-alkyl-sn-glycero-3-phosphate
2-Acyl-1-alkyl-sn-glycero-3-phosphate (Plasmanic acid)
3.1.3.59
3.1.3.4
2.3.1.125
2-Acetyl-1-alkyl-sn-glycerol
2-Acyl-1-alkyl-sn-glycerol
2-Acetyl-3-acyl-1-alkyl-sn-glycerol
2.7.8.2
2.7.8.2
2.7.8.1
2.4.1.47
sn-Glycero-3-Phospho-ethanolamine
2-Acyl-1-alkyl-sn-glycero-3-phosphoethanolamine (Plasmanylethanolamine)
2-Acetyl-1-alkyl-sn-glycero-3-phosphocholine (PAF)
Galactosyl-2-acyl-1-alkyl-sn-glycerol
1-(1-Alkenyl)-sn-glycero-3-phospho-ethanolamine (Lysoplasmalogen)
3.3.2.2
1.14.99.19
2.8.2.11
3.1.1.47
LPCAT
2-Acyl-1-(1alkenyl)-sn-glycero-3-phosphoethanolamine (Plasmenylethanolamine)
LPCAT4
3.1.1.4
1-Alkyl-sn-glycero-3-phosphocholine (lyso PAF)
ENPP6
Seminolipid
LPCAT2
3.1.1.4
2-Acyl-1-alkyl-sn-glycero-3-phosphocholine (Plasmanylcholine)
1-(1-Alkenyl)-sn-glycerol
3.1.4.39
1-(1-Alkenyl)-sn-glycero-3-phosphate
3.1.4.4
2.7.8.1
3.1.4.3
2.3.1.-
2.6.-.-
2-Acyl-1-(1-alkenyl)-sn-glycero-3-phosphate (Plasmenic acid)
3.1.3.-
2-Acyl-1-(1-alkenyl)-sn-glycerol
2.7.8.22
1-(1-Alkenyl)-sn-glycero-3-phosphocholine
2.3.1.25
2-Acyl-1-(1-alkenyl)-sn-glycero-3-phosphocholine (Plasmenylcholine)
3.3.2.2
sn-Glycero-3-phosphocholine
00565 1/10/17
(c) Kanehisa Laboratories

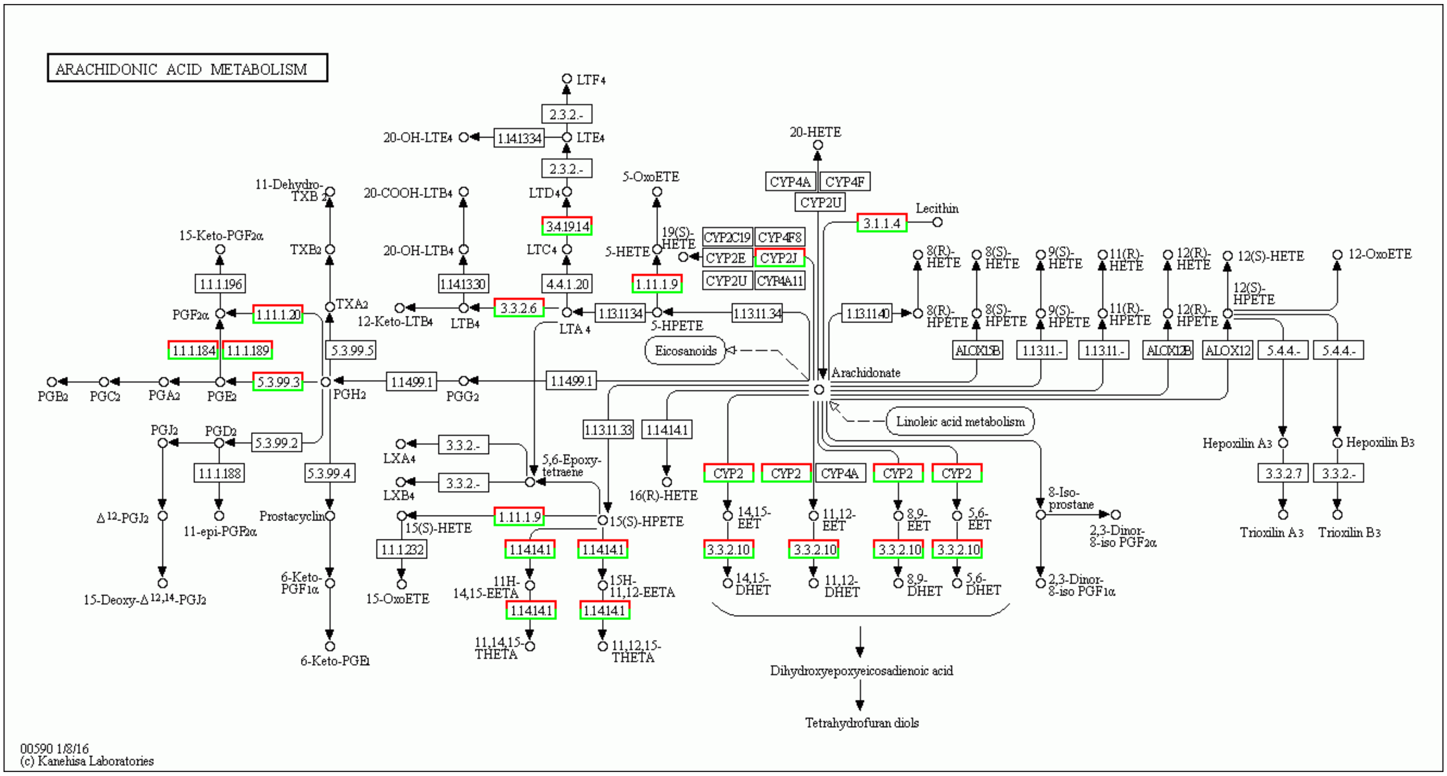
ARACHIDONIC ACID METABOLISM
LTF4
2.3.2.-
20-OH-LTE4
1.14.13.34
LTE4
2.3.2.-
20-HETE
CYP4A
CYP4F
CYP2U
5-OxoETE
11-Dehydro-TXB2
20-COOH-LTB4
LTD4
3.4.19.14
Lecithin
3.1.1.4
19(S)-HETE
CYP2C19
CYP4F8
CYP2E
CYP2J
CYP2U
CYP4A11
15-Keto-PGF2α
TXB2
20-OH-LTB4
LTC4
5-HETE
8(R)-HETE
8(S)-HETE
9(S)-HETE
11(R)-HETE
12(R)-HETE
12(S)-HETE
12-OxoETE
1.1.1.196
1.14.13.30
4.4.1.20
1.11.1.9
12(S)-HPETE
TXA2
PGF2α
1.1.1.20
12-Keto-LTB4
LTB4
3.3.2.6
LTA4
1.13.11.34
5-HPETE
1.13.11.34
1.13.11.40
8(R)-HPETE
8(S)-HPETE
9(S)-HPETE
11(R)-HPETE
12(R)-HPETE
1.1.1.184
1.1.1.189
5.3.99.5
Eicosanoids
ALOX15B
1.13.11.-
1.13.11.-
ALOX12B
ALOX12
5.4.4.-
5.4.4.-
PGB2
PGC2
PGA2
PGE2
5.3.99.3
PGH2
1.14.99.1
PGG2
1.14.99.1
Arachidonate
Linoleic acid metabolism
PGJ2
PGD2
5.3.99.2
1.13.11.33
1.14.14.1
3.3.2.-
LXA4
5,6-Epoxy-tetraene
Hepoxilin A3
Hepoxilin B3
1.1.1.188
5.3.99.4
3.3.2.-
LXB4
CYP2
CYP2
CYP4A
CYP2
CYP2
3.3.2.7
3.3.2.-
Δ12-PGJ2
11-epi-PGF2α
Prostacyclin
1.11.1.9
15(S)-HETE
15(S)-HPETE
16(R)-HETE
14,15-EET
11,12-EET
8,9-EET
5,6-EET
8-Iso-prostane
2,3-Dinor-8-iso PGF2α
Trioxilin A3
Trioxilin B3
1.1.1.232
1.14.14.1
1.14.14.1
3.3.2.10
3.3.2.10
3.3.2.10
3.3.2.10
6-Keto-PGF1α
15-OxoETE
11H-14,15-EETA
15H-11,12-EETA
14,15-DHET
11,12-DHET
8,9-DHET
5,6-DHET
2,3-Dinor-8-iso PGF1α
15-Deoxy-Δ12,14-PGJ2
1.14.14.1
1.14.14.1
11,14,15-THETA
11,12,15-THETA
Dihydroxyepoxyeicosadienoic acid
6-Keto-PGE1
Tetrahydrofuran diols
00590 1/8/16
(c) Kanehisa Laboratories

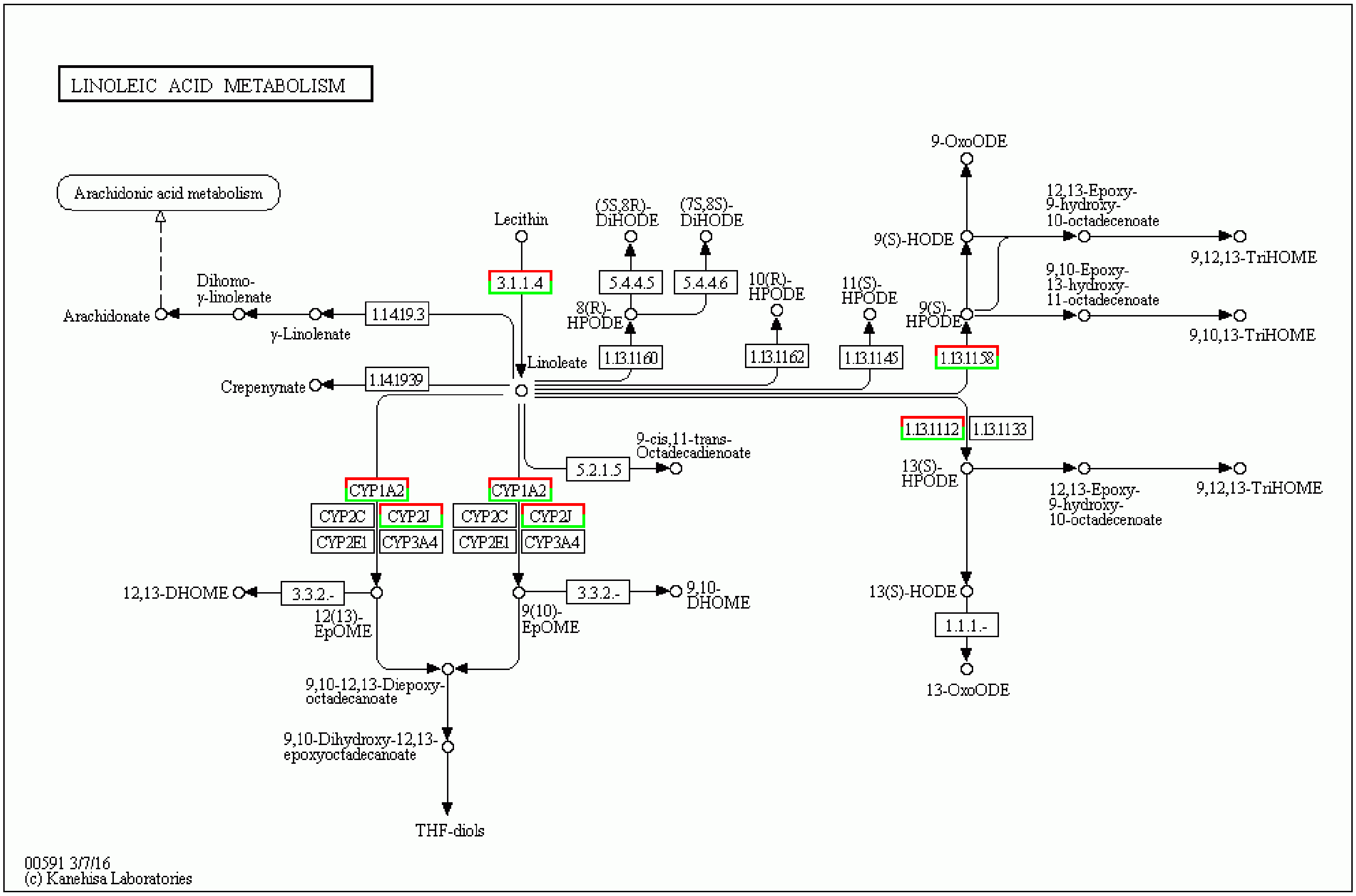
LINOLEIC ACID METABOLISM
9-OxoODE
Arachidonic acid metabolism
12,13-Epoxy-9-hydroxy-10-octadecenoate
Lecithin
(5S,8R)-DiHODE
(7S,8S)-DiHODE
9(S)-HODE
9,12,13-TriHOME
3.1.1.4
5.4.4.5
5.4.4.6
10(R)-HPODE
11(S)-HPODE
9,10-Epoxy-13-hydroxy-11-octadecenoate
Dihomo-γ-linolenate
8(R)-HPODE
9(S)-HPODE
Arachidonate
1.14.19.3
9,10,13-TriHOME
γ-Linolenate
Linoleate
1.13.11.60
1.13.11.62
1.13.11.45
1.13.11.58
Crepenynate
1.14.19.39
1.13.11.12
1.13.11.33
9-cis,11-trans-Octadecadienoate
5.2.1.5
13(S)-HPODE
12,13-Epoxy-9-hydroxy-10-octadecenoate
9,12,13-TriHOME
CYP1A2
CYP1A2
CYP2C
CYP2J
CYP2C
CYP2J
CYP2E1
CYP3A4
CYP2E1
CYP3A4
12,13-DHOME
3.3.2.-
3.3.2.-
9,10-DHOME
13(S)-HODE
12(13)-EpOME
9(10)-EpOME
1.1.1.-
9,10-12,13-Diepoxy-octadecanoate
13-OxoODE
9,10-Dihydroxy-12,13-epoxyoctadecanoate
THF-diols
00591 3/7/16
(c) Kanehisa Laboratories

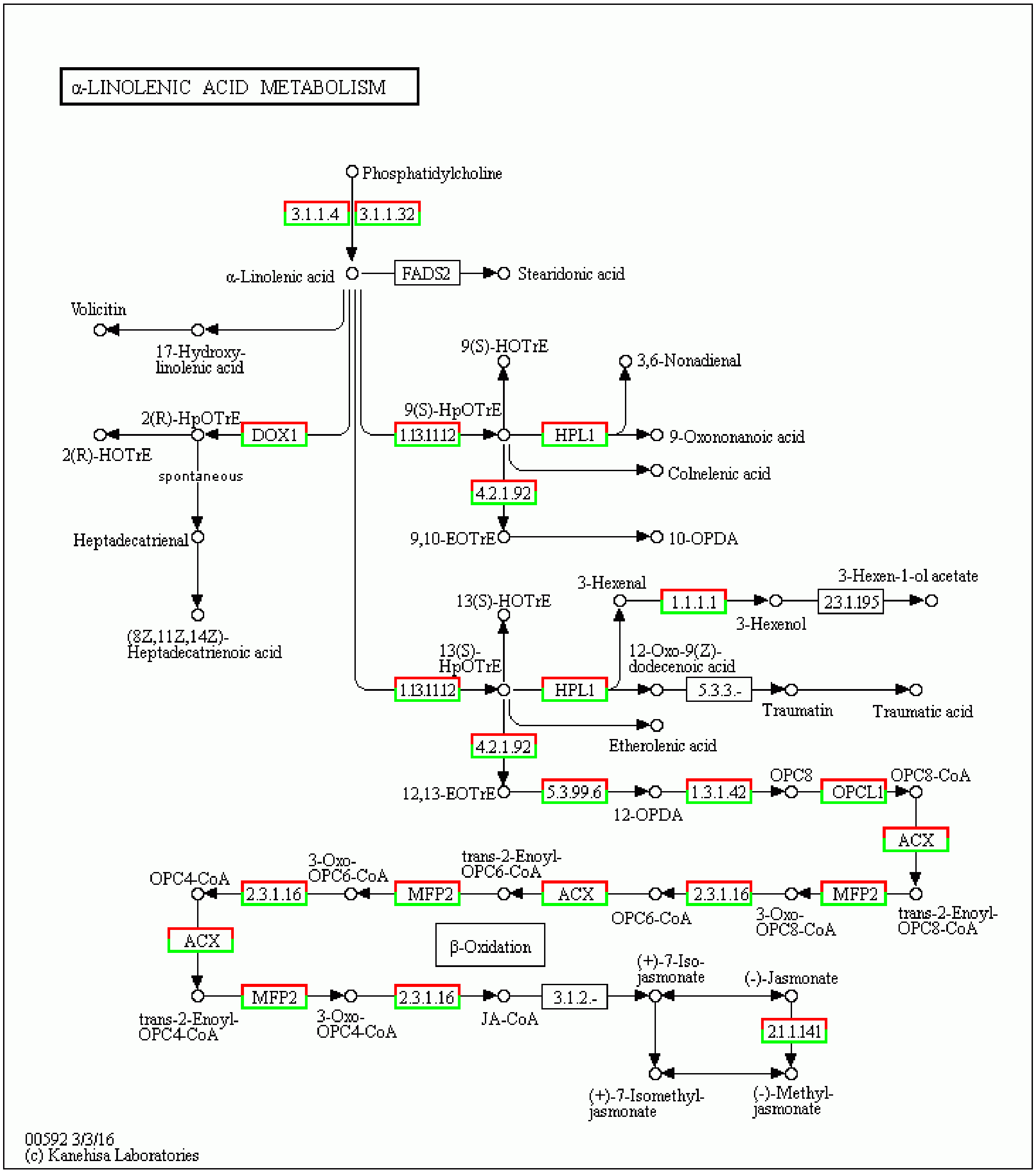
α-LINOLENIC ACID METABOLISM
Phosphatidylcholine
3.1.1.4
3.1.1.32
α-Linolenic acid
FADS2
Stearidonic acid
Volicitin
17-Hydroxy-
linolenic acid
9(S)-HOTrE
3,6-Nonadienal
2(R)-HpOTrE
DOX1
2(R)-HOTrE
9(S)-HpOTrE
1.13.11.12
HPL1
9-Oxononanoic acid
spontaneous
Colnelenic acid
4.2.1.92
Heptadecatrienal
9,10-EOTrE
10-OPDA
3-Hexenal
1.1.1.1
3-Hexen-1-ol acetate
2.3.1.195
13(S)-HOTrE
3-Hexenol
(8Z,11Z,14Z)-
Heptadecatrienoic acid
13(S)-
HpOTrE
12-Oxo-9(Z)-
dodecenoic acid
1.13.11.12
HPL1
5.3.3.-
Traumatin
Traumatic acid
Etherolenic acid
4.2.1.92
OPC8
OPC8-CoA
12,13-EOTrE
5.3.99.6
1.3.1.42
OPCL1
12-OPDA
ACX
3-Oxo-
OPC6-CoA
trans-2-Enoyl-
OPC6-CoA
OPC4-CoA
2.3.1.16
MFP2
ACX
2.3.1.16
MFP2
OPC6-CoA
3-Oxo-
OPC8-CoA
trans-2-Enoyl-
OPC8-CoA
ACX
β-Oxidation
(+)-7-Iso-
jasmonate
(-)-Jasmonate
MFP2
2.3.1.16
3.1.2.-
2.1.1.141
trans-2-Enoyl-
OPC4-CoA
3-Oxo-
OPC4-CoA
JA-CoA
(+)-7-Isomethyl-
jasmonate
(-)-Methyl-
jasmonate
00592 3/3/16
(c) Kanehisa Laboratories

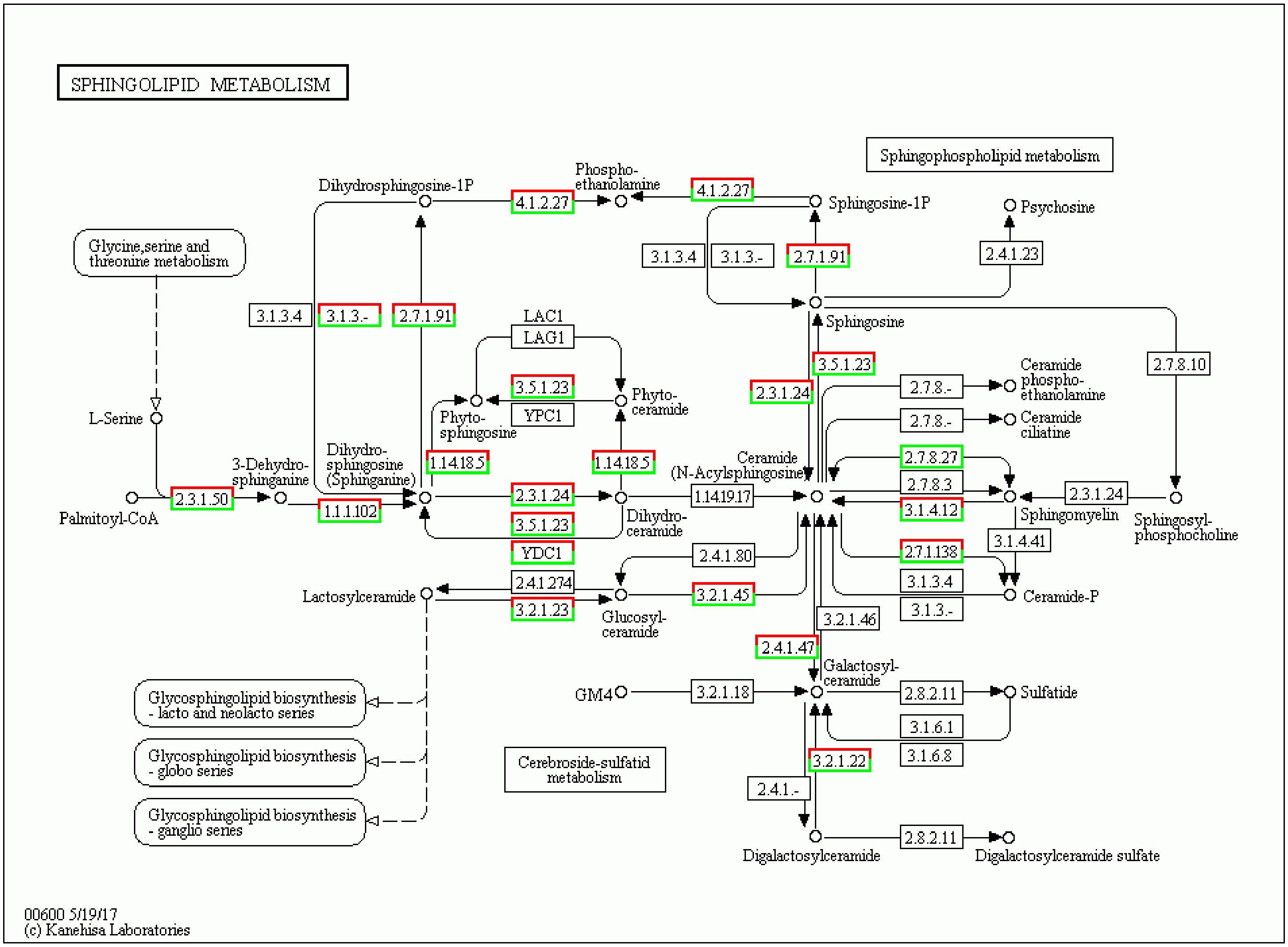
SPHINGOLIPID METABOLISM
Sphingophospholipid metabolism
Glycine,serine and threonine metabolism
Dihydrosphingosine-1P
Phospho-ethanolamine
Sphingosine-1P
Psychosine
Sphingosine
L-Serine
Palmitoyl-CoA
3-Dehydro-sphinganine
Dihydro-sphingosine (Sphinganine)
Phyto-sphingosine
Phyto-ceramide
Dihydro-ceramide
Ceramide (N-Acylsphingosine)
Ceramide phospho-ethanolamine
Ceramide ciliatine
Sphingomyelin
Sphingosyl-phosphocholine
Ceramide-P
Lactosylceramide
Glucosyl-ceramide
Galactosyl-ceramide
GM4
Sulfatide
Digalactosylceramide
Digalactosylceramide sulfate
Glycosphingolipid biosynthesis - lacto and neolacto series
Glycosphingolipid biosynthesis - globo series
Glycosphingolipid biosynthesis - ganglio series
Cerebroside-sulfatid metabolism
LAC1
LAG1
YPC1
YDC1
2.3.1.50
1.1.1.102
3.1.3.4
3.1.3.-
2.7.1.91
4.1.2.27
3.5.1.23
1.14.18.5
2.3.1.24
1.14.19.17
2.7.8.-
2.7.8.27
2.7.8.3
3.1.4.12
2.7.1.138
3.1.3.4
3.1.3.-
3.1.4.41
2.3.1.24
2.7.8.10
2.4.1.23
2.4.1.80
2.4.1.274
3.2.1.23
3.2.1.45
3.2.1.46
2.4.1.47
3.2.1.18
2.8.2.11
3.1.6.1
3.1.6.8
3.2.1.22
2.4.1.-
00600 5/19/17
(c) Kanehisa Laboratories

GLYCOSPHINGOLIPID BIOSYNTHESIS - LACTO AND NEOLACTO SERIES

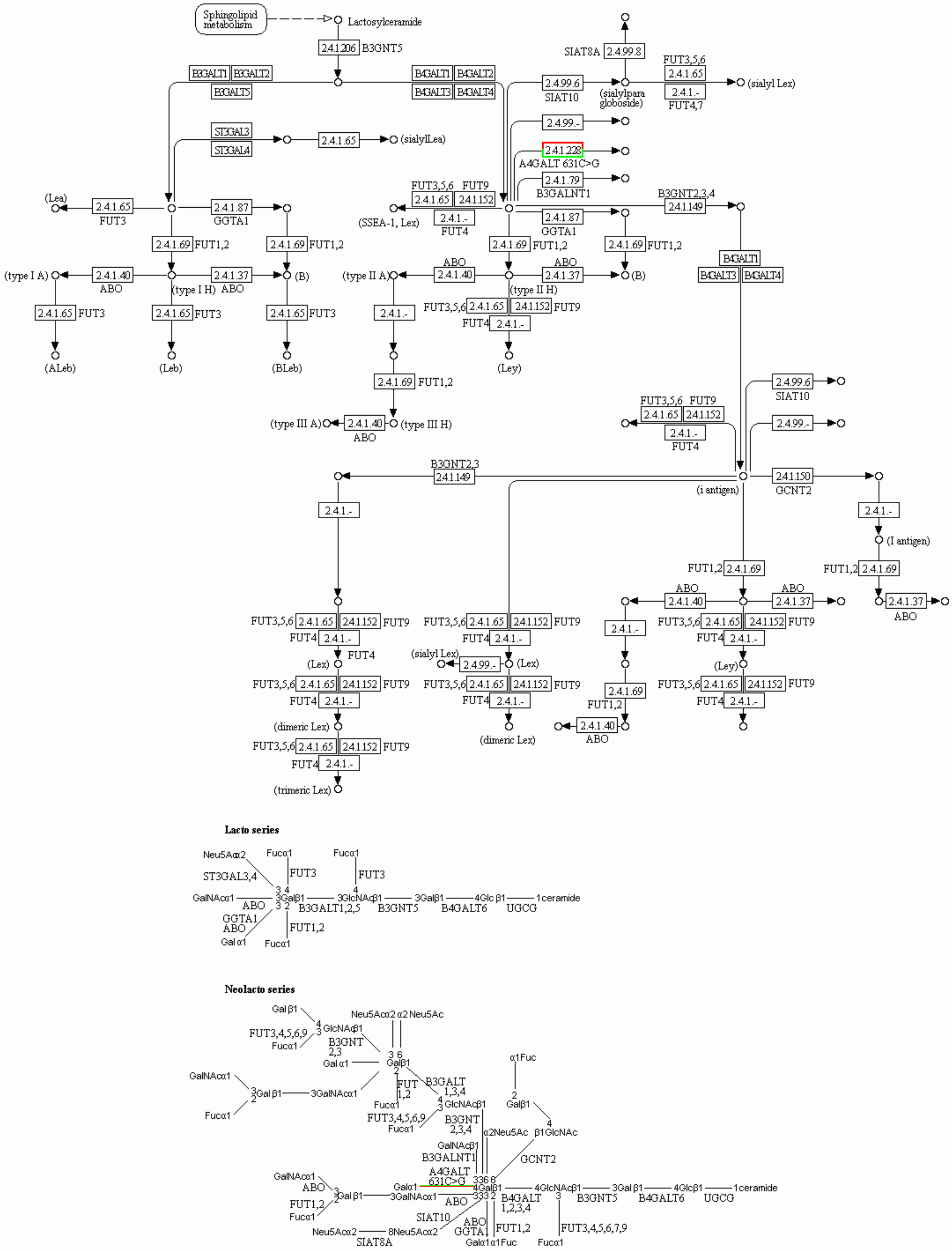

00601 4/11/17
(c) Kanehisa Laboratories

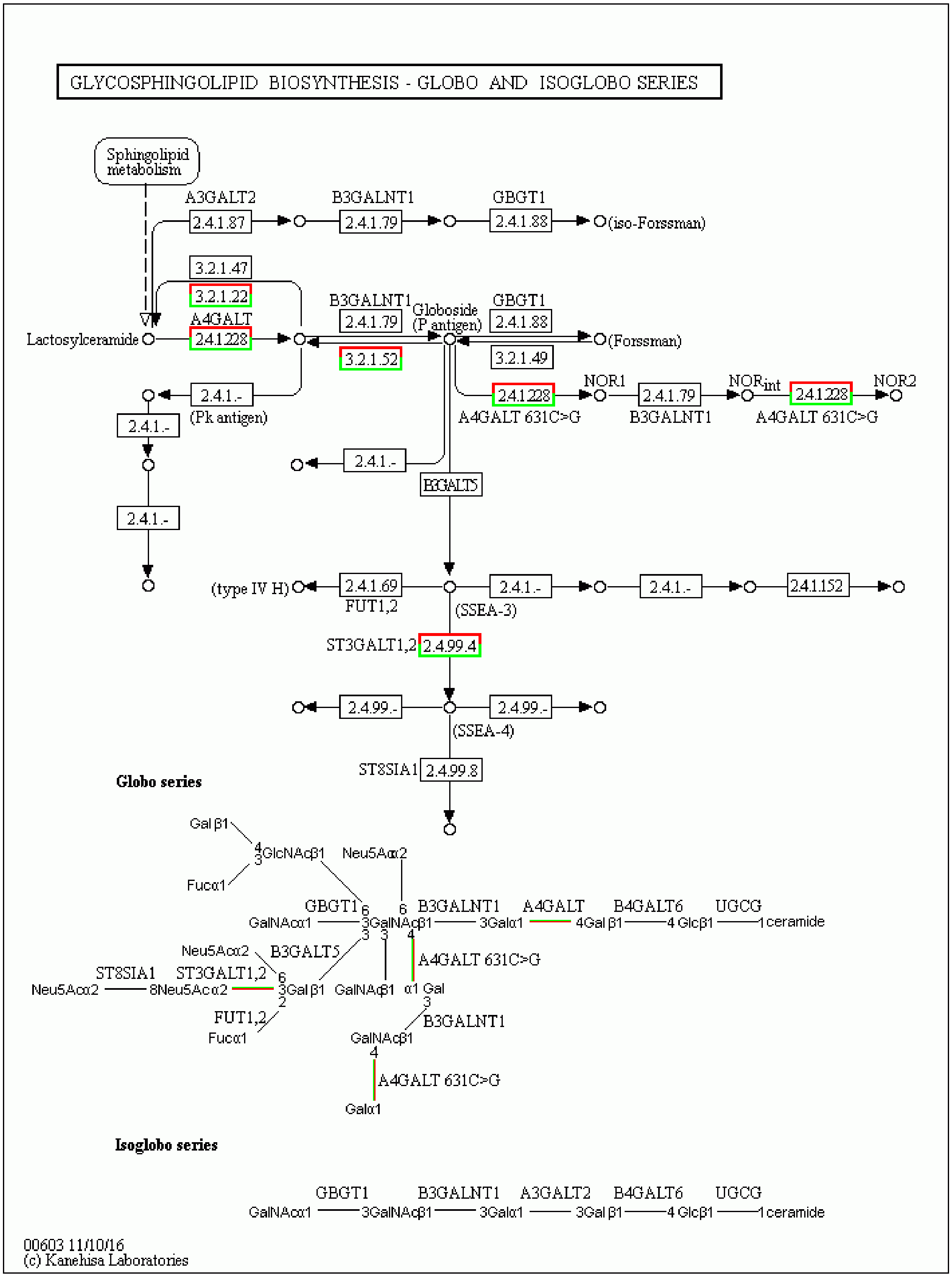
GLYCOSPHINGOLIPID BIOSYNTHESIS - GLOBO AND ISOGLOBO SERIES
Sphingolipid metabolism
A3GALT2
2.4.1.87
B3GALNT1
2.4.1.79
GBGT1
2.4.1.88
(iso-Forssman)
3.2.1.47
3.2.1.22
A4GALT
2.4.1.228
Lactosylceramide
B3GALNT1
2.4.1.79
Globoside (P antigen)
GBGT1
2.4.1.88
(Forssman)
3.2.1.52
3.2.1.49
2.4.1.-
(Pk antigen)
NOR1
NORint
NOR2
2.4.1.228
A4GALT 631C>G
2.4.1.79
B3GALNT1
2.4.1.228
A4GALT 631C>G
2.4.1.-
2.4.1.-
B3GALT5
2.4.1.-
(type IV H)
2.4.1.69
FUT1,2
2.4.1.-
2.4.1.-
2.4.1.152
(SSEA-3)
ST3GALT1,2
2.4.99.4
2.4.99.-
2.4.99.-
(SSEA-4)
ST8SIA1
2.4.99.8
Globo series
Gal β1
4 3 GlcNAcβ1
Fucα1
Neu5Acα2
GBGT1
B3GALNT1
A4GALT
B4GALT6
UGCG
GalNAcα1
3GalNAcβ1
3Galα1
4Gal β1
4 Glcβ1
1ceramide
Neu5Acα2
B3GALT5
ST8SIA1
ST3GALT1,2
Neu5Acα2
8Neu5Acα2
3Gal β1
GalNAcβ1
α1 Gal
A4GALT 631C>G
FUT1,2
Fucα1
B3GALNT1
GalNAcβ1
A4GALT 631C>G
Galα1
Isoglobo series
GBGT1
B3GALNT1
A3GALT2
B4GALT6
UGCG
GalNAcα1
3GalNAcβ1
3Galα1
3Gal β1
4 Glcβ1
1ceramide
00603 11/10/16
(c) Kanehisa Laboratories

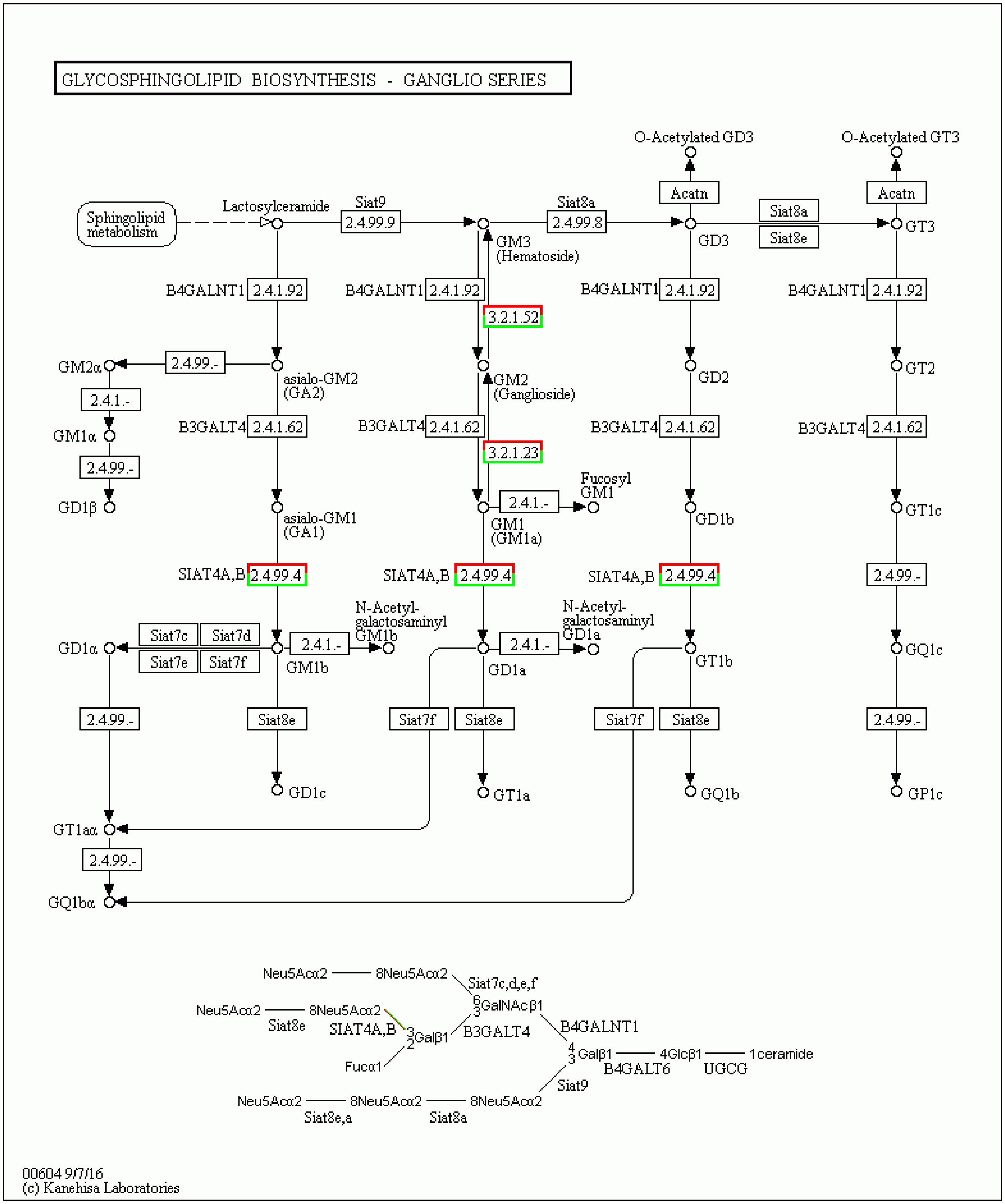
GLYCOSPHINGOLIPID BIOSYNTHESIS - GANGLIO SERIES
O-Acetylated GD3
O-Acetylated GT3
Acatn
Acatn
Sphingolipid metabolism
Lactosylceramide
Siat9
2.4.99.9
Siat8a
2.4.99.8
Siat8a
Siat8e
GM3 (Hematoside)
GD3
GT3
B4GALNT1 2.4.1.92
B4GALNT1 2.4.1.92
B4GALNT1 2.4.1.92
B4GALNT1 2.4.1.92
3.2.1.52
GM2α
2.4.99.-
asialo-GM2 (GA2)
GM2 (Ganglioside)
GD2
GT2
2.4.1.-
B3GALT4 2.4.1.62
B3GALT4 2.4.1.62
B3GALT4 2.4.1.62
B3GALT4 2.4.1.62
GM1α
3.2.1.23
2.4.99.-
Fucosyl GM1
2.4.1.-
GD1β
asialo-GM1 (GA1)
GM1 (GM1a)
GD1b
GT1c
SIAT4A,B 2.4.99.4
SIAT4A,B 2.4.99.4
SIAT4A,B 2.4.99.4
2.4.99.-
Siat7c
Siat7d
Siat7e
Siat7f
N-Acetyl-galactosaminyl GM1b
N-Acetyl-galactosaminyl GD1a
GD1α
2.4.1.-
2.4.1.-
GM1b
GD1a
GT1b
GQ1c
2.4.99.-
Siat8e
Siat7f
Siat8e
Siat7f
Siat8e
2.4.99.-
GD1c
GT1a
GQ1b
GP1c
GT1aα
2.4.99.-
GQ1bα
Neu5Acα2 — 8Neu5Acα2
Siat7c,d,e,f
6 3 GalNAcβ1
Neu5Acα2 — 8Neu5Acα2
Siat8e
SIAT4A,B
3 2 Galβ1
B3GALT4
B4GALNT1
Fucα1
4 3 Galβ1 — 4Glcβ1 — 1ceramide
B4GALT6
UGCG
Siat9
Neu5Acα2 — 8Neu5Acα2 — 8Neu5Acα2
Siat8e,a
Siat8a
00604 9/7/16
(c) Kanehisa Laboratories

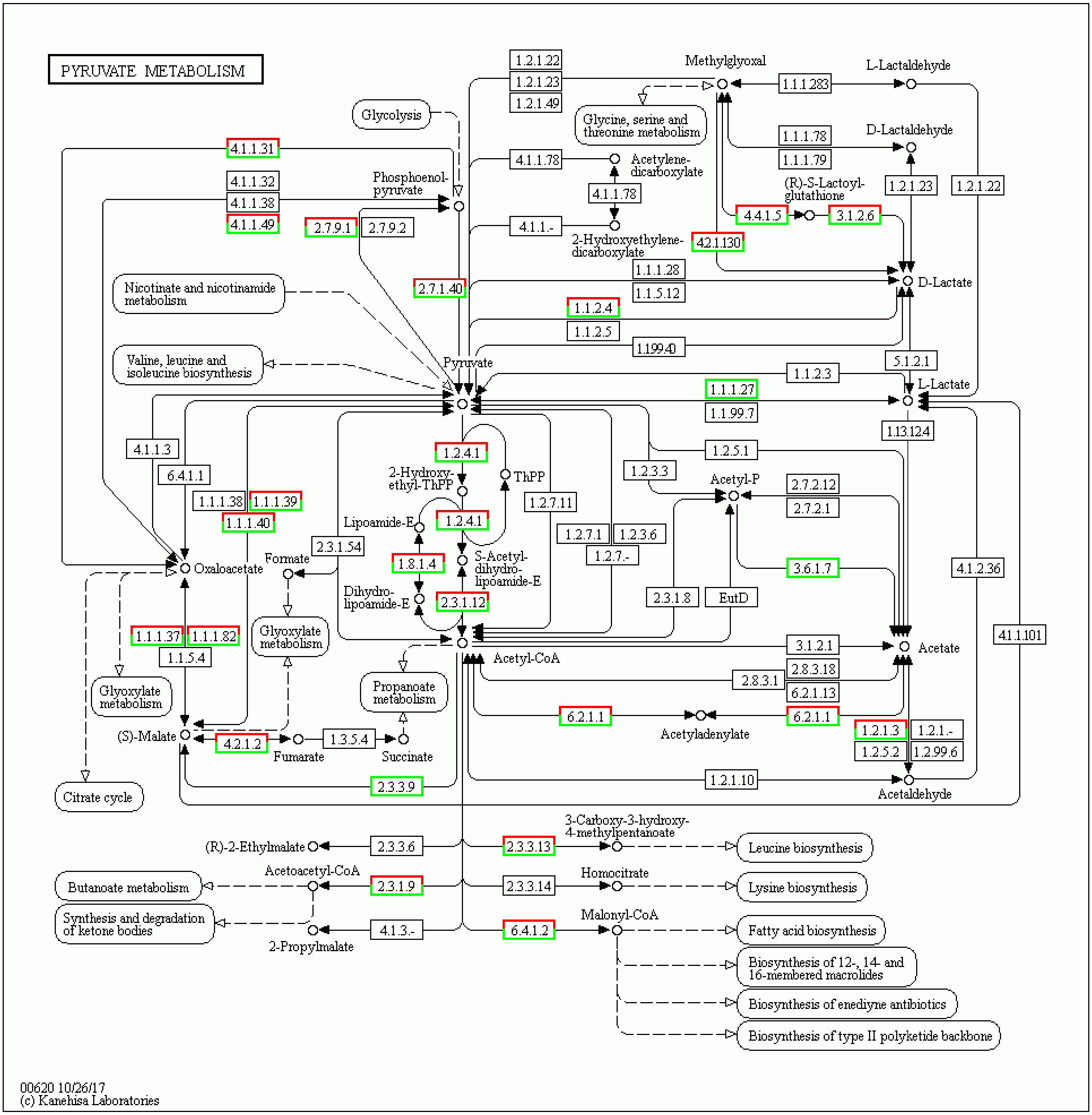
PYRUVATE METABOLISM
Glycolysis
Methylglyoxal
L-Lactaldehyde
1.2.1.22
1.2.1.23
1.2.1.49
1.1.1.283
Glycine, serine and threonine metabolism
1.1.1.78
1.1.1.79
D-Lactaldehyde
4.1.1.31
4.1.1.32
4.1.1.38
4.1.1.49
Phosphoenol-pyruvate
4.1.1.78
Acetylene-dicarboxylate
2.7.9.1
2.7.9.2
4.1.1.-
2-Hydroxyethylene-dicarboxylate
(R)-S-Lactoyl-glutathione
4.4.1.5
3.1.2.6
1.2.1.23
1.2.1.22
4.2.1.130
1.1.1.28
1.1.5.12
D-Lactate
Nicotinate and nicotinamide metabolism
2.7.1.40
1.1.2.4
1.1.2.5
1.1.99.40
Valine, leucine and isoleucine biosynthesis
Pyruvate
5.1.2.1
1.1.2.3
L-Lactate
1.1.1.27
1.1.99.7
1.13.12.4
4.1.1.3
6.4.1.1
1.2.4.1
1.2.5.1
1.2.3.3
2-Hydroxy-ethyl-ThPP
ThPP
Acetyl-P
2.7.2.12
2.7.2.1
1.1.1.38
1.1.1.39
1.1.1.40
1.2.7.11
Lipoamide-E
1.2.4.1
1.2.7.1
1.2.3.6
1.2.7.-
2.3.1.54
Formate
Oxaloacetate
1.8.1.4
S-Acetyl-dihydro-lipoamide-E
3.6.1.7
4.1.2.36
Dihydro-lipoamide-E
2.3.1.12
2.3.1.8
EutD
1.1.1.37
1.1.1.82
Glyoxylate metabolism
4.1.1.101
3.1.2.1
Acetate
1.1.5.4
Acetyl-CoA
2.8.3.18
2.8.3.1
6.2.1.13
Propanoate metabolism
Glyoxylate metabolism
6.2.1.1
Acetyladenylate
6.2.1.1
1.2.1.3
1.2.1.-
1.2.5.2
1.2.99.6
(S)-Malate
4.2.1.2
Fumarate
1.3.5.4
Succinate
2.3.3.9
1.2.1.10
Acetaldehyde
Citrate cycle
3-Carboxy-3-hydroxy-4-methylpentanoate
(R)-2-Ethylmalate
2.3.3.6
2.3.3.13
Leucine biosynthesis
Acetoacetyl-CoA
Homocitrate
Butanoate metabolism
2.3.1.9
2.3.3.14
Lysine biosynthesis
Synthesis and degradation of ketone bodies
Malonyl-CoA
4.1.3.-
6.4.1.2
Fatty acid biosynthesis
2-Propylmalate
Biosynthesis of 12-, 14- and 16-membered macrolides
Biosynthesis of enediyne antibiotics
Biosynthesis of type II polyketide backbone
00620 10/26/17
(c) Kanehisa Laboratories

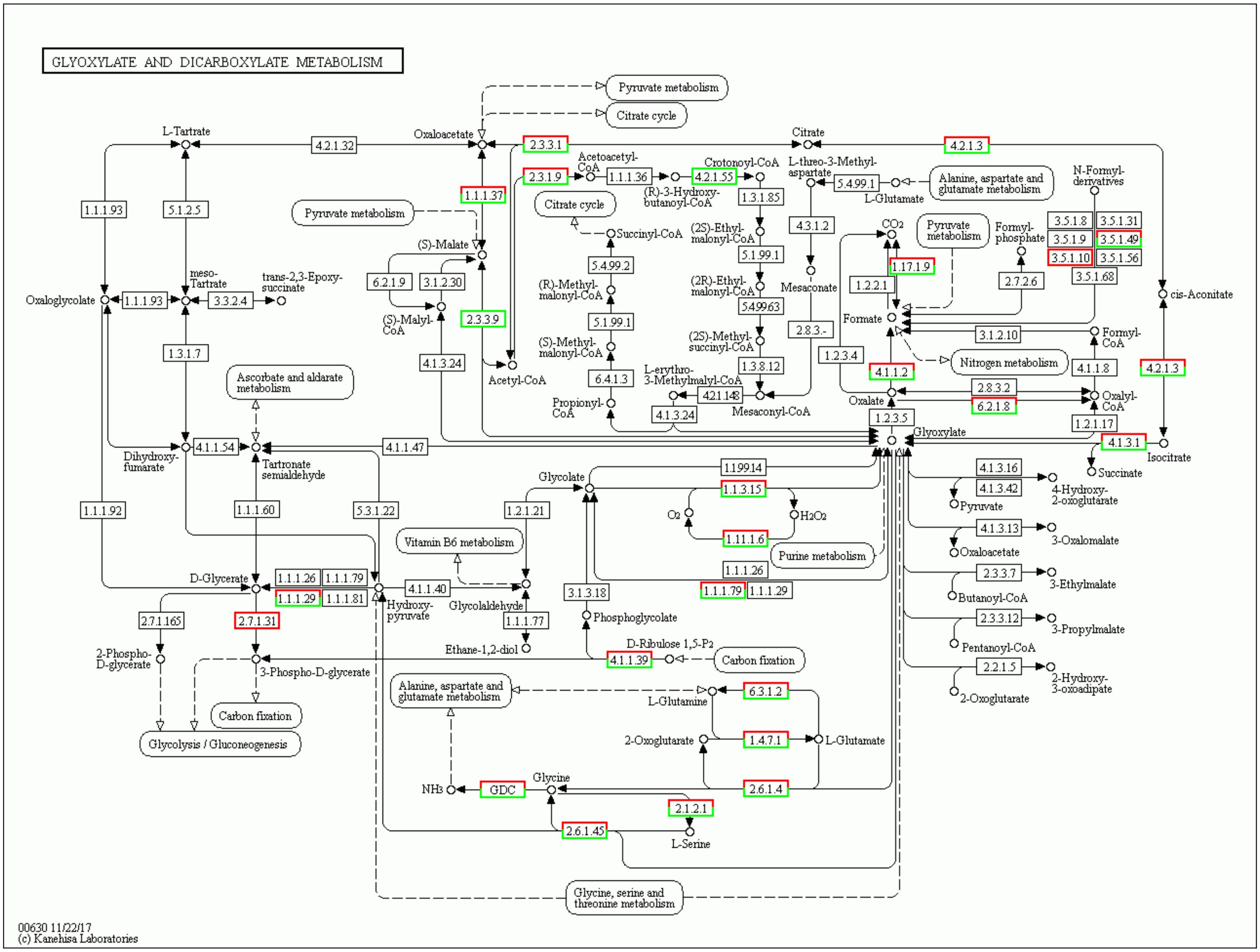
GLYOXYLATE AND DICARBOXYLATE METABOLISM
Pyruvate metabolism
Citrate cycle
L-Tartrate
4.2.1.32
Oxaloacetate
2.3.3.1
Citrate
4.2.1.3
Acetoacetyl-CoA
Crotonoyl-CoA
L-threo-3-Methyl-aspartate
2.3.1.9
1.1.1.36
4.2.1.55
5.4.99.1
Alanine, aspartate and glutamate metabolism
N-Formyl-derivatives
1.1.1.93
5.1.2.5
1.1.1.37
(R)-3-Hydroxy-butanoyl-CoA
1.3.1.85
L-Glutamate
Pyruvate metabolism
Citrate cycle
CO2
Pyruvate metabolism
Formyl-phosphate
3.5.1.8
3.5.1.31
3.5.1.9
3.5.1.49
3.5.1.10
3.5.1.56
3.5.1.68
(S)-Malate
Succinyl-CoA
(2S)-Ethyl-malonyl-CoA
4.3.1.2
1.17.1.9
5.4.99.2
5.1.99.1
meso-Tartrate
trans-2,3-Epoxy-succinate
6.2.1.9
3.1.2.30
(R)-Methyl-malonyl-CoA
(2R)-Ethyl-malonyl-CoA
Mesaconate
1.2.2.1
2.7.2.6
cis-Aconitate
Oxaloglycolate
1.1.1.93
3.3.2.4
2.3.3.9
5.4.99.63
Formate
(S)-Malyl-CoA
5.1.99.1
2.8.3.-
3.1.2.10
(S)-Methyl-malonyl-CoA
(2S)-Methyl-succinyl-CoA
Formyl-CoA
1.3.1.7
4.1.3.24
1.2.3.4
Nitrogen metabolism
Ascorbate and aldarate metabolism
Acetyl-CoA
6.4.1.3
L-erythro-3-Methylmalyl-CoA
1.3.8.12
4.1.1.2
4.1.1.8
4.2.1.3
4.2.1.148
2.8.3.2
Propionyl-CoA
Mesaconyl-CoA
Oxalate
Oxalyl-CoA
4.1.3.24
6.2.1.8
1.2.3.5
1.2.1.17
4.1.1.54
4.1.1.47
Glyoxylate
4.1.3.1
Dihydroxy-fumarate
Tartronate semialdehyde
Isocitrate
1.1.99.14
4.1.3.16
Succinate
Glycolate
1.1.3.15
4.1.3.42
4-Hydroxy-2-oxoglutarate
1.1.1.92
1.1.1.60
5.3.1.22
1.2.1.21
O2
H2O2
Pyruvate
1.11.1.6
4.1.3.13
3-Oxalomalate
Vitamin B6 metabolism
Oxaloacetate
Purine metabolism
1.1.1.26
D-Glycerate
1.1.1.26
1.1.1.79
2.3.3.7
3-Ethylmalate
4.1.1.40
3.1.3.18
1.1.1.79
1.1.1.29
1.1.1.29
1.1.1.81
Hydroxy-pyruvate
Glycolaldehyde
Butanoyl-CoA
2.7.1.165
2.7.1.31
1.1.1.77
Phosphoglycolate
2.3.3.12
3-Propylmalate
Pentanoyl-CoA
D-Ribulose 1,5-P2
2-Phospho-D-glycerate
Ethane-1,2-diol
4.1.1.39
Carbon fixation
3-Phospho-D-glycerate
2.2.1.5
2-Hydroxy-3-oxoadipate
Alanine, aspartate and glutamate metabolism
6.3.1.2
L-Glutamine
2-Oxoglutarate
Carbon fixation
Glycolysis / Gluconeogenesis
2-Oxoglutarate
1.4.7.1
L-Glutamate
Glycine
NH3
GDC
2.6.1.4
2.1.2.1
2.6.1.45
L-Serine
Glycine, serine and threonine metabolism
00630 11/22/17
(c) Kanehisa Laboratories

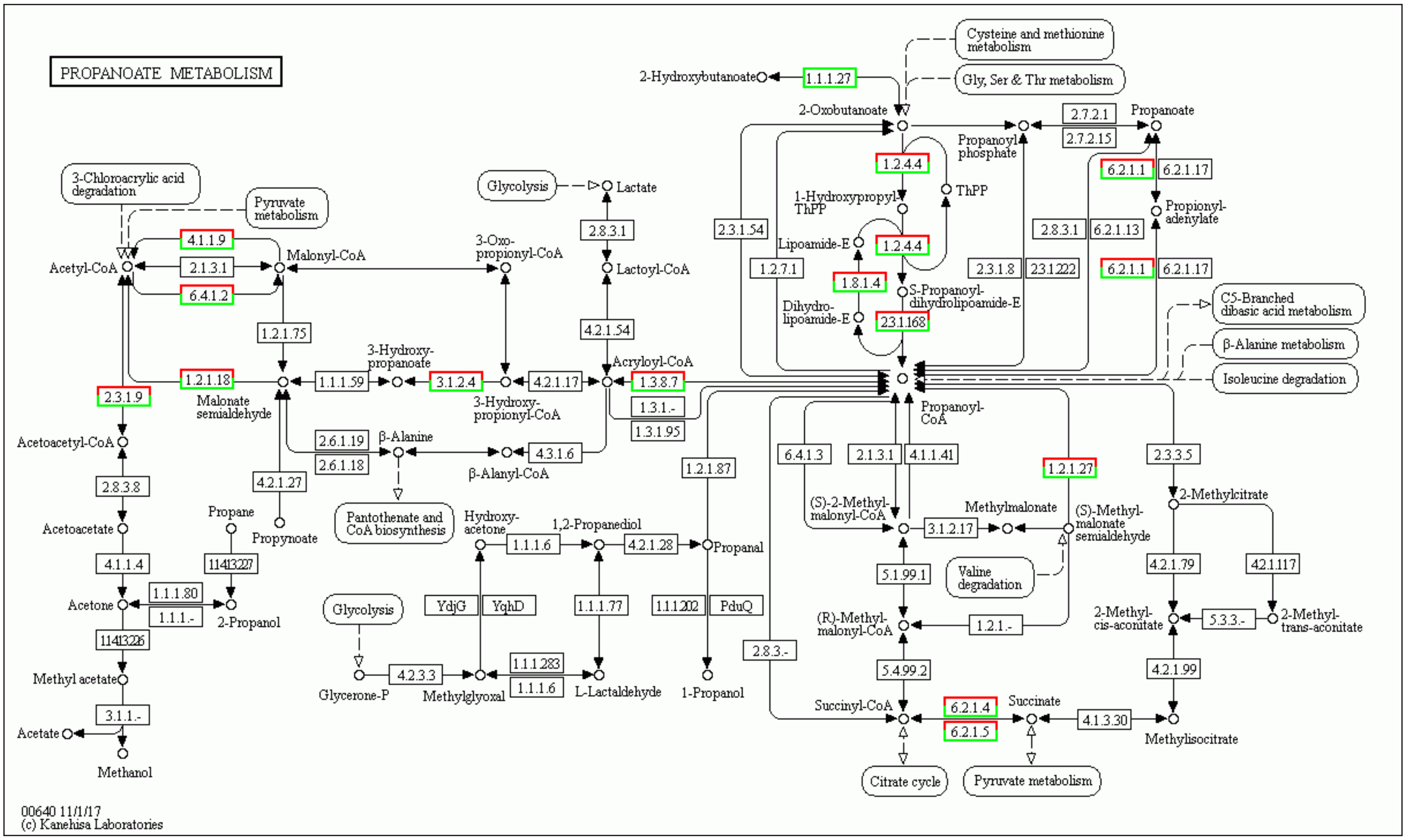
PROPANOATE METABOLISM
2-Hydroxybutanoate
1.1.1.27
Cysteine and methionine metabolism
Gly, Ser & Thr metabolism
2-Oxobutanoate
2.7.2.1
2.7.2.15
Propanoate
Propanoyl phosphate
1.2.4.4
6.2.1.1
6.2.1.17
1-Hydroxypropyl-ThPP
ThPP
Propionyl-adenylate
2.3.1.54
2.8.3.1
6.2.1.13
Lipoamide-E
1.2.4.4
1.2.7.1
2.3.1.8
2.3.1.222
6.2.1.1
6.2.1.17
1.8.1.4
S-Propanoyl-dihydrolipoamide-E
Dihydro-lipoamide-E
2.3.1.168
C5-Branched dibasic acid metabolism
β-Alanine metabolism
Isoleucine degradation
Propanoyl-CoA
3-Chloroacrylic acid degradation
Pyruvate metabolism
Glycolysis
Lactate
4.1.1.9
Malonyl-CoA
3-Oxo-propionyl-CoA
2.8.3.1
Acetyl-CoA
2.1.3.1
Lactoyl-CoA
6.4.1.2
1.2.1.75
4.2.1.54
3-Hydroxy-propanoate
Acryloyl-CoA
1.2.1.18
1.1.1.59
3.1.2.4
4.2.1.17
1.3.8.7
2.3.1.9
Malonate semialdehyde
3-Hydroxy-propionyl-CoA
1.3.1.-
1.3.1.95
Acetoacetyl-CoA
2.6.1.19
2.6.1.18
β-Alanine
4.3.1.6
β-Alanyl-CoA
6.4.1.3
2.1.3.1
4.1.1.41
1.2.1.27
2.3.3.5
1.2.1.87
2-Methylcitrate
2.8.3.8
4.2.1.27
Propane
Pantothenate and CoA biosynthesis
Hydroxy-acetone
1,2-Propanediol
(S)-2-Methyl-malonyl-CoA
Methylmalonate
(S)-Methyl-malonate semialdehyde
Acetoacetate
Propynoate
1.1.1.6
4.2.1.28
Propanal
3.1.2.17
4.1.1.4
1.14.13.227
Valine degradation
5.1.99.1
4.2.1.79
4.2.1.117
1.1.1.80
Glycolysis
YdjG
YqhD
1.1.1.77
1.1.1.202
PduQ
Acetone
1.1.1.-
2-Propanol
(R)-Methyl-malonyl-CoA
1.2.1.-
2-Methyl-cis-aconitate
5.3.3.-
2-Methyl-trans-aconitate
1.14.13.226
2.8.3.-
1.1.1.283
5.4.99.2
4.2.1.99
Methyl acetate
4.2.3.3
1.1.1.6
Glycerone-P
Methylglyoxal
L-Lactaldehyde
1-Propanol
Succinyl-CoA
6.2.1.4
Succinate
4.1.3.30
3.1.1.-
6.2.1.5
Methylisocitrate
Acetate
Methanol
Citrate cycle
Pyruvate metabolism
00640 11/1/17
(c) Kanehisa Laboratories

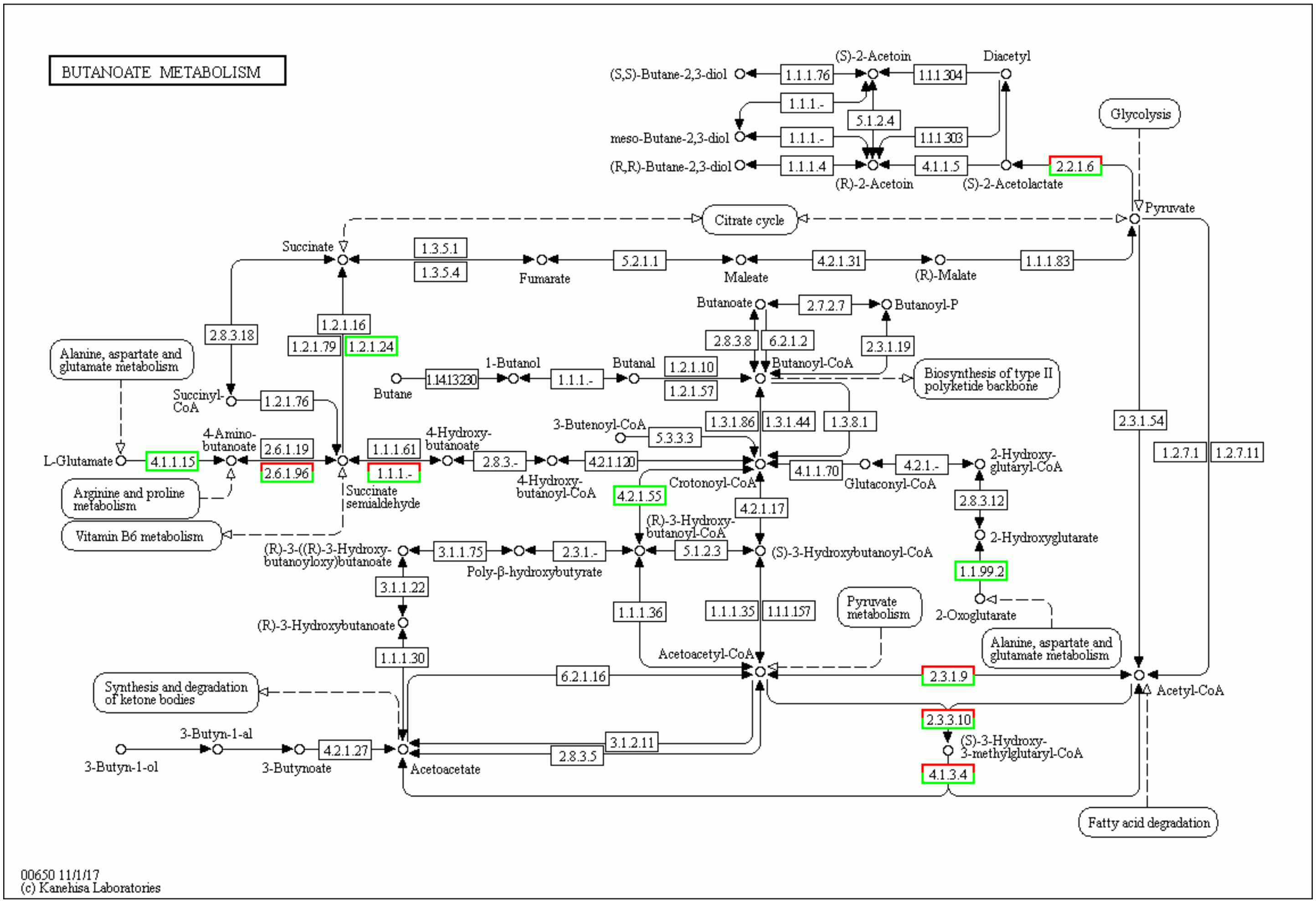
BUTANOATE METABOLISM
(S)-2-Acetoin
Diacetyl
(S,S)-Butane-2,3-diol
1.1.1.76
1.1.1.304
1.1.1.-
5.1.2.4
Glycolysis
meso-Butane-2,3-diol
1.1.1.-
1.1.1.303
(R,R)-Butane-2,3-diol
1.1.1.4
4.1.1.5
2.2.1.6
(R)-2-Acetoin
(S)-2-Acetolactate
Pyruvate
Citrate cycle
Succinate
1.3.5.1
1.3.5.4
5.2.1.1
4.2.1.31
1.1.1.83
Fumarate
Maleate
(R)-Malate
Butanoate
2.7.2.7
Butanoyl-P
2.8.3.18
1.2.1.16
2.8.3.8
6.2.1.2
2.3.1.19
1.2.1.79
1.2.1.24
Alanine, aspartate and glutamate metabolism
1-Butanol
Butanal
1.2.1.10
Butanoyl-CoA
Biosynthesis of type II polyketide backbone
Butane
1.14.13.230
1.1.1.-
1.2.1.57
Succinyl-CoA
1.2.1.76
3-Butenoyl-CoA
1.3.1.86
1.3.1.44
1.3.8.1
2.3.1.54
4-Amino-butanoate
4-Hydroxy-butanoate
5.3.3.3
L-Glutamate
4.1.1.15
2.6.1.19
1.1.1.61
2.8.3.-
4.2.1.120
4.1.1.70
4.2.1.-
2-Hydroxy-glutaryl-CoA
1.2.7.1
1.2.7.11
2.6.1.96
1.1.1.-
4-Hydroxy-butanoyl-CoA
Crotonoyl-CoA
Glutaconyl-CoA
Arginine and proline metabolism
Succinate semialdehyde
4.2.1.55
4.2.1.17
2.8.3.12
(R)-3-Hydroxy-butanoyl-CoA
Vitamin B6 metabolism
2-Hydroxyglutarate
(R)-3-((R)-3-Hydroxy-butanoyloxy)butanoate
3.1.1.75
2.3.1.-
5.1.2.3
(S)-3-Hydroxybutanoyl-CoA
Poly-β-hydroxybutyrate
1.1.99.2
3.1.1.22
1.1.1.36
1.1.1.35
1.1.1.157
Pyruvate metabolism
2-Oxoglutarate
(R)-3-Hydroxybutanoate
Alanine, aspartate and glutamate metabolism
1.1.1.30
Acetoacetyl-CoA
6.2.1.16
2.3.1.9
Acetyl-CoA
Synthesis and degradation of ketone bodies
2.3.3.10
(S)-3-Hydroxy-3-methylglutaryl-CoA
3-Butyn-1-al
3.1.2.11
3-Butyn-1-ol
4.2.1.27
2.8.3.5
3-Butynoate
Acetoacetate
4.1.3.4
Fatty acid degradation
00650 11/1/17
(c) Kanehisa Laboratories

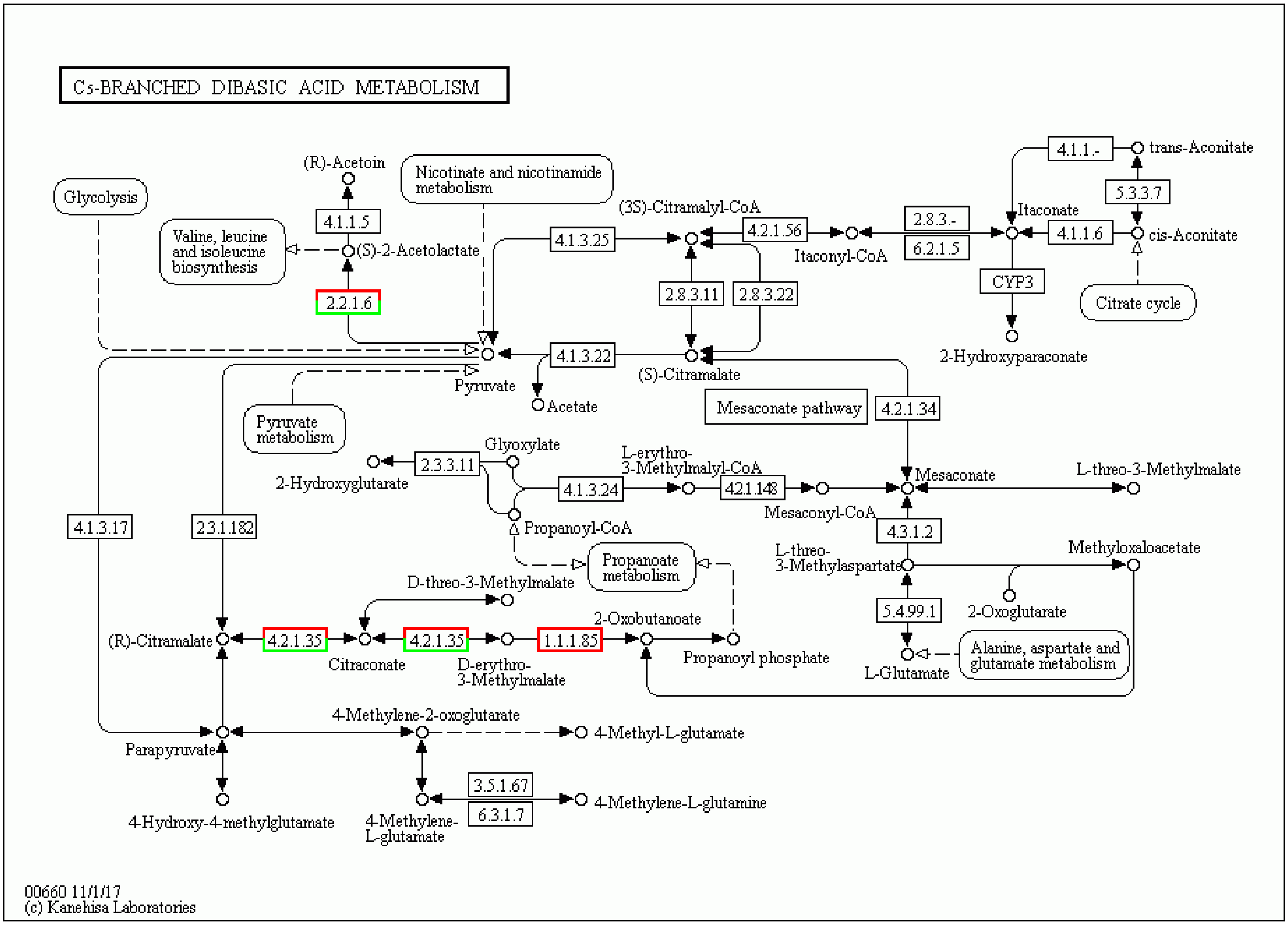
C5-BRANCHED DIBASIC ACID METABOLISM
Glycolysis
(R)-Acetoin
4.1.1.5
Valine, leucine and isoleucine biosynthesis
(S)-2-Acetolactate
2.2.1.6
Nicotinate and nicotinamide metabolism
4.1.3.25
(3S)-Citramalyl-CoA
4.2.1.56
Itaconyl-CoA
2.8.3.-
6.2.1.5
4.1.1.-
trans-Aconitate
5.3.3.7
Itaconate
4.1.1.6
cis-Aconitate
CYP3
Citrate cycle
2-Hydroxyparaconate
2.8.3.11
2.8.3.22
4.1.3.22
Pyruvate
Acetate
(S)-Citramalate
Mesaconate pathway
4.2.1.34
Pyruvate metabolism
2.3.3.11
Glyoxylate
2-Hydroxyglutarate
L-erythro-3-Methylmalyl-CoA
4.1.3.24
4.2.1.148
Mesaconyl-CoA
Mesaconate
L-threo-3-Methylmalate
Propanoyl-CoA
4.1.3.17
2.3.1.182
Propanoate metabolism
4.3.1.2
L-threo-3-Methylaspartate
Methyloxaloacetate
D-threo-3-Methylmalate
5.4.99.1
2-Oxoglutarate
2-Oxobutanoate
(R)-Citramalate
4.2.1.35
Citraconate
4.2.1.35
D-erythro-3-Methylmalate
1.1.1.85
Propanoyl phosphate
L-Glutamate
Alanine, aspartate and glutamate metabolism
4-Methylene-2-oxoglutarate
Parapyruvate
4-Methyl-L-glutamate
3.5.1.67
6.3.1.7
4-Hydroxy-4-methylglutamate
4-Methylene-L-glutamate
4-Methylene-L-glutamine
00660 11/1/17
(c) Kanehisa Laboratories

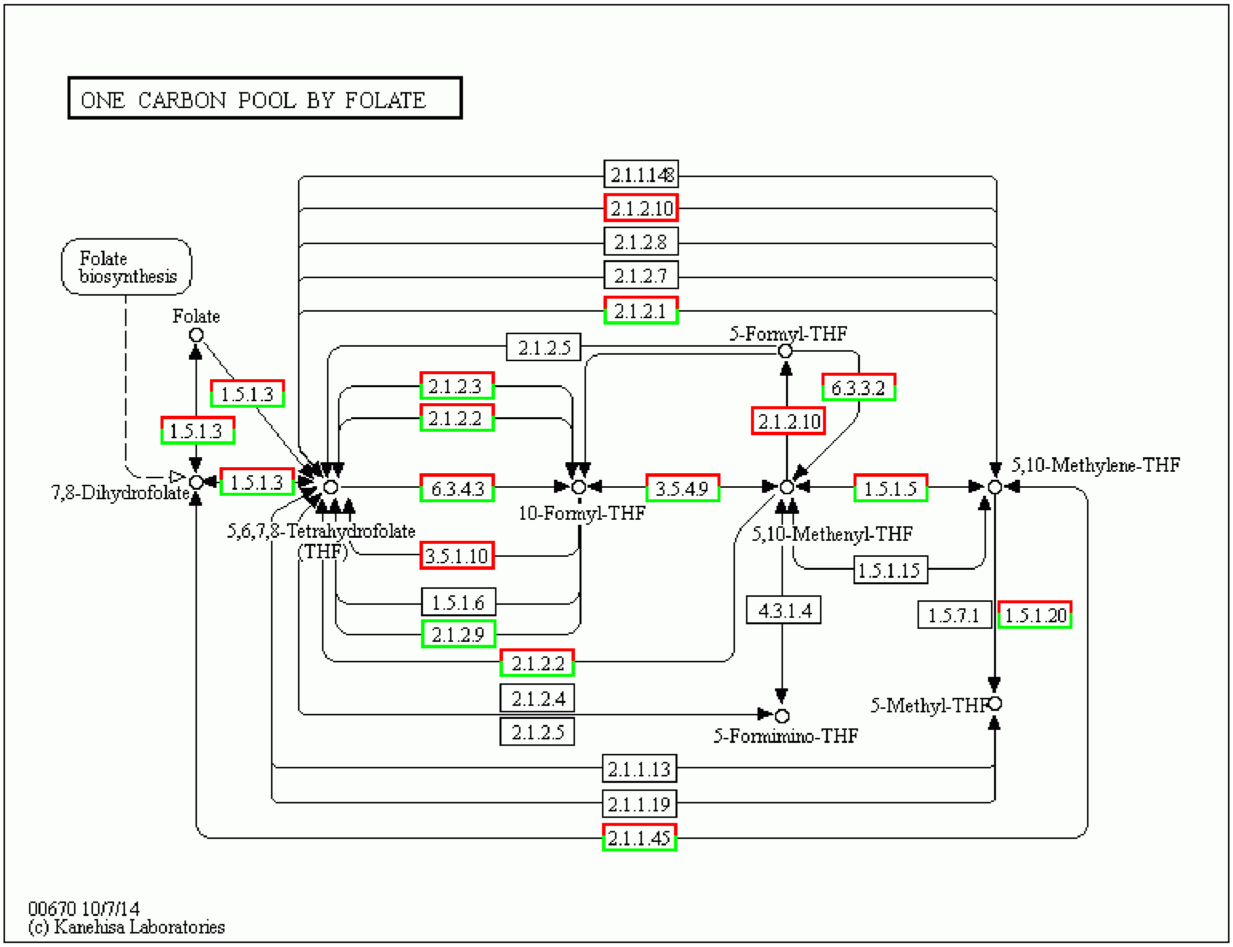
ONE CARBON POOL BY FOLATE
2.1.1.148
2.1.2.10
2.1.2.8
2.1.2.7
2.1.2.1
Folate biosynthesis
Folate
2.1.2.5
5-Formyl-THF
1.5.1.3
2.1.2.3
2.1.2.2
6.3.3.2
1.5.1.3
2.1.2.10
5,10-Methylene-THF
7,8-Dihydrofolate
1.5.1.3
6.3.4.3
3.5.4.9
1.5.1.5
10-Formyl-THF
5,6,7,8-Tetrahydrofolate (THF)
5,10-Methenyl-THF
3.5.1.10
1.5.1.15
1.5.1.6
4.3.1.4
1.5.7.1
1.5.1.20
2.1.2.9
2.1.2.2
2.1.2.4
5-Methyl-THF
2.1.2.5
5-Formimino-THF
2.1.1.13
2.1.1.19
2.1.1.45
00670 10/7/14
(c) Kanehisa Laboratories

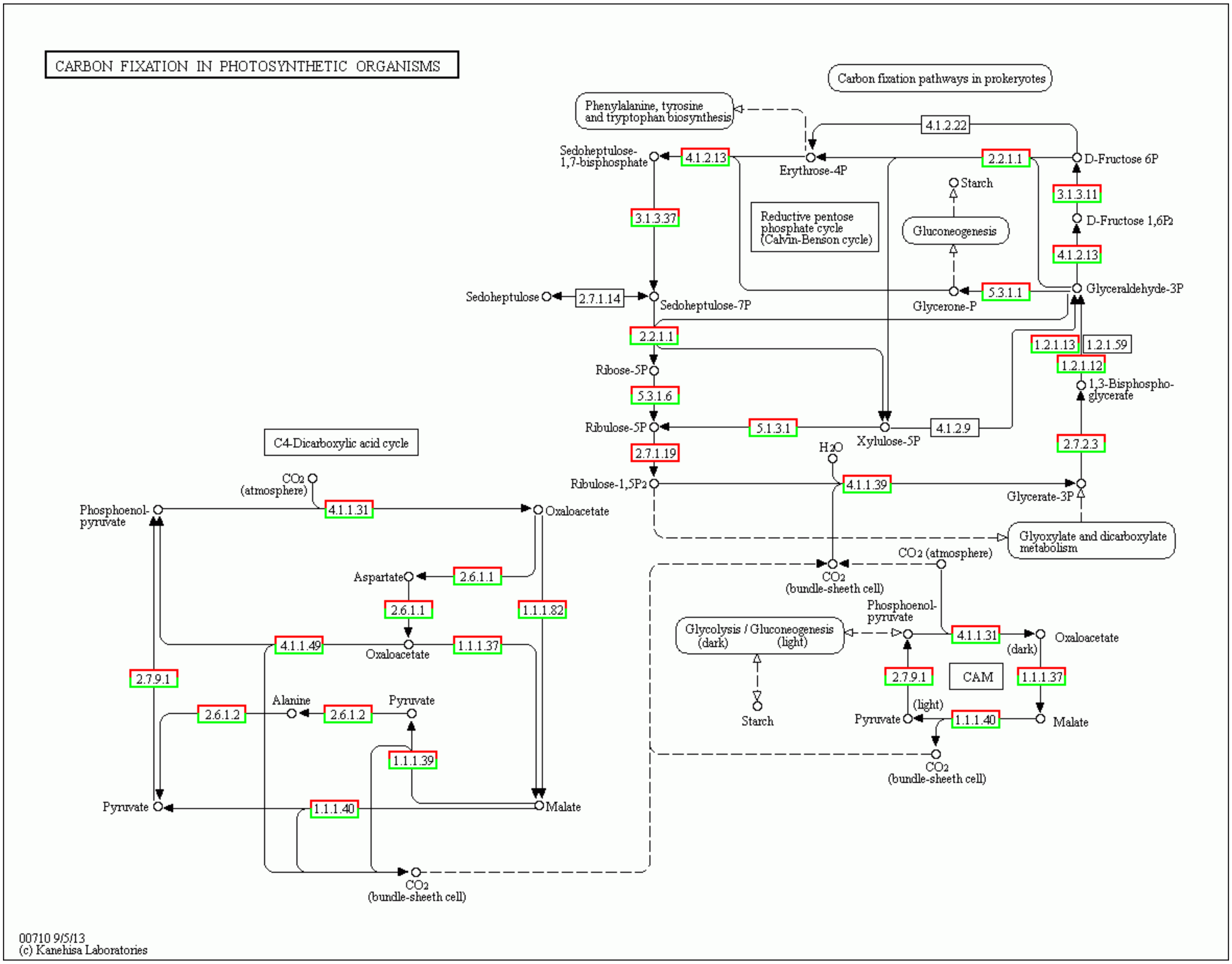
CARBON FIXATION IN PHOTOSYNTHETIC ORGANISMS
Carbon fixation pathways in prokaryotes
Phenylalanine, tyrosine and tryptophan biosynthesis
4.1.2.22
Sedoheptulose-1,7-bisphosphate
4.1.2.13
Erythrose-4P
2.2.1.1
D-Fructose 6P
3.1.3.37
Starch
3.1.3.11
Reductive pentose phosphate cycle (Calvin-Benson cycle)
Gluconeogenesis
D-Fructose 1,6P2
4.1.2.13
Sedoheptulose
2.7.1.14
Sedoheptulose-7P
5.3.1.1
Glycerone-P
Glyceraldehyde-3P
2.2.1.1
1.2.1.13
1.2.1.59
1.2.1.12
Ribose-5P
1,3-Bisphospho-glycerate
5.3.1.6
Ribulose-5P
5.1.3.1
Xylulose-5P
4.1.2.9
C4-Dicarboxylic acid cycle
2.7.1.19
2.7.2.3
H2O
Ribulose-1,5P2
4.1.39
Glycerate-3P
CO2 (atmosphere)
Phosphoenol-pyruvate
4.1.1.31
Oxaloacetate
Glyoxylate and dicarboxylate metabolism
CO2 (atmosphere)
CO2 (bundle-sheath cell)
Aspartate
2.6.1.1
Phosphoenol-pyruvate
2.6.1.1
1.1.1.82
Glycolysis / Gluconeogenesis (dark) (light)
4.1.1.31
Oxaloacetate
4.1.1.49
Oxaloacetate
1.1.1.37
(dark)
2.7.9.1
2.7.9.1
CAM
1.1.1.37
Alanine
Pyruvate
(light)
2.6.1.2
2.6.1.2
Starch
Pyruvate
1.1.1.40
Malate
1.1.1.39
CO2 (bundle-sheath cell)
Pyruvate
1.1.1.40
Malate
CO2 (bundle-sheath cell)
00710 9/5/13
(c) Kanehisa Laboratories

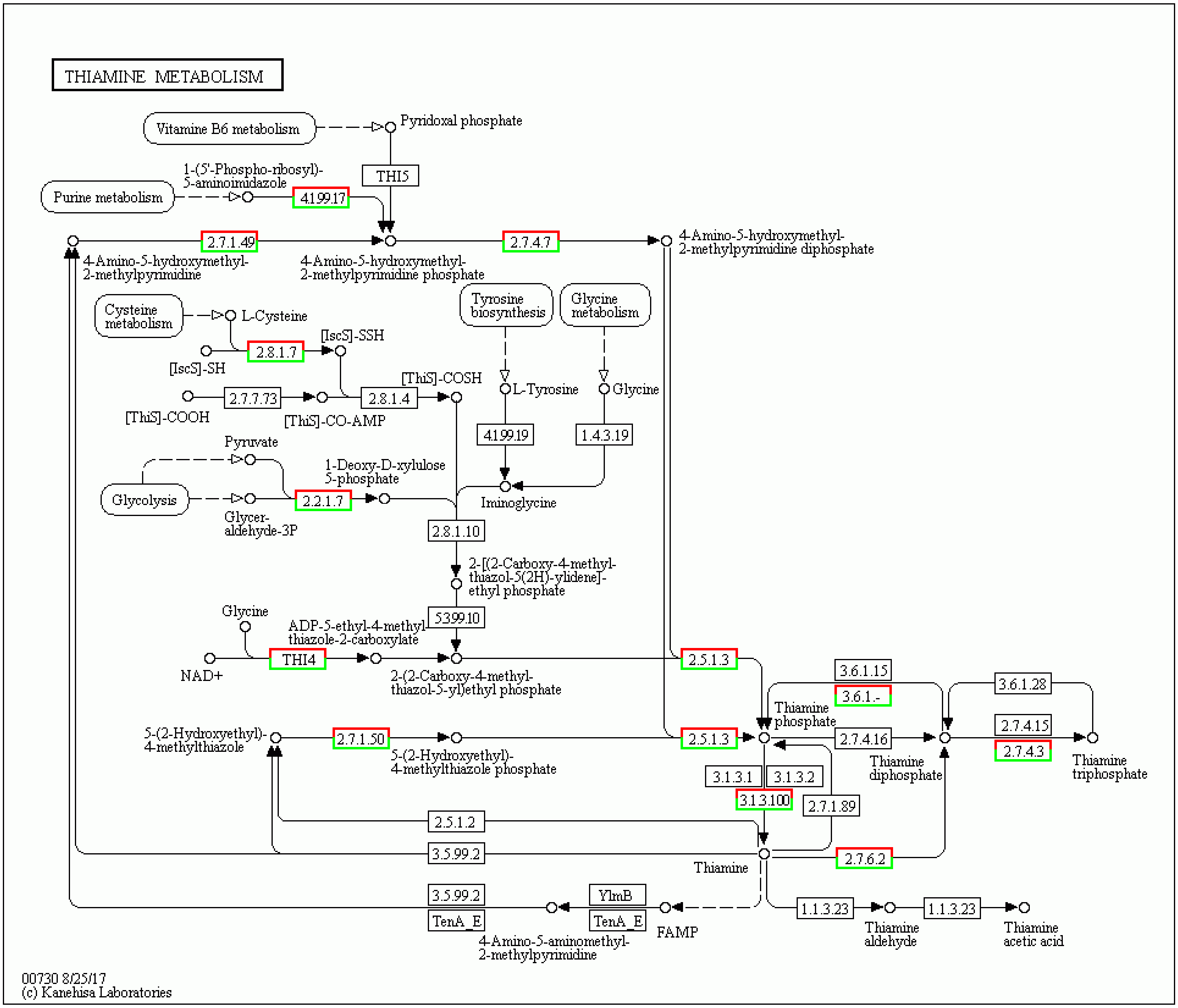
THIAMINE METABOLISM
Vitamine B6 metabolism
Pyridoxal phosphate
THI5
1-(5'-Phospho-ribosyl)-
5-aminoimidazole
Purine metabolism
4.1.99.17
2.7.1.49
2.7.4.7
4-Amino-5-hydroxymethyl-
2-methylpyrimidine
4-Amino-5-hydroxymethyl-
2-methylpyrimidine phosphate
4-Amino-5-hydroxymethyl-
2-methylpyrimidine diphosphate
Cysteine
metabolism
L-Cysteine
Tyrosine
biosynthesis
Glycine
metabolism
2.8.1.7
[IscS]-SSH
[IscS]-SH
[ThiS]-COSH
2.7.7.73
2.8.1.4
[ThiS]-COOH
[ThiS]-CO-AMP
L-Tyrosine
Glycine
4.1.99.19
1.4.3.19
Pyruvate
1-Deoxy-D-xylulose
5-phosphate
Glycolysis
2.2.1.7
Glycer-
aldehyde-3P
Iminoglycine
2.8.1.10
2-[(2-Carboxy-4-methyl-
thiazol-5(2H)-ylidene]-
ethyl phosphate
Glycine
5.3.99.10
ADP-5-ethyl-4-methyl
thiazole-2-carboxylate
NAD+
THI4
2-(2-Carboxy-4-methyl-
thiazol-5-yl)ethyl phosphate
2.5.1.3
3.6.1.15
3.6.1.-
3.6.1.28
Thiamine
phosphate
2.7.4.15
5-(2-Hydroxyethyl)-
4-methylthiazole
2.7.1.50
2.5.1.3
2.7.4.16
2.7.4.3
5-(2-Hydroxyethyl)-
4-methylthiazole phosphate
Thiamine
diphosphate
Thiamine
triphosphate
3.1.3.1
3.1.3.2
3.1.3.100
2.7.1.89
2.5.1.2
3.5.99.2
2.7.6.2
Thiamine
3.5.99.2
YlmB
TenA_E
TenA_E
1.1.3.23
1.1.3.23
4-Amino-5-aminomethyl-
2-methylpyrimidine
FAMP
Thiamine
aldehyde
Thiamine
acetic acid
00730 8/25/17
(c) Kanehisa Laboratories

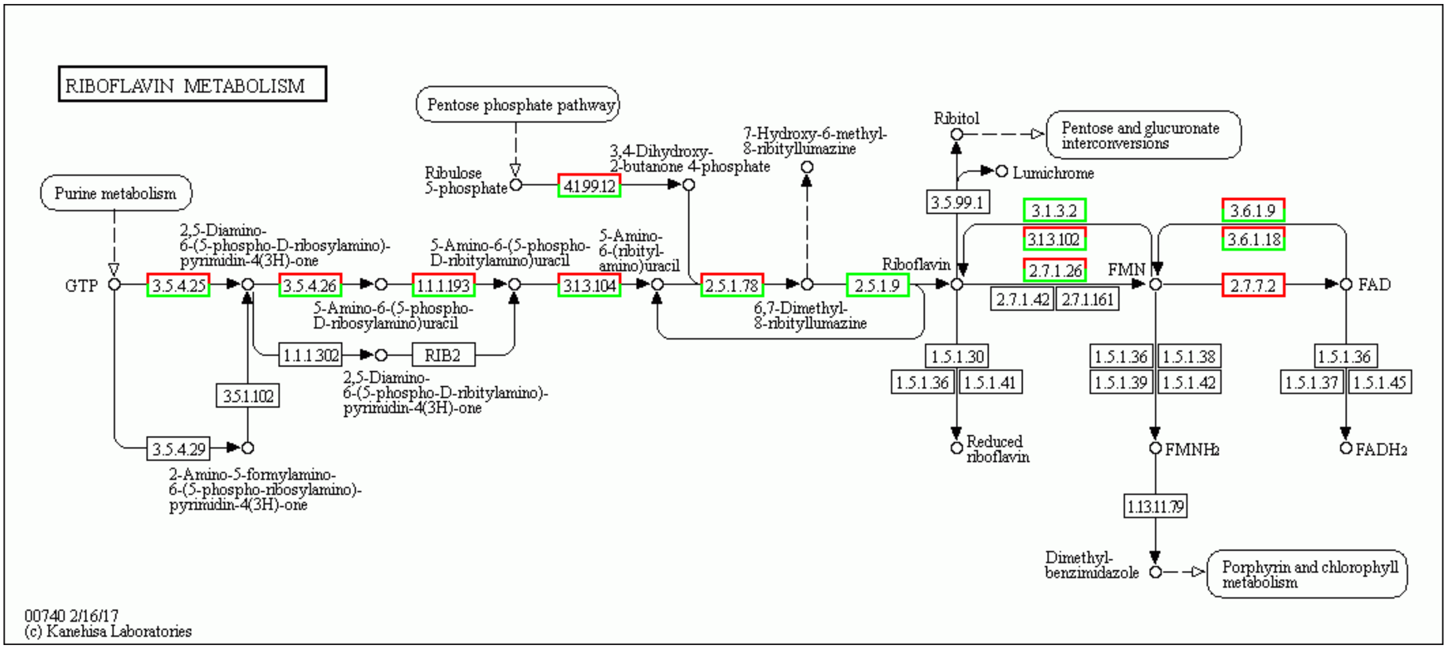
RIBOFLAVIN METABOLISM
Pentose phosphate pathway
Ribulose
5-phosphate
4.1.99.12
3,4-Dihydroxy-
2-butanone 4-phosphate
7-Hydroxy-6-methyl-
8-ribityllumazine
Ribitol
Pentose and glucuronate
interconversions
Lumichrome
Purine metabolism
2,5-Diamino-
6-(5-phospho-D-ribosylamino)-
pyrimidin-4(3H)-one
5-Amino-6-(5-phospho-
D-ribitylamino)uracil
5-Amino-
6-(ribityl-
amino)uracil
3.5.99.1
3.1.3.2
3.6.1.9
3.1.3.102
3.6.1.18
Riboflavin
2.7.1.26
FMN
GTP
3.5.4.25
3.5.4.26
1.1.1.193
3.1.3.104
2.5.1.78
2.5.1.9
2.7.7.2
FAD
5-Amino-6-(5-phospho-
D-ribosylamino)uracil
6,7-Dimethyl-
8-ribityllumazine
2.7.1.42
2.7.1.161
1.1.1.302
RIB2
2,5-Diamino-
6-(5-phospho-D-ribitylamino)-
pyrimidin-4(3H)-one
3.5.1.102
1.5.1.30
1.5.1.36
1.5.1.41
1.5.1.36
1.5.1.38
1.5.1.39
1.5.1.42
1.5.1.36
1.5.1.37
1.5.1.45
3.5.4.29
2-Amino-5-formylamino-
6-(5-phospho-ribosylamino)-
pyrimidin-4(3H)-one
Reduced
riboflavin
FMNH2
FADH2
1.13.11.79
Dimethyl-
benzimidazole
Porphyrin and chlorophyll
metabolism
00740 2/16/17
(c) Kanehisa Laboratories

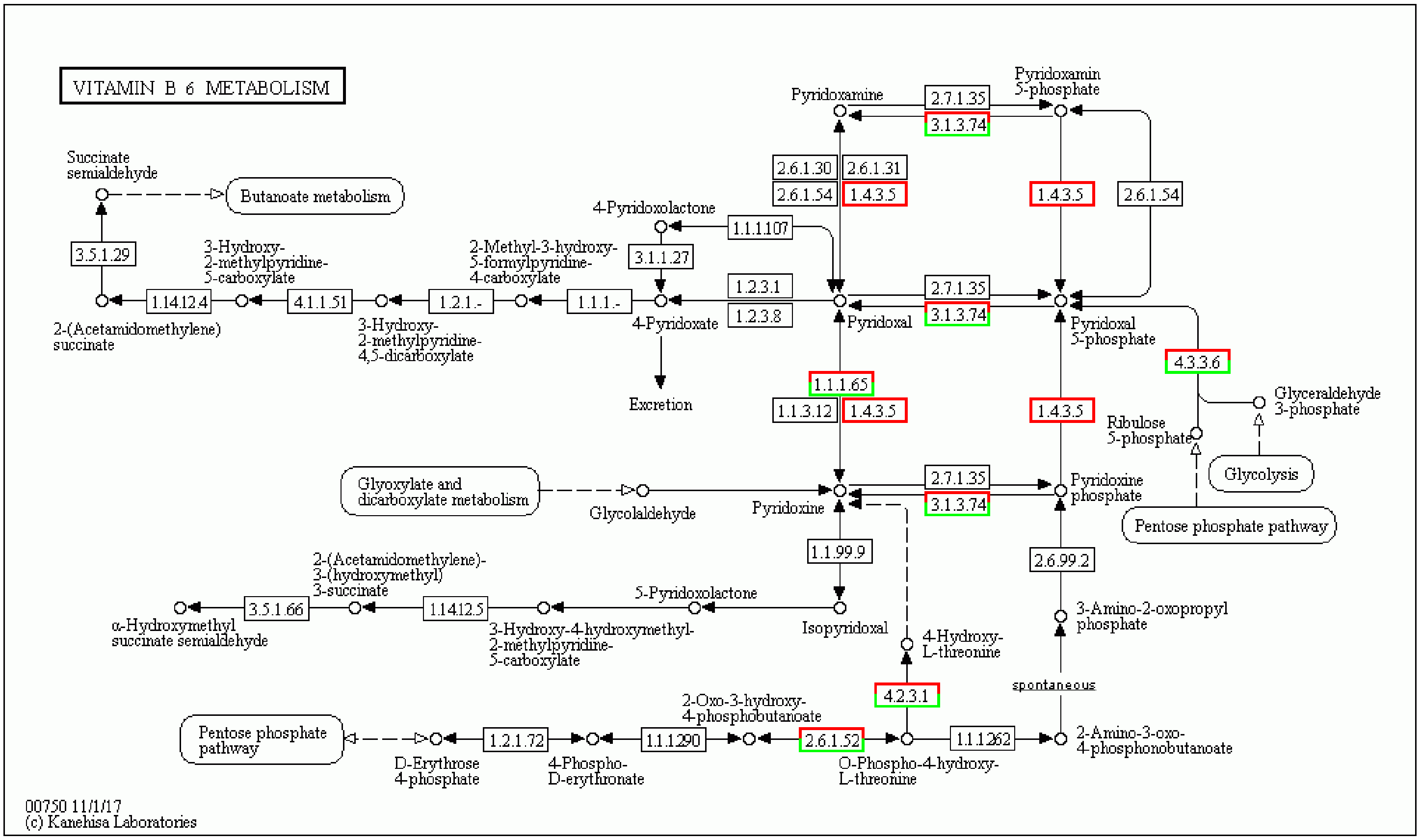
VITAMIN B 6 METABOLISM
Pyridoxamine
2.7.1.35
3.1.3.74
Pyridoxamin
5-phosphate
2.6.1.30
2.6.1.31
2.6.1.54
1.4.3.5
1.4.3.5
2.6.1.54
Succinate
semialdehyde
Butanoate metabolism
4-Pyridoxolactone
1.1.1.107
3.5.1.29
3-Hydroxy-
2-methylpyridine-
5-carboxylate
2-Methyl-3-hydroxy-
5-formylpyridine-
4-carboxylate
3.1.1.27
1.2.3.1
2.7.1.35
1.14.12.4
4.1.1.51
1.2.1.-
1.1.1.-
1.2.3.8
3.1.3.74
2-(Acetamidomethylene)
succinate
3-Hydroxy-
2-methylpyridine-
4,5-dicarboxylate
4-Pyridoxate
Pyridoxal
Pyridoxal
5-phosphate
4.3.3.6
Excretion
1.1.1.65
1.1.3.12
1.4.3.5
1.4.3.5
Glyceraldehyde
3-phosphate
Ribulose
5-phosphate
Glycolysis
Glyoxylate and
dicarboxylate metabolism
Glycolaldehyde
Pyridoxine
2.7.1.35
3.1.3.74
Pyridoxine
phosphate
Pentose phosphate pathway
2-(Acetamidomethylene)-
3-(hydroxymethyl)
3-succinate
1.1.99.9
2.6.99.2
3.5.1.66
1.14.12.5
5-Pyridoxolactone
α-Hydroxymethyl
succinate semialdehyde
3-Hydroxy-4-hydroxymethyl-
2-methylpyridine-
5-carboxylate
Isopyridoxal
4-Hydroxy-
L-threonine
3-Amino-2-oxopropyl
phosphate
spontaneous
4.2.3.1
2-Oxo-3-hydroxy-
4-phosphobutanoate
Pentose phosphate
pathway
1.2.1.72
1.1.1.290
2.6.1.52
1.1.1.262
2-Amino-3-oxo-
4-phosphonobutanoate
D-Erythrose
4-phosphate
4-Phospho-
D-erythronate
O-Phospho-4-hydroxy-
L-threonine
00750 11/1/17
(c) Kanehisa Laboratories

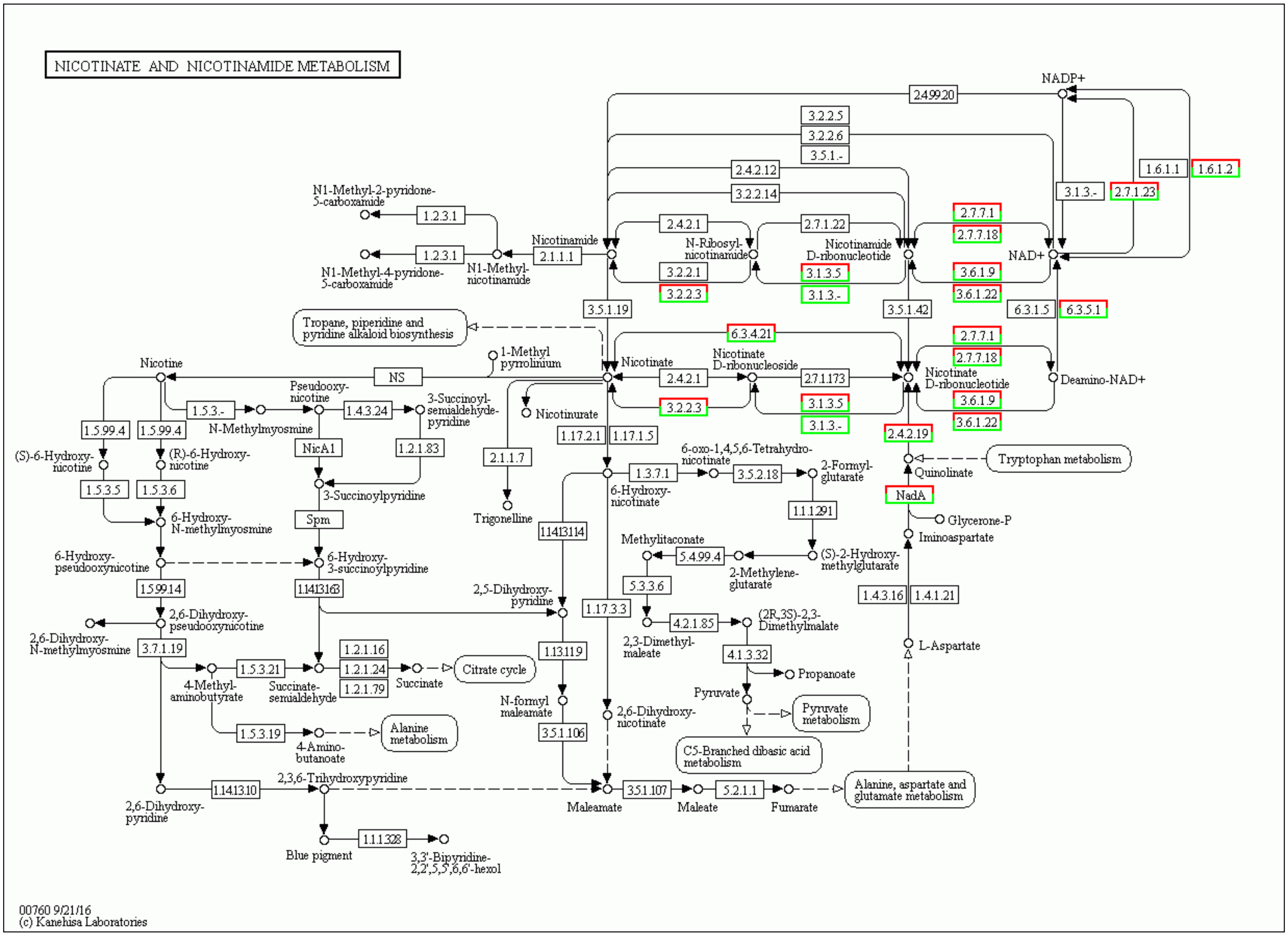
NICOTINATE AND NICOTINAMIDE METABOLISM
NADP+
2.4.99.20
3.2.2.5
3.2.2.6
3.5.1.-
2.4.2.12
1.6.1.1
1.6.1.2
N1-Methyl-2-pyridone-
5-carboxamide
3.2.2.14
3.1.3.-
2.7.1.23
1.2.3.1
2.7.7.1
Nicotinamide
2.4.2.1
2.7.1.22
2.7.7.18
1.2.3.1
2.1.1.1
N-Ribosyl-
nicotinamide
Nicotinamide
D-ribonucleotide
NAD+
N1-Methyl-4-pyridone-
5-carboxamide
N1-Methyl-
nicotinamide
3.2.2.1
3.1.3.5
3.6.1.9
3.2.2.3
3.1.3.-
3.6.1.22
3.5.1.19
3.5.1.42
6.3.1.5
6.3.5.1
Tropane, piperidine and
pyridine alkaloid biosynthesis
6.3.4.21
2.7.7.1
1-Methyl
pyrrolinium
Nicotinate
Nicotinate
D-ribonucleoside
2.7.7.18
Nicotine
NS
2.4.2.1
2.7.1.173
Nicotinate
D-ribonucleotide
Deamino-NAD+
Pseudooxy-
nicotine
3-Succinoyl-
semialdehyde-
pyridine
3.2.2.3
3.1.3.5
3.6.1.9
1.5.3.-
1.4.3.24
Nicotinurate
3.1.3.-
3.6.1.22
1.5.99.4
1.5.99.4
N-Methylmyosmine
1.17.2.1
1.17.1.5
2.4.2.19
(S)-6-Hydroxy-
nicotine
(R)-6-Hydroxy-
nicotine
NicA1
1.2.1.83
6-oxo-1,4,5,6-Tetrahydro-
nicotinate
Tryptophan metabolism
2.1.1.7
2-Formyl-
glutarate
Quinolinate
1.3.7.1
3.5.2.18
1.5.3.5
1.5.3.6
3-Succinoylpyridine
6-Hydroxy-
nicotinate
NadA
Trigonelline
1.1.1.291
Glycerone-P
6-Hydroxy-
N-methylmyosmine
Spm
1.14.13.114
Iminoaspartate
Methylitaconate
6-Hydroxy-
pseudooxynicotine
6-Hydroxy-
3-succinoylpyridine
5.4.99.4
(S)-2-Hydroxy-
methylglutarate
2-Methylene-
glutarate
1.5.99.14
1.14.13.163
5.3.3.6
2,5-Dihydroxy-
pyridine
1.4.3.16
1.4.1.21
1.17.3.3
2,6-Dihydroxy-
pseudooxynicotine
4.2.1.85
(2R,3S)-2,3-
Dimethylmalate
2,6-Dihydroxy-
N-methylmyosmine
3.7.1.19
1.2.1.16
2,3-Dimethyl-
maleate
1.13.11.9
4.1.3.32
L-Aspartate
1.5.3.21
1.2.1.24
Citrate cycle
Propanoate
4-Methyl-
aminobutyrate
Succinate-
semialdehyde
1.2.1.79
Succinate
Pyruvate
N-formyl
maleamate
2,6-Dihydroxy-
nicotinate
Pyruvate
metabolism
1.5.3.19
Alanine
metabolism
3.5.1.106
4-Amino-
butanoate
C5-Branched dibasic acid
metabolism
2,3,6-Trihydroxypyridine
Alanine, aspartate and
glutamate metabolism
1.14.13.10
3.5.1.107
5.2.1.1
2,6-Dihydroxy-
pyridine
Maleamate
Maleate
Fumarate
1.1.1.328
Blue pigment
3,3'-Bipyridine-
2,2',5,5',6,6'-hexol
00760 9/21/16
(c) Kanehisa Laboratories

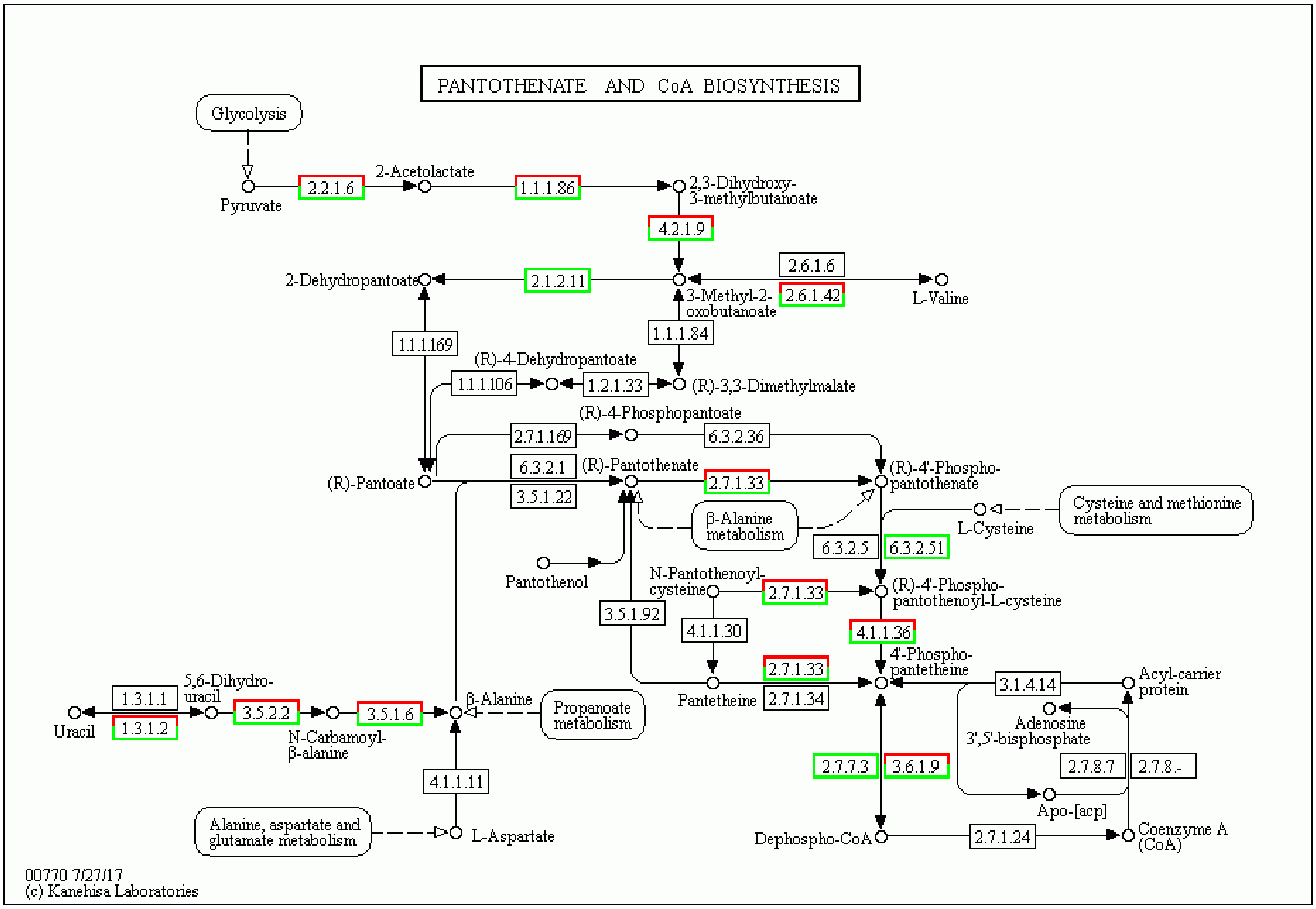
PANTOTHENATE AND CoA BIOSYNTHESIS
Glycolysis
Pyruvate
2.2.1.6
2-Acetolactate
1.1.1.86
2,3-Dihydroxy-3-methylbutanoate
4.2.1.9
2-Dehydropantoate
2.1.2.11
2.6.1.6
2.6.1.42
3-Methyl-2-oxobutanoate
L-Valine
1.1.1.169
1.1.1.84
(R)-4-Dehydropantoate
1.1.1.106
1.2.1.33
(R)-3,3-Dimethylmalate
(R)-4-Phosphopantoate
2.7.1.169
6.3.2.36
(R)-Pantoate
6.3.2.1
(R)-Pantothenate
2.7.1.33
(R)-4'-Phospho-pantothenate
3.5.1.22
β-Alanine metabolism
Cysteine and methionine metabolism
L-Cysteine
6.3.2.5
6.3.2.51
Pantothenol
N-Pantothenoyl-cysteine
2.7.1.33
(R)-4'-Phospho-pantothenoyl-L-cysteine
3.5.1.92
4.1.1.30
4.1.1.36
4'-Phospho-pantetheine
2.7.1.33
Pantetheine
2.7.1.34
3.1.4.14
Acyl-carrier protein
1.3.1.1
5,6-Dihydro-uracil
Uracil
1.3.1.2
3.5.2.2
N-Carbamoyl-β-alanine
3.5.1.6
β-Alanine
Propanoate metabolism
Adenosine 3',5'-bisphosphate
2.7.7.3
3.6.1.9
2.7.8.7
2.7.8.-
Apo-[acp]
4.1.1.11
Alanine, aspartate and glutamate metabolism
L-Aspartate
Dephospho-CoA
2.7.1.24
Coenzyme A (CoA)
00770 7/27/17
(c) Kanehisa Laboratories

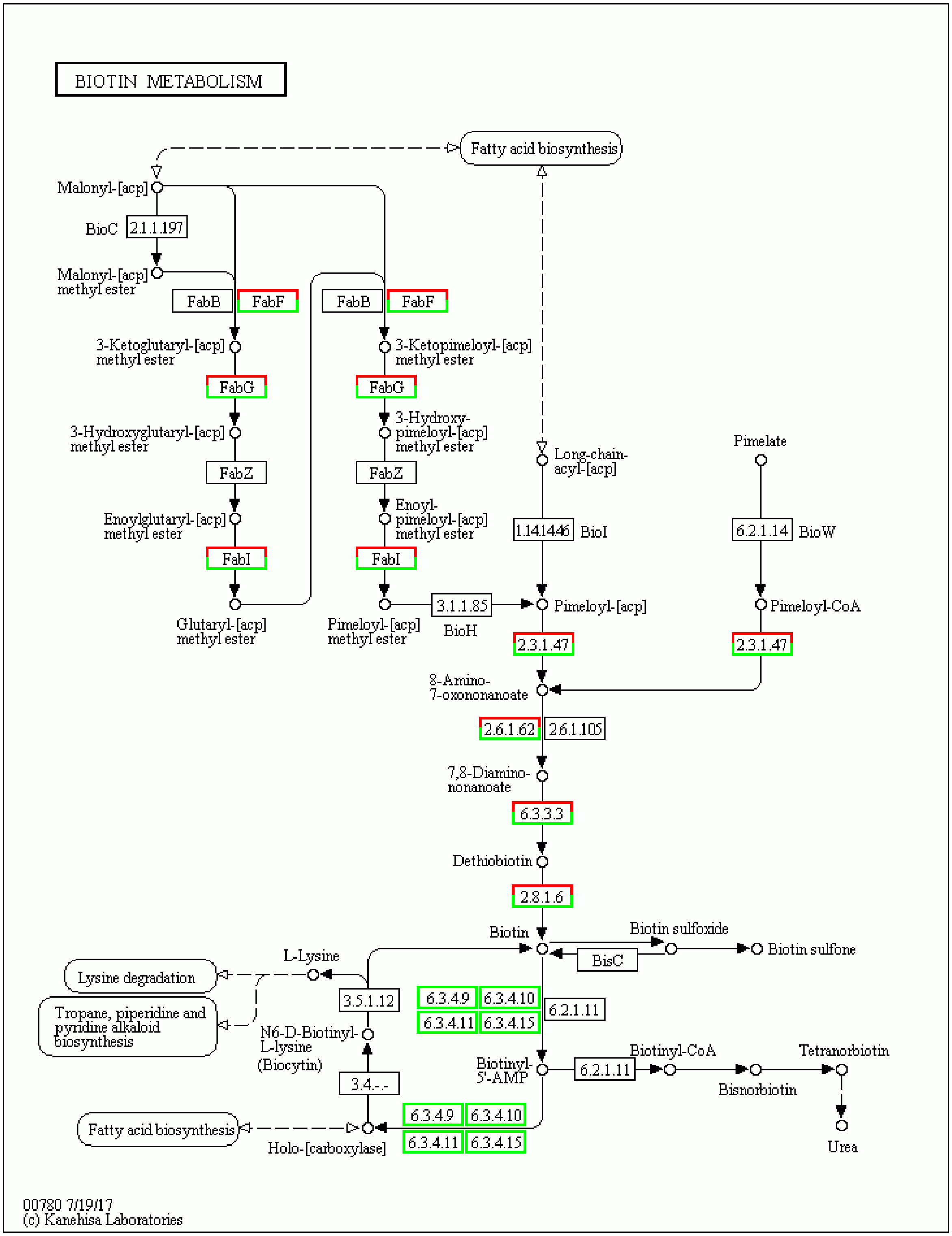
BIOTIN METABOLISM
Fatty acid biosynthesis
Malonyl-[acp]
BioC
2.1.1.197
Malonyl-[acp] methyl ester
FabB
FabF
FabB
FabF
3-Ketoglutaryl-[acp] methyl ester
3-Ketopimeloyl-[acp] methyl ester
FabG
FabG
3-Hydroxyglutaryl-[acp] methyl ester
3-Hydroxy-pimeloyl-[acp] methyl ester
FabZ
FabZ
Enoylglutaryl-[acp] methyl ester
Enoyl-pimeloyl-[acp] methyl ester
FabI
FabI
Glutaryl-[acp] methyl ester
Pimeloyl-[acp] methyl ester
3.1.1.85
BioH
Long-chain-acyl-[acp]
1.14.14.46
BioI
Pimelate
6.2.1.14
BioW
Pimeloyl-[acp]
Pimeloyl-CoA
2.3.1.47
2.3.1.47
8-Amino-7-oxononanoate
2.6.1.62
2.6.1.105
7,8-Diamino-nonanoate
6.3.3.3
Dethiobiotin
2.8.1.6
Biotin
Biotin sulfoxide
Biotin sulfone
BisC
L-Lysine
Lysine degradation
Tropane, piperidine and pyridine alkaloid biosynthesis
3.5.1.12
6.3.4.9
6.3.4.10
6.3.4.11
6.3.4.15
6.2.1.11
N6-D-Biotinyl-L-lysine (Biocytin)
3.4.-.-
Biotinyl-5'-AMP
6.2.1.11
Biotinyl-CoA
Bisnorbiotin
Tetranorbiotin
Urea
6.3.4.9
6.3.4.10
6.3.4.11
6.3.4.15
Fatty acid biosynthesis
Holo-[carboxylase]
00780 7/19/17
(c) Kanehisa Laboratories

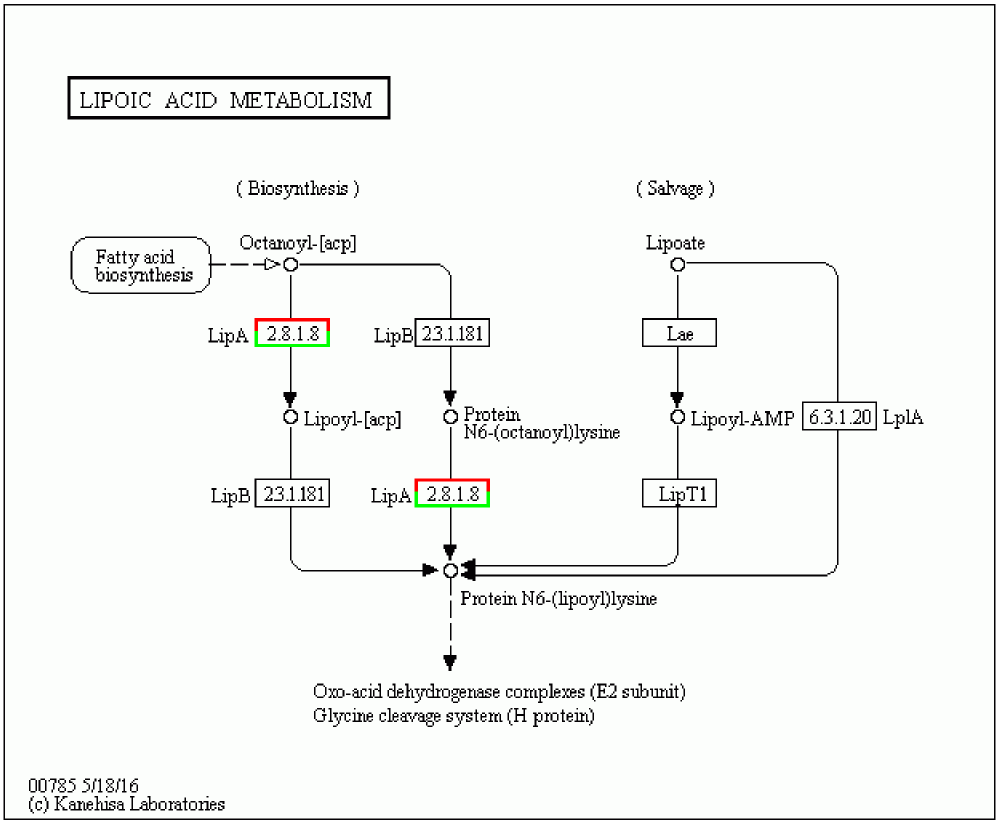
LIPOIC ACID METABOLISM
(Biosynthesis)
(Salvage)
Fatty acid biosynthesis
Octanoyl-[acp]
Lipoate
LipA 2.8.1.8
LipB 2.3.1.181
Lae
Lipoyl-[acp]
Protein N6-(octanoyl)lysine
Lipoyl-AMP
6.3.1.20 LplA
LipB 2.3.1.181
LipA 2.8.1.8
LipT1
Protein N6-(lipoyl)lysine
Oxo-acid dehydrogenase complexes (E2 subunit)
Glycine cleavage system (H protein)
00785 5/18/16
(c) Kanehisa Laboratories

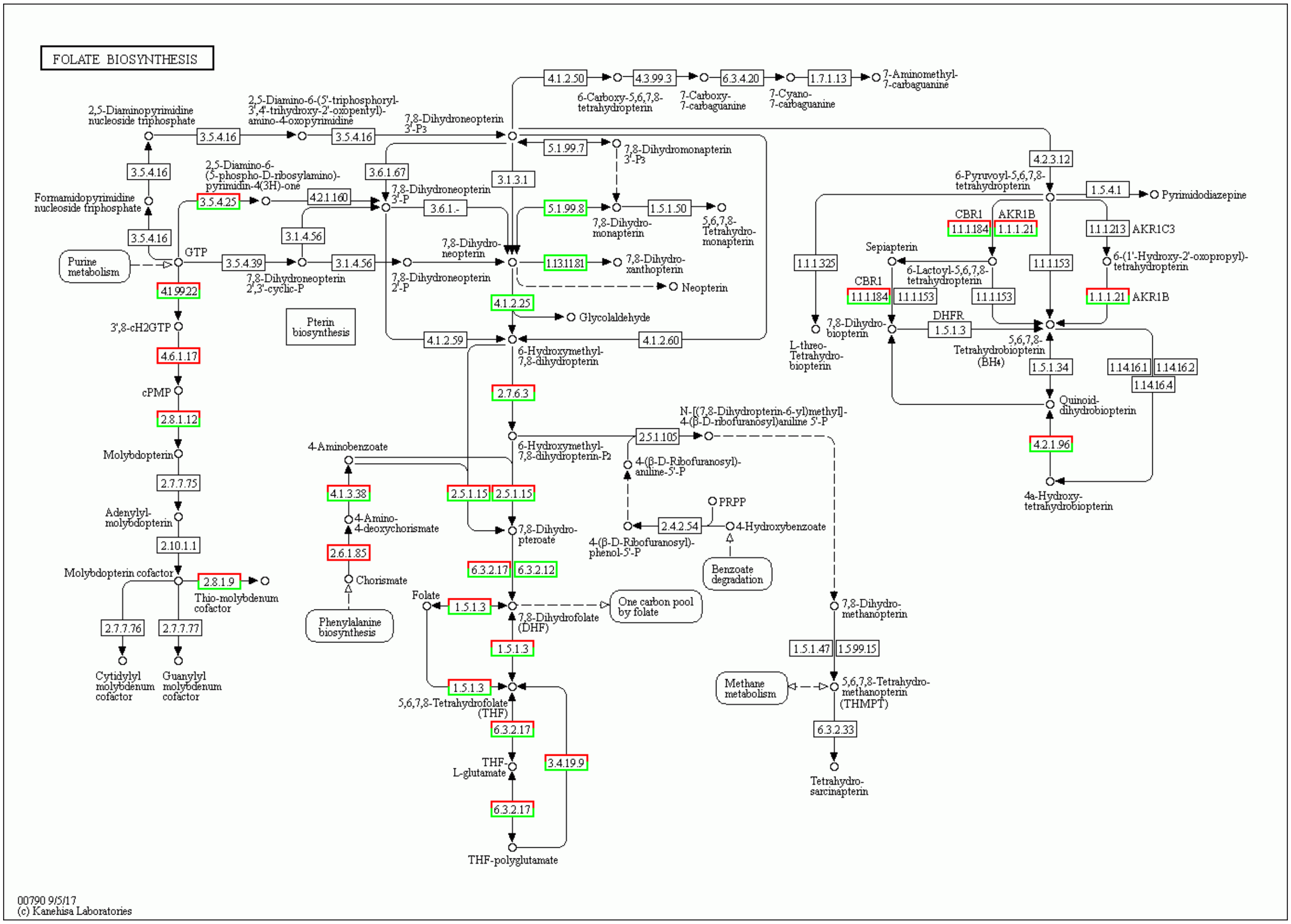
FOLATE BIOSYNTHESIS
GTP
Purine metabolism
2,5-Diaminopyrimidine nucleoside triphosphate
Formamidopyrimidine nucleoside triphosphate
2,5-Diamino-6-(5'-triphosphoryl-3',4'-trihydroxy-2'-oxopentyl)-amino-4-oxopyrimidine
2,5-Diamino-6-(5-phospho-D-ribosylamino)-pyrimidin-4(3H)-one
7,8-Dihydroneopterin 3'-P3
7,8-Dihydroneopterin 3'-P
7,8-Dihydroneopterin 2',3'-cyclic-P
7,8-Dihydroneopterin 2'-P
7,8-Dihydro-neopterin
7,8-Dihydromonapterin 3'-P3
7,8-Dihydro-monapterin
5,6,7,8-Tetrahydro-monapterin
7,8-Dihydro-xanthopterin
Neopterin
Glycolaldehyde
Pterin biosynthesis
6-Hydroxymethyl-7,8-dihydropterin
6-Hydroxymethyl-7,8-dihydropterin-P2
6-Carboxy-5,6,7,8-tetrahydropterin
7-Carboxy-7-carbaguanine
7-Cyano-7-carbaguanine
7-Aminomethyl-7-carbaguanine
3',8-cH2GTP
cPMP
Molybdopterin
Adenylyl-molybdopterin
Molybdopterin cofactor
Thio-molybdenum cofactor
Cytidylyl molybdenum cofactor
Guanylyl molybdenum cofactor
4-Aminobenzoate
4-Amino-4-deoxychorismate
Chorismate
Phenylalanine biosynthesis
7,8-Dihydro-pteroate
Folate
7,8-Dihydrofolate (DHF)
One carbon pool by folate
5,6,7,8-Tetrahydrofolate (THF)
THF-L-glutamate
THF-polyglutamate
N-[(7,8-Dihydropterin-6-yl)methyl]-4-(β-D-ribofuranosyl)aniline 5'-P
4-(β-D-Ribofuranosyl)-aniline-5'-P
4-(β-D-Ribofuranosyl)-phenol-5'-P
PRPP
4-Hydroxybenzoate
Benzoate degradation
7,8-Dihydro-methanopterin
5,6,7,8-Tetrahydro-methanopterin (THMPT)
Methane metabolism
Tetrahydro-sarcinapterin
6-Pyruvoyl-5,6,7,8-tetrahydropterin
Pyrimidodiazepine
Sepiapterin
6-Lactoyl-5,6,7,8-tetrahydropterin
6-(1'-Hydroxy-2'-oxopropyl)-tetrahydropterin
7,8-Dihydro-biopterin
L-threo-Tetrahydro-biopterin
5,6,7,8-Tetrahydrobiopterin (BH4)
Quinoid-dihydrobiopterin
4a-Hydroxy-tetrahydrobiopterin
CBR1
AKR1B
AKR1C3
DHFR
3.5.4.16
3.5.4.25
4.2.1.160
3.5.4.39
3.1.4.56
3.6.1.67
3.6.1.-
3.1.3.1
5.1.99.7
5.1.99.8
1.5.1.50
1.13.11.81
4.1.2.25
4.1.2.59
4.1.2.60
4.1.2.50
4.3.99.3
6.3.4.20
1.7.1.13
4.1.99.22
4.6.1.17
2.8.1.12
2.7.7.75
2.10.1.1
2.8.1.9
2.7.7.76
2.7.7.77
2.7.6.3
2.5.1.105
2.5.1.15
4.1.3.38
2.6.1.85
2.4.2.54
6.3.2.17
6.3.2.12
1.5.1.3
3.4.19.9
1.5.1.47
1.5.99.15
6.3.2.33
4.2.3.12
1.5.4.1
1.1.1.184
1.1.1.21
1.1.1.213
1.1.1.325
1.1.1.153
1.5.1.34
1.14.16.1
1.14.16.2
1.14.16.4
4.2.1.96
00790 9/5/17
(c) Kanehisa Laboratories

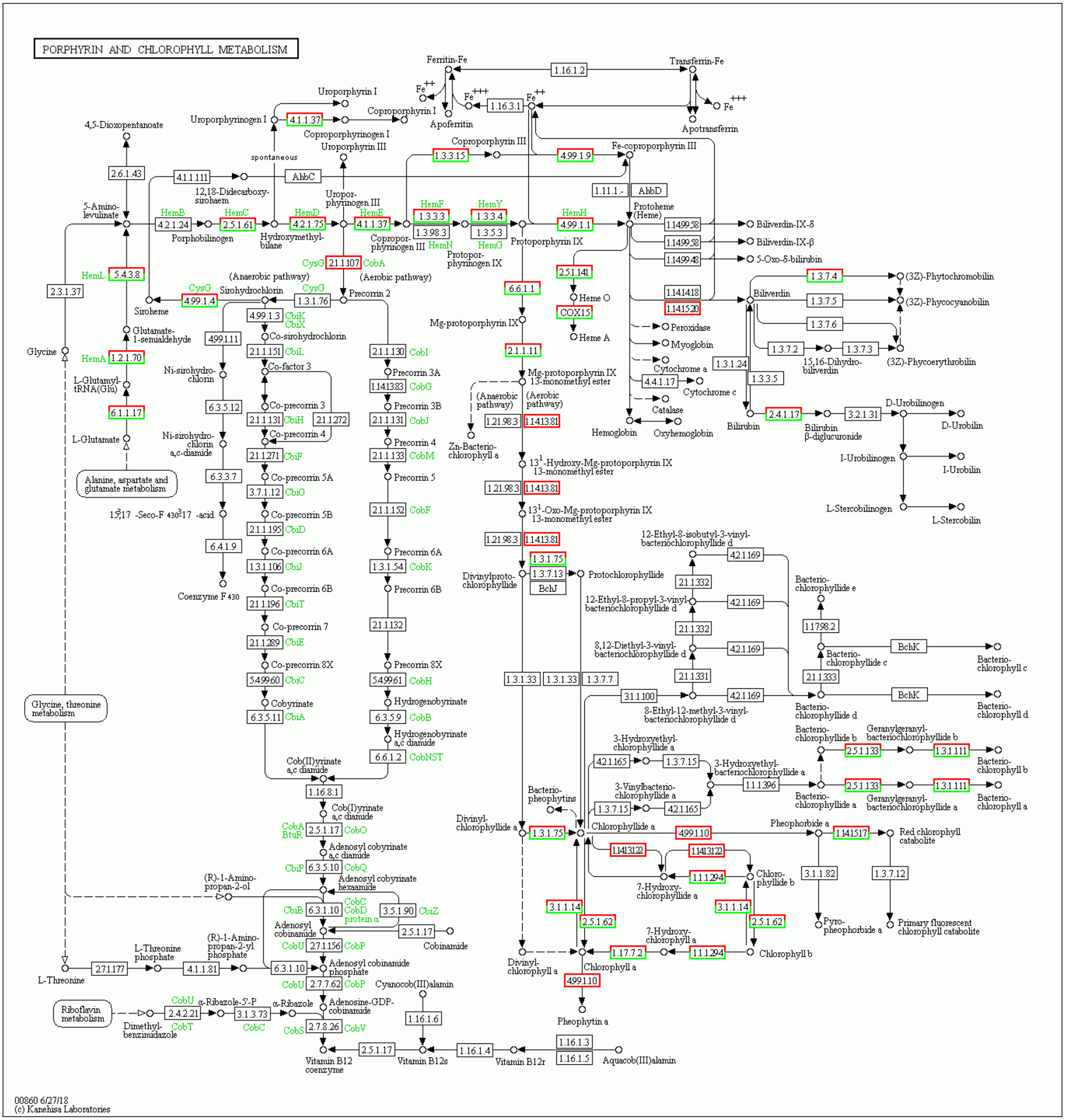

PORPHYRIN AND CHLOROPHYLL METABOLISM
Ferritin-Fe
1.16.1.2
Transferrin-Fe
Fe++
Fe+++
1.16.3.1
Fe++
Fe+++
Apoferritin
Apotransferrin
Uroporphyrin I
Coproporphyrin I
Uroporphyrinogen I
4.1.1.37
Coproporphyrinogen I
Uroporphyrin III
Coproporphyrin III
1.3.3.15
4.99.1.9
Fe-coproporphyrin III
4,5-Dioxopentanoate
2.6.1.43
4.1.1.111
spontaneous
AhbC
12,18-Didecarboxy-siroheam
1.11.1.-
AhbD
5-Amino-levulinate
HemB
4.2.1.24
Porphobilinogen
HemC
2.5.1.61
Hydroxymethyl-bilane
HemD
4.2.1.75
Uropor-phyrinogen III
HemE
4.1.1.37
Copropor-phyrinogen III
HemN
HemF
1.3.3.3
1.3.98.3
HemY
1.3.3.4
1.3.5.3
HemG
Protopor-phyrinogen IX
Protoporphyrin IX
HemH
4.99.1.1
Protoheme (Heme)
1.14.99.58
Biliverdin-IX-δ
1.14.99.58
Biliverdin-IX-β
1.14.99.48
5-Oxo-δ-bilirubin
CysG
2.1.1.107
CobA
HemL
5.4.3.8
(Anaerobic pathway)
(Aerobic pathway)
2.5.1.141
Heme O
COX15
Heme A
1.14.14.18
1.14.15.20
1.3.7.4
(3Z)-Phytochromobilin
Biliverdin
1.3.7.5
(3Z)-Phycocyanobilin
1.3.7.6
2.3.1.37
CysG
4.99.1.4
Sirohydrochlorin
CysG
1.3.1.76
Precorrin 2
6.6.1.1
Siroheme
4.99.1.3
CbiK
CbiX
Mg-protoporphyrin IX
Peroxidase
Glutamate-1-semialdehyde
4.99.1.11
Co-sirohydrochlorin
Myoglobin
Glycine
HemA
1.2.1.70
2.1.1.151
CbiL
2.1.1.130
CobI
2.1.1.11
Cytochrome a
1.3.1.24
1.3.7.2
1.3.7.3
15,16-Dihydro-biliverdin
(3Z)-Phycoerythrobilin
Ni-sirohydro-chlorin
Co-factor 3
Precorrin 3A
Mg-protoporphyrin IX 13-monomethyl ester
4.4.1.17
Cytochrome c
1.3.3.5
L-Glutamyl-tRNA(Glu)
1.14.13.83
CobG
6.3.5.12
Co-precorrin 3
Precorrin 3B
(Anaerobic pathway)
(Aerobic pathway)
Catalase
D-Urobilinogen
6.1.1.17
2.1.1.131
CbiH
2.1.1.272
2.1.1.131
CobJ
1.21.98.3
1.14.13.81
2.4.1.17
3.2.1.31
D-Urobilin
L-Glutamate
Ni-sirohydro-chlorin a,c-diamide
Co-precorrin 4
Precorrin 4
Hemoglobin
Oxyhemoglobin
Bilirubin
Bilirubin β-diglucuronide
2.1.1.271
CbiF
2.1.1.133
CobM
Zn-Bacterio-chlorophyll a
13¹-Hydroxy-Mg-protoporphyrin IX 13-monomethyl ester
I-Urobilinogen
Alanine, aspartate and glutamate metabolism
6.3.3.7
Co-precorrin 5A
Precorrin 5
I-Urobilin
3.7.1.12
CbiG
1.21.98.3
1.14.13.81
15³,17 -Seco-F 430 -17³-acid
Co-precorrin 5B
2.1.1.152
CobF
13¹-Oxo-Mg-protoporphyrin IX 13-monomethyl ester
L-Stercobilinogen
L-Stercobilin
2.1.1.195
CbiD
12-Ethyl-8-isobutyl-3-vinyl-bacteriochlorophyllide d
6.4.1.9
Co-precorrin 6A
Precorrin 6A
1.21.98.3
1.14.13.81
4.2.1.169
1.3.1.106
CbiJ
1.3.1.54
CobK
1.3.1.75
Divinylproto-chlorophyllide
1.3.7.13
Protochlorophyllide
Bacterio-chlorophyllide e
2.1.1.332
Coenzyme F430
Co-precorrin 6B
Precorrin 6B
BchJ
12-Ethyl-8-propyl-3-vinyl-bacteriochlorophyllide d
4.2.1.169
2.1.1.196
CbiT
2.1.1.132
2.1.1.332
1.17.98.2
Co-precorrin 7
8,12-Diethyl-3-vinyl-bacteriochlorophyllide d
4.2.1.169
BchK
2.1.1.289
CbiE
Bacterio-chlorophyllide c
Bacterio-chlorophyll c
Co-precorrin 8X
Precorrin 8X
2.1.1.331
5.4.99.60
CbiC
5.4.99.61
CobH
1.3.1.33
1.3.1.33
1.3.7.7
2.1.1.333
Glycine, threonine metabolism
Hydrogenobyrinate
3.1.1.100
4.2.1.169
BchK
Cobyrinate
8-Ethyl-12-methyl-3-vinyl-bacteriochlorophyllide d
Bacterio-chlorophyllide d
Bacterio-chlorophyll d
6.3.5.11
CbiA
6.3.5.9
CobB
Hydrogenobyrinate a,c diamide
3-Hydroxyethyl-chlorophyllide a
Bacterio-chlorophyllide b
Geranylgeranyl-bacteriochlorophyllide b
Cob(II)yrinate a,c diamide
6.6.1.2
CobNST
4.2.1.165
1.3.7.15
3-Hydroxyethyl-bacteriochlorophyllide a
2.5.1.133
1.3.1.111
Bacterio-chlorophyll b
1.16.8.1
Bacterio-pheophytins
3-Vinylbacterio-chlorophyllide a
1.1.1.396
2.5.1.133
1.3.1.111
Cob(I)yrinate a,c diamide
1.3.7.15
4.2.1.165
Bacterio-chlorophyllide a
Geranylgeranyl-bacteriochlorophyllide a
Bacterio-chlorophyll a
CobA
BtuR
2.5.1.17
CobO
Divinyl-chlorophyllide a
1.3.1.75
Chlorophyllide a
4.99.1.10
Pheophorbide a
1.14.15.17
Red chlorophyll catabolite
Adenosyl cobyrinate a,c diamide
1.14.13.122
1.14.13.122
CbiP
6.3.5.10
CobQ
Adenosyl cobyrinate hexaamide
1.1.1.294
Chloro-phyllide b
3.1.1.82
1.3.7.12
(R)-1-Amino-propan-2-ol
7-Hydroxy-chlorophyllide a
CbiB
6.3.1.10
CobC
CobD
protein α
3.5.1.90
CbiZ
3.1.1.14
2.5.1.62
3.1.1.14
2.5.1.62
Pyro-pheophorbide a
Primary fluorescent chlorophyll catabolite
Adenosyl cobinamide
2.5.1.17
Cobinamide
7-Hydroxy-chlorophyll a
L-Threonine phosphate
(R)-1-Amino-propan-2-yl phosphate
CobU
2.7.1.156
CobP
1.17.7.2
1.1.1.294
Chlorophyll b
2.7.1.177
4.1.1.81
6.3.1.10
Adenosyl cobinamide phosphate
Divinyl-chlorophyll a
Chlorophyll a
L-Threonine
CobU
2.7.7.62
CobP
Cyanocob(III)alamin
4.99.1.10
Riboflavin metabolism
CobU
α-Ribazole-5'-P
α-Ribazole
Adenosine-GDP-cobinamide
2.4.2.21
3.1.3.73
1.16.1.6
Pheophytin a
Dimethyl-benzimidazole
CobT
CobC
CobS
2.7.8.26
CobV
1.16.1.3
2.5.1.17
1.16.1.4
1.16.1.5
Aquacob(III)alamin
Vitamin B12 coenzyme
Vitamin B12s
Vitamin B12r
00860 6/27/18
(c) Kanehisa Laboratories

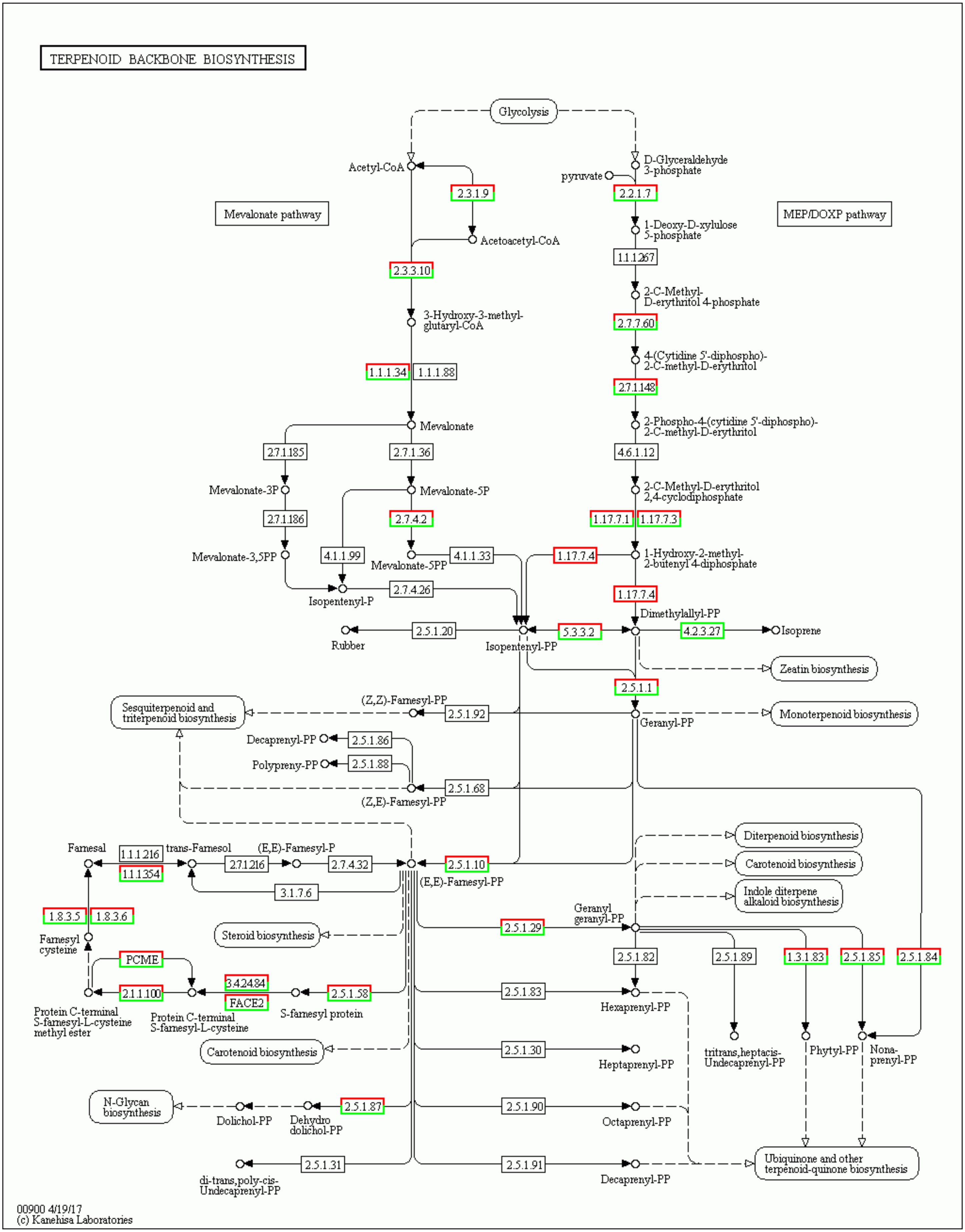

TERPENOID BACKBONE BIOSYNTHESIS
Glycolysis
Acetyl-CoA
2.3.1.9
Acetoacetyl-CoA
2.3.3.10
D-Glyceraldehyde 3-phosphate
pyruvate
2.2.1.7
Mevalonate pathway
MEP/DOXP pathway
1-Deoxy-D-xylulose 5-phosphate
1.1.1.267
2-C-Methyl-D-erythritol 4-phosphate
2.7.7.60
3-Hydroxy-3-methyl-glutaryl-CoA
1.1.1.34
1.1.1.88
4-(Cytidine 5'-diphospho)-2-C-methyl-D-erythritol
2.7.1.148
Mevalonate
2-Phospho-4-(cytidine 5'-diphospho)-2-C-methyl-D-erythritol
2.7.1.185
2.7.1.36
4.6.1.12
Mevalonate-3P
Mevalonate-5P
2-C-Methyl-D-erythritol 2,4-cyclodiphosphate
2.7.1.186
2.7.4.2
1.17.7.1
1.17.7.3
Mevalonate-3,5PP
4.1.1.99
4.1.1.33
Mevalonate-5PP
1.17.7.4
1-Hydroxy-2-methyl-2-butenyl 4-diphosphate
Isopentenyl-P
2.7.4.26
1.17.7.4
Dimethylallyl-PP
Rubber
2.5.1.20
Isopentenyl-PP
5.3.3.2
4.2.3.27
Isoprene
Zeatin biosynthesis
2.5.1.1
Sesquiterpenoid and triterpenoid biosynthesis
(Z,Z)-Farnesyl-PP
2.5.1.92
Geranyl-PP
Monoterpenoid biosynthesis
Decaprenyl-PP
2.5.1.86
Polypreny-PP
2.5.1.88
2.5.1.68
(Z,E)-Farnesyl-PP
Diterpenoid biosynthesis
Carotenoid biosynthesis
Farnesal
1.1.1.216
trans-Farnesol
(E,E)-Farnesyl-P
1.1.1.354
2.7.1.216
2.7.4.32
2.5.1.10
(E,E)-Farnesyl-PP
3.1.7.6
Indole diterpene alkaloid biosynthesis
1.8.3.5
1.8.3.6
Geranyl geranyl-PP
Farnesyl cysteine
Steroid biosynthesis
2.5.1.29
PCME
2.5.1.82
2.5.1.89
1.3.1.83
2.5.1.85
2.5.1.84
3.4.24.84
FACE2
2.1.1.100
2.5.1.58
2.5.1.83
Protein C-terminal S-farnesyl-L-cysteine methyl ester
Protein C-terminal S-farnesyl-L-cysteine
S-farnesyl protein
Hexaprenyl-PP
tritrans,heptacis-Undecaprenyl-PP
Phytyl-PP
Nona-prenyl-PP
Carotenoid biosynthesis
2.5.1.30
Heptaprenyl-PP
N-Glycan biosynthesis
Dolichol-PP
Dehydro dolichol-PP
2.5.1.87
2.5.1.90
Octaprenyl-PP
2.5.1.31
2.5.1.91
di-trans,poly-cis-Undecaprenyl-PP
Decaprenyl-PP
Ubiquinone and other terpenoid-quinone biosynthesis
00900 4/19/17
(c) Kanehisa Laboratories

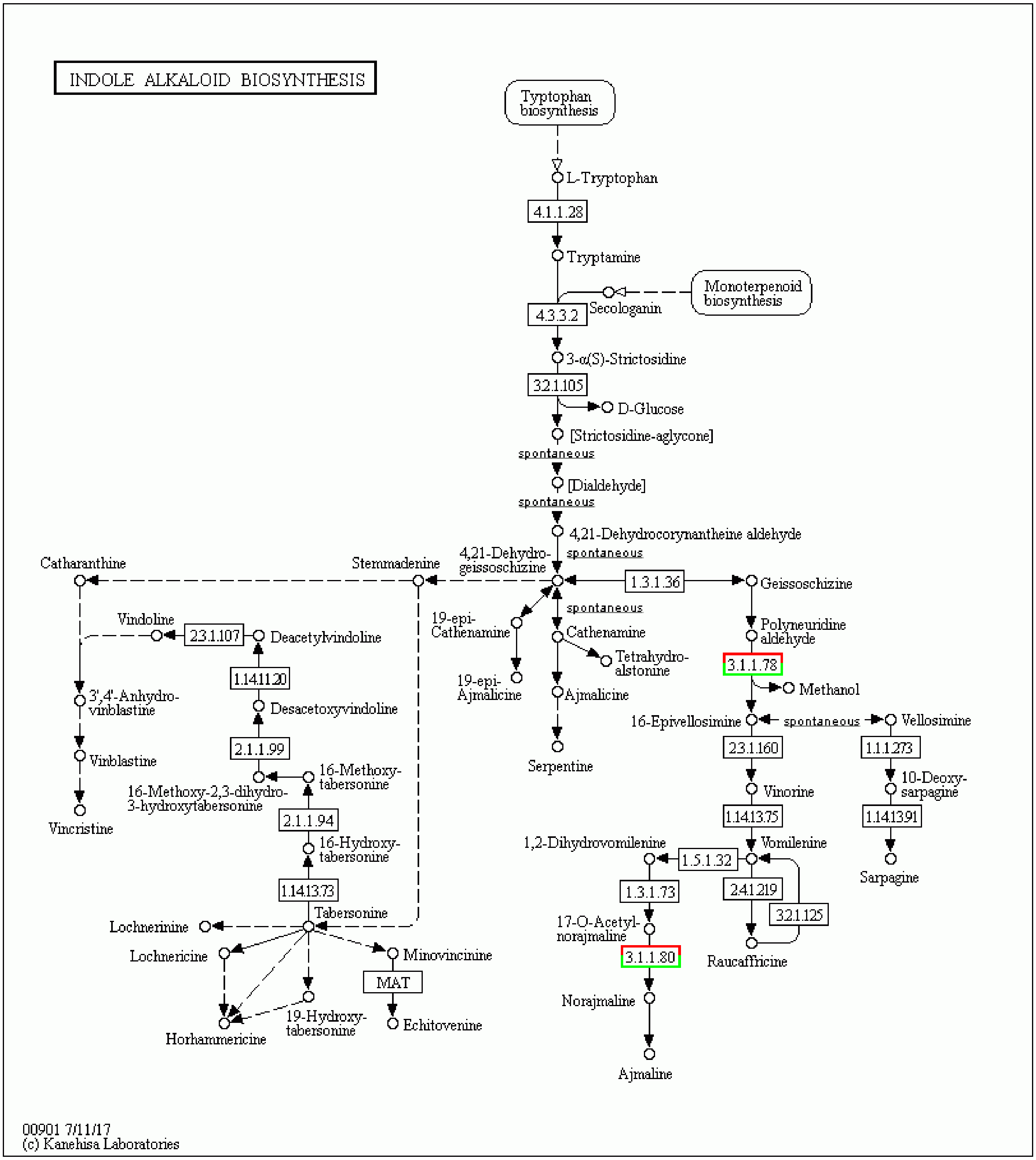
INDOLE ALKALOID BIOSYNTHESIS
Typtophan biosynthesis
L-Tryptophan
4.1.1.28
Tryptamine
Monoterpenoid biosynthesis
Secologanin
4.3.3.2
3-α(S)-Strictosidine
3.2.1.105
D-Glucose
[Strictosidine-aglycone]
spontaneous
[Dialdehyde]
spontaneous
4,21-Dehydrocorynantheine aldehyde
spontaneous
4,21-Dehydro-geissoschizine
1.3.1.36
Geissoschizine
Polyneuridine aldehyde
3.1.1.78
Methanol
16-Epivellosimine
spontaneous
Vellosimine
2.3.1.160
1.1.1.273
Vinorine
10-Deoxy-sarpagine
1.14.13.75
1.14.13.91
Vomilenine
Sarpagine
1,2-Dihydrovomilenine
1.5.1.32
2.4.1.219
3.2.1.125
1.3.1.73
Raucaffricine
17-O-Acetyl-norajmaline
3.1.1.80
Norajmaline
Ajmaline
spontaneous
Cathenamine
Tetrahydro-alstonine
Ajmalicine
Serpentine
19-epi-Cathenamine
19-epi-Ajmalicine
Catharanthine
Stemmadenine
Vindoline
2.3.1.107
Deacetylvindoline
1.14.11.20
Desacetoxyvindoline
2.1.1.99
16-Methoxy-tabersonine
16-Methoxy-2,3-dihydro-3-hydroxytabersonine
2.1.1.94
16-Hydroxy-tabersonine
1.14.13.73
Tabersonine
3',4'-Anhydro-vinblastine
Vinblastine
Vincristine
Lochnerinine
Lochnericine
Minovincinine
MAT
Echitovenine
19-Hydroxy-tabersonine
Horhammericine
00901 7/11/17
(c) Kanehisa Laboratories

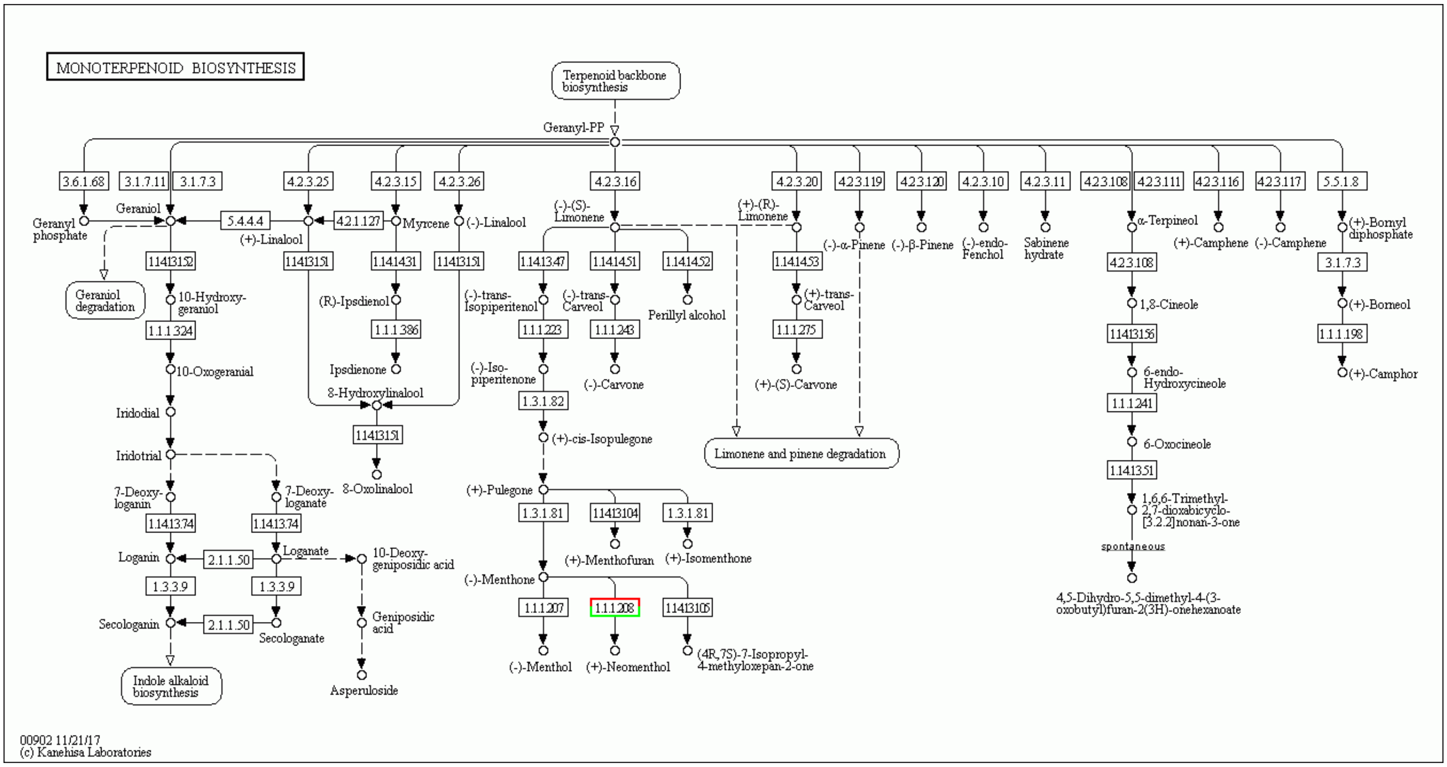
MONOTERPENOID BIOSYNTHESIS
Terpenoid backbone biosynthesis
Geranyl-PP
3.6.1.68
3.1.7.11
3.1.7.3
4.2.3.25
4.2.3.15
4.2.3.26
4.2.3.16
4.2.3.20
4.2.3.119
4.2.3.120
4.2.3.10
4.2.3.11
4.2.3.108
4.2.3.111
4.2.3.116
4.2.3.117
5.5.1.8
Geranyl phosphate
Geraniol
5.4.4.4
4.2.1.127
Myrcene
(-)-Linalool
(+)-Linalool
(-)-(S)-Limonene
(+)-(R)-Limonene
(-)-α-Pinene
(-)-β-Pinene
(-)-endo-Fenchol
Sabinene hydrate
α-Terpineol
(+)-Camphene
(-)-Camphene
(+)-Bornyl diphosphate
1.14.13.152
1.14.13.151
1.14.14.31
1.14.13.47
1.14.14.51
1.14.14.52
1.14.14.53
4.2.3.108
3.1.7.3
Geraniol degradation
10-Hydroxy-geraniol
(R)-Ipsdienol
(-)-trans-Isopiperitenol
(-)-trans-Carveol
Perillyl alcohol
(+)-trans-Carveol
1,8-Cineole
(+)-Borneol
1.1.1.324
1.1.1.386
1.1.1.223
1.1.1.243
1.1.1.275
1.14.13.156
1.1.1.198
10-Oxogeranial
Ipsdienone
(-)-Iso-piperitenone
(-)-Carvone
(+)-(S)-Carvone
6-endo-Hydroxycineole
(+)-Camphor
Iridodial
8-Hydroxylinalool
1.3.1.82
1.1.1.241
1.14.13.151
(+)-cis-Isopulegone
Limonene and pinene degradation
6-Oxocineole
Iridotrial
1.14.13.51
8-Oxolinalool
7-Deoxy-loganin
7-Deoxy-loganate
(+)-Pulegone
1,6,6-Trimethyl-2,7-dioxabicyclo-[3.2.2]nonan-3-one
1.14.13.74
1.3.1.81
1.14.13.104
spontaneous
Loganin
2.1.1.50
Loganate
10-Deoxy-geniposidic acid
(+)-Menthofuran
(+)-Isomenthone
(-)-Menthone
1.3.3.9
4,5-Dihydro-5,5-dimethyl-4-(3-oxobutyl)furan-2(3H)-onehexanoate
1.1.1.207
1.1.1.208
1.14.13.105
Secologanin
Secologanate
Geniposidic acid
(-)-Menthol
(+)-Neomenthol
(4R,7S)-7-Isopropyl-4-methyloxepan-2-one
Indole alkaloid biosynthesis
Asperuloside
00902 11/21/17
(c) Kanehisa Laboratories

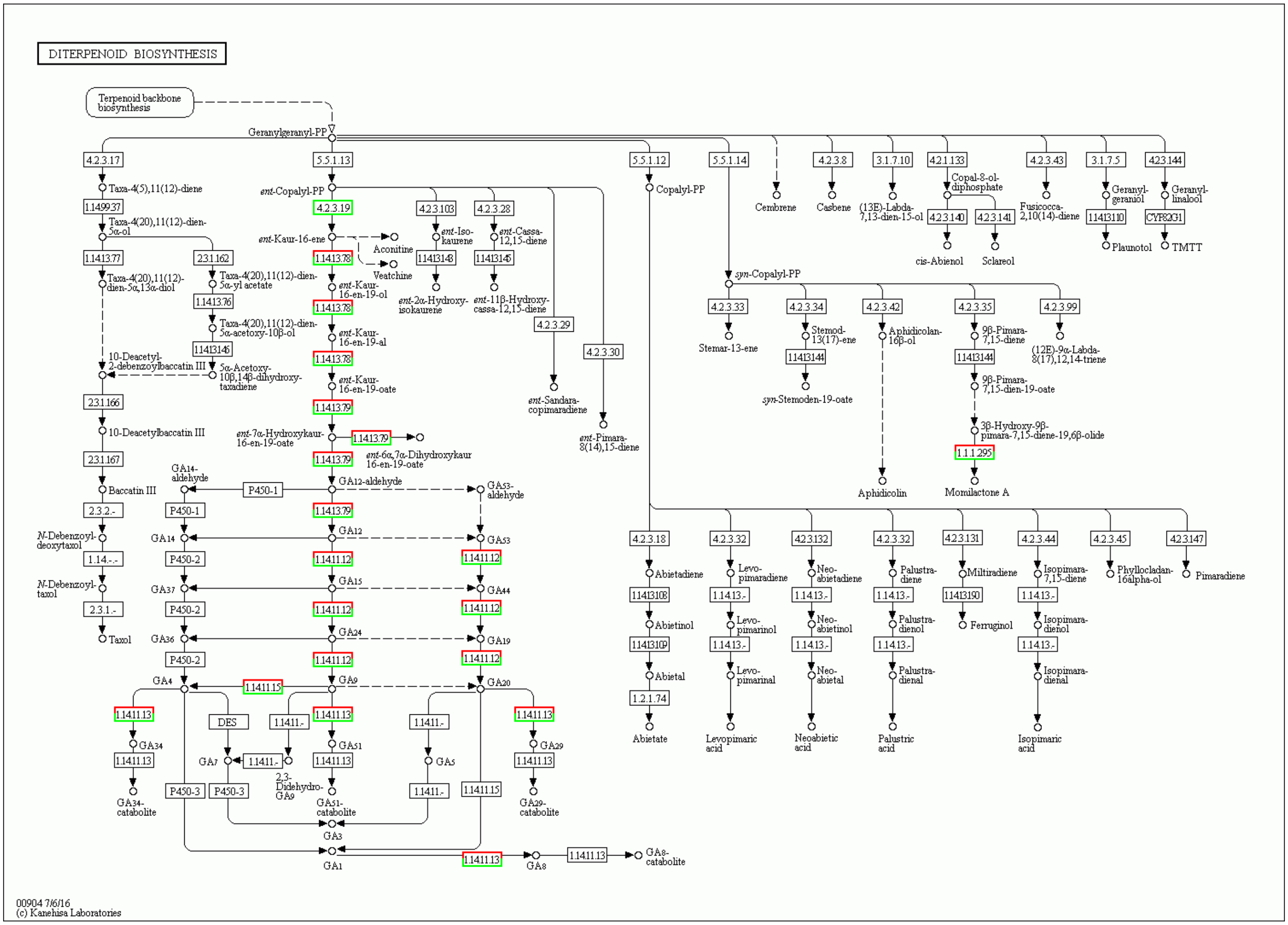
DITERPENOID BIOSYNTHESIS
Terpenoid backbone biosynthesis
Geranylgeranyl-PP
4.2.3.17
5.5.1.13
5.5.1.12
5.5.1.14
4.2.3.8
3.1.7.10
4.2.1.133
4.2.3.43
3.1.7.5
4.2.3.144
Taxa-4(5),11(12)-diene
ent-Copalyl-PP
Copalyl-PP
Cembrene
Casbene
(13E)-Labda-7,13-dien-15-ol
Copal-8-ol-diphosphate
Fusicocca-2,10(14)-diene
Geranyl-geraniol
Geranyl-linalool
1.14.99.37
4.2.3.19
4.2.3.103
4.2.3.28
4.2.3.140
4.2.3.141
1.14.13.110
CYP82G1
Taxa-4(20),11(12)-dien-5α-ol
ent-Kaur-16-ene
Aconitine
ent-Iso-kaurene
ent-Cassa-12,15-diene
cis-Abienol
Sclareol
Plaunotol
TMTT
1.14.13.77
2.3.1.162
1.14.13.78
Veatchine
1.14.13.148
1.14.13.145
syn-Copalyl-PP
Taxa-4(20),11(12)-dien-5α,13α-diol
Taxa-4(20),11(12)-dien-5α-yl acetate
ent-Kaur-16-en-19-ol
ent-2α-Hydroxy-isokaurene
ent-11β-Hydroxy-cassa-12,15-diene
4.2.3.33
4.2.3.34
4.2.3.42
4.2.3.35
4.2.3.99
1.14.13.76
Taxa-4(20),11(12)-dien-5α-acetoxy-10β-ol
4.2.3.29
Stemar-13-ene
Stemod-13(17)-ene
Aphidicolan-16β-ol
9β-Pimara-7,15-diene
(12E)-9α-Labda-8(17),12,14-triene
1.14.13.146
ent-Kaur-16-en-19-al
4.2.3.30
1.14.13.144
10-Deacetyl-2-debenzoylbaccatin III
5α-Acetoxy-10β,14β-dihydroxy-taxadiene
ent-Kaur-16-en-19-oate
9β-Pimara-7,15-dien-19-oate
syn-Stemoden-19-oate
2.3.1.166
1.14.13.79
ent-Sandara-copimaradiene
10-Deacetylbaccatin III
ent-7α-Hydroxykaur-16-en-19-oate
ent-Pimara-8(14),15-diene
3β-Hydroxy-9β-pimara-7,15-diene-19,6β-olide
2.3.1.167
ent-6α,7α-Dihydroxykaur-16-en-19-oate
1.1.1.295
GA14-aldehyde
Baccatin III
P450-1
GA12-aldehyde
GA53-aldehyde
Aphidicolin
Momilactone A
2.3.2.-
N-Debenzoyl-deoxytaxol
GA14
P450-2
GA12
GA53
4.2.3.18
4.2.3.32
4.2.3.132
4.2.3.131
4.2.3.44
4.2.3.45
4.2.3.147
1.14.-.-
1.14.11.12
Abietadiene
Levo-pimaradiene
Neo-abietadiene
Palustra-diene
Miltiradiene
Isopimara-7,15-diene
Phyllocladan-16alpha-ol
Pimaradiene
N-Debenzoyl-taxol
GA37
GA15
GA44
1.14.13.108
1.14.13.-
1.14.13.190
2.3.1.-
Abietinol
Levo-pimarinol
Neo-abietinol
Palustra-dienol
Ferruginol
Isopimara-dienol
Taxol
GA36
GA24
GA19
1.14.13.109
GA4
1.14.11.15
GA9
GA20
Abietal
Levo-pimarinal
Neo-abietal
Palustra-dienal
Isopimara-dienal
1.2.1.74
1.14.11.13
DES
1.14.11.-
GA34
GA51
GA5
GA29
GA7
2,3-Didehydro-GA9
Abietate
Levopimaric acid
Neoabietic acid
Palustric acid
Isopimaric acid
P450-3
GA34-catabolite
GA51-catabolite
GA29-catabolite
GA3
GA1
GA8
GA8-catabolite
00904 7/6/16
(c) Kanehisa Laboratories

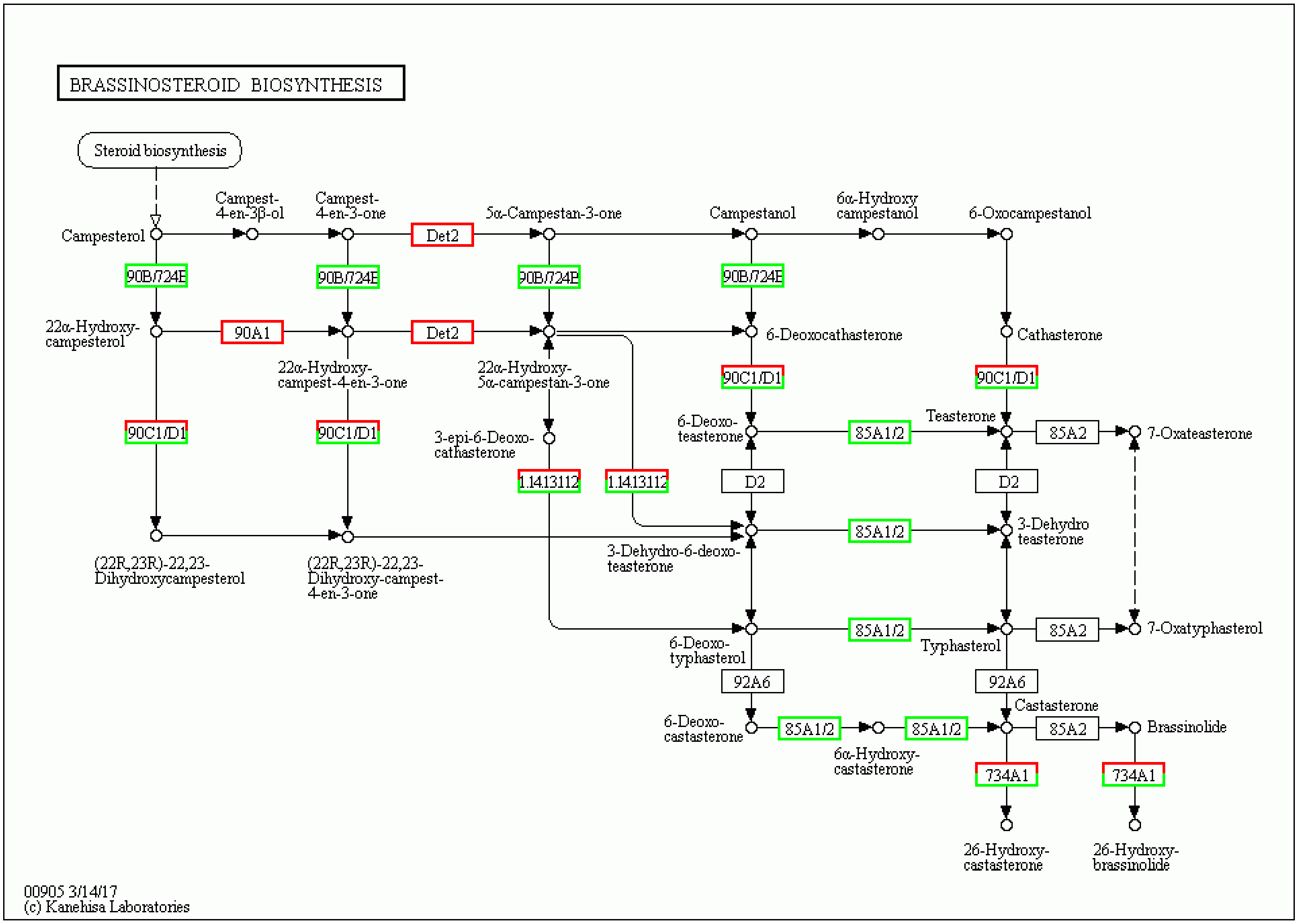
BRASSINOSTEROID BIOSYNTHESIS
Steroid biosynthesis
Campesterol
Campest-4-en-3β-ol
Campest-4-en-3-one
Det2
5α-Campestan-3-one
Campestanol
6α-Hydroxy campestanol
6-Oxocampestanol
90B/724B
22α-Hydroxy-campesterol
90A1
22α-Hydroxy-campest-4-en-3-one
22α-Hydroxy-5α-campestan-3-one
6-Deoxocathasterone
Cathasterone
90C1/D1
3-epi-6-Deoxo-cathasterone
1.14.13.112
6-Deoxo-teasterone
85A1/2
Teasterone
85A2
7-Oxateasterone
D2
3-Dehydro-6-deoxo-teasterone
3-Dehydro teasterone
(22R,23R)-22,23-Dihydroxycampesterol
(22R,23R)-22,23-Dihydroxy-campest-4-en-3-one
6-Deoxo-typhasterol
Typhasterol
7-Oxatyphasterol
92A6
6-Deoxo-castasterone
6α-Hydroxy-castasterone
Castasterone
Brassinolide
734A1
26-Hydroxy-castasterone
26-Hydroxy-brassinolide
00905 3/14/17
(c) Kanehisa Laboratories

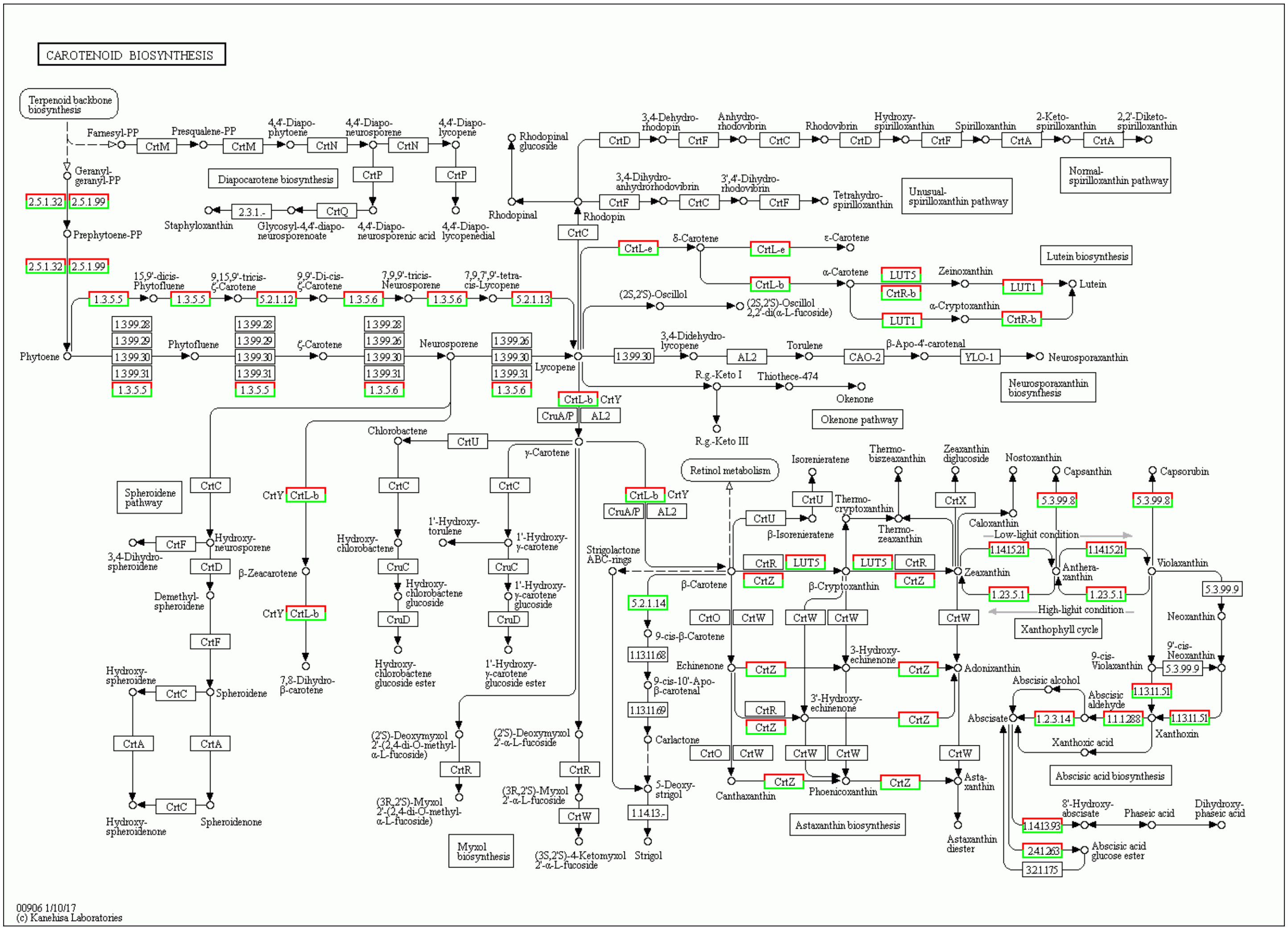
CAROTENOID BIOSYNTHESIS
Terpenoid backbone biosynthesis
Farnesyl-PP
Presqualene-PP
4,4'-Diapo-phytoene
4,4'-Diapo-neurosporene
4,4'-Diapo-lycopene
CrtM
CrtN
CrtP
CrtQ
2.3.1.-
Geranyl-geranyl-PP
Diapocarotene biosynthesis
Staphyloxanthin
Glycosyl-4,4'-diapo-neurosporenoate
4,4'-Diapo-neurosporenic acid
4,4'-Diapo-lycopenedial
2.5.1.32
2.5.1.99
Prephytoene-PP
15,9'-dicis-Phytofluene
9,15,9'-tricis-ζ-Carotene
9,9'-Di-cis-ζ-Carotene
7,9,9'-tricis-Neurosporene
7,9,7',9'-tetra-cis-Lycopene
1.3.5.5
5.2.1.12
1.3.5.6
5.2.1.13
1.3.99.28
1.3.99.29
1.3.99.30
1.3.99.31
1.3.99.26
Phytoene
Phytofluene
ζ-Carotene
Neurosporene
Lycopene
Rhodopinal glucoside
Rhodopinal
Rhodopin
CrtC
CrtD
CrtF
CrtA
3,4-Dehydro-rhodopin
Anhydro-rhodovibrin
Rhodovibrin
Hydroxy-spirilloxanthin
Spirilloxanthin
2-Keto-spirilloxanthin
2,2'-Diketo-spirilloxanthin
Normal-spirilloxanthin pathway
3,4-Dihydro-anhydrorhodovibrin
3',4'-Dihydro-rhodovibrin
Tetrahydro-spirilloxanthin
Unusual-spirilloxanthin pathway
CrtL-e
δ-Carotene
ε-Carotene
Lutein biosynthesis
CrtL-b
α-Carotene
LUT5
CrtR-b
Zeinoxanthin
LUT1
Lutein
α-Cryptoxanthin
(2S,2'S)-Oscillol
(2S,2'S)-Oscillol 2,2'-di(α-L-fucoside)
3,4-Didehydro-lycopene
AL2
Torulene
CAO-2
β-Apo-4'-carotenal
YLO-1
Neurosporaxanthin
Neurosporaxanthin biosynthesis
R.g.-Keto I
Thiothece-474
Okenone
Okenone pathway
R.g.-Keto III
CrtY
CruA/P
Chlorobactene
CrtU
γ-Carotene
Spheroidene pathway
CrtC
Hydroxy-neurosporene
3,4-Dihydro-spheroidene
CrtD
β-Zeacarotene
Demethyl-spheroidene
7,8-Dihydro-β-carotene
Hydroxy-spheroidene
Spheroidene
Hydroxy-spheroidenone
Spheroidenone
1'-Hydroxy-torulene
Hydroxy-chlorobactene
1'-Hydroxy-γ-carotene
CruC
Hydroxy-chlorobactene glucoside
1'-Hydroxy-γ-carotene glucoside
CruD
Hydroxy-chlorobactene glucoside ester
1'-Hydroxy-γ-carotene glucoside ester
(2'S)-Deoxymyxol 2'-(2,4-di-O-methyl-α-L-fucoside)
(2'S)-Deoxymyxol 2'-α-L-fucoside
CrtR
(3R,2'S)-Myxol 2'-(2,4-di-O-methyl-α-L-fucoside)
(3R,2'S)-Myxol 2'-α-L-fucoside
CrtW
Myxol biosynthesis
(3S,2'S)-4-Ketomyxol 2'-α-L-fucoside
Retinol metabolism
Strigolactone ABC-rings
β-Carotene
5.2.1.14
9-cis-β-Carotene
1.13.11.68
9-cis-10'-Apo-β-carotenal
1.13.11.69
Carlactone
5-Deoxy-strigol
1.14.13.-
Strigol
Isorenieratene
β-Isorenieratene
Thermo-biszeaxanthin
Thermo-cryptoxanthin
Thermo-zeaxanthin
Zeaxanthin diglucoside
CrtX
Nostoxanthin
Caloxanthin
Capsanthin
Capsorubin
5.3.99.8
β-Cryptoxanthin
Zeaxanthin
Low-light condition
1.14.15.21
Anthera-xanthin
Violaxanthin
1.23.5.1
High-light condition
Xanthophyll cycle
5.3.99.9
Neoxanthin
CrtO
CrtZ
Echinenone
3-Hydroxy-echinenone
3'-Hydroxy-echinenone
Adonixanthin
9-cis-Violaxanthin
9'-cis-Neoxanthin
Abscisic alcohol
Abscisic aldehyde
1.13.11.51
1.2.3.14
1.1.1.288
Abscisate
Xanthoxin
Xanthoxic acid
Abscisic acid biosynthesis
Canthaxanthin
Phoenicoxanthin
Asta-xanthin
Astaxanthin biosynthesis
Astaxanthin diester
8'-Hydroxy-abscisate
1.14.13.93
Phaseic acid
Dihydroxy-phaseic acid
2.4.1.263
3.2.1.175
Abscisic acid glucose ester
00906 1/10/17
(c) Kanehisa Laboratories

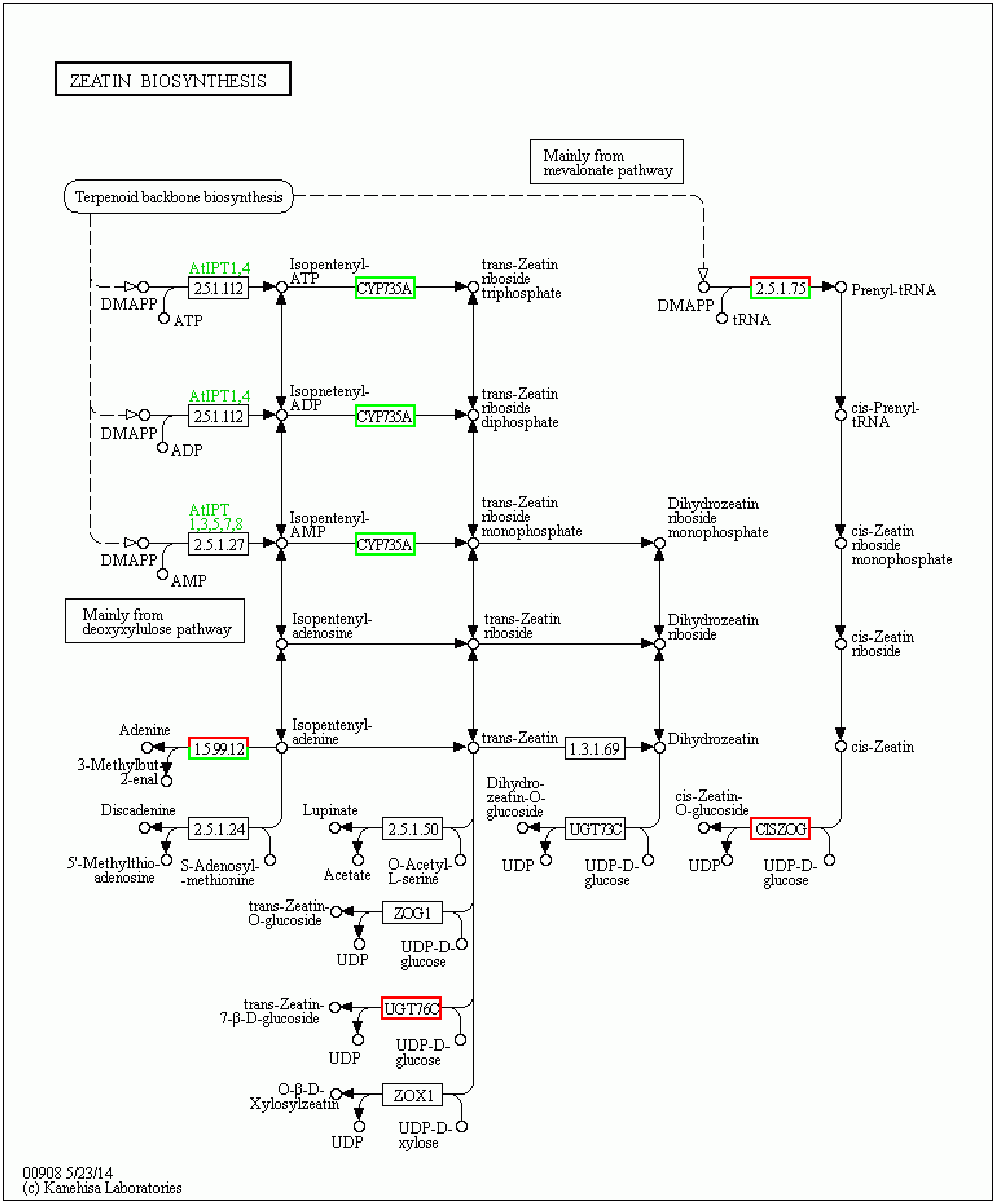
ZEATIN BIOSYNTHESIS
Mainly from mevalonate pathway
Terpenoid backbone biosynthesis
AtIPT1,4
2.5.1.112
Isopentenyl-ATP
CYP735A
trans-Zeatin riboside triphosphate
DMAPP
ATP
2.5.1.75
Prenyl-tRNA
tRNA
Isopnetenyl-ADP
trans-Zeatin riboside diphosphate
ADP
cis-Prenyl-tRNA
AtIPT 1,3,5,7,8
2.5.1.27
Isopentenyl-AMP
trans-Zeatin riboside monophosphate
Dihydrozeatin riboside monophosphate
cis-Zeatin riboside monophosphate
AMP
Mainly from deoxyxylulose pathway
Isopentenyl-adenosine
trans-Zeatin riboside
Dihydrozeatin riboside
cis-Zeatin riboside
Adenine
1.5.99.12
Isopentenyl-adenine
trans-Zeatin
1.3.1.69
Dihydrozeatin
cis-Zeatin
3-Methylbut-2-enal
Dihydro-zeatin-O-glucoside
cis-Zeatin-O-glucoside
Discadenine
2.5.1.24
Lupinate
2.5.1.50
UGT73C
CISZOG
5'-Methylthio-adenosine
S-Adenosyl--methionine
Acetate
O-Acetyl-L-serine
UDP
UDP-D-glucose
trans-Zeatin-O-glucoside
ZOG1
trans-Zeatin-7-β-D-glucoside
UGT76C
O-β-D-Xylosylzeatin
ZOX1
UDP-D-xylose
00908 5/23/14
(c) Kanehisa Laboratories

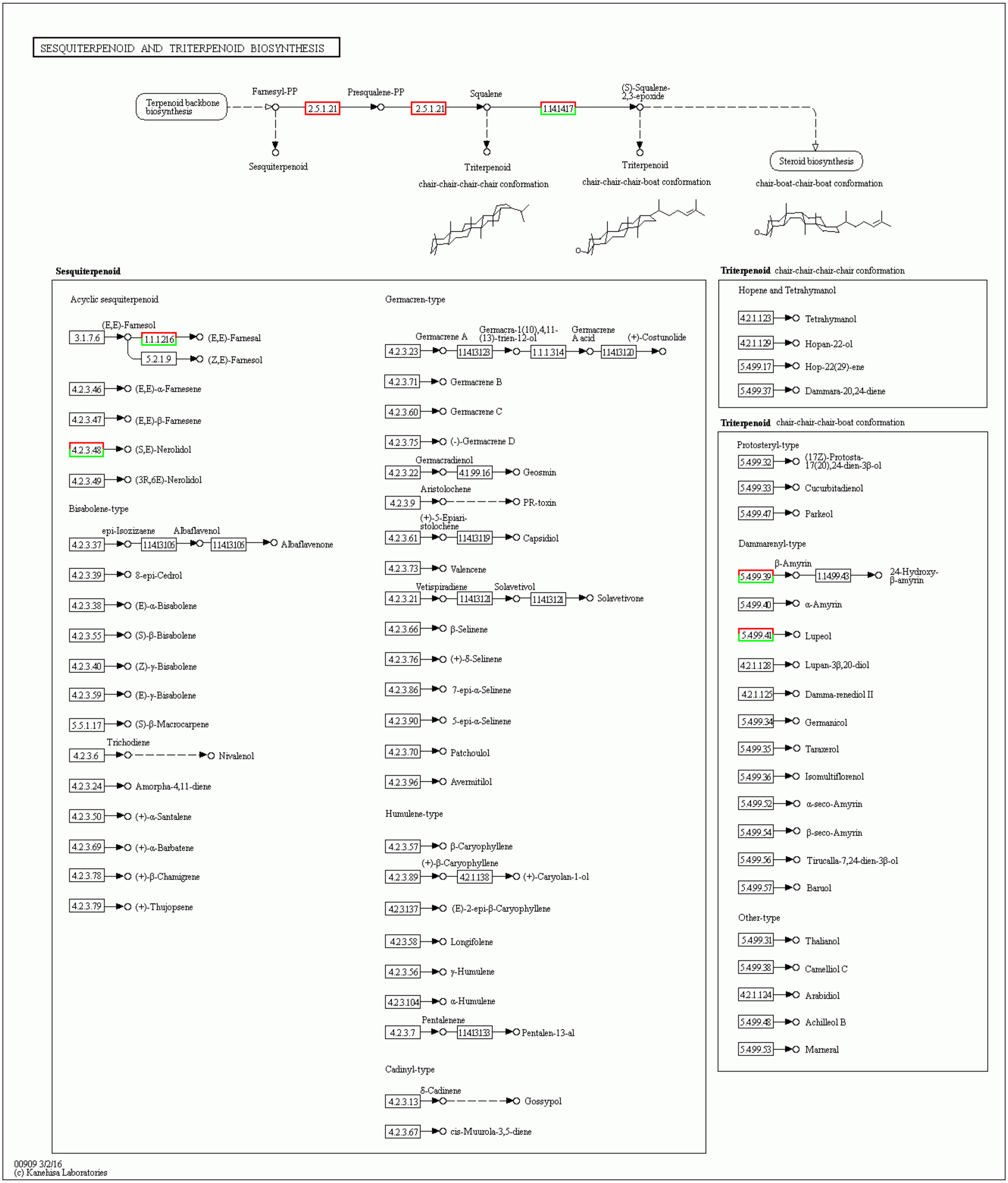
SESQUITERPENOID AND TRITERPENOID BIOSYNTHESIS
Terpenoid backbone biosynthesis
Farnesyl-PP
2.5.1.21
Presqualene-PP
2.5.1.21
Squalene
1.14.14.17
(S)-Squalene-2,3-epoxide
Sesquiterpenoid
Triterpenoid
chair-chair-chair-chair conformation
Triterpenoid
chair-chair-chair-boat conformation
Steroid biosynthesis
chair-boat-chair-boat conformation
Sesquiterpenoid
Acyclic sesquiterpenoid
(E,E)-Farnesol
3.1.7.6
1.1.1.216
(E,E)-Farnesal
5.2.1.9
(Z,E)-Farnesol
4.2.3.46
(E,E)-α-Farnesene
4.2.3.47
(E,E)-β-Farnesene
4.2.3.48
(S,E)-Nerolidol
4.2.3.49
(3R,6E)-Nerolidol
Bisabolene-type
epi-Isozizaene
Albaflavenol
4.2.3.37
1.14.13.106
1.14.13.106
Albaflavenone
4.2.3.39
8-epi-Cedrol
4.2.3.38
(E)-α-Bisabolene
4.2.3.55
(S)-β-Bisabolene
4.2.3.40
(Z)-γ-Bisabolene
4.2.3.59
(E)-γ-Bisabolene
5.5.1.17
(S)-β-Macrocarpene
Trichodiene
4.2.3.6
Nivalenol
4.2.3.24
Amorpha-4,11-diene
4.2.3.50
(+)-α-Santalene
4.2.3.69
(+)-α-Barbatene
4.2.3.78
(+)-β-Chamigrene
4.2.3.79
(+)-Thujopsene
Germacren-type
Germacrene A
Germacra-1(10),4,11-(13)-trien-12-ol
Germacrene A acid
(+)-Costunolide
4.2.3.23
1.14.13.123
1.1.1.314
1.14.13.120
4.2.3.71
Germacrene B
4.2.3.60
Germacrene C
4.2.3.75
(-)-Germacrene D
Germacradienol
4.2.3.22
4.1.99.16
Geosmin
Aristolochene
4.2.3.9
PR-toxin
(+)-5-Epiaristolochene
4.2.3.61
1.14.13.119
Capsidiol
4.2.3.73
Valencene
Vetispiradiene
Solavetivol
4.2.3.21
1.14.13.121
1.14.13.121
Solavetivone
4.2.3.66
β-Selinene
4.2.3.76
(+)-δ-Selinene
4.2.3.86
7-epi-α-Selinene
4.2.3.90
5-epi-α-Selinene
4.2.3.70
Patchoulol
4.2.3.96
Avermitilol
Humulene-type
4.2.3.57
β-Caryophyllene
(+)-β-Caryophyllene
4.2.3.89
4.2.1.138
(+)-Caryolan-1-ol
4.2.3.137
(E)-2-epi-β-Caryophyllene
4.2.3.58
Longifolene
4.2.3.56
γ-Humulene
4.2.3.104
α-Humulene
Pentalenene
4.2.3.7
1.14.13.133
Pentalen-13-al
Cadinyl-type
δ-Cadinene
4.2.3.13
Gossypol
4.2.3.67
cis-Muurola-3,5-diene
Triterpenoid chair-chair-chair-chair conformation
Hopene and Tetrahymanol
4.2.1.123
Tetrahymanol
4.2.1.129
Hopan-22-ol
5.4.99.17
Hop-22(29)-ene
5.4.99.37
Dammara-20,24-diene
Triterpenoid chair-chair-chair-boat conformation
Protosteryl-type
5.4.99.32
(17Z)-Protosta-17(20),24-dien-3β-ol
5.4.99.33
Cucurbitadienol
5.4.99.47
Parkeol
Dammarenyl-type
β-Amyrin
5.4.99.39
1.14.99.43
24-Hydroxy-β-amyrin
5.4.99.40
α-Amyrin
5.4.99.41
Lupeol
4.2.1.128
Lupan-3β,20-diol
4.2.1.125
Dammarenediol II
5.4.99.34
Germanicol
5.4.99.35
Taraxerol
5.4.99.36
Isomultiflorenol
5.4.99.52
α-seco-Amyrin
5.4.99.54
β-seco-Amyrin
5.4.99.56
Tirucalla-7,24-dien-3β-ol
5.4.99.57
Baruol
Other-type
5.4.99.31
Thalianol
5.4.99.38
Camelliol C
4.2.1.124
Arabidiol
5.4.99.48
Achilleol B
5.4.99.53
Marneral
00909 3/2/16
(c) Kanehisa Laboratories

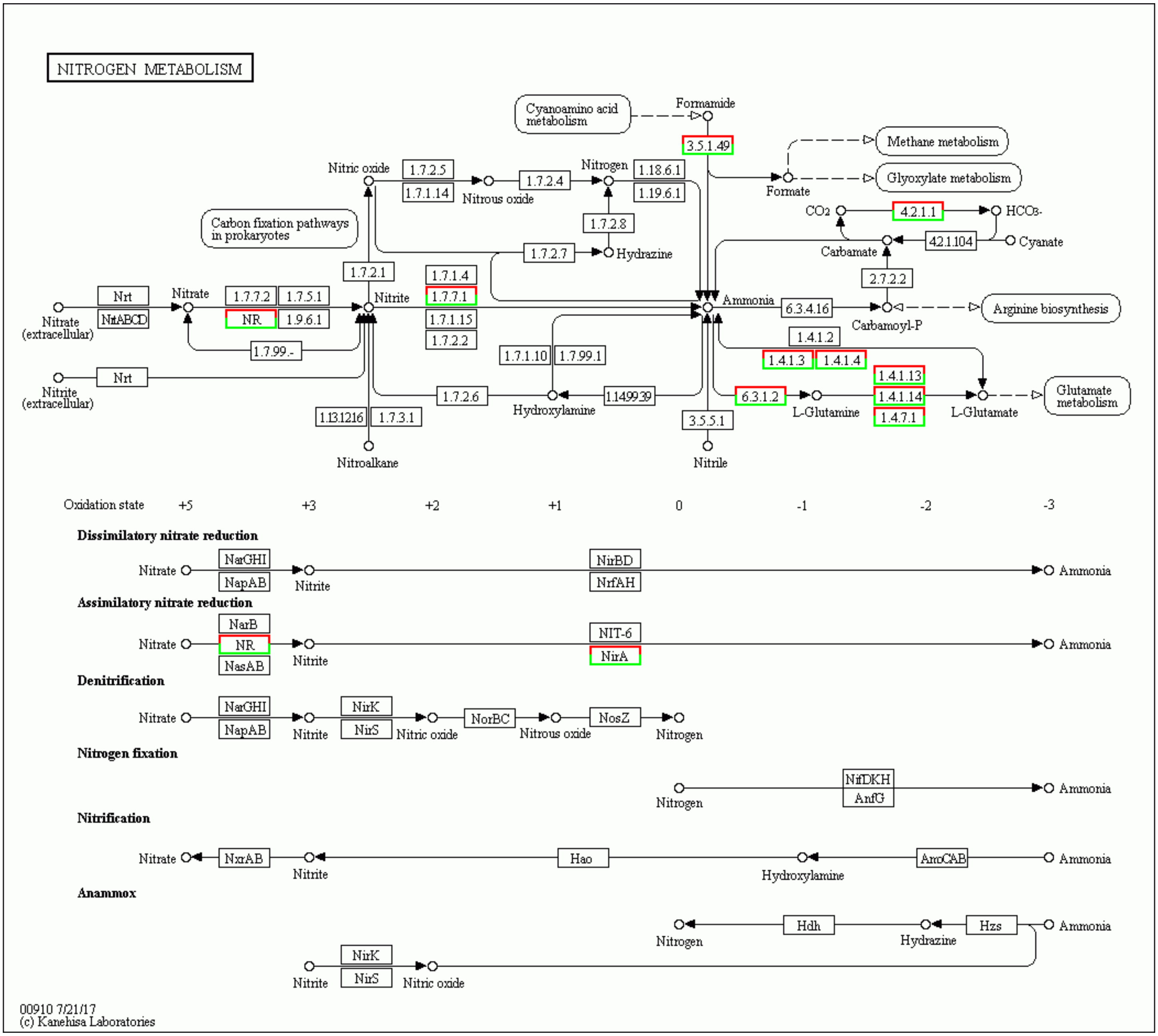
NITROGEN METABOLISM
Cyanoamino acid metabolism
Formamide
3.5.1.49
Methane metabolism
Glyoxylate metabolism
Formate
Nitric oxide
1.7.2.5
1.7.1.14
Nitrous oxide
1.7.2.4
Nitrogen
1.18.6.1
1.19.6.1
CO2
4.2.1.1
HCO3-
Carbon fixation pathways in prokaryotes
1.7.2.8
1.7.2.7
Hydrazine
Carbamate
4.2.1.104
Cyanate
1.7.2.1
1.7.1.4
1.7.7.1
2.7.2.2
Nitrate (extracellular)
Nrt
NrtABCD
Nitrate
1.7.7.2
1.7.5.1
NR
1.9.6.1
Nitrite
1.7.1.15
1.7.2.2
Ammonia
6.3.4.16
Carbamoyl-P
Arginine biosynthesis
1.7.99.-
1.4.1.2
1.4.1.3
1.4.1.4
1.7.1.10
1.7.99.1
Nitrite (extracellular)
1.4.1.13
1.7.2.6
Hydroxylamine
1.14.99.39
6.3.1.2
L-Glutamine
1.4.1.14
L-Glutamate
Glutamate metabolism
1.13.12.16
1.7.3.1
3.5.5.1
1.4.7.1
Nitroalkane
Nitrile
Oxidation state
+5
+3
+2
+1
0
-1
-2
-3
Dissimilatory nitrate reduction
Nitrate
NarGHI
NapAB
Nitrite
NirBD
NrfAH
Ammonia
Assimilatory nitrate reduction
Nitrate
NarB
NR
NasAB
Nitrite
NIT-6
NirA
Ammonia
Denitrification
Nitrate
NarGHI
NapAB
Nitrite
NirK
NirS
Nitric oxide
NorBC
Nitrous oxide
NosZ
Nitrogen
Nitrogen fixation
Nitrogen
NifDKH
AnfG
Ammonia
Nitrification
Nitrate
NxrAB
Nitrite
Hao
Hydroxylamine
AmoCAB
Ammonia
Anammox
Nitrogen
Hdh
Hydrazine
Hzs
Ammonia
Nitrite
NirK
NirS
Nitric oxide
00910 7/21/17
(c) Kanehisa Laboratories

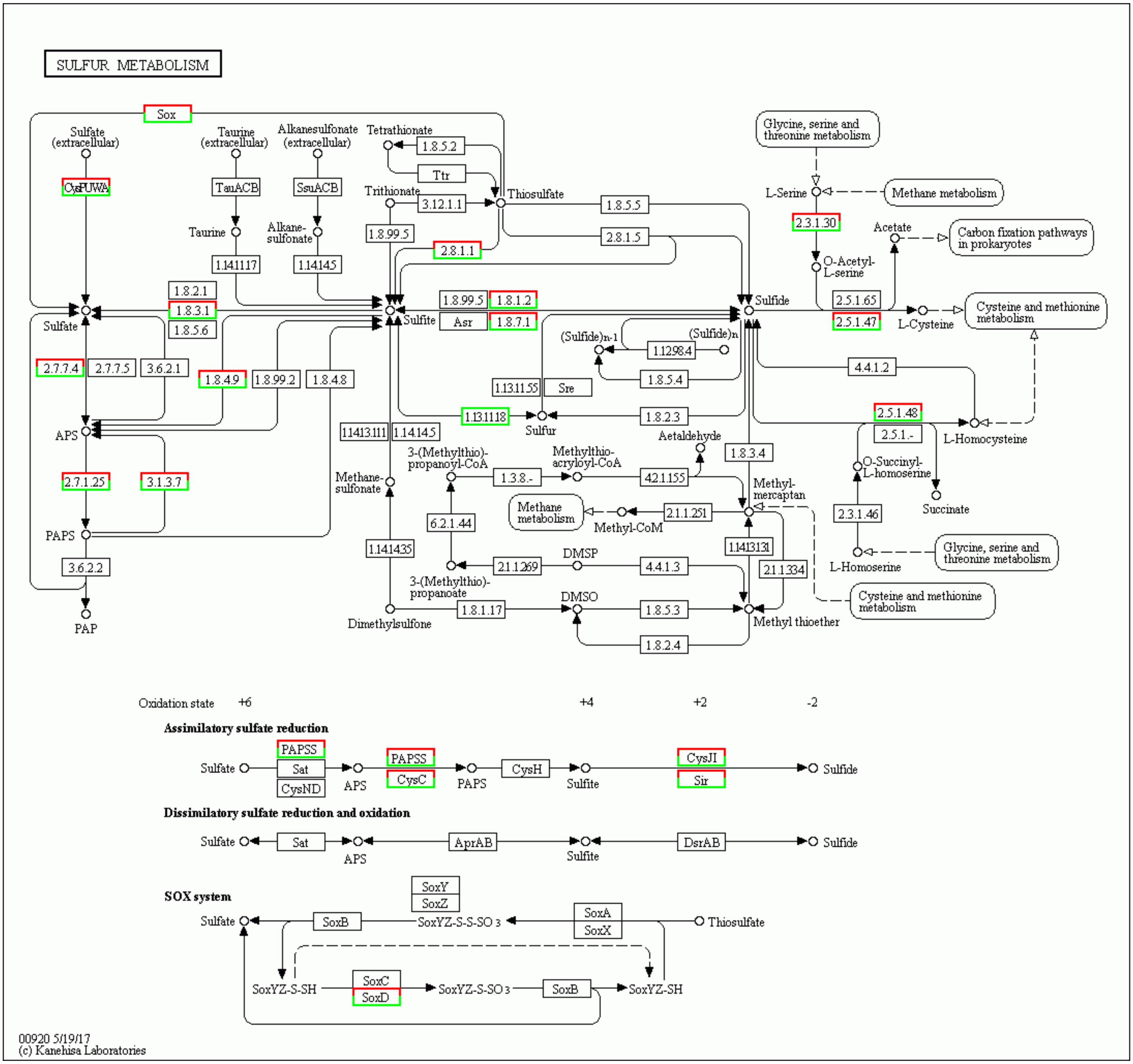
SULFUR METABOLISM
Sox
Sulfate (extracellular)
CysPUWA
Taurine (extracellular)
TauACB
Alkanesulfonate (extracellular)
SsuACB
Tetrathionate
1.8.5.2
Ttr
Trithionate
3.12.1.1
Thiosulfate
1.8.5.5
2.8.1.5
1.8.99.5
2.8.1.1
Taurine
Alkane-sulfonate
1.14.11.17
1.14.14.5
1.8.2.1
1.8.3.1
1.8.5.6
Sulfate
Sulfite
1.8.99.5
1.8.1.2
Asr
1.8.7.1
Sulfide
2.7.7.4
2.7.7.5
3.6.2.1
1.8.4.9
1.8.99.2
1.8.4.8
APS
2.7.1.25
3.1.3.7
PAPS
3.6.2.2
PAP
1.13.11.55
Sre
1.13.11.18
Sulfur
1.8.2.3
1.8.5.4
1.12.98.4
(Sulfide)n-1
(Sulfide)n
1.14.13.111
1.14.14.5
Methane-sulfonate
3-(Methylthio)-propanoyl-CoA
1.3.8.-
Methylthio-acryloyl-CoA
4.2.1.155
Aetaldehyde
1.8.3.4
Methyl-mercaptan
Methane metabolism
Methyl-CoM
2.1.1.251
6.2.1.44
1.14.14.35
2.1.1.269
DMSP
4.4.1.3
1.14.13.131
2.1.1.334
3-(Methylthio)-propanoate
Dimethylsulfone
1.8.1.17
DMSO
1.8.5.3
Methyl thioether
1.8.2.4
Glycine, serine and threonine metabolism
L-Serine
Methane metabolism
2.3.1.30
Acetate
Carbon fixation pathways in prokaryotes
O-Acetyl-L-serine
2.5.1.65
2.5.1.47
L-Cysteine
Cysteine and methionine metabolism
4.4.1.2
2.5.1.48
2.5.1.-
L-Homocysteine
O-Succinyl-L-homoserine
2.3.1.46
Succinate
L-Homoserine
Glycine, serine and threonine metabolism
Cysteine and methionine metabolism
Oxidation state +6 +4 +2 -2
Assimilatory sulfate reduction
Sulfate
PAPSS
Sat
CysND
APS
PAPSS
CysC
PAPS
CysH
Sulfite
CysJI
Sir
Sulfide
Dissimilatory sulfate reduction and oxidation
Sulfate
Sat
APS
AprAB
Sulfite
DsrAB
Sulfide
SOX system
Sulfate
SoxB
SoxY
SoxZ
SoxYZ-S-S-SO3
SoxA
SoxX
Thiosulfate
SoxYZ-S-SH
SoxC
SoxD
SoxYZ-S-S-SO3
SoxB
SoxYZ-SH
00920 5/19/17
(c) Kanehisa Laboratories

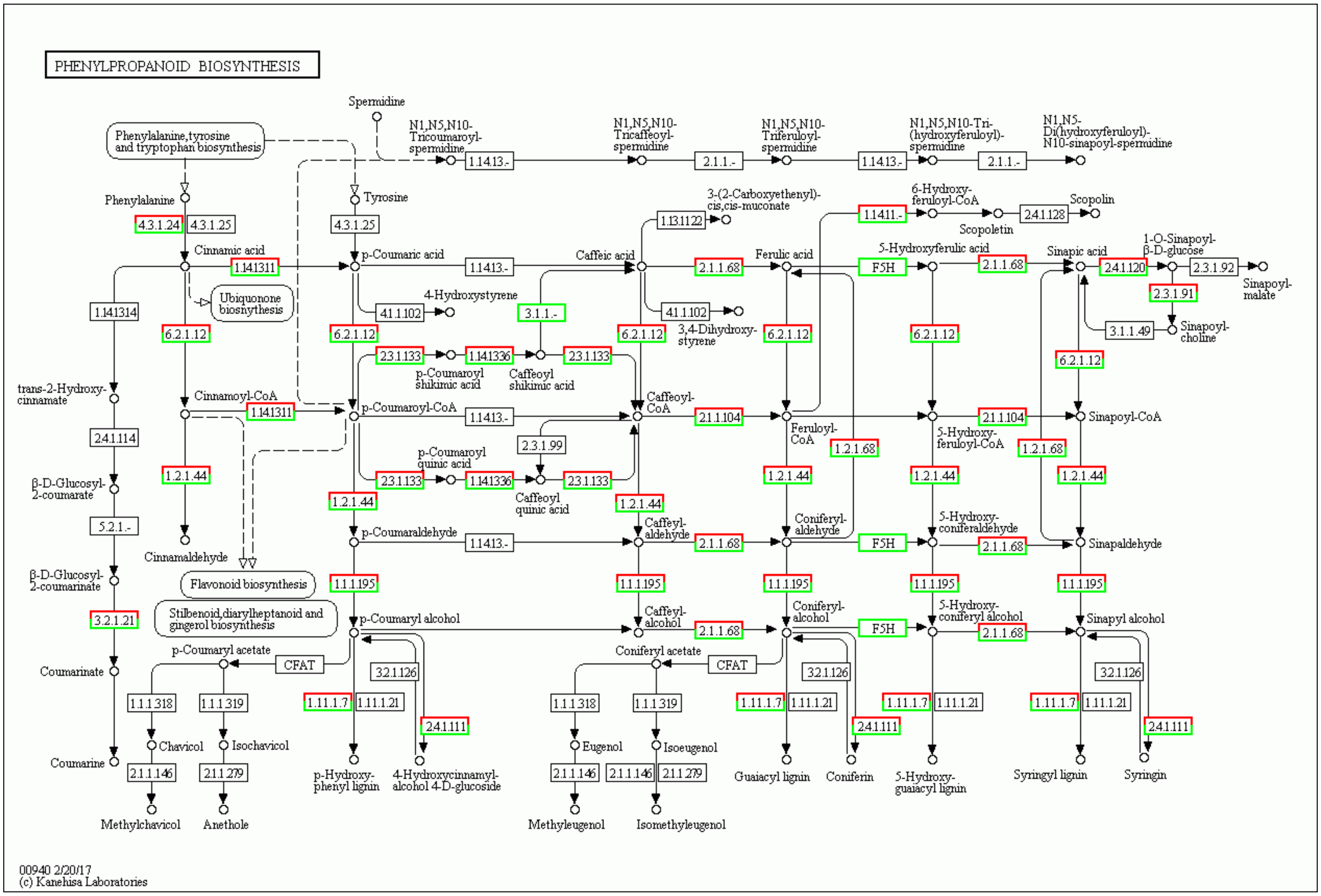
PHENYLPROPANOID BIOSYNTHESIS
Phenylalanine,tyrosine and tryptophan biosynthesis
Spermidine
N1,N5,N10-Tricoumaroyl-spermidine
N1,N5,N10-Tricaffeoyl-spermidine
N1,N5,N10-Triferuloyl-spermidine
N1,N5,N10-Tri-(hydroxyferuloyl)-spermidine
N1,N5-Di(hydroxyferuloyl)-N10-sinapoyl-spermidine
1.14.13.-
2.1.1.-
Phenylalanine
Tyrosine
4.3.1.24
4.3.1.25
3-(2-Carboxyethenyl)-cis,cis-muconate
1.13.11.22
6-Hydroxy-feruloyl-CoA
1.14.11.-
Scopoletin
2.4.1.128
Scopolin
Cinnamic acid
1.14.13.11
p-Coumaric acid
Caffeic acid
Ferulic acid
5-Hydroxyferulic acid
Sinapic acid
1-O-Sinapoyl-β-D-glucose
2.4.1.120
2.3.1.92
Sinapoyl-malate
2.3.1.91
3.1.1.49
Sinapoyl-choline
Ubiquinone biosynthesis
1.14.13.14
4-Hydroxystyrene
4.1.1.102
3.1.1.-
3,4-Dihydroxy-styrene
2.1.1.68
F5H
6.2.1.12
2.3.1.133
1.14.13.36
p-Coumaroyl shikimic acid
Caffeoyl shikimic acid
trans-2-Hydroxy-cinnamate
Cinnamoyl-CoA
p-Coumaroyl-CoA
Caffeoyl-CoA
Feruloyl-CoA
5-Hydroxy-feruloyl-CoA
Sinapoyl-CoA
2.1.1.104
2.4.1.114
2.3.1.99
p-Coumaroyl quinic acid
Caffeoyl quinic acid
β-D-Glucosyl-2-coumarinate
1.2.1.44
5.2.1.-
Cinnamaldehyde
p-Coumaraldehyde
Caffeyl-aldehyde
Coniferyl-aldehyde
5-Hydroxy-coniferaldehyde
Sinapaldehyde
β-D-Glucosyl-2-coumarinate
Flavonoid biosynthesis
Stilbenoid,diarylheptanoid and gingerol biosynthesis
1.1.1.195
3.2.1.21
p-Coumaryl alcohol
Caffeyl-alcohol
Coniferyl-alcohol
5-Hydroxy-coniferyl alcohol
Sinapyl alcohol
p-Coumaryl acetate
Coniferyl acetate
CFAT
Coumarinate
3.2.1.126
1.1.1.318
1.1.1.319
1.11.1.7
1.11.1.21
2.4.1.111
Chavicol
Isochavicol
Eugenol
Isoeugenol
Coumarine
2.1.1.146
2.1.1.279
p-Hydroxy-phenyl lignin
4-Hydroxycinnamyl-alcohol 4-D-glucoside
Guaiacyl lignin
Coniferin
5-Hydroxy-guaiacyl lignin
Syringyl lignin
Syringin
Methylchavicol
Anethole
Methyleugenol
Isomethyleugenol
00940 2/20/17
(c) Kanehisa Laboratories

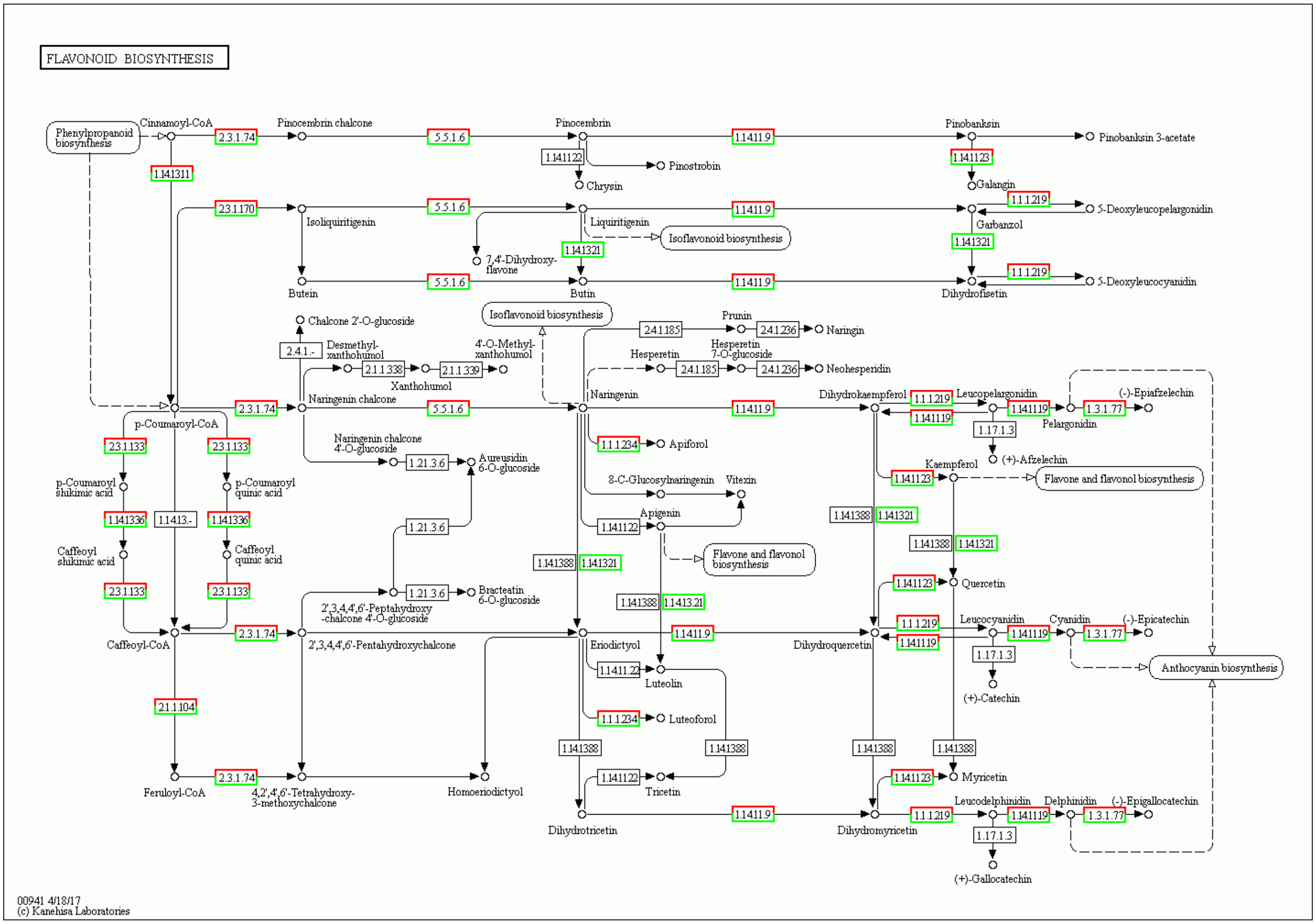
FLAVONOID BIOSYNTHESIS
Phenylpropanoid biosynthesis
Cinnamoyl-CoA
2.3.1.74
Pinocembrin chalcone
5.5.1.6
Pinocembrin
1.14.11.9
Pinobanksin
Pinobanksin 3-acetate
1.14.11.22
Pinostrobin
Chrysin
1.14.11.23
Galangin
1.14.13.11
2.3.1.170
Isoliquiritigenin
Liquiritigenin
1.1.1.219
5-Deoxyleucopelargonidin
Garbanzol
Isoflavonoid biosynthesis
7,4'-Dihydroxy-flavone
1.14.13.21
Butein
Butin
Dihydrofisetin
5-Deoxyleucocyanidin
Chalcone 2'-O-glucoside
2.4.1.-
Desmethyl-xanthohumol
2.1.1.338
Xanthohumol
2.1.1.339
4'-O-Methyl-xanthohumol
2.4.1.185
Prunin
2.4.1.236
Naringin
Hesperetin
Hesperetin 7-O-glucoside
Neohesperidin
Naringenin chalcone
Naringenin
Dihydrokaempferol
Leucopelargonidin
1.14.11.19
Pelargonidin
1.3.1.77
(-)-Epiafzelechin
1.17.1.3
(+)-Afzelechin
p-Coumaroyl-CoA
2.3.1.133
Naringenin chalcone 4'-O-glucoside
1.21.3.6
Aureusidin 6-O-glucoside
1.1.1.234
Apiforol
Kaempferol
Flavone and flavonol biosynthesis
p-Coumaroyl shikimic acid
p-Coumaroyl quinic acid
8-C-Glucosylnaringenin
Vitexin
1.14.13.36
1.14.13.-
Apigenin
1.14.13.88
Caffeoyl shikimic acid
Caffeoyl quinic acid
Flavone and flavonol biosynthesis
Quercetin
Bracteatin 6-O-glucoside
2',3,4,4',6'-Peptahydroxy-chalcone 4'-O-glucoside
Caffeoyl-CoA
2',3,4,4',6'-Pentahydroxychalcone
Eriodictyol
Dihydroquercetin
Leucocyanidin
Cyanidin
(-)-Epicatechin
Anthocyanin biosynthesis
Luteolin
(+)-Catechin
2.1.1.104
Luteoforol
Myricetin
Feruloyl-CoA
4,2',4',6'-Tetrahydroxy-3-methoxychalcone
Homoeriodictyol
Tricetin
Leucodelphinidin
Delphinidin
(-)-Epigallocatechin
Dihydrotricetin
Dihydromyricetin
(+)-Gallocatechin
00941 4/18/17
(c) Kanehisa Laboratories

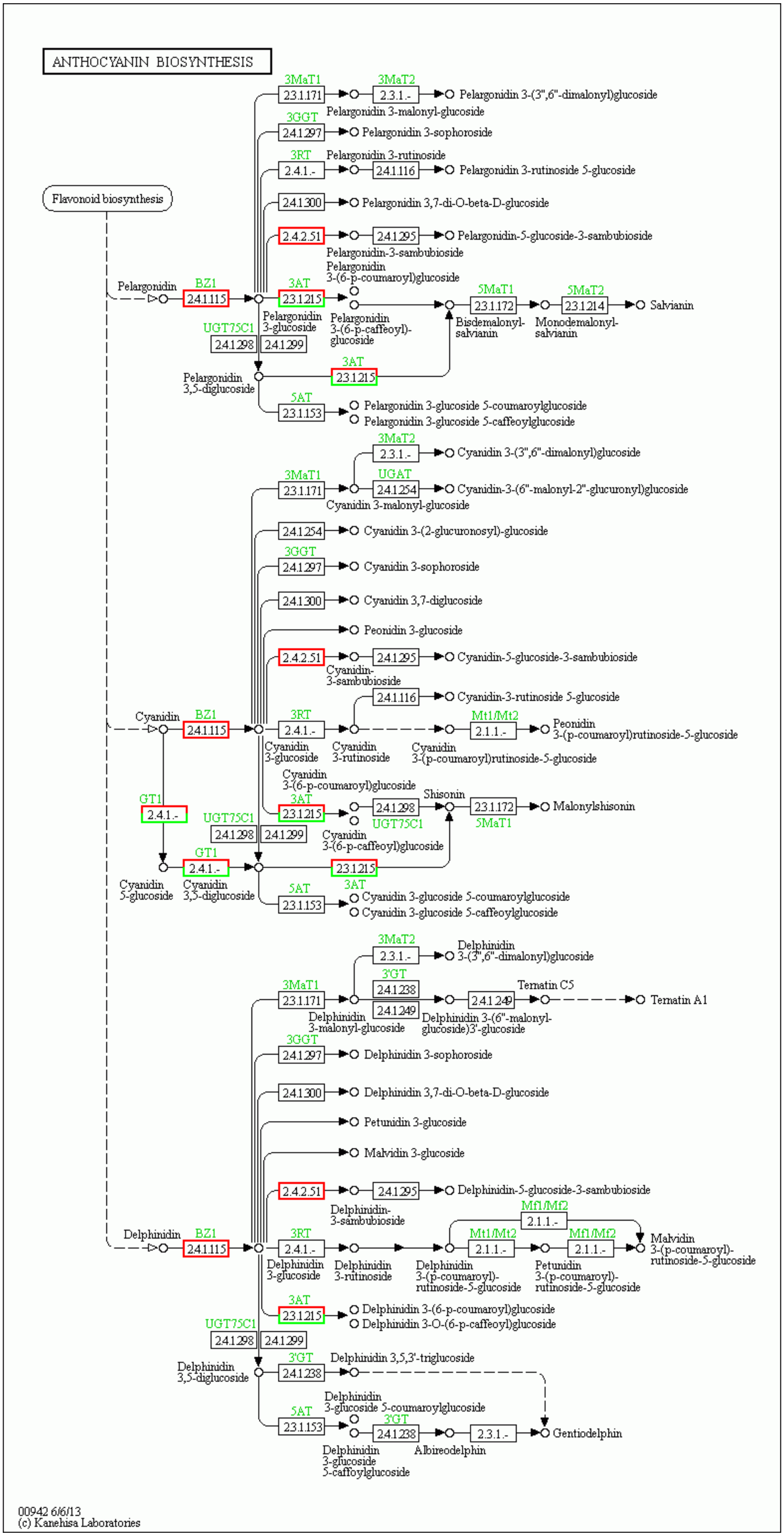
ANTHOCYANIN BIOSYNTHESIS
Flavonoid biosynthesis
Pelargonidin
BZ1
2.4.1.115
3MaT1
2.3.1.171
3MaT2
2.3.1.-
Pelargonidin 3-(3",6"-dimalonyl)glucoside
Pelargonidin 3-malonyl-glucoside
3GGT
2.4.1.297
Pelargonidin 3-sophoroside
3RT
2.4.1.-
Pelargonidin 3-rutinoside
2.4.1.116
Pelargonidin 3-rutinoside 5-glucoside
2.4.1.300
Pelargonidin 3,7-di-O-beta-D-glucoside
2.4.2.51
2.4.1.295
Pelargonidin-5-glucoside-3-sambubioside
Pelargonidin-3-sambubioside
Pelargonidin 3-(6-p-coumaroyl)glucoside
3AT
2.3.1.215
Pelargonidin 3-glucoside
Pelargonidin 3-(6-p-caffeoyl)-glucoside
5MaT1
2.3.1.172
5MaT2
2.3.1.214
Salvianin
Bisdemalonyl-salvianin
Monodemalonyl-salvianin
UGT75C1
2.4.1.298
2.4.1.299
Pelargonidin 3,5-diglucoside
5AT
2.3.1.153
Pelargonidin 3-glucoside 5-coumaroylglucoside
Pelargonidin 3-glucoside 5-caffeoylglucoside
Cyanidin 3-(3",6"-dimalonyl)glucoside
UGAT
2.4.1.254
Cyanidin-3-(6"-malonyl-2"-glucuronyl)glucoside
Cyanidin 3-malonyl-glucoside
Cyanidin 3-(2-glucuronosyl)-glucoside
Cyanidin 3-sophoroside
Cyanidin 3,7-diglucoside
Peonidin 3-glucoside
Cyanidin-5-glucoside-3-sambubioside
Cyanidin-3-sambubioside
Cyanidin-3-rutinoside 5-glucoside
Cyanidin
Mt1/Mt2
2.1.1.-
Peonidin 3-(p-coumaroyl)rutinoside-5-glucoside
Cyanidin 3-glucoside
Cyanidin 3-rutinoside
Cyanidin 3-(p-coumaroyl)rutinoside-5-glucoside
Cyanidin 3-(6-p-coumaroyl)glucoside
Shisonin
Malonylshisonin
GT1
Cyanidin 3-(6-p-caffeoyl)glucoside
Cyanidin 5-glucoside
Cyanidin 3,5-diglucoside
Cyanidin 3-glucoside 5-coumaroylglucoside
Cyanidin 3-glucoside 5-caffeoylglucoside
Delphinidin 3-(3",6"-dimalonyl)glucoside
3'GT
2.4.1.238
2.4.1.249
Ternatin C5
Ternatin A1
Delphinidin 3-malonyl-glucoside
Delphinidin 3-(6"-malonyl-glucoside)3'-glucoside
Delphinidin 3-sophoroside
Delphinidin 3,7-di-O-beta-D-glucoside
Petunidin 3-glucoside
Malvidin 3-glucoside
Delphinidin-5-glucoside-3-sambubioside
Delphinidin-3-sambubioside
Mf1/Mf2
Delphinidin
Malvidin 3-(p-coumaroyl)-rutinoside-5-glucoside
Delphinidin 3-glucoside
Delphinidin 3-rutinoside
Delphinidin 3-(p-coumaroyl)-rutinoside-5-glucoside
Petunidin 3-(p-coumaroyl)-rutinoside-5-glucoside
Delphinidin 3-(6-p-coumaroyl)glucoside
Delphinidin 3-O-(6-p-caffeoyl)glucoside
Delphinidin 3,5-diglucoside
Delphinidin 3,5,3'-triglucoside
Delphinidin 3-glucoside 5-coumaroylglucoside
Gentiodelphin
Delphinidin 3-glucoside 5-caffoylglucoside
Albireodelphin
00942 6/6/13
(c) Kanehisa Laboratories

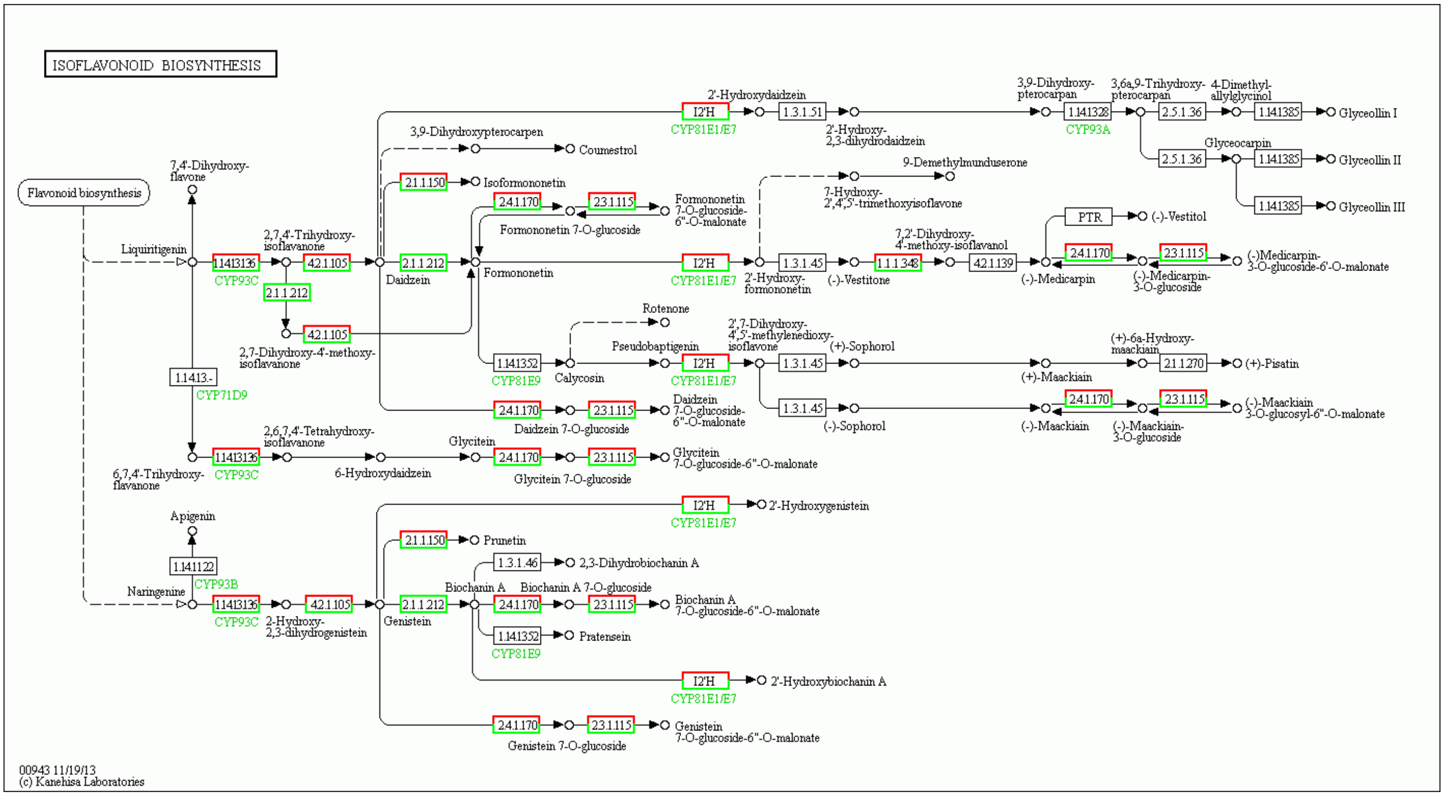
ISOFLAVONOID BIOSYNTHESIS
Flavonoid biosynthesis
7,4'-Dihydroxy-flavone
Liquiritigenin
1.14.13.136
CYP93C
2,7,4'-Trihydroxy-isoflavanone
4.2.1.105
2.1.1.212
2,7-Dihydroxy-4'-methoxy-isoflavanone
1.14.13.-
CYP71D9
Daidzein
3,9-Dihydroxypterocarpen
Coumestrol
2.1.1.150
Isoformononetin
2.4.1.170
2.3.1.115
Formononetin 7-O-glucoside
Formononetin 7-O-glucoside-6''-O-malonate
Formononetin
I2'H
CYP81E1/E7
2'-Hydroxydaidzein
1.3.1.51
2'-Hydroxy-2,3-dihydrodaidzein
3,9-Dihydroxy-pterocarpan
1.14.13.28
CYP93A
3,6a,9-Trihydroxy-pterocarpan
2.5.1.36
4-Dimethyl-allylglycinol
1.14.13.85
Glyceollin I
Glyceocarpin
Glyceollin II
Glyceollin III
9-Demethylmunduserone
7-Hydroxy-2',4',5'-trimethoxyisoflavone
PTR
(-)-Vestitol
7,2'-Dihydroxy-4'-methoxy-isoflavanol
1.3.1.45
(-)-Vestitone
1.1.1.348
4.2.1.139
2'-Hydroxy-formononetin
(-)-Medicarpin
(-)-Medicarpin-3-O-glucoside
(-)-Medicarpin-3-O-glucoside-6'-O-malonate
Rotenone
1.14.13.52
CYP81E9
Calycosin
Pseudobaptigenin
2',7-Dihydroxy-4',5'-methylenedioxy-isoflavone
(+)-Sophorol
(+)-Maackiain
(+)-6a-Hydroxy-maackiain
2.1.1.270
(+)-Pisatin
(-)-Sophorol
(-)-Maackiain
(-)-Maackiain-3-O-glucoside
(-)-Maackiain-3-O-glucosyl-6''-O-malonate
Daidzein 7-O-glucoside
Daidzein 7-O-glucoside-6''-O-malonate
6,7,4'-Trihydroxy-flavanone
2,6,7,4'-Tetrahydroxy-isoflavanone
6-Hydroxydaidzein
Glycitein
Glycitein 7-O-glucoside
Glycitein 7-O-glucoside-6''-O-malonate
2'-Hydroxygenistein
Apigenin
1.14.11.22
CYP93B
Naringenine
2-Hydroxy-2,3-dihydrogenistein
Prunetin
1.3.1.46
2,3-Dihydrobiochanin A
Genistein
Biochanin A
Biochanin A 7-O-glucoside
Biochanin A 7-O-glucoside-6''-O-malonate
Pratensein
2'-Hydroxybiochanin A
Genistein 7-O-glucoside
Genistein 7-O-glucoside-6''-O-malonate
00943 11/19/13
(c) Kanehisa Laboratories

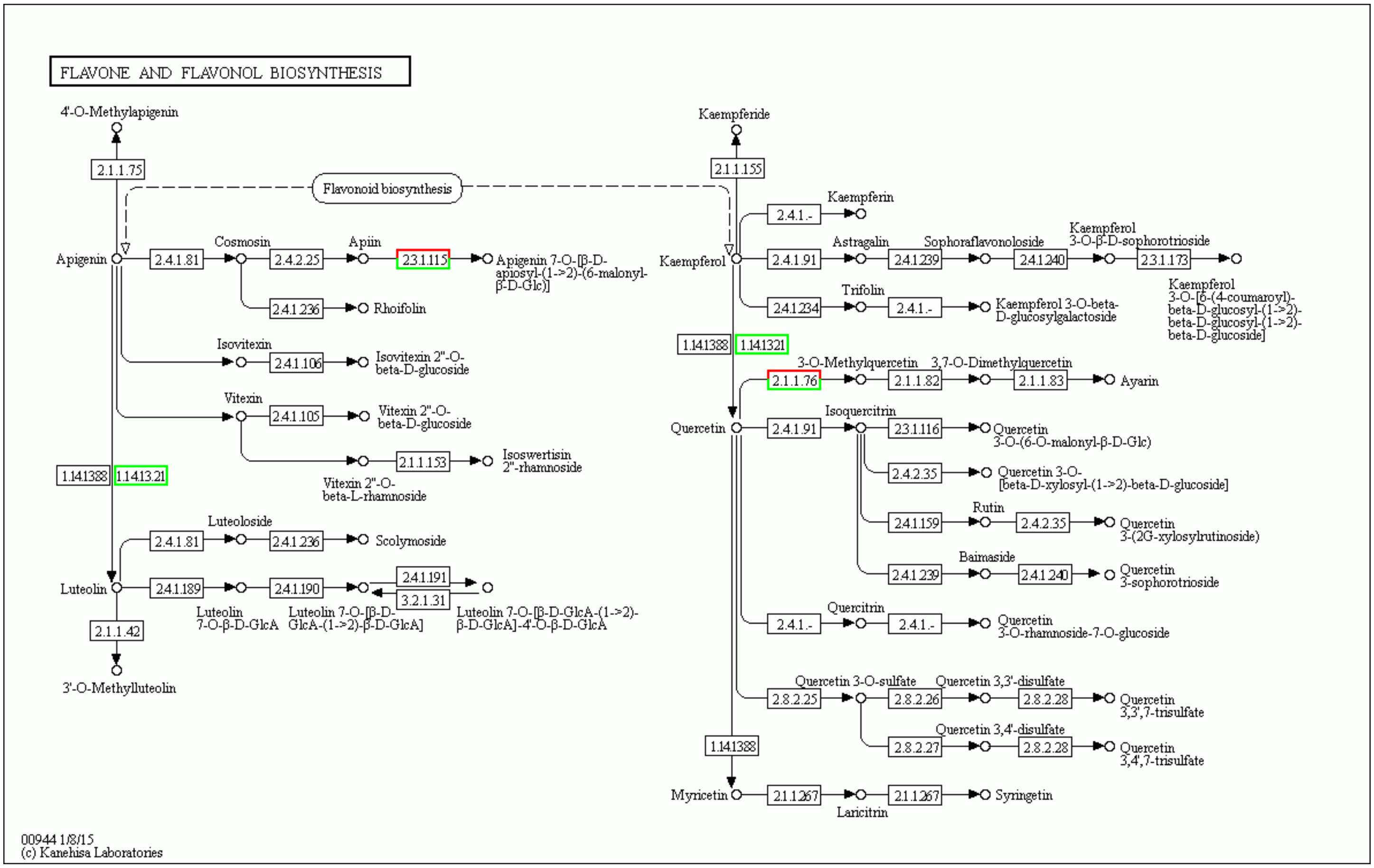
FLAVONE AND FLAVONOL BIOSYNTHESIS
4'-O-Methylapigenin
2.1.1.75
Flavonoid biosynthesis
Apigenin
2.4.1.81
Cosmosin
2.4.2.25
Apiin
2.3.1.115
Apigenin 7-O-[β-D-apiosyl-(1->2)-(6-malonyl-β-D-Glc)]
2.4.1.236
Rhoifolin
Isovitexin
2.4.1.106
Isovitexin 2''-O-beta-D-glucoside
Vitexin
2.4.1.105
Vitexin 2''-O-beta-D-glucoside
2.1.1.153
Isoswertisin 2''-rhamnoside
Vitexin 2''-O-beta-L-rhamnoside
1.14.13.88
1.14.13.21
Luteoloside
Scolymoside
Luteolin
2.4.1.189
2.4.1.190
2.4.1.191
3.2.1.31
Luteolin 7-O-β-D-GlcA
Luteolin 7-O-[β-D-GlcA-(1->2)-β-D-GlcA]
Luteolin 7-O-[β-D-GlcA-(1->2)-β-D-GlcA]-4'-O-β-D-GlcA
2.1.1.42
3'-O-Methylluteolin
Kaempferide
2.1.1.155
Kaempferin
2.4.1.-
Kaempferol
2.4.1.91
Astragalin
2.4.1.239
Sophoraflavonoloside
2.4.1.240
Kaempferol 3-O-β-D-sophorotrioside
2.3.1.173
Kaempferol 3-O-[6-(4-coumaroyl)-beta-D-glucosyl-(1->2)-beta-D-glucosyl-(1->2)-beta-D-glucoside]
2.4.1.234
Trifolin
Kaempferol 3-O-beta-D-glucosylgalactoside
3-O-Methylquercetin
2.1.1.76
2.1.1.82
3,7-O-Dimethylquercetin
2.1.1.83
Ayarin
Quercetin
Isoquercitrin
2.3.1.116
Quercetin 3-O-(6-O-malonyl-β-D-Glc)
2.4.2.35
Quercetin 3-O-[beta-D-xylosyl-(1->2)-beta-D-glucoside]
2.4.1.159
Rutin
Quercetin 3-(2G-xylosylrutinoside)
Baimaside
Quercetin 3-sophorotrioside
Quercitrin
Quercetin 3-O-rhamnoside-7-O-glucoside
Quercetin 3-O-sulfate
2.8.2.25
2.8.2.26
Quercetin 3,3'-disulfate
2.8.2.28
Quercetin 3,3',7-trisulfate
Quercetin 3,4'-disulfate
2.8.2.27
Quercetin 3,4',7-trisulfate
Myricetin
2.1.1.267
Laricitrin
Syringetin
00944 1/8/15
(c) Kanehisa Laboratories

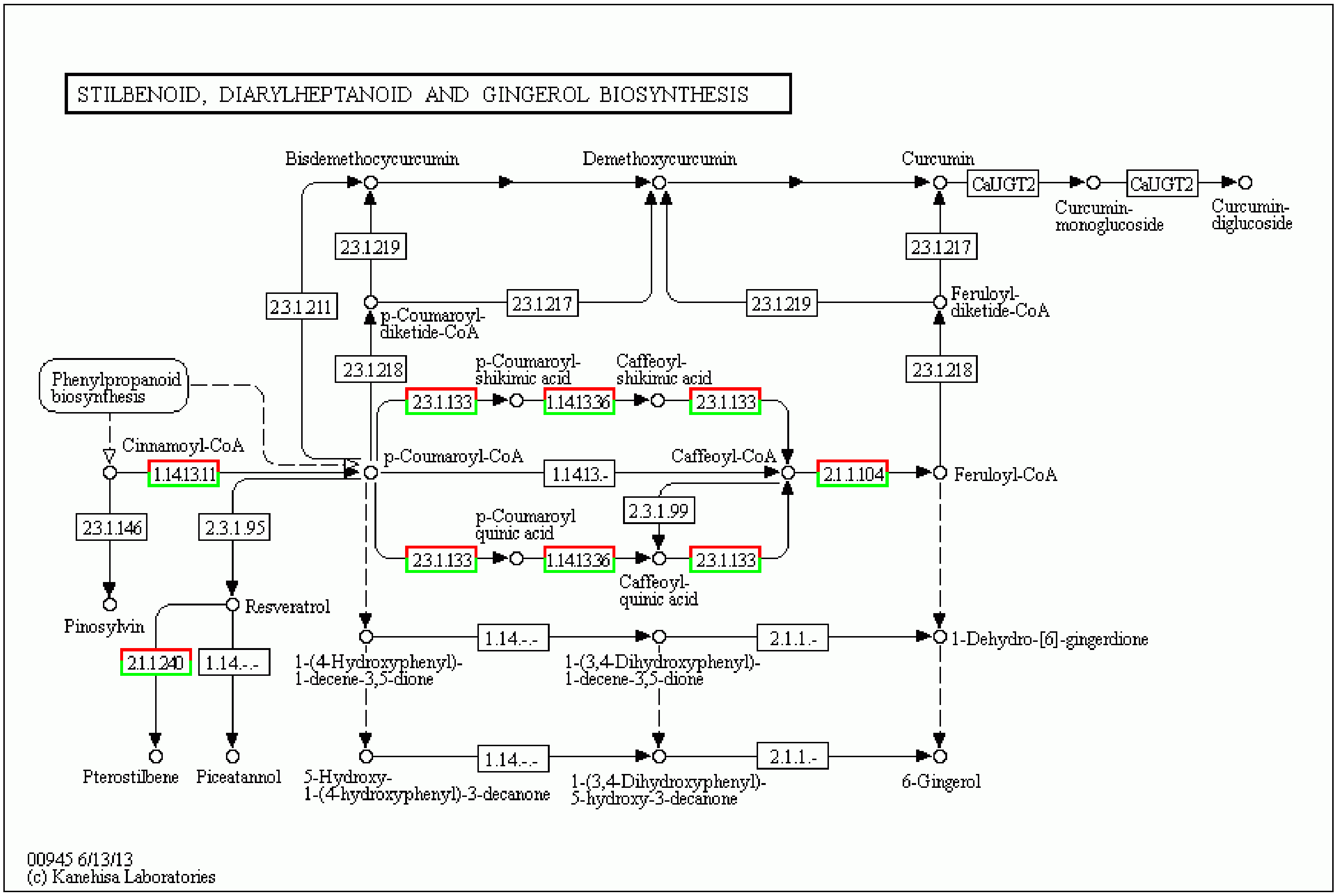
STILBENOID, DIARYLHEPTANOID AND GINGEROL BIOSYNTHESIS
Bisdemethocycurcumin
Demethoxycurcumin
Curcumin
CaUGT2
CaUGT2
Curcumin-monoglucoside
Curcumin-diglucoside
2.3.1.219
2.3.1.217
2.3.1.211
2.3.1.217
2.3.1.219
p-Coumaroyl-diketide-CoA
Feruloyl-diketide-CoA
2.3.1.218
2.3.1.218
Phenylpropanoid biosynthesis
p-Coumaroyl-shikimic acid
Caffeoyl-shikimic acid
2.3.1.133
1.14.13.36
2.3.1.133
Cinnamoyl-CoA
1.14.13.11
p-Coumaroyl-CoA
1.14.13.-
Caffeoyl-CoA
2.1.1.104
Feruloyl-CoA
2.3.1.146
2.3.1.95
2.3.1.99
p-Coumaroyl quinic acid
2.3.1.133
1.14.13.36
2.3.1.133
Caffeoyl-quinic acid
Pinosylvin
Resveratrol
2.1.1.240
1.14.-.-
1.14.-.-
2.1.1.-
1-(4-Hydroxyphenyl)-1-decene-3,5-dione
1-(3,4-Dihydroxyphenyl)-1-decene-3,5-dione
1-Dehydro-[6]-gingerdione
1.14.-.-
2.1.1.-
Pterostilbene
Piceatannol
5-Hydroxy-1-(4-hydroxyphenyl)-3-decanone
1-(3,4-Dihydroxyphenyl)-5-hydroxy-3-decanone
6-Gingerol
00945 6/13/13
(c) Kanehisa Laboratories

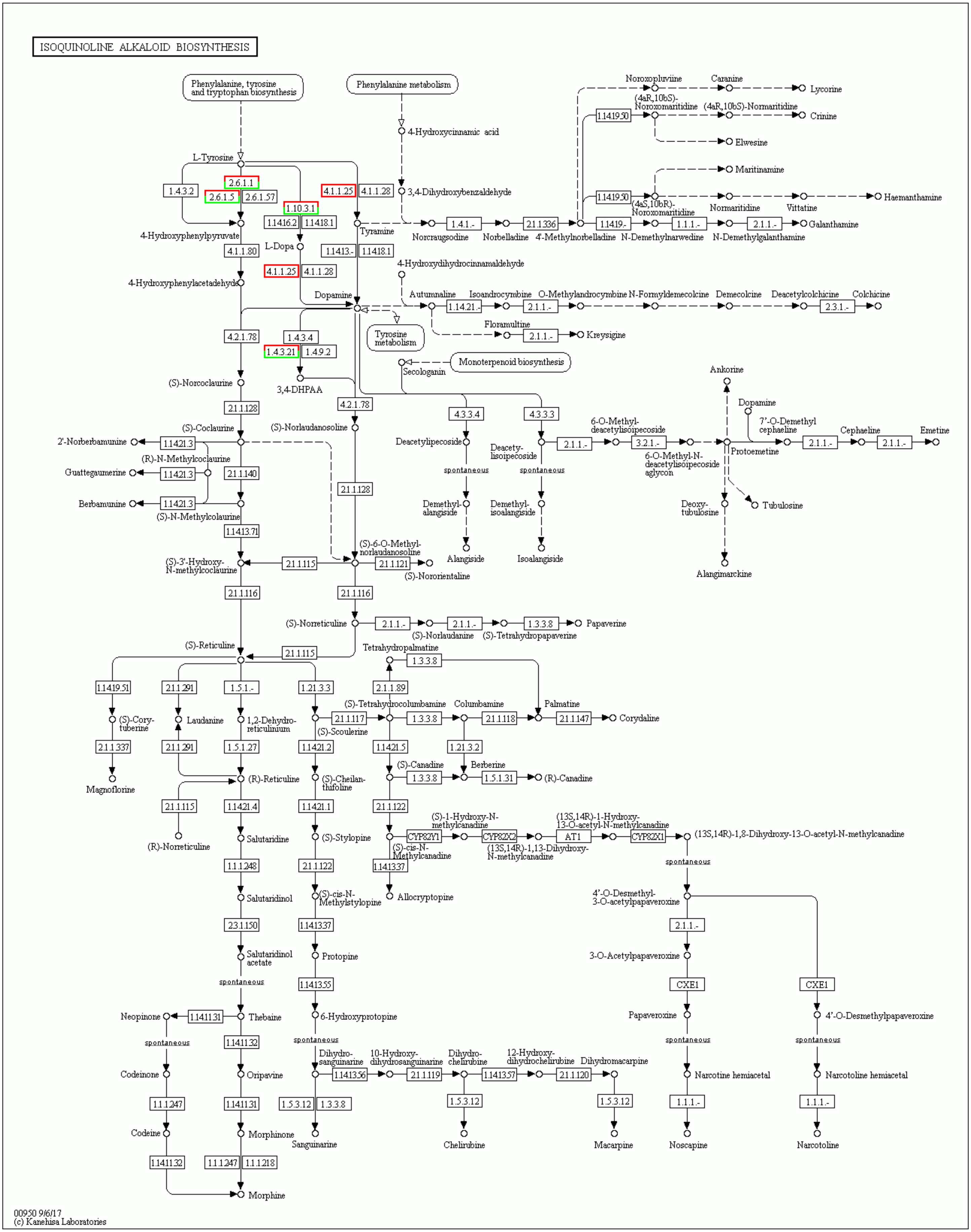

ISOQUINOLINE ALKALOID BIOSYNTHESIS
Phenylalanine, tyrosine and tryptophan biosynthesis
Phenylalanine metabolism
4-Hydroxycinnamic acid
3,4-Dihydroxybenzaldehyde
L-Tyrosine
2.6.1.1
2.6.1.5
2.6.1.57
1.4.3.2
1.10.3.1
1.14.16.2
1.14.18.1
4.1.1.25
4.1.1.28
4-Hydroxyphenylpyruvate
L-Dopa
Tyramine
1.14.13.-
1.14.18.1
4.1.1.80
4-Hydroxyphenylacetaldehyde
4-Hydroxydihydrocinnamaldehyde
Dopamine
Tyrosine metabolism
Monoterpenoid biosynthesis
Secologanin
4.2.1.78
1.4.3.4
1.4.3.21
1.4.9.2
3,4-DHPAA
(S)-Norcoclaurine
2.1.1.128
(S)-Norlaudanosoline
(S)-Coclaurine
2'-Norberbamunine
1.14.21.3
(R)-N-Methylcoclaurine
Guattegaumerine
2.1.1.140
Berbamunine
(S)-N-Methylcoclaurine
1.14.13.71
(S)-3'-Hydroxy-N-methylcoclaurine
2.1.1.115
(S)-6-O-Methyl-norlaudanosoline
2.1.1.121
(S)-Nororientaline
2.1.1.116
(S)-Norreticuline
2.1.1.-
(S)-Norlaudanine
(S)-Tetrahydropapaverine
1.3.3.8
Papaverine
(S)-Reticuline
Tetrahydropalmatine
1.14.19.51
2.1.1.291
1.5.1.-
1.21.3.3
2.1.1.89
(S)-Cory-tuberine
Laudanine
1,2-Dehydro-reticulinium
(S)-Tetrahydrocolumbamine
Columbamine
Palmatine
2.1.1.117
2.1.1.118
2.1.1.147
Corydaline
(S)-Scoulerine
2.1.1.337
1.5.1.27
1.14.21.2
1.14.21.5
1.21.3.2
Magnoflorine
(R)-Reticuline
(S)-Cheilan-thifoline
(S)-Canadine
Berberine
1.5.1.31
(R)-Canadine
2.1.1.115
1.14.21.4
1.14.21.1
2.1.1.122
(R)-Norreticuline
Salutaridine
(S)-Stylopine
(S)-1-Hydroxy-N-methylcanadine
CYP82Y1
CYP82X2
AT1
CYP82X1
(13S,14R)-1-Hydroxy-13-O-acetyl-N-methylcanadine
(13S,14R)-1,8-Dihydroxy-13-O-acetyl-N-methylcanadine
(S)-cis-N-Methylcanadine
(13S,14R)-1,13-Dihydroxy-N-methylcanadine
1.1.1.248
1.14.13.37
Allocryptopine
Salutaridinol
(S)-cis-N-Methylstylopine
spontaneous
4'-O-Desmethyl-3-O-acetylpapaveroxine
2.3.1.150
1.14.13.55
Salutaridinol acetate
Protopine
3-O-Acetylpapaveroxine
CXE1
Thebaine
Neopinone
1.14.11.31
1.14.11.32
6-Hydroxyprotopine
Papaveroxine
4'-O-Desmethylpapaveroxine
Dihydro-sanguinarine
10-Hydroxy-dihydrosanguinarine
Dihydro-chelirubine
12-Hydroxy-dihydrochelirubine
Dihydromacarpine
1.14.13.56
2.1.1.119
1.14.13.57
2.1.1.120
Codeinone
Oripavine
Narcotine hemiacetal
Narcotoline hemiacetal
1.1.1.247
1.5.3.12
1.1.1.-
Codeine
Morphinone
Sanguinarine
Chelirubine
Macarpine
Noscapine
Narcotoline
1.1.1.218
Morphine
Noroxopluviine
Caranine
Lycorine
(4aR,10bS)-Noroxomaritidine
(4aS,10bS)-Normaritidine
Crinine
1.14.19.50
Elwesine
Maritinamine
(4aS,10bR)-Noroxomaritidine
Normaritidine
Vittatine
Haemanthamine
1.4.1.-
2.1.1.336
1.14.19.-
Norcraugsodine
Norbelladine
4'-Methylnorbelladine
N-Demethylnarwedine
N-Demethylgalanthamine
Galanthamine
Autumnaline
Isoandrocymbine
O-Methylandrocymbine
N-Formyldemecolcine
Demecolcine
Deacetylcolchicine
Colchicine
1.14.21.-
2.3.1.-
Floramultine
Kreysigine
4.3.3.4
4.3.3.3
Deacetylipecoside
Deacetyl-isoipecoside
6-O-Methyl-deacetylisoipecoside
3.2.1.-
6-O-Methyl-N-deacetylisoipecoside aglycon
Ankorine
Dopamine
7'-O-Demethyl cephaeline
Cephaeline
Emetine
Protoemetine
Demethyl-alangiside
Demethyl-isoalangiside
Deoxy-tubulosine
Tubulosine
Alangiside
Isoalangiside
Alangimarckine
00950 9/6/17
(c) Kanehisa Laboratories

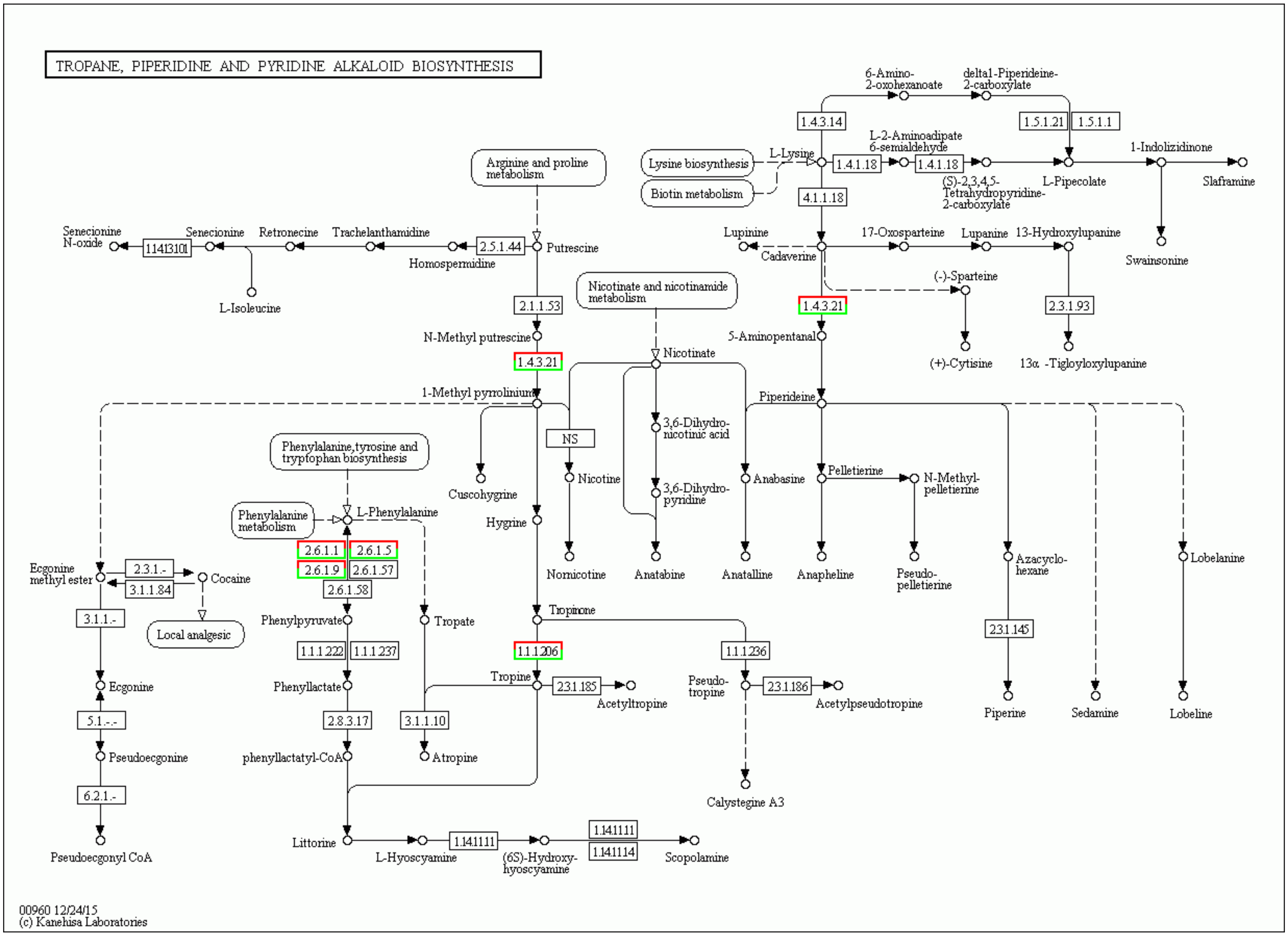
TROPANE, PIPERIDINE AND PYRIDINE ALKALOID BIOSYNTHESIS
6-Amino-
2-oxohexanoate
delta1-Piperideine-
2-carboxylate
1.4.3.14
1.5.1.21
1.5.1.1
L-2-Aminoadipate
6-semialdehyde
L-Lysine
1.4.1.18
1.4.1.18
1-Indolizidinone
Slaframine
Arginine and proline
metabolism
Lysine biosynthesis
Biotin metabolism
4.1.1.18
(S)-2,3,4,5-
Tetrahydropyridine-
2-carboxylate
L-Pipecolate
Senecionine
N-oxide
1.14.13.101
Senecionine
Retronecine
Trachelanthamidine
2.5.1.44
Putrescine
Homospermidine
Lupinine
Cadaverine
17-Oxosparteine
Lupanine
13-Hydroxylupanine
Swainsonine
L-Isoleucine
Nicotinate and nicotinamide
metabolism
(-)-Sparteine
2.1.1.53
1.4.3.21
2.3.1.93
N-Methyl putrescine
5-Aminopentanal
(+)-Cytisine
13α -Tigloyloxylupanine
1.4.3.21
Nicotinate
1-Methyl pyrrolinium
Piperideine
3,6-Dihydro-
nicotinic acid
NS
Phenylalanine,tyrosine and
tryptophan biosynthesis
Cuscohygrine
Nicotine
Anabasine
Pelletierine
N-Methyl-
pelletierine
3,6-Dihydro-
pyridine
Phenylalanine
metabolism
L-Phenylalanine
Hygrine
2.6.1.1
2.6.1.5
2.6.1.9
2.6.1.57
Ecgonine
methyl ester
2.3.1.-
3.1.1.84
Cocaine
2.6.1.58
Nornicotine
Anatabine
Anatalline
Anapheline
Pseudo-
pelletierine
Azacyclo-
hexane
Lobelanine
3.1.1.-
Local analgesic
Phenylpyruvate
Tropate
Tropinone
2.3.1.145
1.1.1.222
1.1.1.237
1.1.1.206
1.1.1.236
Ecgonine
Phenyllactate
Tropine
2.3.1.185
Acetyltropine
Pseudo-
tropine
2.3.1.186
Acetylpseudotropine
Piperine
Sedamine
Lobeline
5.1.-.-
2.8.3.17
3.1.1.10
Pseudoecgonine
phenyllactatyl-CoA
Atropine
6.2.1.-
Calystegine A3
1.14.11.11
Littorine
1.14.11.11
1.14.11.14
Pseudoecgonyl CoA
L-Hyoscyamine
(6S)-Hydroxy-
hyoscyamine
Scopolamine
00960 12/24/15
(c) Kanehisa Laboratories

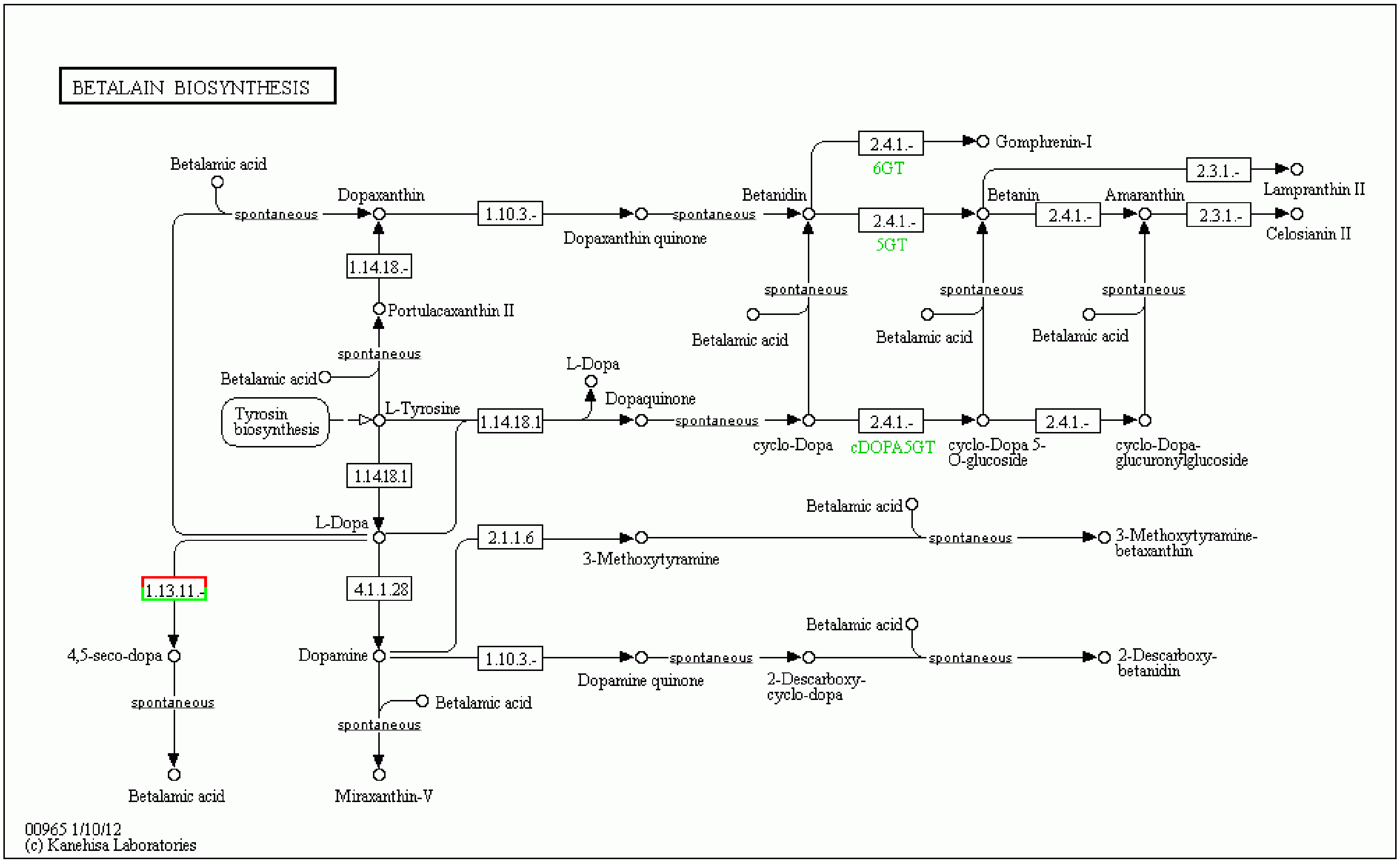
BETALAIN BIOSYNTHESIS
Betalamic acid
spontaneous
Dopaxanthin
1.10.3.-
Dopaxanthin quinone
Betanidin
2.4.1.-
6GT
Gomphrenin-I
2.4.1.-
5GT
Betanin
2.4.1.-
Amaranthin
2.3.1.-
Lampranthin II
2.3.1.-
Celosianin II
1.14.18.-
Portulacaxanthin II
Tyrosin biosynthesis
L-Tyrosine
1.14.18.1
L-Dopa
Dopaquinone
cyclo-Dopa
cDOPA5GT
cyclo-Dopa 5-O-glucoside
cyclo-Dopa-glucuronylglucoside
1.14.18.1
L-Dopa
2.1.1.6
3-Methoxytyramine
3-Methoxytyramine-betaxanthin
1.13.11.-
4.1.1.28
4,5-seco-dopa
Dopamine
Dopamine quinone
2-Descarboxy-cyclo-dopa
2-Descarboxy-betanidin
Miraxanthin-V
00965 1/10/12
(c) Kanehisa Laboratories

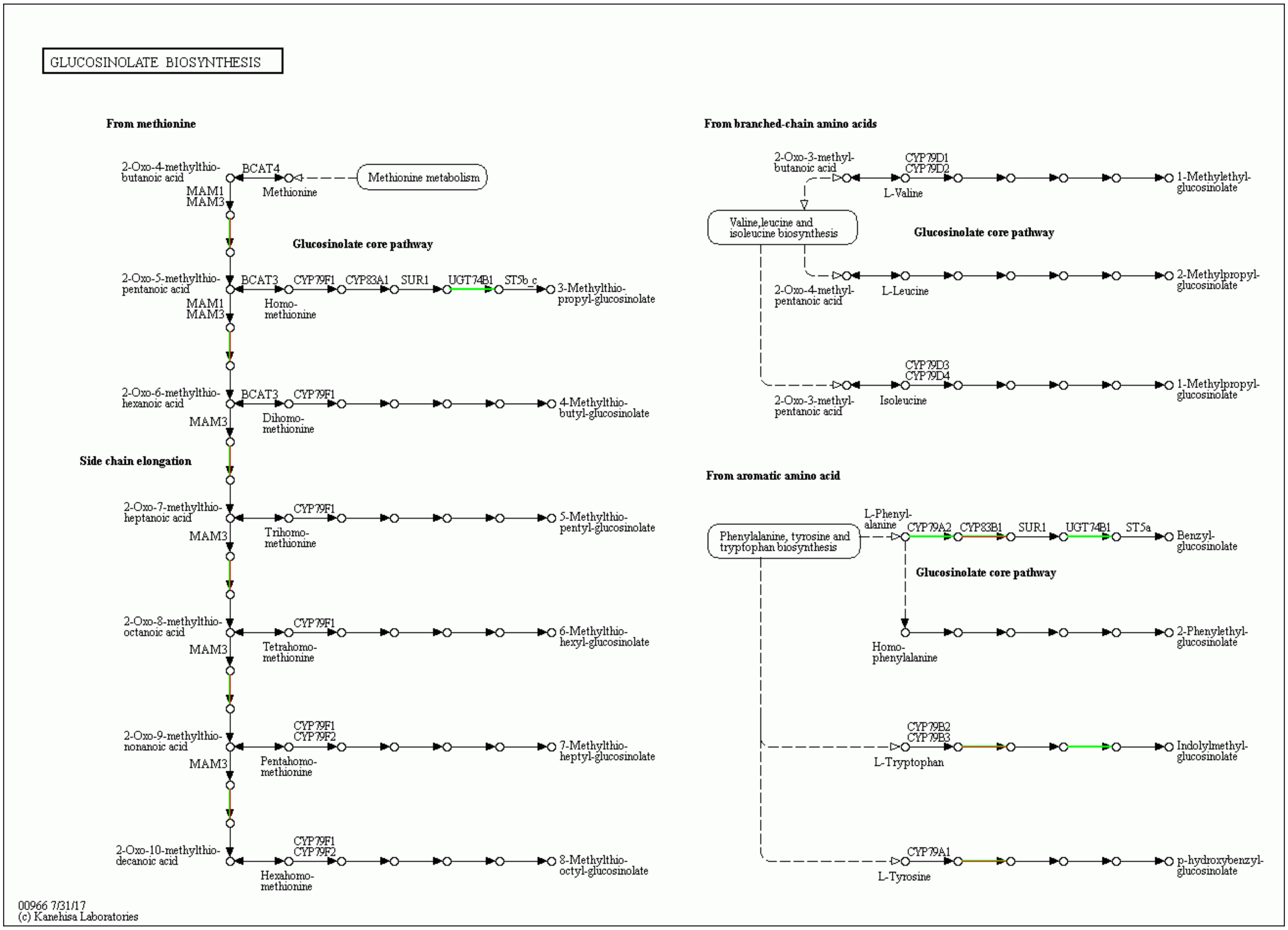

GLUCOSINOLATE BIOSYNTHESIS
From methionine
2-Oxo-4-methylthio-butanoic acid
BCAT4
Methionine
Methionine metabolism
MAM1
MAM3
Glucosinolate core pathway
2-Oxo-5-methylthio-pentanoic acid
BCAT3
CYP79F1
CYP83A1
SUR1
UGT74B1
ST5b_c
3-Methylthio-propyl-glucosinolate
Homo-methionine
2-Oxo-6-methylthio-hexanoic acid
4-Methylthio-butyl-glucosinolate
Dihomo-methionine
Side chain elongation
2-Oxo-7-methylthio-heptanoic acid
5-Methylthio-pentyl-glucosinolate
Trihomo-methionine
2-Oxo-8-methylthio-octanoic acid
6-Methylthio-hexyl-glucosinolate
Tetrahomo-methionine
2-Oxo-9-methylthio-nonanoic acid
CYP79F2
7-Methylthio-heptyl-glucosinolate
Pentahomo-methionine
2-Oxo-10-methylthio-decanoic acid
8-Methylthio-octyl-glucosinolate
Hexahomo-methionine
From branched-chain amino acids
2-Oxo-3-methyl-butanoic acid
CYP79D1
CYP79D2
L-Valine
1-Methylethyl-glucosinolate
Valine, leucine and isoleucine biosynthesis
2-Oxo-4-methyl-pentanoic acid
L-Leucine
2-Methylpropyl-glucosinolate
CYP79D3
CYP79D4
2-Oxo-3-methyl-pentanoic acid
Isoleucine
1-Methylpropyl-glucosinolate
From aromatic amino acid
Phenylalanine, tyrosine and tryptophan biosynthesis
L-Phenyl-alanine
CYP79A2
CYP83B1
ST5a
Benzyl-glucosinolate
Homo-phenylalanine
2-Phenylethyl-glucosinolate
CYP79B2
CYP79B3
L-Tryptophan
Indolylmethyl-glucosinolate
CYP79A1
L-Tyrosine
p-hydroxybenzyl-glucosinolate
00966 7/31/17
(c) Kanehisa Laboratories

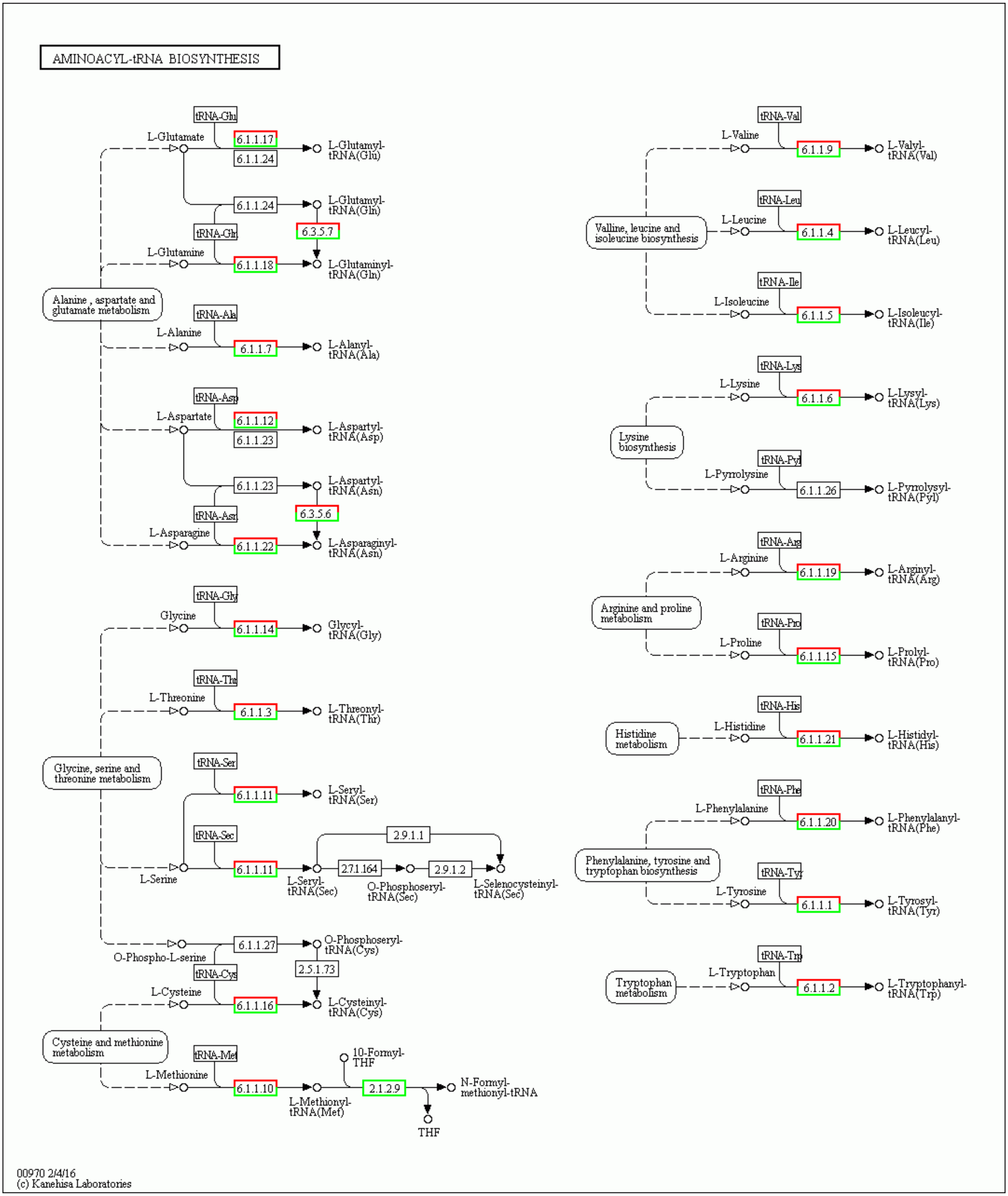
AMINOACYL-tRNA BIOSYNTHESIS
tRNA-Glu
L-Glutamate
6.1.1.17
6.1.1.24
L-Glutamyl-
tRNA(Glu)
6.1.1.24
L-Glutamyl-
tRNA(Gln)
6.3.5.7
tRNA-Gln
L-Glutamine
6.1.1.18
L-Glutaminyl-
tRNA(Gln)
Alanine , aspartate and
glutamate metabolism
tRNA-Ala
L-Alanine
6.1.1.7
L-Alanyl-
tRNA(Ala)
tRNA-Asp
L-Aspartate
6.1.1.12
6.1.1.23
L-Aspartyl-
tRNA(Asp)
6.1.1.23
L-Aspartyl-
tRNA(Asn)
6.3.5.6
tRNA-Asn
L-Asparagine
6.1.1.22
L-Asparaginyl-
tRNA(Asn)
tRNA-Gly
Glycine
6.1.1.14
Glycyl-
tRNA(Gly)
tRNA-Thr
L-Threonine
6.1.1.3
L-Threonyl-
tRNA(Thr)
Glycine, serine and
threonine metabolism
tRNA-Ser
6.1.1.11
L-Seryl-
tRNA(Ser)
tRNA-Sec
2.9.1.1
L-Serine
6.1.1.11
L-Seryl-
tRNA(Sec)
2.7.1.164
O-Phosphoseryl-
tRNA(Sec)
2.9.1.2
L-Selenocysteinyl-
tRNA(Sec)
O-Phospho-L-serine
6.1.1.27
O-Phosphoseryl-
tRNA(Cys)
2.5.1.73
tRNA-Cys
L-Cysteine
6.1.1.16
L-Cysteinyl-
tRNA(Cys)
Cysteine and methionine
metabolism
tRNA-Met
L-Methionine
6.1.1.10
L-Methionyl-
tRNA(Met)
10-Formyl-
THF
2.1.2.9
N-Formyl-
methionyl-tRNA
THF
tRNA-Val
L-Valine
6.1.1.9
L-Valyl-
tRNA(Val)
Valline, leucine and
isoleucine biosynthesis
tRNA-Leu
L-Leucine
6.1.1.4
L-Leucyl-
tRNA(Leu)
tRNA-Ile
L-Isoleucine
6.1.1.5
L-Isoleucyl-
tRNA(Ile)
tRNA-Lys
L-Lysine
6.1.1.6
L-Lysyl-
tRNA(Lys)
Lysine
biosynthesis
tRNA-Pyl
L-Pyrrolysine
6.1.1.26
L-Pyrrolysyl-
tRNA(Pyl)
tRNA-Arg
L-Arginine
6.1.1.19
L-Arginyl-
tRNA(Arg)
Arginine and proline
metabolism
tRNA-Pro
L-Proline
6.1.1.15
L-Prolyl-
tRNA(Pro)
tRNA-His
Histidine
metabolism
L-Histidine
6.1.1.21
L-Histidyl-
tRNA(His)
tRNA-Phe
L-Phenylalanine
6.1.1.20
L-Phenylalanyl-
tRNA(Phe)
Phenylalanine, tyrosine and
tryptophan biosynthesis
tRNA-Tyr
L-Tyrosine
6.1.1.1
L-Tyrosyl-
tRNA(Tyr)
tRNA-Trp
Tryptophan
metabolism
L-Tryptophan
6.1.1.2
L-Tryptophanyl-
tRNA(Trp)
00970 2/4/16
(c) Kanehisa Laboratories

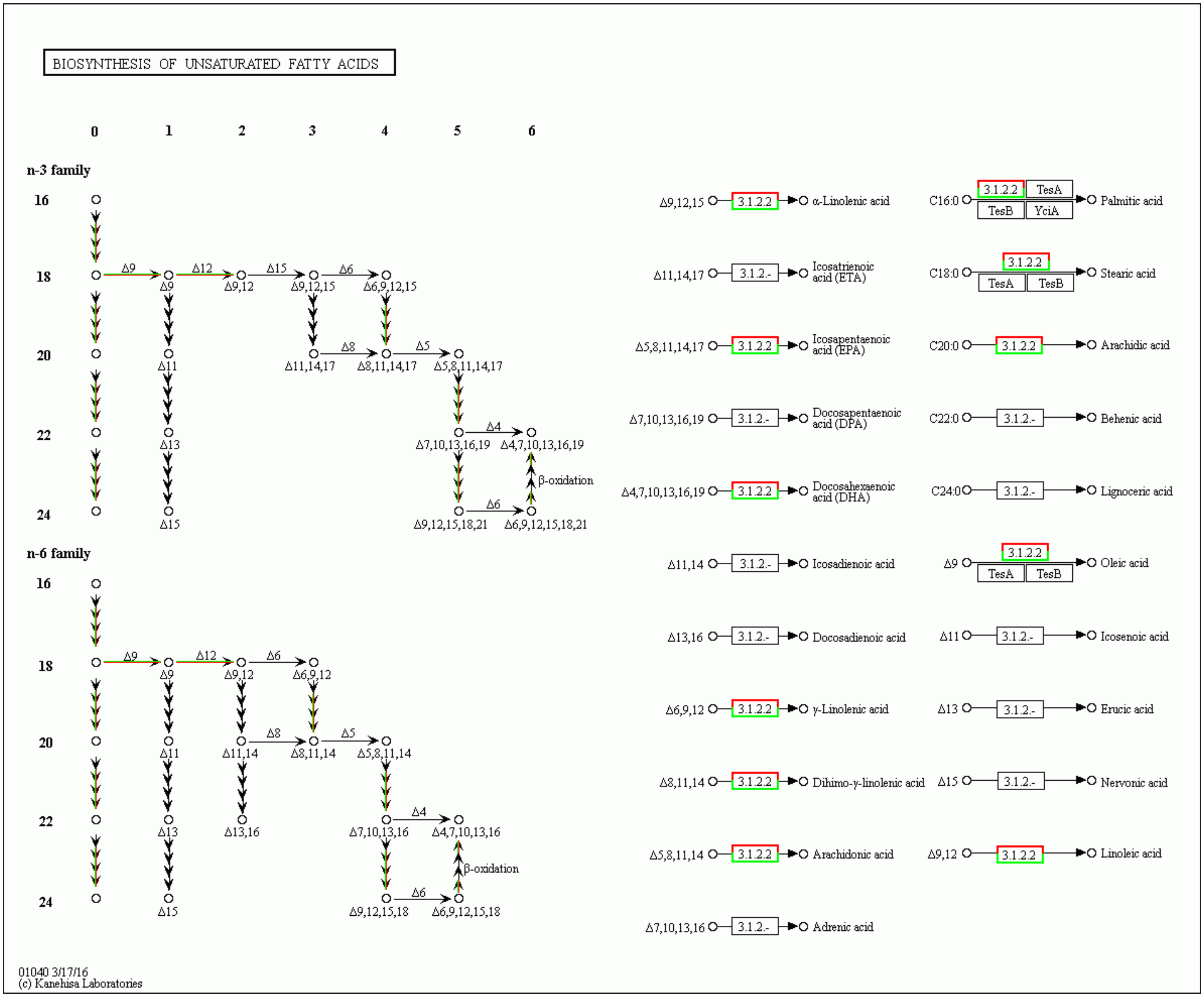

BIOSYNTHESIS OF UNSATURATED FATTY ACIDS
0 1 2 3 4 5 6
n-3 family
16
18
20
22
24
Δ9 Δ12 Δ15 Δ6
Δ9 Δ9,12 Δ9,12,15 Δ6,9,12,15
Δ8 Δ5
Δ11 Δ11,14,17 Δ8,11,14,17 Δ5,8,11,14,17
Δ4
Δ13 Δ7,10,13,16,19 Δ4,7,10,13,16,19
β-oxidation
Δ6
Δ15 Δ9,12,15,18,21 Δ6,9,12,15,18,21
n-6 family
16
18
20
22
24
Δ9 Δ12 Δ6
Δ9 Δ9,12 Δ6,9,12
Δ8 Δ5
Δ11 Δ11,14 Δ8,11,14 Δ5,8,11,14
Δ4
Δ13 Δ13,16 Δ7,10,13,16 Δ4,7,10,13,16
β-oxidation
Δ6
Δ15 Δ9,12,15,18 Δ6,9,12,15,18
Δ9,12,15 3.1.2.2 α-Linolenic acid
Δ11,14,17 3.1.2.- Icosatrienoic acid (ETA)
Δ5,8,11,14,17 3.1.2.2 Icosapentaenoic acid (EPA)
Δ7,10,13,16,19 3.1.2.- Docosapentaenoic acid (DPA)
Δ4,7,10,13,16,19 3.1.2.2 Docosahexaenoic acid (DHA)
Δ11,14 3.1.2.- Icosadienoic acid
Δ13,16 3.1.2.- Docosadienoic acid
Δ6,9,12 3.1.2.2 γ-Linolenic acid
Δ8,11,14 3.1.2.2 Dihimo-γ-linolenic acid
Δ5,8,11,14 3.1.2.2 Arachidonic acid
Δ7,10,13,16 3.1.2.- Adrenic acid
C16:0 3.1.2.2 TesA TesB YciA Palmitic acid
C18:0 3.1.2.2 TesA TesB Stearic acid
C20:0 3.1.2.2 Arachidic acid
C22:0 3.1.2.- Behenic acid
C24:0 3.1.2.- Lignoceric acid
Δ9 3.1.2.2 TesA TesB Oleic acid
Δ11 3.1.2.- Icosenoic acid
Δ13 3.1.2.- Erucic acid
Δ15 3.1.2.- Nervonic acid
Δ9,12 3.1.2.2 Linoleic acid
01040 3/17/16
(c) Kanehisa Laboratories

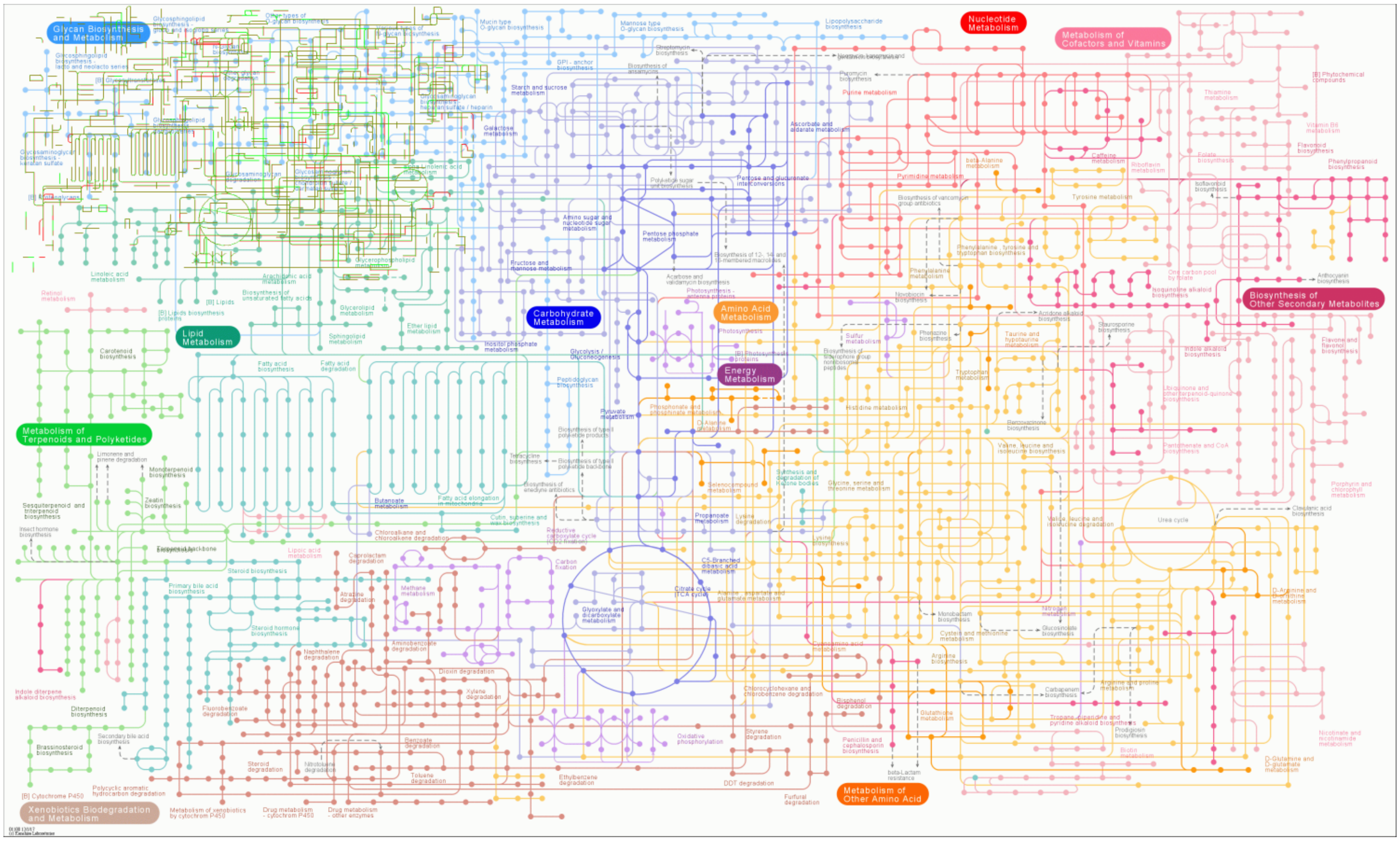
Glycan Biosynthesis and Metabolism
Nucleotide Metabolism
Metabolism of Cofactors and Vitamins
Biosynthesis of Other Secondary Metabolites
Lipid Metabolism
Carbohydrate Metabolism
Amino Acid Metabolism
Energy Metabolism
Metabolism of Terpenoids and Polyketides
Xenobiotics Biodegradation and Metabolism
Metabolism of Other Amino Acid

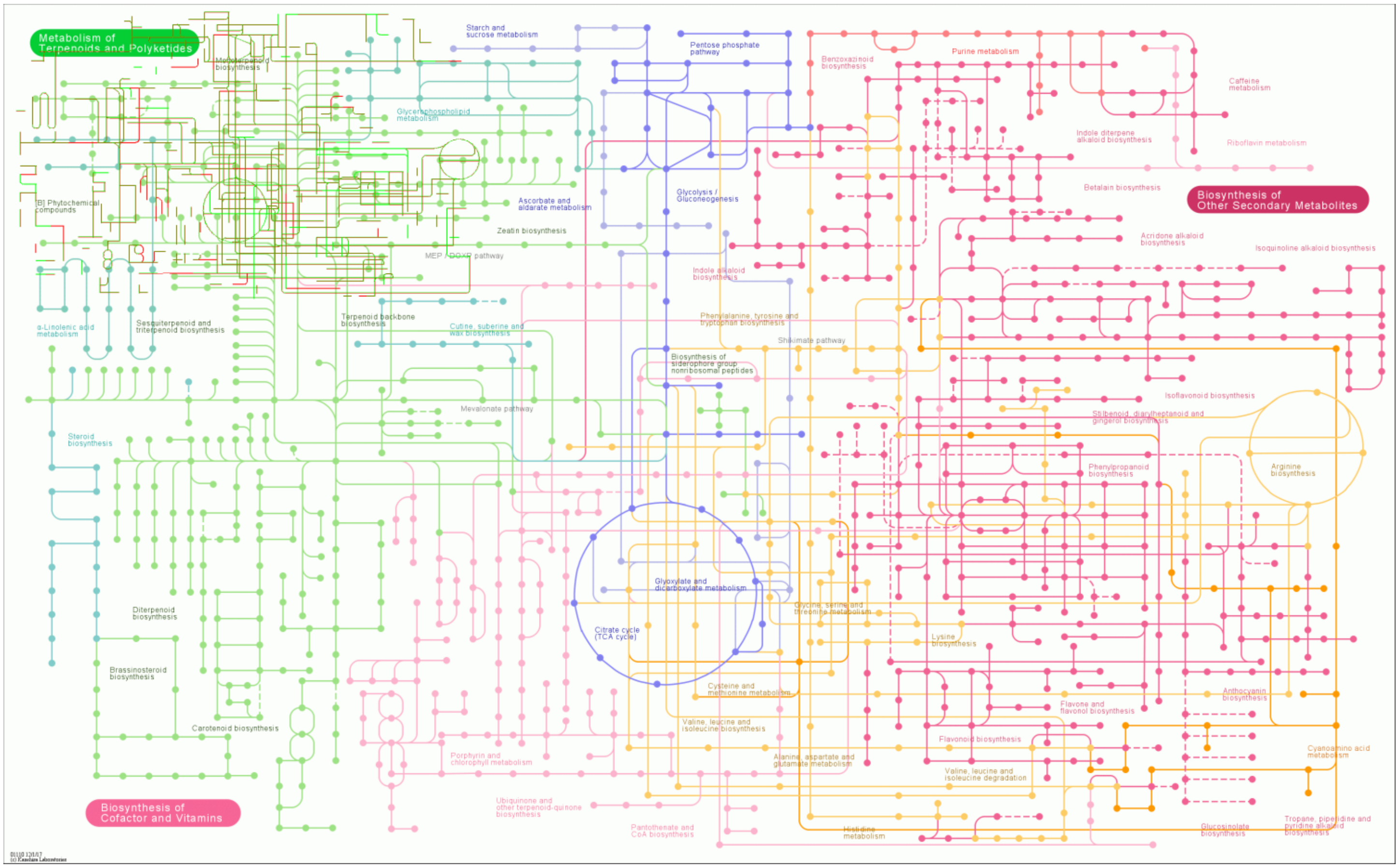
Metabolism of Terpenoids and Polyketides
Biosynthesis of Other Secondary Metabolites
Biosynthesis of Cofactor and Vitamins
Purine metabolism
Glycolysis / Gluconeogenesis
Glyoxylate and dicarboxylate metabolism
Citrate cycle (TCA cycle)
Arginine biosynthesis

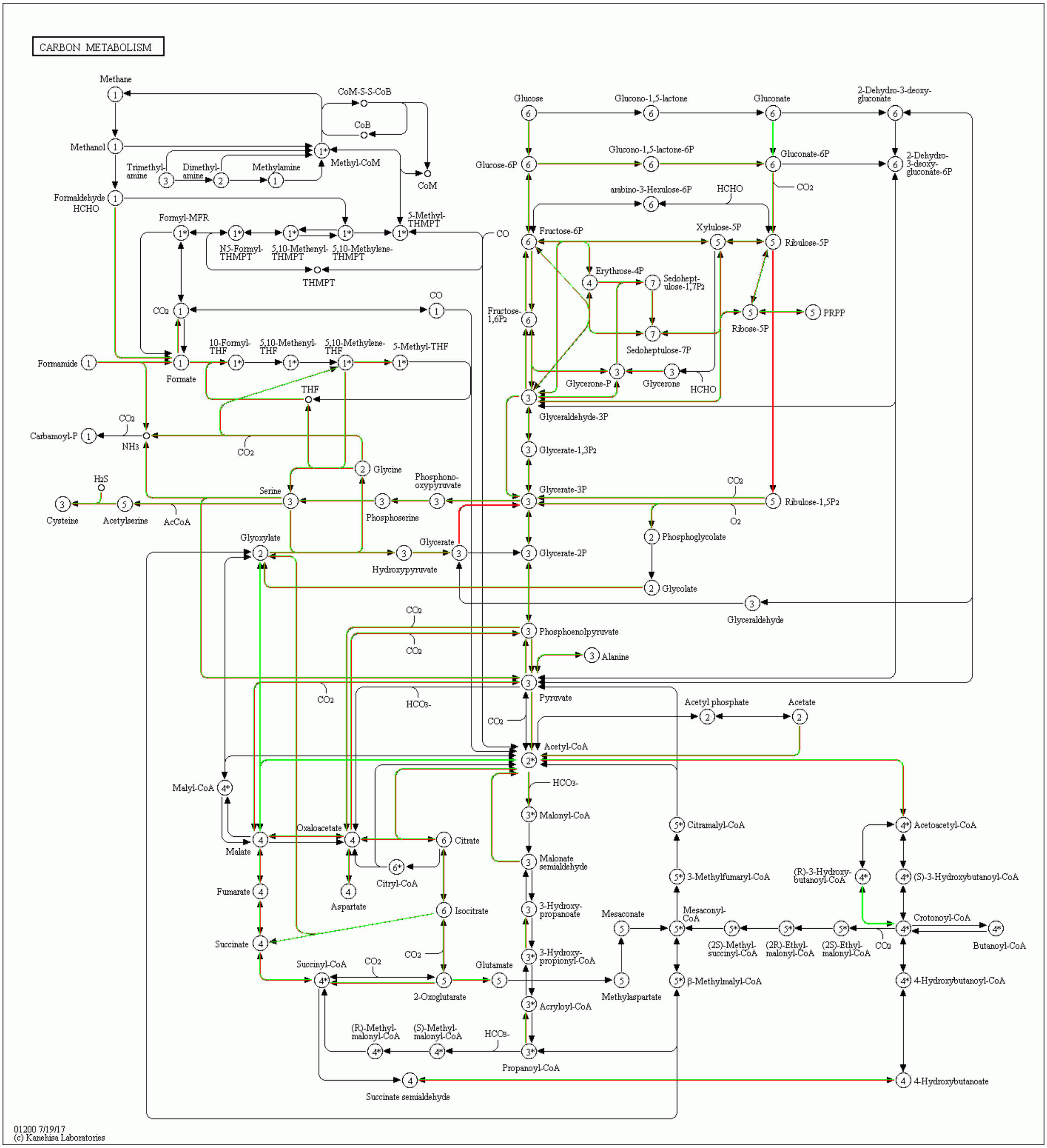

01200 7/19/17
(c) Kanehisa Laboratories

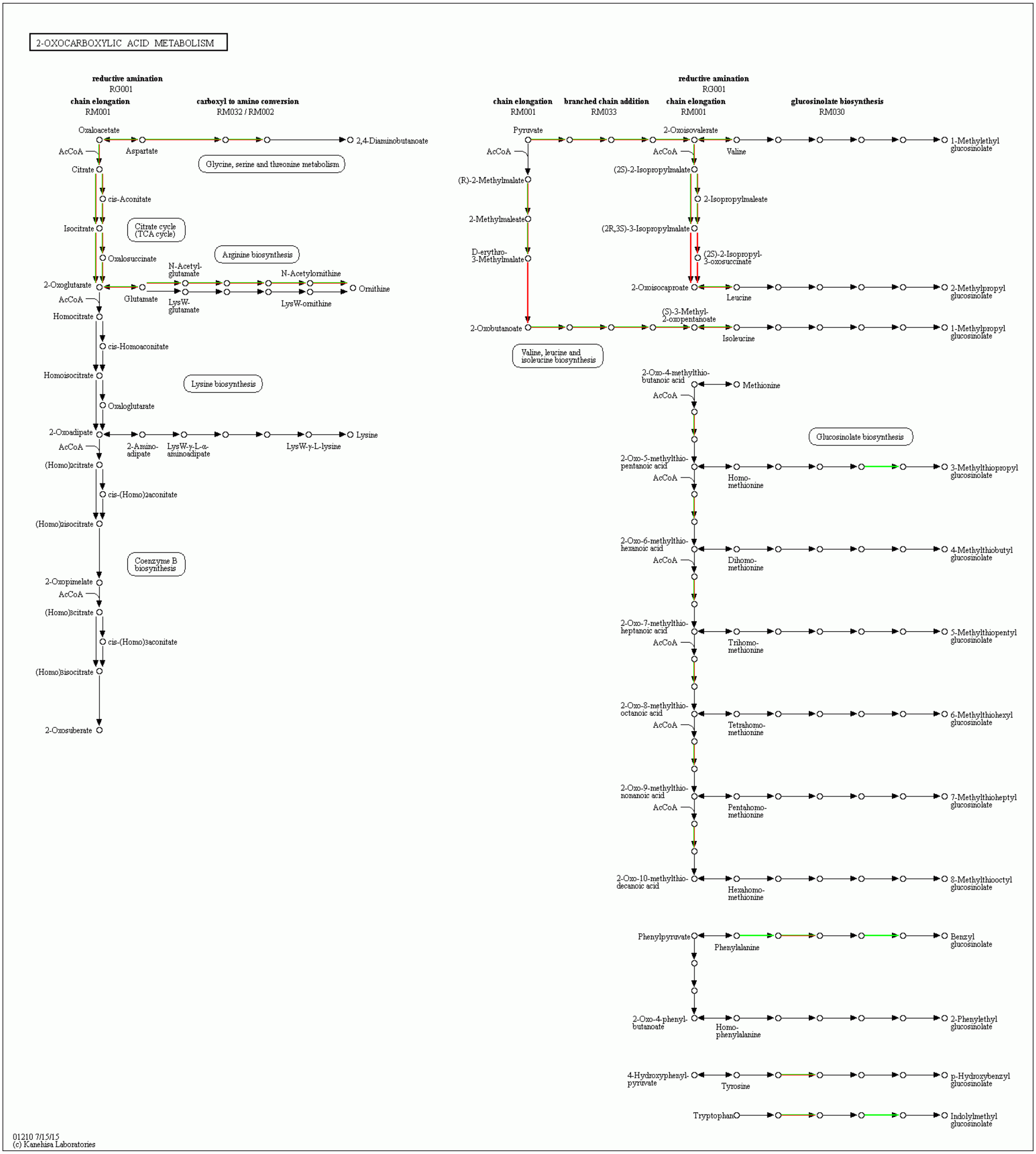
2-OXOCARBOXYLIC ACID METABOLISM
reductive amination
RG001
chain elongation
RM001
carboxyl to amino conversion
RM032 / RM002
Oxaloacetate
Aspartate
2,4-Diaminobutanoate
AcCoA
Glycine, serine and threonine metabolism
Citrate
cis-Aconitate
Isocitrate
Citrate cycle (TCA cycle)
Oxalosuccinate
Arginine biosynthesis
N-Acetyl-glutamate
N-Acetylornithine
2-Oxoglutarate
Glutamate
LysW-glutamate
LysW-ornithine
Ornithine
AcCoA
Homocitrate
cis-Homoaconitate
Homoisocitrate
Lysine biosynthesis
Oxaloglutarate
2-Oxoadipate
2-Amino-adipate
LysW-γ-L-α-aminoadipate
LysW-γ-L-lysine
Lysine
AcCoA
(Homo)2citrate
cis-(Homo)2aconitate
(Homo)2isocitrate
Coenzyme B biosynthesis
2-Oxopimelate
AcCoA
(Homo)3citrate
cis-(Homo)3aconitate
(Homo)3isocitrate
2-Oxosuberate
reductive amination
RG001
chain elongation
RM001
branched chain addition
RM033
chain elongation
RM001
glucosinolate biosynthesis
RM030
Pyruvate
AcCoA
(R)-2-Methylmalate
2-Methylmaleate
D-erythro-3-Methylmalate
2-Oxobutanoate
Valine, leucine and isoleucine biosynthesis
2-Oxoisovalerate
AcCoA
Valine
1-Methylethyl glucosinolate
(2S)-2-Isopropylmalate
2-Isopropylmaleate
(2R,3S)-3-Isopropylmalate
(2S)-2-Isopropyl-3-oxosuccinate
2-Oxoisocaproate
Leucine
2-Methylpropyl glucosinolate
(S)-3-Methyl-2-oxopentanoate
Isoleucine
1-Methylpropyl glucosinolate
2-Oxo-4-methylthio-butanoic acid
Methionine
AcCoA
Glucosinolate biosynthesis
2-Oxo-5-methylthio-pentanoic acid
AcCoA
Homo-methionine
3-Methylthiopropyl glucosinolate
2-Oxo-6-methylthio-hexanoic acid
AcCoA
Dihomo-methionine
4-Methylthiobutyl glucosinolate
2-Oxo-7-methylthio-heptanoic acid
AcCoA
Trihomo-methionine
5-Methylthiopentyl glucosinolate
2-Oxo-8-methylthio-octanoic acid
AcCoA
Tetrahomo-methionine
6-Methylthiohexyl glucosinolate
2-Oxo-9-methylthio-nonanoic acid
AcCoA
Pentahomo-methionine
7-Methylthioheptyl glucosinolate
2-Oxo-10-methylthio-decanoic acid
Hexahomo-methionine
8-Methylthiooctyl glucosinolate
Phenylpyruvate
Phenylalanine
Benzyl glucosinolate
2-Oxo-4-phenyl-butanoate
Homo-phenylalanine
2-Phenylethyl glucosinolate
4-Hydroxyphenyl-pyruvate
Tyrosine
p-Hydroxybenzyl glucosinolate
Tryptophan
Indolylmethyl glucosinolate
01210 7/15/15
(c) Kanehisa Laboratories

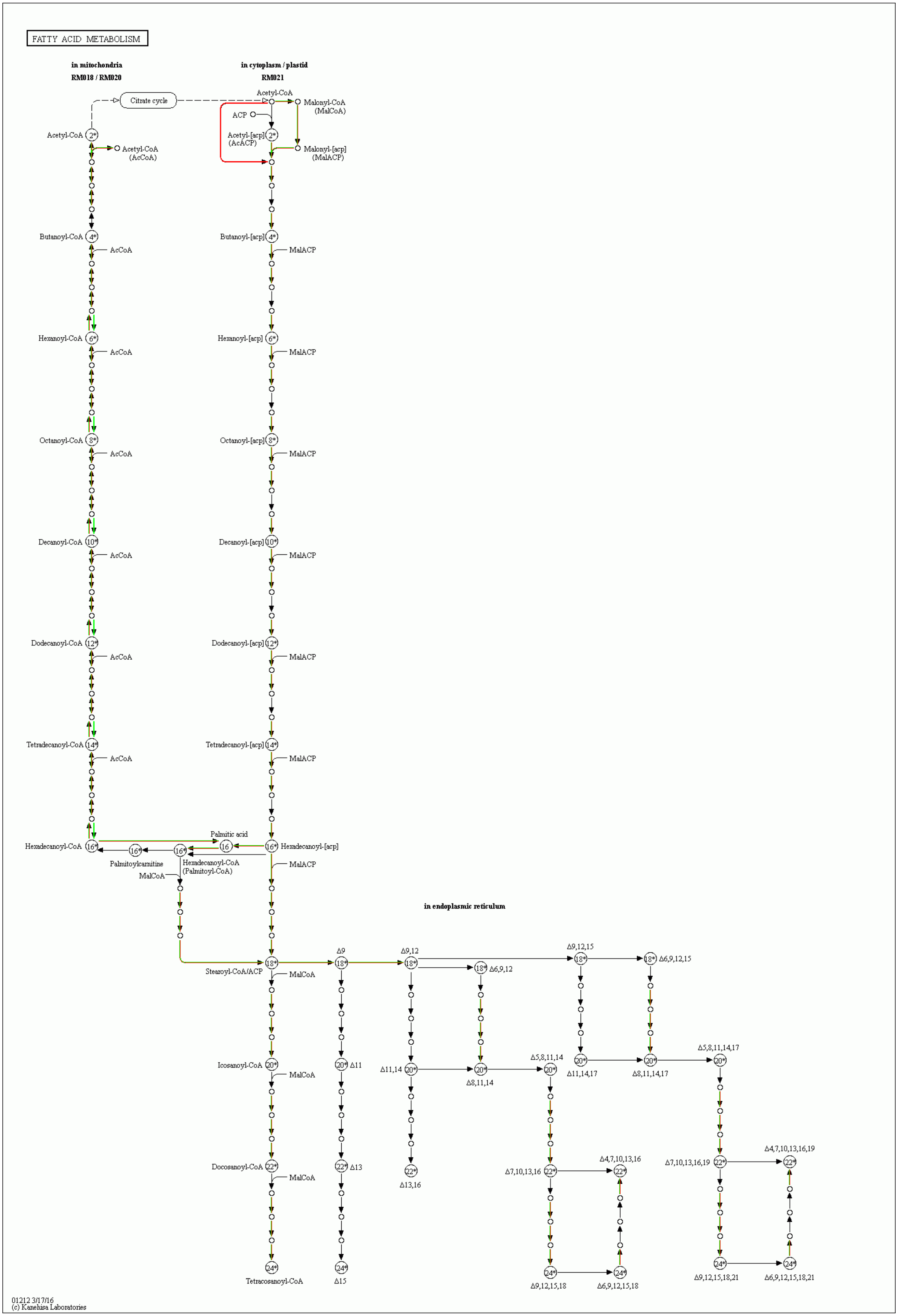
FATTY ACID METABOLISM
in mitochondria
RM018 / RM020
in cytoplasm / plastid
RM021
Citrate cycle
Acetyl-CoA
Malonyl-CoA (MalCoA)
ACP
Acetyl-[acp] (AcACP)
Malonyl-[acp] (MalACP)
Acetyl-CoA
Acetyl-CoA (AcCoA)
Butanoyl-CoA
AcCoA
Butanoyl-[acp]
MalACP
Hexanoyl-CoA
Hexanoyl-[acp]
Octanoyl-CoA
Octanoyl-[acp]
Decanoyl-CoA
Decanoyl-[acp]
Dodecanoyl-CoA
Dodecanoyl-[acp]
Tetradecanoyl-CoA
Tetradecanoyl-[acp]
Hexadecanoyl-CoA
Palmitic acid
Hexadecanoyl-[acp]
Palmitoylcarnitine
Hexadecanoyl-CoA (Palmitoyl-CoA)
MalCoA
in endoplasmic reticulum
Stearoyl-CoA/ACP
Δ9
Δ9,12
Δ6,9,12
Δ9,12,15
Δ6,9,12,15
Icosanoyl-CoA
Δ11
Δ11,14
Δ8,11,14
Δ5,8,11,14
Δ11,14,17
Δ8,11,14,17
Δ5,8,11,14,17
Docosanoyl-CoA
Δ13
Δ13,16
Δ7,10,13,16
Δ4,7,10,13,16
Δ7,10,13,16,19
Δ4,7,10,13,16,19
Tetracosanoyl-CoA
Δ15
Δ9,12,15,18
Δ6,9,12,15,18
Δ9,12,15,18,21
Δ6,9,12,15,18,21
01212 3/17/16
(c) Kanehisa Laboratories

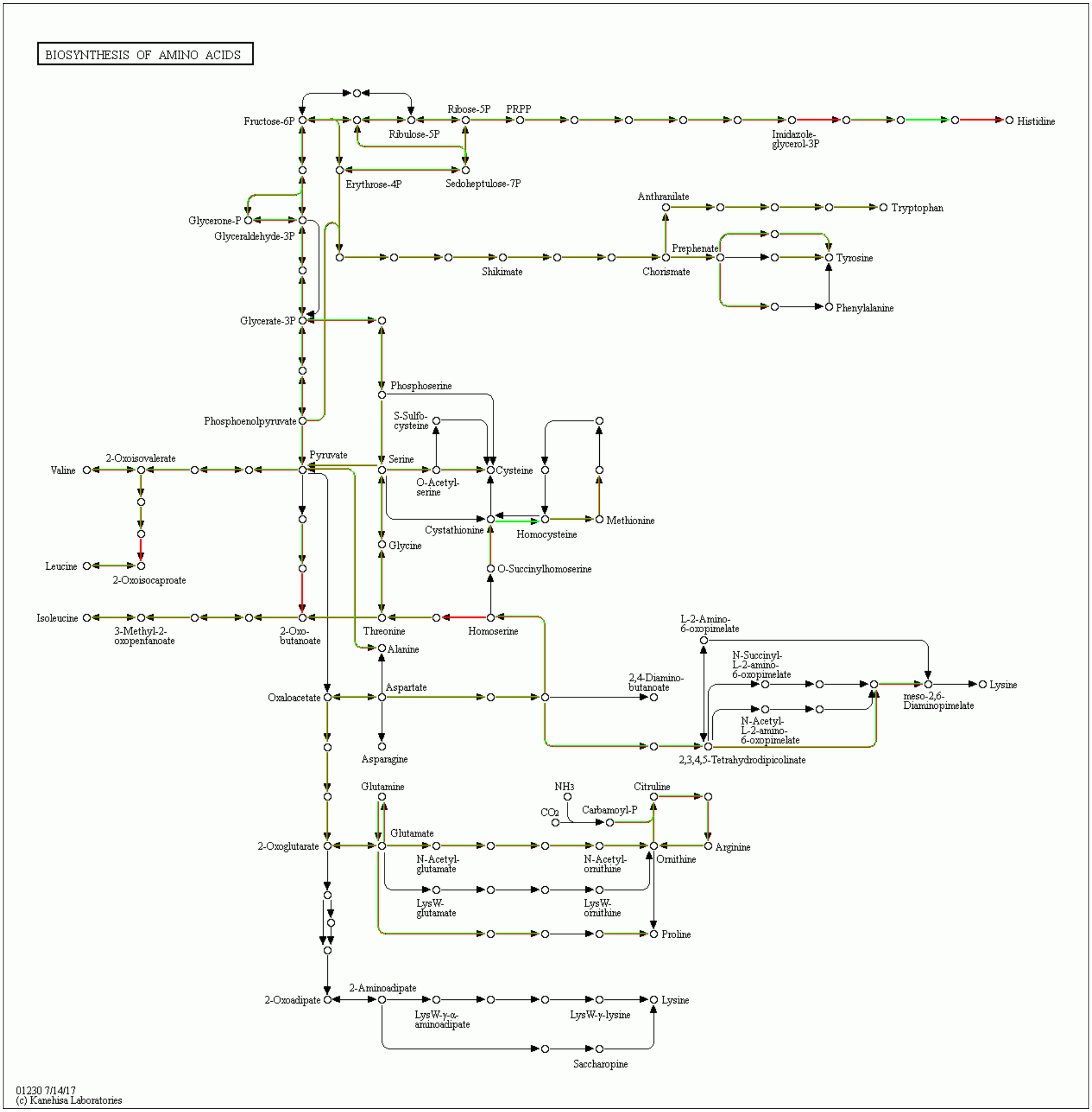
BIOSYNTHESIS OF AMINO ACIDS
Fructose-6P
Ribose-5P
PRPP
Ribulose-5P
Imidazole-glycerol-3P
Histidine
Erythrose-4P
Sedoheptulose-7P
Anthranilate
Tryptophan
Glycerone-P
Glyceraldehyde-3P
Prephenate
Tyrosine
Shikimate
Chorismate
Phenylalanine
Glycerate-3P
Phosphoserine
S-Sulfo-cysteine
Phosphoenolpyruvate
2-Oxoisovalerate
Pyruvate
Serine
Valine
O-Acetyl-serine
Cysteine
Methionine
Cystathionine
Homocysteine
Glycine
Leucine
2-Oxoisocaproate
O-Succinylhomoserine
Isoleucine
3-Methyl-2-oxopentanoate
2-Oxo-butanoate
Threonine
Homoserine
L-2-Amino-6-oxopimelate
Alanine
N-Succinyl-L-2-amino-6-oxopimelate
2,4-Diamino-butanoate
Aspartate
Oxaloacetate
Lysine
meso-2,6-Diaminopimelate
N-Acetyl-L-2-amino-6-oxopimelate
Asparagine
2,3,4,5-Tetrahydrodipicolinate
Glutamine
NH3
Citruline
CO2
Carbamoyl-P
Glutamate
2-Oxoglutarate
Arginine
N-Acetyl-glutamate
N-Acetyl-ornithine
Ornithine
LysW-glutamate
LysW-ornithine
Proline
2-Aminoadipate
2-Oxoadipate
Lysine
LysW-γ-α-aminoadipate
LysW-γ-lysine
Saccharopine
01230 7/14/17
(c) Kanehisa Laboratories

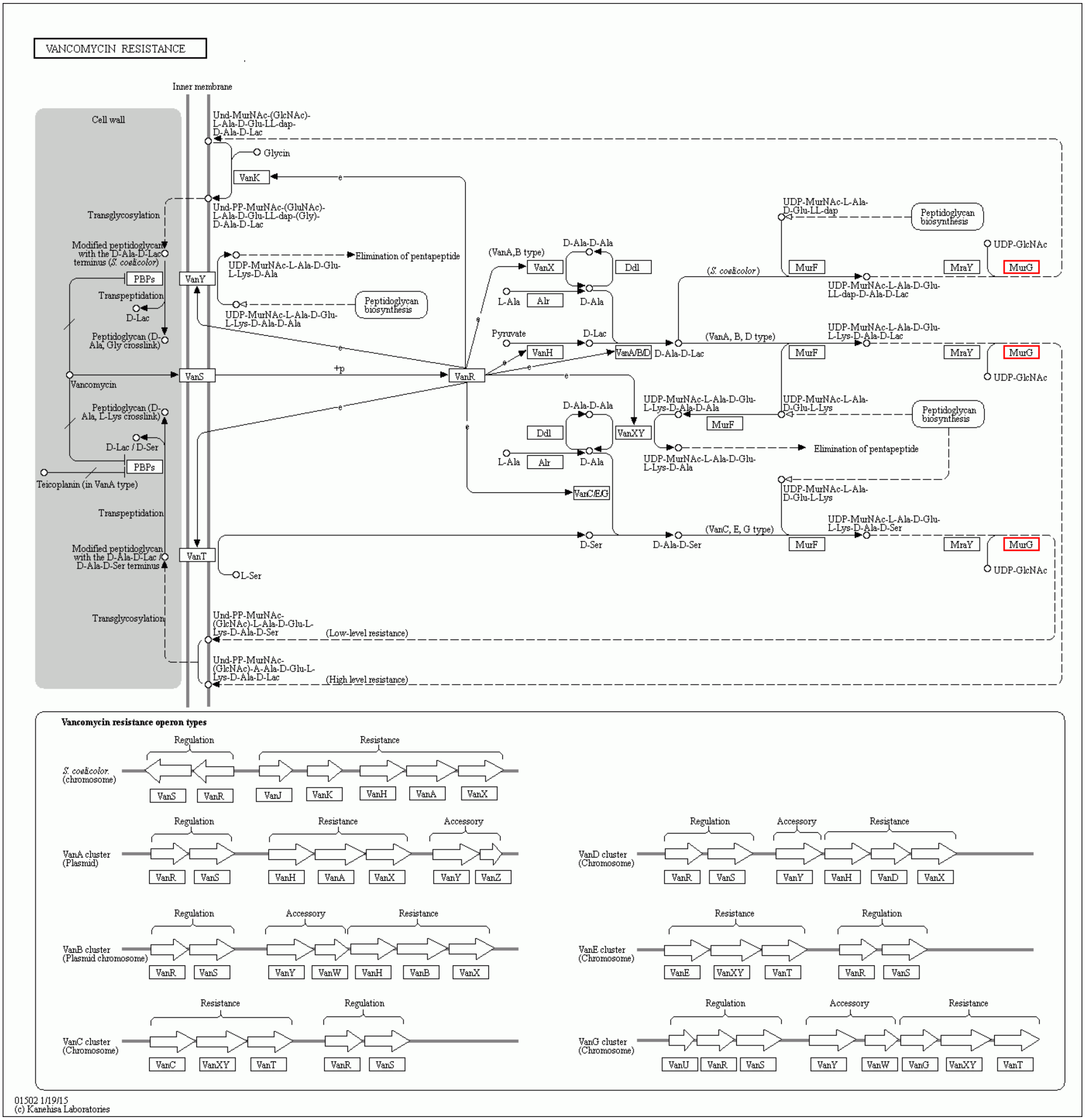
VANCOMYCIN RESISTANCE
Inner membrane
Cell wall
Und-MurNAc-(GlcNAc)-L-Ala-D-Glu-LL-dap-D-Ala-D-Lac
Glycin
VanK
Transglycosylation
Und-PP-MurNAc-(GluNAc)-L-Ala-D-Glu-LL-dap-(Gly)-D-Ala-D-Lac
Modified peptidoglycan with the D-Ala-D-Lac terminus (S. coelicolor)
PBPs
Transpeptidation
D-Lac
Peptidoglycan (D-Ala, Gly crosslink)
VanY
UDP-MurNAc-L-Ala-D-Glu-L-Lys-D-Ala
Elimination of pentapeptide
Peptidoglycan biosynthesis
UDP-MurNAc-L-Ala-D-Glu-L-Lys-D-Ala-D-Ala
Vancomycin
VanS
+p
VanR
Peptidoglycan (D-Ala, L-Lys crosslink)
D-Lac / D-Ser
Teicoplanin (in VanA type)
Transpeptidation
Modified peptidoglycan with the D-Ala-D-Lac / D-Ala-D-Ser terminus
VanT
L-Ser
Transglycosylation
Und-PP-MurNAc-(GlcNAc)-L-Ala-D-Glu-L-Lys-D-Ala-D-Ser
(Low-level resistance)
Und-PP-MurNAc-(GlcNAc)-A-Ala-D-Glu-L-Lys-D-Ala-D-Lac
(High level resistance)
(VanA,B type)
VanX
D-Ala-D-Ala
Ddl
L-Ala
Alr
D-Ala
Pyruvate
D-Lac
VanH
VanA/B/D
D-Ala-D-Lac
(VanA, B, D type)
(S. coelicolor)
UDP-MurNAc-L-Ala-D-Glu-LL-dap
Peptidoglycan biosynthesis
MurF
MraY
MurG
UDP-GlcNAc
UDP-MurNAc-L-Ala-D-Glu-LL-dap-D-Ala-D-Lac
UDP-MurNAc-L-Ala-D-Glu-L-Lys-D-Ala-D-Lac
VanXY
UDP-MurNAc-L-Ala-D-Glu-L-Lys-D-Ala-D-Ala
UDP-MurNAc-L-Ala-D-Glu-L-Lys
UDP-MurNAc-L-Ala-D-Glu-L-Lys-D-Ala
VanC/E/G
UDP-MurNAc-L-Ala-D-Glu-L-Lys
D-Ser
D-Ala-D-Ser
(VanC, E, G type)
UDP-MurNAc-L-Ala-D-Glu-L-Lys-D-Ala-D-Ser
Vancomycin resistance operon types
Regulation
Resistance
Accessory
S. coelicolor (chromosome)
VanS VanR VanJ VanK VanH VanA VanX
VanA cluster (Plasmid)
VanR VanS VanH VanA VanX VanY VanZ
VanB cluster (Plasmid chromosome)
VanR VanS VanY VanW VanH VanB VanX
VanC cluster (Chromosome)
VanC VanXY VanT VanR VanS
VanD cluster (Chromosome)
VanR VanS VanY VanH VanD VanX
VanE cluster (Chromosome)
VanE VanXY VanT VanR VanS
VanG cluster (Chromosome)
VanU VanR VanS VanY VanW VanG VanXY VanT
01502 1/19/15
(c) Kanehisa Laboratories

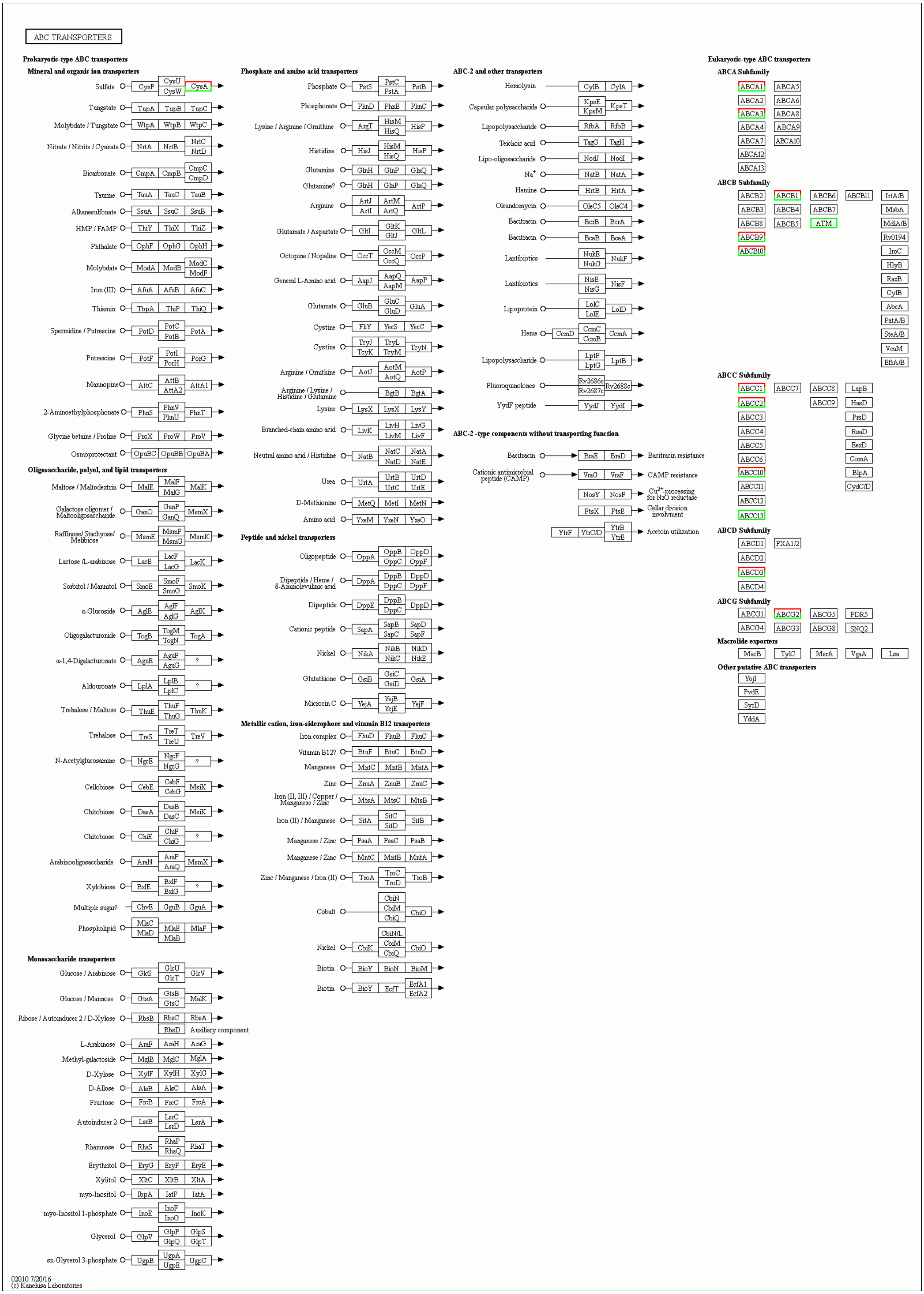

ABC TRANSPORTERS
Prokaryotic-type ABC transporters
Mineral and organic ion transporters
Sulfate CysP CysU CysW CysA
Tungstate TupA TupB TupC
Molybdate / Tungstate WtpA WtpB WtpC
Nitrate / Nitrite / Cyanate NrtA NrtB NrtC NrtD
Bicarbonate CmpA CmpB CmpC CmpD
Taurine TauA TauC TauB
Alkanesulfonate SsuA SsuC SsuB
HMP / FAMP ThiY ThiX ThiZ
Phthalate OphF OphG OphH
Molybdate ModA ModB ModC ModF
Iron (III) AfuA AfuB AfuC
Thiamin TbpA ThiP ThiQ
Spermidine / Putrescine PotD PotC PotB PotA
Putrescine PotF PotI PotH PotG
Mannopine AttC AttB AttA2 AttA1
2-Aminoethylphosphonate PhnS PhnV PhnU PhnT
Glycine betaine / Proline ProX ProW ProV
Osmoprotectant OpuBC OpuBB OpuBA
Oligosaccharide, polyol, and lipid transporters
Maltose / Maltodextrin MalE MalF MalG MalK
Galactose oligomer / Maltooligosaccharide GanO GanP GanQ MsmX
Raffinose/ Stachyose/ Melibiose MsmE MsmF MsmG MsmK
Lactose /L-arabinose LacE LacF LacG LacK
Sorbitol / Mannitol SmoE SmoF SmoG SmoK
α-Glucoside AglE AglF AglG AglK
Oligogalacturonide TogB TogM TogN TogA
α-1,4-Digalacturonate AguE AguF AguG ?
Aldouronate LplA LplB LplC ?
Trehalose / Maltose ThuE ThuF ThuG ThuK
Trehalose TreS TreT TreU TreV
N-Acetylglucosamine NgcE NgcF NgcG ?
Cellobiose CebE CebF CebG MsiK
Chitobiose DasA DasB DasC MsiK
Chitobiose ChiE ChiF ChiG ?
Arabinooligosaccharide AraN AraP AraQ MsmX
Xylobiose BxlE BxlF BxlG ?
Multiple sugar? ChvE GguB GguA
Phospholipid MlaC MlaD MlaE MlaB MlaF
Monosaccharide transporters
Glucose / Arabinose GlcS GlcU GlcT GlcV
Glucose / Mannose GtsA GtsB GtsC MalK
Ribose / Autoinducer 2 / D-Xylose RbsB RbsC RbsA
RbsD Auxiliary component
L-Arabinose AraF AraH AraG
Methyl-galactoside MglB MglC MglA
D-Xylose XylF XylH XylG
D-Allose AlsB AlsC AlsA
Fructose FrcB FrcC FrcA
Autoinducer 2 LsrB LsrC LsrD LsrA
Rhamnose RhaS RhaP RhaQ RhaT
Erythritol EryG EryF EryE
Xylitol XltC XltB XltA
myo-Inositol IbpA IatP IatA
myo-Inositol 1-phosphate InoE InoF InoG InoK
Glycerol GlpV GlpP GlpQ GlpS GlpT
sn-Glycerol 3-phosphate UgpB UgpA UgpE UgpC
Phosphate and amino acid transporters
Phosphate PstS PstC PstA PstB
Phosphonate PhnD PhnE PhnC
Lysine / Arginine / Ornithine ArgT HisM HisQ HisP
Histidine HisJ HisM HisQ HisP
Glutamine GlnH GlnP GlnQ
Glutamine? GlnH GlnP GlnQ
Arginine ArtJ ArtI ArtM ArtQ ArtP
Glutamate / Aspartate GltI GltK GltJ GltL
Octopine / Nopaline OccT OccM OccQ OccP
General L-Amino acid AapJ AapQ AapM AapP
Glutamate GluB GluC GluD GluA
Cystine FliY YecS YecC
Cystine TcyJ TcyK TcyL TcyM TcyN
Arginine / Ornithine AotJ AotM AotQ AotP
Arginine / Lysine / Histidine / Glutamine BgtB BgtA
Lysine LysX LysX LysY
Branched-chain amino acid LivK LivH LivM LivG LivF
Neutral amino acid / Histidine NatB NatC NatD NatA NatE
Urea UrtA UrtB UrtC UrtD UrtE
D-Methionine MetQ MetI MetN
Amino acid YxeM YxeN YxeO
Peptide and nickel transporters
Oligopeptide OppA OppB OppC OppD OppF
Dipeptide / Heme / δ-Aminolevulinic acid DppA DppB DppC DppD DppF
Dipeptide DppE DppB DppC DppD
Cationic peptide SapA SapB SapC SapD SapF
Nickel NikA NikB NikC NikD NikE
Glutathione GsiB GsiC GsiD GsiA
Microcin C YejA YejB YejE YejF
Metallic cation, iron-siderophore and vitamin B12 transporters
Iron complex FhuD FhuB FhuC
Vitamin B12? BtuF BtuC BtuD
Manganese MntC MntB MntA
Zinc ZnuA ZnuB ZnuC
Iron (II, III) / Copper / Manganese / Zinc MtsA MtsC MtsB
Iron (II) / Manganese SitA SitC SitD SitB
Manganese / Zinc PsaA PsaC PsaB
Manganese / Zinc MntC MntB MntA
Zinc / Manganese / Iron (II) TroA TroC TroD TroB
Cobalt CbiN CbiM CbiQ CbiO
Nickel CbiK CbiN/L CbiM CbiQ CbiO
Biotin BioY BioN BioM
Biotin BioY EcfT EcfA1 EcfA2
ABC-2 and other transporters
Hemolysin CylB CylA
Capsular polysaccharide KpsE KpsM KpsT
Lipopolysaccharide RfbA RfbB
Teichoic acid TagG TagH
Lipo-oligosaccharide NodJ NodI
Na+ NatB NatA
Hemine HrtB HrtA
Oleandomycin OleC5 OleC4
Bacitracin BcrB BcrA
Bacitracin BceB BceA
Lantibiotics NukE NukG NukF
Lantibiotics NisE NisG NisF
Lipoprotein LolC LolE LolD
Heme CcmD CcmC CcmB CcmA
Lipopolysaccharide LptF LptG LptB
Fluoroquinolones Rv2686c Rv2687c Rv2688c
YydF peptide YydJ YydI
ABC-2 -type components without transporting function
Bacitracin BraE BraD Bacitracin resistance
Cationic antimicrobial peptide (CAMP) VraG VraF CAMP resistance
NosY NosF Cu2+-processing for N2O reductase
FtsX FtsE Cellar division involvment
YtrF YtrC/D YtrB YtrE Acetoin utilization
Eukaryotic-type ABC transporters
ABCA Subfamily
ABCA1 ABCA5
ABCA2 ABCA6
ABCA3 ABCA8
ABCA4 ABCA9
ABCA7 ABCA10
ABCA12
ABCA13
ABCB Subfamily
ABCB2 ABCB1 ABCB6 ABCB11 IrtA/B
ABCB3 ABCB4 ABCB7 MsbA
ABCB8 ABCB5 ATM MdlA/B
ABCB9 Rv0194
ABCB10 IrtC
HlyB
RaxB
CylB
AbcA
PatA/B
SteA/B
VcaM
EfrA/B
ABCC Subfamily
ABCC1 ABCC7 ABCC8 LapB
ABCC2 ABCC9 HasD
ABCC3 PrsD
ABCC4 RsaD
ABCC5 EexD
ABCC6 ComA
ABCC10 BlpA
ABCC11 CydC/D
ABCC12
ABCC13
ABCD Subfamily
ABCD1 PXA1/2
ABCD2
ABCD3
ABCD4
ABCG Subfamily
ABCG1 ABCG2 ABCG5 PDR5
ABCG4 ABCG3 ABCG8 SNQ2
Macrolide exporters
MacB TylC MsrA VgaA Lsa
Other putative ABC transporters
YojI
PvdE
SyrD
YddA
02010 7/20/16
(c) Kanehisa Laboratories

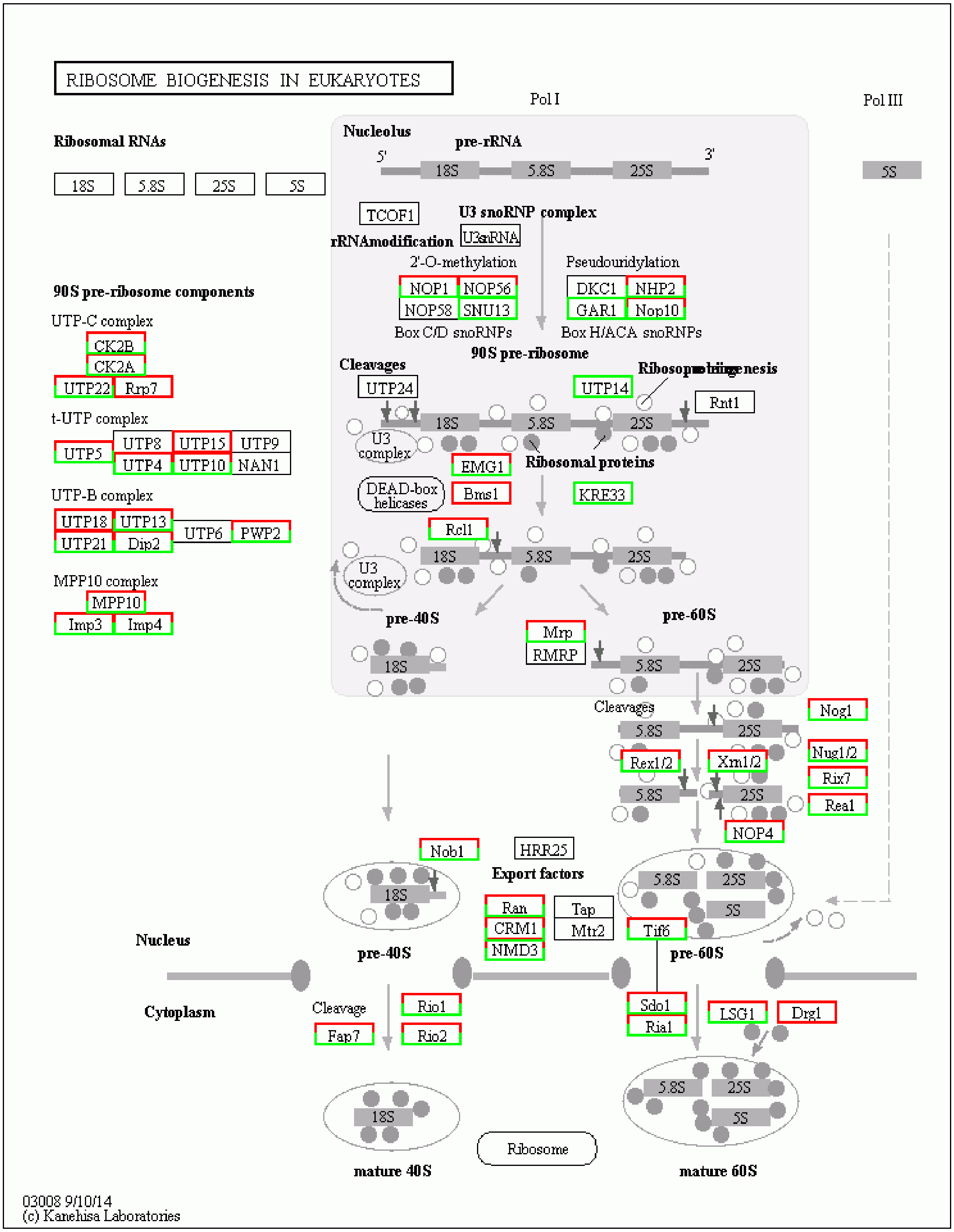
RIBOSOME BIOGENESIS IN EUKARYOTES
Ribosomal RNAs
18S
5.8S
25S
5S
90S pre-ribosome components
UTP-C complex
CK2B
CK2A
UTP22
Rrp7
t-UTP complex
UTP5
UTP8
UTP15
UTP9
UTP4
UTP10
NAN1
UTP-B complex
UTP18
UTP13
UTP6
PWP2
UTP21
Dip2
MPP10 complex
MPP10
Imp3
Imp4
Pol I
Pol III
Nucleolus
pre-rRNA
5'
3'
TCOF1
U3 snoRNP complex
U3snRNA
rRNAmodification
2'-O-methylation
Pseudouridylation
NOP1
NOP56
NOP58
SNU13
DKC1
NHP2
GAR1
Nop10
Box C/D snoRNPs
Box H/ACA snoRNPs
90S pre-ribosome
Cleavages
UTP24
UTP14
Rnt1
U3 complex
EMG1
Ribosomal proteins
DEAD-box helicases
Bms1
KRE33
Rcl1
pre-40S
pre-60S
Mrp
RMRP
Nog1
Nug1/2
Rix7
Rea1
Rex1/2
Xrn1/2
NOP4
Nob1
HRR25
Export factors
Ran
CRM1
NMD3
Tap
Mtr2
Tif6
Nucleus
Cytoplasm
Cleavage
Fap7
Rio1
Rio2
Sdo1
Ria1
LSG1
Drg1
mature 40S
Ribosome
mature 60S
03008 9/10/14
(c) Kanehisa Laboratories

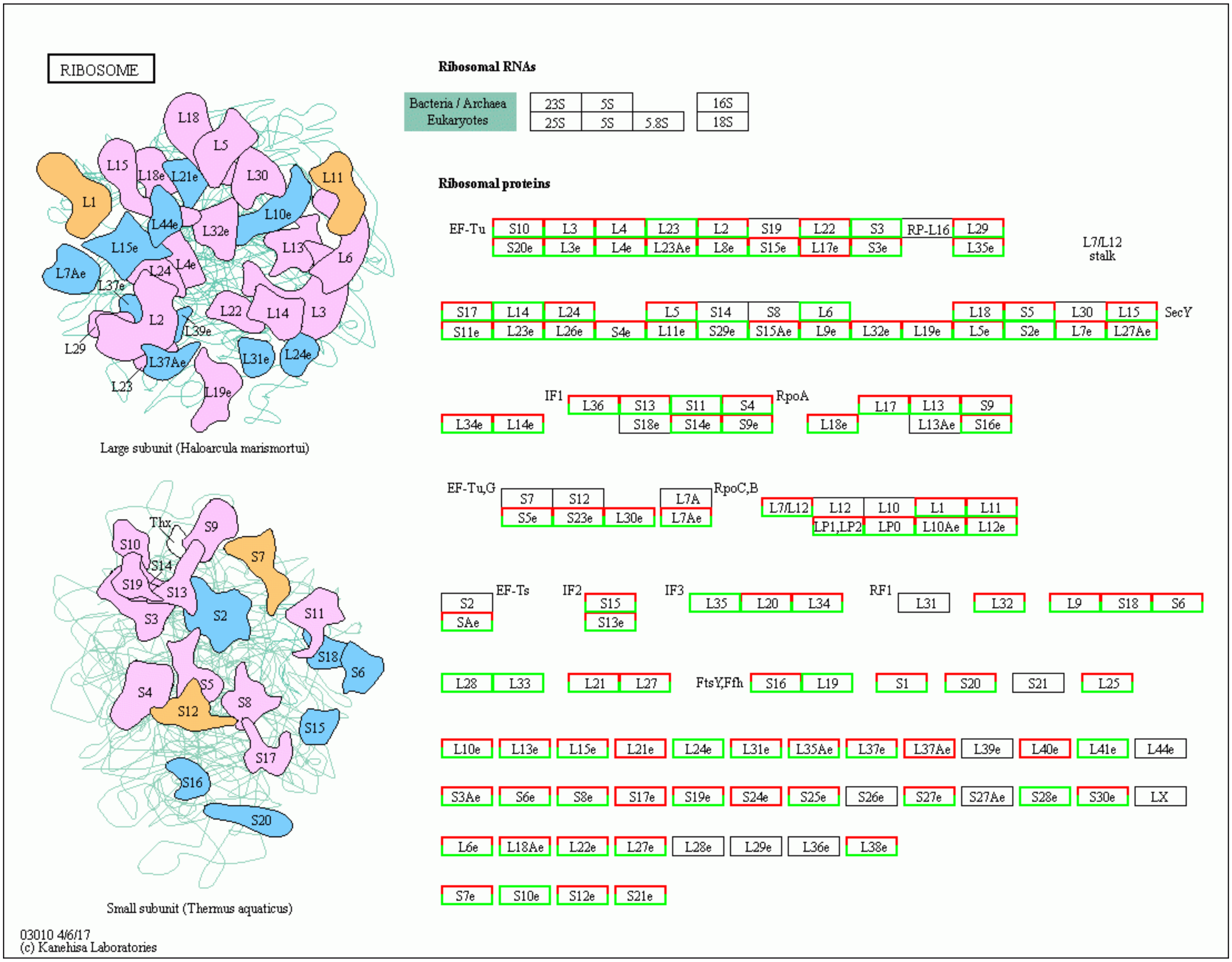
RIBOSOME
L18
L5
L15
L18e
L21e
L30
L11
L1
L10e
L44e
L32e
L15e
L13
L6
L7Ae
L4e
L24
L37e
L2
L22
L14
L3
L39e
L29
L37Ae
L31e
L24e
L23
L19e
Large subunit (Haloarcula marismortui)
Thx
S9
S10
S7
S14
S19
S13
S2
S11
S3
S18
S6
S4
S5
S8
S12
S15
S17
S16
S20
Small subunit (Thermus aquaticus)
Ribosomal RNAs
Bacteria / Archaea
Eukaryotes
23S
5S
16S
25S
5S
5.8S
18S
Ribosomal proteins
EF-Tu
S10 L3 L4 L23 L2 S19 L22 S3 RP-L16 L29
S20e L3e L4e L23Ae L8e S15e L17e S3e L35e
L7/L12
stalk
S17 L14 L24 L5 S14 S8 L6 L18 S5 L30 L15 SecY
S11e L23e L26e S4e L11e S29e S15Ae L9e L32e L19e L5e S2e L7e L27Ae
IF1
L36 S13 S11 S4 RpoA
L34e L14e S18e S14e S9e L18e
L17 L13 S9
L13Ae S16e
EF-Tu,G
S7 S12 L7A RpoC,B
S5e S23e L30e L7Ae
L7/L12 L12 L10 L1 L11
LP1,LP2 LP0 L10Ae L12e
S2 EF-Ts
SAe
IF2
S15
S13e
IF3
L35 L20 L34
RF1
L31 L32 L9 S18 S6
L28 L33 L21 L27 FtsY,Ffh S16 L19 S1 S20 S21 L25
L10e L13e L15e L21e L24e L31e L35Ae L37e L37Ae L39e L40e L41e L44e
S3Ae S6e S8e S17e S19e S24e S25e S26e S27e S27Ae S28e S30e LX
L6e L18Ae L22e L27e L28e L29e L36e L38e
S7e S10e S12e S21e
03010 4/6/17
(c) Kanehisa Laboratories

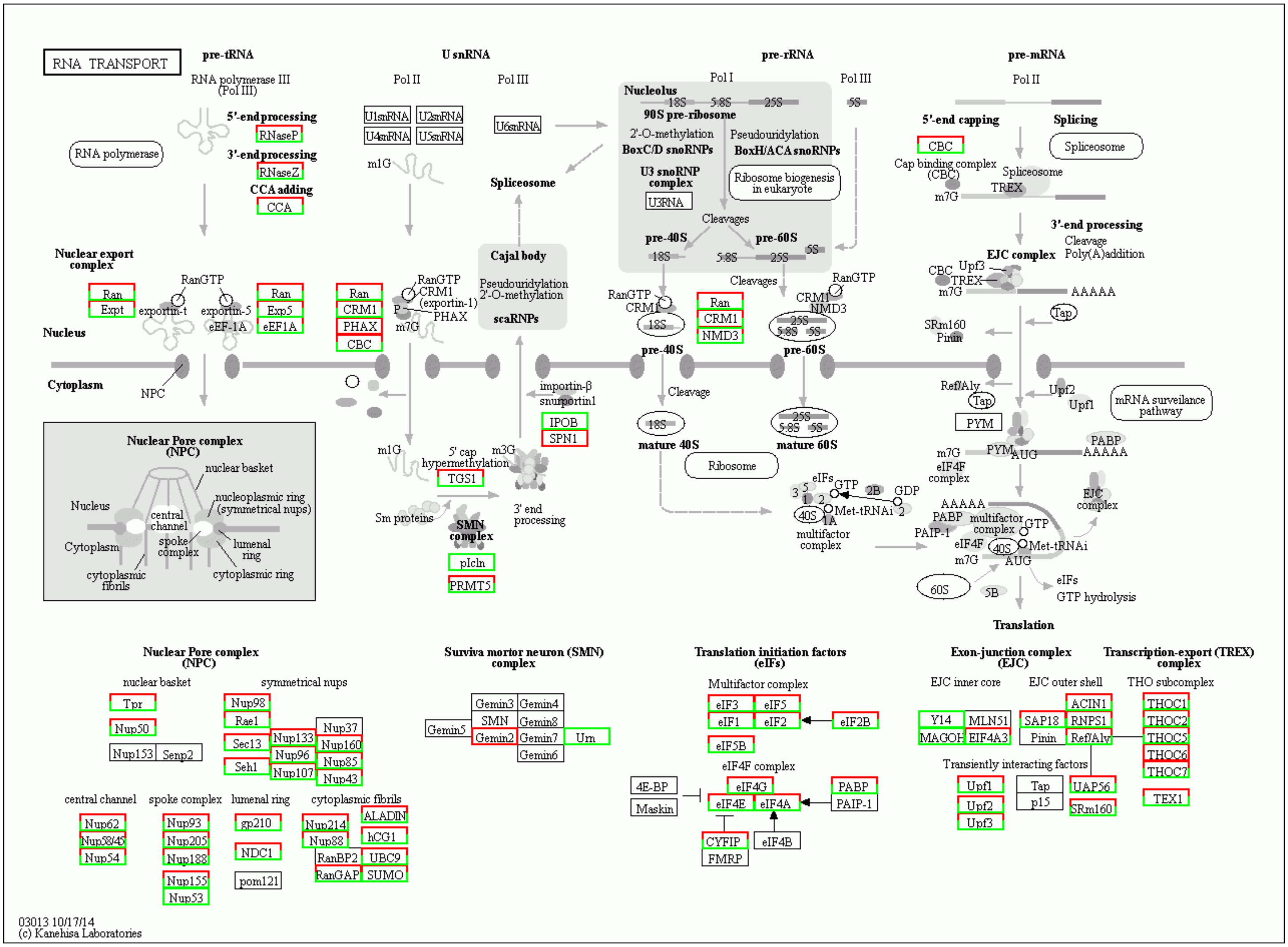
RNA TRANSPORT
pre-tRNA
U snRNA
pre-rRNA
pre-mRNA
RNA polymerase III (Pol III)
5'-end processing
RNaseP
3'-end processing
RNaseZ
CCA adding
CCA
RNA polymerase
Nucleolus
90S pre-ribosome
2'-O-methylation
BoxC/D snoRNPs
Pseudouridylation
BoxH/ACA snoRNPs
U3 snoRNP complex
U3RNA
Ribosome biogenesis in eukaryote
Cleavages
pre-40S
pre-60S
5'-end capping
Splicing
CBC
Spliceosome
Cap binding complex (CBC)
TREX
3'-end processing
Cleavage
Poly(A)addition
EJC complex
Nuclear export complex
Ran
Expt
exportin-t
exportin-5
Exp5
eEF-1A
eEF1A
CRM1
PHAX
Cajal body
Pseudouridylation
2'-O-methylation
scaRNPs
NMD3
SRm160
Pinin
Nucleus
Cytoplasm
NPC
importin-β
snurportin1
IPOB
SPN1
Ref/Aly
Tap
PYM
Upf2
Upf1
mRNA surveilance pathway
Nuclear Pore complex (NPC)
nuclear basket
nucleoplasmic ring (symmetrical nups)
central channel
spoke complex
lumenal ring
cytoplasmic fibrils
cytoplasmic ring
5' cap hypermethylation
TGS1
Sm proteins
SMN complex
3' end processing
pICln
PRMT5
mature 40S
mature 60S
Ribosome
eIFs
GTP
GDP
Met-tRNAi
multifactor complex
eIF4F complex
PABP
PAIP-1
EJC complex
eIFs
GTP hydrolysis
Translation
Nuclear Pore complex (NPC)
nuclear basket
symmetrical nups
Tpr
Nup50
Nup153
Senp2
Nup98
Rae1
Sec13
Seh1
Nup133
Nup96
Nup107
Nup37
Nup160
Nup85
Nup43
central channel
spoke complex
lumenal ring
cytoplasmic fibrils
Nup62
Nup58/45
Nup54
Nup93
Nup205
Nup188
Nup155
Nup53
gp210
NDC1
pom121
Nup214
Nup88
RanBP2
RanGAP
ALADIN
hCG1
UBC9
SUMO
Surviva mortor neuron (SMN) complex
Gemin3
Gemin4
SMN
Gemin8
Gemin5
Gemin2
Gemin7
Urn
Gemin6
Translation initiation factors (eIFs)
Multifactor complex
eIF3
eIF5
eIF1
eIF2
eIF2B
eIF5B
eIF4F complex
4E-BP
Maskin
eIF4G
eIF4E
eIF4A
PABP
PAIP-1
CYFIP
FMRP
eIF4B
Exon-junction complex (EJC)
EJC inner core
EJC outer shell
Y14
MLN51
MAGOH
EIF4A3
SAP18
Pinin
ACIN1
RNPS1
Ref/Aly
Transiently interacting factors
Upf1
Upf2
Upf3
Tap
p15
UAP56
SRm160
Transcription-export (TREX) complex
THO subcomplex
THOC1
THOC2
THOC5
THOC6
THOC7
TEX1
03013 10/17/14
(c) Kanehisa Laboratories

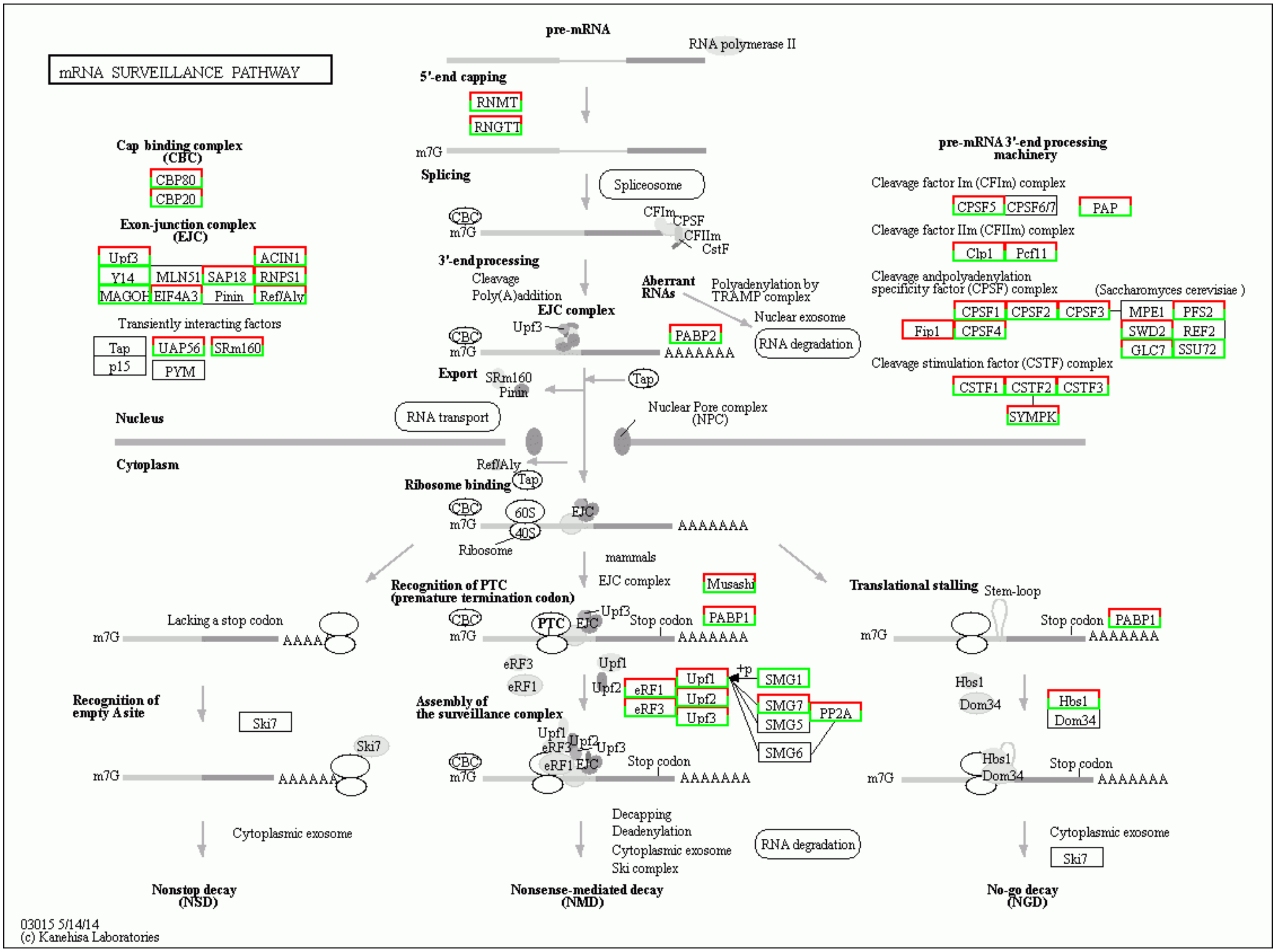
mRNA SURVEILLANCE PATHWAY
pre-mRNA
RNA polymerase II
5'-end capping
RNMT
RNGTT
m7G
Splicing
Spliceosome
Cap binding complex (CBC)
CBP80
CBP20
Exon-junction complex (EJC)
Upf3
ACIN1
Y14
MLN51
SAP18
RNPS1
MAGOH
EIF4A3
Pinin
Ref/Aly
Transiently interacting factors
Tap
UAP56
SRm160
p15
PYM
CBC
CFIm
CPSF
CFIIm
CstF
3'-end processing
Cleavage
Poly(A)addition
EJC complex
Aberrant RNAs
Polyadenylation by TRAMP complex
Nuclear exosome
RNA degradation
PABP2
AAAAAAA
Export
SRm160
Pinin
Tap
RNA transport
Nuclear Pore complex (NPC)
Nucleus
Cytoplasm
Ref/Aly
Ribosome binding
60S
40S
EJC
Ribosome
pre-mRNA 3'-end processing machinery
Cleavage factor Im (CFIm) complex
CPSF5
CPSF6/7
PAP
Cleavage factor IIm (CFIIm) complex
Clp1
Pcf11
Cleavage and polyadenylation specificity factor (CPSF) complex
(Saccharomyces cerevisiae)
CPSF1
CPSF2
CPSF3
MPE1
PFS2
Fip1
CPSF4
SWD2
REF2
GLC7
SSU72
Cleavage stimulation factor (CSTF) complex
CSTF1
CSTF2
CSTF3
SYMPK
mammals
EJC complex
Musashi
Recognition of PTC (premature termination codon)
PTC
Stop codon
PABP1
Translational stalling
Stem-loop
Lacking a stop codon
eRF3
eRF1
Upf1
Upf2
Upf3
SMG1
SMG7
SMG5
PP2A
SMG6
+p
Recognition of empty A site
Ski7
Assembly of the surveillance complex
Hbs1
Dom34
Decapping
Deadenylation
Cytoplasmic exosome
Ski complex
Nonstop decay (NSD)
Nonsense-mediated decay (NMD)
No-go decay (NGD)
03015 5/14/14
(c) Kanehisa Laboratories

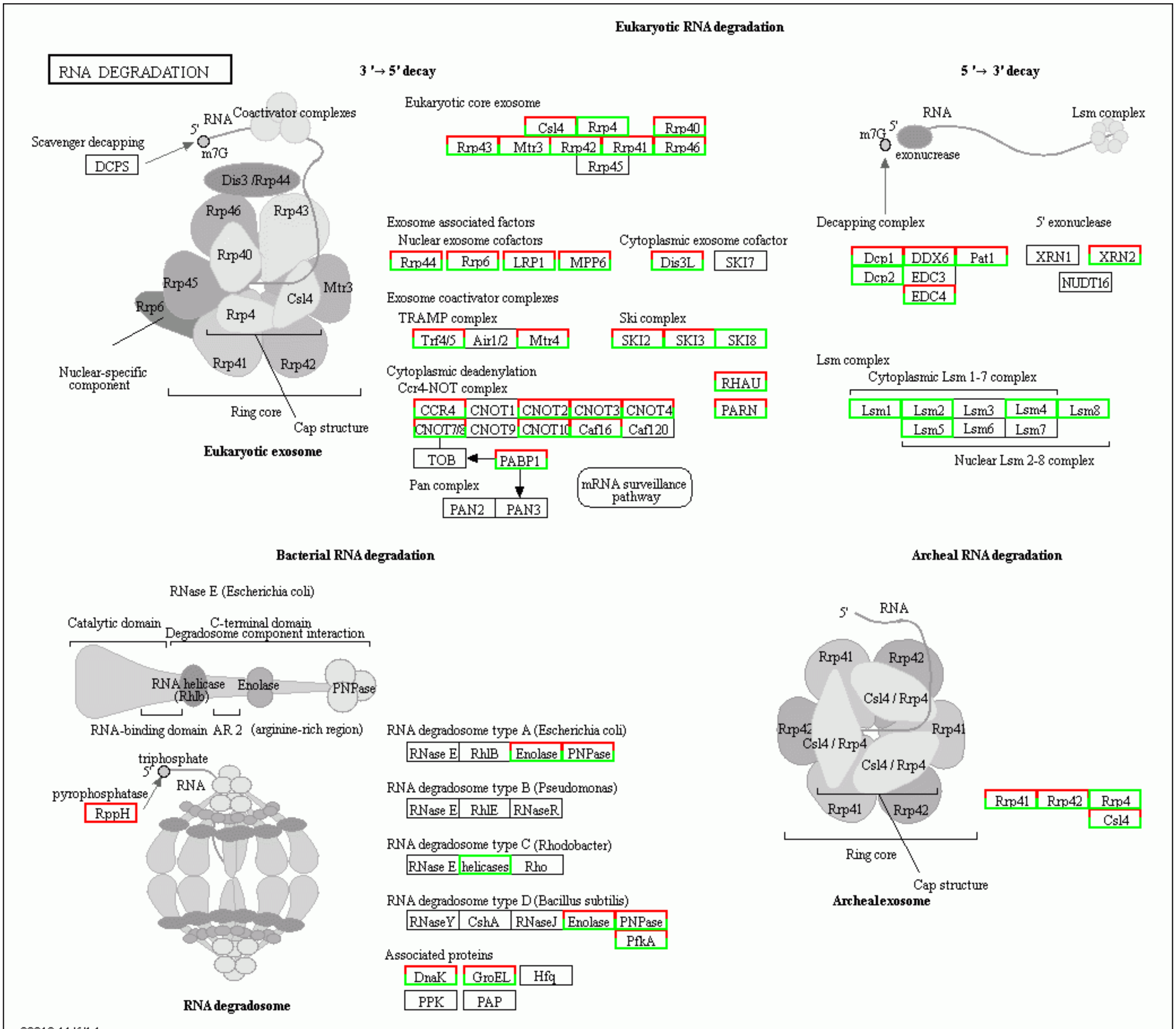

03018 11/6/14
(c) Kanehisa Laboratories

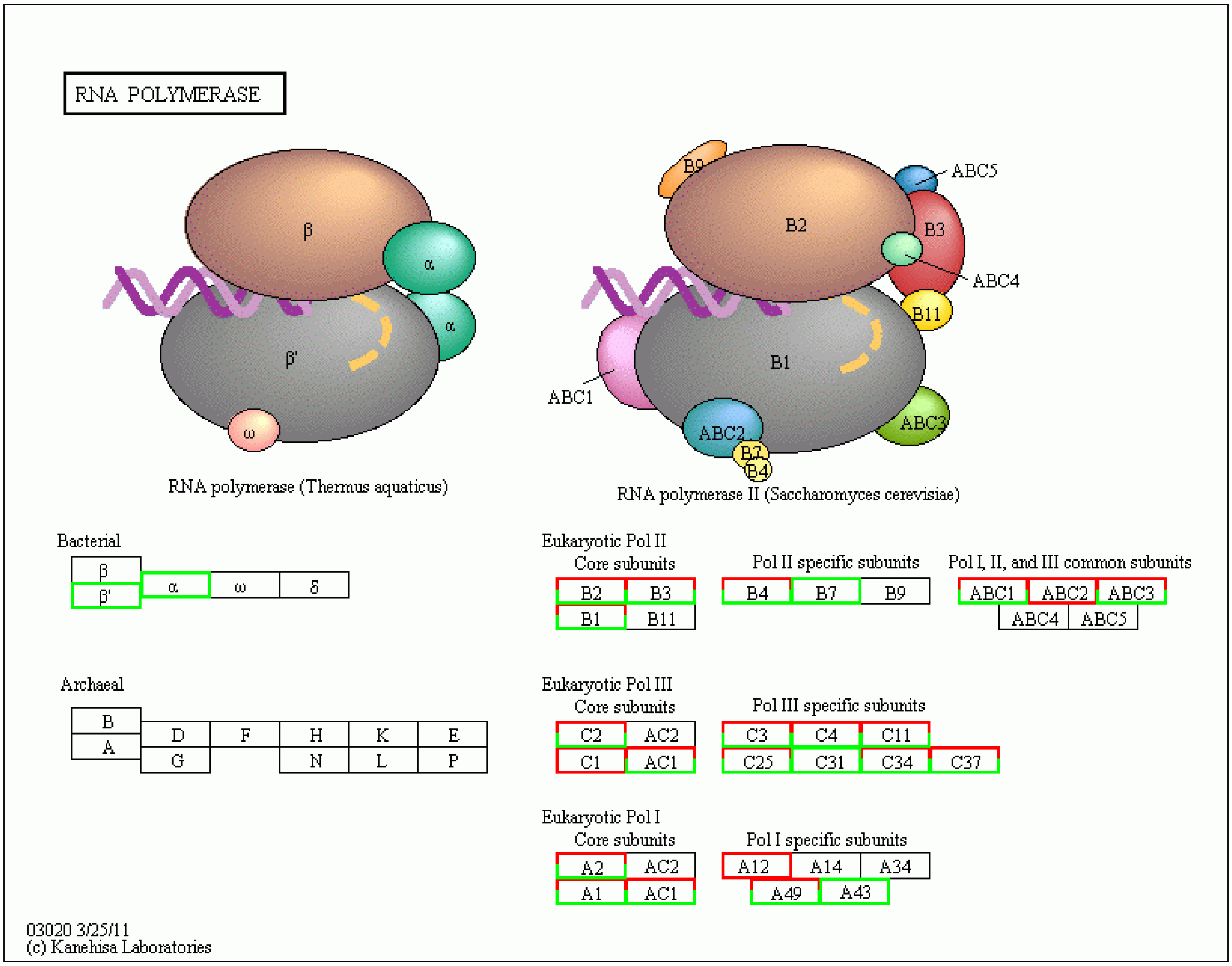
RNA POLYMERASE
β
α
α
β'
ω
RNA polymerase (Thermus aquaticus)
B9
B2
ABC5
B3
ABC4
B11
B1
ABC1
ABC2
ABC3
B7
B4
RNA polymerase II (Saccharomyces cerevisiae)
Bacterial
β
β'
α
ω
δ
Archaeal
B
A
D
G
F
H
N
K
L
E
P
Eukaryotic Pol II
Core subunits
B2
B3
B1
B11
Pol II specific subunits
B4
B7
B9
Pol I, II, and III common subunits
ABC1
ABC2
ABC3
ABC4
ABC5
Eukaryotic Pol III
Core subunits
C2
AC2
C1
AC1
Pol III specific subunits
C3
C4
C11
C25
C31
C34
C37
Eukaryotic Pol I
Core subunits
A2
AC2
A1
AC1
Pol I specific subunits
A12
A14
A34
A49
A43
03020 3/25/11
(c) Kanehisa Laboratories

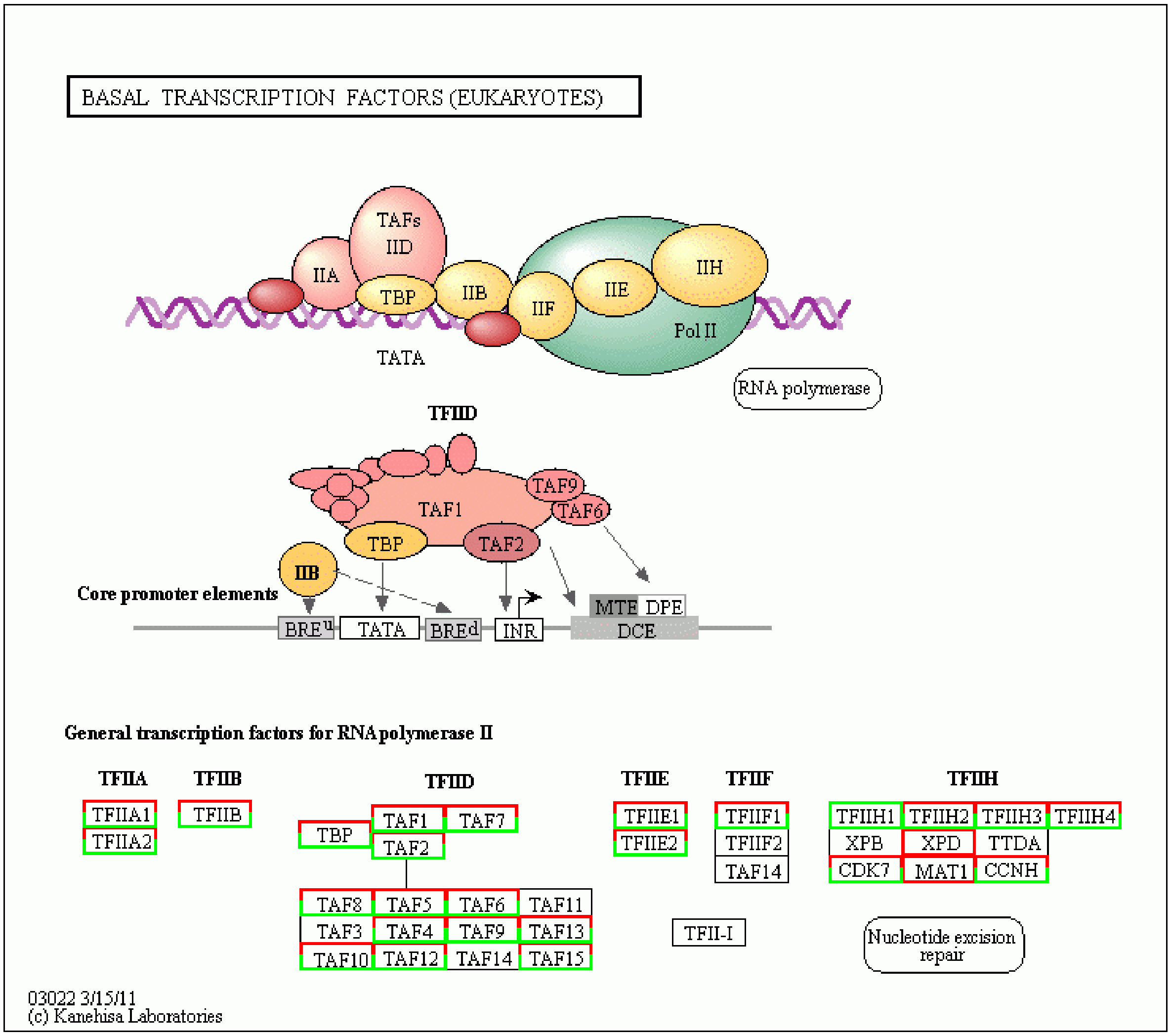
BASAL TRANSCRIPTION FACTORS (EUKARYOTES)
TAFs
IID
IIA
TBP
IIB
IIF
IIE
IIH
Pol II
TATA
RNA polymerase
TFIID
TAF9
TAF6
TAF1
TBP
TAF2
IIB
Core promoter elements
BREu
TATA
BREd
INR
MTE
DPE
DCE
General transcription factors for RNA polymerase II
TFIIA
TFIIA1
TFIIA2
TFIIB
TFIIB
TFIID
TBP
TAF1
TAF7
TAF2
TAF8
TAF5
TAF6
TAF11
TAF3
TAF4
TAF9
TAF13
TAF10
TAF12
TAF14
TAF15
TFIIE
TFIIE1
TFIIE2
TFIIF
TFIIF1
TFIIF2
TAF14
TFII-I
TFIIH
TFIIH1
TFIIH2
TFIIH3
TFIIH4
XPB
XPD
TTDA
CDK7
MAT1
CCNH
Nucleotide excision repair
03022 3/15/11
(c) Kanehisa Laboratories

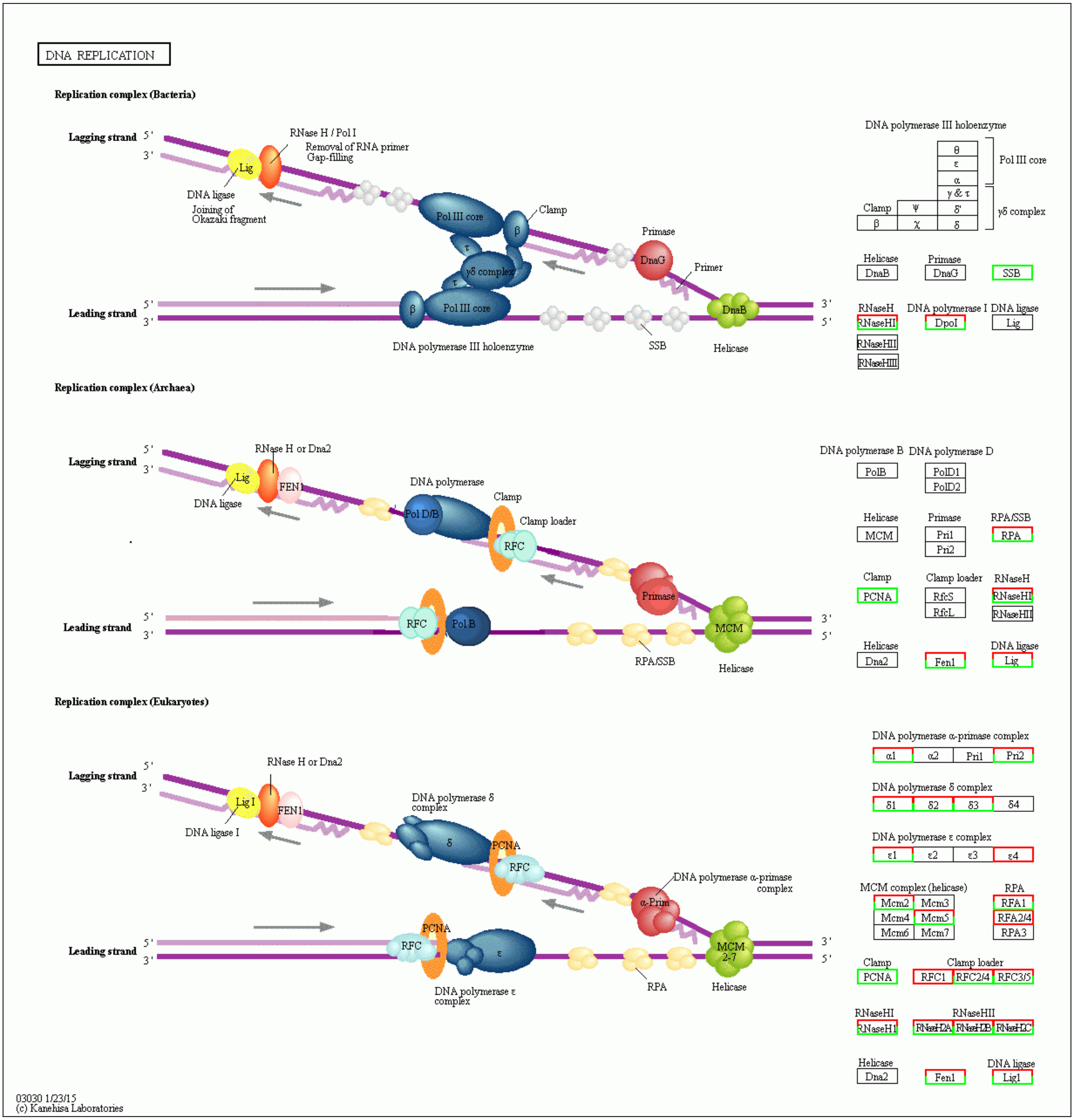
DNA REPLICATION
Replication complex (Bacteria)
Lagging strand
Leading strand
RNase H / Pol I
Removal of RNA primer
Gap-filling
Lig
DNA ligase
Joining of
Okazaki fragment
Pol III core
Clamp
β
τ
γδ complex
Primase
DnaG
Primer
DnaB
DNA polymerase III holoenzyme
SSB
Helicase
θ
ε
α
γ & τ
Ψ
δ'
χ
δ
Pol III core
γδ complex
Helicase
DnaB
Primase
DnaG
SSB
RNaseH
RNaseHI
RNaseHII
RNaseHIII
DNA polymerase I
DpoI
DNA ligase
Lig
Replication complex (Archaea)
RNase H or Dna2
FEN1
DNA polymerase
Pol D/B
Clamp loader
RFC
RPA/SSB
MCM
Pol B
DNA polymerase B
PolB
DNA polymerase D
PolD1
PolD2
MCM
Pri1
Pri2
RPA
PCNA
RfcS
RfcL
Dna2
Fen1
Replication complex (Eukaryotes)
Lig I
DNA ligase I
DNA polymerase δ
complex
PCNA
DNA polymerase α-primase
complex
α-Prim
MCM
2-7
DNA polymerase ε
complex
RPA
DNA polymerase α-primase complex
α1
α2
Pri1
Pri2
DNA polymerase δ complex
δ1
δ2
δ3
δ4
DNA polymerase ε complex
ε1
ε2
ε3
ε4
MCM complex (helicase)
Mcm2
Mcm3
Mcm4
Mcm5
Mcm6
Mcm7
RFA1
RFA2/4
RFA3
RFC1
RFC2/4
RFC3/5
RNaseHI
RNaseH1
RNaseHII
RNaseH2A
RNaseH2B
RNaseH2C
Lig1
03030 1/23/15
(c) Kanehisa Laboratories

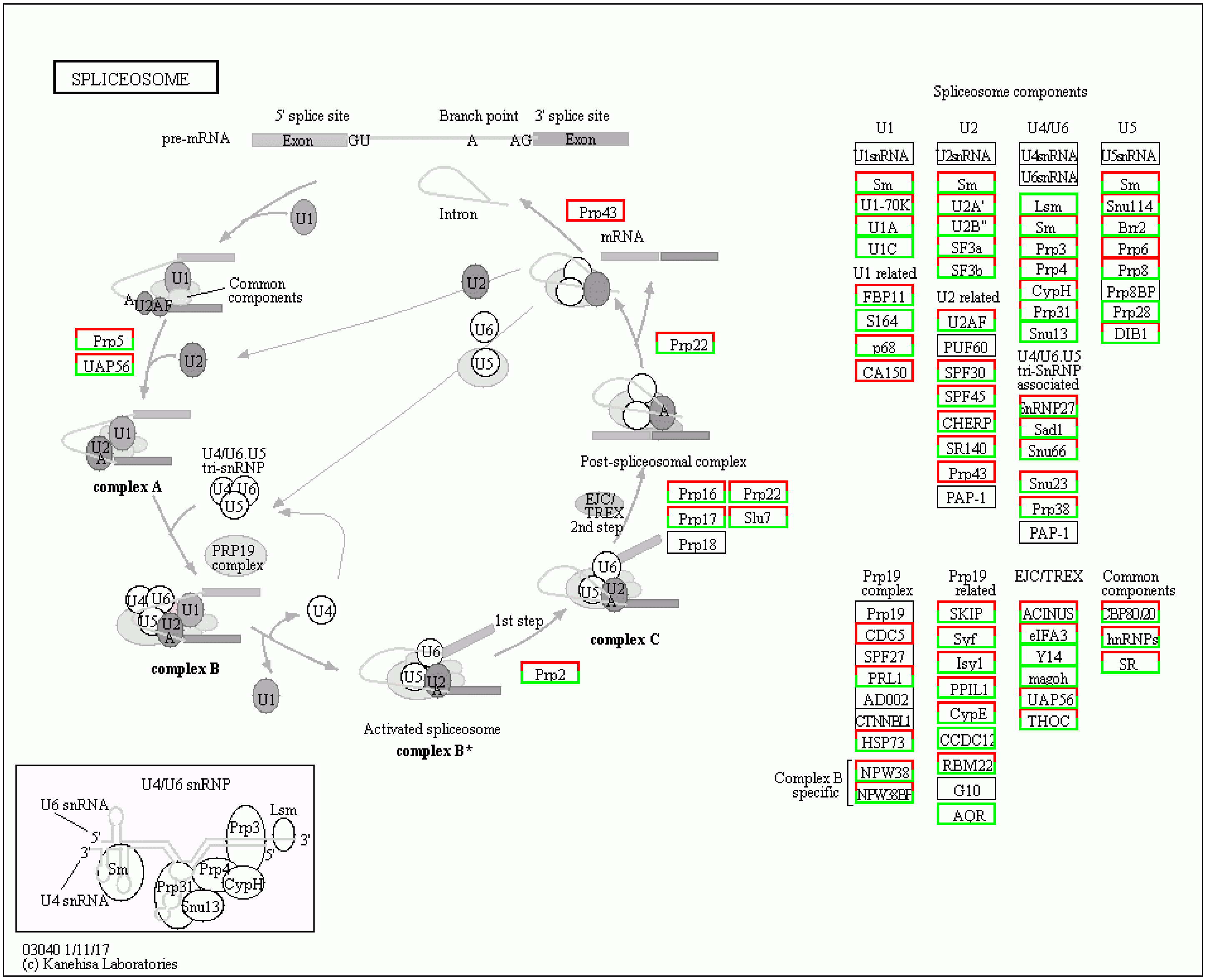

SPLICEOSOME
5' splice site
Branch point
3' splice site
pre-mRNA
Exon
GU
A
AG
Exon
Intron
Prp43
mRNA
Common components
Prp5
UAP56
Prp22
complex A
U4/U6.U5 tri-snRNP
PRP19 complex
Post-spliceosomal complex
EJC/ TREX
2nd step
Prp16
Prp22
Prp17
Slu7
Prp18
complex B
1st step
complex C
Prp2
Activated spliceosome
complex B*
U4/U6 snRNP
U6 snRNA
Lsm
Prp3
Sm
Prp4
CypH
Prp31
Snu13
U4 snRNA
Spliceosome components
U1
U2
U4/U6
U5
U1snRNA
Sm
U1-70K
U1A
U1C
U1 related
FBP11
S164
p68
CA150
U2snRNA
Sm
U2A'
U2B"
SF3a
SF3b
U2 related
U2AF
PUF60
SPF30
SPF45
CHERP
SR140
Prp43
PAP-1
U4snRNA
U6snRNA
Lsm
Sm
Prp3
Prp4
CypH
Prp31
Snu13
U4/U6.U5 tri-SnRNP associated
snRNP27
Sad1
Snu66
Snu23
Prp38
PAP-1
U5snRNA
Sm
Snu114
Brr2
Prp6
Prp8
Prp8BP
Prp28
DIB1
Prp19 complex
Prp19
CDC5
SPF27
PRL1
AD002
CTNNBL1
HSP73
Complex B specific
NPW38
NPW38BP
Prp19 related
SKIP
Syf
Isy1
PPIL1
CypE
CCDC12
RBM22
G10
AQR
EJC/TREX
ACINUS
eIFA3
Y14
magoh
UAP56
THOC
Common components
CBP80/20
hnRNPs
SR
03040 1/11/17
(c) Kanehisa Laboratories

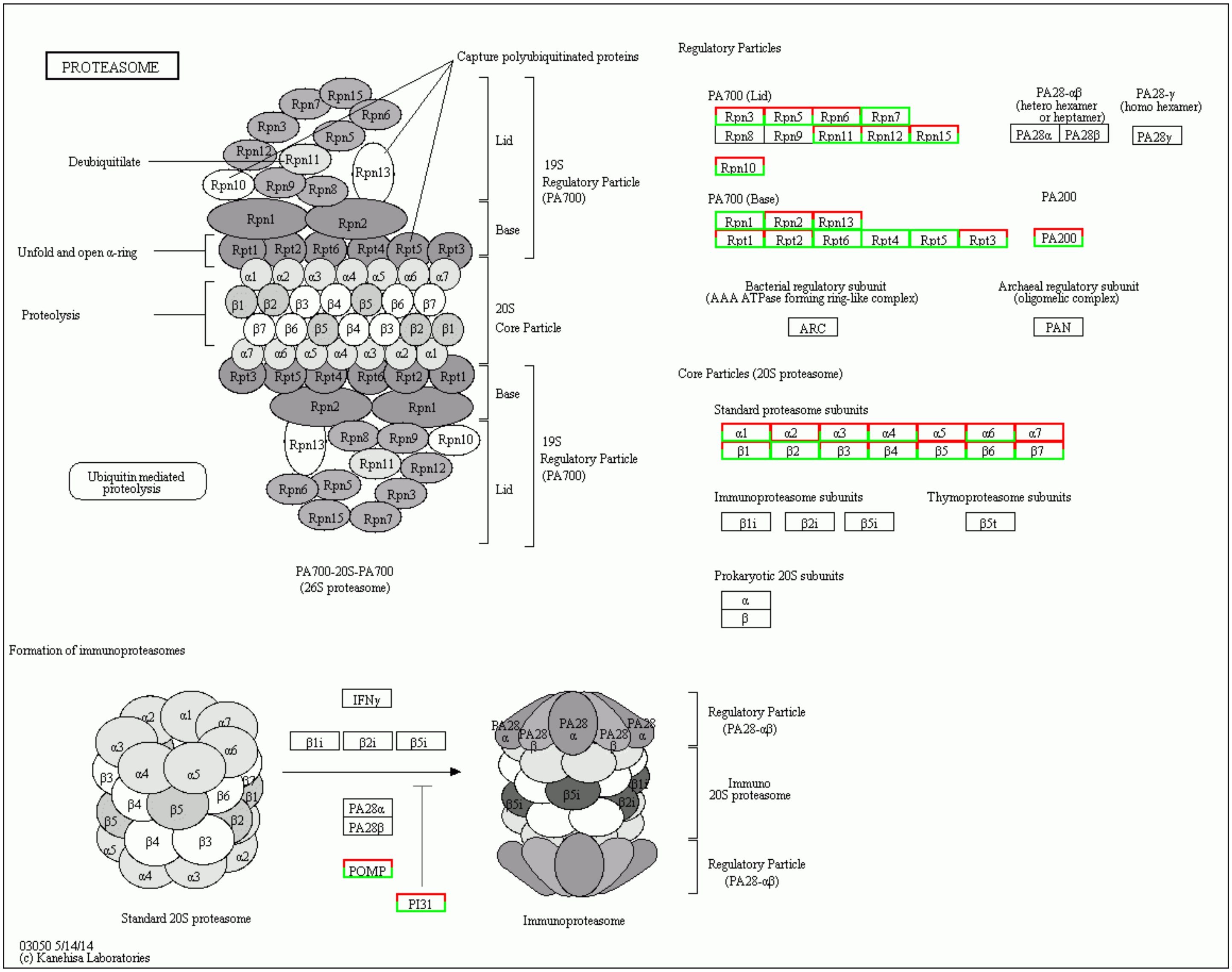
PROTEASOME
Capture polyubiquitinated proteins
Deubiquitilate
Unfold and open α-ring
Proteolysis
Ubiquitin mediated proteolysis
Rpn15
Rpn7
Rpn6
Rpn3
Rpn5
Rpn12
Rpn11
Rpn13
Rpn10
Rpn9
Rpn8
Rpn1
Rpn2
Rpt1
Rpt2
Rpt6
Rpt4
Rpt5
Rpt3
α1 α2 α3 α4 α5 α6 α7
β1 β2 β3 β4 β5 β6 β7
Lid
Base
19S
Regulatory Particle
(PA700)
20S
Core Particle
PA700-20S-PA700
(26S proteasome)
Regulatory Particles
PA700 (Lid)
Rpn3 Rpn5 Rpn6 Rpn7
Rpn8 Rpn9 Rpn11 Rpn12 Rpn15
Rpn10
PA700 (Base)
Rpn1 Rpn2 Rpn13
Rpt1 Rpt2 Rpt6 Rpt4 Rpt5 Rpt3
PA28-αβ
(hetero hexamer
or heptamer)
PA28α PA28β
PA28-γ
(homo hexamer)
PA28γ
PA200
PA200
Bacterial regulatory subunit
(AAA ATPase forming ring-like complex)
ARC
Archaeal regulatory subunit
(oligomelic complex)
PAN
Core Particles (20S proteasome)
Standard proteasome subunits
α1 α2 α3 α4 α5 α6 α7
β1 β2 β3 β4 β5 β6 β7
Immunoproteasome subunits
β1i β2i β5i
Thymoproteasome subunits
β5t
Prokaryotic 20S subunits
α
β
Formation of immunoproteasomes
IFNγ
β1i β2i β5i
PA28α
PA28β
POMP
PI31
Standard 20S proteasome
Immunoproteasome
PA28 α
PA28 β
β5i
β1i
β2i
Regulatory Particle
(PA28-αβ)
Immuno
20S proteasome
Regulatory Particle
(PA28-αβ)
03050 5/14/14
(c) Kanehisa Laboratories

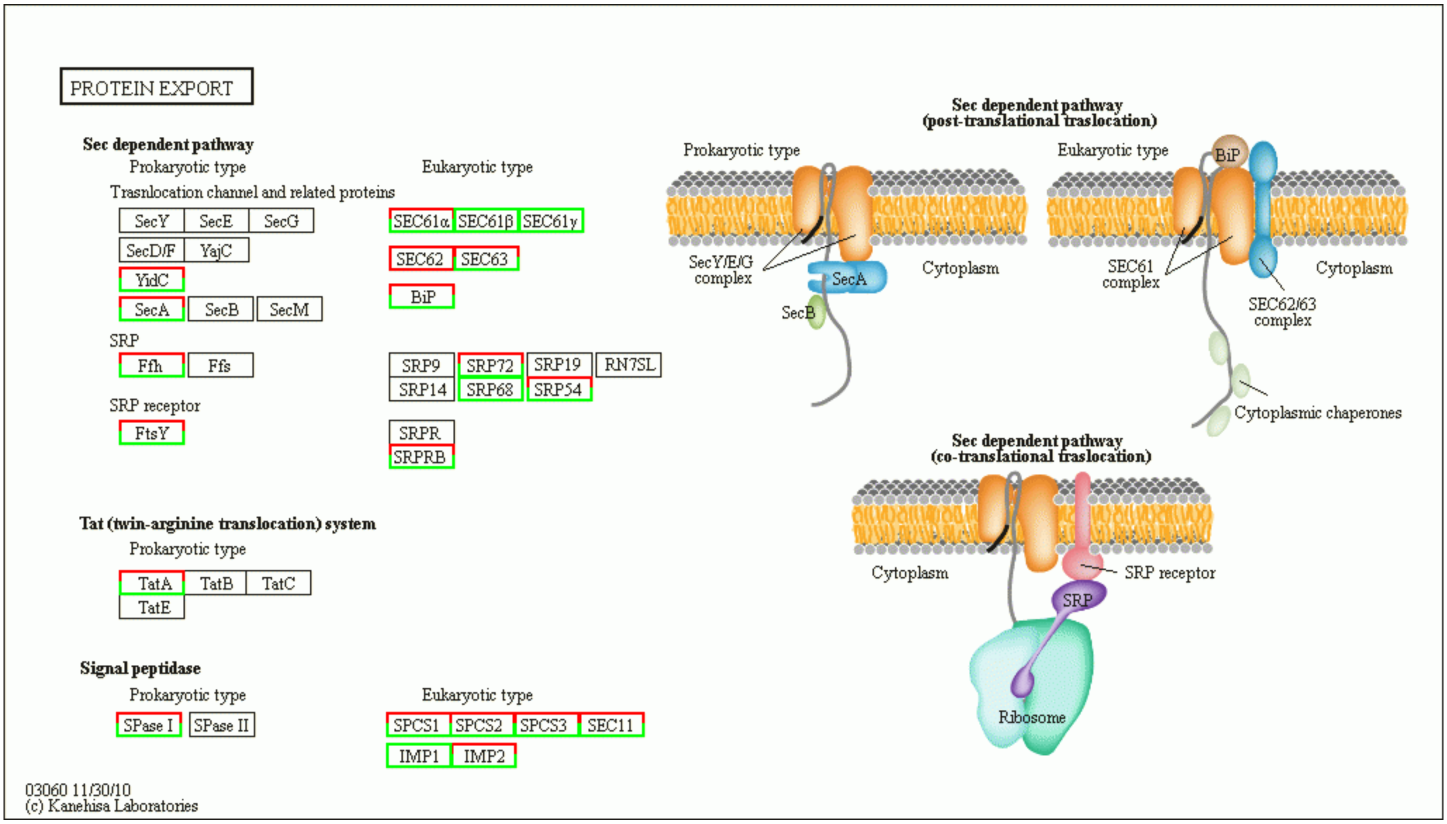
PROTEIN EXPORT
Sec dependent pathway
Prokaryotic type
Trasnlocation channel and related proteins
SecY
SecE
SecG
SecD/F
YajC
YidC
SecA
SecB
SecM
SRP
Ffh
Ffs
SRP receptor
FtsY
Eukaryotic type
SEC61α
SEC61β
SEC61γ
SEC62
SEC63
BiP
SRP9
SRP72
SRP19
RN7SL
SRP14
SRP68
SRP54
SRPR
SRPRB
Tat (twin-arginine translocation) system
Prokaryotic type
TatA
TatB
TatC
TatE
Signal peptidase
Prokaryotic type
SPase I
SPase II
Eukaryotic type
SPCS1
SPCS2
SPCS3
SEC11
IMP1
IMP2
Sec dependent pathway
(post-translational traslocation)
Prokaryotic type
SecY/E/G
complex
SecA
SecB
Cytoplasm
Eukaryotic type
BiP
SEC61
complex
SEC62/63
complex
Cytoplasm
Cytoplasmic chaperones
Sec dependent pathway
(co-translational traslocation)
Cytoplasm
SRP receptor
SRP
Ribosome
03060 11/30/10
(c) Kanehisa Laboratories

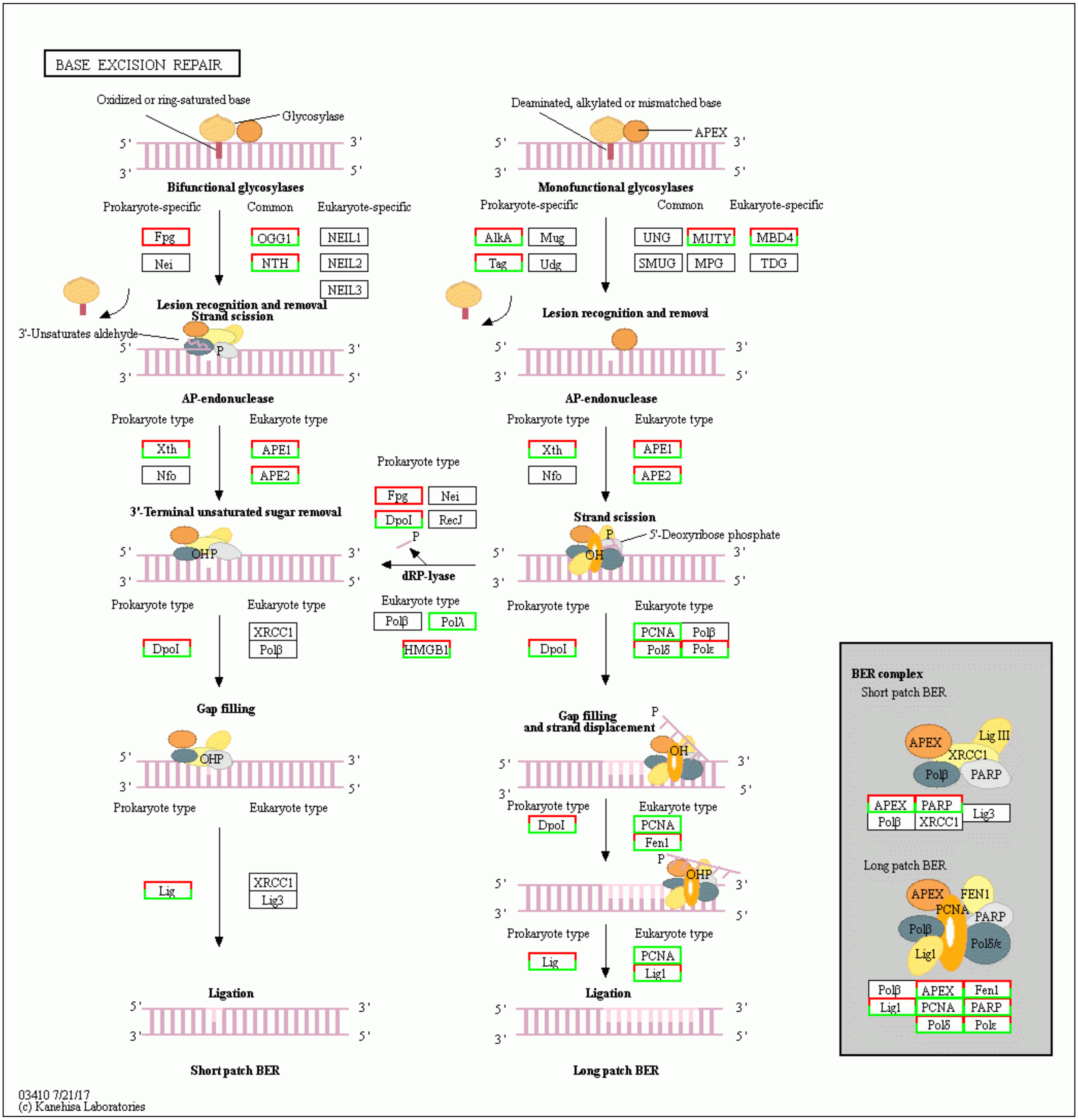
BASE EXCISION REPAIR
Oxidized or ring-saturated base
Glycosylase
Deaminated, alkylated or mismatched base
APEX
Bifunctional glycosylases
Prokaryote-specific
Common
Eukaryote-specific
Fpg
Nei
OGG1
NTH
NEIL1
NEIL2
NEIL3
Monofunctional glycosylases
AlkA
Mug
Tag
Udg
UNG
MUTY
MBD4
SMUG
MPG
TDG
Lesion recognition and removal
Strand scission
3'-Unsaturates aldehyde
AP-endonuclease
Prokaryote type
Eukaryote type
Xth
Nfo
APE1
APE2
Fpg
Nei
DpoI
RecJ
dRP-lyase
Polβ
Polλ
HMGB1
3'-Terminal unsaturated sugar removal
Strand scission
5'-Deoxyribose phosphate
XRCC1
PCNA
Polδ
Polε
Gap filling
Gap filling
and strand displacement
Fen1
Lig
Lig3
Lig1
Ligation
Short patch BER
Long patch BER
BER complex
Short patch BER
Lig III
XRCC1
PARP
APEX
Long patch BER
FEN1
PCNA
Polδ/ε
03410 7/21/17
(c) Kanehisa Laboratories

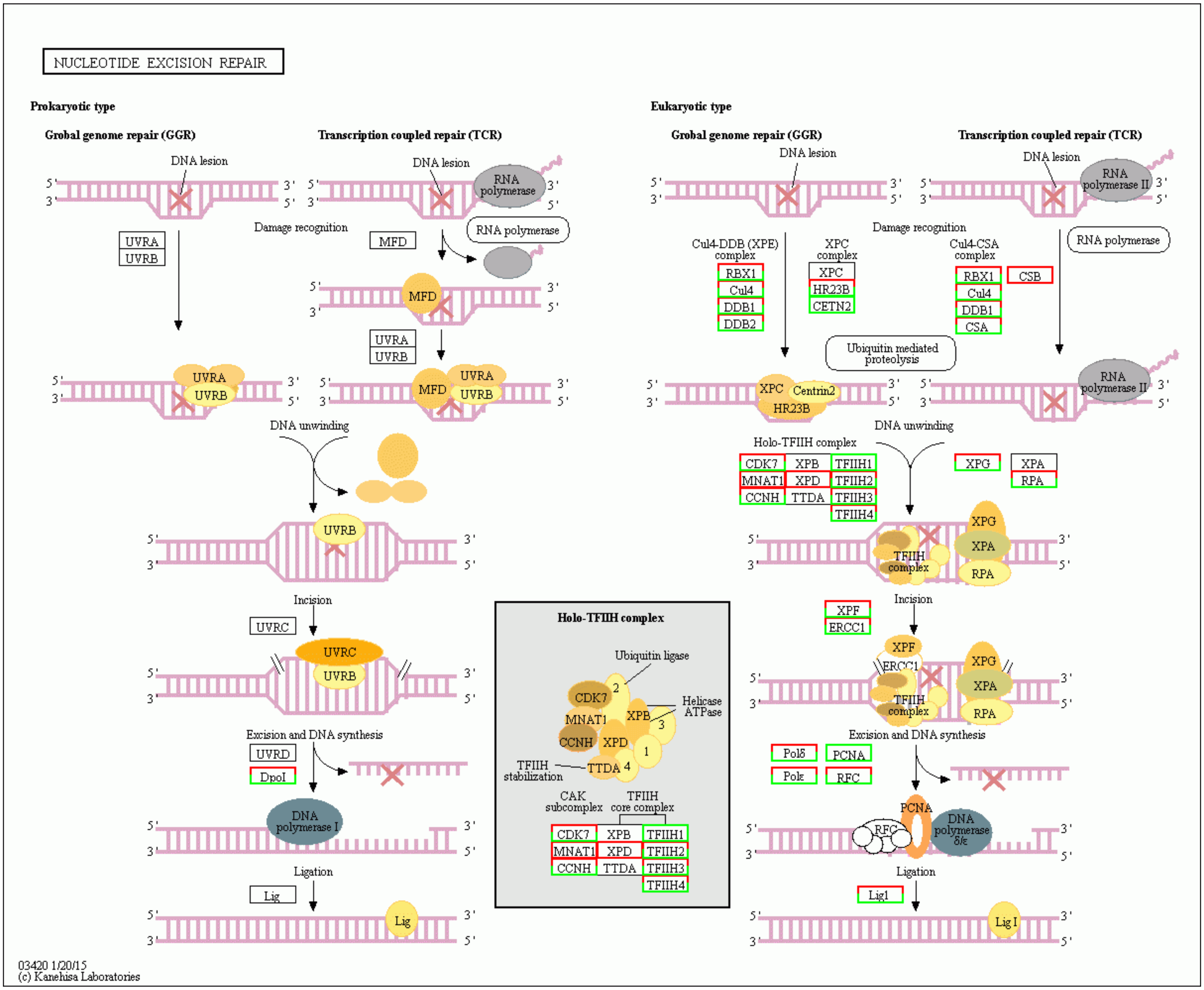
NUCLEOTIDE EXCISION REPAIR
Prokaryotic type
Grobal genome repair (GGR)
Transcription coupled repair (TCR)
DNA lesion
RNA polymerase
Damage recognition
UVRA
UVRB
MFD
DNA unwinding
Incision
UVRC
Excision and DNA synthesis
UVRD
DpoI
DNA polymerase I
Ligation
Lig
Eukaryotic type
Grobal genome repair (GGR)
Transcription coupled repair (TCR)
RNA polymerase II
Cul4-DDB (XPE) complex
RBX1
Cul4
DDB1
DDB2
XPC complex
XPC
HR23B
CETN2
Cul4-CSA complex
CSA
CSB
Ubiquitin mediated proteolysis
Centrin2
Holo-TFIIH complex
CDK7
XPB
TFIIH1
MNAT1
XPD
TFIIH2
CCNH
TTDA
TFIIH3
TFIIH4
XPG
XPA
RPA
TFIIH complex
XPF
ERCC1
Polδ
Polε
PCNA
RFC
DNA polymerase δ/ε
LigI
Lig I
Ubiquitin ligase
Helicase ATPase
TFIIH stabilization
CAK subcomplex
TFIIH core complex
03420 1/20/15
(c) Kanehisa Laboratories

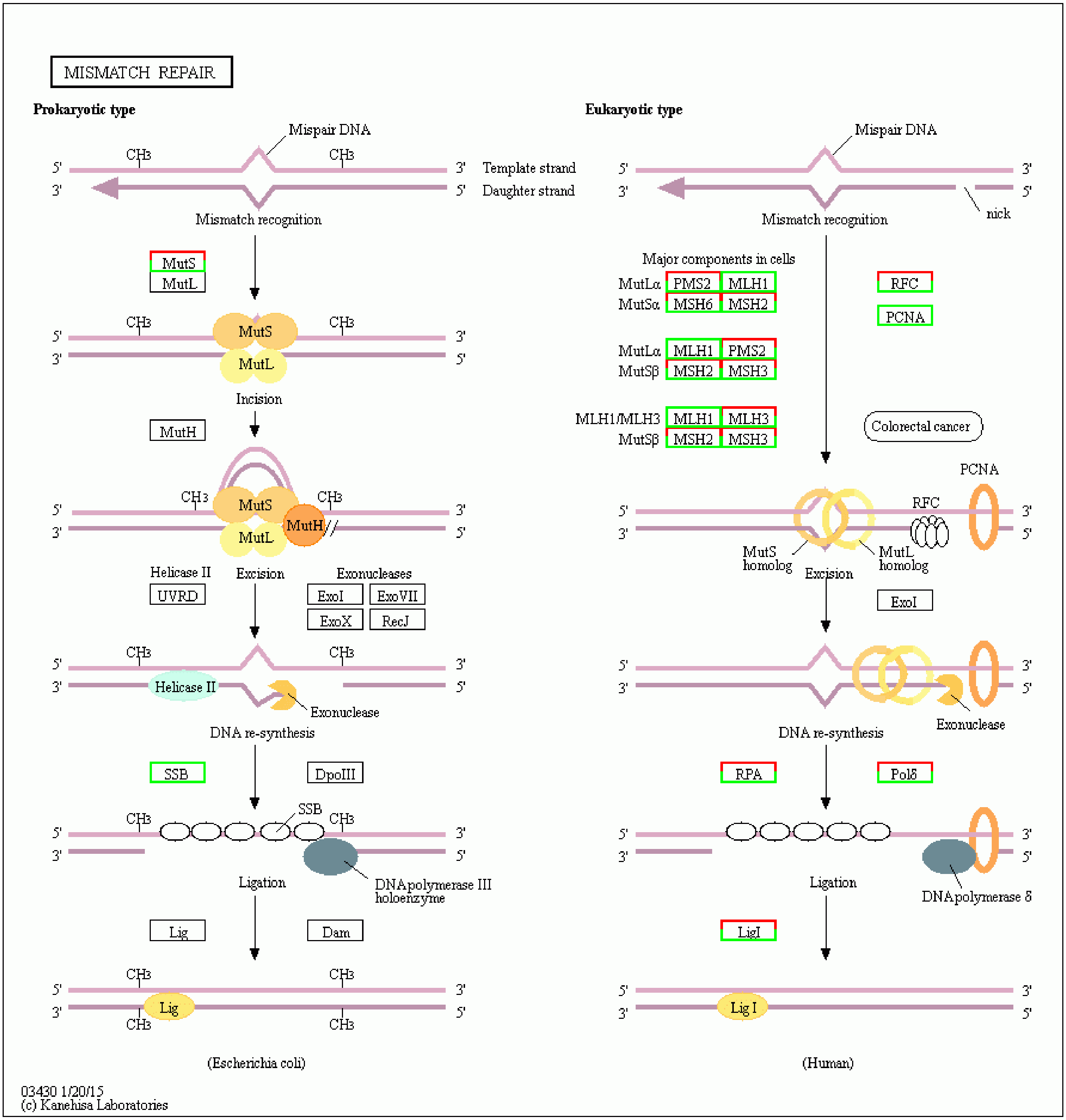
MISMATCH REPAIR
Prokaryotic type
Eukaryotic type
Mispair DNA
CH3
5'
3'
Template strand
Daughter strand
nick
Mismatch recognition
MutS
MutL
Major components in cells
MutLα PMS2 MLH1
MutSα MSH6 MSH2
MutLα MLH1 PMS2
MutSβ MSH2 MSH3
MLH1/MLH3 MLH1 MLH3
MutSβ MSH2 MSH3
RFC
PCNA
Colorectal cancer
Incision
MutH
MutS homolog
MutL homolog
Helicase II
UVRD
Excision
Exonucleases
ExoI
ExoVII
ExoX
RecJ
Exonuclease
DNA re-synthesis
SSB
DpoIII
RPA
Polδ
DNApolymerase III holoenzyme
DNApolymerase δ
Ligation
Lig
Dam
LigI
Lig I
(Escherichia coli)
(Human)
03430 1/20/15
(c) Kanehisa Laboratories

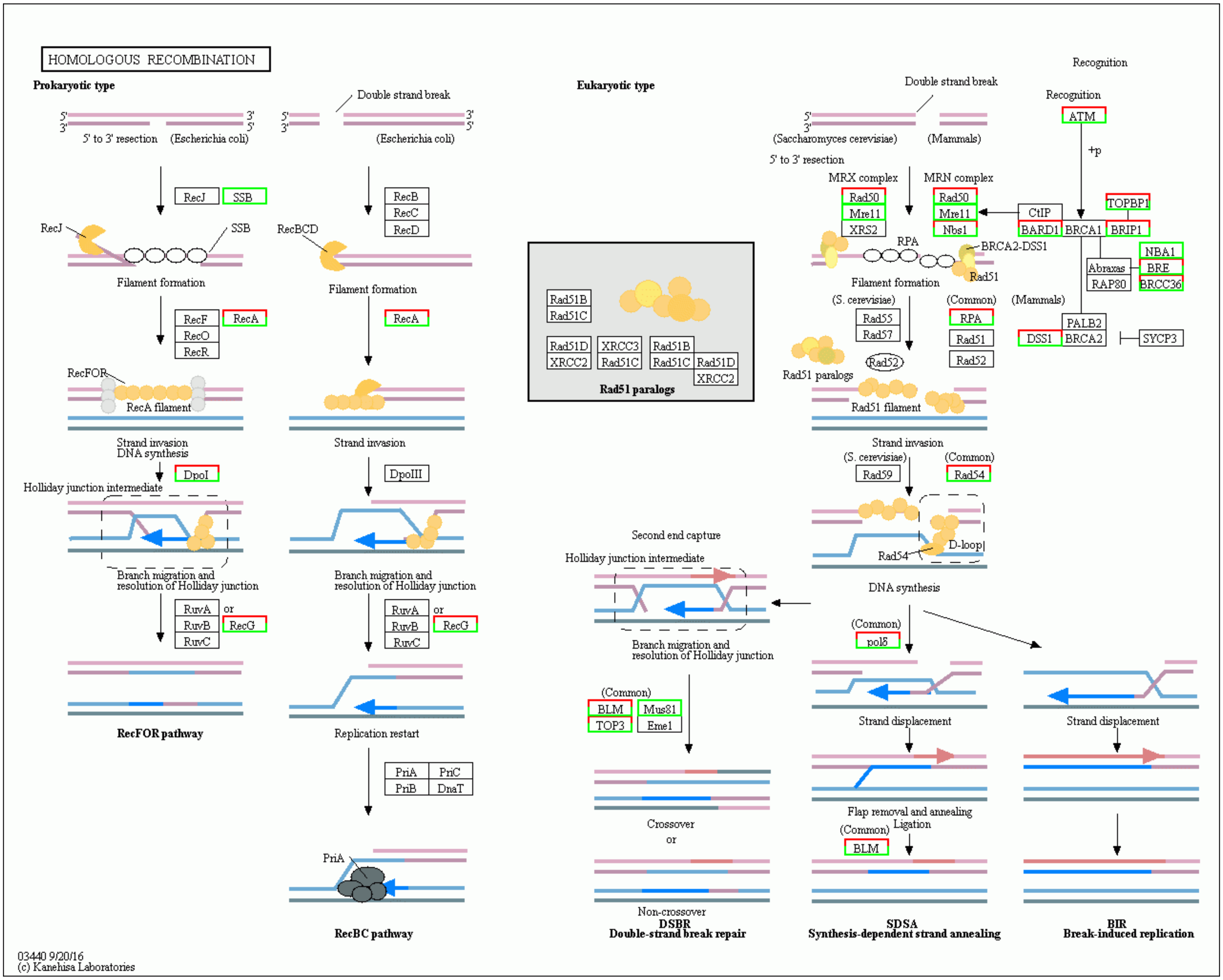

HOMOLOGOUS RECOMBINATION
Prokaryotic type
Eukaryotic type
Double strand break
5' to 3' resection
(Escherichia coli)
RecJ
SSB
RecB
RecC
RecD
RecBCD
Filament formation
RecF
RecO
RecR
RecA
RecFOR
RecA filament
Strand invasion
DNA synthesis
DpoI
DpoIII
Holliday junction intermediate
Branch migration and resolution of Holliday junction
RuvA
RuvB
RuvC
or
RecG
RecFOR pathway
Replication restart
PriA
PriB
PriC
DnaT
RecBC pathway
Rad51B
Rad51C
Rad51D
XRCC2
XRCC3
Rad51 paralogs
(Saccharomyces cerevisiae)
(Mammals)
Recognition
ATM
+p
MRX complex
MRN complex
Rad50
Mre11
XRS2
Nbs1
CtIP
TOPBP1
BARD1
BRCA1
BRIP1
RPA
BRCA2-DSS1
Rad51
NBA1
Abraxas
BRE
RAP80
BRCC36
(S. cerevisiae)
(Common)
Rad55
Rad57
Rad52
PALB2
DSS1
BRCA2
SYCP3
Rad51 filament
Rad59
Rad54
D-loop
Second end capture
DNA synthesis
polδ
BLM
TOP3
Mus81
Eme1
Crossover
or
Non-crossover
DSBR
Double-strand break repair
Strand displacement
Flap removal and annealing
Ligation
SDSA
Synthesis-dependent strand annealing
BIR
Break-induced replication
03440 9/20/16
(c) Kanehisa Laboratories

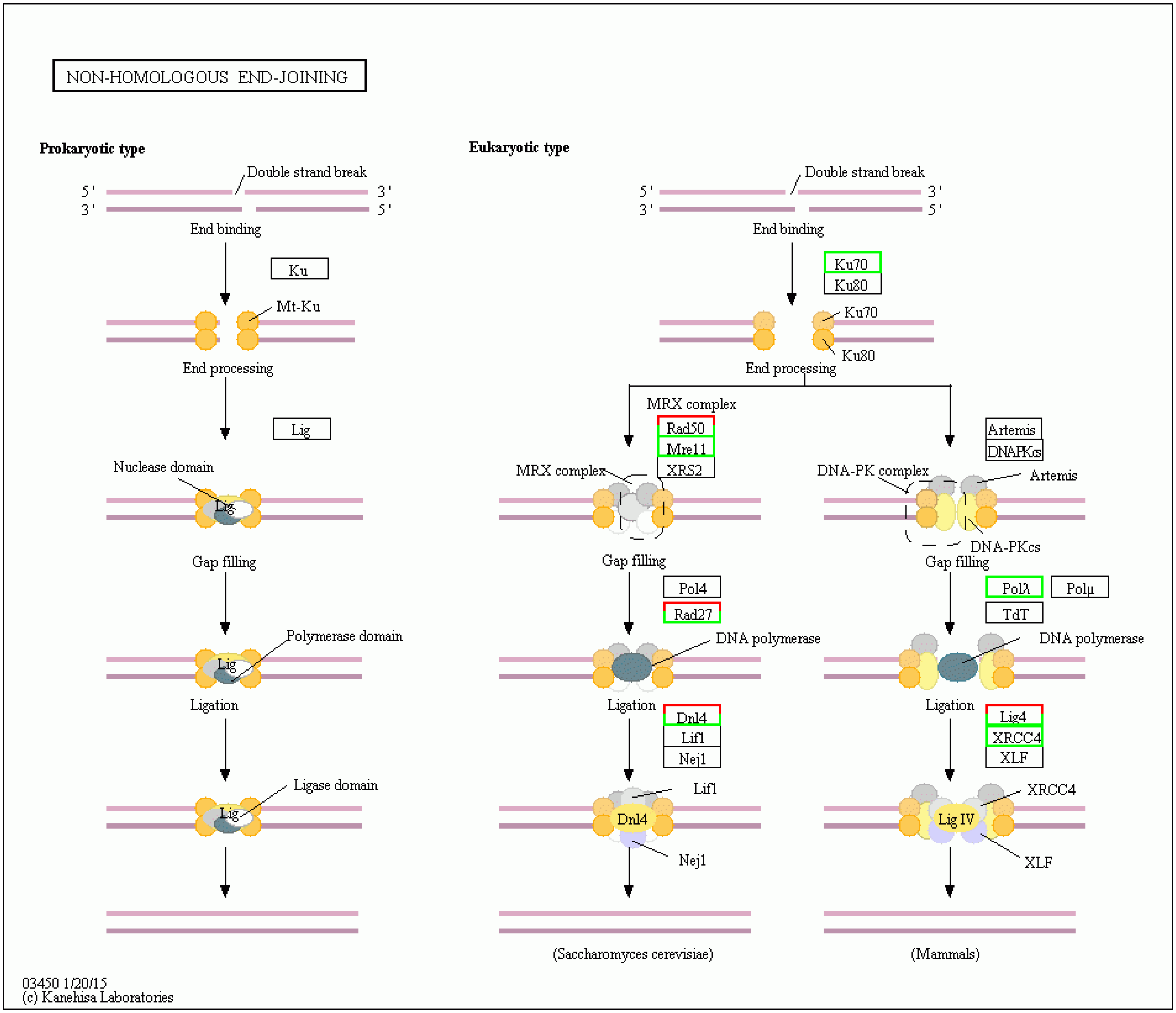
NON-HOMOLOGOUS END-JOINING
Prokaryotic type
Eukaryotic type
Double strand break
5'
3'
End binding
Ku
Mt-Ku
End processing
Lig
Nuclease domain
Gap filling
Polymerase domain
Ligation
Ligase domain
Ku70
Ku80
MRX complex
Rad50
Mre11
XRS2
Artemis
DNAPKcs
DNA-PK complex
DNA-PKcs
Pol4
Rad27
DNA polymerase
Polλ
Polμ
TdT
Dnl4
Lif1
Nej1
Lig4
XRCC4
XLF
Lig IV
(Saccharomyces cerevisiae)
(Mammals)
03450 1/20/15
(c) Kanehisa Laboratories

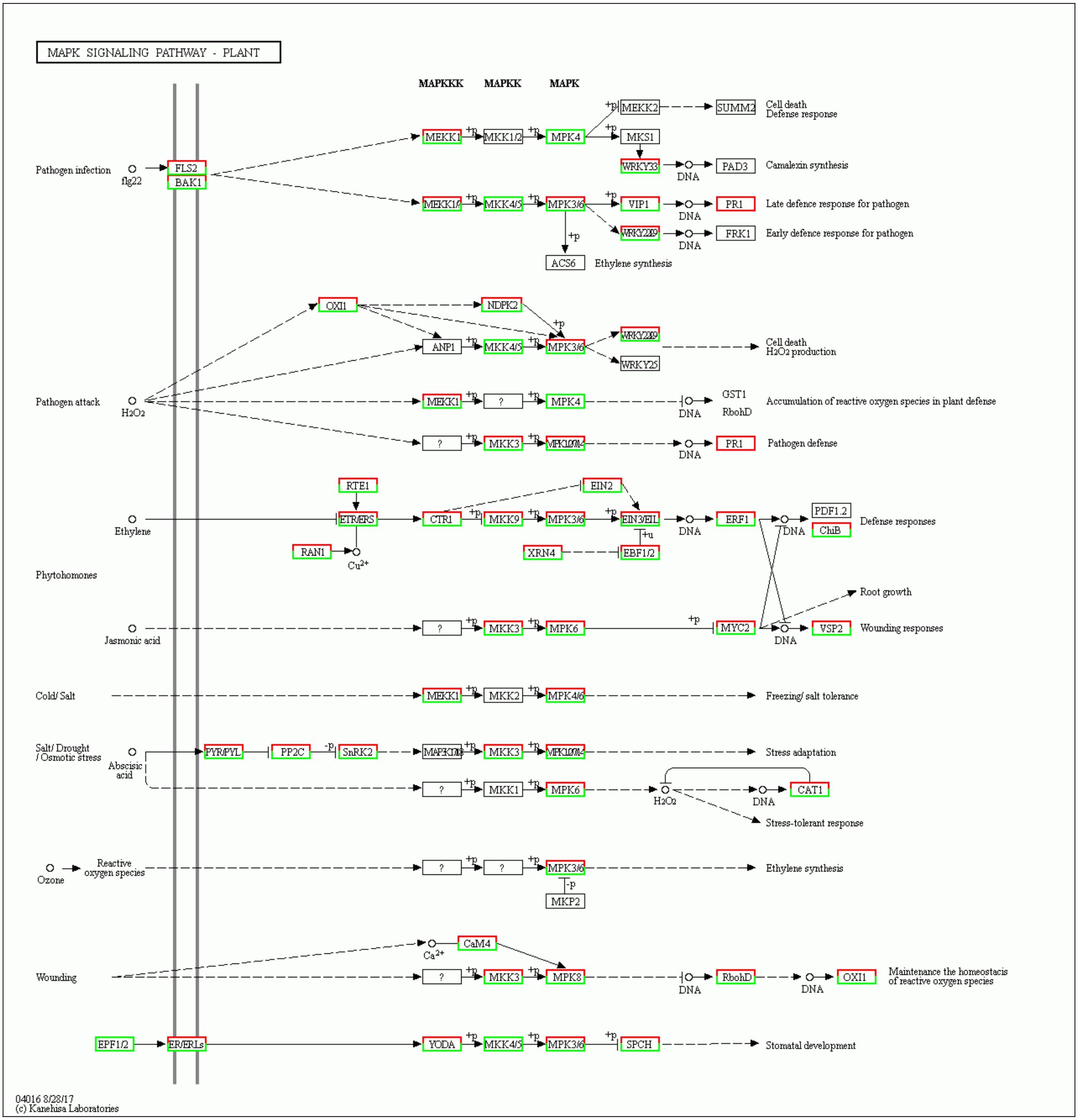
MAPK SIGNALING PATHWAY - PLANT
MAPKKK
MAPKK
MAPK
Pathogen infection
flg22
FLS2
BAK1
MEKK1
MKK1/2
MPK4
MEKK2
SUMM2
Cell death
Defense response
MKS1
WRKY33
DNA
PAD3
Camalexin synthesis
MKK4/5
MPK3/6
VIP1
PR1
Late defence response for pathogen
FRK1
Early defence response for pathogen
ACS6
Ethylene synthesis
OXI1
NDPK2
ANP1
WRKY25
Cell death
H2O2 production
Pathogen attack
H2O2
GST1
RbohD
Accumulation of reactive oxygen species in plant defense
MKK3
Pathogen defense
RTE1
EIN2
Ethylene
ETR/ERS
CTR1
MKK9
EIN3/EIL
ERF1
PDF1.2
ChiB
Defense responses
RAN1
Cu2+
XRN4
EBF1/2
Phytohomones
Root growth
Jasmonic acid
MPK6
MYC2
VSP2
Wounding responses
Cold/ Salt
MKK2
MPK4/6
Freezing/ salt tolerance
Salt/ Drought / Osmotic stress
Abscisic acid
PYR/PYL
PP2C
SnRK2
Stress adaptation
MKK1
CAT1
Stress-tolerant response
Ozone
Reactive oxygen species
Ethylene synthesis
MKP2
Wounding
Ca2+
CaM4
MPK8
RbohD
Maintenance the homeostacis of reactive oxygen species
EPF1/2
ER/ERLs
YODA
SPCH
Stomatal development
04016 8/28/17
(c) Kanehisa Laboratories

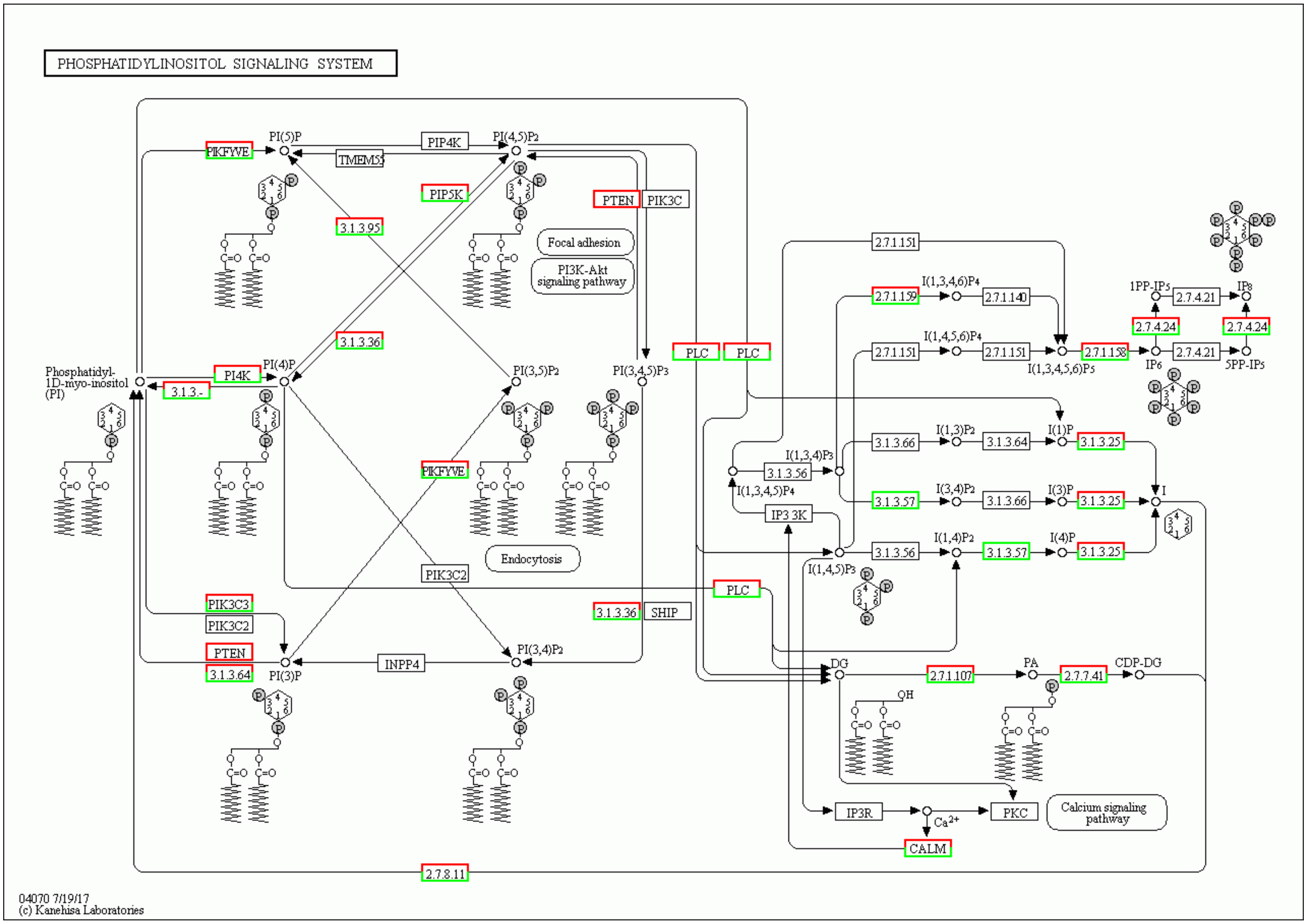
PHOSPHATIDYLINOSITOL SIGNALING SYSTEM
PI(5)P
PIP4K
PI(4,5)P2
PIKFYVE
TMEM55
PIP5K
PTEN
PIK3C
3.1.3.95
Focal adhesion
PI3K-Akt signaling pathway
3.1.3.36
PLC
PLC
Phosphatidyl-1D-myo-inositol (PI)
PI4K
PI(4)P
3.1.3.-
PI(3,5)P2
PI(3,4,5)P3
PIKFYVE
Endocytosis
PIK3C2
PIK3C3
PIK3C2
3.1.3.36
SHIP
PTEN
3.1.3.64
PI(3)P
INPP4
PI(3,4)P2
2.7.1.151
I(1,3,4,6)P4
2.7.1.159
2.7.1.140
I(1,4,5,6)P4
2.7.1.151
2.7.1.151
2.7.1.158
I(1,3,4,5,6)P5
1PP-IP5
2.7.4.21
IP8
2.7.4.24
2.7.4.24
IP6
2.7.4.21
5PP-IP5
I(1,3,4)P3
3.1.3.56
I(1,3,4,5)P4
3.1.3.66
I(1,3)P2
3.1.3.64
I(1)P
3.1.3.25
3.1.3.57
I(3,4)P2
3.1.3.66
I(3)P
3.1.3.25
I
IP3 3K
I(1,4,5)P3
3.1.3.56
I(1,4)P2
3.1.3.57
I(4)P
3.1.3.25
PLC
DG
2.7.1.107
PA
2.7.7.41
CDP-DG
OH
IP3R
Ca2+
PKC
Calcium signaling pathway
CALM
2.7.8.11
04070 7/19/17
(c) Kanehisa Laboratories

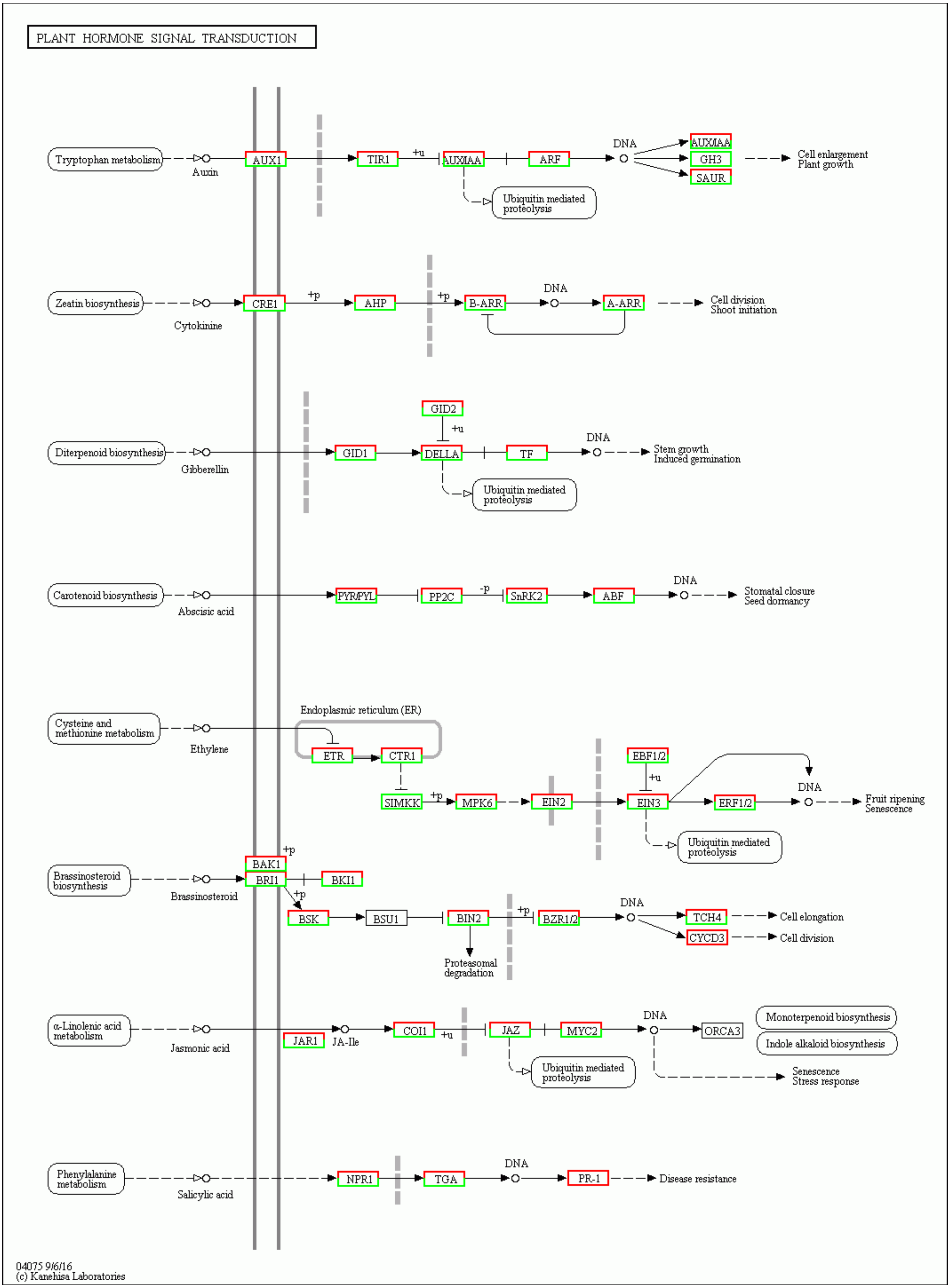
PLANT HORMONE SIGNAL TRANSDUCTION
Tryptophan metabolism
Auxin
AUX1
TIR1
+u
AUX/IAA
ARF
DNA
AUX/IAA
GH3
SAUR
Cell enlargement
Plant growth
Ubiquitin mediated proteolysis
Zeatin biosynthesis
Cytokinine
CRE1
+p
AHP
+p
B-ARR
DNA
A-ARR
Cell division
Shoot initiation
GID2
+u
Diterpenoid biosynthesis
Gibberellin
GID1
DELLA
TF
DNA
Stem growth
Induced germination
Ubiquitin mediated proteolysis
Carotenoid biosynthesis
Abscisic acid
PYR/PYL
PP2C
-p
SnRK2
ABF
DNA
Stomatal closure
Seed dormancy
Endoplasmic reticulum (ER)
Cysteine and methionine metabolism
Ethylene
ETR
CTR1
SIMKK
+p
MPK6
EIN2
EBF1/2
+u
EIN3
ERF1/2
DNA
Fruit ripening
Senescence
Ubiquitin mediated proteolysis
+p
BAK1
Brassinosteroid biosynthesis
Brassinosteroid
BRI1
BKI1
+p
BSK
BSU1
BIN2
+p
BZR1/2
DNA
TCH4
Cell elongation
CYCD3
Cell division
Proteasomal degradation
α-Linolenic acid metabolism
Jasmonic acid
JAR1
JA-Ile
COI1
+u
JAZ
MYC2
DNA
ORCA3
Monoterpenoid biosynthesis
Indole alkaloid biosynthesis
Ubiquitin mediated proteolysis
Senescence
Stress response
Phenylalanine metabolism
Salicylic acid
NPR1
TGA
DNA
PR-1
Disease resistance
04075 9/6/16
(c) Kanehisa Laboratories

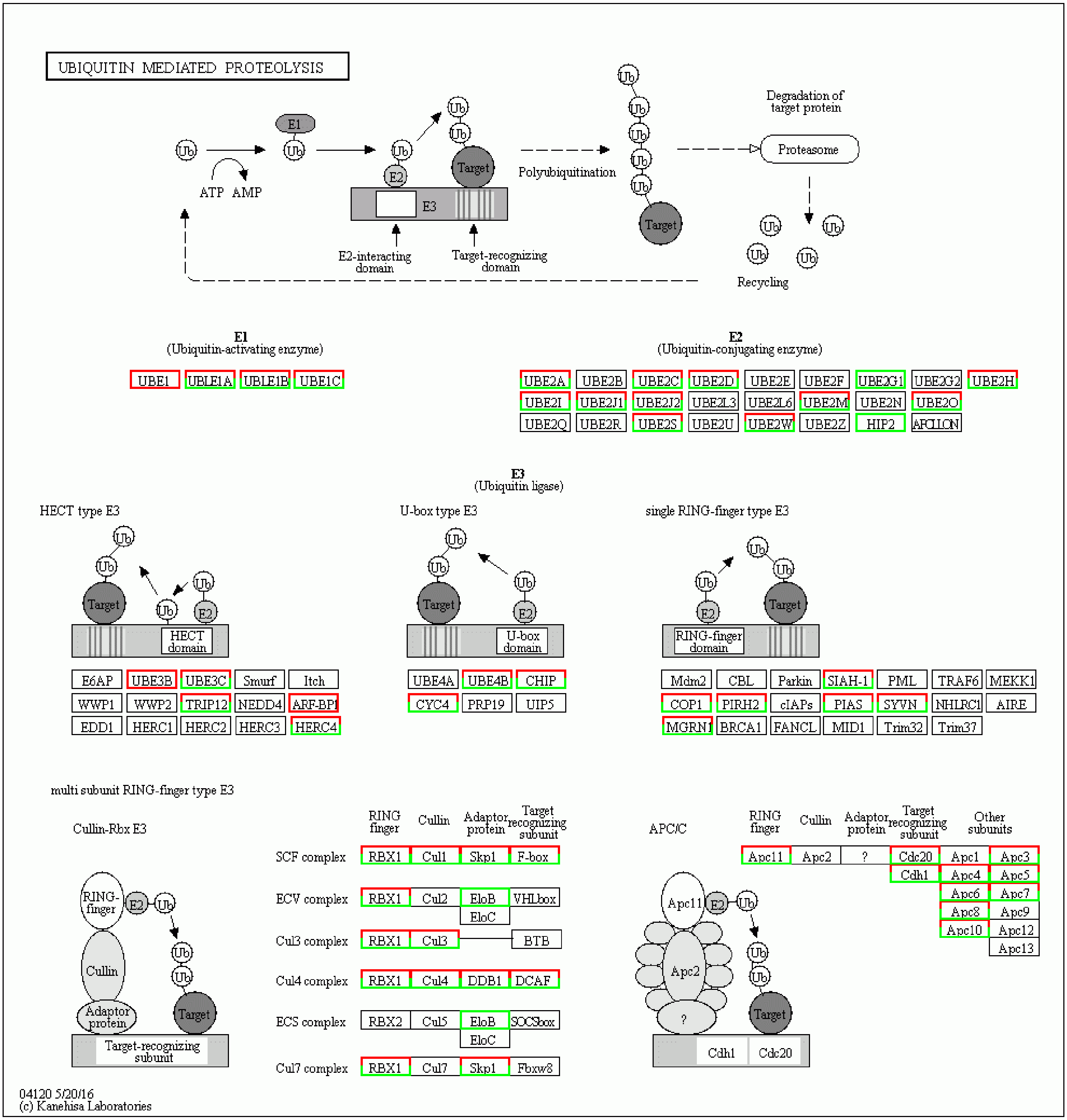
UBIQUITIN MEDIATED PROTEOLYSIS
Ub
E1
ATP AMP
E2
E3
Target
E2-interacting domain
Target-recognizing domain
Polyubiquitination
Degradation of target protein
Proteasome
Recycling
E1
(Ubiquitin-activating enzyme)
UBE1
UBLE1A
UBLE1B
UBE1C
E2
(Ubiquitin-conjugating enzyme)
UBE2A
UBE2B
UBE2C
UBE2D
UBE2E
UBE2F
UBE2G1
UBE2G2
UBE2H
UBE2I
UBE2J1
UBE2J2
UBE2L3
UBE2L6
UBE2M
UBE2N
UBE2O
UBE2Q
UBE2R
UBE2S
UBE2U
UBE2W
UBE2Z
HIP2
APOLLON
E3
(Ubiquitin ligase)
HECT type E3
HECT domain
E6AP
UBE3B
UBE3C
Smurf
Itch
WWP1
WWP2
TRIP12
NEDD4
ARF-BP1
EDD1
HERC1
HERC2
HERC3
HERC4
U-box type E3
U-box domain
UBE4A
UBE4B
CHIP
CYC4
PRP19
UIP5
single RING-finger type E3
RING-finger domain
Mdm2
CBL
Parkin
SIAH-1
PML
TRAF6
MEKK1
COP1
PIRH2
cIAPs
PIAS
SYVN
NHLRC1
AIRE
MGRN1
BRCA1
FANCL
MID1
Trim32
Trim37
multi subunit RING-finger type E3
Cullin-Rbx E3
RING-finger
Cullin
Adaptor protein
Target-recognizing subunit
RING finger
Cullin
Adaptor protein
Target recognizing subunit
SCF complex
RBX1
Cul1
Skp1
F-box
ECV complex
RBX1
Cul2
EloB
VHLbox
EloC
Cul3 complex
RBX1
Cul3
BTB
Cul4 complex
RBX1
Cul4
DDB1
DCAF
ECS complex
RBX2
Cul5
EloB
SOCSbox
EloC
Cul7 complex
RBX1
Cul7
Skp1
Fbxw8
APC/C
Apc11
Apc2
?
Cdh1
Cdc20
RING finger
Cullin
Adaptor protein
Target recognizing subunit
Other subunits
Apc11
Apc2
?
Cdc20
Apc1
Apc3
Cdh1
Apc4
Apc5
Apc6
Apc7
Apc8
Apc9
Apc10
Apc12
Apc13
04120 5/20/16
(c) Kanehisa Laboratories

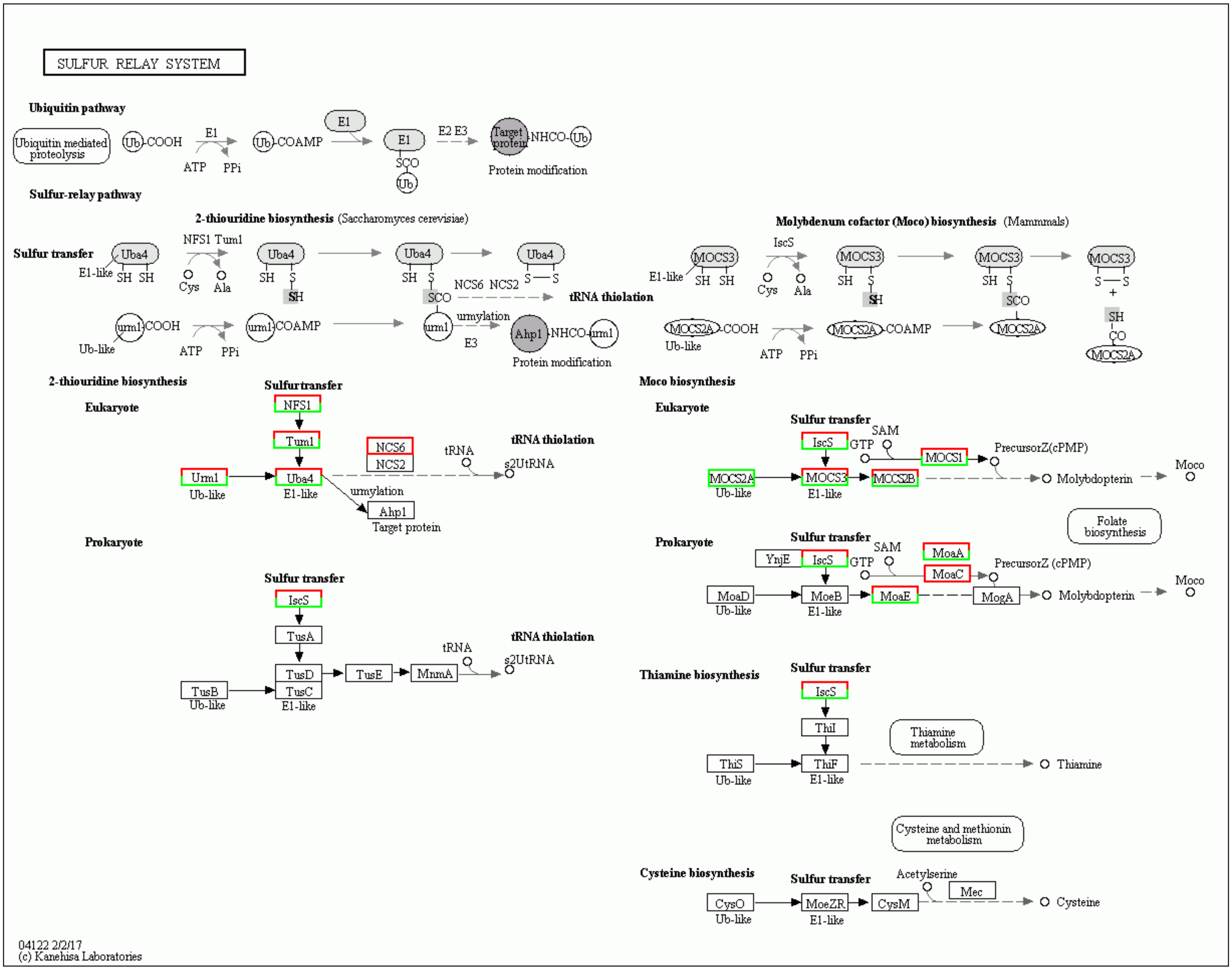
SULFUR RELAY SYSTEM
Ubiquitin pathway
Ubiquitin mediated proteolysis
Ub-COOH
E1
ATP
PPi
Ub-COAMP
E1
SCO
Ub
E2 E3
Target protein
NHCO-Ub
Protein modification
Sulfur-relay pathway
2-thiouridine biosynthesis (Saccharomyces cerevisiae)
Sulfur transfer
Uba4
E1-like
SH SH
NFS1 Tum1
Cys
Ala
NCS6 NCS2
tRNA thiolation
urm1-COOH
Ub-like
urm1-COAMP
urmylation
Ahp1
NHCO-urm1
E3
Protein modification
Molybdenum cofactor (Moco) biosynthesis (Mammals)
MOCS3
IscS
MOCS2A-COOH
Ub-like
MOCS2A-COAMP
MOCS2A
2-thiouridine biosynthesis
Sulfur transfer
Eukaryote
NFS1
Tum1
NCS6
NCS2
tRNA
s2UtRNA
Urm1
Ub-like
Uba4
E1-like
urmylation
Ahp1
Target protein
Prokaryote
IscS
TusA
TusD
TusC
TusB
TusE
MnmA
Moco biosynthesis
Eukaryote
SAM
GTP
MOCS1
PrecursorZ(cPMP)
MOCS2A
MOCS3
MOCS2B
Molybdopterin
Moco
Folate biosynthesis
Prokaryote
YnjE
MoaA
MoaC
PrecursorZ (cPMP)
MoaD
MoeB
MoaE
MogA
Thiamine biosynthesis
ThiI
Thiamine metabolism
ThiS
ThiF
Thiamine
Cysteine and methionin metabolism
Cysteine biosynthesis
Acetylserine
Mec
CysO
MoeZR
CysM
Cysteine
04122 2/2/17
(c) Kanehisa Laboratories

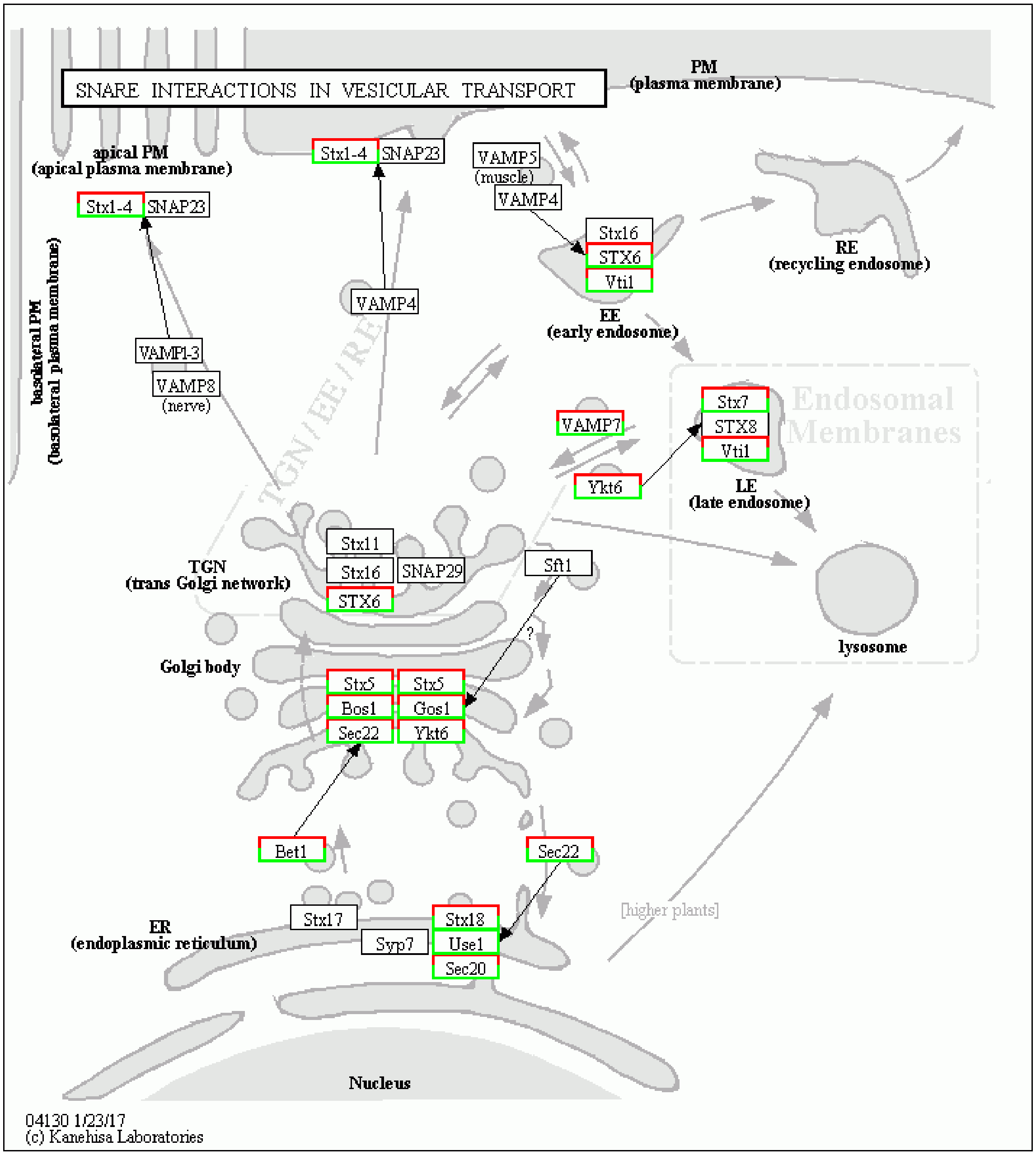
SNARE INTERACTIONS IN VESICULAR TRANSPORT
PM
(plasma membrane)
apical PM
(apical plasma membrane)
basolateral PM
(basolateral plasma membrane)
Stx1-4
SNAP23
Stx1-4
SNAP23
VAMP5
(muscle)
VAMP4
Stx16
STX6
Vti1
EE
(early endosome)
RE
(recycling endosome)
VAMP4
VAMP1-3
VAMP8
(nerve)
TGN / EE / RE
Endosomal Membranes
Stx7
STX8
Vti1
VAMP7
Ykt6
LE
(late endosome)
lysosome
Stx11
Stx16
SNAP29
STX6
TGN
(trans Golgi network)
Sft1
?
Golgi body
Stx5
Bos1
Sec22
Stx5
Gos1
Ykt6
Bet1
Sec22
[higher plants]
Stx17
Syp7
Stx18
Use1
Sec20
ER
(endoplasmic reticulum)
Nucleus
04130 1/23/17
(c) Kanehisa Laboratories

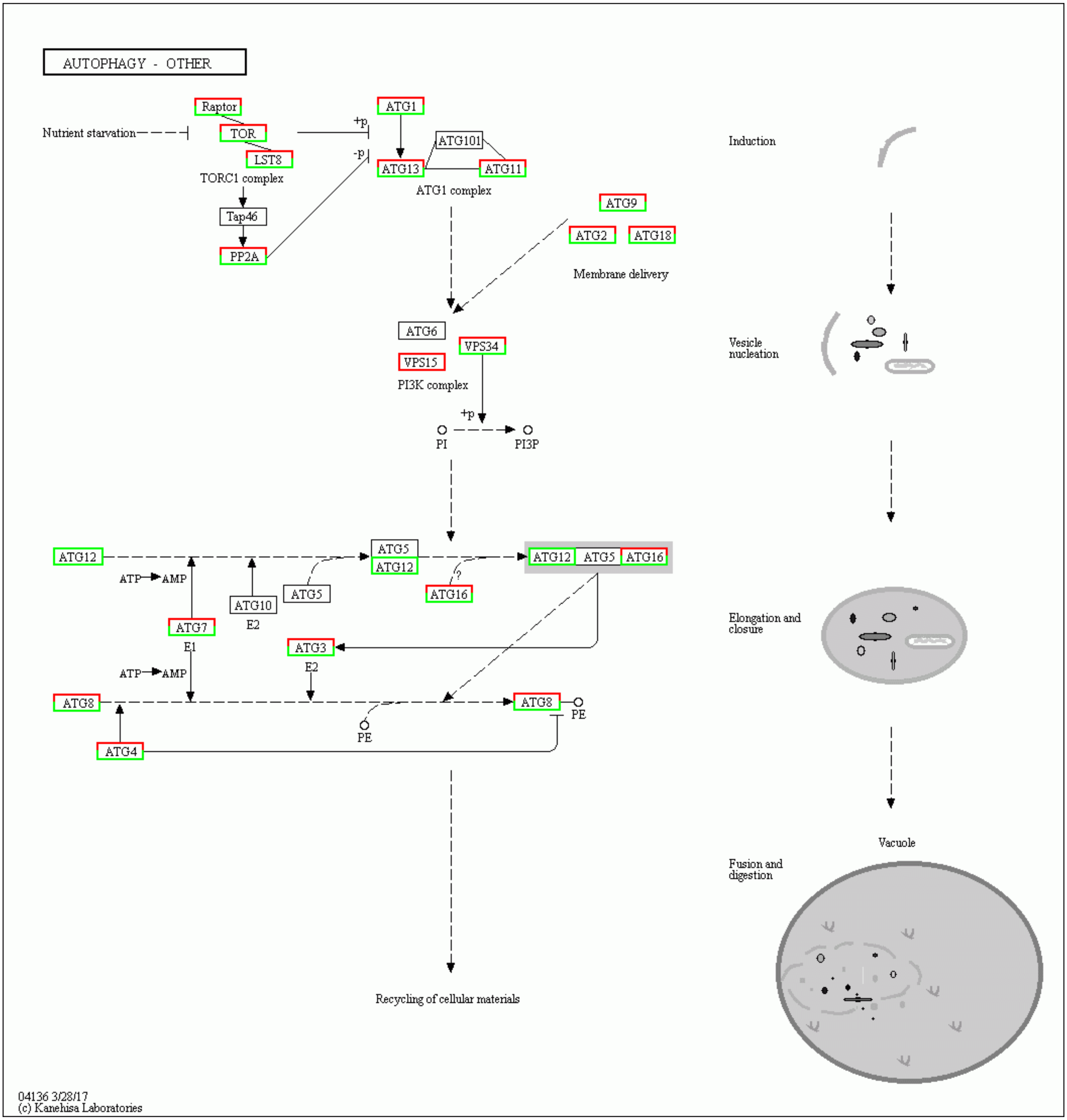
AUTOPHAGY - OTHER
Raptor
TOR
LST8
Nutrient starvation
TORC1 complex
Tap46
PP2A
ATG1
+p
-p
ATG101
ATG13
ATG11
ATG1 complex
Induction
ATG9
ATG2
ATG18
Membrane delivery
ATG6
VPS34
VPS15
PI3K complex
Vesicle nucleation
+p
PI
PI3P
ATG12
ATP
AMP
ATG5
ATG12
ATG12
ATG5
ATG16
?
ATG16
ATG5
ATG10
E2
ATG7
E1
ATG3
E2
Elongation and closure
ATG8
ATG8
PE
PE
ATG4
Vacuole
Fusion and digestion
Recycling of cellular materials
04136 3/28/17
(c) Kanehisa Laboratories

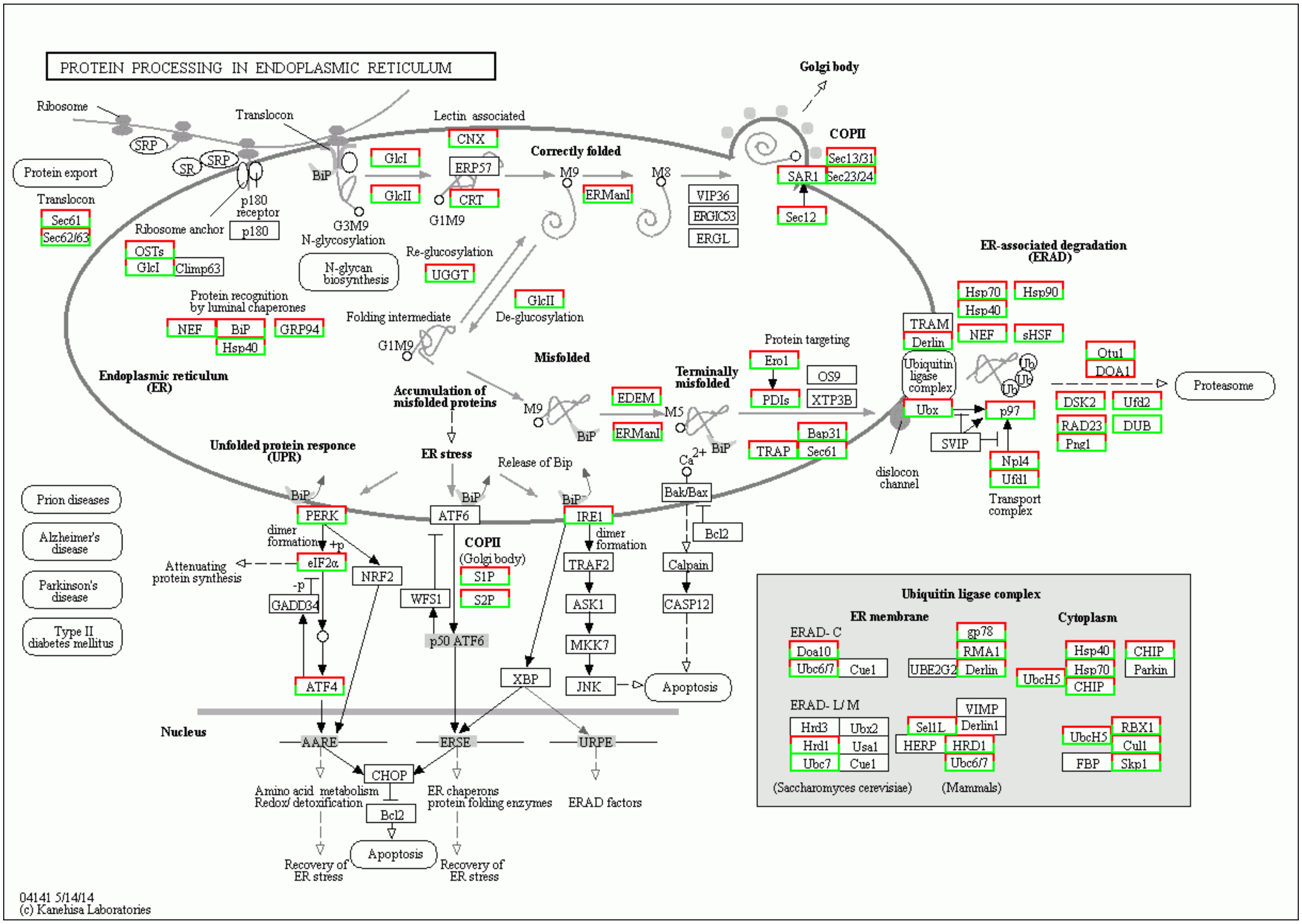
PROTEIN PROCESSING IN ENDOPLASMIC RETICULUM
Golgi body
Ribosome
Translocon
Lectin associated
SRP
SR
Protein export
Translocon
Sec61
Sec62/63
p180 receptor
Ribosome anchor
p180
BiP
GlcI
GlcII
CNX
ERP57
CRT
Correctly folded
M9
M8
ERManI
VIP36
ERGIC53
ERGL
COPII
Sec13/31
Sec23/24
SAR1
Sec12
G3M9
N-glycosylation
G1M9
OSTs
GlcI
Climp63
N-glycan biosynthesis
Re-glucosylation
UGGT
GlcII
De-glucosylation
Protein recognition by luminal chaperones
NEF
BiP
GRP94
Hsp40
Folding intermediate
G1M9
Misfolded
Endoplasmic reticulum (ER)
Accumulation of misfolded proteins
EDEM
ERManI
Terminally misfolded
Protein targeting
Ero1
PDIs
OS9
XTP3B
TRAM
Derlin
Ubiquitin ligase complex
Ubx
p97
SVIP
Npl4
Ufd1
Transport complex
dislocon channel
Bap31
TRAP
Sec61
ER-associated degradation (ERAD)
Hsp70
Hsp90
Hsp40
NEF
sHSF
Otu1
DOA1
Proteasome
DSK2
Ufd2
RAD23
DUB
Png1
Unfolded protein responce (UPR)
ER stress
Release of Bip
Prion diseases
Alzheimer's disease
Parkinson's disease
Type II diabetes mellitus
PERK
ATF6
IRE1
dimer formation
+p
eIF2α
Attenuating protein synthesis
NRF2
-p
GADD34
ATF4
WFS1
COPII (Golgi body)
S1P
S2P
p50 ATF6
TRAF2
ASK1
MKK7
JNK
XBP
Apoptosis
Bak/Bax
Bcl2
Calpain
CASP12
Nucleus
AARE
ERSE
URPE
CHOP
Amino acid metabolism
Redox/ detoxification
Bcl2
Apoptosis
ER chaperons
protein folding enzymes
ERAD factors
Recovery of ER stress
Recovery of ER stress
Ubiquitin ligase complex
ER membrane
Cytoplasm
ERAD- C
Doa10
Ubc6/7
Cue1
gp78
RMA1
UBE2G2
Derlin
Hsp40
Hsp70
CHIP
CHIP
Parkin
UbcH5
ERAD- L/ M
Hrd3
Ubx2
Hrd1
Usa1
Ubc7
Cue1
VIMP
Sel1L
Derlin1
HERP
HRD1
Ubc6/7
UbcH5
RBX1
Cul1
FBP
Skp1
(Saccharomyces cerevisiae)
(Mammals)
04141 5/14/14
(c) Kanehisa Laboratories

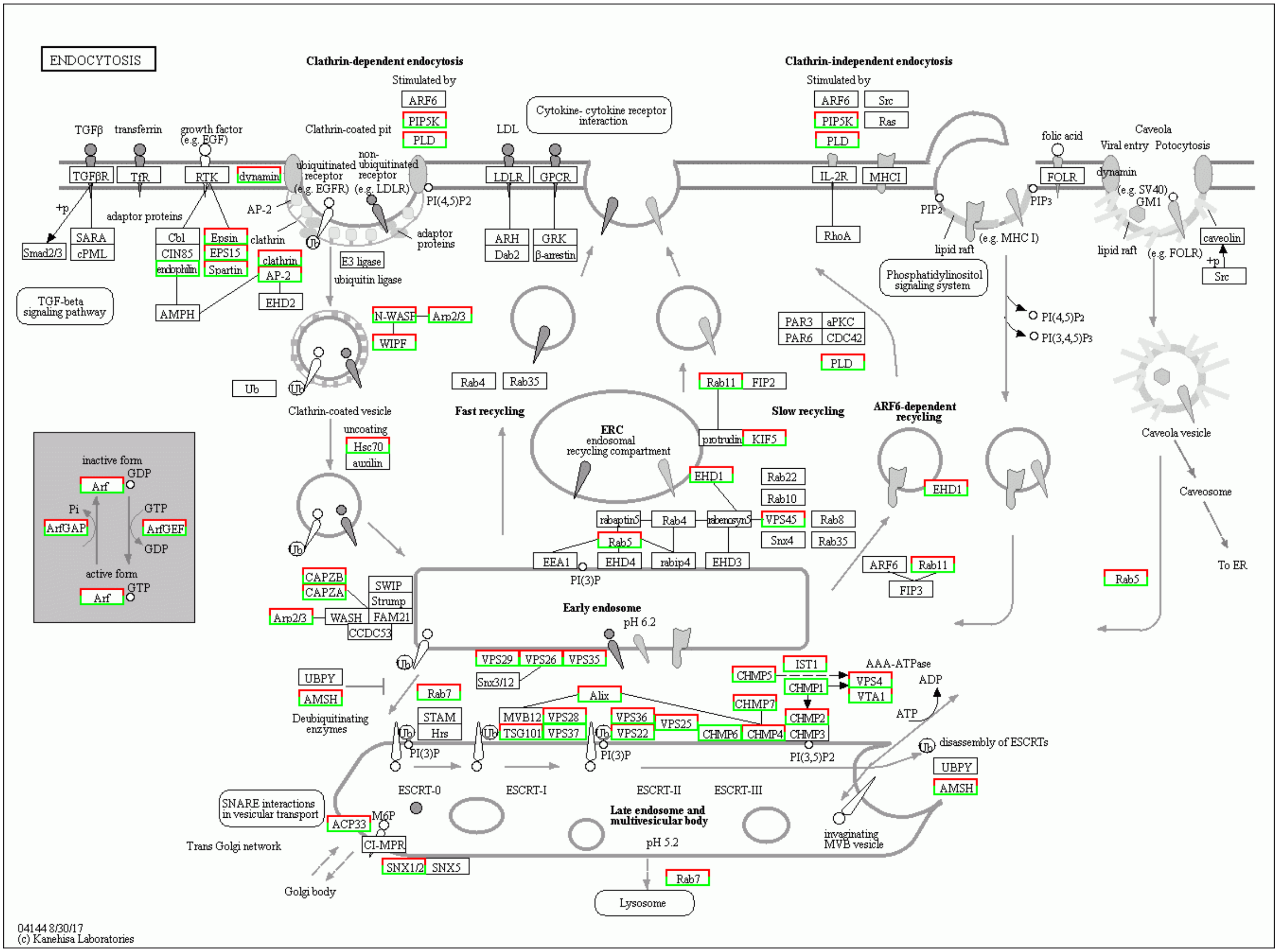
ENDOCYTOSIS
Clathrin-dependent endocytosis
Stimulated by
ARF6
PIP5K
PLD
Cytokine- cytokine receptor interaction
Clathrin-independent endocytosis
TGFβ
transferrin
growth factor (e.g. EGF)
Clathrin-coated pit
LDL
TGFβR
TfR
RTK
dynamin
LDLR
GPCR
IL-2R
MHCI
FOLR
caveolin
Src
Ras
folic acid
Caveola
Viral entry
Potocytosis
SARA
cPML
Smad2/3
Cbl
CIN85
endophilin
Epsin
EPS15
Spartin
clathrin
AP-2
EHD2
AMPH
ARH
Dab2
GRK
β-arrestin
RhoA
Phosphatidylinositol signaling system
TGF-beta signaling pathway
E3 ligase
ubiquitin ligase
N-WASP
Arp2/3
WIPF
Ub
Clathrin-coated vesicle
uncoating
Hsc70
auxilin
Fast recycling
Rab4
Rab35
Rab11
FIP2
ERC
endosomal recycling compartment
protrudin
KIF5
Slow recycling
PAR3
aPKC
PAR6
CDC42
PLD
ARF6-dependent recycling
EHD1
Rab22
Rab10
rabaptin5
Rab5
rabenosyn5
VPS45
Rab8
Snx4
Rab35
EEA1
EHD4
rabip4
EHD3
PI(3)P
Early endosome
pH 6.2
ARF6
FIP3
Caveola vesicle
Caveosome
To ER
Rab5
inactive form
active form
Arf
GDP
GTP
Pi
ArfGAP
ArfGEF
CAPZB
CAPZA
SWIP
Strump
WASH
FAM21
CCDC53
VPS29
VPS26
VPS35
Snx3/12
UBPY
AMSH
Deubiquitinating enzymes
Rab7
Alix
STAM
Hrs
MVB12
VPS28
TSG101
VPS37
VPS36
VPS22
VPS25
CHMP6
CHMP4
CHMP3
CHMP2
CHMP7
CHMP5
CHMP1
IST1
VPS4
VTA1
AAA-ATPase
ADP
ATP
PI(3,5)P2
disassembly of ESCRTs
ESCRT-0
ESCRT-I
ESCRT-II
ESCRT-III
Late endosome and multivesicular body
pH 5.2
invaginating MVB vesicle
SNARE interactions in vesicular transport
Trans Golgi network
ACP33
M6P
CI-MPR
SNX1/2
SNX5
Golgi body
Lysosome
Rab7
04144 8/30/17
(c) Kanehisa Laboratories

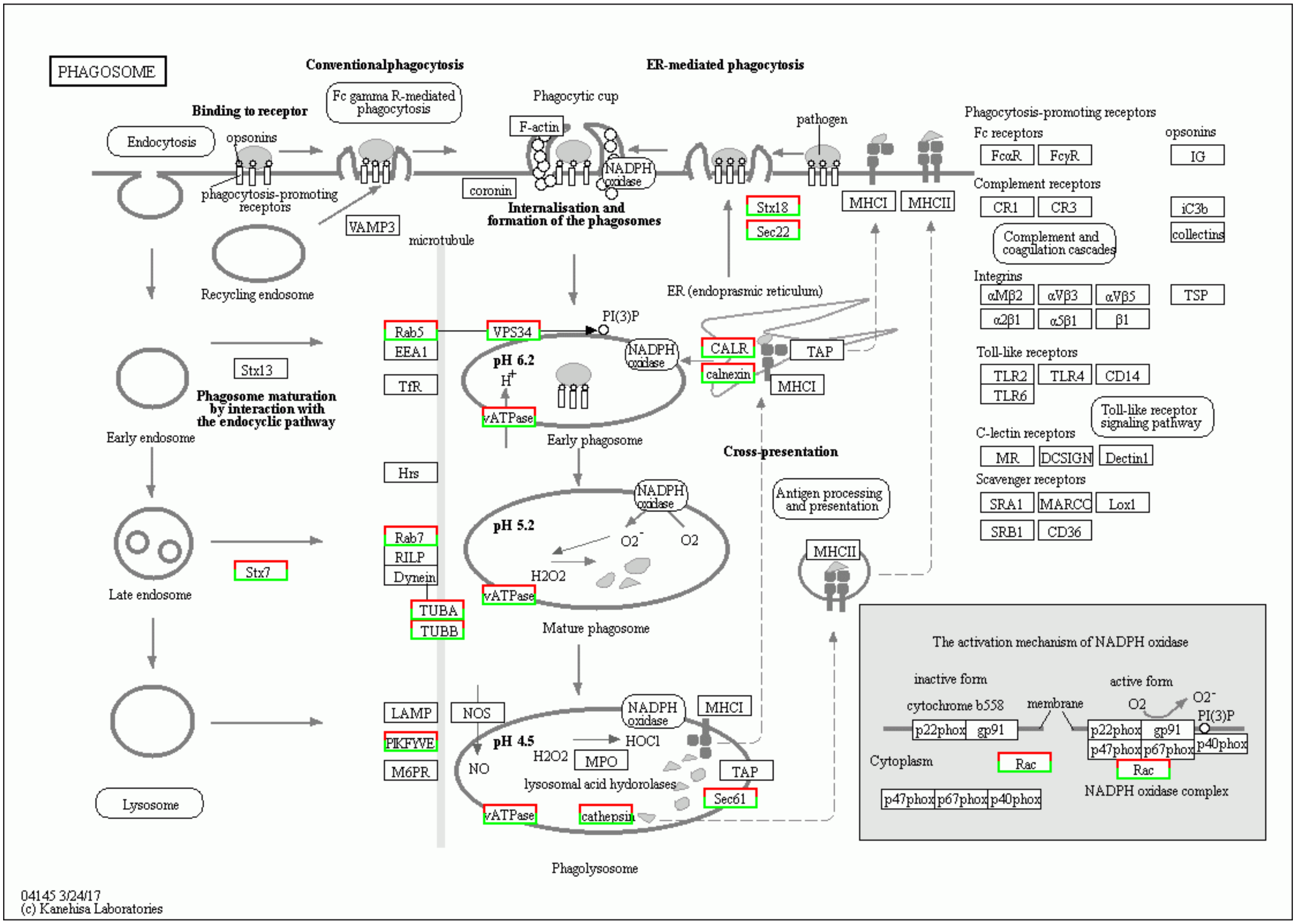
PHAGOSOME
Conventionalphagocytosis
ER-mediated phagocytosis
Binding to receptor
Fc gamma R-mediated phagocytosis
Phagocytic cup
Endocytosis
opsonins
phagocytosis-promoting receptors
F-actin
NADPH oxidase
coronin
Internalisation and formation of the phagosomes
VAMP3
microtubule
Recycling endosome
pathogen
Stx18
Sec22
MHCI
MHCII
Phagocytosis-promoting receptors
Fc receptors
FcαR
FcγR
opsonins
IG
Complement receptors
CR1
CR3
iC3b
Complement and coagulation cascades
collectins
Integrins
αMβ2
αVβ3
αVβ5
TSP
α2β1
α5β1
β1
ER (endoprasmic reticulum)
Rab5
VPS34
PI(3)P
EEA1
pH 6.2
H+
CALR
TAP
calnexin
MHCI
TfR
Stx13
Phagosome maturation by interaction with the endocyclic pathway
Early endosome
vATPase
Early phagosome
Cross-presentation
Toll-like receptors
TLR2
TLR4
CD14
TLR6
Toll-like receptor signaling pathway
C-lectin receptors
MR
DCSIGN
Dectin1
Scavenger receptors
SRA1
MARCO
Lox1
SRB1
CD36
Hrs
Antigen processing and presentation
Rab7
RILP
Dynein
pH 5.2
O2-
O2
H2O2
MHCII
Stx7
Late endosome
TUBA
TUBB
Mature phagosome
The activation mechanism of NADPH oxidase
inactive form
active form
cytochrome b558
membrane
p22phox
gp91
p47phox
p67phox
p40phox
Cytoplasm
Rac
NADPH oxidase complex
LAMP
NOS
MHCI
pH 4.5
HOCl
PIKFYVE
MPO
NO
M6PR
lysosomal acid hydrolases
Lysosome
vATPase
cathepsin
Sec61
Phagolysosome
04145 3/24/17
(c) Kanehisa Laboratories

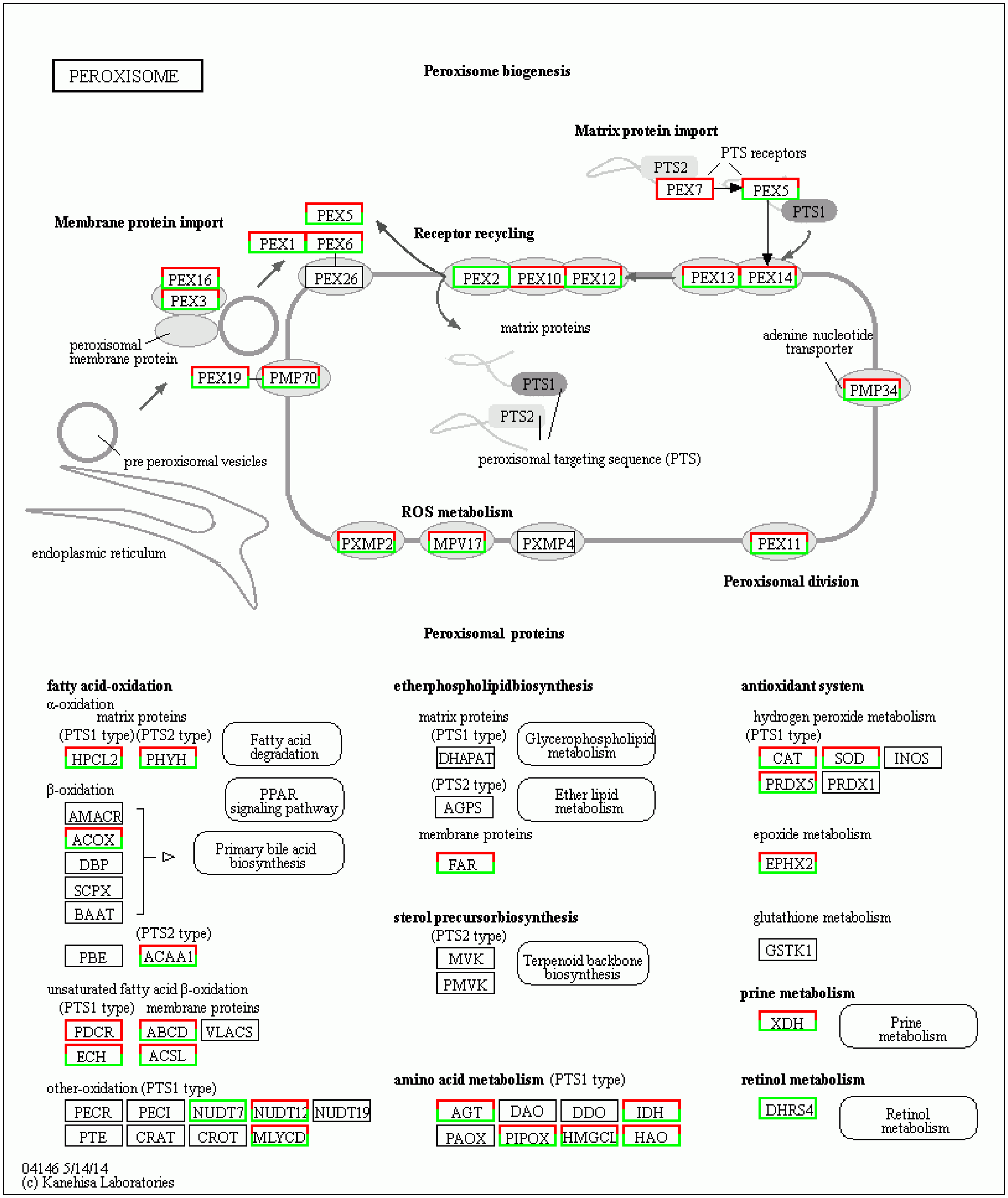
PEROXISOME
Peroxisome biogenesis
Matrix protein import
PTS receptors
PTS2
PEX7
PEX5
PTS1
Membrane protein import
PEX5
PEX1
PEX6
Receptor recycling
PEX16
PEX3
PEX26
PEX2
PEX10
PEX12
PEX13
PEX14
matrix proteins
adenine nucleotide transporter
peroxisomal membrane protein
PEX19
PMP70
PTS1
PMP34
PTS2
pre peroxisomal vesicles
peroxisomal targeting sequence (PTS)
ROS metabolism
endoplasmic reticulum
PXMP2
MPV17
PXMP4
PEX11
Peroxisomal division
Peroxisomal proteins
fatty acid-oxidation
α-oxidation
matrix proteins
(PTS1 type)(PTS2 type)
HPCL2
PHYH
Fatty acid degradation
β-oxidation
PPAR signaling pathway
AMACR
ACOX
DBP
SCPX
BAAT
Primary bile acid biosynthesis
(PTS2 type)
PBE
ACAA1
unsaturated fatty acid β-oxidation
(PTS1 type) membrane proteins
PDCR
ABCD
VLACS
ECH
ACSL
other-oxidation (PTS1 type)
PECR
PECI
NUDT7
NUDT12
NUDT19
PTE
CRAT
CROT
MLYCD
etherphospholipidbiosynthesis
matrix proteins
(PTS1 type)
DHAPAT
Glycerophospholipid metabolism
(PTS2 type)
AGPS
Ether lipid metabolism
membrane proteins
FAR
sterol precursorbiosynthesis
(PTS2 type)
MVK
PMVK
Terpenoid backbone biosynthesis
amino acid metabolism (PTS1 type)
AGT
DAO
DDO
IDH
PAOX
PIPOX
HMGCL
HAO
antioxidant system
hydrogen peroxide metabolism
(PTS1 type)
CAT
SOD
INOS
PRDX5
PRDX1
epoxide metabolism
EPHX2
glutathione metabolism
GSTK1
prine metabolism
XDH
Prine metabolism
retinol metabolism
DHRS4
Retinol metabolism
04146 5/14/14
(c) Kanehisa Laboratories

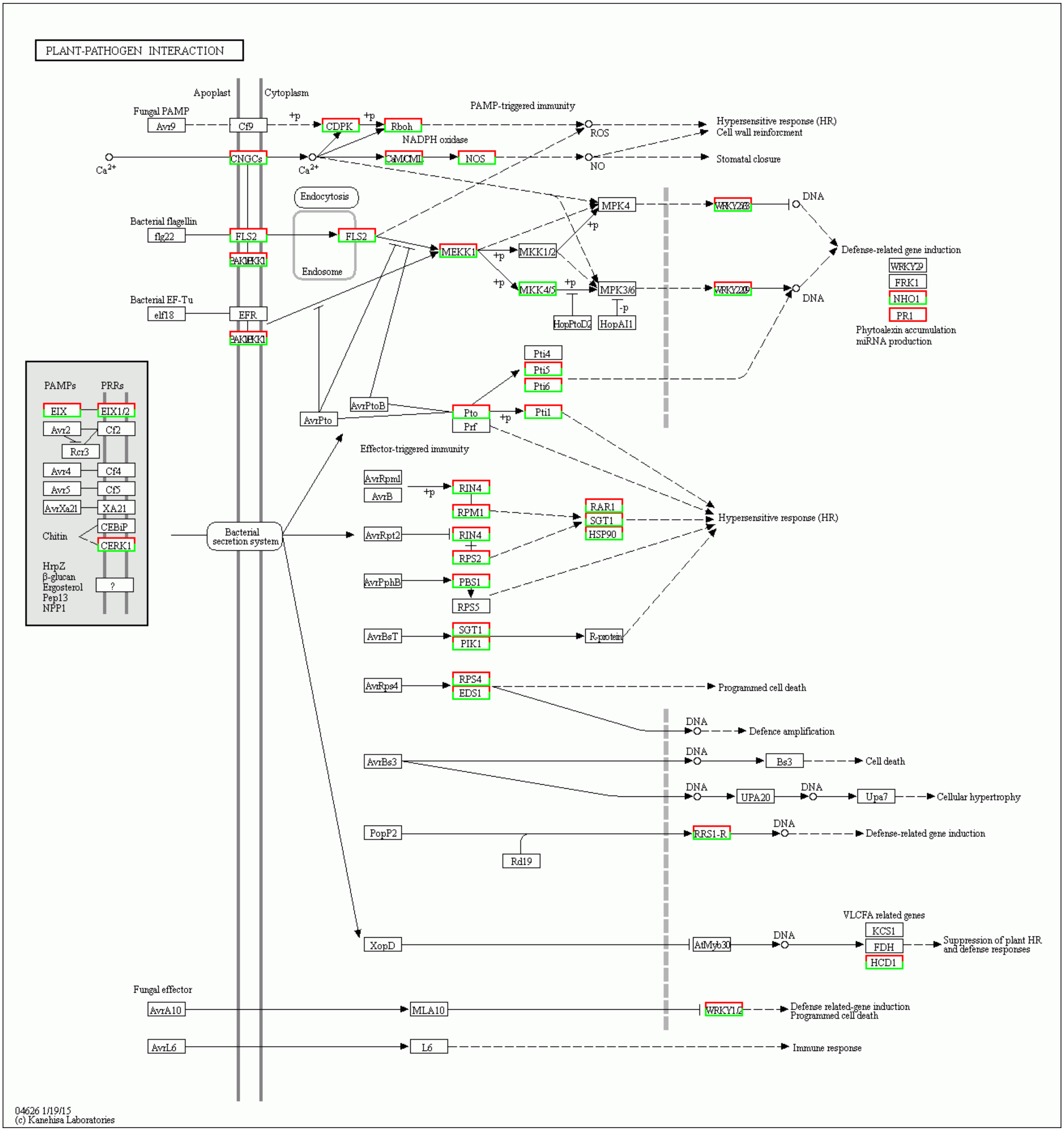
PLANT-PATHOGEN INTERACTION
Apoplast
Cytoplasm
Fungal PAMP
Avr9
Cf9
+p
CDPK
+p
Rboh
NADPH oxidase
PAMP-triggered immunity
ROS
Hypersensitive response (HR)
Cell wall reinforcment
Ca2+
CNGCs
Ca2+
CaM/CML
NOS
NO
Stomatal closure
Endocytosis
Endosome
Bacterial flagellin
flg22
FLS2
BAK1/BKK1
FLS2
MEKK1
MKK1/2
MPK4
WRKY25/33
DNA
+p
+p
+p
MKK4/5
+p
MPK3/6
WRKY22/29
DNA
-p
HopPtoD2
HopAI1
Defense-related gene induction
WRKY29
FRK1
NHO1
PR1
Phytoalexin accumulation
miRNA production
Bacterial EF-Tu
elf18
EFR
BAK1/BKK1
PAMPs
PRRs
EIX
EIX1/2
Avr2
Cf2
Rcr3
Avr4
Cf4
Avr5
Cf5
AvrXa21
XA21
CEBiP
Chitin
CERK1
HrpZ
β-glucan
Ergosterol
Pep13
NPP1
?
Pti4
Pti5
Pti6
AvrPtoB
AvrPto
Pto
Prf
+p
Pti1
Effector-triggered immunity
AvrRpm1
AvrB
+p
RIN4
RPM1
RAR1
SGT1
HSP90
Hypersensitive response (HR)
Bacterial secretion system
AvrRpt2
RIN4
RPS2
AvrPphB
PBS1
RPS5
AvrBsT
SGT1
PIK1
R-protein
AvrRps4
RPS4
EDS1
Programmed cell death
DNA
Defence amplification
AvrBs3
DNA
Bs3
Cell death
DNA
UPA20
DNA
Upa7
Cellular hypertrophy
PopP2
RRS1-R
DNA
Defense-related gene induction
Rd19
VLCFA related genes
KCS1
DNA
XopD
AtMyb30
FDH
Suppression of plant HR and defense responses
HCD1
Fungal effector
AvrA10
MLA10
WRKY1/2
Defense related-gene induction
Programmed cell death
AvrL6
L6
Immune response
04626 1/19/15
(c) Kanehisa Laboratories

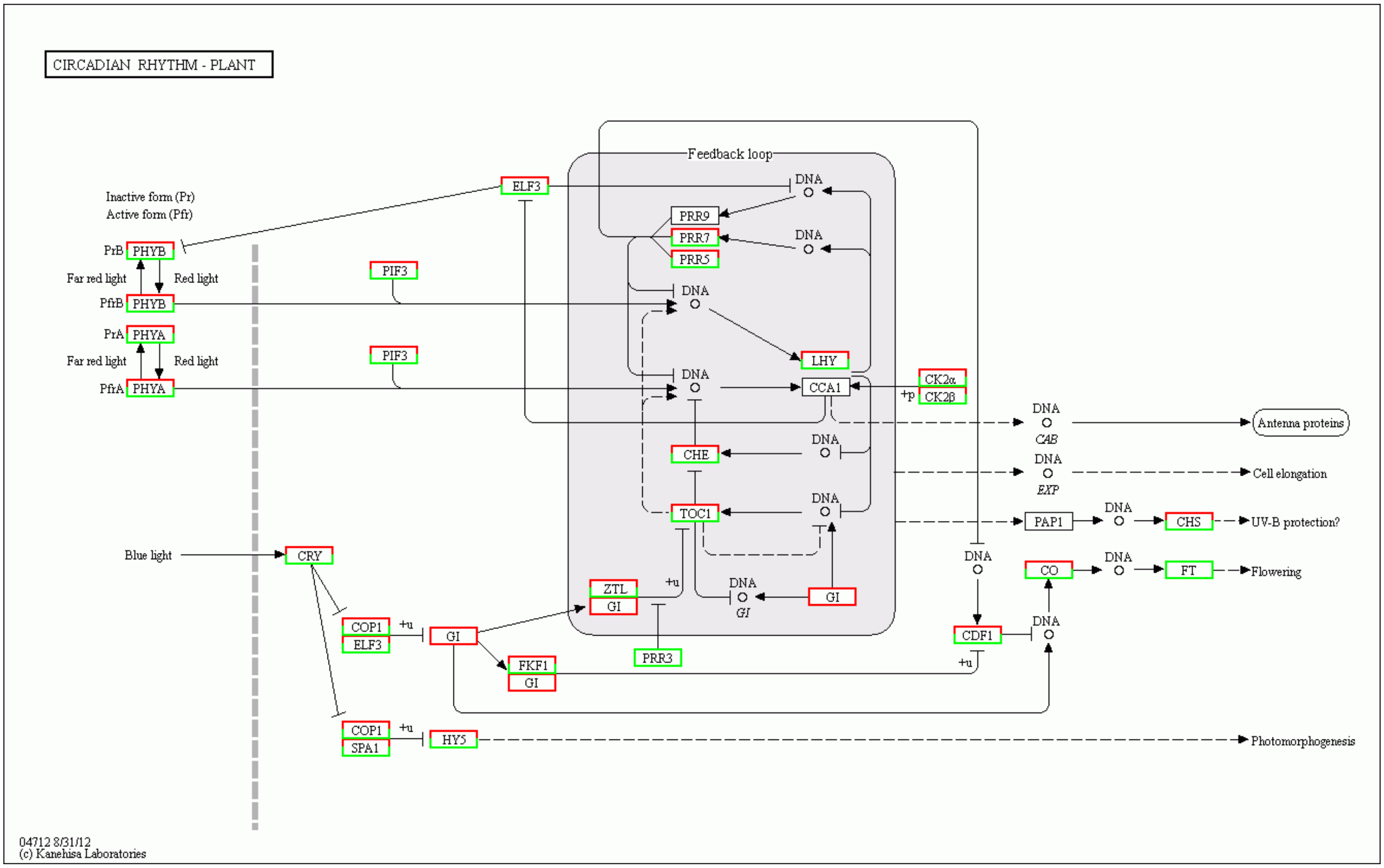
CIRCADIAN RHYTHM - PLANT
Feedback loop
Inactive form (Pr)
Active form (Pfr)
PHYB
PHYA
Far red light
Red light
PIF3
ELF3
PRR9
PRR7
PRR5
DNA
LHY
CCA1
CK2α
CK2β
CHE
TOC1
ZTL
GI
FKF1
PRR3
CRY
Blue light
COP1
SPA1
HY5
CDF1
CO
FT
PAP1
CHS
Antenna proteins
Cell elongation
UV-B protection?
Flowering
Photomorphogenesis
CAB
EXP
04712 8/31/12
(c) Kanehisa Laboratories

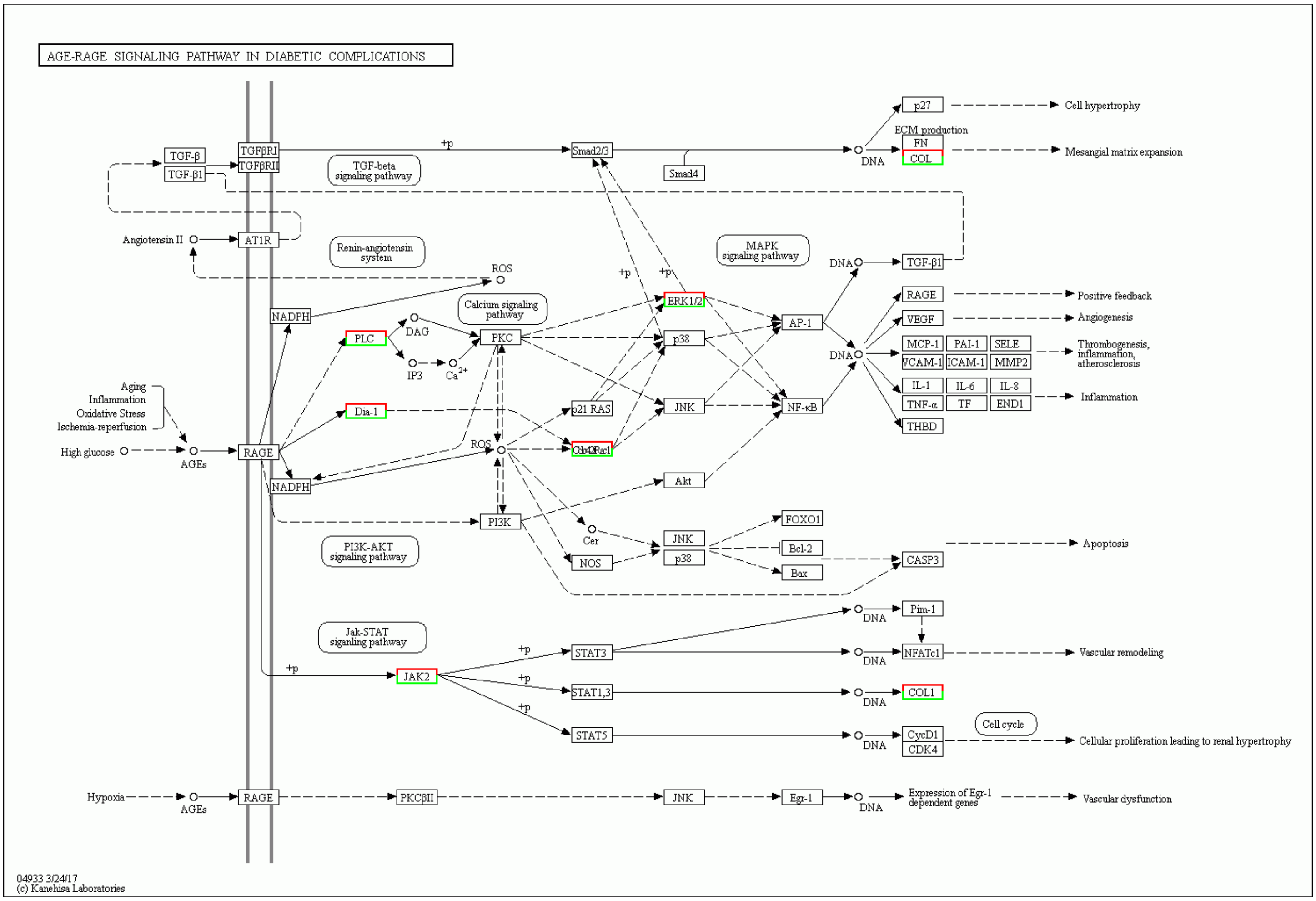
AGE-RAGE SIGNALING PATHWAY IN DIABETIC COMPLICATIONS
TGF-β
TGF-β1
TGFβRI
TGFβRII
TGF-beta signaling pathway
Smad2/3
Smad4
Angiotensin II
AT1R
Renin-angiotensin system
ROS
MAPK signaling pathway
NADPH
PLC
DAG
IP3
PKC
Calcium signaling pathway
ERK1/2
p38
AP-1
Dia-1
p21 RAS
JNK
NF-κB
Cdc42/Rac1
Aging
Inflammation
Oxidative Stress
Ischemia-reperfusion
High glucose
AGEs
RAGE
Akt
PI3K
PI3K-AKT signaling pathway
Cer
NOS
FOXO1
Bcl-2
Bax
CASP3
Jak-STAT signaling pathway
JAK2
STAT3
STAT1,3
STAT5
Pim-1
NFATc1
COL1
CycD1
CDK4
Cell cycle
Hypoxia
PKCβII
Egr-1
Expression of Egr-1 dependent genes
p27
ECM production
FN
COL
TGF-β1
VEGF
MCP-1
PAI-1
SELE
VCAM-1
ICAM-1
MMP2
IL-1
IL-6
IL-8
TNF-α
TF
END1
THBD
Cell hypertrophy
Mesangial matrix expansion
Positive feedback
Angiogenesis
Thrombogenesis, inflammation, atherosclerosis
Inflammation
Apoptosis
Vascular remodeling
Cellular proliferation leading to renal hypertrophy
Vascular dysfunction
04933 3/24/17
(c) Kanehisa Laboratories

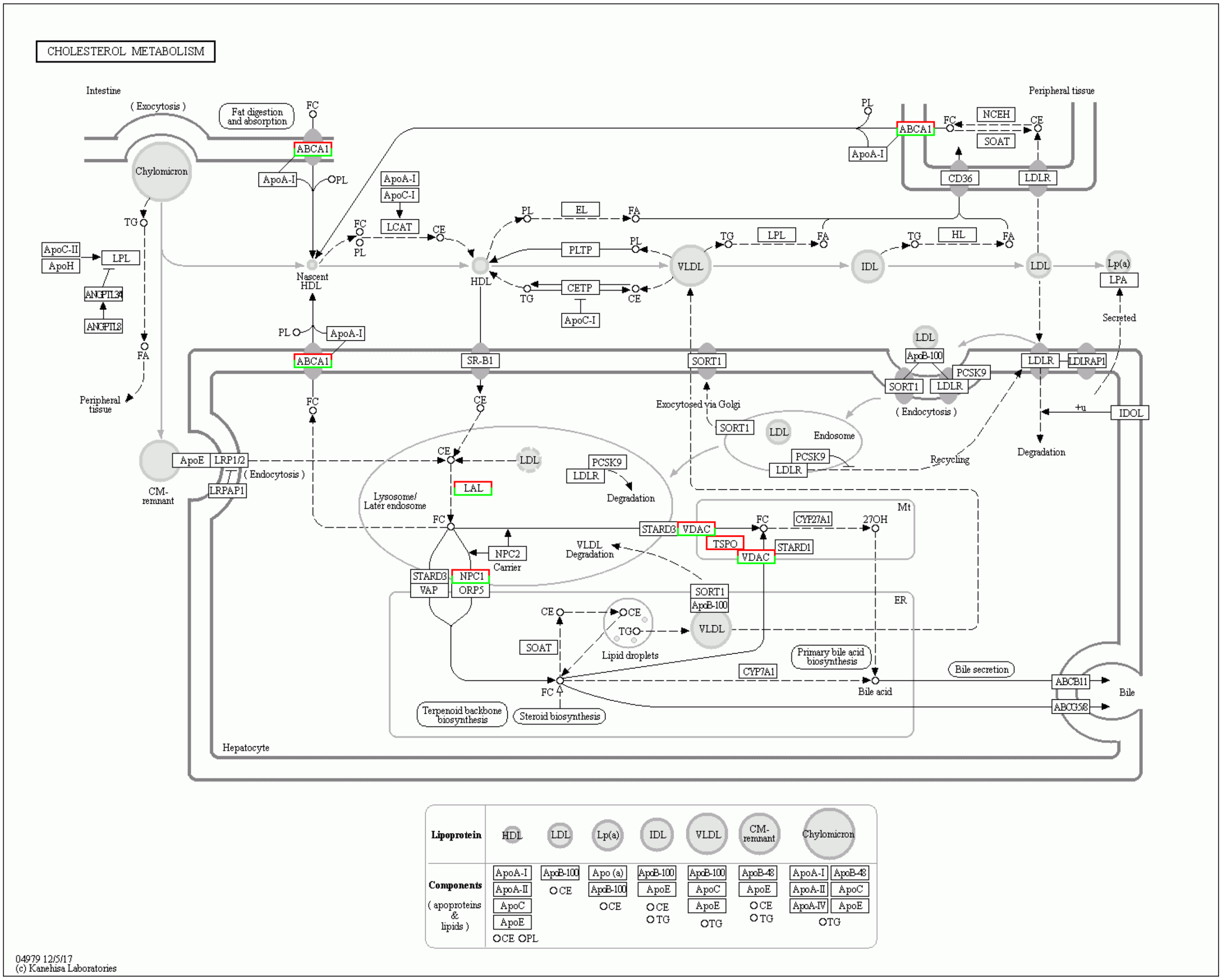
CHOLESTEROL METABOLISM
Intestine
(Exocytosis)
Fat digestion and absorption
Chylomicron
ABCA1
ApoA-I
ApoC-II
ApoH
LPL
ANGPTL3/4
ANGPTL8
Peripheral tissue
Nascent HDL
HDL
LCAT
ApoC-I
EL
PLTP
CETP
VLDL
IDL
LDL
Lp(a)
LPA
Secreted
HL
NCEH
SOAT
CD36
LDLR
LDLRAP1
IDOL
PCSK9
SORT1
ApoB-100
(Endocytosis)
Endosome
Recycling
Degradation
Exocytosed via Golgi
SR-B1
CM-remnant
ApoE
LRP1/2
LRPAP1
Lysosome/ Later endosome
LAL
NPC2
Carrier
STARD3
NPC1
VAP
ORP5
VDAC
TSPO
STARD1
CYP27A1
27OH
Mt
VLDL Degradation
ER
Lipid droplets
Primary bile acid biosynthesis
Bile secretion
CYP7A1
Bile acid
ABCB11
ABCG5/8
Bile
Terpenoid backbone biosynthesis
Steroid biosynthesis
Hepatocyte
Lipoprotein
Components
(apoproteins & lipids)
ApoA-II
ApoC
Apo (a)
ApoB-48
ApoA-IV
04979 12/5/17
(c) Kanehisa Laboratories
