# Supplementary figures and images for: De novo transcriptome assembly of Dalbergia sissoo Roxb. (Fabaceae) under Botryodiplodia theobromae-induced dieback disease
Source: Sci Rep. 2023 Nov 22;13:20503. doi: 10.1038/s41598-023-45982-8 (PMC10665356; doi:10.1038/s41598-023-45982-8)

**Supplementary File S5:** Illustrations of protein-interaction networks of DEGs in all samples.

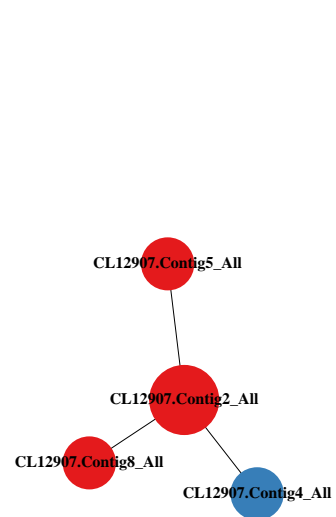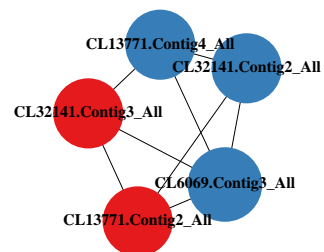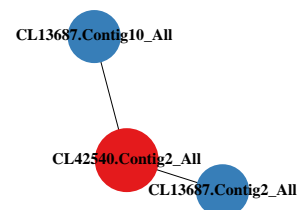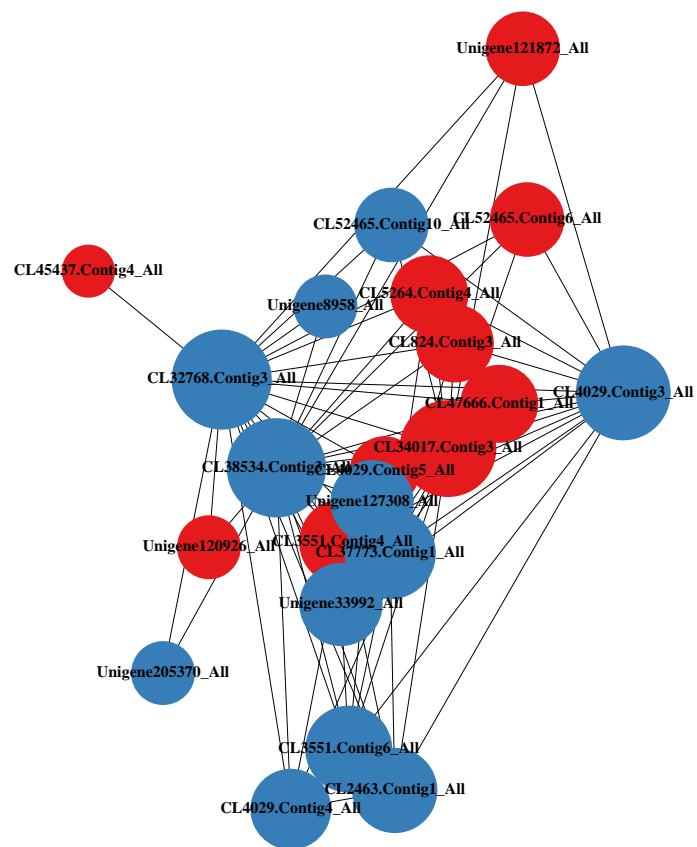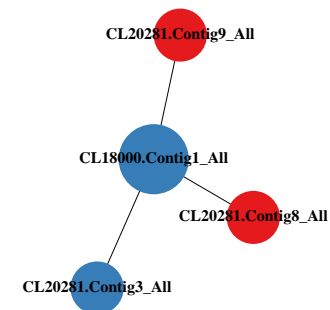

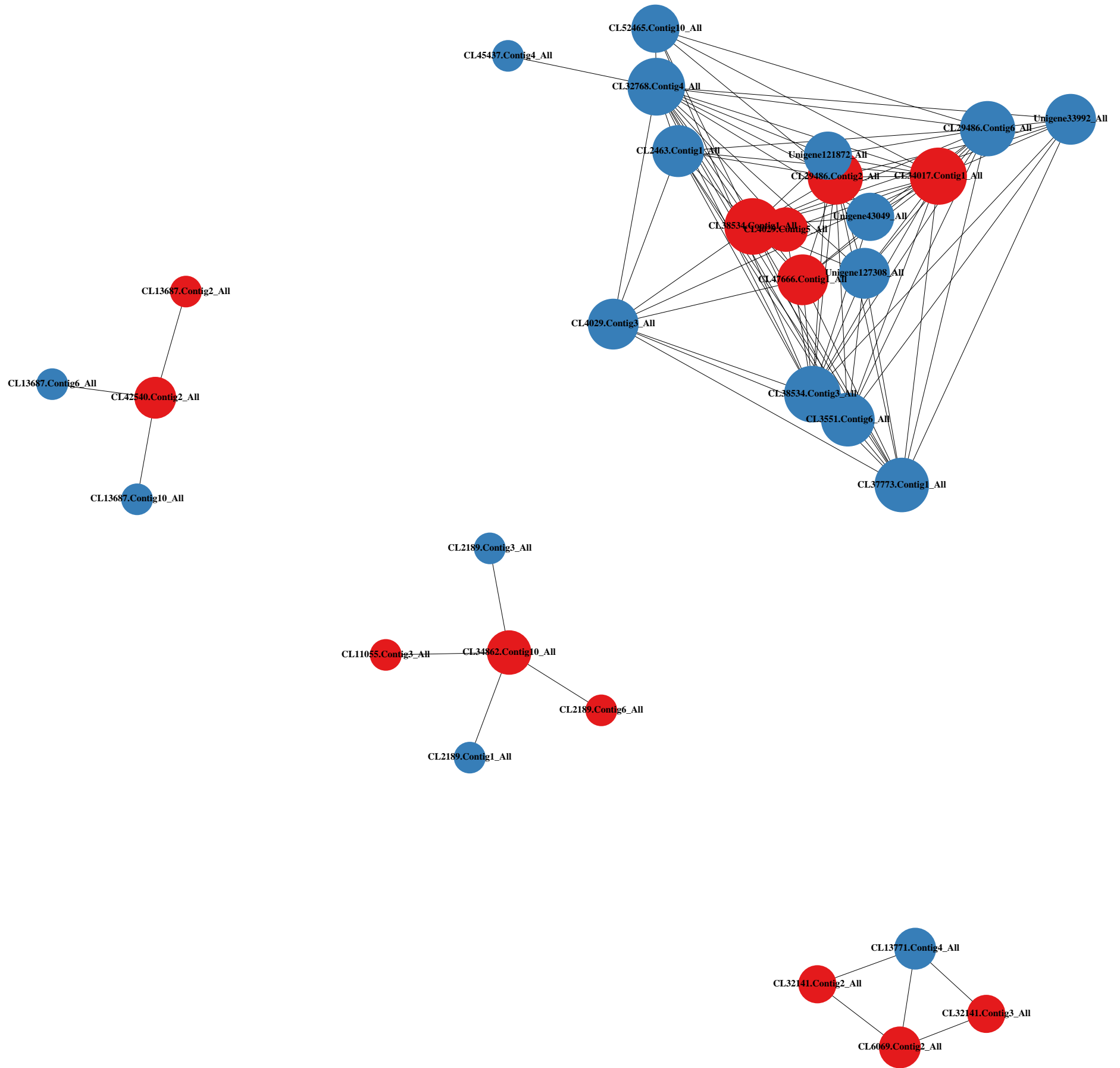

Supplement: Supplementary file 5 — Supplementary Information 5. [file 41598_2023_45982_MOESM5_ESM.pdf]
