# Supplementary material for: De novo transcriptome assembly of Dalbergia sissoo Roxb. (Fabaceae) under Botryodiplodia theobromae-induced dieback disease
Source: Sci Rep. 2023 Nov 22;13:20503. doi: 10.1038/s41598-023-45982-8 (PMC10665356; doi:10.1038/s41598-023-45982-8)
Supplement: Supplementary file 12 — Supplementary Figure S1. [file 41598_2023_45982_MOESM12_ESM.pdf]

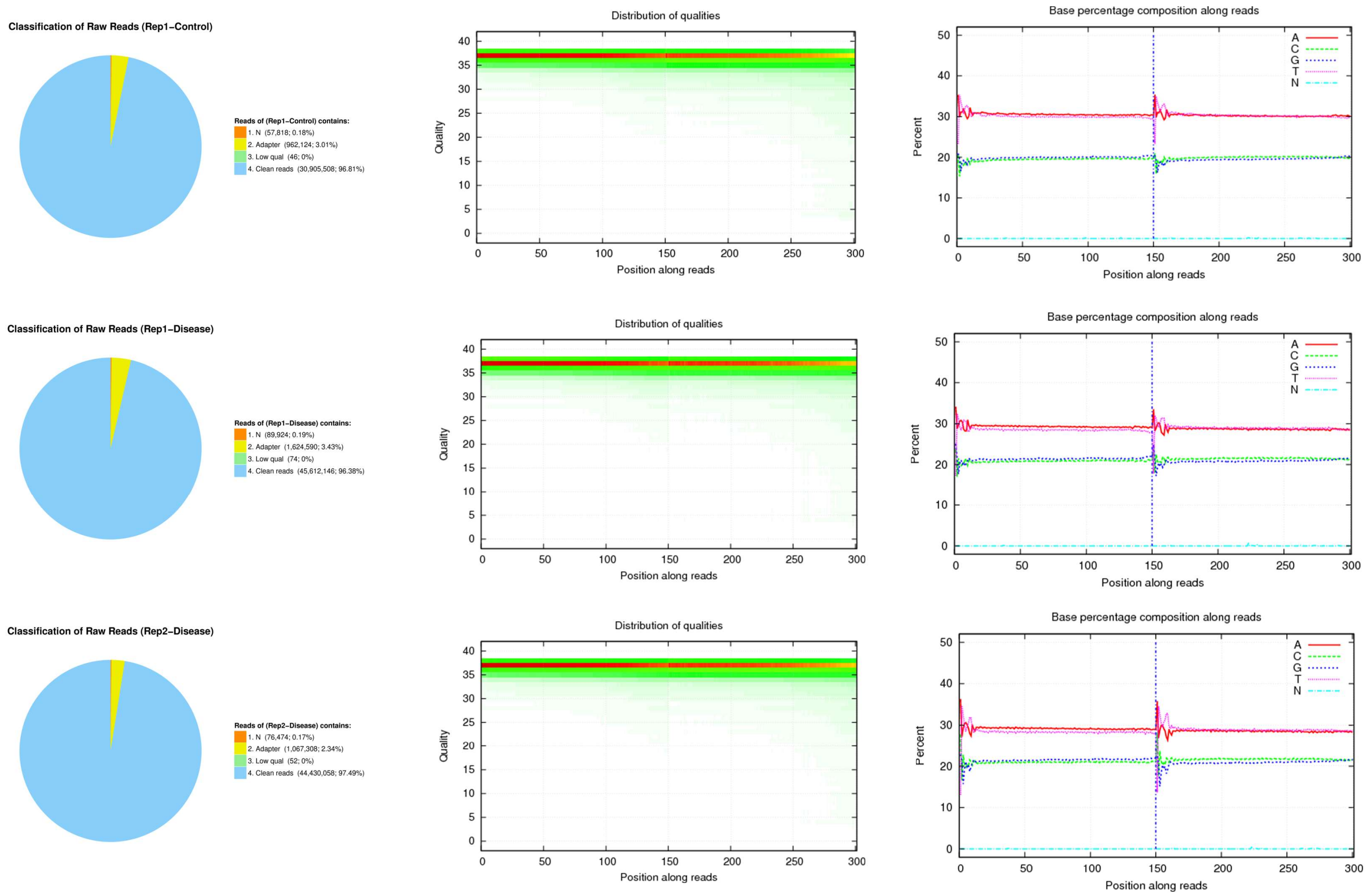

(a) **(b)** **(c)**

Supplementary Figure S1: **(a) Raw data filter composition chart.** N: The total amount of reads that contain more than 5% unknown N base, the N reads ratio; Adapter: The total amount of reads which contain adaptors; the adaptor ratio; Low qual: More than 20% of bases in the total read have a quality score lower than 15; low quality reads ratio; Clean reads: Reads filtered with N reads, reads have adaptors and low-quality reads; clean reads ratio. **(b) Distribution of base quality on clean reads.** The X-axis represents base positions along reads and the Y-axis represents the base quality value. Each dot in the image represents the number of total bases with a certain quality value of the corresponding base along reads. A darker dot color means a greater base number. If the proportion of the bases with low quality (< 20) is very low, that means the sequencing quality of this lane is good. **(c) Base percentage composition along reads.** The X-axis represents base positions along reads and the Y-axis represents the base quality percentage for each (A, C, G, T, and N) base. Each colored line represents a different base.
