# Supplementary material for: De novo transcriptome assembly of Dalbergia sissoo Roxb. (Fabaceae) under Botryodiplodia theobromae-induced dieback disease
Source: Sci Rep. 2023 Nov 22;13:20503. doi: 10.1038/s41598-023-45982-8 (PMC10665356; doi:10.1038/s41598-023-45982-8)
Supplement: Supplementary file 13 — Supplementary Figure S2. [file 41598_2023_45982_MOESM13_ESM.pdf]

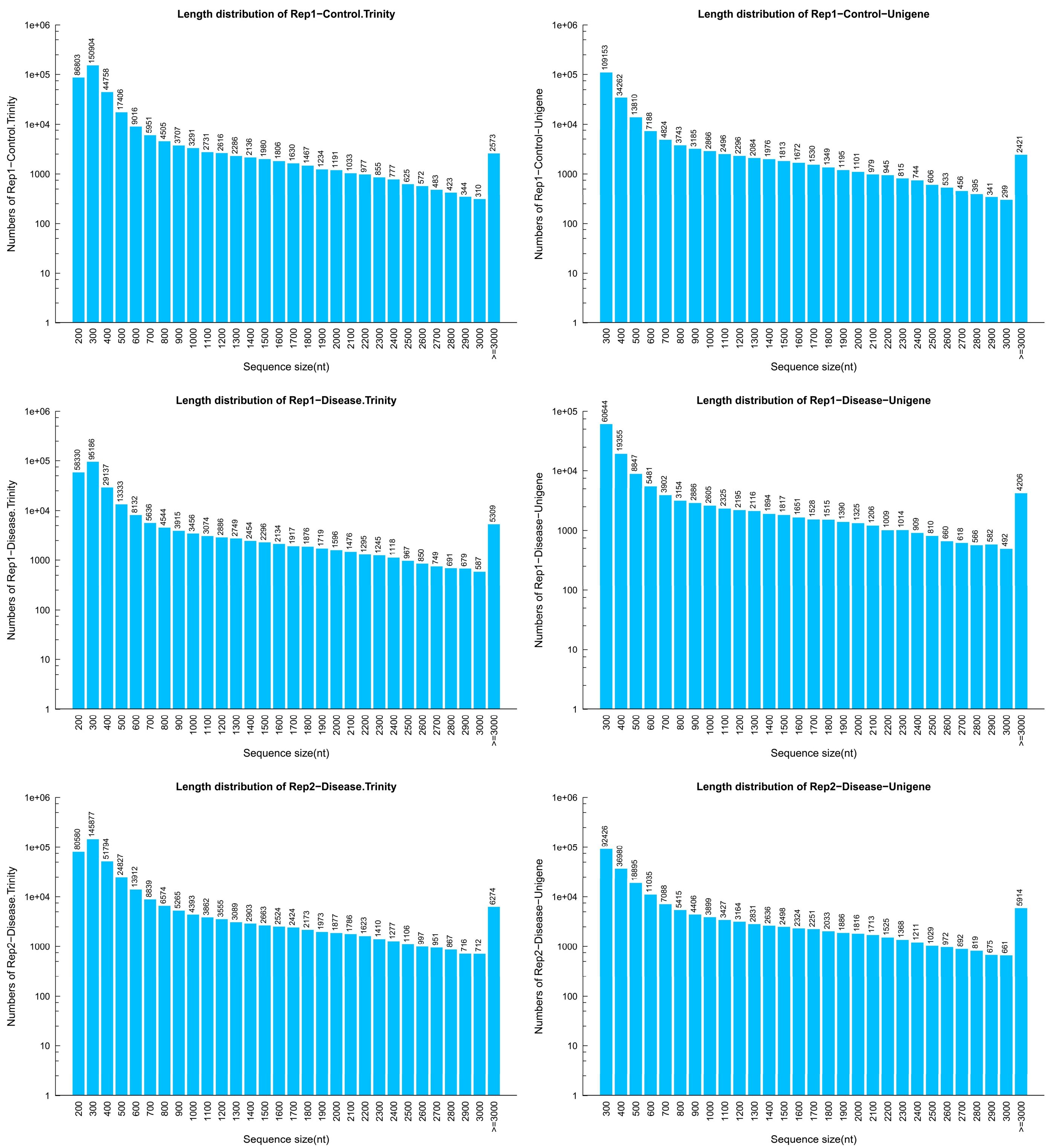

(a) **(b)**

Supplementary Figure S2: **(a) Transcript length distribution.** The X-axis represents the length of transcripts and the Y-axis represents the number of transcripts. **(b) Unigene length distribution.** The X-axis represents the length of Unigenes and the Y-axis represents the number of Unigenes.
