# Supplementary material for: De novo transcriptome assembly of Dalbergia sissoo Roxb. (Fabaceae) under Botryodiplodia theobromae-induced dieback disease
Source: Sci Rep. 2023 Nov 22;13:20503. doi: 10.1038/s41598-023-45982-8 (PMC10665356; doi:10.1038/s41598-023-45982-8)
Supplement: Supplementary file 14 — Supplementary Figure S3. [file 41598_2023_45982_MOESM14_ESM.pdf]

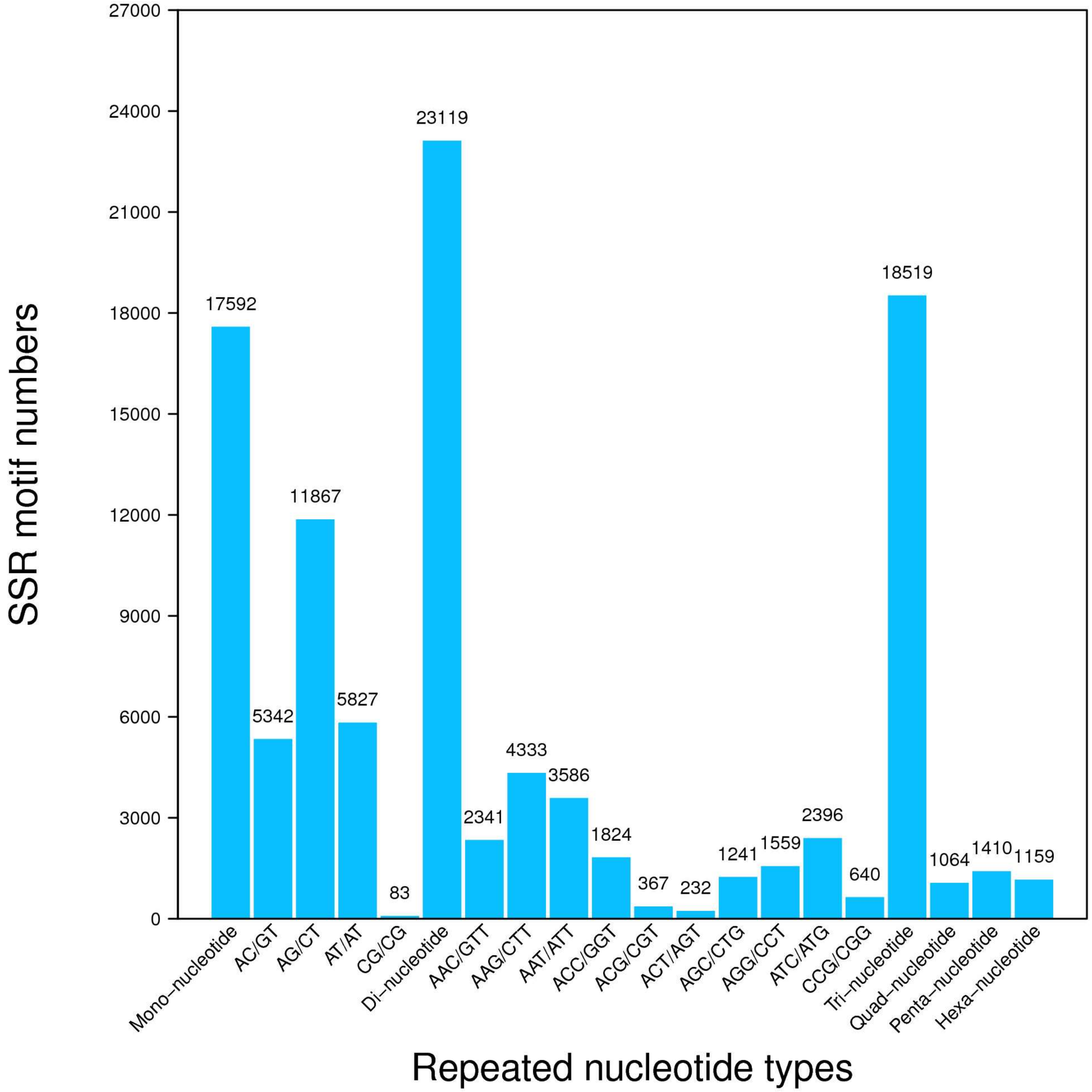

Supplementary Figure S3: **SSR size distribution.** The X-axis represents the type of SSR and the Y-axis represents the number of SSR.
